# Phytochemical composition, antioxidant potential, and enzyme inhibitory properties of *Onosma thracica* extracts: A comparative study of extraction methods

**DOI:** 10.1371/journal.pone.0350995

**Published:** 2026-06-10

**Authors:** Mamdouh Alshammari, Cengiz Sarikurkcu, Fevzi Bardakci, Mousa Alreshidi, Mohd Adnan, Riadh Badraoui, Bektas Tepe

**Affiliations:** 1 Department of Biology, College of Science, University of Ha’il, Ha’il, Saudi Arabia; 2 Faculty of Pharmacy, Afyonkarahisar Health Sciences University, Afyonkarahisar, Türkiye; 3 Department of Molecular Biology and Genetics, Faculty of Science, Kilis 7 Aralik University, Kilis, Türkiye; Institute for Biological Research, University of Belgrade, SERBIA

## Abstract

*Onosma thracica* is a promising natural source of bioactive phenolic compounds, including flavonoids, yet the influence of extraction technique on its chemical profile and biological properties remains unclear. In this study, methanol extracts prepared by maceration (MAC), ultrasound-assisted extraction (UAE), and Soxhlet extraction (SOE) were comparatively investigated in terms of their detailed phenolic composition, antioxidant capacity, enzyme inhibitory activity, and *in silico* interaction patterns of individual compounds. Among the tested methods, UAE yielded the highest total phenolic content (45.41 mg GAEs/g extract), whereas SOE produced the richest flavonoid content (71.05 mg REs/g extract). LC–ESI–MS/MS analysis showed that luteolin-7-glucoside and apigenin-7-glucoside were the dominant flavonoids, while chlorogenic acid and rosmarinic acid were the major phenolic acids. Biological assays revealed method-dependent differences in activity. UAE was more effective in total antioxidant capacity and reducing power, while SOE showed stronger radical scavenging and metal chelating performance. In enzyme inhibition assays, SOE displayed the strongest acetylcholinesterase inhibition, whereas tyrosinase inhibition was similar across the extracts. Correlation analysis indicated that the observed bioactivities were closely associated with the overall phenolic composition (including flavonoid constituents), particularly with luteolin-7-glucoside and rosmarinic acid. Docking studies showed favorable interactions of apigenin-7-glucoside and luteolin-7-glucoside with human α-amylase, apigenin-7-glucoside and luteolin with acetylcholinesterase, and rosmarinic acid with human tyrosinase-related protein 1, suggesting that these major phenolic constituents may contribute to the observed bioactivities of *O. thracica*. The results demonstrate that extraction method markedly shapes the phytochemical composition and bioactivity of *O. thracica*, and they support further work aimed at optimizing its potential pharmacological use.

## Introduction

Tyrosinase (EC 1.14.18.1) is a copper-containing enzyme involved in melanin synthesis across plants, animals, and fungi, catalyzing monophenol hydroxylation and o-diphenol oxidation [1]. The resulting o-quinones undergo autoxidation, leading to enzymatic browning, which causes food discoloration and spoilage while also reducing cosmetic and nutritional value [2]. Inhibiting tyrosinase is crucial in controlling melanin production, with both synthetic (e.g., Depigman, Arbutin) and natural inhibitors (e.g., Haginin A and B from *Alnus* species) used in pharmaceutical and cosmetic applications [3]. Due to concerns over synthetic agents, there is growing interest in natural alternatives, yet research remains limited to specific edible and medicinal plants, highlighting the need for broader studies on tyrosinase inhibition in diverse plant sources [4].

Diabetes has the potential to escalate into a pandemic condition due to its widespread occurrence and significantly contributes to the etiology of various chronic diseases. The enzymes α-amylase and α-glucosidase are well-recognized for their roles in the degradation of starch and the absorption of glucose within the gastrointestinal tract [5]. Elevated blood glucose levels stimulate the pancreas to secrete insulin, which functions to diminish the concentration of glucose in the bloodstream by facilitating its uptake by the tissues. Particularly for type II diabetes, the primary therapeutic strategy involves the inhibition of the enzyme that catalyzes glucose synthesis [6]. The inhibition of α-amylase and α-glucosidase is crucial in mitigating the incidence of hyperglycemia [7]. While pharmacological interventions have been employed in the past to lower hyperglycemia, prolonged administration of these agents can result in substantial adverse effects, including flatulence, diarrhea, and abdominal discomfort. Moreover, the emergence of resistance to these pharmacological treatments has prompted investigations into plant-derived inhibitors [8].

Excessive degradation of acetylcholine (ACh) by acetylcholinesterase (AChE) contributes to Alzheimer’s disease (AD), making AChE inhibition a key therapeutic approach to increasing ACh availability in the brain. Natural sources, particularly plant extracts rich in phenolic compounds, have gained attention as potential AChE inhibitors [9]. Since oxidative stress plays a role in neurodegeneration, antioxidants from plants may also help protect against AD by scavenging reactive oxygen species (ROS). Several medicinal plants have been identified as sources of phenolic compounds with both antioxidant and cholinesterase inhibitory properties, suggesting their potential in AD treatment [10]. Given these findings, further exploration of plant-based bioactive compounds could provide new strategies for drug development.

The genus *Onosma* (Boraginaceae) includes around 70 species found in Central and Southwest Asia, North Africa, and Southern Europe, with 11 species native to Turkey. Traditionally used in herbal medicine, *Onosma* has gained attention in drug discovery due to its diverse phytochemicals with cytotoxic and antimutagenic properties. Widely utilized in Anatolia and beyond, it has been employed for treating malaria, typhoid fever, parasitic infections, rheumatism, and eye diseases, as well as serving as an antiseptic, astringent, diuretic, and blood purifier [11–13].

This study aims to comprehensively investigate the chemical composition, antioxidant potential, and enzyme inhibitory activities of *Onosma thracica* Vel. methanol extracts obtained through maceration (MAC), ultrasound-assisted extraction (UAE), and Soxhlet extraction (SOE). The qualitative and quantitative characterization of the extracts was performed using total phenolic and flavonoid content analysis as well as LC–ESI–MS/MS. The biological activities were systematically evaluated, and the key phytochemicals responsible for these effects were statistically identified. Furthermore, *in silico* modeling was employed to elucidate the molecular interactions between major bioactive compounds and targeted enzymes. A thorough literature review revealed that no prior studies have explored these specific aspects of *O. thracica*, highlighting the novelty of this research in providing the first comprehensive phytochemical and bioactivity profile of the species.

## Materials and methods

### Plant material

The aerial parts of *O. thracica* were collected on June 13, 2023, from a roadside location along the Tufanbeyli-Saimbeyli highway (5th km) in Adana, Türkiye (1385 m altitude, 38°12’037"N, 36°12’059"E). No specific permission was required for the collection of plant material from the sampling site. All field studies and sampling procedures complied with relevant international guidelines, including the IUCN Policy Statement on Research Involving Species at Risk of Extinction and the Convention on the Trade in Endangered Species of Wild Fauna and Flora (CITES). The plant was identified by Prof. Dr. Riza Binzet from the Department of Biology at Mersin University, Türkiye, and deposited under the herbarium number Binzet 201803.

### Methanol extraction

Methanol extracts were prepared using three distinct methods [14]. Maceration (MAC) involved soaking 5 g of dried plant material in 100 mL of methanol for 24 hours. Ultrasound-assisted extraction (UAE) was performed in a sonication bath for one hour while maintaining the same solvent (methanol) and sample-to-solvent ratio (1:20) as MAC. Soxhlet extraction (SOE) followed a standardized procedure using methanol for six hours. The resulting extracts were filtered, evaporated under reduced pressure at 40°C, and stored at 4°C in the dark until analysis. Extraction yields for *O. thracica* obtained via maceration, Soxhlet, and ultrasound-assisted methods were 7.61%, 9.40%, and 5.58%, respectively.

### Determination of the phenolic compositions of the extracts

Total phenolic and flavonoid contents were determined spectrophotometrically [15], while phytochemical profiling was conducted using a previously validated LC–ESI–MS/MS method [16], which was previously validated in terms of linearity (R² > 0.99), limits of detection (LOD), limits of quantification (LOQ), and intra-/inter-day precision, as summarized in S1 and S2 Tables of the supplementary file.

### Biological activity

The experimental protocols for antioxidant [15,17–20] and enzyme inhibitory activity assays [21] are described in the supplementary file. IC₅₀ and EC₅₀ values were calculated by nonlinear regression analysis of concentration–response curves using appropriate software, and results were expressed as mean ± standard deviation of triplicate measurements.

### Molecular docking studies of selected phytochemicals

This study investigated the binding interactions of apigenin-7-glucoside, luteolin-7-glucoside, chlorogenic acid, pinoresinol, rosmarinic acid, hyperoside and luteolin —key phytochemicals isolated from *O. thracica*—with human pancreatic α-amylase (AAMY), AChE, and tyrosinase-related protein 1 (TYRP1) using molecular docking simulations.

The primary criterion for selecting these compounds as key phytochemicals in the docking studies was their quantitative abundance in the extracts, with concentrations exceeding 1000 mg/g, indicating that they were major constituents. The aim was to identify the phytochemical(s) primarily responsible for the enzyme inhibitory activity observed in extracts obtained through maceration, Soxhlet extraction, and ultrasound-assisted extraction of *O. thracica*. Our docking simulations provide a systematic and efficient approach to identifying potential inhibitors of AAMY, AChE, and TYRP1—enzymes implicated in diabetes, Alzheimer’s disease, and skin pigmentation disorders, respectively. These findings complement experimental data and support the discovery of bioactive compounds from *O. thracica* with therapeutic potential.

For molecular docking, the crystal structures of human pancreatic α-amylase (PDB ID: 4W93, resolution: 1.35 Å), human acetylcholinesterase (PDB ID: 7XN1, resolution: 2.85 Å), and human tyrosinase-related protein 1 (PDB ID: 5M8O, resolution: 2.50 Å) were retrieved from the RCSB Protein Data Bank (https://www.rcsb.org/). Only the protein components of these crystal structures, excluding any co-crystallized inhibitors, were used as target receptors for docking. To ensure consistency between the *in vitro* and *in silico* parts of the study and to enable a meaningful comparison with the experimental positive controls, the same standard inhibitors used in the enzyme inhibition assays—acarbose for AAMY, galantamine for AChE, and kojic acid for TYRP1—were also docked into the corresponding receptor binding sites. These standard inhibitors were downloaded from the RCSB Protein Data Bank (https://www.rcsb.org/) in PDB format and optimized prior to docking. The phytochemicals apigenin-7-glucoside, luteolin-7-glucoside, chlorogenic acid, pinoresinol, rosmarinic acid, hyperoside and luteolin were manually generated in ChemOffice 19.1 and saved in MOL format for structure-based modeling [22]. The resulting binding energies (docking score: kcal/mol) of the standart inhibitors were used as reference values to compare the docking scores and intermolecular interactions of the selected phytochemicals within the enzyme active sites.

### Protein and ligand preparation

Before docking, the crystal structures of AAMY, AChE, and TYRP1 were refined using Discovery Studio Visualizer [23] by removing water molecules and extraneous ions. The ligands—apigenin-7-glucoside, luteolin-7-glucoside, chlorogenic acid, pinoresinol, rosmarinic acid, hyperoside and luteolin and standart inhibitors (acarbose, galantamine, and kojic acid)—were energetically minimized using Open Babel scripts with the MMFF94 force field and a steepest descent algorithm (convergence = 10⁻⁷) [24]. The MMFF94 force field, parameterized using quantum mechanical and empirical data, effectively models bonding, nonbonded interactions, and torsional effects for small to mid-sized organic molecules [25,26]. The minimized ligand conformations were saved as MOL2 files, while both ligands and processed protein structures were converted to PDBQT format using AutoDock Tools 1.5.7 [27].

Molecular docking was performed using AutoDock Vina 1.2.6 and AutoDock Tools 1.5.7 to predict how apigenin-7-glucoside, luteolin-7-glucoside, chlorogenic acid, pinoresinol, rosmarinic acid, hyperoside and luteolin and standart inhibitors interact within the active sites of AAMY, AChE, and TYRP1 [27, 28]. Polar hydrogens were retained for both proteins and ligands, while nonpolar hydrogens were removed. Kollman charges were assigned to the receptor proteins, and Gasteiger charges were applied to the ligands. Charge imbalances in receptor residues were resolved through uniform redistribution using AutoDock Tools 1.5.7. A semi-flexible docking approach was used, keeping the protein structures rigid while allowing full flexibility for the ligands.

Grid boxes were centered on the binding sites of the co-crystallized inhibitors to ensure optimal docking conditions:

AAMY: 18 Å × 18 Å × 18 Å (center: x = 11.57 Å, y = 86.33 Å, z = 156.33 Å)
AChE: 15 Å × 15 Å × 15 Å (center: x = 48.32 Å, y = –39.99 Å, z = –30.07 Å)
TYRP1: 14 Å × 14 Å × 14 Å (center: x = 121.08 Å, y = 278.18 Å, z = 216.89 Å)

Each docking simulation was repeated 20 times with an *exhaustiveness* level of 50. The resulting ligand conformations were clustered based on geometric similarity and ranked according to docking scores (Δ*G*, kcal/mol). The most negative binding energy conformation for each ligand was selected for further analysis. These top-ranked conformations were then visualized and analyzed for intermolecular nonbonded interactions using Discovery Studio Visualizer [23].

### Statistical analysis

Information regarding the relative antioxidant capacity index (RACI) [29] and statistical methods used in this study can be found in the supplementary file.

### Inclusivity in global research

Additional information regarding the ethical, cultural, and scientific considerations specific to inclusivity in global research is included in the Supporting Information.‌‌

## Results and discussion

### Chemical composition of the extracts

**Fig 1:**
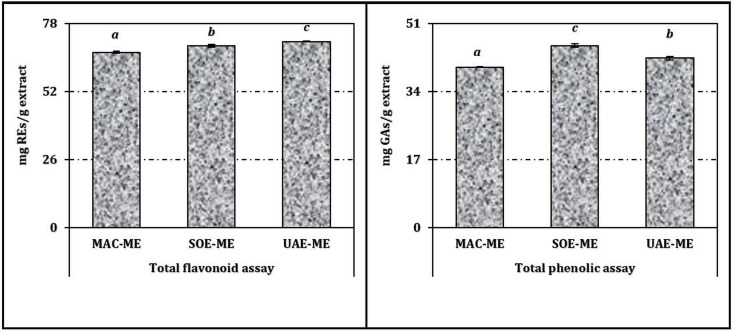
Total flavonoid and phenolic contents of the methanol extracts from *O. thracica.* REs and GAEs: Rutin and gallic acid equivalents, respectively. Values indicated by the same superscripts (a-c) are not different from the honestly significant difference after Tukey’s hoc test at 5% significance level‌‌.

The total phenolic content (TPC) of the methanol extracts obtained from *O. thracica* using different extraction techniques exhibited statistically significant variations (*p* < 0.05) (Fig 1). UAE-ME showed the highest TPC (45.41 mg GAEs/g), followed by SOE-ME (42.33 mg GAEs/g) and MAC-ME (40.06 mg GAEs/g), with statistically significant differences among all extracts, indicating that Soxhlet extraction yielded a moderately improved phenolic recovery compared to maceration but was less effective than ultrasound-assisted extraction.

Similarly, the total flavonoid content (TFC) demonstrated significant differences across extraction methods (*p* < 0.05) (Fig 1). SOE-ME exhibited the highest TFC (71.05 mg REs/g), followed by UAE-ME and MAC-ME, with all differences being statistically significant, suggesting that Soxhlet extraction was the most efficient technique for flavonoid recovery, whereas maceration yielded the lowest flavonoid concentration.

**Fig 2:**
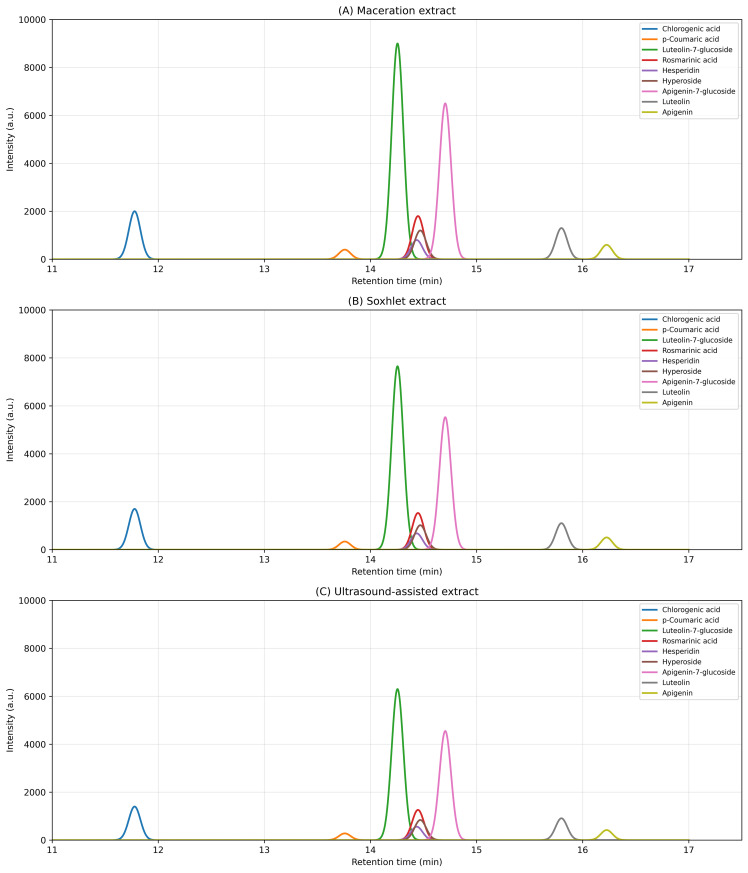
LC–ESI–MS/MS chromatograms of phenolic compounds identified in the extracts. Retention times were assigned based on experimental LC–MS/MS data. Co-eluting compounds (hesperidin, rosmarinic acid and hyperoside) appear at closely overlapping retention times and are distinguished by their specific MRM transitions and color coding.

The LC–ESI–MS/MS analysis revealed significant variations in the concentration of selected phenolic compounds (Table 1, Fig 2). Luteolin-7-glucoside and apigenin-7-glucoside were identified as the dominant flavonoids, whereas chlorogenic acid and rosmarinic acid were the major phenolic acids.

**Table 1:** Concentration (µg/g extract) of selected phenolic compounds in the methanol extracts from *O. thracica.*

Compounds	MAC-ME	SOE-ME	UAE-ME
Luteolin-7-glucoside	9048 ± 27^b^	8798 ± 68^a^	8823 ± 36^a^
Apigenin-7-glucoside	6699 ± 5^b^	5938 ± 43^a^	5860 ± 67^a^
Chlorogenic acid	2021 ± 9^b^	918 ± 4^a^	958 ± 16^a^
Pinoresinol	1797 ± 46^a^	1999 ± 48^b^	2225 ± 13^c^
Rosmarinic acid	1547 ± 12^a^	2218 ± 14^b^	2018 ± 87^b^
Hyperoside	1347 ± 4^c^	1026 ± 2^b^	974 ± 2^a^
Luteolin	1278 ± 15^c^	1209 ± 5^b^	1133 ± 4^a^
Hesperidin	743 ± 10^c^	632 ± 2^b^	588 ± 2^a^
Apigenin	520 ± 5^b^	501 ± 2^a^	499 ± 4^a^
p-Coumaric acid	348 ± 3^b^	319 ± 1^a^	326 ± 9^a^
4-Hydroxybenzoic acid	238 ± 2^b^	207 ± 3^a^	213 ± 2^a^
Syringic acid	206 ± 4^b^	182 ± 8^a^	179 ± 2^a^
Caffeic acid	111 ± 3^c^	64.7 ± 1.3^a^	74.5 ± 1.3^b^
Protocatechuic acid	66.1 ± 0.2^c^	38.3 ± 0.6^a^	42.2 ± 1.4^b^
Ferulic acid	48.8 ± 0.2^b^	42.5 ± 0.1^a^	45.0 ± 2.3^ab^
Vanillin	43.1 ± 2.6^b^	35.4 ± 1.6^a^	31.9 ± 0.8^a^
2,5-Dihydroxybenzoic acid	17.3 ± 0.9^b^	9.82 ± 0.24^a^	9.93 ± 0.27^a^
(-)-Epicatechin	nd	nd	nd
(+)-Catechin	nd	nd	nd
2-Hydroxycinnamic acid	nd	nd	nd
3,4-Dihydroxyphenylacetic acid	nd	nd	nd
3-Hydroxybenzoic acid	nd	nd	nd
Eriodictyol	nd	nd	nd
Gallic acid	nd	nd	nd
Kaempferol	nd	nd	nd
Pyrocatechol	nd	nd	nd
Quercetin	nd	nd	nd
Sinapic acid	nd	nd	nd
Taxifolin	nd	nd	nd
Verbascoside	nd	nd	nd

The values indicated by the same superscripts (a-c) within the same row are not different according to the Tukey’s honestly significant difference post hoc test at 5% significance level. nd: Not detected.

Among the flavonoids, luteolin-7-glucoside was most concentrated in MAC-ME (9048 µg/g), while SOE-ME (8798 µg/g) and UAE-ME (8823 µg/g) exhibited statistically lower but comparable levels (*p* < 0.05). Apigenin-7-glucoside showed a similar distribution pattern, with the highest level in MAC-ME. These results suggest that maceration may be more effective in extracting glycosylated flavonoids compared to the other two methods.

For phenolic acids, chlorogenic acid was most abundant in MAC-ME (2021 µg/g), while SOE-ME and UAE-ME showed significantly lower but comparable levels. Conversely, rosmarinic acid exhibited the highest concentration in SOE-ME (2218 µg/g), followed by UAE-ME (2018 µg/g) and MAC-ME (1547 µg/g). These findings indicate that while maceration favors chlorogenic acid recovery, soxhlet extraction is more effective for rosmarinic acid.

Other flavonoids (e.g., hyperoside, luteolin, hesperidin, and apigenin) generally followed a similar trend, with higher concentrations in MAC-ME. These observations suggest that maceration may be the most effective method for extracting flavonoids, likely due to the prolonged exposure to solvent at ambient temperature, reducing potential thermal degradation.

Among lignans, pinoresinol was detected in the highest concentration in UAE-ME (2225 µg/g), followed by SOE-ME (1999 µg/g) and MAC-ME (1797 µg/g). The UAE method’s superior efficiency in extracting pinoresinol may be attributed to the ultrasonic cavitation process, which facilitates the release of cell wall-bound compounds.

The observed differences in phytochemical profiles reflect the physicochemical characteristics of the extraction techniques. Maceration, performed at ambient temperature, was more effective in extracting the majority of glycosylated flavonoids and some phenolic acids. The lower efficiency of soxhlet extraction for these compounds may be due to their thermal sensitivity and potential degradation under prolonged heating.

These findings indicate that, beyond solvent polarity, parameters such as temperature, extraction time, and mechanical effects play key roles in extraction efficiency.

To our knowledge, this study provides the first comprehensive phytochemical characterization of *O. thracica* and establishes a baseline for future investigations.

A comparison with other *Onosma* species reveals that *O. ambigens* exhibits higher TPC (51.19 mg GAEs/g) and lower TFC (45.39 mg QEs/g) than *O. thracica* [30]. Additionally, *O. oreodoxa* shows an even greater phenolic content (53.76 mg GAEs/g), whereas its flavonoid content (25.29 mg QEs/g) remains significantly lower [31]. These differences suggest species-specific variations in polyphenol biosynthesis, as well as potential influences of extraction methodologies.

Comparison with other Onosma species revealed notable differences in phenolic composition. *O. ambigens* demonstrated remarkably high levels of luteolin-7-glucoside (15756.40 µg/g) and apigenin-7-glucoside (15080.34 µg/g) [30], surpassing those in *O. thracica*. Similarly, *O. bourgaei* and *O. trachytricha* also contained these flavonoids as major constituents [32]. The rosmarinic acid concentration in *O. thracica* was significantly lower than that reported for *O. ambigens* and *O. oreodoxa* [30, 31], highlighting interspecies metabolic differences.

Chlorogenic acid was most abundant in MAC-ME, whereas *O. ambigens* contained a comparable amount (2121.92 µg/g), suggesting consistency in its presence across *Onosma* species [30]. Conversely, the hyperoside content in *O. thracica* was lower than that observed in *O. oreodoxa* (10216.74 µg/g) and *O. gracilis* (7153.26 µg/g) [31], indicating species-dependent variation in flavonol biosynthesis.

The influence of extraction methods is also evident in other *Onosma* species. For example, the methanol extract of *O. ambigens* contained significantly higher amounts of rosmarinic acid (37670.55 µg/g) than that obtained from *O. thracica*, which may be attributed to differences in solvent interaction and plant material composition [30]. Similarly, water extracts of *O. heterophyllum* demonstrated rich phenolic and flavonoid profiles, with protocatechuic acid, syringic acid, and *p*-coumaric acid correlating with antioxidant activity [21]. These findings suggest that solvent polarity and extraction duration play crucial roles in the recovery of bioactive constituents.

### Antioxidant activity of the extracts

**Fig 3:**
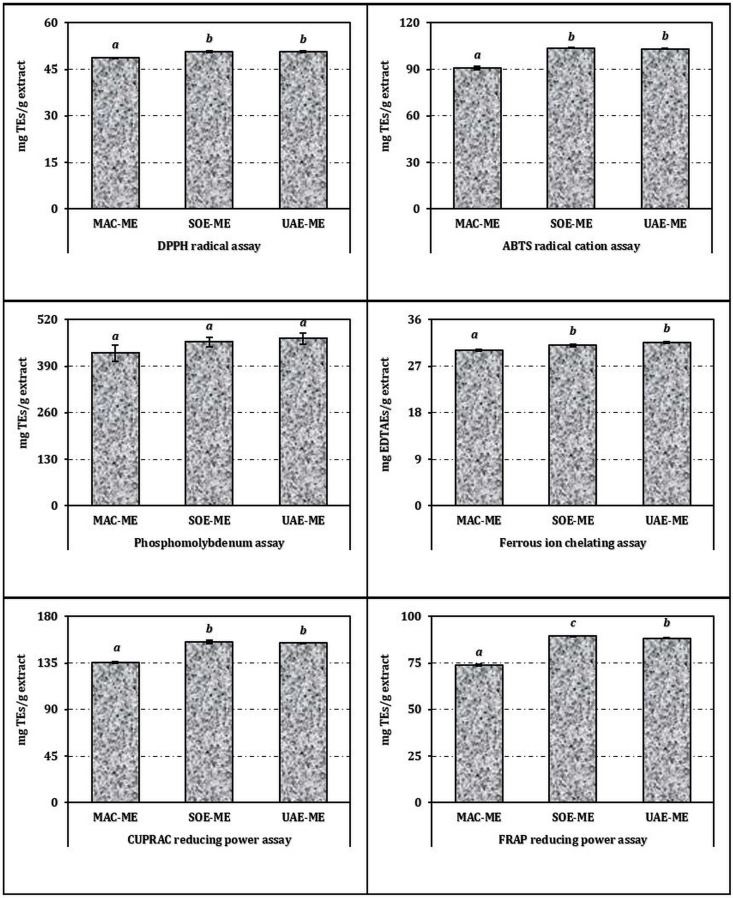
Antioxidant activity of the methanol extracts from *O. thracica.* Values indicated by the same superscripts (a-c) are not different from the honestly significant difference after Tukey’s hoc test at 5% significance level.

The antioxidant activities of the methanol extracts obtained from *O. thracica* using maceration, ultrasound-assisted extraction, and soxhlet extraction were assessed through six different *in vitro* assays, as summarized in Table 2 and Fig 3 (*p* < 0.05).

**Table 2:** Antioxidant activities of the methanol extracts from *O. thracica.*

Assays	MAC-ME	SOE-ME	UAE-ME	Trolox	EDTA
Phosphomolybdenum (EC_50_: mg/mL)	1.15 ± 0.06^b^	1.07 ± 0.03^b^	1.05 ± 0.03^b^	0.50 ± 0.02^a^	–
CUPRAC reducing power (EC_50_: mg/mL)	0.81 ± 0.01^c^	0.71 ± 0.01^b^	0.71 ± 0.001^b^	0.12 ± 0.02^a^	–
FRAP reducing power (EC_50_: mg/mL)	0.60 ± 0.002^d^	0.49 ± 0.001^b^	0.50 ± 0.001^c^	0.044 ± 0.001^a^	–
DPPH radical scavenging (IC_50_: mg/mL)	1.17 ± 0.001^c^	1.12 ± 0.01^b^	1.13 ± 0.01^b^	0.057 ± 0.002^a^	–
ABTS radical cation scavenging (IC_50_: mg/mL)	1.21 ± 0.02^c^	1.06 ± 0.001^b^	1.07 ± 0.003^b^	0.11 ± 0.01^a^	–
Ferrous ion chelating (IC_50_: mg/mL)	1.10 ± 0.004^c^	1.06 ± 0.01^b^	1.05 ± 0.01^b^	–	0.032 ± 0.002^a^

TEs and EDTAEs mean trolox and ethylenediaminetetraacetic acid (disodium salt) equivalents, respectively. Values indicated by the same superscripts (a-d) within the same row are not different from the honestly significant difference after Tukey’s hoc test at 5% significance level.

In the phosphomolybdenum assay, which evaluates total antioxidant capacity, all extracts exhibited comparable EC₅₀ values, with UAE-ME (1.05 mg/mL) showing the lowest value, followed closely by SOE-ME (1.07 mg/mL) and MAC-ME (1.15 mg/mL). All extracts were less active than trolox (0.50 mg/mL).

Reducing power assays (CUPRAC and FRAP) showed a similar pattern. For CUPRAC, SOE-ME and UAE-ME exhibited the highest reducing capacities (EC₅₀: 0.71 mg/mL), significantly outperforming MAC-ME (0.81 mg/mL, *p* < 0.05). A comparable pattern was observed in the FRAP assay, where SOE-ME (0.49 mg/mL) and UAE-ME (0.50 mg/mL) exhibited stronger ferric-reducing abilities than MAC-ME (0.60 mg/mL, *p* < 0.05), but all extracts remained less effective than trolox (EC₅₀: 0.044 mg/mL).

In radical scavenging assays (DPPH and ABTS), SOE-ME (IC₅₀: 1.12 mg/mL) and UAE-ME (IC₅₀: 1.13 mg/mL) had significantly stronger DPPH scavenging capacities than MAC-ME (IC₅₀: 1.17 mg/mL, *p* < 0.05). Similarly, in the ABTS assay, SOE-ME (IC₅₀: 1.06 mg/mL) and UAE-ME (IC₅₀: 1.07 mg/mL) exhibited greater radical scavenging efficiency compared to MAC-ME (IC₅₀: 1.21 mg/mL, *p* < 0.05). All extracts were again less active than trolox (DPPH: 0.057 mg/mL; ABTS: 0.11 mg/mL).

The ferrous ion chelating activity of the extracts followed a similar pattern, with UAE-ME (IC₅₀: 1.05 mg/mL) and SOE-ME (IC₅₀: 1.06 mg/mL) exhibiting stronger metal-chelating abilities than MAC-ME (IC₅₀: 1.10 mg/mL, *p* < 0.05). However, all extracts were markedly weaker than EDTA (IC₅₀: 0.032 mg/mL).

Overall, SOE-ME and UAE-ME consistently showed higher antioxidant activity than MAC-ME across all assays. This difference is likely related to improved extraction efficiency of phenolic compounds under Soxhlet and ultrasound-assisted conditions.

**Fig 4:**
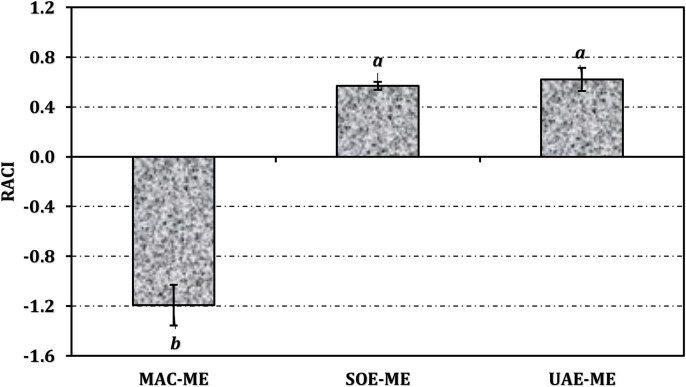
Relative antioxidant capacity index of the methanol extracts from *O. thracica.*

The RACI scores revealed a clear ranking among the extracts (Fig 4). UAE-ME (0.62) and SOE-ME (0.57) exhibited statistically comparable antioxidant capacities, significantly surpassing MAC-ME (−1.19, *p* < 0.05).

These results confirm that UAE-ME and SOE-ME possess higher overall antioxidant potential.

This study provides the first comprehensive evaluation of the antioxidant activity of *O. thracica*. UAE and SOE methods yielded extracts with superior antioxidant properties compared to MAC.

Comparison with other *Onosma* species revealed species-dependent variations in antioxidant activity. Previous studies reported higher antioxidant capacity in some *Onosma* species, particularly those rich in phenolic compounds such as apigenin, luteolin, and rosmarinic acid [33]. The lower EC₅₀ values of DPPH and ABTS assays for *O. lycaonica* suggest that it has a more potent antioxidant profile than *O. thracica*.

Other species such as *O. mutabilis* also exhibited notable antioxidant activity [34]. These values suggest that while *O. mutabilis* has lower radical scavenging activity compared to *O. thracica*, it still possesses notable antioxidant capacity.

Further comparisons with *O. trapezuntea* and *O. rigidum* [35] revealed species-specific differences in antioxidant activity, with chlorogenic acid, rosmarinic acid, and hyperoside contributing to their respective activities. While *O. thracica* demonstrated higher reducing power than some *Onosma* species, its metal chelating ability was relatively weaker compared to *O. rigidum*.

The antioxidant potential of *O. thracica* is likely influenced by its phytochemical composition, particularly phenolic acids and flavonoids. Previous studies on *Onosma* species have reported the presence of apigenin-7-glucoside, luteolin, and luteolin-7-glucoside, compounds known for their free radical scavenging and anti-inflammatory properties [36, 37]. It is plausible that similar bioactive compounds in *O. thracica* contribute to its observed antioxidant effects.

This study provides the first experimental evidence of the antioxidant capacity of *O. thracica*, expanding the knowledge on the genus *Onosma*. The findings suggest that UAE and SOE methods enhance the extraction of bioactive compounds, leading to higher antioxidant activity. While the antioxidant potential of *O. thracica* is moderate compared to other *Onosma* species, its unique phytochemical profile warrants further investigation.

Future research should focus on phytochemical characterization of *O. thracica* to identify key compounds responsible for its antioxidant activity. Additionally, *in vivo* studies are necessary to evaluate the bioavailability and pharmacological relevance of these compounds. Overall, this study underscores the potential of *O. thracica* as a source of natural antioxidants and provides a foundation for further research into its therapeutic applications.

### Enzyme inhibitory activity of the extracts

**Fig 5:**
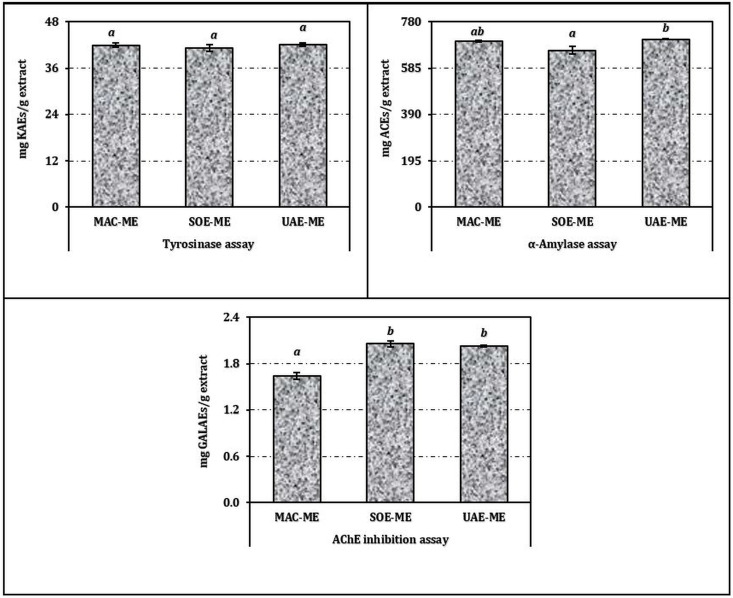
Enzyme inhibition activity of the methanol extracts from *O. thracica.* Values indicated by the same superscripts (a-c) are not different from the honestly significant difference after Tukey’s hoc test at 5% significance level.

The enzyme inhibition activities of methanol extracts from *O. thracica* obtained via maceration, ultrasound-assisted extraction, and Soxhlet extraction were evaluated against AChE, tyrosinase, and α-amylase. The results are summarized in Table 3 and Fig 5.

**Table 3:** Enzyme inhibition activity of the methanol extracts from *O.thracica.*

Samples	AChE inhibition (IC_50_: mg/mL)	Tyrosinase inhibition (IC_50_: mg/mL)	α-Amylase inhibition (IC_50_: mg/mL)
MAC-ME	1.67 ± 0.05^c^	1.93 ± 0.02^b^	1.12 ± 0.01^bc^
SOE-ME	1.33 ± 0.02^b^	1.97 ± 0.04^b^	1.19 ± 0.03^c^
UAE-ME	1.35 ± 0.01^b^	1.92 ± 0.02^b^	1.11 ± 0.002^b^
Galantamine	0.0026 ± 0.0002^a^	–	–
Kojic acid	–	0.082 ± 0.001^a^	–
Acarbose	–	–	0.80 ± 0.02^a^

ACEs, GALAEs and KAEs mean acarbose, galantamine and kojic acid equivalents, respectively. na: not active. Values indicated by the same superscripts (a-c) within the same column are not different from the honestly significant difference after Tukey’s hoc test at 5% significance level.

Among the extracts, SOE-ME exhibited the highest AChE inhibitory activity (IC_50_: 1.33 mg/mL), followed closely by UAE-ME (IC_50_: 1.35 mg/mL). MAC-ME demonstrated significantly lower inhibition (IC_50_: 1.67 mg/mL). Despite these variations, all extracts exhibited significantly weaker inhibition compared to the reference inhibitor, galantamine (IC_50_: 0.0026 mg/mL), indicating moderate but notable inhibitory potential.

The extracts displayed relatively similar tyrosinase inhibition profiles, with IC_50_ values ranging between 1.92 mg/mL (UAE-ME) and 1.97 mg/mL (SOE-ME). Statistical analysis revealed no significant differences among the three extracts (*p* > 0.05). Nevertheless, the inhibitory potency remained markedly lower than that of kojic acid (IC_50_: 0.082 mg/mL), a well-known tyrosinase inhibitor.

The UAE-ME extract demonstrated the highest α-amylase inhibitory activity (IC_50_: 1.11 mg/mL), significantly outperforming SOE-ME (IC_50_: 1.19 mg/mL) (*p* < 0.05). However, all extracts were less effective compared to acarbose (IC_50_: 0.80 mg/mL), suggesting moderate inhibitory potential.

Overall, SOE-ME was more effective against AChE, whereas UAE-ME showed higher α-amylase inhibition, while tyrosinase inhibition remained comparable across extracts. These findings indicate that extraction method influences enzyme inhibitory activity.

Compared to other *Onosma* species, *O. heterophyllum* methanolic extract has been reported to exhibit higher AChE inhibition (79.18 µmol GALAEs/g dry plant) [21]. Similarly, *O. tauricum* var. *tauricum* methanolic extract also demonstrated considerable AChE inhibition (54.62 µmol GALAEs/g dry plant) [38]. The relatively lower inhibitory potency of *O. thracica* extracts may be attributed to phytochemical composition differences. Specifically, *O. ambigens* and *O. gracilis* have also shown certain levels of AChE inhibition, although their activity levels vary [30, 31]. These variations highlight interspecies differences in bioactive compound profiles and the impact of factors such as geographical origin, environmental conditions, and extraction techniques.

Regarding tyrosinase inhibition, *O. thracica* extracts exhibited similar activities, with no statistically significant differences observed among extraction methods. This suggests that bioactive compounds responsible for tyrosinase inhibition are extracted at comparable rates regardless of the technique employed. Comparatively, *O. heterophyllum* (112.44 µmol KAEs/g dry plant) and *O. tauricum* var. *tauricum* (90.66 µmol KAEs/g dry plant) displayed higher tyrosinase inhibition [21, 38]. The relatively lower activity of *O. thracica* may be attributed to the lower concentration or structural variations of its tyrosinase-inhibitory flavonoid compounds.

A noteworthy finding concerns the relationship between flavonoid glycosides and tyrosinase inhibition, as discrepancies exist between the current study and previous reports. Fan et al. noted that flavonoid aglycones exhibit higher tyrosinase inhibition compared to their glycosylated forms [39], whereas this study found that glycosylated flavonoids in *O. thracica* displayed considerable inhibitory activity. This discrepancy underscores the complexity of flavonoid-enzyme interactions and highlights the need for further mechanistic investigations.

Literature reports indicate that *O. heterophyllum* methanolic extract exhibited stronger α-amylase inhibition (10.42 µmol ACEs/g dry plant), while *O. ambigens* showed higher activity (2.64 mg/mL, ethyl acetate extract) [21, 30]. Flavonoid glycosides, particularly luteolin-7-glucoside and apigenin-7-glucoside, have been reported to play a role in α-amylase inhibition [40]. The moderate α-amylase inhibition observed in *O. thracica* may be due to differences in the concentration or structural properties of these inhibitory compounds.

Recent molecular docking studies have also highlighted the potential of flavonoid glycosides in enzyme inhibition. İstifli and Sarıkürkcü investigated the inhibitory effects of apigenin-7-glucoside and luteolin-7-glucoside against AChE, BChE, amyloid precursor protein (APP), and beta-amyloid (Aβ) peptides. Their findings demonstrated that apigenin-7-glucoside and luteolin-7-glucoside exhibited strong binding affinities for AChE and BChE, surpassing even rivastigmine. Furthermore, their interaction with APP and Aβ peptides, though moderate, suggested a multi-targeted therapeutic potential in Alzheimer’s disease [41]. These findings reinforce the significance of flavonoid glycosides in neurodegenerative disease research and suggest that similar compounds in *O. thracica* may contribute to its AChE inhibitory potential.

Overall, this study provides novel and valuable insights into the enzyme inhibitory potential of *O. thracica* extracts. The data reveal that *O. thracica* exerts moderate inhibition on AChE, tyrosinase, and α-amylase enzymes. Compared to other *Onosma* species, *O. thracica* appears to possess a distinct phytochemical composition. Future research should focus on isolating individual active compounds and elucidating their mechanisms to fully uncover the therapeutic potential of this species.

### Correlations among phenolic compounds and assays

The correlation analysis revealed strong relationships between the total phenolic and flavonoid contents and various antioxidant and enzyme inhibitory activities (Table 4). Both total phenolic and flavonoid contents showed strong positive correlations with antioxidant assays, indicating their major contribution to radical scavenging and reducing capacity. Ferrous ion chelating activity was particularly associated with flavonoid content.

**Table 4:** Correlations among phenolic compounds and assays.

	TAP	DPPH	ABTS	CUPRAC	FRAP	FICA	AChEIA	TIA	AAIA
DPPH radical	0.854								
ABTS radical cation	0.810	0.977							
CUPRAC reducing power	0.816	0.989	0.995						
FRAP reducing power	0.766	0.979	0.994	0.993					
Ferrous ion chelating	0.685	0.846	0.887	0.873	0.888				
AChE inhibition	0.746	0.979	0.979	0.990	0.993	0.883			
Tyrosinase inhibition	0.311	−0.137	−0.259	−0.220	−0.304	−0.160	−0.279		
α-Amylase inhibition	−0.126	−0.384	−0.350	−0.386	−0.379	0.065	−0.398	0.477	1.000
Total flavonoid	0.812	0.870	0.896	0.881	0.881	0.976	0.864	−0.012	0.095
Total phenolic	0.532	0.817	0.833	0.830	0.850	0.538	0.831	−0.574	−0.750
Luteolin-7-glucoside	−0.795	−0.985	−0.941	−0.968	−0.957	−0.797	−0.975	0.144	0.468
Apigenin-7-glucoside	−0.838	−0.988	−0.982	−0.986	−0.984	−0.918	−0.981	0.143	0.260
Chlorogenic acid	−0.787	−0.984	−0.995	−0.995	−0.999	−0.897	−0.993	0.265	0.352
Pinoresinol	0.777	0.792	0.823	0.804	0.802	0.962	0.785	0.071	0.238
Rosmarinic acid	0.721	0.955	0.953	0.955	0.966	0.749	0.954	−0.385	−0.561
Hyperoside	−0.815	−0.970	−0.981	−0.980	−0.980	−0.948	−0.975	0.164	0.205
Luteolin	−0.800	−0.808	−0.827	−0.816	−0.807	−0.957	−0.797	−0.114	−0.214
Hesperidin	−0.843	−0.934	−0.947	−0.942	−0.937	−0.967	−0.928	0.050	0.054
Apigenin	−0.828	−0.929	−0.969	−0.965	−0.946	−0.898	−0.934	0.140	0.237
p-Coumaric acid	−0.746	−0.956	−0.915	−0.927	−0.936	−0.723	−0.934	0.272	0.526
4-Hydroxybenzoic acid	−0.723	−0.943	−0.986	−0.974	−0.984	−0.840	−0.963	0.409	0.437
Syringic acid	−0.780	−0.904	−0.949	−0.919	−0.937	−0.916	−0.898	0.260	0.124

Data show the Pearson Correlation Coefﬁcients between the parameters. TAP: total antioxidant activity by phosphomolybdenum method. AAIA, AChEIA, and TIA: α-amylase, acetylcholinesterase, and tyrosinase inhibition activities, respectively. ABTS and DPPH: ABTS and DPPH radical scavenging activities, respectively. CUPRAC and FRAP: CUPRAC and FRAP reducing power potential; respectively. FICA: Ferrous ion chelating activity

Among the individual phytochemicals, pinoresinol and rosmarinic acid showed notable positive correlations with antioxidant assays, reinforcing its known antioxidant potential.

Some compounds, including chlorogenic acid and certain flavonoid glycosides, exhibited negative correlations, suggesting assay-dependent or compound-specific effects.

Regarding enzyme inhibition activities, AChE inhibition was positively associated with antioxidant activity, whereas tyrosinase and α-amylase inhibition showed weaker or inverse relationships.

Overall, the results indicate that phytochemical composition differentially influences antioxidant and enzyme inhibitory responses.

### *In silico* analyses

**Fig 6:**
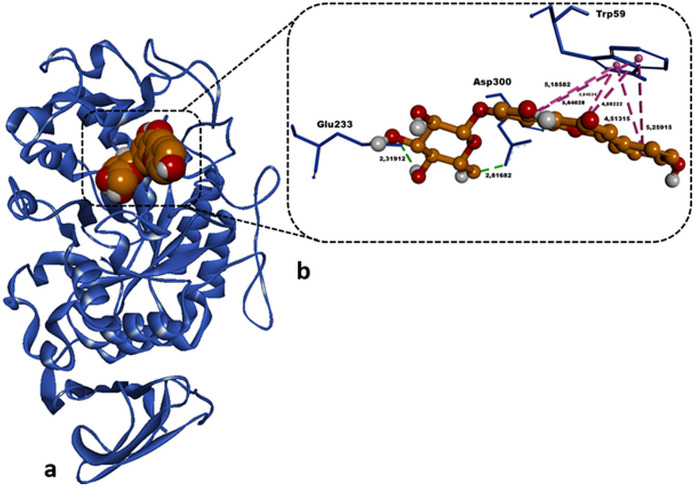
Predicted docking pose of apigenin-7-glucoside within the catalytic site of AAMY. **(a)** Three-dimensional structural representation of the AAMY–apigenin 7-glucoside complex, where AAMY is depicted as a ribbon model, and apigenin-7-glucoside is displayed in Corey–Pauling–Koltun (CPK) representation. **(b)** Magnified view of the ligand binding interface, illustrating critical molecular interactions stabilizing apigenin-7-glucoside within the active site. Hydrogen bonding interactions are denoted by green dashed lines, while hydrophobic contacts are represented by dark purple dashed lines. Non-bonded interaction distances (Å) are annotated in bold black text. Structural analysis and molecular visualization were conducted using DS Studio v16‌‌.

**Fig 7:**
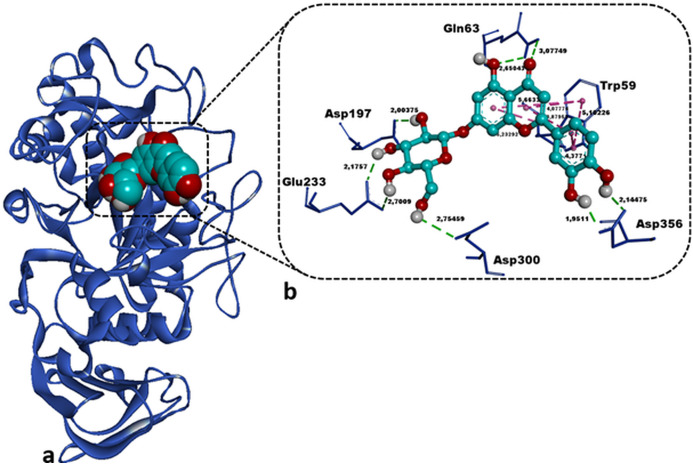
Predicted docking pose of luteolin-7-glucoside within the catalytic site of AAMY. **(a)** Three-dimensional structural representation of the AAMY–luteolin-7-glucoside complex, where AAMY is depicted as a ribbon model, and luteolin-7-glucoside is displayed in Corey–Pauling–Koltun (CPK) representation. **(b)** Magnified view of the ligand binding interface, illustrating critical molecular interactions stabilizing luteolin-7-glucoside within the active site. Hydrogen bonding interactions are denoted by green dashed lines, while hydrophobic contacts are represented by dark purple dashed lines. Non-bonded interaction distances (Å) are annotated in bold black text. Structural analysis and molecular visualization were conducted using DS Studio v16.

**Fig 8:**
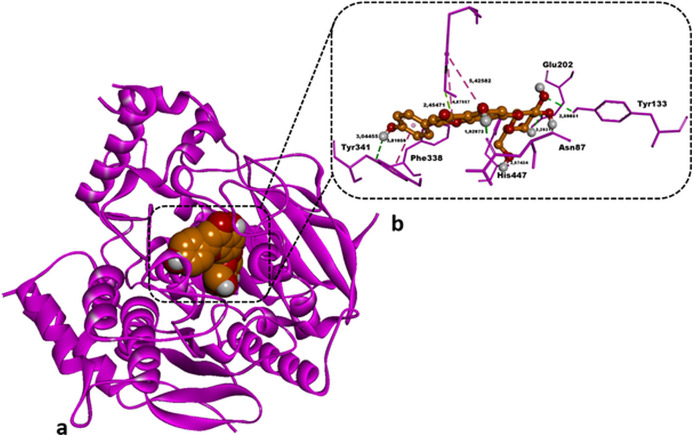
Predicted docking pose of apigenin-7-glucoside within the catalytic site of AChE. **(a)** Three-dimensional structural representation of the AChE–apigenin-7-glucoside complex, where AChE is depicted as a ribbon model, and apigenin-7-glucoside is displayed in Corey–Pauling–Koltun (CPK) representation. **(b)** Magnified view of the ligand binding interface, illustrating critical molecular interactions stabilizing apigenin-7-glucoside within the active site. Hydrogen bonding interactions are denoted by green dashed lines, while hydrophobic contacts are represented by dark purple dashed lines, and the bright lime green color dashed line represents pi-lone pair interaction. Non-bonded interaction distances (Å) are annotated in bold black text. Structural analysis and molecular visualization were conducted using DS Studio v16.

**Fig 9:**
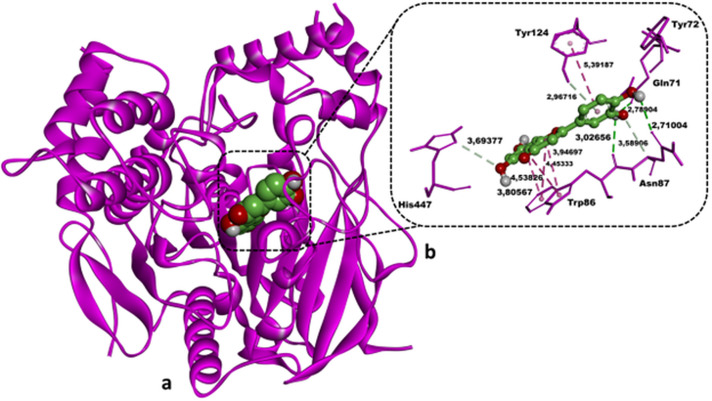
Predicted docking pose of luteolin within the catalytic site of AChE. **(a)** Three-dimensional structural representation of the AChE–luteolin complex, where AChE is depicted as a ribbon model, and luteolin is displayed in Corey–Pauling–Koltun (CPK) representation. **(b)** Magnified view of the ligand binding interface, illustrating critical molecular interactions stabilizing luteolin within the active site. Hydrogen bonding interactions are denoted by green and light pastel green dashed lines, hydrophobic contacts are represented by dark and light purple dashed lines. Non-bonded interaction distances (Å) are annotated in bold black text. Structural analysis and molecular visualization were conducted using DS Studio v16.

**Fig 10:**
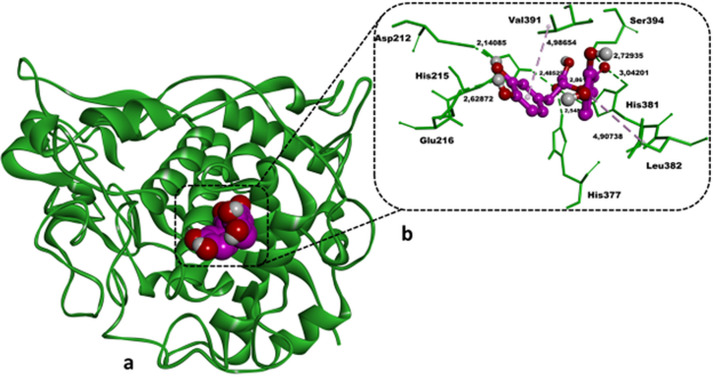
Predicted docking pose of rosmarinic acid within the catalytic site of human tyrosinase-related protein 1 (TYRP1). **(a)** Three-dimensional structural representation of the TYRP1–rosmarinic acid complex, where TYRP1 is depicted as a ribbon model, and rosmarinic acid is displayed in Corey–Pauling–Koltun (CPK) representation. **(b)** Magnified view of the ligand binding interface, illustrating critical molecular interactions stabilizing rosmarinic acid within the active site. Hydrogen bonding interactions are denoted by green dashed lines, hydrophobic contacts are represented by light and dark purple dashed lines. Non-bonded interaction distances (Å) are annotated in bold black text. Structural analysis and molecular visualization were conducted using DS Studio v16.

The docking results are summarized in Table 5–7 and illustrated in Figs 6–10, with additional interaction details of the reference controls provided in S1–S3 Figs. Among the major compounds tested, apigenin-7-glucoside and luteolin-7-glucoside exhibited the most favorable binding affinities toward AAMY, apigenin-7-glucoside and luteolin toward AChE, and rosmarinic acid against TYRP1, supporting their potential contribution to the experimentally observed enzyme inhibitory activities. In the AAMY dockings, apigenin-7-glucoside and luteolin-7-glucoside showed docking scores significantly higher than the reference control acarbose, indicating strong interaction potential (Table 5; S1 Fig). Their interactions involved mainly hydrogen (H) bonding and hydrophobic contacts within the AAMY catalytic site (Figs 6 and7). The identification of apigenin-7-glucoside and luteolin-7-glucoside as the most favorable AAMY-interacting phytochemicals in the present docking analysis is consistent with a previous *in vitro* study showing that both compounds inhibit pancreatic α-amylase, with reported IC₅₀ values of 0.17 and 0.28 mM, respectively [42]. This finding is particularly relevant because it indicates that these glycosylated flavones are not only structurally compatible with the AAMY catalytic region *in silico*, but also possess measurable α-amylase inhibitory activity in enzyme-based assays. In addition, earlier evidence showing flavonoid-mediated inhibition of carbohydrate-hydrolyzing enzymes, including the reported inhibitory effect of luteolin-7-O-glucoside on α-amylase, provides further contextual support for the possible contribution of these glycosylated flavones to the experimentally observed AAMY inhibitory activity [43].

**Table 5:** Overview of docking interactions between key *O. thracica* phytochemicals and standart inhibitors with AAMY including docking scores (kcal/mol) and ligand–residue contact profiles within the enzymes’ active sites.

Compound	Molecular weight (g/mol)	Target protein	Docking score (Δ*G*: kcal/mol)	Classical H-bond	Non-classical H-bond (carbon-hydrogen, pi-donor)	Hydrophobic contact	Electrostatic (pi-cation, pi-anion)	Other, (pi-lone pair)
						π-sigma, alkyl, π-alkyl and π-π stacked, and π-π T-shaped interaction		
Acarbose	645.61	AAMY	−8.20	Gln63 (2.96 Å, 2.99 Å), Thr163 (3.04 Å), Asp197 (3.06 Å), Lys200 (2.21 Å), Glu233 (2.76 Å), Glu240 (2.09 Å), Ala307 (2.94 Å)	Trp59 (3.38 Å)	Leu165 (4.21 Å)	–	–
Apigenin-7-glucoside	432.10	AAMY	−9.50	Glu233 (2.32 Å), Asp300 (2.82 Å)	–	Trp59 (3.85 Å, 4.08 Å, 4.51 Å, 5.19 Å, 5.26 Å, 5.64 Å)	–	–
Luteolin-7-glucoside	448.10	AAMY	−9.46	Gln63 (2.65 Å, 3.08 Å), Asp197 (2.00 Å), Glu233 (2.18 Å, 2.70 Å), Asp300 (2.75 Å), Asp356 (1.95 Å, 2.14 Å)	–	Trp59 (3.88 Å, 4.08 Å, 4.38 Å, 5.23 Å, 5.66 Å, 5.10 Å)	–	–
Chlorogenic acid	354.10	AAMY	−7.66	Gln63 (2.60 Å), His101 (2.80 Å), *Glu233* (2.51 Å, 2.54 Å)	–	*Trp59* (4.07 Å, 4.12 Å)	–	–
Hyperoside	464.10	AAMY	−7.72	Asp197 (2.22 Å), Ala198 (2.92 Å), His201 (2.19 Å), Glu233 (2.26 Å), Asp300 (2.60 Å), His305 (2.50 Å, 2.71 Å, 2.83 Å)	–	–	–	–
Luteolin	286.05	AAMY	−8.75	Gln63 (2.51 Å), Asp197 (1.82 Å)	–	Trp59 (3.90 Å, 4.19 Å, 5.23 Å), Tyr62 (4.09 Å)	–	–
Pinoresinol	358.14	AAMY	−8.45	Asp197 (1.88 Å)	–	Trp59 (4.43 Å), Tyr62 (4.51 Å), Leu162 (5.03 Å), Leu165 (5.38 Å), Ala198 (3.90 Å)	–	–
Rosmarinic acid	360.08	AAMY	−8.34	Gln63 (2.51 Å), Thr163 (1.98 Å), Asp197 (1.99 Å, 2.72 Å), Ala198 (3.02 Å), Glu233 (2.49 Å)	Ala198 (3.27 Å)	Trp58 (5.10 Å), Tyr62 (4.88 Å), Leu162 (3.56 Å), Ala198 (4.76 Å)	–	–

AAMY: Human pancreatic α-amylase (PDB ID: 4W93).

Δ*G*: Docking score (kcal/mol) of the top-ranked binding pose of the ligand.

Residues highlighted in italics and underlined indicate critical interactions between the primary phytochemicals and receptor active sites, which are also contacted by the reference compound in docking. This agreement supports the robustness of the conformational sampling algorithm implemented in AutoDock Vina 1.2.6.

**Table 6:** Overview of docking interactions between key *O. thracica* phytochemicals and standart inhibitors with AChE including docking scores (kcal/mol) and ligand–residue contact profiles within the enzymes’ active sites.

Compound	Molecular weight (g/mol)	Target protein	Docking score (Δ*G*: kcal/mol)	Classical H-bond	Non-classical H-bond (carbon-hydrogen, pi-donor)	Hydrophobic contact	Electrostatic (pi-cation, pi-anion)	Other, (pi-lone pair)
						π-sigma, alkyl, π-alkyl and π-π stacked, and π-π T-shaped interaction		
Galantamine	287.15	AChE	−9.54	–	Asp74 (3.62 Å), Asn87 (3.55 Å), Ser125 (3.01 Å), Tyr337 (3.00 Å), His447 (2.99 Å), Tyr449 (3.71 Å)	Trp86 (4.10 Å, 4.46 Å, 4.57 Å, 5.33 Å, 5.49 Å, 5.50 Å), Tyr337 (3.78 Å, 4.66 Å), Trp439 (4.11 Å, 4.56 Å), Tyr449 (4.84 Å)	–	–
Apigenin-7-glucoside	432.10	AChE	−10.82	Asn87 (1.93 Å), Glu202 (2.29 Å), His447 (2.57 Å), Phe338 (3.04 Å), Tyr133 (2.59 Å)	–	Tyr124 (4.87 Å, 5.43 Å), Tyr341 (3.81 Å)	–	Tyr124 (2.45 Å)
Luteolin-7-glucoside	448.10	AChE	−8.39	Tyr133 (2.44 Å), Glu202 (1.95 Å), Phe295 (1.96 Å, 2.53 Å)	His447 (3.11 Å), Gly448 (3.59 Å)	Tyr124 (4.86 Å, 5.28 Å), Tyr341 (5.38 Å)	–	Tyr124 (2.21 Å)
Chlorogenic acid	354.10	AChE	−9.38	Tyr124 (2.22 Å, 2.55 Å), *Tyr337* (2.32 Å), His447 (2.60 Å)	–	Tyr124 (5.57 Å)	–	–
Hyperoside	464.10	AChE	−8.55	*Asp74* (2.25 Å), *Trp86* (2.12 Å), *Asn87* (2.59 Å), Tyr124 (1.54 Å, 2.91 Å), Glu202 (2.48 Å), *Tyr337* (2.13 Å)	Tyr341 (2.23 Å)	Trp86 (4.02 Å, 4.29 Å, 4.61 Å, 4.86 Å), Tyr124 (5.32 Å)	–	–
Luteolin	286.05	AChE	−10.56	Gln71 (2.79 Å), Trp86 (3.03 Å), Asn87 (2.71 Å)	Asn87 (3.59 Å), Tyr124 (2.97 Å), His447 (3.69 Å)	Trp86 (3.81 Å, 3.95 Å, 4.45 Å, 4.54 Å), Tyr124 (5.39 Å)	–	–
Pinoresinol	358.14	AChE	−6.46	Phe295 (2.03 Å), Phe338 (2.54 Å)	Glu202 (3.17 Å), Arg296 (3.38 Å)	Trp86 (3.75 Å, 4.38 Å, 5.07 Å), Trp286 (4.89 Å), Phe297 (4.26 Å), Phe338 (4.66 Å), Tyr341 (4.50 Å)	–	–
Rosmarinic acid	360.08	AChE	−10.05	Gln71 (3.08 Å), Tyr72 (2.04 Å), Asp74 (2.56 Å), Tyr124 (2.60 Å), Tyr133 (2.90 Å), Tyr337 (2.27 Å)	Tyr124 (3.03 Å)	Trp86 (3.79 Å, 4.36 Å), Tyr124 (5.49 Å)	–	–

AChE: Human acetylcholinesterase (PDB ID: 7XN1).

Δ*G*: Docking score (kcal/mol) of the top-ranked binding pose of the ligand.

Residues highlighted in italics and underlined indicate critical interactions between the primary phytochemicals and receptor active sites, which are also contacted by the reference compound in docking. This agreement supports the robustness of the conformational sampling algorithm implemented in AutoDock Vina 1.2.6.

**Table 7:** Overview of docking interactions between key *O. thracica* phytochemicals and standart inhibitors with TYRP1 including docking scores (kcal/mol) and ligand–residue contact profiles within the enzymes’ active sites.

Compound	Molecular weight (g/mol)	Target protein	Docking score (Δ*G*: kcal/mol)	Classical H-bond	Non-classical H-bond (carbon-hydrogen, pi-donor)	Hydrophobic contact	Electrostatic (pi-cation, pi-anion)	Other, (pi-lone pair)
						π-sigma, alkyl, π-alkyl and π-π stacked, and π-π T-shaped interaction		
Kojic acid	142.11	TYRP1	−5.58	–	–	*His381* (3.62 Å), Val391 (4.89 Å)	His381 (4.01 Å)	–
Apigenin-7-glucoside	432.10	TYRP1	−7.14	Arg321 (2.68 Å), Ser394 (2.33 Å), Val391 (2.33 Å)	Gly389 (3.53 Å), Gln390 (3.39 Å)	His381 (3.97 Å), Leu382 (4.67 Å, 5.03 Å), Val391 (4.62 Å)	His381 (4.11 Å)	–
Luteolin-7-glucoside	448.10	TYRP1	−7.40	Arg321 (2.51 Å), Gly388 (2.71 Å), Ser394 (2.05 Å)	–	His381 (4.18 Å), Val391 (4.48 Å)	–	–
Chlorogenic acid	354.10	TYRP1	−6.46	Asn318 (2.38 Å), Gly388 (2.85 Å), Gly389 (2.54 Å), His381 (2.79 Å), Ser394 (1.85 Å, 2.42 Å)	–	Gln390 (4.62 Å), Val391 (4.23 Å, 4.62 Å)	–	–
Hyperoside	464.10	TYRP1	−8.04	*His381* (2.76 Å), *Val391* (2.02 Å)	–	Leu382 (3.96 Å, 5.44 Å)	–	–
Luteolin	286.05	TYRP1	−7.03	Glu216 (2.20 Å)	–	Phe362 (4.87 Å, 5.37 Å), His381 (4.27 Å), Val391 (4.57 Å)	–	–
Pinoresinol	358.14	TYRP1	−5.69	–	His215 (3.52 Å), His377 (3.17 Å)	His192 (5.34 Å), His215 (4.38 Å), His377 (5.09 Å), His381 (3.85 Å), Leu382 (4.71 Å), Val391 (4.86 Å, 5.15 Å)	–	–
Rosmarinic acid	360.08	TYRP1	−8.60	Asp212 (2.14 Å), His215 (2.49 Å), Glu216 (2.63 Å), His377 (2.55 Å), His381 (2.86 Å, 3.04 Å), Ser394 (2.73 Å)	–	Leu382 (4.91 Å), Val391 (4.99 Å)	–	–

TYRP1: Human tyrosinase-related protein 1 (PDB ID: 5M8O).

Δ*G*: Docking score (kcal/mol) of the top-ranked binding pose of the ligand.

Residues highlighted in italics and underlined indicate critical interactions between the primary phytochemicals and receptor active sites, which are also contacted by the reference compound in docking. This agreement supports the robustness of the conformational sampling algorithm implemented in AutoDock Vina 1.2.6.

In the AChE dockings, apigenin-7-glucoside and luteolin displayed the most favorable docking scores, exceeding that of galantamine (Table 6; Figs 8 and 9; S2 Fig). This result agrees with previous docking studies reporting highly favorable AChE binding for apigenin-7-glucoside and other flavone-based compounds, while experimental evidence for AChE inhibition by luteolin-7-O-glucoside—a glycosylated derivative structurally related to the luteolin scaffold—further supports the AChE-inhibitory potential of this phytochemical class. Thus, apigenin-7-glucoside and luteolin can be regarded as promising AChE-interacting phytochemical hits, with their inhibitory relevance supported by the present docking-based findings and by related experimental observations on structurally analogous flavone compounds [32, 41, 44].

For TYRP1, rosmarinic acid demonstrated the most favorable docking score compared with the reference control kojic acid (Table 7; Fig 10; S3 Fig), suggesting its possible involvement in the tyrosinase-related inhibitory activity. This TYRP1 docking result is consistent with Khan et al., who identified rosmarinic acid as the top hit for tyrosinase inhibition through virtual screening and molecular docking [45]. This computational finding was further supported by L-DOPA-based mTYR/hTYR inhibition assays and B16F10 cell-based evaluation [45]. Because tyrosinase and TYRP1 are functionally related melanogenic metalloenzymes, this evidence supports the plausibility that rosmarinic acid may favorably interact with conserved metal-coordinating and substrate-recognition regions within the tyrosinase-related protein family; nevertheless, since TYRP1 inhibition was not directly tested in that study, the present docking result should be interpreted as a structure-based indication of potential TYRP1 inhibition rather than a biochemical validation.

Taken together, the docking results aligned with the experimental enzyme inhibition data and suggest that the major phenolic constituents of *O. thracica*, including the glycosylated flavones apigenin-7-glucoside and luteolin-7-glucoside, the flavone aglycone luteolin, and the phenolic acid rosmarinic acid, may contribute to the observed bioactivities. To avoid redundancy, detailed residue-level interaction patterns and non-bonded distance values are presented in Table 5–7 and the corresponding figures rather than being repeated extensively in the text.

## Conclusions

The findings of this study provide significant insights into the chemical composition, antioxidant, and enzyme inhibitory properties of methanol extracts obtained from *O. thracica* using maceration, ultrasound-assisted extraction, and Soxhlet extraction. The results highlight the influence of extraction methods on the recovery of bioactive compounds and their corresponding biological activities.

Among the extraction techniques, UAE and SOE demonstrated superior efficacy in extracting phenolic compounds with enhanced antioxidant potential, as evidenced by higher total phenolic and flavonoid contents. In particular, UAE-ME yielded the highest phenolic content, while SOE-ME exhibited the greatest flavonoid concentration. The LC–ESI–MS/MS analysis confirmed that luteolin-7-glucoside and apigenin-7-glucoside were the dominant flavonoids, while chlorogenic acid and rosmarinic acid were the most abundant phenolic acids. Maceration appeared to be more effective in recovering glycosylated flavonoids and chlorogenic acid, whereas Soxhlet extraction favored the accumulation of rosmarinic acid. Additionally, UAE facilitated the extraction of pinoresinol, a lignan with potential pharmacological benefits.

The antioxidant activity assessments demonstrated that UAE-ME and SOE-ME exhibited the highest radical scavenging and reducing power, as supported by CUPRAC, FRAP, DPPH, and ABTS assays, along with the RACI index. However, despite their promising activity, none of the extracts approached the antioxidant efficacy of the reference standard trolox, suggesting that further optimization of extraction conditions may enhance bioactivity.

Enzyme inhibition studies revealed moderate AChE and tyrosinase inhibitory activities. SOE-ME demonstrated the most potent AChE inhibition, followed by UAE-ME, while MAC-ME displayed significantly lower activity. Tyrosinase inhibition was comparable among extracts, indicating that the responsible bioactive constituents were similarly extracted across methods. Nonetheless, the inhibitory capacities of all extracts remained markedly weaker than standard inhibitors, such as galantamine and kojic acid, indicating the need for further purification or synergistic studies with other natural or synthetic inhibitors.

Despite these valuable findings, some limitations remain. The study did not evaluate the thermal and oxidative stability of the bioactive compounds, which could impact their biological efficacy in real-world applications. Additionally, while *in silico* modeling provided insights into potential interactions between major compounds and target enzymes, further *in vitro* and *in vivo* studies are required to confirm these findings and establish pharmacokinetic and toxicological profiles. Future research should focus on optimizing extraction conditions to maximize the yield of bioactive compounds while minimizing solvent use and degradation. Moreover, combinatorial approaches integrating multiple extraction techniques may offer a more comprehensive strategy to enhance bioactivity and compound recovery.

Overall, this study underscores the impact of extraction techniques on the phytochemical profile and bioactivity of *O. thracica* extracts, providing a foundation for further research into their pharmacological potential. The observed antioxidant and enzyme inhibitory activities suggest that these extracts may have potential applications in neuroprotection and skin health, warranting further investigation into their therapeutic and industrial applications.

## Supporting information

S1 Methods Supplementary methods. (DOCX)

S1 Table LC–ESI–MS/MS parameters and analytical characteristics. (DOCX)

S2 Table Calibration curves and sensitivity properties of the method. (DOCX)

S1 Fig Docking conformation of acarbose within AAMY. (TIF)

S2 Fig Docking conformation of galantamine within AChE. (TIF)

S3 Fig Docking conformation of kojic acid within TYRP1. (TIF)

S1 Data Raw LC–MS/MS quantitative analysis data. (PDF)
