# Supplementary material for: Phytochemical composition, antioxidant potential, and enzyme inhibitory properties of Onosma thracica extracts: A comparative study of extraction methods
Source: PLoS One. 2026 Jun 10;21(6):e0350995. doi: 10.1371/journal.pone.0350995 (PMC13252800; doi:10.1371/journal.pone.0350995)
Supplement: S1 Table — (DOCX) [file pone.0350995.s002.docx]

Table S1. ESI–MS/MS Parameters and analytical characteristics for the Analysis of Target Analytes by MRM Negative and Positive Ionization Mode

| Target compounds | Rt (min) | Precursor ion | MRM1 (CE, V) | MRM2 (CE, V) |
| --- | --- | --- | --- | --- |
| *Compounds analyzed by NI mode* |  |  |  |  |
| Gallic acid | 8.891 | 168.9 [M − H]− | 125.0 (10) | – |
| Protocatechuic acid | 10.818 | 152.9 [M − H]− | 108.9 (12) | – |
| 3,4-Dihydroxyphenylacetic acid | 11.224 | 167.0 [M − H]− | 123.0 (2) | – |
| (+)-Catechin | 11.369 | 289.0 [M − H]− | 245.0 (6) | 202.9 (12) |
| Pyrocatechol | 11.506 | 109.0 [M − H]− | 90.6 (18) | 52.9 (16) |
| 2,5-Dihydroxybenzoic acid | 12.412 | 152.9 [M − H]− | 109.0 (10) | – |
| 4-Hydroxybenzoic acid | 12.439 | 136.9 [M − H]− | 93.1 (14) | – |
| Caffeic acid | 12.841 | 179.0 [M − H]− | 135.0 (12) | – |
| Vanillic acid | 12.843 | 166.9 [M − H]− | 151.8 (10) | 122.6 (6) |
| Syringic acid | 12.963 | 196.9 [M − H]− | 181.9 (8) | 152.8 (6) |
| 3-Hydroxybenzoic acid | 13.259 | 137.0 [M − H]− | 93.0 (6) | – |
| Vanillin | 13.397 | 151.0 [M − H]− | 136.0 (10) | – |
| Verbascoside | 13.589 | 623.0 [M − H]− | 461.0 (26) | 160.8 (36) |
| Taxifolin | 13.909 | 303.0 [M − H]− | 285.1 (2) | 125.0 (14) |
| Sinapic acid | 13.992 | 222.9 [M − H]− | 207.9 (6) | 163.8 (6) |
| p-Coumaric acid | 14.022 | 162.9 [M − H]− | 119.0 (12) | – |
| Ferulic acid | 14.120 | 193.0 [M − H]− | 177.8 (8) | 134.0 (12) |
| Luteolin 7-glucoside | 14.266 | 447.1 [M − H]− | 285.0 (24) | – |
| Rosmarinic acid | 14.600 | 359.0 [M − H]− | 196.9 (10) | 160.9 (10) |
| 2-Hydroxycinnamic acid | 15.031 | 162.9 [M − H]− | 119.1 (10) | – |
| Pinoresinol | 15.118 | 357.0 [M − H]− | 151.0 (12) | 135.7 (34) |
| Eriodictyol | 15.247 | 287.0 [M − H]− | 151.0 (4) | 134.9 (22) |
| Quercetin | 15.668 | 301.0 [M − H]− | 178.6 (10) | 151.0 (16) |
| Kaempferol | 16.236 | 285.0 [M − H]− | 242.8 (16) | 229.1 (18) |
| *Compounds analyzed by PI mode* |  |  |  |  |
| Chlorogenic acid | 11.802 | 355.0 [M + H]+ | 163.0 (10) | – |
| (−)-Epicatechin | 12.458 | 291.0 [M + H]+ | 139.1 (12) | 122.9 (36) |
| Hesperidin | 14.412 | 611.1 [M + H]+ | 449.2 (4) | 303.0 (20) |
| Hyperoside | 14.506 | 465.1 [M + H]+ | 303.1 (8) | – |
| Apigenin 7-glucoside | 14.781 | 433.1 [M + H]+ | 271.0 (18) | – |
| Luteolin | 15.923 | 287.0 [M + H]+ | 153.1 (34) | 135.1 (36) |
| Apigenin | 16.382 | 271.0 [M + H]+ | 153.0 (34) | 119.1 (36) |

Rt, retention time; NI, negative ion; and PI, positive ion.
