# Supplementary material for: Phytochemical composition, antioxidant potential, and enzyme inhibitory properties of Onosma thracica extracts: A comparative study of extraction methods
Source: PLoS One. 2026 Jun 10;21(6):e0350995. doi: 10.1371/journal.pone.0350995 (PMC13252800; doi:10.1371/journal.pone.0350995)
Supplement: S2 Table — (DOCX) [file pone.0350995.s003.docx]

**Table S2.** Calibration curves and sensitivity properties of the method

|  | Linearity and sensitivity characteristics | | | |  |
| --- | --- | --- | --- | --- | --- |
| Compounds | Range  (μg/L) | Linear  equation | R^2^ | LOD  (μg/L) | LOQ  (μg/L) |
| Gallic acid | 5–500 | y = 4.82x − 26.48 | 0.9988 | 1.46 | 4.88 |
| Protocatechuic acid | 2.5–500 | y = 5.65x − 9.99 | 0.9990 | 1.17 | 3.88 |
| 3,4-Dihydroxyphenylacetic acid | 5–500 | y = 5.13x − 12.39 | 0.9990 | 1.35 | 4.51 |
| (+)-Catechin | 10–500 | y = 1.45x + 1.95 | 0.9974 | 3.96 | 13.20 |
| Pyrocatechol | 25–400 | y = 0.11x − 0.52 | 0.9916 | 9.62 | 32.08 |
| Chlorogenic acid | 1–500 | y = 12.14x + 32.34 | 0.9995 | 0.55 | 1.82 |
| 2,5-Dihydroxybenzoic acid | 5–500 | y = 3.79x − 14.12 | 0.9980 | 2.12 | 7.08 |
| 4-Hydroxybenzoic acid | 5–500 | y = 7.62x + 22.79 | 0.9996 | 1.72 | 5.72 |
| (−)-Epicatechin | 5–500 | y = 9.11x − 9.99 | 0.9971 | 1.85 | 6.18 |
| Caffeic acid | 5–500 | y = 11.09x + 16.73 | 0.9997 | 3.15 | 10.50 |
| Vanillic acid | 10–500 | y = 0.49x − 1.61 | 0.9968 | 2.56 | 8.54 |
| Syringic acid | 10–500 | y = 0.74x − 1.54 | 0.9975 | 3.75 | 12.50 |
| 3-Hydroxybenzoic acid | 5–500 | y = 3.69x − 12.29 | 0.9991 | 1.86 | 6.20 |
| Vanillin | 50–500 | y = 2.02x + 135.49 | 0.9926 | 15.23 | 50.77 |
| Verbascoside | 2.5–500 | y = 8.59x − 28.05 | 0.9988 | 0.82 | 2.75 |
| Taxifolin | 5–500 | y = 12.32x + 9.98 | 0.9993 | 1.82 | 6.05 |
| Sinapic acid | 5–500 | y = 2.09x − 6.79 | 0.9974 | 2.64 | 8.78 |
| p-Coumaric acid | 5–500 | y = 17.51x + 53.73 | 0.9997 | 1.93 | 6.44 |
| Ferulic acid | 5–500 | y = 3.32x − 4.30 | 0.9992 | 1.43 | 4.76 |
| Luteolin 7-glucoside | 1–500 | y = 45.25x + 156.48 | 0.9996 | 0.45 | 1.51 |
| Hesperidin | 5–500 | y = 5.98x + 0.42 | 0.9993 | 1.73 | 5.77 |
| Hyperoside | 2.5–500 | y = 16.32x − 1.26 | 0.9998 | 0.99 | 3.31 |
| Rosmarinic acid | 1–500 | y = 9.82x − 17.98 | 0.9989 | 0.57 | 1.89 |
| Apigenin 7-glucoside | 1–500 | y = 21.33x − 31.69 | 0.9983 | 0.41 | 1.35 |
| 2-Hydroxycinnamic acid | 1–500 | y = 16.72x − 26.94 | 0.9996 | 0.61 | 2.03 |
| Pinoresinol | 10–500 | y = 0.80x − 2.69 | 0.9966 | 3.94 | 13.12 |
| Eriodictyol | 2.5–500 | y = 14.24x − 0.50 | 0.9998 | 0.80 | 2.68 |
| Quercetin | 5–500 | y = 14.68x − 18.25 | 0.9997 | 1.23 | 4.10 |
| Luteolin | 5–500 | y = 8.96x + 26.80 | 0.9992 | 1.34 | 4.46 |
| Kaempferol | 10–500 | y = 0.82x − 3.06 | 0.9959 | 3.30 | 10.99 |
| Apigenin | 2.5–500 | y = 11.29x + 38.05 | 0.9987 | 0.96 | 3.20 |

LOD and LOQ: limit of detection and limit of quantification, respectively.
