# Supplementary figures and images for: Phytochemical composition, antioxidant potential, and enzyme inhibitory properties of Onosma thracica extracts: A comparative study of extraction methods
Source: PLoS One. 2026 Jun 10;21(6):e0350995. doi: 10.1371/journal.pone.0350995 (PMC13252800; doi:10.1371/journal.pone.0350995)

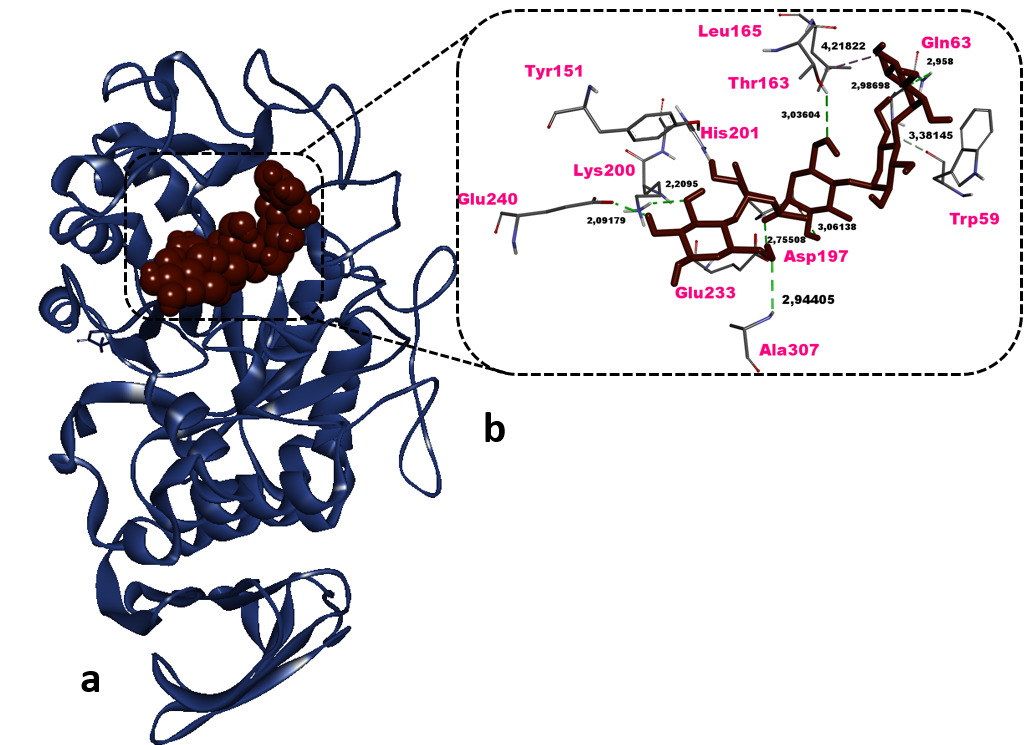

Supplement: S1 Fig — (TIF) [file pone.0350995.s004.tif]

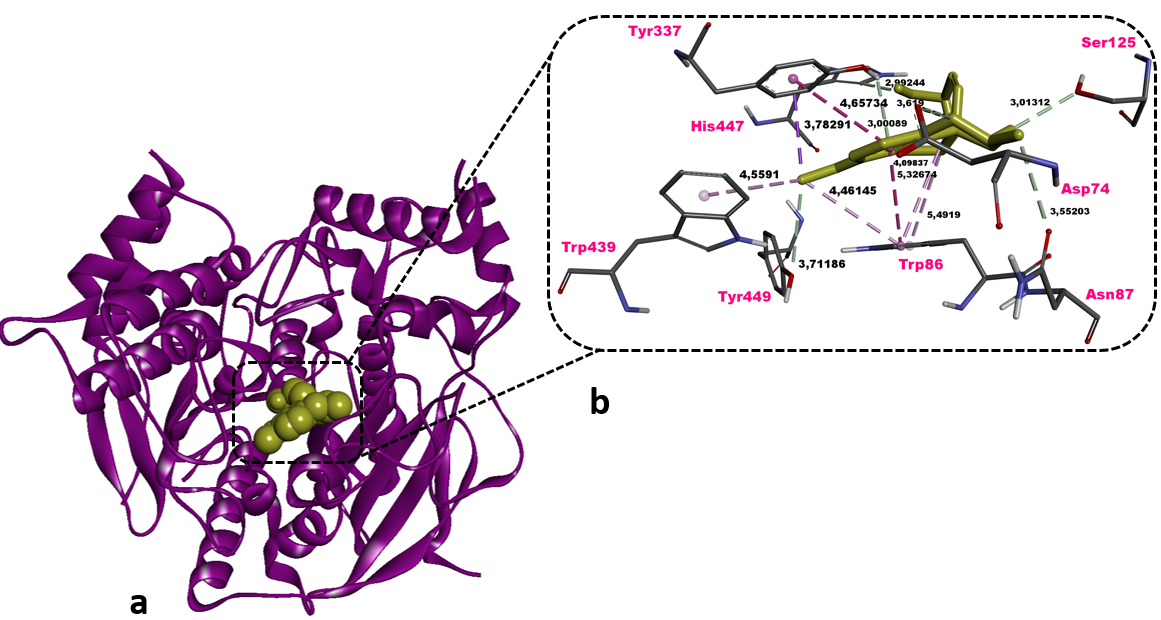

Supplement: S2 Fig — (TIF) [file pone.0350995.s005.tif]

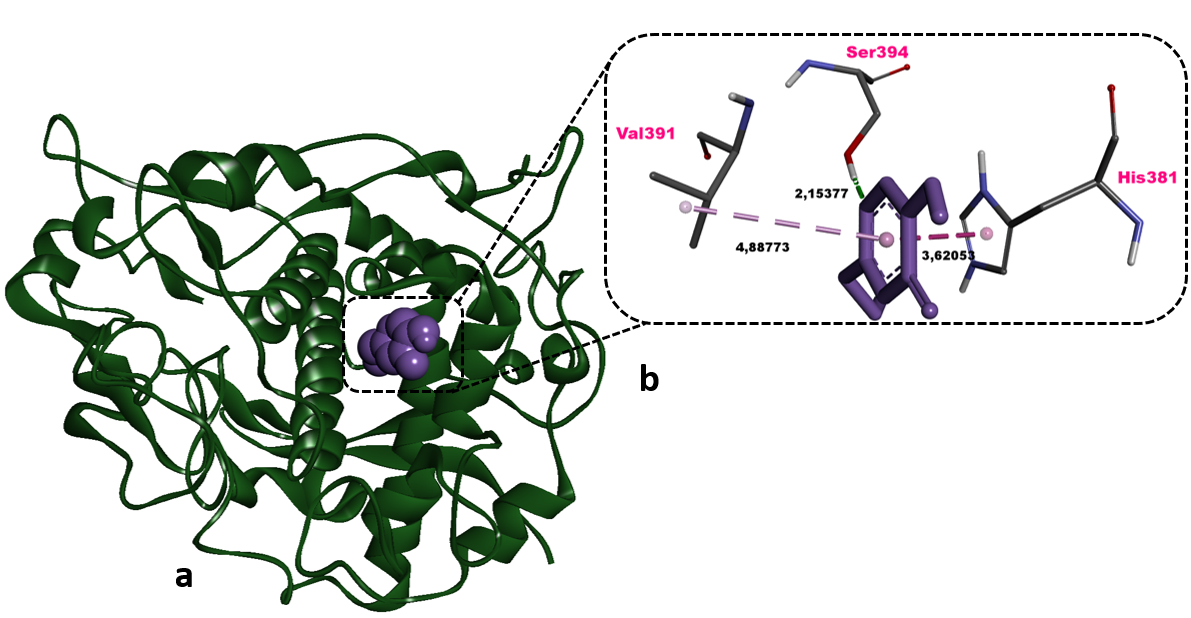

Supplement: S3 Fig — (TIF) [file pone.0350995.s006.tif]
