# Supplementary material for: Phytochemical composition, antioxidant potential, and enzyme inhibitory properties of Onosma thracica extracts: A comparative study of extraction methods
Source: PLoS One. 2026 Jun 10;21(6):e0350995. doi: 10.1371/journal.pone.0350995 (PMC13252800; doi:10.1371/journal.pone.0350995)

22-15A-Maceration-Methanol extract
22-15B-Soxhlet-Methanol extract
22-15C-Ultrasonic-assisted-Methanol extract

Quantitative Analysis Complete Report

Agilent Technologies

**Batch Path**	D:\MassHunter\Data\2022ekim\061022cengizhoca\QuantResults\071022.batch.bin		
**Analysis Time**	10/11/2022 1:33:26 PM	**Analyst Name**	Defam-PC\admin
**Report Time**	10/11/2022 1:33:45 PM	**Reporter Name**	admin
**Last Calib Update**	10/11/2022 1:33:17 PM	**Batch State**	Processed
**Quant Batch Version**	B.07.01	**Quant Report Version**	B.07.01

Data File	Sample Name	Sample Type	Position	Inj Vol	Level	Acq. Method
blank-061022-4.d	blank-061022-4	Sample	Vial 100	-1		FENOLIK_DMRM2021-31bilesen.m
25ppb1.d	25ppb1	Cal	Vial 1	-1	1	FENOLIK_DMRM2021-31bilesen.m
25ppb2.d	25ppb2	Cal	Vial 1	-1	1	FENOLIK_DMRM2021-31bilesen.m
25ppb3.d	25ppb3	Cal	Vial 1	-1	1	FENOLIK_DMRM2021-31bilesen.m
50ppb1.d	50ppb1	Cal	Vial 2	-1	2	FENOLIK_DMRM2021-31bilesen.m
50ppb2.d	50ppb2	Cal	Vial 2	-1	2	FENOLIK_DMRM2021-31bilesen.m
50ppb3.d	50ppb3	Cal	Vial 2	-1	2	FENOLIK_DMRM2021-31bilesen.m
100ppb1.d	100ppb1	Cal	Vial 3	-1	3	FENOLIK_DMRM2021-31bilesen.m
100ppb2.d	100ppb2	Cal	Vial 3	-1	3	FENOLIK_DMRM2021-31bilesen.m
100ppb3.d	100ppb3	Cal	Vial 3	-1	3	FENOLIK_DMRM2021-31bilesen.m
250ppb1.d	250ppb1	Cal	Vial 4	-1	4	FENOLIK_DMRM2021-31bilesen.m
250ppb2.d	250ppb2	Cal	Vial 4	-1	4	FENOLIK_DMRM2021-31bilesen.m
250ppb3.d	250ppb3	Cal	Vial 4	-1	4	FENOLIK_DMRM2021-31bilesen.m
500ppb1.d	500ppb1	Cal	Vial 5	-1	5	FENOLIK_DMRM2021-31bilesen.m
500ppb2.d	500ppb2	Cal	Vial 5	-1	5	FENOLIK_DMRM2021-31bilesen.m
500ppb3.d	500ppb3	Cal	Vial 5	-1	5	FENOLIK_DMRM2021-31bilesen.m
1000ppb1.d	1000ppb1	Cal	Vial 6	-1	6	FENOLIK_DMRM2021-31bilesen.m
1000ppb2.d	1000ppb2	Cal	Vial 6	-1	6	FENOLIK_DMRM2021-31bilesen.m
1000ppb3.d	1000ppb3	Cal	Vial 6	-1	6	FENOLIK_DMRM2021-31bilesen.m
22-1A-1.d	22-1A-1	Sample	Vial 7	-1		FENOLIK_DMRM2021-31bilesen.m
22-1A-2.d	22-1A-2	Sample	Vial 7	-1		FENOLIK_DMRM2021-31bilesen.m
22-1A-3.d	22-1A-3	Sample	Vial 7	-1		FENOLIK_DMRM2021-31bilesen.m
22-2A-1.d	22-2A-1	Sample	Vial 8	-1		FENOLIK_DMRM2021-31bilesen.m
22-2A-2.d	22-2A-2	Sample	Vial 8	-1		FENOLIK_DMRM2021-31bilesen.m
22-2A-3.d	22-2A-3	Sample	Vial 8	-1		FENOLIK_DMRM2021-31bilesen.m
22-3A-1.d	22-3A-1	Sample	Vial 9	-1		FENOLIK_DMRM2021-31bilesen.m
22-3A-2.d	22-3A-2	Sample	Vial 9	-1		FENOLIK_DMRM2021-31bilesen.m
22-3A-3.d	22-3A-3	Sample	Vial 9	-1		FENOLIK_DMRM2021-31bilesen.m
22-4A-1.d	22-4A-1	Sample	Vial 10	-1		FENOLIK_DMRM2021-31bilesen.m
22-4A-2.d	22-4A-2	Sample	Vial 10	-1		FENOLIK_DMRM2021-31bilesen.m
22-4A-3.d	22-4A-3	Sample	Vial 10	-1		FENOLIK_DMRM2021-31bilesen.m
22-5A-1.d	22-5A-1	Sample	Vial 11	-1		FENOLIK_DMRM2021-31bilesen.m
22-5A-2.d	22-5A-2	Sample	Vial 11	-1		FENOLIK_DMRM2021-31bilesen.m
22-5A-3.d	22-5A-3	Sample	Vial 11	-1		FENOLIK_DMRM2021-31bilesen.m
22-6A-1.d	22-6A-1	Sample	Vial 12	-1		FENOLIK_DMRM2021-31bilesen.m
22-6A-2.d	22-6A-2	Sample	Vial 12	-1		FENOLIK_DMRM2021-31bilesen.m

22-6A-3.d	22-6A-3	Sample	Vial 12	-1	FENOLIK_DMRM2021-31bilesen.m
22-7A-1.d	22-7A-1	Sample	Vial 13	-1	FENOLIK_DMRM2021-31bilesen.m
22-7A-2.d	22-7A-2	Sample	Vial 13	-1	FENOLIK_DMRM2021-31bilesen.m
22-7A-3.d	22-7A-3	Sample	Vial 13	-1	FENOLIK_DMRM2021-31bilesen.m
22-8A-1.d	22-8A-1	Sample	Vial 14	-1	FENOLIK_DMRM2021-31bilesen.m
22-8A-2.d	22-8A-2	Sample	Vial 14	-1	FENOLIK_DMRM2021-31bilesen.m
22-8A-3.d	22-8A-3	Sample	Vial 14	-1	FENOLIK_DMRM2021-31bilesen.m
22-9A-1.d	22-9A-1	Sample	Vial 15	-1	FENOLIK_DMRM2021-31bilesen.m
22-9A-2.d	22-9A-2	Sample	Vial 15	-1	FENOLIK_DMRM2021-31bilesen.m
22-9A-3.d	22-9A-3	Sample	Vial 15	-1	FENOLIK_DMRM2021-31bilesen.m
22-10A-1.d	22-10A-1	Sample	Vial 16	-1	FENOLIK_DMRM2021-31bilesen.m
22-10A-2.d	22-10A-2	Sample	Vial 16	-1	FENOLIK_DMRM2021-31bilesen.m
22-10A-3.d	22-10A-3	Sample	Vial 16	-1	FENOLIK_DMRM2021-31bilesen.m
22-11A-1.d	22-11A-1	Sample	Vial 17	-1	FENOLIK_DMRM2021-31bilesen.m
22-11A-2.d	22-11A-2	Sample	Vial 17	-1	FENOLIK_DMRM2021-31bilesen.m
22-11A-3.d	22-11A-3	Sample	Vial 17	-1	FENOLIK_DMRM2021-31bilesen.m
22-12A-1.d	22-12A-1	Sample	Vial 18	-1	FENOLIK_DMRM2021-31bilesen.m
22-12A-2.d	22-12A-2	Sample	Vial 18	-1	FENOLIK_DMRM2021-31bilesen.m
22-12A-3.d	22-12A-3	Sample	Vial 18	-1	FENOLIK_DMRM2021-31bilesen.m
22-13A-1.d	22-13A-1	Sample	Vial 19	-1	FENOLIK_DMRM2021-31bilesen.m
22-13A-2.d	22-13A-2	Sample	Vial 19	-1	FENOLIK_DMRM2021-31bilesen.m
22-13A-3.d	22-13A-3	Sample	Vial 19	-1	FENOLIK_DMRM2021-31bilesen.m
22-14A-1.d	22-14A-1	Sample	Vial 20	-1	FENOLIK_DMRM2021-31bilesen.m
22-14A-2.d	22-14A-2	Sample	Vial 20	-1	FENOLIK_DMRM2021-31bilesen.m
22-14A-3.d	22-14A-3	Sample	Vial 20	-1	FENOLIK_DMRM2021-31bilesen.m
22-15A-1.d	22-15A-1	Sample	Vial 21	-1	FENOLIK_DMRM2021-31bilesen.m
22-15A-2.d	22-15A-2	Sample	Vial 21	-1	FENOLIK_DMRM2021-31bilesen.m
22-15A-3.d	22-15A-3	Sample	Vial 21	-1	FENOLIK_DMRM2021-31bilesen.m
22-16A-1.d	22-16A-1	Sample	Vial 22	-1	FENOLIK_DMRM2021-31bilesen.m
22-16A-2.d	22-16A-2	Sample	Vial 22	-1	FENOLIK_DMRM2021-31bilesen.m
22-16A-3.d	22-16A-3	Sample	Vial 22	-1	FENOLIK_DMRM2021-31bilesen.m
22-17A-1.d	22-17A-1	Sample	Vial 23	-1	FENOLIK_DMRM2021-31bilesen.m
22-17A-2.d	22-17A-2	Sample	Vial 23	-1	FENOLIK_DMRM2021-31bilesen.m
22-17A-3.d	22-17A-3	Sample	Vial 23	-1	FENOLIK_DMRM2021-31bilesen.m
22-18A-1.d	22-18A-1	Sample	Vial 24	-1	FENOLIK_DMRM2021-31bilesen.m
22-18A-2.d	22-18A-2	Sample	Vial 24	-1	FENOLIK_DMRM2021-31bilesen.m
22-18A-3.d	22-18A-3	Sample	Vial 24	-1	FENOLIK_DMRM2021-31bilesen.m
22-1B-1.d	22-1B-1	Sample	Vial 25	-1	FENOLIK_DMRM2021-31bilesen.m
22-1B-2.d	22-1B-2	Sample	Vial 25	-1	FENOLIK_DMRM2021-31bilesen.m
22-1B-3.d	22-1B-3	Sample	Vial 25	-1	FENOLIK_DMRM2021-31bilesen.m
22-2B-1.d	22-2B-1	Sample	Vial 26	-1	FENOLIK_DMRM2021-31bilesen.m
22-2B-2.d	22-2B-2	Sample	Vial 26	-1	FENOLIK_DMRM2021-31bilesen.m

22-2B-3.d	22-2B-3	Sample	Vial 26	-1	FENOLIK_DMRM2021-31bilesen.m
22-3B-1.d	22-3B-1	Sample	Vial 27	-1	FENOLIK_DMRM2021-31bilesen.m
22-3B-2.d	22-3B-2	Sample	Vial 27	-1	FENOLIK_DMRM2021-31bilesen.m
22-3B-3.d	22-3B-3	Sample	Vial 27	-1	FENOLIK_DMRM2021-31bilesen.m
22-4B-1.d	22-4B-1	Sample	Vial 28	-1	FENOLIK_DMRM2021-31bilesen.m
22-4B-2.d	22-4B-2	Sample	Vial 28	-1	FENOLIK_DMRM2021-31bilesen.m
22-4B-3.d	22-4B-3	Sample	Vial 28	-1	FENOLIK_DMRM2021-31bilesen.m
22-5B-1.d	22-5B-1	Sample	Vial 29	-1	FENOLIK_DMRM2021-31bilesen.m
22-5B-2.d	22-5B-2	Sample	Vial 29	-1	FENOLIK_DMRM2021-31bilesen.m
22-5B-3.d	22-5B-3	Sample	Vial 29	-1	FENOLIK_DMRM2021-31bilesen.m
22-6B-1.d	22-6B-1	Sample	Vial 30	-1	FENOLIK_DMRM2021-31bilesen.m
22-6B-2.d	22-6B-2	Sample	Vial 30	-1	FENOLIK_DMRM2021-31bilesen.m
22-6B-3.d	22-6B-3	Sample	Vial 30	-1	FENOLIK_DMRM2021-31bilesen.m
22-7B-1.d	22-7B-1	Sample	Vial 31	-1	FENOLIK_DMRM2021-31bilesen.m
22-7B-2.d	22-7B-2	Sample	Vial 31	-1	FENOLIK_DMRM2021-31bilesen.m
22-7B-3.d	22-7B-3	Sample	Vial 31	-1	FENOLIK_DMRM2021-31bilesen.m
22-8B-1.d	22-8B-1	Sample	Vial 32	-1	FENOLIK_DMRM2021-31bilesen.m
22-8B-2.d	22-8B-2	Sample	Vial 32	-1	FENOLIK_DMRM2021-31bilesen.m
22-8B-3.d	22-8B-3	Sample	Vial 32	-1	FENOLIK_DMRM2021-31bilesen.m
22-9B-1.d	22-9B-1	Sample	Vial 33	-1	FENOLIK_DMRM2021-31bilesen.m
22-9B-2.d	22-9B-2	Sample	Vial 33	-1	FENOLIK_DMRM2021-31bilesen.m
22-9B-3.d	22-9B-3	Sample	Vial 33	-1	FENOLIK_DMRM2021-31bilesen.m
22-10B-1.d	22-10B-1	Sample	Vial 34	-1	FENOLIK_DMRM2021-31bilesen.m
22-10B-2.d	22-10B-2	Sample	Vial 34	-1	FENOLIK_DMRM2021-31bilesen.m
22-10B-3.d	22-10B-3	Sample	Vial 34	-1	FENOLIK_DMRM2021-31bilesen.m
22-11B-1.d	22-11B-1	Sample	Vial 35	-1	FENOLIK_DMRM2021-31bilesen.m
22-11B-2.d	22-11B-2	Sample	Vial 35	-1	FENOLIK_DMRM2021-31bilesen.m
22-11B-3.d	22-11B-3	Sample	Vial 35	-1	FENOLIK_DMRM2021-31bilesen.m
22-12B-1.d	22-12B-1	Sample	Vial 36	-1	FENOLIK_DMRM2021-31bilesen.m
22-12B-2.d	22-12B-2	Sample	Vial 36	-1	FENOLIK_DMRM2021-31bilesen.m
22-12B-3.d	22-12B-3	Sample	Vial 36	-1	FENOLIK_DMRM2021-31bilesen.m
22-13B-1.d	22-13B-1	Sample	Vial 37	-1	FENOLIK_DMRM2021-31bilesen.m
22-13B-2.d	22-13B-2	Sample	Vial 37	-1	FENOLIK_DMRM2021-31bilesen.m
22-13B-3.d	22-13B-3	Sample	Vial 37	-1	FENOLIK_DMRM2021-31bilesen.m
22-14B-1.d	22-14B-1	Sample	Vial 38	-1	FENOLIK_DMRM2021-31bilesen.m
22-14B-2.d	22-14B-2	Sample	Vial 38	-1	FENOLIK_DMRM2021-31bilesen.m
22-14B-3.d	22-14B-3	Sample	Vial 38	-1	FENOLIK_DMRM2021-31bilesen.m
22-15B-1.d	22-15B-1	Sample	Vial 39	-1	FENOLIK_DMRM2021-31bilesen.m
22-15B-2.d	22-15B-2	Sample	Vial 39	-1	FENOLIK_DMRM2021-31bilesen.m
22-15B-3.d	22-15B-3	Sample	Vial 39	-1	FENOLIK_DMRM2021-31bilesen.m
22-16B-1.d	22-16B-1	Sample	Vial 40	-1	FENOLIK_DMRM2021-31bilesen.m
22-16B-2.d	22-16B-2	Sample	Vial 40	-1	FENOLIK_DMRM2021-31bilesen.m

22-16B-3.d	22-16B-3	Sample	Vial 40	-1	FENOLIK_DMRM2021-31bilesen.m
22-17B-1.d	22-17B-1	Sample	Vial 41	-1	FENOLIK_DMRM2021-31bilesen.m
22-17B-2.d	22-17B-2	Sample	Vial 41	-1	FENOLIK_DMRM2021-31bilesen.m
22-17B-3.d	22-17B-3	Sample	Vial 41	-1	FENOLIK_DMRM2021-31bilesen.m
22-18B-1.d	22-18B-1	Sample	Vial 42	-1	FENOLIK_DMRM2021-31bilesen.m
22-18B-2.d	22-18B-2	Sample	Vial 42	-1	FENOLIK_DMRM2021-31bilesen.m
22-18B-3.d	22-18B-3	Sample	Vial 42	-1	FENOLIK_DMRM2021-31bilesen.m
22-1C-1.d	22-1C-1	Sample	Vial 43	-1	FENOLIK_DMRM2021-31bilesen.m
22-1C-2.d	22-1C-2	Sample	Vial 43	-1	FENOLIK_DMRM2021-31bilesen.m
22-1C-3.d	22-1C-3	Sample	Vial 43	-1	FENOLIK_DMRM2021-31bilesen.m
22-2C-1.d	22-2C-1	Sample	Vial 44	-1	FENOLIK_DMRM2021-31bilesen.m
22-2C-2.d	22-2C-2	Sample	Vial 44	-1	FENOLIK_DMRM2021-31bilesen.m
22-2C-3.d	22-2C-3	Sample	Vial 44	-1	FENOLIK_DMRM2021-31bilesen.m
22-3C-1.d	22-3C-1	Sample	Vial 45	-1	FENOLIK_DMRM2021-31bilesen.m
22-3C-2.d	22-3C-2	Sample	Vial 45	-1	FENOLIK_DMRM2021-31bilesen.m
22-3C-3.d	22-3C-3	Sample	Vial 45	-1	FENOLIK_DMRM2021-31bilesen.m
22-4C-1.d	22-4C-1	Sample	Vial 46	-1	FENOLIK_DMRM2021-31bilesen.m
22-4C-2.d	22-4C-2	Sample	Vial 46	-1	FENOLIK_DMRM2021-31bilesen.m
22-4C-3.d	22-4C-3	Sample	Vial 46	-1	FENOLIK_DMRM2021-31bilesen.m
22-5C-1.d	22-5C-1	Sample	Vial 47	-1	FENOLIK_DMRM2021-31bilesen.m
22-5C-2.d	22-5C-2	Sample	Vial 47	-1	FENOLIK_DMRM2021-31bilesen.m
22-5C-3.d	22-5C-3	Sample	Vial 47	-1	FENOLIK_DMRM2021-31bilesen.m
22-6C-1.d	22-6C-1	Sample	Vial 48	-1	FENOLIK_DMRM2021-31bilesen.m
22-6C-2.d	22-6C-2	Sample	Vial 48	-1	FENOLIK_DMRM2021-31bilesen.m
22-6C-3.d	22-6C-3	Sample	Vial 48	-1	FENOLIK_DMRM2021-31bilesen.m
22-7C-1.d	22-7C-1	Sample	Vial 49	-1	FENOLIK_DMRM2021-31bilesen.m
22-7C-2.d	22-7C-2	Sample	Vial 49	-1	FENOLIK_DMRM2021-31bilesen.m
22-7C-3.d	22-7C-3	Sample	Vial 49	-1	FENOLIK_DMRM2021-31bilesen.m
22-8C-1.d	22-8C-1	Sample	Vial 50	-1	FENOLIK_DMRM2021-31bilesen.m
22-8C-2.d	22-8C-2	Sample	Vial 50	-1	FENOLIK_DMRM2021-31bilesen.m
22-8C-3.d	22-8C-3	Sample	Vial 50	-1	FENOLIK_DMRM2021-31bilesen.m
22-9C-1.d	22-9C-1	Sample	Vial 51	-1	FENOLIK_DMRM2021-31bilesen.m
22-9C-2.d	22-9C-2	Sample	Vial 51	-1	FENOLIK_DMRM2021-31bilesen.m
22-9C-3.d	22-9C-3	Sample	Vial 51	-1	FENOLIK_DMRM2021-31bilesen.m
22-10C-1.d	22-10C-1	Sample	Vial 52	-1	FENOLIK_DMRM2021-31bilesen.m
22-10C-2.d	22-10C-2	Sample	Vial 52	-1	FENOLIK_DMRM2021-31bilesen.m
22-10C-3.d	22-10C-3	Sample	Vial 52	-1	FENOLIK_DMRM2021-31bilesen.m
22-11C-1.d	22-11C-1	Sample	Vial 53	-1	FENOLIK_DMRM2021-31bilesen.m
22-11C-2.d	22-11C-2	Sample	Vial 53	-1	FENOLIK_DMRM2021-31bilesen.m
22-11C-3.d	22-11C-3	Sample	Vial 53	-1	FENOLIK_DMRM2021-31bilesen.m
22-12C-1.d	22-12C-1	Sample	Vial 54	-1	FENOLIK_DMRM2021-31bilesen.m
22-12C-2.d	22-12C-2	Sample	Vial 54	-1	FENOLIK_DMRM2021-31bilesen.m

22-12C-3.d	22-12C-3	Sample	Vial 54	-1	FENOLIK_DMRM2021-31bilesen.m
22-13C-1.d	22-13C-1	Sample	Vial 55	-1	FENOLIK_DMRM2021-31bilesen.m
22-13C-2.d	22-13C-2	Sample	Vial 55	-1	FENOLIK_DMRM2021-31bilesen.m
22-13C-3.d	22-13C-3	Sample	Vial 55	-1	FENOLIK_DMRM2021-31bilesen.m
22-14C-1.d	22-14C-1	Sample	Vial 56	-1	FENOLIK_DMRM2021-31bilesen.m
22-14C-2.d	22-14C-2	Sample	Vial 56	-1	FENOLIK_DMRM2021-31bilesen.m
22-14C-3.d	22-14C-3	Sample	Vial 56	-1	FENOLIK_DMRM2021-31bilesen.m
22-15C-1.d	22-15C-1	Sample	Vial 57	-1	FENOLIK_DMRM2021-31bilesen.m
22-15C-2.d	22-15C-2	Sample	Vial 57	-1	FENOLIK_DMRM2021-31bilesen.m
22-15C-3.d	22-15C-3	Sample	Vial 57	-1	FENOLIK_DMRM2021-31bilesen.m
22-16C-1.d	22-16C-1	Sample	Vial 58	-1	FENOLIK_DMRM2021-31bilesen.m
22-16C-2.d	22-16C-2	Sample	Vial 58	-1	FENOLIK_DMRM2021-31bilesen.m
22-16C-3.d	22-16C-3	Sample	Vial 58	-1	FENOLIK_DMRM2021-31bilesen.m
22-17C-1.d	22-17C-1	Sample	Vial 59	-1	FENOLIK_DMRM2021-31bilesen.m
22-17C-2.d	22-17C-2	Sample	Vial 59	-1	FENOLIK_DMRM2021-31bilesen.m
22-17C-3.d	22-17C-3	Sample	Vial 59	-1	FENOLIK_DMRM2021-31bilesen.m
22-18C-1.d	22-18C-1	Sample	Vial 60	-1	FENOLIK_DMRM2021-31bilesen.m
22-18C-2.d	22-18C-2	Sample	Vial 60	-1	FENOLIK_DMRM2021-31bilesen.m
22-18C-3.d	22-18C-3	Sample	Vial 60	-1	FENOLIK_DMRM2021-31bilesen.m

Quantitation Results

Gallic acid

Data File	Compound	Sample Type	RT	Resp.	Final Conc	Exp. Conc	Accuracy
blank-061022-4.d	Gallic acid	Sample	8.925	8	ND		
25ppb1.d	Gallic acid	Calibration	8.666	9	ND		0.00
25ppb2.d	Gallic acid	Calibration	8.808	6	ND		0.00
25ppb3.d	Gallic acid	Calibration	8.666	171	ND		0.00
50ppb1.d	Gallic acid	Calibration	8.708	436	18.8746		37.75
50ppb2.d	Gallic acid	Calibration	8.708	533	27.9968		55.99
50ppb3.d	Gallic acid	Calibration	8.733	581	32.5043		65.01
100ppb1.d	Gallic acid	Calibration	8.733	1398	109.3299		109.33
100ppb2.d	Gallic acid	Calibration	8.741	1358	105.5540		105.55
100ppb3.d	Gallic acid	Calibration	8.741	1360	105.7217		105.72
250ppb1.d	Gallic acid	Calibration	8.750	2981	258.1111		103.24
250ppb2.d	Gallic acid	Calibration	8.750	2978	257.8373		103.13
250ppb3.d	Gallic acid	Calibration	8.758	2962	256.3445		102.54
500ppb1.d	Gallic acid	Calibration	8.766	5975	539.5334		107.91
500ppb2.d	Gallic acid	Calibration	8.775	6025	544.2341		108.85
500ppb3.d	Gallic acid	Calibration	8.775	5984	540.3413		108.07
1000ppb1.d	Gallic acid	Calibration	8.783	11032	1014.7633		101.48
1000ppb2.d	Gallic acid	Calibration	8.783	10374	952.9453		95.29
1000ppb3.d	Gallic acid	Calibration	8.783	10522	966.9094		96.69
22-1A-1.d	Gallic acid	Sample	8.783	4234	375.8901		

Gallic acid

Data File	Compound	Sample Type	RT	Resp.	Final Conc	Exp. Conc	Accuracy
22-1A-2.d	Gallic acid	Sample	8.783	4282	380.3744		
22-1A-3.d	Gallic acid	Sample	8.783	4412	392.5835		
22-2A-1.d	Gallic acid	Sample	8.783	1210	91.6017		
22-2A-2.d	Gallic acid	Sample	8.791	1225	93.0988		
22-2A-3.d	Gallic acid	Sample	8.783	1277	97.9370		
22-3A-1.d	Gallic acid	Sample	8.775	2250	189.4075		
22-3A-2.d	Gallic acid	Sample	8.791	2259	190.1971		
22-3A-3.d	Gallic acid	Sample	8.783	2260	190.3271		
22-4A-1.d	Gallic acid	Sample	8.783	418	17.2194		
22-4A-2.d	Gallic acid	Sample	8.800	454	20.5812		
22-4A-3.d	Gallic acid	Sample	8.791	436	18.8608		
22-5A-1.d	Gallic acid	Sample	8.791	2123	177.4493		
22-5A-2.d	Gallic acid	Sample	8.791	2157	180.6427		
22-5A-3.d	Gallic acid	Sample	8.800	2105	175.7914		
22-6A-1.d	Gallic acid	Sample	8.791	712	44.8324		
22-6A-2.d	Gallic acid	Sample	8.791	843	57.1056		
22-6A-3.d	Gallic acid	Sample	8.800	824	55.3294		
22-7A-1.d	Gallic acid	Sample	8.800	988	70.7921		
22-7A-2.d	Gallic acid	Sample	8.800	1026	74.3973		
22-7A-3.d	Gallic acid	Sample	8.800	1062	77.7655		
22-8A-1.d	Gallic acid	Sample	8.800	845	57.3005		
22-8A-2.d	Gallic acid	Sample	8.791	825	55.4723		
22-8A-3.d	Gallic acid	Sample	8.791	848	57.5834		
22-9A-1.d	Gallic acid	Sample	8.800	505	25.4116		
22-9A-2.d	Gallic acid	Sample	8.800	512	26.0289		
22-9A-3.d	Gallic acid	Sample	8.800	504	25.3345		
22-10A-1.d	Gallic acid	Sample	8.775	264	2.7575		
22-10A-2.d	Gallic acid	Sample	8.783	246	1.0676		
22-10A-3.d	Gallic acid	Sample	8.783	261	2.4613		
22-11A-1.d	Gallic acid	Sample	8.808	121	ND		
22-11A-2.d	Gallic acid	Sample	8.800	114	ND		
22-11A-3.d	Gallic acid	Sample	8.791	115	ND		
22-12A-1.d	Gallic acid	Sample	8.825	15	ND		
22-12A-2.d	Gallic acid	Sample	8.758	7	ND		
22-12A-3.d	Gallic acid	Sample	8.633	0	ND		
22-13A-1.d	Gallic acid	Sample	8.775	1082	79.6453		
22-13A-2.d	Gallic acid	Sample	8.775	1129	84.0480		
22-13A-3.d	Gallic acid	Sample	8.775	1276	97.8451		
22-14A-1.d	Gallic acid	Sample	8.783	440	19.2751		
22-14A-2.d	Gallic acid	Sample	8.775	429	18.2780		

Gallic acid

Data File	Compound	Sample Type	RT	Resp.	Final Conc	Exp. Conc	Accuracy
22-14A-3.d	Gallic acid	Sample	8.783	444	19.6579		
22-15A-1.d	Gallic acid	Sample	8.800	6	ND		
22-15A-2.d	Gallic acid	Sample	8.883	5	ND		
22-15A-3.d	Gallic acid	Sample	8.791	2	ND		
22-16A-1.d	Gallic acid	Sample	8.775	4	ND		
22-16A-2.d	Gallic acid	Sample	8.775	11	ND		
22-16A-3.d	Gallic acid	Sample	8.808	41	ND		
22-17A-1.d	Gallic acid	Sample	8.733	5	ND		
22-17A-2.d	Gallic acid	Sample	8.833	4	ND		
22-17A-3.d	Gallic acid	Sample	8.775	3	ND		
22-18A-1.d	Gallic acid	Sample	8.775	11921	1098.3534		
22-18A-2.d	Gallic acid	Sample	8.758	10132	930.1911		
22-18A-3.d	Gallic acid	Sample	8.758	9255	847.7905		
22-1B-1.d	Gallic acid	Sample	8.758	3607	316.8921		
22-1B-2.d	Gallic acid	Sample	8.758	3446	301.8414		
22-1B-3.d	Gallic acid	Sample	8.766	3685	324.2814		
22-2B-1.d	Gallic acid	Sample	8.766	841	56.9461		
22-2B-2.d	Gallic acid	Sample	8.758	947	66.9576		
22-2B-3.d	Gallic acid	Sample	8.766	985	70.4847		
22-3B-1.d	Gallic acid	Sample	8.758	2440	207.2608		
22-3B-2.d	Gallic acid	Sample	8.758	2280	192.2514		
22-3B-3.d	Gallic acid	Sample	8.758	2458	208.9901		
22-4B-1.d	Gallic acid	Sample	8.758	362	11.9407		
22-4B-2.d	Gallic acid	Sample	8.775	441	19.4018		
22-4B-3.d	Gallic acid	Sample	8.766	397	15.1961		
22-5B-1.d	Gallic acid	Sample	8.758	1911	157.5720		
22-5B-2.d	Gallic acid	Sample	8.766	2013	167.1568		
22-5B-3.d	Gallic acid	Sample	8.766	1972	163.2369		
22-6B-1.d	Gallic acid	Sample	8.766	1168	87.7147		
22-6B-2.d	Gallic acid	Sample	8.766	1230	93.4992		
22-6B-3.d	Gallic acid	Sample	8.766	1279	98.0907		
22-7B-1.d	Gallic acid	Sample	8.766	922	64.6059		
22-7B-2.d	Gallic acid	Sample	8.766	923	64.6867		
22-7B-3.d	Gallic acid	Sample	8.766	948	67.0552		
22-8B-1.d	Gallic acid	Sample	8.766	925	64.8981		
22-8B-2.d	Gallic acid	Sample	8.766	922	64.5405		
22-8B-3.d	Gallic acid	Sample	8.775	965	68.6520		
22-9B-1.d	Gallic acid	Sample	8.766	439	19.1944		
22-9B-2.d	Gallic acid	Sample	8.766	430	18.3402		
22-9B-3.d	Gallic acid	Sample	8.766	406	16.0712		

Gallic acid

Data File	Compound	Sample Type	RT	Resp.	Final Conc	Exp. Conc	Accuracy
22-10B-1.d	Gallic acid	Sample	8.758	278	4.0118		
22-10B-2.d	Gallic acid	Sample	8.758	293	5.4799		
22-10B-3.d	Gallic acid	Sample	8.766	279	4.1026		
22-11B-1.d	Gallic acid	Sample	8.766	126	ND		
22-11B-2.d	Gallic acid	Sample	8.766	128	ND		
22-11B-3.d	Gallic acid	Sample	8.766	134	ND		
22-12B-1.d	Gallic acid	Sample	8.758	24	ND		
22-12B-2.d	Gallic acid	Sample	8.758	14	ND		
22-12B-3.d	Gallic acid	Sample	8.808	22	ND		
22-13B-1.d	Gallic acid	Sample	8.758	1187	89.5100		
22-13B-2.d	Gallic acid	Sample	8.758	1138	84.8388		
22-13B-3.d	Gallic acid	Sample	8.758	1254	95.7358		
22-14B-1.d	Gallic acid	Sample	8.775	494	24.3450		
22-14B-2.d	Gallic acid	Sample	8.766	447	19.8937		
22-14B-3.d	Gallic acid	Sample	8.766	514	26.1820		
22-15B-1.d	Gallic acid	Sample	8.716	2	ND		
22-15B-2.d	Gallic acid	Sample	8.900	3	ND		
22-15B-3.d	Gallic acid	Sample	8.825	6	ND		
22-16B-1.d	Gallic acid	Sample	8.766	40	ND		
22-16B-2.d	Gallic acid	Sample	8.775	39	ND		
22-16B-3.d	Gallic acid	Sample	8.758	37	ND		
22-17B-1.d	Gallic acid	Sample	8.783	32	ND		
22-17B-2.d	Gallic acid	Sample	8.733	26	ND		
22-17B-3.d	Gallic acid	Sample	8.766	16	ND		
22-18B-1.d	Gallic acid	Sample	8.766	7117	646.8790		
22-18B-2.d	Gallic acid	Sample	8.766	7242	658.5912		
22-18B-3.d	Gallic acid	Sample	8.766	7227	657.1982		
22-1C-1.d	Gallic acid	Sample	8.766	3368	294.4738		
22-1C-2.d	Gallic acid	Sample	8.758	3434	300.7144		
22-1C-3.d	Gallic acid	Sample	8.758	3405	297.9940		
22-2C-1.d	Gallic acid	Sample	8.758	727	46.2626		
22-2C-2.d	Gallic acid	Sample	8.758	720	45.6203		
22-2C-3.d	Gallic acid	Sample	8.766	735	46.9569		
22-3C-1.d	Gallic acid	Sample	8.758	1326	102.5925		
22-3C-2.d	Gallic acid	Sample	8.758	1391	108.6704		
22-3C-3.d	Gallic acid	Sample	8.758	1434	112.7082		
22-4C-1.d	Gallic acid	Sample	8.766	291	5.2529		
22-4C-2.d	Gallic acid	Sample	8.766	305	6.5563		
22-4C-3.d	Gallic acid	Sample	8.766	291	5.2929		
22-5C-1.d	Gallic acid	Sample	8.766	1334	103.2612		

Gallic acid

Data File	Compound	Sample Type	RT	Resp.	Final Conc	Exp. Conc	Accuracy
22-5C-2.d	Gallic acid	Sample	8.766	1271	97.3876		
22-5C-3.d	Gallic acid	Sample	8.766	1327	102.6339		
22-6C-1.d	Gallic acid	Sample	8.766	744	47.8818		
22-6C-2.d	Gallic acid	Sample	8.758	781	51.2813		
22-6C-3.d	Gallic acid	Sample	8.766	831	55.9802		
22-7C-1.d	Gallic acid	Sample	8.766	654	39.3693		
22-7C-2.d	Gallic acid	Sample	8.766	677	41.5233		
22-7C-3.d	Gallic acid	Sample	8.766	641	38.1635		
22-8C-1.d	Gallic acid	Sample	8.766	546	29.2117		
22-8C-2.d	Gallic acid	Sample	8.775	502	25.0844		
22-8C-3.d	Gallic acid	Sample	8.775	615	35.6870		
22-9C-1.d	Gallic acid	Sample	8.766	416	17.0194		
22-9C-2.d	Gallic acid	Sample	8.775	405	15.9767		
22-9C-3.d	Gallic acid	Sample	8.775	406	16.1193		
22-10C-1.d	Gallic acid	Sample	8.766	261	2.4720		
22-10C-2.d	Gallic acid	Sample	8.758	285	4.6986		
22-10C-3.d	Gallic acid	Sample	8.766	291	5.3132		
22-11C-1.d	Gallic acid	Sample	8.783	109	ND		
22-11C-2.d	Gallic acid	Sample	8.766	92	ND		
22-11C-3.d	Gallic acid	Sample	8.783	108	ND		
22-12C-1.d	Gallic acid	Sample	8.758	35	ND		
22-12C-2.d	Gallic acid	Sample	8.750	26	ND		
22-12C-3.d	Gallic acid	Sample	8.783	9	ND		
22-13C-1.d	Gallic acid	Sample	8.775	974	69.4411		
22-13C-2.d	Gallic acid	Sample	8.775	934	65.6741		
22-13C-3.d	Gallic acid	Sample	8.775	960	68.1920		
22-14C-1.d	Gallic acid	Sample	8.783	349	10.7446		
22-14C-2.d	Gallic acid	Sample	8.783	368	12.4805		
22-14C-3.d	Gallic acid	Sample	8.775	373	12.9927		
22-15C-1.d	Gallic acid	Sample	8.783	2	ND		
22-15C-2.d	Gallic acid	Sample	8.808	4	ND		
22-15C-3.d	Gallic acid	Sample	8.766	5	ND		
22-16C-1.d	Gallic acid	Sample	8.766	1	ND		
22-16C-2.d	Gallic acid	Sample	8.791	3	ND		
22-16C-3.d	Gallic acid	Sample	8.775	2	ND		
22-17C-1.d	Gallic acid	Sample	8.741	4	ND		
22-17C-2.d	Gallic acid	Sample	8.825	24	ND		
22-17C-3.d	Gallic acid	Sample	8.716	23	ND		
22-18C-1.d	Gallic acid	Sample	8.808	10564	970.7891		
22-18C-2.d	Gallic acid	Sample	8.800	10855	998.1750		

Gallic acid

Data File	Compound	Sample Type	RT	Resp.	Final Conc	Exp. Conc	Accuracy
22-18C-3.d	Gallic acid	Sample	8.800	10920	1004.2407		

Protocatechuic acid

Data File	Compound	Sample Type	RT	Resp.	Final Conc	Exp. Conc	Accuracy
blank-061022-4.d	Protocatechuic acid	Sample	10.518	16	ND		
25ppb1.d	Protocatechuic acid	Calibration	10.501	40	ND		0.00
25ppb2.d	Protocatechuic acid	Calibration	10.956	18	ND		0.00
25ppb3.d	Protocatechuic acid	Calibration	10.426	204	7.9958		31.98
50ppb1.d	Protocatechuic acid	Calibration	10.509	533	39.8123		79.62
50ppb2.d	Protocatechuic acid	Calibration	10.509	486	35.2468		70.49
50ppb3.d	Protocatechuic acid	Calibration	10.526	521	38.6519		77.30
100ppb1.d	Protocatechuic acid	Calibration	10.534	1245	108.6862		108.69
100ppb2.d	Protocatechuic acid	Calibration	10.543	1198	104.1706		104.17
100ppb3.d	Protocatechuic acid	Calibration	10.543	1231	107.3067		107.31
250ppb1.d	Protocatechuic acid	Calibration	10.551	2690	248.4797		99.39
250ppb2.d	Protocatechuic acid	Calibration	10.543	2679	247.4624		98.98
250ppb3.d	Protocatechuic acid	Calibration	10.559	2684	247.9033		99.16
500ppb1.d	Protocatechuic acid	Calibration	10.568	5544	524.6661		104.93
500ppb2.d	Protocatechuic acid	Calibration	10.559	5576	527.7816		105.56
500ppb3.d	Protocatechuic acid	Calibration	10.568	5523	522.5972		104.52
1000ppb1.d	Protocatechuic acid	Calibration	10.576	10639	1017.6295		101.76
1000ppb2.d	Protocatechuic acid	Calibration	10.568	10071	962.6984		96.27
1000ppb3.d	Protocatechuic acid	Calibration	10.568	10291	983.9114		98.39
22-1A-1.d	Protocatechuic acid	Sample	10.576	17217	1654.0897		
22-1A-2.d	Protocatechuic acid	Sample	10.576	17741	1704.7977		
22-1A-3.d	Protocatechuic acid	Sample	10.568	18003	1730.1913		
22-2A-1.d	Protocatechuic acid	Sample	10.576	10188	973.9559		
22-2A-2.d	Protocatechuic acid	Sample	10.576	10263	981.2275		
22-2A-3.d	Protocatechuic acid	Sample	10.568	10206	975.7450		
22-3A-1.d	Protocatechuic acid	Sample	10.568	8330	794.2436		
22-3A-2.d	Protocatechuic acid	Sample	10.576	8417	802.5951		
22-3A-3.d	Protocatechuic acid	Sample	10.576	8469	807.6570		
22-4A-1.d	Protocatechuic acid	Sample	10.584	2830	262.0236		
22-4A-2.d	Protocatechuic acid	Sample	10.584	2815	260.6131		
22-4A-3.d	Protocatechuic acid	Sample	10.584	2768	256.0470		
22-5A-1.d	Protocatechuic acid	Sample	10.568	7934	755.9001		
22-5A-2.d	Protocatechuic acid	Sample	10.576	7858	748.5632		
22-5A-3.d	Protocatechuic acid	Sample	10.576	7937	756.1772		
22-6A-1.d	Protocatechuic acid	Sample	10.568	3229	300.6059		
22-6A-2.d	Protocatechuic acid	Sample	10.576	3161	294.0588		
22-6A-3.d	Protocatechuic acid	Sample	10.576	3298	307.2929		

Protocatechuic acid

Data File	Compound	Sample Type	RT	Resp.	Final Conc	Exp. Conc	Accuracy
22-7A-1.d	Protocatechuic acid	Sample	10.576	5680	537.8252		
22-7A-2.d	Protocatechuic acid	Sample	10.568	5941	563.0561		
22-7A-3.d	Protocatechuic acid	Sample	10.576	5776	547.1270		
22-8A-1.d	Protocatechuic acid	Sample	10.576	3482	325.0861		
22-8A-2.d	Protocatechuic acid	Sample	10.568	3678	344.0898		
22-8A-3.d	Protocatechuic acid	Sample	10.568	3487	325.5678		
22-9A-1.d	Protocatechuic acid	Sample	10.576	918	77.0649		
22-9A-2.d	Protocatechuic acid	Sample	10.584	934	78.6235		
22-9A-3.d	Protocatechuic acid	Sample	10.584	933	78.4823		
22-10A-1.d	Protocatechuic acid	Sample	10.576	1590	142.0682		
22-10A-2.d	Protocatechuic acid	Sample	10.584	1607	143.6945		
22-10A-3.d	Protocatechuic acid	Sample	10.576	1571	140.2593		
22-11A-1.d	Protocatechuic acid	Sample	10.576	519	38.3885		
22-11A-2.d	Protocatechuic acid	Sample	10.576	517	38.2749		
22-11A-3.d	Protocatechuic acid	Sample	10.576	540	40.4801		
22-12A-1.d	Protocatechuic acid	Sample	10.568	1080	92.7614		
22-12A-2.d	Protocatechuic acid	Sample	10.568	1125	97.0239		
22-12A-3.d	Protocatechuic acid	Sample	10.568	1066	91.3139		
22-13A-1.d	Protocatechuic acid	Sample	10.559	5311	502.1363		
22-13A-2.d	Protocatechuic acid	Sample	10.559	5270	498.1674		
22-13A-3.d	Protocatechuic acid	Sample	10.568	5402	510.9201		
22-14A-1.d	Protocatechuic acid	Sample	10.568	3277	305.2686		
22-14A-2.d	Protocatechuic acid	Sample	10.559	3247	302.3808		
22-14A-3.d	Protocatechuic acid	Sample	10.568	3342	311.5902		
22-15A-1.d	Protocatechuic acid	Sample	10.568	796	65.1982		
22-15A-2.d	Protocatechuic acid	Sample	10.559	817	67.2465		
22-15A-3.d	Protocatechuic acid	Sample	10.559	819	67.5074		
22-16A-1.d	Protocatechuic acid	Sample	10.559	1554	138.5899		
22-16A-2.d	Protocatechuic acid	Sample	10.568	1566	139.7726		
22-16A-3.d	Protocatechuic acid	Sample	10.568	1557	138.8356		
22-17A-1.d	Protocatechuic acid	Sample	10.559	1354	119.2309		
22-17A-2.d	Protocatechuic acid	Sample	10.551	1378	121.5842		
22-17A-3.d	Protocatechuic acid	Sample	10.551	1385	122.2275		
22-18A-1.d	Protocatechuic acid	Sample	10.551	63994	6180.1790		
22-18A-2.d	Protocatechuic acid	Sample	10.534	59669	5761.7566		
22-18A-3.d	Protocatechuic acid	Sample	10.543	58865	5683.9631		
22-1B-1.d	Protocatechuic acid	Sample	10.534	20886	2009.0865		
22-1B-2.d	Protocatechuic acid	Sample	10.543	20139	1936.8632		
22-1B-3.d	Protocatechuic acid	Sample	10.543	20957	2015.9426		
22-2B-1.d	Protocatechuic acid	Sample	10.543	11972	1146.5734		

Agilent Technologies

Protocatechuic acid

Data File	Compound	Sample Type	RT	Resp.	Final Conc	Exp. Conc	Accuracy
22-2B-2.d	Protocatechuic acid	Sample	10.534	12166	1165.3749		
22-2B-3.d	Protocatechuic acid	Sample	10.543	12057	1154.8617		
22-3B-1.d	Protocatechuic acid	Sample	10.543	11631	1113.6573		
22-3B-2.d	Protocatechuic acid	Sample	10.543	11461	1097.1311		
22-3B-3.d	Protocatechuic acid	Sample	10.543	11900	1139.6247		
22-4B-1.d	Protocatechuic acid	Sample	10.534	3414	318.5462		
22-4B-2.d	Protocatechuic acid	Sample	10.551	3299	307.3773		
22-4B-3.d	Protocatechuic acid	Sample	10.559	3102	288.4046		
22-5B-1.d	Protocatechuic acid	Sample	10.534	10682	1021.7539		
22-5B-2.d	Protocatechuic acid	Sample	10.543	10452	999.5430		
22-5B-3.d	Protocatechuic acid	Sample	10.543	11195	1071.3962		
22-6B-1.d	Protocatechuic acid	Sample	10.534	4814	453.9701		
22-6B-2.d	Protocatechuic acid	Sample	10.534	5032	475.1215		
22-6B-3.d	Protocatechuic acid	Sample	10.543	5061	477.8979		
22-7B-1.d	Protocatechuic acid	Sample	10.543	6947	660.3533		
22-7B-2.d	Protocatechuic acid	Sample	10.543	7342	698.6062		
22-7B-3.d	Protocatechuic acid	Sample	10.543	7353	699.7088		
22-8B-1.d	Protocatechuic acid	Sample	10.543	4619	435.1883		
22-8B-2.d	Protocatechuic acid	Sample	10.543	4647	437.8353		
22-8B-3.d	Protocatechuic acid	Sample	10.543	4795	452.1746		
22-9B-1.d	Protocatechuic acid	Sample	10.543	998	84.7537		
22-9B-2.d	Protocatechuic acid	Sample	10.551	1054	90.1893		
22-9B-3.d	Protocatechuic acid	Sample	10.543	1001	85.0624		
22-10B-1.d	Protocatechuic acid	Sample	10.551	1726	155.1790		
22-10B-2.d	Protocatechuic acid	Sample	10.543	1650	147.8990		
22-10B-3.d	Protocatechuic acid	Sample	10.543	1663	149.1135		
22-11B-1.d	Protocatechuic acid	Sample	10.543	616	47.8232		
22-11B-2.d	Protocatechuic acid	Sample	10.543	645	50.6439		
22-11B-3.d	Protocatechuic acid	Sample	10.543	568	43.1943		
22-12B-1.d	Protocatechuic acid	Sample	10.543	1210	105.3112		
22-12B-2.d	Protocatechuic acid	Sample	10.543	1273	111.3411		
22-12B-3.d	Protocatechuic acid	Sample	10.543	1256	109.7293		
22-13B-1.d	Protocatechuic acid	Sample	10.543	7187	683.6170		
22-13B-2.d	Protocatechuic acid	Sample	10.543	6992	664.7243		
22-13B-3.d	Protocatechuic acid	Sample	10.543	7304	694.8991		
22-14B-1.d	Protocatechuic acid	Sample	10.543	4084	383.3469		
22-14B-2.d	Protocatechuic acid	Sample	10.543	4320	406.2491		
22-14B-3.d	Protocatechuic acid	Sample	10.543	4148	389.5324		
22-15B-1.d	Protocatechuic acid	Sample	10.551	522	38.7392		
22-15B-2.d	Protocatechuic acid	Sample	10.543	551	41.5173		

Protocatechuic acid

Data File	Compound	Sample Type	RT	Resp.	Final Conc	Exp. Conc	Accuracy
22-15B-3.d	Protocatechuic acid	Sample	10.543	514	37.9121		
22-16B-1.d	Protocatechuic acid	Sample	10.543	1537	136.8856		
22-16B-2.d	Protocatechuic acid	Sample	10.543	1557	138.8944		
22-16B-3.d	Protocatechuic acid	Sample	10.543	1584	141.5147		
22-17B-1.d	Protocatechuic acid	Sample	10.543	1807	163.0716		
22-17B-2.d	Protocatechuic acid	Sample	10.551	1785	160.9445		
22-17B-3.d	Protocatechuic acid	Sample	10.543	1755	158.0508		
22-18B-1.d	Protocatechuic acid	Sample	10.543	12775	1224.3036		
22-18B-2.d	Protocatechuic acid	Sample	10.543	12979	1244.0789		
22-18B-3.d	Protocatechuic acid	Sample	10.543	13263	1271.4930		
22-1C-1.d	Protocatechuic acid	Sample	10.543	21085	2028.3251		
22-1C-2.d	Protocatechuic acid	Sample	10.543	21130	2032.7156		
22-1C-3.d	Protocatechuic acid	Sample	10.543	22138	2130.2143		
22-2C-1.d	Protocatechuic acid	Sample	10.534	10311	985.8616		
22-2C-2.d	Protocatechuic acid	Sample	10.534	10162	971.4307		
22-2C-3.d	Protocatechuic acid	Sample	10.543	10598	1013.6587		
22-3C-1.d	Protocatechuic acid	Sample	10.534	7667	730.0912		
22-3C-2.d	Protocatechuic acid	Sample	10.543	8678	827.9099		
22-3C-3.d	Protocatechuic acid	Sample	10.543	7977	760.0692		
22-4C-1.d	Protocatechuic acid	Sample	10.551	2957	274.3243		
22-4C-2.d	Protocatechuic acid	Sample	10.543	2841	263.0769		
22-4C-3.d	Protocatechuic acid	Sample	10.551	2948	273.4912		
22-5C-1.d	Protocatechuic acid	Sample	10.543	6888	654.6507		
22-5C-2.d	Protocatechuic acid	Sample	10.534	6839	649.9618		
22-5C-3.d	Protocatechuic acid	Sample	10.543	7220	686.7772		
22-6C-1.d	Protocatechuic acid	Sample	10.543	3821	357.9458		
22-6C-2.d	Protocatechuic acid	Sample	10.534	4131	387.9314		
22-6C-3.d	Protocatechuic acid	Sample	10.534	3872	362.8428		
22-7C-1.d	Protocatechuic acid	Sample	10.543	5472	517.7092		
22-7C-2.d	Protocatechuic acid	Sample	10.543	5477	518.1387		
22-7C-3.d	Protocatechuic acid	Sample	10.543	5477	518.1369		
22-8C-1.d	Protocatechuic acid	Sample	10.543	3329	310.3664		
22-8C-2.d	Protocatechuic acid	Sample	10.551	3157	293.7237		
22-8C-3.d	Protocatechuic acid	Sample	10.551	3368	314.1160		
22-9C-1.d	Protocatechuic acid	Sample	10.543	1082	92.9402		
22-9C-2.d	Protocatechuic acid	Sample	10.551	1087	93.3717		
22-9C-3.d	Protocatechuic acid	Sample	10.551	1138	98.3621		
22-10C-1.d	Protocatechuic acid	Sample	10.551	1836	165.8637		
22-10C-2.d	Protocatechuic acid	Sample	10.551	1850	167.1756		
22-10C-3.d	Protocatechuic acid	Sample	10.551	1859	168.1248		

Protocatechuic acid

Data File	Compound	Sample Type	RT	Resp.	Final Conc	Exp. Conc	Accuracy
22-11C-1.d	Protocatechuic acid	Sample	10.551	648	50.9424		
22-11C-2.d	Protocatechuic acid	Sample	10.559	631	49.2411		
22-11C-3.d	Protocatechuic acid	Sample	10.551	633	49.4299		
22-12C-1.d	Protocatechuic acid	Sample	10.551	935	78.7026		
22-12C-2.d	Protocatechuic acid	Sample	10.551	993	84.3157		
22-12C-3.d	Protocatechuic acid	Sample	10.568	884	73.7310		
22-13C-1.d	Protocatechuic acid	Sample	10.559	6496	616.7121		
22-13C-2.d	Protocatechuic acid	Sample	10.559	6507	617.8132		
22-13C-3.d	Protocatechuic acid	Sample	10.559	6531	620.1552		
22-14C-1.d	Protocatechuic acid	Sample	10.559	3246	302.3405		
22-14C-2.d	Protocatechuic acid	Sample	10.551	3378	315.0792		
22-14C-3.d	Protocatechuic acid	Sample	10.559	3220	299.7586		
22-15C-1.d	Protocatechuic acid	Sample	10.559	546	41.0202		
22-15C-2.d	Protocatechuic acid	Sample	10.559	527	39.1793		
22-15C-3.d	Protocatechuic acid	Sample	10.559	512	37.7295		
22-16C-1.d	Protocatechuic acid	Sample	10.568	1167	101.1620		
22-16C-2.d	Protocatechuic acid	Sample	10.568	1130	97.5682		
22-16C-3.d	Protocatechuic acid	Sample	10.568	1174	101.7654		
22-17C-1.d	Protocatechuic acid	Sample	10.568	1387	122.4527		
22-17C-2.d	Protocatechuic acid	Sample	10.568	1388	122.5486		
22-17C-3.d	Protocatechuic acid	Sample	10.576	1362	120.0006		
22-18C-1.d	Protocatechuic acid	Sample	10.576	142646	13790.4353		
22-18C-2.d	Protocatechuic acid	Sample	10.568	144748	13993.8836		
22-18C-3.d	Protocatechuic acid	Sample	10.568	141184	13648.9878		

Pyrocatechol

Data File	Compound	Sample Type	RT	Resp.	Final Conc	Exp. Conc	Accuracy
blank-061022-4.d	Pyrocatechol	Sample	10.899	0	ND		
25ppb1.d	Pyrocatechol	Calibration	10.772	2	ND		0.00
25ppb2.d	Pyrocatechol	Calibration	10.789	2	ND		0.00
25ppb3.d	Pyrocatechol	Calibration	10.856	3	ND		0.00
50ppb1.d	Pyrocatechol	Calibration	10.814	5	ND		0.00
50ppb2.d	Pyrocatechol	Calibration	10.856	26	70.4132		140.83
50ppb3.d	Pyrocatechol	Calibration	10.805	20	47.7965		95.59
100ppb1.d	Pyrocatechol	Calibration	10.805	36	100.4178		100.42
100ppb2.d	Pyrocatechol	Calibration	10.797	44	128.2957		128.30
100ppb3.d	Pyrocatechol	Calibration	10.805	32	87.7827		87.78
250ppb1.d	Pyrocatechol	Calibration	10.831	79	243.5675		97.43
250ppb2.d	Pyrocatechol	Calibration	10.814	78	240.7014		96.28
250ppb3.d	Pyrocatechol	Calibration	10.874	115	360.4919		144.20
500ppb1.d	Pyrocatechol	Calibration	10.831	151	479.3011		95.86

Pyrocatechol

Data File	Compound	Sample Type	RT	Resp.	Final Conc	Exp. Conc	Accuracy
500ppb2.d	Pyrocatechol	Calibration	10.831	151	480.0042		96.00
500ppb3.d	Pyrocatechol	Calibration	10.839	180	575.7839		115.16
1000ppb1.d	Pyrocatechol	Calibration	10.831	315	1020.7597		102.08
1000ppb2.d	Pyrocatechol	Calibration	10.822	309	1000.9603		100.10
1000ppb3.d	Pyrocatechol	Calibration	10.839	339	1099.9595		110.00
22-1A-1.d	Pyrocatechol	Sample	10.864	1	ND		
22-1A-2.d	Pyrocatechol	Sample	10.864	0	ND		
22-1A-3.d	Pyrocatechol	Sample	10.831	2	ND		
22-2A-1.d	Pyrocatechol	Sample	10.856	3	ND		
22-2A-2.d	Pyrocatechol	Sample	10.831	0	ND		
22-2A-3.d	Pyrocatechol	Sample	10.814	4	ND		
22-3A-1.d	Pyrocatechol	Sample	10.797	1	ND		
22-3A-2.d	Pyrocatechol	Sample	10.915	1	ND		
22-3A-3.d	Pyrocatechol	Sample	10.847	0	ND		
22-4A-1.d	Pyrocatechol	Sample	10.797	1	ND		
22-4A-2.d	Pyrocatechol	Sample	10.805	5	ND		
22-4A-3.d	Pyrocatechol	Sample	10.805	1	ND		
22-5A-1.d	Pyrocatechol	Sample	11.116	4	ND		
22-5A-2.d	Pyrocatechol	Sample	10.814	2	ND		
22-5A-3.d	Pyrocatechol	Sample	10.805	3	ND		
22-6A-1.d	Pyrocatechol	Sample	10.797	0	ND		
22-6A-2.d	Pyrocatechol	Sample	10.805	1	ND		
22-6A-3.d	Pyrocatechol	Sample	11.049	1	ND		
22-7A-1.d	Pyrocatechol	Sample	10.805	3	ND		
22-7A-2.d	Pyrocatechol	Sample	10.814	4	ND		
22-7A-3.d	Pyrocatechol	Sample	10.814	1	ND		
22-8A-1.d	Pyrocatechol	Sample	10.831	1	ND		
22-8A-2.d	Pyrocatechol	Sample	10.839	1	ND		
22-8A-3.d	Pyrocatechol	Sample	10.839	2	ND		
22-9A-1.d	Pyrocatechol	Sample	10.814	0	ND		
22-9A-2.d	Pyrocatechol	Sample	10.847	3	ND		
22-9A-3.d	Pyrocatechol	Sample	10.831	2	ND		
22-10A-1.d	Pyrocatechol	Sample	10.899	1	ND		
22-10A-2.d	Pyrocatechol	Sample	10.966	1	ND		
22-10A-3.d	Pyrocatechol	Sample	10.780	1	ND		
22-11A-1.d	Pyrocatechol	Sample	10.839	1	ND		
22-11A-2.d	Pyrocatechol	Sample	11.124	1	ND		
22-11A-3.d	Pyrocatechol	Sample	10.831	0	ND		
22-12A-1.d	Pyrocatechol	Sample	10.856	1	ND		
22-12A-2.d	Pyrocatechol	Sample	10.864	1	ND		

Pyrocatechol

Data File	Compound	Sample Type	RT	Resp.	Final Conc	Exp. Conc	Accuracy
22-12A-3.d	Pyrocatechol	Sample	10.822	0	ND		
22-13A-1.d	Pyrocatechol	Sample	10.940	4	ND		
22-13A-2.d	Pyrocatechol	Sample	10.831	0	ND		
22-13A-3.d	Pyrocatechol	Sample	10.780	2	ND		
22-14A-1.d	Pyrocatechol	Sample	10.856	4	ND		
22-14A-2.d	Pyrocatechol	Sample	10.814	1	ND		
22-14A-3.d	Pyrocatechol	Sample	10.831	5	ND		
22-15A-1.d	Pyrocatechol	Sample	10.814	0	ND		
22-15A-2.d	Pyrocatechol	Sample	10.822	1	ND		
22-15A-3.d	Pyrocatechol	Sample	11.074	2	ND		
22-16A-1.d	Pyrocatechol	Sample	10.789	0	ND		
22-16A-2.d	Pyrocatechol	Sample	10.814	1	ND		
22-16A-3.d	Pyrocatechol	Sample	10.949	2	ND		
22-17A-1.d	Pyrocatechol	Sample	10.814	0	ND		
22-17A-2.d	Pyrocatechol	Sample	10.839	1	ND		
22-17A-3.d	Pyrocatechol	Sample	10.831	1	ND		
22-18A-1.d	Pyrocatechol	Sample	10.822	2	ND		
22-18A-2.d	Pyrocatechol	Sample	10.822	2	ND		
22-18A-3.d	Pyrocatechol	Sample	10.805	2	ND		
22-1B-1.d	Pyrocatechol	Sample	10.847	3	ND		
22-1B-2.d	Pyrocatechol	Sample	10.856	0	ND		
22-1B-3.d	Pyrocatechol	Sample	10.814	1	ND		
22-2B-1.d	Pyrocatechol	Sample	10.831	6	3.6322		
22-2B-2.d	Pyrocatechol	Sample	10.797	6	3.4071		
22-2B-3.d	Pyrocatechol	Sample	10.839	6	3.1898		
22-3B-1.d	Pyrocatechol	Sample	10.856	1	ND		
22-3B-2.d	Pyrocatechol	Sample	10.780	3	ND		
22-3B-3.d	Pyrocatechol	Sample	10.899	2	ND		
22-4B-1.d	Pyrocatechol	Sample	10.814	2	ND		
22-4B-2.d	Pyrocatechol	Sample	10.864	1	ND		
22-4B-3.d	Pyrocatechol	Sample	10.831	3	ND		
22-5B-1.d	Pyrocatechol	Sample	10.822	3	ND		
22-5B-2.d	Pyrocatechol	Sample	10.924	5	ND		
22-5B-3.d	Pyrocatechol	Sample	10.814	0	ND		
22-6B-1.d	Pyrocatechol	Sample	10.847	2	ND		
22-6B-2.d	Pyrocatechol	Sample	10.847	1	ND		
22-6B-3.d	Pyrocatechol	Sample	10.856	1	ND		
22-7B-1.d	Pyrocatechol	Sample	10.856	1	ND		
22-7B-2.d	Pyrocatechol	Sample	10.856	1	ND		
22-7B-3.d	Pyrocatechol	Sample	10.847	1	ND		

Pyrocatechol

Data File	Compound	Sample Type	RT	Resp.	Final Conc	Exp. Conc	Accuracy
22-8B-1.d	Pyrocatechol	Sample	10.839	3	ND		
22-8B-2.d	Pyrocatechol	Sample	10.847	1	ND		
22-8B-3.d	Pyrocatechol	Sample	10.831	3	ND		
22-9B-1.d	Pyrocatechol	Sample	10.805	39	113.3361		
22-9B-2.d	Pyrocatechol	Sample	10.831	30	81.3051		
22-9B-3.d	Pyrocatechol	Sample	10.772	4	ND		
22-10B-1.d	Pyrocatechol	Sample	10.982	0	ND		
22-10B-2.d	Pyrocatechol	Sample	10.856	4	ND		
22-10B-3.d	Pyrocatechol	Sample	10.822	1	ND		
22-11B-1.d	Pyrocatechol	Sample	10.899	1	ND		
22-11B-2.d	Pyrocatechol	Sample	10.847	1	ND		
22-11B-3.d	Pyrocatechol	Sample	10.907	0	ND		
22-12B-1.d	Pyrocatechol	Sample	10.847	1	ND		
22-12B-2.d	Pyrocatechol	Sample	10.890	2	ND		
22-12B-3.d	Pyrocatechol	Sample	10.847	2	ND		
22-13B-1.d	Pyrocatechol	Sample	10.822	2	ND		
22-13B-2.d	Pyrocatechol	Sample	10.789	5	ND		
22-13B-3.d	Pyrocatechol	Sample	10.764	2	ND		
22-14B-1.d	Pyrocatechol	Sample	10.822	2	ND		
22-14B-2.d	Pyrocatechol	Sample	10.797	3	ND		
22-14B-3.d	Pyrocatechol	Sample	10.864	3	ND		
22-15B-1.d	Pyrocatechol	Sample	10.772	3	ND		
22-15B-2.d	Pyrocatechol	Sample	10.822	1	ND		
22-15B-3.d	Pyrocatechol	Sample	10.839	1	ND		
22-16B-1.d	Pyrocatechol	Sample	10.831	3	ND		
22-16B-2.d	Pyrocatechol	Sample	10.856	1	ND		
22-16B-3.d	Pyrocatechol	Sample	10.797	3	ND		
22-17B-1.d	Pyrocatechol	Sample	10.822	1	ND		
22-17B-2.d	Pyrocatechol	Sample	10.864	1	ND		
22-17B-3.d	Pyrocatechol	Sample	10.890	1	ND		
22-18B-1.d	Pyrocatechol	Sample	10.839	2	ND		
22-18B-2.d	Pyrocatechol	Sample	10.822	1	ND		
22-18B-3.d	Pyrocatechol	Sample	10.814	1	ND		
22-1C-1.d	Pyrocatechol	Sample	10.797	4	ND		
22-1C-2.d	Pyrocatechol	Sample	10.856	1	ND		
22-1C-3.d	Pyrocatechol	Sample	10.831	2	ND		
22-2C-1.d	Pyrocatechol	Sample	10.805	3	ND		
22-2C-2.d	Pyrocatechol	Sample	10.847	3	ND		
22-2C-3.d	Pyrocatechol	Sample	10.797	3	ND		
22-3C-1.d	Pyrocatechol	Sample	10.831	2	ND		

Agilent Technologies

Pyrocatechol

Data File	Compound	Sample Type	RT	Resp.	Final Conc	Exp. Conc	Accuracy
22-3C-2.d	Pyrocatechol	Sample	11.032	3	ND		
22-3C-3.d	Pyrocatechol	Sample	10.856	1	ND		
22-4C-1.d	Pyrocatechol	Sample	10.874	1	ND		
22-4C-2.d	Pyrocatechol	Sample	10.822	2	ND		
22-4C-3.d	Pyrocatechol	Sample	10.831	1	ND		
22-5C-1.d	Pyrocatechol	Sample	10.764	2	ND		
22-5C-2.d	Pyrocatechol	Sample	10.822	2	ND		
22-5C-3.d	Pyrocatechol	Sample	10.805	2	ND		
22-6C-1.d	Pyrocatechol	Sample	10.780	0	ND		
22-6C-2.d	Pyrocatechol	Sample	10.882	1	ND		
22-6C-3.d	Pyrocatechol	Sample	10.915	2	ND		
22-7C-1.d	Pyrocatechol	Sample	10.814	1	ND		
22-7C-2.d	Pyrocatechol	Sample	10.805	1	ND		
22-7C-3.d	Pyrocatechol	Sample	10.831	1	ND		
22-8C-1.d	Pyrocatechol	Sample	10.847	1	ND		
22-8C-2.d	Pyrocatechol	Sample	10.831	1	ND		
22-8C-3.d	Pyrocatechol	Sample	10.864	0	ND		
22-9C-1.d	Pyrocatechol	Sample	10.932	4	ND		
22-9C-2.d	Pyrocatechol	Sample	10.822	2	ND		
22-9C-3.d	Pyrocatechol	Sample	10.814	2	ND		
22-10C-1.d	Pyrocatechol	Sample	10.847	1	ND		
22-10C-2.d	Pyrocatechol	Sample	10.814	1	ND		
22-10C-3.d	Pyrocatechol	Sample	10.864	1	ND		
22-11C-1.d	Pyrocatechol	Sample	10.864	3	ND		
22-11C-2.d	Pyrocatechol	Sample	10.839	0	ND		
22-11C-3.d	Pyrocatechol	Sample	10.847	1	ND		
22-12C-1.d	Pyrocatechol	Sample	11.082	1	ND		
22-12C-2.d	Pyrocatechol	Sample	10.907	1	ND		
22-12C-3.d	Pyrocatechol	Sample	10.780	1	ND		
22-13C-1.d	Pyrocatechol	Sample	10.814	1	ND		
22-13C-2.d	Pyrocatechol	Sample	10.882	1	ND		
22-13C-3.d	Pyrocatechol	Sample	10.814	1	ND		
22-14C-1.d	Pyrocatechol	Sample	10.882	2	ND		
22-14C-2.d	Pyrocatechol	Sample	10.847	2	ND		
22-14C-3.d	Pyrocatechol	Sample	10.864	1	ND		
22-15C-1.d	Pyrocatechol	Sample	10.805	3	ND		
22-15C-2.d	Pyrocatechol	Sample	10.864	4	ND		
22-15C-3.d	Pyrocatechol	Sample	10.797	0	ND		
22-16C-1.d	Pyrocatechol	Sample	10.822	1	ND		
22-16C-2.d	Pyrocatechol	Sample	10.856	0	ND		

Pyrocatechol

Data File	Compound	Sample Type	RT	Resp.	Final Conc	Exp. Conc	Accuracy
22-16C-3.d	Pyrocatechol	Sample	10.814	0	ND		
22-17C-1.d	Pyrocatechol	Sample	10.847	5	ND		
22-17C-2.d	Pyrocatechol	Sample	10.780	2	ND		
22-17C-3.d	Pyrocatechol	Sample	10.831	1	ND		
22-18C-1.d	Pyrocatechol	Sample	10.839	8	8.6925		
22-18C-2.d	Pyrocatechol	Sample	10.814	9	11.4106		
22-18C-3.d	Pyrocatechol	Sample	10.874	8	8.1532		

3,4-Dihydroxyphenylacetic acid

Data File	Compound	Sample Type	RT	Resp.	Final Conc	Exp. Conc	Accuracy
blank-061022-4.d	3,4-Dihydroxyphenylacetic acid	Sample	10.954	6	ND		
25ppb1.d	3,4-Dihydroxyphenylacetic acid	Calibration	10.879	3	ND		0.00
25ppb2.d	3,4-Dihydroxyphenylacetic acid	Calibration	10.818	5	ND		0.00
25ppb3.d	3,4-Dihydroxyphenylacetic acid	Calibration	10.760	112	5.2926		21.17
50ppb1.d	3,4-Dihydroxyphenylacetic acid	Calibration	10.826	331	32.9234		65.85
50ppb2.d	3,4-Dihydroxyphenylacetic acid	Calibration	10.843	364	37.0830		74.17
50ppb3.d	3,4-Dihydroxyphenylacetic acid	Calibration	10.843	387	39.9036		79.81
100ppb1.d	3,4-Dihydroxyphenylacetic acid	Calibration	10.860	909	105.7820		105.78
100ppb2.d	3,4-Dihydroxyphenylacetic acid	Calibration	10.860	926	107.9185		107.92
100ppb3.d	3,4-Dihydroxyphenylacetic acid	Calibration	10.860	966	113.0111		113.01
250ppb1.d	3,4-Dihydroxyphenylacetic acid	Calibration	10.871	2023	246.3737		98.55
250ppb2.d	3,4-Dihydroxyphenylacetic acid	Calibration	10.860	2093	255.2710		102.11
250ppb3.d	3,4-Dihydroxyphenylacetic acid	Calibration	10.871	2067	252.0275		100.81
500ppb1.d	3,4-Dihydroxyphenylacetic acid	Calibration	10.879	4201	521.2669		104.25
500ppb2.d	3,4-Dihydroxyphenylacetic acid	Calibration	10.879	4171	517.5872		103.52
500ppb3.d	3,4-Dihydroxyphenylacetic acid	Calibration	10.879	4208	522.1573		104.43
1000ppb1.d	3,4-Dihydroxyphenylacetic acid	Calibration	10.888	8499	1063.7717		106.38
1000ppb2.d	3,4-Dihydroxyphenylacetic acid	Calibration	10.888	7824	978.6204		97.86
1000ppb3.d	3,4-Dihydroxyphenylacetic acid	Calibration	10.879	7913	989.7819		98.98
22-1A-1.d	3,4-Dihydroxyphenylacetic acid	Sample	10.896	78	0.9926		
22-1A-2.d	3,4-Dihydroxyphenylacetic acid	Sample	10.888	81	1.3567		
22-1A-3.d	3,4-Dihydroxyphenylacetic acid	Sample	10.860	78	0.9177		
22-2A-1.d	3,4-Dihydroxyphenylacetic acid	Sample	10.879	9	ND		
22-2A-2.d	3,4-Dihydroxyphenylacetic acid	Sample	10.879	37	ND		
22-2A-3.d	3,4-Dihydroxyphenylacetic acid	Sample	10.851	23	ND		
22-3A-1.d	3,4-Dihydroxyphenylacetic acid	Sample	10.871	29	ND		
22-3A-2.d	3,4-Dihydroxyphenylacetic acid	Sample	10.843	24	ND		
22-3A-3.d	3,4-Dihydroxyphenylacetic acid	Sample	10.896	28	ND		
22-4A-1.d	3,4-Dihydroxyphenylacetic acid	Sample	10.913	30	ND		
22-4A-2.d	3,4-Dihydroxyphenylacetic acid	Sample	10.871	24	ND		
22-4A-3.d	3,4-Dihydroxyphenylacetic acid	Sample	10.860	27	ND		

3,4-Dihydroxyphenylacetic acid

Data File	Compound	Sample Type	RT	Resp.	Final Conc	Exp. Conc	Accuracy
22-5A-1.d	3,4-Dihydroxyphenylacetic acid	Sample	10.879	34	ND		
22-5A-2.d	3,4-Dihydroxyphenylacetic acid	Sample	10.879	31	ND		
22-5A-3.d	3,4-Dihydroxyphenylacetic acid	Sample	10.896	32	ND		
22-6A-1.d	3,4-Dihydroxyphenylacetic acid	Sample	10.904	7	ND		
22-6A-2.d	3,4-Dihydroxyphenylacetic acid	Sample	10.913	3	ND		
22-6A-3.d	3,4-Dihydroxyphenylacetic acid	Sample	10.904	13	ND		
22-7A-1.d	3,4-Dihydroxyphenylacetic acid	Sample	10.879	32	ND		
22-7A-2.d	3,4-Dihydroxyphenylacetic acid	Sample	10.871	33	ND		
22-7A-3.d	3,4-Dihydroxyphenylacetic acid	Sample	10.888	36	ND		
22-8A-1.d	3,4-Dihydroxyphenylacetic acid	Sample	10.879	23	ND		
22-8A-2.d	3,4-Dihydroxyphenylacetic acid	Sample	10.879	42	ND		
22-8A-3.d	3,4-Dihydroxyphenylacetic acid	Sample	10.904	34	ND		
22-9A-1.d	3,4-Dihydroxyphenylacetic acid	Sample	10.879	141	8.9331		
22-9A-2.d	3,4-Dihydroxyphenylacetic acid	Sample	10.896	122	6.4734		
22-9A-3.d	3,4-Dihydroxyphenylacetic acid	Sample	10.871	131	7.6580		
22-10A-1.d	3,4-Dihydroxyphenylacetic acid	Sample	10.879	18	ND		
22-10A-2.d	3,4-Dihydroxyphenylacetic acid	Sample	10.896	19	ND		
22-10A-3.d	3,4-Dihydroxyphenylacetic acid	Sample	10.871	13	ND		
22-11A-1.d	3,4-Dihydroxyphenylacetic acid	Sample	10.896	45	ND		
22-11A-2.d	3,4-Dihydroxyphenylacetic acid	Sample	10.896	26	ND		
22-11A-3.d	3,4-Dihydroxyphenylacetic acid	Sample	10.871	18	ND		
22-12A-1.d	3,4-Dihydroxyphenylacetic acid	Sample	10.851	28	ND		
22-12A-2.d	3,4-Dihydroxyphenylacetic acid	Sample	10.904	17	ND		
22-12A-3.d	3,4-Dihydroxyphenylacetic acid	Sample	10.879	8	ND		
22-13A-1.d	3,4-Dihydroxyphenylacetic acid	Sample	10.879	91	2.6357		
22-13A-2.d	3,4-Dihydroxyphenylacetic acid	Sample	10.871	90	2.4751		
22-13A-3.d	3,4-Dihydroxyphenylacetic acid	Sample	10.888	84	1.7588		
22-14A-1.d	3,4-Dihydroxyphenylacetic acid	Sample	10.879	110	4.9726		
22-14A-2.d	3,4-Dihydroxyphenylacetic acid	Sample	10.879	114	5.4334		
22-14A-3.d	3,4-Dihydroxyphenylacetic acid	Sample	10.888	117	5.8945		
22-15A-1.d	3,4-Dihydroxyphenylacetic acid	Sample	10.996	17	ND		
22-15A-2.d	3,4-Dihydroxyphenylacetic acid	Sample	11.046	2	ND		
22-15A-3.d	3,4-Dihydroxyphenylacetic acid	Sample	11.355	3	ND		
22-16A-1.d	3,4-Dihydroxyphenylacetic acid	Sample	10.896	5	ND		
22-16A-2.d	3,4-Dihydroxyphenylacetic acid	Sample	10.851	18	ND		
22-16A-3.d	3,4-Dihydroxyphenylacetic acid	Sample	10.879	21	ND		
22-17A-1.d	3,4-Dihydroxyphenylacetic acid	Sample	10.871	2	ND		
22-17A-2.d	3,4-Dihydroxyphenylacetic acid	Sample	11.121	8	ND		
22-17A-3.d	3,4-Dihydroxyphenylacetic acid	Sample	10.888	4	ND		
22-18A-1.d	3,4-Dihydroxyphenylacetic acid	Sample	10.860	360	36.5138		

Agilent Technologies

3,4-Dihydroxyphenylacetic acid

Data File	Compound	Sample Type	RT	Resp.	Final Conc	Exp. Conc	Accuracy
22-18A-2.d	3,4-Dihydroxyphenylacetic acid	Sample	10.843	365	37.2284		
22-18A-3.d	3,4-Dihydroxyphenylacetic acid	Sample	10.860	353	35.6748		
22-1B-1.d	3,4-Dihydroxyphenylacetic acid	Sample	10.851	118	5.9541		
22-1B-2.d	3,4-Dihydroxyphenylacetic acid	Sample	10.843	111	5.1004		
22-1B-3.d	3,4-Dihydroxyphenylacetic acid	Sample	10.843	117	5.8962		
22-2B-1.d	3,4-Dihydroxyphenylacetic acid	Sample	10.860	47	ND		
22-2B-2.d	3,4-Dihydroxyphenylacetic acid	Sample	10.835	53	ND		
22-2B-3.d	3,4-Dihydroxyphenylacetic acid	Sample	10.843	44	ND		
22-3B-1.d	3,4-Dihydroxyphenylacetic acid	Sample	10.835	51	ND		
22-3B-2.d	3,4-Dihydroxyphenylacetic acid	Sample	10.851	51	ND		
22-3B-3.d	3,4-Dihydroxyphenylacetic acid	Sample	10.843	68	ND		
22-4B-1.d	3,4-Dihydroxyphenylacetic acid	Sample	10.851	63	ND		
22-4B-2.d	3,4-Dihydroxyphenylacetic acid	Sample	10.860	61	ND		
22-4B-3.d	3,4-Dihydroxyphenylacetic acid	Sample	10.860	70	ND		
22-5B-1.d	3,4-Dihydroxyphenylacetic acid	Sample	10.851	62	ND		
22-5B-2.d	3,4-Dihydroxyphenylacetic acid	Sample	10.879	56	ND		
22-5B-3.d	3,4-Dihydroxyphenylacetic acid	Sample	10.851	47	ND		
22-6B-1.d	3,4-Dihydroxyphenylacetic acid	Sample	10.860	45	ND		
22-6B-2.d	3,4-Dihydroxyphenylacetic acid	Sample	10.888	58	ND		
22-6B-3.d	3,4-Dihydroxyphenylacetic acid	Sample	10.851	29	ND		
22-7B-1.d	3,4-Dihydroxyphenylacetic acid	Sample	10.843	64	ND		
22-7B-2.d	3,4-Dihydroxyphenylacetic acid	Sample	10.860	54	ND		
22-7B-3.d	3,4-Dihydroxyphenylacetic acid	Sample	10.826	43	ND		
22-8B-1.d	3,4-Dihydroxyphenylacetic acid	Sample	10.860	39	ND		
22-8B-2.d	3,4-Dihydroxyphenylacetic acid	Sample	10.843	53	ND		
22-8B-3.d	3,4-Dihydroxyphenylacetic acid	Sample	10.860	46	ND		
22-9B-1.d	3,4-Dihydroxyphenylacetic acid	Sample	10.851	166	12.0736		
22-9B-2.d	3,4-Dihydroxyphenylacetic acid	Sample	10.860	166	11.9941		
22-9B-3.d	3,4-Dihydroxyphenylacetic acid	Sample	10.860	169	12.4500		
22-10B-1.d	3,4-Dihydroxyphenylacetic acid	Sample	10.860	14	ND		
22-10B-2.d	3,4-Dihydroxyphenylacetic acid	Sample	10.879	16	ND		
22-10B-3.d	3,4-Dihydroxyphenylacetic acid	Sample	10.851	24	ND		
22-11B-1.d	3,4-Dihydroxyphenylacetic acid	Sample	10.860	40	ND		
22-11B-2.d	3,4-Dihydroxyphenylacetic acid	Sample	10.851	53	ND		
22-11B-3.d	3,4-Dihydroxyphenylacetic acid	Sample	10.851	45	ND		
22-12B-1.d	3,4-Dihydroxyphenylacetic acid	Sample	10.851	211	17.7920		
22-12B-2.d	3,4-Dihydroxyphenylacetic acid	Sample	10.843	288	27.3927		
22-12B-3.d	3,4-Dihydroxyphenylacetic acid	Sample	10.871	217	18.4756		
22-13B-1.d	3,4-Dihydroxyphenylacetic acid	Sample	10.860	163	11.6690		
22-13B-2.d	3,4-Dihydroxyphenylacetic acid	Sample	10.851	148	9.7480		

3,4-Dihydroxyphenylacetic acid

Data File	Compound	Sample Type	RT	Resp.	Final Conc	Exp. Conc	Accuracy
22-13B-3.d	3,4-Dihydroxyphenylacetic acid	Sample	10.860	167	12.1925		
22-14B-1.d	3,4-Dihydroxyphenylacetic acid	Sample	10.843	313	30.6643		
22-14B-2.d	3,4-Dihydroxyphenylacetic acid	Sample	10.843	310	30.1948		
22-14B-3.d	3,4-Dihydroxyphenylacetic acid	Sample	10.851	340	33.9557		
22-15B-1.d	3,4-Dihydroxyphenylacetic acid	Sample	10.954	6	ND		
22-15B-2.d	3,4-Dihydroxyphenylacetic acid	Sample	10.843	11	ND		
22-15B-3.d	3,4-Dihydroxyphenylacetic acid	Sample	10.954	7	ND		
22-16B-1.d	3,4-Dihydroxyphenylacetic acid	Sample	10.871	23	ND		
22-16B-2.d	3,4-Dihydroxyphenylacetic acid	Sample	10.913	58	ND		
22-16B-3.d	3,4-Dihydroxyphenylacetic acid	Sample	10.938	4	ND		
22-17B-1.d	3,4-Dihydroxyphenylacetic acid	Sample	11.038	10	ND		
22-17B-2.d	3,4-Dihydroxyphenylacetic acid	Sample	10.871	4	ND		
22-17B-3.d	3,4-Dihydroxyphenylacetic acid	Sample	10.888	8	ND		
22-18B-1.d	3,4-Dihydroxyphenylacetic acid	Sample	10.860	84	1.7300		
22-18B-2.d	3,4-Dihydroxyphenylacetic acid	Sample	10.835	87	2.1189		
22-18B-3.d	3,4-Dihydroxyphenylacetic acid	Sample	10.851	79	1.0541		
22-1C-1.d	3,4-Dihydroxyphenylacetic acid	Sample	10.835	93	2.8613		
22-1C-2.d	3,4-Dihydroxyphenylacetic acid	Sample	10.843	98	3.4555		
22-1C-3.d	3,4-Dihydroxyphenylacetic acid	Sample	10.843	92	2.7770		
22-2C-1.d	3,4-Dihydroxyphenylacetic acid	Sample	10.835	38	ND		
22-2C-2.d	3,4-Dihydroxyphenylacetic acid	Sample	10.851	38	ND		
22-2C-3.d	3,4-Dihydroxyphenylacetic acid	Sample	10.871	30	ND		
22-3C-1.d	3,4-Dihydroxyphenylacetic acid	Sample	10.860	38	ND		
22-3C-2.d	3,4-Dihydroxyphenylacetic acid	Sample	10.843	49	ND		
22-3C-3.d	3,4-Dihydroxyphenylacetic acid	Sample	10.904	5	ND		
22-4C-1.d	3,4-Dihydroxyphenylacetic acid	Sample	10.835	38	ND		
22-4C-2.d	3,4-Dihydroxyphenylacetic acid	Sample	10.851	30	ND		
22-4C-3.d	3,4-Dihydroxyphenylacetic acid	Sample	10.843	54	ND		
22-5C-1.d	3,4-Dihydroxyphenylacetic acid	Sample	10.871	48	ND		
22-5C-2.d	3,4-Dihydroxyphenylacetic acid	Sample	10.871	41	ND		
22-5C-3.d	3,4-Dihydroxyphenylacetic acid	Sample	10.843	28	ND		
22-6C-1.d	3,4-Dihydroxyphenylacetic acid	Sample	10.843	4	ND		
22-6C-2.d	3,4-Dihydroxyphenylacetic acid	Sample	10.871	44	ND		
22-6C-3.d	3,4-Dihydroxyphenylacetic acid	Sample	10.913	22	ND		
22-7C-1.d	3,4-Dihydroxyphenylacetic acid	Sample	10.860	57	ND		
22-7C-2.d	3,4-Dihydroxyphenylacetic acid	Sample	10.843	46	ND		
22-7C-3.d	3,4-Dihydroxyphenylacetic acid	Sample	10.871	29	ND		
22-8C-1.d	3,4-Dihydroxyphenylacetic acid	Sample	10.879	32	ND		
22-8C-2.d	3,4-Dihydroxyphenylacetic acid	Sample	10.871	16	ND		
22-8C-3.d	3,4-Dihydroxyphenylacetic acid	Sample	10.843	9	ND		

3,4-Dihydroxyphenylacetic acid

Data File	Compound	Sample Type	RT	Resp.	Final Conc	Exp. Conc	Accuracy
22-9C-1.d	3,4-Dihydroxyphenylacetic acid	Sample	10.843	170	12.5535		
22-9C-2.d	3,4-Dihydroxyphenylacetic acid	Sample	10.860	163	11.6869		
22-9C-3.d	3,4-Dihydroxyphenylacetic acid	Sample	10.851	172	12.7680		
22-10C-1.d	3,4-Dihydroxyphenylacetic acid	Sample	10.913	19	ND		
22-10C-2.d	3,4-Dihydroxyphenylacetic acid	Sample	10.871	29	ND		
22-10C-3.d	3,4-Dihydroxyphenylacetic acid	Sample	10.879	16	ND		
22-11C-1.d	3,4-Dihydroxyphenylacetic acid	Sample	10.879	39	ND		
22-11C-2.d	3,4-Dihydroxyphenylacetic acid	Sample	10.888	40	ND		
22-11C-3.d	3,4-Dihydroxyphenylacetic acid	Sample	10.860	27	ND		
22-12C-1.d	3,4-Dihydroxyphenylacetic acid	Sample	10.871	138	8.4772		
22-12C-2.d	3,4-Dihydroxyphenylacetic acid	Sample	10.860	143	9.1751		
22-12C-3.d	3,4-Dihydroxyphenylacetic acid	Sample	10.871	127	7.0847		
22-13C-1.d	3,4-Dihydroxyphenylacetic acid	Sample	10.860	167	12.1366		
22-13C-2.d	3,4-Dihydroxyphenylacetic acid	Sample	10.871	171	12.6773		
22-13C-3.d	3,4-Dihydroxyphenylacetic acid	Sample	10.871	171	12.7468		
22-14C-1.d	3,4-Dihydroxyphenylacetic acid	Sample	10.860	161	11.4641		
22-14C-2.d	3,4-Dihydroxyphenylacetic acid	Sample	10.871	169	12.4387		
22-14C-3.d	3,4-Dihydroxyphenylacetic acid	Sample	10.879	176	13.3707		
22-15C-1.d	3,4-Dihydroxyphenylacetic acid	Sample	10.921	5	ND		
22-15C-2.d	3,4-Dihydroxyphenylacetic acid	Sample	10.888	1	ND		
22-15C-3.d	3,4-Dihydroxyphenylacetic acid	Sample	10.810	2	ND		
22-16C-1.d	3,4-Dihydroxyphenylacetic acid	Sample	11.013	6	ND		
22-16C-2.d	3,4-Dihydroxyphenylacetic acid	Sample	10.946	2	ND		
22-16C-3.d	3,4-Dihydroxyphenylacetic acid	Sample	11.021	6	ND		
22-17C-1.d	3,4-Dihydroxyphenylacetic acid	Sample	10.921	6	ND		
22-17C-2.d	3,4-Dihydroxyphenylacetic acid	Sample	10.879	7	ND		
22-17C-3.d	3,4-Dihydroxyphenylacetic acid	Sample	10.904	5	ND		
22-18C-1.d	3,4-Dihydroxyphenylacetic acid	Sample	10.871	295	28.3504		
22-18C-2.d	3,4-Dihydroxyphenylacetic acid	Sample	10.879	310	30.1866		
22-18C-3.d	3,4-Dihydroxyphenylacetic acid	Sample	10.871	295	28.3061		

(+)-Catechin

Data File	Compound	Sample Type	RT	Resp.	Final Conc	Exp. Conc	Accuracy
blank-061022-4.d	(+)-Catechin	Sample	11.277	4	ND		
25ppb1.d	(+)-Catechin	Calibration	11.386	48	ND		0.00
25ppb2.d	(+)-Catechin	Calibration	11.336	60	ND		0.00
25ppb3.d	(+)-Catechin	Calibration	11.202	111	4.1059		16.42
50ppb1.d	(+)-Catechin	Calibration	11.261	192	28.8208		57.64
50ppb2.d	(+)-Catechin	Calibration	11.261	198	30.8558		61.71
50ppb3.d	(+)-Catechin	Calibration	11.277	229	40.4031		80.81
100ppb1.d	(+)-Catechin	Calibration	11.286	473	115.7913		115.79

Quantitative Analysis Complete Report

(+)-Catechin

Data File	Compound	Sample Type	RT	Resp.	Final Conc	Exp. Conc	Accuracy
100ppb2.d	(+)-Catechin	Calibration	11.277	506	125.8558		125.86
100ppb3.d	(+)-Catechin	Calibration	11.294	403	94.1036		94.10
250ppb1.d	(+)-Catechin	Calibration	11.294	1006	280.2250		112.09
250ppb2.d	(+)-Catechin	Calibration	11.286	994	276.3034		110.52
250ppb3.d	(+)-Catechin	Calibration	11.302	959	265.7187		106.29
500ppb1.d	(+)-Catechin	Calibration	11.302	1764	514.1201		102.82
500ppb2.d	(+)-Catechin	Calibration	11.294	1869	546.2426		109.25
500ppb3.d	(+)-Catechin	Calibration	11.286	1635	474.1699		94.83
1000ppb1.d	(+)-Catechin	Calibration	11.302	3351	1003.7100		100.37
1000ppb2.d	(+)-Catechin	Calibration	11.302	3317	993.0585		99.31
1000ppb3.d	(+)-Catechin	Calibration	11.302	3236	968.2655		96.83
22-1A-1.d	(+)-Catechin	Sample	11.119	8	ND		
22-1A-2.d	(+)-Catechin	Sample	11.227	7	ND		
22-1A-3.d	(+)-Catechin	Sample	11.294	2	ND		
22-2A-1.d	(+)-Catechin	Sample	11.160	11	ND		
22-2A-2.d	(+)-Catechin	Sample	11.411	42	ND		
22-2A-3.d	(+)-Catechin	Sample	11.361	32	ND		
22-3A-1.d	(+)-Catechin	Sample	11.344	40	ND		
22-3A-2.d	(+)-Catechin	Sample	11.344	35	ND		
22-3A-3.d	(+)-Catechin	Sample	11.336	36	ND		
22-4A-1.d	(+)-Catechin	Sample	11.277	18	ND		
22-4A-2.d	(+)-Catechin	Sample	11.319	62	ND		
22-4A-3.d	(+)-Catechin	Sample	11.319	28	ND		
22-5A-1.d	(+)-Catechin	Sample	11.436	69	ND		
22-5A-2.d	(+)-Catechin	Sample	11.369	42	ND		
22-5A-3.d	(+)-Catechin	Sample	11.319	15	ND		
22-6A-1.d	(+)-Catechin	Sample	11.319	37	ND		
22-6A-2.d	(+)-Catechin	Sample	11.294	43	ND		
22-6A-3.d	(+)-Catechin	Sample	11.319	84	ND		
22-7A-1.d	(+)-Catechin	Sample	11.302	163	19.8995		
22-7A-2.d	(+)-Catechin	Sample	11.294	156	17.8249		
22-7A-3.d	(+)-Catechin	Sample	11.311	183	26.2273		
22-8A-1.d	(+)-Catechin	Sample	11.294	59	ND		
22-8A-2.d	(+)-Catechin	Sample	11.135	16	ND		
22-8A-3.d	(+)-Catechin	Sample	11.102	3	ND		
22-9A-1.d	(+)-Catechin	Sample	11.302	3486	1045.4275		
22-9A-2.d	(+)-Catechin	Sample	11.302	3812	1145.8934		
22-9A-3.d	(+)-Catechin	Sample	11.294	3732	1121.1599		
22-10A-1.d	(+)-Catechin	Sample	11.344	7	ND		
22-10A-2.d	(+)-Catechin	Sample	11.336	34	ND		

(+)-Catechin

Data File	Compound	Sample Type	RT	Resp.	Final Conc	Exp. Conc	Accuracy
22-10A-3.d	(+)-Catechin	Sample	11.353	89	ND		
22-11A-1.d	(+)-Catechin	Sample	11.311	64	ND		
22-11A-2.d	(+)-Catechin	Sample	11.336	9	ND		
22-11A-3.d	(+)-Catechin	Sample	11.336	63	ND		
22-12A-1.d	(+)-Catechin	Sample	11.202	10	ND		
22-12A-2.d	(+)-Catechin	Sample	11.353	18	ND		
22-12A-3.d	(+)-Catechin	Sample	11.428	46	ND		
22-13A-1.d	(+)-Catechin	Sample	11.369	6	ND		
22-13A-2.d	(+)-Catechin	Sample	11.194	6	ND		
22-13A-3.d	(+)-Catechin	Sample	11.361	34	ND		
22-14A-1.d	(+)-Catechin	Sample	11.436	57	ND		
22-14A-2.d	(+)-Catechin	Sample	11.244	7	ND		
22-14A-3.d	(+)-Catechin	Sample	11.344	17	ND		
22-15A-1.d	(+)-Catechin	Sample	11.236	1	ND		
22-15A-2.d	(+)-Catechin	Sample	11.419	2	ND		
22-15A-3.d	(+)-Catechin	Sample	11.378	37	ND		
22-16A-1.d	(+)-Catechin	Sample	11.202	3	ND		
22-16A-2.d	(+)-Catechin	Sample	11.261	3	ND		
22-16A-3.d	(+)-Catechin	Sample	11.261	5	ND		
22-17A-1.d	(+)-Catechin	Sample	11.177	10	ND		
22-17A-2.d	(+)-Catechin	Sample	11.244	6	ND		
22-17A-3.d	(+)-Catechin	Sample	11.244	4	ND		
22-18A-1.d	(+)-Catechin	Sample	11.378	60	ND		
22-18A-2.d	(+)-Catechin	Sample	11.294	31	ND		
22-18A-3.d	(+)-Catechin	Sample	11.562	9	ND		
22-1B-1.d	(+)-Catechin	Sample	11.470	67	ND		
22-1B-2.d	(+)-Catechin	Sample	11.386	35	ND		
22-1B-3.d	(+)-Catechin	Sample	11.160	11	ND		
22-2B-1.d	(+)-Catechin	Sample	11.344	19	ND		
22-2B-2.d	(+)-Catechin	Sample	11.403	41	ND		
22-2B-3.d	(+)-Catechin	Sample	11.403	47	ND		
22-3B-1.d	(+)-Catechin	Sample	11.277	12	ND		
22-3B-2.d	(+)-Catechin	Sample	11.269	14	ND		
22-3B-3.d	(+)-Catechin	Sample	11.277	12	ND		
22-4B-1.d	(+)-Catechin	Sample	11.328	41	ND		
22-4B-2.d	(+)-Catechin	Sample	11.277	10	ND		
22-4B-3.d	(+)-Catechin	Sample	11.261	9	ND		
22-5B-1.d	(+)-Catechin	Sample	11.219	12	ND		
22-5B-2.d	(+)-Catechin	Sample	11.445	38	ND		
22-5B-3.d	(+)-Catechin	Sample	11.378	43	ND		

(+)-Catechin

Data File	Compound	Sample Type	RT	Resp.	Final Conc	Exp. Conc	Accuracy
22-6B-1.d	(+)-Catechin	Sample	11.353	11	ND		
22-6B-2.d	(+)-Catechin	Sample	11.135	14	ND		
22-6B-3.d	(+)-Catechin	Sample	11.252	36	ND		
22-7B-1.d	(+)-Catechin	Sample	11.261	235	42.3714		
22-7B-2.d	(+)-Catechin	Sample	11.261	223	38.6185		
22-7B-3.d	(+)-Catechin	Sample	11.261	300	62.2769		
22-8B-1.d	(+)-Catechin	Sample	11.336	71	ND		
22-8B-2.d	(+)-Catechin	Sample	11.361	68	ND		
22-8B-3.d	(+)-Catechin	Sample	11.261	6	ND		
22-9B-1.d	(+)-Catechin	Sample	11.269	4375	1319.6729		
22-9B-2.d	(+)-Catechin	Sample	11.277	4148	1249.6520		
22-9B-3.d	(+)-Catechin	Sample	11.277	4434	1337.6542		
22-10B-1.d	(+)-Catechin	Sample	11.277	18	ND		
22-10B-2.d	(+)-Catechin	Sample	11.244	13	ND		
22-10B-3.d	(+)-Catechin	Sample	11.261	47	ND		
22-11B-1.d	(+)-Catechin	Sample	11.378	13	ND		
22-11B-2.d	(+)-Catechin	Sample	11.353	8	ND		
22-11B-3.d	(+)-Catechin	Sample	11.269	19	ND		
22-12B-1.d	(+)-Catechin	Sample	11.361	54	ND		
22-12B-2.d	(+)-Catechin	Sample	11.169	3	ND		
22-12B-3.d	(+)-Catechin	Sample	11.211	4	ND		
22-13B-1.d	(+)-Catechin	Sample	11.344	27	ND		
22-13B-2.d	(+)-Catechin	Sample	11.286	22	ND		
22-13B-3.d	(+)-Catechin	Sample	11.353	25	ND		
22-14B-1.d	(+)-Catechin	Sample	11.202	6	ND		
22-14B-2.d	(+)-Catechin	Sample	11.177	39	ND		
22-14B-3.d	(+)-Catechin	Sample	11.035	5	ND		
22-15B-1.d	(+)-Catechin	Sample	11.186	12	ND		
22-15B-2.d	(+)-Catechin	Sample	11.219	9	ND		
22-15B-3.d	(+)-Catechin	Sample	11.461	24	ND		
22-16B-1.d	(+)-Catechin	Sample	11.353	24	ND		
22-16B-2.d	(+)-Catechin	Sample	11.403	75	ND		
22-16B-3.d	(+)-Catechin	Sample	11.369	47	ND		
22-17B-1.d	(+)-Catechin	Sample	11.244	6	ND		
22-17B-2.d	(+)-Catechin	Sample	11.361	20	ND		
22-17B-3.d	(+)-Catechin	Sample	11.236	12	ND		
22-18B-1.d	(+)-Catechin	Sample	11.361	23	ND		
22-18B-2.d	(+)-Catechin	Sample	11.470	92	ND		
22-18B-3.d	(+)-Catechin	Sample	11.394	52	ND		
22-1C-1.d	(+)-Catechin	Sample	11.445	62	ND		

(+)-Catechin

Data File	Compound	Sample Type	RT	Resp.	Final Conc	Exp. Conc	Accuracy
22-1C-2.d	(+)-Catechin	Sample	11.428	56	ND		
22-1C-3.d	(+)-Catechin	Sample	11.186	12	ND		
22-2C-1.d	(+)-Catechin	Sample	11.378	83	ND		
22-2C-2.d	(+)-Catechin	Sample	11.386	56	ND		
22-2C-3.d	(+)-Catechin	Sample	11.378	34	ND		
22-3C-1.d	(+)-Catechin	Sample	11.261	17	ND		
22-3C-2.d	(+)-Catechin	Sample	11.269	28	ND		
22-3C-3.d	(+)-Catechin	Sample	11.328	43	ND		
22-4C-1.d	(+)-Catechin	Sample	11.344	18	ND		
22-4C-2.d	(+)-Catechin	Sample	11.261	31	ND		
22-4C-3.d	(+)-Catechin	Sample	11.252	9	ND		
22-5C-1.d	(+)-Catechin	Sample	11.378	13	ND		
22-5C-2.d	(+)-Catechin	Sample	11.445	85	ND		
22-5C-3.d	(+)-Catechin	Sample	11.353	25	ND		
22-6C-1.d	(+)-Catechin	Sample	11.261	34	ND		
22-6C-2.d	(+)-Catechin	Sample	11.277	89	ND		
22-6C-3.d	(+)-Catechin	Sample	11.286	77	ND		
22-7C-1.d	(+)-Catechin	Sample	11.261	255	48.5150		
22-7C-2.d	(+)-Catechin	Sample	11.277	264	51.1936		
22-7C-3.d	(+)-Catechin	Sample	11.269	245	45.3252		
22-8C-1.d	(+)-Catechin	Sample	11.277	27	ND		
22-8C-2.d	(+)-Catechin	Sample	11.353	91	ND		
22-8C-3.d	(+)-Catechin	Sample	11.353	12	ND		
22-9C-1.d	(+)-Catechin	Sample	11.269	5170	1564.9422		
22-9C-2.d	(+)-Catechin	Sample	11.277	5080	1537.2410		
22-9C-3.d	(+)-Catechin	Sample	11.277	4879	1475.1147		
22-10C-1.d	(+)-Catechin	Sample	11.261	19	ND		
22-10C-2.d	(+)-Catechin	Sample	11.269	18	ND		
22-10C-3.d	(+)-Catechin	Sample	11.319	33	ND		
22-11C-1.d	(+)-Catechin	Sample	11.261	6	ND		
22-11C-2.d	(+)-Catechin	Sample	11.436	27	ND		
22-11C-3.d	(+)-Catechin	Sample	11.344	66	ND		
22-12C-1.d	(+)-Catechin	Sample	11.361	75	ND		
22-12C-2.d	(+)-Catechin	Sample	11.745	15	ND		
22-12C-3.d	(+)-Catechin	Sample	11.428	63	ND		
22-13C-1.d	(+)-Catechin	Sample	11.453	70	ND		
22-13C-2.d	(+)-Catechin	Sample	11.445	8	ND		
22-13C-3.d	(+)-Catechin	Sample	11.562	41	ND		
22-14C-1.d	(+)-Catechin	Sample	11.353	24	ND		
22-14C-2.d	(+)-Catechin	Sample	11.411	31	ND		

(+)-Catechin

Data File	Compound	Sample Type	RT	Resp.	Final Conc	Exp. Conc	Accuracy
22-14C-3.d	(+)-Catechin	Sample	11.194	8	ND		
22-15C-1.d	(+)-Catechin	Sample	11.386	35	ND		
22-15C-2.d	(+)-Catechin	Sample	11.386	68	ND		
22-15C-3.d	(+)-Catechin	Sample	11.069	11	ND		
22-16C-1.d	(+)-Catechin	Sample	11.436	31	ND		
22-16C-2.d	(+)-Catechin	Sample	11.085	3	ND		
22-16C-3.d	(+)-Catechin	Sample	11.378	18	ND		
22-17C-1.d	(+)-Catechin	Sample	11.227	10	ND		
22-17C-2.d	(+)-Catechin	Sample	11.453	58	ND		
22-17C-3.d	(+)-Catechin	Sample	11.018	25	ND		
22-18C-1.d	(+)-Catechin	Sample	11.286	187	27.3187		
22-18C-2.d	(+)-Catechin	Sample	11.286	193	29.2057		
22-18C-3.d	(+)-Catechin	Sample	11.277	216	36.2682		

2,5-Dihydroxybenzoic acid

Data File	Compound	Sample Type	RT	Resp.	Final Conc	Exp. Conc	Accuracy
blank-061022-4.d	2,5-Dihydroxybenzoic acid	Sample	11.963	0	0.5911		
25ppb1.d	2,5-Dihydroxybenzoic acid	Calibration	11.996	18	3.4659		13.86
25ppb2.d	2,5-Dihydroxybenzoic acid	Calibration	12.138	105	17.1393		68.56
25ppb3.d	2,5-Dihydroxybenzoic acid	Calibration	12.013	127	20.6299		82.52
50ppb1.d	2,5-Dihydroxybenzoic acid	Calibration	11.921	216	34.6840		69.37
50ppb2.d	2,5-Dihydroxybenzoic acid	Calibration	11.937	265	42.4716		84.94
50ppb3.d	2,5-Dihydroxybenzoic acid	Calibration	11.946	217	34.8201		69.64
100ppb1.d	2,5-Dihydroxybenzoic acid	Calibration	11.954	674	107.1222		107.12
100ppb2.d	2,5-Dihydroxybenzoic acid	Calibration	11.971	722	114.6293		114.63
100ppb3.d	2,5-Dihydroxybenzoic acid	Calibration	11.954	684	108.6916		108.69
250ppb1.d	2,5-Dihydroxybenzoic acid	Calibration	11.971	1642	260.0777		104.03
250ppb2.d	2,5-Dihydroxybenzoic acid	Calibration	11.954	1503	238.0886		95.24
250ppb3.d	2,5-Dihydroxybenzoic acid	Calibration	11.971	1572	248.8941		99.56
500ppb1.d	2,5-Dihydroxybenzoic acid	Calibration	11.988	3299	521.8580		104.37
500ppb2.d	2,5-Dihydroxybenzoic acid	Calibration	11.988	3589	567.7118		113.54
500ppb3.d	2,5-Dihydroxybenzoic acid	Calibration	11.988	3731	590.1980		118.04
1000ppb1.d	2,5-Dihydroxybenzoic acid	Calibration	11.996	6657	1052.4536		105.25
1000ppb2.d	2,5-Dihydroxybenzoic acid	Calibration	11.996	6265	990.5379		99.05
1000ppb3.d	2,5-Dihydroxybenzoic acid	Calibration	11.988	6313	998.1250		99.81
22-1A-1.d	2,5-Dihydroxybenzoic acid	Sample	11.996	390	62.1896		
22-1A-2.d	2,5-Dihydroxybenzoic acid	Sample	12.004	348	55.5202		
22-1A-3.d	2,5-Dihydroxybenzoic acid	Sample	11.979	366	58.4145		
22-2A-1.d	2,5-Dihydroxybenzoic acid	Sample	11.988	153	24.6833		
22-2A-2.d	2,5-Dihydroxybenzoic acid	Sample	12.021	174	28.0777		
22-2A-3.d	2,5-Dihydroxybenzoic acid	Sample	11.996	168	27.0635		

2,5-Dihydroxybenzoic acid

Data File	Compound	Sample Type	RT	Resp.	Final Conc	Exp. Conc	Accuracy
22-3A-1.d	2,5-Dihydroxybenzoic acid	Sample	11.996	474	75.3801		
22-3A-2.d	2,5-Dihydroxybenzoic acid	Sample	12.004	515	81.9822		
22-3A-3.d	2,5-Dihydroxybenzoic acid	Sample	11.996	504	80.1387		
22-4A-1.d	2,5-Dihydroxybenzoic acid	Sample	11.996	754	119.6404		
22-4A-2.d	2,5-Dihydroxybenzoic acid	Sample	11.996	706	112.0852		
22-4A-3.d	2,5-Dihydroxybenzoic acid	Sample	11.996	707	112.3099		
22-5A-1.d	2,5-Dihydroxybenzoic acid	Sample	11.988	1003	159.1268		
22-5A-2.d	2,5-Dihydroxybenzoic acid	Sample	11.979	1039	164.7900		
22-5A-3.d	2,5-Dihydroxybenzoic acid	Sample	12.004	936	148.4455		
22-6A-1.d	2,5-Dihydroxybenzoic acid	Sample	11.996	431	68.7122		
22-6A-2.d	2,5-Dihydroxybenzoic acid	Sample	11.988	471	74.9891		
22-6A-3.d	2,5-Dihydroxybenzoic acid	Sample	11.988	480	76.3890		
22-7A-1.d	2,5-Dihydroxybenzoic acid	Sample	11.988	253	40.5038		
22-7A-2.d	2,5-Dihydroxybenzoic acid	Sample	11.988	286	45.8000		
22-7A-3.d	2,5-Dihydroxybenzoic acid	Sample	11.988	237	37.9655		
22-8A-1.d	2,5-Dihydroxybenzoic acid	Sample	11.979	94	15.3960		
22-8A-2.d	2,5-Dihydroxybenzoic acid	Sample	11.979	116	18.8639		
22-8A-3.d	2,5-Dihydroxybenzoic acid	Sample	12.004	88	14.5028		
22-9A-1.d	2,5-Dihydroxybenzoic acid	Sample	11.996	157	25.3978		
22-9A-2.d	2,5-Dihydroxybenzoic acid	Sample	12.029	119	19.3035		
22-9A-3.d	2,5-Dihydroxybenzoic acid	Sample	11.988	168	27.1328		
22-10A-1.d	2,5-Dihydroxybenzoic acid	Sample	11.996	234	37.5180		
22-10A-2.d	2,5-Dihydroxybenzoic acid	Sample	12.004	177	28.5827		
22-10A-3.d	2,5-Dihydroxybenzoic acid	Sample	12.004	195	31.3389		
22-11A-1.d	2,5-Dihydroxybenzoic acid	Sample	11.996	379	60.4294		
22-11A-2.d	2,5-Dihydroxybenzoic acid	Sample	11.988	434	69.1295		
22-11A-3.d	2,5-Dihydroxybenzoic acid	Sample	11.988	412	65.5913		
22-12A-1.d	2,5-Dihydroxybenzoic acid	Sample	11.979	350	55.9233		
22-12A-2.d	2,5-Dihydroxybenzoic acid	Sample	11.988	338	53.9277		
22-12A-3.d	2,5-Dihydroxybenzoic acid	Sample	11.971	332	53.0191		
22-13A-1.d	2,5-Dihydroxybenzoic acid	Sample	11.963	167	26.8939		
22-13A-2.d	2,5-Dihydroxybenzoic acid	Sample	11.979	160	25.8798		
22-13A-3.d	2,5-Dihydroxybenzoic acid	Sample	11.988	167	26.9509		
22-14A-1.d	2,5-Dihydroxybenzoic acid	Sample	11.988	1814	287.1537		
22-14A-2.d	2,5-Dihydroxybenzoic acid	Sample	12.004	1780	281.8506		
22-14A-3.d	2,5-Dihydroxybenzoic acid	Sample	11.988	1631	258.3578		
22-15A-1.d	2,5-Dihydroxybenzoic acid	Sample	12.004	90	14.7663		
22-15A-2.d	2,5-Dihydroxybenzoic acid	Sample	11.971	105	17.0672		
22-15A-3.d	2,5-Dihydroxybenzoic acid	Sample	12.013	112	18.3196		
22-16A-1.d	2,5-Dihydroxybenzoic acid	Sample	11.988	324	51.7097		

2,5-Dihydroxybenzoic acid

Data File	Compound	Sample Type	RT	Resp.	Final Conc	Exp. Conc	Accuracy
22-16A-2.d	2,5-Dihydroxybenzoic acid	Sample	11.979	351	56.0752		
22-16A-3.d	2,5-Dihydroxybenzoic acid	Sample	11.996	348	55.5403		
22-17A-1.d	2,5-Dihydroxybenzoic acid	Sample	11.979	244	39.1069		
22-17A-2.d	2,5-Dihydroxybenzoic acid	Sample	11.979	207	33.2094		
22-17A-3.d	2,5-Dihydroxybenzoic acid	Sample	11.971	238	38.1634		
22-18A-1.d	2,5-Dihydroxybenzoic acid	Sample	11.954	1190	188.6142		
22-18A-2.d	2,5-Dihydroxybenzoic acid	Sample	11.954	1250	198.0793		
22-18A-3.d	2,5-Dihydroxybenzoic acid	Sample	11.963	1184	187.7127		
22-1B-1.d	2,5-Dihydroxybenzoic acid	Sample	11.971	189	30.4711		
22-1B-2.d	2,5-Dihydroxybenzoic acid	Sample	11.963	250	40.0377		
22-1B-3.d	2,5-Dihydroxybenzoic acid	Sample	11.971	237	38.0418		
22-2B-1.d	2,5-Dihydroxybenzoic acid	Sample	11.979	305	48.6755		
22-2B-2.d	2,5-Dihydroxybenzoic acid	Sample	11.963	202	32.4649		
22-2B-3.d	2,5-Dihydroxybenzoic acid	Sample	11.963	232	37.1773		
22-3B-1.d	2,5-Dihydroxybenzoic acid	Sample	11.946	685	108.7833		
22-3B-2.d	2,5-Dihydroxybenzoic acid	Sample	11.954	592	94.1266		
22-3B-3.d	2,5-Dihydroxybenzoic acid	Sample	11.954	603	95.7873		
22-4B-1.d	2,5-Dihydroxybenzoic acid	Sample	11.963	743	117.9251		
22-4B-2.d	2,5-Dihydroxybenzoic acid	Sample	11.954	850	134.8282		
22-4B-3.d	2,5-Dihydroxybenzoic acid	Sample	11.963	641	101.8447		
22-5B-1.d	2,5-Dihydroxybenzoic acid	Sample	11.954	925	146.7264		
22-5B-2.d	2,5-Dihydroxybenzoic acid	Sample	11.954	1027	162.7915		
22-5B-3.d	2,5-Dihydroxybenzoic acid	Sample	11.954	983	155.9214		
22-6B-1.d	2,5-Dihydroxybenzoic acid	Sample	11.963	778	123.5247		
22-6B-2.d	2,5-Dihydroxybenzoic acid	Sample	11.954	949	150.4749		
22-6B-3.d	2,5-Dihydroxybenzoic acid	Sample	11.946	908	144.0183		
22-7B-1.d	2,5-Dihydroxybenzoic acid	Sample	11.946	293	46.8635		
22-7B-2.d	2,5-Dihydroxybenzoic acid	Sample	11.954	326	52.0467		
22-7B-3.d	2,5-Dihydroxybenzoic acid	Sample	11.963	288	46.0817		
22-8B-1.d	2,5-Dihydroxybenzoic acid	Sample	11.979	85	13.9091		
22-8B-2.d	2,5-Dihydroxybenzoic acid	Sample	11.971	96	15.7271		
22-8B-3.d	2,5-Dihydroxybenzoic acid	Sample	11.954	100	16.4072		
22-9B-1.d	2,5-Dihydroxybenzoic acid	Sample	11.971	166	26.7241		
22-9B-2.d	2,5-Dihydroxybenzoic acid	Sample	11.954	117	19.0662		
22-9B-3.d	2,5-Dihydroxybenzoic acid	Sample	11.979	197	31.6715		
22-10B-1.d	2,5-Dihydroxybenzoic acid	Sample	11.971	176	28.3119		
22-10B-2.d	2,5-Dihydroxybenzoic acid	Sample	11.946	183	29.4460		
22-10B-3.d	2,5-Dihydroxybenzoic acid	Sample	11.954	181	29.1902		
22-11B-1.d	2,5-Dihydroxybenzoic acid	Sample	11.954	495	78.7090		
22-11B-2.d	2,5-Dihydroxybenzoic acid	Sample	11.963	477	75.9169		

2,5-Dihydroxybenzoic acid

Data File	Compound	Sample Type	RT	Resp.	Final Conc	Exp. Conc	Accuracy
22-11B-3.d	2,5-Dihydroxybenzoic acid	Sample	11.946	474	75.4741		
22-12B-1.d	2,5-Dihydroxybenzoic acid	Sample	11.929	495	78.7943		
22-12B-2.d	2,5-Dihydroxybenzoic acid	Sample	11.954	462	73.5571		
22-12B-3.d	2,5-Dihydroxybenzoic acid	Sample	11.954	409	65.2047		
22-13B-1.d	2,5-Dihydroxybenzoic acid	Sample	11.963	202	32.5227		
22-13B-2.d	2,5-Dihydroxybenzoic acid	Sample	11.971	279	44.7079		
22-13B-3.d	2,5-Dihydroxybenzoic acid	Sample	11.954	235	37.7120		
22-14B-1.d	2,5-Dihydroxybenzoic acid	Sample	11.954	2059	325.8821		
22-14B-2.d	2,5-Dihydroxybenzoic acid	Sample	11.963	2365	374.3465		
22-14B-3.d	2,5-Dihydroxybenzoic acid	Sample	11.963	2192	347.0154		
22-15B-1.d	2,5-Dihydroxybenzoic acid	Sample	11.963	60	9.9936		
22-15B-2.d	2,5-Dihydroxybenzoic acid	Sample	11.954	58	9.6473		
22-15B-3.d	2,5-Dihydroxybenzoic acid	Sample	11.946	67	11.2150		
22-16B-1.d	2,5-Dihydroxybenzoic acid	Sample	11.963	308	49.2219		
22-16B-2.d	2,5-Dihydroxybenzoic acid	Sample	11.963	261	41.7920		
22-16B-3.d	2,5-Dihydroxybenzoic acid	Sample	11.954	285	45.6481		
22-17B-1.d	2,5-Dihydroxybenzoic acid	Sample	11.954	416	66.3497		
22-17B-2.d	2,5-Dihydroxybenzoic acid	Sample	11.954	364	58.0918		
22-17B-3.d	2,5-Dihydroxybenzoic acid	Sample	11.954	299	47.7837		
22-18B-1.d	2,5-Dihydroxybenzoic acid	Sample	11.946	117	18.9762		
22-18B-2.d	2,5-Dihydroxybenzoic acid	Sample	11.954	137	22.1567		
22-18B-3.d	2,5-Dihydroxybenzoic acid	Sample	11.954	145	23.4372		
22-1C-1.d	2,5-Dihydroxybenzoic acid	Sample	11.946	197	31.7040		
22-1C-2.d	2,5-Dihydroxybenzoic acid	Sample	11.946	237	37.9502		
22-1C-3.d	2,5-Dihydroxybenzoic acid	Sample	11.946	247	39.5114		
22-2C-1.d	2,5-Dihydroxybenzoic acid	Sample	11.954	183	29.4501		
22-2C-2.d	2,5-Dihydroxybenzoic acid	Sample	11.954	168	27.1212		
22-2C-3.d	2,5-Dihydroxybenzoic acid	Sample	11.954	161	26.0361		
22-3C-1.d	2,5-Dihydroxybenzoic acid	Sample	11.946	458	72.9660		
22-3C-2.d	2,5-Dihydroxybenzoic acid	Sample	11.954	457	72.7937		
22-3C-3.d	2,5-Dihydroxybenzoic acid	Sample	11.946	407	64.7969		
22-4C-1.d	2,5-Dihydroxybenzoic acid	Sample	11.946	614	97.5217		
22-4C-2.d	2,5-Dihydroxybenzoic acid	Sample	11.946	737	116.9489		
22-4C-3.d	2,5-Dihydroxybenzoic acid	Sample	11.946	618	98.2049		
22-5C-1.d	2,5-Dihydroxybenzoic acid	Sample	11.954	556	88.4355		
22-5C-2.d	2,5-Dihydroxybenzoic acid	Sample	11.946	650	103.1991		
22-5C-3.d	2,5-Dihydroxybenzoic acid	Sample	11.946	582	92.5869		
22-6C-1.d	2,5-Dihydroxybenzoic acid	Sample	11.946	689	109.4396		
22-6C-2.d	2,5-Dihydroxybenzoic acid	Sample	11.946	651	103.4546		
22-6C-3.d	2,5-Dihydroxybenzoic acid	Sample	11.946	752	119.4595		

2,5-Dihydroxybenzoic acid

Data File	Compound	Sample Type	RT	Resp.	Final Conc	Exp. Conc	Accuracy
22-7C-1.d	2,5-Dihydroxybenzoic acid	Sample	11.963	211	33.9343		
22-7C-2.d	2,5-Dihydroxybenzoic acid	Sample	11.954	194	31.2367		
22-7C-3.d	2,5-Dihydroxybenzoic acid	Sample	11.954	187	30.0735		
22-8C-1.d	2,5-Dihydroxybenzoic acid	Sample	11.979	74	12.2186		
22-8C-2.d	2,5-Dihydroxybenzoic acid	Sample	11.971	69	11.5008		
22-8C-3.d	2,5-Dihydroxybenzoic acid	Sample	11.971	62	10.3599		
22-9C-1.d	2,5-Dihydroxybenzoic acid	Sample	11.963	148	23.8906		
22-9C-2.d	2,5-Dihydroxybenzoic acid	Sample	11.979	186	29.9341		
22-9C-3.d	2,5-Dihydroxybenzoic acid	Sample	11.988	147	23.7205		
22-10C-1.d	2,5-Dihydroxybenzoic acid	Sample	11.954	190	30.5826		
22-10C-2.d	2,5-Dihydroxybenzoic acid	Sample	11.946	155	25.0829		
22-10C-3.d	2,5-Dihydroxybenzoic acid	Sample	11.963	209	33.5156		
22-11C-1.d	2,5-Dihydroxybenzoic acid	Sample	11.971	490	77.9762		
22-11C-2.d	2,5-Dihydroxybenzoic acid	Sample	11.971	456	72.6222		
22-11C-3.d	2,5-Dihydroxybenzoic acid	Sample	11.971	471	74.9148		
22-12C-1.d	2,5-Dihydroxybenzoic acid	Sample	11.963	388	61.8971		
22-12C-2.d	2,5-Dihydroxybenzoic acid	Sample	11.971	379	60.4825		
22-12C-3.d	2,5-Dihydroxybenzoic acid	Sample	11.963	428	68.1766		
22-13C-1.d	2,5-Dihydroxybenzoic acid	Sample	11.963	211	33.9256		
22-13C-2.d	2,5-Dihydroxybenzoic acid	Sample	11.996	255	40.9229		
22-13C-3.d	2,5-Dihydroxybenzoic acid	Sample	11.963	207	33.1972		
22-14C-1.d	2,5-Dihydroxybenzoic acid	Sample	11.979	1594	252.4955		
22-14C-2.d	2,5-Dihydroxybenzoic acid	Sample	11.971	1561	247.2439		
22-14C-3.d	2,5-Dihydroxybenzoic acid	Sample	11.971	1546	244.7873		
22-15C-1.d	2,5-Dihydroxybenzoic acid	Sample	11.979	62	10.3447		
22-15C-2.d	2,5-Dihydroxybenzoic acid	Sample	11.954	57	9.6157		
22-15C-3.d	2,5-Dihydroxybenzoic acid	Sample	11.963	55	9.2542		
22-16C-1.d	2,5-Dihydroxybenzoic acid	Sample	11.988	280	44.7871		
22-16C-2.d	2,5-Dihydroxybenzoic acid	Sample	11.971	351	55.9786		
22-16C-3.d	2,5-Dihydroxybenzoic acid	Sample	11.979	310	49.6144		
22-17C-1.d	2,5-Dihydroxybenzoic acid	Sample	11.988	420	66.9412		
22-17C-2.d	2,5-Dihydroxybenzoic acid	Sample	11.988	383	61.0745		
22-17C-3.d	2,5-Dihydroxybenzoic acid	Sample	11.988	449	71.5111		
22-18C-1.d	2,5-Dihydroxybenzoic acid	Sample	11.979	1885	298.3611		
22-18C-2.d	2,5-Dihydroxybenzoic acid	Sample	11.979	2005	317.4514		
22-18C-3.d	2,5-Dihydroxybenzoic acid	Sample	11.979	1963	310.7508		

Chlorogenic acid

Data File	Compound	Sample Type	RT	Resp.	Final Conc	Exp. Conc	Accuracy
blank-061022-4.d	Chlorogenic acid	Sample	11.785	3	5.9545		
25ppb1.d	Chlorogenic acid	Calibration	11.785	10	6.2443		24.98

Chlorogenic acid

Data File	Compound	Sample Type	RT	Resp.	Final Conc	Exp. Conc	Accuracy
25ppb2.d	Chlorogenic acid	Calibration	11.785	7	6.1469		24.59
25ppb3.d	Chlorogenic acid	Calibration	11.793	14	6.4463		25.79
50ppb1.d	Chlorogenic acid	Calibration	11.735	674	37.4146		74.83
50ppb2.d	Chlorogenic acid	Calibration	11.760	731	40.1286		80.26
50ppb3.d	Chlorogenic acid	Calibration	11.768	945	50.1352		100.27
100ppb1.d	Chlorogenic acid	Calibration	11.768	2439	120.2845		120.28
100ppb2.d	Chlorogenic acid	Calibration	11.777	2273	112.5017		112.50
100ppb3.d	Chlorogenic acid	Calibration	11.777	2379	117.4567		117.46
250ppb1.d	Chlorogenic acid	Calibration	11.785	5367	257.7291		103.09
250ppb2.d	Chlorogenic acid	Calibration	11.777	5356	257.2116		102.88
250ppb3.d	Chlorogenic acid	Calibration	11.785	5296	254.3829		101.75
500ppb1.d	Chlorogenic acid	Calibration	11.793	10372	492.6535		98.53
500ppb2.d	Chlorogenic acid	Calibration	11.793	11244	533.5764		106.72
500ppb3.d	Chlorogenic acid	Calibration	11.793	10663	506.2754		101.26
1000ppb1.d	Chlorogenic acid	Calibration	11.802	21670	1022.9514		102.30
1000ppb2.d	Chlorogenic acid	Calibration	11.793	20594	972.4178		97.24
1000ppb3.d	Chlorogenic acid	Calibration	11.793	20777	981.0432		98.10
22-1A-1.d	Chlorogenic acid	Sample	11.785	2501742	117433.1342		
22-1A-2.d	Chlorogenic acid	Sample	11.793	2563537	120333.6810		
22-1A-3.d	Chlorogenic acid	Sample	11.785	2554753	119921.3436		
22-2A-1.d	Chlorogenic acid	Sample	11.793	1669096	78350.2029		
22-2A-2.d	Chlorogenic acid	Sample	11.793	1685794	79133.9669		
22-2A-3.d	Chlorogenic acid	Sample	11.785	1684116	79055.1830		
22-3A-1.d	Chlorogenic acid	Sample	11.785	2191245	102858.9384		
22-3A-2.d	Chlorogenic acid	Sample	11.793	2212485	103855.9186		
22-3A-3.d	Chlorogenic acid	Sample	11.793	2185579	102592.9979		
22-4A-1.d	Chlorogenic acid	Sample	11.785	1389286	65216.4040		
22-4A-2.d	Chlorogenic acid	Sample	11.793	1373952	64496.6619		
22-4A-3.d	Chlorogenic acid	Sample	11.793	1386038	65063.9365		
22-5A-1.d	Chlorogenic acid	Sample	11.785	278967	13099.9940		
22-5A-2.d	Chlorogenic acid	Sample	11.793	273477	12842.2968		
22-5A-3.d	Chlorogenic acid	Sample	11.785	271727	12760.1650		
22-6A-1.d	Chlorogenic acid	Sample	11.785	520163	24421.3402		
22-6A-2.d	Chlorogenic acid	Sample	11.785	520355	24430.3520		
22-6A-3.d	Chlorogenic acid	Sample	11.785	518644	24350.0174		
22-7A-1.d	Chlorogenic acid	Sample	11.785	2313596	108601.8976		
22-7A-2.d	Chlorogenic acid	Sample	11.785	2332143	109472.4223		
22-7A-3.d	Chlorogenic acid	Sample	11.793	2319254	108867.4402		
22-8A-1.d	Chlorogenic acid	Sample	11.793	2195860	103075.5475		
22-8A-2.d	Chlorogenic acid	Sample	11.777	2104896	98805.8703		

Chlorogenic acid

Data File	Compound	Sample Type	RT	Resp.	Final Conc	Exp. Conc	Accuracy
22-8A-3.d	Chlorogenic acid	Sample	11.785	2205672	103536.1217		
22-9A-1.d	Chlorogenic acid	Sample	11.793	51581	2426.9096		
22-9A-2.d	Chlorogenic acid	Sample	11.793	46338	2180.8284		
22-9A-3.d	Chlorogenic acid	Sample	11.785	44906	2113.5947		
22-10A-1.d	Chlorogenic acid	Sample	11.785	9335	443.9546		
22-10A-2.d	Chlorogenic acid	Sample	11.793	8522	405.7840		
22-10A-3.d	Chlorogenic acid	Sample	11.785	8261	393.5690		
22-11A-1.d	Chlorogenic acid	Sample	11.785	2445	120.5640		
22-11A-2.d	Chlorogenic acid	Sample	11.785	2300	113.7450		
22-11A-3.d	Chlorogenic acid	Sample	11.785	2203	109.1987		
22-12A-1.d	Chlorogenic acid	Sample	11.785	2224	110.2047		
22-12A-2.d	Chlorogenic acid	Sample	11.785	2189	108.5633		
22-12A-3.d	Chlorogenic acid	Sample	11.785	2018	100.5162		
22-13A-1.d	Chlorogenic acid	Sample	11.777	1878	93.9516		
22-13A-2.d	Chlorogenic acid	Sample	11.785	1887	94.3655		
22-13A-3.d	Chlorogenic acid	Sample	11.793	1866	93.3667		
22-14A-1.d	Chlorogenic acid	Sample	11.777	5655	271.2431		
22-14A-2.d	Chlorogenic acid	Sample	11.785	5300	254.5573		
22-14A-3.d	Chlorogenic acid	Sample	11.793	5109	245.6011		
22-15A-1.d	Chlorogenic acid	Sample	11.785	43941	2068.2917		
22-15A-2.d	Chlorogenic acid	Sample	11.785	43651	2054.7021		
22-15A-3.d	Chlorogenic acid	Sample	11.785	43407	2043.2324		
22-16A-1.d	Chlorogenic acid	Sample	11.785	228862	10748.1845		
22-16A-2.d	Chlorogenic acid	Sample	11.785	231983	10894.6414		
22-16A-3.d	Chlorogenic acid	Sample	11.785	230546	10827.2209		
22-17A-1.d	Chlorogenic acid	Sample	11.777	6060	290.2639		
22-17A-2.d	Chlorogenic acid	Sample	11.768	4992	240.0924		
22-17A-3.d	Chlorogenic acid	Sample	11.768	4918	236.6535		
22-18A-1.d	Chlorogenic acid	Sample	11.760	20542	970.0055		
22-18A-2.d	Chlorogenic acid	Sample	11.752	18170	858.6705		
22-18A-3.d	Chlorogenic acid	Sample	11.768	17336	819.5024		
22-1B-1.d	Chlorogenic acid	Sample	11.752	3043269	142851.4467		
22-1B-2.d	Chlorogenic acid	Sample	11.752	3115501	146241.8617		
22-1B-3.d	Chlorogenic acid	Sample	11.752	3072471	144222.1107		
22-2B-1.d	Chlorogenic acid	Sample	11.760	1769667	83070.8119		
22-2B-2.d	Chlorogenic acid	Sample	11.752	1781600	83630.9327		
22-2B-3.d	Chlorogenic acid	Sample	11.752	1781189	83611.6560		
22-3B-1.d	Chlorogenic acid	Sample	11.752	2876585	135027.5867		
22-3B-2.d	Chlorogenic acid	Sample	11.760	2790460	130985.0262		
22-3B-3.d	Chlorogenic acid	Sample	11.752	2913638	136766.7893		

Chlorogenic acid

Data File	Compound	Sample Type	RT	Resp.	Final Conc	Exp. Conc	Accuracy
22-4B-1.d	Chlorogenic acid	Sample	11.760	1637821	76882.1945		
22-4B-2.d	Chlorogenic acid	Sample	11.760	1655213	77698.5454		
22-4B-3.d	Chlorogenic acid	Sample	11.760	1674585	78607.8377		
22-5B-1.d	Chlorogenic acid	Sample	11.760	381989	17935.6578		
22-5B-2.d	Chlorogenic acid	Sample	11.760	399938	18778.1860		
22-5B-3.d	Chlorogenic acid	Sample	11.760	381746	17924.2777		
22-6B-1.d	Chlorogenic acid	Sample	11.760	730323	34285.8584		
22-6B-2.d	Chlorogenic acid	Sample	11.752	757671	35569.5366		
22-6B-3.d	Chlorogenic acid	Sample	11.760	740221	34750.4639		
22-7B-1.d	Chlorogenic acid	Sample	11.752	2629752	123441.6591		
22-7B-2.d	Chlorogenic acid	Sample	11.752	2671419	125397.4682		
22-7B-3.d	Chlorogenic acid	Sample	11.760	2660273	124874.2597		
22-8B-1.d	Chlorogenic acid	Sample	11.760	2646480	124226.8399		
22-8B-2.d	Chlorogenic acid	Sample	11.760	2620266	122996.4027		
22-8B-3.d	Chlorogenic acid	Sample	11.752	2601967	122137.4812		
22-9B-1.d	Chlorogenic acid	Sample	11.760	52683	2478.6241		
22-9B-2.d	Chlorogenic acid	Sample	11.760	46939	2209.0051		
22-9B-3.d	Chlorogenic acid	Sample	11.760	47426	2231.9023		
22-10B-1.d	Chlorogenic acid	Sample	11.760	9719	461.9932		
22-10B-2.d	Chlorogenic acid	Sample	11.760	8939	425.3883		
22-10B-3.d	Chlorogenic acid	Sample	11.760	8201	390.7503		
22-11B-1.d	Chlorogenic acid	Sample	11.752	3548	172.3211		
22-11B-2.d	Chlorogenic acid	Sample	11.768	3460	168.2001		
22-11B-3.d	Chlorogenic acid	Sample	11.760	3440	167.2576		
22-12B-1.d	Chlorogenic acid	Sample	11.760	3161	154.1532		
22-12B-2.d	Chlorogenic acid	Sample	11.752	3553	172.5817		
22-12B-3.d	Chlorogenic acid	Sample	11.760	3563	173.0188		
22-13B-1.d	Chlorogenic acid	Sample	11.752	2753	135.0011		
22-13B-2.d	Chlorogenic acid	Sample	11.760	2529	124.4980		
22-13B-3.d	Chlorogenic acid	Sample	11.760	2511	123.6428		
22-14B-1.d	Chlorogenic acid	Sample	11.760	5893	282.4252		
22-14B-2.d	Chlorogenic acid	Sample	11.760	5540	265.8481		
22-14B-3.d	Chlorogenic acid	Sample	11.760	5712	273.8833		
22-15B-1.d	Chlorogenic acid	Sample	11.760	19375	915.2301		
22-15B-2.d	Chlorogenic acid	Sample	11.752	19721	931.4722		
22-15B-3.d	Chlorogenic acid	Sample	11.752	19492	920.7244		
22-16B-1.d	Chlorogenic acid	Sample	11.760	171667	8063.5480		
22-16B-2.d	Chlorogenic acid	Sample	11.760	171246	8043.7788		
22-16B-3.d	Chlorogenic acid	Sample	11.760	171523	8056.7672		
22-17B-1.d	Chlorogenic acid	Sample	11.752	4639	223.5260		

Chlorogenic acid

Data File	Compound	Sample Type	RT	Resp.	Final Conc	Exp. Conc	Accuracy
22-17B-2.d	Chlorogenic acid	Sample	11.760	4022	194.6032		
22-17B-3.d	Chlorogenic acid	Sample	11.752	3775	182.9717		
22-18B-1.d	Chlorogenic acid	Sample	11.760	9767	464.2626		
22-18B-2.d	Chlorogenic acid	Sample	11.752	10212	485.1462		
22-18B-3.d	Chlorogenic acid	Sample	11.752	10357	491.9538		
22-1C-1.d	Chlorogenic acid	Sample	11.752	2368691	111187.9388		
22-1C-2.d	Chlorogenic acid	Sample	11.752	2508172	117734.9132		
22-1C-3.d	Chlorogenic acid	Sample	11.752	2421614	113672.0550		
22-2C-1.d	Chlorogenic acid	Sample	11.752	1321646	62041.5009		
22-2C-2.d	Chlorogenic acid	Sample	11.752	1320914	62007.1710		
22-2C-3.d	Chlorogenic acid	Sample	11.760	1303430	61186.4839		
22-3C-1.d	Chlorogenic acid	Sample	11.752	1871698	87859.9860		
22-3C-2.d	Chlorogenic acid	Sample	11.752	1840818	86410.5078		
22-3C-3.d	Chlorogenic acid	Sample	11.752	1928334	90518.3500		
22-4C-1.d	Chlorogenic acid	Sample	11.752	1345880	63178.9971		
22-4C-2.d	Chlorogenic acid	Sample	11.744	1465980	68816.2865		
22-4C-3.d	Chlorogenic acid	Sample	11.752	1389334	65218.6536		
22-5C-1.d	Chlorogenic acid	Sample	11.760	178157	8368.1627		
22-5C-2.d	Chlorogenic acid	Sample	11.752	173493	8149.2279		
22-5C-3.d	Chlorogenic acid	Sample	11.752	177348	8330.1948		
22-6C-1.d	Chlorogenic acid	Sample	11.752	550690	25854.1837		
22-6C-2.d	Chlorogenic acid	Sample	11.752	553268	25975.2312		
22-6C-3.d	Chlorogenic acid	Sample	11.752	569894	26755.5836		
22-7C-1.d	Chlorogenic acid	Sample	11.752	2022464	94936.6784		
22-7C-2.d	Chlorogenic acid	Sample	11.760	2048165	96142.9964		
22-7C-3.d	Chlorogenic acid	Sample	11.752	2013300	94506.4985		
22-8C-1.d	Chlorogenic acid	Sample	11.760	1847505	86724.3935		
22-8C-2.d	Chlorogenic acid	Sample	11.760	1882087	88347.5938		
22-8C-3.d	Chlorogenic acid	Sample	11.760	1796894	84348.7941		
22-9C-1.d	Chlorogenic acid	Sample	11.760	46041	2166.9008		
22-9C-2.d	Chlorogenic acid	Sample	11.768	44458	2092.5862		
22-9C-3.d	Chlorogenic acid	Sample	11.768	41078	1933.9135		
22-10C-1.d	Chlorogenic acid	Sample	11.760	9375	445.8309		
22-10C-2.d	Chlorogenic acid	Sample	11.760	8746	416.3010		
22-10C-3.d	Chlorogenic acid	Sample	11.768	8352	397.8075		
22-11C-1.d	Chlorogenic acid	Sample	11.768	2744	134.6154		
22-11C-2.d	Chlorogenic acid	Sample	11.768	2319	114.6429		
22-11C-3.d	Chlorogenic acid	Sample	11.760	2421	119.4165		
22-12C-1.d	Chlorogenic acid	Sample	11.760	1959	97.7652		
22-12C-2.d	Chlorogenic acid	Sample	11.768	1892	94.5798		

Chlorogenic acid

Data File	Compound	Sample Type	RT	Resp.	Final Conc	Exp. Conc	Accuracy
22-12C-3.d	Chlorogenic acid	Sample	11.768	1851	92.6590		
22-13C-1.d	Chlorogenic acid	Sample	11.768	1739	87.4273		
22-13C-2.d	Chlorogenic acid	Sample	11.768	1809	90.6868		
22-13C-3.d	Chlorogenic acid	Sample	11.768	1606	81.1722		
22-14C-1.d	Chlorogenic acid	Sample	11.768	3916	189.5822		
22-14C-2.d	Chlorogenic acid	Sample	11.768	3843	186.1807		
22-14C-3.d	Chlorogenic acid	Sample	11.768	3930	190.2436		
22-15C-1.d	Chlorogenic acid	Sample	11.777	18178	859.0215		
22-15C-2.d	Chlorogenic acid	Sample	11.760	19487	920.4916		
22-15C-3.d	Chlorogenic acid	Sample	11.768	19031	899.0568		
22-16C-1.d	Chlorogenic acid	Sample	11.768	105880	4975.6015		
22-16C-2.d	Chlorogenic acid	Sample	11.777	110099	5173.6618		
22-16C-3.d	Chlorogenic acid	Sample	11.777	111115	5221.3159		
22-17C-1.d	Chlorogenic acid	Sample	11.768	3353	163.1833		
22-17C-2.d	Chlorogenic acid	Sample	11.777	2923	142.9976		
22-17C-3.d	Chlorogenic acid	Sample	11.777	2829	138.5871		
22-18C-1.d	Chlorogenic acid	Sample	11.777	7208	344.1140		
22-18C-2.d	Chlorogenic acid	Sample	11.768	7789	371.3926		
22-18C-3.d	Chlorogenic acid	Sample	11.768	7483	357.0188		

3-Hydroxybenzoic acid

Data File	Compound	Sample Type	RT	Resp.	Final Conc	Exp. Conc	Accuracy
blank-061022-4.d	3-Hydroxybenzoic acid	Sample	12.803	31	20.8467		
25ppb1.d	3-Hydroxybenzoic acid	Calibration	12.803	5	15.9093		63.64
25ppb2.d	3-Hydroxybenzoic acid	Calibration	12.845	7	16.3334		65.33
25ppb3.d	3-Hydroxybenzoic acid	Calibration	12.911	16	18.0074		72.03
50ppb1.d	3-Hydroxybenzoic acid	Calibration	12.794	144	41.9387		83.88
50ppb2.d	3-Hydroxybenzoic acid	Calibration	12.794	198	52.0752		104.15
50ppb3.d	3-Hydroxybenzoic acid	Calibration	12.803	200	52.4551		104.91
100ppb1.d	3-Hydroxybenzoic acid	Calibration	12.828	482	105.1845		105.18
100ppb2.d	3-Hydroxybenzoic acid	Calibration	12.828	430	95.5195		95.52
100ppb3.d	3-Hydroxybenzoic acid	Calibration	12.819	534	114.8329		114.83
250ppb1.d	3-Hydroxybenzoic acid	Calibration	12.836	1239	246.6953		98.68
250ppb2.d	3-Hydroxybenzoic acid	Calibration	12.836	1158	231.6025		92.64
250ppb3.d	3-Hydroxybenzoic acid	Calibration	12.845	1273	253.1454		101.26
500ppb1.d	3-Hydroxybenzoic acid	Calibration	12.845	2642	509.1901		101.84
500ppb2.d	3-Hydroxybenzoic acid	Calibration	12.853	2732	525.9839		105.20
500ppb3.d	3-Hydroxybenzoic acid	Calibration	12.853	2698	519.6662		103.93
1000ppb1.d	3-Hydroxybenzoic acid	Calibration	12.861	5429	1030.3545		103.04
1000ppb2.d	3-Hydroxybenzoic acid	Calibration	12.853	4978	946.1062		94.61
1000ppb3.d	3-Hydroxybenzoic acid	Calibration	12.845	4136	788.6899		78.87

Agilent Technologies

3-Hydroxybenzoic acid

Data File	Compound	Sample Type	RT	Resp.	Final Conc	Exp. Conc	Accuracy
22-1A-1.d	3-Hydroxybenzoic acid	Sample	12.811	18	18.3511		
22-1A-2.d	3-Hydroxybenzoic acid	Sample	12.794	7	16.3791		
22-1A-3.d	3-Hydroxybenzoic acid	Sample	12.828	17	18.2302		
22-2A-1.d	3-Hydroxybenzoic acid	Sample	12.878	11	17.1512		
22-2A-2.d	3-Hydroxybenzoic acid	Sample	12.861	12	17.2935		
22-2A-3.d	3-Hydroxybenzoic acid	Sample	12.861	3	15.6186		
22-3A-1.d	3-Hydroxybenzoic acid	Sample	12.811	10	16.8947		
22-3A-2.d	3-Hydroxybenzoic acid	Sample	12.828	8	16.6351		
22-3A-3.d	3-Hydroxybenzoic acid	Sample	12.803	15	17.8247		
22-4A-1.d	3-Hydroxybenzoic acid	Sample	12.861	40	22.5081		
22-4A-2.d	3-Hydroxybenzoic acid	Sample	12.811	52	24.8173		
22-4A-3.d	3-Hydroxybenzoic acid	Sample	12.819	19	18.5333		
22-5A-1.d	3-Hydroxybenzoic acid	Sample	12.778	78	29.6590		
22-5A-2.d	3-Hydroxybenzoic acid	Sample	12.836	8	16.6241		
22-5A-3.d	3-Hydroxybenzoic acid	Sample	12.870	20	18.7723		
22-6A-1.d	3-Hydroxybenzoic acid	Sample	12.828	45	23.4304		
22-6A-2.d	3-Hydroxybenzoic acid	Sample	12.845	22	19.1089		
22-6A-3.d	3-Hydroxybenzoic acid	Sample	12.861	34	21.4191		
22-7A-1.d	3-Hydroxybenzoic acid	Sample	12.836	20	18.8766		
22-7A-2.d	3-Hydroxybenzoic acid	Sample	12.836	31	20.7974		
22-7A-3.d	3-Hydroxybenzoic acid	Sample	12.811	9	16.7820		
22-8A-1.d	3-Hydroxybenzoic acid	Sample	12.811	3	15.6808		
22-8A-2.d	3-Hydroxybenzoic acid	Sample	12.819	10	16.8329		
22-8A-3.d	3-Hydroxybenzoic acid	Sample	12.794	8	16.4921		
22-9A-1.d	3-Hydroxybenzoic acid	Sample	12.794	4	15.7185		
22-9A-2.d	3-Hydroxybenzoic acid	Sample	12.853	12	17.3700		
22-9A-3.d	3-Hydroxybenzoic acid	Sample	12.786	4	15.7920		
22-10A-1.d	3-Hydroxybenzoic acid	Sample	12.786	8	16.5507		
22-10A-2.d	3-Hydroxybenzoic acid	Sample	12.778	4	15.8126		
22-10A-3.d	3-Hydroxybenzoic acid	Sample	12.794	7	16.4463		
22-11A-1.d	3-Hydroxybenzoic acid	Sample	12.819	8	16.4765		
22-11A-2.d	3-Hydroxybenzoic acid	Sample	12.794	10	16.9222		
22-11A-3.d	3-Hydroxybenzoic acid	Sample	12.819	9	16.6987		
22-12A-1.d	3-Hydroxybenzoic acid	Sample	12.853	15	17.8930		
22-12A-2.d	3-Hydroxybenzoic acid	Sample	12.878	18	18.3616		
22-12A-3.d	3-Hydroxybenzoic acid	Sample	12.836	13	17.5020		
22-13A-1.d	3-Hydroxybenzoic acid	Sample	12.828	23	19.4046		
22-13A-2.d	3-Hydroxybenzoic acid	Sample	12.895	32	21.0543		
22-13A-3.d	3-Hydroxybenzoic acid	Sample	12.861	23	19.3580		
22-14A-1.d	3-Hydroxybenzoic acid	Sample	12.828	22	19.1762		

3-Hydroxybenzoic acid

Data File	Compound	Sample Type	RT	Resp.	Final Conc	Exp. Conc	Accuracy
22-14A-2.d	3-Hydroxybenzoic acid	Sample	12.845	35	21.6049		
22-14A-3.d	3-Hydroxybenzoic acid	Sample	12.828	65	27.2828		
22-15A-1.d	3-Hydroxybenzoic acid	Sample	12.853	32	21.0380		
22-15A-2.d	3-Hydroxybenzoic acid	Sample	12.836	13	17.5453		
22-15A-3.d	3-Hydroxybenzoic acid	Sample	12.811	14	17.6302		
22-16A-1.d	3-Hydroxybenzoic acid	Sample	12.853	28	20.3019		
22-16A-2.d	3-Hydroxybenzoic acid	Sample	12.836	45	23.3912		
22-16A-3.d	3-Hydroxybenzoic acid	Sample	12.861	35	21.6264		
22-17A-1.d	3-Hydroxybenzoic acid	Sample	12.819	44	23.2920		
22-17A-2.d	3-Hydroxybenzoic acid	Sample	12.811	23	19.2719		
22-17A-3.d	3-Hydroxybenzoic acid	Sample	12.828	20	18.7256		
22-18A-1.d	3-Hydroxybenzoic acid	Sample	12.836	85	30.8923		
22-18A-2.d	3-Hydroxybenzoic acid	Sample	12.811	60	26.3185		
22-18A-3.d	3-Hydroxybenzoic acid	Sample	12.819	88	31.5319		
22-1B-1.d	3-Hydroxybenzoic acid	Sample	12.778	19	18.5210		
22-1B-2.d	3-Hydroxybenzoic acid	Sample	12.778	39	22.3326		
22-1B-3.d	3-Hydroxybenzoic acid	Sample	12.971	19	18.5662		
22-2B-1.d	3-Hydroxybenzoic acid	Sample	12.794	27	20.1517		
22-2B-2.d	3-Hydroxybenzoic acid	Sample	12.803	26	19.9260		
22-2B-3.d	3-Hydroxybenzoic acid	Sample	12.778	18	18.4287		
22-3B-1.d	3-Hydroxybenzoic acid	Sample	12.971	11	17.0401		
22-3B-2.d	3-Hydroxybenzoic acid	Sample	12.962	4	15.8500		
22-3B-3.d	3-Hydroxybenzoic acid	Sample	12.794	11	17.0293		
22-4B-1.d	3-Hydroxybenzoic acid	Sample	12.811	13	17.4594		
22-4B-2.d	3-Hydroxybenzoic acid	Sample	12.803	15	17.9119		
22-4B-3.d	3-Hydroxybenzoic acid	Sample	12.819	17	18.2277		
22-5B-1.d	3-Hydroxybenzoic acid	Sample	12.819	28	20.2408		
22-5B-2.d	3-Hydroxybenzoic acid	Sample	12.803	14	17.6630		
22-5B-3.d	3-Hydroxybenzoic acid	Sample	12.778	15	17.8704		
22-6B-1.d	3-Hydroxybenzoic acid	Sample	12.811	21	18.8828		
22-6B-2.d	3-Hydroxybenzoic acid	Sample	12.819	5	15.9278		
22-6B-3.d	3-Hydroxybenzoic acid	Sample	12.811	14	17.7242		
22-7B-1.d	3-Hydroxybenzoic acid	Sample	12.811	25	19.6654		
22-7B-2.d	3-Hydroxybenzoic acid	Sample	12.819	24	19.5156		
22-7B-3.d	3-Hydroxybenzoic acid	Sample	12.845	24	19.4897		
22-8B-1.d	3-Hydroxybenzoic acid	Sample	12.811	11	17.1693		
22-8B-2.d	3-Hydroxybenzoic acid	Sample	12.811	10	16.8859		
22-8B-3.d	3-Hydroxybenzoic acid	Sample	12.811	18	18.4069		
22-9B-1.d	3-Hydroxybenzoic acid	Sample	12.886	7	16.3585		
22-9B-2.d	3-Hydroxybenzoic acid	Sample	12.819	8	16.5939		

3-Hydroxybenzoic acid

Data File	Compound	Sample Type	RT	Resp.	Final Conc	Exp. Conc	Accuracy
22-9B-3.d	3-Hydroxybenzoic acid	Sample	12.803	4	15.7893		
22-10B-1.d	3-Hydroxybenzoic acid	Sample	12.811	6	16.1498		
22-10B-2.d	3-Hydroxybenzoic acid	Sample	12.811	4	15.8336		
22-10B-3.d	3-Hydroxybenzoic acid	Sample	12.895	11	17.1867		
22-11B-1.d	3-Hydroxybenzoic acid	Sample	12.786	12	17.2381		
22-11B-2.d	3-Hydroxybenzoic acid	Sample	12.778	13	17.5118		
22-11B-3.d	3-Hydroxybenzoic acid	Sample	12.794	10	16.9236		
22-12B-1.d	3-Hydroxybenzoic acid	Sample	12.819	30	20.7352		
22-12B-2.d	3-Hydroxybenzoic acid	Sample	12.794	14	17.6053		
22-12B-3.d	3-Hydroxybenzoic acid	Sample	12.819	13	17.4464		
22-13B-1.d	3-Hydroxybenzoic acid	Sample	12.836	16	17.9509		
22-13B-2.d	3-Hydroxybenzoic acid	Sample	12.819	17	18.2734		
22-13B-3.d	3-Hydroxybenzoic acid	Sample	12.861	30	20.6976		
22-14B-1.d	3-Hydroxybenzoic acid	Sample	12.828	46	23.6192		
22-14B-2.d	3-Hydroxybenzoic acid	Sample	12.853	48	24.0224		
22-14B-3.d	3-Hydroxybenzoic acid	Sample	12.811	18	18.4550		
22-15B-1.d	3-Hydroxybenzoic acid	Sample	12.778	9	16.6615		
22-15B-2.d	3-Hydroxybenzoic acid	Sample	12.819	14	17.6661		
22-15B-3.d	3-Hydroxybenzoic acid	Sample	12.836	17	18.2555		
22-16B-1.d	3-Hydroxybenzoic acid	Sample	12.811	30	20.7233		
22-16B-2.d	3-Hydroxybenzoic acid	Sample	12.819	10	16.9924		
22-16B-3.d	3-Hydroxybenzoic acid	Sample	12.794	9	16.7849		
22-17B-1.d	3-Hydroxybenzoic acid	Sample	12.845	3	15.5872		
22-17B-2.d	3-Hydroxybenzoic acid	Sample	12.828	35	21.5628		
22-17B-3.d	3-Hydroxybenzoic acid	Sample	12.803	25	19.7116		
22-18B-1.d	3-Hydroxybenzoic acid	Sample	12.819	19	18.6275		
22-18B-2.d	3-Hydroxybenzoic acid	Sample	12.845	8	16.5479		
22-18B-3.d	3-Hydroxybenzoic acid	Sample	12.803	16	18.0288		
22-1C-1.d	3-Hydroxybenzoic acid	Sample	12.803	5	16.0068		
22-1C-2.d	3-Hydroxybenzoic acid	Sample	12.819	4	15.8005		
22-1C-3.d	3-Hydroxybenzoic acid	Sample	12.811	10	16.9852		
22-2C-1.d	3-Hydroxybenzoic acid	Sample	12.845	8	16.5046		
22-2C-2.d	3-Hydroxybenzoic acid	Sample	12.794	9	16.7449		
22-2C-3.d	3-Hydroxybenzoic acid	Sample	12.811	3	15.6616		
22-3C-1.d	3-Hydroxybenzoic acid	Sample	12.836	8	16.5165		
22-3C-2.d	3-Hydroxybenzoic acid	Sample	12.836	11	17.0564		
22-3C-3.d	3-Hydroxybenzoic acid	Sample	12.803	17	18.2676		
22-4C-1.d	3-Hydroxybenzoic acid	Sample	12.845	8	16.5199		
22-4C-2.d	3-Hydroxybenzoic acid	Sample	12.861	9	16.7989		
22-4C-3.d	3-Hydroxybenzoic acid	Sample	12.845	8	16.6067		

3-Hydroxybenzoic acid

Data File	Compound	Sample Type	RT	Resp.	Final Conc	Exp. Conc	Accuracy
22-5C-1.d	3-Hydroxybenzoic acid	Sample	12.819	23	19.3879		
22-5C-2.d	3-Hydroxybenzoic acid	Sample	12.878	3	15.6763		
22-5C-3.d	3-Hydroxybenzoic acid	Sample	12.853	11	17.1793		
22-6C-1.d	3-Hydroxybenzoic acid	Sample	12.803	3	15.5206		
22-6C-2.d	3-Hydroxybenzoic acid	Sample	12.803	36	21.7693		
22-6C-3.d	3-Hydroxybenzoic acid	Sample	12.828	47	23.7517		
22-7C-1.d	3-Hydroxybenzoic acid	Sample	12.853	8	16.5971		
22-7C-2.d	3-Hydroxybenzoic acid	Sample	12.811	17	18.3021		
22-7C-3.d	3-Hydroxybenzoic acid	Sample	12.811	6	16.0832		
22-8C-1.d	3-Hydroxybenzoic acid	Sample	12.828	12	17.2068		
22-8C-2.d	3-Hydroxybenzoic acid	Sample	12.828	4	15.8163		
22-8C-3.d	3-Hydroxybenzoic acid	Sample	12.803	9	16.7576		
22-9C-1.d	3-Hydroxybenzoic acid	Sample	12.803	4	15.8308		
22-9C-2.d	3-Hydroxybenzoic acid	Sample	12.778	5	16.0521		
22-9C-3.d	3-Hydroxybenzoic acid	Sample	12.778	3	15.6720		
22-10C-1.d	3-Hydroxybenzoic acid	Sample	12.786	6	16.1080		
22-10C-2.d	3-Hydroxybenzoic acid	Sample	12.861	2	15.4674		
22-10C-3.d	3-Hydroxybenzoic acid	Sample	12.778	4	15.8584		
22-11C-1.d	3-Hydroxybenzoic acid	Sample	12.794	8	16.4605		
22-11C-2.d	3-Hydroxybenzoic acid	Sample	12.845	11	17.0987		
22-11C-3.d	3-Hydroxybenzoic acid	Sample	12.870	8	16.5538		
22-12C-1.d	3-Hydroxybenzoic acid	Sample	12.861	17	18.1370		
22-12C-2.d	3-Hydroxybenzoic acid	Sample	12.811	62	26.5499		
22-12C-3.d	3-Hydroxybenzoic acid	Sample	12.853	23	19.4327		
22-13C-1.d	3-Hydroxybenzoic acid	Sample	12.853	71	28.3541		
22-13C-2.d	3-Hydroxybenzoic acid	Sample	12.836	18	18.4075		
22-13C-3.d	3-Hydroxybenzoic acid	Sample	12.828	30	20.7500		
22-14C-1.d	3-Hydroxybenzoic acid	Sample	12.819	44	23.3078		
22-14C-2.d	3-Hydroxybenzoic acid	Sample	12.836	54	25.0986		
22-14C-3.d	3-Hydroxybenzoic acid	Sample	12.819	19	18.6375		
22-15C-1.d	3-Hydroxybenzoic acid	Sample	12.828	13	17.4722		
22-15C-2.d	3-Hydroxybenzoic acid	Sample	12.819	7	16.3213		
22-15C-3.d	3-Hydroxybenzoic acid	Sample	12.836	10	16.8853		
22-16C-1.d	3-Hydroxybenzoic acid	Sample	12.811	15	17.9059		
22-16C-2.d	3-Hydroxybenzoic acid	Sample	12.828	14	17.5804		
22-16C-3.d	3-Hydroxybenzoic acid	Sample	12.828	49	24.2178		
22-17C-1.d	3-Hydroxybenzoic acid	Sample	12.819	18	18.4931		
22-17C-2.d	3-Hydroxybenzoic acid	Sample	12.819	16	18.0650		
22-17C-3.d	3-Hydroxybenzoic acid	Sample	12.836	47	23.8278		
22-18C-1.d	3-Hydroxybenzoic acid	Sample	12.828	23	19.4380		

3-Hydroxybenzoic acid

Data File	Compound	Sample Type	RT	Resp.	Final Conc	Exp. Conc	Accuracy
22-18C-2.d	3-Hydroxybenzoic acid	Sample	12.819	31	20.8762		
22-18C-3.d	3-Hydroxybenzoic acid	Sample	12.811	26	19.9422		

4-Hydroxybenzoic acid

Data File	Compound	Sample Type	RT	Resp.	Final Conc	Exp. Conc	Accuracy
blank-061022-4.d	4-Hydroxybenzoic acid	Sample	12.653	167	ND		
25ppb1.d	4-Hydroxybenzoic acid	Calibration	12.577	391	19.3507		77.40
25ppb2.d	4-Hydroxybenzoic acid	Calibration	12.047	26	ND		0.00
25ppb3.d	4-Hydroxybenzoic acid	Calibration	12.097	8	ND		0.00
50ppb1.d	4-Hydroxybenzoic acid	Calibration	12.039	495	29.0138		58.03
50ppb2.d	4-Hydroxybenzoic acid	Calibration	12.047	576	36.6119		73.22
50ppb3.d	4-Hydroxybenzoic acid	Calibration	12.055	590	37.9583		75.92
100ppb1.d	4-Hydroxybenzoic acid	Calibration	12.064	1269	101.3861		101.39
100ppb2.d	4-Hydroxybenzoic acid	Calibration	12.081	1218	96.6080		96.61
100ppb3.d	4-Hydroxybenzoic acid	Calibration	12.072	1272	101.7121		101.71
250ppb1.d	4-Hydroxybenzoic acid	Calibration	12.081	2764	241.2311		96.49
250ppb2.d	4-Hydroxybenzoic acid	Calibration	12.072	2922	255.9975		102.40
250ppb3.d	4-Hydroxybenzoic acid	Calibration	12.089	2843	248.6277		99.45
500ppb1.d	4-Hydroxybenzoic acid	Calibration	12.089	5913	535.6665		107.13
500ppb2.d	4-Hydroxybenzoic acid	Calibration	12.089	5860	530.6833		106.14
500ppb3.d	4-Hydroxybenzoic acid	Calibration	12.097	5837	528.5849		105.72
1000ppb1.d	4-Hydroxybenzoic acid	Calibration	12.097	11222	1032.0475		103.20
1000ppb2.d	4-Hydroxybenzoic acid	Calibration	12.097	10421	957.1763		95.72
1000ppb3.d	4-Hydroxybenzoic acid	Calibration	12.097	10523	966.6949		96.67
22-1A-1.d	4-Hydroxybenzoic acid	Sample	12.097	2274	195.4307		
22-1A-2.d	4-Hydroxybenzoic acid	Sample	12.097	2386	205.8667		
22-1A-3.d	4-Hydroxybenzoic acid	Sample	12.089	2369	204.2941		
22-2A-1.d	4-Hydroxybenzoic acid	Sample	12.089	1902	160.6000		
22-2A-2.d	4-Hydroxybenzoic acid	Sample	12.089	1880	158.5777		
22-2A-3.d	4-Hydroxybenzoic acid	Sample	12.097	1936	163.7994		
22-3A-1.d	4-Hydroxybenzoic acid	Sample	12.097	3756	333.9991		
22-3A-2.d	4-Hydroxybenzoic acid	Sample	12.106	3878	345.4071		
22-3A-3.d	4-Hydroxybenzoic acid	Sample	12.097	3726	331.1625		
22-4A-1.d	4-Hydroxybenzoic acid	Sample	12.089	5132	462.6166		
22-4A-2.d	4-Hydroxybenzoic acid	Sample	12.097	5197	468.6867		
22-4A-3.d	4-Hydroxybenzoic acid	Sample	12.106	5026	452.7124		
22-5A-1.d	4-Hydroxybenzoic acid	Sample	12.089	5413	488.9173		
22-5A-2.d	4-Hydroxybenzoic acid	Sample	12.097	5511	498.0855		
22-5A-3.d	4-Hydroxybenzoic acid	Sample	12.097	5442	491.5913		
22-6A-1.d	4-Hydroxybenzoic acid	Sample	12.097	24131	2239.2134		
22-6A-2.d	4-Hydroxybenzoic acid	Sample	12.097	24704	2292.7947		

4-Hydroxybenzoic acid

Data File	Compound	Sample Type	RT	Resp.	Final Conc	Exp. Conc	Accuracy
22-6A-3.d	4-Hydroxybenzoic acid	Sample	12.097	24791	2300.8602		
22-7A-1.d	4-Hydroxybenzoic acid	Sample	12.097	3193	281.3543		
22-7A-2.d	4-Hydroxybenzoic acid	Sample	12.089	3510	310.9874		
22-7A-3.d	4-Hydroxybenzoic acid	Sample	12.097	3331	294.2179		
22-8A-1.d	4-Hydroxybenzoic acid	Sample	12.097	1780	149.1877		
22-8A-2.d	4-Hydroxybenzoic acid	Sample	12.089	1776	148.8202		
22-8A-3.d	4-Hydroxybenzoic acid	Sample	12.097	1725	144.0694		
22-9A-1.d	4-Hydroxybenzoic acid	Sample	12.097	1112	86.6956		
22-9A-2.d	4-Hydroxybenzoic acid	Sample	12.106	1028	78.8760		
22-9A-3.d	4-Hydroxybenzoic acid	Sample	12.097	1146	89.9355		
22-10A-1.d	4-Hydroxybenzoic acid	Sample	12.097	4215	376.8804		
22-10A-2.d	4-Hydroxybenzoic acid	Sample	12.106	4253	380.4110		
22-10A-3.d	4-Hydroxybenzoic acid	Sample	12.097	4287	383.6616		
22-11A-1.d	4-Hydroxybenzoic acid	Sample	12.097	990	75.3499		
22-11A-2.d	4-Hydroxybenzoic acid	Sample	12.089	1020	78.1079		
22-11A-3.d	4-Hydroxybenzoic acid	Sample	12.097	999	76.1637		
22-12A-1.d	4-Hydroxybenzoic acid	Sample	12.089	2104	179.5159		
22-12A-2.d	4-Hydroxybenzoic acid	Sample	12.081	2039	173.4387		
22-12A-3.d	4-Hydroxybenzoic acid	Sample	12.089	2059	175.3203		
22-13A-1.d	4-Hydroxybenzoic acid	Sample	12.081	22745	2109.5400		
22-13A-2.d	4-Hydroxybenzoic acid	Sample	12.089	23293	2160.8168		
22-13A-3.d	4-Hydroxybenzoic acid	Sample	12.089	23270	2158.6274		
22-14A-1.d	4-Hydroxybenzoic acid	Sample	12.089	17098	1581.5239		
22-14A-2.d	4-Hydroxybenzoic acid	Sample	12.089	17334	1603.6218		
22-14A-3.d	4-Hydroxybenzoic acid	Sample	12.097	17545	1623.3234		
22-15A-1.d	4-Hydroxybenzoic acid	Sample	12.089	2766	241.3607		
22-15A-2.d	4-Hydroxybenzoic acid	Sample	12.089	2801	244.7058		
22-15A-3.d	4-Hydroxybenzoic acid	Sample	12.097	2798	244.4272		
22-16A-1.d	4-Hydroxybenzoic acid	Sample	12.089	3808	338.8176		
22-16A-2.d	4-Hydroxybenzoic acid	Sample	12.089	3824	340.3304		
22-16A-3.d	4-Hydroxybenzoic acid	Sample	12.097	3818	339.7323		
22-17A-1.d	4-Hydroxybenzoic acid	Sample	12.081	5455	492.8357		
22-17A-2.d	4-Hydroxybenzoic acid	Sample	12.081	5454	492.7091		
22-17A-3.d	4-Hydroxybenzoic acid	Sample	12.081	5386	486.3729		
22-18A-1.d	4-Hydroxybenzoic acid	Sample	12.064	123680	11547.7697		
22-18A-2.d	4-Hydroxybenzoic acid	Sample	12.064	123034	11487.3184		
22-18A-3.d	4-Hydroxybenzoic acid	Sample	12.064	125440	11712.3287		
22-1B-1.d	4-Hydroxybenzoic acid	Sample	12.055	2507	217.1711		
22-1B-2.d	4-Hydroxybenzoic acid	Sample	12.055	2579	223.9451		
22-1B-3.d	4-Hydroxybenzoic acid	Sample	12.064	2524	218.7709		

4-Hydroxybenzoic acid

Data File	Compound	Sample Type	RT	Resp.	Final Conc	Exp. Conc	Accuracy
22-2B-1.d	4-Hydroxybenzoic acid	Sample	12.064	2326	200.2176		
22-2B-2.d	4-Hydroxybenzoic acid	Sample	12.064	2246	192.7371		
22-2B-3.d	4-Hydroxybenzoic acid	Sample	12.064	2384	205.6615		
22-3B-1.d	4-Hydroxybenzoic acid	Sample	12.055	5440	491.4292		
22-3B-2.d	4-Hydroxybenzoic acid	Sample	12.064	5355	483.5259		
22-3B-3.d	4-Hydroxybenzoic acid	Sample	12.055	5523	499.1892		
22-4B-1.d	4-Hydroxybenzoic acid	Sample	12.064	6878	625.8807		
22-4B-2.d	4-Hydroxybenzoic acid	Sample	12.064	6928	630.5333		
22-4B-3.d	4-Hydroxybenzoic acid	Sample	12.064	7219	657.7427		
22-5B-1.d	4-Hydroxybenzoic acid	Sample	12.055	7141	650.5005		
22-5B-2.d	4-Hydroxybenzoic acid	Sample	12.055	7424	676.9809		
22-5B-3.d	4-Hydroxybenzoic acid	Sample	12.064	7474	681.6702		
22-6B-1.d	4-Hydroxybenzoic acid	Sample	12.055	36899	3433.1239		
22-6B-2.d	4-Hydroxybenzoic acid	Sample	12.055	38938	3623.6963		
22-6B-3.d	4-Hydroxybenzoic acid	Sample	12.064	37715	3509.3898		
22-7B-1.d	4-Hydroxybenzoic acid	Sample	12.064	4372	391.6025		
22-7B-2.d	4-Hydroxybenzoic acid	Sample	12.064	4430	396.9974		
22-7B-3.d	4-Hydroxybenzoic acid	Sample	12.064	4467	400.4754		
22-8B-1.d	4-Hydroxybenzoic acid	Sample	12.072	2341	201.6449		
22-8B-2.d	4-Hydroxybenzoic acid	Sample	12.072	2432	210.1616		
22-8B-3.d	4-Hydroxybenzoic acid	Sample	12.064	2334	201.0305		
22-9B-1.d	4-Hydroxybenzoic acid	Sample	12.072	1362	110.0940		
22-9B-2.d	4-Hydroxybenzoic acid	Sample	12.072	1231	97.8169		
22-9B-3.d	4-Hydroxybenzoic acid	Sample	12.055	1348	108.8080		
22-10B-1.d	4-Hydroxybenzoic acid	Sample	12.064	4713	423.4917		
22-10B-2.d	4-Hydroxybenzoic acid	Sample	12.055	4726	424.6370		
22-10B-3.d	4-Hydroxybenzoic acid	Sample	12.064	4721	424.1981		
22-11B-1.d	4-Hydroxybenzoic acid	Sample	12.064	1228	97.6221		
22-11B-2.d	4-Hydroxybenzoic acid	Sample	12.072	1259	100.5022		
22-11B-3.d	4-Hydroxybenzoic acid	Sample	12.064	1298	104.1563		
22-12B-1.d	4-Hydroxybenzoic acid	Sample	12.064	2867	250.8042		
22-12B-2.d	4-Hydroxybenzoic acid	Sample	12.064	2658	231.2752		
22-12B-3.d	4-Hydroxybenzoic acid	Sample	12.064	2849	249.1065		
22-13B-1.d	4-Hydroxybenzoic acid	Sample	12.064	31239	2903.8504		
22-13B-2.d	4-Hydroxybenzoic acid	Sample	12.064	32664	3037.1047		
22-13B-3.d	4-Hydroxybenzoic acid	Sample	12.064	31762	2952.7756		
22-14B-1.d	4-Hydroxybenzoic acid	Sample	12.064	24313	2256.2171		
22-14B-2.d	4-Hydroxybenzoic acid	Sample	12.064	22701	2105.4528		
22-14B-3.d	4-Hydroxybenzoic acid	Sample	12.064	23405	2171.2526		
22-15B-1.d	4-Hydroxybenzoic acid	Sample	12.064	2510	217.4602		

4-Hydroxybenzoic acid

Data File	Compound	Sample Type	RT	Resp.	Final Conc	Exp. Conc	Accuracy
22-15B-2.d	4-Hydroxybenzoic acid	Sample	12.064	2376	204.9290		
22-15B-3.d	4-Hydroxybenzoic acid	Sample	12.064	2424	209.4151		
22-16B-1.d	4-Hydroxybenzoic acid	Sample	12.072	4194	374.9533		
22-16B-2.d	4-Hydroxybenzoic acid	Sample	12.064	4123	368.2482		
22-16B-3.d	4-Hydroxybenzoic acid	Sample	12.064	3900	347.4678		
22-17B-1.d	4-Hydroxybenzoic acid	Sample	12.055	7337	668.7923		
22-17B-2.d	4-Hydroxybenzoic acid	Sample	12.064	7401	674.7567		
22-17B-3.d	4-Hydroxybenzoic acid	Sample	12.055	7404	675.0742		
22-18B-1.d	4-Hydroxybenzoic acid	Sample	12.064	4184	374.0308		
22-18B-2.d	4-Hydroxybenzoic acid	Sample	12.055	4268	381.8327		
22-18B-3.d	4-Hydroxybenzoic acid	Sample	12.055	4240	379.1975		
22-1C-1.d	4-Hydroxybenzoic acid	Sample	12.055	2861	250.2627		
22-1C-2.d	4-Hydroxybenzoic acid	Sample	12.055	2905	254.4067		
22-1C-3.d	4-Hydroxybenzoic acid	Sample	12.055	2919	255.6539		
22-2C-1.d	4-Hydroxybenzoic acid	Sample	12.055	1941	164.2178		
22-2C-2.d	4-Hydroxybenzoic acid	Sample	12.055	2045	173.9602		
22-2C-3.d	4-Hydroxybenzoic acid	Sample	12.064	2006	170.3607		
22-3C-1.d	4-Hydroxybenzoic acid	Sample	12.055	4856	436.8158		
22-3C-2.d	4-Hydroxybenzoic acid	Sample	12.055	4796	431.1847		
22-3C-3.d	4-Hydroxybenzoic acid	Sample	12.055	4860	437.1796		
22-4C-1.d	4-Hydroxybenzoic acid	Sample	12.055	6274	569.4254		
22-4C-2.d	4-Hydroxybenzoic acid	Sample	12.055	6380	579.2889		
22-4C-3.d	4-Hydroxybenzoic acid	Sample	12.055	6359	577.3363		
22-5C-1.d	4-Hydroxybenzoic acid	Sample	12.064	5314	479.6371		
22-5C-2.d	4-Hydroxybenzoic acid	Sample	12.055	5269	475.4429		
22-5C-3.d	4-Hydroxybenzoic acid	Sample	12.064	5319	480.1094		
22-6C-1.d	4-Hydroxybenzoic acid	Sample	12.055	33461	3111.5936		
22-6C-2.d	4-Hydroxybenzoic acid	Sample	12.055	33034	3071.6748		
22-6C-3.d	4-Hydroxybenzoic acid	Sample	12.055	33902	3152.8708		
22-7C-1.d	4-Hydroxybenzoic acid	Sample	12.064	3503	310.3047		
22-7C-2.d	4-Hydroxybenzoic acid	Sample	12.064	3549	314.6254		
22-7C-3.d	4-Hydroxybenzoic acid	Sample	12.055	3661	325.0348		
22-8C-1.d	4-Hydroxybenzoic acid	Sample	12.064	1805	151.5405		
22-8C-2.d	4-Hydroxybenzoic acid	Sample	12.072	1752	146.6103		
22-8C-3.d	4-Hydroxybenzoic acid	Sample	12.072	1707	142.3292		
22-9C-1.d	4-Hydroxybenzoic acid	Sample	12.064	1347	108.7151		
22-9C-2.d	4-Hydroxybenzoic acid	Sample	12.072	1379	111.7404		
22-9C-3.d	4-Hydroxybenzoic acid	Sample	12.072	1312	105.4416		
22-10C-1.d	4-Hydroxybenzoic acid	Sample	12.064	5532	500.0726		
22-10C-2.d	4-Hydroxybenzoic acid	Sample	12.072	5518	498.7376		

4-Hydroxybenzoic acid

Data File	Compound	Sample Type	RT	Resp.	Final Conc	Exp. Conc	Accuracy
22-10C-3.d	4-Hydroxybenzoic acid	Sample	12.072	5529	499.7173		
22-11C-1.d	4-Hydroxybenzoic acid	Sample	12.081	1450	118.3786		
22-11C-2.d	4-Hydroxybenzoic acid	Sample	12.072	1485	121.6398		
22-11C-3.d	4-Hydroxybenzoic acid	Sample	12.072	1517	124.5705		
22-12C-1.d	4-Hydroxybenzoic acid	Sample	12.072	2292	197.0838		
22-12C-2.d	4-Hydroxybenzoic acid	Sample	12.072	2357	203.1004		
22-12C-3.d	4-Hydroxybenzoic acid	Sample	12.081	2286	196.4719		
22-13C-1.d	4-Hydroxybenzoic acid	Sample	12.081	30519	2836.4896		
22-13C-2.d	4-Hydroxybenzoic acid	Sample	12.081	30749	2857.9809		
22-13C-3.d	4-Hydroxybenzoic acid	Sample	12.072	31680	2945.0236		
22-14C-1.d	4-Hydroxybenzoic acid	Sample	12.081	19513	1807.3147		
22-14C-2.d	4-Hydroxybenzoic acid	Sample	12.072	19749	1829.4442		
22-14C-3.d	4-Hydroxybenzoic acid	Sample	12.072	19796	1833.7948		
22-15C-1.d	4-Hydroxybenzoic acid	Sample	12.081	2367	204.0907		
22-15C-2.d	4-Hydroxybenzoic acid	Sample	12.072	2520	218.3544		
22-15C-3.d	4-Hydroxybenzoic acid	Sample	12.072	2332	200.8389		
22-16C-1.d	4-Hydroxybenzoic acid	Sample	12.081	3512	311.1788		
22-16C-2.d	4-Hydroxybenzoic acid	Sample	12.089	3549	314.5760		
22-16C-3.d	4-Hydroxybenzoic acid	Sample	12.089	3511	311.0214		
22-17C-1.d	4-Hydroxybenzoic acid	Sample	12.089	5993	543.1787		
22-17C-2.d	4-Hydroxybenzoic acid	Sample	12.089	6050	548.4410		
22-17C-3.d	4-Hydroxybenzoic acid	Sample	12.097	6190	561.5369		
22-18C-1.d	4-Hydroxybenzoic acid	Sample	12.089	12328	1135.5282		
22-18C-2.d	4-Hydroxybenzoic acid	Sample	12.081	12082	1112.4923		
22-18C-3.d	4-Hydroxybenzoic acid	Sample	12.081	12336	1136.2475		

(-)-Epicatechin

Data File	Compound	Sample Type	RT	Resp.	Final Conc	Exp. Conc	Accuracy
blank-061022-4.d	(-)-Epicatechin	Sample	12.436	3	3.4570		
25ppb1.d	(-)-Epicatechin	Calibration	12.293	59	10.5488		42.20
25ppb2.d	(-)-Epicatechin	Calibration	12.293	19	5.4843		21.94
25ppb3.d	(-)-Epicatechin	Calibration	12.211	133	19.9201		79.68
50ppb1.d	(-)-Epicatechin	Calibration	12.302	225	31.4174		62.83
50ppb2.d	(-)-Epicatechin	Calibration	12.326	308	41.9072		83.81
50ppb3.d	(-)-Epicatechin	Calibration	12.334	359	48.2992		96.60
100ppb1.d	(-)-Epicatechin	Calibration	12.343	743	96.7673		96.77
100ppb2.d	(-)-Epicatechin	Calibration	12.351	794	103.1343		103.13
100ppb3.d	(-)-Epicatechin	Calibration	12.351	816	105.9278		105.93
250ppb1.d	(-)-Epicatechin	Calibration	12.361	1942	247.7801		99.11
250ppb2.d	(-)-Epicatechin	Calibration	12.351	1912	244.0519		97.62
250ppb3.d	(-)-Epicatechin	Calibration	12.361	1940	247.5823		99.03

(-)-Epicatechin

Data File	Compound	Sample Type	RT	Resp.	Final Conc	Exp. Conc	Accuracy
500ppb1.d	(-)-Epicatechin	Calibration	12.369	4072	516.2789		103.26
500ppb2.d	(-)-Epicatechin	Calibration	12.369	4097	519.3311		103.87
500ppb3.d	(-)-Epicatechin	Calibration	12.369	4210	533.5963		106.72
1000ppb1.d	(-)-Epicatechin	Calibration	12.369	8001	1011.3057		101.13
1000ppb2.d	(-)-Epicatechin	Calibration	12.369	7749	979.6259		97.96
1000ppb3.d	(-)-Epicatechin	Calibration	12.361	7737	978.0745		97.81
22-1A-1.d	(-)-Epicatechin	Sample	12.277	3	3.4230		
22-1A-2.d	(-)-Epicatechin	Sample	12.219	2	3.3290		
22-1A-3.d	(-)-Epicatechin	Sample	12.219	4	3.5400		
22-2A-1.d	(-)-Epicatechin	Sample	12.169	2	3.3913		
22-2A-2.d	(-)-Epicatechin	Sample	12.277	2	3.3563		
22-2A-3.d	(-)-Epicatechin	Sample	12.269	4	3.5480		
22-3A-1.d	(-)-Epicatechin	Sample	12.302	4	3.5886		
22-3A-2.d	(-)-Epicatechin	Sample	12.302	3	3.5076		
22-3A-3.d	(-)-Epicatechin	Sample	12.302	5	3.6889		
22-4A-1.d	(-)-Epicatechin	Sample	12.252	3	3.4664		
22-4A-2.d	(-)-Epicatechin	Sample	12.453	3	3.4604		
22-4A-3.d	(-)-Epicatechin	Sample	12.227	3	3.5253		
22-5A-1.d	(-)-Epicatechin	Sample	12.302	5	3.7917		
22-5A-2.d	(-)-Epicatechin	Sample	12.293	4	3.5752		
22-5A-3.d	(-)-Epicatechin	Sample	12.302	4	3.5560		
22-6A-1.d	(-)-Epicatechin	Sample	12.386	4	3.6082		
22-6A-2.d	(-)-Epicatechin	Sample	12.285	10	4.4108		
22-6A-3.d	(-)-Epicatechin	Sample	12.277	3	3.4938		
22-7A-1.d	(-)-Epicatechin	Sample	12.369	4073	516.3611		
22-7A-2.d	(-)-Epicatechin	Sample	12.361	4122	522.5514		
22-7A-3.d	(-)-Epicatechin	Sample	12.369	4094	518.9831		
22-8A-1.d	(-)-Epicatechin	Sample	12.361	773	100.5525		
22-8A-2.d	(-)-Epicatechin	Sample	12.351	833	108.0248		
22-8A-3.d	(-)-Epicatechin	Sample	12.351	791	102.8288		
22-9A-1.d	(-)-Epicatechin	Sample	12.361	45367	5719.9451		
22-9A-2.d	(-)-Epicatechin	Sample	12.369	43517	5486.8067		
22-9A-3.d	(-)-Epicatechin	Sample	12.361	44448	5604.2153		
22-10A-1.d	(-)-Epicatechin	Sample	12.361	4	3.5831		
22-10A-2.d	(-)-Epicatechin	Sample	12.261	6	3.8042		
22-10A-3.d	(-)-Epicatechin	Sample	12.302	3	3.5134		
22-11A-1.d	(-)-Epicatechin	Sample	12.302	8	4.1558		
22-11A-2.d	(-)-Epicatechin	Sample	12.351	12	4.5808		
22-11A-3.d	(-)-Epicatechin	Sample	12.361	5	3.7338		
22-12A-1.d	(-)-Epicatechin	Sample	12.310	4	3.5830		

(-)-Epicatechin

Data File	Compound	Sample Type	RT	Resp.	Final Conc	Exp. Conc	Accuracy
22-12A-2.d	(-)-Epicatechin	Sample	12.302	2	3.3423		
22-12A-3.d	(-)-Epicatechin	Sample	12.302	4	3.5477		
22-13A-1.d	(-)-Epicatechin	Sample	12.277	4	3.5781		
22-13A-2.d	(-)-Epicatechin	Sample	12.277	3	3.4649		
22-13A-3.d	(-)-Epicatechin	Sample	12.269	6	3.8983		
22-14A-1.d	(-)-Epicatechin	Sample	12.302	4	3.6197		
22-14A-2.d	(-)-Epicatechin	Sample	12.293	3	3.4226		
22-14A-3.d	(-)-Epicatechin	Sample	12.302	3	3.5284		
22-15A-1.d	(-)-Epicatechin	Sample	12.293	3	3.4232		
22-15A-2.d	(-)-Epicatechin	Sample	12.285	5	3.7248		
22-15A-3.d	(-)-Epicatechin	Sample	12.302	3	3.4495		
22-16A-1.d	(-)-Epicatechin	Sample	12.293	4	3.5823		
22-16A-2.d	(-)-Epicatechin	Sample	12.302	9	4.2226		
22-16A-3.d	(-)-Epicatechin	Sample	12.378	8	4.0570		
22-17A-1.d	(-)-Epicatechin	Sample	12.302	3	3.4518		
22-17A-2.d	(-)-Epicatechin	Sample	12.334	32	7.0945		
22-17A-3.d	(-)-Epicatechin	Sample	12.302	3	3.4889		
22-18A-1.d	(-)-Epicatechin	Sample	12.277	2	3.3796		
22-18A-2.d	(-)-Epicatechin	Sample	12.302	3	3.4417		
22-18A-3.d	(-)-Epicatechin	Sample	12.302	5	3.7401		
22-1B-1.d	(-)-Epicatechin	Sample	12.277	4	3.5909		
22-1B-2.d	(-)-Epicatechin	Sample	12.236	4	3.6068		
22-1B-3.d	(-)-Epicatechin	Sample	12.293	4	3.6628		
22-2B-1.d	(-)-Epicatechin	Sample	12.302	3	3.5001		
22-2B-2.d	(-)-Epicatechin	Sample	12.302	16	5.0702		
22-2B-3.d	(-)-Epicatechin	Sample	12.302	4	3.6208		
22-3B-1.d	(-)-Epicatechin	Sample	12.310	16	5.1046		
22-3B-2.d	(-)-Epicatechin	Sample	12.318	27	6.5141		
22-3B-3.d	(-)-Epicatechin	Sample	12.285	13	4.6908		
22-4B-1.d	(-)-Epicatechin	Sample	12.302	19	5.5192		
22-4B-2.d	(-)-Epicatechin	Sample	12.302	8	4.1007		
22-4B-3.d	(-)-Epicatechin	Sample	12.302	4	3.6229		
22-5B-1.d	(-)-Epicatechin	Sample	12.277	4	3.6046		
22-5B-2.d	(-)-Epicatechin	Sample	12.302	5	3.7281		
22-5B-3.d	(-)-Epicatechin	Sample	12.302	6	3.9173		
22-6B-1.d	(-)-Epicatechin	Sample	12.310	4	3.6179		
22-6B-2.d	(-)-Epicatechin	Sample	12.285	11	4.5066		
22-6B-3.d	(-)-Epicatechin	Sample	12.302	8	4.1473		
22-7B-1.d	(-)-Epicatechin	Sample	12.326	3141	398.8751		
22-7B-2.d	(-)-Epicatechin	Sample	12.334	3238	411.0897		

(-)-Epicatechin

Data File	Compound	Sample Type	RT	Resp.	Final Conc	Exp. Conc	Accuracy
22-7B-3.d	(-)-Epicatechin	Sample	12.334	3187	404.7624		
22-8B-1.d	(-)-Epicatechin	Sample	12.343	824	106.9539		
22-8B-2.d	(-)-Epicatechin	Sample	12.326	814	105.6404		
22-8B-3.d	(-)-Epicatechin	Sample	12.326	827	107.2760		
22-9B-1.d	(-)-Epicatechin	Sample	12.334	41609	5246.3870		
22-9B-2.d	(-)-Epicatechin	Sample	12.334	42332	5337.4994		
22-9B-3.d	(-)-Epicatechin	Sample	12.334	42290	5332.2710		
22-10B-1.d	(-)-Epicatechin	Sample	12.310	11	4.5019		
22-10B-2.d	(-)-Epicatechin	Sample	12.261	15	4.9393		
22-10B-3.d	(-)-Epicatechin	Sample	12.302	5	3.6943		
22-11B-1.d	(-)-Epicatechin	Sample	12.293	19	5.5363		
22-11B-2.d	(-)-Epicatechin	Sample	12.310	19	5.4970		
22-11B-3.d	(-)-Epicatechin	Sample	12.310	14	4.8450		
22-12B-1.d	(-)-Epicatechin	Sample	12.293	5	3.6706		
22-12B-2.d	(-)-Epicatechin	Sample	12.293	4	3.5861		
22-12B-3.d	(-)-Epicatechin	Sample	12.261	4	3.6330		
22-13B-1.d	(-)-Epicatechin	Sample	12.302	5	3.7668		
22-13B-2.d	(-)-Epicatechin	Sample	12.277	5	3.6777		
22-13B-3.d	(-)-Epicatechin	Sample	12.302	5	3.6792		
22-14B-1.d	(-)-Epicatechin	Sample	12.302	4	3.5599		
22-14B-2.d	(-)-Epicatechin	Sample	12.310	11	4.4741		
22-14B-3.d	(-)-Epicatechin	Sample	12.302	6	3.8508		
22-15B-1.d	(-)-Epicatechin	Sample	12.277	9	4.2483		
22-15B-2.d	(-)-Epicatechin	Sample	12.302	6	3.8968		
22-15B-3.d	(-)-Epicatechin	Sample	12.302	4	3.6080		
22-16B-1.d	(-)-Epicatechin	Sample	12.302	7	3.9643		
22-16B-2.d	(-)-Epicatechin	Sample	12.302	7	4.0130		
22-16B-3.d	(-)-Epicatechin	Sample	12.302	6	3.8922		
22-17B-1.d	(-)-Epicatechin	Sample	12.285	2	3.4042		
22-17B-2.d	(-)-Epicatechin	Sample	12.351	19	5.5365		
22-17B-3.d	(-)-Epicatechin	Sample	12.293	11	4.4904		
22-18B-1.d	(-)-Epicatechin	Sample	12.219	4	3.6281		
22-18B-2.d	(-)-Epicatechin	Sample	12.302	3	3.4191		
22-18B-3.d	(-)-Epicatechin	Sample	12.302	4	3.5670		
22-1C-1.d	(-)-Epicatechin	Sample	12.293	3	3.4811		
22-1C-2.d	(-)-Epicatechin	Sample	12.285	5	3.6822		
22-1C-3.d	(-)-Epicatechin	Sample	12.285	7	4.0297		
22-2C-1.d	(-)-Epicatechin	Sample	12.293	6	3.9102		
22-2C-2.d	(-)-Epicatechin	Sample	12.302	4	3.6327		
22-2C-3.d	(-)-Epicatechin	Sample	12.302	3	3.4999		

(-)-Epicatechin

Data File	Compound	Sample Type	RT	Resp.	Final Conc	Exp. Conc	Accuracy
22-3C-1.d	(-)-Epicatechin	Sample	12.310	21	5.7154		
22-3C-2.d	(-)-Epicatechin	Sample	12.334	33	7.3180		
22-3C-3.d	(-)-Epicatechin	Sample	12.310	22	5.8372		
22-4C-1.d	(-)-Epicatechin	Sample	12.302	6	3.8271		
22-4C-2.d	(-)-Epicatechin	Sample	12.277	9	4.2584		
22-4C-3.d	(-)-Epicatechin	Sample	12.302	12	4.6013		
22-5C-1.d	(-)-Epicatechin	Sample	12.285	9	4.2353		
22-5C-2.d	(-)-Epicatechin	Sample	12.302	5	3.7818		
22-5C-3.d	(-)-Epicatechin	Sample	12.277	16	5.0757		
22-6C-1.d	(-)-Epicatechin	Sample	12.310	17	5.2808		
22-6C-2.d	(-)-Epicatechin	Sample	12.302	14	4.8654		
22-6C-3.d	(-)-Epicatechin	Sample	12.302	29	6.7436		
22-7C-1.d	(-)-Epicatechin	Sample	12.326	3880	492.0732		
22-7C-2.d	(-)-Epicatechin	Sample	12.326	3788	480.4771		
22-7C-3.d	(-)-Epicatechin	Sample	12.326	3758	476.6424		
22-8C-1.d	(-)-Epicatechin	Sample	12.334	825	107.0928		
22-8C-2.d	(-)-Epicatechin	Sample	12.334	823	106.8467		
22-8C-3.d	(-)-Epicatechin	Sample	12.334	857	111.0841		
22-9C-1.d	(-)-Epicatechin	Sample	12.334	50209	6330.2016		
22-9C-2.d	(-)-Epicatechin	Sample	12.334	51017	6431.9782		
22-9C-3.d	(-)-Epicatechin	Sample	12.334	50409	6355.3969		
22-10C-1.d	(-)-Epicatechin	Sample	12.310	15	5.0392		
22-10C-2.d	(-)-Epicatechin	Sample	12.302	7	3.9308		
22-10C-3.d	(-)-Epicatechin	Sample	12.302	5	3.6904		
22-11C-1.d	(-)-Epicatechin	Sample	12.343	18	5.3570		
22-11C-2.d	(-)-Epicatechin	Sample	12.310	21	5.6953		
22-11C-3.d	(-)-Epicatechin	Sample	12.318	19	5.4396		
22-12C-1.d	(-)-Epicatechin	Sample	12.285	4	3.5631		
22-12C-2.d	(-)-Epicatechin	Sample	12.302	5	3.7651		
22-12C-3.d	(-)-Epicatechin	Sample	12.261	10	4.3634		
22-13C-1.d	(-)-Epicatechin	Sample	12.302	5	3.7806		
22-13C-2.d	(-)-Epicatechin	Sample	12.302	2	3.3111		
22-13C-3.d	(-)-Epicatechin	Sample	12.310	4	3.6139		
22-14C-1.d	(-)-Epicatechin	Sample	12.285	4	3.5485		
22-14C-2.d	(-)-Epicatechin	Sample	12.310	3	3.4868		
22-14C-3.d	(-)-Epicatechin	Sample	12.293	4	3.5481		
22-15C-1.d	(-)-Epicatechin	Sample	12.293	4	3.6051		
22-15C-2.d	(-)-Epicatechin	Sample	12.293	4	3.6638		
22-15C-3.d	(-)-Epicatechin	Sample	12.302	7	3.9700		
22-16C-1.d	(-)-Epicatechin	Sample	12.302	2	3.3564		

(-)-Epicatechin

Data File	Compound	Sample Type	RT	Resp.	Final Conc	Exp. Conc	Accuracy
22-16C-2.d	(-)-Epicatechin	Sample	12.293	3	3.5094		
22-16C-3.d	(-)-Epicatechin	Sample	12.302	3	3.5188		
22-17C-1.d	(-)-Epicatechin	Sample	12.302	3	3.5130		
22-17C-2.d	(-)-Epicatechin	Sample	12.310	5	3.7749		
22-17C-3.d	(-)-Epicatechin	Sample	12.302	5	3.7090		
22-18C-1.d	(-)-Epicatechin	Sample	12.310	11	4.4285		
22-18C-2.d	(-)-Epicatechin	Sample	12.310	8	4.0459		
22-18C-3.d	(-)-Epicatechin	Sample	12.310	16	5.1168		

Caffeic acid

Data File	Compound	Sample Type	RT	Resp.	Final Conc	Exp. Conc	Accuracy
blank-061022-4.d	Caffeic acid	Sample	12.725	9	ND		
25ppb1.d	Caffeic acid	Calibration	12.691	30	ND		0.00
25ppb2.d	Caffeic acid	Calibration	12.633	44	ND		0.00
25ppb3.d	Caffeic acid	Calibration	12.474	371	ND		0.00
50ppb1.d	Caffeic acid	Calibration	12.574	1120	19.3875		38.78
50ppb2.d	Caffeic acid	Calibration	12.591	1226	23.9716		47.94
50ppb3.d	Caffeic acid	Calibration	12.599	1223	23.8337		47.67
100ppb1.d	Caffeic acid	Calibration	12.616	2941	97.9899		97.99
100ppb2.d	Caffeic acid	Calibration	12.616	3185	108.5444		108.54
100ppb3.d	Caffeic acid	Calibration	12.616	3025	101.6371		101.64
250ppb1.d	Caffeic acid	Calibration	12.633	6437	248.8870		99.55
250ppb2.d	Caffeic acid	Calibration	12.624	6580	255.0503		102.02
250ppb3.d	Caffeic acid	Calibration	12.633	6725	261.3145		104.53
500ppb1.d	Caffeic acid	Calibration	12.641	13194	540.5373		108.11
500ppb2.d	Caffeic acid	Calibration	12.641	13046	534.1772		106.84
500ppb3.d	Caffeic acid	Calibration	12.641	13298	545.0329		109.01
1000ppb1.d	Caffeic acid	Calibration	12.641	24200	1015.6110		101.56
1000ppb2.d	Caffeic acid	Calibration	12.641	23033	965.2409		96.52
1000ppb3.d	Caffeic acid	Calibration	12.641	22884	958.7847		95.88
22-1A-1.d	Caffeic acid	Sample	12.641	32023	1353.2347		
22-1A-2.d	Caffeic acid	Sample	12.649	33448	1414.7815		
22-1A-3.d	Caffeic acid	Sample	12.641	33571	1420.0547		
22-2A-1.d	Caffeic acid	Sample	12.641	31138	1315.0636		
22-2A-2.d	Caffeic acid	Sample	12.641	31428	1327.5757		
22-2A-3.d	Caffeic acid	Sample	12.641	31051	1311.3226		
22-3A-1.d	Caffeic acid	Sample	12.633	26753	1125.7988		
22-3A-2.d	Caffeic acid	Sample	12.641	27032	1137.8526		
22-3A-3.d	Caffeic acid	Sample	12.641	26850	1129.9910		
22-4A-1.d	Caffeic acid	Sample	12.641	10743	434.7464		
22-4A-2.d	Caffeic acid	Sample	12.641	10666	431.4307		

Caffeic acid

Data File	Compound	Sample Type	RT	Resp.	Final Conc	Exp. Conc	Accuracy
22-4A-3.d	Caffeic acid	Sample	12.641	10648	430.6487		
22-5A-1.d	Caffeic acid	Sample	12.633	11228	455.6973		
22-5A-2.d	Caffeic acid	Sample	12.641	11436	464.6832		
22-5A-3.d	Caffeic acid	Sample	12.641	11147	452.1899		
22-6A-1.d	Caffeic acid	Sample	12.641	6060	232.6399		
22-6A-2.d	Caffeic acid	Sample	12.641	5855	223.7757		
22-6A-3.d	Caffeic acid	Sample	12.641	6045	231.9572		
22-7A-1.d	Caffeic acid	Sample	12.641	7006	273.4770		
22-7A-2.d	Caffeic acid	Sample	12.641	6947	270.8885		
22-7A-3.d	Caffeic acid	Sample	12.641	6823	265.5755		
22-8A-1.d	Caffeic acid	Sample	12.641	56058	2390.6871		
22-8A-2.d	Caffeic acid	Sample	12.633	57967	2473.0832		
22-8A-3.d	Caffeic acid	Sample	12.633	56283	2400.3927		
22-9A-1.d	Caffeic acid	Sample	12.649	624	ND		
22-9A-2.d	Caffeic acid	Sample	12.649	644	ND		
22-9A-3.d	Caffeic acid	Sample	12.633	617	ND		
22-10A-1.d	Caffeic acid	Sample	12.633	1082	17.7543		
22-10A-2.d	Caffeic acid	Sample	12.641	1179	21.9685		
22-10A-3.d	Caffeic acid	Sample	12.633	1279	26.2734		
22-11A-1.d	Caffeic acid	Sample	12.641	1053	16.5216		
22-11A-2.d	Caffeic acid	Sample	12.633	1071	17.3040		
22-11A-3.d	Caffeic acid	Sample	12.633	1026	15.3376		
22-12A-1.d	Caffeic acid	Sample	12.633	6637	257.5118		
22-12A-2.d	Caffeic acid	Sample	12.633	6856	266.9685		
22-12A-3.d	Caffeic acid	Sample	12.641	6662	258.5920		
22-13A-1.d	Caffeic acid	Sample	12.624	6923	269.8908		
22-13A-2.d	Caffeic acid	Sample	12.624	7024	274.2231		
22-13A-3.d	Caffeic acid	Sample	12.633	7132	278.8927		
22-14A-1.d	Caffeic acid	Sample	12.633	36489	1546.0178		
22-14A-2.d	Caffeic acid	Sample	12.633	35783	1515.5542		
22-14A-3.d	Caffeic acid	Sample	12.641	36150	1531.3974		
22-15A-1.d	Caffeic acid	Sample	12.633	3333	114.9407		
22-15A-2.d	Caffeic acid	Sample	12.633	3068	103.4612		
22-15A-3.d	Caffeic acid	Sample	12.633	3246	111.1733		
22-16A-1.d	Caffeic acid	Sample	12.633	13735	563.8948		
22-16A-2.d	Caffeic acid	Sample	12.641	14177	582.9586		
22-16A-3.d	Caffeic acid	Sample	12.641	13931	572.3460		
22-17A-1.d	Caffeic acid	Sample	12.633	5923	226.7181		
22-17A-2.d	Caffeic acid	Sample	12.616	5947	227.7462		
22-17A-3.d	Caffeic acid	Sample	12.624	6008	230.3957		

Caffeic acid

Data File	Compound	Sample Type	RT	Resp.	Final Conc	Exp. Conc	Accuracy
22-18A-1.d	Caffeic acid	Sample	12.608	20471	854.6545		
22-18A-2.d	Caffeic acid	Sample	12.608	19199	799.7359		
22-18A-3.d	Caffeic acid	Sample	12.608	19286	803.4863		
22-1B-1.d	Caffeic acid	Sample	12.608	35500	1503.3122		
22-1B-2.d	Caffeic acid	Sample	12.608	36592	1550.4603		
22-1B-3.d	Caffeic acid	Sample	12.608	36129	1530.4708		
22-2B-1.d	Caffeic acid	Sample	12.608	37878	1605.9830		
22-2B-2.d	Caffeic acid	Sample	12.599	36724	1556.1621		
22-2B-3.d	Caffeic acid	Sample	12.608	37916	1607.5985		
22-3B-1.d	Caffeic acid	Sample	12.608	34025	1439.6549		
22-3B-2.d	Caffeic acid	Sample	12.608	34794	1472.8387		
22-3B-3.d	Caffeic acid	Sample	12.599	34714	1469.4140		
22-4B-1.d	Caffeic acid	Sample	12.608	12899	527.8066		
22-4B-2.d	Caffeic acid	Sample	12.608	12846	525.5163		
22-4B-3.d	Caffeic acid	Sample	12.608	12525	511.6542		
22-5B-1.d	Caffeic acid	Sample	12.608	13412	549.9769		
22-5B-2.d	Caffeic acid	Sample	12.608	13559	556.3041		
22-5B-3.d	Caffeic acid	Sample	12.608	13847	568.7407		
22-6B-1.d	Caffeic acid	Sample	12.608	7722	304.3490		
22-6B-2.d	Caffeic acid	Sample	12.608	7965	314.8676		
22-6B-3.d	Caffeic acid	Sample	12.608	7872	310.8539		
22-7B-1.d	Caffeic acid	Sample	12.608	7468	293.3971		
22-7B-2.d	Caffeic acid	Sample	12.608	7755	305.7685		
22-7B-3.d	Caffeic acid	Sample	12.608	7711	303.8853		
22-8B-1.d	Caffeic acid	Sample	12.616	66187	2827.8781		
22-8B-2.d	Caffeic acid	Sample	12.616	67309	2876.2951		
22-8B-3.d	Caffeic acid	Sample	12.608	66993	2862.6518		
22-9B-1.d	Caffeic acid	Sample	12.616	1063	16.9227		
22-9B-2.d	Caffeic acid	Sample	12.608	1055	16.5776		
22-9B-3.d	Caffeic acid	Sample	12.608	1134	20.0139		
22-10B-1.d	Caffeic acid	Sample	12.608	1346	29.1707		
22-10B-2.d	Caffeic acid	Sample	12.599	1273	26.0218		
22-10B-3.d	Caffeic acid	Sample	12.608	1338	28.7964		
22-11B-1.d	Caffeic acid	Sample	12.599	2458	77.1328		
22-11B-2.d	Caffeic acid	Sample	12.599	2468	77.5772		
22-11B-3.d	Caffeic acid	Sample	12.608	2344	72.2518		
22-12B-1.d	Caffeic acid	Sample	12.608	7727	304.5927		
22-12B-2.d	Caffeic acid	Sample	12.608	7970	315.0784		
22-12B-3.d	Caffeic acid	Sample	12.608	7934	313.5303		
22-13B-1.d	Caffeic acid	Sample	12.599	7827	308.8959		

Caffeic acid

Data File	Compound	Sample Type	RT	Resp.	Final Conc	Exp. Conc	Accuracy
22-13B-2.d	Caffeic acid	Sample	12.599	8089	320.2222		
22-13B-3.d	Caffeic acid	Sample	12.608	7940	313.7842		
22-14B-1.d	Caffeic acid	Sample	12.608	45774	1946.7823		
22-14B-2.d	Caffeic acid	Sample	12.608	46157	1963.3242		
22-14B-3.d	Caffeic acid	Sample	12.616	44908	1909.4103		
22-15B-1.d	Caffeic acid	Sample	12.608	2148	63.7779		
22-15B-2.d	Caffeic acid	Sample	12.608	2192	65.6664		
22-15B-3.d	Caffeic acid	Sample	12.599	2242	67.8166		
22-16B-1.d	Caffeic acid	Sample	12.608	12327	503.1295		
22-16B-2.d	Caffeic acid	Sample	12.608	12452	508.5229		
22-16B-3.d	Caffeic acid	Sample	12.608	12357	504.4073		
22-17B-1.d	Caffeic acid	Sample	12.608	6673	259.0783		
22-17B-2.d	Caffeic acid	Sample	12.608	6520	252.4623		
22-17B-3.d	Caffeic acid	Sample	12.599	6550	253.7807		
22-18B-1.d	Caffeic acid	Sample	12.608	5269	198.4691		
22-18B-2.d	Caffeic acid	Sample	12.599	5219	196.3204		
22-18B-3.d	Caffeic acid	Sample	12.599	5254	197.8164		
22-1C-1.d	Caffeic acid	Sample	12.599	35392	1498.6872		
22-1C-2.d	Caffeic acid	Sample	12.608	36584	1550.1026		
22-1C-3.d	Caffeic acid	Sample	12.599	37605	1594.1847		
22-2C-1.d	Caffeic acid	Sample	12.599	29195	1231.1984		
22-2C-2.d	Caffeic acid	Sample	12.599	29642	1250.4847		
22-2C-3.d	Caffeic acid	Sample	12.608	29521	1245.2581		
22-3C-1.d	Caffeic acid	Sample	12.599	25332	1064.4441		
22-3C-2.d	Caffeic acid	Sample	12.599	26592	1118.8422		
22-3C-3.d	Caffeic acid	Sample	12.599	25984	1092.6159		
22-4C-1.d	Caffeic acid	Sample	12.599	10912	442.0612		
22-4C-2.d	Caffeic acid	Sample	12.599	11278	457.8387		
22-4C-3.d	Caffeic acid	Sample	12.599	11272	457.6022		
22-5C-1.d	Caffeic acid	Sample	12.608	8699	346.5158		
22-5C-2.d	Caffeic acid	Sample	12.599	8941	356.9686		
22-5C-3.d	Caffeic acid	Sample	12.599	9208	368.5090		
22-6C-1.d	Caffeic acid	Sample	12.599	6437	248.8998		
22-6C-2.d	Caffeic acid	Sample	12.599	6538	253.2413		
22-6C-3.d	Caffeic acid	Sample	12.599	6441	249.0508		
22-7C-1.d	Caffeic acid	Sample	12.608	6496	251.4500		
22-7C-2.d	Caffeic acid	Sample	12.608	6313	243.5638		
22-7C-3.d	Caffeic acid	Sample	12.608	6573	254.7778		
22-8C-1.d	Caffeic acid	Sample	12.608	53067	2261.5844		
22-8C-2.d	Caffeic acid	Sample	12.608	53099	2262.9418		

Caffeic acid

Data File	Compound	Sample Type	RT	Resp.	Final Conc	Exp. Conc	Accuracy
22-8C-3.d	Caffeic acid	Sample	12.608	54832	2337.7739		
22-9C-1.d	Caffeic acid	Sample	12.616	653	ND		
22-9C-2.d	Caffeic acid	Sample	12.616	613	ND		
22-9C-3.d	Caffeic acid	Sample	12.608	599	ND		
22-10C-1.d	Caffeic acid	Sample	12.599	1364	29.9135		
22-10C-2.d	Caffeic acid	Sample	12.608	1348	29.2393		
22-10C-3.d	Caffeic acid	Sample	12.608	1390	31.0516		
22-11C-1.d	Caffeic acid	Sample	12.608	1381	30.6473		
22-11C-2.d	Caffeic acid	Sample	12.608	1348	29.2283		
22-11C-3.d	Caffeic acid	Sample	12.608	1293	26.8705		
22-12C-1.d	Caffeic acid	Sample	12.624	5887	225.1504		
22-12C-2.d	Caffeic acid	Sample	12.608	6298	242.8835		
22-12C-3.d	Caffeic acid	Sample	12.616	5975	228.9760		
22-13C-1.d	Caffeic acid	Sample	12.608	7547	296.7974		
22-13C-2.d	Caffeic acid	Sample	12.616	7712	303.9185		
22-13C-3.d	Caffeic acid	Sample	12.608	7647	301.1366		
22-14C-1.d	Caffeic acid	Sample	12.616	33573	1420.1529		
22-14C-2.d	Caffeic acid	Sample	12.616	35464	1501.8001		
22-14C-3.d	Caffeic acid	Sample	12.616	34305	1451.7698		
22-15C-1.d	Caffeic acid	Sample	12.624	2434	76.1257		
22-15C-2.d	Caffeic acid	Sample	12.616	2331	71.6853		
22-15C-3.d	Caffeic acid	Sample	12.616	2291	69.9383		
22-16C-1.d	Caffeic acid	Sample	12.616	10529	425.5292		
22-16C-2.d	Caffeic acid	Sample	12.624	10282	414.8452		
22-16C-3.d	Caffeic acid	Sample	12.624	10117	407.7505		
22-17C-1.d	Caffeic acid	Sample	12.624	5609	213.1782		
22-17C-2.d	Caffeic acid	Sample	12.624	5602	212.8647		
22-17C-3.d	Caffeic acid	Sample	12.624	5776	220.3792		
22-18C-1.d	Caffeic acid	Sample	12.624	129618	5565.7068		
22-18C-2.d	Caffeic acid	Sample	12.616	130474	5602.6801		
22-18C-3.d	Caffeic acid	Sample	12.616	129970	5580.9229		

Syringic acid

Data File	Compound	Sample Type	RT	Resp.	Final Conc	Exp. Conc	Accuracy
blank-061022-4.d	Syringic acid	Sample	12.773	0	3.5315		
25ppb1.d	Syringic acid	Calibration	12.824	86	122.7660		491.06
25ppb2.d	Syringic acid	Calibration	12.681	50	73.4044		293.62
25ppb3.d	Syringic acid	Calibration	12.556	14	22.0241		88.10
50ppb1.d	Syringic acid	Calibration	12.715	32	47.7785		95.56
50ppb2.d	Syringic acid	Calibration	12.731	39	56.7659		113.53
50ppb3.d	Syringic acid	Calibration	12.748	34	50.3990		100.80

Syringic acid

Data File	Compound	Sample Type	RT	Resp.	Final Conc	Exp. Conc	Accuracy
100ppb1.d	Syringic acid	Calibration	12.757	70	100.8741		100.87
100ppb2.d	Syringic acid	Calibration	12.740	86	122.5376		122.54
100ppb3.d	Syringic acid	Calibration	12.765	73	104.2785		104.28
250ppb1.d	Syringic acid	Calibration	12.757	190	267.7617		107.10
250ppb2.d	Syringic acid	Calibration	12.748	167	235.9900		94.40
250ppb3.d	Syringic acid	Calibration	12.765	181	255.5301		102.21
500ppb1.d	Syringic acid	Calibration	12.748	353	494.8108		98.96
500ppb2.d	Syringic acid	Calibration	12.765	418	586.0106		117.20
500ppb3.d	Syringic acid	Calibration	12.765	387	543.2235		108.64
1000ppb1.d	Syringic acid	Calibration	12.773	734	1026.8009		102.68
1000ppb2.d	Syringic acid	Calibration	12.757	708	990.2416		99.02
1000ppb3.d	Syringic acid	Calibration	12.757	705	986.9296		98.69
22-1A-1.d	Syringic acid	Sample	12.765	59	85.2610		
22-1A-2.d	Syringic acid	Sample	12.757	48	69.4675		
22-1A-3.d	Syringic acid	Sample	12.757	47	68.9183		
22-2A-1.d	Syringic acid	Sample	12.748	23	35.5285		
22-2A-2.d	Syringic acid	Sample	12.790	14	22.9997		
22-2A-3.d	Syringic acid	Sample	12.807	22	33.3569		
22-3A-1.d	Syringic acid	Sample	12.757	42	61.9106		
22-3A-2.d	Syringic acid	Sample	12.773	43	62.9684		
22-3A-3.d	Syringic acid	Sample	12.748	40	58.2615		
22-4A-1.d	Syringic acid	Sample	12.757	136	192.6977		
22-4A-2.d	Syringic acid	Sample	12.757	154	217.5243		
22-4A-3.d	Syringic acid	Sample	12.773	144	203.1514		
22-5A-1.d	Syringic acid	Sample	12.765	112	159.6340		
22-5A-2.d	Syringic acid	Sample	12.757	103	147.3319		
22-5A-3.d	Syringic acid	Sample	12.765	109	154.8238		
22-6A-1.d	Syringic acid	Sample	12.757	69	99.6474		
22-6A-2.d	Syringic acid	Sample	12.765	88	126.0077		
22-6A-3.d	Syringic acid	Sample	12.790	76	108.3235		
22-7A-1.d	Syringic acid	Sample	12.815	52	75.0292		
22-7A-2.d	Syringic acid	Sample	12.757	43	63.3202		
22-7A-3.d	Syringic acid	Sample	12.765	41	60.4796		
22-8A-1.d	Syringic acid	Sample	12.765	39	57.8248		
22-8A-2.d	Syringic acid	Sample	12.740	42	61.8804		
22-8A-3.d	Syringic acid	Sample	12.765	36	52.5419		
22-9A-1.d	Syringic acid	Sample	12.740	41	60.4814		
22-9A-2.d	Syringic acid	Sample	12.773	38	55.6975		
22-9A-3.d	Syringic acid	Sample	12.748	36	53.6695		
22-10A-1.d	Syringic acid	Sample	12.748	46	67.0598		

Syringic acid

Data File	Compound	Sample Type	RT	Resp.	Final Conc	Exp. Conc	Accuracy
22-10A-2.d	Syringic acid	Sample	12.765	45	66.0071		
22-10A-3.d	Syringic acid	Sample	12.765	45	65.0739		
22-11A-1.d	Syringic acid	Sample	12.748	9	15.4137		
22-11A-2.d	Syringic acid	Sample	12.790	12	20.4132		
22-11A-3.d	Syringic acid	Sample	12.773	13	21.2990		
22-12A-1.d	Syringic acid	Sample	12.757	126	179.1152		
22-12A-2.d	Syringic acid	Sample	12.765	113	160.6869		
22-12A-3.d	Syringic acid	Sample	12.782	156	220.0693		
22-13A-1.d	Syringic acid	Sample	12.765	38	56.2308		
22-13A-2.d	Syringic acid	Sample	12.765	32	47.0367		
22-13A-3.d	Syringic acid	Sample	12.782	35	51.4589		
22-14A-1.d	Syringic acid	Sample	12.748	97	137.7120		
22-14A-2.d	Syringic acid	Sample	12.773	97	138.8652		
22-14A-3.d	Syringic acid	Sample	12.748	101	144.4200		
22-15A-1.d	Syringic acid	Sample	12.757	146	206.7221		
22-15A-2.d	Syringic acid	Sample	12.782	138	194.9232		
22-15A-3.d	Syringic acid	Sample	12.782	150	212.5223		
22-16A-1.d	Syringic acid	Sample	12.765	162	229.2189		
22-16A-2.d	Syringic acid	Sample	12.765	136	193.2969		
22-16A-3.d	Syringic acid	Sample	12.757	163	230.3181		
22-17A-1.d	Syringic acid	Sample	12.740	84	119.7554		
22-17A-2.d	Syringic acid	Sample	12.748	86	123.3339		
22-17A-3.d	Syringic acid	Sample	12.740	85	121.4876		
22-18A-1.d	Syringic acid	Sample	12.706	32	47.9702		
22-18A-2.d	Syringic acid	Sample	12.723	35	51.6835		
22-18A-3.d	Syringic acid	Sample	12.740	29	43.3740		
22-1B-1.d	Syringic acid	Sample	12.731	64	92.8676		
22-1B-2.d	Syringic acid	Sample	12.723	70	100.8704		
22-1B-3.d	Syringic acid	Sample	12.723	56	80.8968		
22-2B-1.d	Syringic acid	Sample	12.715	29	43.3082		
22-2B-2.d	Syringic acid	Sample	12.723	36	52.8720		
22-2B-3.d	Syringic acid	Sample	12.681	39	57.8204		
22-3B-1.d	Syringic acid	Sample	12.731	54	78.0654		
22-3B-2.d	Syringic acid	Sample	12.715	58	84.1587		
22-3B-3.d	Syringic acid	Sample	12.748	44	64.5884		
22-4B-1.d	Syringic acid	Sample	12.748	175	247.0156		
22-4B-2.d	Syringic acid	Sample	12.723	180	253.8365		
22-4B-3.d	Syringic acid	Sample	12.715	187	264.0732		
22-5B-1.d	Syringic acid	Sample	12.731	166	234.7572		
22-5B-2.d	Syringic acid	Sample	12.723	161	227.9747		

Syringic acid

Data File	Compound	Sample Type	RT	Resp.	Final Conc	Exp. Conc	Accuracy
22-5B-3.d	Syringic acid	Sample	12.723	197	277.6678		
22-6B-1.d	Syringic acid	Sample	12.731	95	135.5494		
22-6B-2.d	Syringic acid	Sample	12.715	100	142.2153		
22-6B-3.d	Syringic acid	Sample	12.731	111	157.6810		
22-7B-1.d	Syringic acid	Sample	12.715	42	62.0190		
22-7B-2.d	Syringic acid	Sample	12.731	65	93.4694		
22-7B-3.d	Syringic acid	Sample	12.731	60	86.0386		
22-8B-1.d	Syringic acid	Sample	12.731	56	81.6417		
22-8B-2.d	Syringic acid	Sample	12.748	56	81.4411		
22-8B-3.d	Syringic acid	Sample	12.740	63	90.6943		
22-9B-1.d	Syringic acid	Sample	12.740	46	67.3496		
22-9B-2.d	Syringic acid	Sample	12.740	45	66.2540		
22-9B-3.d	Syringic acid	Sample	12.715	47	69.1295		
22-10B-1.d	Syringic acid	Sample	12.723	44	63.7631		
22-10B-2.d	Syringic acid	Sample	12.748	41	59.7303		
22-10B-3.d	Syringic acid	Sample	12.748	44	63.8524		
22-11B-1.d	Syringic acid	Sample	12.723	18	27.5295		
22-11B-2.d	Syringic acid	Sample	12.757	23	34.8801		
22-11B-3.d	Syringic acid	Sample	12.723	11	18.7177		
22-12B-1.d	Syringic acid	Sample	12.731	184	259.1650		
22-12B-2.d	Syringic acid	Sample	12.731	171	241.1941		
22-12B-3.d	Syringic acid	Sample	12.715	159	224.1256		
22-13B-1.d	Syringic acid	Sample	12.740	46	66.6859		
22-13B-2.d	Syringic acid	Sample	12.731	64	91.8446		
22-13B-3.d	Syringic acid	Sample	12.748	63	91.0282		
22-14B-1.d	Syringic acid	Sample	12.731	118	167.7921		
22-14B-2.d	Syringic acid	Sample	12.723	141	199.3237		
22-14B-3.d	Syringic acid	Sample	12.740	121	171.9862		
22-15B-1.d	Syringic acid	Sample	12.740	150	212.3293		
22-15B-2.d	Syringic acid	Sample	12.723	124	175.6957		
22-15B-3.d	Syringic acid	Sample	12.723	132	187.6359		
22-16B-1.d	Syringic acid	Sample	12.740	181	255.6330		
22-16B-2.d	Syringic acid	Sample	12.723	177	249.1582		
22-16B-3.d	Syringic acid	Sample	12.757	155	218.7242		
22-17B-1.d	Syringic acid	Sample	12.723	118	167.3613		
22-17B-2.d	Syringic acid	Sample	12.723	141	199.7072		
22-17B-3.d	Syringic acid	Sample	12.731	124	175.4154		
22-18B-1.d	Syringic acid	Sample	12.765	5	9.9191		
22-18B-2.d	Syringic acid	Sample	12.748	3	7.4259		
22-18B-3.d	Syringic acid	Sample	12.748	4	8.5513		

Syringic acid

Data File	Compound	Sample Type	RT	Resp.	Final Conc	Exp. Conc	Accuracy
22-1C-1.d	Syringic acid	Sample	12.757	76	108.5145		
22-1C-2.d	Syringic acid	Sample	12.731	74	106.3247		
22-1C-3.d	Syringic acid	Sample	12.715	79	113.3005		
22-2C-1.d	Syringic acid	Sample	12.723	28	41.8556		
22-2C-2.d	Syringic acid	Sample	12.731	27	40.7995		
22-2C-3.d	Syringic acid	Sample	12.698	34	50.0856		
22-3C-1.d	Syringic acid	Sample	12.715	33	49.1706		
22-3C-2.d	Syringic acid	Sample	12.706	48	69.6813		
22-3C-3.d	Syringic acid	Sample	12.706	36	53.2676		
22-4C-1.d	Syringic acid	Sample	12.731	197	278.3165		
22-4C-2.d	Syringic acid	Sample	12.715	150	211.7132		
22-4C-3.d	Syringic acid	Sample	12.723	188	265.4415		
22-5C-1.d	Syringic acid	Sample	12.715	86	123.5091		
22-5C-2.d	Syringic acid	Sample	12.740	112	158.6824		
22-5C-3.d	Syringic acid	Sample	12.723	101	144.0865		
22-6C-1.d	Syringic acid	Sample	12.698	82	117.3754		
22-6C-2.d	Syringic acid	Sample	12.706	83	119.3490		
22-6C-3.d	Syringic acid	Sample	12.723	88	125.1863		
22-7C-1.d	Syringic acid	Sample	12.731	51	74.0738		
22-7C-2.d	Syringic acid	Sample	12.715	43	63.6168		
22-7C-3.d	Syringic acid	Sample	12.731	51	74.7698		
22-8C-1.d	Syringic acid	Sample	12.748	58	84.3016		
22-8C-2.d	Syringic acid	Sample	12.731	53	77.2046		
22-8C-3.d	Syringic acid	Sample	12.706	57	82.5972		
22-9C-1.d	Syringic acid	Sample	12.765	42	61.2481		
22-9C-2.d	Syringic acid	Sample	12.715	47	68.4973		
22-9C-3.d	Syringic acid	Sample	12.723	47	67.9406		
22-10C-1.d	Syringic acid	Sample	12.715	52	75.3726		
22-10C-2.d	Syringic acid	Sample	12.740	46	67.6929		
22-10C-3.d	Syringic acid	Sample	12.731	49	70.7673		
22-11C-1.d	Syringic acid	Sample	12.723	23	34.8267		
22-11C-2.d	Syringic acid	Sample	12.706	32	47.6163		
22-11C-3.d	Syringic acid	Sample	12.807	24	36.0216		
22-12C-1.d	Syringic acid	Sample	12.740	128	181.1024		
22-12C-2.d	Syringic acid	Sample	12.740	132	186.8109		
22-12C-3.d	Syringic acid	Sample	12.731	147	207.5433		
22-13C-1.d	Syringic acid	Sample	12.731	51	73.7500		
22-13C-2.d	Syringic acid	Sample	12.731	49	71.8619		
22-13C-3.d	Syringic acid	Sample	12.757	54	77.8068		
22-14C-1.d	Syringic acid	Sample	12.723	108	153.4966		

Syringic acid

Data File	Compound	Sample Type	RT	Resp.	Final Conc	Exp. Conc	Accuracy
22-14C-2.d	Syringic acid	Sample	12.757	110	156.0275		
22-14C-3.d	Syringic acid	Sample	12.740	96	136.9484		
22-15C-1.d	Syringic acid	Sample	12.740	109	154.9537		
22-15C-2.d	Syringic acid	Sample	12.731	120	171.0326		
22-15C-3.d	Syringic acid	Sample	12.723	118	168.1878		
22-16C-1.d	Syringic acid	Sample	12.740	169	238.1485		
22-16C-2.d	Syringic acid	Sample	12.748	152	214.9144		
22-16C-3.d	Syringic acid	Sample	12.731	162	229.4832		
22-17C-1.d	Syringic acid	Sample	12.731	102	145.2300		
22-17C-2.d	Syringic acid	Sample	12.740	107	152.2823		
22-17C-3.d	Syringic acid	Sample	12.731	107	152.1974		
22-18C-1.d	Syringic acid	Sample	12.723	12	19.3148		
22-18C-2.d	Syringic acid	Sample	12.748	8	14.7576		
22-18C-3.d	Syringic acid	Sample	12.715	10	16.9158		

Vanillin

Data File	Compound	Sample Type	RT	Resp.	Final Conc	Exp. Conc	Accuracy
blank-061022-4.d	Vanillin	Sample	13.078	3	ND		
25ppb1.d	Vanillin	Calibration	13.053	1	ND		0.00
25ppb2.d	Vanillin	Calibration	13.028	16	ND		0.00
25ppb3.d	Vanillin	Calibration	12.953	45	ND		0.00
50ppb1.d	Vanillin	Calibration	13.012	175	31.3814		62.76
50ppb2.d	Vanillin	Calibration	13.037	258	56.2214		112.44
50ppb3.d	Vanillin	Calibration	13.037	272	60.6644		121.33
100ppb1.d	Vanillin	Calibration	13.037	388	95.4356		95.44
100ppb2.d	Vanillin	Calibration	13.045	379	92.6379		92.64
100ppb3.d	Vanillin	Calibration	13.045	394	97.2655		97.27
250ppb1.d	Vanillin	Calibration	13.053	942	261.5460		104.62
250ppb2.d	Vanillin	Calibration	13.037	884	244.1660		97.67
250ppb3.d	Vanillin	Calibration	13.053	807	221.0151		88.41
500ppb1.d	Vanillin	Calibration	13.053	1941	561.2160		112.24
500ppb2.d	Vanillin	Calibration	13.062	1761	507.1726		101.43
500ppb3.d	Vanillin	Calibration	13.053	1724	496.2029		99.24
1000ppb1.d	Vanillin	Calibration	13.053	3457	1016.2171		101.62
1000ppb2.d	Vanillin	Calibration	13.062	3313	972.8767		97.29
1000ppb3.d	Vanillin	Calibration	13.053	3356	985.9814		98.60
22-1A-1.d	Vanillin	Sample	13.062	378	92.4092		
22-1A-2.d	Vanillin	Sample	13.070	361	87.2215		
22-1A-3.d	Vanillin	Sample	13.053	410	101.9014		
22-2A-1.d	Vanillin	Sample	13.062	360	86.7942		
22-2A-2.d	Vanillin	Sample	13.062	337	80.0829		

Vanillin

Data File	Compound	Sample Type	RT	Resp.	Final Conc	Exp. Conc	Accuracy
22-2A-3.d	Vanillin	Sample	13.053	326	76.6660		
22-3A-1.d	Vanillin	Sample	13.062	174	30.9990		
22-3A-2.d	Vanillin	Sample	13.062	193	36.9351		
22-3A-3.d	Vanillin	Sample	13.062	166	28.5898		
22-4A-1.d	Vanillin	Sample	13.045	665	178.4442		
22-4A-2.d	Vanillin	Sample	13.045	687	185.0347		
22-4A-3.d	Vanillin	Sample	13.045	790	215.9329		
22-5A-1.d	Vanillin	Sample	13.053	914	253.1943		
22-5A-2.d	Vanillin	Sample	13.053	932	258.4712		
22-5A-3.d	Vanillin	Sample	13.053	995	277.3201		
22-6A-1.d	Vanillin	Sample	13.053	2246	652.6891		
22-6A-2.d	Vanillin	Sample	13.045	2420	704.9516		
22-6A-3.d	Vanillin	Sample	13.053	2206	640.6717		
22-7A-1.d	Vanillin	Sample	13.062	529	137.7184		
22-7A-2.d	Vanillin	Sample	13.062	613	162.8440		
22-7A-3.d	Vanillin	Sample	13.062	537	140.1496		
22-8A-1.d	Vanillin	Sample	13.037	603	159.8957		
22-8A-2.d	Vanillin	Sample	13.037	525	136.5216		
22-8A-3.d	Vanillin	Sample	13.053	543	141.8283		
22-9A-1.d	Vanillin	Sample	13.062	265	58.2944		
22-9A-2.d	Vanillin	Sample	13.053	239	50.5934		
22-9A-3.d	Vanillin	Sample	13.045	263	57.7704		
22-10A-1.d	Vanillin	Sample	13.062	239	50.5573		
22-10A-2.d	Vanillin	Sample	13.053	223	45.8745		
22-10A-3.d	Vanillin	Sample	13.053	213	42.7033		
22-11A-1.d	Vanillin	Sample	13.045	480	122.9571		
22-11A-2.d	Vanillin	Sample	13.053	481	123.3339		
22-11A-3.d	Vanillin	Sample	13.045	538	140.2589		
22-12A-1.d	Vanillin	Sample	13.045	182	33.4678		
22-12A-2.d	Vanillin	Sample	13.045	195	37.5502		
22-12A-3.d	Vanillin	Sample	13.045	176	31.8078		
22-13A-1.d	Vanillin	Sample	13.037	604	160.1203		
22-13A-2.d	Vanillin	Sample	13.037	608	161.4579		
22-13A-3.d	Vanillin	Sample	13.053	648	173.3011		
22-14A-1.d	Vanillin	Sample	13.037	1064	298.0279		
22-14A-2.d	Vanillin	Sample	13.045	1020	284.8509		
22-14A-3.d	Vanillin	Sample	13.053	969	269.6028		
22-15A-1.d	Vanillin	Sample	13.045	211	42.1012		
22-15A-2.d	Vanillin	Sample	13.045	190	35.8794		
22-15A-3.d	Vanillin	Sample	13.053	236	49.7218		

Vanillin

Data File	Compound	Sample Type	RT	Resp.	Final Conc	Exp. Conc	Accuracy
22-16A-1.d	Vanillin	Sample	13.045	186	34.6856		
22-16A-2.d	Vanillin	Sample	13.037	212	42.4526		
22-16A-3.d	Vanillin	Sample	13.053	208	41.3925		
22-17A-1.d	Vanillin	Sample	13.028	422	105.5491		
22-17A-2.d	Vanillin	Sample	13.028	448	113.1984		
22-17A-3.d	Vanillin	Sample	13.037	457	116.1759		
22-18A-1.d	Vanillin	Sample	13.020	890	245.9467		
22-18A-2.d	Vanillin	Sample	13.020	811	222.3397		
22-18A-3.d	Vanillin	Sample	13.028	872	240.6008		
22-1B-1.d	Vanillin	Sample	13.037	375	91.5274		
22-1B-2.d	Vanillin	Sample	13.020	389	95.5974		
22-1B-3.d	Vanillin	Sample	13.003	398	98.2997		
22-2B-1.d	Vanillin	Sample	13.028	372	90.5143		
22-2B-2.d	Vanillin	Sample	13.028	425	106.4999		
22-2B-3.d	Vanillin	Sample	13.028	410	101.9722		
22-3B-1.d	Vanillin	Sample	13.012	331	78.2640		
22-3B-2.d	Vanillin	Sample	13.053	353	84.8108		
22-3B-3.d	Vanillin	Sample	13.003	329	77.5870		
22-4B-1.d	Vanillin	Sample	13.012	1084	304.1009		
22-4B-2.d	Vanillin	Sample	13.012	1058	296.2970		
22-4B-3.d	Vanillin	Sample	13.012	1121	315.2577		
22-5B-1.d	Vanillin	Sample	13.020	1481	423.2231		
22-5B-2.d	Vanillin	Sample	13.020	1366	388.6541		
22-5B-3.d	Vanillin	Sample	13.020	1329	377.5551		
22-6B-1.d	Vanillin	Sample	13.028	2473	720.8536		
22-6B-2.d	Vanillin	Sample	13.020	2684	784.0911		
22-6B-3.d	Vanillin	Sample	13.028	2686	784.9379		
22-7B-1.d	Vanillin	Sample	13.028	590	155.8389		
22-7B-2.d	Vanillin	Sample	13.028	644	172.2745		
22-7B-3.d	Vanillin	Sample	13.028	677	181.9252		
22-8B-1.d	Vanillin	Sample	13.028	710	191.8302		
22-8B-2.d	Vanillin	Sample	13.020	776	211.6604		
22-8B-3.d	Vanillin	Sample	13.012	663	177.8948		
22-9B-1.d	Vanillin	Sample	13.020	296	67.8463		
22-9B-2.d	Vanillin	Sample	13.020	307	70.9722		
22-9B-3.d	Vanillin	Sample	13.020	328	77.4709		
22-10B-1.d	Vanillin	Sample	13.020	262	57.5608		
22-10B-2.d	Vanillin	Sample	13.003	225	46.2937		
22-10B-3.d	Vanillin	Sample	13.012	263	57.9437		
22-11B-1.d	Vanillin	Sample	13.020	624	166.1426		

Vanillin

Data File	Compound	Sample Type	RT	Resp.	Final Conc	Exp. Conc	Accuracy
22-11B-2.d	Vanillin	Sample	13.012	610	161.9757		
22-11B-3.d	Vanillin	Sample	13.028	571	150.3672		
22-12B-1.d	Vanillin	Sample	13.020	242	51.5009		
22-12B-2.d	Vanillin	Sample	13.012	276	61.7742		
22-12B-3.d	Vanillin	Sample	13.020	260	56.9000		
22-13B-1.d	Vanillin	Sample	13.037	773	210.8255		
22-13B-2.d	Vanillin	Sample	13.028	791	216.2055		
22-13B-3.d	Vanillin	Sample	13.028	842	231.5115		
22-14B-1.d	Vanillin	Sample	13.028	1039	290.8000		
22-14B-2.d	Vanillin	Sample	13.028	1141	321.1316		
22-14B-3.d	Vanillin	Sample	13.028	1168	329.2849		
22-15B-1.d	Vanillin	Sample	13.028	192	36.5526		
22-15B-2.d	Vanillin	Sample	13.012	232	48.6369		
22-15B-3.d	Vanillin	Sample	13.028	184	34.2396		
22-16B-1.d	Vanillin	Sample	13.028	224	46.1054		
22-16B-2.d	Vanillin	Sample	13.037	225	46.3650		
22-16B-3.d	Vanillin	Sample	13.028	180	33.0104		
22-17B-1.d	Vanillin	Sample	13.012	703	189.8601		
22-17B-2.d	Vanillin	Sample	13.012	695	187.5427		
22-17B-3.d	Vanillin	Sample	13.012	626	166.6395		
22-18B-1.d	Vanillin	Sample	13.028	154	24.9937		
22-18B-2.d	Vanillin	Sample	13.037	133	18.9642		
22-18B-3.d	Vanillin	Sample	13.037	118	14.4122		
22-1C-1.d	Vanillin	Sample	13.012	398	98.4270		
22-1C-2.d	Vanillin	Sample	13.003	388	95.4832		
22-1C-3.d	Vanillin	Sample	13.012	397	98.0444		
22-2C-1.d	Vanillin	Sample	13.020	306	70.7690		
22-2C-2.d	Vanillin	Sample	13.012	304	70.2419		
22-2C-3.d	Vanillin	Sample	13.012	335	79.3275		
22-3C-1.d	Vanillin	Sample	13.028	204	40.1472		
22-3C-2.d	Vanillin	Sample	12.986	196	37.7867		
22-3C-3.d	Vanillin	Sample	13.020	216	43.7334		
22-4C-1.d	Vanillin	Sample	13.003	886	244.6437		
22-4C-2.d	Vanillin	Sample	13.012	900	248.9578		
22-4C-3.d	Vanillin	Sample	13.012	924	256.1700		
22-5C-1.d	Vanillin	Sample	13.020	1062	297.5708		
22-5C-2.d	Vanillin	Sample	13.012	941	261.1815		
22-5C-3.d	Vanillin	Sample	13.003	1028	287.4036		
22-6C-1.d	Vanillin	Sample	13.020	2498	728.4205		
22-6C-2.d	Vanillin	Sample	13.020	2553	745.0164		

Vanillin

Data File	Compound	Sample Type	RT	Resp.	Final Conc	Exp. Conc	Accuracy
22-6C-3.d	Vanillin	Sample	13.012	2517	734.1727		
22-7C-1.d	Vanillin	Sample	13.028	533	138.7280		
22-7C-2.d	Vanillin	Sample	13.037	498	128.3703		
22-7C-3.d	Vanillin	Sample	13.028	569	149.6024		
22-8C-1.d	Vanillin	Sample	13.020	674	180.9990		
22-8C-2.d	Vanillin	Sample	13.012	464	118.1183		
22-8C-3.d	Vanillin	Sample	13.012	589	155.6826		
22-9C-1.d	Vanillin	Sample	13.003	364	88.0442		
22-9C-2.d	Vanillin	Sample	13.020	335	79.5501		
22-9C-3.d	Vanillin	Sample	13.028	293	66.8482		
22-10C-1.d	Vanillin	Sample	13.020	271	60.0900		
22-10C-2.d	Vanillin	Sample	13.020	263	57.7235		
22-10C-3.d	Vanillin	Sample	13.028	284	63.9955		
22-11C-1.d	Vanillin	Sample	13.028	667	179.0191		
22-11C-2.d	Vanillin	Sample	13.028	682	183.4674		
22-11C-3.d	Vanillin	Sample	13.028	647	172.9842		
22-12C-1.d	Vanillin	Sample	13.037	245	52.5309		
22-12C-2.d	Vanillin	Sample	13.028	205	40.2916		
22-12C-3.d	Vanillin	Sample	13.012	202	39.5404		
22-13C-1.d	Vanillin	Sample	13.028	847	233.0441		
22-13C-2.d	Vanillin	Sample	13.028	746	202.8165		
22-13C-3.d	Vanillin	Sample	13.028	762	207.5405		
22-14C-1.d	Vanillin	Sample	13.028	1117	313.9804		
22-14C-2.d	Vanillin	Sample	13.028	1185	334.5845		
22-14C-3.d	Vanillin	Sample	13.020	1157	325.9701		
22-15C-1.d	Vanillin	Sample	13.028	140	20.7779		
22-15C-2.d	Vanillin	Sample	13.020	173	30.8544		
22-15C-3.d	Vanillin	Sample	13.045	169	29.7363		
22-16C-1.d	Vanillin	Sample	13.045	199	38.4905		
22-16C-2.d	Vanillin	Sample	13.020	181	33.2380		
22-16C-3.d	Vanillin	Sample	13.037	169	29.5859		
22-17C-1.d	Vanillin	Sample	13.045	511	132.1653		
22-17C-2.d	Vanillin	Sample	13.037	492	126.5183		
22-17C-3.d	Vanillin	Sample	13.003	579	152.5103		
22-18C-1.d	Vanillin	Sample	13.087	58	ND		
22-18C-2.d	Vanillin	Sample	13.012	43	ND		
22-18C-3.d	Vanillin	Sample	13.045	17	ND		

Verbascoside

Data File	Compound	Sample Type	RT	Resp.	Final Conc	Exp. Conc	Accuracy
blank-061022-4.d	Verbascoside	Sample	13.166	33	ND		

Verbascoside

Data File	Compound	Sample Type	RT	Resp.	Final Conc	Exp. Conc	Accuracy
25ppb1.d	Verbascoside	Calibration	14.129	44	ND		0.00
25ppb2.d	Verbascoside	Calibration	13.434	9	ND		0.00
25ppb3.d	Verbascoside	Calibration	13.334	395	26.0474		104.19
50ppb1.d	Verbascoside	Calibration	13.434	585	45.0338		90.07
50ppb2.d	Verbascoside	Calibration	13.434	529	39.4022		78.80
50ppb3.d	Verbascoside	Calibration	13.467	517	38.2816		76.56
100ppb1.d	Verbascoside	Calibration	13.459	1102	96.8839		96.88
100ppb2.d	Verbascoside	Calibration	13.459	1133	100.0341		100.03
100ppb3.d	Verbascoside	Calibration	13.451	1182	104.9154		104.92
250ppb1.d	Verbascoside	Calibration	13.467	2508	237.9791		95.19
250ppb2.d	Verbascoside	Calibration	13.459	2607	247.8597		99.14
250ppb3.d	Verbascoside	Calibration	13.459	2670	254.1681		101.67
500ppb1.d	Verbascoside	Calibration	13.467	5431	531.0617		106.21
500ppb2.d	Verbascoside	Calibration	13.476	5287	516.6662		103.33
500ppb3.d	Verbascoside	Calibration	13.467	5258	513.7557		102.75
1000ppb1.d	Verbascoside	Calibration	13.467	10297	1019.1654		101.92
1000ppb2.d	Verbascoside	Calibration	13.476	9803	969.6688		96.97
1000ppb3.d	Verbascoside	Calibration	13.459	9947	984.0769		98.41
22-1A-1.d	Verbascoside	Sample	13.467	97	ND		
22-1A-2.d	Verbascoside	Sample	13.467	24	ND		
22-1A-3.d	Verbascoside	Sample	13.451	29	ND		
22-2A-1.d	Verbascoside	Sample	13.551	7	ND		
22-2A-2.d	Verbascoside	Sample	13.693	12	ND		
22-2A-3.d	Verbascoside	Sample	13.459	16	ND		
22-3A-1.d	Verbascoside	Sample	13.459	4568	444.5351		
22-3A-2.d	Verbascoside	Sample	13.467	4468	434.5020		
22-3A-3.d	Verbascoside	Sample	13.467	4691	456.9138		
22-4A-1.d	Verbascoside	Sample	13.392	53	ND		
22-4A-2.d	Verbascoside	Sample	13.501	11	ND		
22-4A-3.d	Verbascoside	Sample	13.442	5	ND		
22-5A-1.d	Verbascoside	Sample	13.459	15	ND		
22-5A-2.d	Verbascoside	Sample	13.493	22	ND		
22-5A-3.d	Verbascoside	Sample	13.467	15	ND		
22-6A-1.d	Verbascoside	Sample	13.359	25	ND		
22-6A-2.d	Verbascoside	Sample	13.300	3	ND		
22-6A-3.d	Verbascoside	Sample	13.484	4	ND		
22-7A-1.d	Verbascoside	Sample	13.777	5	ND		
22-7A-2.d	Verbascoside	Sample	13.476	4	ND		
22-7A-3.d	Verbascoside	Sample	13.459	9	ND		
22-8A-1.d	Verbascoside	Sample	13.459	22	ND		

Verbascoside

Data File	Compound	Sample Type	RT	Resp.	Final Conc	Exp. Conc	Accuracy
22-8A-2.d	Verbascoside	Sample	13.426	37	ND		
22-8A-3.d	Verbascoside	Sample	13.518	6	ND		
22-9A-1.d	Verbascoside	Sample	13.459	371	23.6309		
22-9A-2.d	Verbascoside	Sample	13.459	407	27.1848		
22-9A-3.d	Verbascoside	Sample	13.459	415	27.9903		
22-10A-1.d	Verbascoside	Sample	13.467	27	ND		
22-10A-2.d	Verbascoside	Sample	13.367	60	ND		
22-10A-3.d	Verbascoside	Sample	13.350	71	ND		
22-11A-1.d	Verbascoside	Sample	13.493	3	ND		
22-11A-2.d	Verbascoside	Sample	13.928	4	ND		
22-11A-3.d	Verbascoside	Sample	13.493	5	ND		
22-12A-1.d	Verbascoside	Sample	13.426	4	ND		
22-12A-2.d	Verbascoside	Sample	13.493	4	ND		
22-12A-3.d	Verbascoside	Sample	13.467	10	ND		
22-13A-1.d	Verbascoside	Sample	13.292	2	ND		
22-13A-2.d	Verbascoside	Sample	13.501	2	ND		
22-13A-3.d	Verbascoside	Sample	13.409	7	ND		
22-14A-1.d	Verbascoside	Sample	13.568	12	ND		
22-14A-2.d	Verbascoside	Sample	13.551	21	ND		
22-14A-3.d	Verbascoside	Sample	13.375	3	ND		
22-15A-1.d	Verbascoside	Sample	13.292	3	ND		
22-15A-2.d	Verbascoside	Sample	13.685	2	ND		
22-15A-3.d	Verbascoside	Sample	13.618	1	ND		
22-16A-1.d	Verbascoside	Sample	13.576	5	ND		
22-16A-2.d	Verbascoside	Sample	13.635	3	ND		
22-16A-3.d	Verbascoside	Sample	13.417	7	ND		
22-17A-1.d	Verbascoside	Sample	13.543	3	ND		
22-17A-2.d	Verbascoside	Sample	13.484	3	ND		
22-17A-3.d	Verbascoside	Sample	13.467	4	ND		
22-18A-1.d	Verbascoside	Sample	13.442	46	ND		
22-18A-2.d	Verbascoside	Sample	13.434	61	ND		
22-18A-3.d	Verbascoside	Sample	13.426	74	ND		
22-1B-1.d	Verbascoside	Sample	13.442	16	ND		
22-1B-2.d	Verbascoside	Sample	13.451	15	ND		
22-1B-3.d	Verbascoside	Sample	13.426	11	ND		
22-2B-1.d	Verbascoside	Sample	13.526	2	ND		
22-2B-2.d	Verbascoside	Sample	13.409	7	ND		
22-2B-3.d	Verbascoside	Sample	13.459	4	ND		
22-3B-1.d	Verbascoside	Sample	13.426	7063	694.7648		
22-3B-2.d	Verbascoside	Sample	13.434	6283	616.5870		

Verbascoside

Data File	Compound	Sample Type	RT	Resp.	Final Conc	Exp. Conc	Accuracy
22-3B-3.d	Verbascoside	Sample	13.426	6978	686.2895		
22-4B-1.d	Verbascoside	Sample	13.426	25	ND		
22-4B-2.d	Verbascoside	Sample	13.350	47	ND		
22-4B-3.d	Verbascoside	Sample	13.476	3	ND		
22-5B-1.d	Verbascoside	Sample	13.417	22	ND		
22-5B-2.d	Verbascoside	Sample	13.426	31	ND		
22-5B-3.d	Verbascoside	Sample	13.451	35	ND		
22-6B-1.d	Verbascoside	Sample	13.434	13	ND		
22-6B-2.d	Verbascoside	Sample	13.442	4	ND		
22-6B-3.d	Verbascoside	Sample	13.626	40	ND		
22-7B-1.d	Verbascoside	Sample	13.467	3	ND		
22-7B-2.d	Verbascoside	Sample	13.434	13	ND		
22-7B-3.d	Verbascoside	Sample	13.442	9	ND		
22-8B-1.d	Verbascoside	Sample	13.459	12	ND		
22-8B-2.d	Verbascoside	Sample	13.426	47	ND		
22-8B-3.d	Verbascoside	Sample	13.392	42	ND		
22-9B-1.d	Verbascoside	Sample	13.442	576	44.1494		
22-9B-2.d	Verbascoside	Sample	13.434	634	50.0157		
22-9B-3.d	Verbascoside	Sample	13.442	652	51.7988		
22-10B-1.d	Verbascoside	Sample	13.334	81	ND		
22-10B-2.d	Verbascoside	Sample	13.342	59	ND		
22-10B-3.d	Verbascoside	Sample	13.576	8	ND		
22-11B-1.d	Verbascoside	Sample	13.442	10	ND		
22-11B-2.d	Verbascoside	Sample	13.518	4	ND		
22-11B-3.d	Verbascoside	Sample	13.434	5	ND		
22-12B-1.d	Verbascoside	Sample	13.426	7	ND		
22-12B-2.d	Verbascoside	Sample	13.434	11	ND		
22-12B-3.d	Verbascoside	Sample	13.518	3	ND		
22-13B-1.d	Verbascoside	Sample	13.484	2	ND		
22-13B-2.d	Verbascoside	Sample	13.551	3	ND		
22-13B-3.d	Verbascoside	Sample	13.543	6	ND		
22-14B-1.d	Verbascoside	Sample	13.359	3	ND		
22-14B-2.d	Verbascoside	Sample	13.518	6	ND		
22-14B-3.d	Verbascoside	Sample	13.267	5	ND		
22-15B-1.d	Verbascoside	Sample	14.154	75	ND		
22-15B-2.d	Verbascoside	Sample	13.317	18	ND		
22-15B-3.d	Verbascoside	Sample	13.576	3	ND		
22-16B-1.d	Verbascoside	Sample	14.229	70	ND		
22-16B-2.d	Verbascoside	Sample	13.392	7	ND		
22-16B-3.d	Verbascoside	Sample	13.493	1	ND		

Agilent Technologies

Verbascoside

Data File	Compound	Sample Type	RT	Resp.	Final Conc	Exp. Conc	Accuracy
22-17B-1.d	Verbascoside	Sample	13.426	42	ND		
22-17B-2.d	Verbascoside	Sample	13.467	13	ND		
22-17B-3.d	Verbascoside	Sample	13.426	57	ND		
22-18B-1.d	Verbascoside	Sample	13.417	63	ND		
22-18B-2.d	Verbascoside	Sample	13.417	67	ND		
22-18B-3.d	Verbascoside	Sample	13.434	82	ND		
22-1C-1.d	Verbascoside	Sample	13.434	19	ND		
22-1C-2.d	Verbascoside	Sample	13.417	23	ND		
22-1C-3.d	Verbascoside	Sample	13.426	18	ND		
22-2C-1.d	Verbascoside	Sample	13.426	2	ND		
22-2C-2.d	Verbascoside	Sample	13.451	11	ND		
22-2C-3.d	Verbascoside	Sample	13.610	12	ND		
22-3C-1.d	Verbascoside	Sample	13.434	5142	502.1009		
22-3C-2.d	Verbascoside	Sample	13.426	5634	551.4449		
22-3C-3.d	Verbascoside	Sample	13.426	5443	532.3261		
22-4C-1.d	Verbascoside	Sample	13.576	10	ND		
22-4C-2.d	Verbascoside	Sample	13.476	20	ND		
22-4C-3.d	Verbascoside	Sample	13.559	7	ND		
22-5C-1.d	Verbascoside	Sample	13.426	21	ND		
22-5C-2.d	Verbascoside	Sample	13.434	14	ND		
22-5C-3.d	Verbascoside	Sample	13.467	7	ND		
22-6C-1.d	Verbascoside	Sample	14.221	66	ND		
22-6C-2.d	Verbascoside	Sample	13.409	7	ND		
22-6C-3.d	Verbascoside	Sample	13.375	14	ND		
22-7C-1.d	Verbascoside	Sample	13.459	16	ND		
22-7C-2.d	Verbascoside	Sample	13.417	8	ND		
22-7C-3.d	Verbascoside	Sample	13.417	5	ND		
22-8C-1.d	Verbascoside	Sample	13.426	31	ND		
22-8C-2.d	Verbascoside	Sample	13.493	4	ND		
22-8C-3.d	Verbascoside	Sample	13.434	12	ND		
22-9C-1.d	Verbascoside	Sample	13.434	529	39.4723		
22-9C-2.d	Verbascoside	Sample	13.426	516	38.1724		
22-9C-3.d	Verbascoside	Sample	13.434	549	41.4033		
22-10C-1.d	Verbascoside	Sample	13.325	85	ND		
22-10C-2.d	Verbascoside	Sample	13.334	54	ND		
22-10C-3.d	Verbascoside	Sample	13.467	10	ND		
22-11C-1.d	Verbascoside	Sample	13.534	3	ND		
22-11C-2.d	Verbascoside	Sample	13.442	8	ND		
22-11C-3.d	Verbascoside	Sample	13.777	7	ND		
22-12C-1.d	Verbascoside	Sample	13.484	8	ND		

Verbascoside

Data File	Compound	Sample Type	RT	Resp.	Final Conc	Exp. Conc	Accuracy
22-12C-2.d	Verbascoside	Sample	13.459	6	ND		
22-12C-3.d	Verbascoside	Sample	13.426	5	ND		
22-13C-1.d	Verbascoside	Sample	14.062	23	ND		
22-13C-2.d	Verbascoside	Sample	13.192	1	ND		
22-13C-3.d	Verbascoside	Sample	13.526	3	ND		
22-14C-1.d	Verbascoside	Sample	14.271	82	ND		
22-14C-2.d	Verbascoside	Sample	13.467	1	ND		
22-14C-3.d	Verbascoside	Sample	13.509	6	ND		
22-15C-1.d	Verbascoside	Sample	13.710	3	ND		
22-15C-2.d	Verbascoside	Sample	13.852	2	ND		
22-15C-3.d	Verbascoside	Sample	14.229	74	ND		
22-16C-1.d	Verbascoside	Sample	13.978	55	ND		
22-16C-2.d	Verbascoside	Sample	13.576	16	ND		
22-16C-3.d	Verbascoside	Sample	13.610	5	ND		
22-17C-1.d	Verbascoside	Sample	13.434	21	ND		
22-17C-2.d	Verbascoside	Sample	13.434	2	ND		
22-17C-3.d	Verbascoside	Sample	13.467	3	ND		
22-18C-1.d	Verbascoside	Sample	14.187	74	ND		
22-18C-2.d	Verbascoside	Sample	13.451	2	ND		
22-18C-3.d	Verbascoside	Sample	13.543	3	ND		

Taxifolin

Data File	Compound	Sample Type	RT	Resp.	Final Conc	Exp. Conc	Accuracy
blank-061022-4.d	Taxifolin	Sample	13.695	1	ND		
25ppb1.d	Taxifolin	Calibration	13.779	3	ND		0.00
25ppb2.d	Taxifolin	Calibration	13.695	2	ND		0.00
25ppb3.d	Taxifolin	Calibration	13.595	932	ND		0.00
50ppb1.d	Taxifolin	Calibration	13.661	1923	29.9648		59.93
50ppb2.d	Taxifolin	Calibration	13.670	1650	19.0826		38.17
50ppb3.d	Taxifolin	Calibration	13.678	1717	21.7492		43.50
100ppb1.d	Taxifolin	Calibration	13.695	3803	104.9985		105.00
100ppb2.d	Taxifolin	Calibration	13.695	3529	94.0705		94.07
100ppb3.d	Taxifolin	Calibration	13.695	3736	102.3347		102.33
250ppb1.d	Taxifolin	Calibration	13.703	7653	258.6576		103.46
250ppb2.d	Taxifolin	Calibration	13.695	7668	259.2388		103.70
250ppb3.d	Taxifolin	Calibration	13.695	7709	260.8713		104.35
500ppb1.d	Taxifolin	Calibration	13.712	15077	554.9196		110.98
500ppb2.d	Taxifolin	Calibration	13.712	14931	549.1162		109.82
500ppb3.d	Taxifolin	Calibration	13.712	15410	568.2097		113.64
1000ppb1.d	Taxifolin	Calibration	13.712	25309	963.2799		96.33
1000ppb2.d	Taxifolin	Calibration	13.712	25452	969.0002		96.90

Taxifolin

Data File	Compound	Sample Type	RT	Resp.	Final Conc	Exp. Conc	Accuracy
1000ppb3.d	Taxifolin	Calibration	13.703	25705	979.0896		97.91
22-1A-1.d	Taxifolin	Sample	13.712	41290	1601.0538		
22-1A-2.d	Taxifolin	Sample	13.712	42976	1668.3462		
22-1A-3.d	Taxifolin	Sample	13.703	42533	1650.6729		
22-2A-1.d	Taxifolin	Sample	13.728	401	ND		
22-2A-2.d	Taxifolin	Sample	13.720	377	ND		
22-2A-3.d	Taxifolin	Sample	13.703	415	ND		
22-3A-1.d	Taxifolin	Sample	13.695	387	ND		
22-3A-2.d	Taxifolin	Sample	13.703	336	ND		
22-3A-3.d	Taxifolin	Sample	13.712	325	ND		
22-4A-1.d	Taxifolin	Sample	13.703	719	ND		
22-4A-2.d	Taxifolin	Sample	13.712	764	ND		
22-4A-3.d	Taxifolin	Sample	13.703	666	ND		
22-5A-1.d	Taxifolin	Sample	13.712	883	ND		
22-5A-2.d	Taxifolin	Sample	13.703	805	ND		
22-5A-3.d	Taxifolin	Sample	13.695	941	ND		
22-6A-1.d	Taxifolin	Sample	13.703	4826	145.8184		
22-6A-2.d	Taxifolin	Sample	13.695	4721	141.6305		
22-6A-3.d	Taxifolin	Sample	13.703	4821	145.6243		
22-7A-1.d	Taxifolin	Sample	13.695	20	ND		
22-7A-2.d	Taxifolin	Sample	13.678	21	ND		
22-7A-3.d	Taxifolin	Sample	13.728	16	ND		
22-8A-1.d	Taxifolin	Sample	13.703	289	ND		
22-8A-2.d	Taxifolin	Sample	13.686	261	ND		
22-8A-3.d	Taxifolin	Sample	13.703	256	ND		
22-9A-1.d	Taxifolin	Sample	13.703	88	ND		
22-9A-2.d	Taxifolin	Sample	13.712	121	ND		
22-9A-3.d	Taxifolin	Sample	13.695	120	ND		
22-10A-1.d	Taxifolin	Sample	13.712	176	ND		
22-10A-2.d	Taxifolin	Sample	13.712	153	ND		
22-10A-3.d	Taxifolin	Sample	13.703	173	ND		
22-11A-1.d	Taxifolin	Sample	13.703	574	ND		
22-11A-2.d	Taxifolin	Sample	13.695	672	ND		
22-11A-3.d	Taxifolin	Sample	13.703	554	ND		
22-12A-1.d	Taxifolin	Sample	13.703	51	ND		
22-12A-2.d	Taxifolin	Sample	13.720	62	ND		
22-12A-3.d	Taxifolin	Sample	13.712	49	ND		
22-13A-1.d	Taxifolin	Sample	13.712	36	ND		
22-13A-2.d	Taxifolin	Sample	13.703	38	ND		
22-13A-3.d	Taxifolin	Sample	13.745	50	ND		

Taxifolin

Data File	Compound	Sample Type	RT	Resp.	Final Conc	Exp. Conc	Accuracy
22-14A-1.d	Taxifolin	Sample	13.686	55	ND		
22-14A-2.d	Taxifolin	Sample	13.703	34	ND		
22-14A-3.d	Taxifolin	Sample	13.678	20	ND		
22-15A-1.d	Taxifolin	Sample	13.712	5	ND		
22-15A-2.d	Taxifolin	Sample	13.670	8	ND		
22-15A-3.d	Taxifolin	Sample	13.661	33	ND		
22-16A-1.d	Taxifolin	Sample	13.712	105	ND		
22-16A-2.d	Taxifolin	Sample	13.720	59	ND		
22-16A-3.d	Taxifolin	Sample	13.720	75	ND		
22-17A-1.d	Taxifolin	Sample	13.728	9	ND		
22-17A-2.d	Taxifolin	Sample	13.712	19	ND		
22-17A-3.d	Taxifolin	Sample	13.913	10	ND		
22-18A-1.d	Taxifolin	Sample	13.678	2427	50.1077		
22-18A-2.d	Taxifolin	Sample	13.653	2202	41.1109		
22-18A-3.d	Taxifolin	Sample	13.678	2241	42.6738		
22-1B-1.d	Taxifolin	Sample	13.670	45908	1785.3433		
22-1B-2.d	Taxifolin	Sample	13.670	44115	1713.8127		
22-1B-3.d	Taxifolin	Sample	13.670	43936	1706.6519		
22-2B-1.d	Taxifolin	Sample	13.678	438	ND		
22-2B-2.d	Taxifolin	Sample	13.678	483	ND		
22-2B-3.d	Taxifolin	Sample	13.661	389	ND		
22-3B-1.d	Taxifolin	Sample	13.661	379	ND		
22-3B-2.d	Taxifolin	Sample	13.661	358	ND		
22-3B-3.d	Taxifolin	Sample	13.670	408	ND		
22-4B-1.d	Taxifolin	Sample	13.670	803	ND		
22-4B-2.d	Taxifolin	Sample	13.661	783	ND		
22-4B-3.d	Taxifolin	Sample	13.670	681	ND		
22-5B-1.d	Taxifolin	Sample	13.661	1140	ND		
22-5B-2.d	Taxifolin	Sample	13.661	1120	ND		
22-5B-3.d	Taxifolin	Sample	13.678	1138	ND		
22-6B-1.d	Taxifolin	Sample	13.670	5905	188.9019		
22-6B-2.d	Taxifolin	Sample	13.670	6047	194.5682		
22-6B-3.d	Taxifolin	Sample	13.661	5998	192.6075		
22-7B-1.d	Taxifolin	Sample	13.720	18	ND		
22-7B-2.d	Taxifolin	Sample	13.712	21	ND		
22-7B-3.d	Taxifolin	Sample	13.753	8	ND		
22-8B-1.d	Taxifolin	Sample	13.686	293	ND		
22-8B-2.d	Taxifolin	Sample	13.678	297	ND		
22-8B-3.d	Taxifolin	Sample	13.670	302	ND		
22-9B-1.d	Taxifolin	Sample	13.703	173	ND		

Taxifolin

Data File	Compound	Sample Type	RT	Resp.	Final Conc	Exp. Conc	Accuracy
22-9B-2.d	Taxifolin	Sample	13.661	145	ND		
22-9B-3.d	Taxifolin	Sample	13.670	182	ND		
22-10B-1.d	Taxifolin	Sample	13.670	148	ND		
22-10B-2.d	Taxifolin	Sample	13.678	161	ND		
22-10B-3.d	Taxifolin	Sample	13.678	106	ND		
22-11B-1.d	Taxifolin	Sample	13.670	656	ND		
22-11B-2.d	Taxifolin	Sample	13.661	694	ND		
22-11B-3.d	Taxifolin	Sample	13.678	831	ND		
22-12B-1.d	Taxifolin	Sample	13.653	58	ND		
22-12B-2.d	Taxifolin	Sample	13.695	42	ND		
22-12B-3.d	Taxifolin	Sample	13.678	75	ND		
22-13B-1.d	Taxifolin	Sample	13.728	55	ND		
22-13B-2.d	Taxifolin	Sample	13.678	58	ND		
22-13B-3.d	Taxifolin	Sample	13.661	23	ND		
22-14B-1.d	Taxifolin	Sample	13.712	5	ND		
22-14B-2.d	Taxifolin	Sample	13.670	22	ND		
22-14B-3.d	Taxifolin	Sample	13.678	36	ND		
22-15B-1.d	Taxifolin	Sample	13.695	19	ND		
22-15B-2.d	Taxifolin	Sample	13.661	28	ND		
22-15B-3.d	Taxifolin	Sample	13.728	8	ND		
22-16B-1.d	Taxifolin	Sample	13.670	39	ND		
22-16B-2.d	Taxifolin	Sample	13.670	66	ND		
22-16B-3.d	Taxifolin	Sample	13.670	79	ND		
22-17B-1.d	Taxifolin	Sample	13.720	5	ND		
22-17B-2.d	Taxifolin	Sample	13.753	3	ND		
22-17B-3.d	Taxifolin	Sample	13.720	10	ND		
22-18B-1.d	Taxifolin	Sample	13.670	143	ND		
22-18B-2.d	Taxifolin	Sample	13.695	179	ND		
22-18B-3.d	Taxifolin	Sample	13.678	176	ND		
22-1C-1.d	Taxifolin	Sample	13.661	48987	1908.2237		
22-1C-2.d	Taxifolin	Sample	13.661	48724	1897.7397		
22-1C-3.d	Taxifolin	Sample	13.661	49073	1911.6634		
22-2C-1.d	Taxifolin	Sample	13.670	727	ND		
22-2C-2.d	Taxifolin	Sample	13.661	567	ND		
22-2C-3.d	Taxifolin	Sample	13.678	655	ND		
22-3C-1.d	Taxifolin	Sample	13.661	422	ND		
22-3C-2.d	Taxifolin	Sample	13.653	491	ND		
22-3C-3.d	Taxifolin	Sample	13.661	484	ND		
22-4C-1.d	Taxifolin	Sample	13.670	645	ND		
22-4C-2.d	Taxifolin	Sample	13.661	802	ND		

Taxifolin

Data File	Compound	Sample Type	RT	Resp.	Final Conc	Exp. Conc	Accuracy
22-4C-3.d	Taxifolin	Sample	13.661	736	ND		
22-5C-1.d	Taxifolin	Sample	13.670	743	ND		
22-5C-2.d	Taxifolin	Sample	13.661	649	ND		
22-5C-3.d	Taxifolin	Sample	13.678	754	ND		
22-6C-1.d	Taxifolin	Sample	13.661	5140	158.3776		
22-6C-2.d	Taxifolin	Sample	13.661	5288	164.2905		
22-6C-3.d	Taxifolin	Sample	13.661	5490	172.3213		
22-7C-1.d	Taxifolin	Sample	13.745	14	ND		
22-7C-2.d	Taxifolin	Sample	13.745	11	ND		
22-7C-3.d	Taxifolin	Sample	13.628	18	ND		
22-8C-1.d	Taxifolin	Sample	13.686	251	ND		
22-8C-2.d	Taxifolin	Sample	13.686	280	ND		
22-8C-3.d	Taxifolin	Sample	13.678	256	ND		
22-9C-1.d	Taxifolin	Sample	13.670	135	ND		
22-9C-2.d	Taxifolin	Sample	13.670	161	ND		
22-9C-3.d	Taxifolin	Sample	13.670	143	ND		
22-10C-1.d	Taxifolin	Sample	13.670	200	ND		
22-10C-2.d	Taxifolin	Sample	13.695	180	ND		
22-10C-3.d	Taxifolin	Sample	13.670	166	ND		
22-11C-1.d	Taxifolin	Sample	13.678	857	ND		
22-11C-2.d	Taxifolin	Sample	13.686	694	ND		
22-11C-3.d	Taxifolin	Sample	13.678	756	ND		
22-12C-1.d	Taxifolin	Sample	13.686	91	ND		
22-12C-2.d	Taxifolin	Sample	13.661	59	ND		
22-12C-3.d	Taxifolin	Sample	13.678	83	ND		
22-13C-1.d	Taxifolin	Sample	13.678	33	ND		
22-13C-2.d	Taxifolin	Sample	13.686	34	ND		
22-13C-3.d	Taxifolin	Sample	13.686	32	ND		
22-14C-1.d	Taxifolin	Sample	13.678	6	ND		
22-14C-2.d	Taxifolin	Sample	13.703	3	ND		
22-14C-3.d	Taxifolin	Sample	13.653	17	ND		
22-15C-1.d	Taxifolin	Sample	13.653	29	ND		
22-15C-2.d	Taxifolin	Sample	13.745	10	ND		
22-15C-3.d	Taxifolin	Sample	13.670	8	ND		
22-16C-1.d	Taxifolin	Sample	13.678	51	ND		
22-16C-2.d	Taxifolin	Sample	13.720	71	ND		
22-16C-3.d	Taxifolin	Sample	13.686	61	ND		
22-17C-1.d	Taxifolin	Sample	13.695	8	ND		
22-17C-2.d	Taxifolin	Sample	13.728	6	ND		
22-17C-3.d	Taxifolin	Sample	13.737	11	ND		

Taxifolin

Data File	Compound	Sample Type	RT	Resp.	Final Conc	Exp. Conc	Accuracy
22-18C-1.d	Taxifolin	Sample	13.686	1756	23.3301		
22-18C-2.d	Taxifolin	Sample	13.686	1711	21.5091		
22-18C-3.d	Taxifolin	Sample	13.686	1753	23.2146		

p-Coumaric acid

Data File	Compound	Sample Type	RT	Resp.	Final Conc	Exp. Conc	Accuracy
blank-061022-4.d	p-Coumaric acid	Sample	13.758	13	ND		
25ppb1.d	p-Coumaric acid	Calibration	13.758	29	ND		0.00
25ppb2.d	p-Coumaric acid	Calibration	14.117	243	0.1075		0.43
25ppb3.d	p-Coumaric acid	Calibration	13.632	486	9.5755		38.30
50ppb1.d	p-Coumaric acid	Calibration	13.741	1106	33.7852		67.57
50ppb2.d	p-Coumaric acid	Calibration	13.750	1400	45.2729		90.55
50ppb3.d	p-Coumaric acid	Calibration	13.766	1381	44.5407		89.08
100ppb1.d	p-Coumaric acid	Calibration	13.775	3027	108.8030		108.80
100ppb2.d	p-Coumaric acid	Calibration	13.784	3079	110.8228		110.82
100ppb3.d	p-Coumaric acid	Calibration	13.784	3173	114.5153		114.52
250ppb1.d	p-Coumaric acid	Calibration	13.792	6865	258.6283		103.45
250ppb2.d	p-Coumaric acid	Calibration	13.784	6933	261.2789		104.51
250ppb3.d	p-Coumaric acid	Calibration	13.784	6909	260.3475		104.14
500ppb1.d	p-Coumaric acid	Calibration	13.792	13899	533.2423		106.65
500ppb2.d	p-Coumaric acid	Calibration	13.801	13748	527.3728		105.47
500ppb3.d	p-Coumaric acid	Calibration	13.792	13718	526.1957		105.24
1000ppb1.d	p-Coumaric acid	Calibration	13.801	25973	1004.6535		100.47
1000ppb2.d	p-Coumaric acid	Calibration	13.801	24851	960.8410		96.08
1000ppb3.d	p-Coumaric acid	Calibration	13.792	25426	983.2819		98.33
22-1A-1.d	p-Coumaric acid	Sample	13.775	1520	49.9776		
22-1A-2.d	p-Coumaric acid	Sample	13.784	1572	51.9794		
22-1A-3.d	p-Coumaric acid	Sample	13.775	1557	51.4130		
22-2A-1.d	p-Coumaric acid	Sample	13.775	2530	89.3859		
22-2A-2.d	p-Coumaric acid	Sample	13.784	2571	91.0097		
22-2A-3.d	p-Coumaric acid	Sample	13.775	2449	86.2313		
22-3A-1.d	p-Coumaric acid	Sample	13.775	3138	113.1174		
22-3A-2.d	p-Coumaric acid	Sample	13.784	3254	117.6580		
22-3A-3.d	p-Coumaric acid	Sample	13.784	3123	112.5359		
22-4A-1.d	p-Coumaric acid	Sample	13.784	3973	145.7404		
22-4A-2.d	p-Coumaric acid	Sample	13.784	4247	156.4355		
22-4A-3.d	p-Coumaric acid	Sample	13.784	4099	150.6368		
22-5A-1.d	p-Coumaric acid	Sample	13.792	12359	473.1371		
22-5A-2.d	p-Coumaric acid	Sample	13.784	12367	473.4393		
22-5A-3.d	p-Coumaric acid	Sample	13.792	12491	478.2846		
22-6A-1.d	p-Coumaric acid	Sample	13.784	8426	319.5871		

Agilent Technologies

p-Coumaric acid

Data File	Compound	Sample Type	RT	Resp.	Final Conc	Exp. Conc	Accuracy
22-6A-2.d	p-Coumaric acid	Sample	13.784	7849	297.0465		
22-6A-3.d	p-Coumaric acid	Sample	13.792	8234	312.0734		
22-7A-1.d	p-Coumaric acid	Sample	13.801	4334	159.8320		
22-7A-2.d	p-Coumaric acid	Sample	13.801	4304	158.6582		
22-7A-3.d	p-Coumaric acid	Sample	13.801	4286	157.9477		
22-8A-1.d	p-Coumaric acid	Sample	13.784	3012	108.2035		
22-8A-2.d	p-Coumaric acid	Sample	13.775	3049	109.6473		
22-8A-3.d	p-Coumaric acid	Sample	13.775	3302	119.5486		
22-9A-1.d	p-Coumaric acid	Sample	13.792	5164	192.2131		
22-9A-2.d	p-Coumaric acid	Sample	13.792	5355	199.6807		
22-9A-3.d	p-Coumaric acid	Sample	13.784	5405	201.6514		
22-10A-1.d	p-Coumaric acid	Sample	13.842	5009	186.1928		
22-10A-2.d	p-Coumaric acid	Sample	13.809	5174	192.6067		
22-10A-3.d	p-Coumaric acid	Sample	13.859	5110	190.1245		
22-11A-1.d	p-Coumaric acid	Sample	13.792	4366	161.0671		
22-11A-2.d	p-Coumaric acid	Sample	13.792	4417	163.0553		
22-11A-3.d	p-Coumaric acid	Sample	13.792	4331	159.7057		
22-12A-1.d	p-Coumaric acid	Sample	13.792	2341	82.0294		
22-12A-2.d	p-Coumaric acid	Sample	13.784	2383	83.6579		
22-12A-3.d	p-Coumaric acid	Sample	13.792	2419	85.0802		
22-13A-1.d	p-Coumaric acid	Sample	13.775	8893	337.8193		
22-13A-2.d	p-Coumaric acid	Sample	13.775	8095	306.6793		
22-13A-3.d	p-Coumaric acid	Sample	13.784	8394	318.3203		
22-14A-1.d	p-Coumaric acid	Sample	13.784	18773	723.5635		
22-14A-2.d	p-Coumaric acid	Sample	13.792	18746	722.4935		
22-14A-3.d	p-Coumaric acid	Sample	13.792	18625	717.7674		
22-15A-1.d	p-Coumaric acid	Sample	13.792	9368	356.3704		
22-15A-2.d	p-Coumaric acid	Sample	13.792	9271	352.5828		
22-15A-3.d	p-Coumaric acid	Sample	13.792	9484	360.8727		
22-16A-1.d	p-Coumaric acid	Sample	13.792	7905	299.2460		
22-16A-2.d	p-Coumaric acid	Sample	13.801	7924	299.9765		
22-16A-3.d	p-Coumaric acid	Sample	13.792	8055	305.1060		
22-17A-1.d	p-Coumaric acid	Sample	13.784	4508	166.6115		
22-17A-2.d	p-Coumaric acid	Sample	13.775	4590	169.8385		
22-17A-3.d	p-Coumaric acid	Sample	13.775	4468	165.0557		
22-18A-1.d	p-Coumaric acid	Sample	13.758	22916	885.2972		
22-18A-2.d	p-Coumaric acid	Sample	13.758	22105	853.6441		
22-18A-3.d	p-Coumaric acid	Sample	13.758	21860	844.0767		
22-1B-1.d	p-Coumaric acid	Sample	13.741	2144	74.3109		
22-1B-2.d	p-Coumaric acid	Sample	13.741	2091	72.2410		

p-Coumaric acid

Data File	Compound	Sample Type	RT	Resp.	Final Conc	Exp. Conc	Accuracy
22-1B-3.d	p-Coumaric acid	Sample	13.733	2055	70.8451		
22-2B-1.d	p-Coumaric acid	Sample	13.741	3211	115.9940		
22-2B-2.d	p-Coumaric acid	Sample	13.741	3400	123.3781		
22-2B-3.d	p-Coumaric acid	Sample	13.741	3047	109.5664		
22-3B-1.d	p-Coumaric acid	Sample	13.741	4924	182.8666		
22-3B-2.d	p-Coumaric acid	Sample	13.741	4628	171.3075		
22-3B-3.d	p-Coumaric acid	Sample	13.741	5149	191.6358		
22-4B-1.d	p-Coumaric acid	Sample	13.750	5290	197.1657		
22-4B-2.d	p-Coumaric acid	Sample	13.750	5792	216.7637		
22-4B-3.d	p-Coumaric acid	Sample	13.750	5470	204.1825		
22-5B-1.d	p-Coumaric acid	Sample	13.750	16853	648.5963		
22-5B-2.d	p-Coumaric acid	Sample	13.750	16980	653.5302		
22-5B-3.d	p-Coumaric acid	Sample	13.750	17121	659.0516		
22-6B-1.d	p-Coumaric acid	Sample	13.758	14769	567.2114		
22-6B-2.d	p-Coumaric acid	Sample	13.750	13804	529.5652		
22-6B-3.d	p-Coumaric acid	Sample	13.758	13941	534.8976		
22-7B-1.d	p-Coumaric acid	Sample	13.766	4977	184.9179		
22-7B-2.d	p-Coumaric acid	Sample	13.766	5021	186.6335		
22-7B-3.d	p-Coumaric acid	Sample	13.775	5095	189.5260		
22-8B-1.d	p-Coumaric acid	Sample	13.750	4210	155.0012		
22-8B-2.d	p-Coumaric acid	Sample	13.750	4037	148.2455		
22-8B-3.d	p-Coumaric acid	Sample	13.750	4307	158.7647		
22-9B-1.d	p-Coumaric acid	Sample	13.758	7048	265.7696		
22-9B-2.d	p-Coumaric acid	Sample	13.758	6587	247.7825		
22-9B-3.d	p-Coumaric acid	Sample	13.758	6700	252.2158		
22-10B-1.d	p-Coumaric acid	Sample	13.826	5806	217.2876		
22-10B-2.d	p-Coumaric acid	Sample	13.826	5962	223.3733		
22-10B-3.d	p-Coumaric acid	Sample	13.809	5721	213.9745		
22-11B-1.d	p-Coumaric acid	Sample	13.758	5884	220.3500		
22-11B-2.d	p-Coumaric acid	Sample	13.758	5869	219.7585		
22-11B-3.d	p-Coumaric acid	Sample	13.758	5941	222.5496		
22-12B-1.d	p-Coumaric acid	Sample	13.758	3152	113.6707		
22-12B-2.d	p-Coumaric acid	Sample	13.758	3342	121.0772		
22-12B-3.d	p-Coumaric acid	Sample	13.758	3140	113.2112		
22-13B-1.d	p-Coumaric acid	Sample	13.758	10445	398.3974		
22-13B-2.d	p-Coumaric acid	Sample	13.750	11218	428.5710		
22-13B-3.d	p-Coumaric acid	Sample	13.750	11055	422.2307		
22-14B-1.d	p-Coumaric acid	Sample	13.758	24685	954.3519		
22-14B-2.d	p-Coumaric acid	Sample	13.758	24614	951.6063		
22-14B-3.d	p-Coumaric acid	Sample	13.758	25026	967.6789		

p-Coumaric acid

Data File	Compound	Sample Type	RT	Resp.	Final Conc	Exp. Conc	Accuracy
22-15B-1.d	p-Coumaric acid	Sample	13.758	8401	318.6026		
22-15B-2.d	p-Coumaric acid	Sample	13.758	8546	324.2599		
22-15B-3.d	p-Coumaric acid	Sample	13.758	8441	320.1794		
22-16B-1.d	p-Coumaric acid	Sample	13.758	8082	306.1590		
22-16B-2.d	p-Coumaric acid	Sample	13.758	7952	301.0919		
22-16B-3.d	p-Coumaric acid	Sample	13.758	7789	294.7185		
22-17B-1.d	p-Coumaric acid	Sample	13.758	6022	225.7146		
22-17B-2.d	p-Coumaric acid	Sample	13.750	6388	240.0005		
22-17B-3.d	p-Coumaric acid	Sample	13.758	6033	226.1713		
22-18B-1.d	p-Coumaric acid	Sample	13.750	3931	144.0803		
22-18B-2.d	p-Coumaric acid	Sample	13.750	3914	143.4280		
22-18B-3.d	p-Coumaric acid	Sample	13.750	4046	148.5644		
22-1C-1.d	p-Coumaric acid	Sample	13.733	1944	66.5239		
22-1C-2.d	p-Coumaric acid	Sample	13.741	2021	69.5415		
22-1C-3.d	p-Coumaric acid	Sample	13.733	2055	70.8432		
22-2C-1.d	p-Coumaric acid	Sample	13.733	2837	101.3888		
22-2C-2.d	p-Coumaric acid	Sample	13.733	2616	92.7667		
22-2C-3.d	p-Coumaric acid	Sample	13.733	2673	94.9710		
22-3C-1.d	p-Coumaric acid	Sample	13.741	3590	130.7912		
22-3C-2.d	p-Coumaric acid	Sample	13.741	3546	129.0481		
22-3C-3.d	p-Coumaric acid	Sample	13.741	3612	131.6557		
22-4C-1.d	p-Coumaric acid	Sample	13.750	4906	182.1569		
22-4C-2.d	p-Coumaric acid	Sample	13.741	5091	189.3614		
22-4C-3.d	p-Coumaric acid	Sample	13.741	4847	179.8475		
22-5C-1.d	p-Coumaric acid	Sample	13.750	12838	491.8457		
22-5C-2.d	p-Coumaric acid	Sample	13.750	12821	491.1588		
22-5C-3.d	p-Coumaric acid	Sample	13.750	13413	514.3007		
22-6C-1.d	p-Coumaric acid	Sample	13.750	9827	374.2912		
22-6C-2.d	p-Coumaric acid	Sample	13.750	10909	416.5169		
22-6C-3.d	p-Coumaric acid	Sample	13.750	10434	397.9828		
22-7C-1.d	p-Coumaric acid	Sample	13.766	4406	162.6197		
22-7C-2.d	p-Coumaric acid	Sample	13.775	4732	175.3457		
22-7C-3.d	p-Coumaric acid	Sample	13.758	4323	159.3843		
22-8C-1.d	p-Coumaric acid	Sample	13.750	3510	127.6462		
22-8C-2.d	p-Coumaric acid	Sample	13.750	3421	124.1922		
22-8C-3.d	p-Coumaric acid	Sample	13.750	3439	124.8994		
22-9C-1.d	p-Coumaric acid	Sample	13.758	6561	246.7550		
22-9C-2.d	p-Coumaric acid	Sample	13.758	6443	242.1513		
22-9C-3.d	p-Coumaric acid	Sample	13.758	6391	240.1374		
22-10C-1.d	p-Coumaric acid	Sample	13.817	6579	247.4879		

p-Coumaric acid

Data File	Compound	Sample Type	RT	Resp.	Final Conc	Exp. Conc	Accuracy
22-10C-2.d	p-Coumaric acid	Sample	13.826	6426	241.5190		
22-10C-3.d	p-Coumaric acid	Sample	13.826	6616	248.9187		
22-11C-1.d	p-Coumaric acid	Sample	13.766	6121	229.6120		
22-11C-2.d	p-Coumaric acid	Sample	13.766	6060	227.1930		
22-11C-3.d	p-Coumaric acid	Sample	13.766	6101	228.8062		
22-12C-1.d	p-Coumaric acid	Sample	13.766	2762	98.4458		
22-12C-2.d	p-Coumaric acid	Sample	13.766	2884	103.2299		
22-12C-3.d	p-Coumaric acid	Sample	13.766	2933	105.1253		
22-13C-1.d	p-Coumaric acid	Sample	13.766	9812	373.6981		
22-13C-2.d	p-Coumaric acid	Sample	13.766	11071	422.8409		
22-13C-3.d	p-Coumaric acid	Sample	13.758	10321	393.5805		
22-14C-1.d	p-Coumaric acid	Sample	13.766	21153	816.4673		
22-14C-2.d	p-Coumaric acid	Sample	13.766	20213	779.7518		
22-14C-3.d	p-Coumaric acid	Sample	13.766	21045	812.2371		
22-15C-1.d	p-Coumaric acid	Sample	13.766	8028	304.0516		
22-15C-2.d	p-Coumaric acid	Sample	13.766	7856	297.3239		
22-15C-3.d	p-Coumaric acid	Sample	13.766	8338	316.1573		
22-16C-1.d	p-Coumaric acid	Sample	13.766	6957	262.2286		
22-16C-2.d	p-Coumaric acid	Sample	13.775	6875	259.0336		
22-16C-3.d	p-Coumaric acid	Sample	13.775	7105	267.9945		
22-17C-1.d	p-Coumaric acid	Sample	13.775	5424	202.3656		
22-17C-2.d	p-Coumaric acid	Sample	13.775	5341	199.1392		
22-17C-3.d	p-Coumaric acid	Sample	13.775	5272	196.4390		
22-18C-1.d	p-Coumaric acid	Sample	13.842	18066	695.9568		
22-18C-2.d	p-Coumaric acid	Sample	13.834	18445	710.7462		
22-18C-3.d	p-Coumaric acid	Sample	13.834	18615	717.3695		

Sinapic acid

Data File	Compound	Sample Type	RT	Resp.	Final Conc	Exp. Conc	Accuracy
blank-061022-4.d	Sinapic acid	Sample	13.806	11	7.8742		
25ppb1.d	Sinapic acid	Calibration	13.839	1	4.5522		18.21
25ppb2.d	Sinapic acid	Calibration	13.823	16	9.3416		37.37
25ppb3.d	Sinapic acid	Calibration	13.730	50	19.9996		80.00
50ppb1.d	Sinapic acid	Calibration	13.806	120	42.2062		84.41
50ppb2.d	Sinapic acid	Calibration	13.814	119	41.8883		83.78
50ppb3.d	Sinapic acid	Calibration	13.839	151	51.8771		103.75
100ppb1.d	Sinapic acid	Calibration	13.873	344	112.7960		112.80
100ppb2.d	Sinapic acid	Calibration	13.848	323	106.1399		106.14
100ppb3.d	Sinapic acid	Calibration	13.856	343	112.6130		112.61
250ppb1.d	Sinapic acid	Calibration	13.856	735	235.9321		94.37
250ppb2.d	Sinapic acid	Calibration	13.856	803	257.6513		103.06

Sinapic acid

Data File	Compound	Sample Type	RT	Resp.	Final Conc	Exp. Conc	Accuracy
250ppb3.d	Sinapic acid	Calibration	13.848	796	255.4346		102.17
500ppb1.d	Sinapic acid	Calibration	13.864	1571	499.7134		99.94
500ppb2.d	Sinapic acid	Calibration	13.864	1652	525.1850		105.04
500ppb3.d	Sinapic acid	Calibration	13.864	1657	526.5985		105.32
1000ppb1.d	Sinapic acid	Calibration	13.873	3258	1031.5171		103.15
1000ppb2.d	Sinapic acid	Calibration	13.873	3138	993.7140		99.37
1000ppb3.d	Sinapic acid	Calibration	13.864	2993	947.8402		94.78
22-1A-1.d	Sinapic acid	Sample	13.772	30	13.7365		
22-1A-2.d	Sinapic acid	Sample	13.721	5	5.9375		
22-1A-3.d	Sinapic acid	Sample	13.798	4	5.7472		
22-2A-1.d	Sinapic acid	Sample	13.755	5	6.0942		
22-2A-2.d	Sinapic acid	Sample	13.823	2	5.0930		
22-2A-3.d	Sinapic acid	Sample	13.755	4	5.5230		
22-3A-1.d	Sinapic acid	Sample	13.823	36	15.7593		
22-3A-2.d	Sinapic acid	Sample	13.864	37	15.9980		
22-3A-3.d	Sinapic acid	Sample	13.881	48	19.3768		
22-4A-1.d	Sinapic acid	Sample	13.831	41	17.2465		
22-4A-2.d	Sinapic acid	Sample	13.848	28	13.1343		
22-4A-3.d	Sinapic acid	Sample	13.864	30	13.8809		
22-5A-1.d	Sinapic acid	Sample	13.864	13	8.5612		
22-5A-2.d	Sinapic acid	Sample	13.864	15	8.9641		
22-5A-3.d	Sinapic acid	Sample	13.839	16	9.5635		
22-6A-1.d	Sinapic acid	Sample	13.781	8	6.8039		
22-6A-2.d	Sinapic acid	Sample	13.856	5	5.8321		
22-6A-3.d	Sinapic acid	Sample	13.890	6	6.3064		
22-7A-1.d	Sinapic acid	Sample	13.831	13	8.5687		
22-7A-2.d	Sinapic acid	Sample	13.772	15	8.9731		
22-7A-3.d	Sinapic acid	Sample	13.873	17	9.8777		
22-8A-1.d	Sinapic acid	Sample	13.798	28	13.2430		
22-8A-2.d	Sinapic acid	Sample	13.848	28	13.1782		
22-8A-3.d	Sinapic acid	Sample	13.839	20	10.8029		
22-9A-1.d	Sinapic acid	Sample	13.881	43	18.0291		
22-9A-2.d	Sinapic acid	Sample	13.873	48	19.4139		
22-9A-3.d	Sinapic acid	Sample	13.856	70	26.2887		
22-10A-1.d	Sinapic acid	Sample	13.864	83	30.6591		
22-10A-2.d	Sinapic acid	Sample	13.873	97	35.0527		
22-10A-3.d	Sinapic acid	Sample	13.839	106	37.7254		
22-11A-1.d	Sinapic acid	Sample	13.873	52	20.6223		
22-11A-2.d	Sinapic acid	Sample	13.839	64	24.6130		
22-11A-3.d	Sinapic acid	Sample	13.856	66	25.1573		

Sinapic acid

Data File	Compound	Sample Type	RT	Resp.	Final Conc	Exp. Conc	Accuracy
22-12A-1.d	Sinapic acid	Sample	13.848	96	34.7032		
22-12A-2.d	Sinapic acid	Sample	13.848	90	32.7091		
22-12A-3.d	Sinapic acid	Sample	13.856	120	42.3481		
22-13A-1.d	Sinapic acid	Sample	13.848	1134	361.8530		
22-13A-2.d	Sinapic acid	Sample	13.848	1129	360.1893		
22-13A-3.d	Sinapic acid	Sample	13.856	1159	369.7347		
22-14A-1.d	Sinapic acid	Sample	13.856	758	243.2793		
22-14A-2.d	Sinapic acid	Sample	13.864	767	246.0097		
22-14A-3.d	Sinapic acid	Sample	13.864	796	255.3381		
22-15A-1.d	Sinapic acid	Sample	13.856	21	10.8654		
22-15A-2.d	Sinapic acid	Sample	13.856	37	15.9931		
22-15A-3.d	Sinapic acid	Sample	13.864	32	14.6022		
22-16A-1.d	Sinapic acid	Sample	13.839	99	35.6910		
22-16A-2.d	Sinapic acid	Sample	13.873	133	46.2572		
22-16A-3.d	Sinapic acid	Sample	13.881	103	36.7033		
22-17A-1.d	Sinapic acid	Sample	13.873	54	21.3869		
22-17A-2.d	Sinapic acid	Sample	13.831	35	15.3889		
22-17A-3.d	Sinapic acid	Sample	13.848	53	20.9475		
22-18A-1.d	Sinapic acid	Sample	13.848	41	17.2107		
22-18A-2.d	Sinapic acid	Sample	13.839	31	13.9945		
22-18A-3.d	Sinapic acid	Sample	13.823	32	14.4295		
22-1B-1.d	Sinapic acid	Sample	13.831	6	6.3947		
22-1B-2.d	Sinapic acid	Sample	13.823	24	11.9494		
22-1B-3.d	Sinapic acid	Sample	13.873	3	5.3163		
22-2B-1.d	Sinapic acid	Sample	13.721	25	12.2957		
22-2B-2.d	Sinapic acid	Sample	13.755	3	5.3685		
22-2B-3.d	Sinapic acid	Sample	13.763	10	7.4633		
22-3B-1.d	Sinapic acid	Sample	13.814	55	21.7399		
22-3B-2.d	Sinapic acid	Sample	13.806	52	20.6176		
22-3B-3.d	Sinapic acid	Sample	13.848	59	23.1188		
22-4B-1.d	Sinapic acid	Sample	13.814	35	15.5183		
22-4B-2.d	Sinapic acid	Sample	13.831	49	19.7609		
22-4B-3.d	Sinapic acid	Sample	13.831	48	19.5446		
22-5B-1.d	Sinapic acid	Sample	13.839	14	8.7429		
22-5B-2.d	Sinapic acid	Sample	13.806	16	9.3944		
22-5B-3.d	Sinapic acid	Sample	13.806	23	11.7387		
22-6B-1.d	Sinapic acid	Sample	13.823	17	9.7875		
22-6B-2.d	Sinapic acid	Sample	13.881	4	5.6981		
22-6B-3.d	Sinapic acid	Sample	13.814	10	7.5395		
22-7B-1.d	Sinapic acid	Sample	13.814	37	15.9330		

Sinapic acid

Data File	Compound	Sample Type	RT	Resp.	Final Conc	Exp. Conc	Accuracy
22-7B-2.d	Sinapic acid	Sample	13.823	47	19.1449		
22-7B-3.d	Sinapic acid	Sample	13.823	34	15.2178		
22-8B-1.d	Sinapic acid	Sample	13.831	13	8.4238		
22-8B-2.d	Sinapic acid	Sample	13.831	30	13.7791		
22-8B-3.d	Sinapic acid	Sample	13.839	18	10.0782		
22-9B-1.d	Sinapic acid	Sample	13.823	60	23.1487		
22-9B-2.d	Sinapic acid	Sample	13.823	74	27.7604		
22-9B-3.d	Sinapic acid	Sample	13.839	54	21.4588		
22-10B-1.d	Sinapic acid	Sample	13.823	100	35.9420		
22-10B-2.d	Sinapic acid	Sample	13.839	106	37.7969		
22-10B-3.d	Sinapic acid	Sample	13.823	114	40.2872		
22-11B-1.d	Sinapic acid	Sample	13.831	76	28.4464		
22-11B-2.d	Sinapic acid	Sample	13.814	88	32.2588		
22-11B-3.d	Sinapic acid	Sample	13.831	81	29.8163		
22-12B-1.d	Sinapic acid	Sample	13.839	182	61.7427		
22-12B-2.d	Sinapic acid	Sample	13.839	155	53.1527		
22-12B-3.d	Sinapic acid	Sample	13.839	171	58.3078		
22-13B-1.d	Sinapic acid	Sample	13.831	1668	530.2325		
22-13B-2.d	Sinapic acid	Sample	13.839	1605	510.3504		
22-13B-3.d	Sinapic acid	Sample	13.831	1737	551.9793		
22-14B-1.d	Sinapic acid	Sample	13.831	1102	351.7207		
22-14B-2.d	Sinapic acid	Sample	13.831	1032	329.8440		
22-14B-3.d	Sinapic acid	Sample	13.831	1083	345.7100		
22-15B-1.d	Sinapic acid	Sample	13.814	47	19.1009		
22-15B-2.d	Sinapic acid	Sample	13.823	43	17.8946		
22-15B-3.d	Sinapic acid	Sample	13.831	52	20.7404		
22-16B-1.d	Sinapic acid	Sample	13.848	94	34.0377		
22-16B-2.d	Sinapic acid	Sample	13.864	137	47.4905		
22-16B-3.d	Sinapic acid	Sample	13.831	93	33.5641		
22-17B-1.d	Sinapic acid	Sample	13.848	61	23.5504		
22-17B-2.d	Sinapic acid	Sample	13.823	54	21.4833		
22-17B-3.d	Sinapic acid	Sample	13.831	65	24.8857		
22-18B-1.d	Sinapic acid	Sample	13.823	7	6.4816		
22-18B-2.d	Sinapic acid	Sample	13.915	1	4.6618		
22-18B-3.d	Sinapic acid	Sample	13.873	3	5.3039		
22-1C-1.d	Sinapic acid	Sample	13.823	4	5.5784		
22-1C-2.d	Sinapic acid	Sample	13.873	4	5.7245		
22-1C-3.d	Sinapic acid	Sample	13.864	0	4.4836		
22-2C-1.d	Sinapic acid	Sample	13.688	7	6.4327		
22-2C-2.d	Sinapic acid	Sample	13.831	1	4.5598		

Sinapic acid

Data File	Compound	Sample Type	RT	Resp.	Final Conc	Exp. Conc	Accuracy
22-2C-3.d	Sinapic acid	Sample	13.856	2	4.8785		
22-3C-1.d	Sinapic acid	Sample	13.864	18	10.1392		
22-3C-2.d	Sinapic acid	Sample	13.814	44	18.2659		
22-3C-3.d	Sinapic acid	Sample	13.823	45	18.6041		
22-4C-1.d	Sinapic acid	Sample	13.831	66	25.1816		
22-4C-2.d	Sinapic acid	Sample	13.814	41	17.4245		
22-4C-3.d	Sinapic acid	Sample	13.839	62	23.9045		
22-5C-1.d	Sinapic acid	Sample	13.831	12	8.0609		
22-5C-2.d	Sinapic acid	Sample	13.814	19	10.2785		
22-5C-3.d	Sinapic acid	Sample	13.814	18	9.9990		
22-6C-1.d	Sinapic acid	Sample	13.873	2	5.0356		
22-6C-2.d	Sinapic acid	Sample	13.839	9	7.0458		
22-6C-3.d	Sinapic acid	Sample	13.848	5	5.8194		
22-7C-1.d	Sinapic acid	Sample	13.798	25	12.1764		
22-7C-2.d	Sinapic acid	Sample	13.848	48	19.5123		
22-7C-3.d	Sinapic acid	Sample	13.806	31	14.0507		
22-8C-1.d	Sinapic acid	Sample	13.856	39	16.7316		
22-8C-2.d	Sinapic acid	Sample	13.856	48	19.4536		
22-8C-3.d	Sinapic acid	Sample	13.806	22	11.1503		
22-9C-1.d	Sinapic acid	Sample	13.814	58	22.6932		
22-9C-2.d	Sinapic acid	Sample	13.823	50	20.2581		
22-9C-3.d	Sinapic acid	Sample	13.839	56	22.1364		
22-10C-1.d	Sinapic acid	Sample	13.839	132	46.0793		
22-10C-2.d	Sinapic acid	Sample	13.831	128	44.8423		
22-10C-3.d	Sinapic acid	Sample	13.839	135	46.7960		
22-11C-1.d	Sinapic acid	Sample	13.839	82	30.0591		
22-11C-2.d	Sinapic acid	Sample	13.831	82	30.1731		
22-11C-3.d	Sinapic acid	Sample	13.898	100	35.9495		
22-12C-1.d	Sinapic acid	Sample	13.831	120	42.0561		
22-12C-2.d	Sinapic acid	Sample	13.814	138	47.8991		
22-12C-3.d	Sinapic acid	Sample	13.848	134	46.6613		
22-13C-1.d	Sinapic acid	Sample	13.831	1563	497.0656		
22-13C-2.d	Sinapic acid	Sample	13.831	1654	525.9228		
22-13C-3.d	Sinapic acid	Sample	13.839	1569	499.0699		
22-14C-1.d	Sinapic acid	Sample	13.831	931	297.8887		
22-14C-2.d	Sinapic acid	Sample	13.831	834	267.2850		
22-14C-3.d	Sinapic acid	Sample	13.831	863	276.5254		
22-15C-1.d	Sinapic acid	Sample	13.831	26	12.6472		
22-15C-2.d	Sinapic acid	Sample	13.823	33	14.8436		
22-15C-3.d	Sinapic acid	Sample	13.831	38	16.4048		

Sinapic acid

Data File	Compound	Sample Type	RT	Resp.	Final Conc	Exp. Conc	Accuracy
22-16C-1.d	Sinapic acid	Sample	13.856	83	30.6062		
22-16C-2.d	Sinapic acid	Sample	13.848	70	26.3871		
22-16C-3.d	Sinapic acid	Sample	13.839	96	34.6131		
22-17C-1.d	Sinapic acid	Sample	13.839	70	26.3338		
22-17C-2.d	Sinapic acid	Sample	13.856	59	22.9503		
22-17C-3.d	Sinapic acid	Sample	13.856	57	22.2391		
22-18C-1.d	Sinapic acid	Sample	13.839	84	30.8676		
22-18C-2.d	Sinapic acid	Sample	13.831	80	29.7214		
22-18C-3.d	Sinapic acid	Sample	13.831	78	28.9252		

Ferulic acid

Data File	Compound	Sample Type	RT	Resp.	Final Conc	Exp. Conc	Accuracy
blank-061022-4.d	Ferulic acid	Sample	13.950	0	0.2546		
25ppb1.d	Ferulic acid	Calibration	13.891	2	0.5666		2.27
25ppb2.d	Ferulic acid	Calibration	13.950	3	0.7802		3.12
25ppb3.d	Ferulic acid	Calibration	13.891	9	2.0503		8.20
50ppb1.d	Ferulic acid	Calibration	13.891	222	42.7595		85.52
50ppb2.d	Ferulic acid	Calibration	13.908	244	47.0246		94.05
50ppb3.d	Ferulic acid	Calibration	13.916	226	43.7032		87.41
100ppb1.d	Ferulic acid	Calibration	13.916	617	118.6712		118.67
100ppb2.d	Ferulic acid	Calibration	13.925	648	124.5307		124.53
100ppb3.d	Ferulic acid	Calibration	13.925	637	122.4015		122.40
250ppb1.d	Ferulic acid	Calibration	13.934	1264	242.7344		97.09
250ppb2.d	Ferulic acid	Calibration	13.916	1327	254.9409		101.98
250ppb3.d	Ferulic acid	Calibration	13.925	1281	245.9975		98.40
500ppb1.d	Ferulic acid	Calibration	13.934	2647	508.0690		101.61
500ppb2.d	Ferulic acid	Calibration	13.934	2724	522.9891		104.60
500ppb3.d	Ferulic acid	Calibration	13.934	2768	531.3465		106.27
1000ppb1.d	Ferulic acid	Calibration	13.934	5358	1028.3674		102.84
1000ppb2.d	Ferulic acid	Calibration	13.934	5066	972.2530		97.23
1000ppb3.d	Ferulic acid	Calibration	13.925	5032	965.8144		96.58
22-1A-1.d	Ferulic acid	Sample	13.942	725	139.4097		
22-1A-2.d	Ferulic acid	Sample	13.950	674	129.5684		
22-1A-3.d	Ferulic acid	Sample	13.950	664	127.6188		
22-2A-1.d	Ferulic acid	Sample	13.934	623	119.8329		
22-2A-2.d	Ferulic acid	Sample	13.942	647	124.3604		
22-2A-3.d	Ferulic acid	Sample	13.934	671	129.0229		
22-3A-1.d	Ferulic acid	Sample	13.934	576	110.7939		
22-3A-2.d	Ferulic acid	Sample	13.942	544	104.5737		
22-3A-3.d	Ferulic acid	Sample	13.925	555	106.8018		
22-4A-1.d	Ferulic acid	Sample	13.934	534	102.7066		

Ferulic acid

Data File	Compound	Sample Type	RT	Resp.	Final Conc	Exp. Conc	Accuracy
22-4A-2.d	Ferulic acid	Sample	13.925	579	111.3506		
22-4A-3.d	Ferulic acid	Sample	13.934	551	105.9467		
22-5A-1.d	Ferulic acid	Sample	13.925	296	57.1304		
22-5A-2.d	Ferulic acid	Sample	13.925	299	57.5596		
22-5A-3.d	Ferulic acid	Sample	13.925	299	57.5617		
22-6A-1.d	Ferulic acid	Sample	13.934	22295	4278.3654		
22-6A-2.d	Ferulic acid	Sample	13.925	22288	4277.0242		
22-6A-3.d	Ferulic acid	Sample	13.934	22355	4289.7434		
22-7A-1.d	Ferulic acid	Sample	13.925	790	151.8853		
22-7A-2.d	Ferulic acid	Sample	13.934	830	159.6055		
22-7A-3.d	Ferulic acid	Sample	13.934	757	145.5974		
22-8A-1.d	Ferulic acid	Sample	13.934	1423	273.3365		
22-8A-2.d	Ferulic acid	Sample	13.925	1251	240.3604		
22-8A-3.d	Ferulic acid	Sample	13.925	1362	261.5464		
22-9A-1.d	Ferulic acid	Sample	13.925	430	82.8530		
22-9A-2.d	Ferulic acid	Sample	13.925	449	86.4606		
22-9A-3.d	Ferulic acid	Sample	13.925	409	78.7580		
22-10A-1.d	Ferulic acid	Sample	13.925	1949	374.1769		
22-10A-2.d	Ferulic acid	Sample	13.925	2081	399.4829		
22-10A-3.d	Ferulic acid	Sample	13.916	1996	383.2723		
22-11A-1.d	Ferulic acid	Sample	13.925	582	111.8643		
22-11A-2.d	Ferulic acid	Sample	13.934	526	101.1953		
22-11A-3.d	Ferulic acid	Sample	13.925	517	99.5262		
22-12A-1.d	Ferulic acid	Sample	13.916	462	88.8051		
22-12A-2.d	Ferulic acid	Sample	13.916	500	96.2066		
22-12A-3.d	Ferulic acid	Sample	13.925	451	86.7507		
22-13A-1.d	Ferulic acid	Sample	13.916	6537	1254.5328		
22-13A-2.d	Ferulic acid	Sample	13.916	6590	1264.8382		
22-13A-3.d	Ferulic acid	Sample	13.925	6574	1261.6492		
22-14A-1.d	Ferulic acid	Sample	13.925	2317	444.9077		
22-14A-2.d	Ferulic acid	Sample	13.925	2251	432.0993		
22-14A-3.d	Ferulic acid	Sample	13.925	2286	438.9602		
22-15A-1.d	Ferulic acid	Sample	13.934	252	48.5086		
22-15A-2.d	Ferulic acid	Sample	13.925	259	49.9390		
22-15A-3.d	Ferulic acid	Sample	13.925	257	49.5839		
22-16A-1.d	Ferulic acid	Sample	13.925	682	131.1355		
22-16A-2.d	Ferulic acid	Sample	13.925	676	130.0381		
22-16A-3.d	Ferulic acid	Sample	13.942	742	142.7011		
22-17A-1.d	Ferulic acid	Sample	13.925	250	48.2306		
22-17A-2.d	Ferulic acid	Sample	13.916	244	47.0204		

Agilent Technologies

Ferulic acid

Data File	Compound	Sample Type	RT	Resp.	Final Conc	Exp. Conc	Accuracy
22-17A-3.d	Ferulic acid	Sample	13.908	306	58.9838		
22-18A-1.d	Ferulic acid	Sample	13.899	2047	393.0741		
22-18A-2.d	Ferulic acid	Sample	13.899	1904	365.5334		
22-18A-3.d	Ferulic acid	Sample	13.899	2099	403.0358		
22-1B-1.d	Ferulic acid	Sample	13.908	720	138.3915		
22-1B-2.d	Ferulic acid	Sample	13.908	719	138.2841		
22-1B-3.d	Ferulic acid	Sample	13.908	801	153.8831		
22-2B-1.d	Ferulic acid	Sample	13.908	793	152.4983		
22-2B-2.d	Ferulic acid	Sample	13.899	842	161.8375		
22-2B-3.d	Ferulic acid	Sample	13.899	843	162.0540		
22-3B-1.d	Ferulic acid	Sample	13.891	780	149.8404		
22-3B-2.d	Ferulic acid	Sample	13.899	773	148.6389		
22-3B-3.d	Ferulic acid	Sample	13.899	796	152.9287		
22-4B-1.d	Ferulic acid	Sample	13.899	689	132.3676		
22-4B-2.d	Ferulic acid	Sample	13.899	824	158.4194		
22-4B-3.d	Ferulic acid	Sample	13.899	793	152.3464		
22-5B-1.d	Ferulic acid	Sample	13.891	397	76.4182		
22-5B-2.d	Ferulic acid	Sample	13.891	380	73.1606		
22-5B-3.d	Ferulic acid	Sample	13.891	386	74.2950		
22-6B-1.d	Ferulic acid	Sample	13.899	30331	5820.2947		
22-6B-2.d	Ferulic acid	Sample	13.899	30873	5924.3310		
22-6B-3.d	Ferulic acid	Sample	13.899	30806	5911.3155		
22-7B-1.d	Ferulic acid	Sample	13.899	891	171.1822		
22-7B-2.d	Ferulic acid	Sample	13.908	995	191.1030		
22-7B-3.d	Ferulic acid	Sample	13.883	1009	193.8274		
22-8B-1.d	Ferulic acid	Sample	13.908	1532	294.1275		
22-8B-2.d	Ferulic acid	Sample	13.899	1515	290.9258		
22-8B-3.d	Ferulic acid	Sample	13.899	1569	301.2845		
22-9B-1.d	Ferulic acid	Sample	13.891	591	113.5585		
22-9B-2.d	Ferulic acid	Sample	13.908	518	99.7010		
22-9B-3.d	Ferulic acid	Sample	13.899	687	132.1640		
22-10B-1.d	Ferulic acid	Sample	13.899	2392	459.3002		
22-10B-2.d	Ferulic acid	Sample	13.891	2360	453.0172		
22-10B-3.d	Ferulic acid	Sample	13.899	2307	442.9583		
22-11B-1.d	Ferulic acid	Sample	13.891	866	166.3994		
22-11B-2.d	Ferulic acid	Sample	13.891	772	148.3046		
22-11B-3.d	Ferulic acid	Sample	13.899	780	149.9915		
22-12B-1.d	Ferulic acid	Sample	13.891	679	130.5144		
22-12B-2.d	Ferulic acid	Sample	13.899	683	131.3513		
22-12B-3.d	Ferulic acid	Sample	13.899	632	121.5614		

Ferulic acid

Data File	Compound	Sample Type	RT	Resp.	Final Conc	Exp. Conc	Accuracy
22-13B-1.d	Ferulic acid	Sample	13.899	8427	1617.1672		
22-13B-2.d	Ferulic acid	Sample	13.891	7879	1512.0579		
22-13B-3.d	Ferulic acid	Sample	13.899	8941	1715.8572		
22-14B-1.d	Ferulic acid	Sample	13.899	3020	579.6533		
22-14B-2.d	Ferulic acid	Sample	13.899	2952	566.7054		
22-14B-3.d	Ferulic acid	Sample	13.891	2940	564.3382		
22-15B-1.d	Ferulic acid	Sample	13.908	221	42.5636		
22-15B-2.d	Ferulic acid	Sample	13.899	260	50.1531		
22-15B-3.d	Ferulic acid	Sample	13.899	220	42.3990		
22-16B-1.d	Ferulic acid	Sample	13.899	730	140.3987		
22-16B-2.d	Ferulic acid	Sample	13.899	719	138.3063		
22-16B-3.d	Ferulic acid	Sample	13.891	690	132.5963		
22-17B-1.d	Ferulic acid	Sample	13.899	328	63.2616		
22-17B-2.d	Ferulic acid	Sample	13.883	390	75.0661		
22-17B-3.d	Ferulic acid	Sample	13.908	386	74.2355		
22-18B-1.d	Ferulic acid	Sample	13.883	421	81.1154		
22-18B-2.d	Ferulic acid	Sample	13.899	465	89.4641		
22-18B-3.d	Ferulic acid	Sample	13.899	403	77.6472		
22-1C-1.d	Ferulic acid	Sample	13.899	849	163.1487		
22-1C-2.d	Ferulic acid	Sample	13.899	940	180.6165		
22-1C-3.d	Ferulic acid	Sample	13.899	824	158.4392		
22-2C-1.d	Ferulic acid	Sample	13.899	684	131.5738		
22-2C-2.d	Ferulic acid	Sample	13.899	647	124.4703		
22-2C-3.d	Ferulic acid	Sample	13.899	704	135.2807		
22-3C-1.d	Ferulic acid	Sample	13.891	642	123.3457		
22-3C-2.d	Ferulic acid	Sample	13.891	581	111.7549		
22-3C-3.d	Ferulic acid	Sample	13.883	628	120.7209		
22-4C-1.d	Ferulic acid	Sample	13.891	622	119.5556		
22-4C-2.d	Ferulic acid	Sample	13.891	632	121.4579		
22-4C-3.d	Ferulic acid	Sample	13.883	695	133.5146		
22-5C-1.d	Ferulic acid	Sample	13.883	300	57.7415		
22-5C-2.d	Ferulic acid	Sample	13.891	315	60.6468		
22-5C-3.d	Ferulic acid	Sample	13.883	279	53.8648		
22-6C-1.d	Ferulic acid	Sample	13.891	28899	5545.5358		
22-6C-2.d	Ferulic acid	Sample	13.891	29471	5655.2483		
22-6C-3.d	Ferulic acid	Sample	13.891	29910	5739.4801		
22-7C-1.d	Ferulic acid	Sample	13.891	740	142.3367		
22-7C-2.d	Ferulic acid	Sample	13.891	902	173.2442		
22-7C-3.d	Ferulic acid	Sample	13.891	808	155.3661		
22-8C-1.d	Ferulic acid	Sample	13.899	1531	294.0792		

Ferulic acid

Data File	Compound	Sample Type	RT	Resp.	Final Conc	Exp. Conc	Accuracy
22-8C-2.d	Ferulic acid	Sample	13.899	1353	259.9172		
22-8C-3.d	Ferulic acid	Sample	13.899	1383	265.5945		
22-9C-1.d	Ferulic acid	Sample	13.891	523	100.5098		
22-9C-2.d	Ferulic acid	Sample	13.891	548	105.3422		
22-9C-3.d	Ferulic acid	Sample	13.908	638	122.6570		
22-10C-1.d	Ferulic acid	Sample	13.891	2470	474.2175		
22-10C-2.d	Ferulic acid	Sample	13.899	2531	485.9754		
22-10C-3.d	Ferulic acid	Sample	13.899	2530	485.7146		
22-11C-1.d	Ferulic acid	Sample	13.908	793	152.4717		
22-11C-2.d	Ferulic acid	Sample	13.908	835	160.4931		
22-11C-3.d	Ferulic acid	Sample	13.899	833	160.1252		
22-12C-1.d	Ferulic acid	Sample	13.891	577	110.9811		
22-12C-2.d	Ferulic acid	Sample	13.899	555	106.6858		
22-12C-3.d	Ferulic acid	Sample	13.908	586	112.6525		
22-13C-1.d	Ferulic acid	Sample	13.908	8275	1588.0455		
22-13C-2.d	Ferulic acid	Sample	13.899	8801	1689.0776		
22-13C-3.d	Ferulic acid	Sample	13.899	8373	1606.8392		
22-14C-1.d	Ferulic acid	Sample	13.908	2591	497.4279		
22-14C-2.d	Ferulic acid	Sample	13.899	2543	488.1515		
22-14C-3.d	Ferulic acid	Sample	13.899	2730	524.0993		
22-15C-1.d	Ferulic acid	Sample	13.899	229	44.2342		
22-15C-2.d	Ferulic acid	Sample	13.899	213	41.1756		
22-15C-3.d	Ferulic acid	Sample	13.908	245	47.2855		
22-16C-1.d	Ferulic acid	Sample	13.899	607	116.6552		
22-16C-2.d	Ferulic acid	Sample	13.916	648	124.6460		
22-16C-3.d	Ferulic acid	Sample	13.908	559	107.5007		
22-17C-1.d	Ferulic acid	Sample	13.899	263	50.8060		
22-17C-2.d	Ferulic acid	Sample	13.916	358	69.0076		
22-17C-3.d	Ferulic acid	Sample	13.908	285	54.9550		
22-18C-1.d	Ferulic acid	Sample	13.908	410	78.8959		
22-18C-2.d	Ferulic acid	Sample	13.899	450	86.5807		
22-18C-3.d	Ferulic acid	Sample	13.899	417	80.3336		

Luteolin 7-glucoside

Data File	Compound	Sample Type	RT	Resp.	Final Conc	Exp. Conc	Accuracy
blank-061022-4.d	Luteolin 7-glucoside	Sample	14.355	145	ND		
25ppb1.d	Luteolin 7-glucoside	Calibration	14.531	92	ND		0.00
25ppb2.d	Luteolin 7-glucoside	Calibration	14.038	47	ND		0.00
25ppb3.d	Luteolin 7-glucoside	Calibration	14.104	4730	18.3184		73.27
50ppb1.d	Luteolin 7-glucoside	Calibration	14.222	7068	44.0082		88.02
50ppb2.d	Luteolin 7-glucoside	Calibration	14.239	5992	32.1840		64.37

Luteolin 7-glucoside

Data File	Compound	Sample Type	RT	Resp.	Final Conc	Exp. Conc	Accuracy
50ppb3.d	Luteolin 7-glucoside	Calibration	14.247	5445	26.1764		52.35
100ppb1.d	Luteolin 7-glucoside	Calibration	14.255	12136	99.6874		99.69
100ppb2.d	Luteolin 7-glucoside	Calibration	14.255	11834	96.3644		96.36
100ppb3.d	Luteolin 7-glucoside	Calibration	14.255	12280	101.2657		101.27
250ppb1.d	Luteolin 7-glucoside	Calibration	14.264	25423	245.6612		98.26
250ppb2.d	Luteolin 7-glucoside	Calibration	14.264	26577	258.3454		103.34
250ppb3.d	Luteolin 7-glucoside	Calibration	14.264	26253	254.7851		101.91
500ppb1.d	Luteolin 7-glucoside	Calibration	14.264	51277	529.7120		105.94
500ppb2.d	Luteolin 7-glucoside	Calibration	14.272	51110	527.8735		105.57
500ppb3.d	Luteolin 7-glucoside	Calibration	14.272	51994	537.5842		107.52
1000ppb1.d	Luteolin 7-glucoside	Calibration	14.272	94912	1009.1075		100.91
1000ppb2.d	Luteolin 7-glucoside	Calibration	14.272	89837	953.3428		95.33
1000ppb3.d	Luteolin 7-glucoside	Calibration	14.264	93226	990.5837		99.06
22-1A-1.d	Luteolin 7-glucoside	Sample	14.272	28603	280.6030		
22-1A-2.d	Luteolin 7-glucoside	Sample	14.272	28476	279.2081		
22-1A-3.d	Luteolin 7-glucoside	Sample	14.264	28204	276.2142		
22-2A-1.d	Luteolin 7-glucoside	Sample	14.272	132437	1421.3701		
22-2A-2.d	Luteolin 7-glucoside	Sample	14.272	133578	1433.9112		
22-2A-3.d	Luteolin 7-glucoside	Sample	14.272	134147	1440.1575		
22-3A-1.d	Luteolin 7-glucoside	Sample	14.264	94474	1004.2970		
22-3A-2.d	Luteolin 7-glucoside	Sample	14.272	94404	1003.5177		
22-3A-3.d	Luteolin 7-glucoside	Sample	14.272	93986	998.9302		
22-4A-1.d	Luteolin 7-glucoside	Sample	14.264	186202	2012.0566		
22-4A-2.d	Luteolin 7-glucoside	Sample	14.264	187556	2026.9417		
22-4A-3.d	Luteolin 7-glucoside	Sample	14.264	186383	2014.0560		
22-5A-1.d	Luteolin 7-glucoside	Sample	14.255	117999	1262.7450		
22-5A-2.d	Luteolin 7-glucoside	Sample	14.255	118688	1270.3220		
22-5A-3.d	Luteolin 7-glucoside	Sample	14.255	116560	1246.9375		
22-6A-1.d	Luteolin 7-glucoside	Sample	14.264	29312	288.3932		
22-6A-2.d	Luteolin 7-glucoside	Sample	14.264	29747	293.1650		
22-6A-3.d	Luteolin 7-glucoside	Sample	14.264	28900	283.8613		
22-7A-1.d	Luteolin 7-glucoside	Sample	14.264	1160	ND		
22-7A-2.d	Luteolin 7-glucoside	Sample	14.255	1152	ND		
22-7A-3.d	Luteolin 7-glucoside	Sample	14.264	1152	ND		
22-8A-1.d	Luteolin 7-glucoside	Sample	14.264	8865	63.7488		
22-8A-2.d	Luteolin 7-glucoside	Sample	14.264	8595	60.7788		
22-8A-3.d	Luteolin 7-glucoside	Sample	14.264	8929	64.4459		
22-9A-1.d	Luteolin 7-glucoside	Sample	14.264	16697	149.7894		
22-9A-2.d	Luteolin 7-glucoside	Sample	14.264	16693	149.7548		
22-9A-3.d	Luteolin 7-glucoside	Sample	14.264	16981	152.9174		

Luteolin 7-glucoside

Data File	Compound	Sample Type	RT	Resp.	Final Conc	Exp. Conc	Accuracy
22-10A-1.d	Luteolin 7-glucoside	Sample	14.264	2976	ND		
22-10A-2.d	Luteolin 7-glucoside	Sample	14.255	2520	ND		
22-10A-3.d	Luteolin 7-glucoside	Sample	14.264	2891	ND		
22-11A-1.d	Luteolin 7-glucoside	Sample	14.264	2281	ND		
22-11A-2.d	Luteolin 7-glucoside	Sample	14.272	2085	ND		
22-11A-3.d	Luteolin 7-glucoside	Sample	14.264	2531	ND		
22-12A-1.d	Luteolin 7-glucoside	Sample	14.264	640682	7005.2060		
22-12A-2.d	Luteolin 7-glucoside	Sample	14.264	648163	7087.4007		
22-12A-3.d	Luteolin 7-glucoside	Sample	14.264	647702	7082.3330		
22-13A-1.d	Luteolin 7-glucoside	Sample	14.264	46685	479.2592		
22-13A-2.d	Luteolin 7-glucoside	Sample	14.264	42064	428.4895		
22-13A-3.d	Luteolin 7-glucoside	Sample	14.264	39887	404.5669		
22-14A-1.d	Luteolin 7-glucoside	Sample	14.264	805587	8816.9382		
22-14A-2.d	Luteolin 7-glucoside	Sample	14.264	807545	8838.4449		
22-14A-3.d	Luteolin 7-glucoside	Sample	14.264	803365	8792.5216		
22-15A-1.d	Luteolin 7-glucoside	Sample	14.264	844830	9248.0826		
22-15A-2.d	Luteolin 7-glucoside	Sample	14.264	841347	9209.8113		
22-15A-3.d	Luteolin 7-glucoside	Sample	14.264	847693	9279.5317		
22-16A-1.d	Luteolin 7-glucoside	Sample	14.264	89894	953.9711		
22-16A-2.d	Luteolin 7-glucoside	Sample	14.264	78639	830.3201		
22-16A-3.d	Luteolin 7-glucoside	Sample	14.264	76304	804.6706		
22-17A-1.d	Luteolin 7-glucoside	Sample	14.264	429706	4687.3207		
22-17A-2.d	Luteolin 7-glucoside	Sample	14.255	444762	4852.7338		
22-17A-3.d	Luteolin 7-glucoside	Sample	14.255	442212	4824.7163		
22-18A-1.d	Luteolin 7-glucoside	Sample	14.255	6530	38.0899		
22-18A-2.d	Luteolin 7-glucoside	Sample	14.239	4375	14.4205		
22-18A-3.d	Luteolin 7-glucoside	Sample	14.255	4124	11.6633		
22-1B-1.d	Luteolin 7-glucoside	Sample	14.239	31930	317.1479		
22-1B-2.d	Luteolin 7-glucoside	Sample	14.239	30907	305.9170		
22-1B-3.d	Luteolin 7-glucoside	Sample	14.239	31939	317.2560		
22-2B-1.d	Luteolin 7-glucoside	Sample	14.247	146475	1575.6048		
22-2B-2.d	Luteolin 7-glucoside	Sample	14.239	146935	1580.6568		
22-2B-3.d	Luteolin 7-glucoside	Sample	14.239	146730	1578.4024		
22-3B-1.d	Luteolin 7-glucoside	Sample	14.239	122623	1313.5538		
22-3B-2.d	Luteolin 7-glucoside	Sample	14.239	119284	1276.8628		
22-3B-3.d	Luteolin 7-glucoside	Sample	14.239	121623	1302.5615		
22-4B-1.d	Luteolin 7-glucoside	Sample	14.239	197173	2132.5991		
22-4B-2.d	Luteolin 7-glucoside	Sample	14.247	195294	2111.9492		
22-4B-3.d	Luteolin 7-glucoside	Sample	14.247	197683	2138.2016		
22-5B-1.d	Luteolin 7-glucoside	Sample	14.222	116145	1242.3851		

Luteolin 7-glucoside

Data File	Compound	Sample Type	RT	Resp.	Final Conc	Exp. Conc	Accuracy
22-5B-2.d	Luteolin 7-glucoside	Sample	14.213	117329	1255.3922		
22-5B-3.d	Luteolin 7-glucoside	Sample	14.222	113469	1212.9793		
22-6B-1.d	Luteolin 7-glucoside	Sample	14.239	27750	271.2246		
22-6B-2.d	Luteolin 7-glucoside	Sample	14.239	28156	275.6938		
22-6B-3.d	Luteolin 7-glucoside	Sample	14.239	26215	254.3633		
22-7B-1.d	Luteolin 7-glucoside	Sample	14.247	1500	ND		
22-7B-2.d	Luteolin 7-glucoside	Sample	14.239	1412	ND		
22-7B-3.d	Luteolin 7-glucoside	Sample	14.239	1514	ND		
22-8B-1.d	Luteolin 7-glucoside	Sample	14.247	7784	51.8720		
22-8B-2.d	Luteolin 7-glucoside	Sample	14.239	8168	56.0921		
22-8B-3.d	Luteolin 7-glucoside	Sample	14.239	7934	53.5200		
22-9B-1.d	Luteolin 7-glucoside	Sample	14.247	18483	169.4208		
22-9B-2.d	Luteolin 7-glucoside	Sample	14.247	17367	157.1590		
22-9B-3.d	Luteolin 7-glucoside	Sample	14.247	17995	164.0501		
22-10B-1.d	Luteolin 7-glucoside	Sample	14.230	2382	ND		
22-10B-2.d	Luteolin 7-glucoside	Sample	14.230	2554	ND		
22-10B-3.d	Luteolin 7-glucoside	Sample	14.239	2336	ND		
22-11B-1.d	Luteolin 7-glucoside	Sample	14.247	2638	ND		
22-11B-2.d	Luteolin 7-glucoside	Sample	14.247	1736	ND		
22-11B-3.d	Luteolin 7-glucoside	Sample	14.247	1818	ND		
22-12B-1.d	Luteolin 7-glucoside	Sample	14.247	685619	7498.9146		
22-12B-2.d	Luteolin 7-glucoside	Sample	14.239	677020	7404.4409		
22-12B-3.d	Luteolin 7-glucoside	Sample	14.247	678738	7423.3090		
22-13B-1.d	Luteolin 7-glucoside	Sample	14.247	52161	539.4156		
22-13B-2.d	Luteolin 7-glucoside	Sample	14.247	48987	504.5536		
22-13B-3.d	Luteolin 7-glucoside	Sample	14.247	46854	481.1091		
22-14B-1.d	Luteolin 7-glucoside	Sample	14.247	906579	9926.4851		
22-14B-2.d	Luteolin 7-glucoside	Sample	14.239	880305	9637.8263		
22-14B-3.d	Luteolin 7-glucoside	Sample	14.239	903149	9888.8067		
22-15B-1.d	Luteolin 7-glucoside	Sample	14.247	799450	8749.5163		
22-15B-2.d	Luteolin 7-glucoside	Sample	14.247	792794	8676.3882		
22-15B-3.d	Luteolin 7-glucoside	Sample	14.247	808190	8845.5367		
22-16B-1.d	Luteolin 7-glucoside	Sample	14.247	99321	1057.5418		
22-16B-2.d	Luteolin 7-glucoside	Sample	14.247	87763	930.5572		
22-16B-3.d	Luteolin 7-glucoside	Sample	14.247	87483	927.4886		
22-17B-1.d	Luteolin 7-glucoside	Sample	14.239	472992	5162.8859		
22-17B-2.d	Luteolin 7-glucoside	Sample	14.239	478739	5226.0235		
22-17B-3.d	Luteolin 7-glucoside	Sample	14.239	477379	5211.0778		
22-18B-1.d	Luteolin 7-glucoside	Sample	14.239	6603	38.8912		
22-18B-2.d	Luteolin 7-glucoside	Sample	14.230	4386	14.5399		

Agilent Technologies

Luteolin 7-glucoside

Data File	Compound	Sample Type	RT	Resp.	Final Conc	Exp. Conc	Accuracy
22-18B-3.d	Luteolin 7-glucoside	Sample	14.239	4154	11.9893		
22-1C-1.d	Luteolin 7-glucoside	Sample	14.230	29968	295.5925		
22-1C-2.d	Luteolin 7-glucoside	Sample	14.239	29851	294.3062		
22-1C-3.d	Luteolin 7-glucoside	Sample	14.239	30135	297.4319		
22-2C-1.d	Luteolin 7-glucoside	Sample	14.239	127543	1367.5994		
22-2C-2.d	Luteolin 7-glucoside	Sample	14.239	128049	1373.1675		
22-2C-3.d	Luteolin 7-glucoside	Sample	14.239	127346	1365.4372		
22-3C-1.d	Luteolin 7-glucoside	Sample	14.239	107732	1149.9457		
22-3C-2.d	Luteolin 7-glucoside	Sample	14.239	106268	1133.8620		
22-3C-3.d	Luteolin 7-glucoside	Sample	14.239	110173	1176.7659		
22-4C-1.d	Luteolin 7-glucoside	Sample	14.239	185093	1999.8806		
22-4C-2.d	Luteolin 7-glucoside	Sample	14.239	193611	2093.4597		
22-4C-3.d	Luteolin 7-glucoside	Sample	14.239	187732	2028.8676		
22-5C-1.d	Luteolin 7-glucoside	Sample	14.230	121787	1304.3715		
22-5C-2.d	Luteolin 7-glucoside	Sample	14.230	121117	1297.0062		
22-5C-3.d	Luteolin 7-glucoside	Sample	14.230	122478	1311.9605		
22-6C-1.d	Luteolin 7-glucoside	Sample	14.239	27127	264.3825		
22-6C-2.d	Luteolin 7-glucoside	Sample	14.239	25989	251.8778		
22-6C-3.d	Luteolin 7-glucoside	Sample	14.230	26882	261.6936		
22-7C-1.d	Luteolin 7-glucoside	Sample	14.239	1252	ND		
22-7C-2.d	Luteolin 7-glucoside	Sample	14.239	1208	ND		
22-7C-3.d	Luteolin 7-glucoside	Sample	14.230	1147	ND		
22-8C-1.d	Luteolin 7-glucoside	Sample	14.239	9120	66.5499		
22-8C-2.d	Luteolin 7-glucoside	Sample	14.239	9161	66.9975		
22-8C-3.d	Luteolin 7-glucoside	Sample	14.239	8891	64.0355		
22-9C-1.d	Luteolin 7-glucoside	Sample	14.247	19133	176.5623		
22-9C-2.d	Luteolin 7-glucoside	Sample	14.247	18742	172.2663		
22-9C-3.d	Luteolin 7-glucoside	Sample	14.247	18686	171.6414		
22-10C-1.d	Luteolin 7-glucoside	Sample	14.247	2506	ND		
22-10C-2.d	Luteolin 7-glucoside	Sample	14.239	2391	ND		
22-10C-3.d	Luteolin 7-glucoside	Sample	14.239	2428	ND		
22-11C-1.d	Luteolin 7-glucoside	Sample	14.247	3396	3.6660		
22-11C-2.d	Luteolin 7-glucoside	Sample	14.247	4150	11.9492		
22-11C-3.d	Luteolin 7-glucoside	Sample	14.247	3451	4.2690		
22-12C-1.d	Luteolin 7-glucoside	Sample	14.247	600109	6559.4548		
22-12C-2.d	Luteolin 7-glucoside	Sample	14.247	599021	6547.5023		
22-12C-3.d	Luteolin 7-glucoside	Sample	14.247	617106	6746.1873		
22-13C-1.d	Luteolin 7-glucoside	Sample	14.255	42883	437.4903		
22-13C-2.d	Luteolin 7-glucoside	Sample	14.255	37566	379.0767		
22-13C-3.d	Luteolin 7-glucoside	Sample	14.255	35195	353.0254		

Luteolin 7-glucoside

Data File	Compound	Sample Type	RT	Resp.	Final Conc	Exp. Conc	Accuracy
22-14C-1.d	Luteolin 7-glucoside	Sample	14.247	794834	8698.7983		
22-14C-2.d	Luteolin 7-glucoside	Sample	14.247	773618	8465.7141		
22-14C-3.d	Luteolin 7-glucoside	Sample	14.247	811013	8876.5467		
22-15C-1.d	Luteolin 7-glucoside	Sample	14.255	748291	8187.4585		
22-15C-2.d	Luteolin 7-glucoside	Sample	14.247	768189	8406.0607		
22-15C-3.d	Luteolin 7-glucoside	Sample	14.255	763750	8357.2973		
22-16C-1.d	Luteolin 7-glucoside	Sample	14.247	77502	817.8263		
22-16C-2.d	Luteolin 7-glucoside	Sample	14.255	75310	793.7415		
22-16C-3.d	Luteolin 7-glucoside	Sample	14.255	76327	804.9190		
22-17C-1.d	Luteolin 7-glucoside	Sample	14.255	399168	4351.8142		
22-17C-2.d	Luteolin 7-glucoside	Sample	14.255	404688	4412.4616		
22-17C-3.d	Luteolin 7-glucoside	Sample	14.255	404995	4415.8321		
22-18C-1.d	Luteolin 7-glucoside	Sample	14.247	3998	10.2779		
22-18C-2.d	Luteolin 7-glucoside	Sample	14.247	4193	12.4244		
22-18C-3.d	Luteolin 7-glucoside	Sample	14.247	3878	8.9565		

Hesperidin

Data File	Compound	Sample Type	RT	Resp.	Final Conc	Exp. Conc	Accuracy
blank-061022-4.d	Hesperidin	Sample	14.319	0	4.2147		
25ppb1.d	Hesperidin	Calibration	14.419	4	4.7150		18.86
25ppb2.d	Hesperidin	Calibration	14.319	2	4.3764		17.51
25ppb3.d	Hesperidin	Calibration	14.202	99	16.1794		64.72
50ppb1.d	Hesperidin	Calibration	14.286	308	41.6171		83.23
50ppb2.d	Hesperidin	Calibration	14.302	328	43.9704		87.94
50ppb3.d	Hesperidin	Calibration	14.302	391	51.5761		103.15
100ppb1.d	Hesperidin	Calibration	14.302	823	104.0248		104.02
100ppb2.d	Hesperidin	Calibration	14.302	877	110.6730		110.67
100ppb3.d	Hesperidin	Calibration	14.310	925	116.3887		116.39
250ppb1.d	Hesperidin	Calibration	14.310	2063	254.5285		101.81
250ppb2.d	Hesperidin	Calibration	14.310	1805	223.2001		89.28
250ppb3.d	Hesperidin	Calibration	14.310	2096	258.5218		103.41
500ppb1.d	Hesperidin	Calibration	14.319	4319	528.3534		105.67
500ppb2.d	Hesperidin	Calibration	14.319	4313	527.5245		105.50
500ppb3.d	Hesperidin	Calibration	14.310	4334	530.1521		106.03
1000ppb1.d	Hesperidin	Calibration	14.319	8206	1000.0435		100.00
1000ppb2.d	Hesperidin	Calibration	14.319	7866	958.7951		95.88
1000ppb3.d	Hesperidin	Calibration	14.310	8209	1000.3602		100.04
22-1A-1.d	Hesperidin	Sample	14.469	166276	20182.4122		
22-1A-2.d	Hesperidin	Sample	14.469	171329	20795.6479		
22-1A-3.d	Hesperidin	Sample	14.469	171150	20773.8500		
22-2A-1.d	Hesperidin	Sample	14.469	41490	5039.1770		

Quantitative Analysis Complete Report

Agilent Technologies

Hesperidin

Data File	Compound	Sample Type	RT	Resp.	Final Conc	Exp. Conc	Accuracy
22-2A-2.d	Hesperidin	Sample	14.469	42268	5133.5475		
22-2A-3.d	Hesperidin	Sample	14.469	42102	5113.4679		
22-3A-1.d	Hesperidin	Sample	14.461	75930	9218.5385		
22-3A-2.d	Hesperidin	Sample	14.461	77895	9457.0406		
22-3A-3.d	Hesperidin	Sample	14.469	77324	9387.7620		
22-4A-1.d	Hesperidin	Sample	14.469	203159	24658.2352		
22-4A-2.d	Hesperidin	Sample	14.461	222640	27022.3521		
22-4A-3.d	Hesperidin	Sample	14.461	221271	26856.2541		
22-5A-1.d	Hesperidin	Sample	14.461	1320	164.3962		
22-5A-2.d	Hesperidin	Sample	14.469	909	114.4986		
22-5A-3.d	Hesperidin	Sample	14.461	821	103.8701		
22-6A-1.d	Hesperidin	Sample	14.536	227101	27563.7610		
22-6A-2.d	Hesperidin	Sample	14.536	230401	27964.1868		
22-6A-3.d	Hesperidin	Sample	14.536	231125	28052.0539		
22-7A-1.d	Hesperidin	Sample	14.461	565859	68673.2500		
22-7A-2.d	Hesperidin	Sample	14.461	566900	68799.5863		
22-7A-3.d	Hesperidin	Sample	14.461	567062	68819.2435		
22-8A-1.d	Hesperidin	Sample	14.444	5997	731.9445		
22-8A-2.d	Hesperidin	Sample	14.452	5690	694.6998		
22-8A-3.d	Hesperidin	Sample	14.444	5988	730.8234		
22-9A-1.d	Hesperidin	Sample	14.461	6512	794.3846		
22-9A-2.d	Hesperidin	Sample	14.469	6318	770.8509		
22-9A-3.d	Hesperidin	Sample	14.461	6374	777.7369		
22-10A-1.d	Hesperidin	Sample	14.227	6082	742.2770		
22-10A-2.d	Hesperidin	Sample	14.227	6277	765.9827		
22-10A-3.d	Hesperidin	Sample	14.227	5764	703.7122		
22-11A-1.d	Hesperidin	Sample	14.461	37742	4584.3026		
22-11A-2.d	Hesperidin	Sample	14.461	38084	4625.7826		
22-11A-3.d	Hesperidin	Sample	14.461	37456	4549.5603		
22-12A-1.d	Hesperidin	Sample	14.319	16	6.0966		
22-12A-2.d	Hesperidin	Sample	14.302	37	8.6498		
22-12A-3.d	Hesperidin	Sample	14.319	44	9.5416		
22-13A-1.d	Hesperidin	Sample	14.461	2912	357.6052		
22-13A-2.d	Hesperidin	Sample	14.461	2899	356.0422		
22-13A-3.d	Hesperidin	Sample	14.461	2863	351.6092		
22-14A-1.d	Hesperidin	Sample	14.461	10015	1219.5895		
22-14A-2.d	Hesperidin	Sample	14.461	10307	1254.9604		
22-14A-3.d	Hesperidin	Sample	14.461	10216	1243.8854		
22-15A-1.d	Hesperidin	Sample	14.461	6147	750.1395		
22-15A-2.d	Hesperidin	Sample	14.469	6381	778.5929		

Hesperidin

Data File	Compound	Sample Type	RT	Resp.	Final Conc	Exp. Conc	Accuracy
22-15A-3.d	Hesperidin	Sample	14.469	6270	765.1268		
22-16A-1.d	Hesperidin	Sample	14.461	930	117.0218		
22-16A-2.d	Hesperidin	Sample	14.469	804	101.7598		
22-16A-3.d	Hesperidin	Sample	14.461	886	111.7634		
22-17A-1.d	Hesperidin	Sample	14.461	31844	3868.5278		
22-17A-2.d	Hesperidin	Sample	14.452	31789	3861.9454		
22-17A-3.d	Hesperidin	Sample	14.452	28624	3477.7688		
22-18A-1.d	Hesperidin	Sample	14.436	358561	43516.8777		
22-18A-2.d	Hesperidin	Sample	14.436	346145	42010.1331		
22-18A-3.d	Hesperidin	Sample	14.436	347250	42144.2166		
22-1B-1.d	Hesperidin	Sample	14.436	211169	25630.2653		
22-1B-2.d	Hesperidin	Sample	14.436	218036	26463.6159		
22-1B-3.d	Hesperidin	Sample	14.427	207263	25156.3338		
22-2B-1.d	Hesperidin	Sample	14.436	46695	5670.7369		
22-2B-2.d	Hesperidin	Sample	14.436	46059	5593.5828		
22-2B-3.d	Hesperidin	Sample	14.436	46893	5694.8715		
22-3B-1.d	Hesperidin	Sample	14.419	93332	11330.3560		
22-3B-2.d	Hesperidin	Sample	14.427	91513	11109.6533		
22-3B-3.d	Hesperidin	Sample	14.427	95237	11561.5359		
22-4B-1.d	Hesperidin	Sample	14.427	242310	29409.3804		
22-4B-2.d	Hesperidin	Sample	14.427	241251	29280.8683		
22-4B-3.d	Hesperidin	Sample	14.436	241323	29289.6719		
22-5B-1.d	Hesperidin	Sample	14.436	1560	193.5006		
22-5B-2.d	Hesperidin	Sample	14.427	1261	157.2201		
22-5B-3.d	Hesperidin	Sample	14.427	1170	146.1617		
22-6B-1.d	Hesperidin	Sample	14.503	297312	36084.0695		
22-6B-2.d	Hesperidin	Sample	14.503	316053	38358.3746		
22-6B-3.d	Hesperidin	Sample	14.503	303585	36845.3730		
22-7B-1.d	Hesperidin	Sample	14.436	592198	71869.5980		
22-7B-2.d	Hesperidin	Sample	14.436	592904	71955.2574		
22-7B-3.d	Hesperidin	Sample	14.427	592051	71851.8040		
22-8B-1.d	Hesperidin	Sample	14.419	6919	843.8603		
22-8B-2.d	Hesperidin	Sample	14.419	6331	772.4996		
22-8B-3.d	Hesperidin	Sample	14.419	6089	743.1613		
22-9B-1.d	Hesperidin	Sample	14.436	7683	936.5548		
22-9B-2.d	Hesperidin	Sample	14.436	7084	863.8485		
22-9B-3.d	Hesperidin	Sample	14.436	7512	915.8358		
22-10B-1.d	Hesperidin	Sample	14.202	6079	741.9089		
22-10B-2.d	Hesperidin	Sample	14.194	6504	793.5069		
22-10B-3.d	Hesperidin	Sample	14.202	6101	744.6108		

Hesperidin

Data File	Compound	Sample Type	RT	Resp.	Final Conc	Exp. Conc	Accuracy
22-11B-1.d	Hesperidin	Sample	14.436	51186	6215.8311		
22-11B-2.d	Hesperidin	Sample	14.436	53150	6454.1043		
22-11B-3.d	Hesperidin	Sample	14.436	51206	6218.2416		
22-12B-1.d	Hesperidin	Sample	14.436	295	39.9321		
22-12B-2.d	Hesperidin	Sample	14.436	205	29.0343		
22-12B-3.d	Hesperidin	Sample	14.427	186	26.8004		
22-13B-1.d	Hesperidin	Sample	14.436	3895	476.8188		
22-13B-2.d	Hesperidin	Sample	14.427	3946	483.0389		
22-13B-3.d	Hesperidin	Sample	14.436	3977	486.7906		
22-14B-1.d	Hesperidin	Sample	14.436	11994	1459.7445		
22-14B-2.d	Hesperidin	Sample	14.436	11166	1359.2122		
22-14B-3.d	Hesperidin	Sample	14.436	11368	1383.7227		
22-15B-1.d	Hesperidin	Sample	14.436	5185	633.4380		
22-15B-2.d	Hesperidin	Sample	14.427	5000	610.9026		
22-15B-3.d	Hesperidin	Sample	14.436	5167	631.2382		
22-16B-1.d	Hesperidin	Sample	14.444	860	108.5311		
22-16B-2.d	Hesperidin	Sample	14.436	816	103.2578		
22-16B-3.d	Hesperidin	Sample	14.436	775	98.2507		
22-17B-1.d	Hesperidin	Sample	14.436	31850	3869.3255		
22-17B-2.d	Hesperidin	Sample	14.436	31719	3853.3756		
22-17B-3.d	Hesperidin	Sample	14.436	30169	3665.2507		
22-18B-1.d	Hesperidin	Sample	14.427	118809	14422.1332		
22-18B-2.d	Hesperidin	Sample	14.427	130733	15869.0758		
22-18B-3.d	Hesperidin	Sample	14.427	128409	15587.1199		
22-1C-1.d	Hesperidin	Sample	14.427	149576	18155.8129		
22-1C-2.d	Hesperidin	Sample	14.427	155703	18899.2876		
22-1C-3.d	Hesperidin	Sample	14.427	152606	18523.4744		
22-2C-1.d	Hesperidin	Sample	14.427	38313	4653.6246		
22-2C-2.d	Hesperidin	Sample	14.427	39178	4758.5410		
22-2C-3.d	Hesperidin	Sample	14.427	36989	4492.9236		
22-3C-1.d	Hesperidin	Sample	14.427	75666	9186.5328		
22-3C-2.d	Hesperidin	Sample	14.419	71507	8681.8303		
22-3C-3.d	Hesperidin	Sample	14.427	78627	9545.8716		
22-4C-1.d	Hesperidin	Sample	14.427	210228	25516.1329		
22-4C-2.d	Hesperidin	Sample	14.427	229710	27880.3954		
22-4C-3.d	Hesperidin	Sample	14.427	219440	26634.0208		
22-5C-1.d	Hesperidin	Sample	14.427	1280	159.5688		
22-5C-2.d	Hesperidin	Sample	14.427	1109	138.7440		
22-5C-3.d	Hesperidin	Sample	14.427	944	118.7956		
22-6C-1.d	Hesperidin	Sample	14.503	262168	31819.1821		

Hesperidin

Data File	Compound	Sample Type	RT	Resp.	Final Conc	Exp. Conc	Accuracy
22-6C-2.d	Hesperidin	Sample	14.503	257479	31250.2004		
22-6C-3.d	Hesperidin	Sample	14.503	268549	32593.6523		
22-7C-1.d	Hesperidin	Sample	14.427	499985	60679.1598		
22-7C-2.d	Hesperidin	Sample	14.436	507949	61645.6129		
22-7C-3.d	Hesperidin	Sample	14.427	511169	62036.4550		
22-8C-1.d	Hesperidin	Sample	14.419	4884	596.8464		
22-8C-2.d	Hesperidin	Sample	14.419	4390	536.8853		
22-8C-3.d	Hesperidin	Sample	14.427	3927	480.6952		
22-9C-1.d	Hesperidin	Sample	14.436	7340	894.9432		
22-9C-2.d	Hesperidin	Sample	14.436	7455	908.8435		
22-9C-3.d	Hesperidin	Sample	14.444	7127	869.0541		
22-10C-1.d	Hesperidin	Sample	14.202	6295	768.1555		
22-10C-2.d	Hesperidin	Sample	14.202	6636	809.5024		
22-10C-3.d	Hesperidin	Sample	14.202	6072	741.0285		
22-11C-1.d	Hesperidin	Sample	14.444	46706	5672.1552		
22-11C-2.d	Hesperidin	Sample	14.444	47084	5718.0108		
22-11C-3.d	Hesperidin	Sample	14.444	46631	5663.0076		
22-12C-1.d	Hesperidin	Sample	14.444	266	36.5123		
22-12C-2.d	Hesperidin	Sample	14.444	193	27.5592		
22-12C-3.d	Hesperidin	Sample	14.452	178	25.8105		
22-13C-1.d	Hesperidin	Sample	14.452	2812	345.4865		
22-13C-2.d	Hesperidin	Sample	14.444	3147	386.1079		
22-13C-3.d	Hesperidin	Sample	14.444	3258	399.5841		
22-14C-1.d	Hesperidin	Sample	14.444	8132	991.0654		
22-14C-2.d	Hesperidin	Sample	14.444	8161	994.5926		
22-14C-3.d	Hesperidin	Sample	14.444	8378	1020.8678		
22-15C-1.d	Hesperidin	Sample	14.444	4561	557.7034		
22-15C-2.d	Hesperidin	Sample	14.444	4580	559.9765		
22-15C-3.d	Hesperidin	Sample	14.444	4375	535.1084		
22-16C-1.d	Hesperidin	Sample	14.436	723	91.8781		
22-16C-2.d	Hesperidin	Sample	14.452	661	84.3911		
22-16C-3.d	Hesperidin	Sample	14.452	637	81.5250		
22-17C-1.d	Hesperidin	Sample	14.444	24480	2974.9626		
22-17C-2.d	Hesperidin	Sample	14.444	25295	3073.8070		
22-17C-3.d	Hesperidin	Sample	14.452	26639	3236.9611		
22-18C-1.d	Hesperidin	Sample	14.444	88658	10763.1950		
22-18C-2.d	Hesperidin	Sample	14.436	88557	10750.9534		
22-18C-3.d	Hesperidin	Sample	14.436	91794	11143.7327		

Hyperoside

Data File	Compound	Sample Type	RT	Resp.	Final Conc	Exp. Conc	Accuracy
blank-061022-4.d	Hyperoside	Sample	14.262	2	4.0406		
25ppb1.d	Hyperoside	Calibration	14.805	177	12.8758		51.50
25ppb2.d	Hyperoside	Calibration	14.637	155	11.7859		47.14
25ppb3.d	Hyperoside	Calibration	14.303	196	13.8627		55.45
50ppb1.d	Hyperoside	Calibration	14.445	655	37.0741		74.15
50ppb2.d	Hyperoside	Calibration	14.454	744	41.5821		83.16
50ppb3.d	Hyperoside	Calibration	14.462	799	44.3805		88.76
100ppb1.d	Hyperoside	Calibration	14.479	1959	103.0652		103.07
100ppb2.d	Hyperoside	Calibration	14.470	1966	103.4669		103.47
100ppb3.d	Hyperoside	Calibration	14.470	2113	110.8718		110.87
250ppb1.d	Hyperoside	Calibration	14.479	4890	251.4230		100.57
250ppb2.d	Hyperoside	Calibration	14.479	4636	238.5983		95.44
250ppb3.d	Hyperoside	Calibration	14.479	4525	232.9429		93.18
500ppb1.d	Hyperoside	Calibration	14.487	10086	514.4364		102.89
500ppb2.d	Hyperoside	Calibration	14.495	10310	525.7668		105.15
500ppb3.d	Hyperoside	Calibration	14.487	10630	541.9716		108.39
1000ppb1.d	Hyperoside	Calibration	14.487	20105	1021.5063		102.15
1000ppb2.d	Hyperoside	Calibration	14.495	19114	971.3325		97.13
1000ppb3.d	Hyperoside	Calibration	14.487	19141	972.7189		97.27
22-1A-1.d	Hyperoside	Sample	14.504	58274	2953.3150		
22-1A-2.d	Hyperoside	Sample	14.504	60014	3041.4037		
22-1A-3.d	Hyperoside	Sample	14.495	59601	3020.4884		
22-2A-1.d	Hyperoside	Sample	14.487	21793	1106.9613		
22-2A-2.d	Hyperoside	Sample	14.495	21881	1111.3882		
22-2A-3.d	Hyperoside	Sample	14.487	22274	1131.2689		
22-3A-1.d	Hyperoside	Sample	14.495	463505	23463.1077		
22-3A-2.d	Hyperoside	Sample	14.504	469566	23769.8578		
22-3A-3.d	Hyperoside	Sample	14.504	469228	23752.7707		
22-4A-1.d	Hyperoside	Sample	14.495	152304	7712.4520		
22-4A-2.d	Hyperoside	Sample	14.495	150235	7607.7181		
22-4A-3.d	Hyperoside	Sample	14.495	149103	7550.4255		
22-5A-1.d	Hyperoside	Sample	14.495	29158	1479.7109		
22-5A-2.d	Hyperoside	Sample	14.504	27716	1406.7111		
22-5A-3.d	Hyperoside	Sample	14.495	28237	1433.0990		
22-6A-1.d	Hyperoside	Sample	14.487	283016	14328.0694		
22-6A-2.d	Hyperoside	Sample	14.487	284568	14406.6372		
22-6A-3.d	Hyperoside	Sample	14.487	281087	14230.4494		
22-7A-1.d	Hyperoside	Sample	14.504	162197	8213.1354		
22-7A-2.d	Hyperoside	Sample	14.495	162927	8250.0675		
22-7A-3.d	Hyperoside	Sample	14.495	163929	8300.7865		

Agilent Technologies

Hyperoside

Data File	Compound	Sample Type	RT	Resp.	Final Conc	Exp. Conc	Accuracy
22-8A-1.d	Hyperoside	Sample	14.487	1773022	89741.0733		
22-8A-2.d	Hyperoside	Sample	14.487	1737625	87949.5208		
22-8A-3.d	Hyperoside	Sample	14.479	1782094	90200.2241		
22-9A-1.d	Hyperoside	Sample	14.495	87202	4417.4361		
22-9A-2.d	Hyperoside	Sample	14.495	82532	4181.1053		
22-9A-3.d	Hyperoside	Sample	14.495	82197	4164.1363		
22-10A-1.d	Hyperoside	Sample	14.487	7137	365.1782		
22-10A-2.d	Hyperoside	Sample	14.495	6894	352.8405		
22-10A-3.d	Hyperoside	Sample	14.487	6374	326.5181		
22-11A-1.d	Hyperoside	Sample	14.495	12937	658.7249		
22-11A-2.d	Hyperoside	Sample	14.495	13196	671.8065		
22-11A-3.d	Hyperoside	Sample	14.495	12681	645.7774		
22-12A-1.d	Hyperoside	Sample	14.495	3565	184.3851		
22-12A-2.d	Hyperoside	Sample	14.495	3560	184.1184		
22-12A-3.d	Hyperoside	Sample	14.495	3607	186.4899		
22-13A-1.d	Hyperoside	Sample	14.487	551	31.8292		
22-13A-2.d	Hyperoside	Sample	14.479	603	34.4457		
22-13A-3.d	Hyperoside	Sample	14.504	595	34.0340		
22-14A-1.d	Hyperoside	Sample	14.495	25689	1304.1026		
22-14A-2.d	Hyperoside	Sample	14.495	25988	1319.2494		
22-14A-3.d	Hyperoside	Sample	14.495	25823	1310.9167		
22-15A-1.d	Hyperoside	Sample	14.504	27466	1394.0576		
22-15A-2.d	Hyperoside	Sample	14.504	27122	1376.6424		
22-15A-3.d	Hyperoside	Sample	14.504	27019	1371.4377		
22-16A-1.d	Hyperoside	Sample	14.504	9689	494.3172		
22-16A-2.d	Hyperoside	Sample	14.504	9563	487.9508		
22-16A-3.d	Hyperoside	Sample	14.504	9683	494.0018		
22-17A-1.d	Hyperoside	Sample	14.495	17980	913.9272		
22-17A-2.d	Hyperoside	Sample	14.487	18284	929.3306		
22-17A-3.d	Hyperoside	Sample	14.487	18030	916.4640		
22-18A-1.d	Hyperoside	Sample	14.479	116462	5898.3893		
22-18A-2.d	Hyperoside	Sample	14.470	110845	5614.0772		
22-18A-3.d	Hyperoside	Sample	14.470	112396	5692.5931		
22-1B-1.d	Hyperoside	Sample	14.470	74501	3774.6340		
22-1B-2.d	Hyperoside	Sample	14.470	76335	3867.4600		
22-1B-3.d	Hyperoside	Sample	14.462	73980	3748.2727		
22-2B-1.d	Hyperoside	Sample	14.462	24357	1236.7069		
22-2B-2.d	Hyperoside	Sample	14.454	24381	1237.9128		
22-2B-3.d	Hyperoside	Sample	14.462	24354	1236.5673		
22-3B-1.d	Hyperoside	Sample	14.462	620326	31400.2201		

Hyperoside

Data File	Compound	Sample Type	RT	Resp.	Final Conc	Exp. Conc	Accuracy
22-3B-2.d	Hyperoside	Sample	14.470	612273	30992.6252		
22-3B-3.d	Hyperoside	Sample	14.462	634788	32132.1836		
22-4B-1.d	Hyperoside	Sample	14.462	161005	8152.8232		
22-4B-2.d	Hyperoside	Sample	14.462	160586	8131.5821		
22-4B-3.d	Hyperoside	Sample	14.462	158994	8051.0025		
22-5B-1.d	Hyperoside	Sample	14.470	36384	1845.4148		
22-5B-2.d	Hyperoside	Sample	14.470	39087	1982.2382		
22-5B-3.d	Hyperoside	Sample	14.470	39526	2004.4406		
22-6B-1.d	Hyperoside	Sample	14.454	363823	18417.9455		
22-6B-2.d	Hyperoside	Sample	14.454	386029	19541.8276		
22-6B-3.d	Hyperoside	Sample	14.454	374321	18949.2573		
22-7B-1.d	Hyperoside	Sample	14.470	187391	9488.2849		
22-7B-2.d	Hyperoside	Sample	14.479	188743	9556.7100		
22-7B-3.d	Hyperoside	Sample	14.470	189035	9571.4576		
22-8B-1.d	Hyperoside	Sample	14.462	2133921	108007.1049		
22-8B-2.d	Hyperoside	Sample	14.454	2144012	108517.8183		
22-8B-3.d	Hyperoside	Sample	14.454	2074403	104994.7505		
22-9B-1.d	Hyperoside	Sample	14.470	92853	4703.4613		
22-9B-2.d	Hyperoside	Sample	14.470	86554	4384.6395		
22-9B-3.d	Hyperoside	Sample	14.470	88553	4485.8392		
22-10B-1.d	Hyperoside	Sample	14.462	8135	415.6548		
22-10B-2.d	Hyperoside	Sample	14.462	7782	397.8148		
22-10B-3.d	Hyperoside	Sample	14.462	6774	346.7862		
22-11B-1.d	Hyperoside	Sample	14.470	16930	860.8140		
22-11B-2.d	Hyperoside	Sample	14.470	16923	860.4598		
22-11B-3.d	Hyperoside	Sample	14.470	16493	838.7058		
22-12B-1.d	Hyperoside	Sample	14.470	5293	271.8239		
22-12B-2.d	Hyperoside	Sample	14.470	4901	251.9672		
22-12B-3.d	Hyperoside	Sample	14.470	5154	264.7832		
22-13B-1.d	Hyperoside	Sample	14.479	795	44.1517		
22-13B-2.d	Hyperoside	Sample	14.462	750	41.9099		
22-13B-3.d	Hyperoside	Sample	14.462	750	41.8880		
22-14B-1.d	Hyperoside	Sample	14.479	28651	1454.0254		
22-14B-2.d	Hyperoside	Sample	14.470	30053	1525.0197		
22-14B-3.d	Hyperoside	Sample	14.470	30726	1559.0727		
22-15B-1.d	Hyperoside	Sample	14.470	20155	1024.0287		
22-15B-2.d	Hyperoside	Sample	14.470	20224	1027.5511		
22-15B-3.d	Hyperoside	Sample	14.470	20404	1036.6394		
22-16B-1.d	Hyperoside	Sample	14.470	10211	520.7194		
22-16B-2.d	Hyperoside	Sample	14.470	9968	508.4459		

Hyperoside

Data File	Compound	Sample Type	RT	Resp.	Final Conc	Exp. Conc	Accuracy
22-16B-3.d	Hyperoside	Sample	14.470	9995	509.7867		
22-17B-1.d	Hyperoside	Sample	14.470	18585	944.5970		
22-17B-2.d	Hyperoside	Sample	14.470	18593	944.9997		
22-17B-3.d	Hyperoside	Sample	14.470	18546	942.5816		
22-18B-1.d	Hyperoside	Sample	14.462	65270	3307.3994		
22-18B-2.d	Hyperoside	Sample	14.462	71816	3638.7472		
22-18B-3.d	Hyperoside	Sample	14.462	70594	3576.8705		
22-1C-1.d	Hyperoside	Sample	14.462	52272	2649.5666		
22-1C-2.d	Hyperoside	Sample	14.462	57879	2933.3423		
22-1C-3.d	Hyperoside	Sample	14.462	55541	2815.0092		
22-2C-1.d	Hyperoside	Sample	14.454	19998	1016.0787		
22-2C-2.d	Hyperoside	Sample	14.454	20203	1026.4759		
22-2C-3.d	Hyperoside	Sample	14.454	19123	971.8134		
22-3C-1.d	Hyperoside	Sample	14.462	436078	22074.9459		
22-3C-2.d	Hyperoside	Sample	14.462	429182	21725.9136		
22-3C-3.d	Hyperoside	Sample	14.462	448623	22709.8794		
22-4C-1.d	Hyperoside	Sample	14.462	143176	7250.4625		
22-4C-2.d	Hyperoside	Sample	14.462	151226	7657.8480		
22-4C-3.d	Hyperoside	Sample	14.462	149621	7576.6251		
22-5C-1.d	Hyperoside	Sample	14.470	22584	1146.9755		
22-5C-2.d	Hyperoside	Sample	14.462	21431	1088.6159		
22-5C-3.d	Hyperoside	Sample	14.470	21690	1101.7062		
22-6C-1.d	Hyperoside	Sample	14.454	311162	15752.6119		
22-6C-2.d	Hyperoside	Sample	14.454	314112	15901.9625		
22-6C-3.d	Hyperoside	Sample	14.454	323679	16386.1279		
22-7C-1.d	Hyperoside	Sample	14.470	143573	7270.5310		
22-7C-2.d	Hyperoside	Sample	14.470	144591	7322.0410		
22-7C-3.d	Hyperoside	Sample	14.470	145495	7367.7948		
22-8C-1.d	Hyperoside	Sample	14.454	1516150	76740.1428		
22-8C-2.d	Hyperoside	Sample	14.454	1445027	73140.4109		
22-8C-3.d	Hyperoside	Sample	14.462	1524363	77155.8316		
22-9C-1.d	Hyperoside	Sample	14.470	97462	4936.7621		
22-9C-2.d	Hyperoside	Sample	14.470	92897	4705.6962		
22-9C-3.d	Hyperoside	Sample	14.470	87580	4436.5949		
22-10C-1.d	Hyperoside	Sample	14.470	7625	389.8672		
22-10C-2.d	Hyperoside	Sample	14.470	7742	395.7982		
22-10C-3.d	Hyperoside	Sample	14.470	7398	378.3754		
22-11C-1.d	Hyperoside	Sample	14.479	15695	798.2852		
22-11C-2.d	Hyperoside	Sample	14.479	15545	790.7290		
22-11C-3.d	Hyperoside	Sample	14.479	15355	781.0971		

Hyperoside

Data File	Compound	Sample Type	RT	Resp.	Final Conc	Exp. Conc	Accuracy
22-12C-1.d	Hyperoside	Sample	14.479	4044	208.6391		
22-12C-2.d	Hyperoside	Sample	14.470	3823	197.4424		
22-12C-3.d	Hyperoside	Sample	14.479	3733	192.8741		
22-13C-1.d	Hyperoside	Sample	14.470	655	37.0972		
22-13C-2.d	Hyperoside	Sample	14.470	612	34.9309		
22-13C-3.d	Hyperoside	Sample	14.470	645	36.5916		
22-14C-1.d	Hyperoside	Sample	14.470	19267	979.0778		
22-14C-2.d	Hyperoside	Sample	14.470	19339	982.7217		
22-14C-3.d	Hyperoside	Sample	14.470	20089	1020.6995		
22-15C-1.d	Hyperoside	Sample	14.479	17769	903.2709		
22-15C-2.d	Hyperoside	Sample	14.479	18172	923.6934		
22-15C-3.d	Hyperoside	Sample	14.479	18217	925.9603		
22-16C-1.d	Hyperoside	Sample	14.470	6836	349.9362		
22-16C-2.d	Hyperoside	Sample	14.487	7019	359.1886		
22-16C-3.d	Hyperoside	Sample	14.479	6929	354.6321		
22-17C-1.d	Hyperoside	Sample	14.479	14017	713.3510		
22-17C-2.d	Hyperoside	Sample	14.479	14643	745.0736		
22-17C-3.d	Hyperoside	Sample	14.479	14769	751.4289		
22-18C-1.d	Hyperoside	Sample	14.479	27747	1408.2707		
22-18C-2.d	Hyperoside	Sample	14.470	28423	1442.4932		
22-18C-3.d	Hyperoside	Sample	14.470	28401	1441.4086		

Rosmarinic acid

Data File	Compound	Sample Type	RT	Resp.	Final Conc	Exp. Conc	Accuracy
blank-061022-4.d	Rosmarinic acid	Sample	14.347	12	ND		
25ppb1.d	Rosmarinic acid	Calibration	14.355	13	ND		0.00
25ppb2.d	Rosmarinic acid	Calibration	14.397	2	ND		0.00
25ppb3.d	Rosmarinic acid	Calibration	14.372	207	15.2585		61.03
50ppb1.d	Rosmarinic acid	Calibration	14.464	517	40.0507		80.10
50ppb2.d	Rosmarinic acid	Calibration	14.456	537	41.6587		83.32
50ppb3.d	Rosmarinic acid	Calibration	14.464	600	46.6701		93.34
100ppb1.d	Rosmarinic acid	Calibration	14.473	1331	105.1277		105.13
100ppb2.d	Rosmarinic acid	Calibration	14.473	1299	102.5878		102.59
100ppb3.d	Rosmarinic acid	Calibration	14.481	1445	114.2891		114.29
250ppb1.d	Rosmarinic acid	Calibration	14.481	3025	240.6256		96.25
250ppb2.d	Rosmarinic acid	Calibration	14.481	3169	252.1411		100.86
250ppb3.d	Rosmarinic acid	Calibration	14.481	2932	233.1788		93.27
500ppb1.d	Rosmarinic acid	Calibration	14.489	6474	516.4535		103.29
500ppb2.d	Rosmarinic acid	Calibration	14.489	6530	520.9417		104.19
500ppb3.d	Rosmarinic acid	Calibration	14.489	6508	519.1533		103.83
1000ppb1.d	Rosmarinic acid	Calibration	14.489	12837	1025.2666		102.53

Rosmarinic acid

Data File	Compound	Sample Type	RT	Resp.	Final Conc	Exp. Conc	Accuracy
1000ppb2.d	Rosmarinic acid	Calibration	14.489	12233	976.9791		97.70
1000ppb3.d	Rosmarinic acid	Calibration	14.481	12204	974.6178		97.46
22-1A-1.d	Rosmarinic acid	Sample	14.489	311	23.5645		
22-1A-2.d	Rosmarinic acid	Sample	14.498	248	18.5152		
22-1A-3.d	Rosmarinic acid	Sample	14.489	206	15.2092		
22-2A-1.d	Rosmarinic acid	Sample	14.473	46	2.3993		
22-2A-2.d	Rosmarinic acid	Sample	14.523	29	0.9995		
22-2A-3.d	Rosmarinic acid	Sample	14.489	35	1.5334		
22-3A-1.d	Rosmarinic acid	Sample	14.473	1507	119.2376		
22-3A-2.d	Rosmarinic acid	Sample	14.481	1402	110.8691		
22-3A-3.d	Rosmarinic acid	Sample	14.481	1319	104.1949		
22-4A-1.d	Rosmarinic acid	Sample	14.489	8	ND		
22-4A-2.d	Rosmarinic acid	Sample	14.481	14	ND		
22-4A-3.d	Rosmarinic acid	Sample	14.489	10	ND		
22-5A-1.d	Rosmarinic acid	Sample	14.489	8	ND		
22-5A-2.d	Rosmarinic acid	Sample	14.498	13	ND		
22-5A-3.d	Rosmarinic acid	Sample	14.464	3	ND		
22-6A-1.d	Rosmarinic acid	Sample	14.406	10	ND		
22-6A-2.d	Rosmarinic acid	Sample	14.464	9	ND		
22-6A-3.d	Rosmarinic acid	Sample	14.506	2	ND		
22-7A-1.d	Rosmarinic acid	Sample	14.464	3	ND		
22-7A-2.d	Rosmarinic acid	Sample	14.515	3	ND		
22-7A-3.d	Rosmarinic acid	Sample	14.347	13	ND		
22-8A-1.d	Rosmarinic acid	Sample	14.473	5	ND		
22-8A-2.d	Rosmarinic acid	Sample	14.481	9	ND		
22-8A-3.d	Rosmarinic acid	Sample	14.456	5	ND		
22-9A-1.d	Rosmarinic acid	Sample	14.473	4	ND		
22-9A-2.d	Rosmarinic acid	Sample	14.456	3	ND		
22-9A-3.d	Rosmarinic acid	Sample	14.464	12	ND		
22-10A-1.d	Rosmarinic acid	Sample	14.439	3	ND		
22-10A-2.d	Rosmarinic acid	Sample	14.498	16	0.0277		
22-10A-3.d	Rosmarinic acid	Sample	14.464	7	ND		
22-11A-1.d	Rosmarinic acid	Sample	14.297	2	ND		
22-11A-2.d	Rosmarinic acid	Sample	14.481	4	ND		
22-11A-3.d	Rosmarinic acid	Sample	14.481	6	ND		
22-12A-1.d	Rosmarinic acid	Sample	14.481	43560	3482.1165		
22-12A-2.d	Rosmarinic acid	Sample	14.473	44153	3529.5746		
22-12A-3.d	Rosmarinic acid	Sample	14.481	43569	3482.8152		
22-13A-1.d	Rosmarinic acid	Sample	14.481	20650	1650.0571		
22-13A-2.d	Rosmarinic acid	Sample	14.481	20718	1655.5280		

Rosmarinic acid

Data File	Compound	Sample Type	RT	Resp.	Final Conc	Exp. Conc	Accuracy
22-13A-3.d	Rosmarinic acid	Sample	14.481	20743	1657.5136		
22-14A-1.d	Rosmarinic acid	Sample	14.481	375865	30055.9430		
22-14A-2.d	Rosmarinic acid	Sample	14.481	370899	29658.8019		
22-14A-3.d	Rosmarinic acid	Sample	14.489	376580	30113.1279		
22-15A-1.d	Rosmarinic acid	Sample	14.481	23495	1877.5640		
22-15A-2.d	Rosmarinic acid	Sample	14.481	19857	1586.6509		
22-15A-3.d	Rosmarinic acid	Sample	14.481	19640	1569.3202		
22-16A-1.d	Rosmarinic acid	Sample	14.489	63195	5052.3323		
22-16A-2.d	Rosmarinic acid	Sample	14.489	65003	5196.8775		
22-16A-3.d	Rosmarinic acid	Sample	14.489	64617	5165.9830		
22-17A-1.d	Rosmarinic acid	Sample	14.481	42871	3427.0593		
22-17A-2.d	Rosmarinic acid	Sample	14.473	42921	3431.0166		
22-17A-3.d	Rosmarinic acid	Sample	14.473	43171	3451.0232		
22-18A-1.d	Rosmarinic acid	Sample	14.464	308	23.3430		
22-18A-2.d	Rosmarinic acid	Sample	14.464	200	14.7220		
22-18A-3.d	Rosmarinic acid	Sample	14.464	146	10.4258		
22-1B-1.d	Rosmarinic acid	Sample	14.448	372	28.4896		
22-1B-2.d	Rosmarinic acid	Sample	14.464	416	31.9463		
22-1B-3.d	Rosmarinic acid	Sample	14.464	385	29.4831		
22-2B-1.d	Rosmarinic acid	Sample	14.456	61	3.5681		
22-2B-2.d	Rosmarinic acid	Sample	14.456	104	7.0340		
22-2B-3.d	Rosmarinic acid	Sample	14.439	49	2.6110		
22-3B-1.d	Rosmarinic acid	Sample	14.456	1985	157.4584		
22-3B-2.d	Rosmarinic acid	Sample	14.448	1930	153.0229		
22-3B-3.d	Rosmarinic acid	Sample	14.448	2053	162.9096		
22-4B-1.d	Rosmarinic acid	Sample	14.439	43	2.1331		
22-4B-2.d	Rosmarinic acid	Sample	14.481	44	2.2221		
22-4B-3.d	Rosmarinic acid	Sample	14.473	31	1.2186		
22-5B-1.d	Rosmarinic acid	Sample	14.439	30	1.1555		
22-5B-2.d	Rosmarinic acid	Sample	14.439	35	1.5114		
22-5B-3.d	Rosmarinic acid	Sample	14.439	30	1.1110		
22-6B-1.d	Rosmarinic acid	Sample	14.448	15	ND		
22-6B-2.d	Rosmarinic acid	Sample	14.489	4	ND		
22-6B-3.d	Rosmarinic acid	Sample	14.481	9	ND		
22-7B-1.d	Rosmarinic acid	Sample	14.489	3	ND		
22-7B-2.d	Rosmarinic acid	Sample	14.515	9	ND		
22-7B-3.d	Rosmarinic acid	Sample	14.489	10	ND		
22-8B-1.d	Rosmarinic acid	Sample	14.515	3	ND		
22-8B-2.d	Rosmarinic acid	Sample	14.506	12	ND		
22-8B-3.d	Rosmarinic acid	Sample	14.515	5	ND		

Rosmarinic acid

Data File	Compound	Sample Type	RT	Resp.	Final Conc	Exp. Conc	Accuracy
22-9B-1.d	Rosmarinic acid	Sample	14.498	10	ND		
22-9B-2.d	Rosmarinic acid	Sample	14.498	13	ND		
22-9B-3.d	Rosmarinic acid	Sample	14.464	15	ND		
22-10B-1.d	Rosmarinic acid	Sample	14.448	26	0.7955		
22-10B-2.d	Rosmarinic acid	Sample	14.473	30	1.1366		
22-10B-3.d	Rosmarinic acid	Sample	14.414	31	1.1844		
22-11B-1.d	Rosmarinic acid	Sample	14.506	7	ND		
22-11B-2.d	Rosmarinic acid	Sample	14.448	7	ND		
22-11B-3.d	Rosmarinic acid	Sample	14.489	11	ND		
22-12B-1.d	Rosmarinic acid	Sample	14.456	66611	5325.4715		
22-12B-2.d	Rosmarinic acid	Sample	14.456	62480	4995.1505		
22-12B-3.d	Rosmarinic acid	Sample	14.456	66220	5294.1771		
22-13B-1.d	Rosmarinic acid	Sample	14.456	27948	2233.6491		
22-13B-2.d	Rosmarinic acid	Sample	14.456	29261	2338.6951		
22-13B-3.d	Rosmarinic acid	Sample	14.456	27925	2231.8558		
22-14B-1.d	Rosmarinic acid	Sample	14.456	466758	37324.5268		
22-14B-2.d	Rosmarinic acid	Sample	14.456	440269	35206.2357		
22-14B-3.d	Rosmarinic acid	Sample	14.456	442966	35421.9320		
22-15B-1.d	Rosmarinic acid	Sample	14.456	32002	2557.8949		
22-15B-2.d	Rosmarinic acid	Sample	14.456	27875	2227.8208		
22-15B-3.d	Rosmarinic acid	Sample	14.448	27626	2207.9561		
22-16B-1.d	Rosmarinic acid	Sample	14.456	77347	6183.9884		
22-16B-2.d	Rosmarinic acid	Sample	14.456	75526	6038.4233		
22-16B-3.d	Rosmarinic acid	Sample	14.448	76768	6137.7259		
22-17B-1.d	Rosmarinic acid	Sample	14.456	67712	5413.5486		
22-17B-2.d	Rosmarinic acid	Sample	14.456	67148	5368.4490		
22-17B-3.d	Rosmarinic acid	Sample	14.456	68299	5460.4331		
22-18B-1.d	Rosmarinic acid	Sample	14.448	598	46.5081		
22-18B-2.d	Rosmarinic acid	Sample	14.456	358	27.3855		
22-18B-3.d	Rosmarinic acid	Sample	14.464	260	19.4954		
22-1C-1.d	Rosmarinic acid	Sample	14.448	327	24.8687		
22-1C-2.d	Rosmarinic acid	Sample	14.464	314	23.7937		
22-1C-3.d	Rosmarinic acid	Sample	14.439	291	21.9674		
22-2C-1.d	Rosmarinic acid	Sample	14.431	121	8.4094		
22-2C-2.d	Rosmarinic acid	Sample	14.448	96	6.3854		
22-2C-3.d	Rosmarinic acid	Sample	14.431	61	3.5757		
22-3C-1.d	Rosmarinic acid	Sample	14.448	1581	125.1324		
22-3C-2.d	Rosmarinic acid	Sample	14.456	1425	112.6920		
22-3C-3.d	Rosmarinic acid	Sample	14.439	1572	124.4486		
22-4C-1.d	Rosmarinic acid	Sample	14.431	56	3.2246		

Rosmarinic acid

Data File	Compound	Sample Type	RT	Resp.	Final Conc	Exp. Conc	Accuracy
22-4C-2.d	Rosmarinic acid	Sample	14.456	50	2.7094		
22-4C-3.d	Rosmarinic acid	Sample	14.439	30	1.1517		
22-5C-1.d	Rosmarinic acid	Sample	14.464	58	3.3281		
22-5C-2.d	Rosmarinic acid	Sample	14.431	44	2.2425		
22-5C-3.d	Rosmarinic acid	Sample	14.464	42	2.1141		
22-6C-1.d	Rosmarinic acid	Sample	14.431	28	0.9232		
22-6C-2.d	Rosmarinic acid	Sample	14.448	27	0.8808		
22-6C-3.d	Rosmarinic acid	Sample	14.397	39	1.8609		
22-7C-1.d	Rosmarinic acid	Sample	14.439	22	0.4888		
22-7C-2.d	Rosmarinic acid	Sample	14.464	22	0.5127		
22-7C-3.d	Rosmarinic acid	Sample	14.489	29	1.0416		
22-8C-1.d	Rosmarinic acid	Sample	14.448	12	ND		
22-8C-2.d	Rosmarinic acid	Sample	14.489	11	ND		
22-8C-3.d	Rosmarinic acid	Sample	14.456	14	ND		
22-9C-1.d	Rosmarinic acid	Sample	14.464	19	0.2337		
22-9C-2.d	Rosmarinic acid	Sample	14.464	29	1.0720		
22-9C-3.d	Rosmarinic acid	Sample	14.464	29	1.0358		
22-10C-1.d	Rosmarinic acid	Sample	14.431	13	ND		
22-10C-2.d	Rosmarinic acid	Sample	14.489	22	0.4394		
22-10C-3.d	Rosmarinic acid	Sample	14.456	21	0.3984		
22-11C-1.d	Rosmarinic acid	Sample	14.456	20	0.3285		
22-11C-2.d	Rosmarinic acid	Sample	14.473	28	0.9344		
22-11C-3.d	Rosmarinic acid	Sample	14.464	24	0.6592		
22-12C-1.d	Rosmarinic acid	Sample	14.456	51462	4114.0292		
22-12C-2.d	Rosmarinic acid	Sample	14.456	51324	4103.0413		
22-12C-3.d	Rosmarinic acid	Sample	14.464	53145	4248.6486		
22-13C-1.d	Rosmarinic acid	Sample	14.464	25303	2022.1675		
22-13C-2.d	Rosmarinic acid	Sample	14.464	25329	2024.2581		
22-13C-3.d	Rosmarinic acid	Sample	14.464	24953	1994.2026		
22-14C-1.d	Rosmarinic acid	Sample	14.464	378221	30244.3465		
22-14C-2.d	Rosmarinic acid	Sample	14.456	373022	29828.6264		
22-14C-3.d	Rosmarinic acid	Sample	14.456	388880	31096.7526		
22-15C-1.d	Rosmarinic acid	Sample	14.464	24720	1975.5095		
22-15C-2.d	Rosmarinic acid	Sample	14.456	23258	1858.6418		
22-15C-3.d	Rosmarinic acid	Sample	14.464	21826	1744.0792		
22-16C-1.d	Rosmarinic acid	Sample	14.456	49911	3990.0301		
22-16C-2.d	Rosmarinic acid	Sample	14.464	52933	4231.6960		
22-16C-3.d	Rosmarinic acid	Sample	14.464	51639	4128.1930		
22-17C-1.d	Rosmarinic acid	Sample	14.464	51823	4142.8978		
22-17C-2.d	Rosmarinic acid	Sample	14.464	54604	4365.2781		

Rosmarinic acid

Data File	Compound	Sample Type	RT	Resp.	Final Conc	Exp. Conc	Accuracy
22-17C-3.d	Rosmarinic acid	Sample	14.464	54665	4370.1906		
22-18C-1.d	Rosmarinic acid	Sample	14.456	567	44.0889		
22-18C-2.d	Rosmarinic acid	Sample	14.456	357	27.2928		
22-18C-3.d	Rosmarinic acid	Sample	14.456	311	23.5842		

Apigenin 7-glucoside

Data File	Compound	Sample Type	RT	Resp.	Final Conc	Exp. Conc	Accuracy
blank-061022-4.d	Apigenin 7-glucoside	Sample	14.772	38	ND		
25ppb1.d	Apigenin 7-glucoside	Calibration	14.755	79	1.0439		4.18
25ppb2.d	Apigenin 7-glucoside	Calibration	14.596	213	5.0206		20.08
25ppb3.d	Apigenin 7-glucoside	Calibration	14.630	337	8.7149		34.86
50ppb1.d	Apigenin 7-glucoside	Calibration	14.713	990	28.1372		56.27
50ppb2.d	Apigenin 7-glucoside	Calibration	14.730	1120	31.9925		63.99
50ppb3.d	Apigenin 7-glucoside	Calibration	14.739	1412	40.6706		81.34
100ppb1.d	Apigenin 7-glucoside	Calibration	14.739	3368	98.8548		98.85
100ppb2.d	Apigenin 7-glucoside	Calibration	14.739	3542	104.0308		104.03
100ppb3.d	Apigenin 7-glucoside	Calibration	14.739	3776	110.9796		110.98
250ppb1.d	Apigenin 7-glucoside	Calibration	14.747	8380	247.9263		99.17
250ppb2.d	Apigenin 7-glucoside	Calibration	14.739	8641	255.6770		102.27
250ppb3.d	Apigenin 7-glucoside	Calibration	14.747	8633	255.4256		102.17
500ppb1.d	Apigenin 7-glucoside	Calibration	14.747	17437	517.2855		103.46
500ppb2.d	Apigenin 7-glucoside	Calibration	14.755	17975	533.2879		106.66
500ppb3.d	Apigenin 7-glucoside	Calibration	14.755	18072	536.1615		107.23
1000ppb1.d	Apigenin 7-glucoside	Calibration	14.747	33663	999.8474		99.98
1000ppb2.d	Apigenin 7-glucoside	Calibration	14.755	32942	978.4026		97.84
1000ppb3.d	Apigenin 7-glucoside	Calibration	14.747	32915	977.6059		97.76
22-1A-1.d	Apigenin 7-glucoside	Sample	14.747	19657	583.3102		
22-1A-2.d	Apigenin 7-glucoside	Sample	14.747	20258	601.1615		
22-1A-3.d	Apigenin 7-glucoside	Sample	14.747	20248	600.8917		
22-2A-1.d	Apigenin 7-glucoside	Sample	14.747	68999	2050.7749		
22-2A-2.d	Apigenin 7-glucoside	Sample	14.747	70035	2081.5739		
22-2A-3.d	Apigenin 7-glucoside	Sample	14.747	69358	2061.4343		
22-3A-1.d	Apigenin 7-glucoside	Sample	14.739	152719	4540.6495		
22-3A-2.d	Apigenin 7-glucoside	Sample	14.747	155311	4617.7454		
22-3A-3.d	Apigenin 7-glucoside	Sample	14.747	154641	4597.8256		
22-4A-1.d	Apigenin 7-glucoside	Sample	14.747	4487	132.1235		
22-4A-2.d	Apigenin 7-glucoside	Sample	14.747	4844	142.7630		
22-4A-3.d	Apigenin 7-glucoside	Sample	14.747	4866	143.4063		
22-5A-1.d	Apigenin 7-glucoside	Sample	14.739	161765	4809.6937		
22-5A-2.d	Apigenin 7-glucoside	Sample	14.739	161153	4791.4854		
22-5A-3.d	Apigenin 7-glucoside	Sample	14.739	160294	4765.9440		

Apigenin 7-glucoside

Data File	Compound	Sample Type	RT	Resp.	Final Conc	Exp. Conc	Accuracy
22-6A-1.d	Apigenin 7-glucoside	Sample	14.747	9504	281.3287		
22-6A-2.d	Apigenin 7-glucoside	Sample	14.747	9495	281.0714		
22-6A-3.d	Apigenin 7-glucoside	Sample	14.755	9457	279.9356		
22-7A-1.d	Apigenin 7-glucoside	Sample	14.747	88	1.2997		
22-7A-2.d	Apigenin 7-glucoside	Sample	14.739	87	1.2762		
22-7A-3.d	Apigenin 7-glucoside	Sample	14.722	110	1.9716		
22-8A-1.d	Apigenin 7-glucoside	Sample	14.739	585	16.0979		
22-8A-2.d	Apigenin 7-glucoside	Sample	14.747	628	17.3585		
22-8A-3.d	Apigenin 7-glucoside	Sample	14.739	630	17.4207		
22-9A-1.d	Apigenin 7-glucoside	Sample	14.647	2376	69.3382		
22-9A-2.d	Apigenin 7-glucoside	Sample	14.647	2376	69.3485		
22-9A-3.d	Apigenin 7-glucoside	Sample	14.638	2651	77.5450		
22-10A-1.d	Apigenin 7-glucoside	Sample	14.755	161	3.4900		
22-10A-2.d	Apigenin 7-glucoside	Sample	14.755	215	5.0791		
22-10A-3.d	Apigenin 7-glucoside	Sample	14.747	203	4.7107		
22-11A-1.d	Apigenin 7-glucoside	Sample	14.739	13452	398.7640		
22-11A-2.d	Apigenin 7-glucoside	Sample	14.747	12251	363.0414		
22-11A-3.d	Apigenin 7-glucoside	Sample	14.747	12599	373.3990		
22-12A-1.d	Apigenin 7-glucoside	Sample	14.739	268889	7995.6199		
22-12A-2.d	Apigenin 7-glucoside	Sample	14.730	271807	8082.4090		
22-12A-3.d	Apigenin 7-glucoside	Sample	14.739	272220	8094.7037		
22-13A-1.d	Apigenin 7-glucoside	Sample	14.747	25579	759.4120		
22-13A-2.d	Apigenin 7-glucoside	Sample	14.747	24534	728.3334		
22-13A-3.d	Apigenin 7-glucoside	Sample	14.755	25187	747.7721		
22-14A-1.d	Apigenin 7-glucoside	Sample	14.739	503108	14961.4603		
22-14A-2.d	Apigenin 7-glucoside	Sample	14.739	505627	15036.3705		
22-14A-3.d	Apigenin 7-glucoside	Sample	14.739	501538	14914.7647		
22-15A-1.d	Apigenin 7-glucoside	Sample	14.730	209380	6225.7884		
22-15A-2.d	Apigenin 7-glucoside	Sample	14.739	229895	6835.9352		
22-15A-3.d	Apigenin 7-glucoside	Sample	14.739	229675	6829.3646		
22-16A-1.d	Apigenin 7-glucoside	Sample	14.755	26757	794.4460		
22-16A-2.d	Apigenin 7-glucoside	Sample	14.755	26710	793.0735		
22-16A-3.d	Apigenin 7-glucoside	Sample	14.747	26476	786.1050		
22-17A-1.d	Apigenin 7-glucoside	Sample	14.739	25418	754.6395		
22-17A-2.d	Apigenin 7-glucoside	Sample	14.730	25437	755.2054		
22-17A-3.d	Apigenin 7-glucoside	Sample	14.730	25127	745.9848		
22-18A-1.d	Apigenin 7-glucoside	Sample	14.739	1048	29.8632		
22-18A-2.d	Apigenin 7-glucoside	Sample	14.730	1062	30.2789		
22-18A-3.d	Apigenin 7-glucoside	Sample	14.739	977	27.7585		
22-1B-1.d	Apigenin 7-glucoside	Sample	14.713	21975	652.2336		

Apigenin 7-glucoside

Data File	Compound	Sample Type	RT	Resp.	Final Conc	Exp. Conc	Accuracy
22-1B-2.d	Apigenin 7-glucoside	Sample	14.713	23178	688.0084		
22-1B-3.d	Apigenin 7-glucoside	Sample	14.713	22391	664.5990		
22-2B-1.d	Apigenin 7-glucoside	Sample	14.713	81864	2433.3699		
22-2B-2.d	Apigenin 7-glucoside	Sample	14.713	79932	2375.9297		
22-2B-3.d	Apigenin 7-glucoside	Sample	14.713	83322	2476.7291		
22-3B-1.d	Apigenin 7-glucoside	Sample	14.705	186479	5544.7022		
22-3B-2.d	Apigenin 7-glucoside	Sample	14.713	188304	5598.9708		
22-3B-3.d	Apigenin 7-glucoside	Sample	14.713	187399	5572.0625		
22-4B-1.d	Apigenin 7-glucoside	Sample	14.722	6131	181.0321		
22-4B-2.d	Apigenin 7-glucoside	Sample	14.722	5757	169.8951		
22-4B-3.d	Apigenin 7-glucoside	Sample	14.730	5869	173.2305		
22-5B-1.d	Apigenin 7-glucoside	Sample	14.713	182440	5424.5785		
22-5B-2.d	Apigenin 7-glucoside	Sample	14.705	192739	5730.8864		
22-5B-3.d	Apigenin 7-glucoside	Sample	14.713	191625	5697.7358		
22-6B-1.d	Apigenin 7-glucoside	Sample	14.730	11821	350.2452		
22-6B-2.d	Apigenin 7-glucoside	Sample	14.730	12496	370.3298		
22-6B-3.d	Apigenin 7-glucoside	Sample	14.730	11899	352.5861		
22-7B-1.d	Apigenin 7-glucoside	Sample	14.722	110	1.9565		
22-7B-2.d	Apigenin 7-glucoside	Sample	14.722	129	2.5163		
22-7B-3.d	Apigenin 7-glucoside	Sample	14.713	182	4.0924		
22-8B-1.d	Apigenin 7-glucoside	Sample	14.713	865	24.4096		
22-8B-2.d	Apigenin 7-glucoside	Sample	14.730	756	21.1820		
22-8B-3.d	Apigenin 7-glucoside	Sample	14.722	820	23.0610		
22-9B-1.d	Apigenin 7-glucoside	Sample	14.722	2180	63.5111		
22-9B-2.d	Apigenin 7-glucoside	Sample	14.713	2147	62.5510		
22-9B-3.d	Apigenin 7-glucoside	Sample	14.722	2125	61.8884		
22-10B-1.d	Apigenin 7-glucoside	Sample	14.722	235	5.6795		
22-10B-2.d	Apigenin 7-glucoside	Sample	14.705	168	3.6704		
22-10B-3.d	Apigenin 7-glucoside	Sample	14.705	234	5.6321		
22-11B-1.d	Apigenin 7-glucoside	Sample	14.722	16128	478.3594		
22-11B-2.d	Apigenin 7-glucoside	Sample	14.713	16819	498.8903		
22-11B-3.d	Apigenin 7-glucoside	Sample	14.722	16197	480.4110		
22-12B-1.d	Apigenin 7-glucoside	Sample	14.713	293944	8740.7833		
22-12B-2.d	Apigenin 7-glucoside	Sample	14.713	278193	8272.3374		
22-12B-3.d	Apigenin 7-glucoside	Sample	14.713	293439	8725.7727		
22-13B-1.d	Apigenin 7-glucoside	Sample	14.730	17984	533.5352		
22-13B-2.d	Apigenin 7-glucoside	Sample	14.730	19559	580.3735		
22-13B-3.d	Apigenin 7-glucoside	Sample	14.730	19692	584.3295		
22-14B-1.d	Apigenin 7-glucoside	Sample	14.713	555787	16528.1745		
22-14B-2.d	Apigenin 7-glucoside	Sample	14.713	540025	16059.4042		

Apigenin 7-glucoside

Data File	Compound	Sample Type	RT	Resp.	Final Conc	Exp. Conc	Accuracy
22-14B-3.d	Apigenin 7-glucoside	Sample	14.713	542134	16122.1254		
22-15B-1.d	Apigenin 7-glucoside	Sample	14.713	194854	5793.7795		
22-15B-2.d	Apigenin 7-glucoside	Sample	14.713	200726	5968.4125		
22-15B-3.d	Apigenin 7-glucoside	Sample	14.705	198669	5907.2303		
22-16B-1.d	Apigenin 7-glucoside	Sample	14.722	27160	806.4481		
22-16B-2.d	Apigenin 7-glucoside	Sample	14.722	26298	780.8077		
22-16B-3.d	Apigenin 7-glucoside	Sample	14.722	26826	796.5204		
22-17B-1.d	Apigenin 7-glucoside	Sample	14.713	28098	834.3551		
22-17B-2.d	Apigenin 7-glucoside	Sample	14.713	27655	821.1522		
22-17B-3.d	Apigenin 7-glucoside	Sample	14.713	28153	835.9840		
22-18B-1.d	Apigenin 7-glucoside	Sample	14.722	1207	34.5834		
22-18B-2.d	Apigenin 7-glucoside	Sample	14.722	1219	34.9401		
22-18B-3.d	Apigenin 7-glucoside	Sample	14.722	1282	36.8162		
22-1C-1.d	Apigenin 7-glucoside	Sample	14.713	19945	591.8615		
22-1C-2.d	Apigenin 7-glucoside	Sample	14.713	20994	623.0525		
22-1C-3.d	Apigenin 7-glucoside	Sample	14.713	20562	610.2106		
22-2C-1.d	Apigenin 7-glucoside	Sample	14.713	57685	1714.2857		
22-2C-2.d	Apigenin 7-glucoside	Sample	14.713	59628	1772.0620		
22-2C-3.d	Apigenin 7-glucoside	Sample	14.713	56729	1685.8434		
22-3C-1.d	Apigenin 7-glucoside	Sample	14.713	155322	4618.0598		
22-3C-2.d	Apigenin 7-glucoside	Sample	14.705	156281	4646.5972		
22-3C-3.d	Apigenin 7-glucoside	Sample	14.705	159888	4753.8694		
22-4C-1.d	Apigenin 7-glucoside	Sample	14.713	5309	156.5764		
22-4C-2.d	Apigenin 7-glucoside	Sample	14.713	4955	146.0497		
22-4C-3.d	Apigenin 7-glucoside	Sample	14.713	5161	152.1758		
22-5C-1.d	Apigenin 7-glucoside	Sample	14.713	148359	4410.9717		
22-5C-2.d	Apigenin 7-glucoside	Sample	14.713	152657	4538.8027		
22-5C-3.d	Apigenin 7-glucoside	Sample	14.713	148434	4413.2132		
22-6C-1.d	Apigenin 7-glucoside	Sample	14.722	8650	255.9421		
22-6C-2.d	Apigenin 7-glucoside	Sample	14.730	8281	244.9610		
22-6C-3.d	Apigenin 7-glucoside	Sample	14.722	8603	254.5319		
22-7C-1.d	Apigenin 7-glucoside	Sample	14.713	157	3.3510		
22-7C-2.d	Apigenin 7-glucoside	Sample	14.730	165	3.5819		
22-7C-3.d	Apigenin 7-glucoside	Sample	14.730	142	2.9013		
22-8C-1.d	Apigenin 7-glucoside	Sample	14.713	518	14.0913		
22-8C-2.d	Apigenin 7-glucoside	Sample	14.730	596	16.4086		
22-8C-3.d	Apigenin 7-glucoside	Sample	14.730	518	14.0975		
22-9C-1.d	Apigenin 7-glucoside	Sample	14.713	2269	66.1692		
22-9C-2.d	Apigenin 7-glucoside	Sample	14.705	2412	70.4331		
22-9C-3.d	Apigenin 7-glucoside	Sample	14.722	2292	66.8671		

Agilent Technologies

Apigenin 7-glucoside

Data File	Compound	Sample Type	RT	Resp.	Final Conc	Exp. Conc	Accuracy
22-10C-1.d	Apigenin 7-glucoside	Sample	14.722	188	4.2678		
22-10C-2.d	Apigenin 7-glucoside	Sample	14.713	204	4.7580		
22-10C-3.d	Apigenin 7-glucoside	Sample	14.730	218	5.1575		
22-11C-1.d	Apigenin 7-glucoside	Sample	14.722	17124	507.9545		
22-11C-2.d	Apigenin 7-glucoside	Sample	14.722	17406	516.3417		
22-11C-3.d	Apigenin 7-glucoside	Sample	14.722	15938	472.6815		
22-12C-1.d	Apigenin 7-glucoside	Sample	14.713	267287	7947.9864		
22-12C-2.d	Apigenin 7-glucoside	Sample	14.713	269950	8027.1769		
22-12C-3.d	Apigenin 7-glucoside	Sample	14.722	268945	7997.3027		
22-13C-1.d	Apigenin 7-glucoside	Sample	14.739	23422	695.2616		
22-13C-2.d	Apigenin 7-glucoside	Sample	14.730	22511	668.1815		
22-13C-3.d	Apigenin 7-glucoside	Sample	14.730	22806	676.9420		
22-14C-1.d	Apigenin 7-glucoside	Sample	14.713	421911	12546.6092		
22-14C-2.d	Apigenin 7-glucoside	Sample	14.713	422398	12561.0906		
22-14C-3.d	Apigenin 7-glucoside	Sample	14.713	429289	12766.0408		
22-15C-1.d	Apigenin 7-glucoside	Sample	14.713	185729	5522.3942		
22-15C-2.d	Apigenin 7-glucoside	Sample	14.713	195029	5798.9756		
22-15C-3.d	Apigenin 7-glucoside	Sample	14.713	188747	5612.1636		
22-16C-1.d	Apigenin 7-glucoside	Sample	14.722	22702	673.8685		
22-16C-2.d	Apigenin 7-glucoside	Sample	14.730	22304	662.0147		
22-16C-3.d	Apigenin 7-glucoside	Sample	14.730	21815	647.4728		
22-17C-1.d	Apigenin 7-glucoside	Sample	14.722	22028	653.8050		
22-17C-2.d	Apigenin 7-glucoside	Sample	14.722	22872	678.9218		
22-17C-3.d	Apigenin 7-glucoside	Sample	14.730	23048	684.1495		
22-18C-1.d	Apigenin 7-glucoside	Sample	14.730	142	2.8971		
22-18C-2.d	Apigenin 7-glucoside	Sample	14.722	100	1.6526		
22-18C-3.d	Apigenin 7-glucoside	Sample	14.713	78	1.0079		

Pinoresinol

Data File	Compound	Sample Type	RT	Resp.	Final Conc	Exp. Conc	Accuracy
blank-061022-4.d	Pinoresinol	Sample	14.767	4	ND		
25ppb1.d	Pinoresinol	Calibration	14.934	7	ND		0.00
25ppb2.d	Pinoresinol	Calibration	14.900	6	ND		0.00
25ppb3.d	Pinoresinol	Calibration	14.909	14	29.4558		117.82
50ppb1.d	Pinoresinol	Calibration	14.976	29	86.4868		172.97
50ppb2.d	Pinoresinol	Calibration	15.001	19	48.7333		97.47
50ppb3.d	Pinoresinol	Calibration	14.967	21	56.3671		112.73
100ppb1.d	Pinoresinol	Calibration	14.967	29	87.8772		87.88
100ppb2.d	Pinoresinol	Calibration	14.950	28	83.4822		83.48
100ppb3.d	Pinoresinol	Calibration	14.942	27	80.8620		80.86
250ppb1.d	Pinoresinol	Calibration	14.959	73	261.3065		104.52

Pinoresinol

Data File	Compound	Sample Type	RT	Resp.	Final Conc	Exp. Conc	Accuracy
250ppb2.d	Pinoresinol	Calibration	14.967	48	162.4476		64.98
250ppb3.d	Pinoresinol	Calibration	14.967	54	186.4562		74.58
500ppb1.d	Pinoresinol	Calibration	14.967	131	489.3227		97.86
500ppb2.d	Pinoresinol	Calibration	14.967	132	492.0144		98.40
500ppb3.d	Pinoresinol	Calibration	14.959	112	415.5208		83.10
1000ppb1.d	Pinoresinol	Calibration	14.950	263	1011.2129		101.12
1000ppb2.d	Pinoresinol	Calibration	14.967	255	979.0101		97.90
1000ppb3.d	Pinoresinol	Calibration	14.976	265	1018.8690		101.89
22-1A-1.d	Pinoresinol	Sample	14.959	23	62.5411		
22-1A-2.d	Pinoresinol	Sample	14.959	20	51.2717		
22-1A-3.d	Pinoresinol	Sample	14.959	18	44.7502		
22-2A-1.d	Pinoresinol	Sample	14.976	13	25.5653		
22-2A-2.d	Pinoresinol	Sample	14.942	11	17.7095		
22-2A-3.d	Pinoresinol	Sample	14.967	20	52.1759		
22-3A-1.d	Pinoresinol	Sample	14.909	12	20.4614		
22-3A-2.d	Pinoresinol	Sample	14.883	11	14.6006		
22-3A-3.d	Pinoresinol	Sample	14.934	11	15.3365		
22-4A-1.d	Pinoresinol	Sample	14.950	40	131.6106		
22-4A-2.d	Pinoresinol	Sample	14.967	40	130.2681		
22-4A-3.d	Pinoresinol	Sample	14.967	54	184.3889		
22-5A-1.d	Pinoresinol	Sample	15.085	322	1244.3434		
22-5A-2.d	Pinoresinol	Sample	15.093	374	1449.3466		
22-5A-3.d	Pinoresinol	Sample	15.093	351	1355.9227		
22-6A-1.d	Pinoresinol	Sample	14.959	259	993.8494		
22-6A-2.d	Pinoresinol	Sample	14.967	307	1183.1755		
22-6A-3.d	Pinoresinol	Sample	14.959	301	1161.4626		
22-7A-1.d	Pinoresinol	Sample	14.950	43	142.6740		
22-7A-2.d	Pinoresinol	Sample	14.976	47	159.3503		
22-7A-3.d	Pinoresinol	Sample	14.950	40	132.0517		
22-8A-1.d	Pinoresinol	Sample	14.967	15	31.6060		
22-8A-2.d	Pinoresinol	Sample	14.993	14	29.1794		
22-8A-3.d	Pinoresinol	Sample	14.959	17	38.3161		
22-9A-1.d	Pinoresinol	Sample	14.959	6	ND		
22-9A-2.d	Pinoresinol	Sample	14.842	4	ND		
22-9A-3.d	Pinoresinol	Sample	14.942	5	ND		
22-10A-1.d	Pinoresinol	Sample	14.967	44	145.3739		
22-10A-2.d	Pinoresinol	Sample	14.967	38	124.5685		
22-10A-3.d	Pinoresinol	Sample	14.942	48	163.1643		
22-11A-1.d	Pinoresinol	Sample	14.976	13	25.7130		
22-11A-2.d	Pinoresinol	Sample	14.925	12	21.9196		

Pinoresinol

Data File	Compound	Sample Type	RT	Resp.	Final Conc	Exp. Conc	Accuracy
22-11A-3.d	Pinoresinol	Sample	14.925	14	27.6940		
22-12A-1.d	Pinoresinol	Sample	14.950	38	122.8844		
22-12A-2.d	Pinoresinol	Sample	14.942	41	134.7565		
22-12A-3.d	Pinoresinol	Sample	14.967	48	162.4963		
22-13A-1.d	Pinoresinol	Sample	14.976	53	183.3602		
22-13A-2.d	Pinoresinol	Sample	14.967	34	108.5169		
22-13A-3.d	Pinoresinol	Sample	14.976	35	110.4667		
22-14A-1.d	Pinoresinol	Sample	14.959	148	556.7809		
22-14A-2.d	Pinoresinol	Sample	14.967	152	571.0508		
22-14A-3.d	Pinoresinol	Sample	14.976	137	514.0646		
22-15A-1.d	Pinoresinol	Sample	14.959	480	1866.0389		
22-15A-2.d	Pinoresinol	Sample	14.967	463	1799.1250		
22-15A-3.d	Pinoresinol	Sample	14.959	520	2025.8040		
22-16A-1.d	Pinoresinol	Sample	14.959	120	445.1933		
22-16A-2.d	Pinoresinol	Sample	14.967	135	504.0991		
22-16A-3.d	Pinoresinol	Sample	14.959	140	526.8193		
22-17A-1.d	Pinoresinol	Sample	14.967	120	447.8518		
22-17A-2.d	Pinoresinol	Sample	14.942	114	423.8344		
22-17A-3.d	Pinoresinol	Sample	14.934	117	435.0613		
22-18A-1.d	Pinoresinol	Sample	14.967	48	160.9531		
22-18A-2.d	Pinoresinol	Sample	14.917	38	122.7862		
22-18A-3.d	Pinoresinol	Sample	14.942	40	129.8687		
22-1B-1.d	Pinoresinol	Sample	14.934	24	65.7470		
22-1B-2.d	Pinoresinol	Sample	14.917	30	90.2261		
22-1B-3.d	Pinoresinol	Sample	14.917	24	69.2009		
22-2B-1.d	Pinoresinol	Sample	14.917	24	69.0513		
22-2B-2.d	Pinoresinol	Sample	14.925	24	66.5840		
22-2B-3.d	Pinoresinol	Sample	14.950	25	71.6331		
22-3B-1.d	Pinoresinol	Sample	15.043	1	ND		
22-3B-2.d	Pinoresinol	Sample	14.950	5	ND		
22-3B-3.d	Pinoresinol	Sample	14.892	6	ND		
22-4B-1.d	Pinoresinol	Sample	14.934	97	356.0271		
22-4B-2.d	Pinoresinol	Sample	14.934	93	339.6299		
22-4B-3.d	Pinoresinol	Sample	14.934	90	327.6885		
22-5B-1.d	Pinoresinol	Sample	15.076	379	1469.7582		
22-5B-2.d	Pinoresinol	Sample	15.068	423	1642.2048		
22-5B-3.d	Pinoresinol	Sample	15.076	412	1598.5351		
22-6B-1.d	Pinoresinol	Sample	14.925	446	1732.2238		
22-6B-2.d	Pinoresinol	Sample	14.934	458	1778.1675		
22-6B-3.d	Pinoresinol	Sample	14.934	422	1638.6117		

Pinoresinol

Data File	Compound	Sample Type	RT	Resp.	Final Conc	Exp. Conc	Accuracy
22-7B-1.d	Pinoresinol	Sample	14.959	58	201.1262		
22-7B-2.d	Pinoresinol	Sample	14.942	65	228.2004		
22-7B-3.d	Pinoresinol	Sample	14.950	73	261.6032		
22-8B-1.d	Pinoresinol	Sample	14.934	5	ND		
22-8B-2.d	Pinoresinol	Sample	14.917	6	ND		
22-8B-3.d	Pinoresinol	Sample	14.917	7	ND		
22-9B-1.d	Pinoresinol	Sample	14.934	3	ND		
22-9B-2.d	Pinoresinol	Sample	14.942	3	ND		
22-9B-3.d	Pinoresinol	Sample	14.917	0	ND		
22-10B-1.d	Pinoresinol	Sample	14.925	54	184.9897		
22-10B-2.d	Pinoresinol	Sample	14.917	53	183.2422		
22-10B-3.d	Pinoresinol	Sample	14.934	47	156.7242		
22-11B-1.d	Pinoresinol	Sample	14.934	8	5.3223		
22-11B-2.d	Pinoresinol	Sample	14.909	10	10.6071		
22-11B-3.d	Pinoresinol	Sample	14.917	11	14.4730		
22-12B-1.d	Pinoresinol	Sample	14.917	78	278.7749		
22-12B-2.d	Pinoresinol	Sample	14.934	49	168.2172		
22-12B-3.d	Pinoresinol	Sample	14.934	57	196.7747		
22-13B-1.d	Pinoresinol	Sample	14.925	47	157.5889		
22-13B-2.d	Pinoresinol	Sample	14.942	53	180.0874		
22-13B-3.d	Pinoresinol	Sample	14.950	62	216.9351		
22-14B-1.d	Pinoresinol	Sample	14.934	241	923.7439		
22-14B-2.d	Pinoresinol	Sample	14.934	250	960.3970		
22-14B-3.d	Pinoresinol	Sample	14.934	223	850.7402		
22-15B-1.d	Pinoresinol	Sample	14.942	505	1964.9871		
22-15B-2.d	Pinoresinol	Sample	14.925	623	2430.9142		
22-15B-3.d	Pinoresinol	Sample	14.925	548	2133.1541		
22-16B-1.d	Pinoresinol	Sample	14.925	164	618.4035		
22-16B-2.d	Pinoresinol	Sample	14.950	141	528.3469		
22-16B-3.d	Pinoresinol	Sample	14.917	144	539.4781		
22-17B-1.d	Pinoresinol	Sample	14.934	183	696.4515		
22-17B-2.d	Pinoresinol	Sample	14.934	196	746.9889		
22-17B-3.d	Pinoresinol	Sample	14.925	189	718.8471		
22-18B-1.d	Pinoresinol	Sample	14.967	14	27.2211		
22-18B-2.d	Pinoresinol	Sample	14.909	14	28.7637		
22-18B-3.d	Pinoresinol	Sample	14.984	11	17.7097		
22-1C-1.d	Pinoresinol	Sample	14.925	28	82.7081		
22-1C-2.d	Pinoresinol	Sample	14.959	34	107.7574		
22-1C-3.d	Pinoresinol	Sample	14.959	33	104.4313		
22-2C-1.d	Pinoresinol	Sample	14.900	21	57.7952		

Pinoresinol

Data File	Compound	Sample Type	RT	Resp.	Final Conc	Exp. Conc	Accuracy
22-2C-2.d	Pinoresinol	Sample	14.934	19	47.4388		
22-2C-3.d	Pinoresinol	Sample	14.925	23	62.0839		
22-3C-1.d	Pinoresinol	Sample	14.892	1	ND		
22-3C-2.d	Pinoresinol	Sample	14.858	2	ND		
22-3C-3.d	Pinoresinol	Sample	14.934	3	ND		
22-4C-1.d	Pinoresinol	Sample	14.917	62	216.1038		
22-4C-2.d	Pinoresinol	Sample	14.925	59	204.3838		
22-4C-3.d	Pinoresinol	Sample	14.909	55	191.3765		
22-5C-1.d	Pinoresinol	Sample	15.076	350	1354.9615		
22-5C-2.d	Pinoresinol	Sample	15.076	299	1153.3834		
22-5C-3.d	Pinoresinol	Sample	15.076	308	1189.4748		
22-6C-1.d	Pinoresinol	Sample	14.925	416	1612.9898		
22-6C-2.d	Pinoresinol	Sample	14.934	407	1577.2492		
22-6C-3.d	Pinoresinol	Sample	14.925	445	1727.2249		
22-7C-1.d	Pinoresinol	Sample	14.942	58	203.6702		
22-7C-2.d	Pinoresinol	Sample	14.942	40	132.0206		
22-7C-3.d	Pinoresinol	Sample	14.934	60	208.7069		
22-8C-1.d	Pinoresinol	Sample	14.950	13	23.9216		
22-8C-2.d	Pinoresinol	Sample	14.917	12	21.3211		
22-8C-3.d	Pinoresinol	Sample	14.917	11	17.2596		
22-9C-1.d	Pinoresinol	Sample	14.791	8	6.2752		
22-9C-2.d	Pinoresinol	Sample	14.909	12	19.3304		
22-9C-3.d	Pinoresinol	Sample	14.984	11	14.7134		
22-10C-1.d	Pinoresinol	Sample	14.942	46	156.1410		
22-10C-2.d	Pinoresinol	Sample	14.917	58	200.5512		
22-10C-3.d	Pinoresinol	Sample	14.950	44	146.9453		
22-11C-1.d	Pinoresinol	Sample	14.934	11	17.9655		
22-11C-2.d	Pinoresinol	Sample	14.925	12	20.4914		
22-11C-3.d	Pinoresinol	Sample	14.925	12	20.3435		
22-12C-1.d	Pinoresinol	Sample	14.950	55	191.6947		
22-12C-2.d	Pinoresinol	Sample	14.942	54	185.9656		
22-12C-3.d	Pinoresinol	Sample	14.934	37	120.8513		
22-13C-1.d	Pinoresinol	Sample	14.967	43	144.5188		
22-13C-2.d	Pinoresinol	Sample	14.934	37	117.1302		
22-13C-3.d	Pinoresinol	Sample	14.917	53	182.4789		
22-14C-1.d	Pinoresinol	Sample	14.950	176	669.0403		
22-14C-2.d	Pinoresinol	Sample	14.934	167	630.8378		
22-14C-3.d	Pinoresinol	Sample	14.934	168	634.7953		
22-15C-1.d	Pinoresinol	Sample	14.934	499	1942.3786		
22-15C-2.d	Pinoresinol	Sample	14.934	545	2122.7550		

Pinoresinol

Data File	Compound	Sample Type	RT	Resp.	Final Conc	Exp. Conc	Accuracy
22-15C-3.d	Pinoresinol	Sample	14.934	540	2104.9272		
22-16C-1.d	Pinoresinol	Sample	14.934	114	420.9939		
22-16C-2.d	Pinoresinol	Sample	14.942	117	432.7426		
22-16C-3.d	Pinoresinol	Sample	14.934	127	473.1015		
22-17C-1.d	Pinoresinol	Sample	14.934	156	587.3236		
22-17C-2.d	Pinoresinol	Sample	14.934	164	620.7027		
22-17C-3.d	Pinoresinol	Sample	14.942	160	604.7518		
22-18C-1.d	Pinoresinol	Sample	14.892	45	151.3446		
22-18C-2.d	Pinoresinol	Sample	14.892	32	98.0870		
22-18C-3.d	Pinoresinol	Sample	14.883	46	154.3744		

2-Hydroxycinnamic acid

Data File	Compound	Sample Type	RT	Resp.	Final Conc	Exp. Conc	Accuracy
blank-061022-4.d	2-Hydroxycinnamic acid	Sample	15.072	13	ND		
25ppb1.d	2-Hydroxycinnamic acid	Calibration	15.383	119	3.8097		15.24
25ppb2.d	2-Hydroxycinnamic acid	Calibration	14.635	31	ND		0.00
25ppb3.d	2-Hydroxycinnamic acid	Calibration	14.693	322	13.3673		53.47
50ppb1.d	2-Hydroxycinnamic acid	Calibration	14.760	879	39.6209		79.24
50ppb2.d	2-Hydroxycinnamic acid	Calibration	14.760	900	40.6437		81.29
50ppb3.d	2-Hydroxycinnamic acid	Calibration	14.777	952	43.0716		86.14
100ppb1.d	2-Hydroxycinnamic acid	Calibration	14.777	2320	107.5845		107.58
100ppb2.d	2-Hydroxycinnamic acid	Calibration	14.786	2217	102.7210		102.72
100ppb3.d	2-Hydroxycinnamic acid	Calibration	14.777	2296	106.4425		106.44
250ppb1.d	2-Hydroxycinnamic acid	Calibration	14.786	5293	247.7823		99.11
250ppb2.d	2-Hydroxycinnamic acid	Calibration	14.786	5239	245.2251		98.09
250ppb3.d	2-Hydroxycinnamic acid	Calibration	14.786	5392	252.4420		100.98
500ppb1.d	2-Hydroxycinnamic acid	Calibration	14.794	11011	517.3781		103.48
500ppb2.d	2-Hydroxycinnamic acid	Calibration	14.802	11001	516.9123		103.38
500ppb3.d	2-Hydroxycinnamic acid	Calibration	14.794	10976	515.7341		103.15
1000ppb1.d	2-Hydroxycinnamic acid	Calibration	14.794	21790	1025.6140		102.56
1000ppb2.d	2-Hydroxycinnamic acid	Calibration	14.794	20803	979.0892		97.91
1000ppb3.d	2-Hydroxycinnamic acid	Calibration	14.794	20640	971.3714		97.14
22-1A-1.d	2-Hydroxycinnamic acid	Sample	14.861	34	ND		
22-1A-2.d	2-Hydroxycinnamic acid	Sample	14.996	25	ND		
22-1A-3.d	2-Hydroxycinnamic acid	Sample	14.936	11	ND		
22-2A-1.d	2-Hydroxycinnamic acid	Sample	14.853	4	ND		
22-2A-2.d	2-Hydroxycinnamic acid	Sample	15.383	24	ND		
22-2A-3.d	2-Hydroxycinnamic acid	Sample	14.786	30	ND		
22-3A-1.d	2-Hydroxycinnamic acid	Sample	15.004	4	ND		
22-3A-2.d	2-Hydroxycinnamic acid	Sample	15.030	7	ND		
22-3A-3.d	2-Hydroxycinnamic acid	Sample	15.163	13	ND		

2-Hydroxycinnamic acid

Data File	Compound	Sample Type	RT	Resp.	Final Conc	Exp. Conc	Accuracy
22-4A-1.d	2-Hydroxycinnamic acid	Sample	15.038	34	ND		
22-4A-2.d	2-Hydroxycinnamic acid	Sample	14.853	16	ND		
22-4A-3.d	2-Hydroxycinnamic acid	Sample	14.869	15	ND		
22-5A-1.d	2-Hydroxycinnamic acid	Sample	15.055	33	ND		
22-5A-2.d	2-Hydroxycinnamic acid	Sample	15.272	19	ND		
22-5A-3.d	2-Hydroxycinnamic acid	Sample	14.786	10	ND		
22-6A-1.d	2-Hydroxycinnamic acid	Sample	14.794	169	6.1508		
22-6A-2.d	2-Hydroxycinnamic acid	Sample	14.786	184	6.8519		
22-6A-3.d	2-Hydroxycinnamic acid	Sample	14.794	281	11.4335		
22-7A-1.d	2-Hydroxycinnamic acid	Sample	15.030	40	0.0836		
22-7A-2.d	2-Hydroxycinnamic acid	Sample	15.163	20	ND		
22-7A-3.d	2-Hydroxycinnamic acid	Sample	14.811	20	ND		
22-8A-1.d	2-Hydroxycinnamic acid	Sample	15.055	10	ND		
22-8A-2.d	2-Hydroxycinnamic acid	Sample	14.794	32	ND		
22-8A-3.d	2-Hydroxycinnamic acid	Sample	14.635	28	ND		
22-9A-1.d	2-Hydroxycinnamic acid	Sample	15.030	10	ND		
22-9A-2.d	2-Hydroxycinnamic acid	Sample	15.030	9	ND		
22-9A-3.d	2-Hydroxycinnamic acid	Sample	15.113	23	ND		
22-10A-1.d	2-Hydroxycinnamic acid	Sample	14.811	18	ND		
22-10A-2.d	2-Hydroxycinnamic acid	Sample	15.105	11	ND		
22-10A-3.d	2-Hydroxycinnamic acid	Sample	15.038	16	ND		
22-11A-1.d	2-Hydroxycinnamic acid	Sample	14.886	32	ND		
22-11A-2.d	2-Hydroxycinnamic acid	Sample	14.819	27	ND		
22-11A-3.d	2-Hydroxycinnamic acid	Sample	15.072	5	ND		
22-12A-1.d	2-Hydroxycinnamic acid	Sample	15.097	33	ND		
22-12A-2.d	2-Hydroxycinnamic acid	Sample	15.088	14	ND		
22-12A-3.d	2-Hydroxycinnamic acid	Sample	15.046	28	ND		
22-13A-1.d	2-Hydroxycinnamic acid	Sample	15.063	7	ND		
22-13A-2.d	2-Hydroxycinnamic acid	Sample	15.063	17	ND		
22-13A-3.d	2-Hydroxycinnamic acid	Sample	15.055	13	ND		
22-14A-1.d	2-Hydroxycinnamic acid	Sample	14.920	38	ND		
22-14A-2.d	2-Hydroxycinnamic acid	Sample	15.038	21	ND		
22-14A-3.d	2-Hydroxycinnamic acid	Sample	15.038	27	ND		
22-15A-1.d	2-Hydroxycinnamic acid	Sample	14.987	12	ND		
22-15A-2.d	2-Hydroxycinnamic acid	Sample	14.945	19	ND		
22-15A-3.d	2-Hydroxycinnamic acid	Sample	14.735	26	ND		
22-16A-1.d	2-Hydroxycinnamic acid	Sample	15.088	37	ND		
22-16A-2.d	2-Hydroxycinnamic acid	Sample	15.113	8	ND		
22-16A-3.d	2-Hydroxycinnamic acid	Sample	15.021	14	ND		
22-17A-1.d	2-Hydroxycinnamic acid	Sample	15.122	19	ND		

2-Hydroxycinnamic acid

Data File	Compound	Sample Type	RT	Resp.	Final Conc	Exp. Conc	Accuracy
22-17A-2.d	2-Hydroxycinnamic acid	Sample	15.021	6	ND		
22-17A-3.d	2-Hydroxycinnamic acid	Sample	15.055	11	ND		
22-18A-1.d	2-Hydroxycinnamic acid	Sample	15.072	19	ND		
22-18A-2.d	2-Hydroxycinnamic acid	Sample	15.138	21	ND		
22-18A-3.d	2-Hydroxycinnamic acid	Sample	14.760	2	ND		
22-1B-1.d	2-Hydroxycinnamic acid	Sample	15.105	16	ND		
22-1B-2.d	2-Hydroxycinnamic acid	Sample	15.021	20	ND		
22-1B-3.d	2-Hydroxycinnamic acid	Sample	14.869	9	ND		
22-2B-1.d	2-Hydroxycinnamic acid	Sample	14.760	32	ND		
22-2B-2.d	2-Hydroxycinnamic acid	Sample	14.961	18	ND		
22-2B-3.d	2-Hydroxycinnamic acid	Sample	14.811	25	ND		
22-3B-1.d	2-Hydroxycinnamic acid	Sample	15.105	27	ND		
22-3B-2.d	2-Hydroxycinnamic acid	Sample	15.097	4	ND		
22-3B-3.d	2-Hydroxycinnamic acid	Sample	15.105	3	ND		
22-4B-1.d	2-Hydroxycinnamic acid	Sample	15.046	11	ND		
22-4B-2.d	2-Hydroxycinnamic acid	Sample	15.046	23	ND		
22-4B-3.d	2-Hydroxycinnamic acid	Sample	15.030	26	ND		
22-5B-1.d	2-Hydroxycinnamic acid	Sample	14.786	4	ND		
22-5B-2.d	2-Hydroxycinnamic acid	Sample	15.063	18	ND		
22-5B-3.d	2-Hydroxycinnamic acid	Sample	15.021	20	ND		
22-6B-1.d	2-Hydroxycinnamic acid	Sample	14.752	604	26.6434		
22-6B-2.d	2-Hydroxycinnamic acid	Sample	14.743	601	26.5346		
22-6B-3.d	2-Hydroxycinnamic acid	Sample	14.760	588	25.9130		
22-7B-1.d	2-Hydroxycinnamic acid	Sample	14.894	31	ND		
22-7B-2.d	2-Hydroxycinnamic acid	Sample	14.769	6	ND		
22-7B-3.d	2-Hydroxycinnamic acid	Sample	15.046	17	ND		
22-8B-1.d	2-Hydroxycinnamic acid	Sample	15.046	35	ND		
22-8B-2.d	2-Hydroxycinnamic acid	Sample	14.987	9	ND		
22-8B-3.d	2-Hydroxycinnamic acid	Sample	14.928	29	ND		
22-9B-1.d	2-Hydroxycinnamic acid	Sample	14.970	8	ND		
22-9B-2.d	2-Hydroxycinnamic acid	Sample	15.113	10	ND		
22-9B-3.d	2-Hydroxycinnamic acid	Sample	15.072	24	ND		
22-10B-1.d	2-Hydroxycinnamic acid	Sample	15.063	10	ND		
22-10B-2.d	2-Hydroxycinnamic acid	Sample	14.710	6	ND		
22-10B-3.d	2-Hydroxycinnamic acid	Sample	15.423	25	ND		
22-11B-1.d	2-Hydroxycinnamic acid	Sample	15.046	9	ND		
22-11B-2.d	2-Hydroxycinnamic acid	Sample	14.811	6	ND		
22-11B-3.d	2-Hydroxycinnamic acid	Sample	15.038	11	ND		
22-12B-1.d	2-Hydroxycinnamic acid	Sample	14.996	22	ND		
22-12B-2.d	2-Hydroxycinnamic acid	Sample	14.794	8	ND		

2-Hydroxycinnamic acid

Data File	Compound	Sample Type	RT	Resp.	Final Conc	Exp. Conc	Accuracy
22-12B-3.d	2-Hydroxycinnamic acid	Sample	15.038	13	ND		
22-13B-1.d	2-Hydroxycinnamic acid	Sample	14.777	6	ND		
22-13B-2.d	2-Hydroxycinnamic acid	Sample	15.307	30	ND		
22-13B-3.d	2-Hydroxycinnamic acid	Sample	14.996	18	ND		
22-14B-1.d	2-Hydroxycinnamic acid	Sample	15.046	7	ND		
22-14B-2.d	2-Hydroxycinnamic acid	Sample	15.072	20	ND		
22-14B-3.d	2-Hydroxycinnamic acid	Sample	14.903	28	ND		
22-15B-1.d	2-Hydroxycinnamic acid	Sample	15.155	6	ND		
22-15B-2.d	2-Hydroxycinnamic acid	Sample	14.743	4	ND		
22-15B-3.d	2-Hydroxycinnamic acid	Sample	14.911	9	ND		
22-16B-1.d	2-Hydroxycinnamic acid	Sample	15.080	19	ND		
22-16B-2.d	2-Hydroxycinnamic acid	Sample	15.189	26	ND		
22-16B-3.d	2-Hydroxycinnamic acid	Sample	15.046	24	ND		
22-17B-1.d	2-Hydroxycinnamic acid	Sample	14.811	14	ND		
22-17B-2.d	2-Hydroxycinnamic acid	Sample	14.996	9	ND		
22-17B-3.d	2-Hydroxycinnamic acid	Sample	15.021	27	ND		
22-18B-1.d	2-Hydroxycinnamic acid	Sample	14.752	18	ND		
22-18B-2.d	2-Hydroxycinnamic acid	Sample	15.063	4	ND		
22-18B-3.d	2-Hydroxycinnamic acid	Sample	15.256	16	ND		
22-1C-1.d	2-Hydroxycinnamic acid	Sample	15.214	5	ND		
22-1C-2.d	2-Hydroxycinnamic acid	Sample	15.004	10	ND		
22-1C-3.d	2-Hydroxycinnamic acid	Sample	15.171	10	ND		
22-2C-1.d	2-Hydroxycinnamic acid	Sample	14.786	8	ND		
22-2C-2.d	2-Hydroxycinnamic acid	Sample	14.911	22	ND		
22-2C-3.d	2-Hydroxycinnamic acid	Sample	15.163	28	ND		
22-3C-1.d	2-Hydroxycinnamic acid	Sample	15.222	24	ND		
22-3C-2.d	2-Hydroxycinnamic acid	Sample	15.030	7	ND		
22-3C-3.d	2-Hydroxycinnamic acid	Sample	15.399	12	ND		
22-4C-1.d	2-Hydroxycinnamic acid	Sample	14.769	36	ND		
22-4C-2.d	2-Hydroxycinnamic acid	Sample	15.004	16	ND		
22-4C-3.d	2-Hydroxycinnamic acid	Sample	15.030	20	ND		
22-5C-1.d	2-Hydroxycinnamic acid	Sample	15.021	19	ND		
22-5C-2.d	2-Hydroxycinnamic acid	Sample	14.794	21	ND		
22-5C-3.d	2-Hydroxycinnamic acid	Sample	14.987	37	ND		
22-6C-1.d	2-Hydroxycinnamic acid	Sample	14.743	441	18.9659		
22-6C-2.d	2-Hydroxycinnamic acid	Sample	14.760	300	12.3386		
22-6C-3.d	2-Hydroxycinnamic acid	Sample	14.752	383	16.2626		
22-7C-1.d	2-Hydroxycinnamic acid	Sample	15.072	6	ND		
22-7C-2.d	2-Hydroxycinnamic acid	Sample	15.097	28	ND		
22-7C-3.d	2-Hydroxycinnamic acid	Sample	14.693	7	ND		

2-Hydroxycinnamic acid

Data File	Compound	Sample Type	RT	Resp.	Final Conc	Exp. Conc	Accuracy
22-8C-1.d	2-Hydroxycinnamic acid	Sample	15.021	13	ND		
22-8C-2.d	2-Hydroxycinnamic acid	Sample	15.046	35	ND		
22-8C-3.d	2-Hydroxycinnamic acid	Sample	15.055	16	ND		
22-9C-1.d	2-Hydroxycinnamic acid	Sample	15.004	26	ND		
22-9C-2.d	2-Hydroxycinnamic acid	Sample	15.105	30	ND		
22-9C-3.d	2-Hydroxycinnamic acid	Sample	15.012	5	ND		
22-10C-1.d	2-Hydroxycinnamic acid	Sample	14.626	28	ND		
22-10C-2.d	2-Hydroxycinnamic acid	Sample	15.012	14	ND		
22-10C-3.d	2-Hydroxycinnamic acid	Sample	15.063	23	ND		
22-11C-1.d	2-Hydroxycinnamic acid	Sample	14.794	24	ND		
22-11C-2.d	2-Hydroxycinnamic acid	Sample	15.030	12	ND		
22-11C-3.d	2-Hydroxycinnamic acid	Sample	15.105	15	ND		
22-12C-1.d	2-Hydroxycinnamic acid	Sample	15.080	11	ND		
22-12C-2.d	2-Hydroxycinnamic acid	Sample	15.021	3	ND		
22-12C-3.d	2-Hydroxycinnamic acid	Sample	14.643	9	ND		
22-13C-1.d	2-Hydroxycinnamic acid	Sample	14.979	14	ND		
22-13C-2.d	2-Hydroxycinnamic acid	Sample	14.760	12	ND		
22-13C-3.d	2-Hydroxycinnamic acid	Sample	14.819	26	ND		
22-14C-1.d	2-Hydroxycinnamic acid	Sample	15.072	34	ND		
22-14C-2.d	2-Hydroxycinnamic acid	Sample	14.861	18	ND		
22-14C-3.d	2-Hydroxycinnamic acid	Sample	14.894	13	ND		
22-15C-1.d	2-Hydroxycinnamic acid	Sample	15.063	27	ND		
22-15C-2.d	2-Hydroxycinnamic acid	Sample	14.911	19	ND		
22-15C-3.d	2-Hydroxycinnamic acid	Sample	14.945	22	ND		
22-16C-1.d	2-Hydroxycinnamic acid	Sample	15.523	4	ND		
22-16C-2.d	2-Hydroxycinnamic acid	Sample	15.072	23	ND		
22-16C-3.d	2-Hydroxycinnamic acid	Sample	15.163	7	ND		
22-17C-1.d	2-Hydroxycinnamic acid	Sample	14.844	5	ND		
22-17C-2.d	2-Hydroxycinnamic acid	Sample	15.146	15	ND		
22-17C-3.d	2-Hydroxycinnamic acid	Sample	15.122	5	ND		
22-18C-1.d	2-Hydroxycinnamic acid	Sample	15.012	8	ND		
22-18C-2.d	2-Hydroxycinnamic acid	Sample	15.122	3	ND		
22-18C-3.d	2-Hydroxycinnamic acid	Sample	15.088	14	ND		

Eriodictyol

Data File	Compound	Sample Type	RT	Resp.	Final Conc	Exp. Conc	Accuracy
blank-061022-4.d	Eriodictyol	Sample	15.087	1	ND		
25ppb1.d	Eriodictyol	Calibration	15.152	3	ND		0.00
25ppb2.d	Eriodictyol	Calibration	15.103	5	ND		0.00
25ppb3.d	Eriodictyol	Calibration	15.002	482	ND		0.00
50ppb1.d	Eriodictyol	Calibration	15.053	1372	35.1731		70.35

Eriodictyol

Data File	Compound	Sample Type	RT	Resp.	Final Conc	Exp. Conc	Accuracy
50ppb2.d	Eriodictyol	Calibration	15.061	1379	35.4807		70.96
50ppb3.d	Eriodictyol	Calibration	15.061	1421	37.1279		74.26
100ppb1.d	Eriodictyol	Calibration	15.070	3048	101.7680		101.77
100ppb2.d	Eriodictyol	Calibration	15.070	3082	103.0961		103.10
100ppb3.d	Eriodictyol	Calibration	15.078	3150	105.8178		105.82
250ppb1.d	Eriodictyol	Calibration	15.078	6962	257.2121		102.88
250ppb2.d	Eriodictyol	Calibration	15.070	7007	259.0168		103.61
250ppb3.d	Eriodictyol	Calibration	15.087	6297	230.8125		92.33
500ppb1.d	Eriodictyol	Calibration	15.078	14052	538.8617		107.77
500ppb2.d	Eriodictyol	Calibration	15.087	14212	545.2018		109.04
500ppb3.d	Eriodictyol	Calibration	15.078	13840	530.4438		106.09
1000ppb1.d	Eriodictyol	Calibration	15.078	25696	1001.3855		100.14
1000ppb2.d	Eriodictyol	Calibration	15.087	25161	980.1297		98.01
1000ppb3.d	Eriodictyol	Calibration	15.078	24746	963.6427		96.36
22-1A-1.d	Eriodictyol	Sample	15.061	1194	28.1003		
22-1A-2.d	Eriodictyol	Sample	15.061	1385	35.7037		
22-1A-3.d	Eriodictyol	Sample	15.061	1450	38.2774		
22-2A-1.d	Eriodictyol	Sample	15.087	449	ND		
22-2A-2.d	Eriodictyol	Sample	15.078	443	ND		
22-2A-3.d	Eriodictyol	Sample	15.078	410	ND		
22-3A-1.d	Eriodictyol	Sample	15.078	98	ND		
22-3A-2.d	Eriodictyol	Sample	15.070	25	ND		
22-3A-3.d	Eriodictyol	Sample	15.095	97	ND		
22-4A-1.d	Eriodictyol	Sample	15.078	1487	39.7528		
22-4A-2.d	Eriodictyol	Sample	15.078	1480	39.4950		
22-4A-3.d	Eriodictyol	Sample	15.078	1510	40.6797		
22-5A-1.d	Eriodictyol	Sample	15.078	2005	60.3393		
22-5A-2.d	Eriodictyol	Sample	15.078	2019	60.8910		
22-5A-3.d	Eriodictyol	Sample	15.078	2070	62.9058		
22-6A-1.d	Eriodictyol	Sample	15.078	2834	93.2524		
22-6A-2.d	Eriodictyol	Sample	15.070	2744	89.6737		
22-6A-3.d	Eriodictyol	Sample	15.078	2901	95.9236		
22-7A-1.d	Eriodictyol	Sample	15.087	98	ND		
22-7A-2.d	Eriodictyol	Sample	15.087	74	ND		
22-7A-3.d	Eriodictyol	Sample	15.078	88	ND		
22-8A-1.d	Eriodictyol	Sample	15.087	60	ND		
22-8A-2.d	Eriodictyol	Sample	15.087	89	ND		
22-8A-3.d	Eriodictyol	Sample	15.070	63	ND		
22-9A-1.d	Eriodictyol	Sample	15.061	18	ND		
22-9A-2.d	Eriodictyol	Sample	15.103	37	ND		

Agilent Technologies

Eriodictyol

Data File	Compound	Sample Type	RT	Resp.	Final Conc	Exp. Conc	Accuracy
22-9A-3.d	Eriodictyol	Sample	15.078	41	ND		
22-10A-1.d	Eriodictyol	Sample	15.078	473	ND		
22-10A-2.d	Eriodictyol	Sample	15.087	485	ND		
22-10A-3.d	Eriodictyol	Sample	15.078	398	ND		
22-11A-1.d	Eriodictyol	Sample	15.087	39	ND		
22-11A-2.d	Eriodictyol	Sample	15.078	42	ND		
22-11A-3.d	Eriodictyol	Sample	15.087	49	ND		
22-12A-1.d	Eriodictyol	Sample	15.070	64	ND		
22-12A-2.d	Eriodictyol	Sample	15.078	59	ND		
22-12A-3.d	Eriodictyol	Sample	15.087	43	ND		
22-13A-1.d	Eriodictyol	Sample	15.053	27	ND		
22-13A-2.d	Eriodictyol	Sample	15.061	12	ND		
22-13A-3.d	Eriodictyol	Sample	15.078	42	ND		
22-14A-1.d	Eriodictyol	Sample	15.061	19	ND		
22-14A-2.d	Eriodictyol	Sample	15.070	32	ND		
22-14A-3.d	Eriodictyol	Sample	15.070	36	ND		
22-15A-1.d	Eriodictyol	Sample	15.078	110	ND		
22-15A-2.d	Eriodictyol	Sample	15.087	105	ND		
22-15A-3.d	Eriodictyol	Sample	15.070	131	ND		
22-16A-1.d	Eriodictyol	Sample	15.095	73	ND		
22-16A-2.d	Eriodictyol	Sample	15.087	77	ND		
22-16A-3.d	Eriodictyol	Sample	15.070	70	ND		
22-17A-1.d	Eriodictyol	Sample	15.087	11	ND		
22-17A-2.d	Eriodictyol	Sample	15.103	13	ND		
22-17A-3.d	Eriodictyol	Sample	15.070	8	ND		
22-18A-1.d	Eriodictyol	Sample	15.053	364	ND		
22-18A-2.d	Eriodictyol	Sample	15.053	327	ND		
22-18A-3.d	Eriodictyol	Sample	15.053	395	ND		
22-1B-1.d	Eriodictyol	Sample	15.028	1678	47.3474		
22-1B-2.d	Eriodictyol	Sample	15.028	1640	45.8481		
22-1B-3.d	Eriodictyol	Sample	15.028	1646	46.0815		
22-2B-1.d	Eriodictyol	Sample	15.036	452	ND		
22-2B-2.d	Eriodictyol	Sample	15.045	449	ND		
22-2B-3.d	Eriodictyol	Sample	15.045	455	ND		
22-3B-1.d	Eriodictyol	Sample	15.053	99	ND		
22-3B-2.d	Eriodictyol	Sample	15.053	119	ND		
22-3B-3.d	Eriodictyol	Sample	15.061	101	ND		
22-4B-1.d	Eriodictyol	Sample	15.045	2436	77.4704		
22-4B-2.d	Eriodictyol	Sample	15.053	2272	70.9479		
22-4B-3.d	Eriodictyol	Sample	15.045	2407	76.3008		

Eriodictyol

Data File	Compound	Sample Type	RT	Resp.	Final Conc	Exp. Conc	Accuracy
22-5B-1.d	Eriodictyol	Sample	15.045	2651	86.0048		
22-5B-2.d	Eriodictyol	Sample	15.045	2717	88.6263		
22-5B-3.d	Eriodictyol	Sample	15.053	2705	88.1357		
22-6B-1.d	Eriodictyol	Sample	15.053	3770	130.4524		
22-6B-2.d	Eriodictyol	Sample	15.053	3890	135.1918		
22-6B-3.d	Eriodictyol	Sample	15.053	3818	132.3342		
22-7B-1.d	Eriodictyol	Sample	15.045	106	ND		
22-7B-2.d	Eriodictyol	Sample	15.061	87	ND		
22-7B-3.d	Eriodictyol	Sample	15.045	95	ND		
22-8B-1.d	Eriodictyol	Sample	15.036	42	ND		
22-8B-2.d	Eriodictyol	Sample	15.045	112	ND		
22-8B-3.d	Eriodictyol	Sample	15.045	95	ND		
22-9B-1.d	Eriodictyol	Sample	15.070	28	ND		
22-9B-2.d	Eriodictyol	Sample	15.045	57	ND		
22-9B-3.d	Eriodictyol	Sample	15.045	55	ND		
22-10B-1.d	Eriodictyol	Sample	15.053	2044	61.8848		
22-10B-2.d	Eriodictyol	Sample	15.053	2044	61.8752		
22-10B-3.d	Eriodictyol	Sample	15.045	2037	61.6003		
22-11B-1.d	Eriodictyol	Sample	15.061	64	ND		
22-11B-2.d	Eriodictyol	Sample	15.053	57	ND		
22-11B-3.d	Eriodictyol	Sample	15.061	50	ND		
22-12B-1.d	Eriodictyol	Sample	15.036	86	ND		
22-12B-2.d	Eriodictyol	Sample	15.053	56	ND		
22-12B-3.d	Eriodictyol	Sample	15.053	75	ND		
22-13B-1.d	Eriodictyol	Sample	15.053	84	ND		
22-13B-2.d	Eriodictyol	Sample	15.045	86	ND		
22-13B-3.d	Eriodictyol	Sample	15.053	91	ND		
22-14B-1.d	Eriodictyol	Sample	15.061	52	ND		
22-14B-2.d	Eriodictyol	Sample	15.045	63	ND		
22-14B-3.d	Eriodictyol	Sample	15.061	37	ND		
22-15B-1.d	Eriodictyol	Sample	15.045	160	ND		
22-15B-2.d	Eriodictyol	Sample	15.036	142	ND		
22-15B-3.d	Eriodictyol	Sample	15.045	130	ND		
22-16B-1.d	Eriodictyol	Sample	15.053	85	ND		
22-16B-2.d	Eriodictyol	Sample	15.045	69	ND		
22-16B-3.d	Eriodictyol	Sample	15.019	91	ND		
22-17B-1.d	Eriodictyol	Sample	15.087	9	ND		
22-17B-2.d	Eriodictyol	Sample	15.095	4	ND		
22-17B-3.d	Eriodictyol	Sample	15.061	7	ND		
22-18B-1.d	Eriodictyol	Sample	15.061	48	ND		

Eriodictyol

Data File	Compound	Sample Type	RT	Resp.	Final Conc	Exp. Conc	Accuracy
22-18B-2.d	Eriodictyol	Sample	15.061	58	ND		
22-18B-3.d	Eriodictyol	Sample	15.036	55	ND		
22-1C-1.d	Eriodictyol	Sample	15.019	1621	45.0616		
22-1C-2.d	Eriodictyol	Sample	15.028	1628	45.3392		
22-1C-3.d	Eriodictyol	Sample	15.019	1725	49.2192		
22-2C-1.d	Eriodictyol	Sample	15.036	533	1.8770		
22-2C-2.d	Eriodictyol	Sample	15.045	519	1.3132		
22-2C-3.d	Eriodictyol	Sample	15.036	567	3.2095		
22-3C-1.d	Eriodictyol	Sample	15.061	119	ND		
22-3C-2.d	Eriodictyol	Sample	15.045	118	ND		
22-3C-3.d	Eriodictyol	Sample	15.028	113	ND		
22-4C-1.d	Eriodictyol	Sample	15.036	1623	45.1511		
22-4C-2.d	Eriodictyol	Sample	15.045	1684	47.5608		
22-4C-3.d	Eriodictyol	Sample	15.045	1783	51.4971		
22-5C-1.d	Eriodictyol	Sample	15.045	1841	53.7996		
22-5C-2.d	Eriodictyol	Sample	15.053	1871	55.0081		
22-5C-3.d	Eriodictyol	Sample	15.053	1838	53.7151		
22-6C-1.d	Eriodictyol	Sample	15.045	3388	115.2718		
22-6C-2.d	Eriodictyol	Sample	15.053	3392	115.4347		
22-6C-3.d	Eriodictyol	Sample	15.053	3323	112.6997		
22-7C-1.d	Eriodictyol	Sample	15.045	118	ND		
22-7C-2.d	Eriodictyol	Sample	15.045	105	ND		
22-7C-3.d	Eriodictyol	Sample	15.045	99	ND		
22-8C-1.d	Eriodictyol	Sample	15.036	67	ND		
22-8C-2.d	Eriodictyol	Sample	15.053	76	ND		
22-8C-3.d	Eriodictyol	Sample	15.053	51	ND		
22-9C-1.d	Eriodictyol	Sample	15.070	47	ND		
22-9C-2.d	Eriodictyol	Sample	15.070	43	ND		
22-9C-3.d	Eriodictyol	Sample	15.078	61	ND		
22-10C-1.d	Eriodictyol	Sample	15.045	732	9.7665		
22-10C-2.d	Eriodictyol	Sample	15.061	693	8.2336		
22-10C-3.d	Eriodictyol	Sample	15.053	738	10.0008		
22-11C-1.d	Eriodictyol	Sample	15.070	54	ND		
22-11C-2.d	Eriodictyol	Sample	15.045	60	ND		
22-11C-3.d	Eriodictyol	Sample	15.053	49	ND		
22-12C-1.d	Eriodictyol	Sample	15.036	60	ND		
22-12C-2.d	Eriodictyol	Sample	15.045	69	ND		
22-12C-3.d	Eriodictyol	Sample	15.045	77	ND		
22-13C-1.d	Eriodictyol	Sample	15.045	65	ND		
22-13C-2.d	Eriodictyol	Sample	15.045	63	ND		

Agilent Technologies

Eriodictyol

Data File	Compound	Sample Type	RT	Resp.	Final Conc	Exp. Conc	Accuracy
22-13C-3.d	Eriodictyol	Sample	15.061	42	ND		
22-14C-1.d	Eriodictyol	Sample	15.061	39	ND		
22-14C-2.d	Eriodictyol	Sample	15.045	28	ND		
22-14C-3.d	Eriodictyol	Sample	15.070	41	ND		
22-15C-1.d	Eriodictyol	Sample	15.053	129	ND		
22-15C-2.d	Eriodictyol	Sample	15.045	113	ND		
22-15C-3.d	Eriodictyol	Sample	15.053	131	ND		
22-16C-1.d	Eriodictyol	Sample	15.045	95	ND		
22-16C-2.d	Eriodictyol	Sample	15.061	77	ND		
22-16C-3.d	Eriodictyol	Sample	15.045	79	ND		
22-17C-1.d	Eriodictyol	Sample	15.061	6	ND		
22-17C-2.d	Eriodictyol	Sample	15.053	5	ND		
22-17C-3.d	Eriodictyol	Sample	15.053	11	ND		
22-18C-1.d	Eriodictyol	Sample	15.053	154	ND		
22-18C-2.d	Eriodictyol	Sample	15.045	171	ND		
22-18C-3.d	Eriodictyol	Sample	15.061	173	ND		

Quercetin

Data File	Compound	Sample Type	RT	Resp.	Final Conc	Exp. Conc	Accuracy
blank-061022-4.d	Quercetin	Sample	15.460	5	ND		
25ppb1.d	Quercetin	Calibration	15.668	14	ND		0.00
25ppb2.d	Quercetin	Calibration	16.120	2	ND		0.00
25ppb3.d	Quercetin	Calibration	15.502	290	5.5347		22.14
50ppb1.d	Quercetin	Calibration	15.553	954	34.5532		69.11
50ppb2.d	Quercetin	Calibration	15.553	1023	37.6091		75.22
50ppb3.d	Quercetin	Calibration	15.561	1025	37.6559		75.31
100ppb1.d	Quercetin	Calibration	15.578	2412	98.2920		98.29
100ppb2.d	Quercetin	Calibration	15.578	2527	103.3176		103.32
100ppb3.d	Quercetin	Calibration	15.578	2534	103.6220		103.62
250ppb1.d	Quercetin	Calibration	15.578	5693	241.7059		96.68
250ppb2.d	Quercetin	Calibration	15.578	5892	250.3854		100.15
250ppb3.d	Quercetin	Calibration	15.586	6108	259.8316		103.93
500ppb1.d	Quercetin	Calibration	15.586	12336	532.0472		106.41
500ppb2.d	Quercetin	Calibration	15.586	12240	527.8523		105.57
500ppb3.d	Quercetin	Calibration	15.594	12566	542.1020		108.42
1000ppb1.d	Quercetin	Calibration	15.586	23388	1015.1219		101.51
1000ppb2.d	Quercetin	Calibration	15.586	22272	966.3349		96.63
1000ppb3.d	Quercetin	Calibration	15.586	22333	969.0342		96.90
22-1A-1.d	Quercetin	Sample	15.586	90495	3948.2836		
22-1A-2.d	Quercetin	Sample	15.586	91119	3975.5559		
22-1A-3.d	Quercetin	Sample	15.586	92204	4022.9651		

Quercetin

Data File	Compound	Sample Type	RT	Resp.	Final Conc	Exp. Conc	Accuracy
22-2A-1.d	Quercetin	Sample	15.586	66563	2902.2297		
22-2A-2.d	Quercetin	Sample	15.586	65635	2861.6734		
22-2A-3.d	Quercetin	Sample	15.586	66250	2888.5627		
22-3A-1.d	Quercetin	Sample	15.578	6401	272.6616		
22-3A-2.d	Quercetin	Sample	15.586	5155	218.1918		
22-3A-3.d	Quercetin	Sample	15.594	4794	202.4264		
22-4A-1.d	Quercetin	Sample	15.586	9210	395.4476		
22-4A-2.d	Quercetin	Sample	15.586	8999	386.2148		
22-4A-3.d	Quercetin	Sample	15.578	8998	386.1518		
22-5A-1.d	Quercetin	Sample	15.578	3620	151.1055		
22-5A-2.d	Quercetin	Sample	15.578	3292	136.7505		
22-5A-3.d	Quercetin	Sample	15.578	3349	139.2396		
22-6A-1.d	Quercetin	Sample	15.620	802	27.9367		
22-6A-2.d	Quercetin	Sample	15.635	769	26.4685		
22-6A-3.d	Quercetin	Sample	15.603	812	28.3659		
22-7A-1.d	Quercetin	Sample	15.578	6967	297.3733		
22-7A-2.d	Quercetin	Sample	15.578	7356	314.3787		
22-7A-3.d	Quercetin	Sample	15.578	7428	317.5520		
22-8A-1.d	Quercetin	Sample	15.578	11157	480.5260		
22-8A-2.d	Quercetin	Sample	15.578	11040	475.4368		
22-8A-3.d	Quercetin	Sample	15.578	10506	452.0944		
22-9A-1.d	Quercetin	Sample	15.569	995	36.3751		
22-9A-2.d	Quercetin	Sample	15.586	705	23.7081		
22-9A-3.d	Quercetin	Sample	15.578	698	23.3735		
22-10A-1.d	Quercetin	Sample	15.578	890	31.7572		
22-10A-2.d	Quercetin	Sample	15.594	911	32.6829		
22-10A-3.d	Quercetin	Sample	15.578	851	30.0813		
22-11A-1.d	Quercetin	Sample	15.578	449	12.4845		
22-11A-2.d	Quercetin	Sample	15.578	387	9.7818		
22-11A-3.d	Quercetin	Sample	15.569	356	8.4215		
22-12A-1.d	Quercetin	Sample	15.594	85	ND		
22-12A-2.d	Quercetin	Sample	15.561	26	ND		
22-12A-3.d	Quercetin	Sample	15.594	44	ND		
22-13A-1.d	Quercetin	Sample	15.578	946	34.2116		
22-13A-2.d	Quercetin	Sample	15.578	962	34.9099		
22-13A-3.d	Quercetin	Sample	15.586	935	33.7577		
22-14A-1.d	Quercetin	Sample	15.586	126	ND		
22-14A-2.d	Quercetin	Sample	15.561	85	ND		
22-14A-3.d	Quercetin	Sample	15.569	89	ND		
22-15A-1.d	Quercetin	Sample	15.586	23	ND		

Quercetin

Data File	Compound	Sample Type	RT	Resp.	Final Conc	Exp. Conc	Accuracy
22-15A-2.d	Quercetin	Sample	15.553	9	ND		
22-15A-3.d	Quercetin	Sample	15.635	12	ND		
22-16A-1.d	Quercetin	Sample	15.611	26	ND		
22-16A-2.d	Quercetin	Sample	15.578	29	ND		
22-16A-3.d	Quercetin	Sample	15.578	32	ND		
22-17A-1.d	Quercetin	Sample	15.578	12	ND		
22-17A-2.d	Quercetin	Sample	15.553	17	ND		
22-17A-3.d	Quercetin	Sample	15.586	5	ND		
22-18A-1.d	Quercetin	Sample	15.561	257185	11234.0096		
22-18A-2.d	Quercetin	Sample	15.561	255232	11148.6613		
22-18A-3.d	Quercetin	Sample	15.561	250201	10928.7766		
22-1B-1.d	Quercetin	Sample	15.553	109279	4769.2759		
22-1B-2.d	Quercetin	Sample	15.553	106977	4668.6781		
22-1B-3.d	Quercetin	Sample	15.553	106117	4631.0606		
22-2B-1.d	Quercetin	Sample	15.553	87857	3832.9699		
22-2B-2.d	Quercetin	Sample	15.553	86009	3752.1937		
22-2B-3.d	Quercetin	Sample	15.561	86690	3781.9727		
22-3B-1.d	Quercetin	Sample	15.553	10871	468.0425		
22-3B-2.d	Quercetin	Sample	15.553	9300	399.3520		
22-3B-3.d	Quercetin	Sample	15.553	8881	381.0658		
22-4B-1.d	Quercetin	Sample	15.553	14578	630.0691		
22-4B-2.d	Quercetin	Sample	15.553	14979	647.5911		
22-4B-3.d	Quercetin	Sample	15.553	14305	618.1365		
22-5B-1.d	Quercetin	Sample	15.553	5177	219.1522		
22-5B-2.d	Quercetin	Sample	15.544	4673	197.1063		
22-5B-3.d	Quercetin	Sample	15.553	4667	196.8703		
22-6B-1.d	Quercetin	Sample	15.553	2198	88.9420		
22-6B-2.d	Quercetin	Sample	15.544	2116	85.3734		
22-6B-3.d	Quercetin	Sample	15.553	2157	87.1516		
22-7B-1.d	Quercetin	Sample	15.561	12091	521.3492		
22-7B-2.d	Quercetin	Sample	15.553	12601	543.6492		
22-7B-3.d	Quercetin	Sample	15.553	12604	543.7737		
22-8B-1.d	Quercetin	Sample	15.553	22222	964.1699		
22-8B-2.d	Quercetin	Sample	15.553	22014	955.0631		
22-8B-3.d	Quercetin	Sample	15.553	23013	998.7151		
22-9B-1.d	Quercetin	Sample	15.553	1627	63.9803		
22-9B-2.d	Quercetin	Sample	15.561	1092	40.6013		
22-9B-3.d	Quercetin	Sample	15.553	979	35.6776		
22-10B-1.d	Quercetin	Sample	15.553	1052	38.8711		
22-10B-2.d	Quercetin	Sample	15.553	961	34.8810		

Quercetin

Data File	Compound	Sample Type	RT	Resp.	Final Conc	Exp. Conc	Accuracy
22-10B-3.d	Quercetin	Sample	15.561	1013	37.1530		
22-11B-1.d	Quercetin	Sample	15.553	886	31.5948		
22-11B-2.d	Quercetin	Sample	15.544	761	26.1166		
22-11B-3.d	Quercetin	Sample	15.553	788	27.2962		
22-12B-1.d	Quercetin	Sample	15.561	547	16.7784		
22-12B-2.d	Quercetin	Sample	15.553	547	16.7830		
22-12B-3.d	Quercetin	Sample	15.553	497	14.6104		
22-13B-1.d	Quercetin	Sample	15.561	1439	55.7883		
22-13B-2.d	Quercetin	Sample	15.561	1413	54.6347		
22-13B-3.d	Quercetin	Sample	15.553	1408	54.4243		
22-14B-1.d	Quercetin	Sample	15.561	350	8.1904		
22-14B-2.d	Quercetin	Sample	15.544	292	5.6263		
22-14B-3.d	Quercetin	Sample	15.553	272	4.7671		
22-15B-1.d	Quercetin	Sample	15.569	104	ND		
22-15B-2.d	Quercetin	Sample	15.544	100	ND		
22-15B-3.d	Quercetin	Sample	15.544	64	ND		
22-16B-1.d	Quercetin	Sample	15.553	318	6.7561		
22-16B-2.d	Quercetin	Sample	15.553	325	7.0683		
22-16B-3.d	Quercetin	Sample	15.561	296	5.8186		
22-17B-1.d	Quercetin	Sample	15.578	70	ND		
22-17B-2.d	Quercetin	Sample	15.553	72	ND		
22-17B-3.d	Quercetin	Sample	15.561	55	ND		
22-18B-1.d	Quercetin	Sample	15.553	90557	3950.9827		
22-18B-2.d	Quercetin	Sample	15.553	100104	4368.2803		
22-18B-3.d	Quercetin	Sample	15.553	103639	4522.7522		
22-1C-1.d	Quercetin	Sample	15.544	96156	4195.7122		
22-1C-2.d	Quercetin	Sample	15.553	94613	4128.2476		
22-1C-3.d	Quercetin	Sample	15.553	98121	4281.5702		
22-2C-1.d	Quercetin	Sample	15.553	73524	3206.4958		
22-2C-2.d	Quercetin	Sample	15.553	72647	3168.1717		
22-2C-3.d	Quercetin	Sample	15.553	73277	3195.6983		
22-3C-1.d	Quercetin	Sample	15.561	7987	341.9700		
22-3C-2.d	Quercetin	Sample	15.553	6638	282.9900		
22-3C-3.d	Quercetin	Sample	15.553	6000	255.1064		
22-4C-1.d	Quercetin	Sample	15.544	10880	468.4218		
22-4C-2.d	Quercetin	Sample	15.553	10833	466.3823		
22-4C-3.d	Quercetin	Sample	15.553	11106	478.3093		
22-5C-1.d	Quercetin	Sample	15.561	3230	134.0481		
22-5C-2.d	Quercetin	Sample	15.553	3074	127.2535		
22-5C-3.d	Quercetin	Sample	15.553	3050	126.1795		

Quercetin

Data File	Compound	Sample Type	RT	Resp.	Final Conc	Exp. Conc	Accuracy
22-6C-1.d	Quercetin	Sample	15.561	1259	47.9183		
22-6C-2.d	Quercetin	Sample	15.578	1212	45.8433		
22-6C-3.d	Quercetin	Sample	15.561	1219	46.1771		
22-7C-1.d	Quercetin	Sample	15.553	8415	360.6906		
22-7C-2.d	Quercetin	Sample	15.553	8586	368.1723		
22-7C-3.d	Quercetin	Sample	15.553	8716	373.8528		
22-8C-1.d	Quercetin	Sample	15.553	12611	544.0598		
22-8C-2.d	Quercetin	Sample	15.553	11869	511.6513		
22-8C-3.d	Quercetin	Sample	15.561	12342	532.3159		
22-9C-1.d	Quercetin	Sample	15.561	1083	40.2006		
22-9C-2.d	Quercetin	Sample	15.553	819	28.6677		
22-9C-3.d	Quercetin	Sample	15.561	776	26.7788		
22-10C-1.d	Quercetin	Sample	15.561	952	34.5023		
22-10C-2.d	Quercetin	Sample	15.569	1011	37.0443		
22-10C-3.d	Quercetin	Sample	15.561	987	35.9941		
22-11C-1.d	Quercetin	Sample	15.561	579	18.1978		
22-11C-2.d	Quercetin	Sample	15.553	523	15.7285		
22-11C-3.d	Quercetin	Sample	15.561	558	17.2583		
22-12C-1.d	Quercetin	Sample	15.561	330	7.3199		
22-12C-2.d	Quercetin	Sample	15.553	343	7.8775		
22-12C-3.d	Quercetin	Sample	15.561	337	7.5894		
22-13C-1.d	Quercetin	Sample	15.561	1290	49.2490		
22-13C-2.d	Quercetin	Sample	15.569	1239	47.0394		
22-13C-3.d	Quercetin	Sample	15.553	1275	48.5984		
22-14C-1.d	Quercetin	Sample	15.553	143	ND		
22-14C-2.d	Quercetin	Sample	15.561	163	ND		
22-14C-3.d	Quercetin	Sample	15.569	149	ND		
22-15C-1.d	Quercetin	Sample	15.569	80	ND		
22-15C-2.d	Quercetin	Sample	15.561	90	ND		
22-15C-3.d	Quercetin	Sample	15.578	53	ND		
22-16C-1.d	Quercetin	Sample	15.553	65	ND		
22-16C-2.d	Quercetin	Sample	15.553	59	ND		
22-16C-3.d	Quercetin	Sample	15.569	58	ND		
22-17C-1.d	Quercetin	Sample	15.578	41	ND		
22-17C-2.d	Quercetin	Sample	15.561	51	ND		
22-17C-3.d	Quercetin	Sample	15.578	69	ND		
22-18C-1.d	Quercetin	Sample	15.569	755	25.8613		
22-18C-2.d	Quercetin	Sample	15.544	868	30.8065		
22-18C-3.d	Quercetin	Sample	15.561	816	28.5450		

Quantitative Analysis Complete Report

Luteolin

Data File	Compound	Sample Type	RT	Resp.	Final Conc	Exp. Conc	Accuracy
blank-061022-4.d	Luteolin	Sample	15.842	5	ND		
25ppb1.d	Luteolin	Calibration	15.758	11	ND		0.00
25ppb2.d	Luteolin	Calibration	15.808	15	ND		0.00
25ppb3.d	Luteolin	Calibration	15.733	178	6.2745		25.10
50ppb1.d	Luteolin	Calibration	15.800	512	27.3825		54.76
50ppb2.d	Luteolin	Calibration	15.791	633	34.9900		69.98
50ppb3.d	Luteolin	Calibration	15.808	659	36.6506		73.30
100ppb1.d	Luteolin	Calibration	15.816	1619	97.2974		97.30
100ppb2.d	Luteolin	Calibration	15.825	1760	106.2186		106.22
100ppb3.d	Luteolin	Calibration	15.816	1849	111.7955		111.80
250ppb1.d	Luteolin	Calibration	15.825	3905	241.7447		96.70
250ppb2.d	Luteolin	Calibration	15.808	3846	238.0102		95.20
250ppb3.d	Luteolin	Calibration	15.825	4280	265.4377		106.18
500ppb1.d	Luteolin	Calibration	15.825	8430	527.5986		105.52
500ppb2.d	Luteolin	Calibration	15.833	8691	544.1054		108.82
500ppb3.d	Luteolin	Calibration	15.833	8647	541.3173		108.26
1000ppb1.d	Luteolin	Calibration	15.825	15722	988.3047		98.83
1000ppb2.d	Luteolin	Calibration	15.825	15325	963.2163		96.32
1000ppb3.d	Luteolin	Calibration	15.825	15823	994.6560		99.47
22-1A-1.d	Luteolin	Sample	15.825	1145	67.3439		
22-1A-2.d	Luteolin	Sample	15.825	941	54.4576		
22-1A-3.d	Luteolin	Sample	15.816	945	54.7423		
22-2A-1.d	Luteolin	Sample	15.825	6773	422.9232		
22-2A-2.d	Luteolin	Sample	15.825	6554	409.0838		
22-2A-3.d	Luteolin	Sample	15.825	6861	428.4739		
22-3A-1.d	Luteolin	Sample	15.825	1631	98.0551		
22-3A-2.d	Luteolin	Sample	15.816	1564	93.8022		
22-3A-3.d	Luteolin	Sample	15.825	1574	94.4192		
22-4A-1.d	Luteolin	Sample	15.825	17393	1093.8224		
22-4A-2.d	Luteolin	Sample	15.825	17860	1123.3470		
22-4A-3.d	Luteolin	Sample	15.816	17778	1118.1583		
22-5A-1.d	Luteolin	Sample	15.825	23414	1474.2170		
22-5A-2.d	Luteolin	Sample	15.825	23419	1474.5450		
22-5A-3.d	Luteolin	Sample	15.816	23762	1496.2027		
22-6A-1.d	Luteolin	Sample	15.825	17639	1109.3625		
22-6A-2.d	Luteolin	Sample	15.825	17981	1131.0123		
22-6A-3.d	Luteolin	Sample	15.825	17640	1109.4839		
22-7A-1.d	Luteolin	Sample	15.825	759	42.9674		
22-7A-2.d	Luteolin	Sample	15.833	370	18.3797		
22-7A-3.d	Luteolin	Sample	15.816	344	16.7253		

Luteolin

Data File	Compound	Sample Type	RT	Resp.	Final Conc	Exp. Conc	Accuracy
22-8A-1.d	Luteolin	Sample	15.816	390	19.6650		
22-8A-2.d	Luteolin	Sample	15.816	310	14.5930		
22-8A-3.d	Luteolin	Sample	15.808	266	11.8252		
22-9A-1.d	Luteolin	Sample	15.816	341	16.5497		
22-9A-2.d	Luteolin	Sample	15.816	282	12.7977		
22-9A-3.d	Luteolin	Sample	15.816	283	12.8623		
22-10A-1.d	Luteolin	Sample	15.808	703	39.4448		
22-10A-2.d	Luteolin	Sample	15.816	683	38.1507		
22-10A-3.d	Luteolin	Sample	15.808	577	31.4903		
22-11A-1.d	Luteolin	Sample	15.816	956	55.3855		
22-11A-2.d	Luteolin	Sample	15.825	991	57.6292		
22-11A-3.d	Luteolin	Sample	15.825	946	54.7990		
22-12A-1.d	Luteolin	Sample	15.816	9095	569.6176		
22-12A-2.d	Luteolin	Sample	15.816	9440	591.4296		
22-12A-3.d	Luteolin	Sample	15.816	9610	602.1256		
22-13A-1.d	Luteolin	Sample	15.816	715	40.1570		
22-13A-2.d	Luteolin	Sample	15.816	614	33.8312		
22-13A-3.d	Luteolin	Sample	15.825	550	29.7746		
22-14A-1.d	Luteolin	Sample	15.816	10113	633.9133		
22-14A-2.d	Luteolin	Sample	15.816	10400	652.0197		
22-14A-3.d	Luteolin	Sample	15.825	10138	635.5158		
22-15A-1.d	Luteolin	Sample	15.825	20541	1292.7200		
22-15A-2.d	Luteolin	Sample	15.825	20896	1315.1641		
22-15A-3.d	Luteolin	Sample	15.825	20875	1313.8207		
22-16A-1.d	Luteolin	Sample	15.825	2999	184.4459		
22-16A-2.d	Luteolin	Sample	15.825	2718	166.7263		
22-16A-3.d	Luteolin	Sample	15.825	2576	157.7640		
22-17A-1.d	Luteolin	Sample	15.825	4053	251.0867		
22-17A-2.d	Luteolin	Sample	15.808	4042	250.3930		
22-17A-3.d	Luteolin	Sample	15.808	4088	253.2691		
22-18A-1.d	Luteolin	Sample	16.100	8111	507.4623		
22-18A-2.d	Luteolin	Sample	16.100	8207	513.5079		
22-18A-3.d	Luteolin	Sample	16.100	7849	490.8735		
22-1B-1.d	Luteolin	Sample	15.783	923	53.3429		
22-1B-2.d	Luteolin	Sample	15.791	1010	58.7940		
22-1B-3.d	Luteolin	Sample	15.791	1008	58.6861		
22-2B-1.d	Luteolin	Sample	15.791	8672	542.9092		
22-2B-2.d	Luteolin	Sample	15.791	8903	557.5012		
22-2B-3.d	Luteolin	Sample	15.791	8683	543.6032		
22-3B-1.d	Luteolin	Sample	15.791	2092	127.1785		

Luteolin

Data File	Compound	Sample Type	RT	Resp.	Final Conc	Exp. Conc	Accuracy
22-3B-2.d	Luteolin	Sample	15.791	2096	127.4253		
22-3B-3.d	Luteolin	Sample	15.791	2130	129.6050		
22-4B-1.d	Luteolin	Sample	15.791	23236	1462.9708		
22-4B-2.d	Luteolin	Sample	15.791	24141	1520.1417		
22-4B-3.d	Luteolin	Sample	15.791	24637	1551.4803		
22-5B-1.d	Luteolin	Sample	15.791	28430	1791.1026		
22-5B-2.d	Luteolin	Sample	15.791	28122	1771.6650		
22-5B-3.d	Luteolin	Sample	15.791	29510	1859.3434		
22-6B-1.d	Luteolin	Sample	15.791	24377	1535.0731		
22-6B-2.d	Luteolin	Sample	15.791	24551	1546.0877		
22-6B-3.d	Luteolin	Sample	15.791	24918	1569.2861		
22-7B-1.d	Luteolin	Sample	15.800	830	47.4619		
22-7B-2.d	Luteolin	Sample	15.800	451	23.5054		
22-7B-3.d	Luteolin	Sample	15.800	370	18.3724		
22-8B-1.d	Luteolin	Sample	15.791	429	22.1015		
22-8B-2.d	Luteolin	Sample	15.791	408	20.7611		
22-8B-3.d	Luteolin	Sample	15.791	362	17.8476		
22-9B-1.d	Luteolin	Sample	15.791	406	20.6686		
22-9B-2.d	Luteolin	Sample	15.791	363	17.9396		
22-9B-3.d	Luteolin	Sample	15.800	348	17.0127		
22-10B-1.d	Luteolin	Sample	15.791	792	45.0245		
22-10B-2.d	Luteolin	Sample	15.791	657	36.5281		
22-10B-3.d	Luteolin	Sample	15.791	703	39.4233		
22-11B-1.d	Luteolin	Sample	15.791	1145	67.3437		
22-11B-2.d	Luteolin	Sample	15.791	1195	70.4940		
22-11B-3.d	Luteolin	Sample	15.800	1169	68.8660		
22-12B-1.d	Luteolin	Sample	15.791	11852	743.8094		
22-12B-2.d	Luteolin	Sample	15.791	12461	782.2859		
22-12B-3.d	Luteolin	Sample	15.800	12103	759.6501		
22-13B-1.d	Luteolin	Sample	15.800	862	49.4982		
22-13B-2.d	Luteolin	Sample	15.791	735	41.4133		
22-13B-3.d	Luteolin	Sample	15.791	704	39.5020		
22-14B-1.d	Luteolin	Sample	15.800	12563	788.7336		
22-14B-2.d	Luteolin	Sample	15.800	12982	815.1928		
22-14B-3.d	Luteolin	Sample	15.800	13000	816.3297		
22-15B-1.d	Luteolin	Sample	15.800	18931	1191.0294		
22-15B-2.d	Luteolin	Sample	15.791	19162	1205.5929		
22-15B-3.d	Luteolin	Sample	15.791	19274	1212.7005		
22-16B-1.d	Luteolin	Sample	15.791	3083	189.7594		
22-16B-2.d	Luteolin	Sample	15.800	2865	176.0283		

Luteolin

Data File	Compound	Sample Type	RT	Resp.	Final Conc	Exp. Conc	Accuracy
22-16B-3.d	Luteolin	Sample	15.800	2836	174.1508		
22-17B-1.d	Luteolin	Sample	15.800	5671	353.2786		
22-17B-2.d	Luteolin	Sample	15.791	5637	351.1638		
22-17B-3.d	Luteolin	Sample	15.800	5496	342.2287		
22-18B-1.d	Luteolin	Sample	15.791	105	1.6121		
22-18B-2.d	Luteolin	Sample	15.783	103	1.5246		
22-18B-3.d	Luteolin	Sample	15.791	96	1.0939		
22-1C-1.d	Luteolin	Sample	15.791	1035	60.3996		
22-1C-2.d	Luteolin	Sample	15.791	975	56.6262		
22-1C-3.d	Luteolin	Sample	15.791	1050	61.3591		
22-2C-1.d	Luteolin	Sample	15.791	7796	487.5184		
22-2C-2.d	Luteolin	Sample	15.791	7860	491.5956		
22-2C-3.d	Luteolin	Sample	15.791	8044	503.1858		
22-3C-1.d	Luteolin	Sample	15.791	1846	111.6194		
22-3C-2.d	Luteolin	Sample	15.791	1969	119.4207		
22-3C-3.d	Luteolin	Sample	15.783	2039	123.8035		
22-4C-1.d	Luteolin	Sample	15.791	17821	1120.8933		
22-4C-2.d	Luteolin	Sample	15.791	17674	1111.6265		
22-4C-3.d	Luteolin	Sample	15.791	17944	1128.6473		
22-5C-1.d	Luteolin	Sample	15.791	19719	1240.8262		
22-5C-2.d	Luteolin	Sample	15.791	19598	1233.1837		
22-5C-3.d	Luteolin	Sample	15.791	20262	1275.0751		
22-6C-1.d	Luteolin	Sample	15.791	18226	1146.4844		
22-6C-2.d	Luteolin	Sample	15.791	19035	1197.5861		
22-6C-3.d	Luteolin	Sample	15.791	18608	1170.5913		
22-7C-1.d	Luteolin	Sample	15.800	699	39.1906		
22-7C-2.d	Luteolin	Sample	15.791	319	15.1807		
22-7C-3.d	Luteolin	Sample	15.800	237	10.0026		
22-8C-1.d	Luteolin	Sample	15.800	326	15.6170		
22-8C-2.d	Luteolin	Sample	15.783	335	16.1866		
22-8C-3.d	Luteolin	Sample	15.800	266	11.8199		
22-9C-1.d	Luteolin	Sample	15.791	321	15.2606		
22-9C-2.d	Luteolin	Sample	15.800	340	16.5029		
22-9C-3.d	Luteolin	Sample	15.800	343	16.6686		
22-10C-1.d	Luteolin	Sample	15.791	714	40.1019		
22-10C-2.d	Luteolin	Sample	15.791	707	39.6622		
22-10C-3.d	Luteolin	Sample	15.800	752	42.4917		
22-11C-1.d	Luteolin	Sample	15.808	1104	64.7813		
22-11C-2.d	Luteolin	Sample	15.800	1068	62.4931		
22-11C-3.d	Luteolin	Sample	15.800	1245	73.6795		

Luteolin

Data File	Compound	Sample Type	RT	Resp.	Final Conc	Exp. Conc	Accuracy
22-12C-1.d	Luteolin	Sample	15.800	9695	607.5313		
22-12C-2.d	Luteolin	Sample	15.800	10007	627.2019		
22-12C-3.d	Luteolin	Sample	15.800	9826	615.7958		
22-13C-1.d	Luteolin	Sample	15.808	783	44.4842		
22-13C-2.d	Luteolin	Sample	15.800	655	36.4119		
22-13C-3.d	Luteolin	Sample	15.791	589	32.2300		
22-14C-1.d	Luteolin	Sample	15.800	8877	555.8528		
22-14C-2.d	Luteolin	Sample	15.800	8935	559.5208		
22-14C-3.d	Luteolin	Sample	15.800	8888	556.5323		
22-15C-1.d	Luteolin	Sample	15.800	17156	1078.8880		
22-15C-2.d	Luteolin	Sample	15.800	17077	1073.8532		
22-15C-3.d	Luteolin	Sample	15.800	17300	1088.0007		
22-16C-1.d	Luteolin	Sample	15.800	2620	160.5637		
22-16C-2.d	Luteolin	Sample	15.808	2199	133.9273		
22-16C-3.d	Luteolin	Sample	15.808	2079	126.3717		
22-17C-1.d	Luteolin	Sample	15.800	4097	253.8274		
22-17C-2.d	Luteolin	Sample	15.808	4249	263.4697		
22-17C-3.d	Luteolin	Sample	15.808	4168	258.3397		
22-18C-1.d	Luteolin	Sample	15.808	70	ND		
22-18C-2.d	Luteolin	Sample	15.800	52	ND		
22-18C-3.d	Luteolin	Sample	15.791	72	ND		

Kaempferol

Data File	Compound	Sample Type	RT	Resp.	Final Conc	Exp. Conc	Accuracy
blank-061022-4.d	Kaempferol	Sample	15.779	120	ND		
25ppb1.d	Kaempferol	Calibration	15.821	222	21.9577		87.83
25ppb2.d	Kaempferol	Calibration	15.796	186	13.1101		52.44
25ppb3.d	Kaempferol	Calibration	16.046	98	ND		0.00
50ppb1.d	Kaempferol	Calibration	16.088	224	22.6002		45.20
50ppb2.d	Kaempferol	Calibration	16.113	240	26.6800		53.36
50ppb3.d	Kaempferol	Calibration	16.113	272	34.5596		69.12
100ppb1.d	Kaempferol	Calibration	16.121	573	109.8097		109.81
100ppb2.d	Kaempferol	Calibration	16.121	594	115.0103		115.01
100ppb3.d	Kaempferol	Calibration	16.105	580	111.4005		111.40
250ppb1.d	Kaempferol	Calibration	16.130	1210	268.8920		107.56
250ppb2.d	Kaempferol	Calibration	16.121	1107	243.0488		97.22
250ppb3.d	Kaempferol	Calibration	16.113	1138	250.8880		100.36
500ppb1.d	Kaempferol	Calibration	16.121	2318	545.5338		109.11
500ppb2.d	Kaempferol	Calibration	16.130	2325	547.3324		109.47
500ppb3.d	Kaempferol	Calibration	16.121	2193	514.3050		102.86
1000ppb1.d	Kaempferol	Calibration	16.121	3962	955.9408		95.59

Kaempferol

Data File	Compound	Sample Type	RT	Resp.	Final Conc	Exp. Conc	Accuracy
1000ppb2.d	Kaempferol	Calibration	16.130	3807	917.4037		91.74
1000ppb3.d	Kaempferol	Calibration	16.121	4089	987.7973		98.78
22-1A-1.d	Kaempferol	Sample	16.113	1397	315.5101		
22-1A-2.d	Kaempferol	Sample	16.121	1408	318.3060		
22-1A-3.d	Kaempferol	Sample	16.121	1278	285.7029		
22-2A-1.d	Kaempferol	Sample	16.254	389	63.8702		
22-2A-2.d	Kaempferol	Sample	16.254	446	78.0932		
22-2A-3.d	Kaempferol	Sample	16.262	387	63.1922		
22-3A-1.d	Kaempferol	Sample	16.121	3278	785.2949		
22-3A-2.d	Kaempferol	Sample	16.121	3199	765.4004		
22-3A-3.d	Kaempferol	Sample	16.130	3561	855.9496		
22-4A-1.d	Kaempferol	Sample	16.113	2408	567.9501		
22-4A-2.d	Kaempferol	Sample	16.113	2338	550.5825		
22-4A-3.d	Kaempferol	Sample	16.121	2239	525.7765		
22-5A-1.d	Kaempferol	Sample	16.271	3310	793.2407		
22-5A-2.d	Kaempferol	Sample	16.262	3303	791.6080		
22-5A-3.d	Kaempferol	Sample	16.254	3283	786.3778		
22-6A-1.d	Kaempferol	Sample	16.121	322	46.9456		
22-6A-2.d	Kaempferol	Sample	16.113	300	41.5558		
22-6A-3.d	Kaempferol	Sample	16.105	289	38.8462		
22-7A-1.d	Kaempferol	Sample	16.113	478	85.9817		
22-7A-2.d	Kaempferol	Sample	16.130	448	78.5498		
22-7A-3.d	Kaempferol	Sample	16.105	529	98.6994		
22-8A-1.d	Kaempferol	Sample	16.121	294	40.0981		
22-8A-2.d	Kaempferol	Sample	16.130	287	38.3477		
22-8A-3.d	Kaempferol	Sample	16.105	362	56.9119		
22-9A-1.d	Kaempferol	Sample	16.138	22	ND		
22-9A-2.d	Kaempferol	Sample	16.180	11	ND		
22-9A-3.d	Kaempferol	Sample	16.138	6	ND		
22-10A-1.d	Kaempferol	Sample	16.105	63	ND		
22-10A-2.d	Kaempferol	Sample	16.147	40	ND		
22-10A-3.d	Kaempferol	Sample	16.130	63	ND		
22-11A-1.d	Kaempferol	Sample	16.088	47	ND		
22-11A-2.d	Kaempferol	Sample	16.121	26	ND		
22-11A-3.d	Kaempferol	Sample	16.130	25	ND		
22-12A-1.d	Kaempferol	Sample	16.096	95	ND		
22-12A-2.d	Kaempferol	Sample	16.088	129	ND		
22-12A-3.d	Kaempferol	Sample	16.147	100	ND		
22-13A-1.d	Kaempferol	Sample	16.121	272	34.6219		
22-13A-2.d	Kaempferol	Sample	16.113	254	30.1846		

Quantitative Analysis Complete Report

Agilent Technologies

Kaempferol

Data File	Compound	Sample Type	RT	Resp.	Final Conc	Exp. Conc	Accuracy
22-13A-3.d	Kaempferol	Sample	16.113	244	27.5796		
22-14A-1.d	Kaempferol	Sample	16.121	54	ND		
22-14A-2.d	Kaempferol	Sample	16.138	40	ND		
22-14A-3.d	Kaempferol	Sample	16.121	88	ND		
22-15A-1.d	Kaempferol	Sample	16.155	43	ND		
22-15A-2.d	Kaempferol	Sample	16.130	48	ND		
22-15A-3.d	Kaempferol	Sample	16.105	38	ND		
22-16A-1.d	Kaempferol	Sample	16.121	281	36.7497		
22-16A-2.d	Kaempferol	Sample	16.121	303	42.2108		
22-16A-3.d	Kaempferol	Sample	16.121	288	38.4986		
22-17A-1.d	Kaempferol	Sample	16.138	19	ND		
22-17A-2.d	Kaempferol	Sample	16.121	16	ND		
22-17A-3.d	Kaempferol	Sample	16.130	26	ND		
22-18A-1.d	Kaempferol	Sample	16.096	2996	714.8884		
22-18A-2.d	Kaempferol	Sample	16.105	3334	799.3356		
22-18A-3.d	Kaempferol	Sample	16.096	3105	741.9812		
22-1B-1.d	Kaempferol	Sample	16.080	1436	325.3128		
22-1B-2.d	Kaempferol	Sample	16.088	1396	315.3233		
22-1B-3.d	Kaempferol	Sample	16.088	1404	317.3758		
22-2B-1.d	Kaempferol	Sample	16.220	429	73.8515		
22-2B-2.d	Kaempferol	Sample	16.220	439	76.2150		
22-2B-3.d	Kaempferol	Sample	16.220	475	85.2115		
22-3B-1.d	Kaempferol	Sample	16.096	3316	794.8027		
22-3B-2.d	Kaempferol	Sample	16.096	3464	831.7150		
22-3B-3.d	Kaempferol	Sample	16.096	3445	826.8891		
22-4B-1.d	Kaempferol	Sample	16.088	3452	828.7320		
22-4B-2.d	Kaempferol	Sample	16.088	3411	818.5726		
22-4B-3.d	Kaempferol	Sample	16.088	3427	822.5341		
22-5B-1.d	Kaempferol	Sample	16.237	3655	879.4753		
22-5B-2.d	Kaempferol	Sample	16.229	3751	903.2605		
22-5B-3.d	Kaempferol	Sample	16.245	3529	847.8750		
22-6B-1.d	Kaempferol	Sample	16.088	381	61.9043		
22-6B-2.d	Kaempferol	Sample	16.096	401	66.7457		
22-6B-3.d	Kaempferol	Sample	16.096	392	64.4519		
22-7B-1.d	Kaempferol	Sample	16.088	560	106.5212		
22-7B-2.d	Kaempferol	Sample	16.096	583	112.3115		
22-7B-3.d	Kaempferol	Sample	16.088	615	120.1965		
22-8B-1.d	Kaempferol	Sample	16.096	431	74.3155		
22-8B-2.d	Kaempferol	Sample	16.088	402	66.9689		
22-8B-3.d	Kaempferol	Sample	16.080	424	72.6275		

Kaempferol

Data File	Compound	Sample Type	RT	Resp.	Final Conc	Exp. Conc	Accuracy
22-9B-1.d	Kaempferol	Sample	16.105	25	ND		
22-9B-2.d	Kaempferol	Sample	16.071	17	ND		
22-9B-3.d	Kaempferol	Sample	15.787	104	ND		
22-10B-1.d	Kaempferol	Sample	16.071	61	ND		
22-10B-2.d	Kaempferol	Sample	16.088	63	ND		
22-10B-3.d	Kaempferol	Sample	16.105	52	ND		
22-11B-1.d	Kaempferol	Sample	16.080	49	ND		
22-11B-2.d	Kaempferol	Sample	16.071	33	ND		
22-11B-3.d	Kaempferol	Sample	16.105	43	ND		
22-12B-1.d	Kaempferol	Sample	16.088	198	16.0237		
22-12B-2.d	Kaempferol	Sample	16.113	224	22.6862		
22-12B-3.d	Kaempferol	Sample	16.080	240	26.5341		
22-13B-1.d	Kaempferol	Sample	16.096	239	26.3934		
22-13B-2.d	Kaempferol	Sample	16.088	249	28.8037		
22-13B-3.d	Kaempferol	Sample	16.113	297	40.7454		
22-14B-1.d	Kaempferol	Sample	16.088	88	ND		
22-14B-2.d	Kaempferol	Sample	16.096	91	ND		
22-14B-3.d	Kaempferol	Sample	16.088	64	ND		
22-15B-1.d	Kaempferol	Sample	16.096	43	ND		
22-15B-2.d	Kaempferol	Sample	16.096	52	ND		
22-15B-3.d	Kaempferol	Sample	16.071	65	ND		
22-16B-1.d	Kaempferol	Sample	16.096	315	45.2498		
22-16B-2.d	Kaempferol	Sample	16.113	303	42.3239		
22-16B-3.d	Kaempferol	Sample	16.088	318	46.1525		
22-17B-1.d	Kaempferol	Sample	16.096	67	ND		
22-17B-2.d	Kaempferol	Sample	16.096	71	ND		
22-17B-3.d	Kaempferol	Sample	16.130	45	ND		
22-18B-1.d	Kaempferol	Sample	16.080	819	171.1956		
22-18B-2.d	Kaempferol	Sample	16.096	881	186.6232		
22-18B-3.d	Kaempferol	Sample	16.088	824	172.3376		
22-1C-1.d	Kaempferol	Sample	16.088	1466	332.7536		
22-1C-2.d	Kaempferol	Sample	16.088	1422	321.6264		
22-1C-3.d	Kaempferol	Sample	16.088	1496	340.2373		
22-2C-1.d	Kaempferol	Sample	16.220	435	75.3470		
22-2C-2.d	Kaempferol	Sample	16.212	457	80.6673		
22-2C-3.d	Kaempferol	Sample	16.220	487	88.2622		
22-3C-1.d	Kaempferol	Sample	16.088	3691	888.4557		
22-3C-2.d	Kaempferol	Sample	16.096	3605	866.9086		
22-3C-3.d	Kaempferol	Sample	16.088	3520	845.7349		
22-4C-1.d	Kaempferol	Sample	16.088	2746	652.3394		

Kaempferol

Data File	Compound	Sample Type	RT	Resp.	Final Conc	Exp. Conc	Accuracy
22-4C-2.d	Kaempferol	Sample	16.088	2660	630.8498		
22-4C-3.d	Kaempferol	Sample	16.088	2645	627.2147		
22-5C-1.d	Kaempferol	Sample	16.237	3522	846.2518		
22-5C-2.d	Kaempferol	Sample	16.237	3664	881.6171		
22-5C-3.d	Kaempferol	Sample	16.237	3487	837.5352		
22-6C-1.d	Kaempferol	Sample	16.088	315	45.1899		
22-6C-2.d	Kaempferol	Sample	16.088	306	43.1304		
22-6C-3.d	Kaempferol	Sample	16.071	270	34.1809		
22-7C-1.d	Kaempferol	Sample	16.088	452	79.5643		
22-7C-2.d	Kaempferol	Sample	16.088	403	67.2510		
22-7C-3.d	Kaempferol	Sample	16.080	420	71.5404		
22-8C-1.d	Kaempferol	Sample	16.096	302	41.9954		
22-8C-2.d	Kaempferol	Sample	16.096	310	44.1218		
22-8C-3.d	Kaempferol	Sample	16.096	276	35.6391		
22-9C-1.d	Kaempferol	Sample	16.138	24	ND		
22-9C-2.d	Kaempferol	Sample	16.088	10	ND		
22-9C-3.d	Kaempferol	Sample	16.113	17	ND		
22-10C-1.d	Kaempferol	Sample	16.055	61	ND		
22-10C-2.d	Kaempferol	Sample	16.113	60	ND		
22-10C-3.d	Kaempferol	Sample	16.096	86	ND		
22-11C-1.d	Kaempferol	Sample	16.105	22	ND		
22-11C-2.d	Kaempferol	Sample	16.096	30	ND		
22-11C-3.d	Kaempferol	Sample	16.105	17	ND		
22-12C-1.d	Kaempferol	Sample	16.080	221	21.6989		
22-12C-2.d	Kaempferol	Sample	16.096	177	10.8379		
22-12C-3.d	Kaempferol	Sample	16.105	191	14.3660		
22-13C-1.d	Kaempferol	Sample	16.096	293	39.7458		
22-13C-2.d	Kaempferol	Sample	16.105	315	45.1867		
22-13C-3.d	Kaempferol	Sample	16.096	339	51.2586		
22-14C-1.d	Kaempferol	Sample	16.096	32	ND		
22-14C-2.d	Kaempferol	Sample	16.088	52	ND		
22-14C-3.d	Kaempferol	Sample	16.096	67	ND		
22-15C-1.d	Kaempferol	Sample	16.080	18	ND		
22-15C-2.d	Kaempferol	Sample	16.096	10	ND		
22-15C-3.d	Kaempferol	Sample	16.088	28	ND		
22-16C-1.d	Kaempferol	Sample	16.105	245	27.7336		
22-16C-2.d	Kaempferol	Sample	16.105	235	25.3452		
22-16C-3.d	Kaempferol	Sample	16.105	228	23.6795		
22-17C-1.d	Kaempferol	Sample	16.088	60	ND		
22-17C-2.d	Kaempferol	Sample	16.080	49	ND		

Agilent Technologies

Kaempferol

Data File	Compound	Sample Type	RT	Resp.	Final Conc	Exp. Conc	Accuracy
22-17C-3.d	Kaempferol	Sample	16.105	40	ND		
22-18C-1.d	Kaempferol	Sample	16.080	54	ND		
22-18C-2.d	Kaempferol	Sample	15.854	111	ND		
22-18C-3.d	Kaempferol	Sample	16.071	83	ND		

Apigenin

Data File	Compound	Sample Type	RT	Resp.	Final Conc	Exp. Conc	Accuracy
blank-061022-4.d	Apigenin	Sample	16.260	68	ND		
25ppb1.d	Apigenin	Calibration	16.302	48	ND		0.00
25ppb2.d	Apigenin	Calibration	16.001	5	ND		0.00
25ppb3.d	Apigenin	Calibration	16.193	177	3.6625		14.65
50ppb1.d	Apigenin	Calibration	16.226	644	28.9204		57.84
50ppb2.d	Apigenin	Calibration	16.243	747	34.5354		69.07
50ppb3.d	Apigenin	Calibration	16.235	816	38.2329		76.47
100ppb1.d	Apigenin	Calibration	16.251	1895	96.6902		96.69
100ppb2.d	Apigenin	Calibration	16.260	1904	97.1300		97.13
100ppb3.d	Apigenin	Calibration	16.251	2155	110.7457		110.75
250ppb1.d	Apigenin	Calibration	16.251	4746	251.0300		100.41
250ppb2.d	Apigenin	Calibration	16.260	4753	251.3925		100.56
250ppb3.d	Apigenin	Calibration	16.260	4803	254.0694		101.63
500ppb1.d	Apigenin	Calibration	16.260	9884	529.1451		105.83
500ppb2.d	Apigenin	Calibration	16.260	10152	543.6772		108.74
500ppb3.d	Apigenin	Calibration	16.260	10133	542.6208		108.52
1000ppb1.d	Apigenin	Calibration	16.260	18564	999.0581		99.91
1000ppb2.d	Apigenin	Calibration	16.260	18232	981.0949		98.11
1000ppb3.d	Apigenin	Calibration	16.260	17898	962.9949		96.30
22-1A-1.d	Apigenin	Sample	16.260	2228	114.7152		
22-1A-2.d	Apigenin	Sample	16.251	2190	112.6559		
22-1A-3.d	Apigenin	Sample	16.260	2204	113.3874		
22-2A-1.d	Apigenin	Sample	16.251	1617	81.6399		
22-2A-2.d	Apigenin	Sample	16.260	1639	82.8152		
22-2A-3.d	Apigenin	Sample	16.260	1710	86.6668		
22-3A-1.d	Apigenin	Sample	16.260	4483	236.7516		
22-3A-2.d	Apigenin	Sample	16.260	4445	234.7015		
22-3A-3.d	Apigenin	Sample	16.260	4776	252.6236		
22-4A-1.d	Apigenin	Sample	16.251	12915	693.2326		
22-4A-2.d	Apigenin	Sample	16.243	12592	675.7579		
22-4A-3.d	Apigenin	Sample	16.251	12813	687.7019		
22-5A-1.d	Apigenin	Sample	16.243	82275	4448.1481		
22-5A-2.d	Apigenin	Sample	16.243	81927	4429.3198		
22-5A-3.d	Apigenin	Sample	16.235	80522	4353.2454		

Apigenin

Data File	Compound	Sample Type	RT	Resp.	Final Conc	Exp. Conc	Accuracy
22-6A-1.d	Apigenin	Sample	16.251	10296	551.4870		
22-6A-2.d	Apigenin	Sample	16.251	9521	509.5350		
22-6A-3.d	Apigenin	Sample	16.251	9576	512.4888		
22-7A-1.d	Apigenin	Sample	16.251	323	11.5600		
22-7A-2.d	Apigenin	Sample	16.251	223	6.1337		
22-7A-3.d	Apigenin	Sample	16.260	193	4.5380		
22-8A-1.d	Apigenin	Sample	16.251	227	6.3720		
22-8A-2.d	Apigenin	Sample	16.251	241	7.1383		
22-8A-3.d	Apigenin	Sample	16.251	228	6.4437		
22-9A-1.d	Apigenin	Sample	16.268	282	9.3482		
22-9A-2.d	Apigenin	Sample	16.268	257	8.0070		
22-9A-3.d	Apigenin	Sample	16.251	264	8.3654		
22-10A-1.d	Apigenin	Sample	16.285	64	ND		
22-10A-2.d	Apigenin	Sample	16.268	107	ND		
22-10A-3.d	Apigenin	Sample	16.235	95	ND		
22-11A-1.d	Apigenin	Sample	16.251	6581	350.3605		
22-11A-2.d	Apigenin	Sample	16.251	6638	353.4110		
22-11A-3.d	Apigenin	Sample	16.251	6647	353.9115		
22-12A-1.d	Apigenin	Sample	16.251	8365	446.9165		
22-12A-2.d	Apigenin	Sample	16.243	8403	448.9836		
22-12A-3.d	Apigenin	Sample	16.243	8334	445.2325		
22-13A-1.d	Apigenin	Sample	16.251	7251	386.6265		
22-13A-2.d	Apigenin	Sample	16.243	7348	391.8562		
22-13A-3.d	Apigenin	Sample	16.251	7381	393.6820		
22-14A-1.d	Apigenin	Sample	16.251	16147	868.2189		
22-14A-2.d	Apigenin	Sample	16.251	16091	865.2021		
22-14A-3.d	Apigenin	Sample	16.251	16084	864.8168		
22-15A-1.d	Apigenin	Sample	16.251	9842	526.8651		
22-15A-2.d	Apigenin	Sample	16.251	9665	517.2847		
22-15A-3.d	Apigenin	Sample	16.251	9965	533.5579		
22-16A-1.d	Apigenin	Sample	16.251	4083	215.1172		
22-16A-2.d	Apigenin	Sample	16.260	4064	214.0767		
22-16A-3.d	Apigenin	Sample	16.260	4001	210.6820		
22-17A-1.d	Apigenin	Sample	16.251	984	47.3285		
22-17A-2.d	Apigenin	Sample	16.243	963	46.2134		
22-17A-3.d	Apigenin	Sample	16.243	938	44.8411		
22-18A-1.d	Apigenin	Sample	16.235	71	ND		
22-18A-2.d	Apigenin	Sample	16.243	76	ND		
22-18A-3.d	Apigenin	Sample	16.210	85	ND		
22-1B-1.d	Apigenin	Sample	16.226	2388	123.3629		

Apigenin

Data File	Compound	Sample Type	RT	Resp.	Final Conc	Exp. Conc	Accuracy
22-1B-2.d	Apigenin	Sample	16.235	2292	118.1726		
22-1B-3.d	Apigenin	Sample	16.226	2500	129.3934		
22-2B-1.d	Apigenin	Sample	16.226	2013	103.0317		
22-2B-2.d	Apigenin	Sample	16.218	2039	104.4704		
22-2B-3.d	Apigenin	Sample	16.218	1982	101.3521		
22-3B-1.d	Apigenin	Sample	16.226	5899	313.4539		
22-3B-2.d	Apigenin	Sample	16.226	5627	298.7227		
22-3B-3.d	Apigenin	Sample	16.226	5821	309.2203		
22-4B-1.d	Apigenin	Sample	16.218	15026	807.5294		
22-4B-2.d	Apigenin	Sample	16.218	15623	839.8449		
22-4B-3.d	Apigenin	Sample	16.218	15229	818.5183		
22-5B-1.d	Apigenin	Sample	16.210	91264	4934.7764		
22-5B-2.d	Apigenin	Sample	16.210	91758	4961.5196		
22-5B-3.d	Apigenin	Sample	16.210	91258	4934.4607		
22-6B-1.d	Apigenin	Sample	16.218	12952	695.2498		
22-6B-2.d	Apigenin	Sample	16.218	12098	649.0064		
22-6B-3.d	Apigenin	Sample	16.218	11706	627.8092		
22-7B-1.d	Apigenin	Sample	16.235	427	17.2171		
22-7B-2.d	Apigenin	Sample	16.243	286	9.5528		
22-7B-3.d	Apigenin	Sample	16.226	245	7.3691		
22-8B-1.d	Apigenin	Sample	16.226	460	18.9907		
22-8B-2.d	Apigenin	Sample	16.243	447	18.2673		
22-8B-3.d	Apigenin	Sample	16.226	373	14.2907		
22-9B-1.d	Apigenin	Sample	16.218	324	11.5928		
22-9B-2.d	Apigenin	Sample	16.226	282	9.3487		
22-9B-3.d	Apigenin	Sample	16.226	206	5.2528		
22-10B-1.d	Apigenin	Sample	16.218	68	ND		
22-10B-2.d	Apigenin	Sample	16.243	75	ND		
22-10B-3.d	Apigenin	Sample	16.218	69	ND		
22-11B-1.d	Apigenin	Sample	16.226	7956	424.7908		
22-11B-2.d	Apigenin	Sample	16.226	7850	419.0389		
22-11B-3.d	Apigenin	Sample	16.226	8110	433.1099		
22-12B-1.d	Apigenin	Sample	16.226	10130	542.4847		
22-12B-2.d	Apigenin	Sample	16.218	10390	556.5279		
22-12B-3.d	Apigenin	Sample	16.226	9960	533.2772		
22-13B-1.d	Apigenin	Sample	16.226	8329	444.9833		
22-13B-2.d	Apigenin	Sample	16.226	8007	427.5564		
22-13B-3.d	Apigenin	Sample	16.226	8337	445.4014		
22-14B-1.d	Apigenin	Sample	16.226	19599	1055.0830		
22-14B-2.d	Apigenin	Sample	16.226	19763	1063.9574		

Apigenin

Data File	Compound	Sample Type	RT	Resp.	Final Conc	Exp. Conc	Accuracy
22-14B-3.d	Apigenin	Sample	16.226	19758	1063.6941		
22-15B-1.d	Apigenin	Sample	16.226	9343	499.8910		
22-15B-2.d	Apigenin	Sample	16.226	9394	502.6294		
22-15B-3.d	Apigenin	Sample	16.226	9272	496.0243		
22-16B-1.d	Apigenin	Sample	16.226	4359	230.0533		
22-16B-2.d	Apigenin	Sample	16.226	4338	228.9316		
22-16B-3.d	Apigenin	Sample	16.226	4290	226.3027		
22-17B-1.d	Apigenin	Sample	16.235	1256	62.0626		
22-17B-2.d	Apigenin	Sample	16.226	1157	56.7271		
22-17B-3.d	Apigenin	Sample	16.226	1194	58.7057		
22-18B-1.d	Apigenin	Sample	16.235	66	ND		
22-18B-2.d	Apigenin	Sample	16.235	43	ND		
22-18B-3.d	Apigenin	Sample	16.226	51	ND		
22-1C-1.d	Apigenin	Sample	16.226	2350	121.3133		
22-1C-2.d	Apigenin	Sample	16.218	2407	124.4032		
22-1C-3.d	Apigenin	Sample	16.226	2455	126.9787		
22-2C-1.d	Apigenin	Sample	16.226	1703	86.2882		
22-2C-2.d	Apigenin	Sample	16.218	1686	85.3336		
22-2C-3.d	Apigenin	Sample	16.218	1661	84.0134		
22-3C-1.d	Apigenin	Sample	16.226	4770	252.2896		
22-3C-2.d	Apigenin	Sample	16.226	4955	262.3274		
22-3C-3.d	Apigenin	Sample	16.226	5111	270.7790		
22-4C-1.d	Apigenin	Sample	16.218	13280	713.0039		
22-4C-2.d	Apigenin	Sample	16.218	14130	759.0171		
22-4C-3.d	Apigenin	Sample	16.218	13574	728.9248		
22-5C-1.d	Apigenin	Sample	16.210	69432	3752.8837		
22-5C-2.d	Apigenin	Sample	16.210	68386	3696.2572		
22-5C-3.d	Apigenin	Sample	16.210	70138	3791.1068		
22-6C-1.d	Apigenin	Sample	16.218	10252	549.0985		
22-6C-2.d	Apigenin	Sample	16.218	10128	542.3489		
22-6C-3.d	Apigenin	Sample	16.218	10136	542.8084		
22-7C-1.d	Apigenin	Sample	16.226	343	12.6365		
22-7C-2.d	Apigenin	Sample	16.226	249	7.5860		
22-7C-3.d	Apigenin	Sample	16.235	208	5.3287		
22-8C-1.d	Apigenin	Sample	16.226	211	5.5077		
22-8C-2.d	Apigenin	Sample	16.235	231	6.5598		
22-8C-3.d	Apigenin	Sample	16.218	221	6.0567		
22-9C-1.d	Apigenin	Sample	16.235	324	11.6280		
22-9C-2.d	Apigenin	Sample	16.235	276	9.0455		
22-9C-3.d	Apigenin	Sample	16.235	312	10.9524		

Agilent Technologies

Apigenin

Data File	Compound	Sample Type	RT	Resp.	Final Conc	Exp. Conc	Accuracy
22-10C-1.d	Apigenin	Sample	16.251	59	ND		
22-10C-2.d	Apigenin	Sample	16.218	82	ND		
22-10C-3.d	Apigenin	Sample	16.235	59	ND		
22-11C-1.d	Apigenin	Sample	16.235	8700	465.0615		
22-11C-2.d	Apigenin	Sample	16.235	8942	478.1772		
22-11C-3.d	Apigenin	Sample	16.235	8859	473.6955		
22-12C-1.d	Apigenin	Sample	16.226	8723	466.3051		
22-12C-2.d	Apigenin	Sample	16.226	8814	471.2408		
22-12C-3.d	Apigenin	Sample	16.235	8856	473.5295		
22-13C-1.d	Apigenin	Sample	16.235	7948	424.3517		
22-13C-2.d	Apigenin	Sample	16.235	8351	446.1842		
22-13C-3.d	Apigenin	Sample	16.226	8135	434.4968		
22-14C-1.d	Apigenin	Sample	16.226	14899	800.6549		
22-14C-2.d	Apigenin	Sample	16.226	14833	797.0931		
22-14C-3.d	Apigenin	Sample	16.226	15010	806.6856		
22-15C-1.d	Apigenin	Sample	16.226	8997	481.1655		
22-15C-2.d	Apigenin	Sample	16.226	8819	471.5055		
22-15C-3.d	Apigenin	Sample	16.235	8922	477.1049		
22-16C-1.d	Apigenin	Sample	16.235	3654	191.8726		
22-16C-2.d	Apigenin	Sample	16.235	3500	183.5556		
22-16C-3.d	Apigenin	Sample	16.235	3665	192.5079		
22-17C-1.d	Apigenin	Sample	16.235	977	46.9842		
22-17C-2.d	Apigenin	Sample	16.243	935	44.7220		
22-17C-3.d	Apigenin	Sample	16.235	913	43.4928		
22-18C-1.d	Apigenin	Sample	16.243	45	ND		
22-18C-2.d	Apigenin	Sample	16.235	60	ND		
22-18C-3.d	Apigenin	Sample	16.218	45	ND		

Quantitative Analysis Complete Report

Agilent Technologies

**Batch Path**	D:\MassHunter\Data\2022ekim\061022cengizhoca\QuantResults\071022.batch.bin		
**Analysis Time**	10/11/2022 1:33:26 PM	**Analyst Name**	Defam-PC\admin
**Report Time**	10/11/2022 1:33:46 PM	**Reporter Name**	admin
**Last Calib Update**	10/11/2022 1:33:17 PM	**Batch State**	Processed
**Quant Batch Version**	B.07.01	**Quant Report Version**	B.07.01

Gallic acid

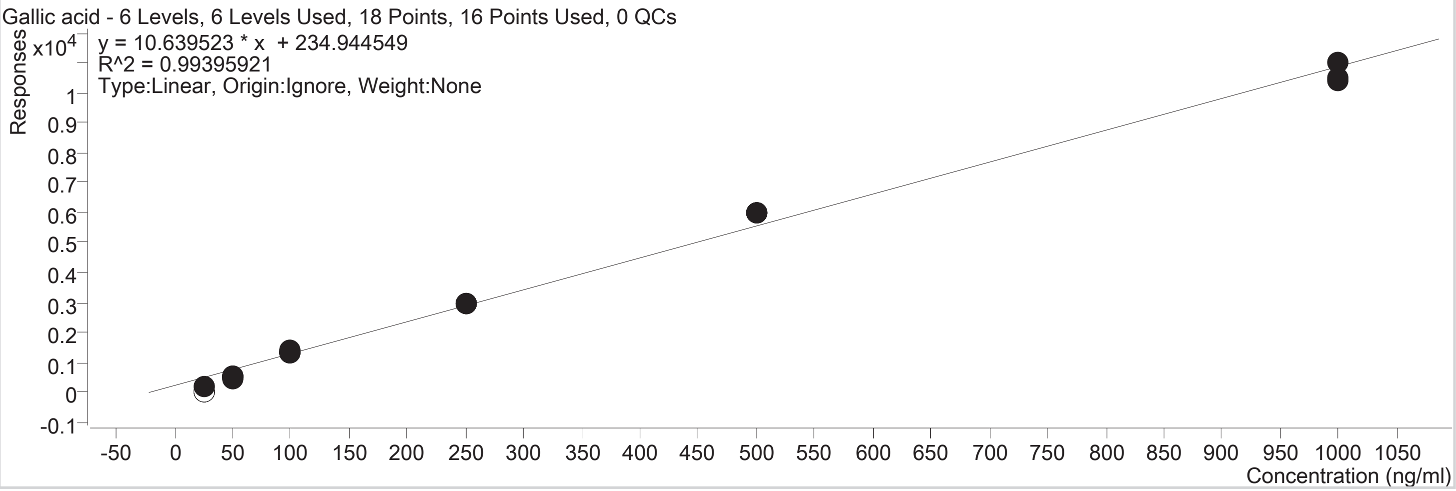

Calibration STD Path	Cal Type	Level	Enabled	Resp.	Exp. Conc	Resp. Factor
D:\MassHunter\Data\2022ekim\061022cengizhoca\25ppb1.d	Calibration	1		9	25.0000	0.3503
D:\MassHunter\Data\2022ekim\061022cengizhoca\25ppb2.d	Calibration	1		6	25.0000	0.2287
D:\MassHunter\Data\2022ekim\061022cengizhoca\25ppb3.d	Calibration	1	x	171	25.0000	6.8438
D:\MassHunter\Data\2022ekim\061022cengizhoca\50ppb1.d	Calibration	2	x	436	50.0000	8.7152
D:\MassHunter\Data\2022ekim\061022cengizhoca\50ppb2.d	Calibration	2	x	533	50.0000	10.6563
D:\MassHunter\Data\2022ekim\061022cengizhoca\50ppb3.d	Calibration	2	x	581	50.0000	11.6155
D:\MassHunter\Data\2022ekim\061022cengizhoca\100ppb1.d	Calibration	3	x	1398	100.0000	13.9816
D:\MassHunter\Data\2022ekim\061022cengizhoca\100ppb2.d	Calibration	3	x	1358	100.0000	13.5799
D:\MassHunter\Data\2022ekim\061022cengizhoca\100ppb3.d	Calibration	3	x	1360	100.0000	13.5977
D:\MassHunter\Data\2022ekim\061022cengizhoca\250ppb1.d	Calibration	4	x	2981	250.0000	11.9245
D:\MassHunter\Data\2022ekim\061022cengizhoca\250ppb2.d	Calibration	4	x	2978	250.0000	11.9128
D:\MassHunter\Data\2022ekim\061022cengizhoca\250ppb3.d	Calibration	4	x	2962	250.0000	11.8493
D:\MassHunter\Data\2022ekim\061022cengizhoca\500ppb1.d	Calibration	5	x	5975	500.0000	11.9506
D:\MassHunter\Data\2022ekim\061022cengizhoca\500ppb2.d	Calibration	5	x	6025	500.0000	12.0507
D:\MassHunter\Data\2022ekim\061022cengizhoca\500ppb3.d	Calibration	5	x	5984	500.0000	11.9678
D:\MassHunter\Data\2022ekim\061022cengizhoca\1000ppb1.d	Calibration	6	x	11032	1000.0000	11.0315
D:\MassHunter\Data\2022ekim\061022cengizhoca\1000ppb2.d	Calibration	6	x	10374	1000.0000	10.3738
D:\MassHunter\Data\2022ekim\061022cengizhoca\1000ppb3.d	Calibration	6	x	10522	1000.0000	10.5224

Quantitative Analysis Complete Report

**Batch Path**	D:\MassHunter\Data\2022ekim\061022cengizhoca\QuantResults\071022.batch.bin		
**Analysis Time**	10/11/2022 1:33:26 PM	**Analyst Name**	Defam-PC\admin
**Report Time**	10/11/2022 1:33:47 PM	**Reporter Name**	admin
**Last Calib Update**	10/11/2022 1:33:17 PM	**Batch State**	Processed
**Quant Batch Version**	B.07.01	**Quant Report Version**	B.07.01

Protocatechuic acid

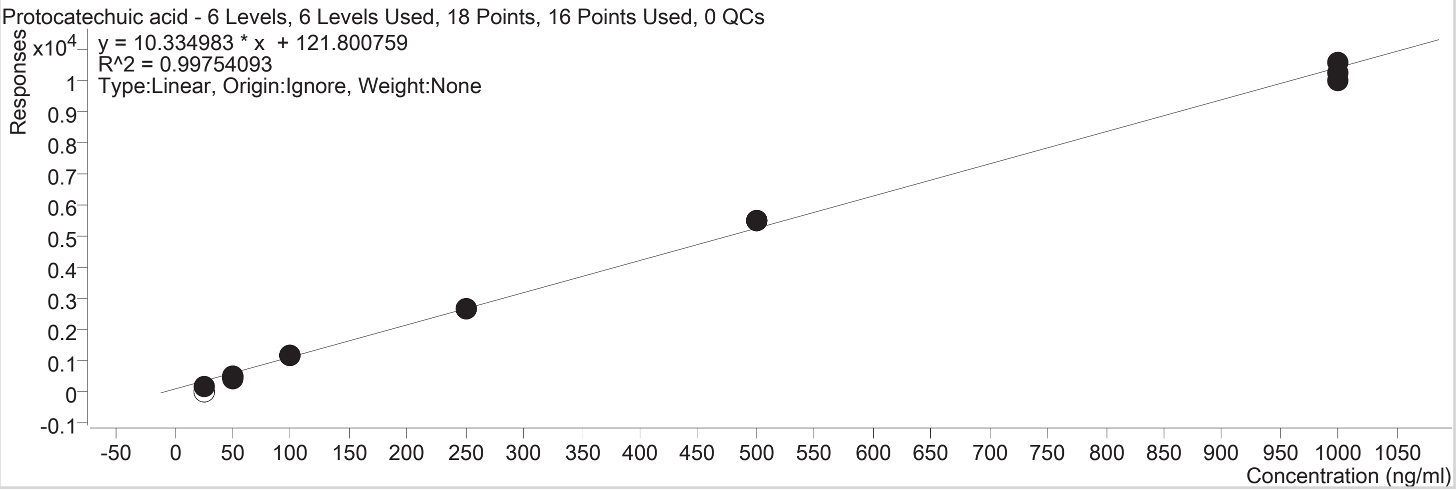

Calibration STD Path	Cal Type	Level	Enabled	Resp.	Exp. Conc	Resp. Factor
D:\MassHunter\Data\2022ekim\061022cengizhoca \25ppb1.d	Calibration	1		40	25.0000	1.6173
D:\MassHunter\Data\2022ekim\061022cengizhoca \25ppb2.d	Calibration	1		18	25.0000	0.7007
D:\MassHunter\Data\2022ekim\061022cengizhoca \25ppb3.d	Calibration	1	x	204	25.0000	8.1775
D:\MassHunter\Data\2022ekim\061022cengizhoca \50ppb1.d	Calibration	2	x	533	50.0000	10.6652
D:\MassHunter\Data\2022ekim\061022cengizhoca \50ppb2.d	Calibration	2	x	486	50.0000	9.7215
D:\MassHunter\Data\2022ekim\061022cengizhoca \50ppb3.d	Calibration	2	x	521	50.0000	10.4254
D:\MassHunter\Data\2022ekim\061022cengizhoca \100ppb1.d	Calibration	3	x	1245	100.0000	12.4507
D:\MassHunter\Data\2022ekim\061022cengizhoca \100ppb2.d	Calibration	3	x	1198	100.0000	11.9840
D:\MassHunter\Data\2022ekim\061022cengizhoca \100ppb3.d	Calibration	3	x	1231	100.0000	12.3081
D:\MassHunter\Data\2022ekim\061022cengizhoca \250ppb1.d	Calibration	4	x	2690	250.0000	10.7593
D:\MassHunter\Data\2022ekim\061022cengizhoca \250ppb2.d	Calibration	4	x	2679	250.0000	10.7173
D:\MassHunter\Data\2022ekim\061022cengizhoca \250ppb3.d	Calibration	4	x	2684	250.0000	10.7355
D:\MassHunter\Data\2022ekim\061022cengizhoca \500ppb1.d	Calibration	5	x	5544	500.0000	11.0884
D:\MassHunter\Data\2022ekim\061022cengizhoca \500ppb2.d	Calibration	5	x	5576	500.0000	11.1528
D:\MassHunter\Data\2022ekim\061022cengizhoca \500ppb3.d	Calibration	5	x	5523	500.0000	11.0457
D:\MassHunter\Data\2022ekim\061022cengizhoca \1000ppb1.d	Calibration	6	x	10639	1000.0000	10.6390
D:\MassHunter\Data\2022ekim\061022cengizhoca \1000ppb2.d	Calibration	6	x	10071	1000.0000	10.0713
D:\MassHunter\Data\2022ekim\061022cengizhoca \1000ppb3.d	Calibration	6	x	10291	1000.0000	10.2905

**Batch Path**	D:\MassHunter\Data\2022ekim\061022cengizhoca\QuantResults\071022.batch.bin		
**Analysis Time**	10/11/2022 1:33:26 PM	**Analyst Name**	Defam-PC\admin
**Report Time**	10/11/2022 1:33:47 PM	**Reporter Name**	admin
**Last Calib Update**	10/11/2022 1:33:17 PM	**Batch State**	Processed
**Quant Batch Version**	B.07.01	**Quant Report Version**	B.07.01

Pyrocatechol

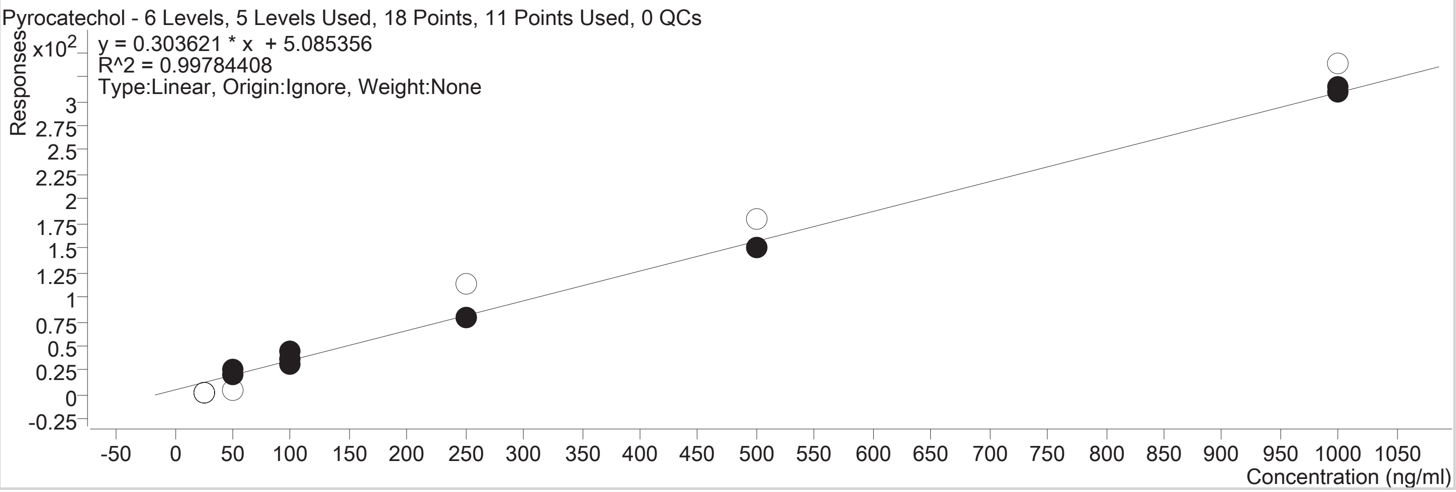

Calibration STD Path	Cal Type	Level	Enabled	Resp.	Exp. Conc	Resp. Factor
D:\MassHunter\Data\2022ekim\061022cengizhoca\25ppb1.d	Calibration	1		2	25.0000	0.0835
D:\MassHunter\Data\2022ekim\061022cengizhoca\25ppb2.d	Calibration	1		2	25.0000	0.0675
D:\MassHunter\Data\2022ekim\061022cengizhoca\25ppb3.d	Calibration	1		3	25.0000	0.1137
D:\MassHunter\Data\2022ekim\061022cengizhoca\50ppb1.d	Calibration	2		5	50.0000	0.0983
D:\MassHunter\Data\2022ekim\061022cengizhoca\50ppb2.d	Calibration	2	x	26	50.0000	0.5293
D:\MassHunter\Data\2022ekim\061022cengizhoca\50ppb3.d	Calibration	2	x	20	50.0000	0.3919
D:\MassHunter\Data\2022ekim\061022cengizhoca\100ppb1.d	Calibration	3	x	36	100.0000	0.3557
D:\MassHunter\Data\2022ekim\061022cengizhoca\100ppb2.d	Calibration	3	x	44	100.0000	0.4404
D:\MassHunter\Data\2022ekim\061022cengizhoca\100ppb3.d	Calibration	3	x	32	100.0000	0.3174
D:\MassHunter\Data\2022ekim\061022cengizhoca\250ppb1.d	Calibration	4	x	79	250.0000	0.3162
D:\MassHunter\Data\2022ekim\061022cengizhoca\250ppb2.d	Calibration	4	x	78	250.0000	0.3127
D:\MassHunter\Data\2022ekim\061022cengizhoca\250ppb3.d	Calibration	4		115	250.0000	0.4582
D:\MassHunter\Data\2022ekim\061022cengizhoca\500ppb1.d	Calibration	5	x	151	500.0000	0.3012
D:\MassHunter\Data\2022ekim\061022cengizhoca\500ppb2.d	Calibration	5	x	151	500.0000	0.3016
D:\MassHunter\Data\2022ekim\061022cengizhoca\500ppb3.d	Calibration	5		180	500.0000	0.3598
D:\MassHunter\Data\2022ekim\061022cengizhoca\1000ppb1.d	Calibration	6	x	315	1000.0000	0.3150
D:\MassHunter\Data\2022ekim\061022cengizhoca\1000ppb2.d	Calibration	6	x	309	1000.0000	0.3090
D:\MassHunter\Data\2022ekim\061022cengizhoca\1000ppb3.d	Calibration	6		339	1000.0000	0.3391

Quantitative Analysis Complete Report

Agilent Technologies

**Batch Path**	D:\MassHunter\Data\2022ekim\061022cengizhoca\QuantResults\071022.batch.bin		
**Analysis Time**	10/11/2022 1:33:26 PM	**Analyst Name**	Defam-PC\admin
**Report Time**	10/11/2022 1:33:47 PM	**Reporter Name**	admin
**Last Calib Update**	10/11/2022 1:33:17 PM	**Batch State**	Processed
**Quant Batch Version**	B.07.01	**Quant Report Version**	B.07.01

3,4-Dihydroxyphenylacetic acid

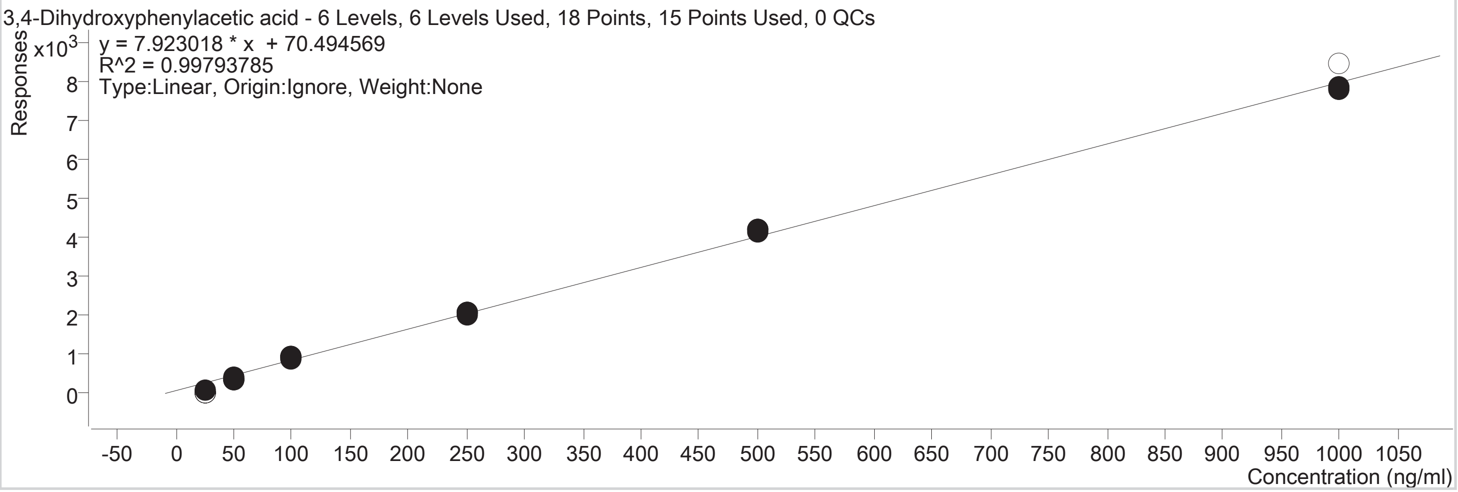

Calibration STD Path	Cal Type	Level	Enabled	Resp.	Exp. Conc	Resp. Factor
D:\MassHunter\Data\2022ekim\061022cengizhoca\25ppb1.d	Calibration	1		3	25.0000	0.1123
D:\MassHunter\Data\2022ekim\061022cengizhoca\25ppb2.d	Calibration	1		5	25.0000	0.1822
D:\MassHunter\Data\2022ekim\061022cengizhoca\25ppb3.d	Calibration	1	x	112	25.0000	4.4971
D:\MassHunter\Data\2022ekim\061022cengizhoca\50ppb1.d	Calibration	2	x	331	50.0000	6.6270
D:\MassHunter\Data\2022ekim\061022cengizhoca\50ppb2.d	Calibration	2	x	364	50.0000	7.2861
D:\MassHunter\Data\2022ekim\061022cengizhoca\50ppb3.d	Calibration	2	x	387	50.0000	7.7330
D:\MassHunter\Data\2022ekim\061022cengizhoca\100ppb1.d	Calibration	3	x	909	100.0000	9.0861
D:\MassHunter\Data\2022ekim\061022cengizhoca\100ppb2.d	Calibration	3	x	926	100.0000	9.2554
D:\MassHunter\Data\2022ekim\061022cengizhoca\100ppb3.d	Calibration	3	x	966	100.0000	9.6588
D:\MassHunter\Data\2022ekim\061022cengizhoca\250ppb1.d	Calibration	4	x	2023	250.0000	8.0901
D:\MassHunter\Data\2022ekim\061022cengizhoca\250ppb2.d	Calibration	4	x	2093	250.0000	8.3720
D:\MassHunter\Data\2022ekim\061022cengizhoca\250ppb3.d	Calibration	4	x	2067	250.0000	8.2693
D:\MassHunter\Data\2022ekim\061022cengizhoca\500ppb1.d	Calibration	5	x	4201	500.0000	8.4010
D:\MassHunter\Data\2022ekim\061022cengizhoca\500ppb2.d	Calibration	5	x	4171	500.0000	8.3427
D:\MassHunter\Data\2022ekim\061022cengizhoca\500ppb3.d	Calibration	5	x	4208	500.0000	8.4151
D:\MassHunter\Data\2022ekim\061022cengizhoca\1000ppb1.d	Calibration	6		8499	1000.0000	8.4988
D:\MassHunter\Data\2022ekim\061022cengizhoca\1000ppb2.d	Calibration	6	x	7824	1000.0000	7.8241
D:\MassHunter\Data\2022ekim\061022cengizhoca\1000ppb3.d	Calibration	6	x	7913	1000.0000	7.9126

Quantitative Analysis Complete Report

Agilent Technologies

**Batch Path**	D:\MassHunter\Data\2022ekim\061022cengizhoca\QuantResults\071022.batch.bin		
**Analysis Time**	10/11/2022 1:33:26 PM	**Analyst Name**	Defam-PC\admin
**Report Time**	10/11/2022 1:33:47 PM	**Reporter Name**	admin
**Last Calib Update**	10/11/2022 1:33:17 PM	**Batch State**	Processed
**Quant Batch Version**	B.07.01	**Quant Report Version**	B.07.01

(+)-Catechin

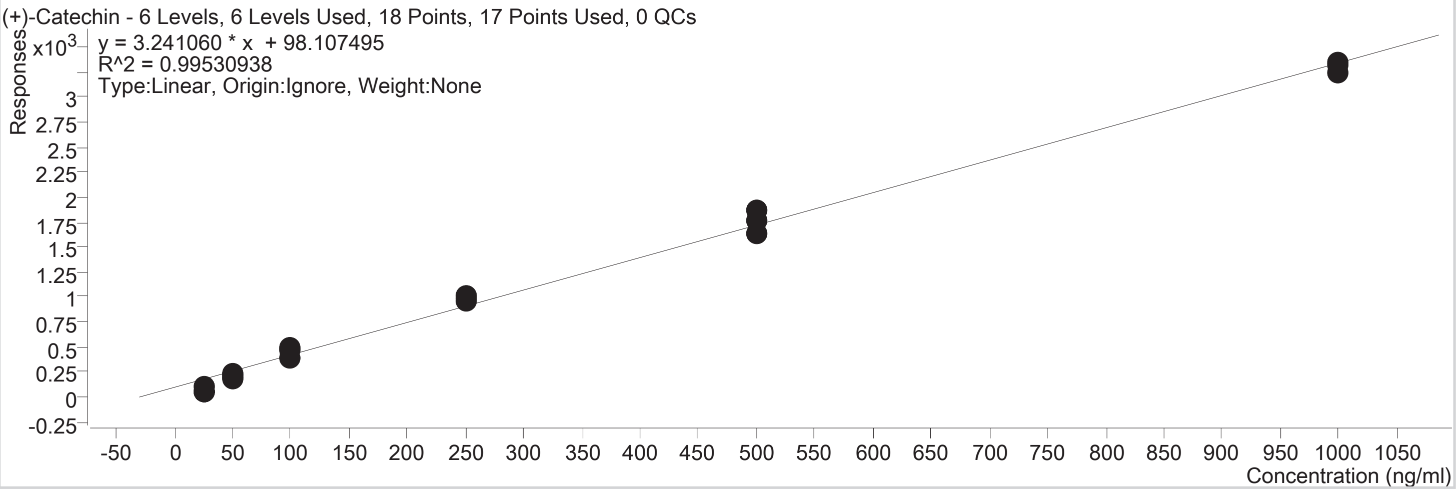

Calibration STD Path	Cal Type	Level	Enabled	Resp.	Exp. Conc	Resp. Factor
D:\MassHunter\Data\2022ekim\061022cengizhoca\25ppb1.d	Calibration	1		48	25.0000	1.9010
D:\MassHunter\Data\2022ekim\061022cengizhoca\25ppb2.d	Calibration	1	x	60	25.0000	2.4010
D:\MassHunter\Data\2022ekim\061022cengizhoca\25ppb3.d	Calibration	1	x	111	25.0000	4.4566
D:\MassHunter\Data\2022ekim\061022cengizhoca\50ppb1.d	Calibration	2	x	192	50.0000	3.8303
D:\MassHunter\Data\2022ekim\061022cengizhoca\50ppb2.d	Calibration	2	x	198	50.0000	3.9623
D:\MassHunter\Data\2022ekim\061022cengizhoca\50ppb3.d	Calibration	2	x	229	50.0000	4.5811
D:\MassHunter\Data\2022ekim\061022cengizhoca\100ppb1.d	Calibration	3	x	473	100.0000	4.7339
D:\MassHunter\Data\2022ekim\061022cengizhoca\100ppb2.d	Calibration	3	x	506	100.0000	5.0601
D:\MassHunter\Data\2022ekim\061022cengizhoca\100ppb3.d	Calibration	3	x	403	100.0000	4.0310
D:\MassHunter\Data\2022ekim\061022cengizhoca\250ppb1.d	Calibration	4	x	1006	250.0000	4.0253
D:\MassHunter\Data\2022ekim\061022cengizhoca\250ppb2.d	Calibration	4	x	994	250.0000	3.9745
D:\MassHunter\Data\2022ekim\061022cengizhoca\250ppb3.d	Calibration	4	x	959	250.0000	3.8373
D:\MassHunter\Data\2022ekim\061022cengizhoca\500ppb1.d	Calibration	5	x	1764	500.0000	3.5288
D:\MassHunter\Data\2022ekim\061022cengizhoca\500ppb2.d	Calibration	5	x	1869	500.0000	3.7370
D:\MassHunter\Data\2022ekim\061022cengizhoca\500ppb3.d	Calibration	5	x	1635	500.0000	3.2698
D:\MassHunter\Data\2022ekim\061022cengizhoca\1000ppb1.d	Calibration	6	x	3351	1000.0000	3.3512
D:\MassHunter\Data\2022ekim\061022cengizhoca\1000ppb2.d	Calibration	6	x	3317	1000.0000	3.3167
D:\MassHunter\Data\2022ekim\061022cengizhoca\1000ppb3.d	Calibration	6	x	3236	1000.0000	3.2363

Quantitative Analysis Complete Report

**Batch Path**	D:\MassHunter\Data\2022ekim\061022cengizhoca\QuantResults\071022.batch.bin		
**Analysis Time**	10/11/2022 1:33:26 PM	**Analyst Name**	Defam-PC\admin
**Report Time**	10/11/2022 1:33:47 PM	**Reporter Name**	admin
**Last Calib Update**	10/11/2022 1:33:17 PM	**Batch State**	Processed
**Quant Batch Version**	B.07.01	**Quant Report Version**	B.07.01

2,5-Dihydroxybenzoic acid

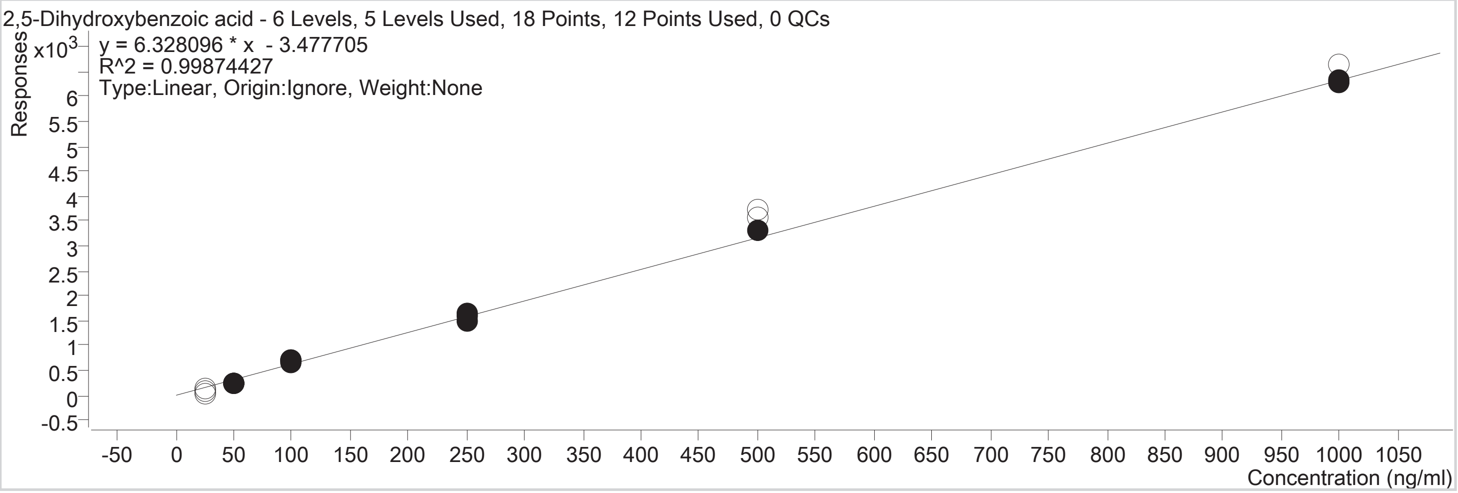

Calibration STD Path	Cal Type	Level	Enabled	Resp.	Exp. Conc	Resp. Factor
D:\MassHunter\Data\2022ekim\061022cengizhoca\25ppb1.d	Calibration	1		18	25.0000	0.7382
D:\MassHunter\Data\2022ekim\061022cengizhoca\25ppb2.d	Calibration	1		105	25.0000	4.1993
D:\MassHunter\Data\2022ekim\061022cengizhoca\25ppb3.d	Calibration	1		127	25.0000	5.0828
D:\MassHunter\Data\2022ekim\061022cengizhoca\50ppb1.d	Calibration	2	x	216	50.0000	4.3201
D:\MassHunter\Data\2022ekim\061022cengizhoca\50ppb2.d	Calibration	2	x	265	50.0000	5.3057
D:\MassHunter\Data\2022ekim\061022cengizhoca\50ppb3.d	Calibration	2	x	217	50.0000	4.3373
D:\MassHunter\Data\2022ekim\061022cengizhoca\100ppb1.d	Calibration	3	x	674	100.0000	6.7440
D:\MassHunter\Data\2022ekim\061022cengizhoca\100ppb2.d	Calibration	3	x	722	100.0000	7.2191
D:\MassHunter\Data\2022ekim\061022cengizhoca\100ppb3.d	Calibration	3	x	684	100.0000	6.8433
D:\MassHunter\Data\2022ekim\061022cengizhoca\250ppb1.d	Calibration	4	x	1642	250.0000	6.5693
D:\MassHunter\Data\2022ekim\061022cengizhoca\250ppb2.d	Calibration	4	x	1503	250.0000	6.0127
D:\MassHunter\Data\2022ekim\061022cengizhoca\250ppb3.d	Calibration	4	x	1572	250.0000	6.2862
D:\MassHunter\Data\2022ekim\061022cengizhoca\500ppb1.d	Calibration	5	x	3299	500.0000	6.5978
D:\MassHunter\Data\2022ekim\061022cengizhoca\500ppb2.d	Calibration	5		3589	500.0000	7.1781
D:\MassHunter\Data\2022ekim\061022cengizhoca\500ppb3.d	Calibration	5		3731	500.0000	7.4627
D:\MassHunter\Data\2022ekim\061022cengizhoca\1000ppb1.d	Calibration	6		6657	1000.0000	6.6565
D:\MassHunter\Data\2022ekim\061022cengizhoca\1000ppb2.d	Calibration	6	x	6265	1000.0000	6.2647
D:\MassHunter\Data\2022ekim\061022cengizhoca\1000ppb3.d	Calibration	6	x	6313	1000.0000	6.3128

Quantitative Analysis Complete Report

Agilent Technologies

**Batch Path**	D:\MassHunter\Data\2022ekim\061022cengizhoca\QuantResults\071022.batch.bin		
**Analysis Time**	10/11/2022 1:33:26 PM	**Analyst Name**	Defam-PC\admin
**Report Time**	10/11/2022 1:33:47 PM	**Reporter Name**	admin
**Last Calib Update**	10/11/2022 1:33:17 PM	**Batch State**	Processed
**Quant Batch Version**	B.07.01	**Quant Report Version**	B.07.01

Chlorogenic acid

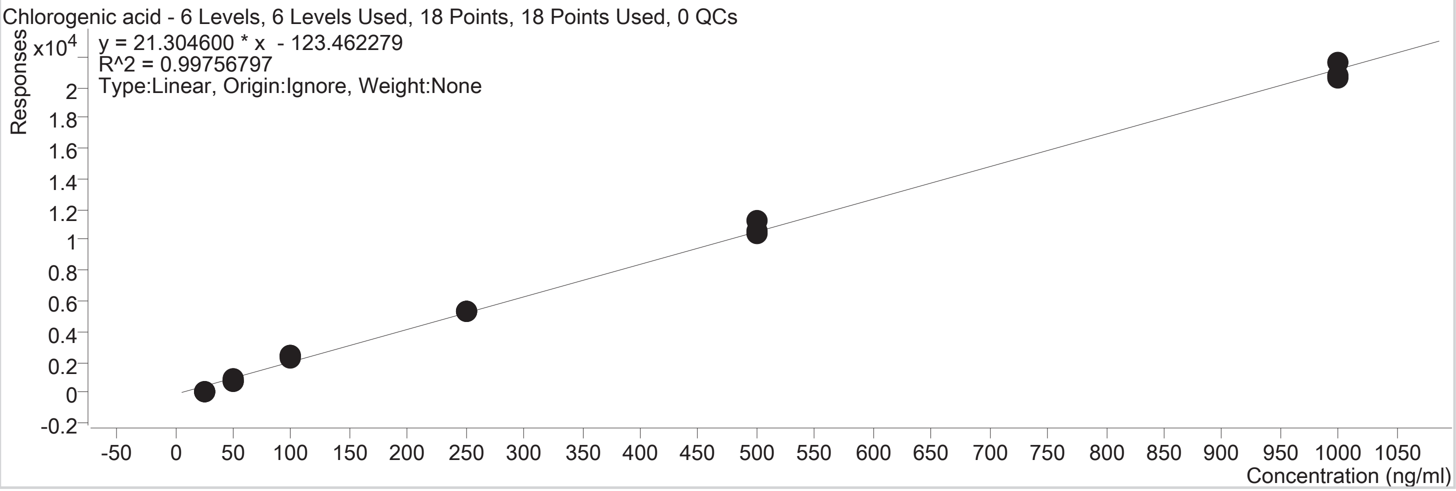

Calibration STD Path	Cal Type	Level	Enabled	Resp.	Exp. Conc	Resp. Factor
D:\MassHunter\Data\2022ekim\061022cengizhoca\25ppb1.d	Calibration	1	x	10	25.0000	0.3828
D:\MassHunter\Data\2022ekim\061022cengizhoca\25ppb2.d	Calibration	1	x	7	25.0000	0.2998
D:\MassHunter\Data\2022ekim\061022cengizhoca\25ppb3.d	Calibration	1	x	14	25.0000	0.5550
D:\MassHunter\Data\2022ekim\061022cengizhoca\50ppb1.d	Calibration	2	x	674	50.0000	13.4728
D:\MassHunter\Data\2022ekim\061022cengizhoca\50ppb2.d	Calibration	2	x	731	50.0000	14.6292
D:\MassHunter\Data\2022ekim\061022cengizhoca\50ppb3.d	Calibration	2	x	945	50.0000	18.8930
D:\MassHunter\Data\2022ekim\061022cengizhoca\100ppb1.d	Calibration	3	x	2439	100.0000	24.3915
D:\MassHunter\Data\2022ekim\061022cengizhoca\100ppb2.d	Calibration	3	x	2273	100.0000	22.7334
D:\MassHunter\Data\2022ekim\061022cengizhoca\100ppb3.d	Calibration	3	x	2379	100.0000	23.7890
D:\MassHunter\Data\2022ekim\061022cengizhoca\250ppb1.d	Calibration	4	x	5367	250.0000	21.4694
D:\MassHunter\Data\2022ekim\061022cengizhoca\250ppb2.d	Calibration	4	x	5356	250.0000	21.4253
D:\MassHunter\Data\2022ekim\061022cengizhoca\250ppb3.d	Calibration	4	x	5296	250.0000	21.1843
D:\MassHunter\Data\2022ekim\061022cengizhoca\500ppb1.d	Calibration	5	x	10372	500.0000	20.7446
D:\MassHunter\Data\2022ekim\061022cengizhoca\500ppb2.d	Calibration	5	x	11244	500.0000	22.4883
D:\MassHunter\Data\2022ekim\061022cengizhoca\500ppb3.d	Calibration	5	x	10663	500.0000	21.3251
D:\MassHunter\Data\2022ekim\061022cengizhoca\1000ppb1.d	Calibration	6	x	21670	1000.0000	21.6701
D:\MassHunter\Data\2022ekim\061022cengizhoca\1000ppb2.d	Calibration	6	x	20594	1000.0000	20.5935
D:\MassHunter\Data\2022ekim\061022cengizhoca\1000ppb3.d	Calibration	6	x	20777	1000.0000	20.7773

Quantitative Analysis Complete Report

**Batch Path**	D:\MassHunter\Data\2022ekim\061022cengizhoca\QuantResults\071022.batch.bin		
**Analysis Time**	10/11/2022 1:33:26 PM	**Analyst Name**	Defam-PC\admin
**Report Time**	10/11/2022 1:33:47 PM	**Reporter Name**	admin
**Last Calib Update**	10/11/2022 1:33:17 PM	**Batch State**	Processed
**Quant Batch Version**	B.07.01	**Quant Report Version**	B.07.01

3-Hydroxybenzoic acid

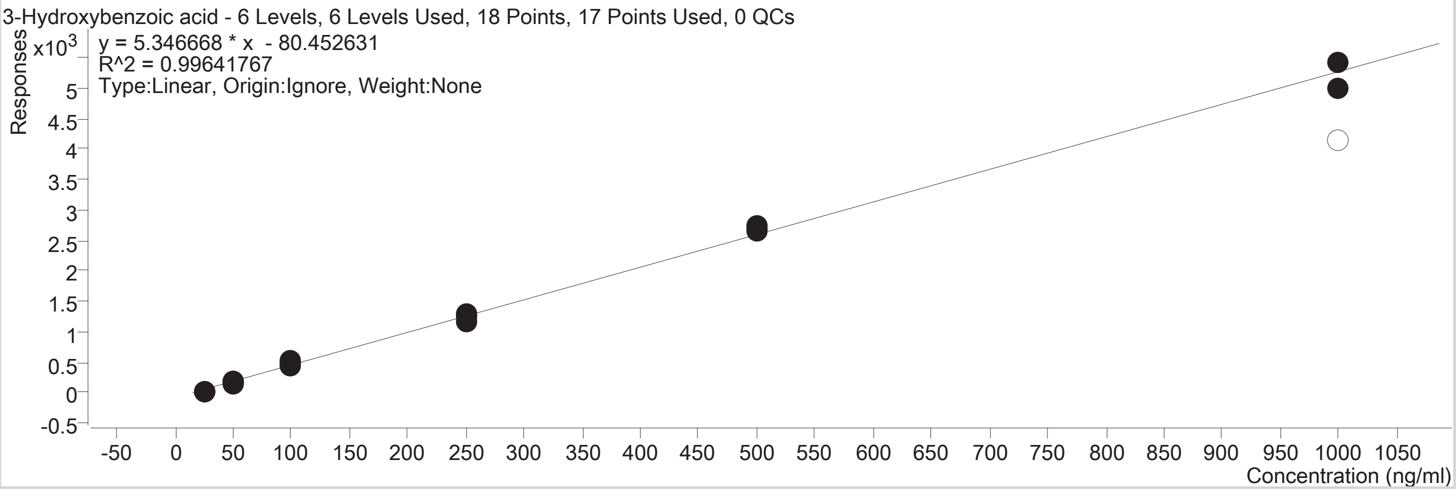

Calibration STD Path	Cal Type	Level	Enabled	Resp.	Exp. Conc	Resp. Factor
D:\MassHunter\Data\2022ekim\061022cengizhoca\25ppb1.d	Calibration	1	x	5	25.0000	0.1844
D:\MassHunter\Data\2022ekim\061022cengizhoca\25ppb2.d	Calibration	1	x	7	25.0000	0.2751
D:\MassHunter\Data\2022ekim\061022cengizhoca\25ppb3.d	Calibration	1	x	16	25.0000	0.6331
D:\MassHunter\Data\2022ekim\061022cengizhoca\50ppb1.d	Calibration	2	x	144	50.0000	2.8756
D:\MassHunter\Data\2022ekim\061022cengizhoca\50ppb2.d	Calibration	2	x	198	50.0000	3.9595
D:\MassHunter\Data\2022ekim\061022cengizhoca\50ppb3.d	Calibration	2	x	200	50.0000	4.0001
D:\MassHunter\Data\2022ekim\061022cengizhoca\100ppb1.d	Calibration	3	x	482	100.0000	4.8193
D:\MassHunter\Data\2022ekim\061022cengizhoca\100ppb2.d	Calibration	3	x	430	100.0000	4.3026
D:\MassHunter\Data\2022ekim\061022cengizhoca\100ppb3.d	Calibration	3	x	534	100.0000	5.3352
D:\MassHunter\Data\2022ekim\061022cengizhoca\250ppb1.d	Calibration	4	x	1239	250.0000	4.9542
D:\MassHunter\Data\2022ekim\061022cengizhoca\250ppb2.d	Calibration	4	x	1158	250.0000	4.6314
D:\MassHunter\Data\2022ekim\061022cengizhoca\250ppb3.d	Calibration	4	x	1273	250.0000	5.0921
D:\MassHunter\Data\2022ekim\061022cengizhoca\500ppb1.d	Calibration	5	x	2642	500.0000	5.2840
D:\MassHunter\Data\2022ekim\061022cengizhoca\500ppb2.d	Calibration	5	x	2732	500.0000	5.4636
D:\MassHunter\Data\2022ekim\061022cengizhoca\500ppb3.d	Calibration	5	x	2698	500.0000	5.3961
D:\MassHunter\Data\2022ekim\061022cengizhoca\1000ppb1.d	Calibration	6	x	5429	1000.0000	5.4285
D:\MassHunter\Data\2022ekim\061022cengizhoca\1000ppb2.d	Calibration	6	x	4978	1000.0000	4.9781
D:\MassHunter\Data\2022ekim\061022cengizhoca\1000ppb3.d	Calibration	6		4136	1000.0000	4.1364

Quantitative Analysis Complete Report

**Batch Path**	D:\MassHunter\Data\2022ekim\061022cengizhoca\QuantResults\071022.batch.bin		
**Analysis Time**	10/11/2022 1:33:26 PM	**Analyst Name**	Defam-PC\admin
**Report Time**	10/11/2022 1:33:47 PM	**Reporter Name**	admin
**Last Calib Update**	10/11/2022 1:33:17 PM	**Batch State**	Processed
**Quant Batch Version**	B.07.01	**Quant Report Version**	B.07.01

4-Hydroxybenzoic acid

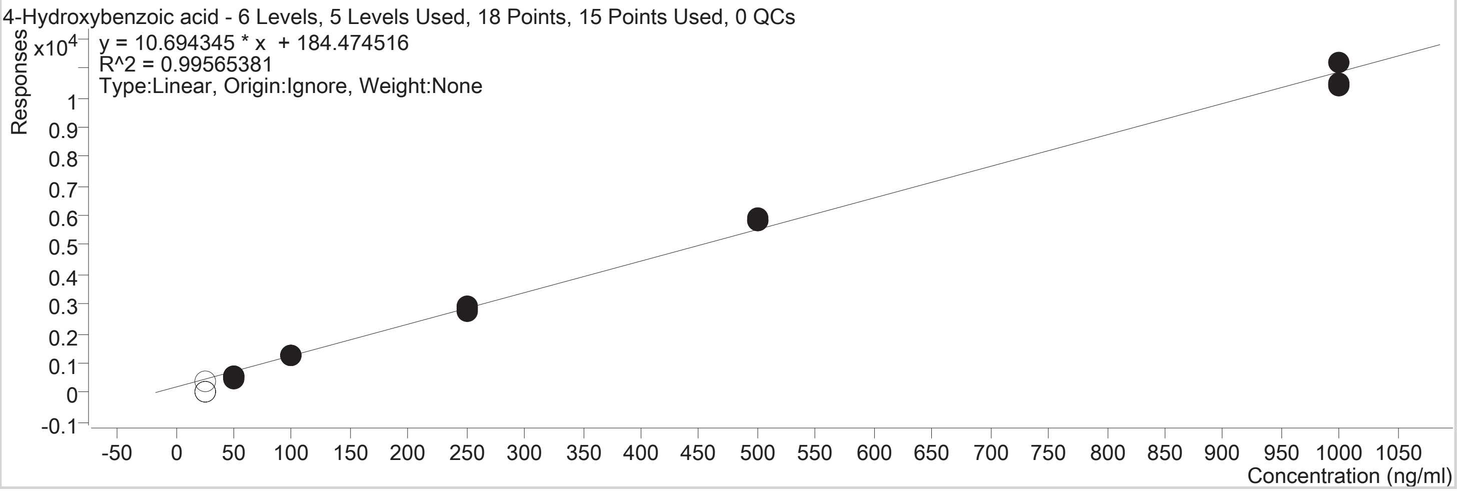

Calibration STD Path	Cal Type	Level	Enabled	Resp.	Exp. Conc	Resp. Factor
D:\MassHunter\Data\2022ekim\061022cengizhoca\25ppb1.d	Calibration	1		391	25.0000	15.6567
D:\MassHunter\Data\2022ekim\061022cengizhoca\25ppb2.d	Calibration	1		26	25.0000	1.0411
D:\MassHunter\Data\2022ekim\061022cengizhoca\25ppb3.d	Calibration	1		8	25.0000	0.3165
D:\MassHunter\Data\2022ekim\061022cengizhoca\50ppb1.d	Calibration	2	x	495	50.0000	9.8952
D:\MassHunter\Data\2022ekim\061022cengizhoca\50ppb2.d	Calibration	2	x	576	50.0000	11.5203
D:\MassHunter\Data\2022ekim\061022cengizhoca\50ppb3.d	Calibration	2	x	590	50.0000	11.8083
D:\MassHunter\Data\2022ekim\061022cengizhoca\100ppb1.d	Calibration	3	x	1269	100.0000	12.6873
D:\MassHunter\Data\2022ekim\061022cengizhoca\100ppb2.d	Calibration	3	x	1218	100.0000	12.1763
D:\MassHunter\Data\2022ekim\061022cengizhoca\100ppb3.d	Calibration	3	x	1272	100.0000	12.7222
D:\MassHunter\Data\2022ekim\061022cengizhoca\250ppb1.d	Calibration	4	x	2764	250.0000	11.0571
D:\MassHunter\Data\2022ekim\061022cengizhoca\250ppb2.d	Calibration	4	x	2922	250.0000	11.6888
D:\MassHunter\Data\2022ekim\061022cengizhoca\250ppb3.d	Calibration	4	x	2843	250.0000	11.3735
D:\MassHunter\Data\2022ekim\061022cengizhoca\500ppb1.d	Calibration	5	x	5913	500.0000	11.8262
D:\MassHunter\Data\2022ekim\061022cengizhoca\500ppb2.d	Calibration	5	x	5860	500.0000	11.7196
D:\MassHunter\Data\2022ekim\061022cengizhoca\500ppb3.d	Calibration	5	x	5837	500.0000	11.6747
D:\MassHunter\Data\2022ekim\061022cengizhoca\1000ppb1.d	Calibration	6	x	11222	1000.0000	11.2215
D:\MassHunter\Data\2022ekim\061022cengizhoca\1000ppb2.d	Calibration	6	x	10421	1000.0000	10.4208
D:\MassHunter\Data\2022ekim\061022cengizhoca\1000ppb3.d	Calibration	6	x	10523	1000.0000	10.5226

Quantitative Analysis Complete Report

Agilent Technologies

**Batch Path**	D:\MassHunter\Data\2022ekim\061022cengizhoca\QuantResults\071022.batch.bin		
**Analysis Time**	10/11/2022 1:33:26 PM	**Analyst Name**	Defam-PC\admin
**Report Time**	10/11/2022 1:33:47 PM	**Reporter Name**	admin
**Last Calib Update**	10/11/2022 1:33:17 PM	**Batch State**	Processed
**Quant Batch Version**	B.07.01	**Quant Report Version**	B.07.01

(-)-Epicatechin

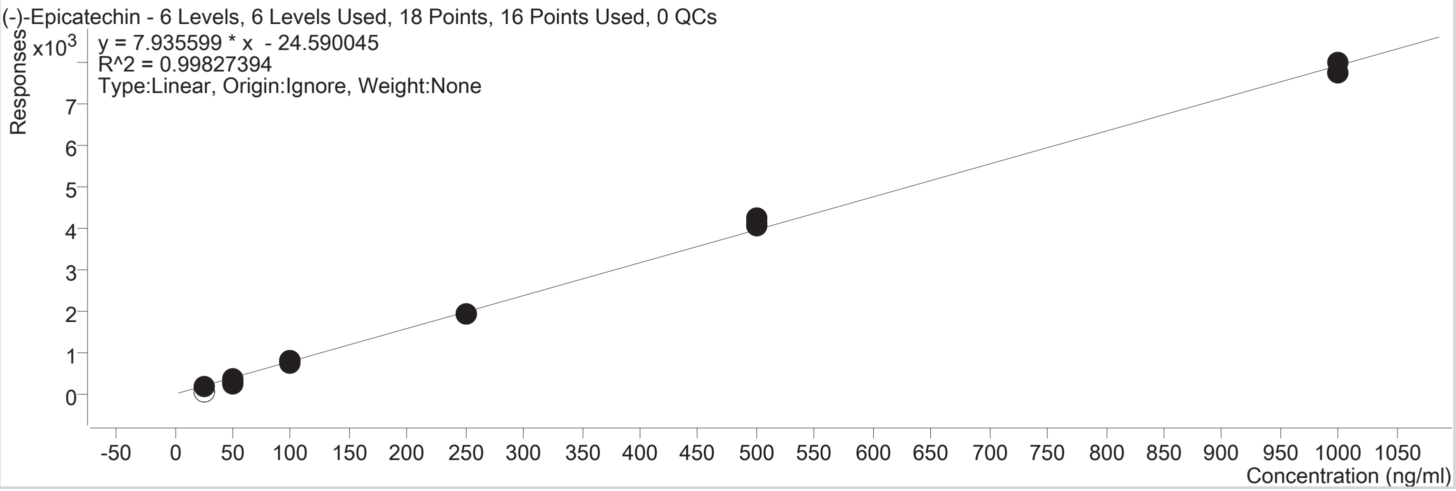

Calibration STD Path	Cal Type	Level	Enabled	Resp.	Exp. Conc	Resp. Factor
D:\MassHunter\Data\2022ekim\061022cengizhoca\25ppb1.d	Calibration	1		59	25.0000	2.3648
D:\MassHunter\Data\2022ekim\061022cengizhoca\25ppb2.d	Calibration	1		19	25.0000	0.7572
D:\MassHunter\Data\2022ekim\061022cengizhoca\25ppb3.d	Calibration	1	x	133	25.0000	5.3395
D:\MassHunter\Data\2022ekim\061022cengizhoca\50ppb1.d	Calibration	2	x	225	50.0000	4.4945
D:\MassHunter\Data\2022ekim\061022cengizhoca\50ppb2.d	Calibration	2	x	308	50.0000	6.1594
D:\MassHunter\Data\2022ekim\061022cengizhoca\50ppb3.d	Calibration	2	x	359	50.0000	7.1739
D:\MassHunter\Data\2022ekim\061022cengizhoca\100ppb1.d	Calibration	3	x	743	100.0000	7.4332
D:\MassHunter\Data\2022ekim\061022cengizhoca\100ppb2.d	Calibration	3	x	794	100.0000	7.9384
D:\MassHunter\Data\2022ekim\061022cengizhoca\100ppb3.d	Calibration	3	x	816	100.0000	8.1601
D:\MassHunter\Data\2022ekim\061022cengizhoca\250ppb1.d	Calibration	4	x	1942	250.0000	7.7668
D:\MassHunter\Data\2022ekim\061022cengizhoca\250ppb2.d	Calibration	4	x	1912	250.0000	7.6484
D:\MassHunter\Data\2022ekim\061022cengizhoca\250ppb3.d	Calibration	4	x	1940	250.0000	7.7605
D:\MassHunter\Data\2022ekim\061022cengizhoca\500ppb1.d	Calibration	5	x	4072	500.0000	8.1448
D:\MassHunter\Data\2022ekim\061022cengizhoca\500ppb2.d	Calibration	5	x	4097	500.0000	8.1932
D:\MassHunter\Data\2022ekim\061022cengizhoca\500ppb3.d	Calibration	5	x	4210	500.0000	8.4196
D:\MassHunter\Data\2022ekim\061022cengizhoca\1000ppb1.d	Calibration	6	x	8001	1000.0000	8.0007
D:\MassHunter\Data\2022ekim\061022cengizhoca\1000ppb2.d	Calibration	6	x	7749	1000.0000	7.7493
D:\MassHunter\Data\2022ekim\061022cengizhoca\1000ppb3.d	Calibration	6	x	7737	1000.0000	7.7370

Quantitative Analysis Complete Report

Agilent Technologies

**Batch Path**	D:\MassHunter\Data\2022ekim\061022cengizhoca\QuantResults\071022.batch.bin		
**Analysis Time**	10/11/2022 1:33:26 PM	**Analyst Name**	Defam-PC\admin
**Report Time**	10/11/2022 1:33:47 PM	**Reporter Name**	admin
**Last Calib Update**	10/11/2022 1:33:17 PM	**Batch State**	Processed
**Quant Batch Version**	B.07.01	**Quant Report Version**	B.07.01

Caffeic acid

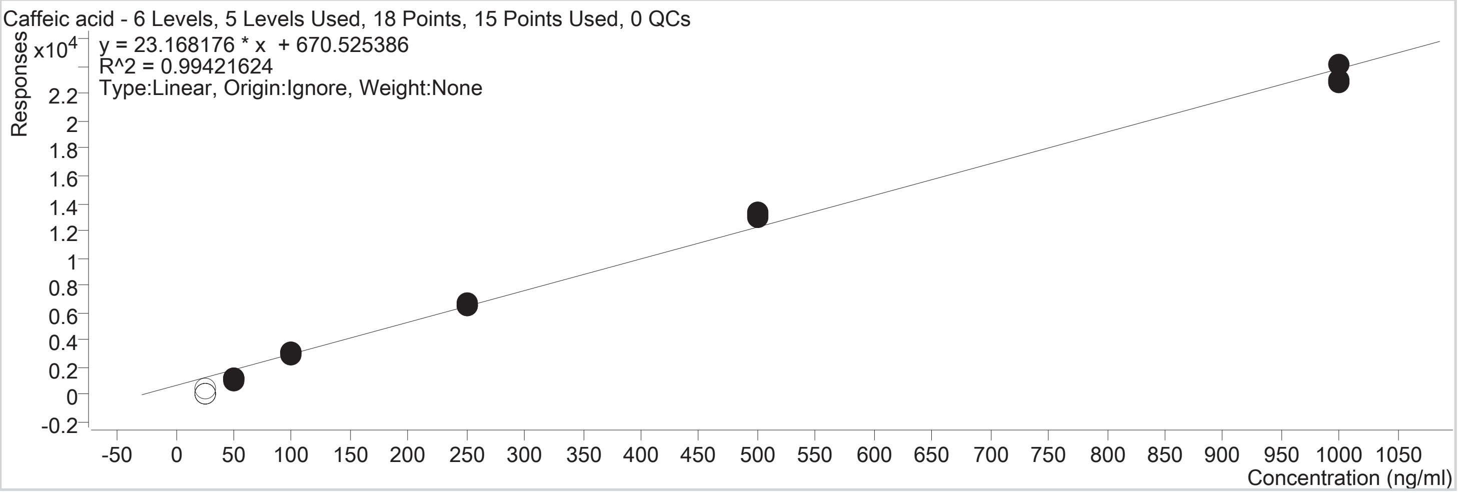

Calibration STD Path	Cal Type	Level	Enabled	Resp.	Exp. Conc	Resp. Factor
D:\MassHunter\Data\2022ekim\061022cengizhoca \25ppb1.d	Calibration	1		30	25.0000	1.2038
D:\MassHunter\Data\2022ekim\061022cengizhoca \25ppb2.d	Calibration	1		44	25.0000	1.7555
D:\MassHunter\Data\2022ekim\061022cengizhoca \25ppb3.d	Calibration	1		371	25.0000	14.8504
D:\MassHunter\Data\2022ekim\061022cengizhoca \50ppb1.d	Calibration	2	x	1120	50.0000	22.3940
D:\MassHunter\Data\2022ekim\061022cengizhoca \50ppb2.d	Calibration	2	x	1226	50.0000	24.5181
D:\MassHunter\Data\2022ekim\061022cengizhoca \50ppb3.d	Calibration	2	x	1223	50.0000	24.4542
D:\MassHunter\Data\2022ekim\061022cengizhoca \100ppb1.d	Calibration	3	x	2941	100.0000	29.4077
D:\MassHunter\Data\2022ekim\061022cengizhoca \100ppb2.d	Calibration	3	x	3185	100.0000	31.8530
D:\MassHunter\Data\2022ekim\061022cengizhoca \100ppb3.d	Calibration	3	x	3025	100.0000	30.2527
D:\MassHunter\Data\2022ekim\061022cengizhoca \250ppb1.d	Calibration	4	x	6437	250.0000	25.7471
D:\MassHunter\Data\2022ekim\061022cengizhoca \250ppb2.d	Calibration	4	x	6580	250.0000	26.3183
D:\MassHunter\Data\2022ekim\061022cengizhoca \250ppb3.d	Calibration	4	x	6725	250.0000	26.8988
D:\MassHunter\Data\2022ekim\061022cengizhoca \500ppb1.d	Calibration	5	x	13194	500.0000	26.3876
D:\MassHunter\Data\2022ekim\061022cengizhoca \500ppb2.d	Calibration	5	x	13046	500.0000	26.0929
D:\MassHunter\Data\2022ekim\061022cengizhoca \500ppb3.d	Calibration	5	x	13298	500.0000	26.5959
D:\MassHunter\Data\2022ekim\061022cengizhoca \1000ppb1.d	Calibration	6	x	24200	1000.0000	24.2004
D:\MassHunter\Data\2022ekim\061022cengizhoca \1000ppb2.d	Calibration	6	x	23033	1000.0000	23.0334
D:\MassHunter\Data\2022ekim\061022cengizhoca \1000ppb3.d	Calibration	6	x	22884	1000.0000	22.8838

Quantitative Analysis Complete Report

**Batch Path**	D:\MassHunter\Data\2022ekim\061022cengizhoca\QuantResults\071022.batch.bin		
**Analysis Time**	10/11/2022 1:33:26 PM	**Analyst Name**	Defam-PC\admin
**Report Time**	10/11/2022 1:33:47 PM	**Reporter Name**	admin
**Last Calib Update**	10/11/2022 1:33:17 PM	**Batch State**	Processed
**Quant Batch Version**	B.07.01	**Quant Report Version**	B.07.01

Syringic acid

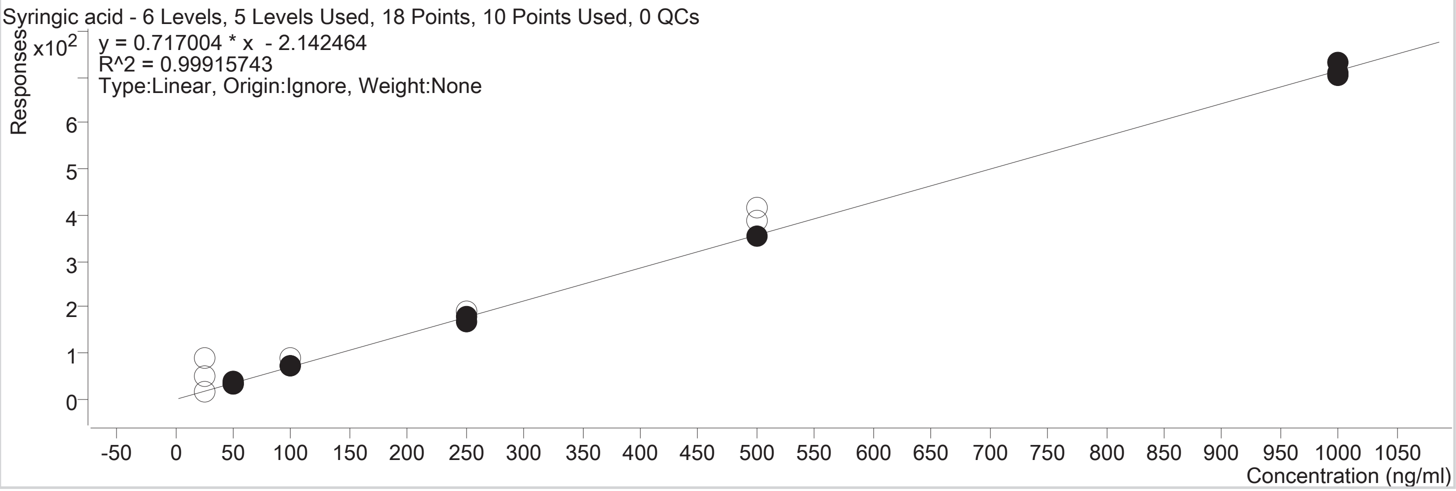

Calibration STD Path	Cal Type	Level	Enabled	Resp.	Exp. Conc	Resp. Factor
D:\MassHunter\Data\2022ekim\061022cengizhoca\25ppb1.d	Calibration	1		86	25.0000	3.4353
D:\MassHunter\Data\2022ekim\061022cengizhoca\25ppb2.d	Calibration	1		50	25.0000	2.0196
D:\MassHunter\Data\2022ekim\061022cengizhoca\25ppb3.d	Calibration	1		14	25.0000	0.5460
D:\MassHunter\Data\2022ekim\061022cengizhoca\50ppb1.d	Calibration	2	x	32	50.0000	0.6423
D:\MassHunter\Data\2022ekim\061022cengizhoca\50ppb2.d	Calibration	2	x	39	50.0000	0.7712
D:\MassHunter\Data\2022ekim\061022cengizhoca\50ppb3.d	Calibration	2		34	50.0000	0.6799
D:\MassHunter\Data\2022ekim\061022cengizhoca\100ppb1.d	Calibration	3	x	70	100.0000	0.7018
D:\MassHunter\Data\2022ekim\061022cengizhoca\100ppb2.d	Calibration	3		86	100.0000	0.8572
D:\MassHunter\Data\2022ekim\061022cengizhoca\100ppb3.d	Calibration	3	x	73	100.0000	0.7263
D:\MassHunter\Data\2022ekim\061022cengizhoca\250ppb1.d	Calibration	4		190	250.0000	0.7594
D:\MassHunter\Data\2022ekim\061022cengizhoca\250ppb2.d	Calibration	4	x	167	250.0000	0.6683
D:\MassHunter\Data\2022ekim\061022cengizhoca\250ppb3.d	Calibration	4	x	181	250.0000	0.7243
D:\MassHunter\Data\2022ekim\061022cengizhoca\500ppb1.d	Calibration	5	x	353	500.0000	0.7053
D:\MassHunter\Data\2022ekim\061022cengizhoca\500ppb2.d	Calibration	5		418	500.0000	0.8361
D:\MassHunter\Data\2022ekim\061022cengizhoca\500ppb3.d	Calibration	5		387	500.0000	0.7747
D:\MassHunter\Data\2022ekim\061022cengizhoca\1000ppb1.d	Calibration	6	x	734	1000.0000	0.7341
D:\MassHunter\Data\2022ekim\061022cengizhoca\1000ppb2.d	Calibration	6	x	708	1000.0000	0.7079
D:\MassHunter\Data\2022ekim\061022cengizhoca\1000ppb3.d	Calibration	6	x	705	1000.0000	0.7055

Quantitative Analysis Complete Report

Agilent Technologies

**Batch Path**	D:\MassHunter\Data\2022ekim\061022cengizhoca\QuantResults\071022.batch.bin		
**Analysis Time**	10/11/2022 1:33:26 PM	**Analyst Name**	Defam-PC\admin
**Report Time**	10/11/2022 1:33:47 PM	**Reporter Name**	admin
**Last Calib Update**	10/11/2022 1:33:17 PM	**Batch State**	Processed
**Quant Batch Version**	B.07.01	**Quant Report Version**	B.07.01

Vanillin

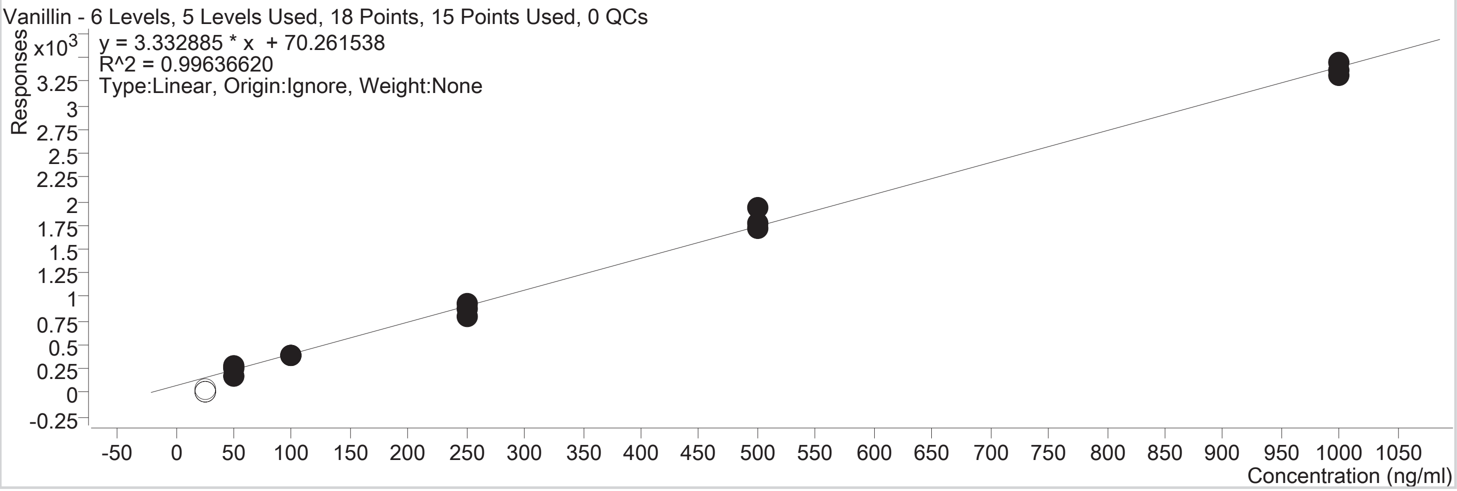

Calibration STD Path	Cal Type	Level	Enabled	Resp.	Exp. Conc	Resp. Factor
D:\MassHunter\Data\2022ekim\061022cengizhoca\25ppb1.d	Calibration	1		1	25.0000	0.0595
D:\MassHunter\Data\2022ekim\061022cengizhoca\25ppb2.d	Calibration	1		16	25.0000	0.6390
D:\MassHunter\Data\2022ekim\061022cengizhoca\25ppb3.d	Calibration	1		45	25.0000	1.8181
D:\MassHunter\Data\2022ekim\061022cengizhoca\50ppb1.d	Calibration	2	x	175	50.0000	3.4970
D:\MassHunter\Data\2022ekim\061022cengizhoca\50ppb2.d	Calibration	2	x	258	50.0000	5.1528
D:\MassHunter\Data\2022ekim\061022cengizhoca\50ppb3.d	Calibration	2	x	272	50.0000	5.4490
D:\MassHunter\Data\2022ekim\061022cengizhoca\100ppb1.d	Calibration	3	x	388	100.0000	3.8834
D:\MassHunter\Data\2022ekim\061022cengizhoca\100ppb2.d	Calibration	3	x	379	100.0000	3.7901
D:\MassHunter\Data\2022ekim\061022cengizhoca\100ppb3.d	Calibration	3	x	394	100.0000	3.9444
D:\MassHunter\Data\2022ekim\061022cengizhoca\250ppb1.d	Calibration	4	x	942	250.0000	3.7679
D:\MassHunter\Data\2022ekim\061022cengizhoca\250ppb2.d	Calibration	4	x	884	250.0000	3.5362
D:\MassHunter\Data\2022ekim\061022cengizhoca\250ppb3.d	Calibration	4	x	807	250.0000	3.2275
D:\MassHunter\Data\2022ekim\061022cengizhoca\500ppb1.d	Calibration	5	x	1941	500.0000	3.8815
D:\MassHunter\Data\2022ekim\061022cengizhoca\500ppb2.d	Calibration	5	x	1761	500.0000	3.5212
D:\MassHunter\Data\2022ekim\061022cengizhoca\500ppb3.d	Calibration	5	x	1724	500.0000	3.4481
D:\MassHunter\Data\2022ekim\061022cengizhoca\1000ppb1.d	Calibration	6	x	3457	1000.0000	3.4572
D:\MassHunter\Data\2022ekim\061022cengizhoca\1000ppb2.d	Calibration	6	x	3313	1000.0000	3.3127
D:\MassHunter\Data\2022ekim\061022cengizhoca\1000ppb3.d	Calibration	6	x	3356	1000.0000	3.3564

Quantitative Analysis Complete Report

**Batch Path**	D:\MassHunter\Data\2022ekim\061022cengizhoca\QuantResults\071022.batch.bin		
**Analysis Time**	10/11/2022 1:33:26 PM	**Analyst Name**	Defam-PC\admin
**Report Time**	10/11/2022 1:33:47 PM	**Reporter Name**	admin
**Last Calib Update**	10/11/2022 1:33:17 PM	**Batch State**	Processed
**Quant Batch Version**	B.07.01	**Quant Report Version**	B.07.01

Verbascoside

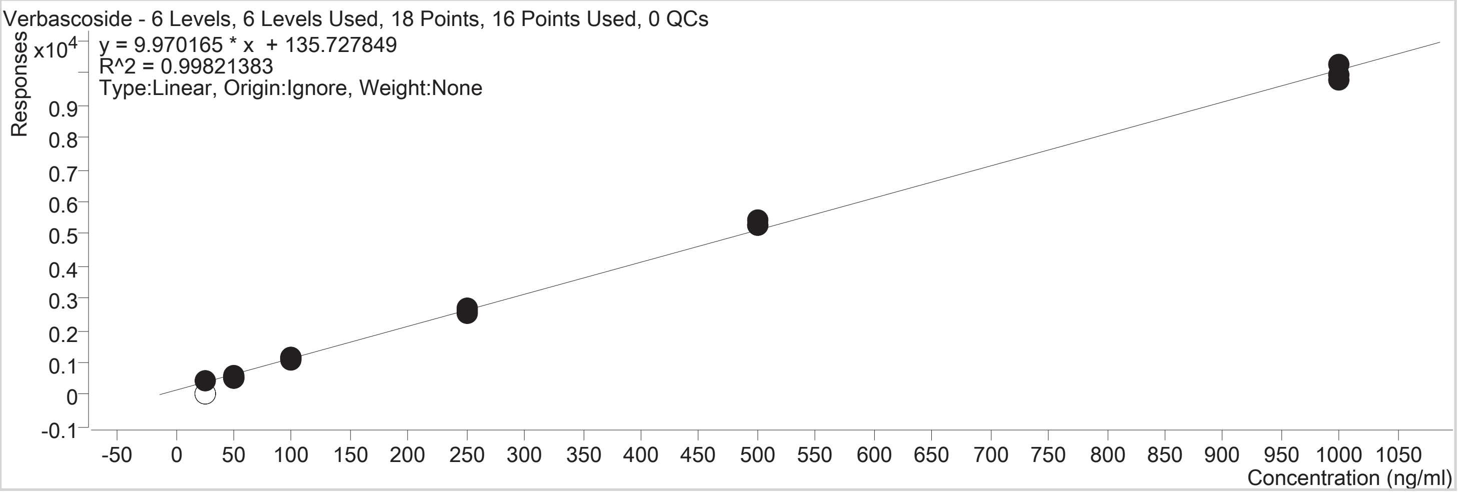

Calibration STD Path	Cal Type	Level	Enabled	Resp.	Exp. Conc	Resp. Factor
D:\MassHunter\Data\2022ekim\061022cengizhoca\25ppb1.d	Calibration	1		44	25.0000	1.7489
D:\MassHunter\Data\2022ekim\061022cengizhoca\25ppb2.d	Calibration	1		9	25.0000	0.3701
D:\MassHunter\Data\2022ekim\061022cengizhoca\25ppb3.d	Calibration	1	x	395	25.0000	15.8170
D:\MassHunter\Data\2022ekim\061022cengizhoca\50ppb1.d	Calibration	2	x	585	50.0000	11.6944
D:\MassHunter\Data\2022ekim\061022cengizhoca\50ppb2.d	Calibration	2	x	529	50.0000	10.5715
D:\MassHunter\Data\2022ekim\061022cengizhoca\50ppb3.d	Calibration	2	x	517	50.0000	10.3480
D:\MassHunter\Data\2022ekim\061022cengizhoca\100ppb1.d	Calibration	3	x	1102	100.0000	11.0168
D:\MassHunter\Data\2022ekim\061022cengizhoca\100ppb2.d	Calibration	3	x	1133	100.0000	11.3308
D:\MassHunter\Data\2022ekim\061022cengizhoca\100ppb3.d	Calibration	3	x	1182	100.0000	11.8175
D:\MassHunter\Data\2022ekim\061022cengizhoca\250ppb1.d	Calibration	4	x	2508	250.0000	10.0337
D:\MassHunter\Data\2022ekim\061022cengizhoca\250ppb2.d	Calibration	4	x	2607	250.0000	10.4277
D:\MassHunter\Data\2022ekim\061022cengizhoca\250ppb3.d	Calibration	4	x	2670	250.0000	10.6793
D:\MassHunter\Data\2022ekim\061022cengizhoca\500ppb1.d	Calibration	5	x	5431	500.0000	10.8610
D:\MassHunter\Data\2022ekim\061022cengizhoca\500ppb2.d	Calibration	5	x	5287	500.0000	10.5740
D:\MassHunter\Data\2022ekim\061022cengizhoca\500ppb3.d	Calibration	5	x	5258	500.0000	10.5159
D:\MassHunter\Data\2022ekim\061022cengizhoca\1000ppb1.d	Calibration	6	x	10297	1000.0000	10.2970
D:\MassHunter\Data\2022ekim\061022cengizhoca\1000ppb2.d	Calibration	6	x	9803	1000.0000	9.8035
D:\MassHunter\Data\2022ekim\061022cengizhoca\1000ppb3.d	Calibration	6	x	9947	1000.0000	9.9471

Quantitative Analysis Complete Report

**Batch Path**	D:\MassHunter\Data\2022ekim\061022cengizhoca\QuantResults\071022.batch.bin		
**Analysis Time**	10/11/2022 1:33:26 PM	**Analyst Name**	Defam-PC\admin
**Report Time**	10/11/2022 1:33:47 PM	**Reporter Name**	admin
**Last Calib Update**	10/11/2022 1:33:17 PM	**Batch State**	Processed
**Quant Batch Version**	B.07.01	**Quant Report Version**	B.07.01

Taxifolin

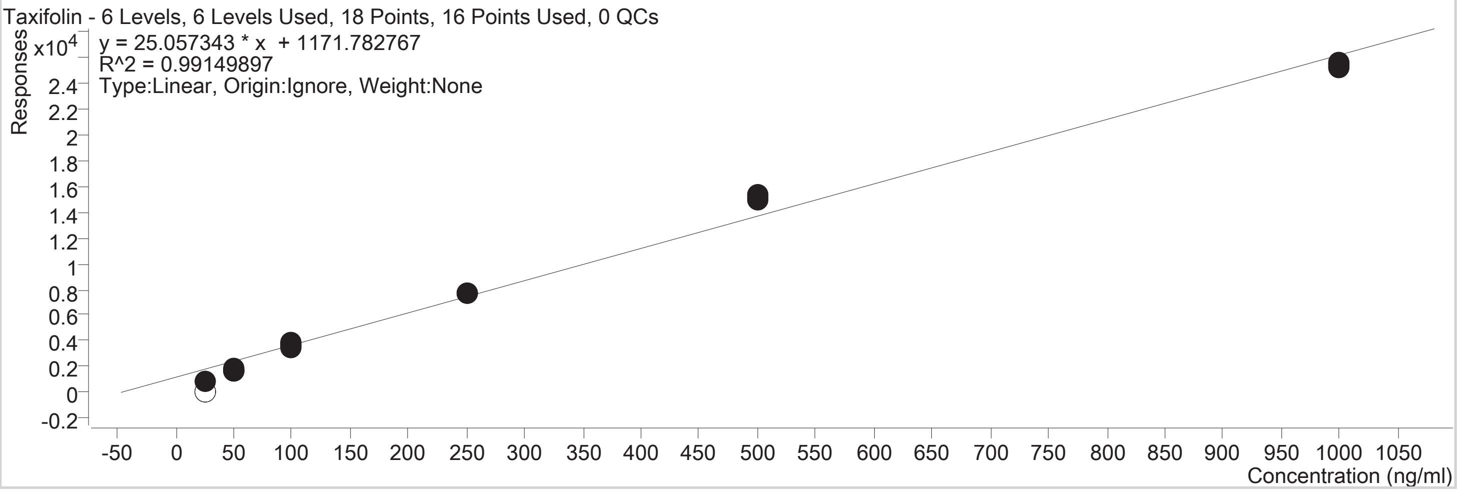

Calibration STD Path	Cal Type	Level	Enabled	Resp.	Exp. Conc	Resp. Factor
D:\MassHunter\Data\2022ekim\061022cengizhoca\25ppb1.d	Calibration	1		3	25.0000	0.1014
D:\MassHunter\Data\2022ekim\061022cengizhoca\25ppb2.d	Calibration	1		2	25.0000	0.0852
D:\MassHunter\Data\2022ekim\061022cengizhoca\25ppb3.d	Calibration	1	x	932	25.0000	37.2661
D:\MassHunter\Data\2022ekim\061022cengizhoca\50ppb1.d	Calibration	2	x	1923	50.0000	38.4524
D:\MassHunter\Data\2022ekim\061022cengizhoca\50ppb2.d	Calibration	2	x	1650	50.0000	32.9988
D:\MassHunter\Data\2022ekim\061022cengizhoca\50ppb3.d	Calibration	2	x	1717	50.0000	34.3352
D:\MassHunter\Data\2022ekim\061022cengizhoca\100ppb1.d	Calibration	3	x	3803	100.0000	38.0277
D:\MassHunter\Data\2022ekim\061022cengizhoca\100ppb2.d	Calibration	3	x	3529	100.0000	35.2894
D:\MassHunter\Data\2022ekim\061022cengizhoca\100ppb3.d	Calibration	3	x	3736	100.0000	37.3602
D:\MassHunter\Data\2022ekim\061022cengizhoca\250ppb1.d	Calibration	4	x	7653	250.0000	30.6122
D:\MassHunter\Data\2022ekim\061022cengizhoca\250ppb2.d	Calibration	4	x	7668	250.0000	30.6705
D:\MassHunter\Data\2022ekim\061022cengizhoca\250ppb3.d	Calibration	4	x	7709	250.0000	30.8341
D:\MassHunter\Data\2022ekim\061022cengizhoca\500ppb1.d	Calibration	5	x	15077	500.0000	30.1532
D:\MassHunter\Data\2022ekim\061022cengizhoca\500ppb2.d	Calibration	5	x	14931	500.0000	29.8624
D:\MassHunter\Data\2022ekim\061022cengizhoca\500ppb3.d	Calibration	5	x	15410	500.0000	30.8192
D:\MassHunter\Data\2022ekim\061022cengizhoca\1000ppb1.d	Calibration	6	x	25309	1000.0000	25.3090
D:\MassHunter\Data\2022ekim\061022cengizhoca\1000ppb2.d	Calibration	6	x	25452	1000.0000	25.4524
D:\MassHunter\Data\2022ekim\061022cengizhoca\1000ppb3.d	Calibration	6	x	25705	1000.0000	25.7052

**Batch Path**	D:\MassHunter\Data\2022ekim\061022cengizhoca\QuantResults\071022.batch.bin		
**Analysis Time**	10/11/2022 1:33:26 PM	**Analyst Name**	Defam-PC\admin
**Report Time**	10/11/2022 1:33:47 PM	**Reporter Name**	admin
**Last Calib Update**	10/11/2022 1:33:17 PM	**Batch State**	Processed
**Quant Batch Version**	B.07.01	**Quant Report Version**	B.07.01

p-Coumaric acid

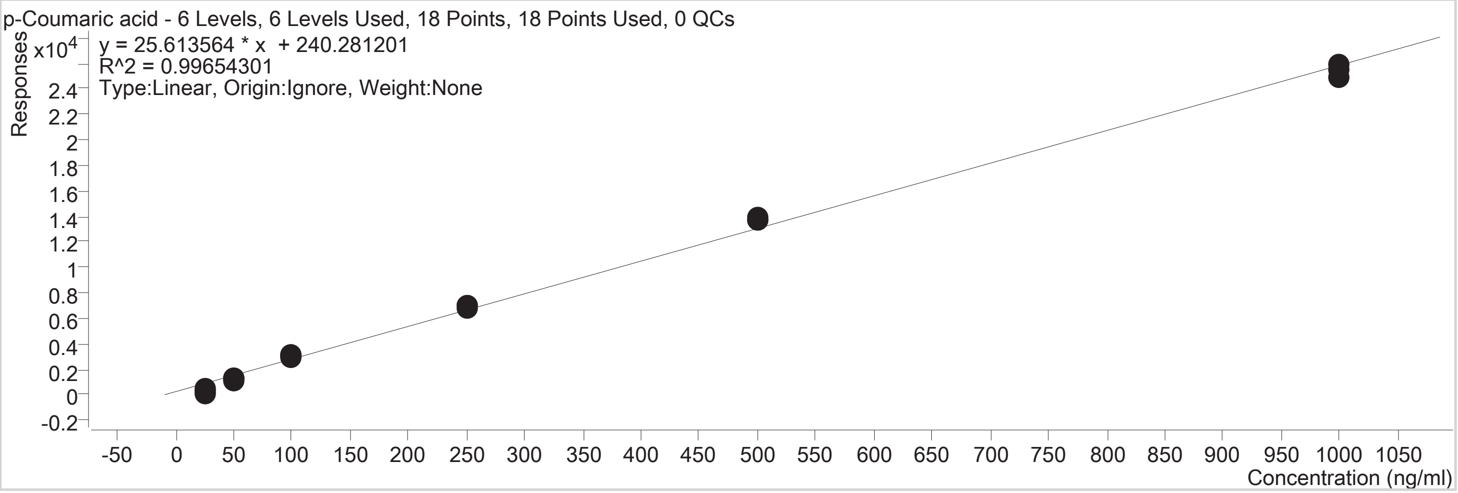

Calibration STD Path	Cal Type	Level	Enabled	Resp.	Exp. Conc	Resp. Factor
D:\MassHunter\Data\2022ekim\061022cengizhoca\25ppb1.d	Calibration	1	x	29	25.0000	1.1436
D:\MassHunter\Data\2022ekim\061022cengizhoca\25ppb2.d	Calibration	1	x	243	25.0000	9.7214
D:\MassHunter\Data\2022ekim\061022cengizhoca\25ppb3.d	Calibration	1	x	486	25.0000	19.4217
D:\MassHunter\Data\2022ekim\061022cengizhoca\50ppb1.d	Calibration	2	x	1106	50.0000	22.1128
D:\MassHunter\Data\2022ekim\061022cengizhoca\50ppb2.d	Calibration	2	x	1400	50.0000	27.9976
D:\MassHunter\Data\2022ekim\061022cengizhoca\50ppb3.d	Calibration	2	x	1381	50.0000	27.6226
D:\MassHunter\Data\2022ekim\061022cengizhoca\100ppb1.d	Calibration	3	x	3027	100.0000	30.2711
D:\MassHunter\Data\2022ekim\061022cengizhoca\100ppb2.d	Calibration	3	x	3079	100.0000	30.7885
D:\MassHunter\Data\2022ekim\061022cengizhoca\100ppb3.d	Calibration	3	x	3173	100.0000	31.7343
D:\MassHunter\Data\2022ekim\061022cengizhoca\250ppb1.d	Calibration	4	x	6865	250.0000	27.4587
D:\MassHunter\Data\2022ekim\061022cengizhoca\250ppb2.d	Calibration	4	x	6933	250.0000	27.7303
D:\MassHunter\Data\2022ekim\061022cengizhoca\250ppb3.d	Calibration	4	x	6909	250.0000	27.6348
D:\MassHunter\Data\2022ekim\061022cengizhoca\500ppb1.d	Calibration	5	x	13899	500.0000	27.7970
D:\MassHunter\Data\2022ekim\061022cengizhoca\500ppb2.d	Calibration	5	x	13748	500.0000	27.4964
D:\MassHunter\Data\2022ekim\061022cengizhoca\500ppb3.d	Calibration	5	x	13718	500.0000	27.4361
D:\MassHunter\Data\2022ekim\061022cengizhoca\1000ppb1.d	Calibration	6	x	25973	1000.0000	25.9730
D:\MassHunter\Data\2022ekim\061022cengizhoca\1000ppb2.d	Calibration	6	x	24851	1000.0000	24.8508
D:\MassHunter\Data\2022ekim\061022cengizhoca\1000ppb3.d	Calibration	6	x	25426	1000.0000	25.4256

Quantitative Analysis Complete Report

Agilent Technologies

**Batch Path**	D:\MassHunter\Data\2022ekim\061022cengizhoca\QuantResults\071022.batch.bin		
**Analysis Time**	10/11/2022 1:33:26 PM	**Analyst Name**	Defam-PC\admin
**Report Time**	10/11/2022 1:33:47 PM	**Reporter Name**	admin
**Last Calib Update**	10/11/2022 1:33:17 PM	**Batch State**	Processed
**Quant Batch Version**	B.07.01	**Quant Report Version**	B.07.01

Sinapic acid

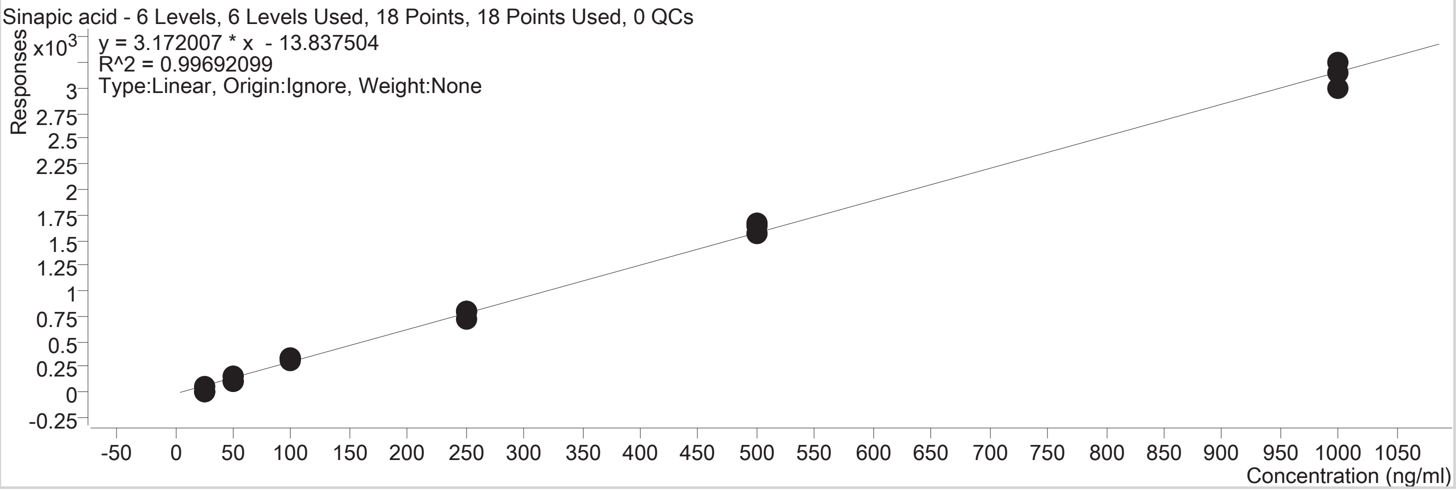

Calibration STD Path	Cal Type	Level	Enabled	Resp.	Exp. Conc	Resp. Factor
D:\MassHunter\Data\2022ekim\061022cengizhoca\25ppb1.d	Calibration	1	x	1	25.0000	0.0241
D:\MassHunter\Data\2022ekim\061022cengizhoca\25ppb2.d	Calibration	1	x	16	25.0000	0.6318
D:\MassHunter\Data\2022ekim\061022cengizhoca\25ppb3.d	Calibration	1	x	50	25.0000	1.9841
D:\MassHunter\Data\2022ekim\061022cengizhoca\50ppb1.d	Calibration	2	x	120	50.0000	2.4008
D:\MassHunter\Data\2022ekim\061022cengizhoca\50ppb2.d	Calibration	2	x	119	50.0000	2.3807
D:\MassHunter\Data\2022ekim\061022cengizhoca\50ppb3.d	Calibration	2	x	151	50.0000	3.0143
D:\MassHunter\Data\2022ekim\061022cengizhoca\100ppb1.d	Calibration	3	x	344	100.0000	3.4395
D:\MassHunter\Data\2022ekim\061022cengizhoca\100ppb2.d	Calibration	3	x	323	100.0000	3.2284
D:\MassHunter\Data\2022ekim\061022cengizhoca\100ppb3.d	Calibration	3	x	343	100.0000	3.4337
D:\MassHunter\Data\2022ekim\061022cengizhoca\250ppb1.d	Calibration	4	x	735	250.0000	2.9382
D:\MassHunter\Data\2022ekim\061022cengizhoca\250ppb2.d	Calibration	4	x	803	250.0000	3.2137
D:\MassHunter\Data\2022ekim\061022cengizhoca\250ppb3.d	Calibration	4	x	796	250.0000	3.1856
D:\MassHunter\Data\2022ekim\061022cengizhoca\500ppb1.d	Calibration	5	x	1571	500.0000	3.1425
D:\MassHunter\Data\2022ekim\061022cengizhoca\500ppb2.d	Calibration	5	x	1652	500.0000	3.3041
D:\MassHunter\Data\2022ekim\061022cengizhoca\500ppb3.d	Calibration	5	x	1657	500.0000	3.3131
D:\MassHunter\Data\2022ekim\061022cengizhoca\1000ppb1.d	Calibration	6	x	3258	1000.0000	3.2581
D:\MassHunter\Data\2022ekim\061022cengizhoca\1000ppb2.d	Calibration	6	x	3138	1000.0000	3.1382
D:\MassHunter\Data\2022ekim\061022cengizhoca\1000ppb3.d	Calibration	6	x	2993	1000.0000	2.9927

Quantitative Analysis Complete Report

Agilent Technologies

**Batch Path**	D:\MassHunter\Data\2022ekim\061022cengizhoca\QuantResults\071022.batch.bin		
**Analysis Time**	10/11/2022 1:33:26 PM	**Analyst Name**	Defam-PC\admin
**Report Time**	10/11/2022 1:33:47 PM	**Reporter Name**	admin
**Last Calib Update**	10/11/2022 1:33:17 PM	**Batch State**	Processed
**Quant Batch Version**	B.07.01	**Quant Report Version**	B.07.01

Ferulic acid

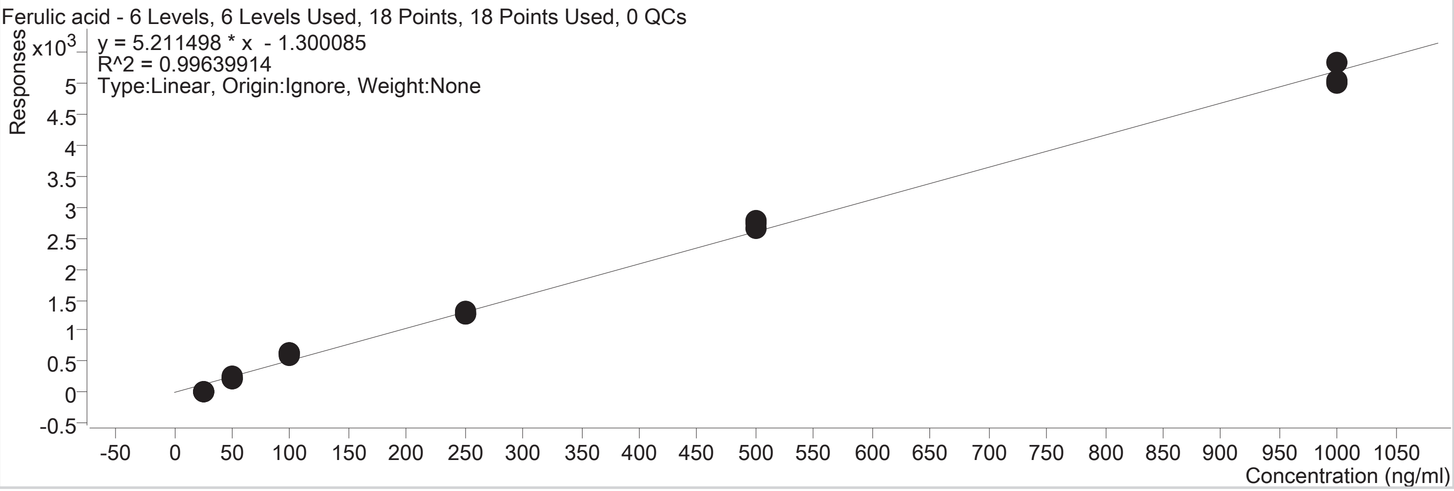

Calibration STD Path	Cal Type	Level	Enabled	Resp.	Exp. Conc	Resp. Factor
D:\MassHunter\Data\2022ekim\061022cengizhoca\25ppb1.d	Calibration	1	x	2	25.0000	0.0661
D:\MassHunter\Data\2022ekim\061022cengizhoca\25ppb2.d	Calibration	1	x	3	25.0000	0.1106
D:\MassHunter\Data\2022ekim\061022cengizhoca\25ppb3.d	Calibration	1	x	9	25.0000	0.3754
D:\MassHunter\Data\2022ekim\061022cengizhoca\50ppb1.d	Calibration	2	x	222	50.0000	4.4308
D:\MassHunter\Data\2022ekim\061022cengizhoca\50ppb2.d	Calibration	2	x	244	50.0000	4.8754
D:\MassHunter\Data\2022ekim\061022cengizhoca\50ppb3.d	Calibration	2	x	226	50.0000	4.5292
D:\MassHunter\Data\2022ekim\061022cengizhoca\100ppb1.d	Calibration	3	x	617	100.0000	6.1715
D:\MassHunter\Data\2022ekim\061022cengizhoca\100ppb2.d	Calibration	3	x	648	100.0000	6.4769
D:\MassHunter\Data\2022ekim\061022cengizhoca\100ppb3.d	Calibration	3	x	637	100.0000	6.3659
D:\MassHunter\Data\2022ekim\061022cengizhoca\250ppb1.d	Calibration	4	x	1264	250.0000	5.0548
D:\MassHunter\Data\2022ekim\061022cengizhoca\250ppb2.d	Calibration	4	x	1327	250.0000	5.3093
D:\MassHunter\Data\2022ekim\061022cengizhoca\250ppb3.d	Calibration	4	x	1281	250.0000	5.1229
D:\MassHunter\Data\2022ekim\061022cengizhoca\500ppb1.d	Calibration	5	x	2647	500.0000	5.2930
D:\MassHunter\Data\2022ekim\061022cengizhoca\500ppb2.d	Calibration	5	x	2724	500.0000	5.4485
D:\MassHunter\Data\2022ekim\061022cengizhoca\500ppb3.d	Calibration	5	x	2768	500.0000	5.5356
D:\MassHunter\Data\2022ekim\061022cengizhoca\1000ppb1.d	Calibration	6	x	5358	1000.0000	5.3580
D:\MassHunter\Data\2022ekim\061022cengizhoca\1000ppb2.d	Calibration	6	x	5066	1000.0000	5.0656
D:\MassHunter\Data\2022ekim\061022cengizhoca\1000ppb3.d	Calibration	6	x	5032	1000.0000	5.0320

Quantitative Analysis Complete Report

Agilent Technologies

**Batch Path**	D:\MassHunter\Data\2022ekim\061022cengizhoca\QuantResults\071022.batch.bin		
**Analysis Time**	10/11/2022 1:33:26 PM	**Analyst Name**	Defam-PC\admin
**Report Time**	10/11/2022 1:33:47 PM	**Reporter Name**	admin
**Last Calib Update**	10/11/2022 1:33:17 PM	**Batch State**	Processed
**Quant Batch Version**	B.07.01	**Quant Report Version**	B.07.01

Luteolin 7-glucoside

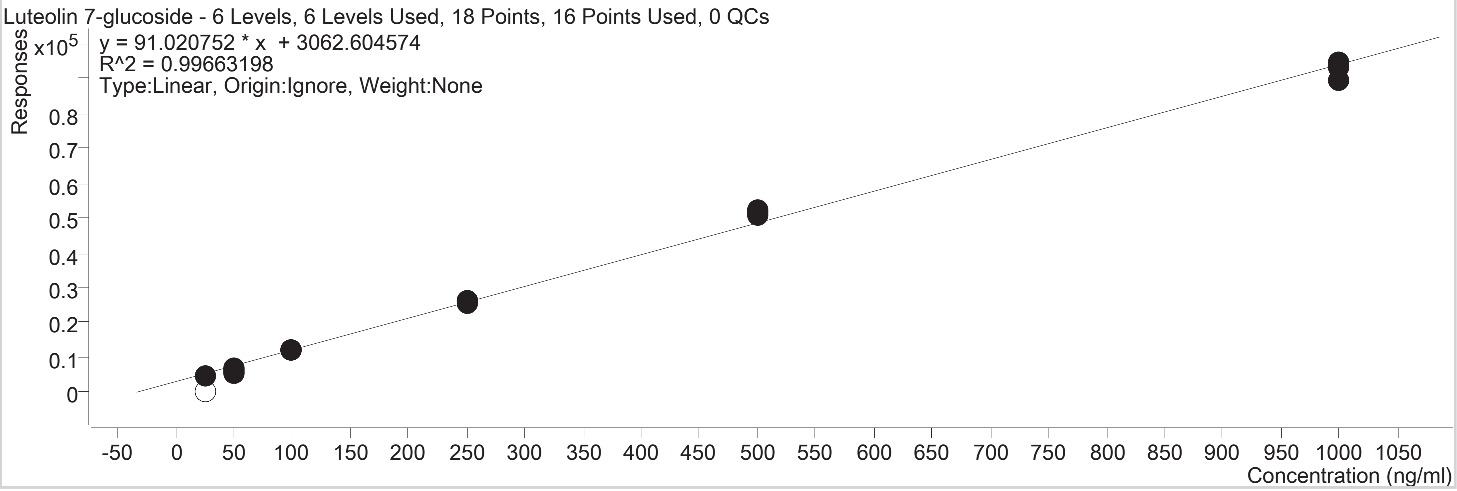

Calibration STD Path	Cal Type	Level	Enabled	Resp.	Exp. Conc	Resp. Factor
D:\MassHunter\Data\2022ekim\061022cengizhoca \25ppb1.d	Calibration	1		92	25.0000	3.6850
D:\MassHunter\Data\2022ekim\061022cengizhoca \25ppb2.d	Calibration	1		47	25.0000	1.8719
D:\MassHunter\Data\2022ekim\061022cengizhoca \25ppb3.d	Calibration	1	x	4730	25.0000	189.1984
D:\MassHunter\Data\2022ekim\061022cengizhoca \50ppb1.d	Calibration	2	x	7068	50.0000	141.3653
D:\MassHunter\Data\2022ekim\061022cengizhoca \50ppb2.d	Calibration	2	x	5992	50.0000	119.8403
D:\MassHunter\Data\2022ekim\061022cengizhoca \50ppb3.d	Calibration	2	x	5445	50.0000	108.9039
D:\MassHunter\Data\2022ekim\061022cengizhoca \100ppb1.d	Calibration	3	x	12136	100.0000	121.3622
D:\MassHunter\Data\2022ekim\061022cengizhoca \100ppb2.d	Calibration	3	x	11834	100.0000	118.3376
D:\MassHunter\Data\2022ekim\061022cengizhoca \100ppb3.d	Calibration	3	x	12280	100.0000	122.7989
D:\MassHunter\Data\2022ekim\061022cengizhoca \250ppb1.d	Calibration	4	x	25423	250.0000	101.6915
D:\MassHunter\Data\2022ekim\061022cengizhoca \250ppb2.d	Calibration	4	x	26577	250.0000	106.3096
D:\MassHunter\Data\2022ekim\061022cengizhoca \250ppb3.d	Calibration	4	x	26253	250.0000	105.0134
D:\MassHunter\Data\2022ekim\061022cengizhoca \500ppb1.d	Calibration	5	x	51277	500.0000	102.5548
D:\MassHunter\Data\2022ekim\061022cengizhoca \500ppb2.d	Calibration	5	x	51110	500.0000	102.2201
D:\MassHunter\Data\2022ekim\061022cengizhoca \500ppb3.d	Calibration	5	x	51994	500.0000	103.9878
D:\MassHunter\Data\2022ekim\061022cengizhoca \1000ppb1.d	Calibration	6	x	94912	1000.0000	94.9123
D:\MassHunter\Data\2022ekim\061022cengizhoca \1000ppb2.d	Calibration	6	x	89837	1000.0000	89.8366
D:\MassHunter\Data\2022ekim\061022cengizhoca \1000ppb3.d	Calibration	6	x	93226	1000.0000	93.2263

Quantitative Analysis Complete Report

**Batch Path**	D:\MassHunter\Data\2022ekim\061022cengizhoca\QuantResults\071022.batch.bin		
**Analysis Time**	10/11/2022 1:33:26 PM	**Analyst Name**	Defam-PC\admin
**Report Time**	10/11/2022 1:33:47 PM	**Reporter Name**	admin
**Last Calib Update**	10/11/2022 1:33:17 PM	**Batch State**	Processed
**Quant Batch Version**	B.07.01	**Quant Report Version**	B.07.01

Hesperidin

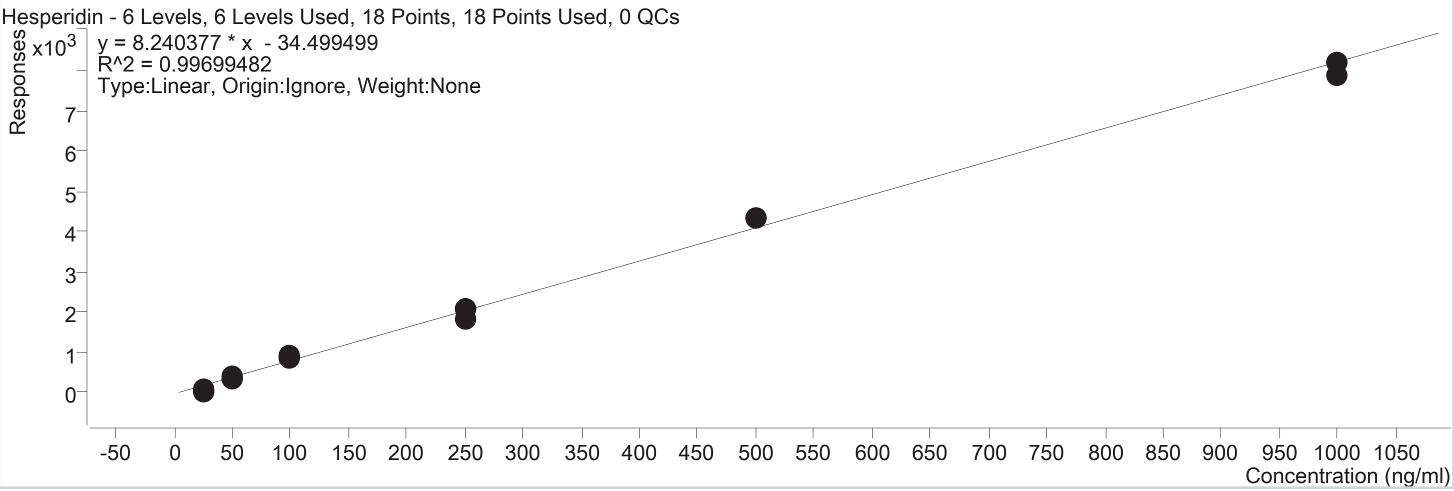

Calibration STD Path	Cal Type	Level	Enabled	Resp.	Exp. Conc	Resp. Factor
D:\MassHunter\Data\2022ekim\061022cengizhoca\25ppb1.d	Calibration	1	x	4	25.0000	0.1741
D:\MassHunter\Data\2022ekim\061022cengizhoca\25ppb2.d	Calibration	1	x	2	25.0000	0.0625
D:\MassHunter\Data\2022ekim\061022cengizhoca\25ppb3.d	Calibration	1	x	99	25.0000	3.9530
D:\MassHunter\Data\2022ekim\061022cengizhoca\50ppb1.d	Calibration	2	x	308	50.0000	6.1688
D:\MassHunter\Data\2022ekim\061022cengizhoca\50ppb2.d	Calibration	2	x	328	50.0000	6.5567
D:\MassHunter\Data\2022ekim\061022cengizhoca\50ppb3.d	Calibration	2	x	391	50.0000	7.8101
D:\MassHunter\Data\2022ekim\061022cengizhoca\100ppb1.d	Calibration	3	x	823	100.0000	8.2270
D:\MassHunter\Data\2022ekim\061022cengizhoca\100ppb2.d	Calibration	3	x	877	100.0000	8.7749
D:\MassHunter\Data\2022ekim\061022cengizhoca\100ppb3.d	Calibration	3	x	925	100.0000	9.2459
D:\MassHunter\Data\2022ekim\061022cengizhoca\250ppb1.d	Calibration	4	x	2063	250.0000	8.2516
D:\MassHunter\Data\2022ekim\061022cengizhoca\250ppb2.d	Calibration	4	x	1805	250.0000	7.2190
D:\MassHunter\Data\2022ekim\061022cengizhoca\250ppb3.d	Calibration	4	x	2096	250.0000	8.3833
D:\MassHunter\Data\2022ekim\061022cengizhoca\500ppb1.d	Calibration	5	x	4319	500.0000	8.6387
D:\MassHunter\Data\2022ekim\061022cengizhoca\500ppb2.d	Calibration	5	x	4313	500.0000	8.6250
D:\MassHunter\Data\2022ekim\061022cengizhoca\500ppb3.d	Calibration	5	x	4334	500.0000	8.6683
D:\MassHunter\Data\2022ekim\061022cengizhoca\1000ppb1.d	Calibration	6	x	8206	1000.0000	8.2062
D:\MassHunter\Data\2022ekim\061022cengizhoca\1000ppb2.d	Calibration	6	x	7866	1000.0000	7.8663
D:\MassHunter\Data\2022ekim\061022cengizhoca\1000ppb3.d	Calibration	6	x	8209	1000.0000	8.2088

Quantitative Analysis Complete Report

**Batch Path**	D:\MassHunter\Data\2022ekim\061022cengizhoca\QuantResults\071022.batch.bin		
**Analysis Time**	10/11/2022 1:33:26 PM	**Analyst Name**	Defam-PC\admin
**Report Time**	10/11/2022 1:33:47 PM	**Reporter Name**	admin
**Last Calib Update**	10/11/2022 1:33:17 PM	**Batch State**	Processed
**Quant Batch Version**	B.07.01	**Quant Report Version**	B.07.01

Hyperoside

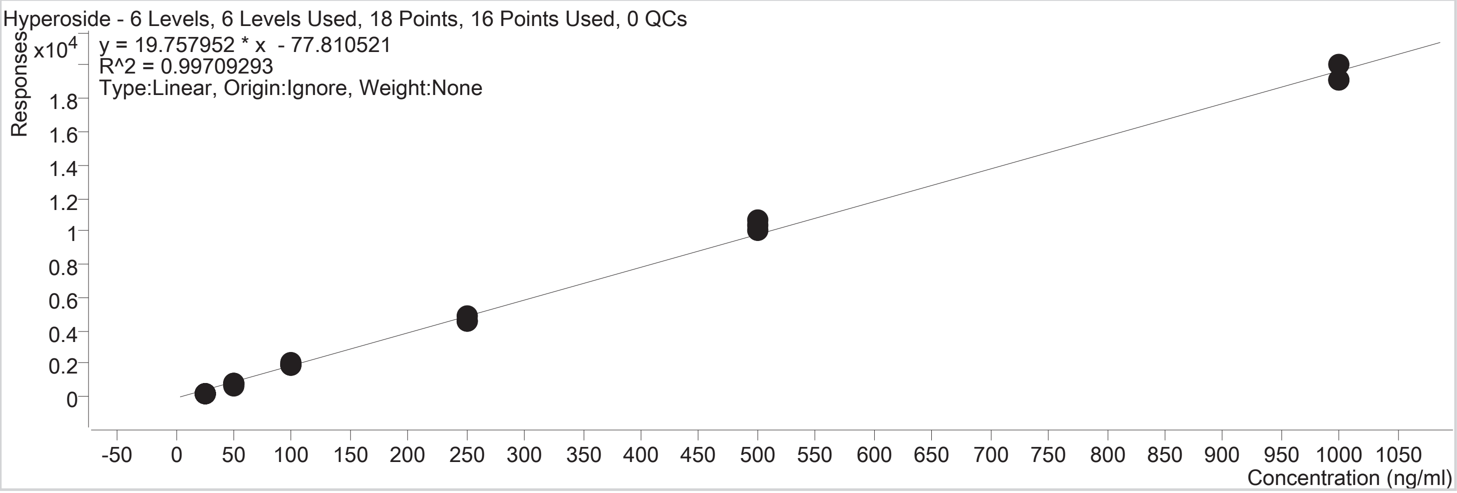

Calibration STD Path	Cal Type	Level	Enabled	Resp.	Exp. Conc	Resp. Factor
D:\MassHunter\Data\2022ekim\061022cengizhoca\25ppb1.d	Calibration	1		177	25.0000	7.0636
D:\MassHunter\Data\2022ekim\061022cengizhoca\25ppb2.d	Calibration	1		155	25.0000	6.2022
D:\MassHunter\Data\2022ekim\061022cengizhoca\25ppb3.d	Calibration	1	x	196	25.0000	7.8435
D:\MassHunter\Data\2022ekim\061022cengizhoca\50ppb1.d	Calibration	2	x	655	50.0000	13.0939
D:\MassHunter\Data\2022ekim\061022cengizhoca\50ppb2.d	Calibration	2	x	744	50.0000	14.8753
D:\MassHunter\Data\2022ekim\061022cengizhoca\50ppb3.d	Calibration	2	x	799	50.0000	15.9811
D:\MassHunter\Data\2022ekim\061022cengizhoca\100ppb1.d	Calibration	3	x	1959	100.0000	19.5855
D:\MassHunter\Data\2022ekim\061022cengizhoca\100ppb2.d	Calibration	3	x	1966	100.0000	19.6648
D:\MassHunter\Data\2022ekim\061022cengizhoca\100ppb3.d	Calibration	3	x	2113	100.0000	21.1279
D:\MassHunter\Data\2022ekim\061022cengizhoca\250ppb1.d	Calibration	4	x	4890	250.0000	19.5592
D:\MassHunter\Data\2022ekim\061022cengizhoca\250ppb2.d	Calibration	4	x	4636	250.0000	18.5456
D:\MassHunter\Data\2022ekim\061022cengizhoca\250ppb3.d	Calibration	4	x	4525	250.0000	18.0987
D:\MassHunter\Data\2022ekim\061022cengizhoca\500ppb1.d	Calibration	5	x	10086	500.0000	20.1728
D:\MassHunter\Data\2022ekim\061022cengizhoca\500ppb2.d	Calibration	5	x	10310	500.0000	20.6205
D:\MassHunter\Data\2022ekim\061022cengizhoca\500ppb3.d	Calibration	5	x	10630	500.0000	21.2609
D:\MassHunter\Data\2022ekim\061022cengizhoca\1000ppb1.d	Calibration	6	x	20105	1000.0000	20.1051
D:\MassHunter\Data\2022ekim\061022cengizhoca\1000ppb2.d	Calibration	6	x	19114	1000.0000	19.1137
D:\MassHunter\Data\2022ekim\061022cengizhoca\1000ppb3.d	Calibration	6	x	19141	1000.0000	19.1411

Quantitative Analysis Complete Report

Agilent Technologies

**Batch Path**	D:\MassHunter\Data\2022ekim\061022cengizhoca\QuantResults\071022.batch.bin		
**Analysis Time**	10/11/2022 1:33:26 PM	**Analyst Name**	Defam-PC\admin
**Report Time**	10/11/2022 1:33:47 PM	**Reporter Name**	admin
**Last Calib Update**	10/11/2022 1:33:17 PM	**Batch State**	Processed
**Quant Batch Version**	B.07.01	**Quant Report Version**	B.07.01

Rosmarinic acid

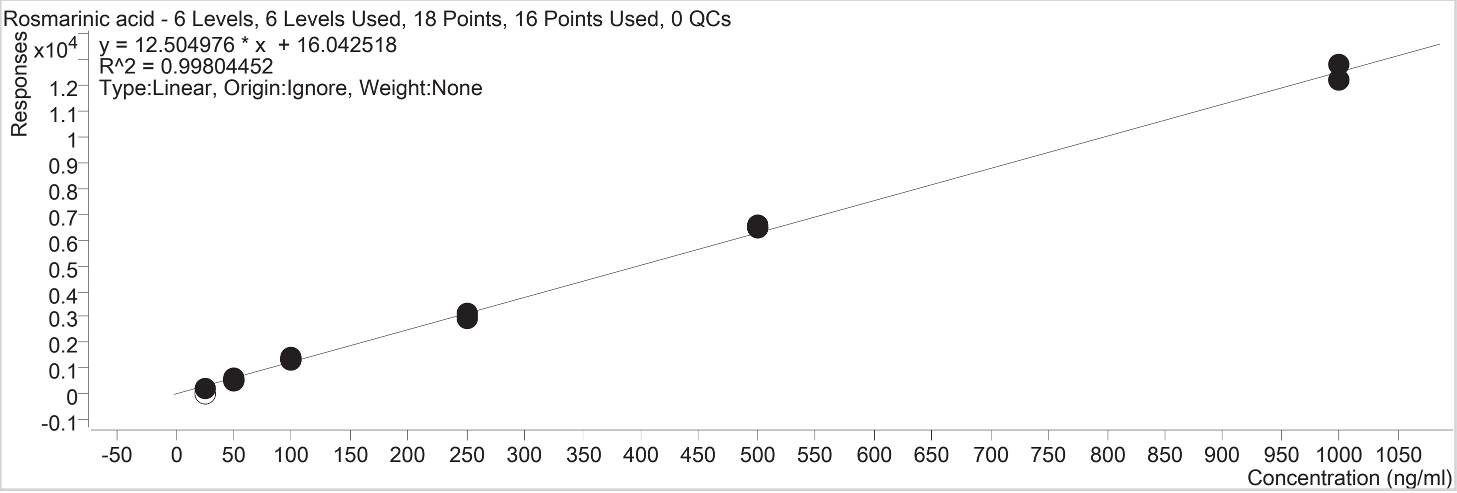

Calibration STD Path	Cal Type	Level	Enabled	Resp.	Exp. Conc	Resp. Factor
D:\MassHunter\Data\2022ekim\061022cengizhoca\25ppb1.d	Calibration	1		13	25.0000	0.5023
D:\MassHunter\Data\2022ekim\061022cengizhoca\25ppb2.d	Calibration	1		2	25.0000	0.0866
D:\MassHunter\Data\2022ekim\061022cengizhoca\25ppb3.d	Calibration	1	x	207	25.0000	8.2740
D:\MassHunter\Data\2022ekim\061022cengizhoca\50ppb1.d	Calibration	2	x	517	50.0000	10.3375
D:\MassHunter\Data\2022ekim\061022cengizhoca\50ppb2.d	Calibration	2	x	537	50.0000	10.7397
D:\MassHunter\Data\2022ekim\061022cengizhoca\50ppb3.d	Calibration	2	x	600	50.0000	11.9930
D:\MassHunter\Data\2022ekim\061022cengizhoca\100ppb1.d	Calibration	3	x	1331	100.0000	13.3066
D:\MassHunter\Data\2022ekim\061022cengizhoca\100ppb2.d	Calibration	3	x	1299	100.0000	12.9890
D:\MassHunter\Data\2022ekim\061022cengizhoca\100ppb3.d	Calibration	3	x	1445	100.0000	14.4522
D:\MassHunter\Data\2022ekim\061022cengizhoca\250ppb1.d	Calibration	4	x	3025	250.0000	12.1002
D:\MassHunter\Data\2022ekim\061022cengizhoca\250ppb2.d	Calibration	4	x	3169	250.0000	12.6762
D:\MassHunter\Data\2022ekim\061022cengizhoca\250ppb3.d	Calibration	4	x	2932	250.0000	11.7278
D:\MassHunter\Data\2022ekim\061022cengizhoca\500ppb1.d	Calibration	5	x	6474	500.0000	12.9486
D:\MassHunter\Data\2022ekim\061022cengizhoca\500ppb2.d	Calibration	5	x	6530	500.0000	13.0608
D:\MassHunter\Data\2022ekim\061022cengizhoca\500ppb3.d	Calibration	5	x	6508	500.0000	13.0161
D:\MassHunter\Data\2022ekim\061022cengizhoca\1000ppb1.d	Calibration	6	x	12837	1000.0000	12.8370
D:\MassHunter\Data\2022ekim\061022cengizhoca\1000ppb2.d	Calibration	6	x	12233	1000.0000	12.2331
D:\MassHunter\Data\2022ekim\061022cengizhoca\1000ppb3.d	Calibration	6	x	12204	1000.0000	12.2036

Agilent Technologies

**Batch Path**	D:\MassHunter\Data\2022ekim\061022cengizhoca\QuantResults\071022.batch.bin		
**Analysis Time**	10/11/2022 1:33:26 PM	**Analyst Name**	Defam-PC\admin
**Report Time**	10/11/2022 1:33:47 PM	**Reporter Name**	admin
**Last Calib Update**	10/11/2022 1:33:17 PM	**Batch State**	Processed
**Quant Batch Version**	B.07.01	**Quant Report Version**	B.07.01

Apigenin 7-glucoside

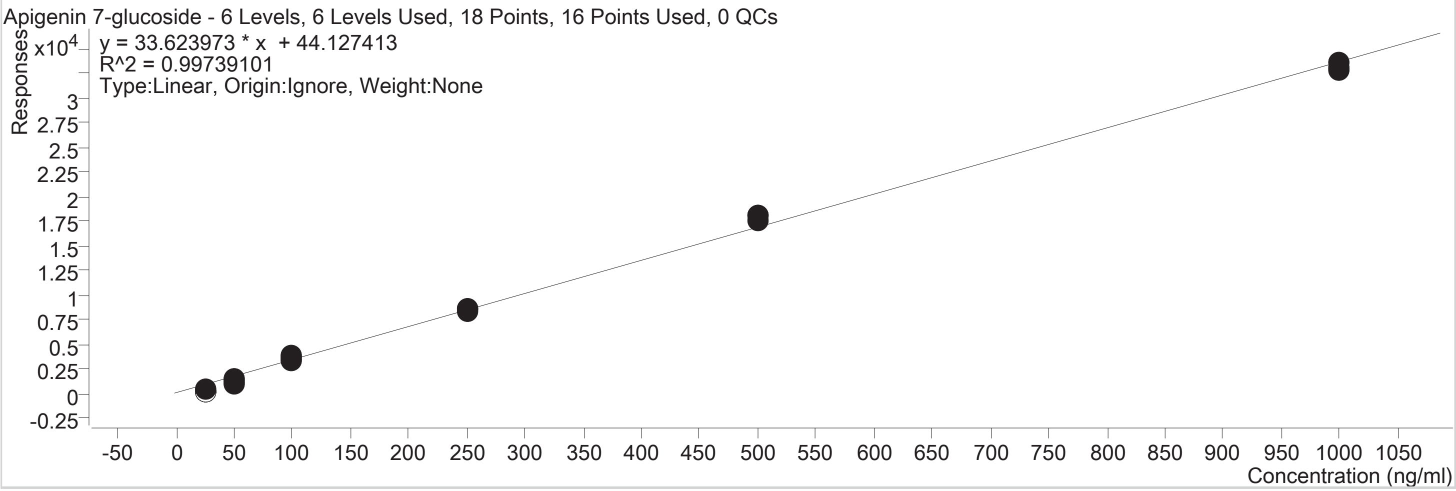

Calibration STD Path	Cal Type	Level	Enabled	Resp.	Exp. Conc	Resp. Factor
D:\MassHunter\Data\2022ekim\061022cengizhoca\25ppb1.d	Calibration	1		79	25.0000	3.1691
D:\MassHunter\Data\2022ekim\061022cengizhoca\25ppb2.d	Calibration	1		213	25.0000	8.5176
D:\MassHunter\Data\2022ekim\061022cengizhoca\25ppb3.d	Calibration	1	x	337	25.0000	13.4863
D:\MassHunter\Data\2022ekim\061022cengizhoca\50ppb1.d	Calibration	2	x	990	50.0000	19.8042
D:\MassHunter\Data\2022ekim\061022cengizhoca\50ppb2.d	Calibration	2	x	1120	50.0000	22.3969
D:\MassHunter\Data\2022ekim\061022cengizhoca\50ppb3.d	Calibration	2	x	1412	50.0000	28.2327
D:\MassHunter\Data\2022ekim\061022cengizhoca\100ppb1.d	Calibration	3	x	3368	100.0000	33.6802
D:\MassHunter\Data\2022ekim\061022cengizhoca\100ppb2.d	Calibration	3	x	3542	100.0000	35.4206
D:\MassHunter\Data\2022ekim\061022cengizhoca\100ppb3.d	Calibration	3	x	3776	100.0000	37.7570
D:\MassHunter\Data\2022ekim\061022cengizhoca\250ppb1.d	Calibration	4	x	8380	250.0000	33.5216
D:\MassHunter\Data\2022ekim\061022cengizhoca\250ppb2.d	Calibration	4	x	8641	250.0000	34.5640
D:\MassHunter\Data\2022ekim\061022cengizhoca\250ppb3.d	Calibration	4	x	8633	250.0000	34.5302
D:\MassHunter\Data\2022ekim\061022cengizhoca\500ppb1.d	Calibration	5	x	17437	500.0000	34.8746
D:\MassHunter\Data\2022ekim\061022cengizhoca\500ppb2.d	Calibration	5	x	17975	500.0000	35.9508
D:\MassHunter\Data\2022ekim\061022cengizhoca\500ppb3.d	Calibration	5	x	18072	500.0000	36.1440
D:\MassHunter\Data\2022ekim\061022cengizhoca\1000ppb1.d	Calibration	6	x	33663	1000.0000	33.6630
D:\MassHunter\Data\2022ekim\061022cengizhoca\1000ppb2.d	Calibration	6	x	32942	1000.0000	32.9419
D:\MassHunter\Data\2022ekim\061022cengizhoca\1000ppb3.d	Calibration	6	x	32915	1000.0000	32.9151

Quantitative Analysis Complete Report

**Batch Path**	D:\MassHunter\Data\2022ekim\061022cengizhoca\QuantResults\071022.batch.bin		
**Analysis Time**	10/11/2022 1:33:26 PM	**Analyst Name**	Defam-PC\admin
**Report Time**	10/11/2022 1:33:47 PM	**Reporter Name**	admin
**Last Calib Update**	10/11/2022 1:33:17 PM	**Batch State**	Processed
**Quant Batch Version**	B.07.01	**Quant Report Version**	B.07.01

Pinoresinol

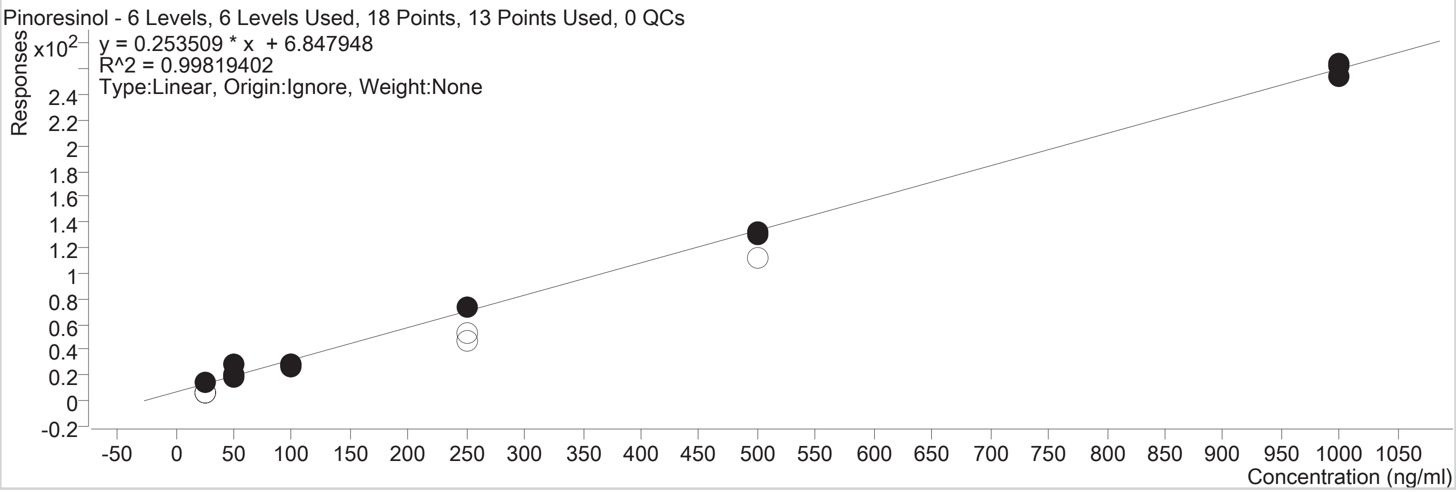

Calibration STD Path	Cal Type	Level	Enabled	Resp.	Exp. Conc	Resp. Factor
D:\MassHunter\Data\2022ekim\061022cengizhoca\25ppb1.d	Calibration	1		7	25.0000	0.2644
D:\MassHunter\Data\2022ekim\061022cengizhoca\25ppb2.d	Calibration	1		6	25.0000	0.2345
D:\MassHunter\Data\2022ekim\061022cengizhoca\25ppb3.d	Calibration	1	x	14	25.0000	0.5726
D:\MassHunter\Data\2022ekim\061022cengizhoca\50ppb1.d	Calibration	2	x	29	50.0000	0.5755
D:\MassHunter\Data\2022ekim\061022cengizhoca\50ppb2.d	Calibration	2	x	19	50.0000	0.3840
D:\MassHunter\Data\2022ekim\061022cengizhoca\50ppb3.d	Calibration	2	x	21	50.0000	0.4228
D:\MassHunter\Data\2022ekim\061022cengizhoca\100ppb1.d	Calibration	3	x	29	100.0000	0.2913
D:\MassHunter\Data\2022ekim\061022cengizhoca\100ppb2.d	Calibration	3	x	28	100.0000	0.2801
D:\MassHunter\Data\2022ekim\061022cengizhoca\100ppb3.d	Calibration	3	x	27	100.0000	0.2735
D:\MassHunter\Data\2022ekim\061022cengizhoca\250ppb1.d	Calibration	4	x	73	250.0000	0.2924
D:\MassHunter\Data\2022ekim\061022cengizhoca\250ppb2.d	Calibration	4		48	250.0000	0.1921
D:\MassHunter\Data\2022ekim\061022cengizhoca\250ppb3.d	Calibration	4		54	250.0000	0.2165
D:\MassHunter\Data\2022ekim\061022cengizhoca\500ppb1.d	Calibration	5	x	131	500.0000	0.2618
D:\MassHunter\Data\2022ekim\061022cengizhoca\500ppb2.d	Calibration	5	x	132	500.0000	0.2632
D:\MassHunter\Data\2022ekim\061022cengizhoca\500ppb3.d	Calibration	5		112	500.0000	0.2244
D:\MassHunter\Data\2022ekim\061022cengizhoca\1000ppb1.d	Calibration	6	x	263	1000.0000	0.2632
D:\MassHunter\Data\2022ekim\061022cengizhoca\1000ppb2.d	Calibration	6	x	255	1000.0000	0.2550
D:\MassHunter\Data\2022ekim\061022cengizhoca\1000ppb3.d	Calibration	6	x	265	1000.0000	0.2651

Quantitative Analysis Complete Report

**Batch Path**	D:\MassHunter\Data\2022ekim\061022cengizhoca\QuantResults\071022.batch.bin		
**Analysis Time**	10/11/2022 1:33:26 PM	**Analyst Name**	Defam-PC\admin
**Report Time**	10/11/2022 1:33:47 PM	**Reporter Name**	admin
**Last Calib Update**	10/11/2022 1:33:17 PM	**Batch State**	Processed
**Quant Batch Version**	B.07.01	**Quant Report Version**	B.07.01

2-Hydroxycinnamic acid

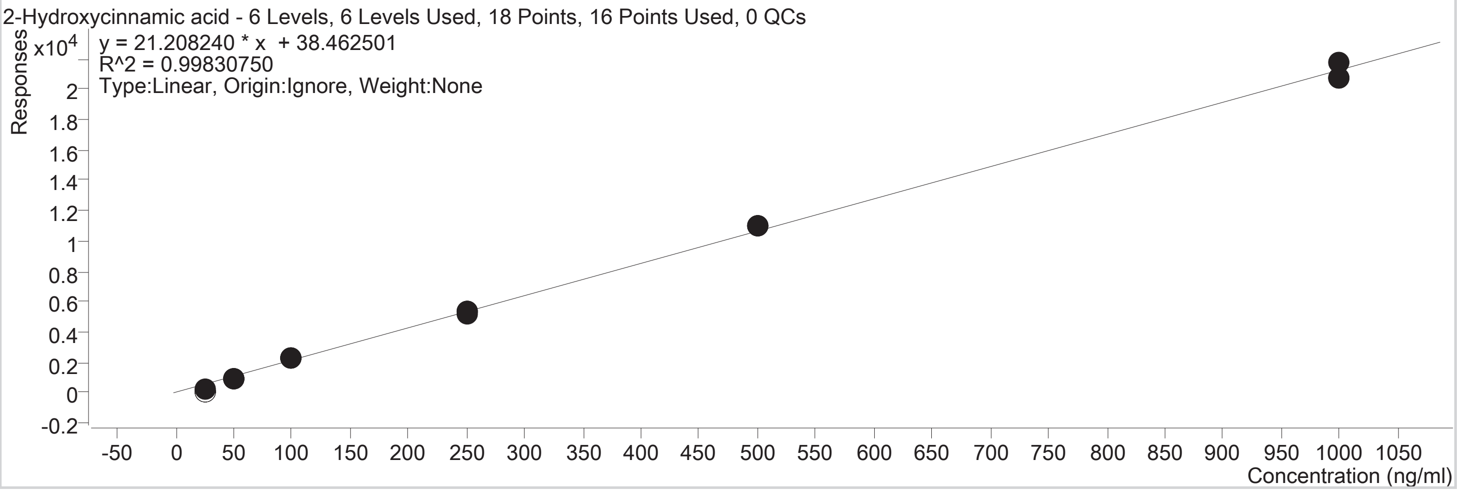

Calibration STD Path	Cal Type	Level	Enabled	Resp.	Exp. Conc	Resp. Factor
D:\MassHunter\Data\2022ekim\061022cengizhoca\25ppb1.d	Calibration	1		119	25.0000	4.7704
D:\MassHunter\Data\2022ekim\061022cengizhoca\25ppb2.d	Calibration	1		31	25.0000	1.2390
D:\MassHunter\Data\2022ekim\061022cengizhoca\25ppb3.d	Calibration	1	x	322	25.0000	12.8784
D:\MassHunter\Data\2022ekim\061022cengizhoca\50ppb1.d	Calibration	2	x	879	50.0000	17.5750
D:\MassHunter\Data\2022ekim\061022cengizhoca\50ppb2.d	Calibration	2	x	900	50.0000	18.0089
D:\MassHunter\Data\2022ekim\061022cengizhoca\50ppb3.d	Calibration	2	x	952	50.0000	19.0387
D:\MassHunter\Data\2022ekim\061022cengizhoca\100ppb1.d	Calibration	3	x	2320	100.0000	23.2014
D:\MassHunter\Data\2022ekim\061022cengizhoca\100ppb2.d	Calibration	3	x	2217	100.0000	22.1699
D:\MassHunter\Data\2022ekim\061022cengizhoca\100ppb3.d	Calibration	3	x	2296	100.0000	22.9592
D:\MassHunter\Data\2022ekim\061022cengizhoca\250ppb1.d	Calibration	4	x	5293	250.0000	21.1740
D:\MassHunter\Data\2022ekim\061022cengizhoca\250ppb2.d	Calibration	4	x	5239	250.0000	20.9570
D:\MassHunter\Data\2022ekim\061022cengizhoca\250ppb3.d	Calibration	4	x	5392	250.0000	21.5693
D:\MassHunter\Data\2022ekim\061022cengizhoca\500ppb1.d	Calibration	5	x	11011	500.0000	22.0223
D:\MassHunter\Data\2022ekim\061022cengizhoca\500ppb2.d	Calibration	5	x	11001	500.0000	22.0025
D:\MassHunter\Data\2022ekim\061022cengizhoca\500ppb3.d	Calibration	5	x	10976	500.0000	21.9525
D:\MassHunter\Data\2022ekim\061022cengizhoca\1000ppb1.d	Calibration	6	x	21790	1000.0000	21.7899
D:\MassHunter\Data\2022ekim\061022cengizhoca\1000ppb2.d	Calibration	6	x	20803	1000.0000	20.8032
D:\MassHunter\Data\2022ekim\061022cengizhoca\1000ppb3.d	Calibration	6	x	20640	1000.0000	20.6395

Quantitative Analysis Complete Report

Agilent Technologies

**Batch Path**	D:\MassHunter\Data\2022ekim\061022cengizhoca\QuantResults\071022.batch.bin		
**Analysis Time**	10/11/2022 1:33:26 PM	**Analyst Name**	Defam-PC\admin
**Report Time**	10/11/2022 1:33:47 PM	**Reporter Name**	admin
**Last Calib Update**	10/11/2022 1:33:17 PM	**Batch State**	Processed
**Quant Batch Version**	B.07.01	**Quant Report Version**	B.07.01

Eriodictyol

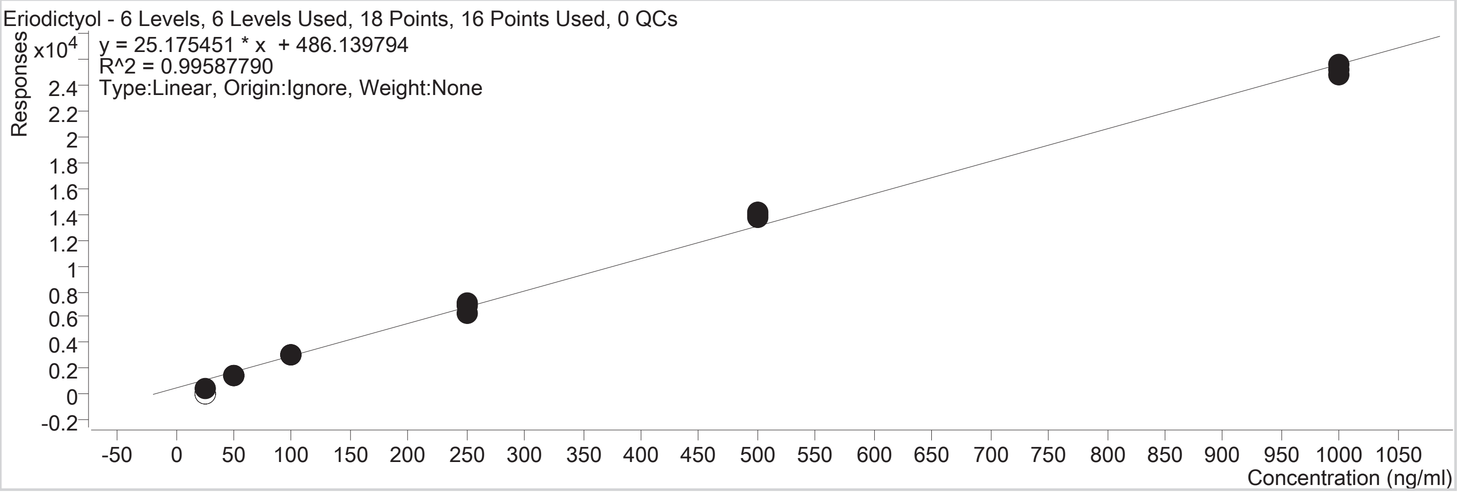

Calibration STD Path	Cal Type	Level	Enabled	Resp.	Exp. Conc	Resp. Factor
D:\MassHunter\Data\2022ekim\061022cengizhoca\25ppb1.d	Calibration	1		3	25.0000	0.1211
D:\MassHunter\Data\2022ekim\061022cengizhoca\25ppb2.d	Calibration	1		5	25.0000	0.1931
D:\MassHunter\Data\2022ekim\061022cengizhoca\25ppb3.d	Calibration	1	x	482	25.0000	19.2740
D:\MassHunter\Data\2022ekim\061022cengizhoca\50ppb1.d	Calibration	2	x	1372	50.0000	27.4328
D:\MassHunter\Data\2022ekim\061022cengizhoca\50ppb2.d	Calibration	2	x	1379	50.0000	27.5876
D:\MassHunter\Data\2022ekim\061022cengizhoca\50ppb3.d	Calibration	2	x	1421	50.0000	28.4170
D:\MassHunter\Data\2022ekim\061022cengizhoca\100ppb1.d	Calibration	3	x	3048	100.0000	30.4820
D:\MassHunter\Data\2022ekim\061022cengizhoca\100ppb2.d	Calibration	3	x	3082	100.0000	30.8163
D:\MassHunter\Data\2022ekim\061022cengizhoca\100ppb3.d	Calibration	3	x	3150	100.0000	31.5015
D:\MassHunter\Data\2022ekim\061022cengizhoca\250ppb1.d	Calibration	4	x	6962	250.0000	27.8463
D:\MassHunter\Data\2022ekim\061022cengizhoca\250ppb2.d	Calibration	4	x	7007	250.0000	28.0280
D:\MassHunter\Data\2022ekim\061022cengizhoca\250ppb3.d	Calibration	4	x	6297	250.0000	25.1878
D:\MassHunter\Data\2022ekim\061022cengizhoca\500ppb1.d	Calibration	5	x	14052	500.0000	28.1045
D:\MassHunter\Data\2022ekim\061022cengizhoca\500ppb2.d	Calibration	5	x	14212	500.0000	28.4237
D:\MassHunter\Data\2022ekim\061022cengizhoca\500ppb3.d	Calibration	5	x	13840	500.0000	27.6806
D:\MassHunter\Data\2022ekim\061022cengizhoca\1000ppb1.d	Calibration	6	x	25696	1000.0000	25.6965
D:\MassHunter\Data\2022ekim\061022cengizhoca\1000ppb2.d	Calibration	6	x	25161	1000.0000	25.1613
D:\MassHunter\Data\2022ekim\061022cengizhoca\1000ppb3.d	Calibration	6	x	24746	1000.0000	24.7463

Quantitative Analysis Complete Report

**Batch Path**	D:\MassHunter\Data\2022ekim\061022cengizhoca\QuantResults\071022.batch.bin		
**Analysis Time**	10/11/2022 1:33:26 PM	**Analyst Name**	Defam-PC\admin
**Report Time**	10/11/2022 1:33:47 PM	**Reporter Name**	admin
**Last Calib Update**	10/11/2022 1:33:17 PM	**Batch State**	Processed
**Quant Batch Version**	B.07.01	**Quant Report Version**	B.07.01

Quercetin

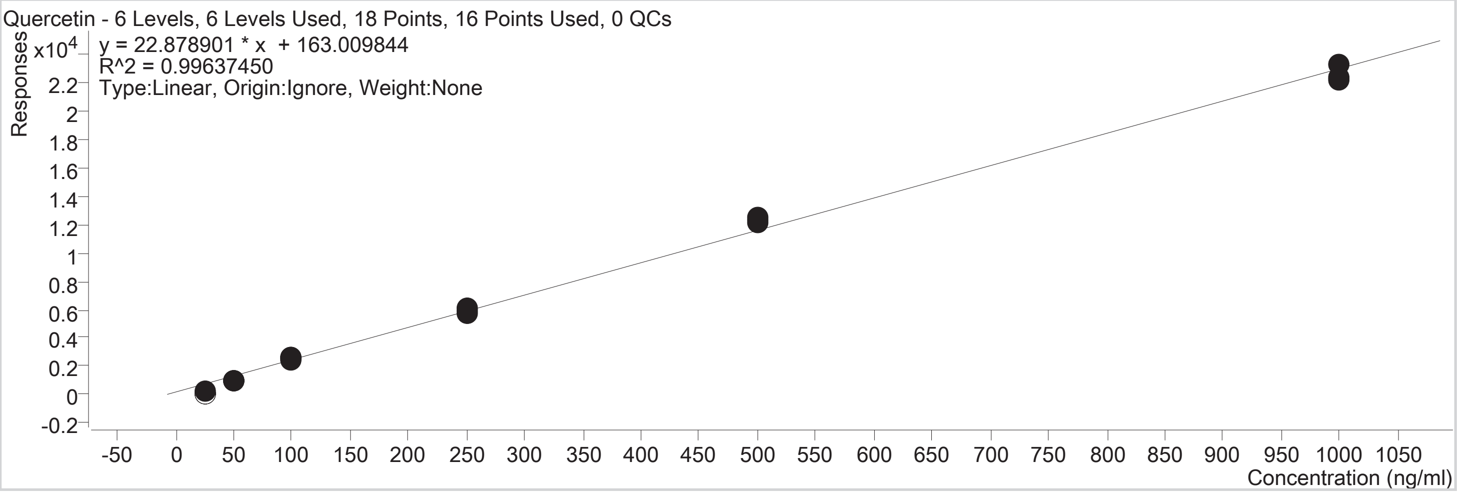

Calibration STD Path	Cal Type	Level	Enabled	Resp.	Exp. Conc	Resp. Factor
D:\MassHunter\Data\2022ekim\061022cengizhoca\25ppb1.d	Calibration	1		14	25.0000	0.5670
D:\MassHunter\Data\2022ekim\061022cengizhoca\25ppb2.d	Calibration	1		2	25.0000	0.0624
D:\MassHunter\Data\2022ekim\061022cengizhoca\25ppb3.d	Calibration	1	x	290	25.0000	11.5855
D:\MassHunter\Data\2022ekim\061022cengizhoca\50ppb1.d	Calibration	2	x	954	50.0000	19.0710
D:\MassHunter\Data\2022ekim\061022cengizhoca\50ppb2.d	Calibration	2	x	1023	50.0000	20.4693
D:\MassHunter\Data\2022ekim\061022cengizhoca\50ppb3.d	Calibration	2	x	1025	50.0000	20.4907
D:\MassHunter\Data\2022ekim\061022cengizhoca\100ppb1.d	Calibration	3	x	2412	100.0000	24.1182
D:\MassHunter\Data\2022ekim\061022cengizhoca\100ppb2.d	Calibration	3	x	2527	100.0000	25.2680
D:\MassHunter\Data\2022ekim\061022cengizhoca\100ppb3.d	Calibration	3	x	2534	100.0000	25.3377
D:\MassHunter\Data\2022ekim\061022cengizhoca\250ppb1.d	Calibration	4	x	5693	250.0000	22.7719
D:\MassHunter\Data\2022ekim\061022cengizhoca\250ppb2.d	Calibration	4	x	5892	250.0000	23.5662
D:\MassHunter\Data\2022ekim\061022cengizhoca\250ppb3.d	Calibration	4	x	6108	250.0000	24.4307
D:\MassHunter\Data\2022ekim\061022cengizhoca\500ppb1.d	Calibration	5	x	12336	500.0000	24.6713
D:\MassHunter\Data\2022ekim\061022cengizhoca\500ppb2.d	Calibration	5	x	12240	500.0000	24.4794
D:\MassHunter\Data\2022ekim\061022cengizhoca\500ppb3.d	Calibration	5	x	12566	500.0000	25.1314
D:\MassHunter\Data\2022ekim\061022cengizhoca\1000ppb1.d	Calibration	6	x	23388	1000.0000	23.3879
D:\MassHunter\Data\2022ekim\061022cengizhoca\1000ppb2.d	Calibration	6	x	22272	1000.0000	22.2717
D:\MassHunter\Data\2022ekim\061022cengizhoca\1000ppb3.d	Calibration	6	x	22333	1000.0000	22.3334

Quantitative Analysis Complete Report

Agilent Technologies

**Batch Path**	D:\MassHunter\Data\2022ekim\061022cengizhoca\QuantResults\071022.batch.bin		
**Analysis Time**	10/11/2022 1:33:26 PM	**Analyst Name**	Defam-PC\admin
**Report Time**	10/11/2022 1:33:47 PM	**Reporter Name**	admin
**Last Calib Update**	10/11/2022 1:33:17 PM	**Batch State**	Processed
**Quant Batch Version**	B.07.01	**Quant Report Version**	B.07.01

Luteolin

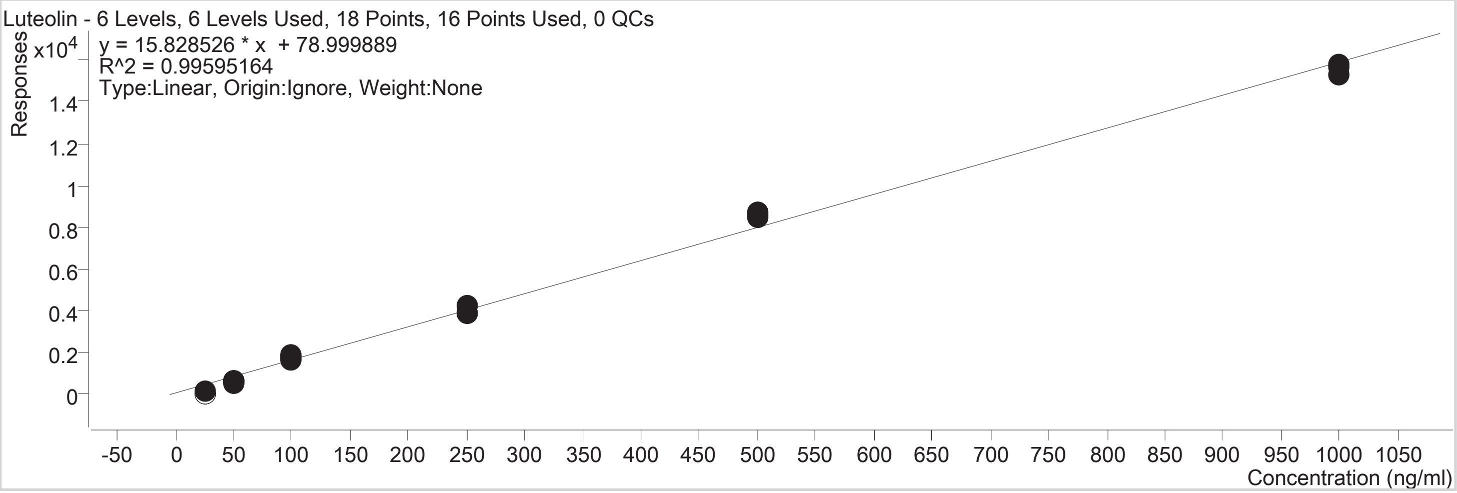

Calibration STD Path	Cal Type	Level	Enabled	Resp.	Exp. Conc	Resp. Factor
D:\MassHunter\Data\2022ekim\061022cengizhoca \25ppb1.d	Calibration	1		11	25.0000	0.4275
D:\MassHunter\Data\2022ekim\061022cengizhoca \25ppb2.d	Calibration	1		15	25.0000	0.5841
D:\MassHunter\Data\2022ekim\061022cengizhoca \25ppb3.d	Calibration	1	x	178	25.0000	7.1327
D:\MassHunter\Data\2022ekim\061022cengizhoca \50ppb1.d	Calibration	2	x	512	50.0000	10.2485
D:\MassHunter\Data\2022ekim\061022cengizhoca \50ppb2.d	Calibration	2	x	633	50.0000	12.6568
D:\MassHunter\Data\2022ekim\061022cengizhoca \50ppb3.d	Calibration	2	x	659	50.0000	13.1825
D:\MassHunter\Data\2022ekim\061022cengizhoca \100ppb1.d	Calibration	3	x	1619	100.0000	16.1907
D:\MassHunter\Data\2022ekim\061022cengizhoca \100ppb2.d	Calibration	3	x	1760	100.0000	17.6028
D:\MassHunter\Data\2022ekim\061022cengizhoca \100ppb3.d	Calibration	3	x	1849	100.0000	18.4856
D:\MassHunter\Data\2022ekim\061022cengizhoca \250ppb1.d	Calibration	4	x	3905	250.0000	15.6219
D:\MassHunter\Data\2022ekim\061022cengizhoca \250ppb2.d	Calibration	4	x	3846	250.0000	15.3854
D:\MassHunter\Data\2022ekim\061022cengizhoca \250ppb3.d	Calibration	4	x	4280	250.0000	17.1219
D:\MassHunter\Data\2022ekim\061022cengizhoca \500ppb1.d	Calibration	5	x	8430	500.0000	16.8602
D:\MassHunter\Data\2022ekim\061022cengizhoca \500ppb2.d	Calibration	5	x	8691	500.0000	17.3828
D:\MassHunter\Data\2022ekim\061022cengizhoca \500ppb3.d	Calibration	5	x	8647	500.0000	17.2945
D:\MassHunter\Data\2022ekim\061022cengizhoca \1000ppb1.d	Calibration	6	x	15722	1000.0000	15.7224
D:\MassHunter\Data\2022ekim\061022cengizhoca \1000ppb2.d	Calibration	6	x	15325	1000.0000	15.3253
D:\MassHunter\Data\2022ekim\061022cengizhoca \1000ppb3.d	Calibration	6	x	15823	1000.0000	15.8229

Quantitative Analysis Complete Report

**Batch Path**	D:\MassHunter\Data\2022ekim\061022cengizhoca\QuantResults\071022.batch.bin		
**Analysis Time**	10/11/2022 1:33:26 PM	**Analyst Name**	Defam-PC\admin
**Report Time**	10/11/2022 1:33:47 PM	**Reporter Name**	admin
**Last Calib Update**	10/11/2022 1:33:17 PM	**Batch State**	Processed
**Quant Batch Version**	B.07.01	**Quant Report Version**	B.07.01

Kaempferol

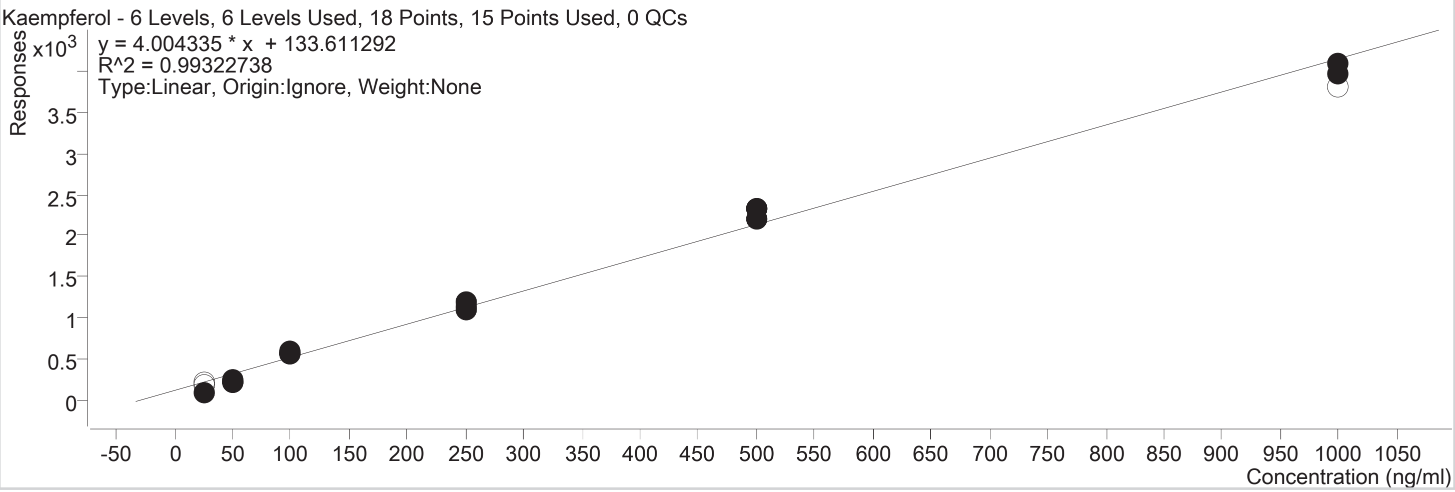

Calibration STD Path	Cal Type	Level	Enabled	Resp.	Exp. Conc	Resp. Factor
D:\MassHunter\Data\2022ekim\061022cengizhoca\25ppb1.d	Calibration	1		222	25.0000	8.8615
D:\MassHunter\Data\2022ekim\061022cengizhoca\25ppb2.d	Calibration	1		186	25.0000	7.4443
D:\MassHunter\Data\2022ekim\061022cengizhoca\25ppb3.d	Calibration	1	x	98	25.0000	3.9352
D:\MassHunter\Data\2022ekim\061022cengizhoca\50ppb1.d	Calibration	2	x	224	50.0000	4.4822
D:\MassHunter\Data\2022ekim\061022cengizhoca\50ppb2.d	Calibration	2	x	240	50.0000	4.8089
D:\MassHunter\Data\2022ekim\061022cengizhoca\50ppb3.d	Calibration	2	x	272	50.0000	5.4400
D:\MassHunter\Data\2022ekim\061022cengizhoca\100ppb1.d	Calibration	3	x	573	100.0000	5.7333
D:\MassHunter\Data\2022ekim\061022cengizhoca\100ppb2.d	Calibration	3	x	594	100.0000	5.9415
D:\MassHunter\Data\2022ekim\061022cengizhoca\100ppb3.d	Calibration	3	x	580	100.0000	5.7970
D:\MassHunter\Data\2022ekim\061022cengizhoca\250ppb1.d	Calibration	4	x	1210	250.0000	4.8414
D:\MassHunter\Data\2022ekim\061022cengizhoca\250ppb2.d	Calibration	4	x	1107	250.0000	4.4274
D:\MassHunter\Data\2022ekim\061022cengizhoca\250ppb3.d	Calibration	4	x	1138	250.0000	4.5530
D:\MassHunter\Data\2022ekim\061022cengizhoca\500ppb1.d	Calibration	5	x	2318	500.0000	4.6362
D:\MassHunter\Data\2022ekim\061022cengizhoca\500ppb2.d	Calibration	5	x	2325	500.0000	4.6506
D:\MassHunter\Data\2022ekim\061022cengizhoca\500ppb3.d	Calibration	5	x	2193	500.0000	4.3861
D:\MassHunter\Data\2022ekim\061022cengizhoca\1000ppb1.d	Calibration	6	x	3962	1000.0000	3.9615
D:\MassHunter\Data\2022ekim\061022cengizhoca\1000ppb2.d	Calibration	6		3807	1000.0000	3.8072
D:\MassHunter\Data\2022ekim\061022cengizhoca\1000ppb3.d	Calibration	6	x	4089	1000.0000	4.0891

Quantitative Analysis Complete Report

**Batch Path**	D:\MassHunter\Data\2022ekim\061022cengizhoca\QuantResults\071022.batch.bin		
**Analysis Time**	10/11/2022 1:33:26 PM	**Analyst Name**	Defam-PC\admin
**Report Time**	10/11/2022 1:33:47 PM	**Reporter Name**	admin
**Last Calib Update**	10/11/2022 1:33:17 PM	**Batch State**	Processed
**Quant Batch Version**	B.07.01	**Quant Report Version**	B.07.01

Apigenin

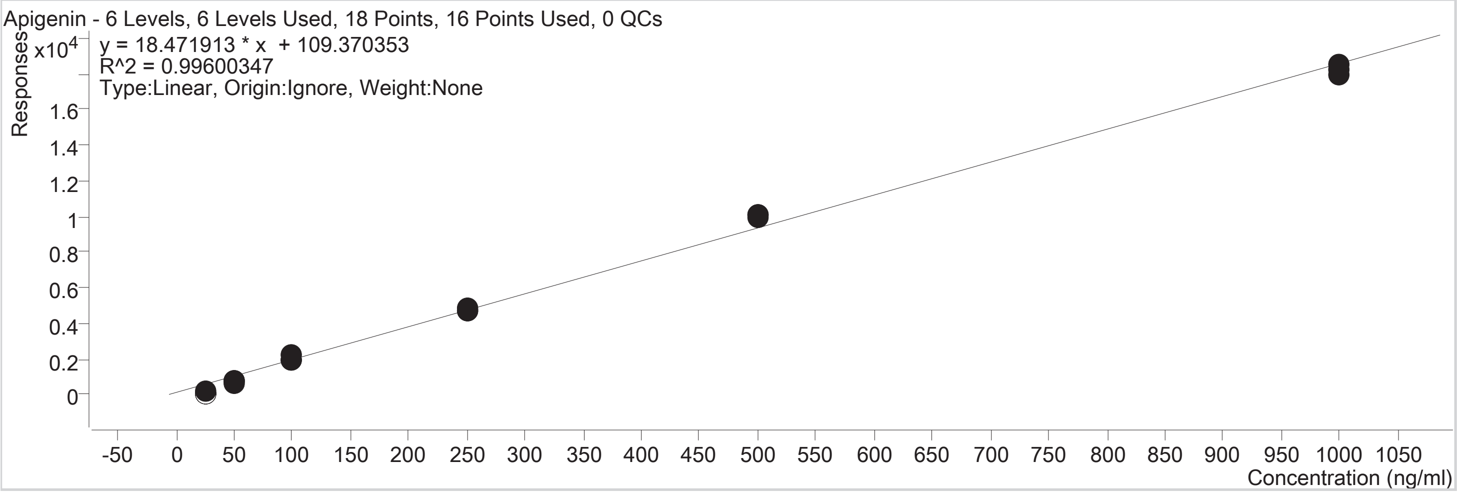

Calibration STD Path	Cal Type	Level	Enabled	Resp.	Exp. Conc	Resp. Factor
D:\MassHunter\Data\2022ekim\061022cengizhoca\25ppb1.d	Calibration	1		48	25.0000	1.9093
D:\MassHunter\Data\2022ekim\061022cengizhoca\25ppb2.d	Calibration	1		5	25.0000	0.2019
D:\MassHunter\Data\2022ekim\061022cengizhoca\25ppb3.d	Calibration	1	x	177	25.0000	7.0810
D:\MassHunter\Data\2022ekim\061022cengizhoca\50ppb1.d	Calibration	2	x	644	50.0000	12.8717
D:\MassHunter\Data\2022ekim\061022cengizhoca\50ppb2.d	Calibration	2	x	747	50.0000	14.9461
D:\MassHunter\Data\2022ekim\061022cengizhoca\50ppb3.d	Calibration	2	x	816	50.0000	16.3121
D:\MassHunter\Data\2022ekim\061022cengizhoca\100ppb1.d	Calibration	3	x	1895	100.0000	18.9542
D:\MassHunter\Data\2022ekim\061022cengizhoca\100ppb2.d	Calibration	3	x	1904	100.0000	19.0355
D:\MassHunter\Data\2022ekim\061022cengizhoca\100ppb3.d	Calibration	3	x	2155	100.0000	21.5505
D:\MassHunter\Data\2022ekim\061022cengizhoca\250ppb1.d	Calibration	4	x	4746	250.0000	18.9855
D:\MassHunter\Data\2022ekim\061022cengizhoca\250ppb2.d	Calibration	4	x	4753	250.0000	19.0123
D:\MassHunter\Data\2022ekim\061022cengizhoca\250ppb3.d	Calibration	4	x	4803	250.0000	19.2101
D:\MassHunter\Data\2022ekim\061022cengizhoca\500ppb1.d	Calibration	5	x	9884	500.0000	19.7674
D:\MassHunter\Data\2022ekim\061022cengizhoca\500ppb2.d	Calibration	5	x	10152	500.0000	20.3043
D:\MassHunter\Data\2022ekim\061022cengizhoca\500ppb3.d	Calibration	5	x	10133	500.0000	20.2652
D:\MassHunter\Data\2022ekim\061022cengizhoca\1000ppb1.d	Calibration	6	x	18564	1000.0000	18.5639
D:\MassHunter\Data\2022ekim\061022cengizhoca\1000ppb2.d	Calibration	6	x	18232	1000.0000	18.2321
D:\MassHunter\Data\2022ekim\061022cengizhoca\1000ppb3.d	Calibration	6	x	17898	1000.0000	17.8977

Quantitative Analysis Complete Report

**Batch Path**	D:\MassHunter\Data\2022ekim\061022cengizhoca\QuantResults\071022.batch.bin		
**Analysis Time**	10/11/2022 1:33:26 PM	**Analyst Name**	Defam-PC\admin
**Report Time**	10/11/2022 1:33:47 PM	**Reporter Name**	admin
**Last Calib Update**	10/11/2022 1:33:17 PM	**Batch State**	Processed
**Quant Batch Version**	B.07.01	**Quant Report Version**	B.07.01

**Acq. Time**	10/6/2022 3:33:30 PM	**Data File**	blank-061022-4.d
**Sample Type**	Sample	**Sample Name**	blank-061022-4
**Dilution**	1	**Acq. Method**	FENOLIK_DMRM2021-31bilesen.m

Sample Chromatogram

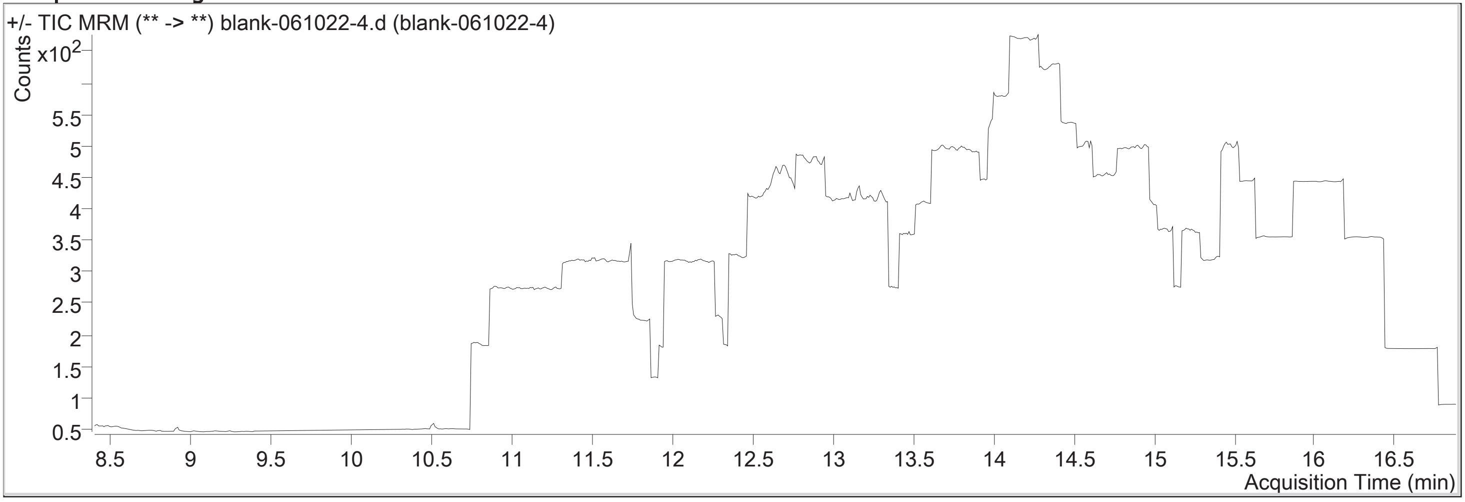

Compound	Transition	RT	Resp.	Final Conc	Units
Gallic acid	168.9 -> 125.0	8.925	8	ND	ng/ml
Protocatechuic acid	152.9 -> 108.9	10.518	16	ND	ng/ml
Pyrocatechol	109.0 -> 52.9	10.899	0	ND	ng/ml
3,4-Dihydroxyphenylacetic acid	167.0 -> 123.0	10.954	6	ND	ng/ml
(+)-Catechin	289.0 -> 245.0	11.277	4	ND	ng/ml
2,5-Dihydroxybenzoic acid	152.9 -> 109.0	11.963	0	0.5911	ng/ml
Chlorogenic acid	355.0 -> 163.0	11.785	3	5.9545	ng/ml
3-Hydroxybenzoic acid	137.0 -> 93.0	12.803	31	20.8467	ng/ml
4-Hydroxybenzoic acid	136.9 -> 93.1	12.653	167	ND	ng/ml
(-)-Epicatechin	291.0 -> 139.1	12.436	3	3.4570	ng/ml
Caffeic acid	179.0 -> 135.0	12.725	9	ND	ng/ml
Syringic acid	196.9 -> 181.9	12.773	0	3.5315	ng/ml
Vanillin	151.0 -> 136.0	13.078	3	ND	ng/ml
Verbascoside	623.0 -> 160.8	13.166	33	ND	ng/ml
Taxifolin	303.0 -> 285.1	13.695	1	ND	ng/ml
p-Coumaric acid	162.9 -> 119.0	13.758	13	ND	ng/ml
Sinapic acid	222.9 -> 207.9	13.806	11	7.8742	ng/ml
Ferulic acid	193.0 -> 134.0	13.950	0	0.2546	ng/ml
Luteolin 7-glucoside	447.1 -> 285.0	14.355	145	ND	ng/ml
Hesperidin	611.1 -> 303.0	14.319	0	4.2147	ng/ml
Hyperoside	465.1 -> 303.1	14.262	2	4.0406	ng/ml
Rosmarinic acid	359.0 -> 160.9	14.347	12	ND	ng/ml
Apigenin 7-glucoside	433.1 -> 271.0	14.772	38	ND	ng/ml
Pinoresinol	357.0 -> 151.0	14.767	4	ND	ng/ml
2-Hydroxycinnamic acid	162.9 -> 119.1	15.072	13	ND	ng/ml
Eriodictyol	287.0 -> 151.0	15.087	1	ND	ng/ml
Quercetin	301.0 -> 151.0	15.460	5	ND	ng/ml
Luteolin	287.0 -> 153.1	15.842	5	ND	ng/ml
Kaempferol	285.0 -> 229.1	15.779	120	ND	ng/ml

Quantitative Analysis Complete Report

Agilent Technologies

Compound	Transition	RT	Resp.	Final Conc	Units
Apigenin	271.0 -> 153.0	16.260	68	ND	ng/ml

Gallic acid

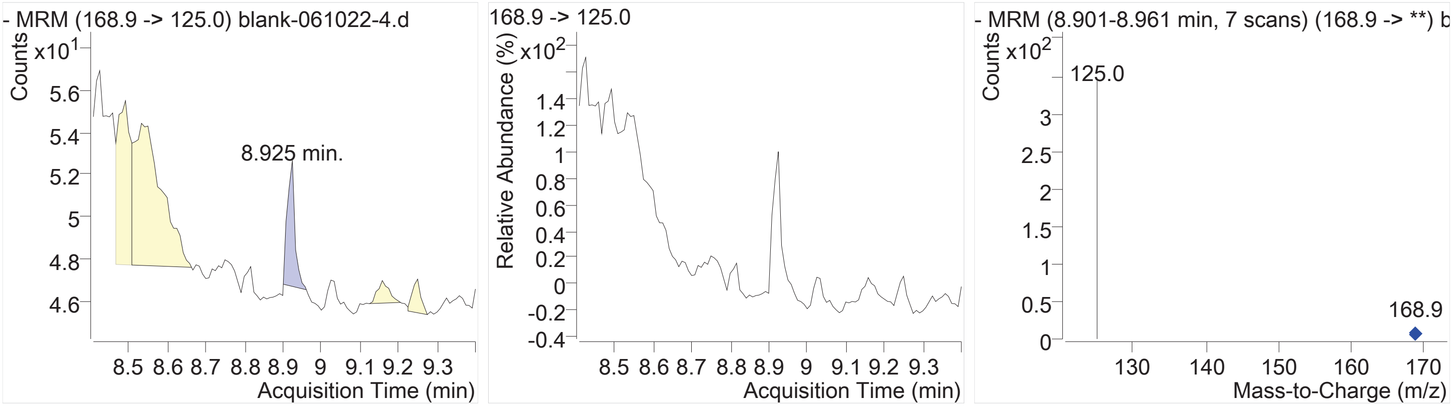

Protocatechuic acid

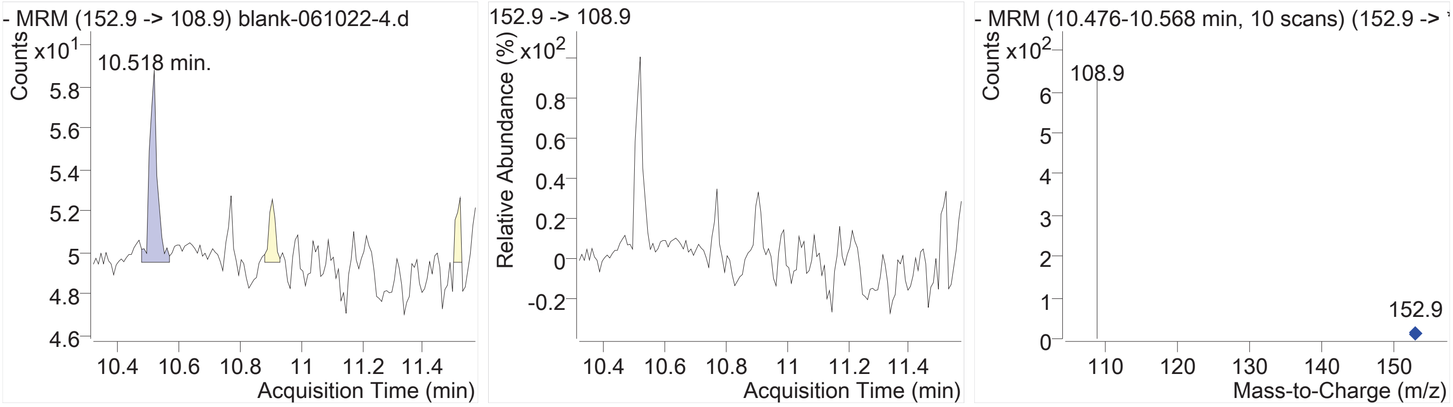

Pyrocatechol

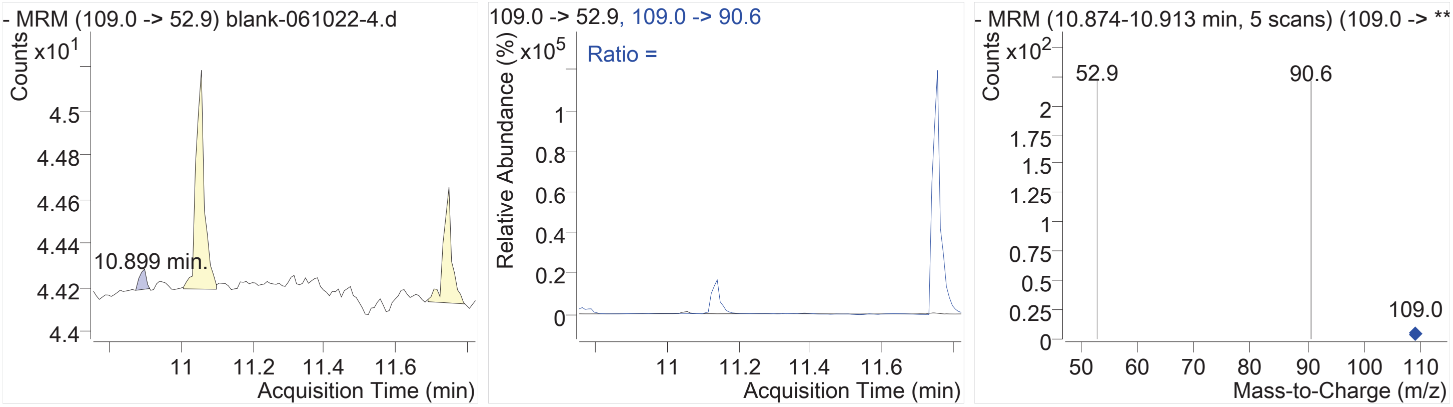

3,4-Dihydroxyphenylacetic acid

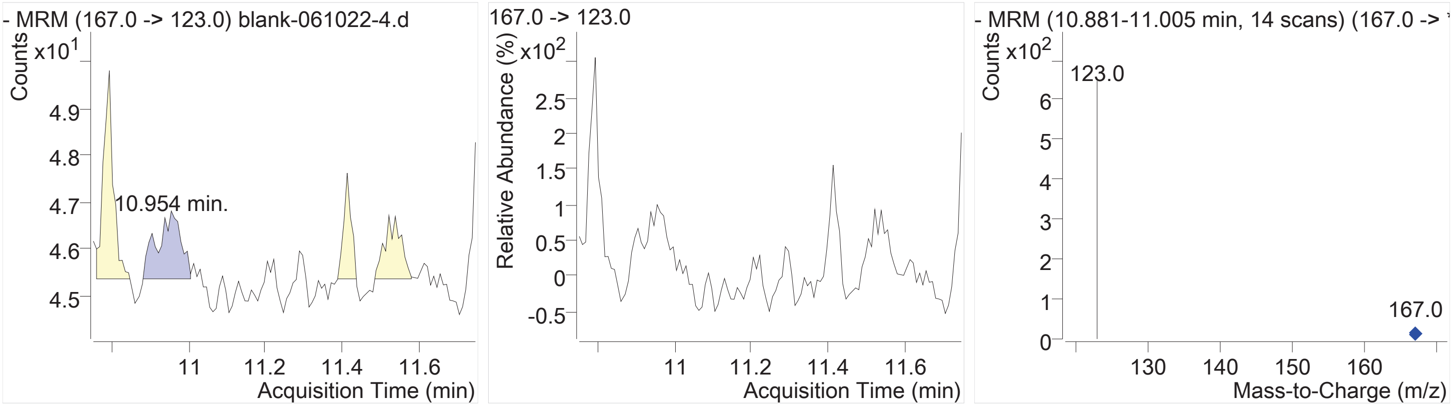

Agilent Technologies

(+)-Catechin

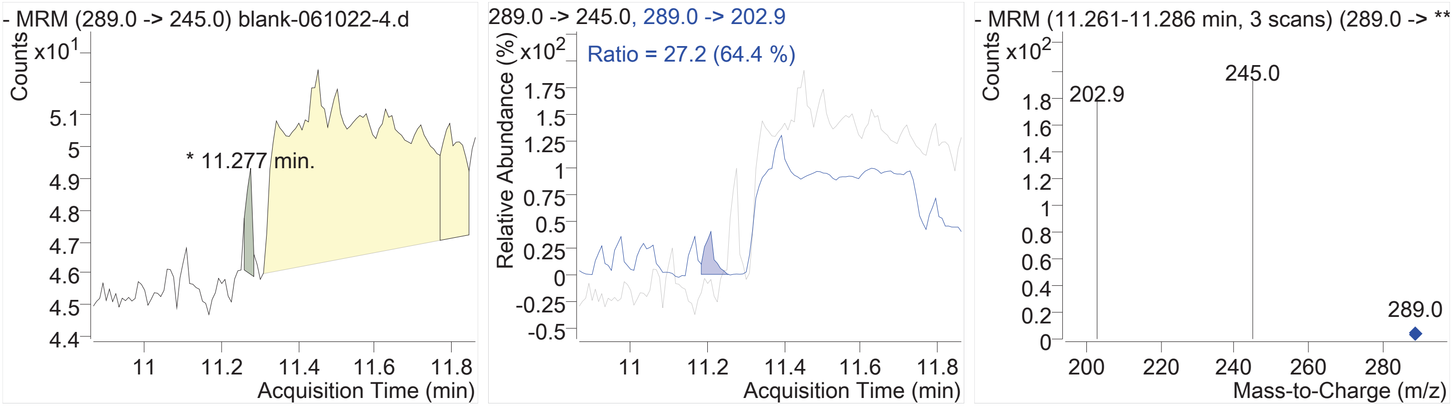

2,5-Dihydroxybenzoic acid

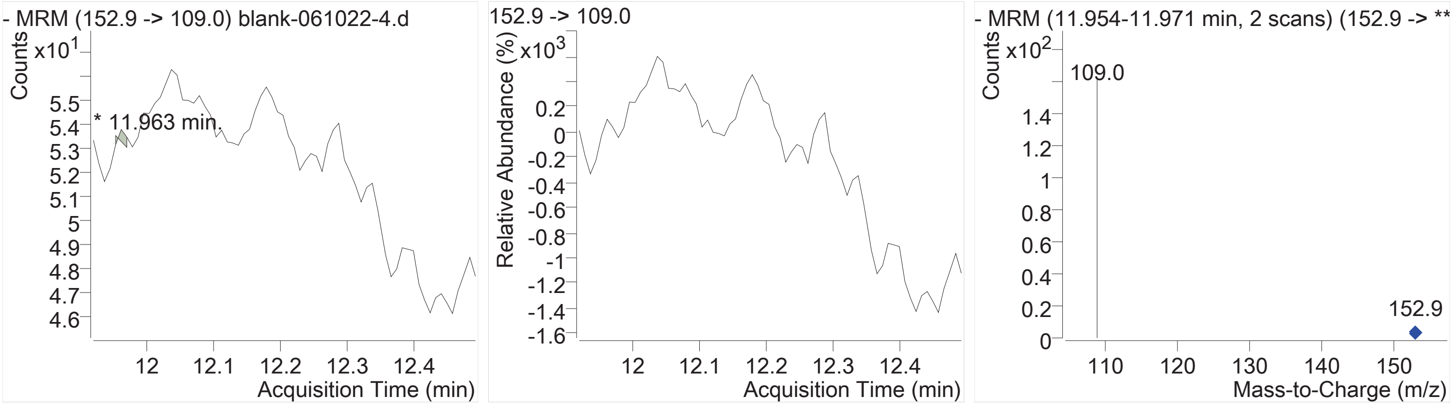

Chlorogenic acid

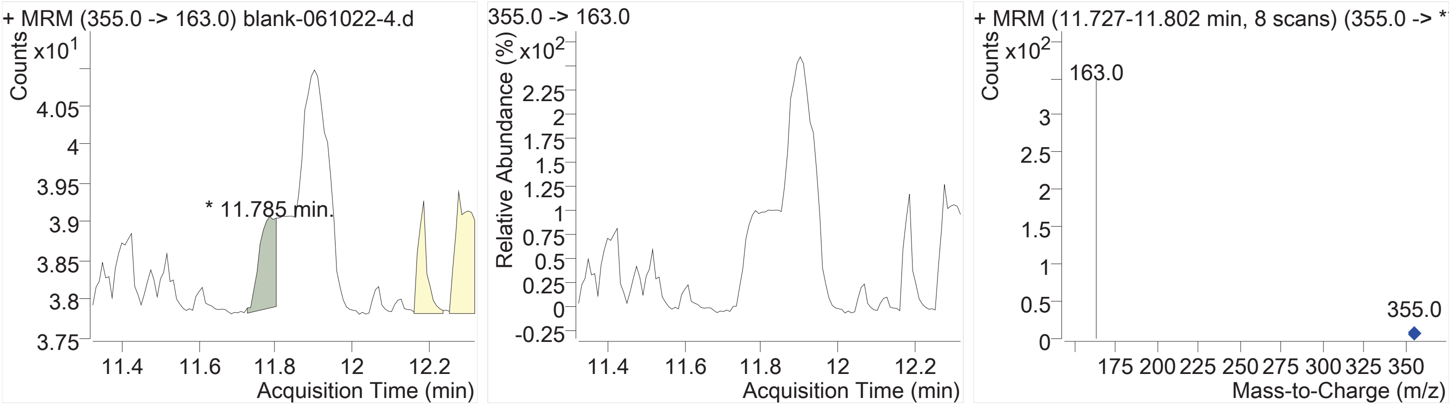

3-Hydroxybenzoic acid

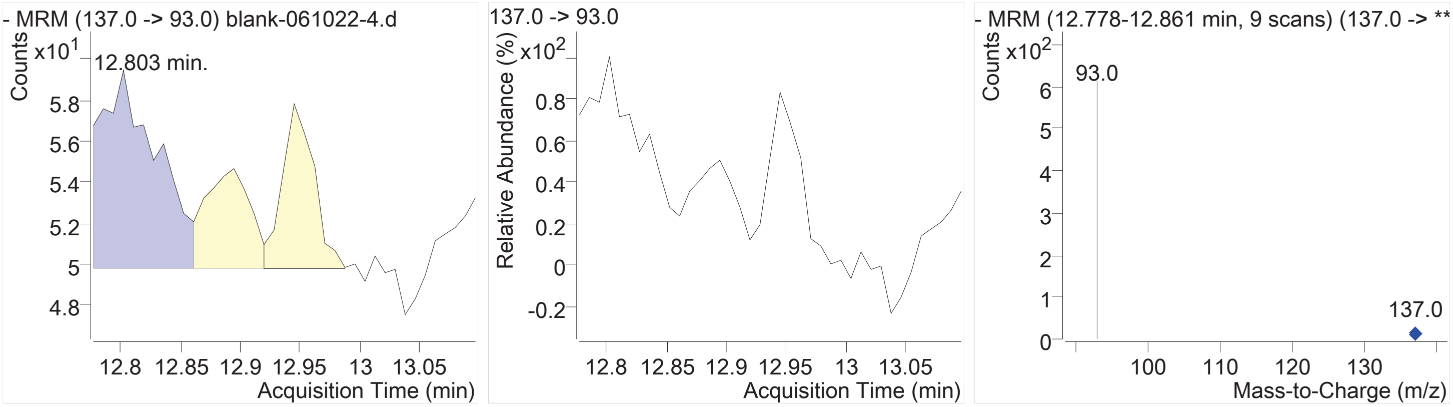

4-Hydroxybenzoic acid

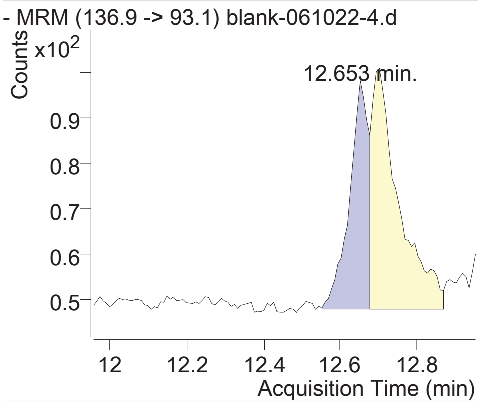

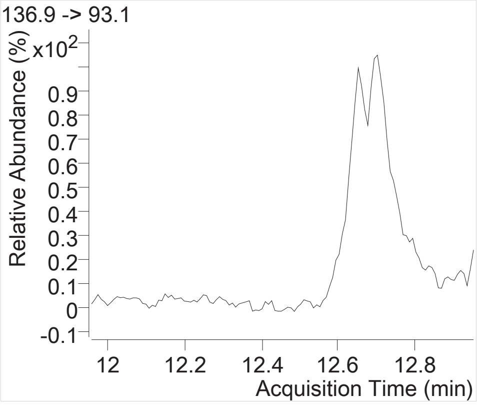

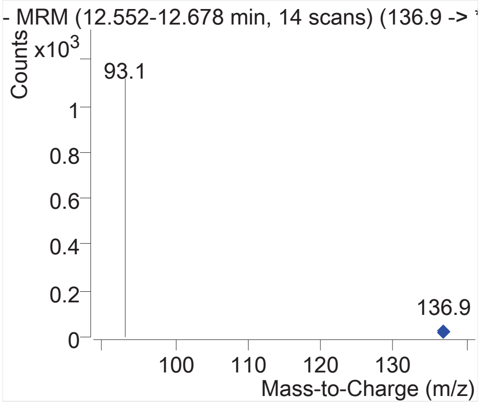

(-)-Epicatechin

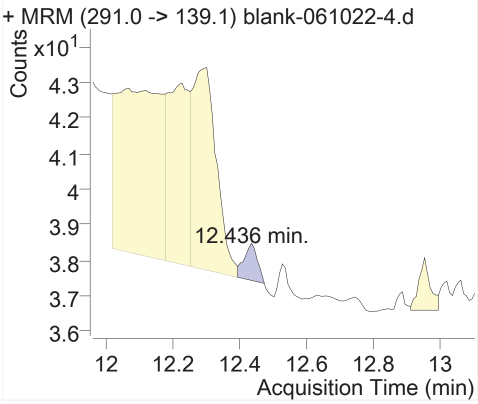

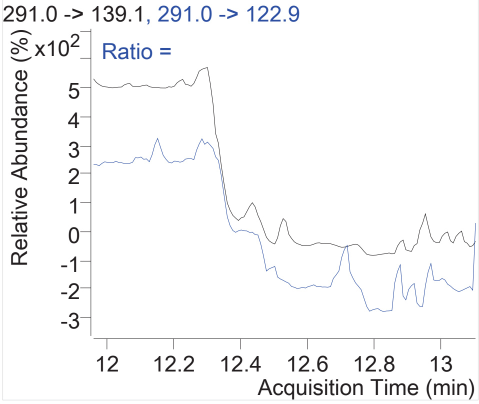

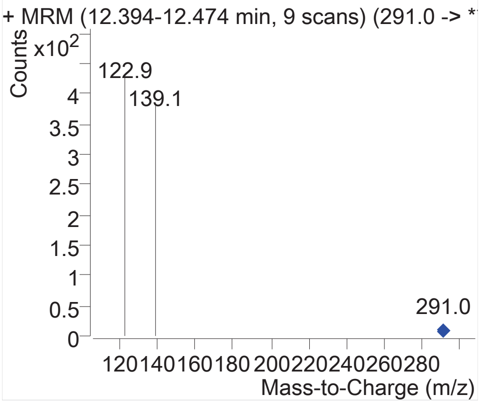

Caffeic acid

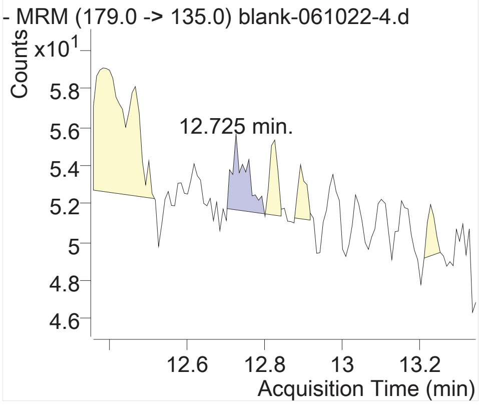

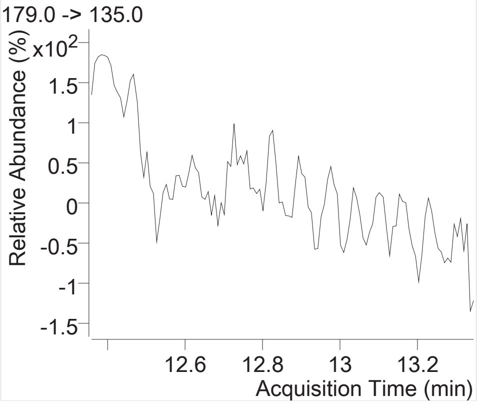

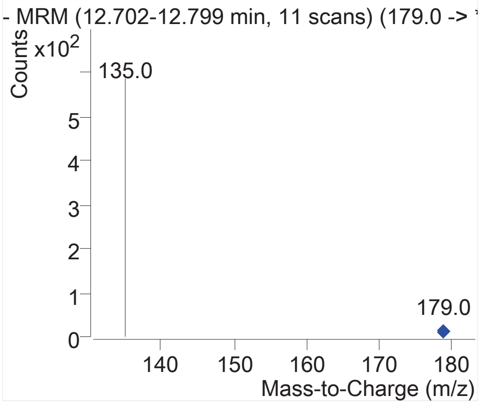

Syringic acid

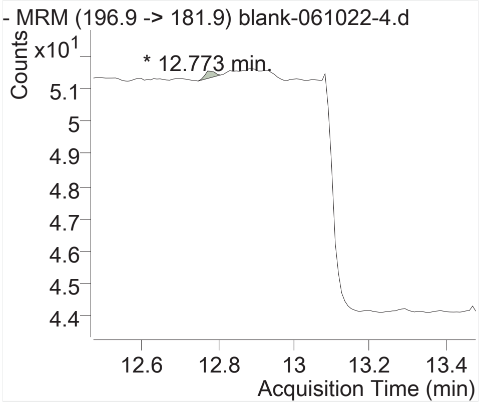

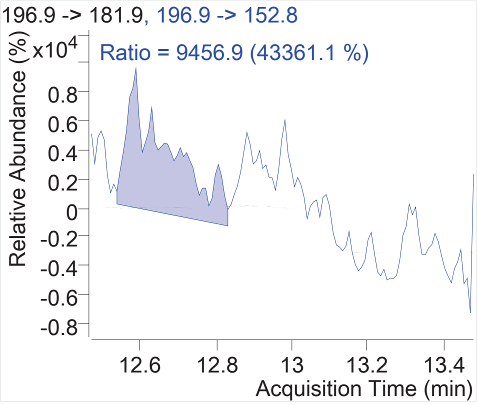

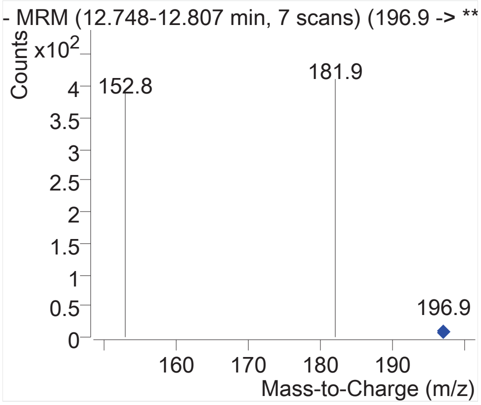

Vanillin

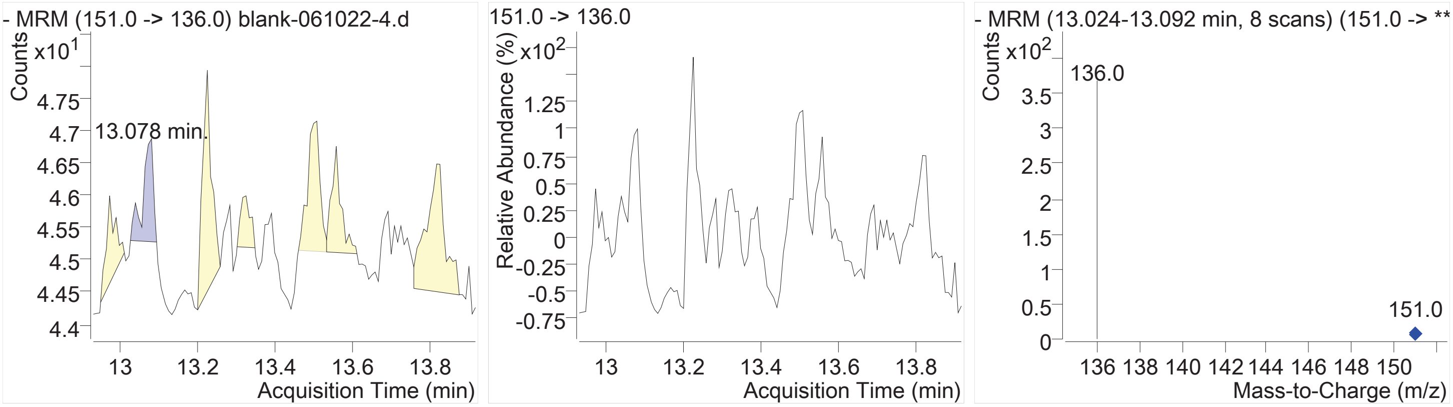

Verbascoside

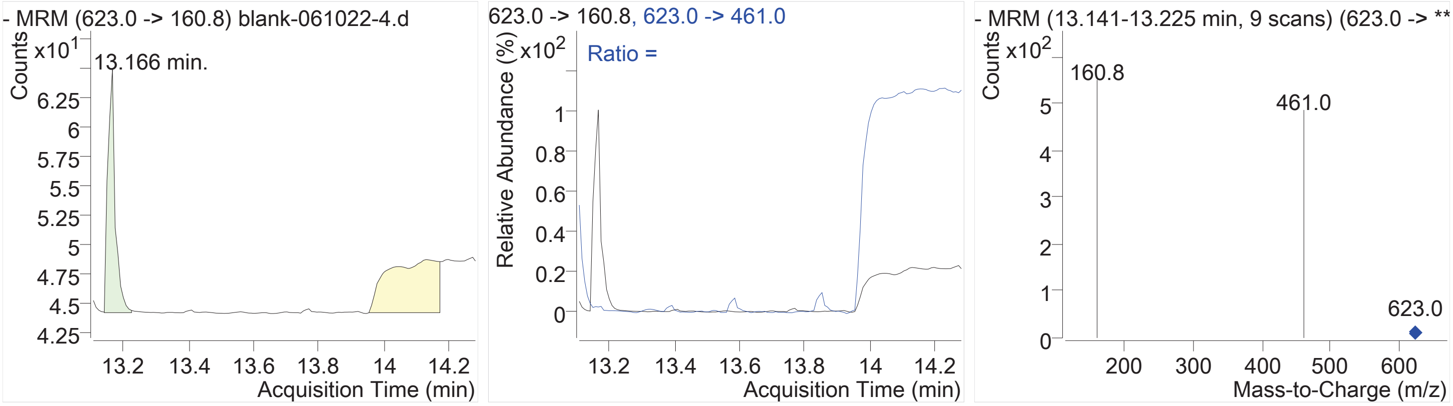

Taxifolin

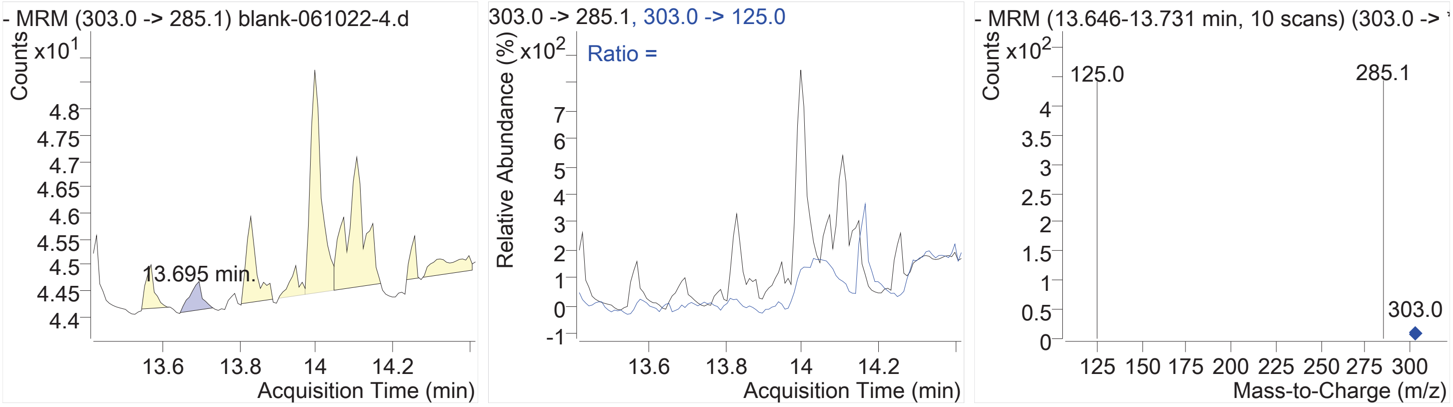

p-Coumaric acid

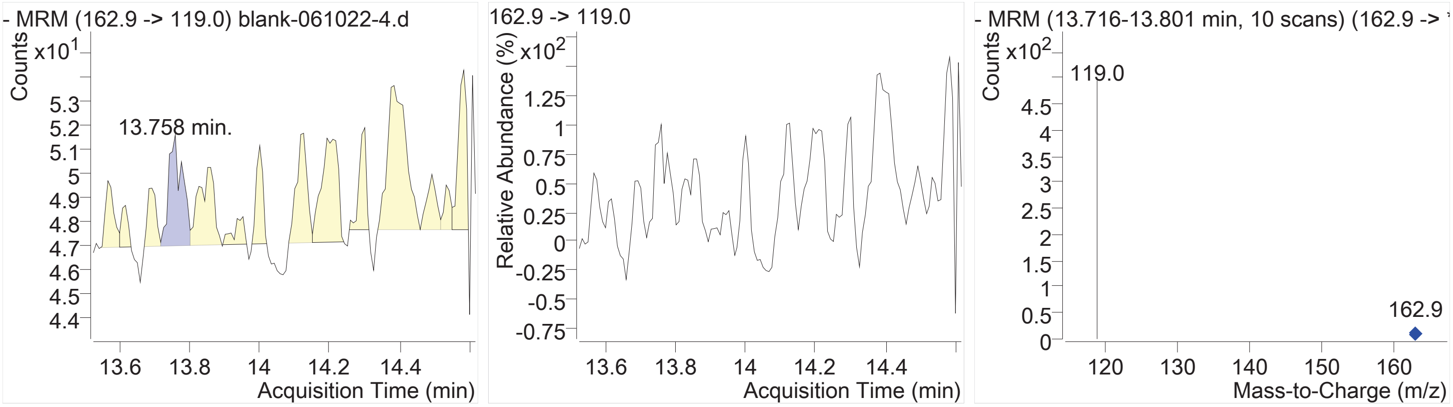

Agilent Technologies

Sinapic acid

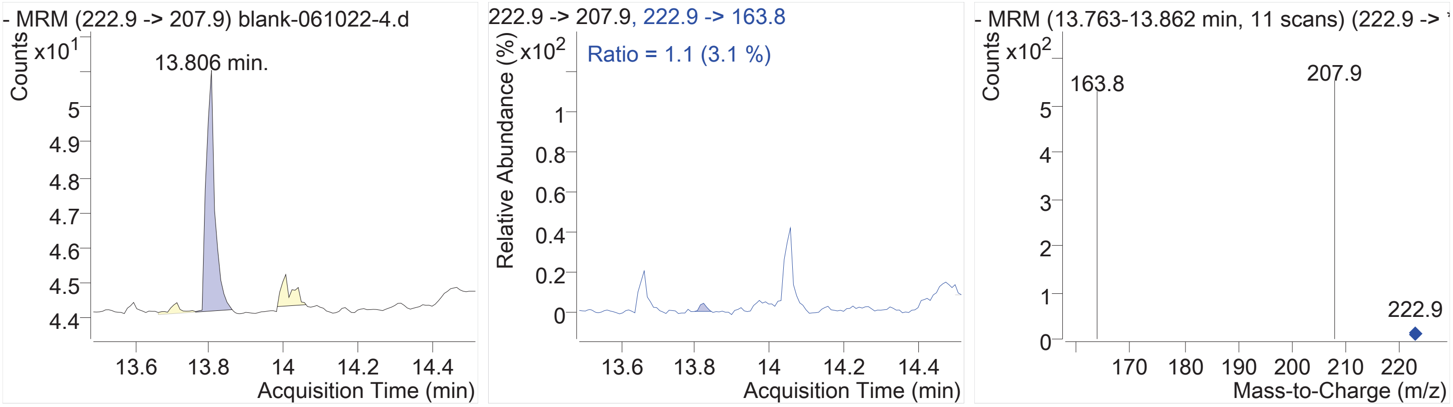

Ferulic acid

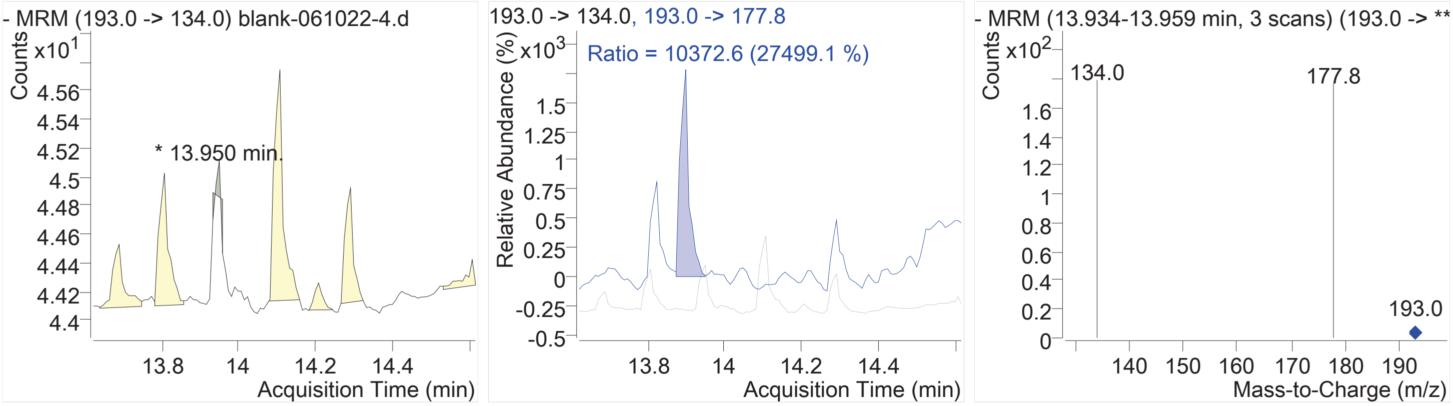

Luteolin 7-glucoside

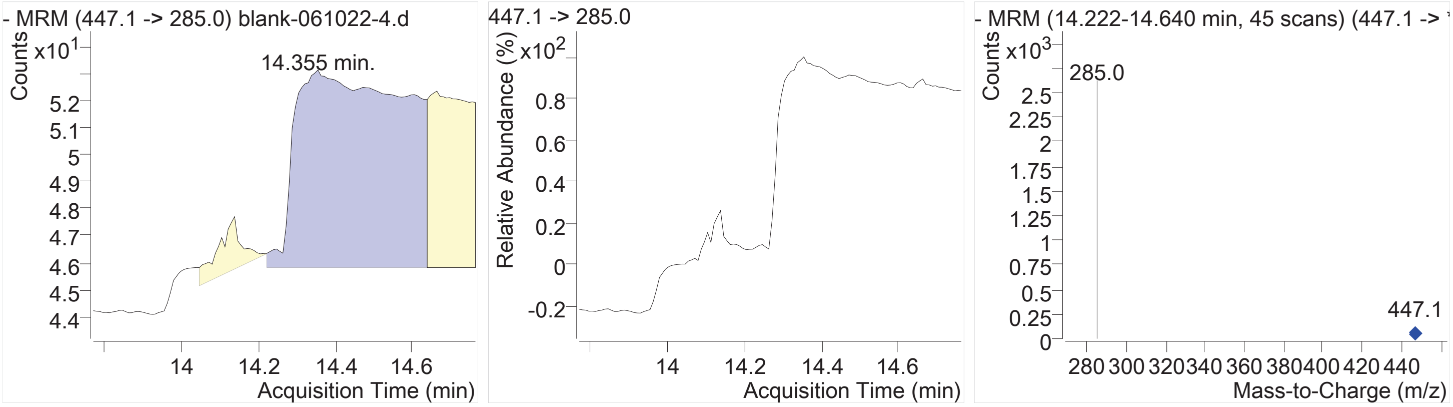

Hesperidin

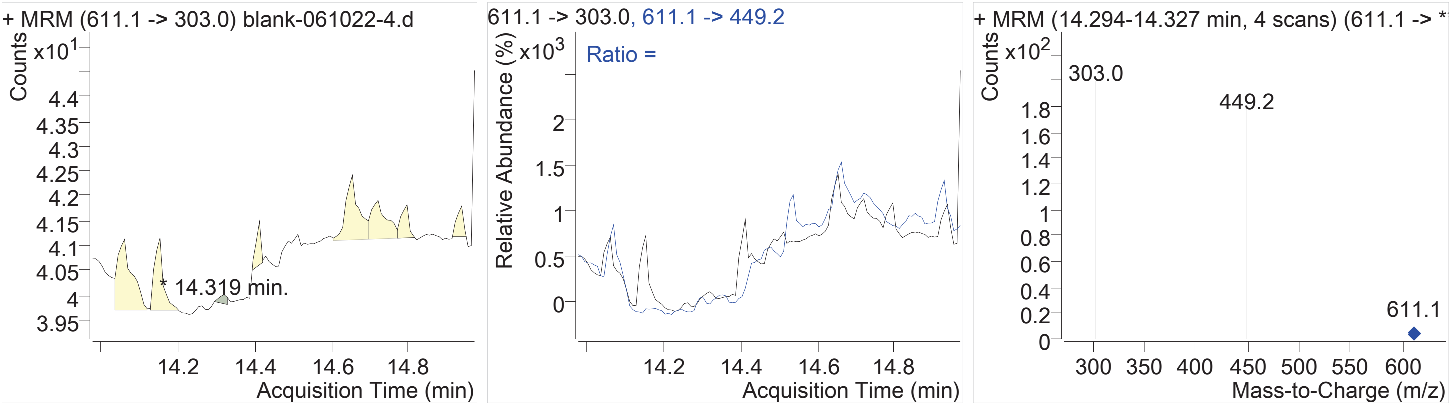

Hyperoside

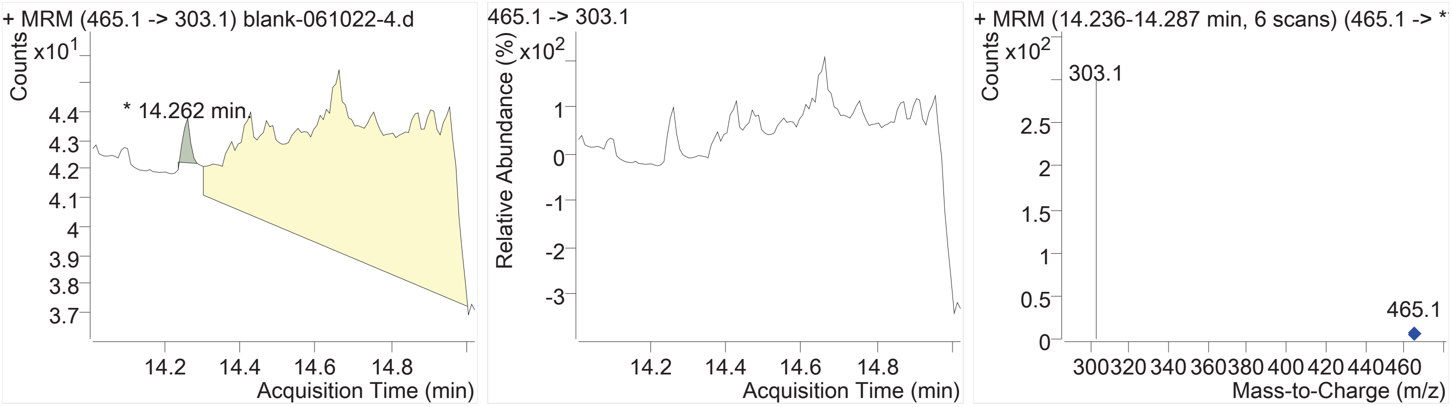

Rosmarinic acid

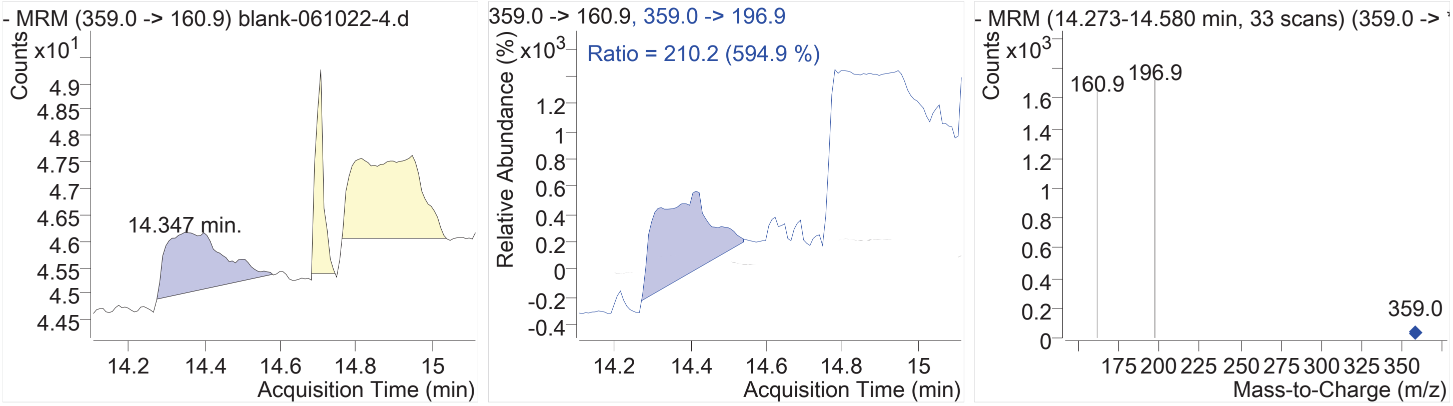

Apigenin 7-glucoside

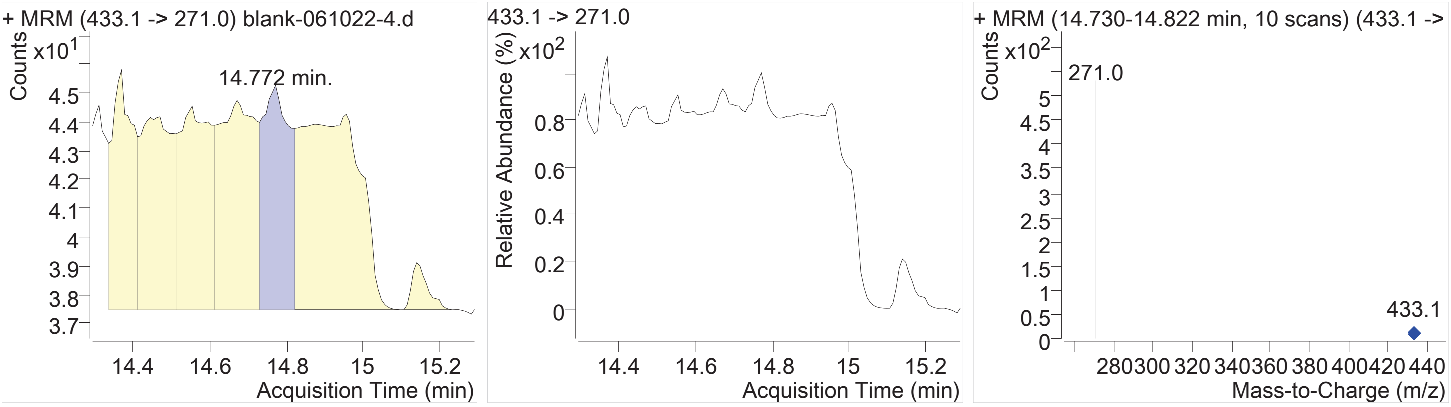

Pinoresinol

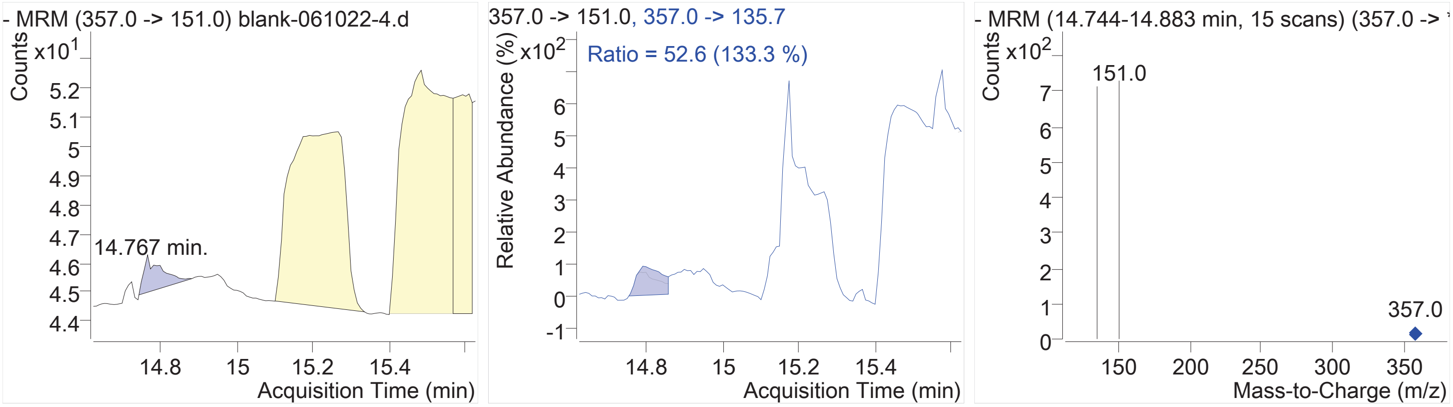

2-Hydroxycinnamic acid

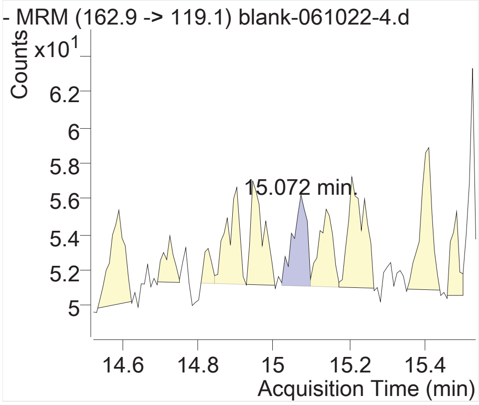

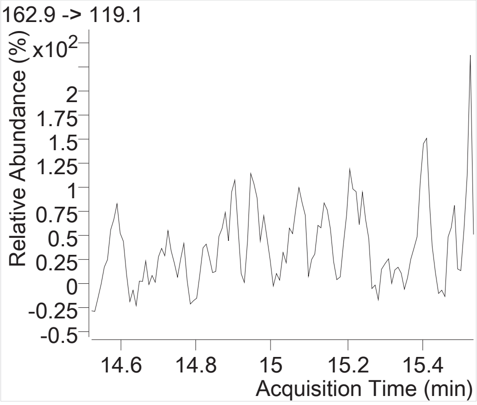

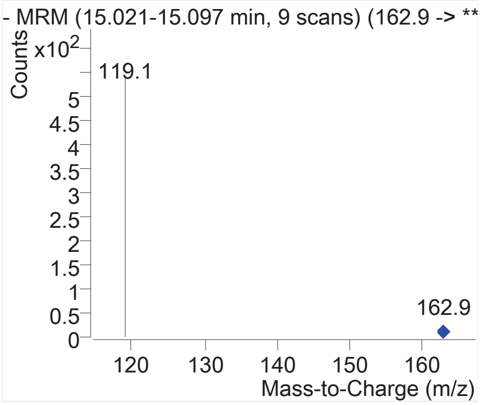

Eriodictyol

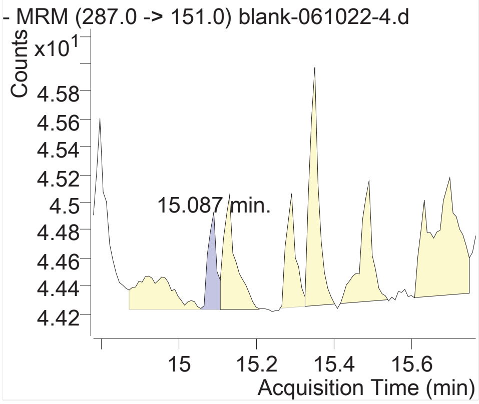

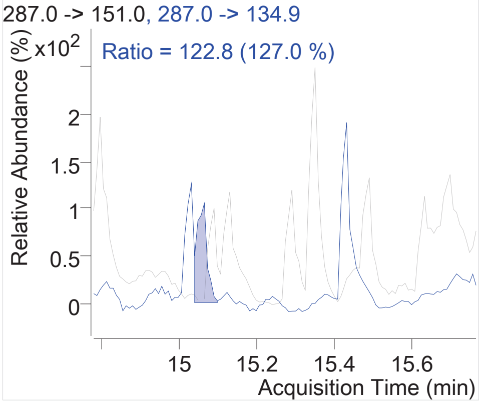

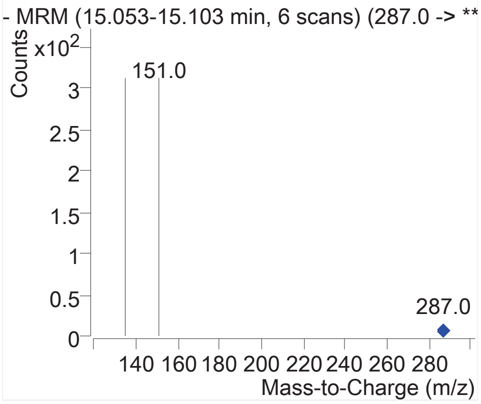

Quercetin

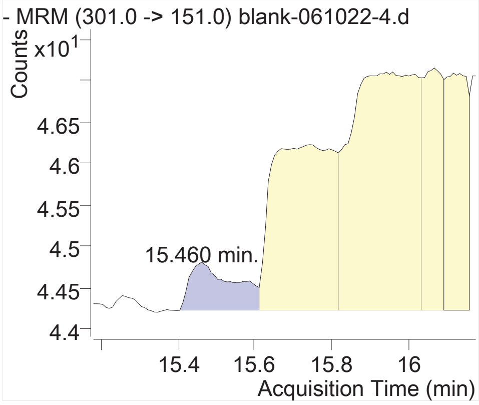

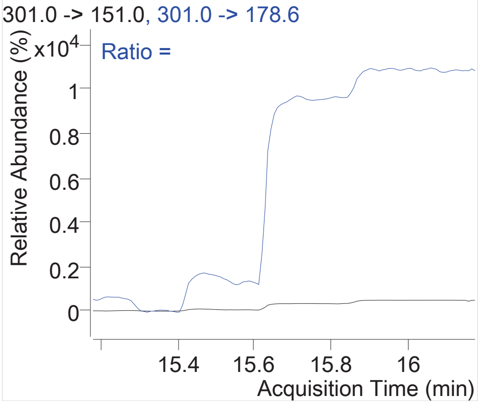

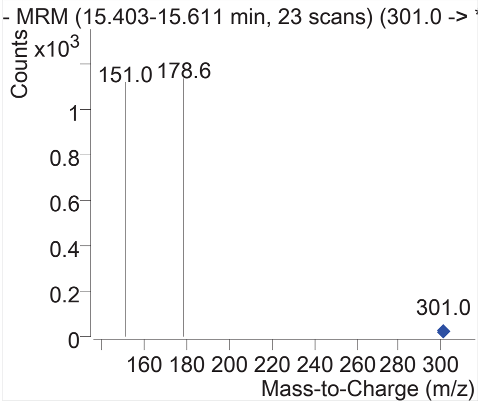

Luteolin

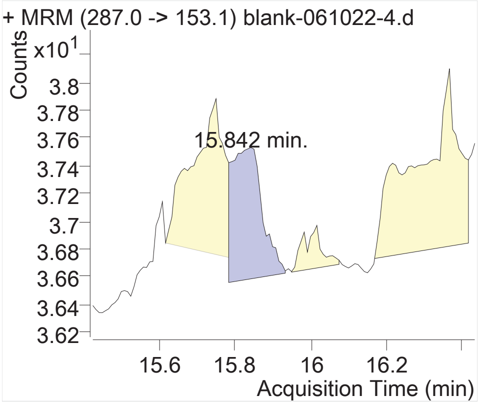

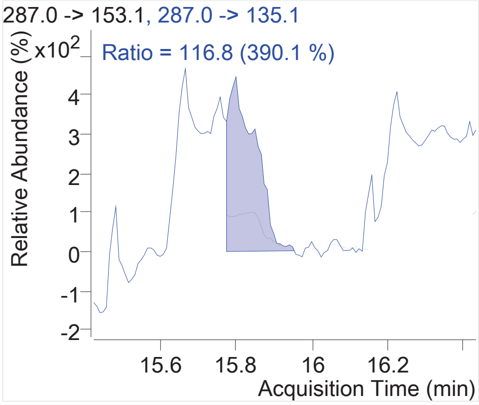

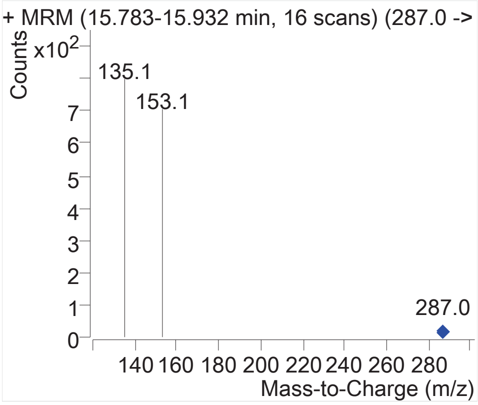

Kaempferol

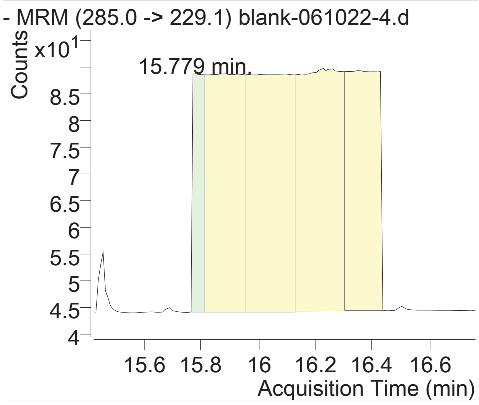

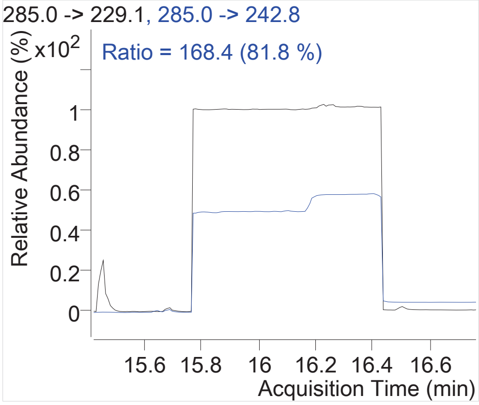

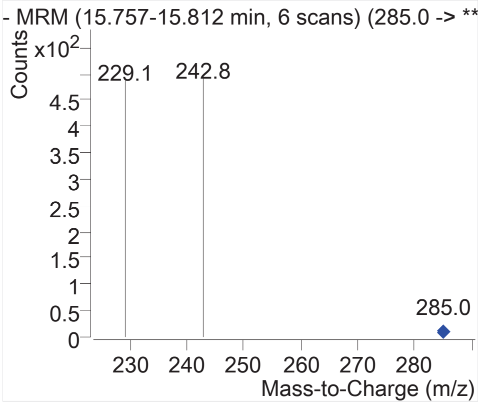

Apigenin

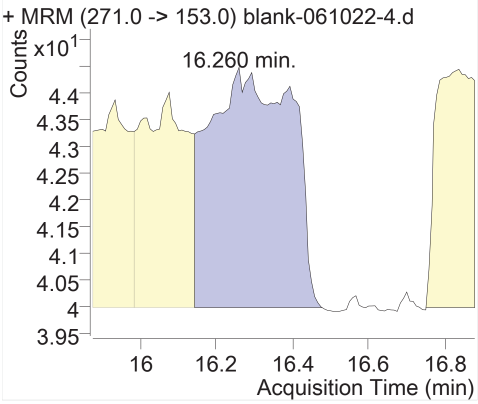

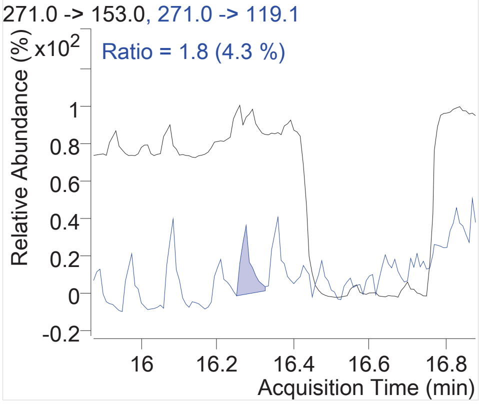

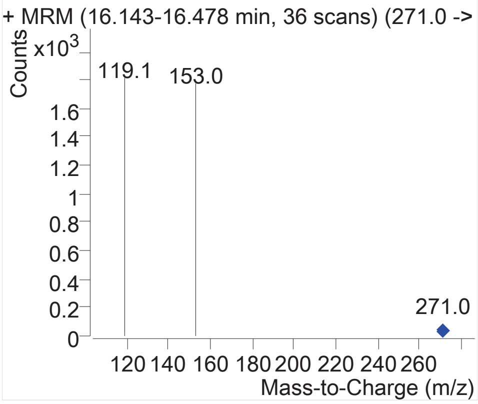

Quantitative Analysis Complete Report

Agilent Technologies

**Batch Path**	D:\MassHunter\Data\2022ekim\061022cengizhoca\QuantResults\071022.batch.bin		
**Analysis Time**	10/11/2022 1:33:26 PM	**Analyst Name**	Defam-PC\admin
**Report Time**	10/11/2022 1:33:50 PM	**Reporter Name**	admin
**Last Calib Update**	10/11/2022 1:33:17 PM	**Batch State**	Processed
**Quant Batch Version**	B.07.01	**Quant Report Version**	B.07.01

**Acq. Time**	10/6/2022 3:55:14 PM	**Data File**	25ppb1.d
**Sample Type**	Cal	**Sample Name**	25ppb1
**Dilution**	1	**Acq. Method**	FENOLIK_DMRM2021-31bilesen.m

Sample Chromatogram

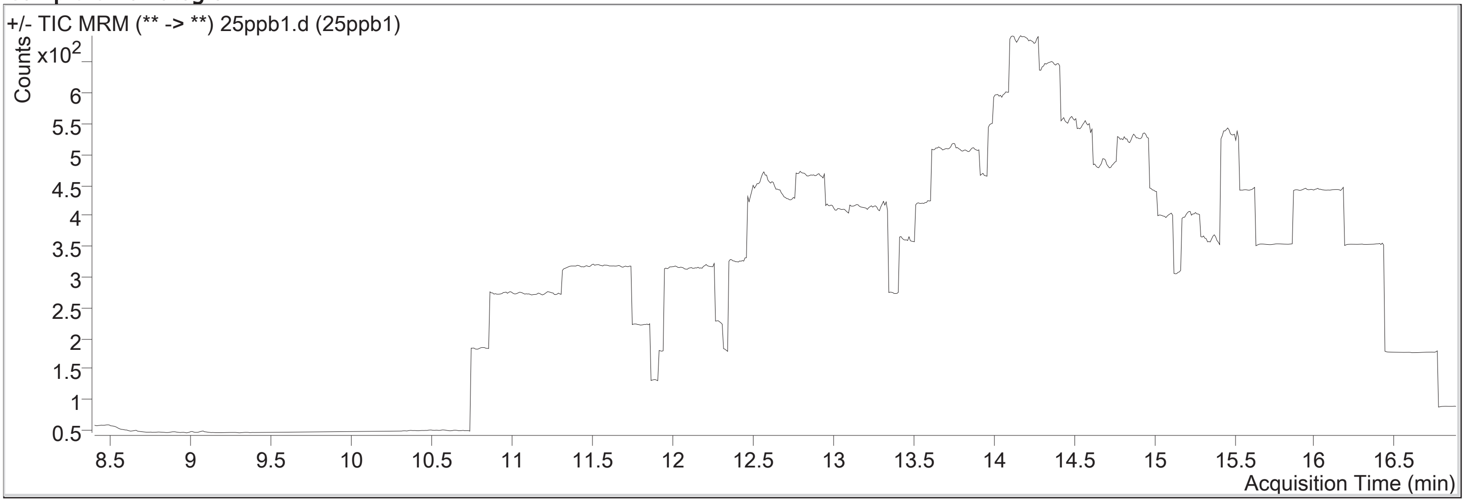

Compound	Transition	RT	Resp.	Final Conc	Units
Gallic acid	168.9 -> 125.0	8.666	9	ND	ng/ml
Protocatechuic acid	152.9 -> 108.9	10.501	40	ND	ng/ml
Pyrocatechol	109.0 -> 52.9	10.772	2	ND	ng/ml
3,4-Dihydroxyphenylacetic acid	167.0 -> 123.0	10.879	3	ND	ng/ml
(+)-Catechin	289.0 -> 245.0	11.386	48	ND	ng/ml
2,5-Dihydroxybenzoic acid	152.9 -> 109.0	11.996	18	3.4659	ng/ml
Chlorogenic acid	355.0 -> 163.0	11.785	10	6.2443	ng/ml
3-Hydroxybenzoic acid	137.0 -> 93.0	12.803	5	15.9093	ng/ml
4-Hydroxybenzoic acid	136.9 -> 93.1	12.577	391	19.3507	ng/ml
(-)-Epicatechin	291.0 -> 139.1	12.293	59	10.5488	ng/ml
Caffeic acid	179.0 -> 135.0	12.691	30	ND	ng/ml
Syringic acid	196.9 -> 181.9	12.824	86	122.7660	ng/ml
Vanillin	151.0 -> 136.0	13.053	1	ND	ng/ml
Verbascoside	623.0 -> 160.8	14.129	44	ND	ng/ml
Taxifolin	303.0 -> 285.1	13.779	3	ND	ng/ml
p-Coumaric acid	162.9 -> 119.0	13.758	29	ND	ng/ml
Sinapic acid	222.9 -> 207.9	13.839	1	4.5522	ng/ml
Ferulic acid	193.0 -> 134.0	13.891	2	0.5666	ng/ml
Luteolin 7-glucoside	447.1 -> 285.0	14.531	92	ND	ng/ml
Hesperidin	611.1 -> 303.0	14.419	4	4.7150	ng/ml
Hyperoside	465.1 -> 303.1	14.805	177	12.8758	ng/ml
Rosmarinic acid	359.0 -> 160.9	14.355	13	ND	ng/ml
Apigenin 7-glucoside	433.1 -> 271.0	14.755	79	1.0439	ng/ml
Pinoresinol	357.0 -> 151.0	14.934	7	ND	ng/ml
2-Hydroxycinnamic acid	162.9 -> 119.1	15.383	119	3.8097	ng/ml
Eriodictyol	287.0 -> 151.0	15.152	3	ND	ng/ml
Quercetin	301.0 -> 151.0	15.668	14	ND	ng/ml
Luteolin	287.0 -> 153.1	15.758	11	ND	ng/ml
Kaempferol	285.0 -> 229.1	15.821	222	21.9577	ng/ml

Quantitative Analysis Complete Report

Compound	Transition	RT	Resp.	Final Conc	Units
Apigenin	271.0 -> 153.0	16.302	48	ND	ng/ml

Gallic acid

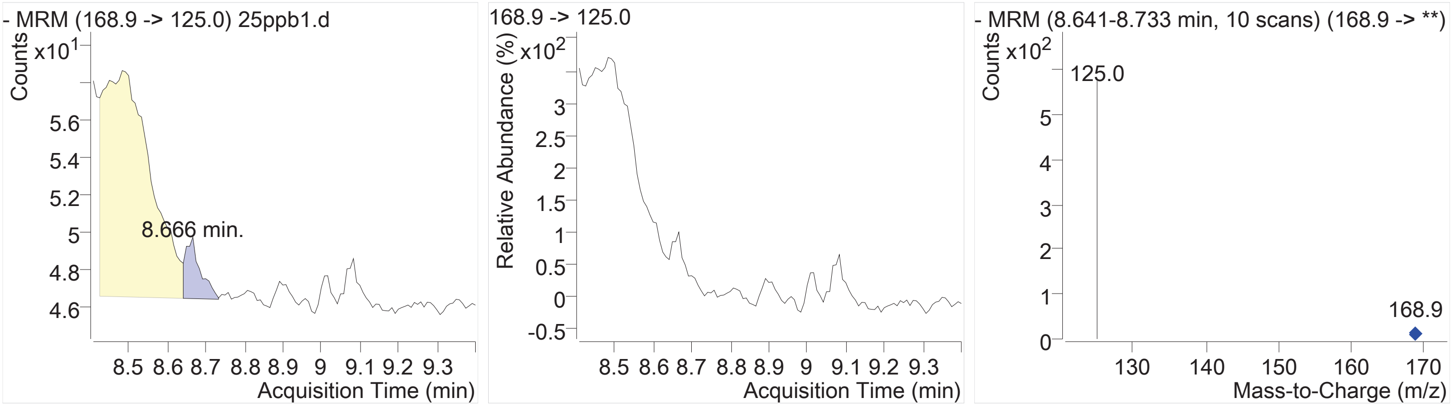

Protocatechuic acid

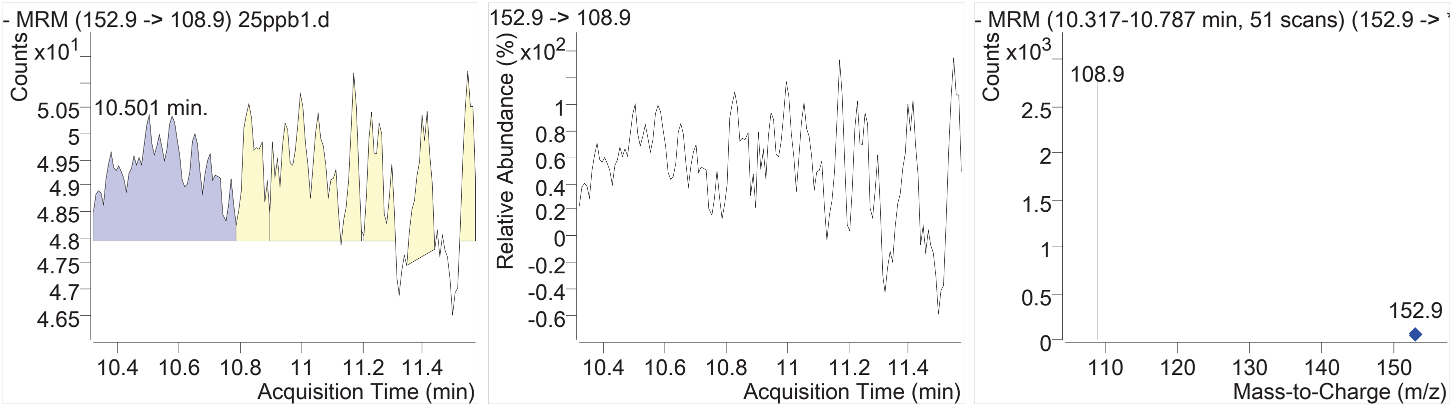

Pyrocatechol

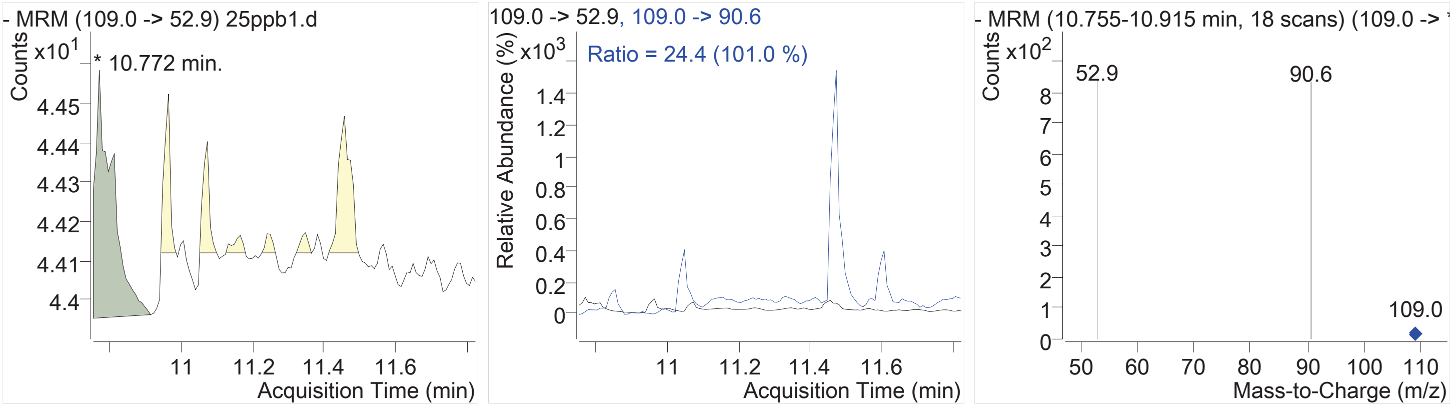

3,4-Dihydroxyphenylacetic acid

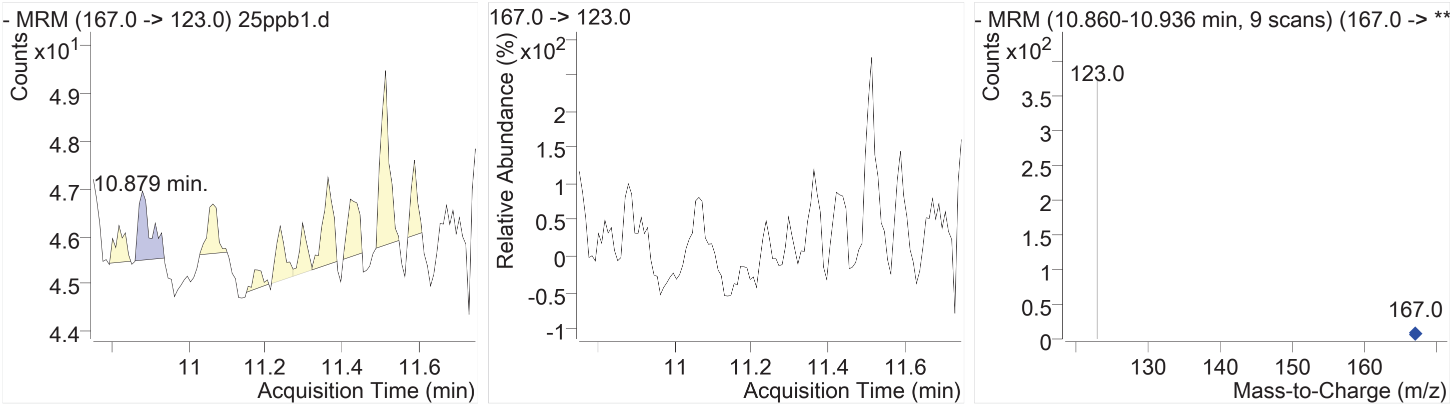

Quantitative Analysis Complete Report

Agilent Technologies

(+)-Catechin

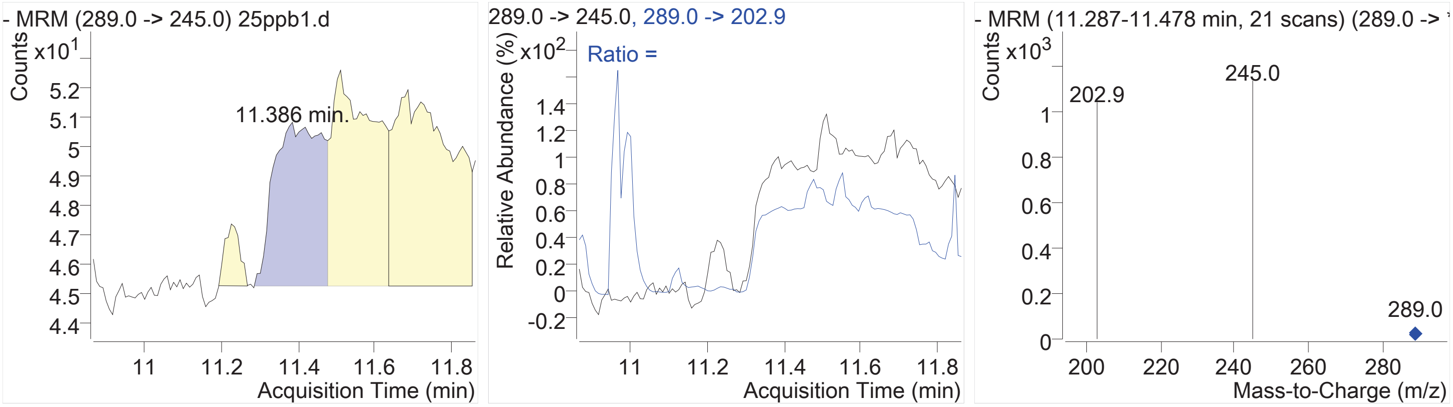

2,5-Dihydroxybenzoic acid

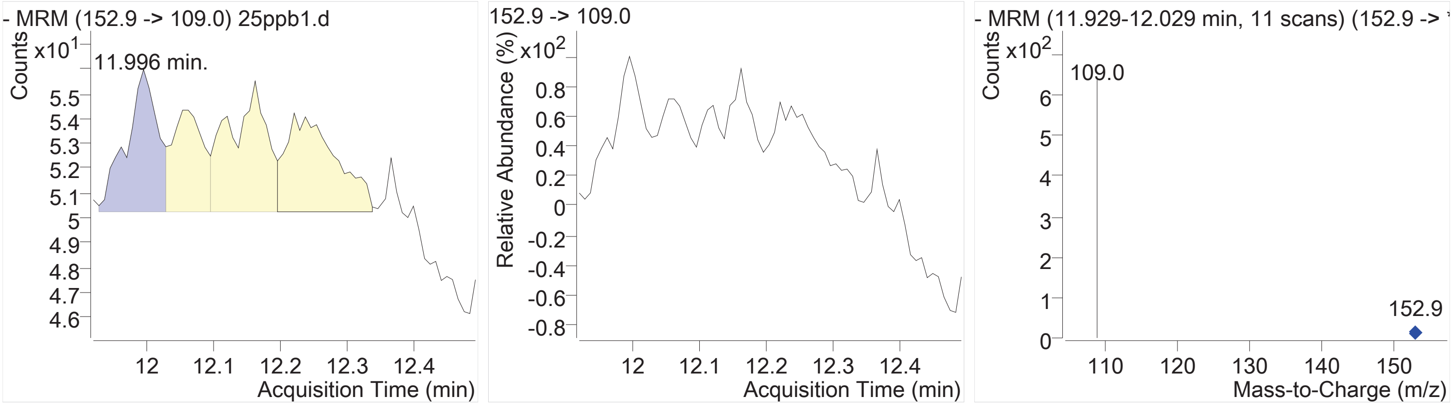

Chlorogenic acid

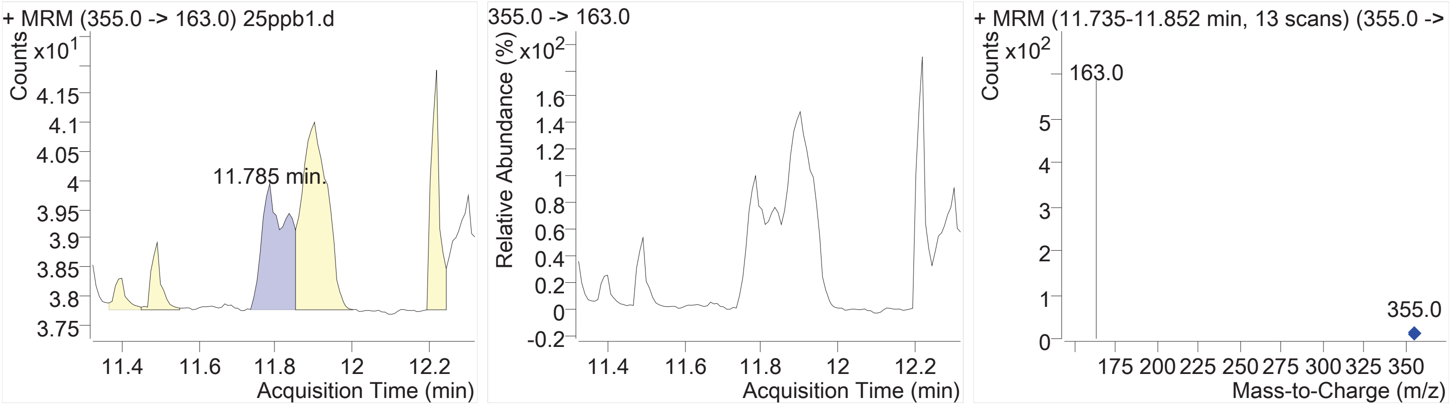

3-Hydroxybenzoic acid

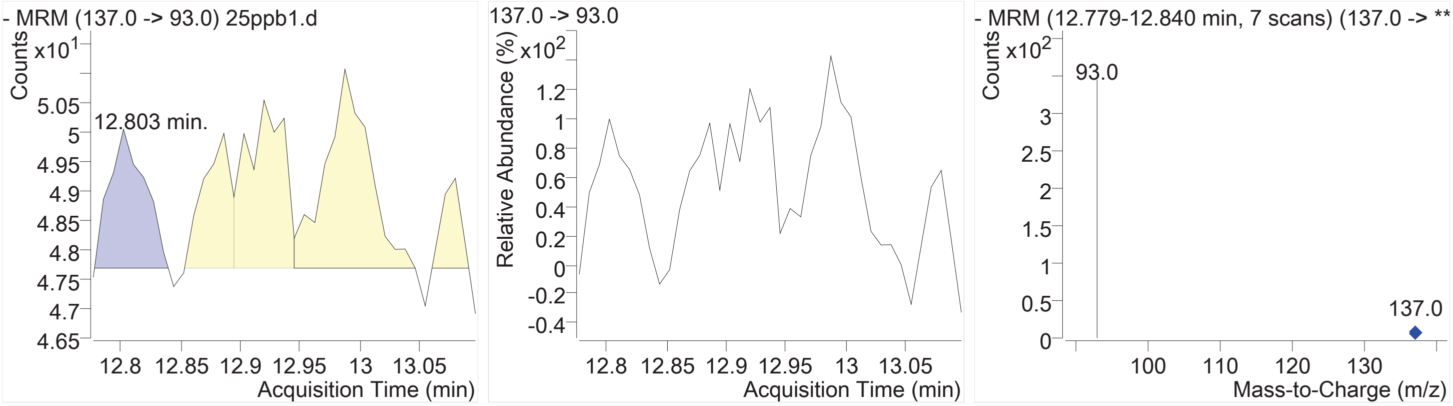

4-Hydroxybenzoic acid

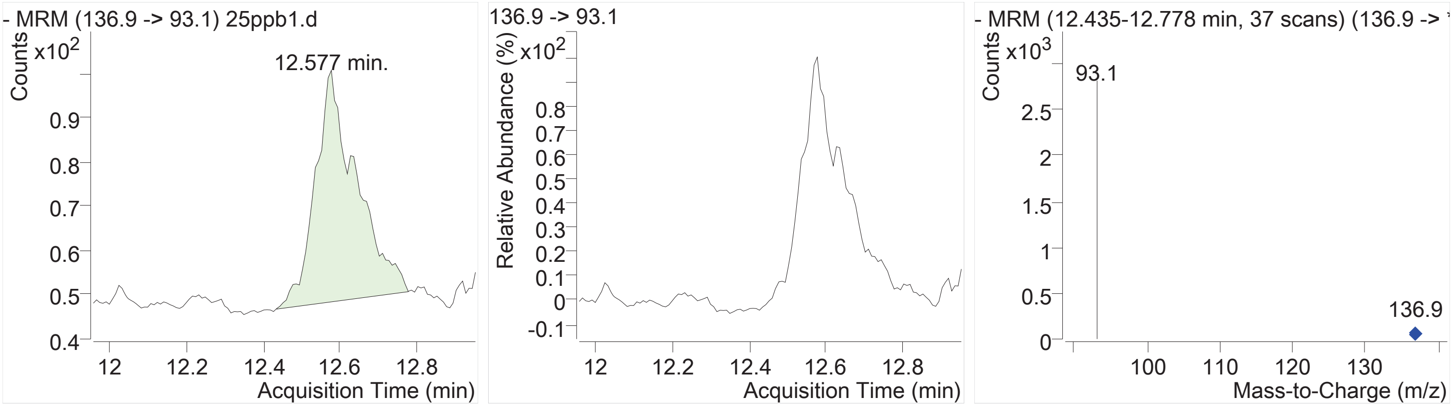

(-)-Epicatechin

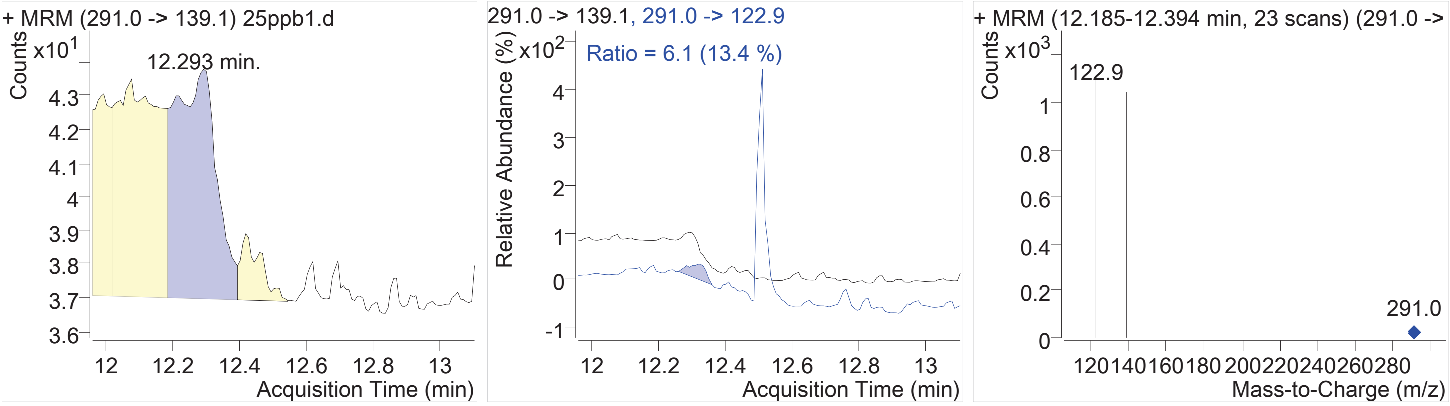

Caffeic acid

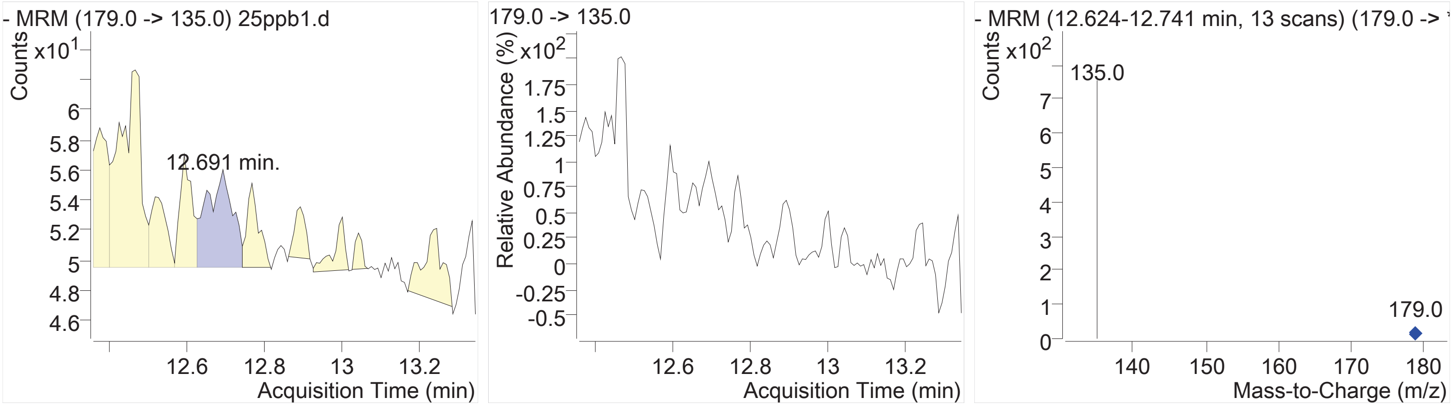

Syringic acid

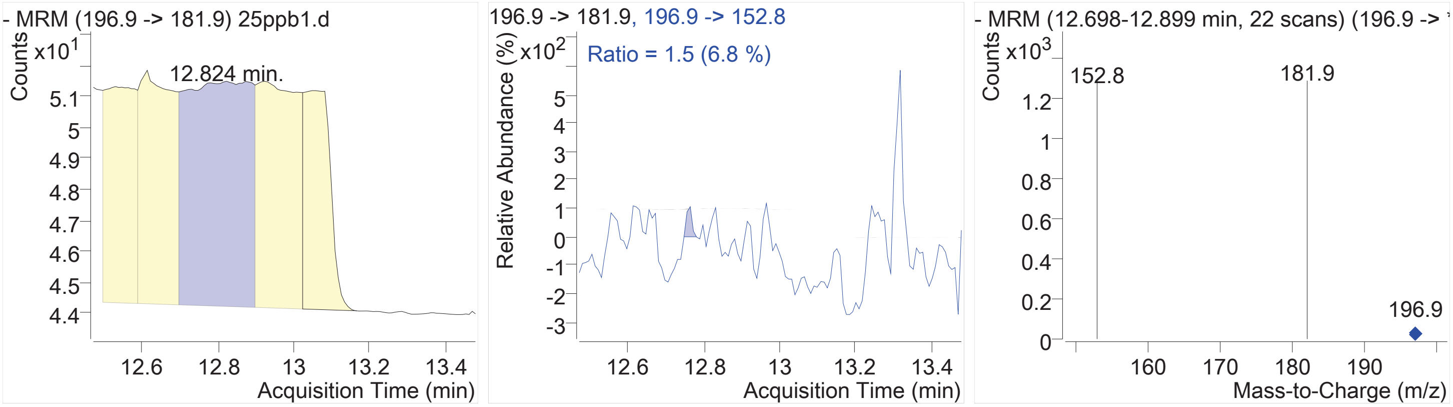

Vanillin

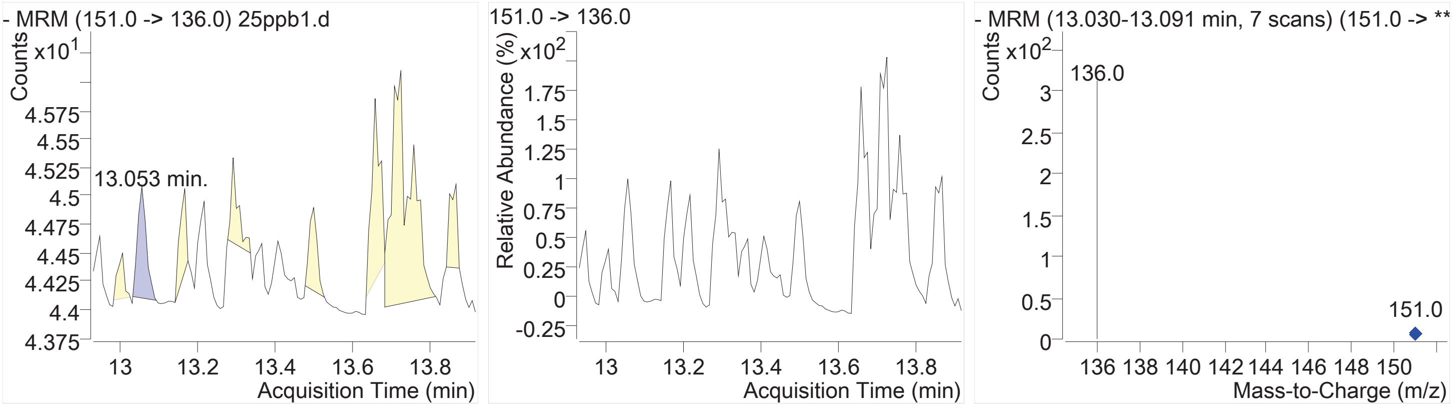

Verbascoside

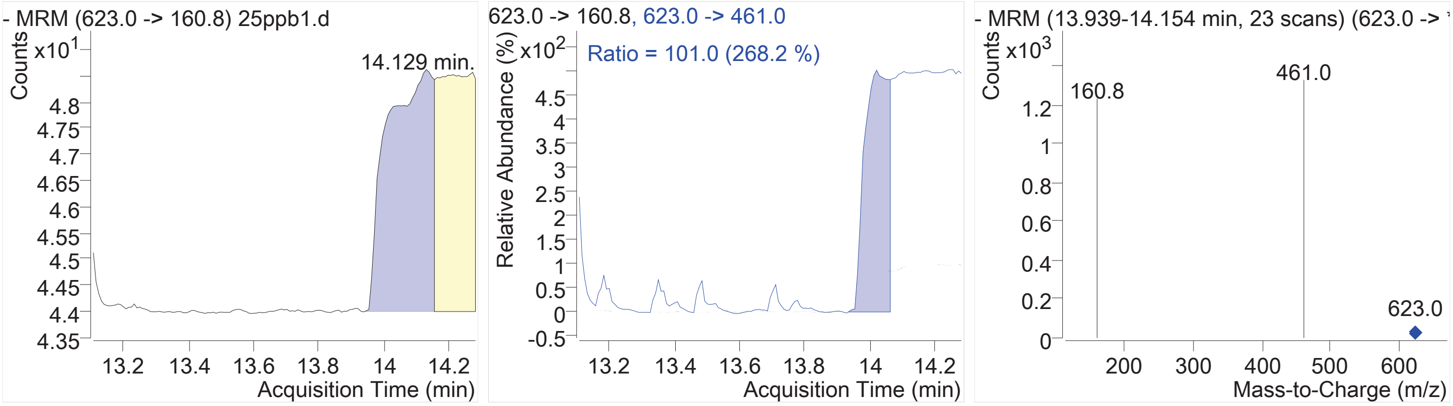

Taxifolin

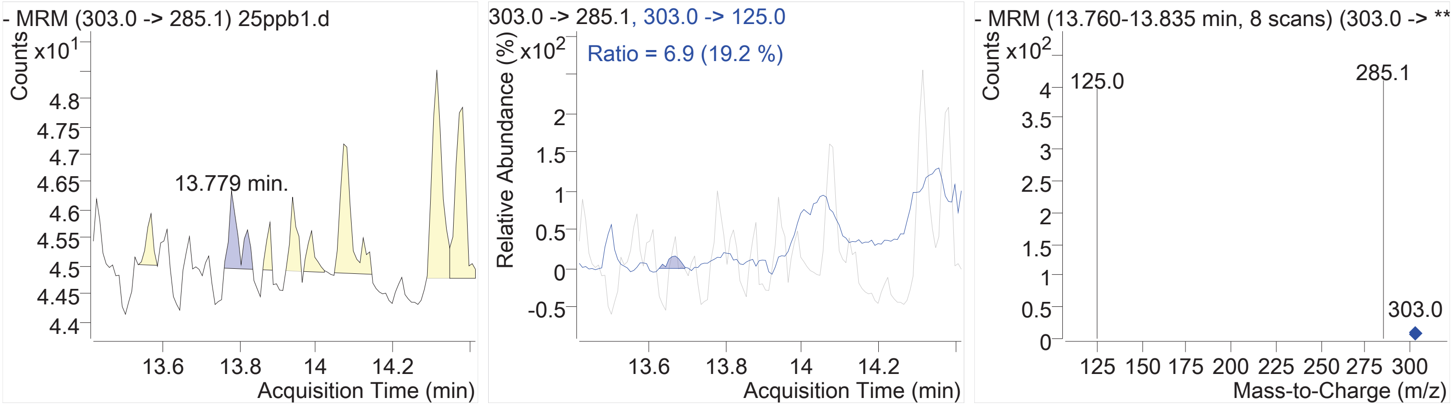

p-Coumaric acid

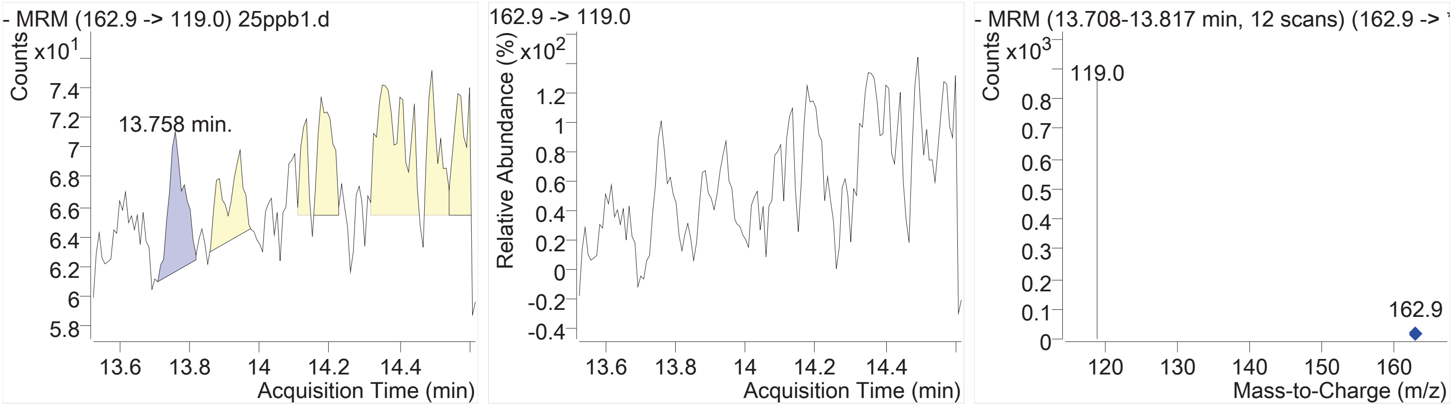

Sinapic acid

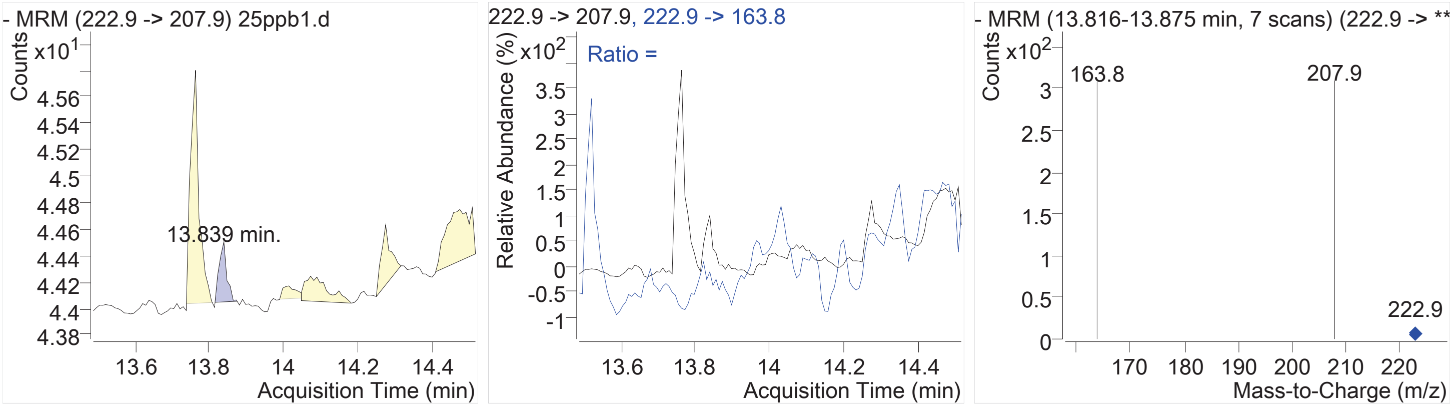

Ferulic acid

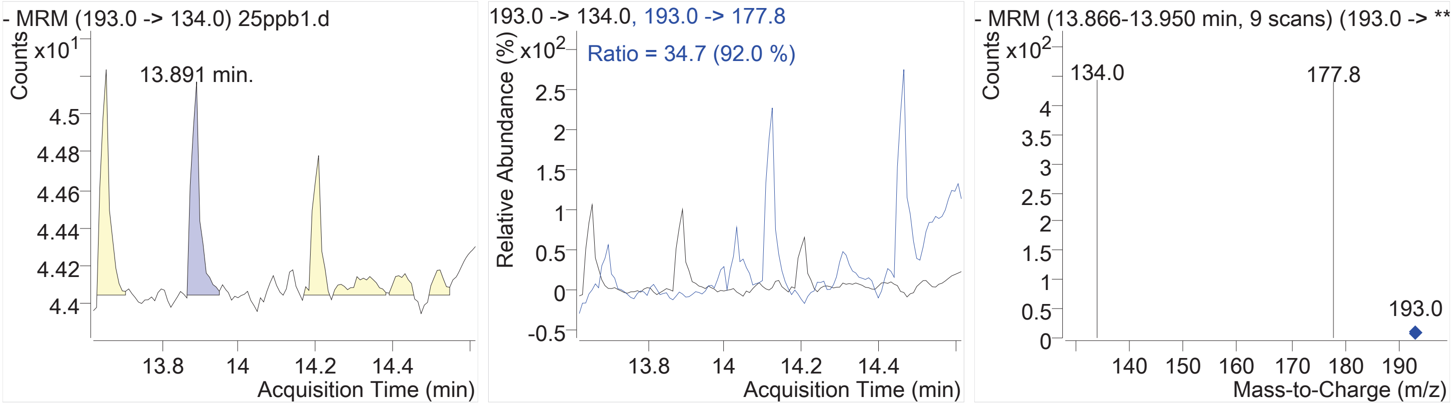

Luteolin 7-glucoside

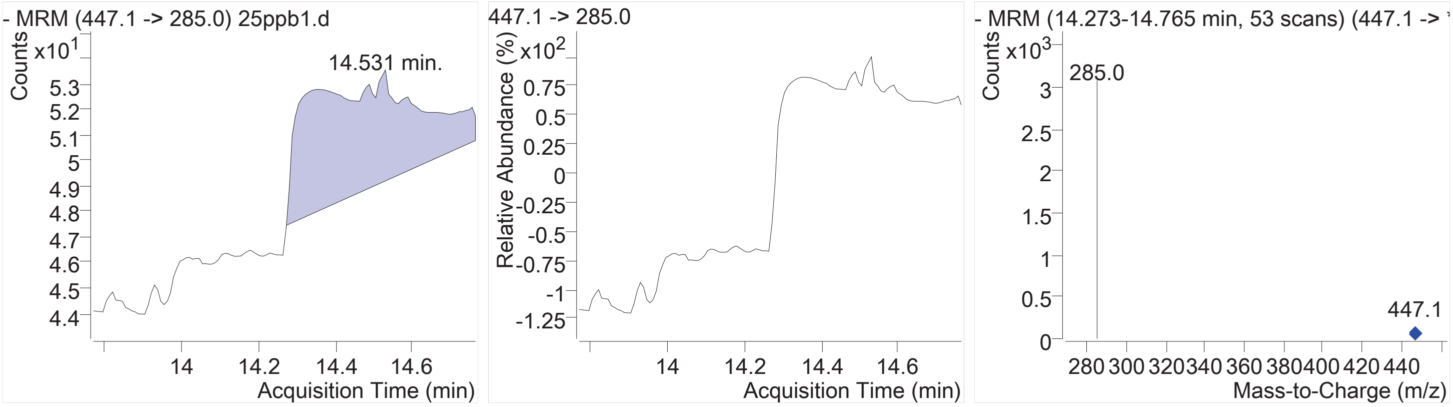

Hesperidin

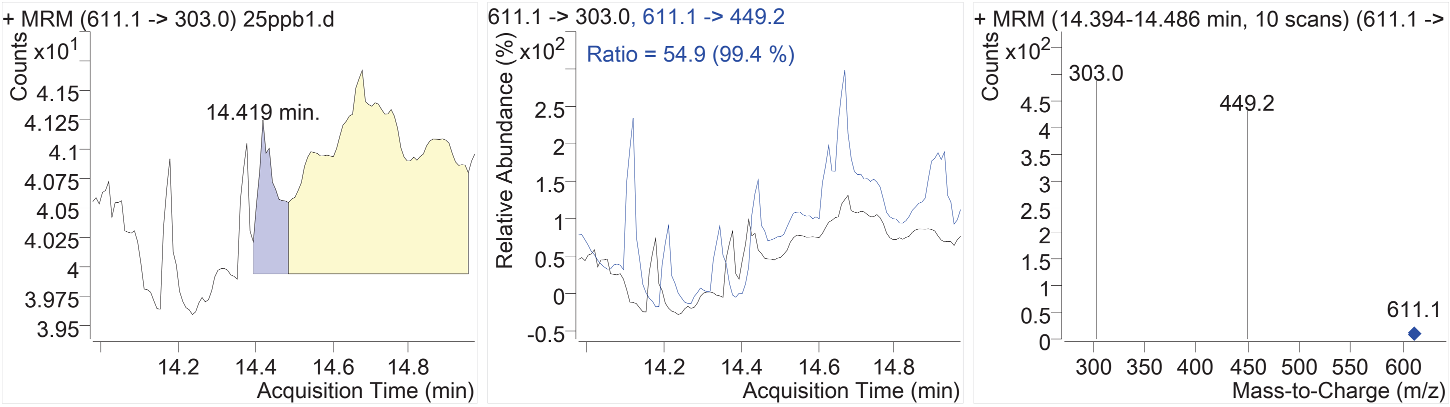

Hyperoside

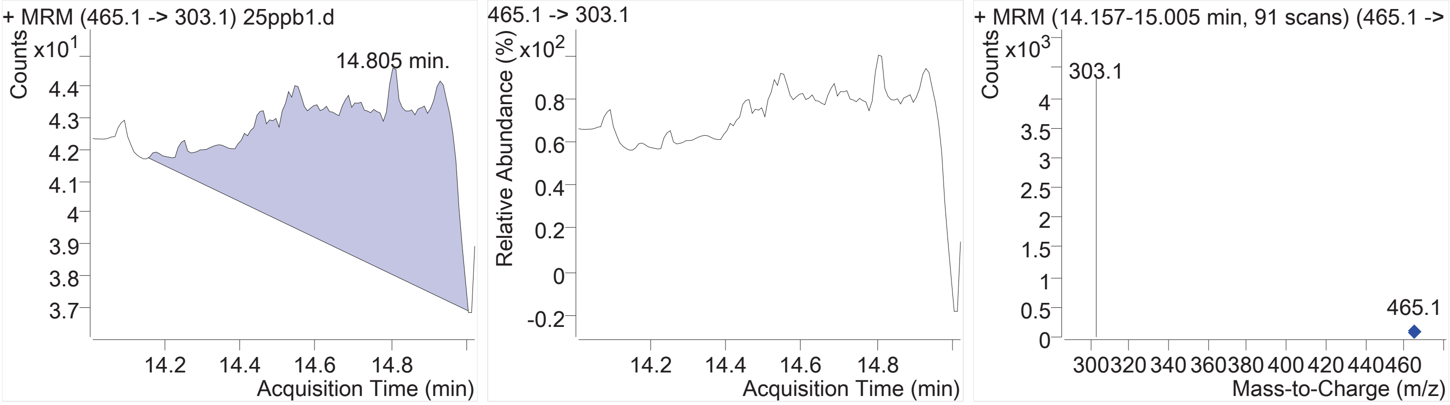

Rosmarinic acid

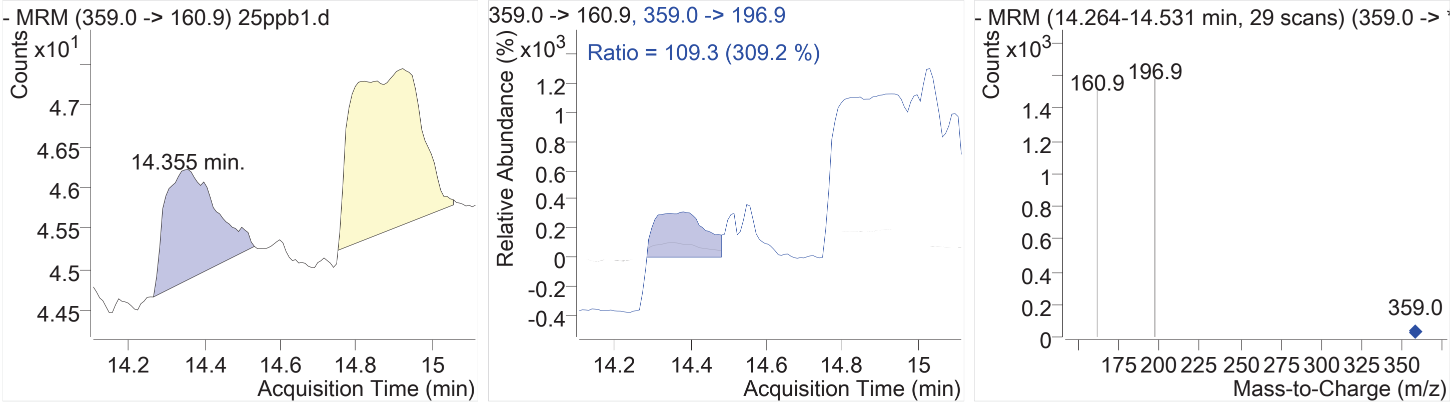

Apigenin 7-glucoside

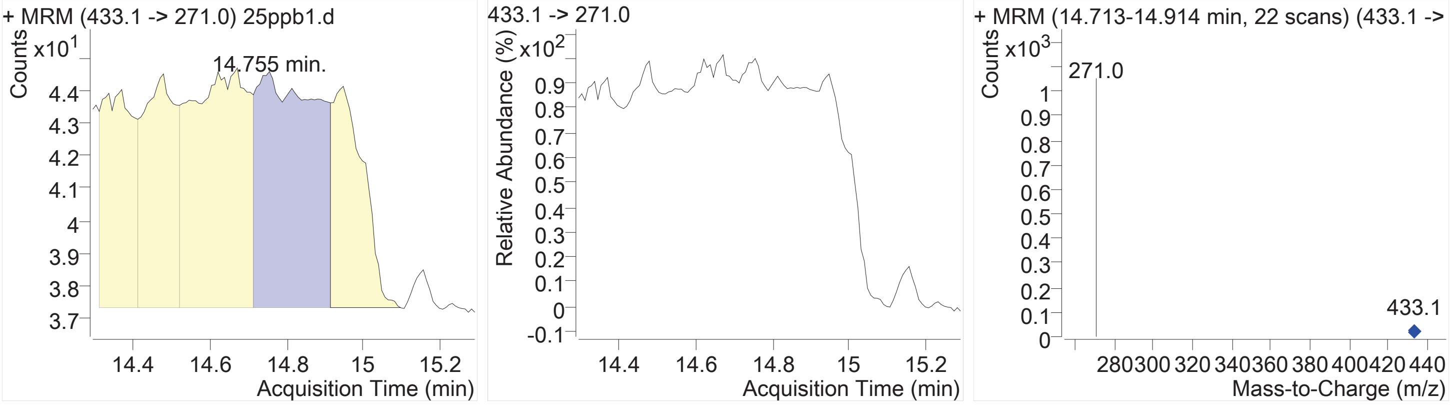

Pinoresinol

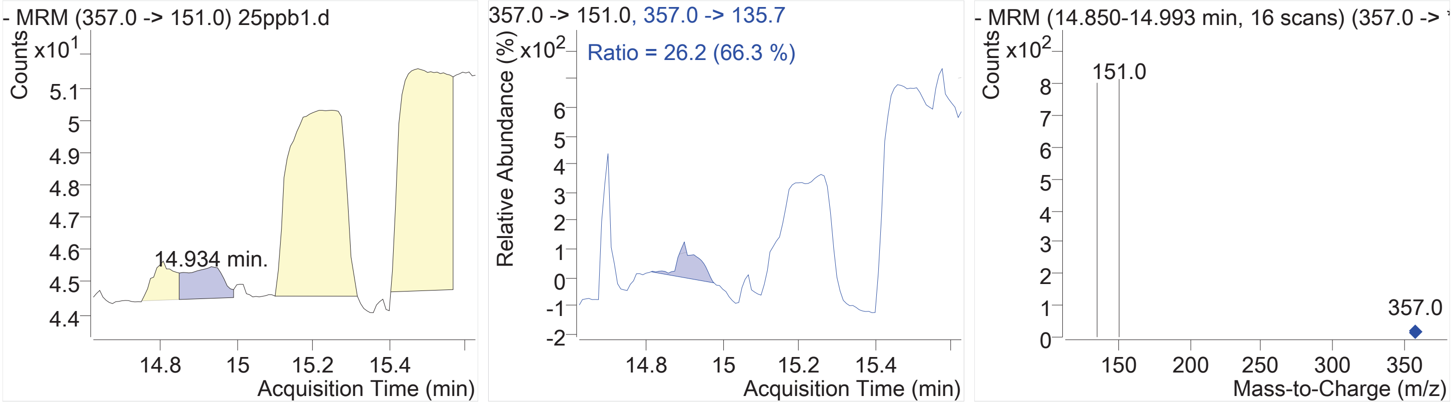

2-Hydroxycinnamic acid

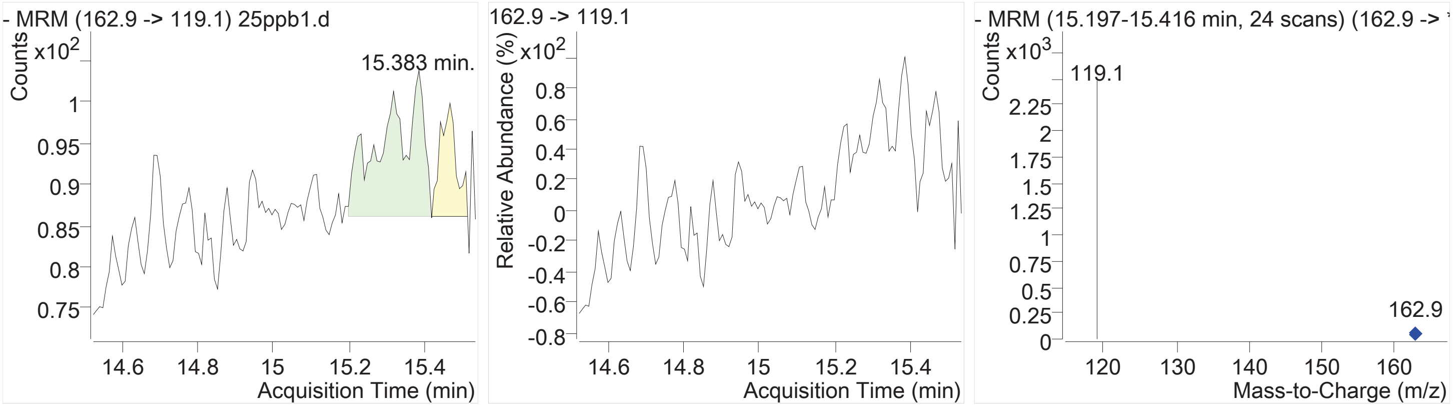

Eriodictyol

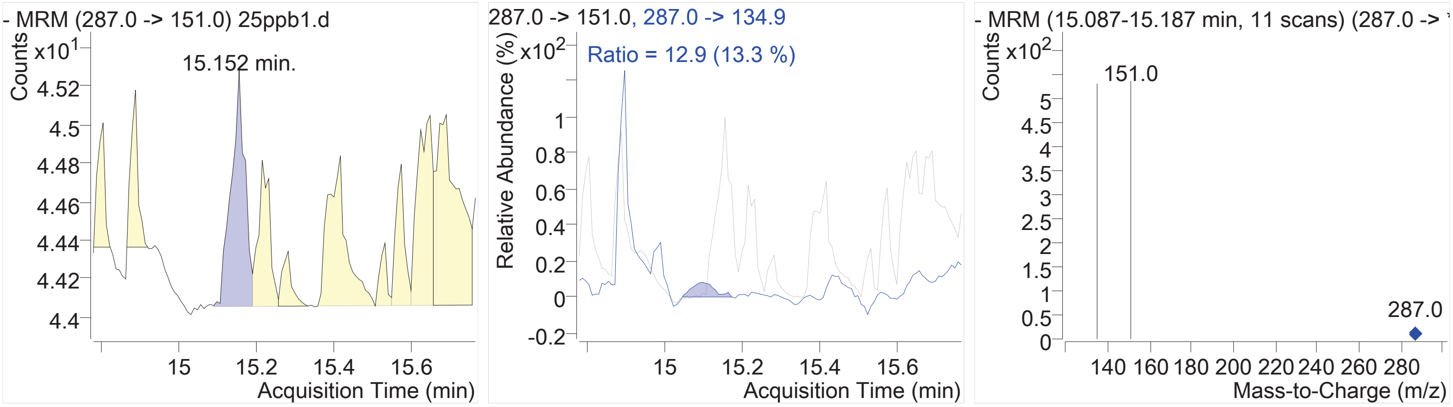

Quercetin

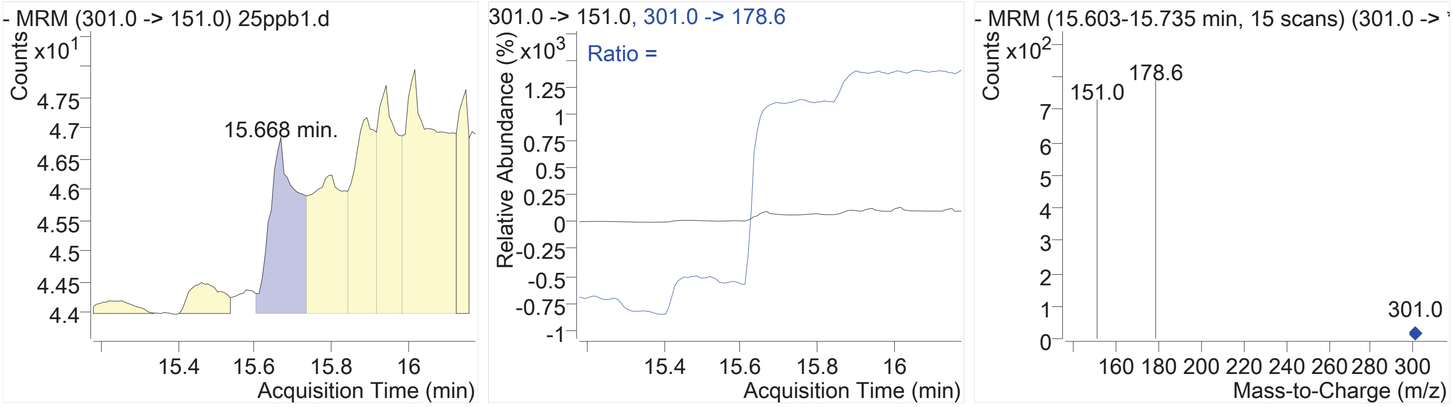

Luteolin

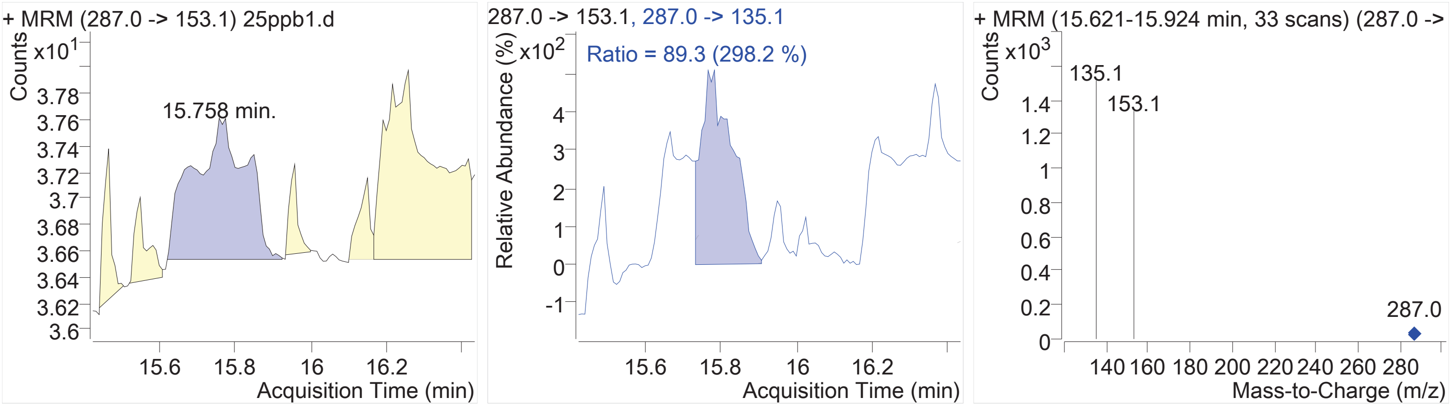

Kaempferol

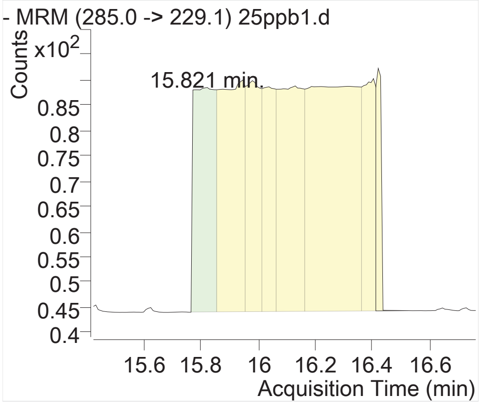

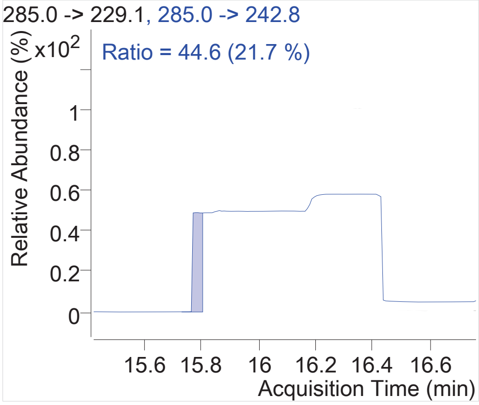

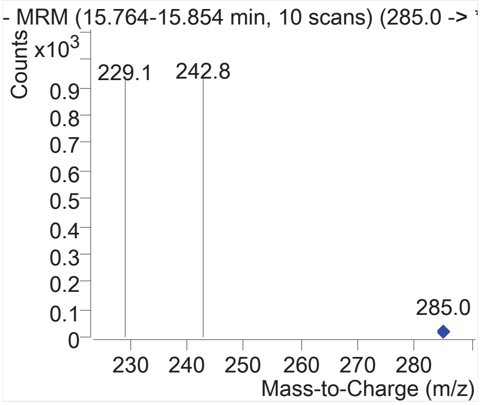

Apigenin

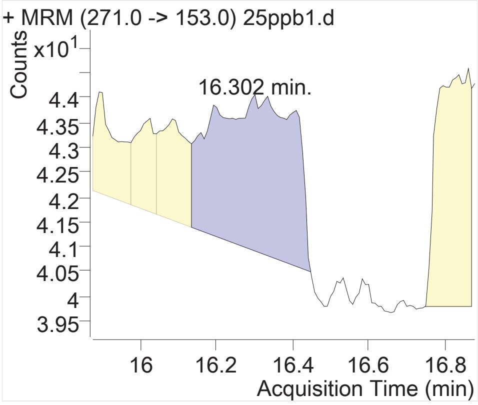

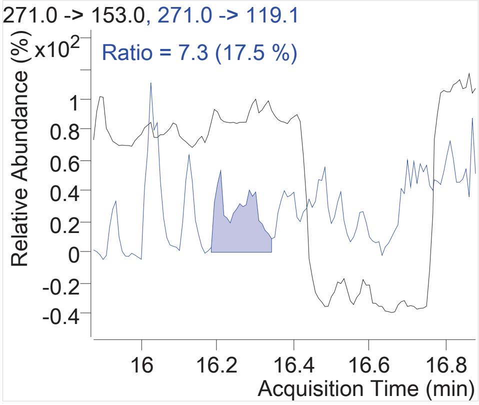

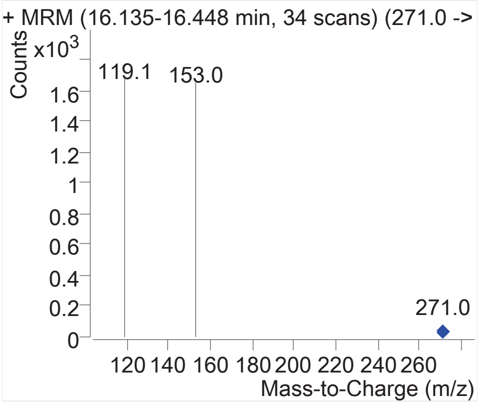

Quantitative Analysis Complete Report

Agilent Technologies

**Batch Path**	D:\MassHunter\Data\2022ekim\061022cengizhoca\QuantResults\071022.batch.bin		
**Analysis Time**	10/11/2022 1:33:26 PM	**Analyst Name**	Defam-PC\admin
**Report Time**	10/11/2022 1:33:53 PM	**Reporter Name**	admin
**Last Calib Update**	10/11/2022 1:33:17 PM	**Batch State**	Processed
**Quant Batch Version**	B.07.01	**Quant Report Version**	B.07.01

**Acq. Time**	10/6/2022 4:16:54 PM	**Data File**	25ppb2.d
**Sample Type**	Cal	**Sample Name**	25ppb2
**Dilution**	1	**Acq. Method**	FENOLIK_DMRM2021-31bilesen.m

Sample Chromatogram

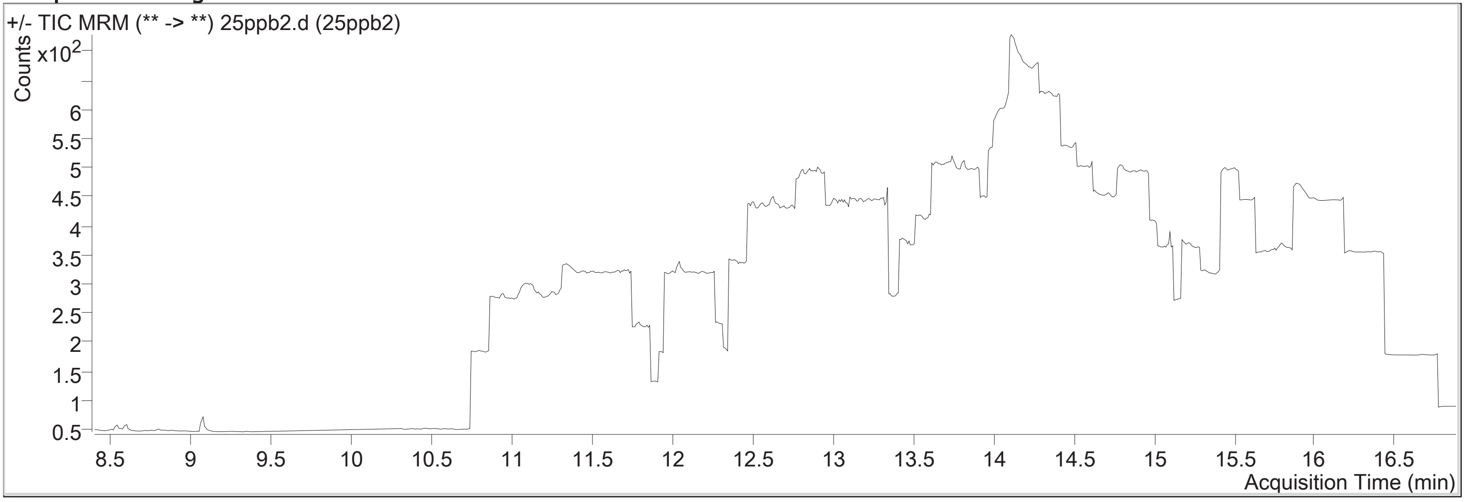

Compound	Transition	RT	Resp.	Final Conc	Units
Gallic acid	168.9 -> 125.0	8.808	6	ND	ng/ml
Protocatechuic acid	152.9 -> 108.9	10.956	18	ND	ng/ml
Pyrocatechol	109.0 -> 52.9	10.789	2	ND	ng/ml
3,4-Dihydroxyphenylacetic acid	167.0 -> 123.0	10.818	5	ND	ng/ml
(+)-Catechin	289.0 -> 245.0	11.336	60	ND	ng/ml
2,5-Dihydroxybenzoic acid	152.9 -> 109.0	12.138	105	17.1393	ng/ml
Chlorogenic acid	355.0 -> 163.0	11.785	7	6.1469	ng/ml
3-Hydroxybenzoic acid	137.0 -> 93.0	12.845	7	16.3334	ng/ml
4-Hydroxybenzoic acid	136.9 -> 93.1	12.047	26	ND	ng/ml
(-)-Epicatechin	291.0 -> 139.1	12.293	19	5.4843	ng/ml
Caffeic acid	179.0 -> 135.0	12.633	44	ND	ng/ml
Syringic acid	196.9 -> 181.9	12.681	50	73.4044	ng/ml
Vanillin	151.0 -> 136.0	13.028	16	ND	ng/ml
Verbascoside	623.0 -> 160.8	13.434	9	ND	ng/ml
Taxifolin	303.0 -> 285.1	13.695	2	ND	ng/ml
p-Coumaric acid	162.9 -> 119.0	14.117	243	0.1075	ng/ml
Sinapic acid	222.9 -> 207.9	13.823	16	9.3416	ng/ml
Ferulic acid	193.0 -> 134.0	13.950	3	0.7802	ng/ml
Luteolin 7-glucoside	447.1 -> 285.0	14.038	47	ND	ng/ml
Hesperidin	611.1 -> 303.0	14.319	2	4.3764	ng/ml
Hyperoside	465.1 -> 303.1	14.637	155	11.7859	ng/ml
Rosmarinic acid	359.0 -> 160.9	14.397	2	ND	ng/ml
Apigenin 7-glucoside	433.1 -> 271.0	14.596	213	5.0206	ng/ml
Pinoresinol	357.0 -> 151.0	14.900	6	ND	ng/ml
2-Hydroxycinnamic acid	162.9 -> 119.1	14.635	31	ND	ng/ml
Eriodictyol	287.0 -> 151.0	15.103	5	ND	ng/ml
Quercetin	301.0 -> 151.0	16.120	2	ND	ng/ml
Luteolin	287.0 -> 153.1	15.808	15	ND	ng/ml
Kaempferol	285.0 -> 229.1	15.796	186	13.1101	ng/ml

Quantitative Analysis Complete Report

Compound	Transition	RT	Resp.	Final Conc	Units
Apigenin	271.0 -> 153.0	16.001	5	ND	ng/ml

Gallic acid

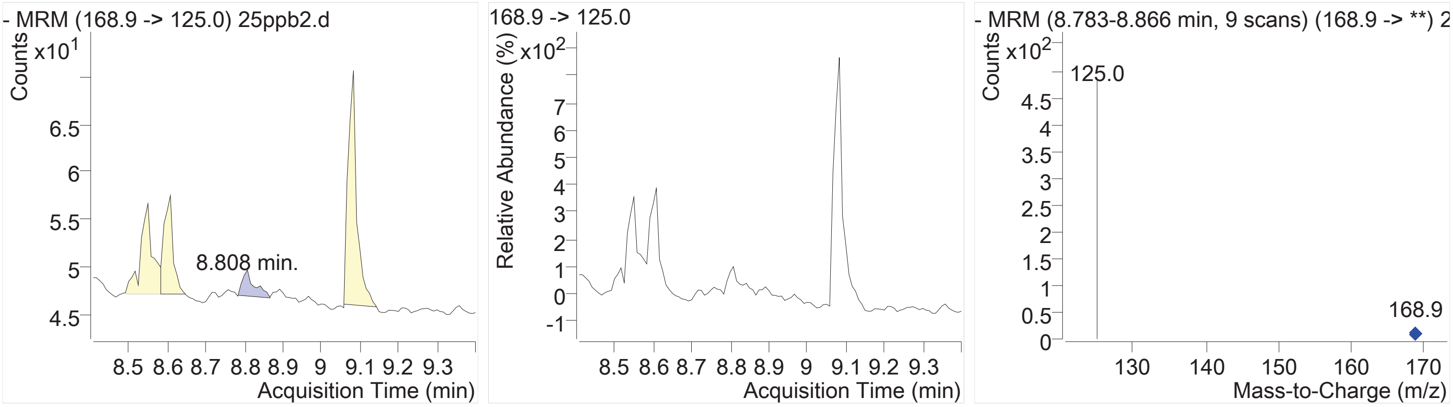

Protocatechuic acid

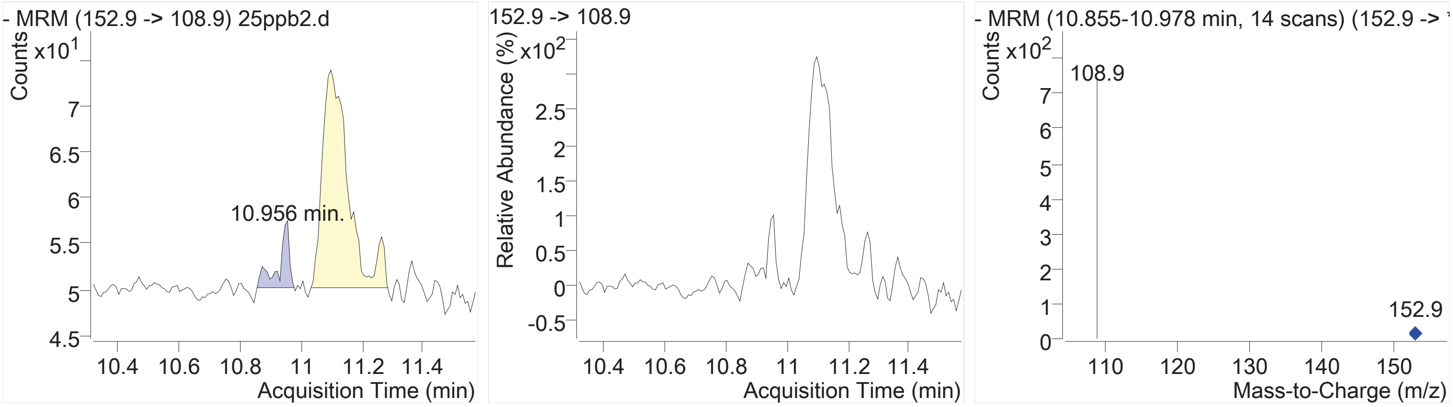

Pyrocatechol

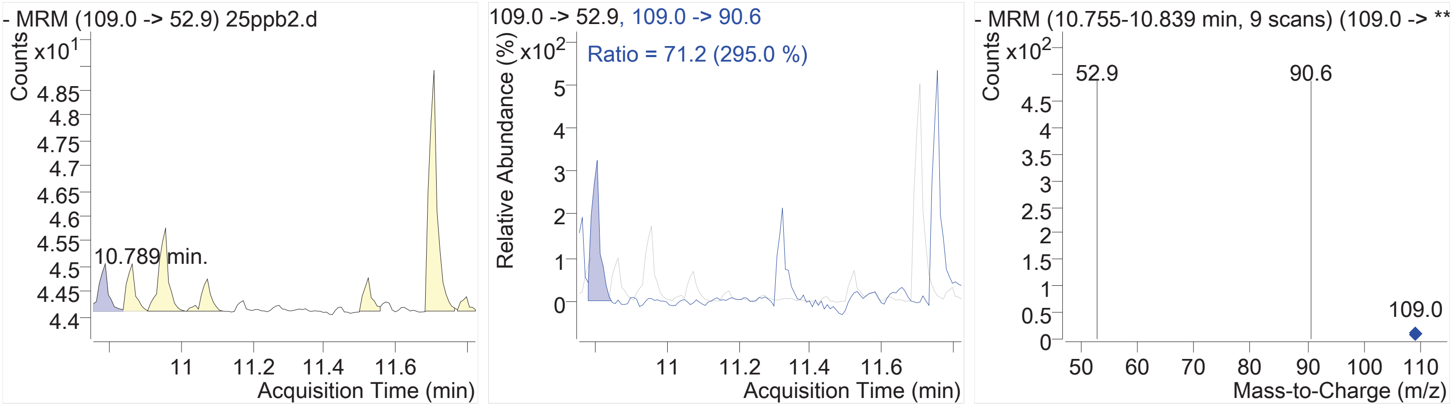

3,4-Dihydroxyphenylacetic acid

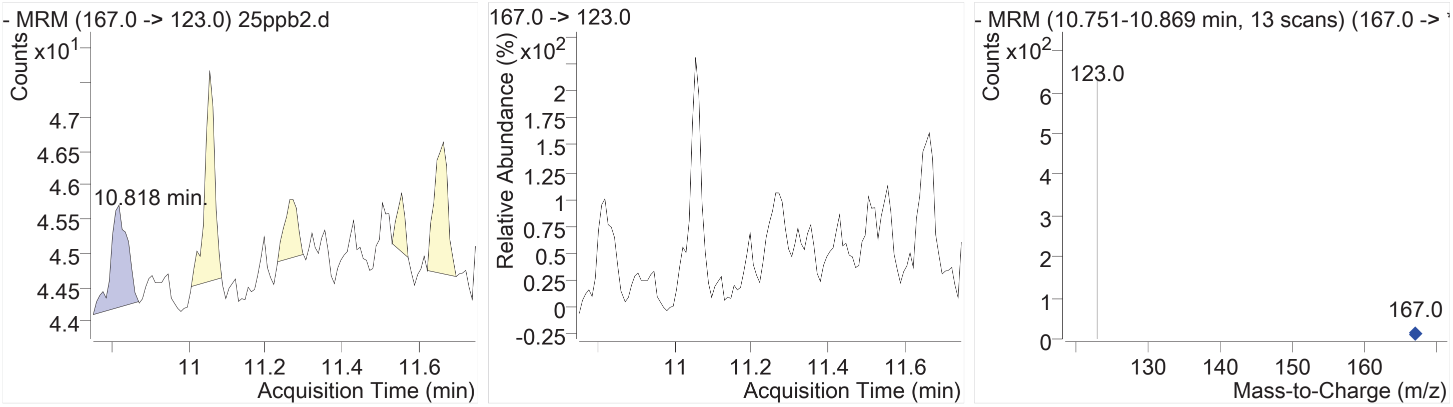

Agilent Technologies

(+)-Catechin

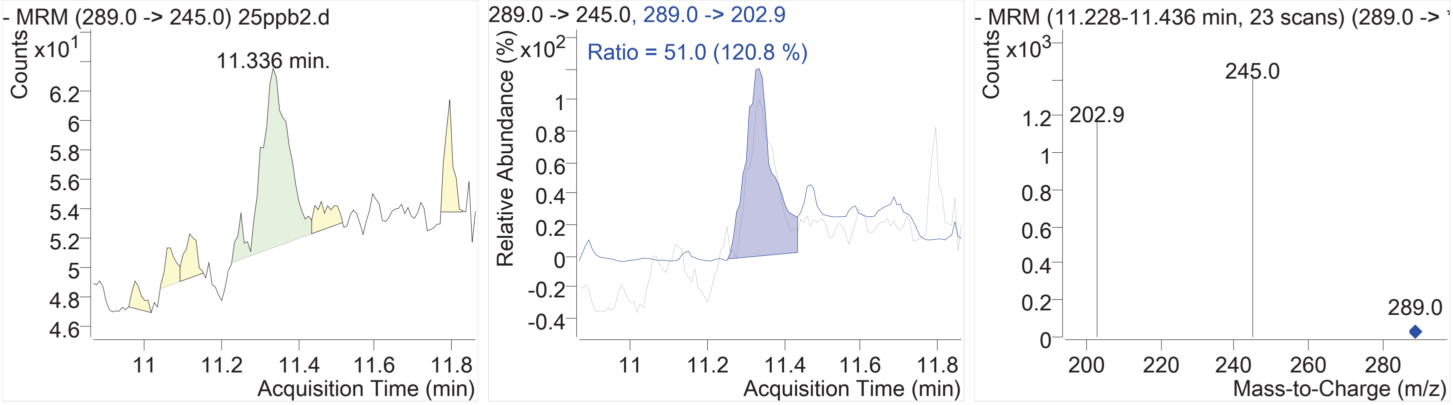

2,5-Dihydroxybenzoic acid

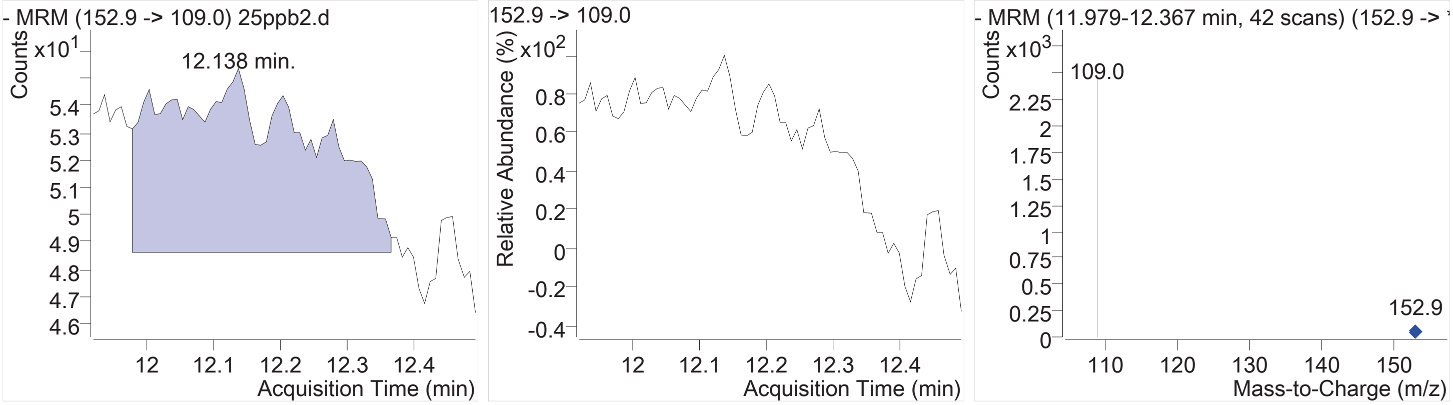

Chlorogenic acid

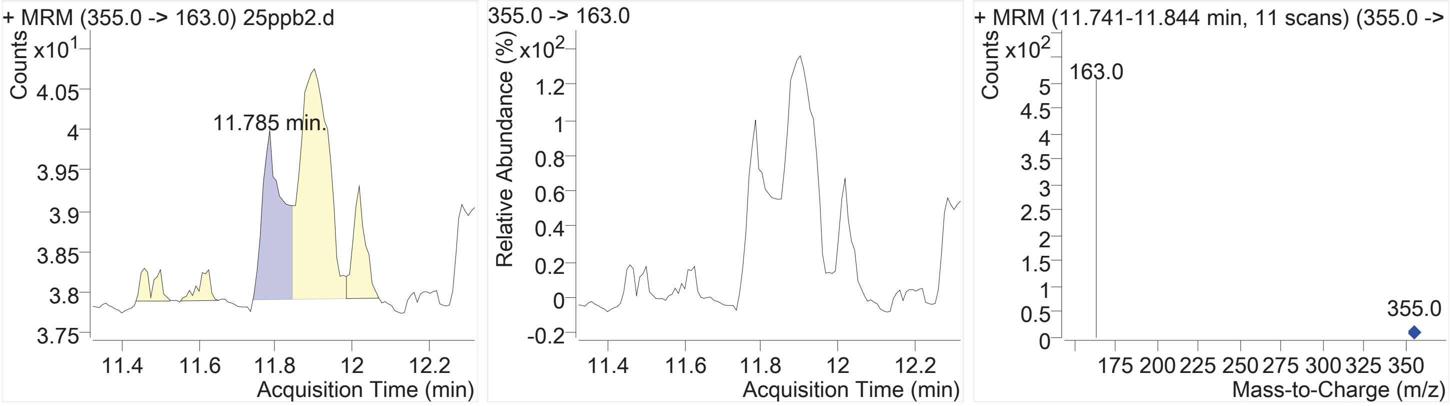

3-Hydroxybenzoic acid

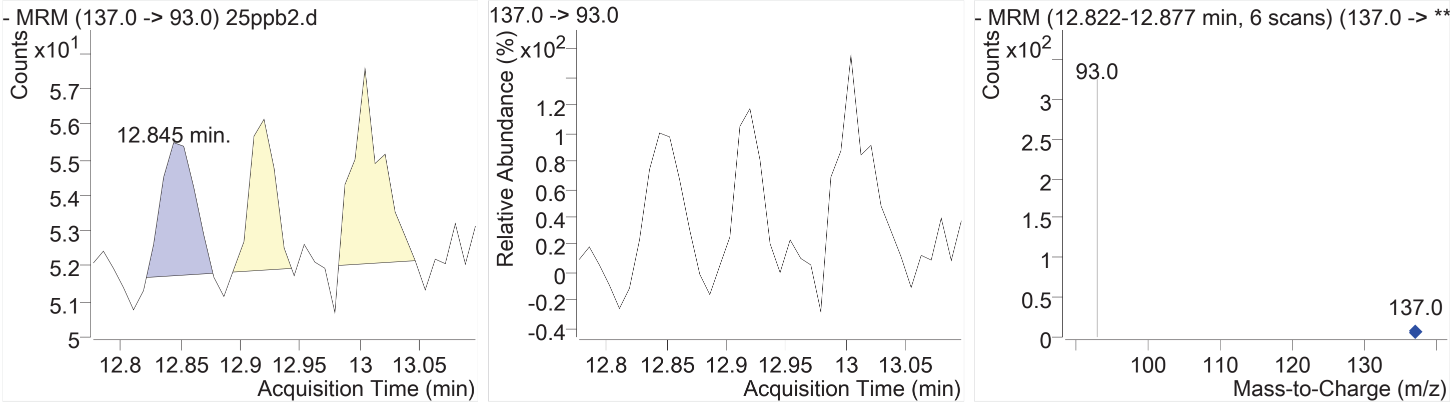

4-Hydroxybenzoic acid

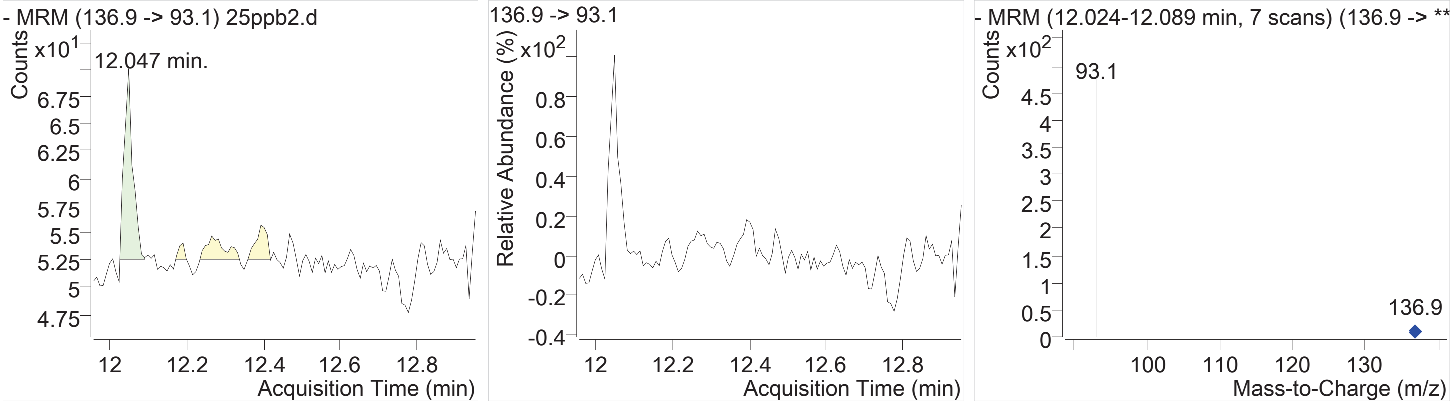

(-)-Epicatechin

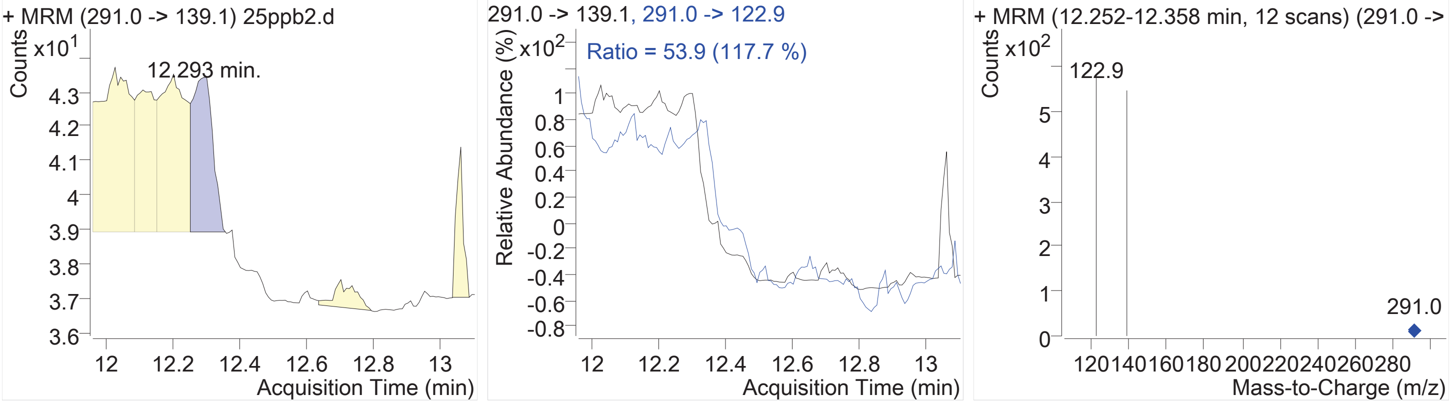

Caffeic acid

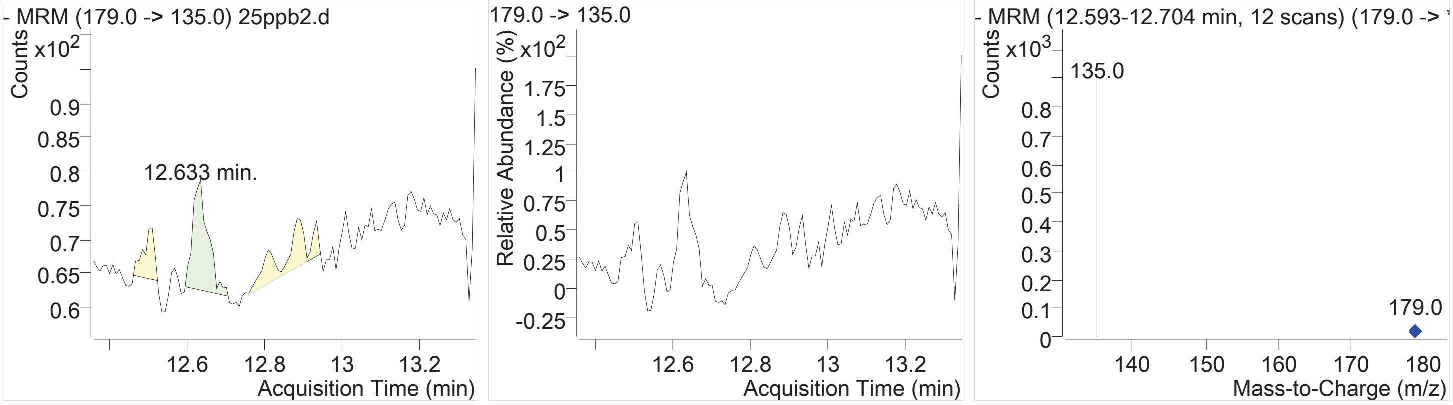

Syringic acid

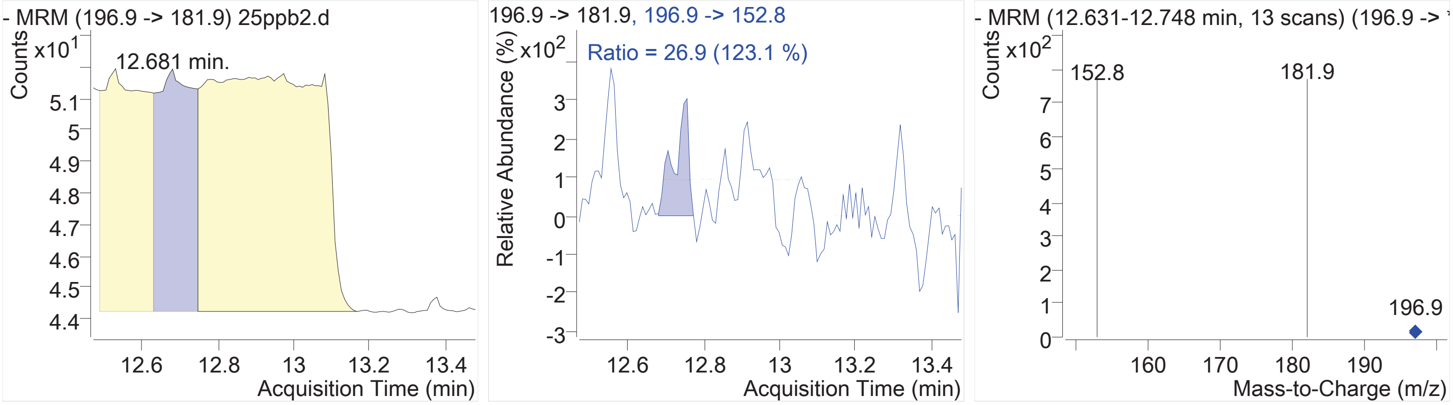

Vanillin

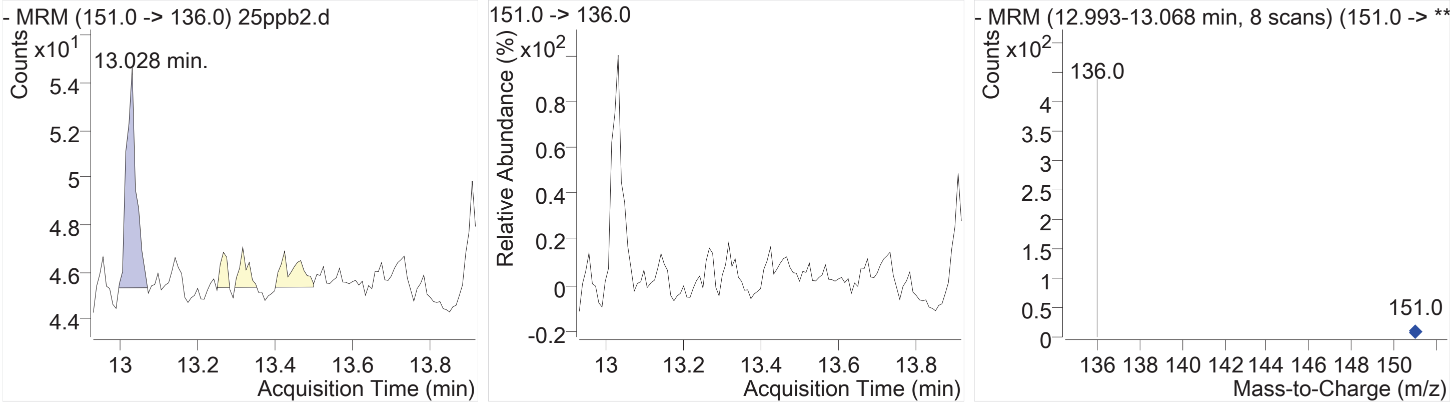

Verbascoside

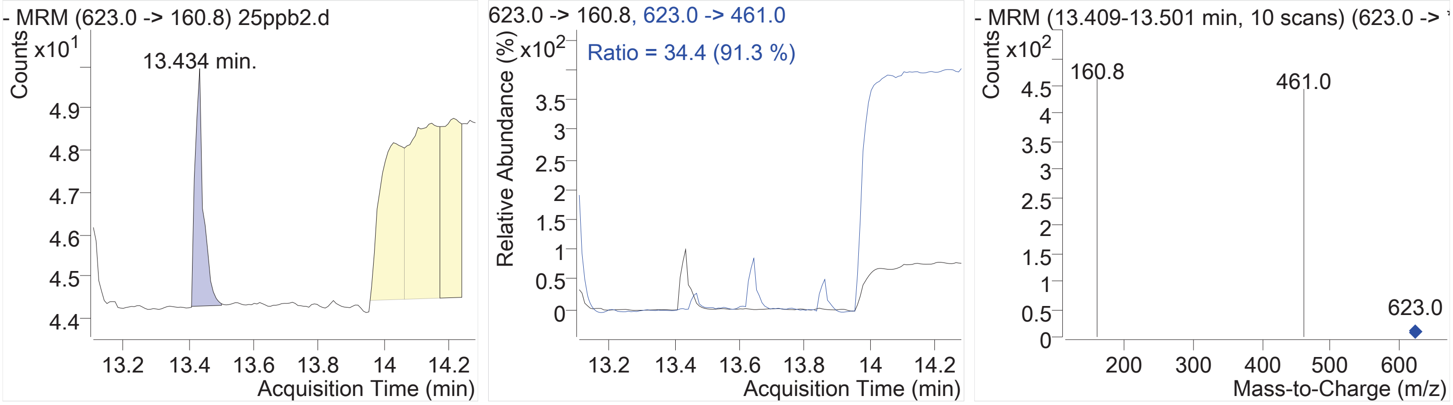

Taxifolin

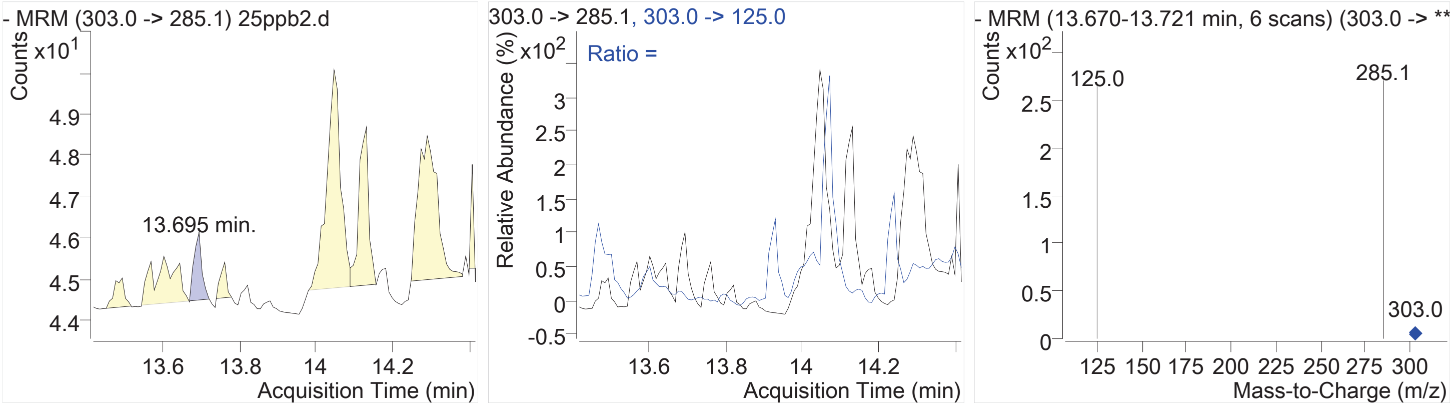

p-Coumaric acid

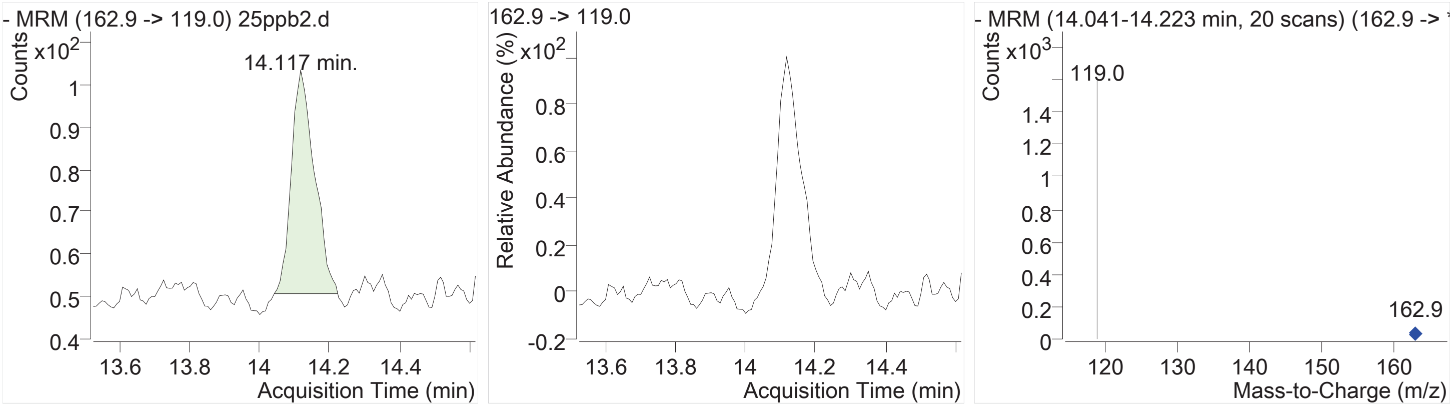

Sinapic acid

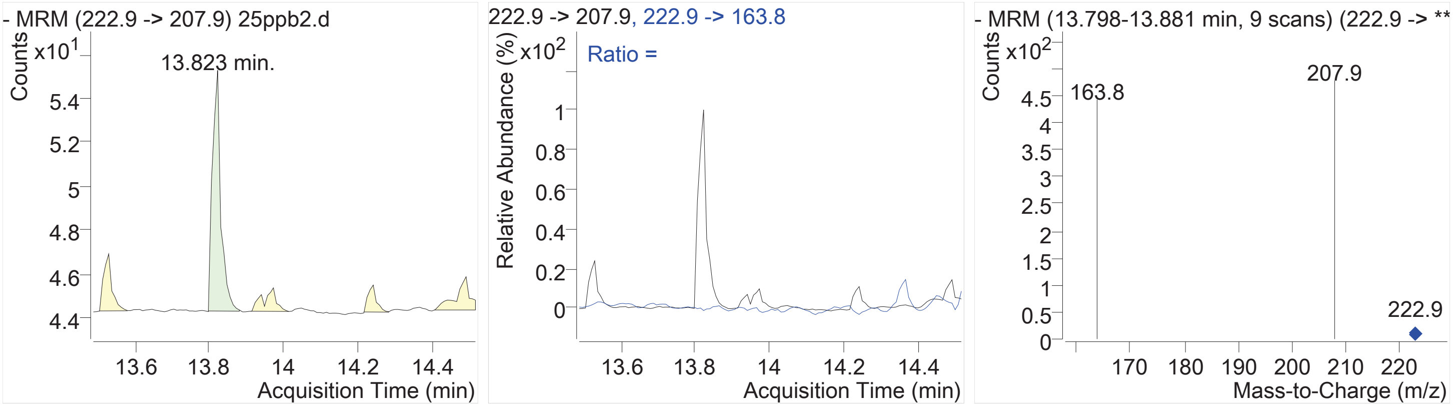

Ferulic acid

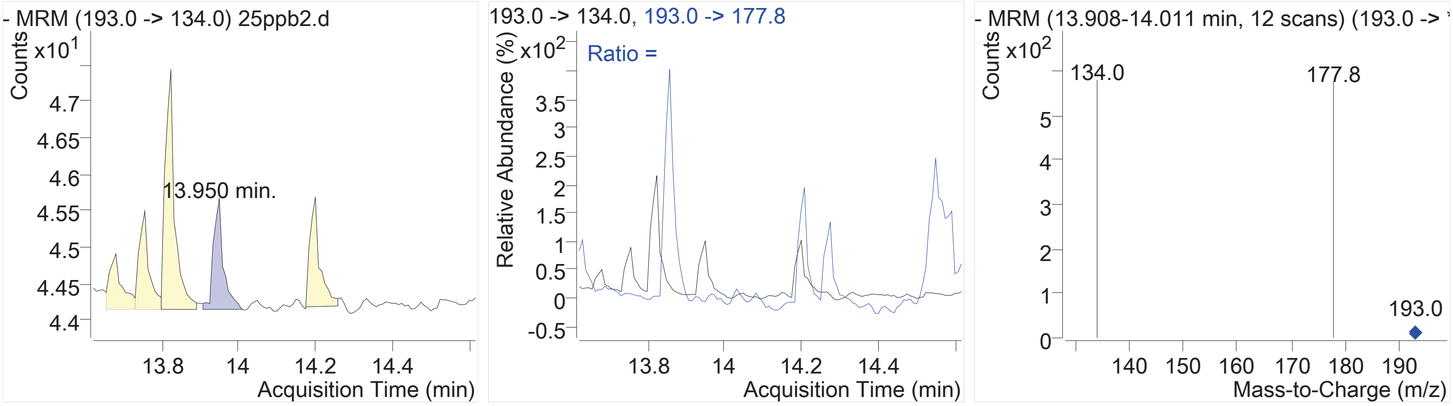

Luteolin 7-glucoside

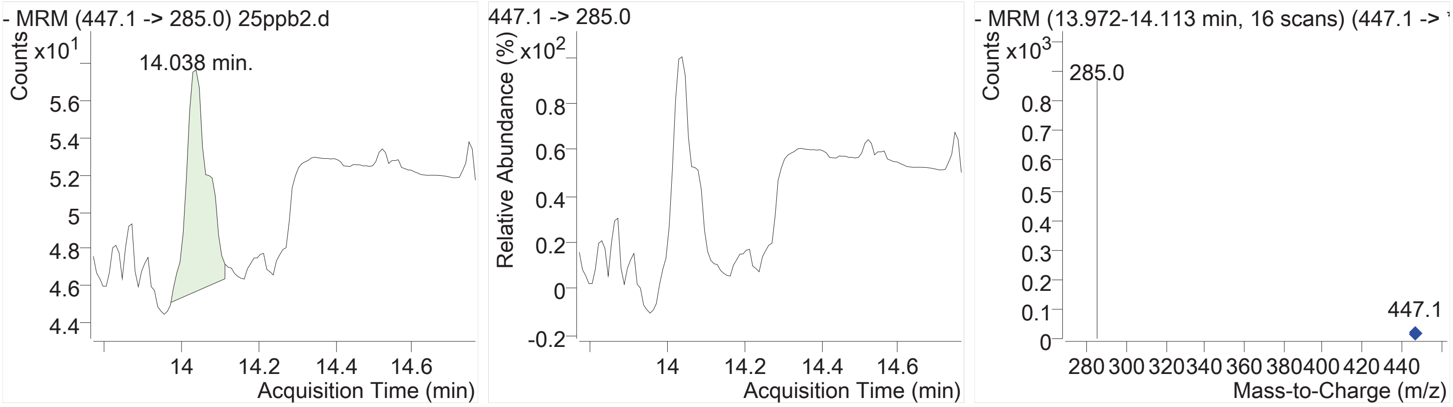

Hesperidin

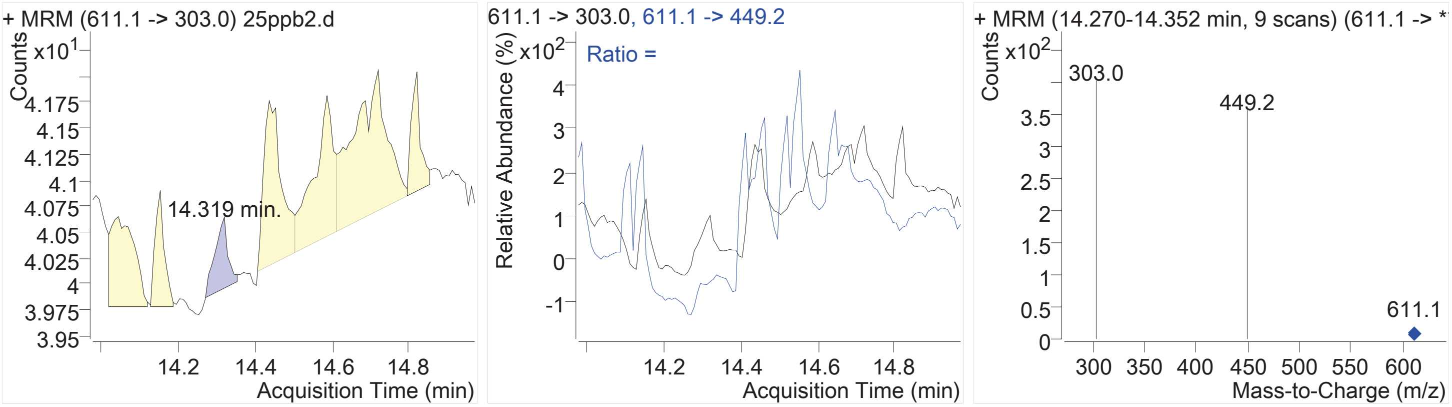

Agilent Technologies

Hyperoside

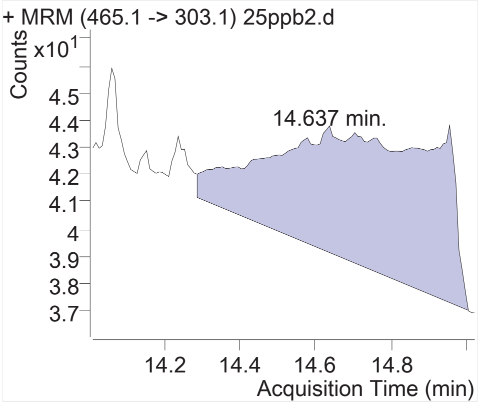

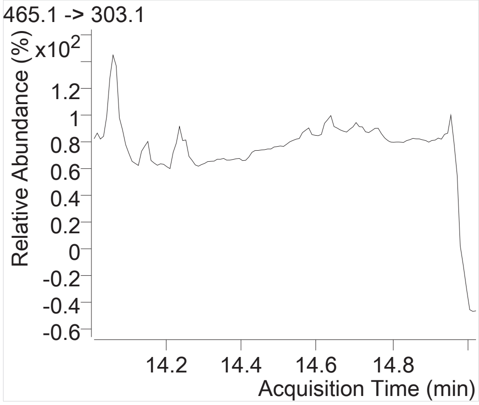

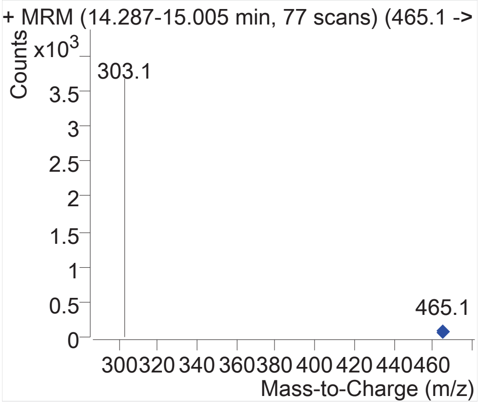

Rosmarinic acid

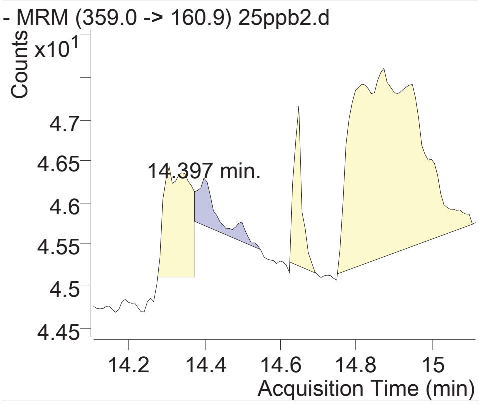

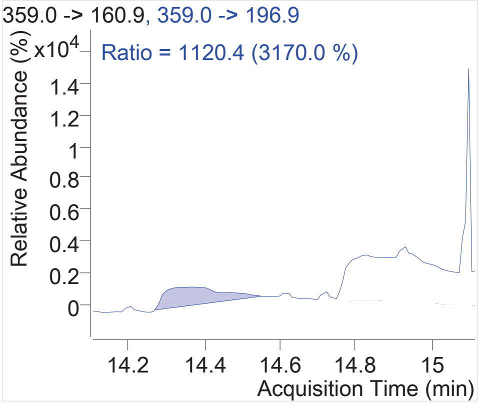

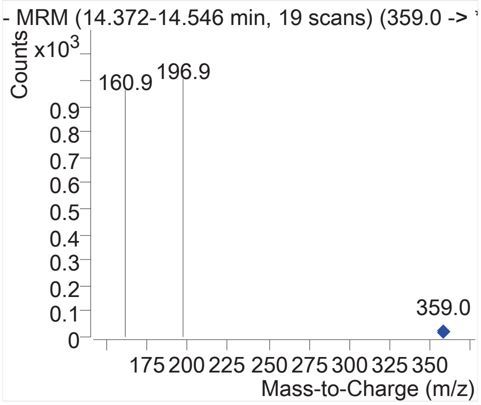

Apigenin 7-glucoside

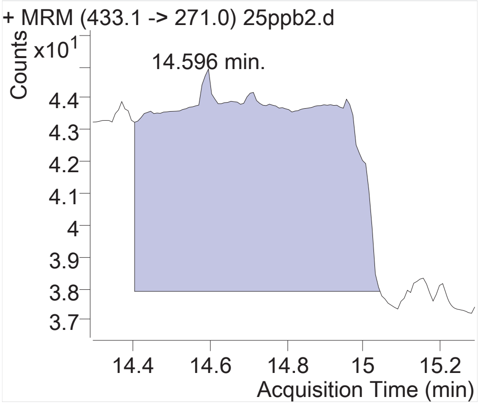

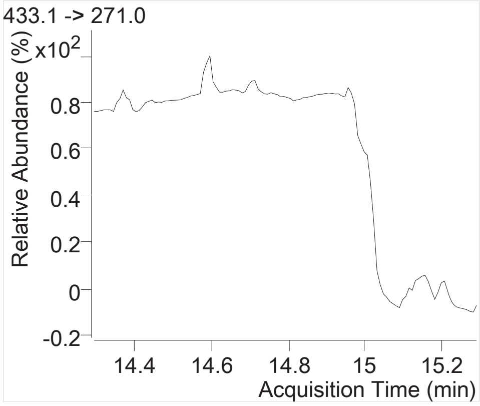

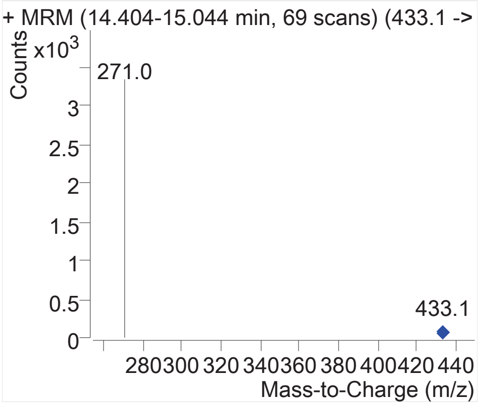

Pinoresinol

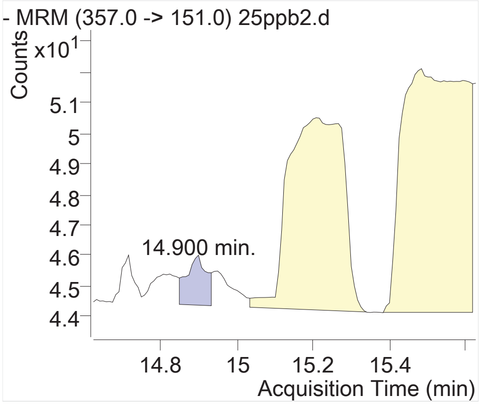

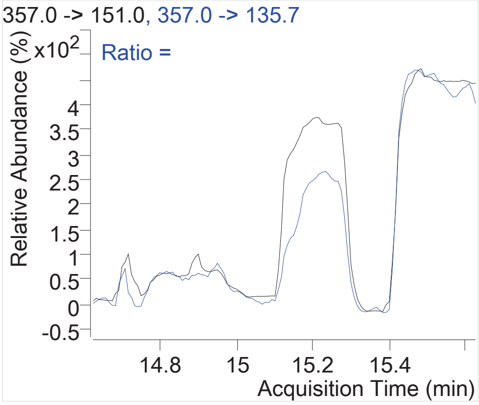

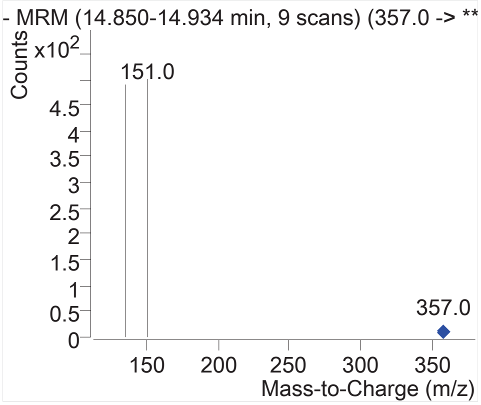

2-Hydroxycinnamic acid

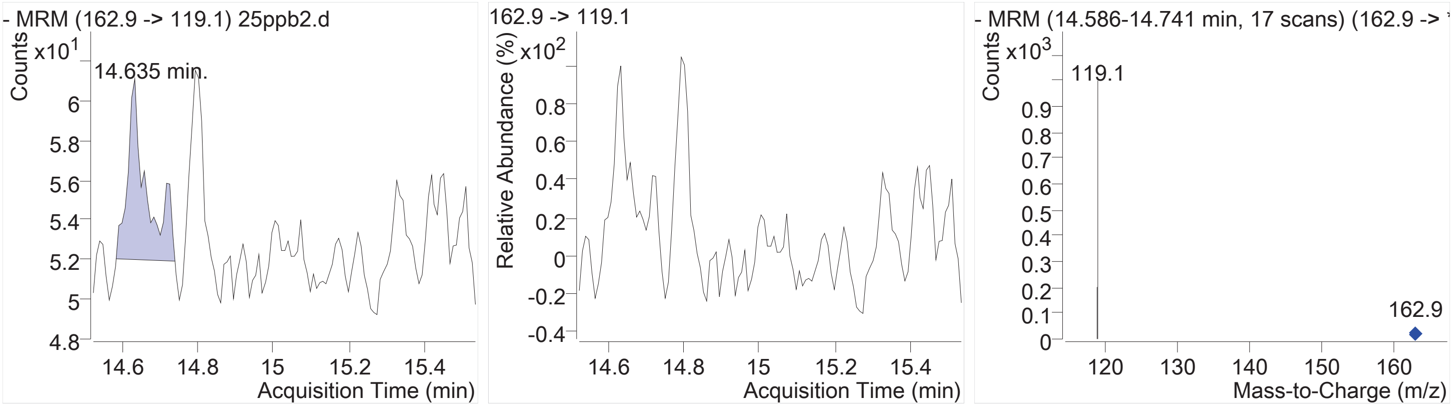

Eriodictyol

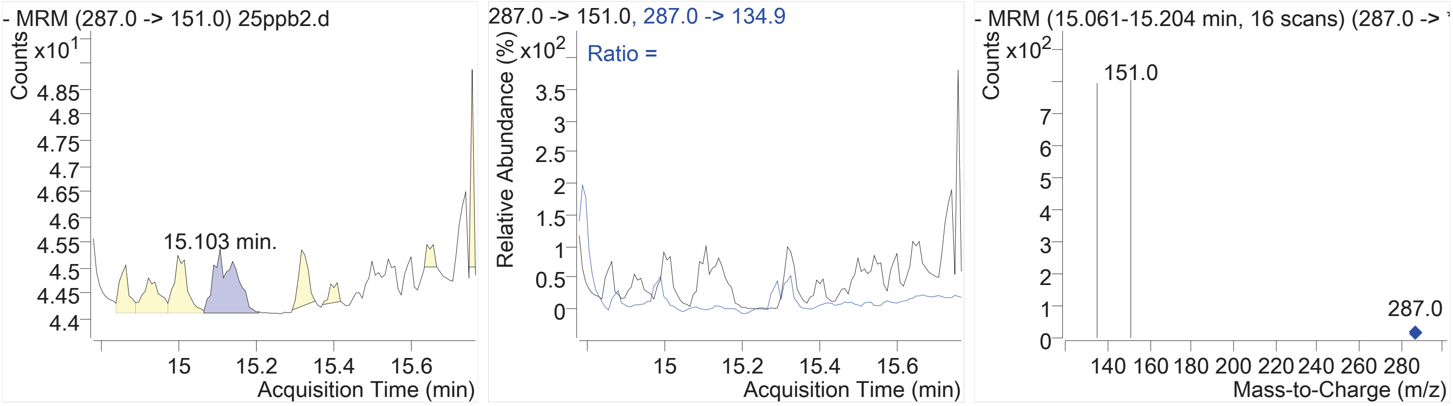

Quercetin

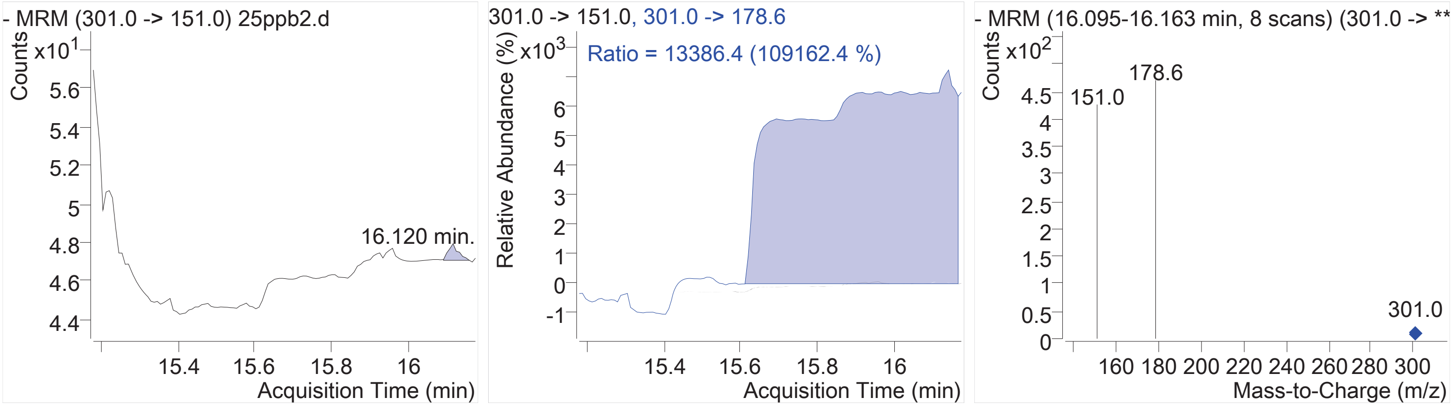

Luteolin

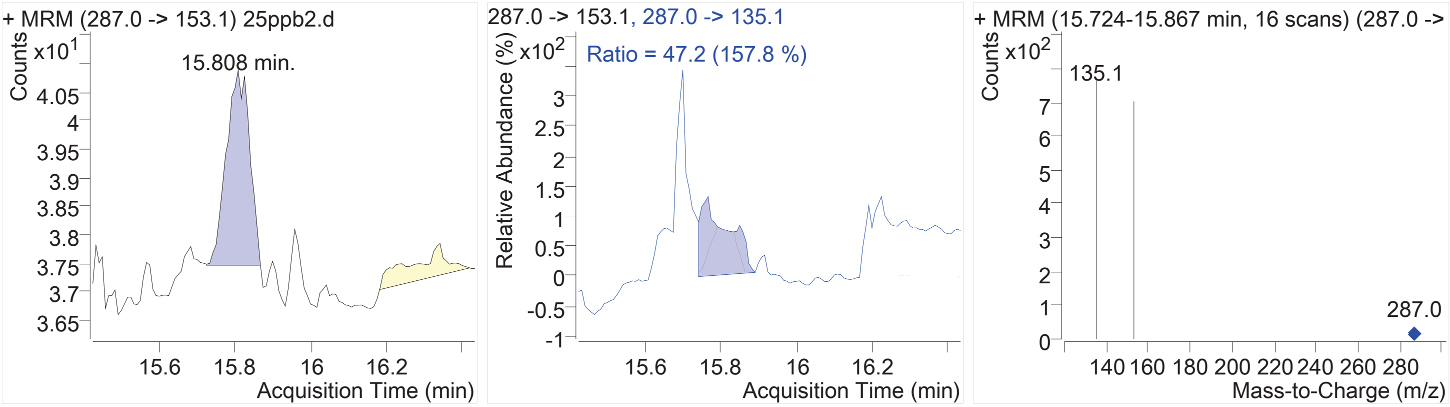

Kaempferol

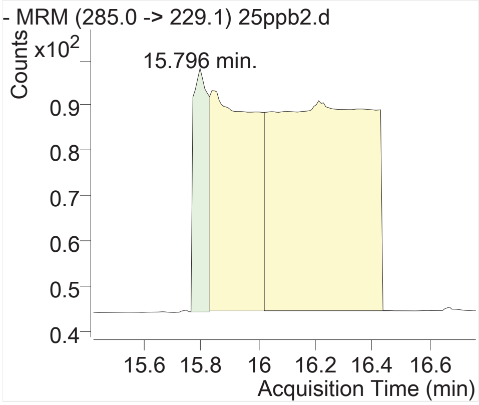

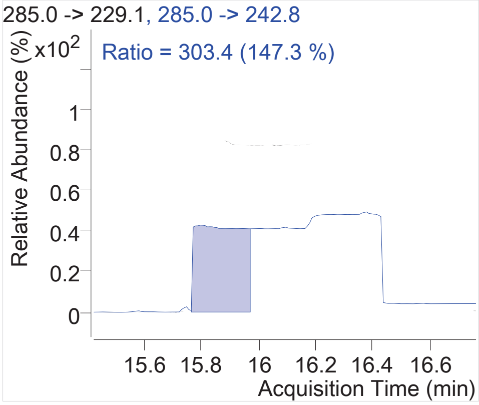

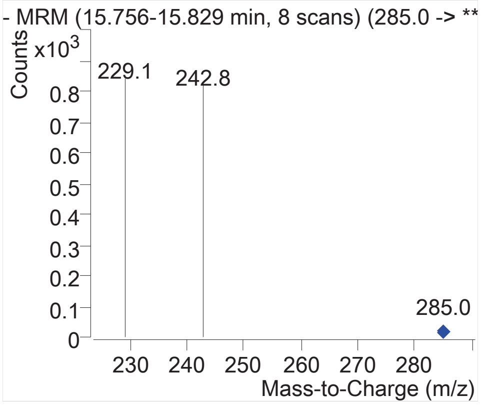

Apigenin

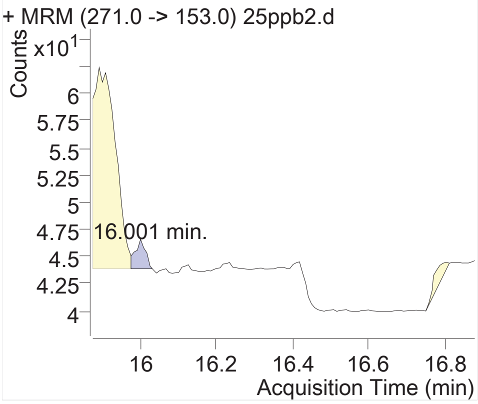

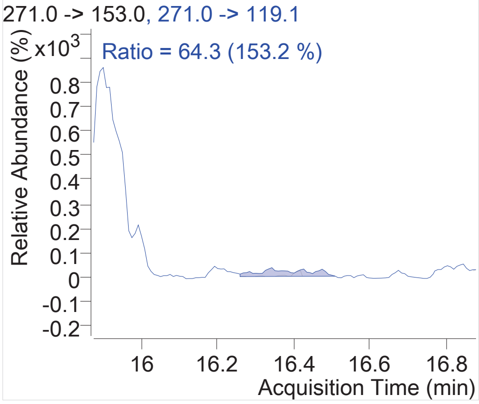

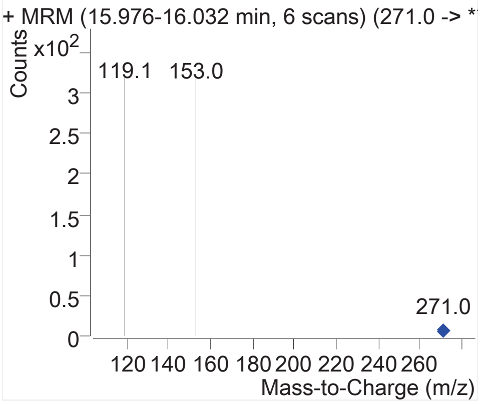

Quantitative Analysis Complete Report

**Batch Path**	D:\MassHunter\Data\2022ekim\061022cengizhoca\QuantResults\071022.batch.bin		
**Analysis Time**	10/11/2022 1:33:26 PM	**Analyst Name**	Defam-PC\admin
**Report Time**	10/11/2022 1:33:56 PM	**Reporter Name**	admin
**Last Calib Update**	10/11/2022 1:33:17 PM	**Batch State**	Processed
**Quant Batch Version**	B.07.01	**Quant Report Version**	B.07.01

**Acq. Time**	10/6/2022 4:38:34 PM	**Data File**	25ppb3.d
**Sample Type**	Cal	**Sample Name**	25ppb3
**Dilution**	1	**Acq. Method**	FENOLIK_DMRM2021-31bilesen.m

Sample Chromatogram

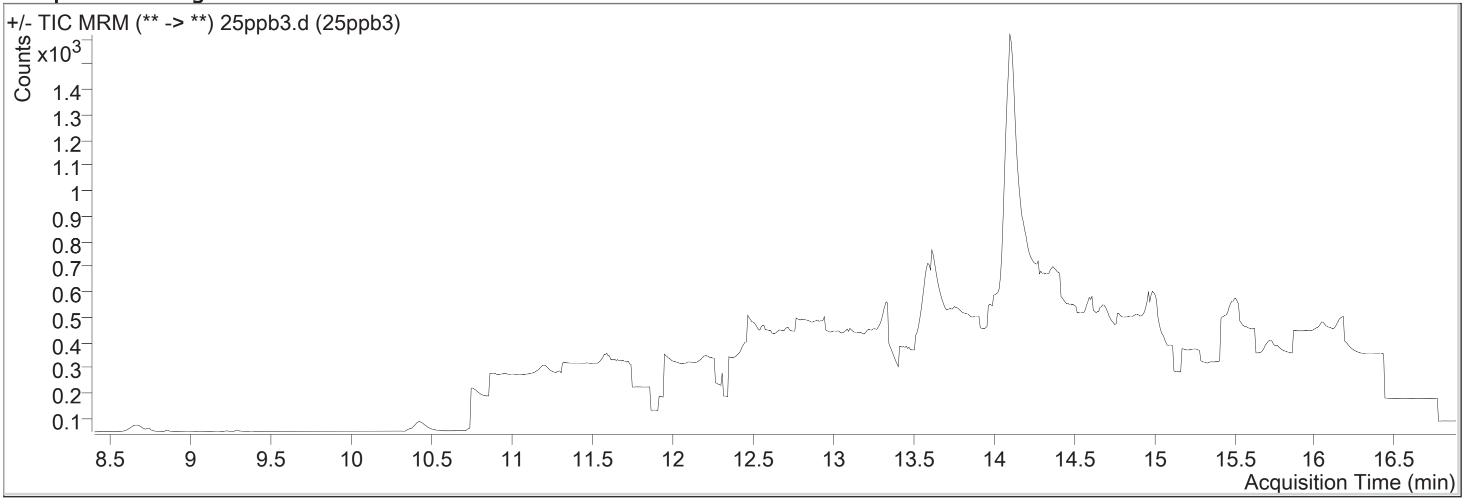

Compound	Transition	RT	Resp.	Final Conc	Units
Gallic acid	168.9 -> 125.0	8.666	171	ND	ng/ml
Protocatechuic acid	152.9 -> 108.9	10.426	204	7.9958	ng/ml
Pyrocatechol	109.0 -> 52.9	10.856	3	ND	ng/ml
3,4-Dihydroxyphenylacetic acid	167.0 -> 123.0	10.760	112	5.2926	ng/ml
(+)-Catechin	289.0 -> 245.0	11.202	111	4.1059	ng/ml
2,5-Dihydroxybenzoic acid	152.9 -> 109.0	12.013	127	20.6299	ng/ml
Chlorogenic acid	355.0 -> 163.0	11.793	14	6.4463	ng/ml
3-Hydroxybenzoic acid	137.0 -> 93.0	12.911	16	18.0074	ng/ml
4-Hydroxybenzoic acid	136.9 -> 93.1	12.097	8	ND	ng/ml
(-)-Epicatechin	291.0 -> 139.1	12.211	133	19.9201	ng/ml
Caffeic acid	179.0 -> 135.0	12.474	371	ND	ng/ml
Syringic acid	196.9 -> 181.9	12.556	14	22.0241	ng/ml
Vanillin	151.0 -> 136.0	12.953	45	ND	ng/ml
Verbascoside	623.0 -> 160.8	13.334	395	26.0474	ng/ml
Taxifolin	303.0 -> 285.1	13.595	932	ND	ng/ml
p-Coumaric acid	162.9 -> 119.0	13.632	486	9.5755	ng/ml
Sinapic acid	222.9 -> 207.9	13.730	50	19.9996	ng/ml
Ferulic acid	193.0 -> 134.0	13.891	9	2.0503	ng/ml
Luteolin 7-glucoside	447.1 -> 285.0	14.104	4730	18.3184	ng/ml
Hesperidin	611.1 -> 303.0	14.202	99	16.1794	ng/ml
Hyperoside	465.1 -> 303.1	14.303	196	13.8627	ng/ml
Rosmarinic acid	359.0 -> 160.9	14.372	207	15.2585	ng/ml
Apigenin 7-glucoside	433.1 -> 271.0	14.630	337	8.7149	ng/ml
Pinoresinol	357.0 -> 151.0	14.909	14	29.4558	ng/ml
2-Hydroxycinnamic acid	162.9 -> 119.1	14.693	322	13.3673	ng/ml
Eriodictyol	287.0 -> 151.0	15.002	482	ND	ng/ml
Quercetin	301.0 -> 151.0	15.502	290	5.5347	ng/ml
Luteolin	287.0 -> 153.1	15.733	178	6.2745	ng/ml
Kaempferol	285.0 -> 229.1	16.046	98	ND	ng/ml

Compound	Transition	RT	Resp.	Final Conc	Units
Apigenin	271.0 -> 153.0	16.193	177	3.6625	ng/ml

Gallic acid

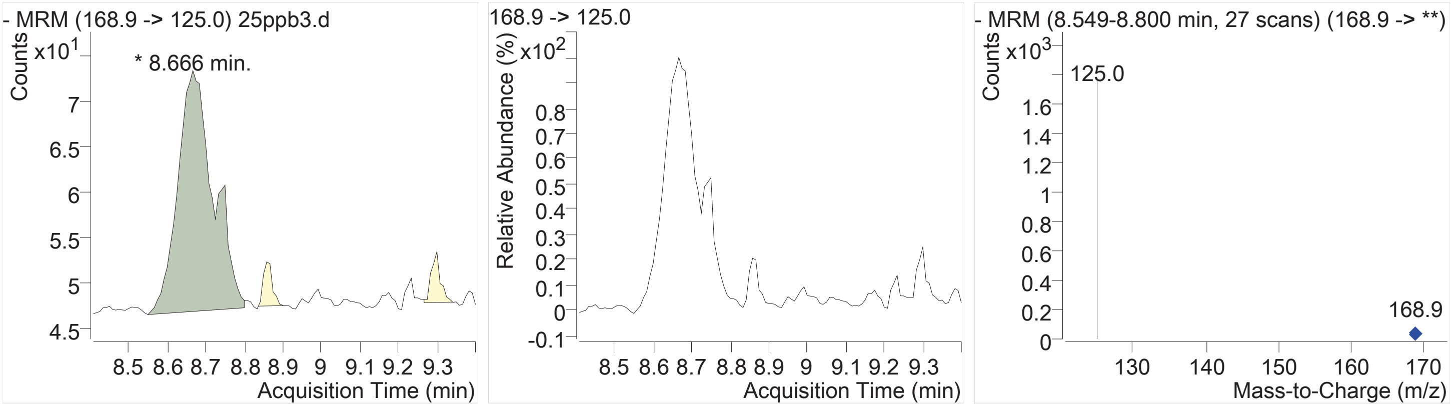

Protocatechuic acid

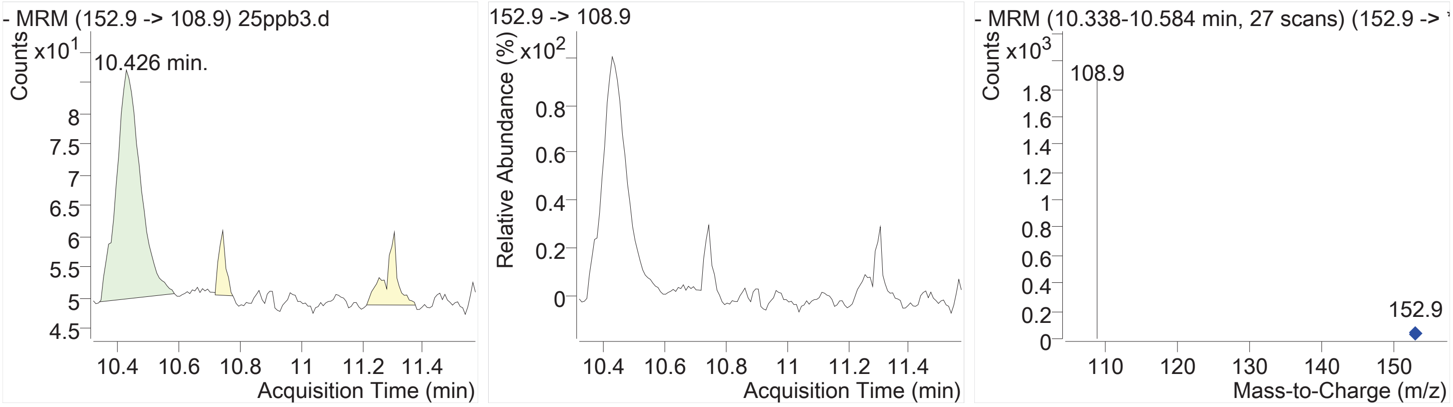

Pyrocatechol

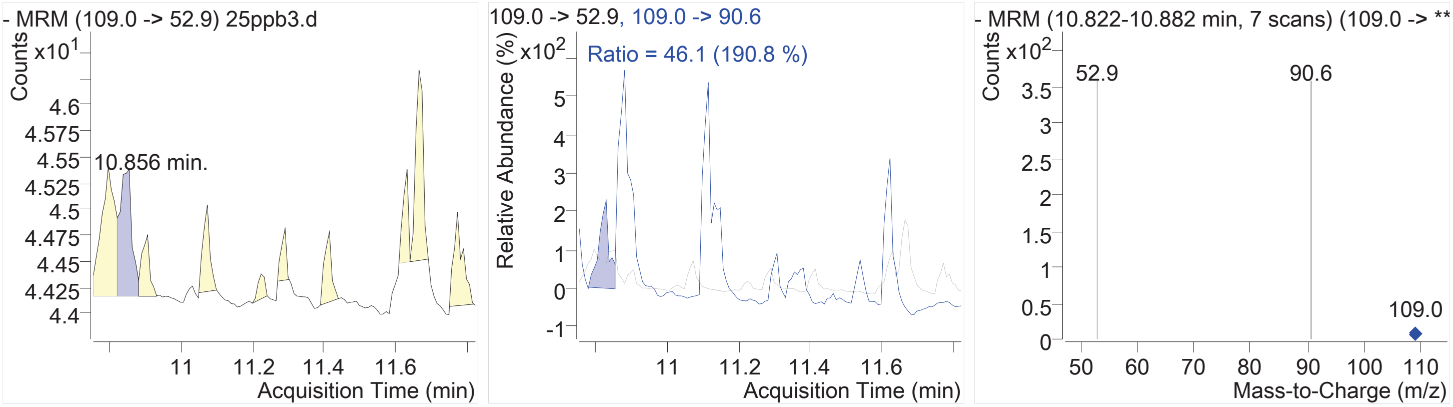

3,4-Dihydroxyphenylacetic acid

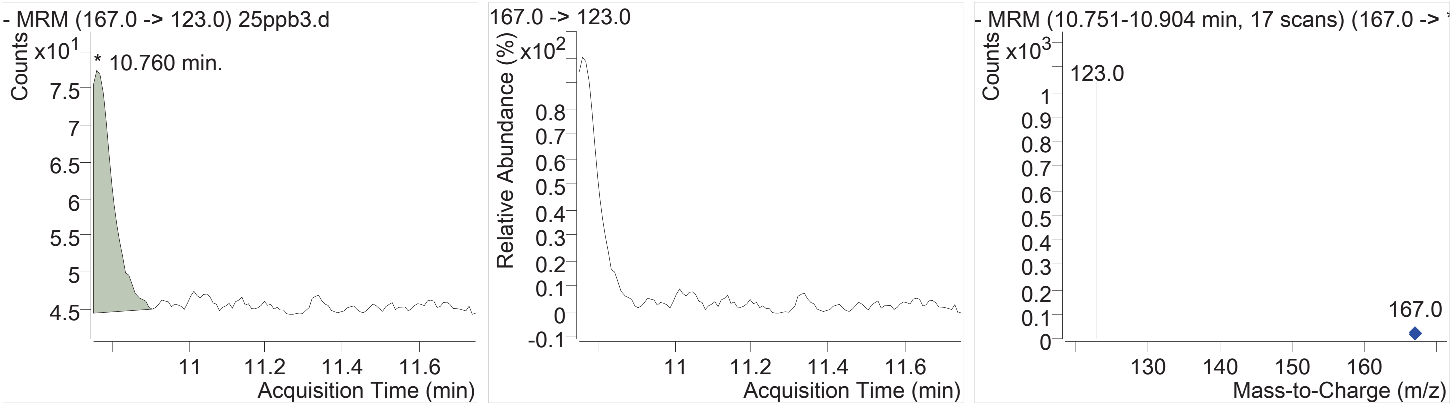

Agilent Technologies

(+)-Catechin

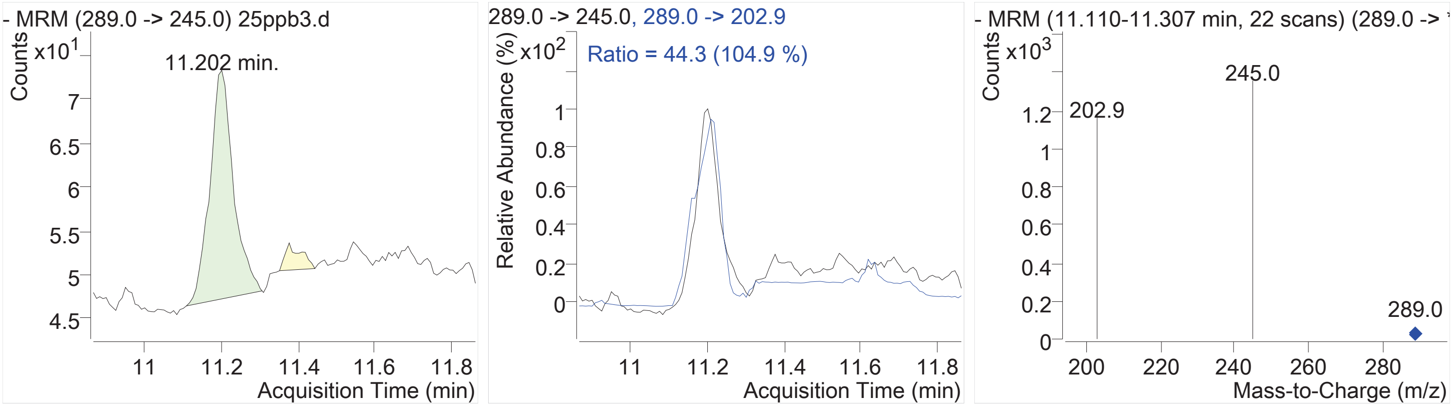

2,5-Dihydroxybenzoic acid

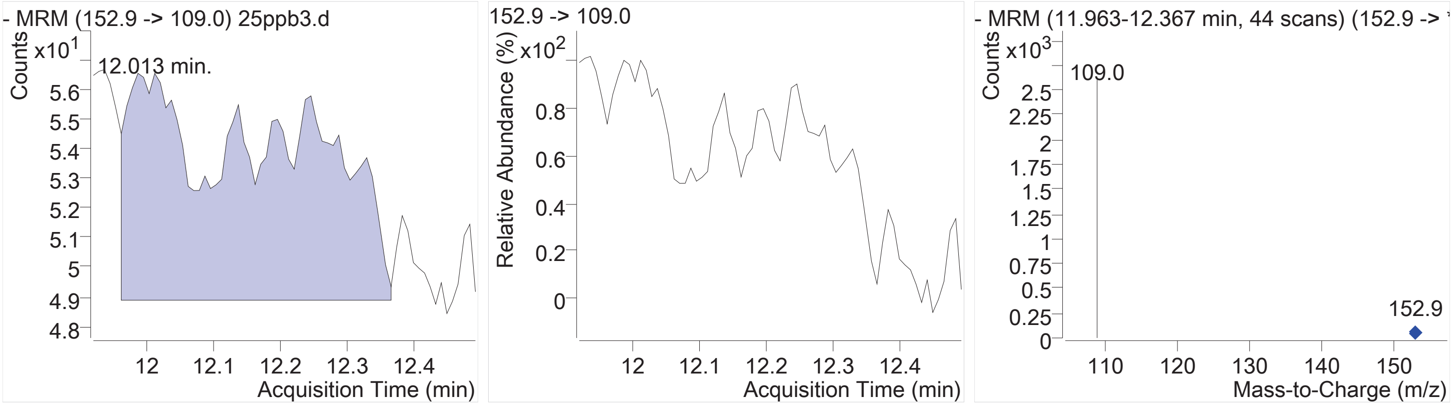

Chlorogenic acid

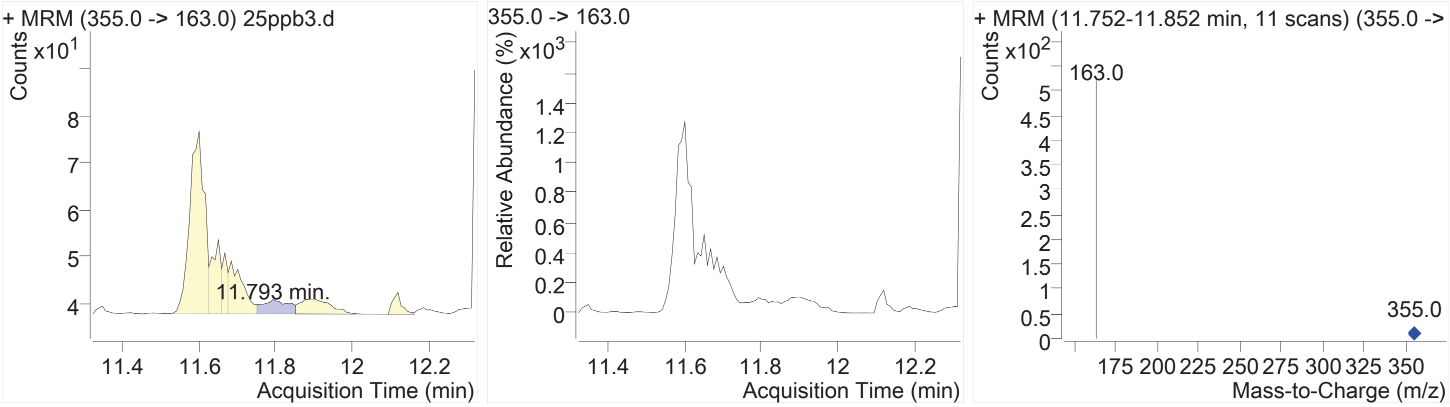

3-Hydroxybenzoic acid

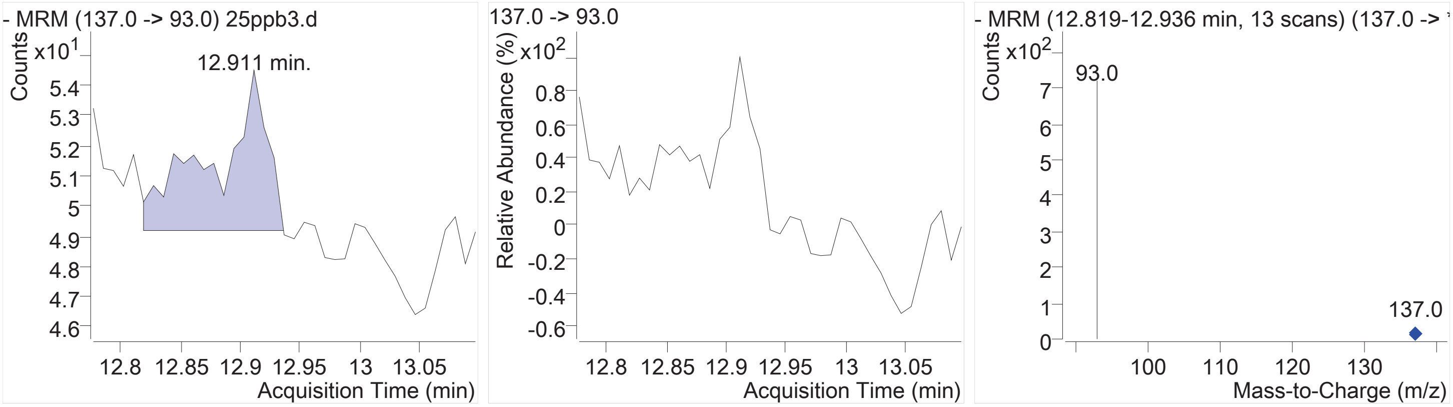

4-Hydroxybenzoic acid

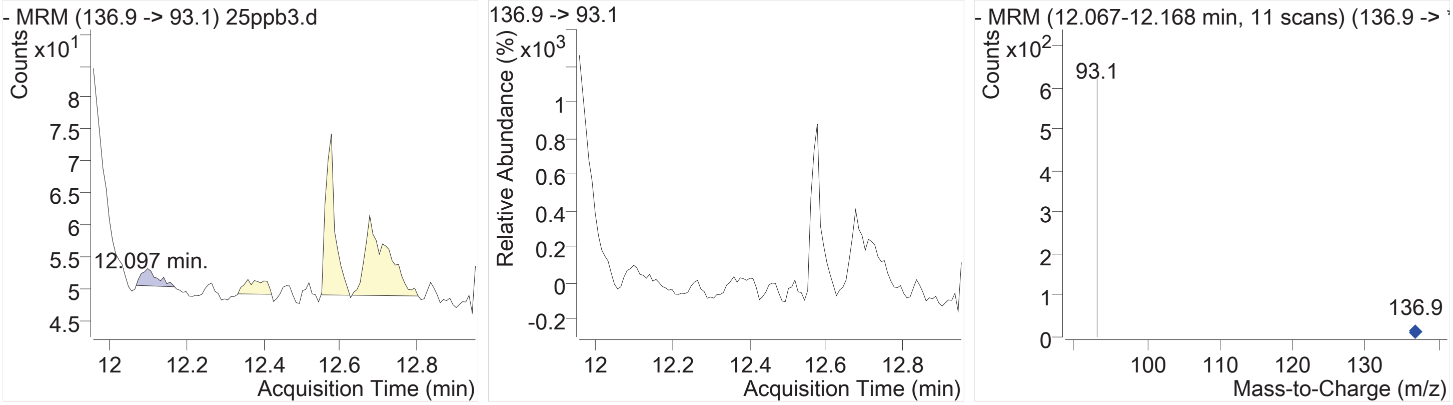

(-)-Epicatechin

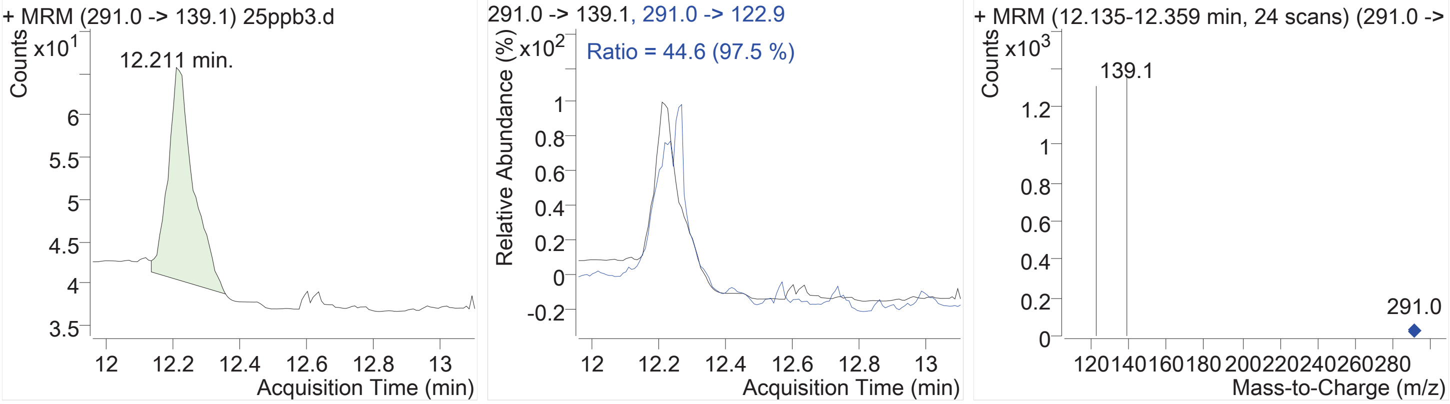

Caffeic acid

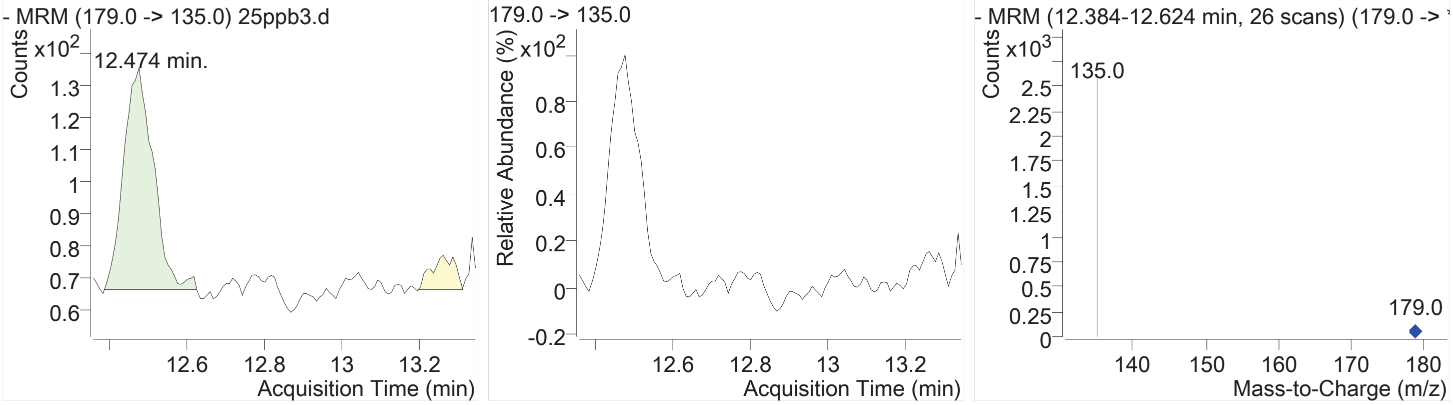

Syringic acid

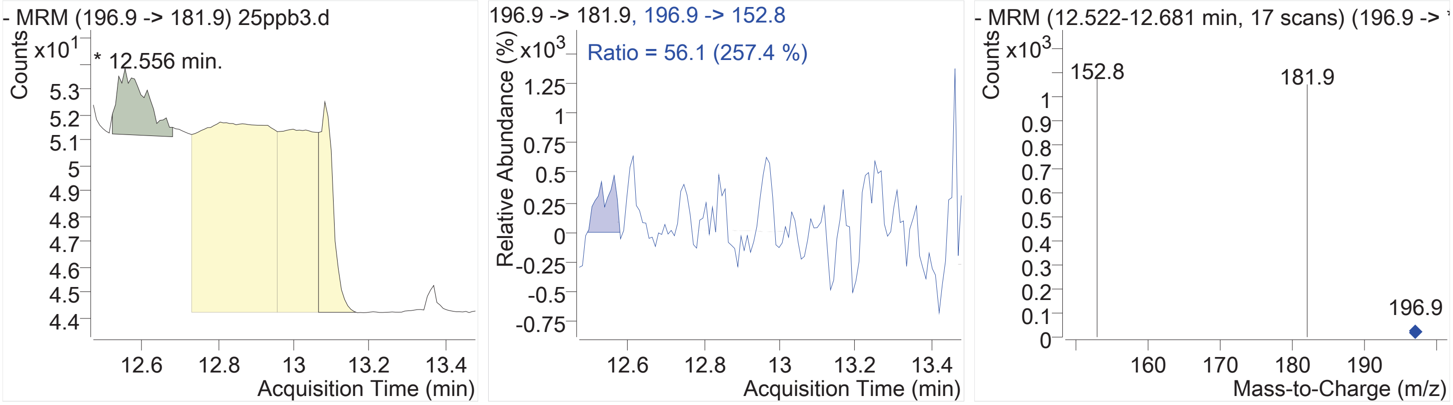

Vanillin

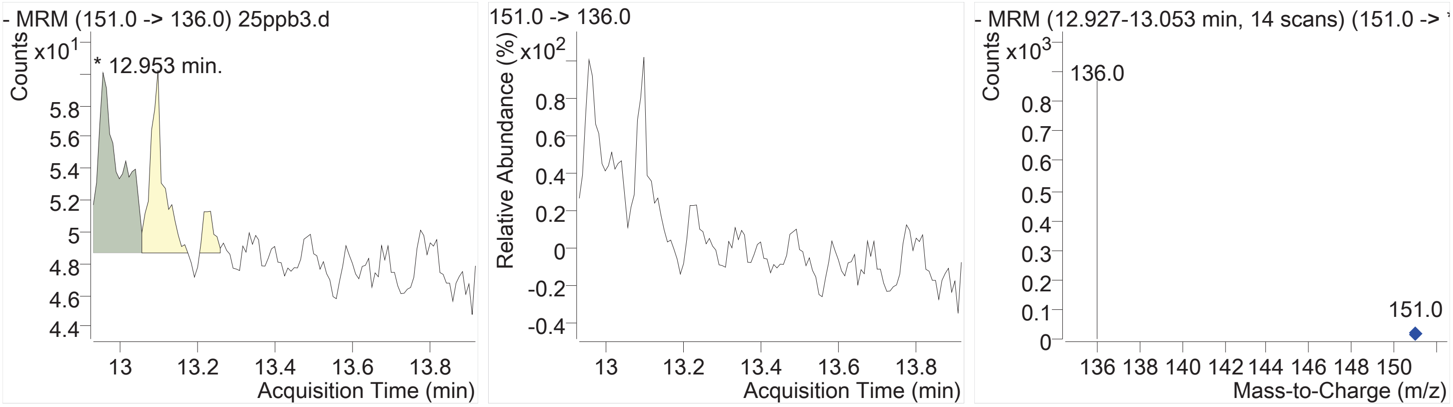

Verbascoside

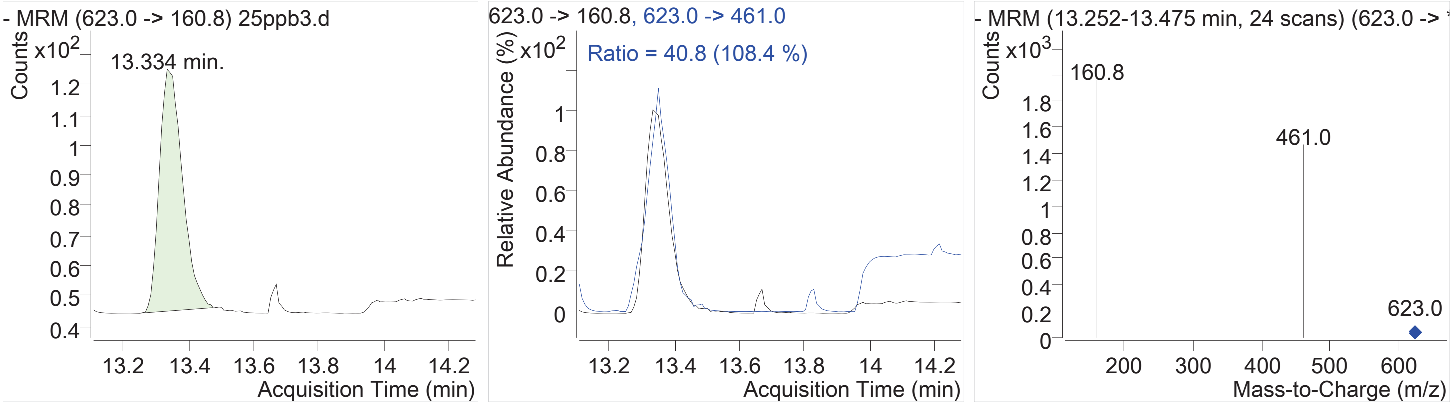

Taxifolin

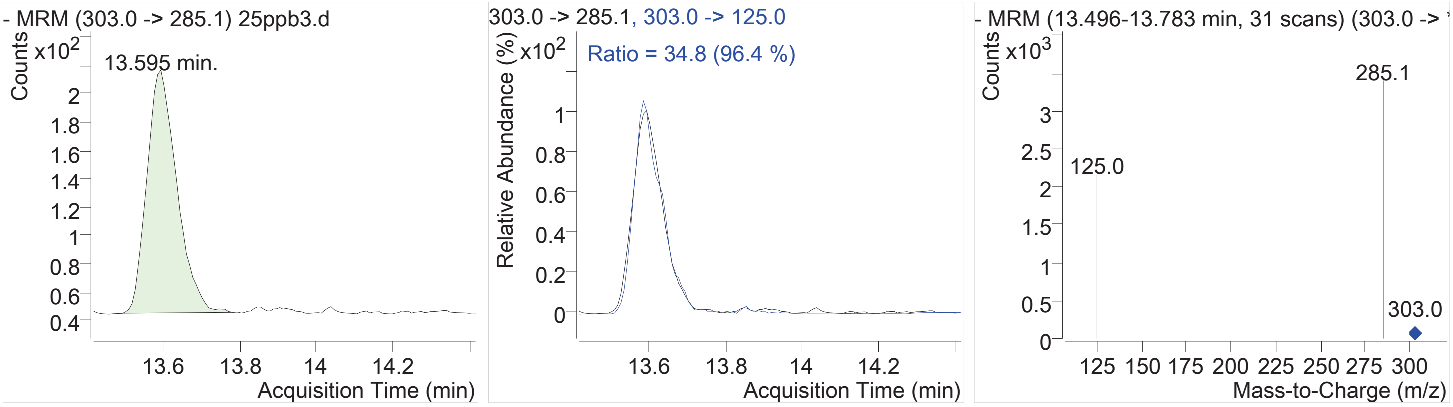

p-Coumaric acid

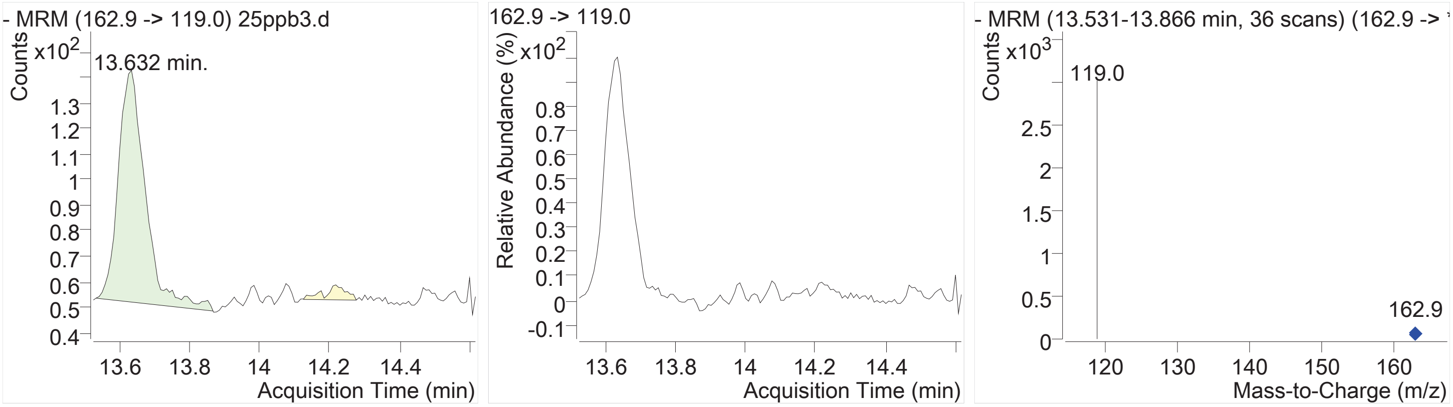

Sinapic acid

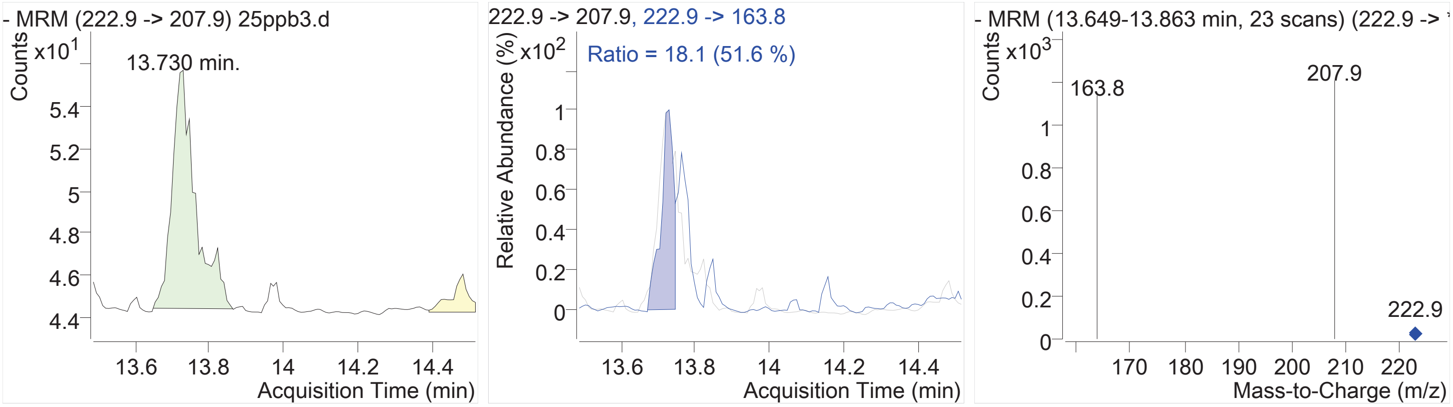

Ferulic acid

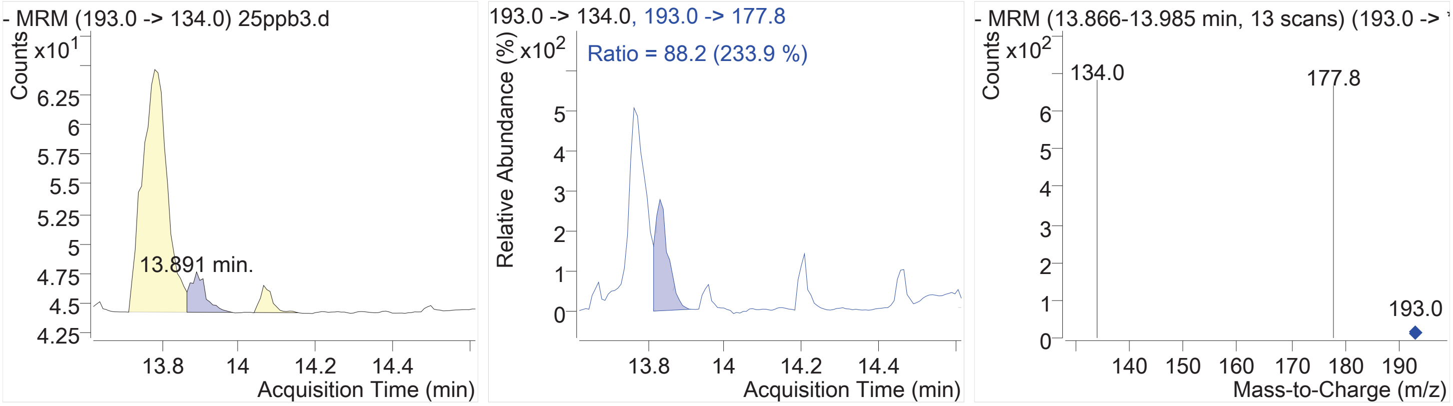

Luteolin 7-glucoside

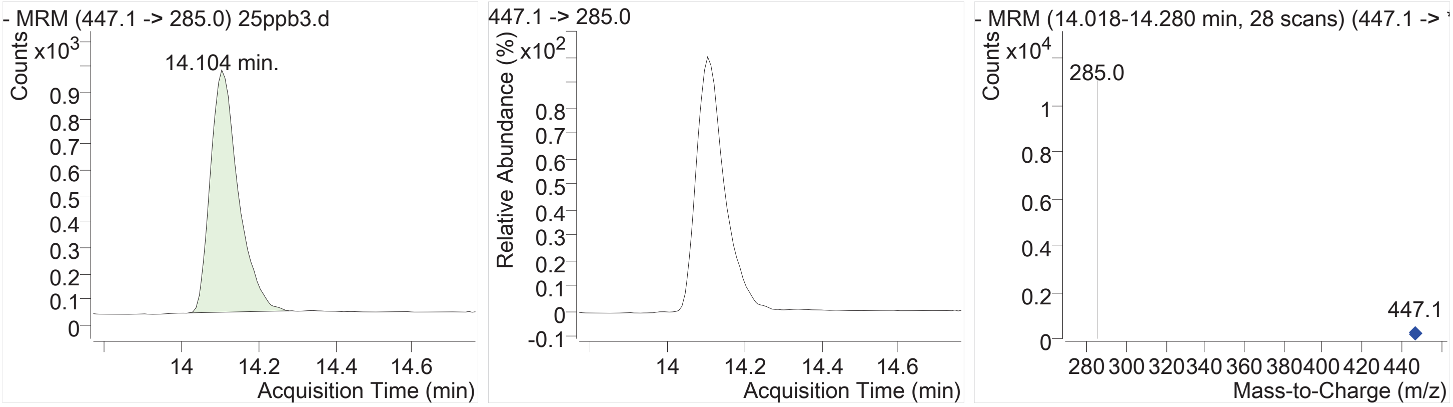

Hesperidin

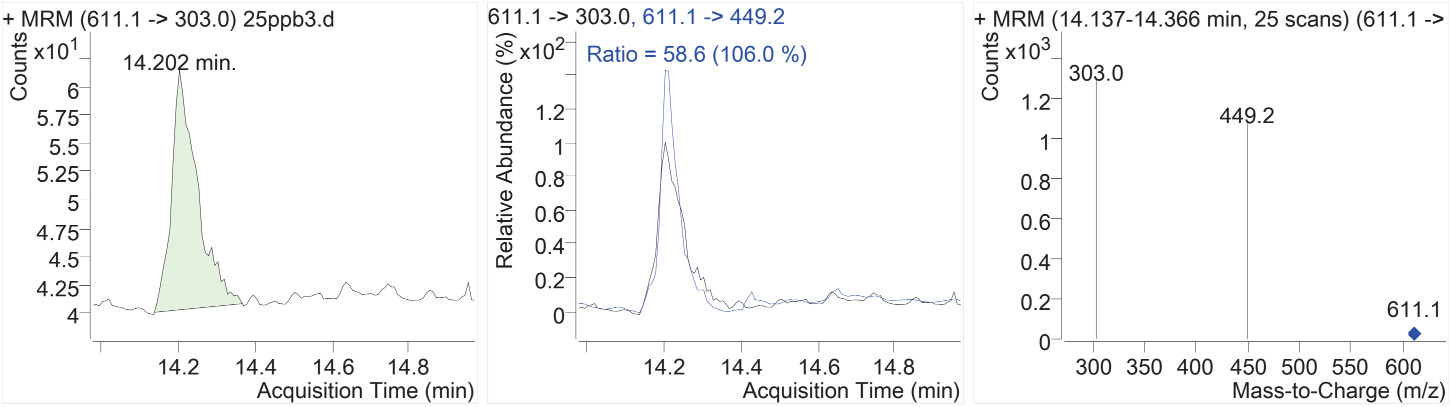

Hyperoside

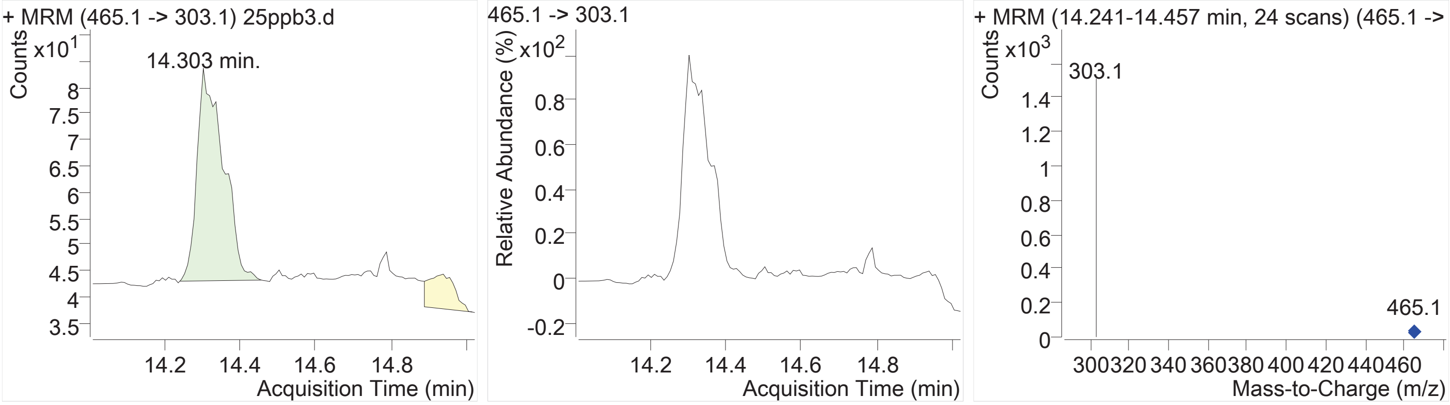

Rosmarinic acid

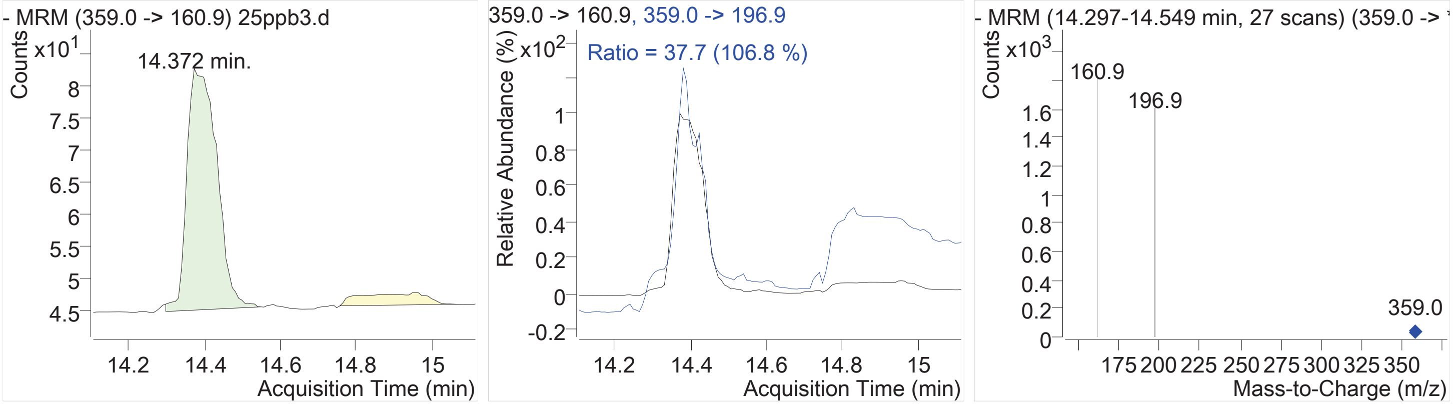

Apigenin 7-glucoside

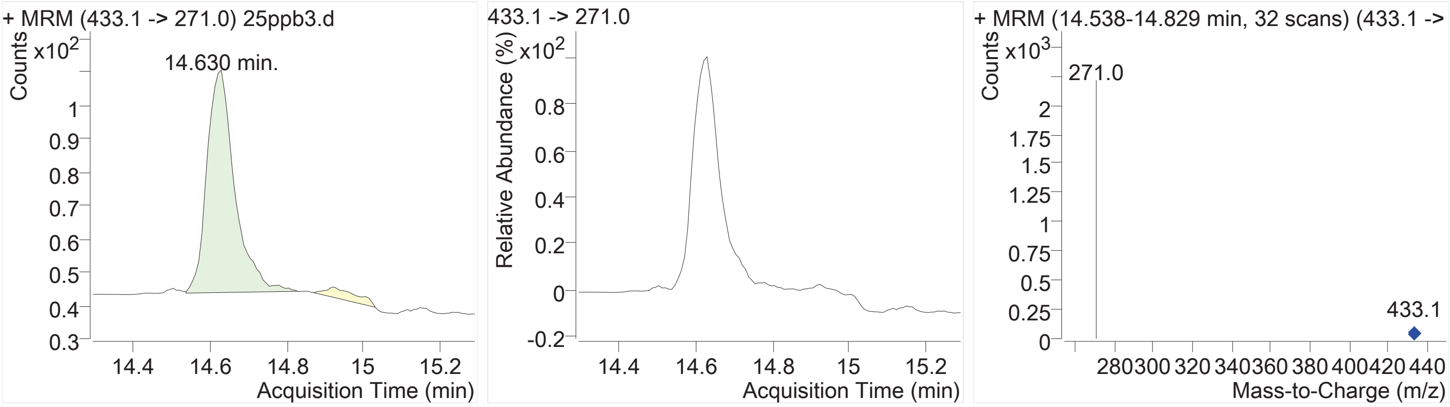

Pinoresinol

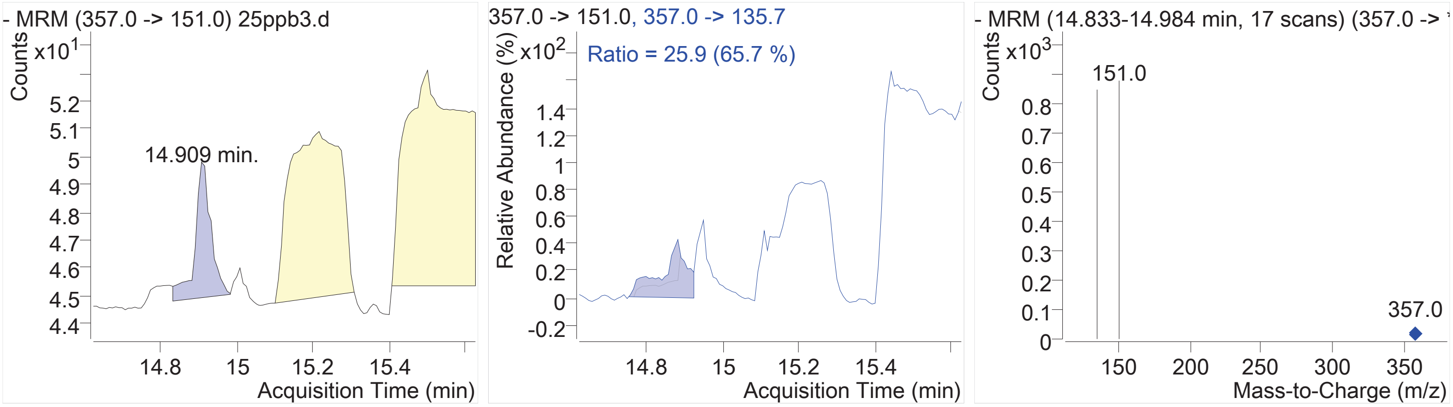

Agilent Technologies

2-Hydroxycinnamic acid

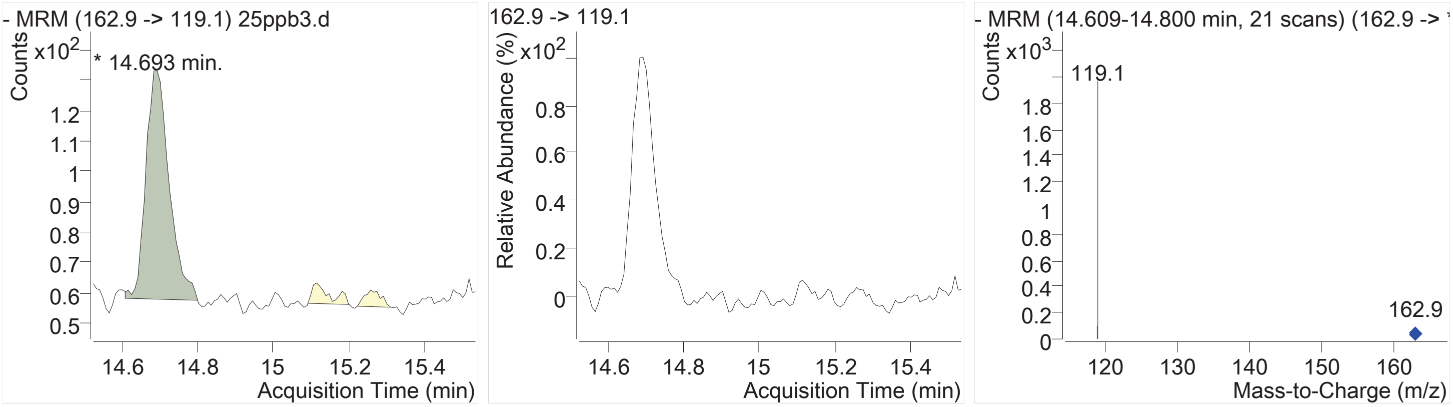

Eriodictyol

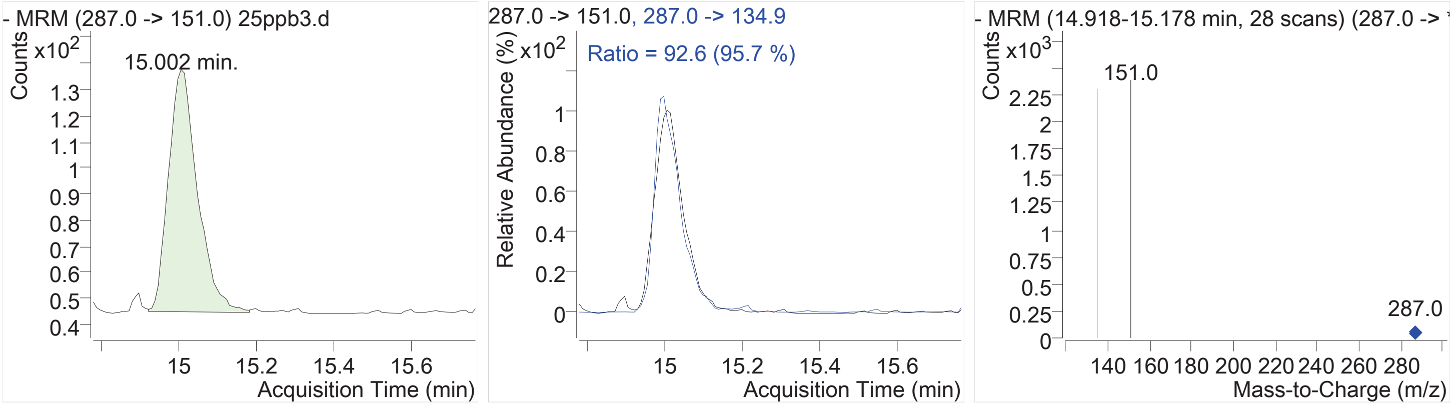

Quercetin

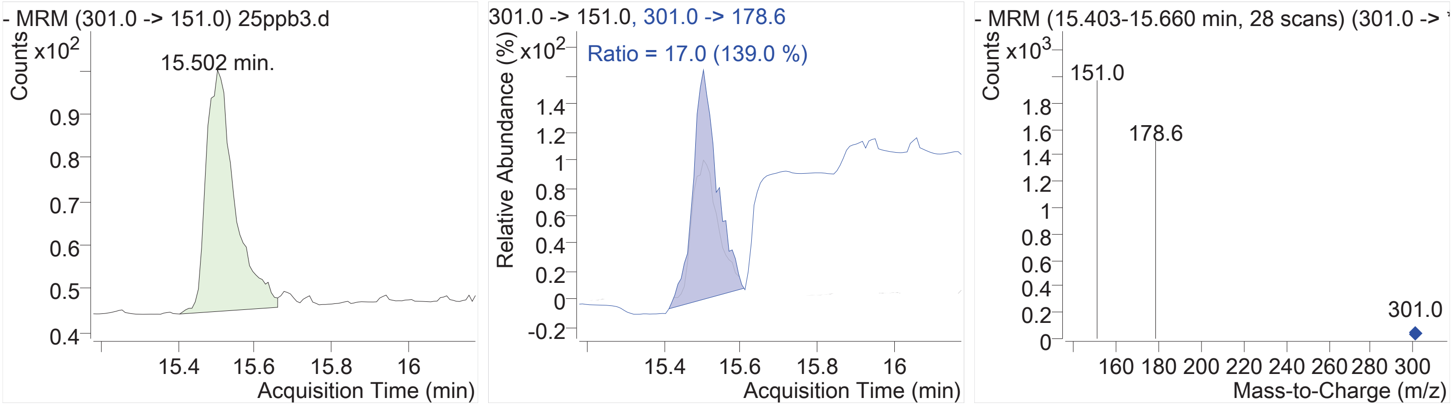

Luteolin

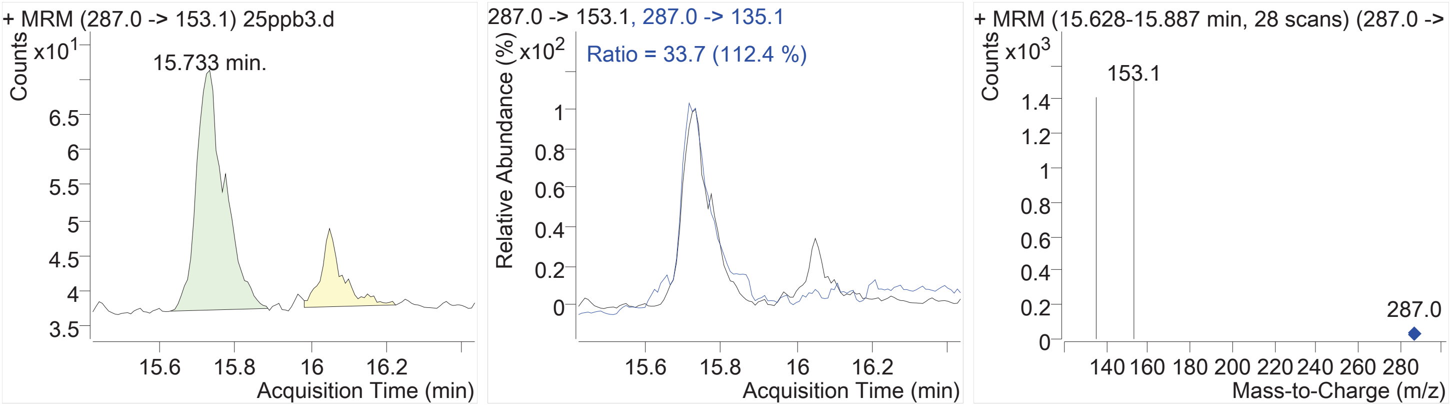

Kaempferol

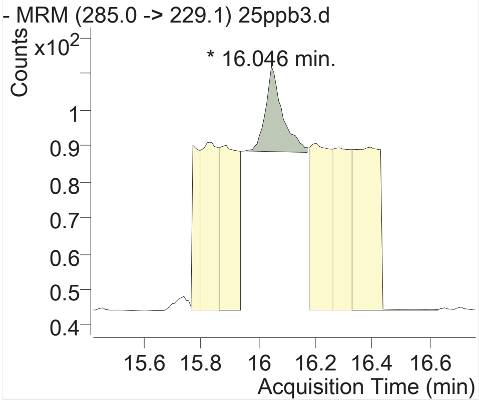

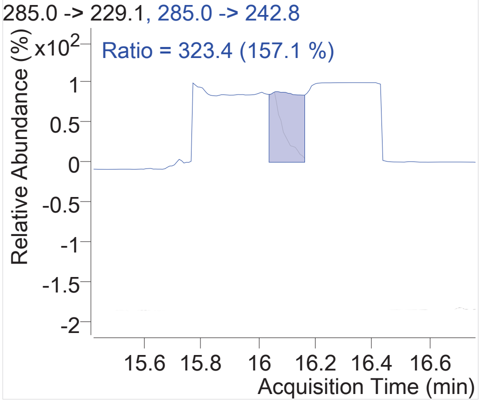

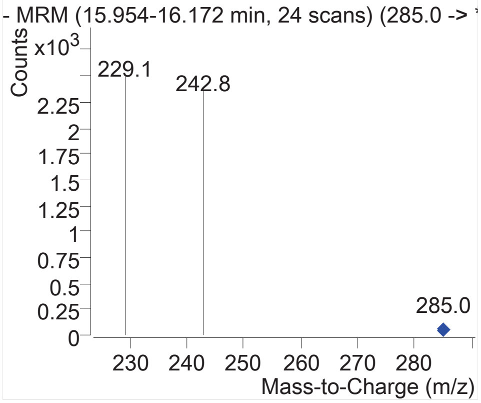

Apigenin

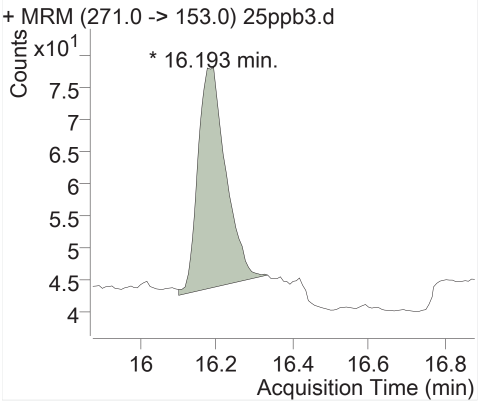

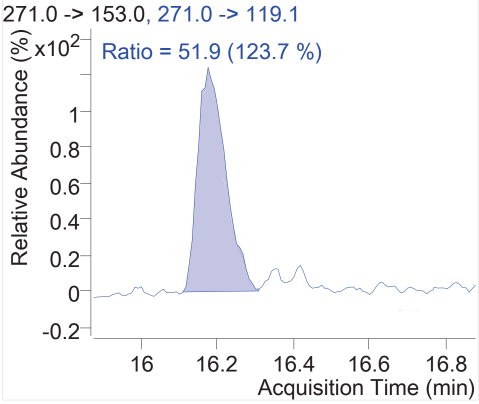

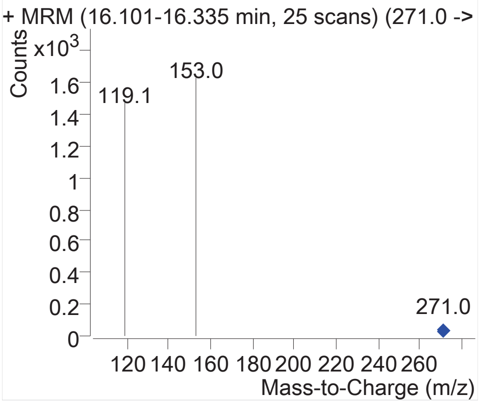

**Batch Path**	D:\MassHunter\Data\2022ekim\061022cengizhoca\QuantResults\071022.batch.bin		
**Analysis Time**	10/11/2022 1:33:26 PM	**Analyst Name**	Defam-PC\admin
**Report Time**	10/11/2022 1:33:59 PM	**Reporter Name**	admin
**Last Calib Update**	10/11/2022 1:33:17 PM	**Batch State**	Processed
**Quant Batch Version**	B.07.01	**Quant Report Version**	B.07.01

**Acq. Time**	10/6/2022 5:00:14 PM	**Data File**	50ppb1.d
**Sample Type**	Cal	**Sample Name**	50ppb1
**Dilution**	1	**Acq. Method**	FENOLIK_DMRM2021-31bilesen.m

Sample Chromatogram

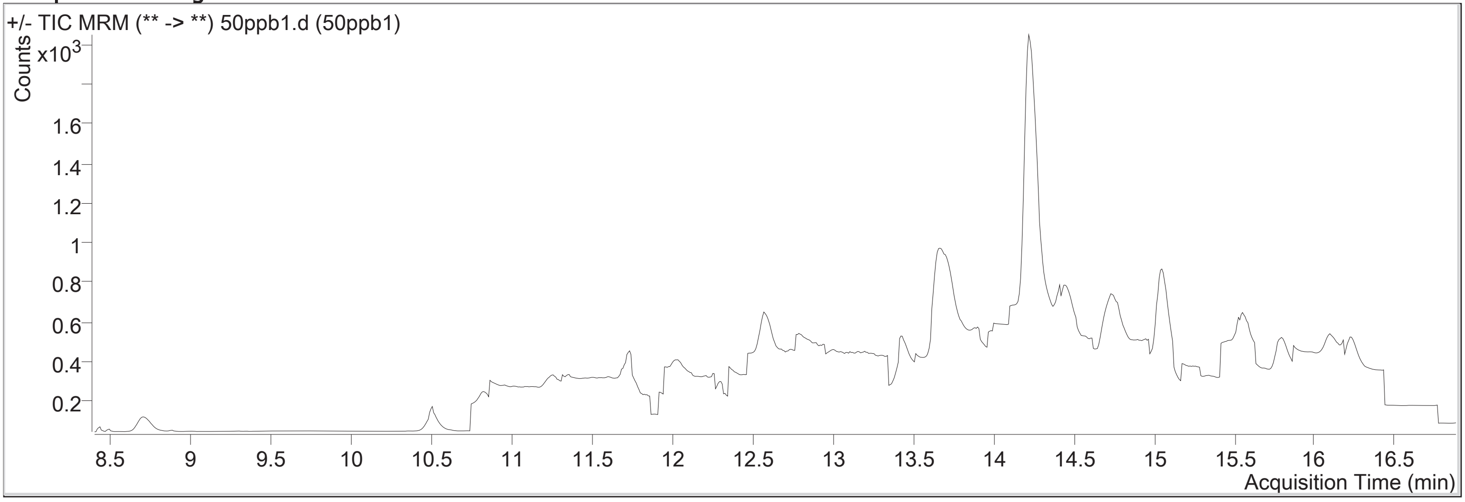

Compound	Transition	RT	Resp.	Final Conc	Units
Gallic acid	168.9 -> 125.0	8.708	436	18.8746	ng/ml
Protocatechuic acid	152.9 -> 108.9	10.509	533	39.8123	ng/ml
Pyrocatechol	109.0 -> 52.9	10.814	5	ND	ng/ml
3,4-Dihydroxyphenylacetic acid	167.0 -> 123.0	10.826	331	32.9234	ng/ml
(+)-Catechin	289.0 -> 245.0	11.261	192	28.8208	ng/ml
2,5-Dihydroxybenzoic acid	152.9 -> 109.0	11.921	216	34.6840	ng/ml
Chlorogenic acid	355.0 -> 163.0	11.735	674	37.4146	ng/ml
3-Hydroxybenzoic acid	137.0 -> 93.0	12.794	144	41.9387	ng/ml
4-Hydroxybenzoic acid	136.9 -> 93.1	12.039	495	29.0138	ng/ml
(-)-Epicatechin	291.0 -> 139.1	12.302	225	31.4174	ng/ml
Caffeic acid	179.0 -> 135.0	12.574	1120	19.3875	ng/ml
Syringic acid	196.9 -> 181.9	12.715	32	47.7785	ng/ml
Vanillin	151.0 -> 136.0	13.012	175	31.3814	ng/ml
Verbascoside	623.0 -> 160.8	13.434	585	45.0338	ng/ml
Taxifolin	303.0 -> 285.1	13.661	1923	29.9648	ng/ml
p-Coumaric acid	162.9 -> 119.0	13.741	1106	33.7852	ng/ml
Sinapic acid	222.9 -> 207.9	13.806	120	42.2062	ng/ml
Ferulic acid	193.0 -> 134.0	13.891	222	42.7595	ng/ml
Luteolin 7-glucoside	447.1 -> 285.0	14.222	7068	44.0082	ng/ml
Hesperidin	611.1 -> 303.0	14.286	308	41.6171	ng/ml
Hyperoside	465.1 -> 303.1	14.445	655	37.0741	ng/ml
Rosmarinic acid	359.0 -> 160.9	14.464	517	40.0507	ng/ml
Apigenin 7-glucoside	433.1 -> 271.0	14.713	990	28.1372	ng/ml
Pinoresinol	357.0 -> 151.0	14.976	29	86.4868	ng/ml
2-Hydroxycinnamic acid	162.9 -> 119.1	14.760	879	39.6209	ng/ml
Eriodictyol	287.0 -> 151.0	15.053	1372	35.1731	ng/ml
Quercetin	301.0 -> 151.0	15.553	954	34.5532	ng/ml
Luteolin	287.0 -> 153.1	15.800	512	27.3825	ng/ml
Kaempferol	285.0 -> 229.1	16.088	224	22.6002	ng/ml

Quantitative Analysis Complete Report

Agilent Technologies

Compound	Transition	RT	Resp.	Final Conc	Units
Apigenin	271.0 -> 153.0	16.226	644	28.9204	ng/ml

Gallic acid

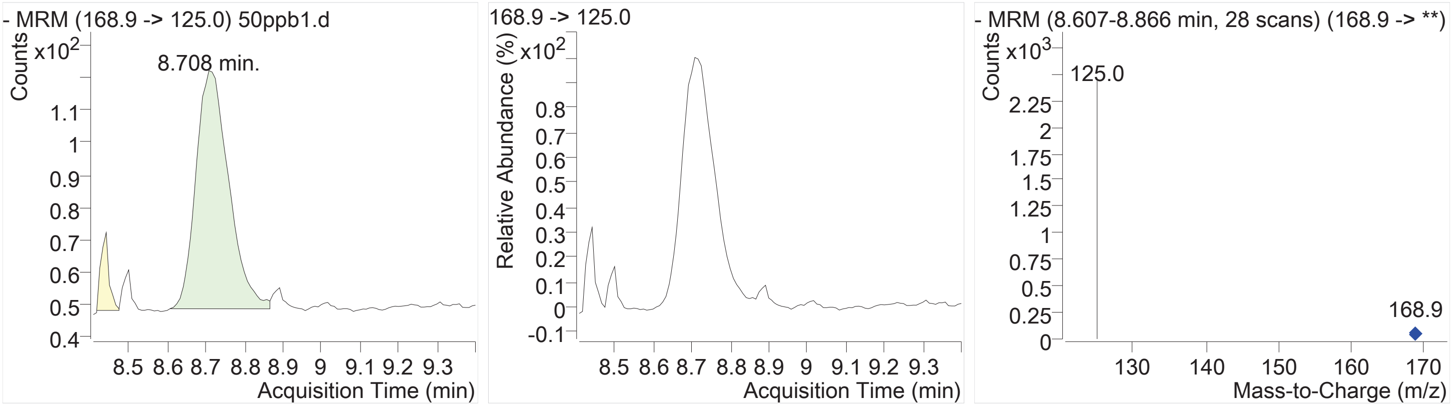

Protocatechuic acid

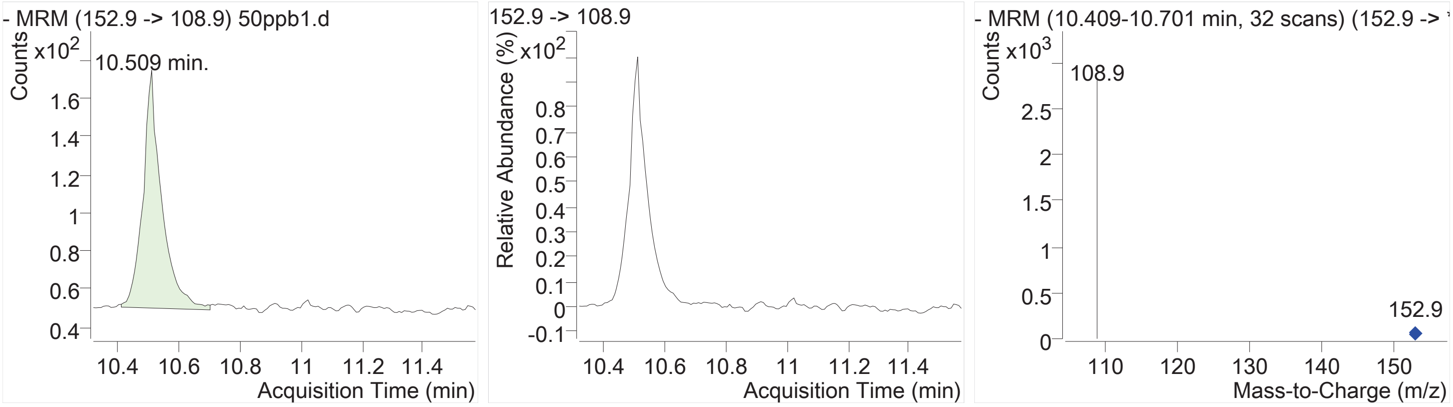

Pyrocatechol

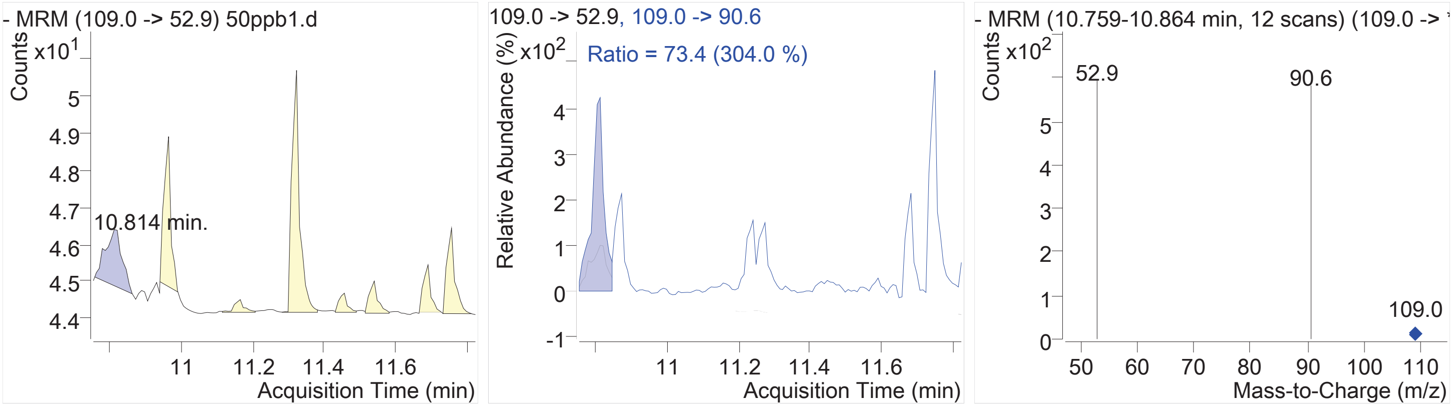

3,4-Dihydroxyphenylacetic acid

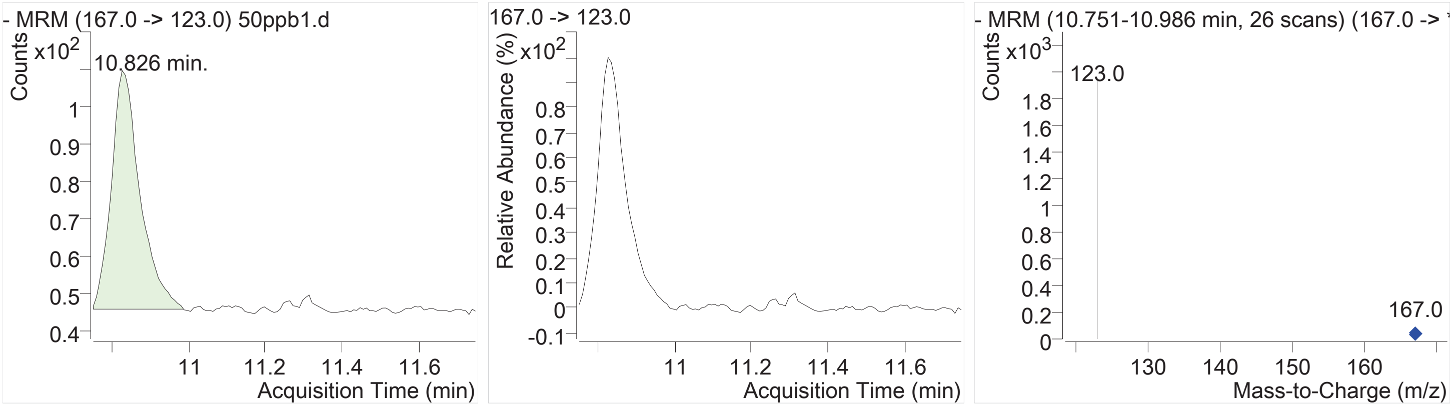

Generated at 1:33 PM on 10/11/2022

(+)-Catechin

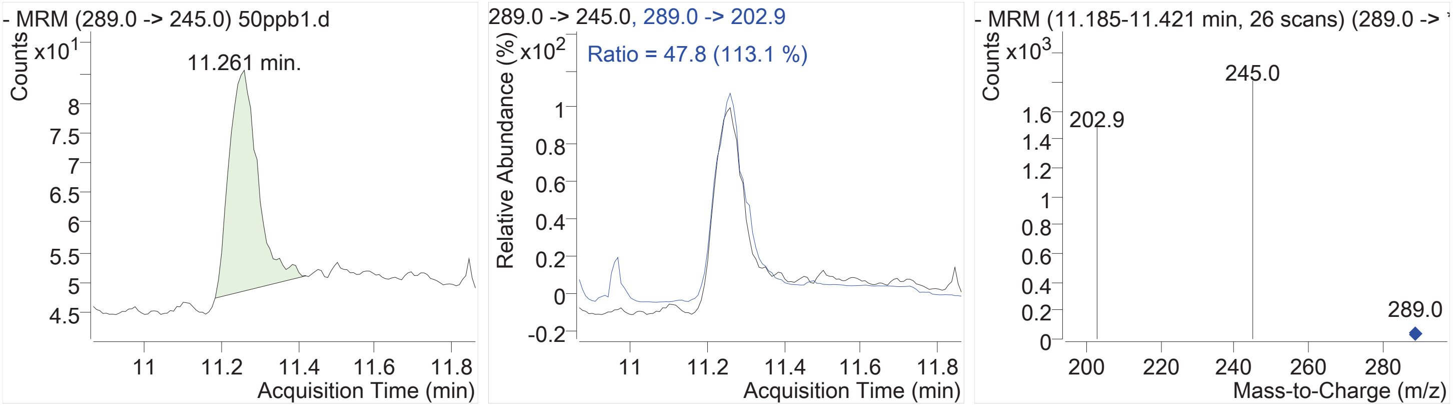

2,5-Dihydroxybenzoic acid

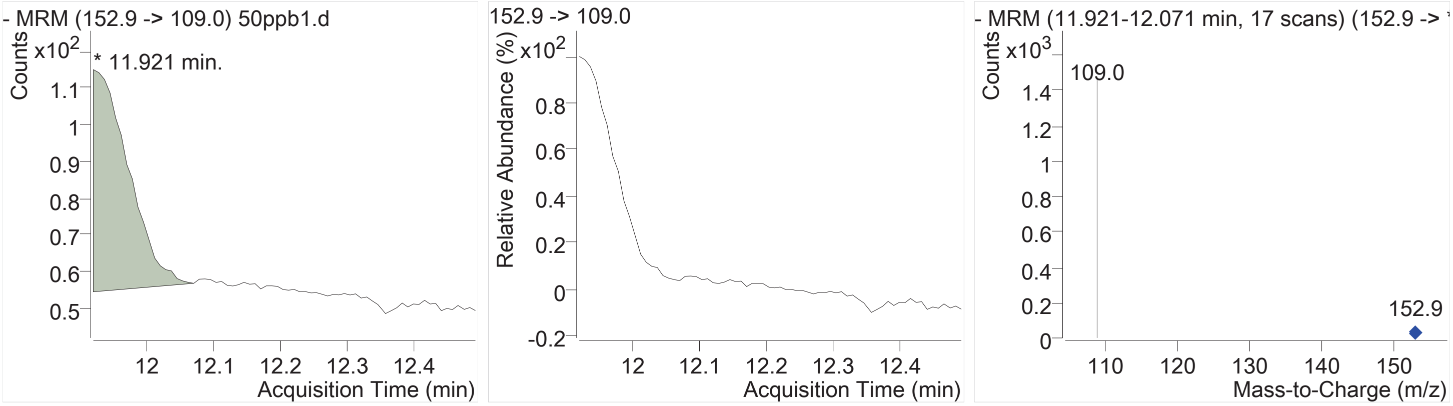

Chlorogenic acid

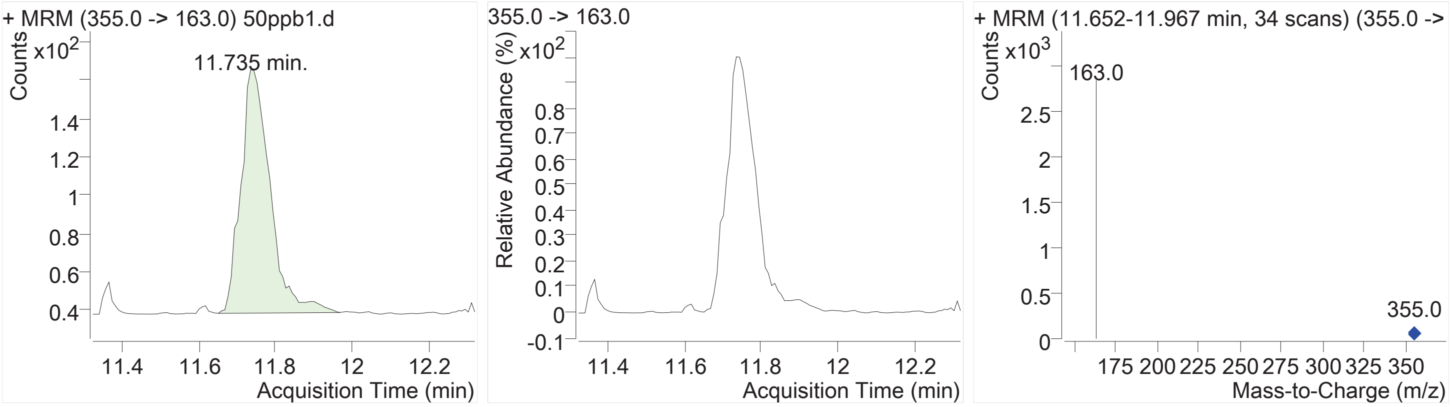

3-Hydroxybenzoic acid

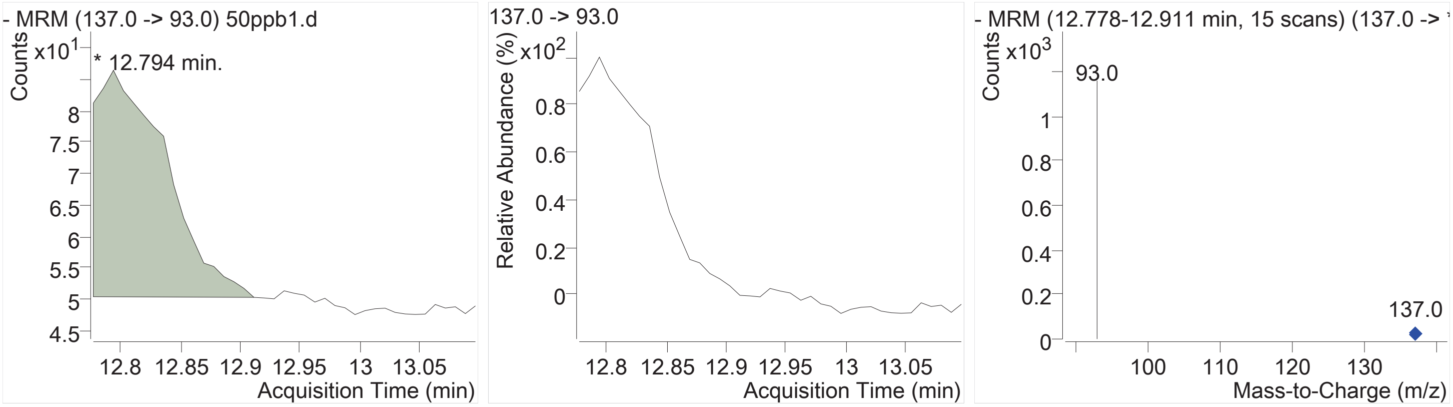

4-Hydroxybenzoic acid

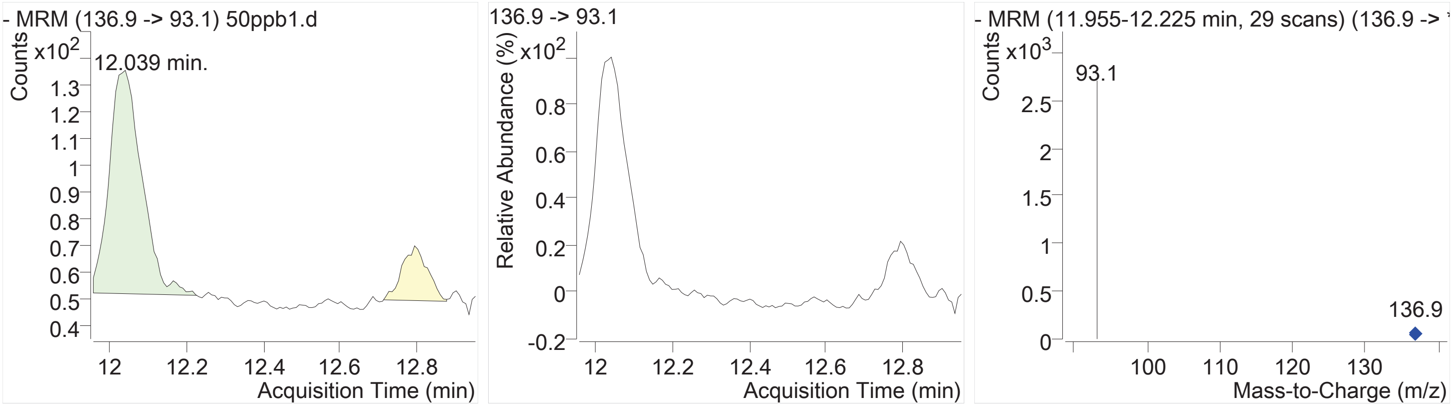

(-)-Epicatechin

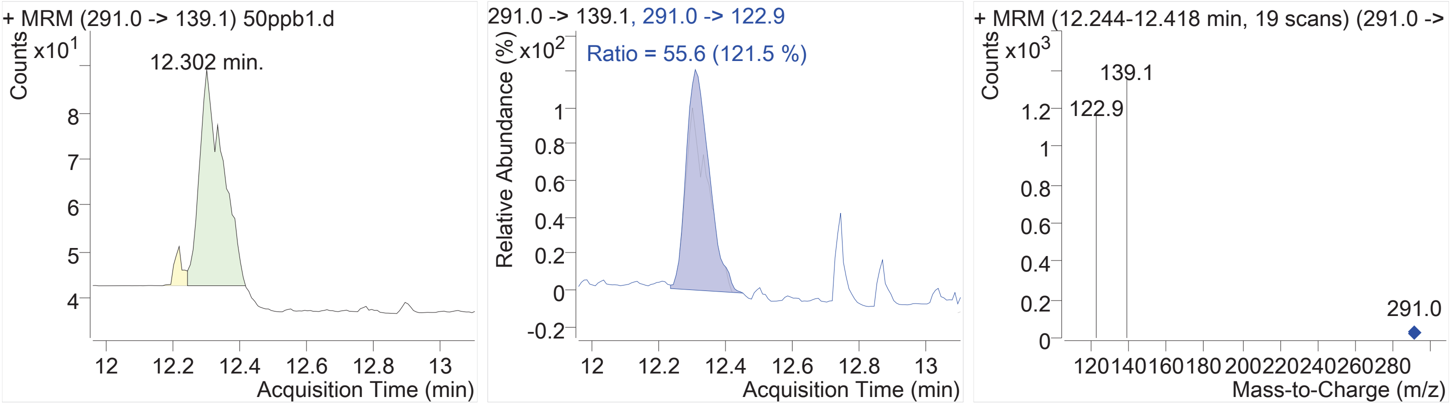

Caffeic acid

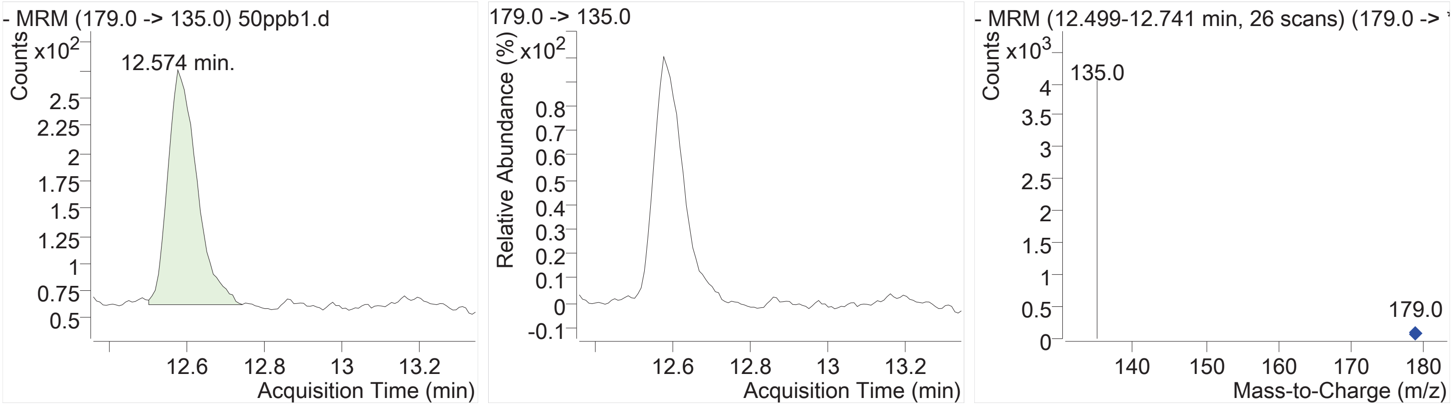

Syringic acid

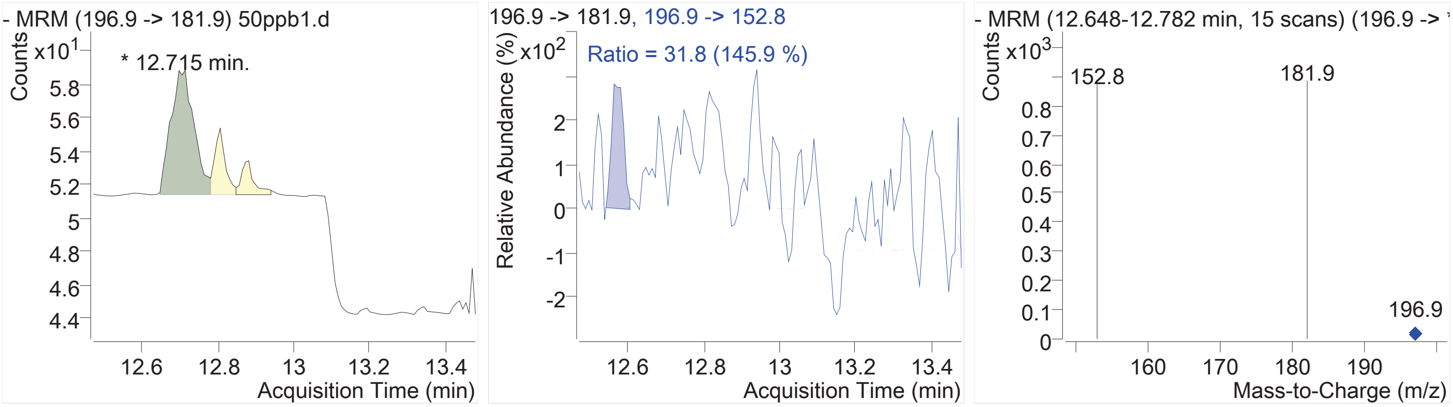

Vanillin

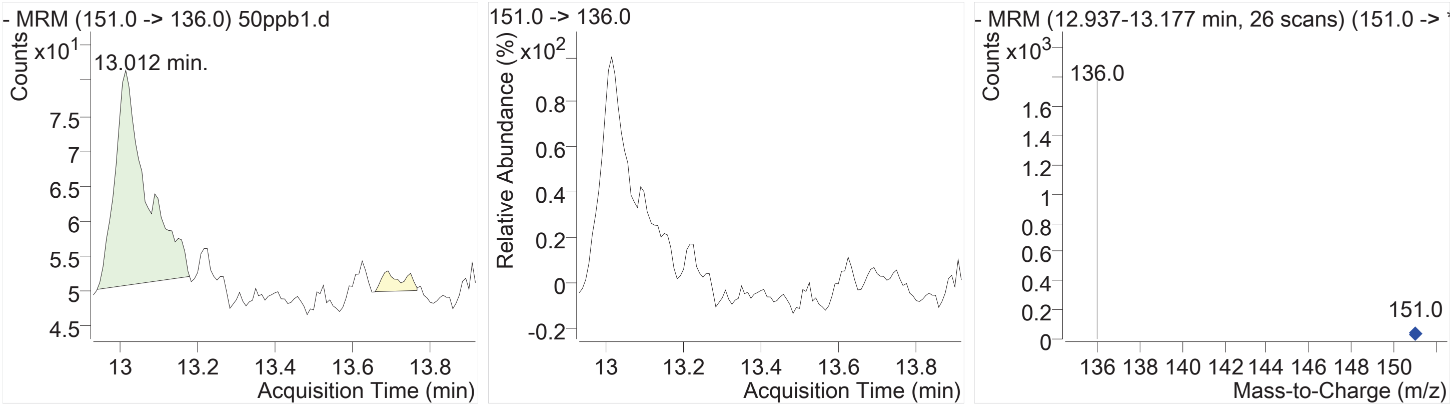

Verbascoside

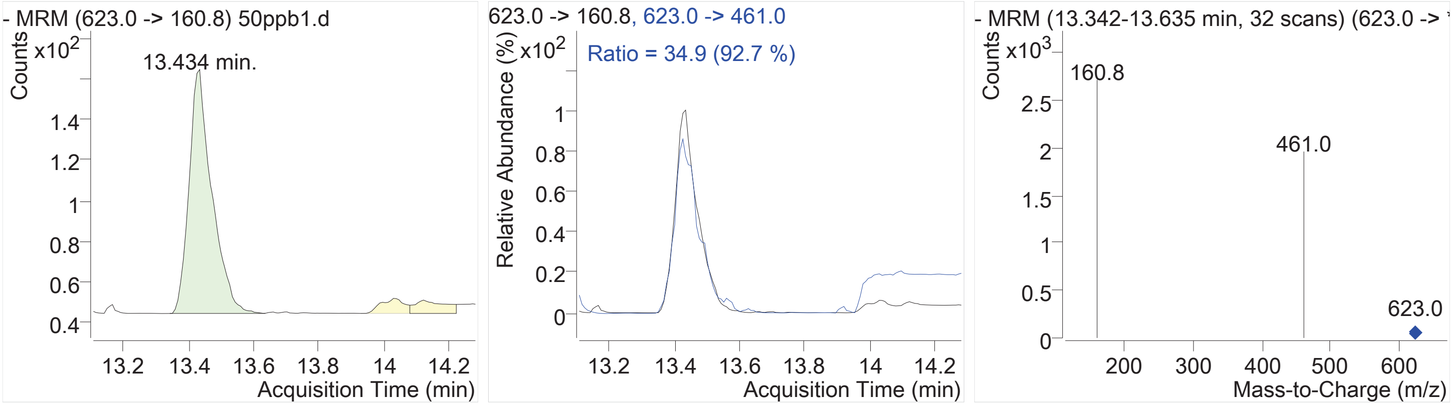

Taxifolin

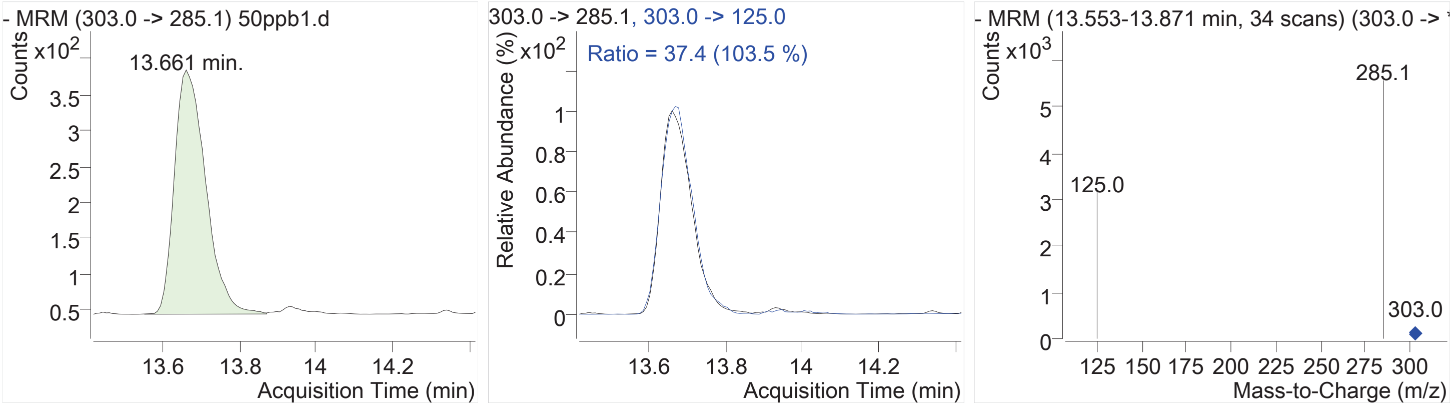

p-Coumaric acid

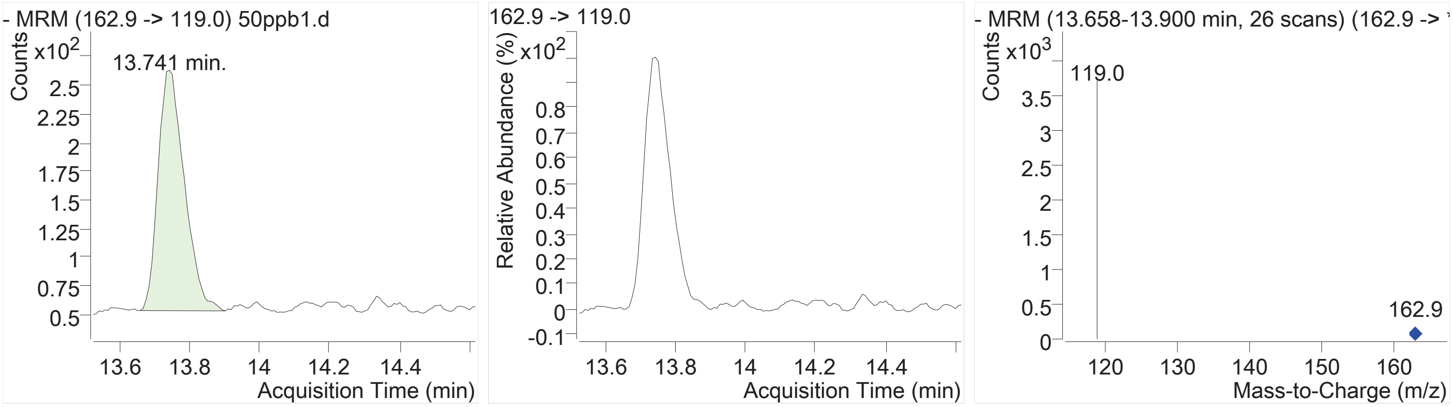

Sinapic acid

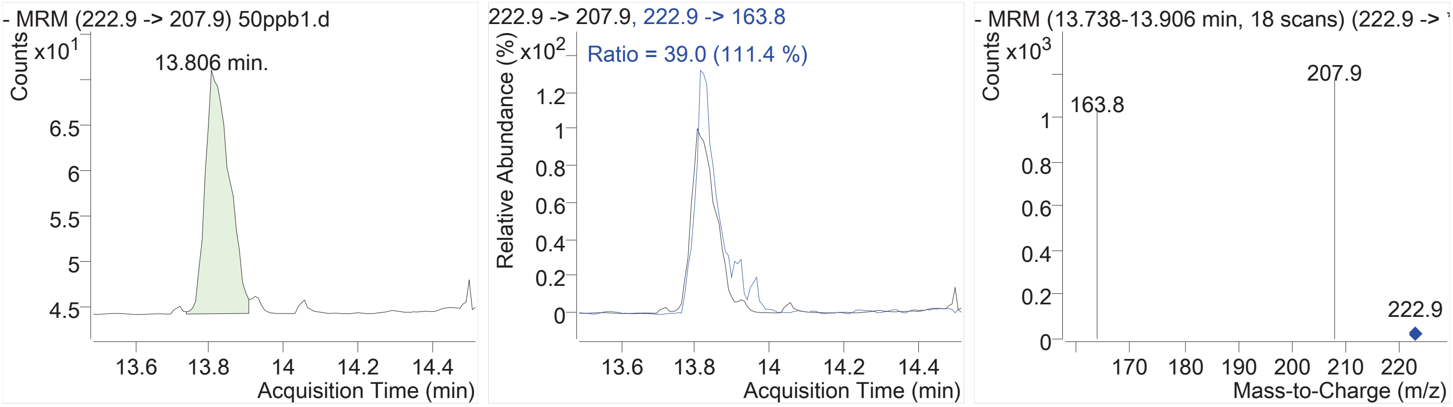

Ferulic acid

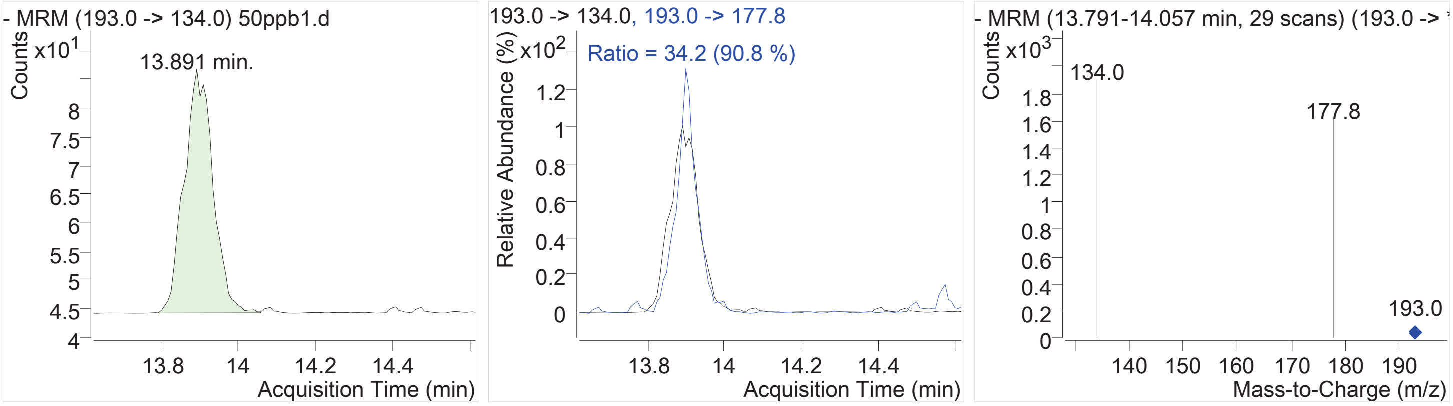

Luteolin 7-glucoside

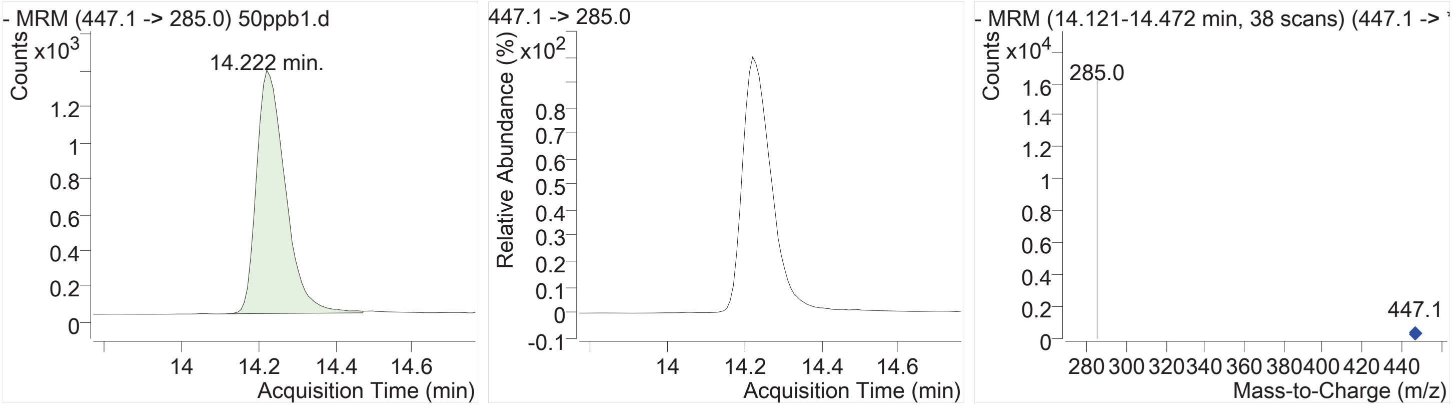

Hesperidin

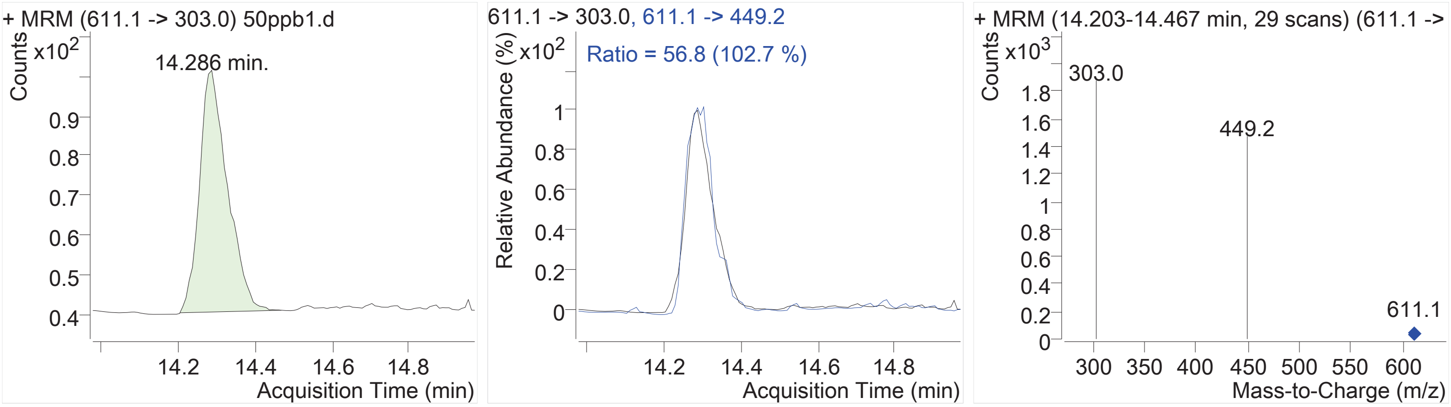

Agilent Technologies

Hyperoside

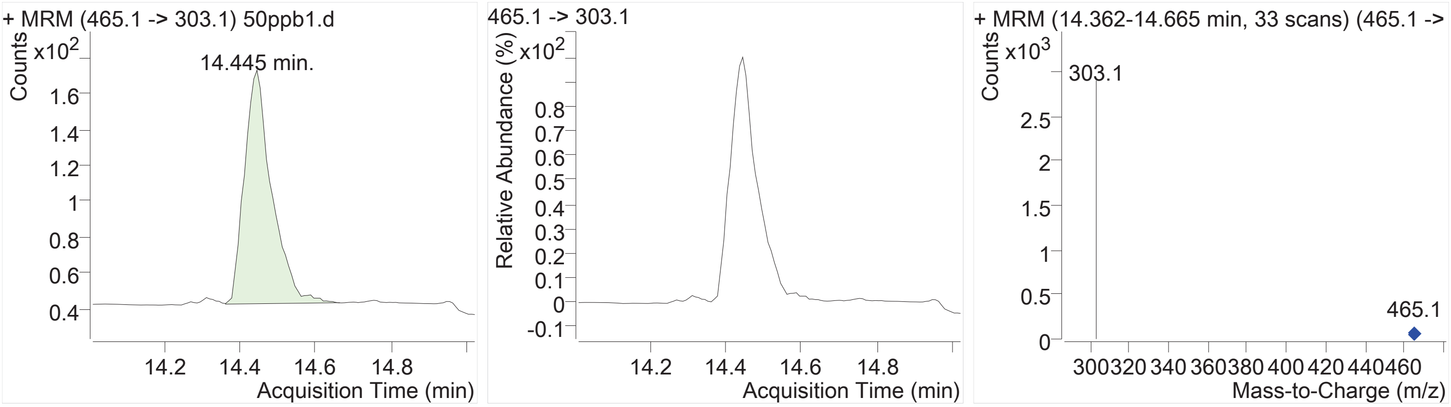

Rosmarinic acid

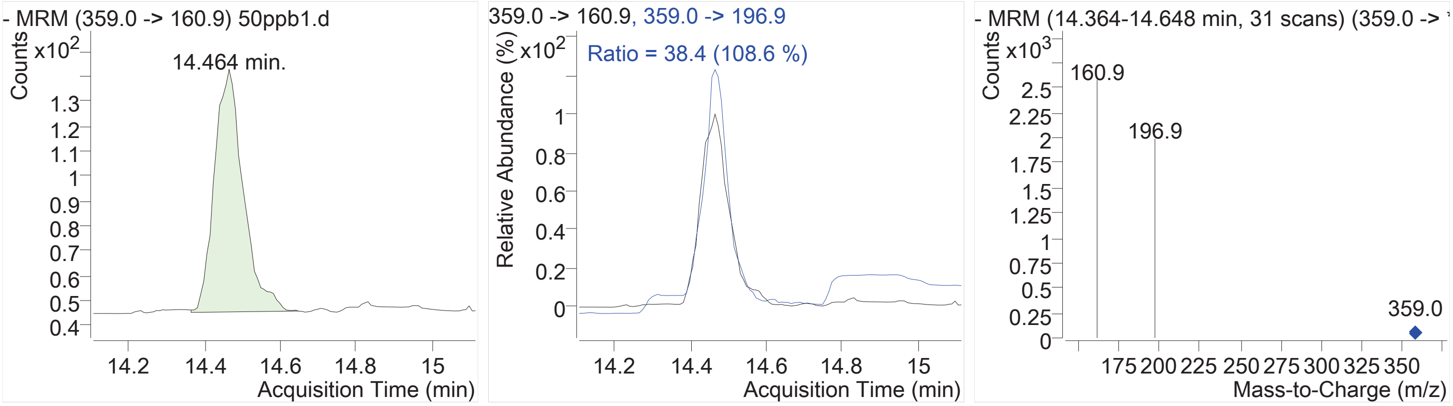

Apigenin 7-glucoside

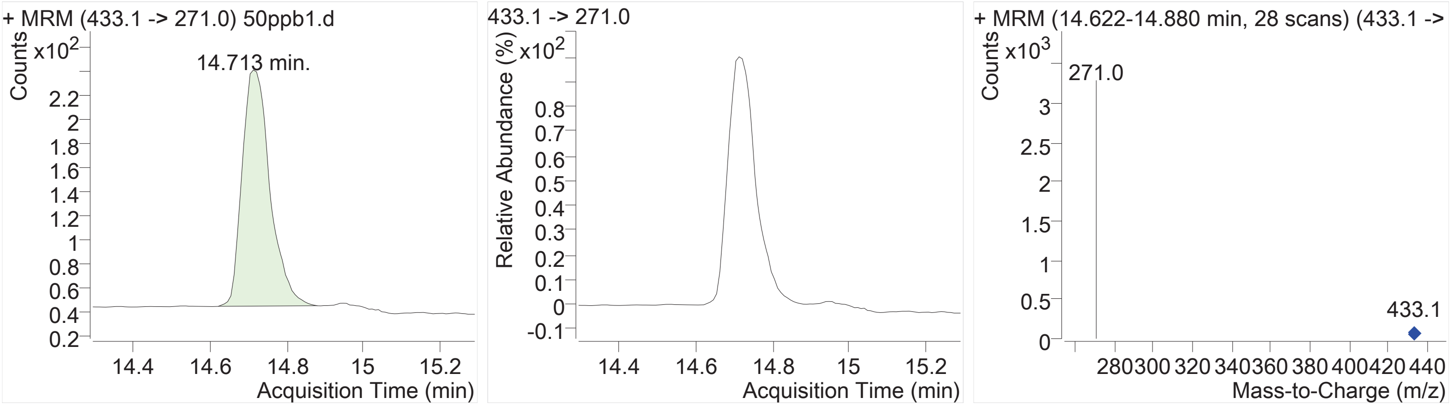

Pinoresinol

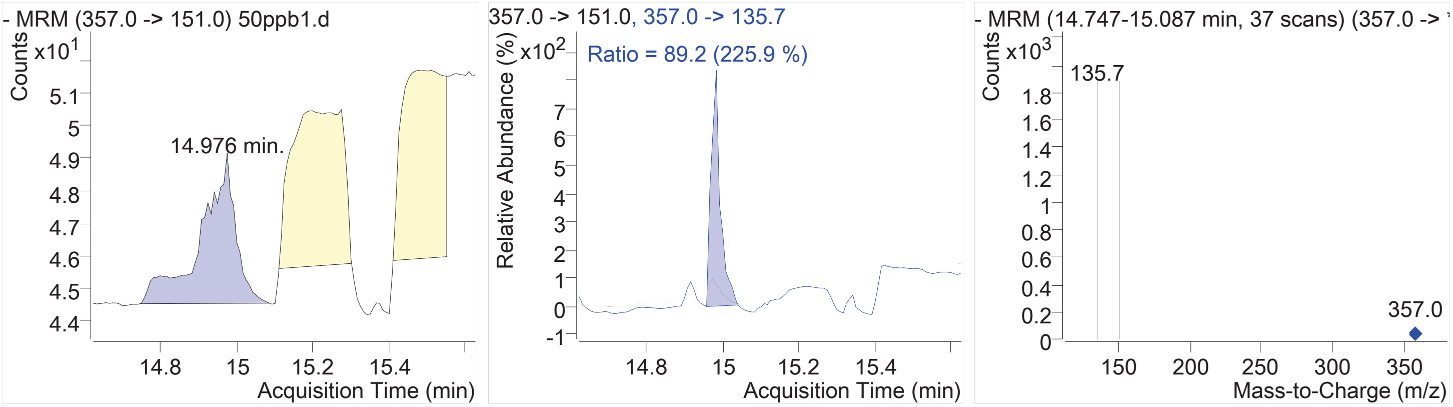

2-Hydroxycinnamic acid

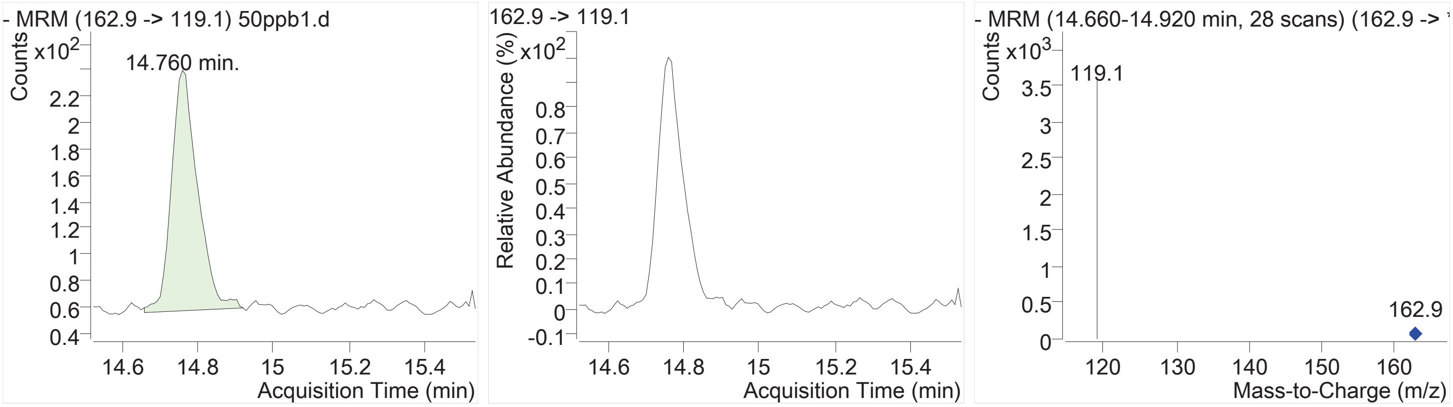

Eriodictyol

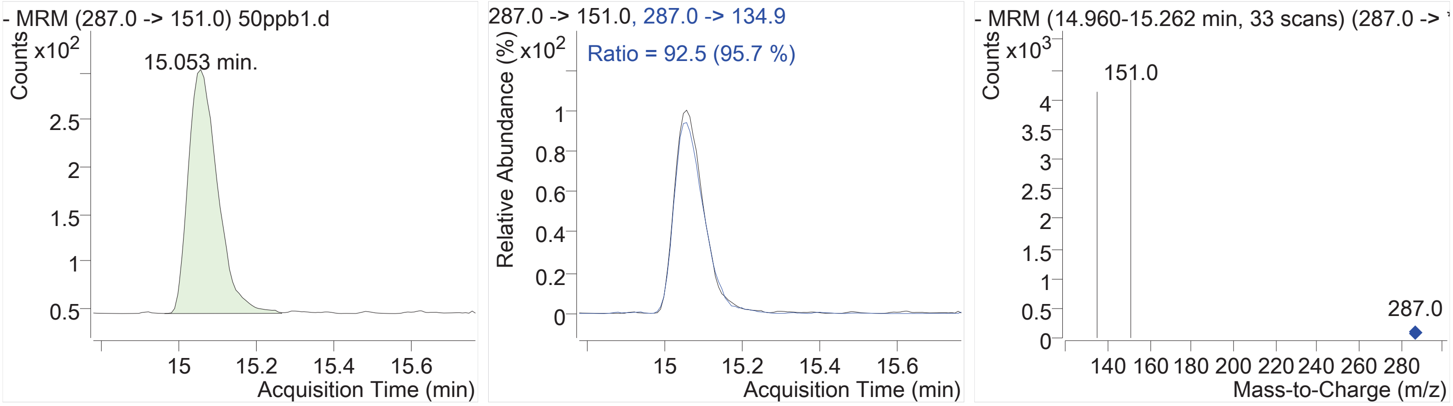

Quercetin

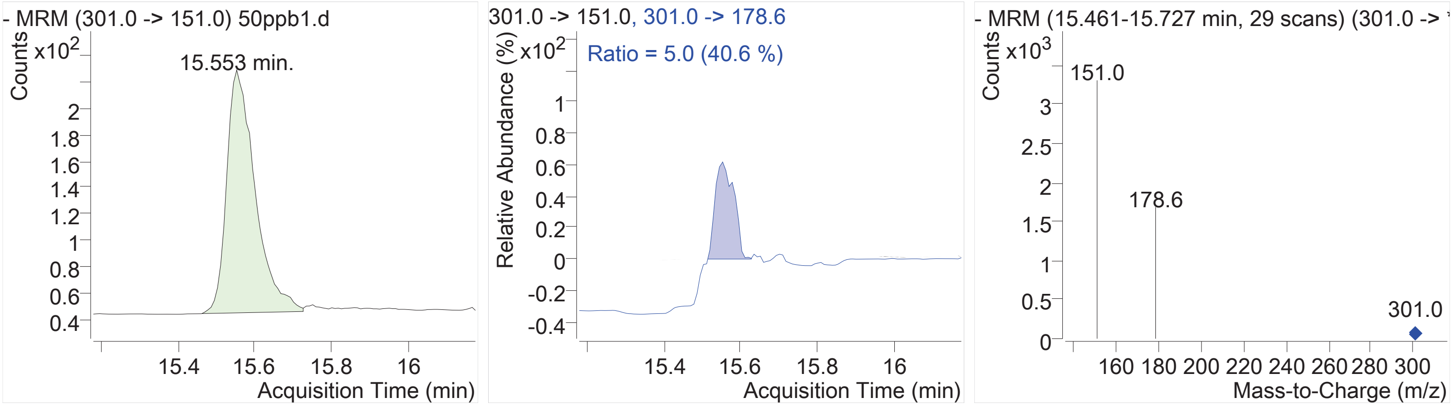

Luteolin

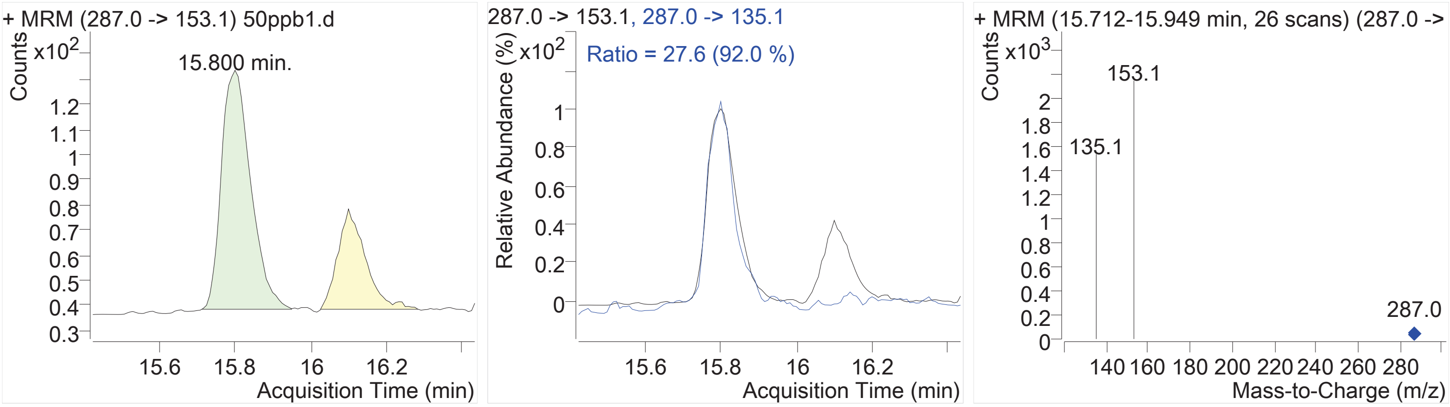

Kaempferol

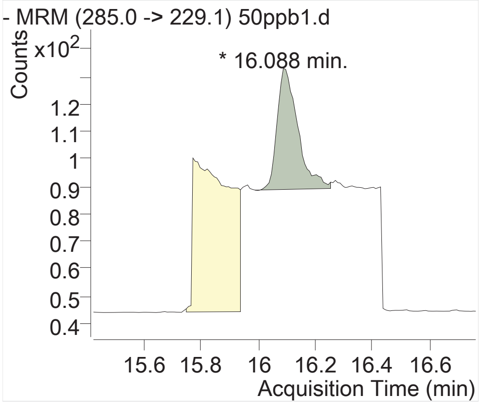

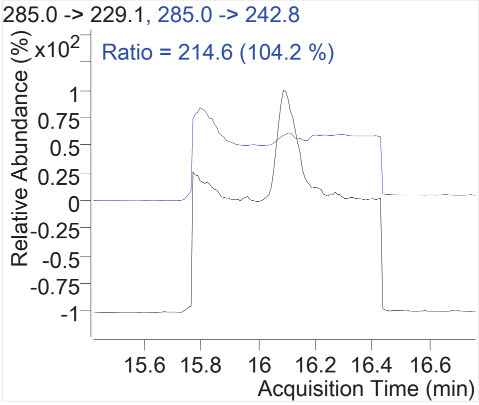

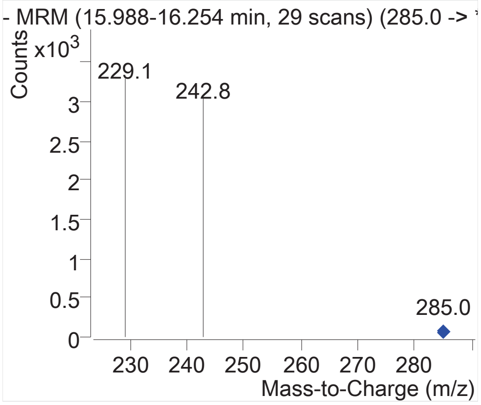

Apigenin

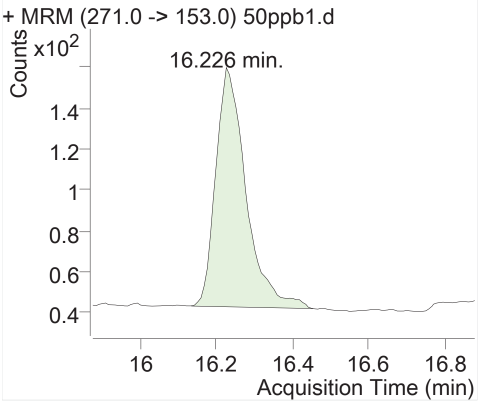

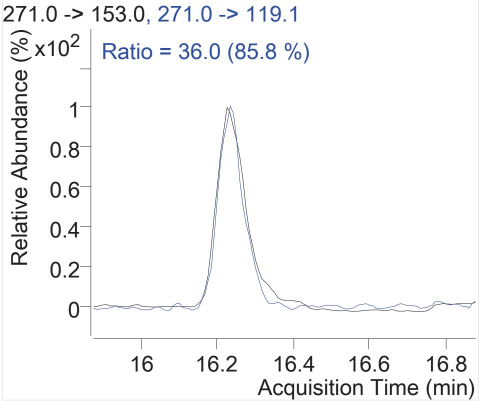

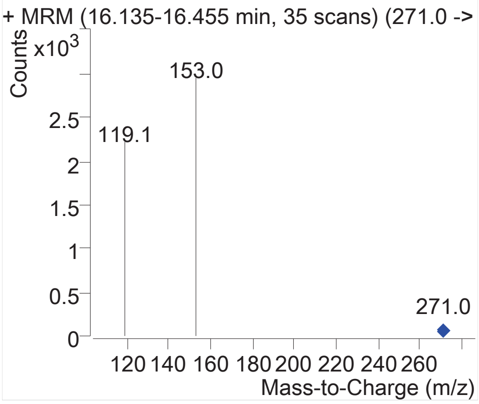

Quantitative Analysis Complete Report

**Batch Path**	D:\MassHunter\Data\2022ekim\061022cengizhoca\QuantResults\071022.batch.bin		
**Analysis Time**	10/11/2022 1:33:26 PM	**Analyst Name**	Defam-PC\admin
**Report Time**	10/11/2022 1:34:02 PM	**Reporter Name**	admin
**Last Calib Update**	10/11/2022 1:33:17 PM	**Batch State**	Processed
**Quant Batch Version**	B.07.01	**Quant Report Version**	B.07.01

**Acq. Time**	10/6/2022 5:21:54 PM	**Data File**	50ppb2.d
**Sample Type**	Cal	**Sample Name**	50ppb2
**Dilution**	1	**Acq. Method**	FENOLIK_DMRM2021-31bilesen.m

Sample Chromatogram

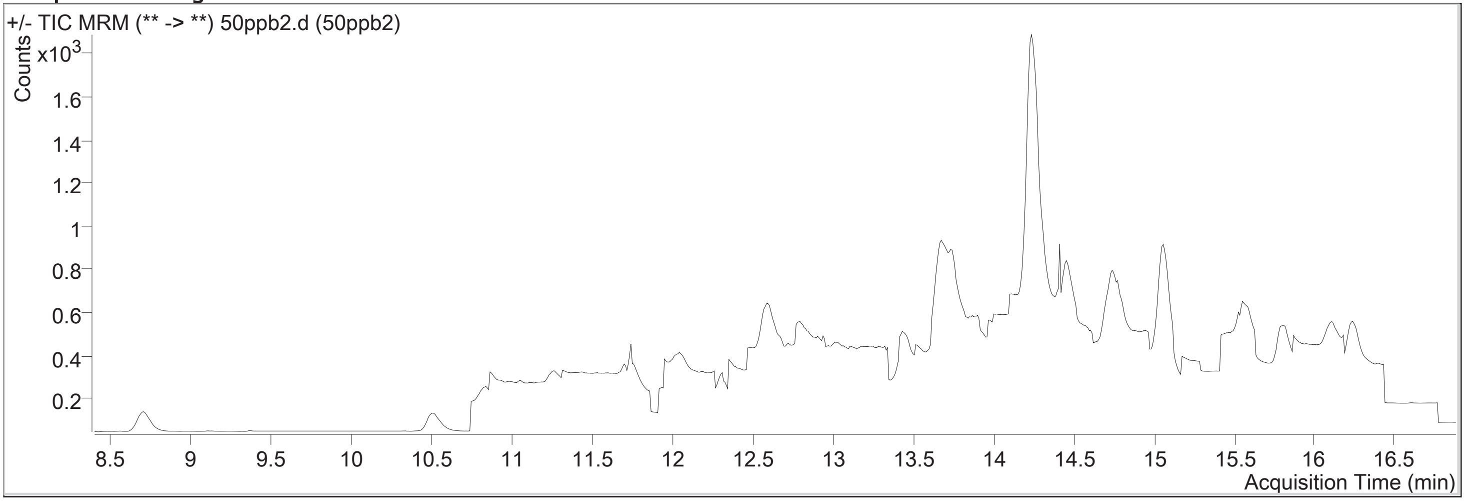

Compound	Transition	RT	Resp.	Final Conc	Units
Gallic acid	168.9 -> 125.0	8.708	533	27.9968	ng/ml
Protocatechuic acid	152.9 -> 108.9	10.509	486	35.2468	ng/ml
Pyrocatechol	109.0 -> 52.9	10.856	26	70.4132	ng/ml
3,4-Dihydroxyphenylacetic acid	167.0 -> 123.0	10.843	364	37.0830	ng/ml
(+)-Catechin	289.0 -> 245.0	11.261	198	30.8558	ng/ml
2,5-Dihydroxybenzoic acid	152.9 -> 109.0	11.937	265	42.4716	ng/ml
Chlorogenic acid	355.0 -> 163.0	11.760	731	40.1286	ng/ml
3-Hydroxybenzoic acid	137.0 -> 93.0	12.794	198	52.0752	ng/ml
4-Hydroxybenzoic acid	136.9 -> 93.1	12.047	576	36.6119	ng/ml
(-)-Epicatechin	291.0 -> 139.1	12.326	308	41.9072	ng/ml
Caffeic acid	179.0 -> 135.0	12.591	1226	23.9716	ng/ml
Syringic acid	196.9 -> 181.9	12.731	39	56.7659	ng/ml
Vanillin	151.0 -> 136.0	13.037	258	56.2214	ng/ml
Verbascoside	623.0 -> 160.8	13.434	529	39.4022	ng/ml
Taxifolin	303.0 -> 285.1	13.670	1650	19.0826	ng/ml
p-Coumaric acid	162.9 -> 119.0	13.750	1400	45.2729	ng/ml
Sinapic acid	222.9 -> 207.9	13.814	119	41.8883	ng/ml
Ferulic acid	193.0 -> 134.0	13.908	244	47.0246	ng/ml
Luteolin 7-glucoside	447.1 -> 285.0	14.239	5992	32.1840	ng/ml
Hesperidin	611.1 -> 303.0	14.302	328	43.9704	ng/ml
Hyperoside	465.1 -> 303.1	14.454	744	41.5821	ng/ml
Rosmarinic acid	359.0 -> 160.9	14.456	537	41.6587	ng/ml
Apigenin 7-glucoside	433.1 -> 271.0	14.730	1120	31.9925	ng/ml
Pinoresinol	357.0 -> 151.0	15.001	19	48.7333	ng/ml
2-Hydroxycinnamic acid	162.9 -> 119.1	14.760	900	40.6437	ng/ml
Eriodictyol	287.0 -> 151.0	15.061	1379	35.4807	ng/ml
Quercetin	301.0 -> 151.0	15.553	1023	37.6091	ng/ml
Luteolin	287.0 -> 153.1	15.791	633	34.9900	ng/ml
Kaempferol	285.0 -> 229.1	16.113	240	26.6800	ng/ml

Agilent Technologies

Compound	Transition	RT	Resp.	Final Conc	Units
Apigenin	271.0 -> 153.0	16.243	747	34.5354	ng/ml

Gallic acid

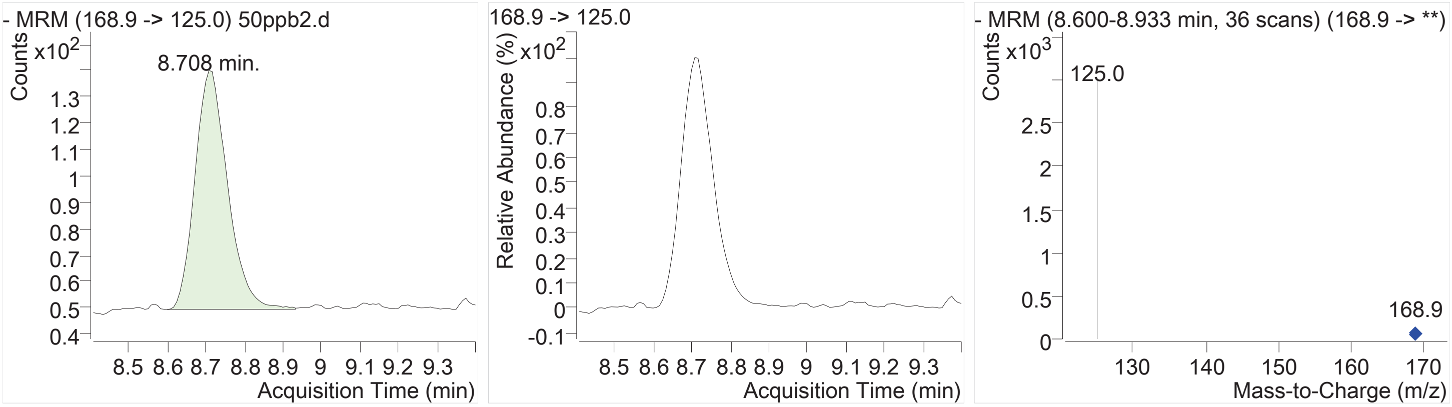

Protocatechuic acid

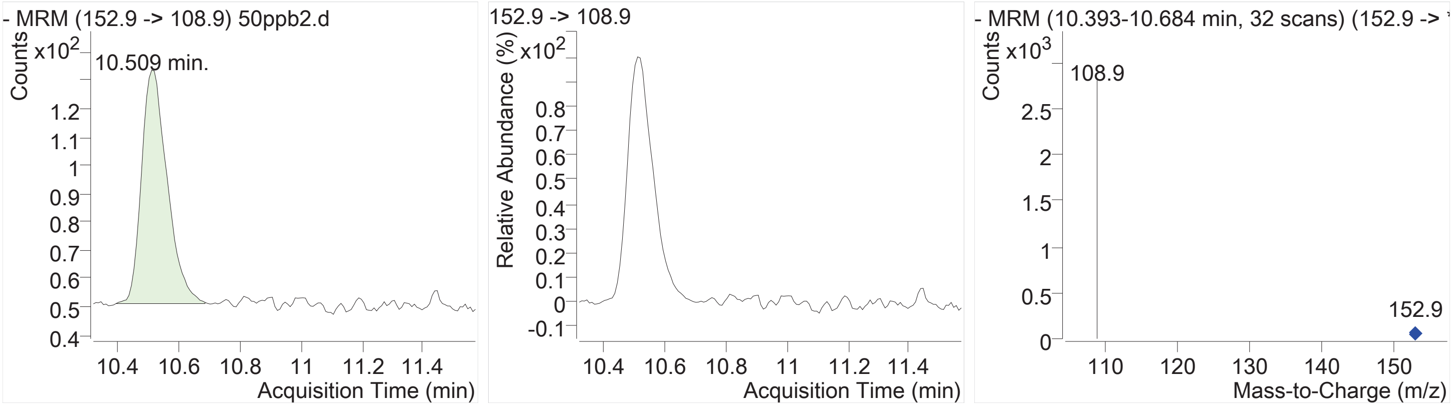

Pyrocatechol

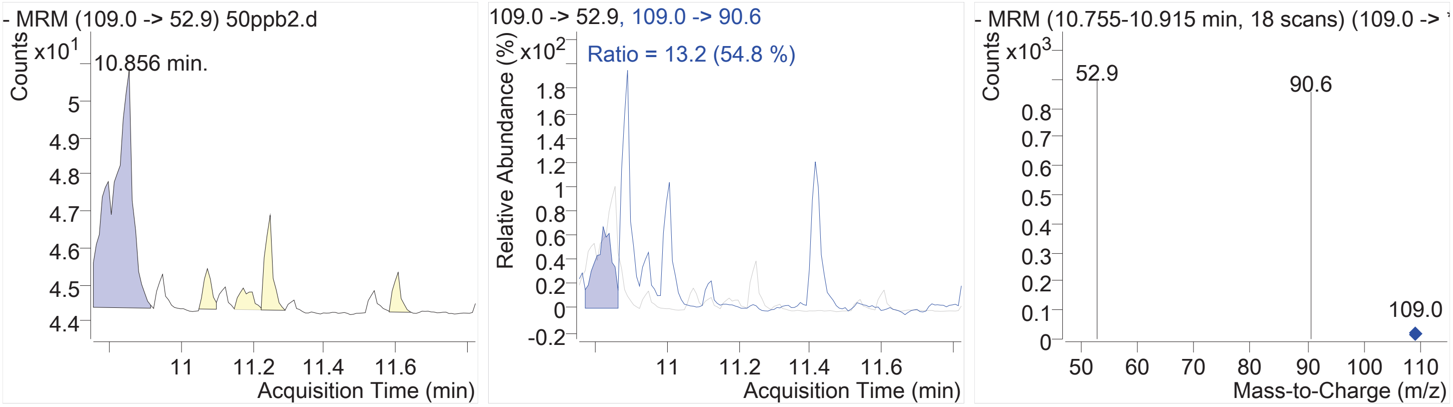

3,4-Dihydroxyphenylacetic acid

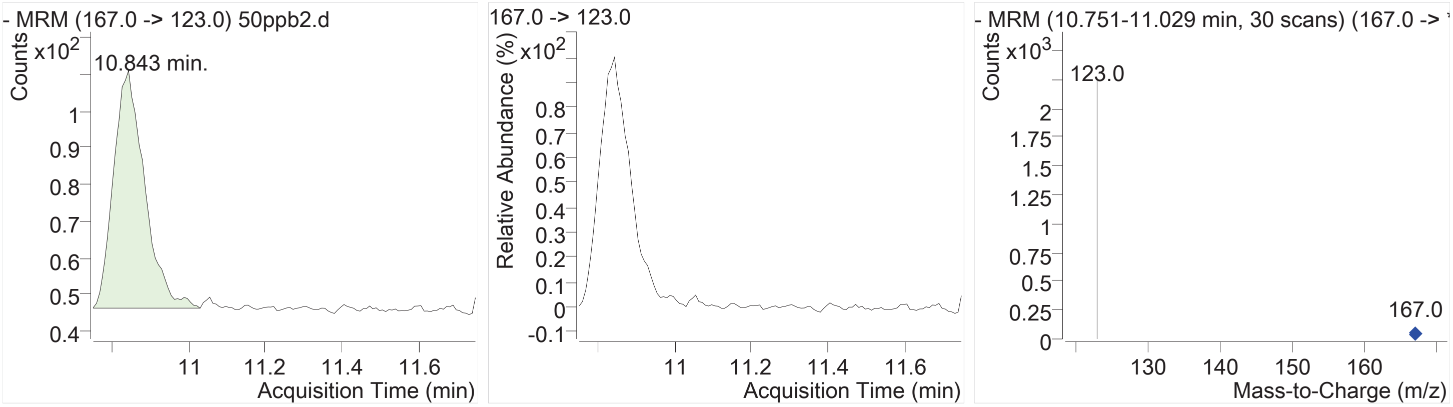

(+)-Catechin

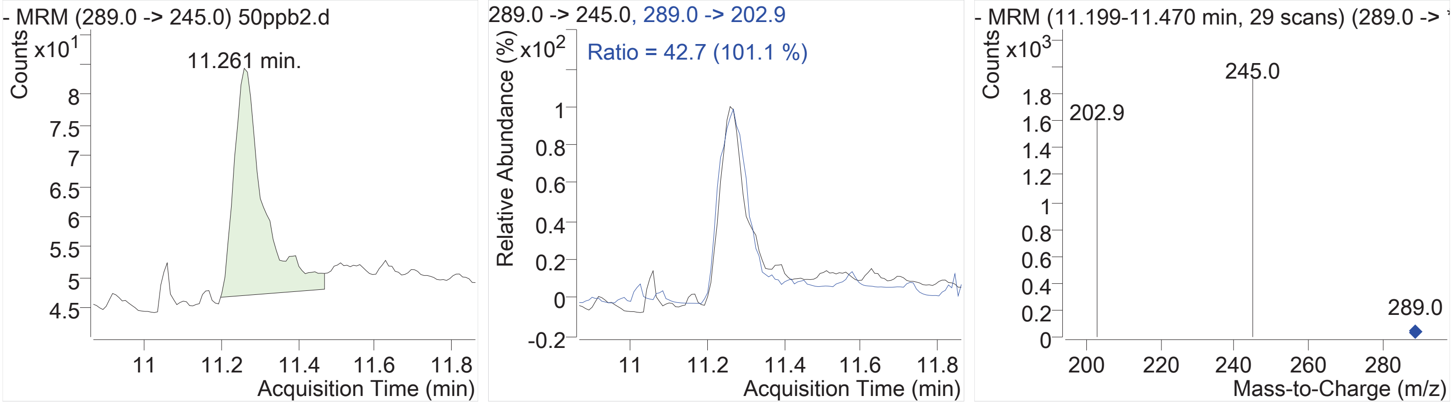

2,5-Dihydroxybenzoic acid

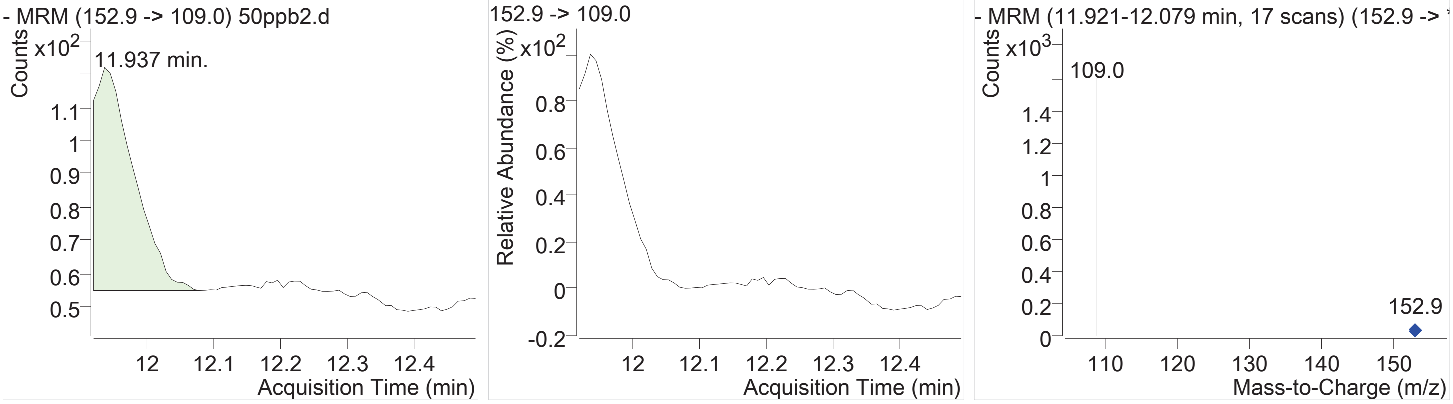

Chlorogenic acid

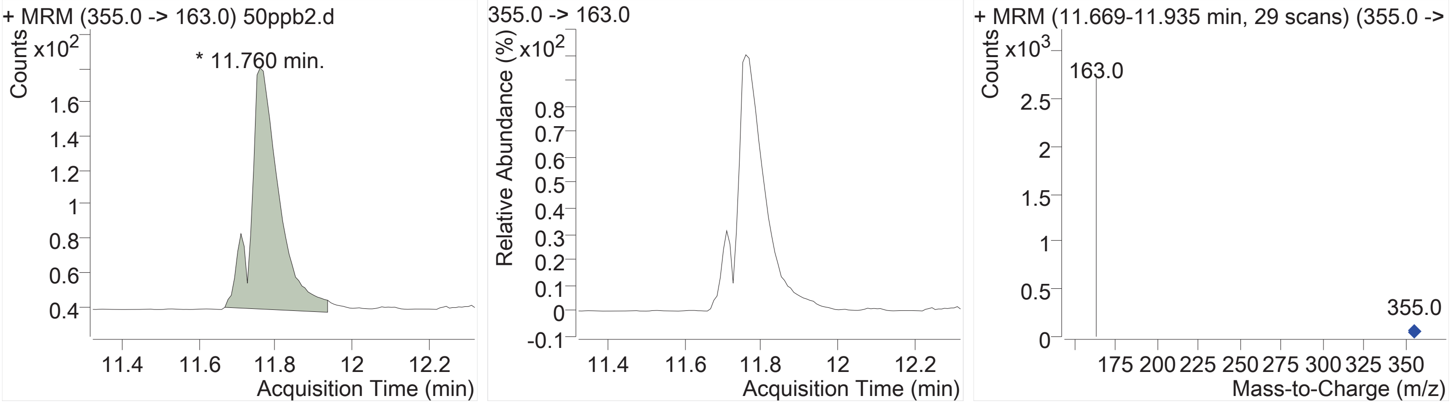

3-Hydroxybenzoic acid

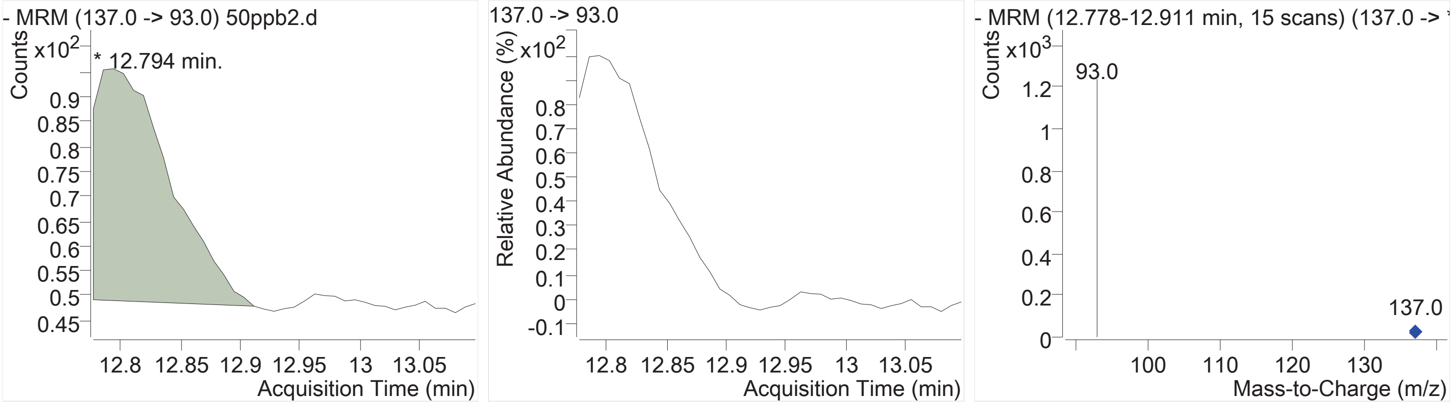

4-Hydroxybenzoic acid

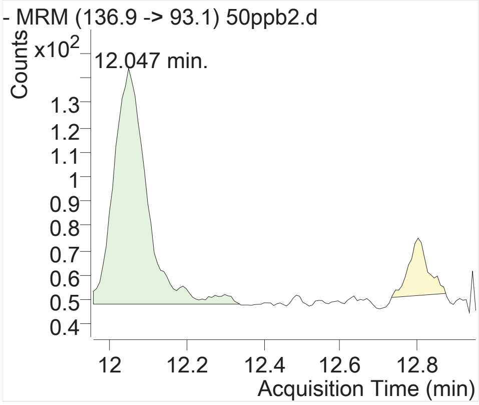

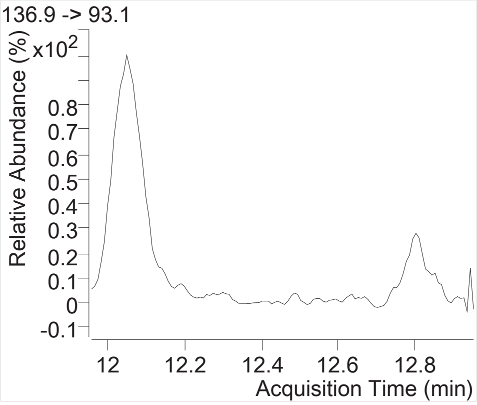

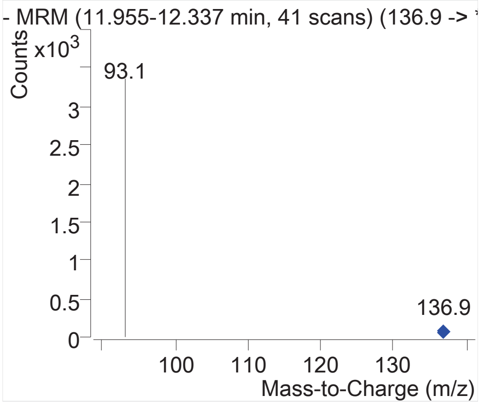

(-)-Epicatechin

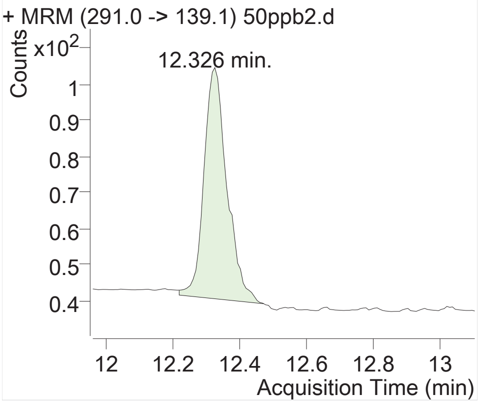

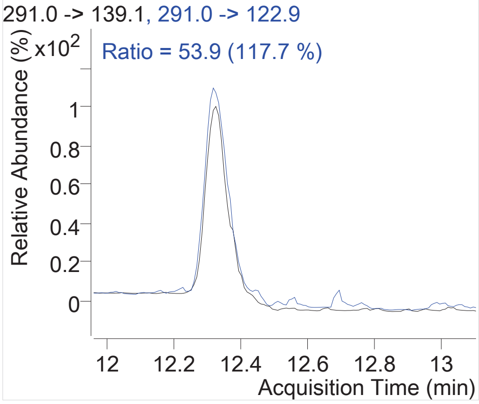

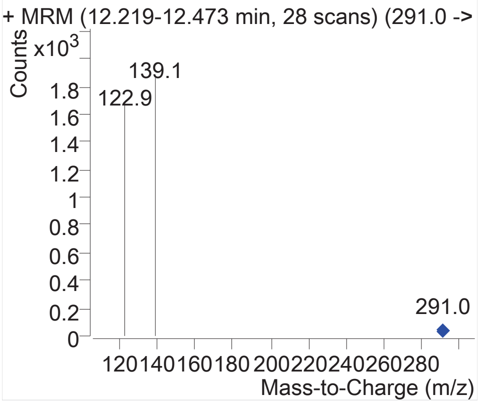

Caffeic acid

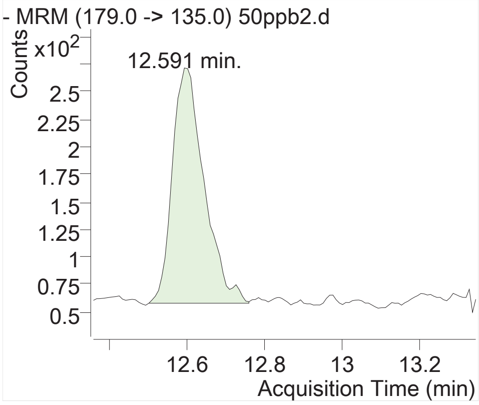

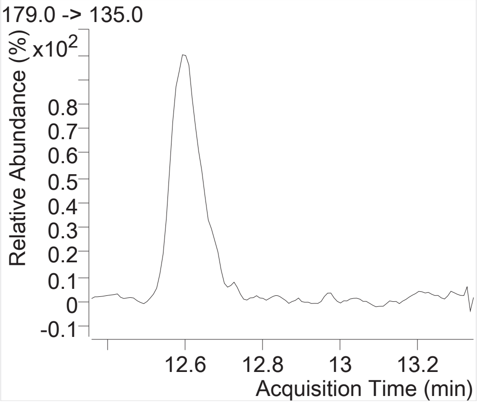

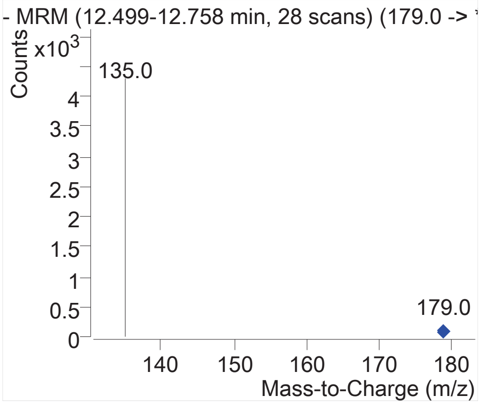

Syringic acid

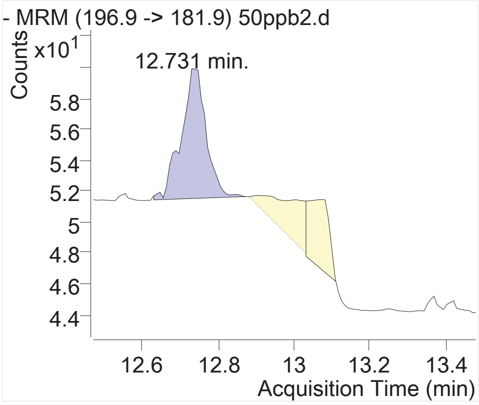

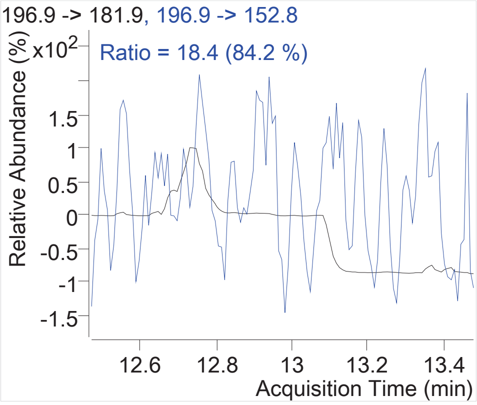

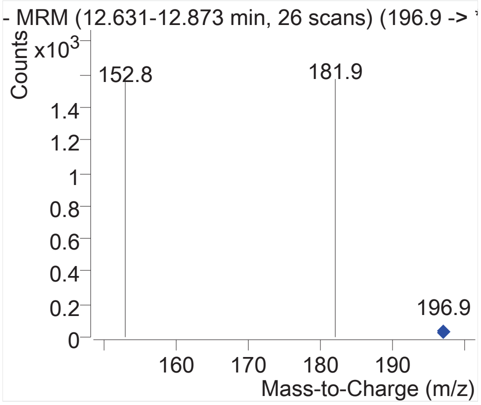

Vanillin

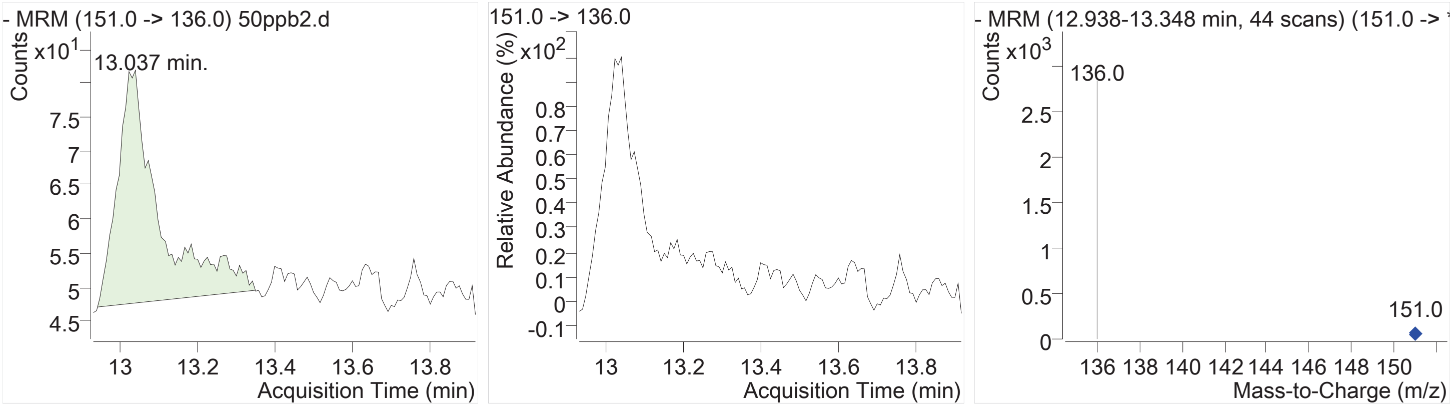

Verbascoside

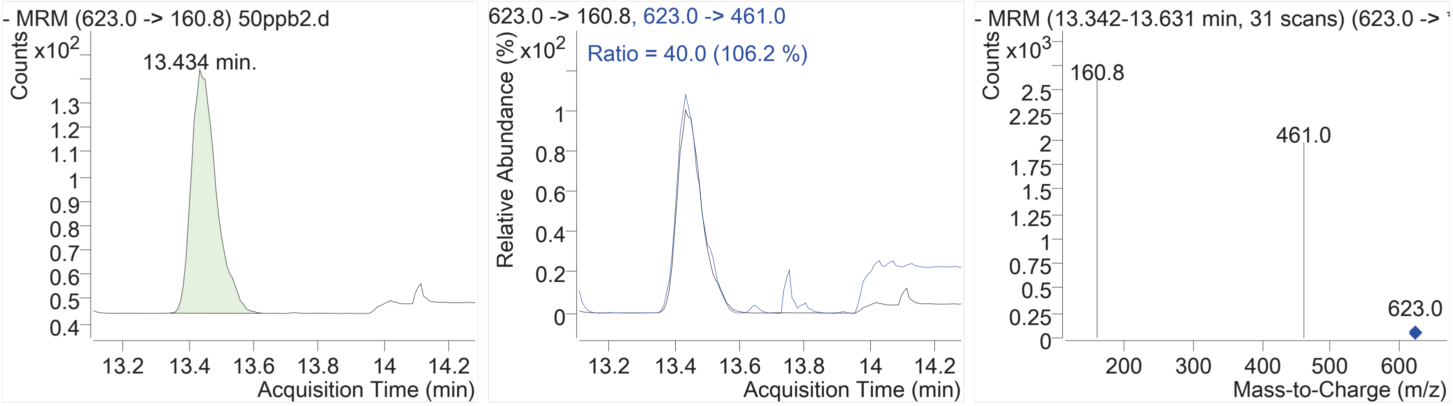

Taxifolin

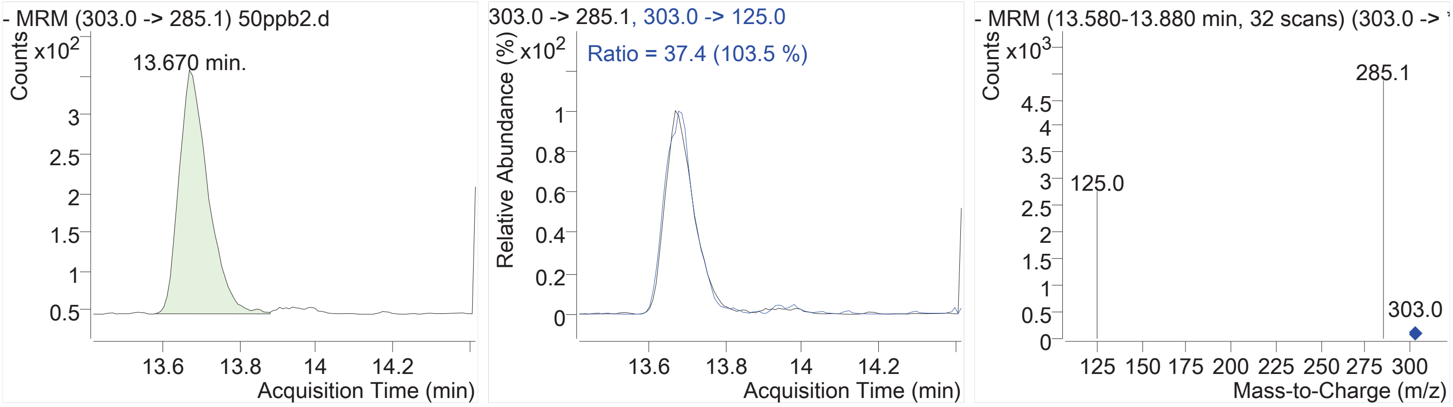

p-Coumaric acid

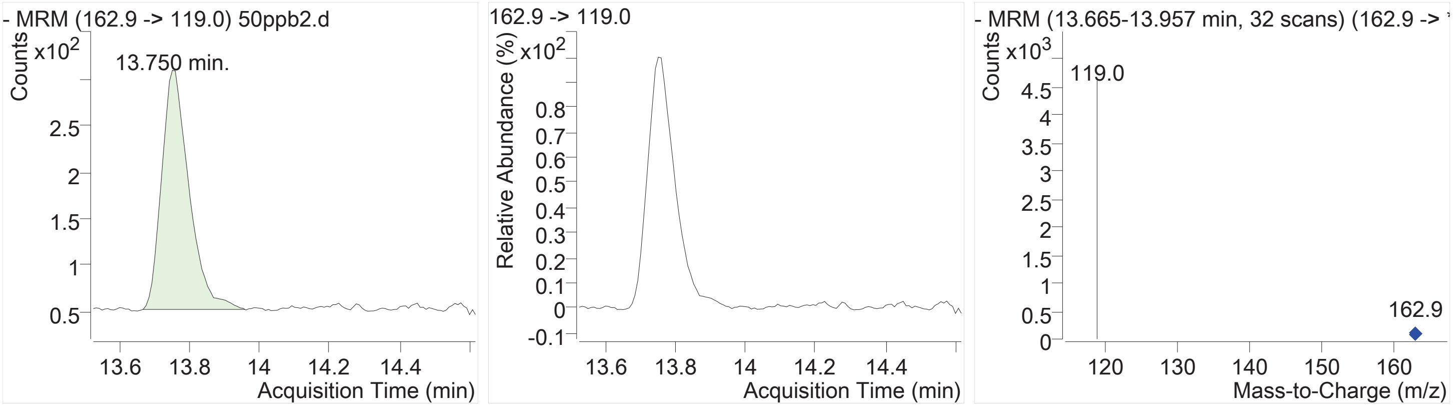

Sinapic acid

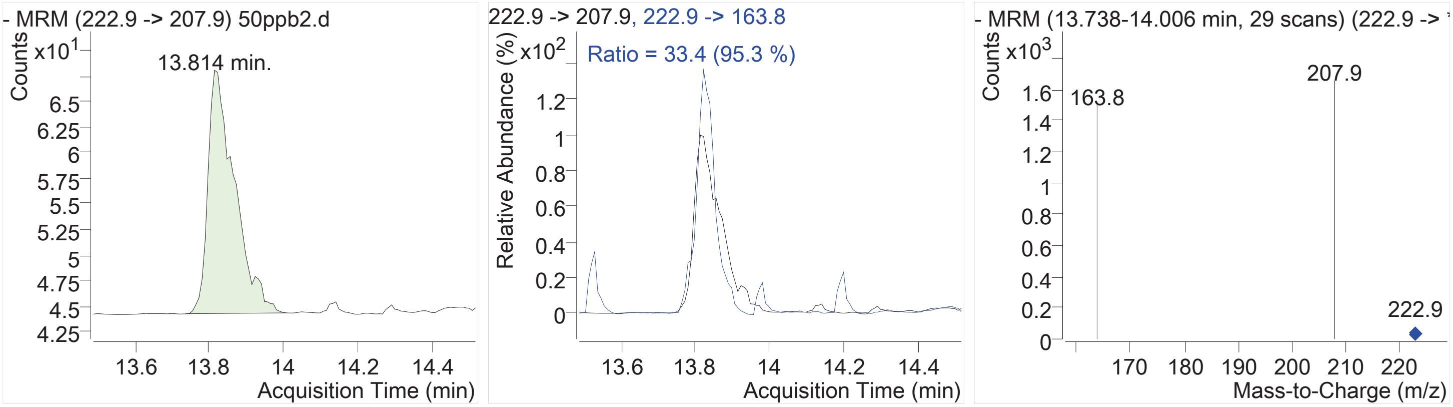

Ferulic acid

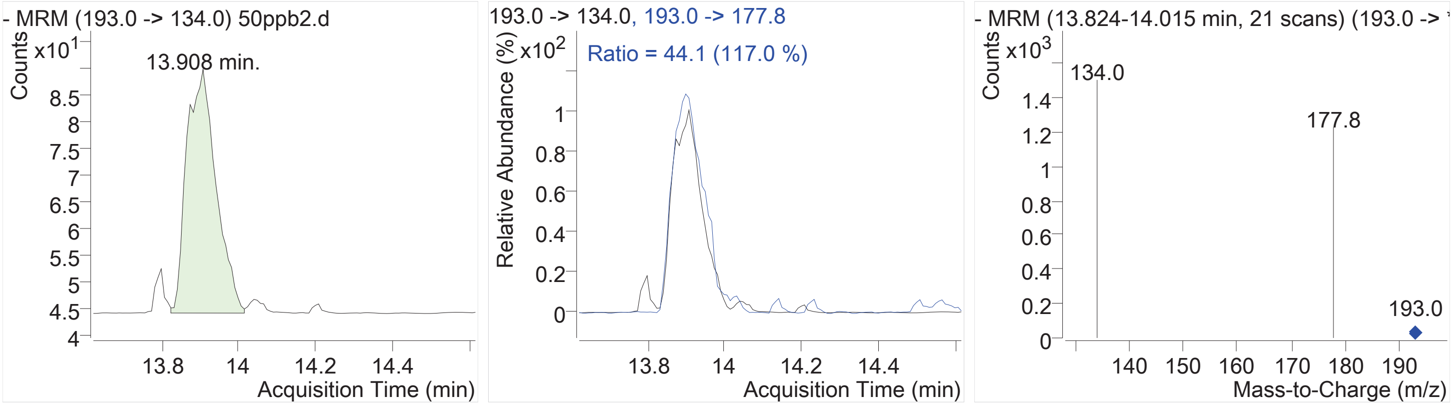

Luteolin 7-glucoside

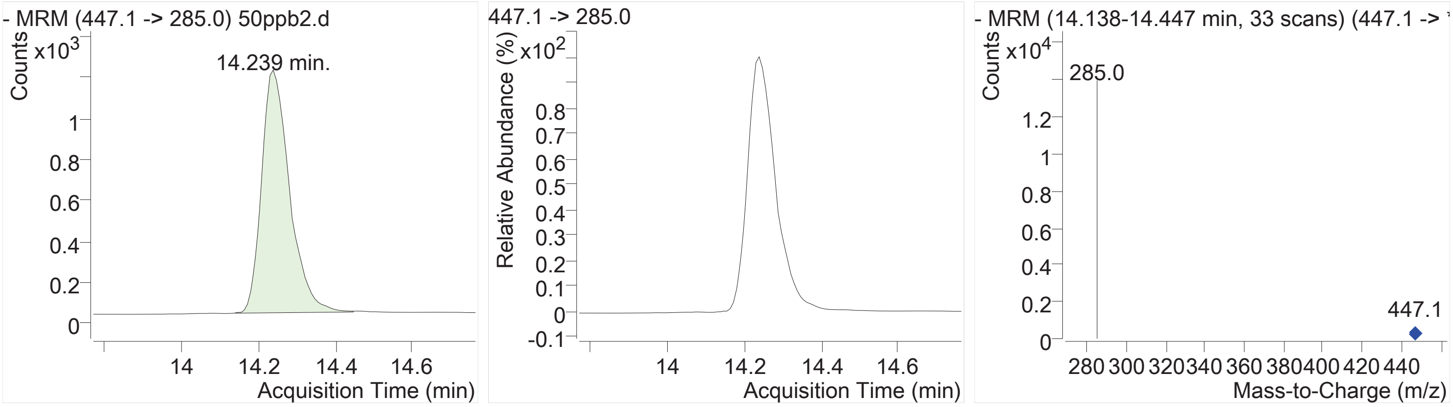

Hesperidin

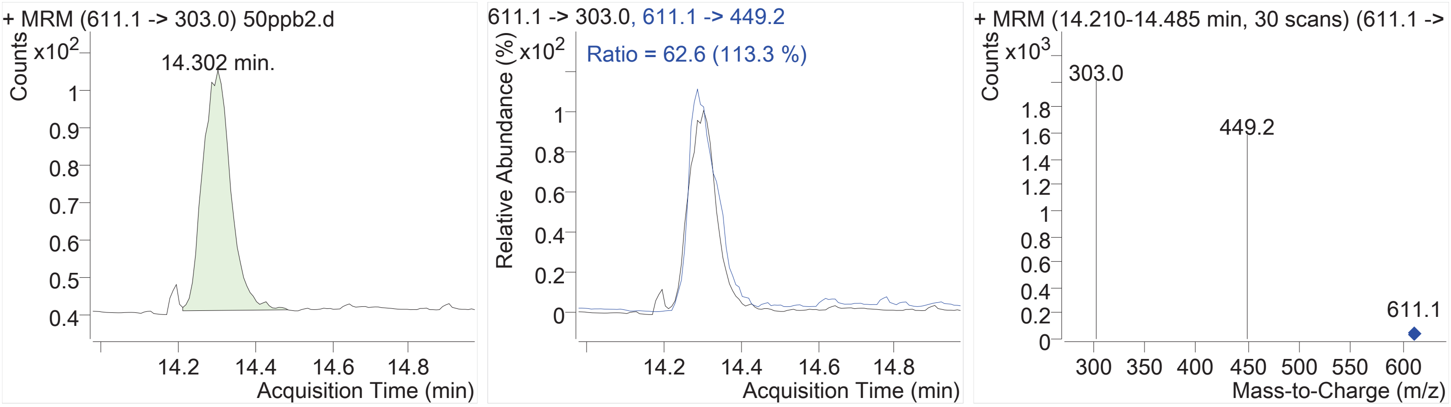

Hyperoside

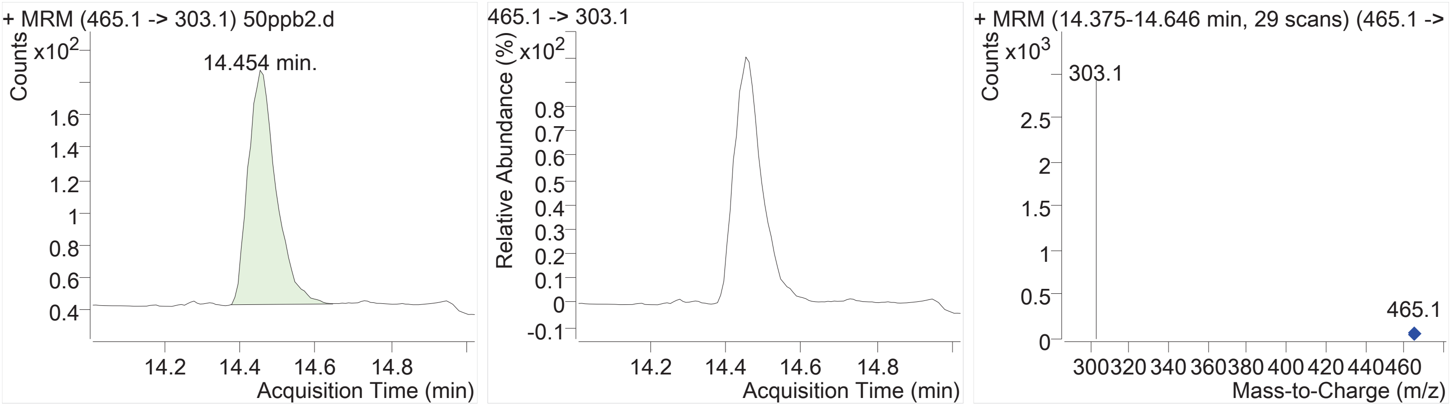

Rosmarinic acid

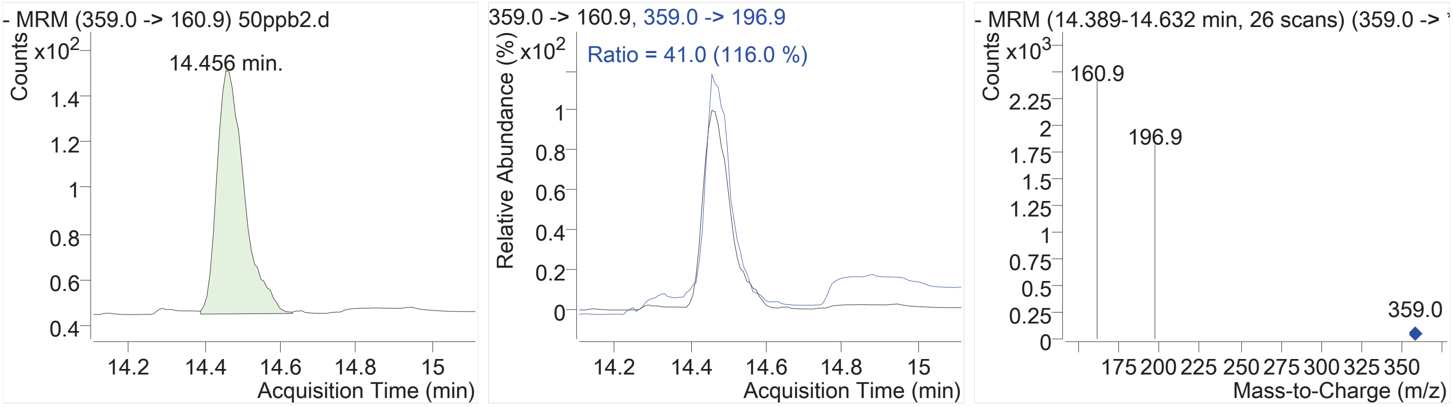

Apigenin 7-glucoside

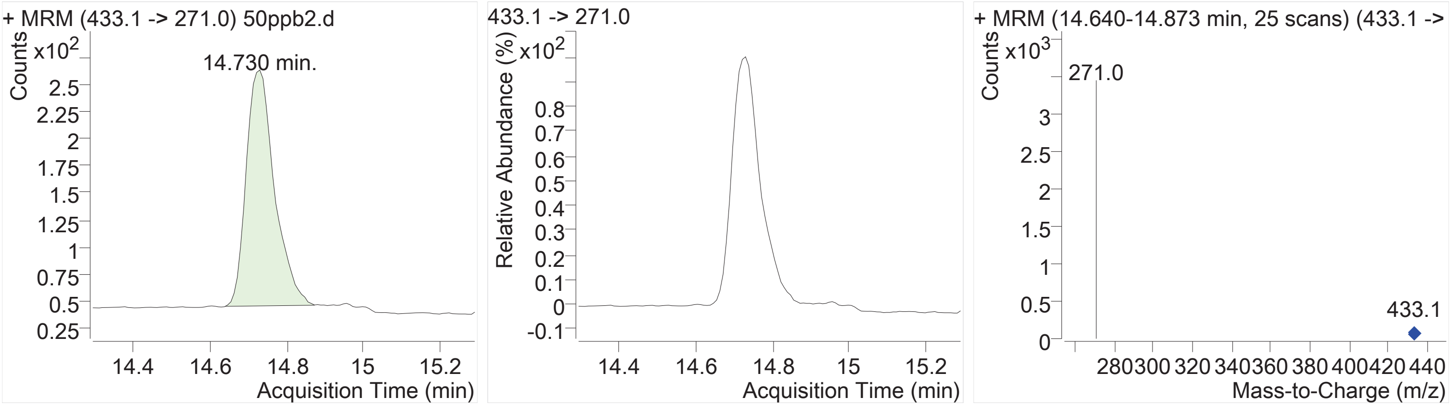

Pinoresinol

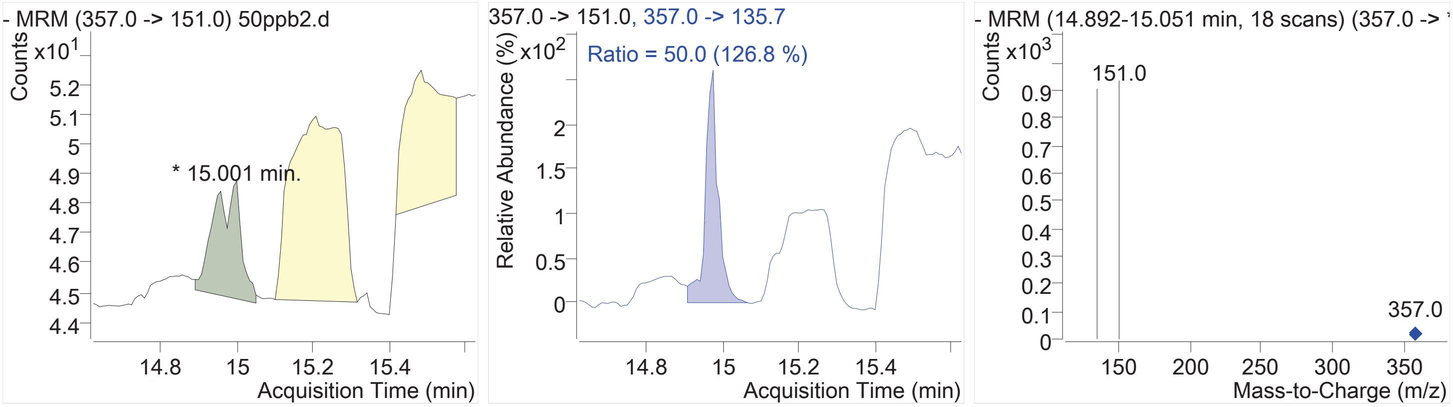

Agilent Technologies

2-Hydroxycinnamic acid

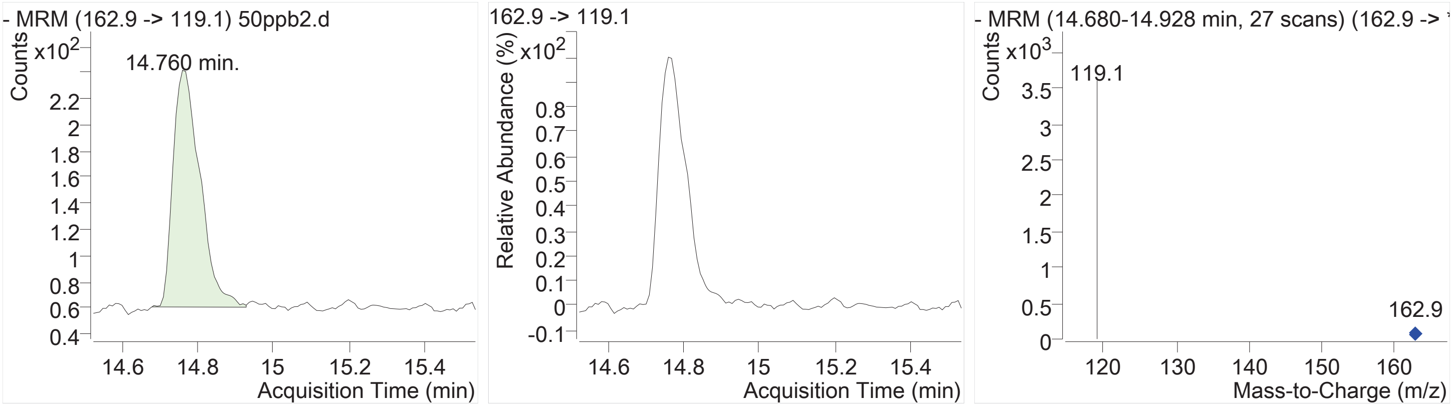

Eriodictyol

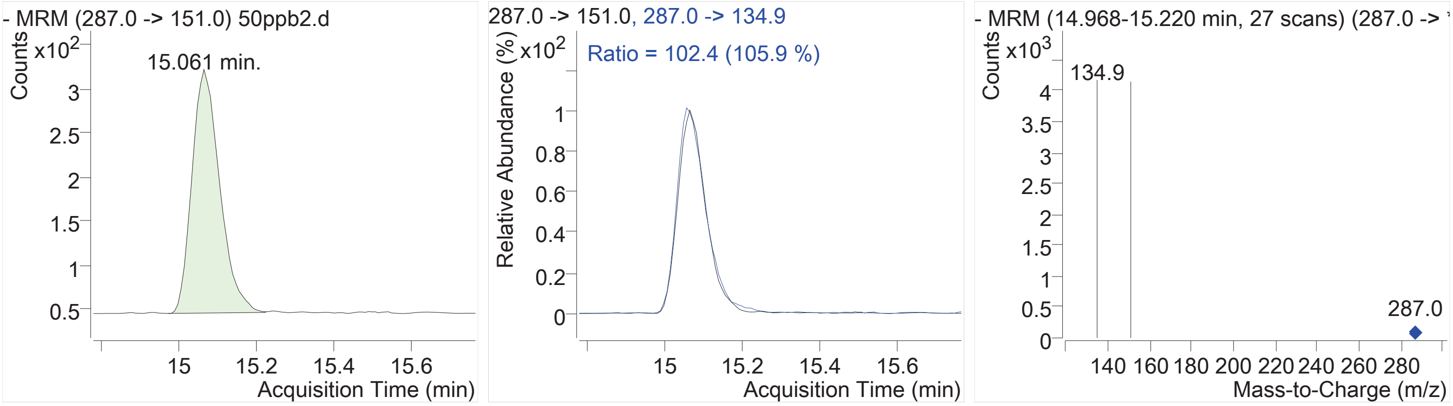

Quercetin

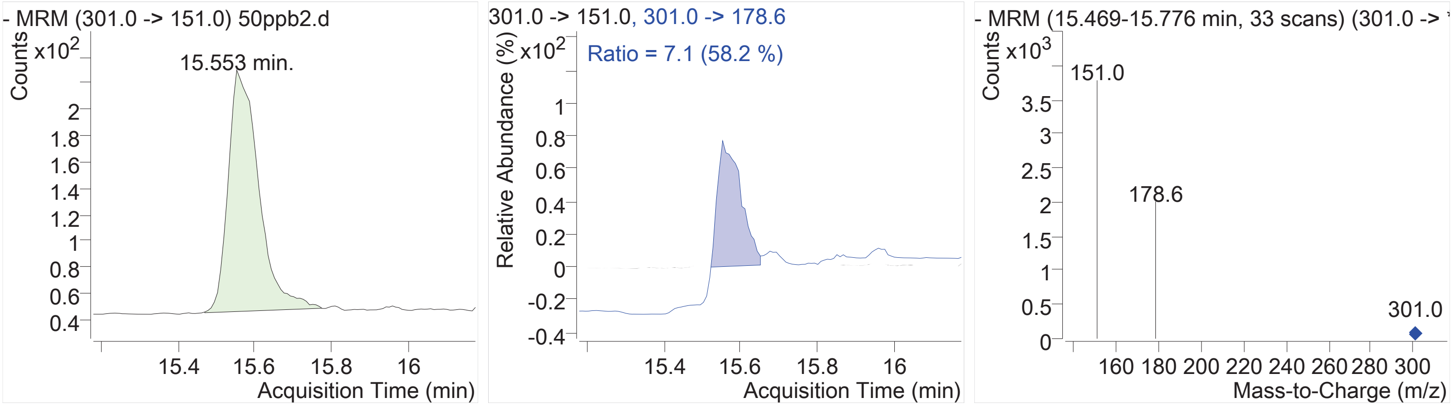

Luteolin

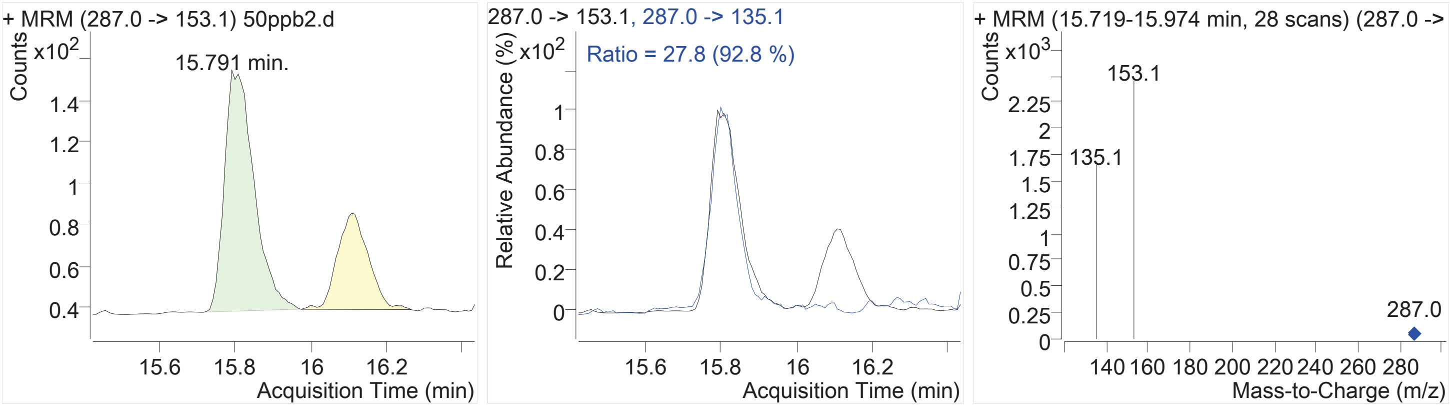

Kaempferol

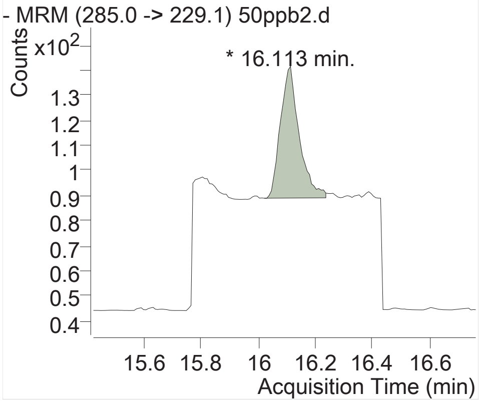

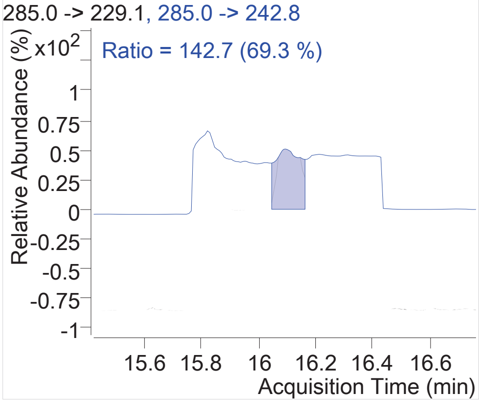

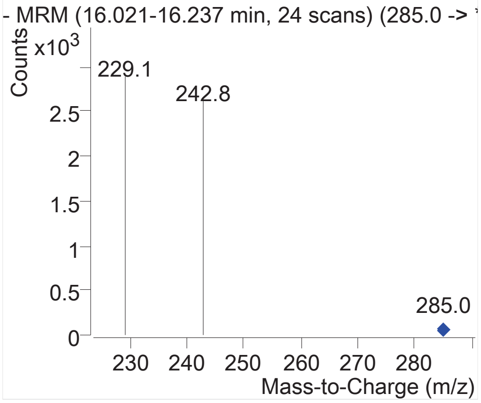

Apigenin

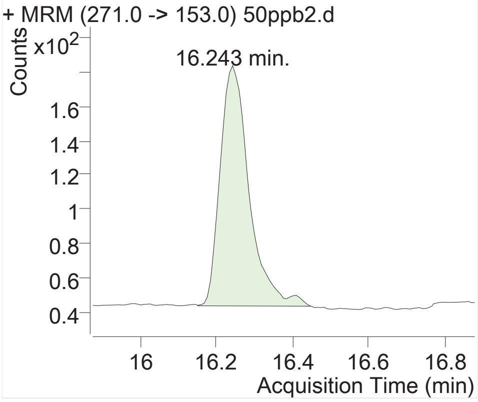

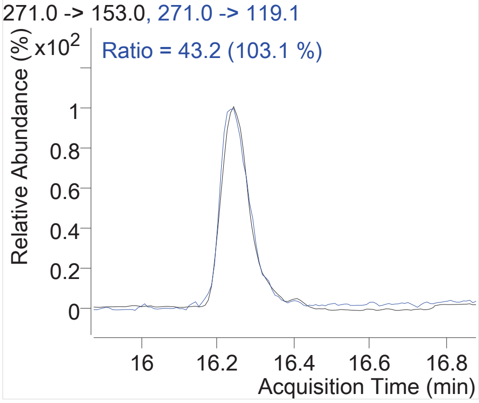

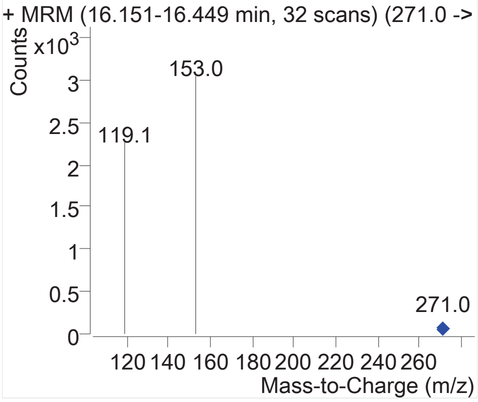

Quantitative Analysis Complete Report

**Batch Path**	D:\MassHunter\Data\2022ekim\061022cengizhoca\QuantResults\071022.batch.bin		
**Analysis Time**	10/11/2022 1:33:26 PM	**Analyst Name**	Defam-PC\admin
**Report Time**	10/11/2022 1:34:05 PM	**Reporter Name**	admin
**Last Calib Update**	10/11/2022 1:33:17 PM	**Batch State**	Processed
**Quant Batch Version**	B.07.01	**Quant Report Version**	B.07.01

**Acq. Time**	10/6/2022 5:43:35 PM	**Data File**	50ppb3.d
**Sample Type**	Cal	**Sample Name**	50ppb3
**Dilution**	1	**Acq. Method**	FENOLIK_DMRM2021-31bilesen.m

Sample Chromatogram

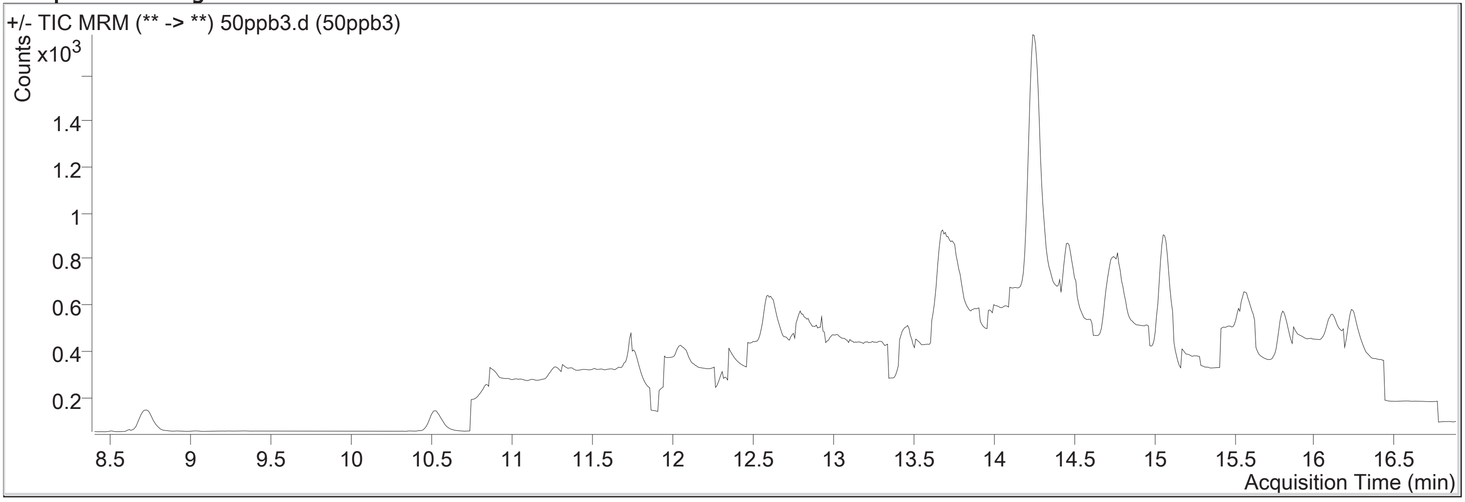

Compound	Transition	RT	Resp.	Final Conc	Units
Gallic acid	168.9 -> 125.0	8.733	581	32.5043	ng/ml
Protocatechuic acid	152.9 -> 108.9	10.526	521	38.6519	ng/ml
Pyrocatechol	109.0 -> 52.9	10.805	20	47.7965	ng/ml
3,4-Dihydroxyphenylacetic acid	167.0 -> 123.0	10.843	387	39.9036	ng/ml
(+)-Catechin	289.0 -> 245.0	11.277	229	40.4031	ng/ml
2,5-Dihydroxybenzoic acid	152.9 -> 109.0	11.946	217	34.8201	ng/ml
Chlorogenic acid	355.0 -> 163.0	11.768	945	50.1352	ng/ml
3-Hydroxybenzoic acid	137.0 -> 93.0	12.803	200	52.4551	ng/ml
4-Hydroxybenzoic acid	136.9 -> 93.1	12.055	590	37.9583	ng/ml
(-)-Epicatechin	291.0 -> 139.1	12.334	359	48.2992	ng/ml
Caffeic acid	179.0 -> 135.0	12.599	1223	23.8337	ng/ml
Syringic acid	196.9 -> 181.9	12.748	34	50.3990	ng/ml
Vanillin	151.0 -> 136.0	13.037	272	60.6644	ng/ml
Verbascoside	623.0 -> 160.8	13.467	517	38.2816	ng/ml
Taxifolin	303.0 -> 285.1	13.678	1717	21.7492	ng/ml
p-Coumaric acid	162.9 -> 119.0	13.766	1381	44.5407	ng/ml
Sinapic acid	222.9 -> 207.9	13.839	151	51.8771	ng/ml
Ferulic acid	193.0 -> 134.0	13.916	226	43.7032	ng/ml
Luteolin 7-glucoside	447.1 -> 285.0	14.247	5445	26.1764	ng/ml
Hesperidin	611.1 -> 303.0	14.302	391	51.5761	ng/ml
Hyperoside	465.1 -> 303.1	14.462	799	44.3805	ng/ml
Rosmarinic acid	359.0 -> 160.9	14.464	600	46.6701	ng/ml
Apigenin 7-glucoside	433.1 -> 271.0	14.739	1412	40.6706	ng/ml
Pinoresinol	357.0 -> 151.0	14.967	21	56.3671	ng/ml
2-Hydroxycinnamic acid	162.9 -> 119.1	14.777	952	43.0716	ng/ml
Eriodictyol	287.0 -> 151.0	15.061	1421	37.1279	ng/ml
Quercetin	301.0 -> 151.0	15.561	1025	37.6559	ng/ml
Luteolin	287.0 -> 153.1	15.808	659	36.6506	ng/ml
Kaempferol	285.0 -> 229.1	16.113	272	34.5596	ng/ml

Quantitative Analysis Complete Report

Agilent Technologies

Compound	Transition	RT	Resp.	Final Conc	Units
Apigenin	271.0 -> 153.0	16.235	816	38.2329	ng/ml

Gallic acid

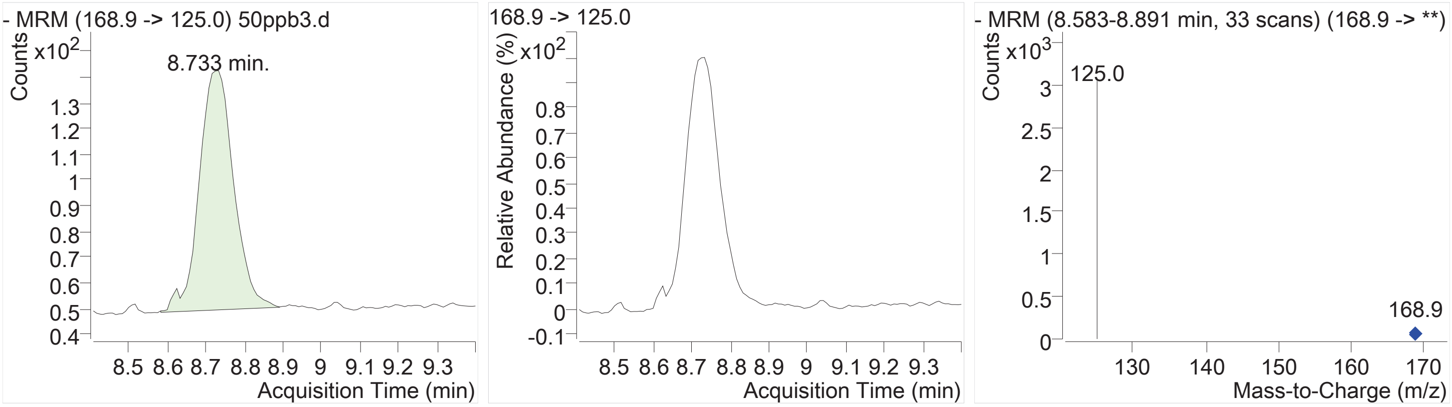

Protocatechuic acid

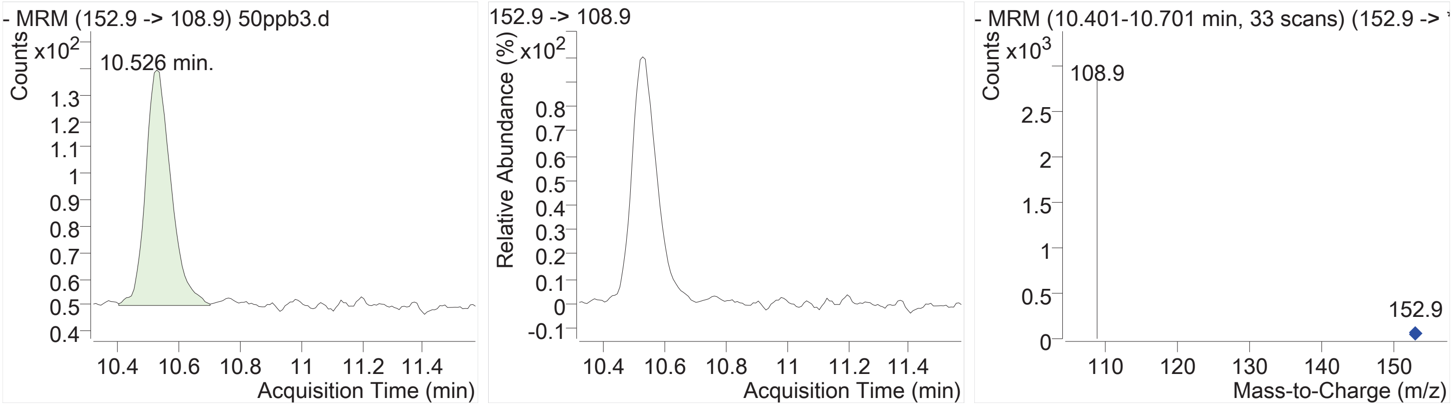

Pyrocatechol

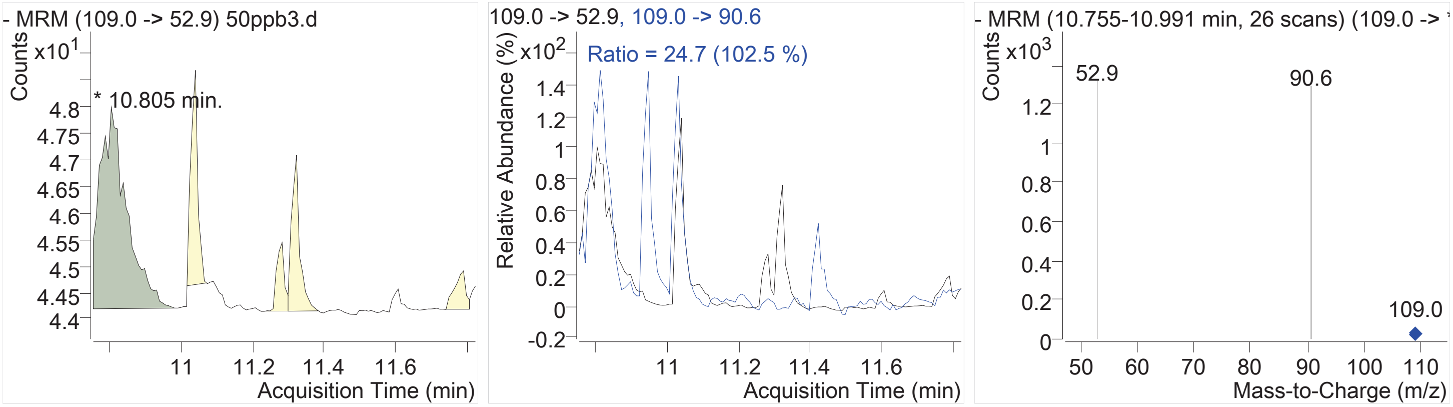

3,4-Dihydroxyphenylacetic acid

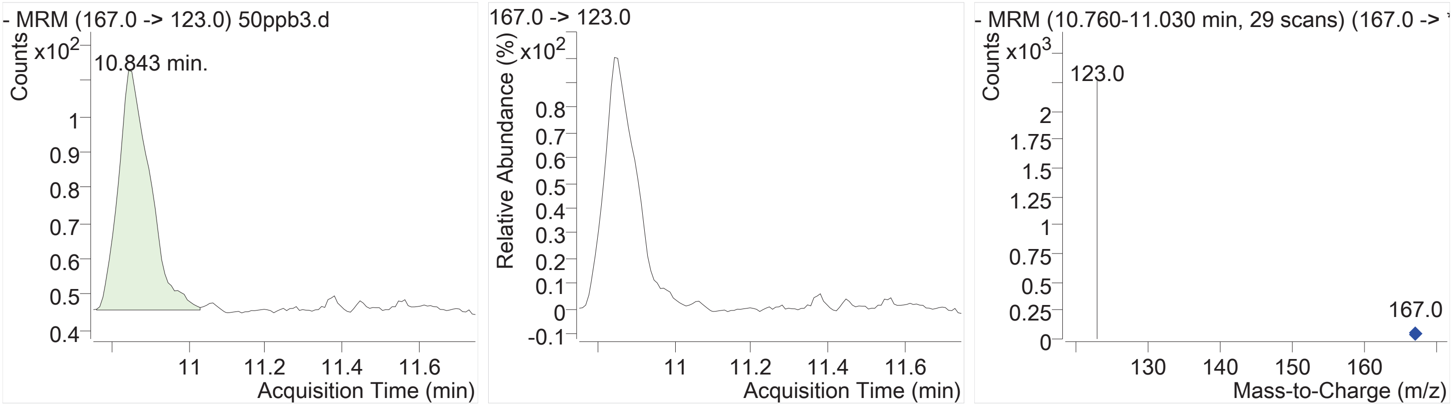

(+)-Catechin

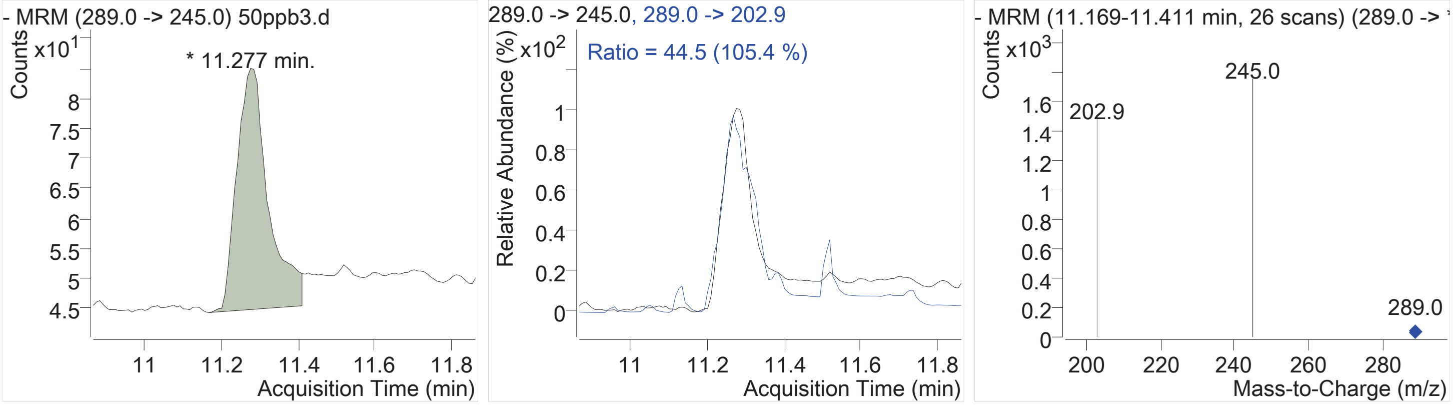

2,5-Dihydroxybenzoic acid

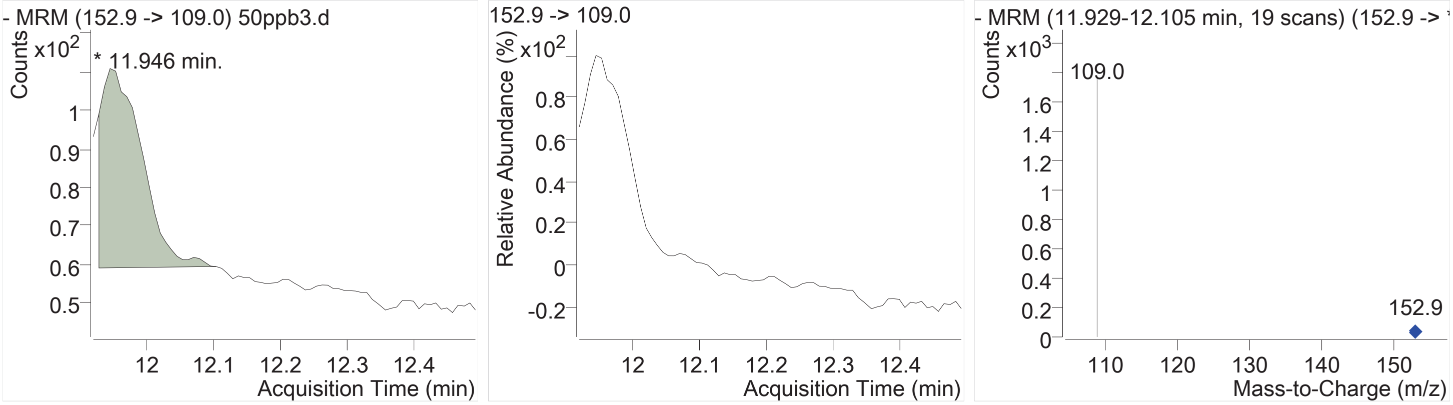

Chlorogenic acid

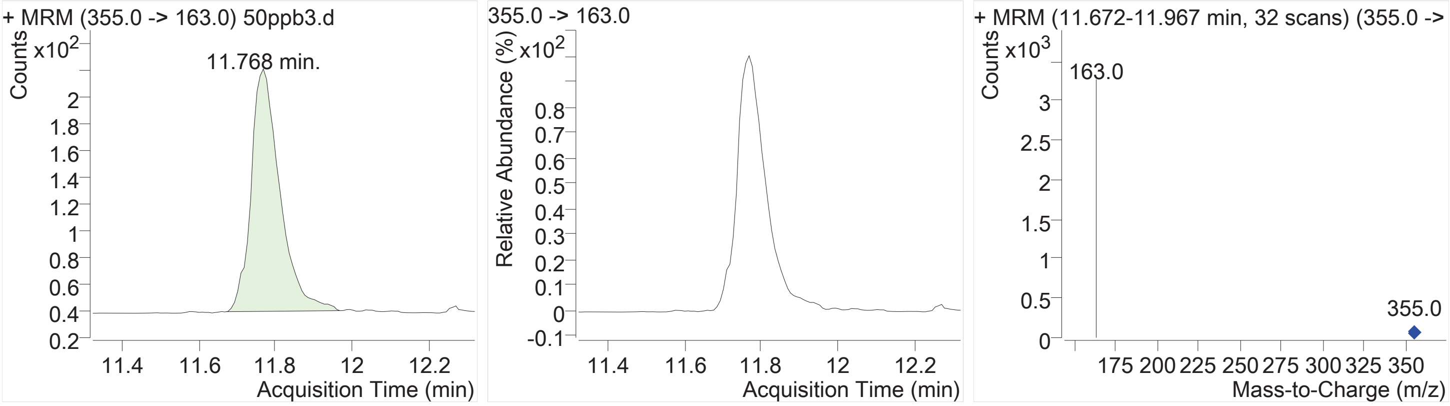

3-Hydroxybenzoic acid

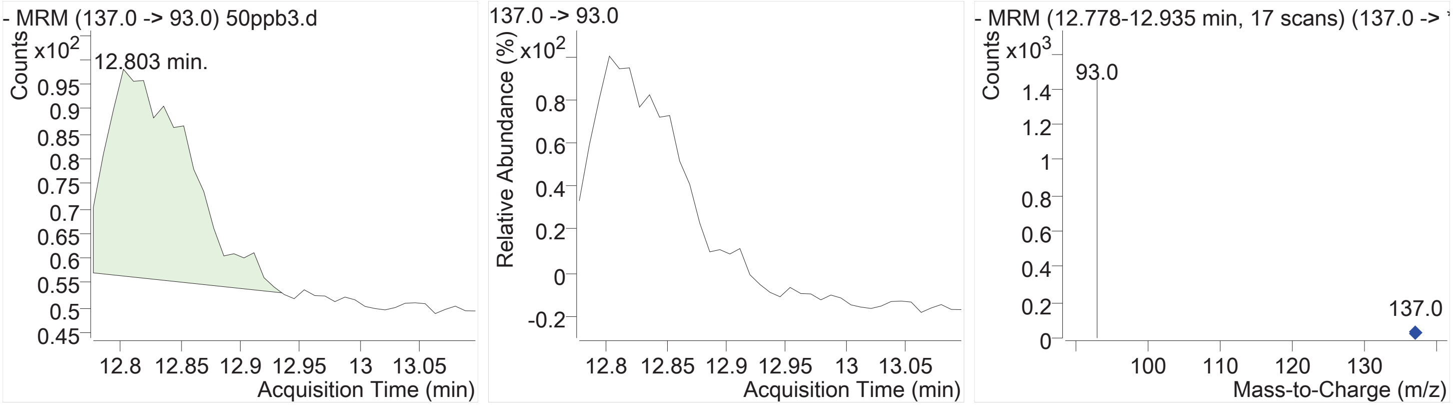

4-Hydroxybenzoic acid

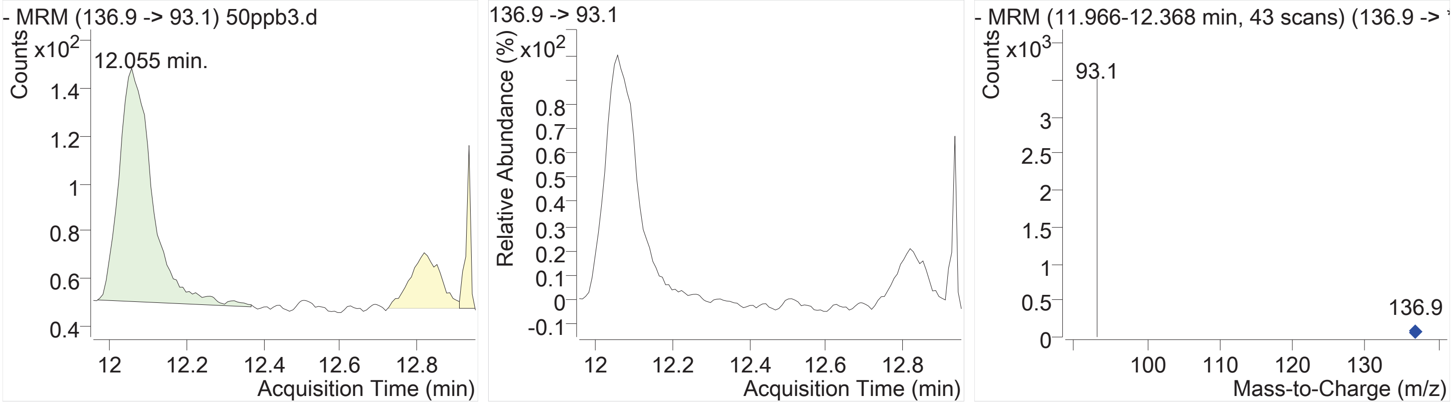

(-)-Epicatechin

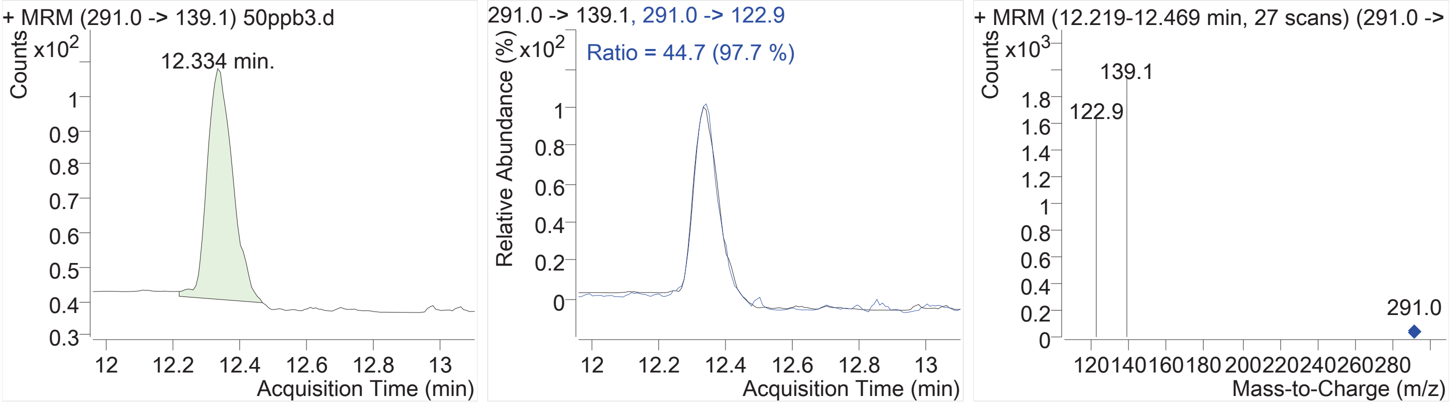

Caffeic acid

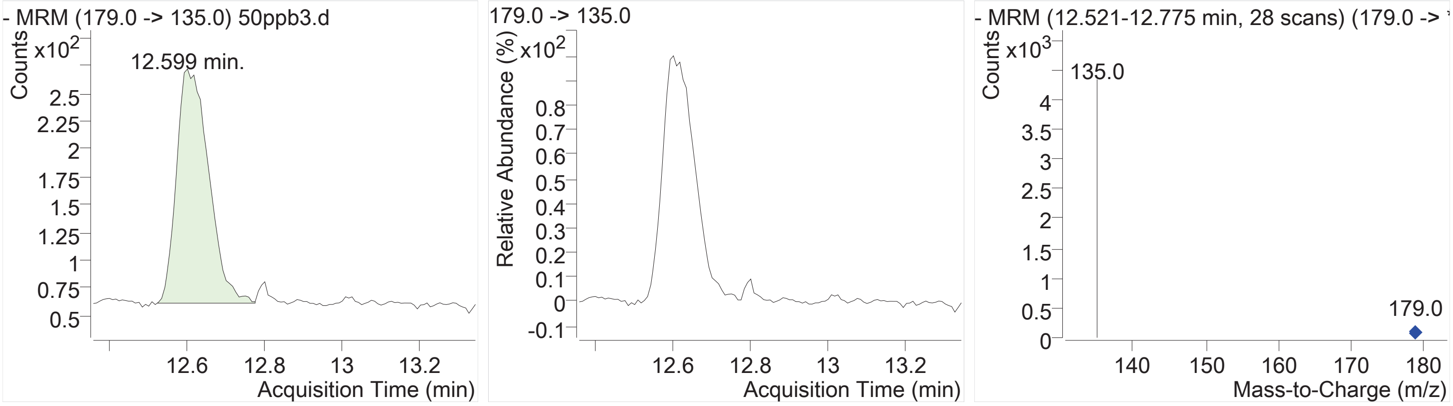

Syringic acid

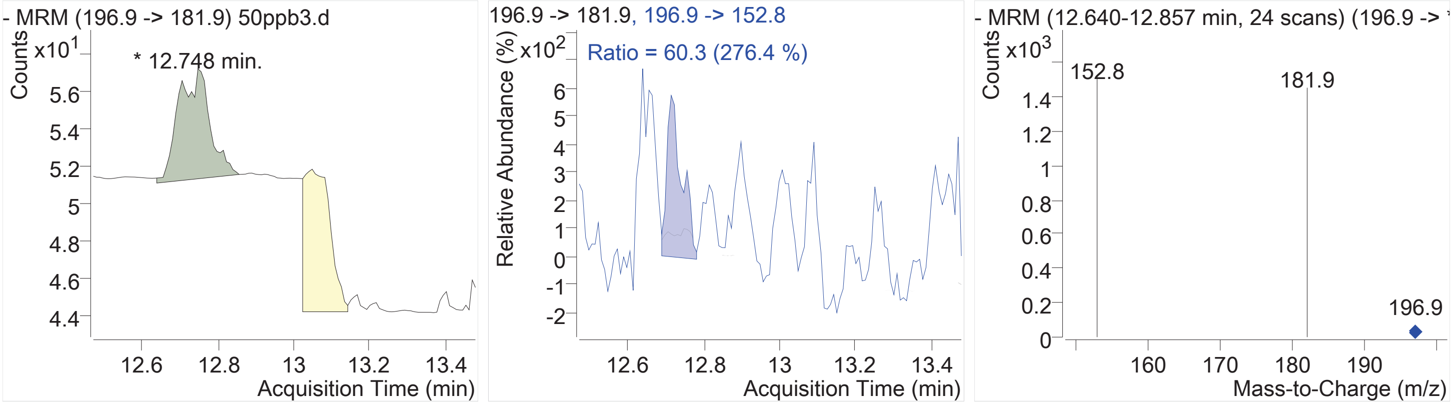

Vanillin

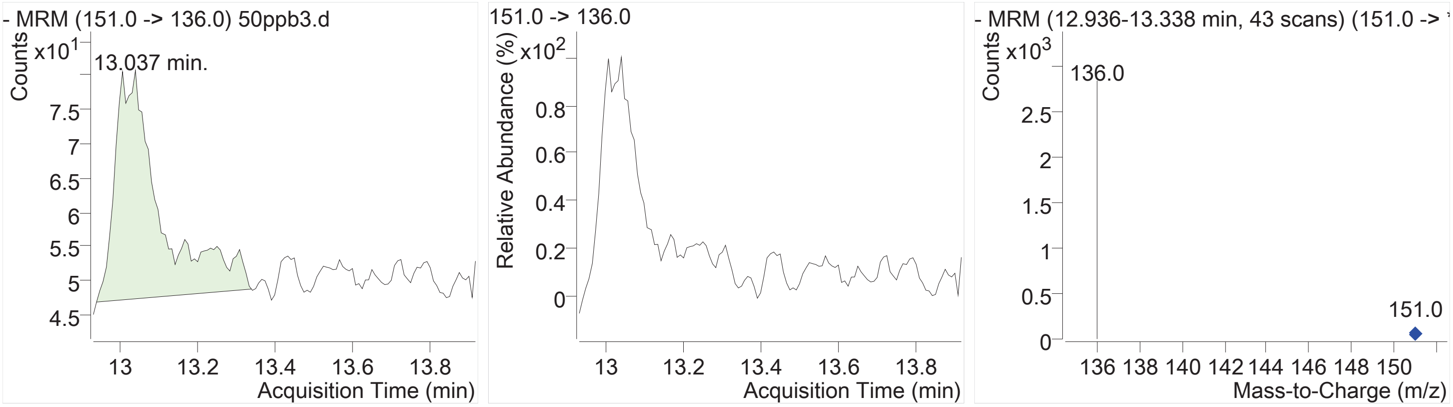

Verbascoside

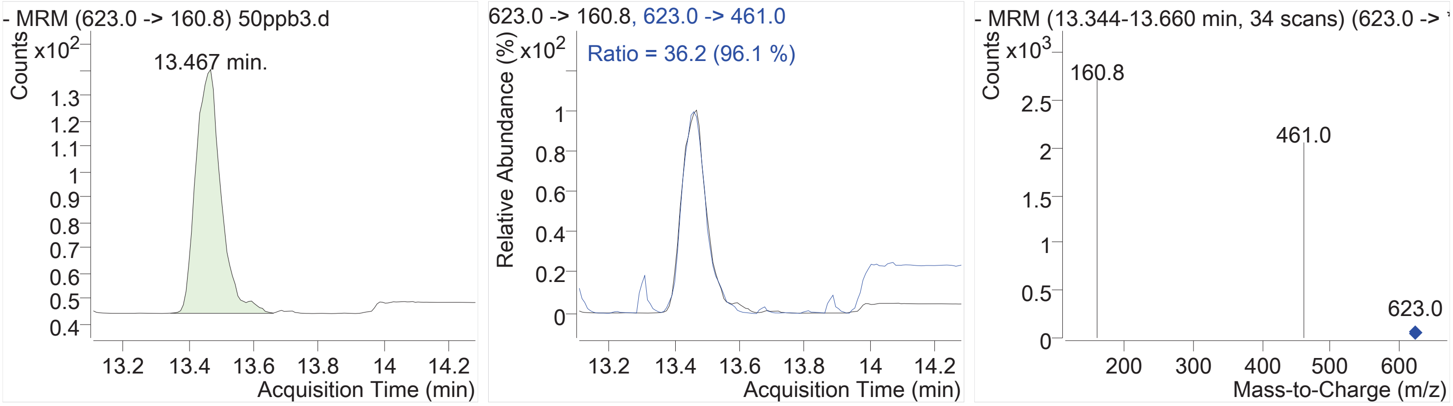

Taxifolin

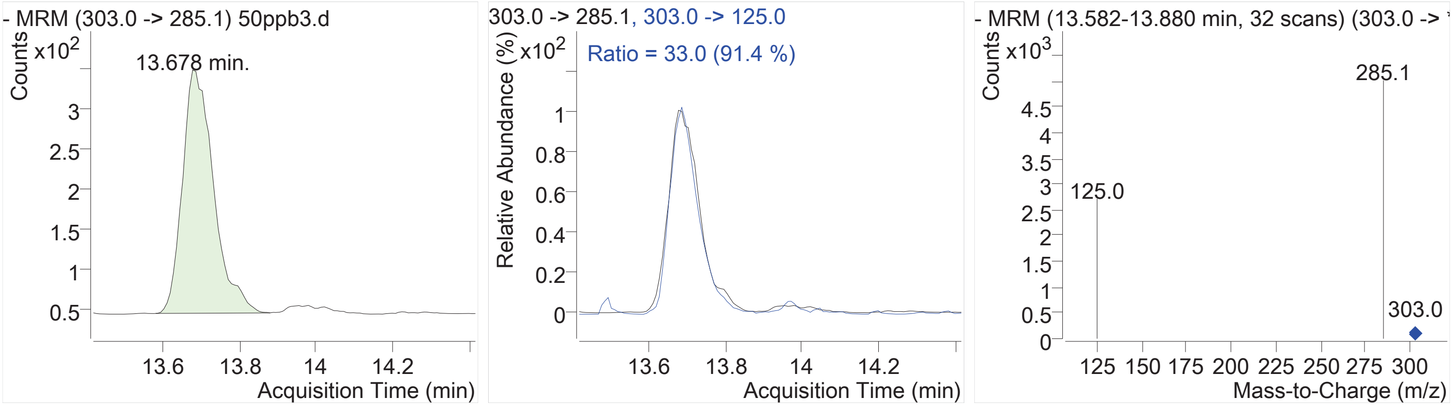

p-Coumaric acid

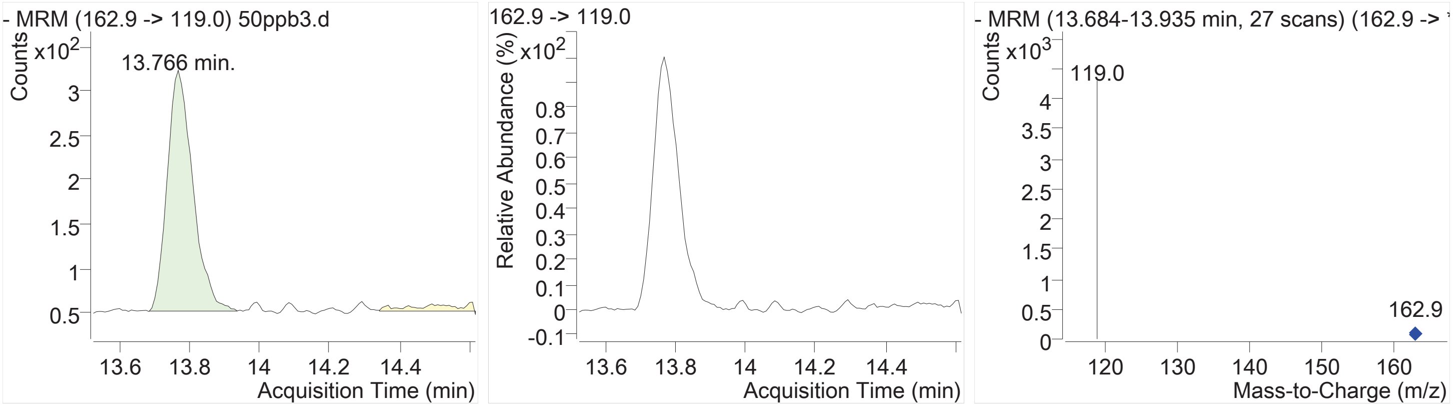

Sinapic acid

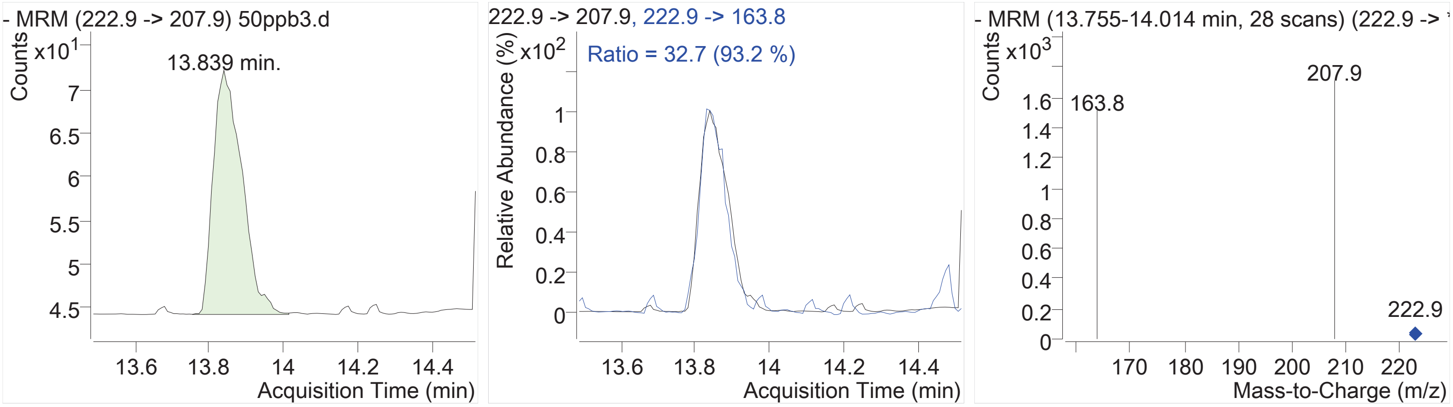

Ferulic acid

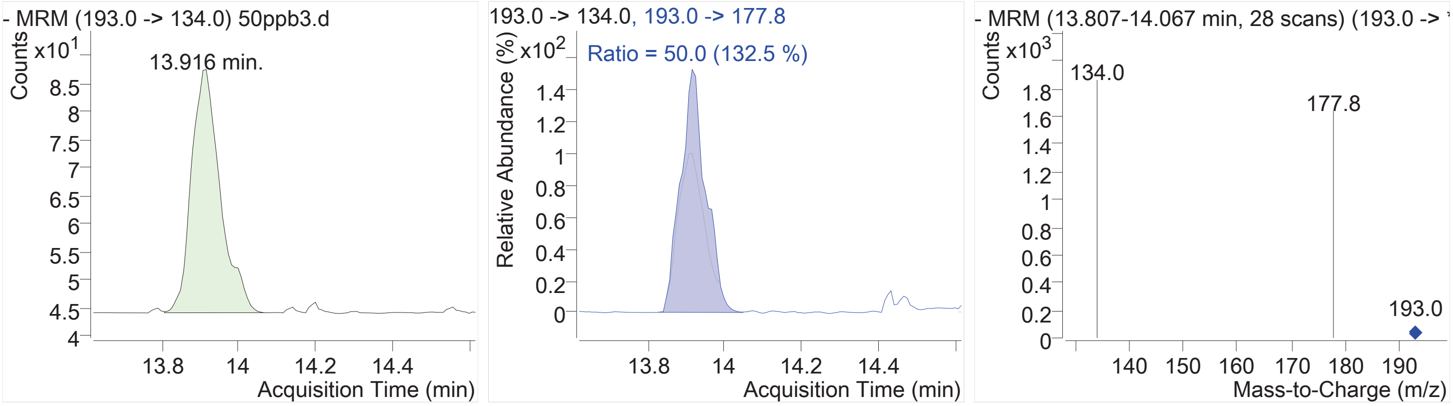

Luteolin 7-glucoside

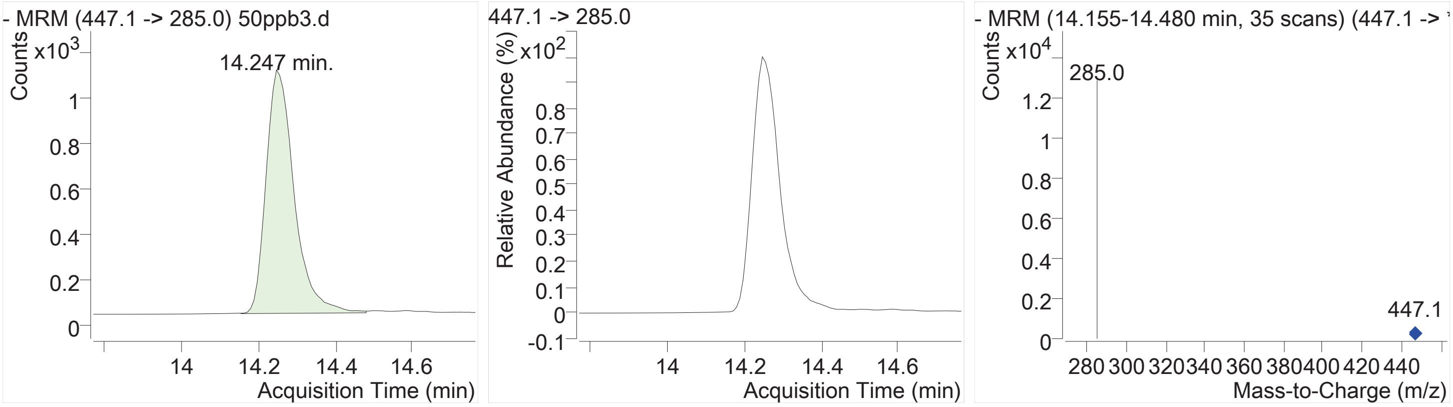

Hesperidin

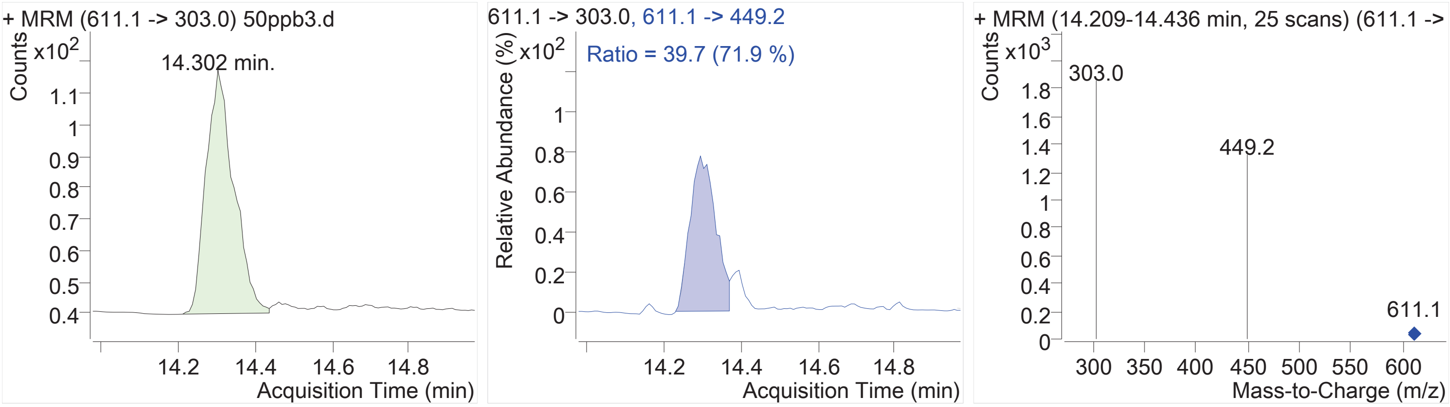

Hyperoside

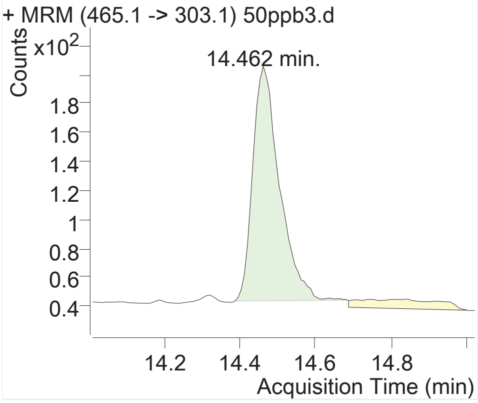

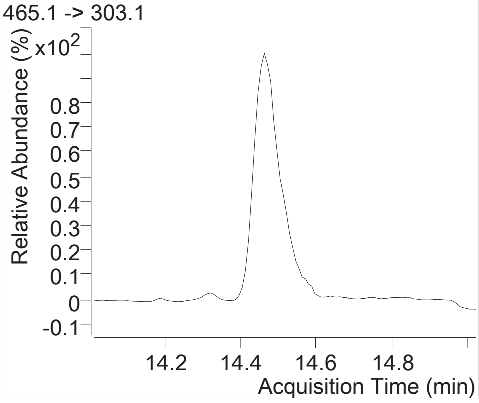

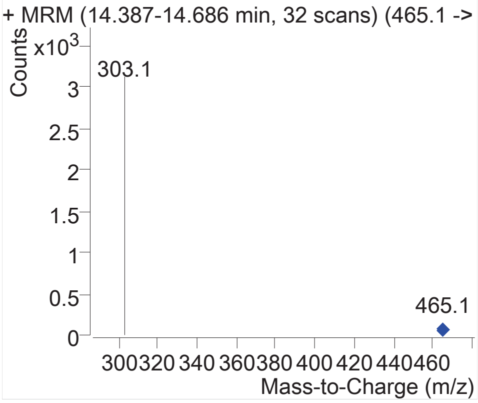

Rosmarinic acid

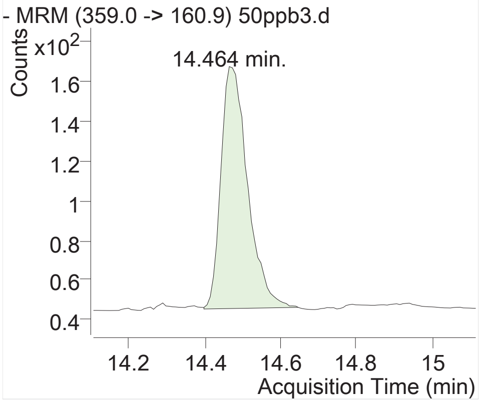

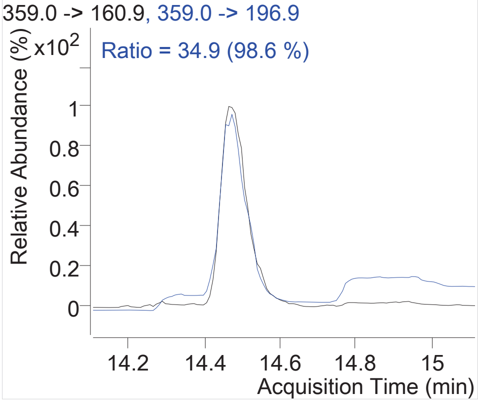

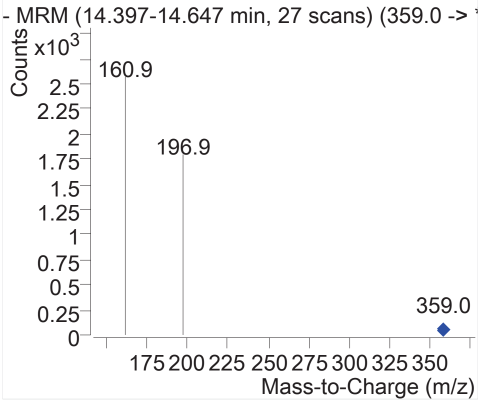

Apigenin 7-glucoside

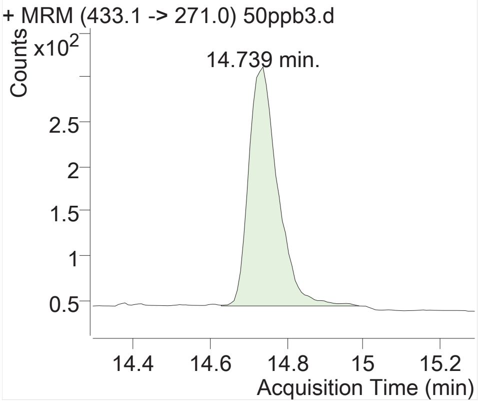

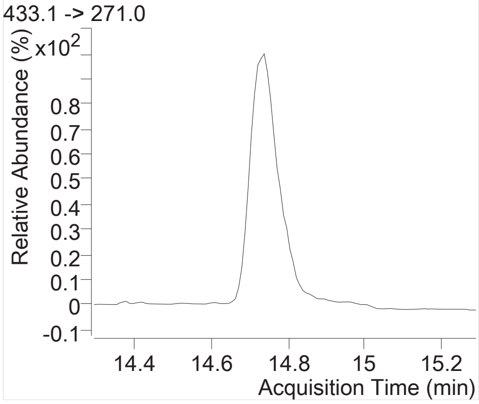

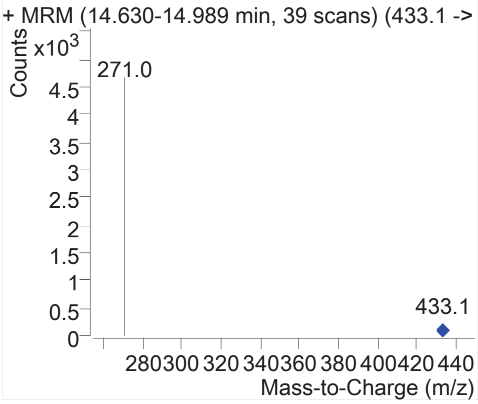

Pinoresinol

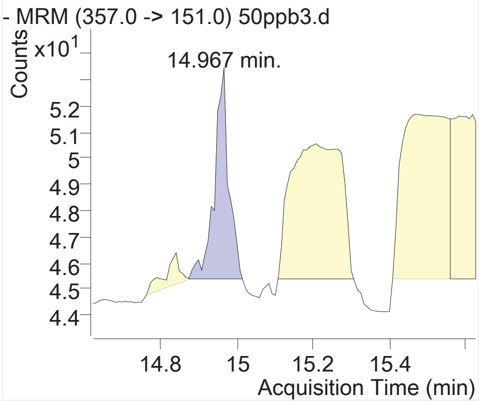

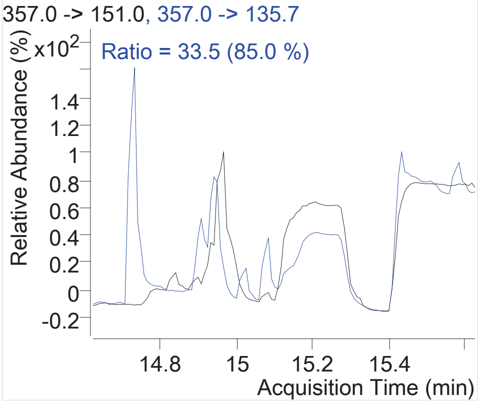

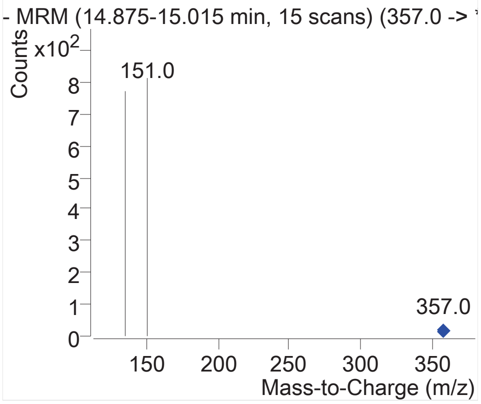

2-Hydroxycinnamic acid

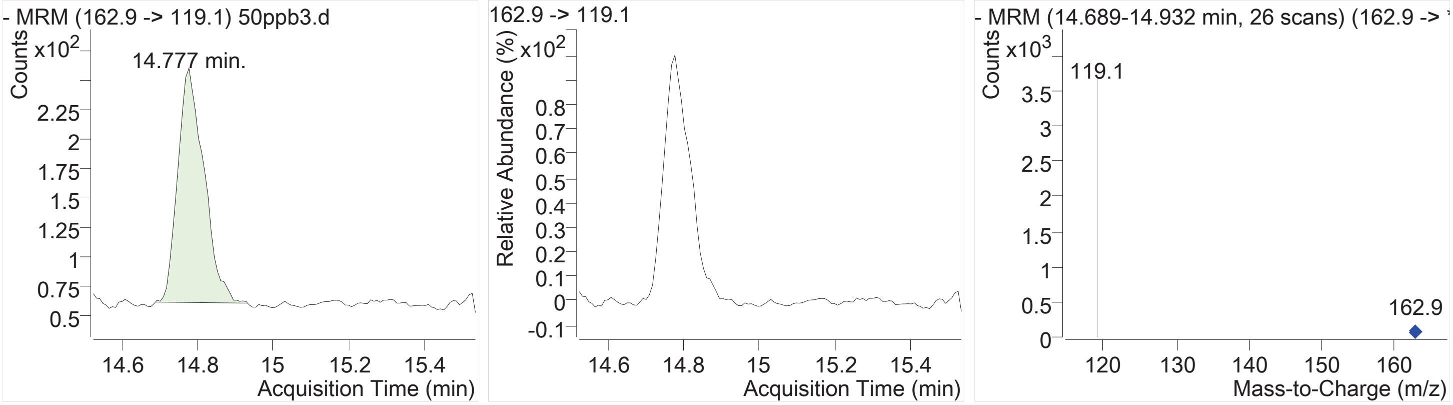

Eriodictyol

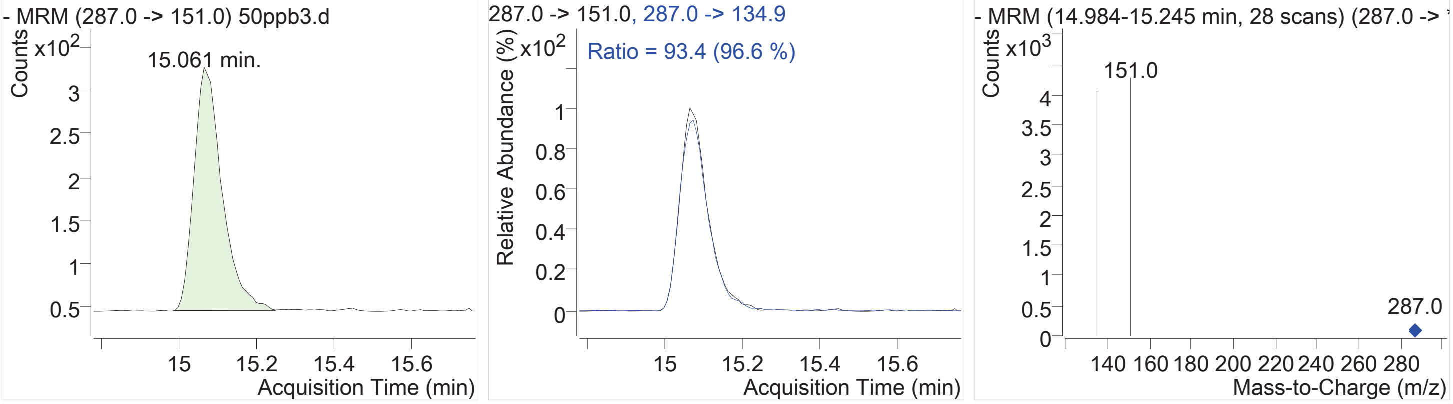

Quercetin

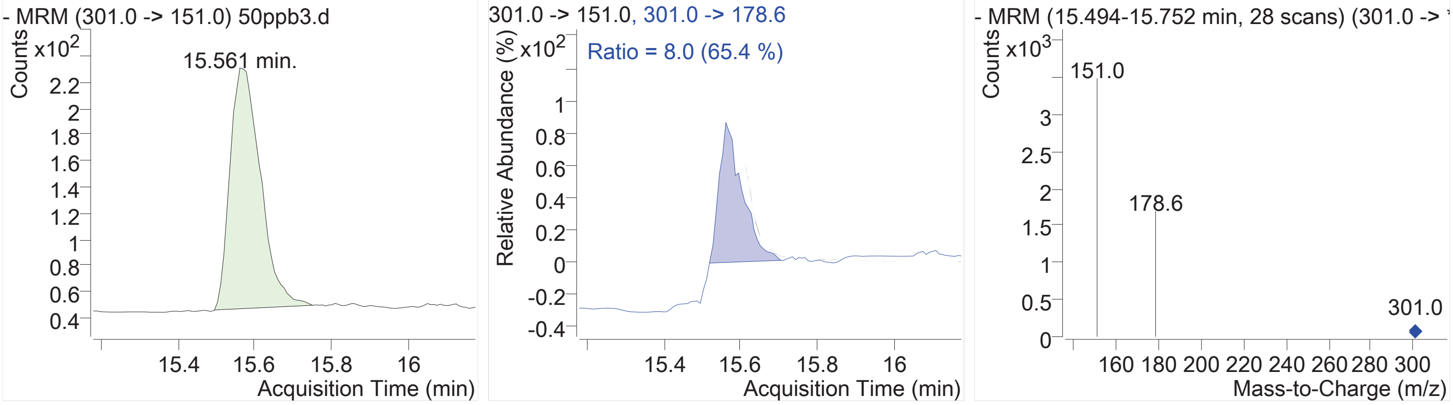

Luteolin

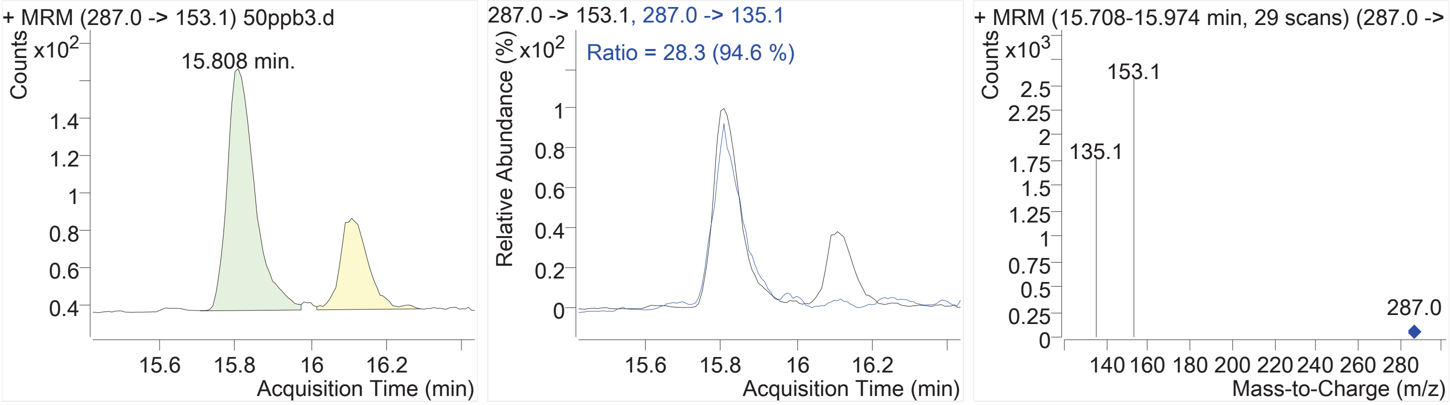

Kaempferol

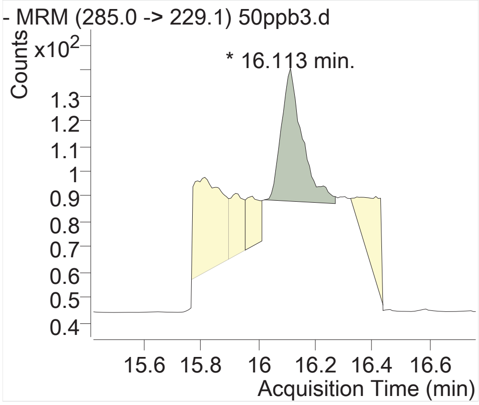

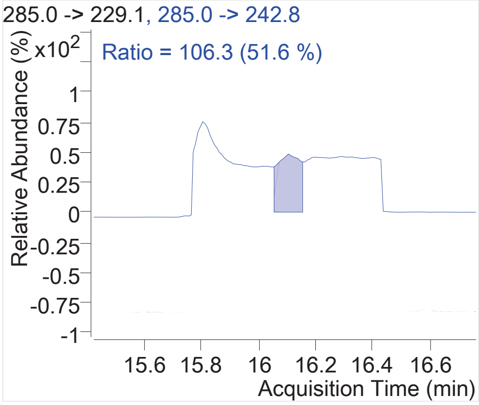

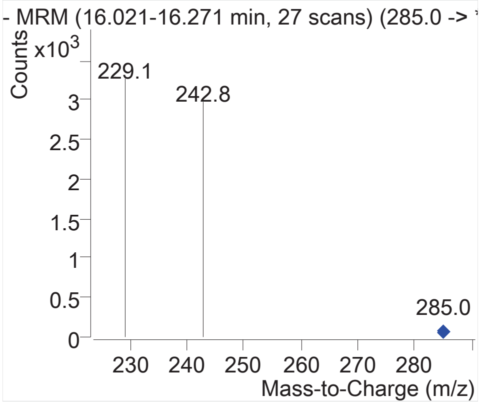

Apigenin

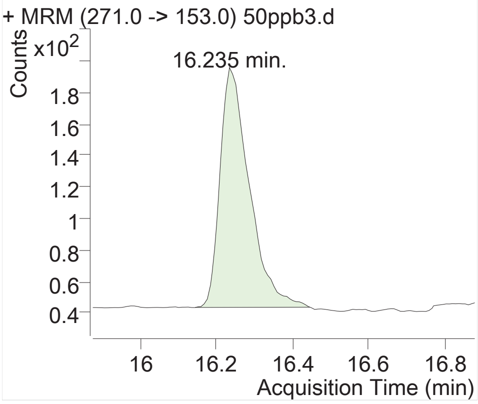

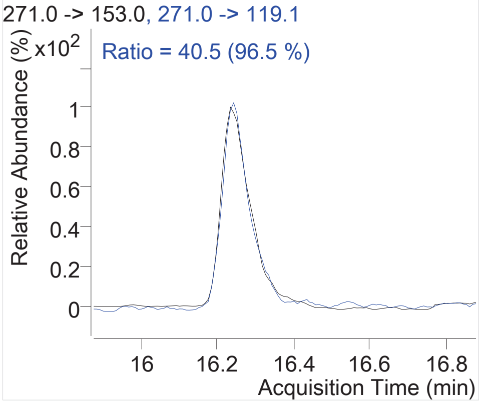

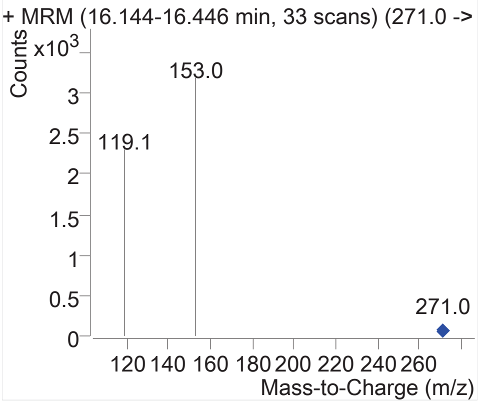

Quantitative Analysis Complete Report

Agilent Technologies

**Batch Path**	D:\MassHunter\Data\2022ekim\061022cengizhoca\QuantResults\071022.batch.bin		
**Analysis Time**	10/11/2022 1:33:26 PM	**Analyst Name**	Defam-PC\admin
**Report Time**	10/11/2022 1:34:08 PM	**Reporter Name**	admin
**Last Calib Update**	10/11/2022 1:33:17 PM	**Batch State**	Processed
**Quant Batch Version**	B.07.01	**Quant Report Version**	B.07.01

**Acq. Time**	10/6/2022 6:05:15 PM	**Data File**	100ppb1.d
**Sample Type**	Cal	**Sample Name**	100ppb1
**Dilution**	1	**Acq. Method**	FENOLIK_DMRM2021-31bilesen.m

Sample Chromatogram

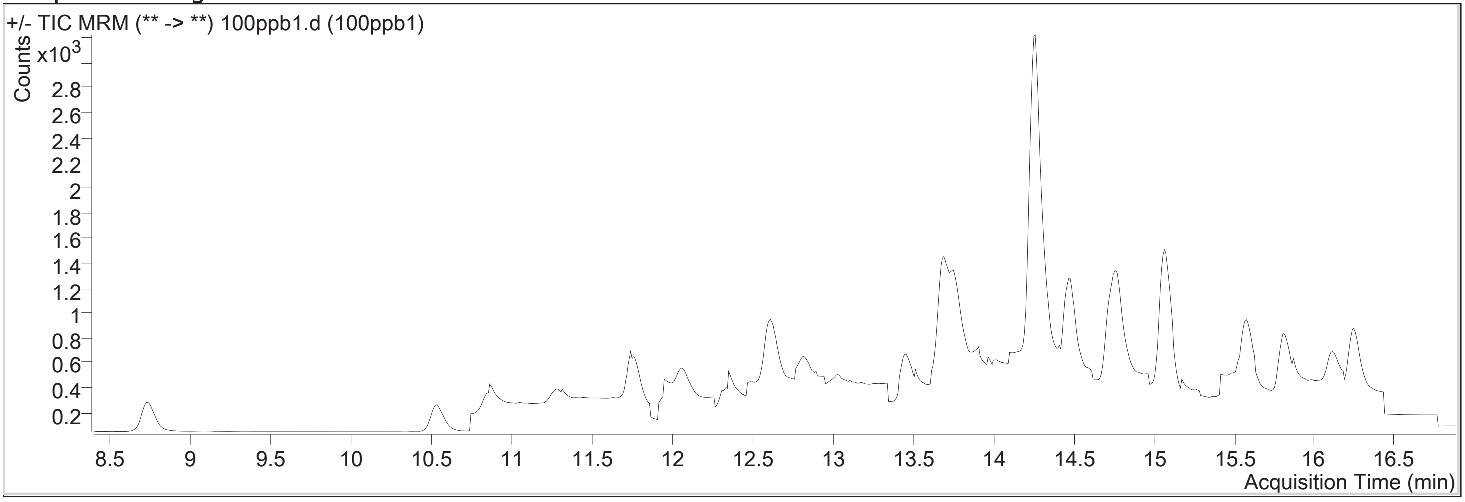

Compound	Transition	RT	Resp.	Final Conc	Units
Gallic acid	168.9 -> 125.0	8.733	1398	109.3299	ng/ml
Protocatechuic acid	152.9 -> 108.9	10.534	1245	108.6862	ng/ml
Pyrocatechol	109.0 -> 52.9	10.805	36	100.4178	ng/ml
3,4-Dihydroxyphenylacetic acid	167.0 -> 123.0	10.860	909	105.7820	ng/ml
(+)-Catechin	289.0 -> 245.0	11.286	473	115.7913	ng/ml
2,5-Dihydroxybenzoic acid	152.9 -> 109.0	11.954	674	107.1222	ng/ml
Chlorogenic acid	355.0 -> 163.0	11.768	2439	120.2845	ng/ml
3-Hydroxybenzoic acid	137.0 -> 93.0	12.828	482	105.1845	ng/ml
4-Hydroxybenzoic acid	136.9 -> 93.1	12.064	1269	101.3861	ng/ml
(-)-Epicatechin	291.0 -> 139.1	12.343	743	96.7673	ng/ml
Caffeic acid	179.0 -> 135.0	12.616	2941	97.9899	ng/ml
Syringic acid	196.9 -> 181.9	12.757	70	100.8741	ng/ml
Vanillin	151.0 -> 136.0	13.037	388	95.4356	ng/ml
Verbascoside	623.0 -> 160.8	13.459	1102	96.8839	ng/ml
Taxifolin	303.0 -> 285.1	13.695	3803	104.9985	ng/ml
p-Coumaric acid	162.9 -> 119.0	13.775	3027	108.8030	ng/ml
Sinapic acid	222.9 -> 207.9	13.873	344	112.7960	ng/ml
Ferulic acid	193.0 -> 134.0	13.916	617	118.6712	ng/ml
Luteolin 7-glucoside	447.1 -> 285.0	14.255	12136	99.6874	ng/ml
Hesperidin	611.1 -> 303.0	14.302	823	104.0248	ng/ml
Hyperoside	465.1 -> 303.1	14.479	1959	103.0652	ng/ml
Rosmarinic acid	359.0 -> 160.9	14.473	1331	105.1277	ng/ml
Apigenin 7-glucoside	433.1 -> 271.0	14.739	3368	98.8548	ng/ml
Pinoresinol	357.0 -> 151.0	14.967	29	87.8772	ng/ml
2-Hydroxycinnamic acid	162.9 -> 119.1	14.777	2320	107.5845	ng/ml
Eriodictyol	287.0 -> 151.0	15.070	3048	101.7680	ng/ml
Quercetin	301.0 -> 151.0	15.578	2412	98.2920	ng/ml
Luteolin	287.0 -> 153.1	15.816	1619	97.2974	ng/ml
Kaempferol	285.0 -> 229.1	16.121	573	109.8097	ng/ml

Compound	Transition	RT	Resp.	Final Conc	Units
Apigenin	271.0 -> 153.0	16.251	1895	96.6902	ng/ml

Gallic acid

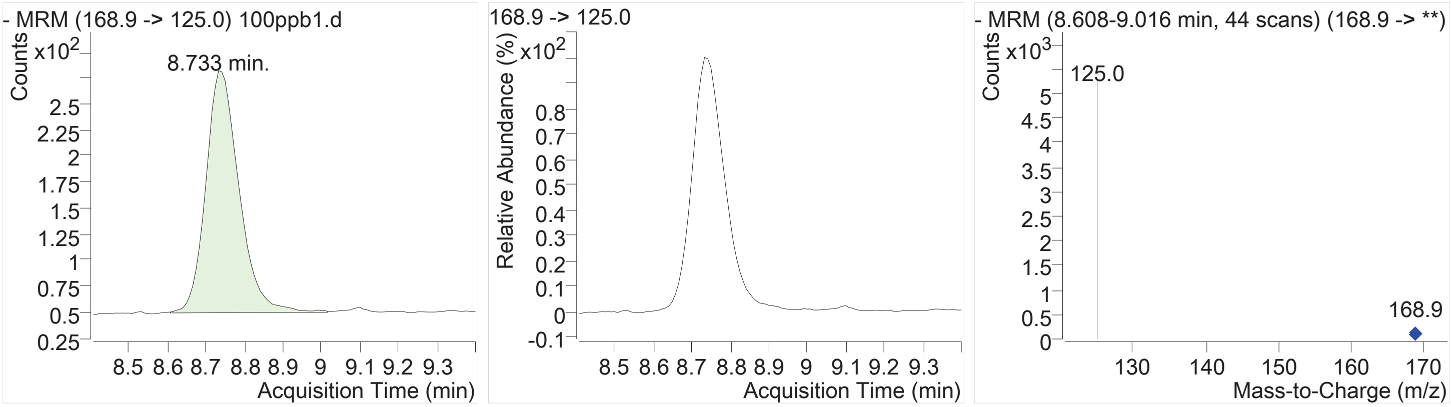

Protocatechuic acid

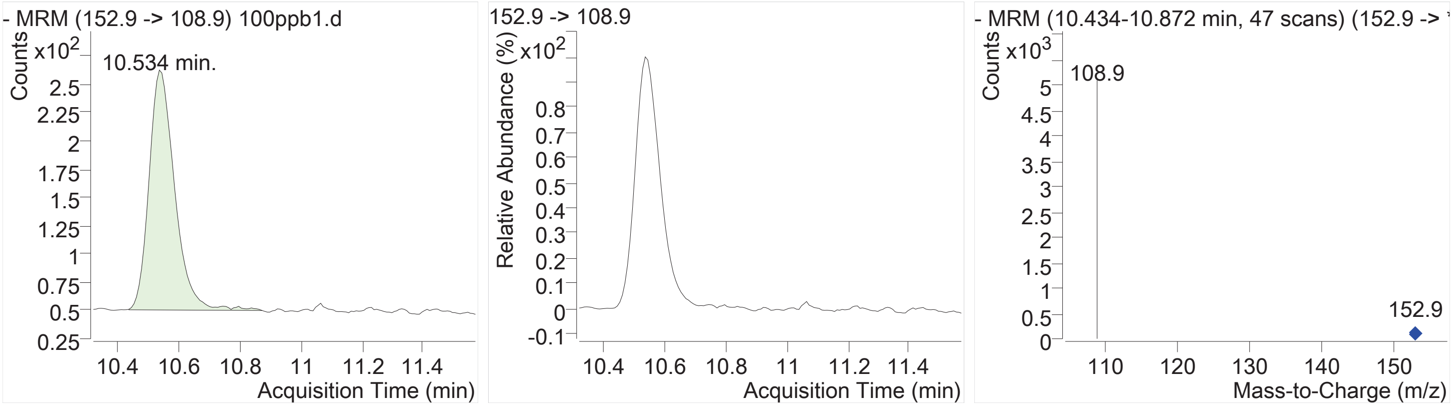

Pyrocatechol

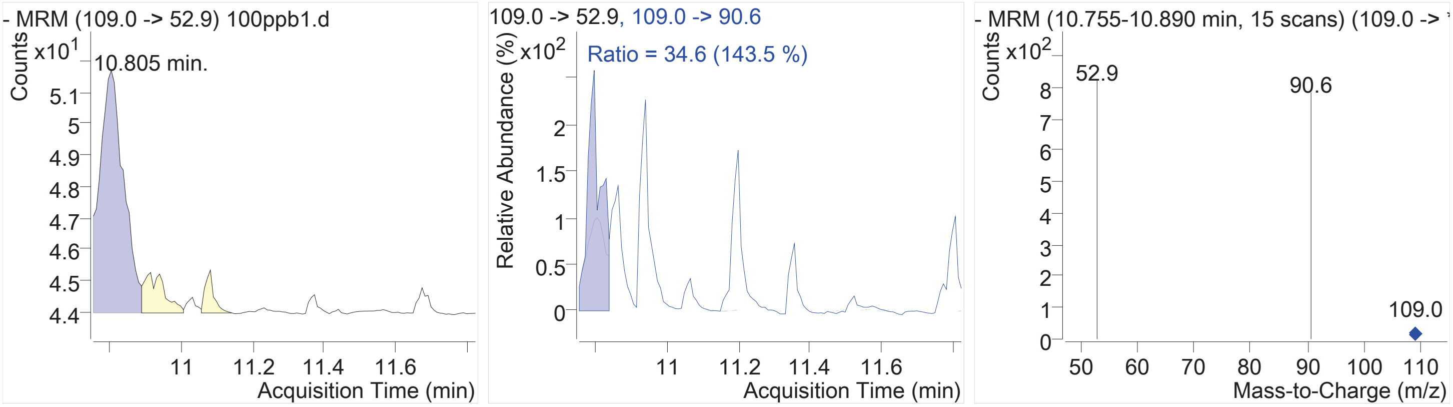

3,4-Dihydroxyphenylacetic acid

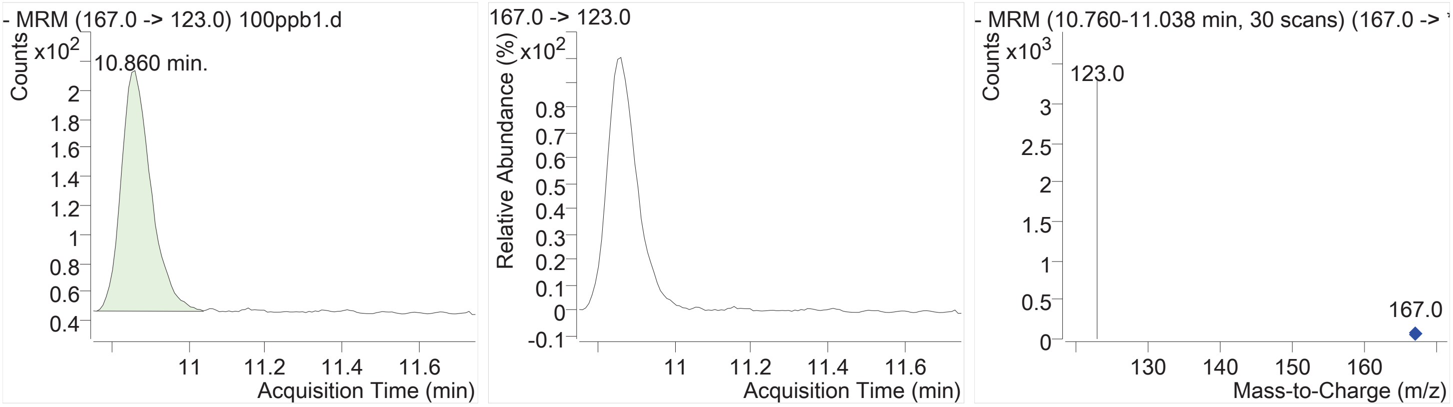

(+)-Catechin

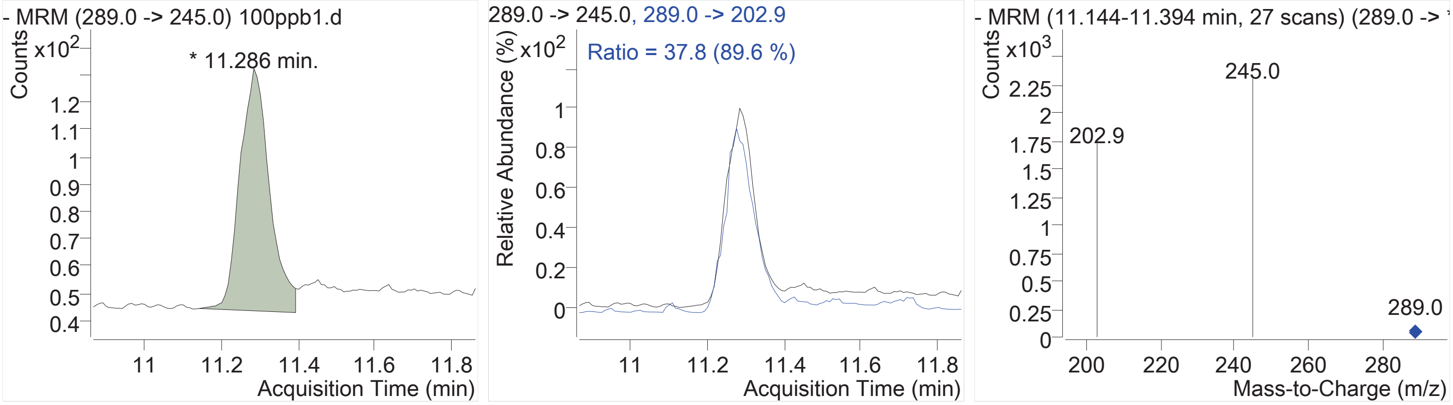

2,5-Dihydroxybenzoic acid

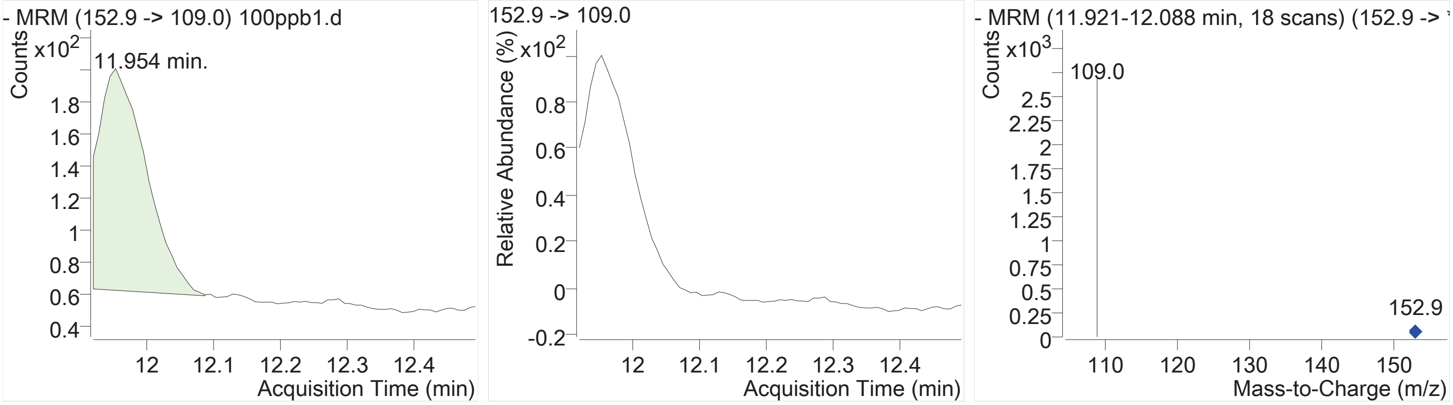

Chlorogenic acid

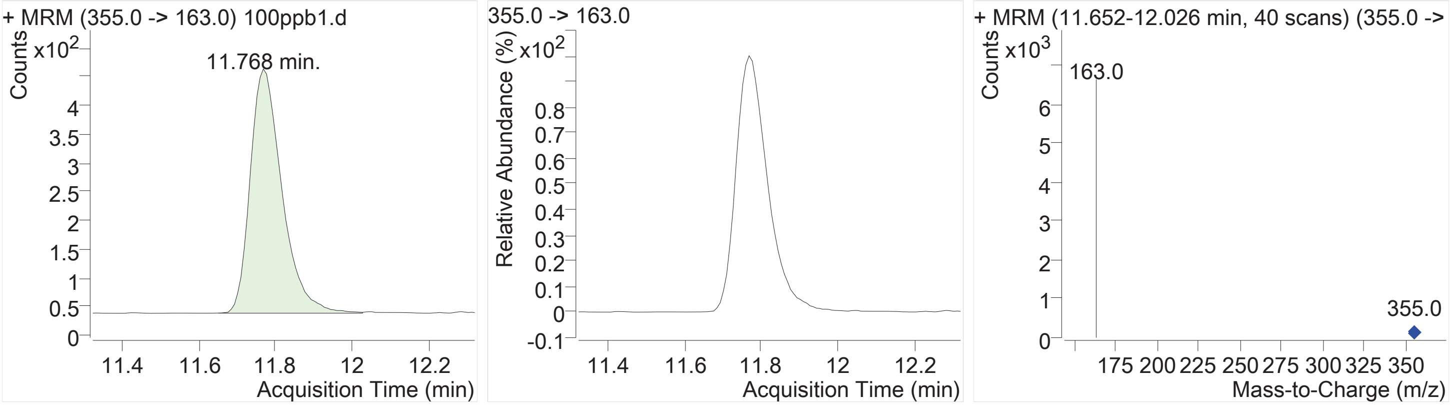

3-Hydroxybenzoic acid

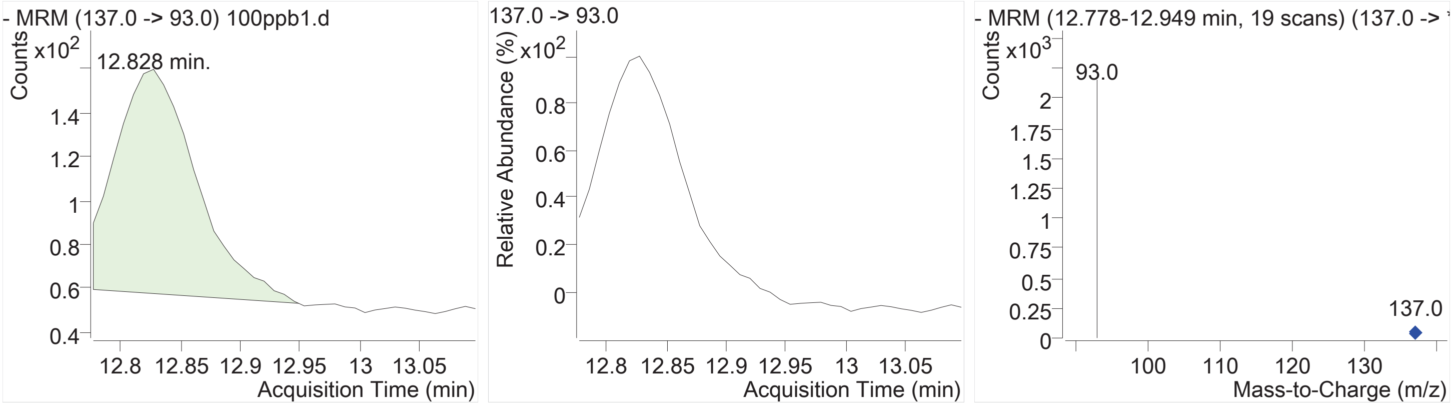

4-Hydroxybenzoic acid

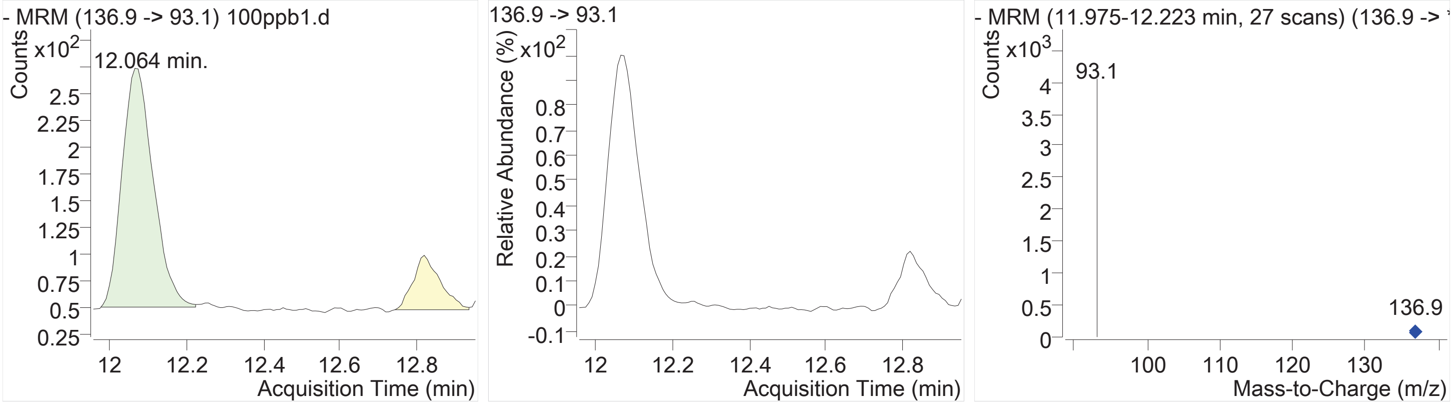

(-)-Epicatechin

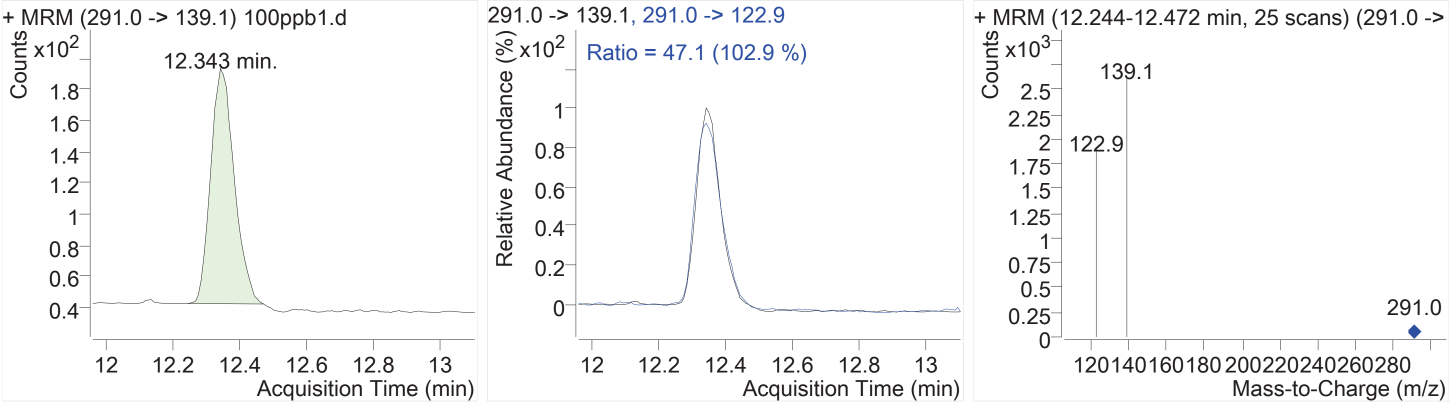

Caffeic acid

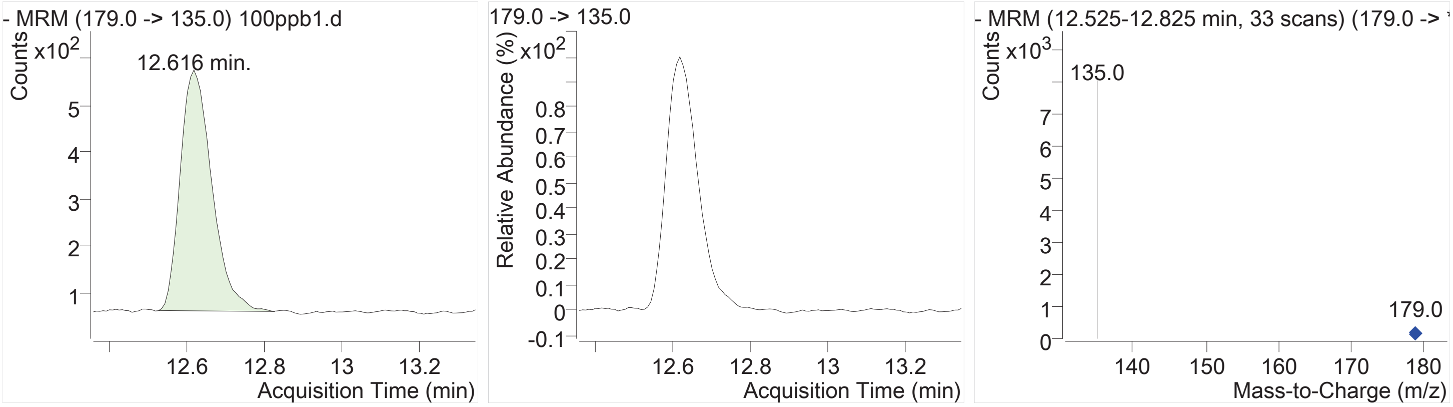

Syringic acid

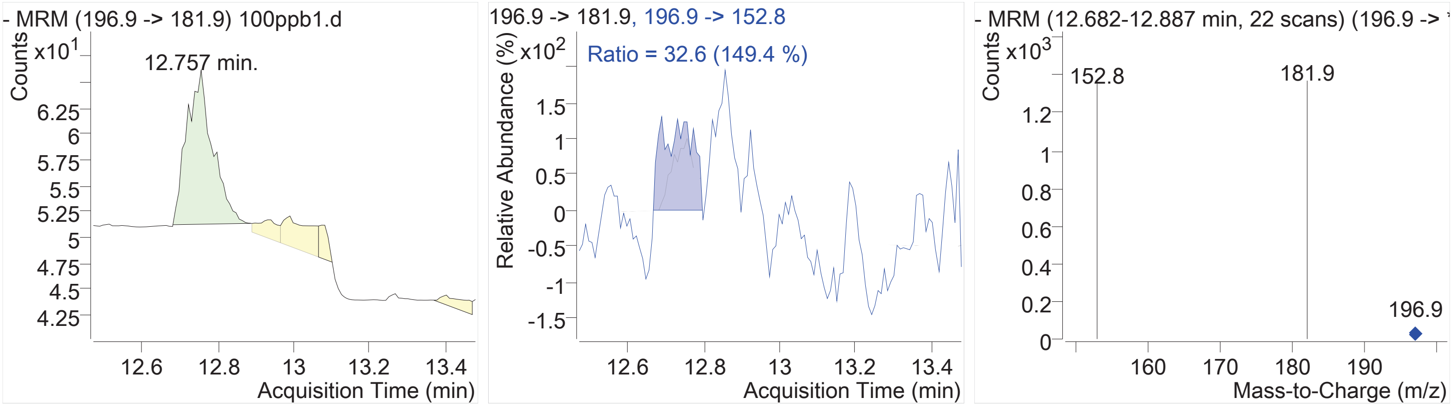

Vanillin

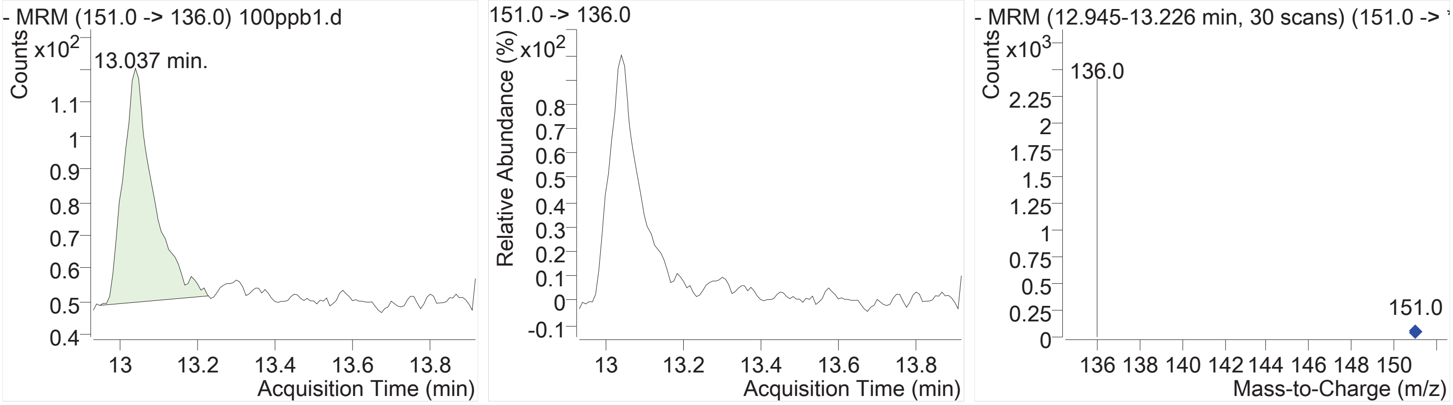

Verbascoside

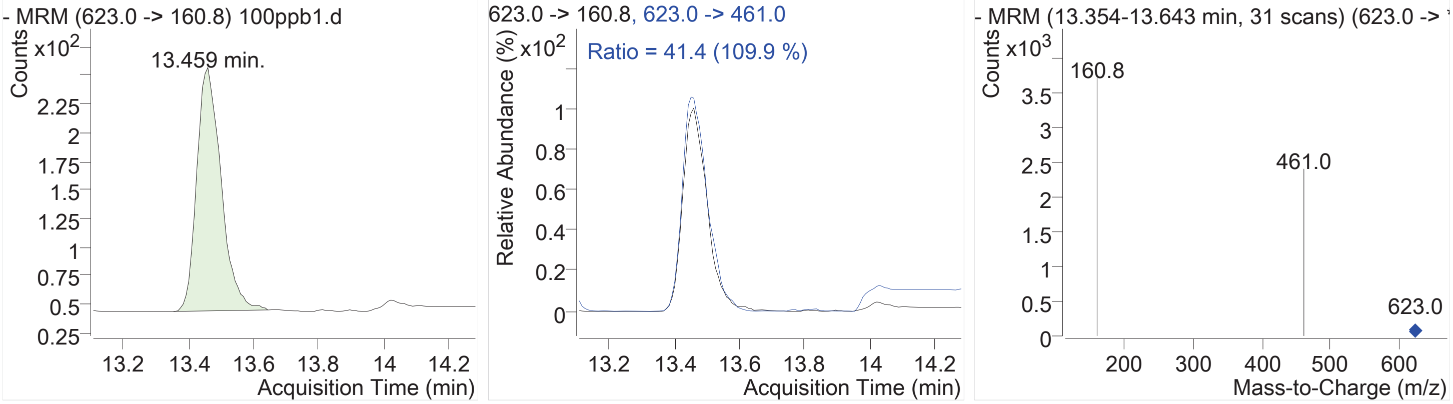

Taxifolin

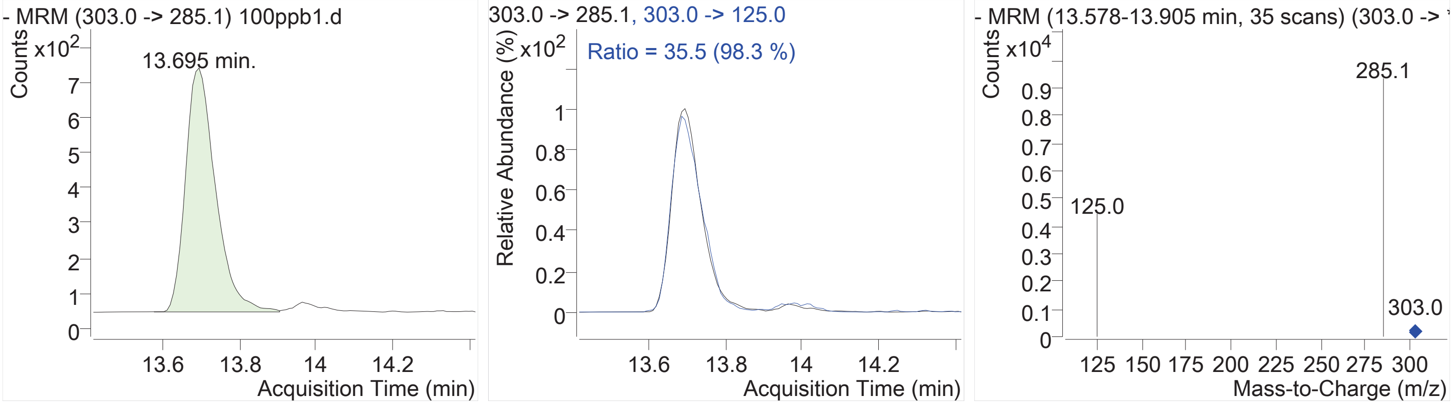

p-Coumaric acid

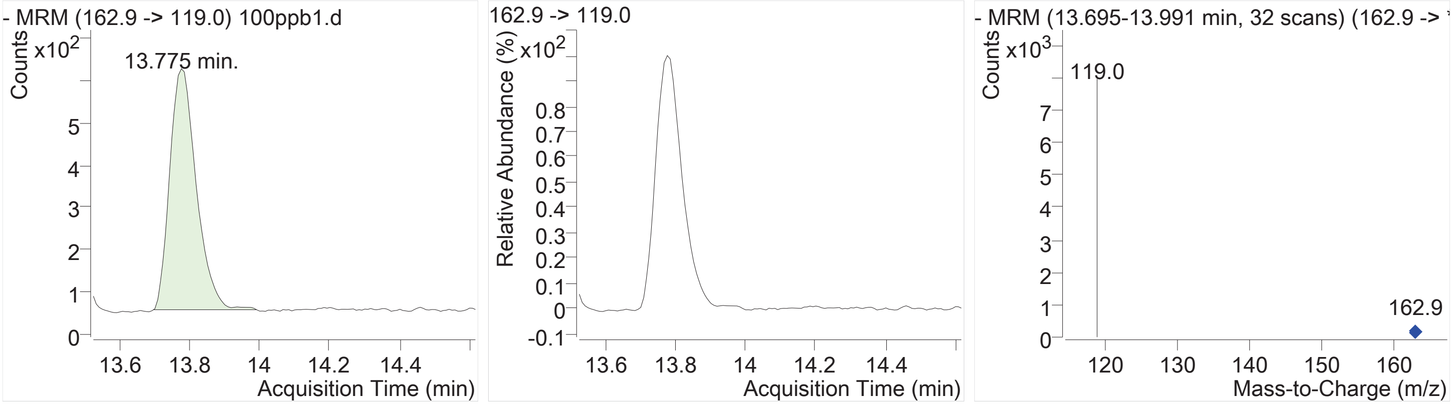

Sinapic acid

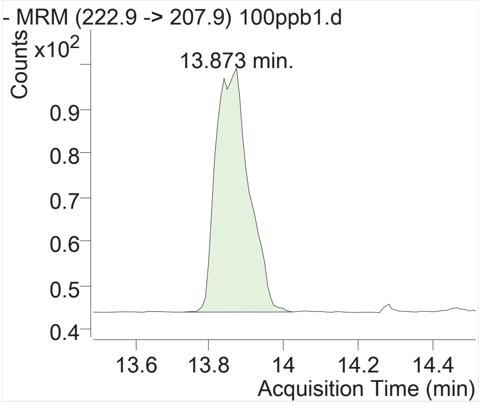

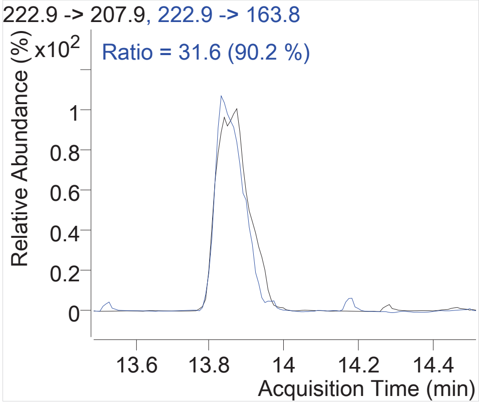

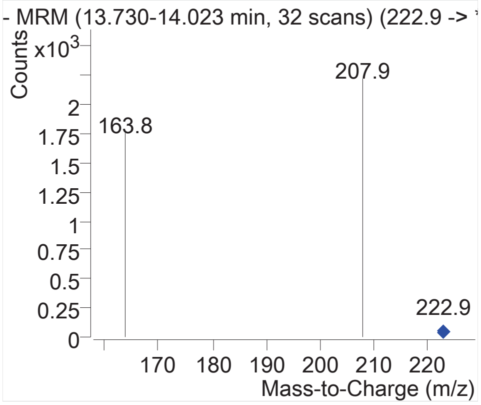

Ferulic acid

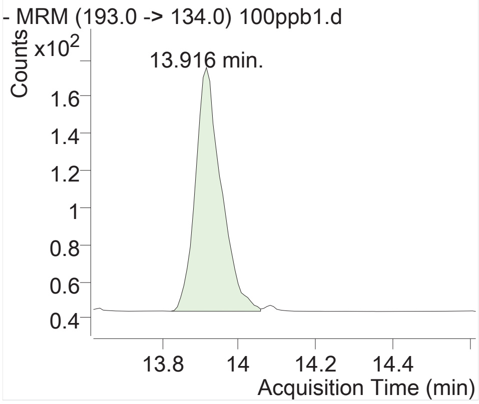

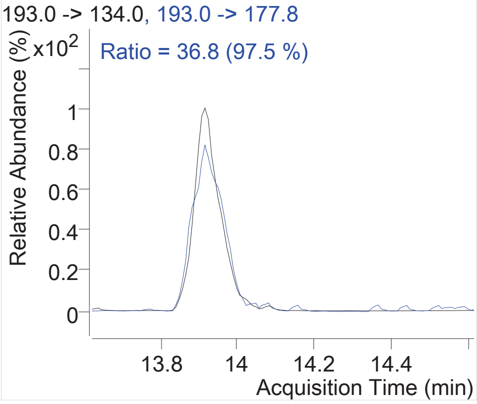

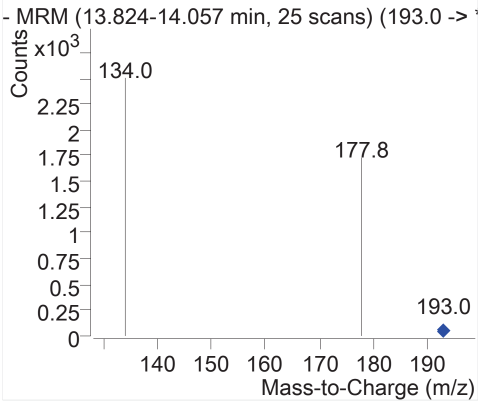

Luteolin 7-glucoside

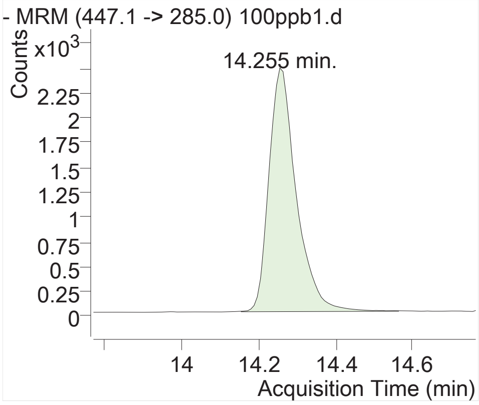

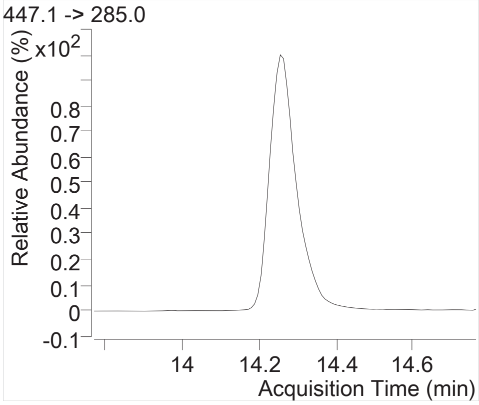

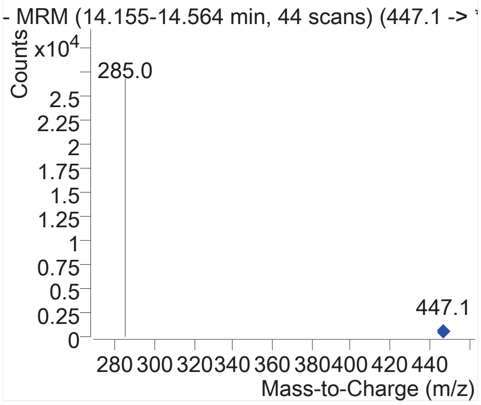

Hesperidin

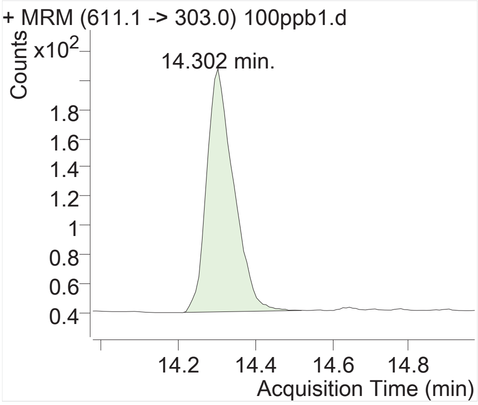

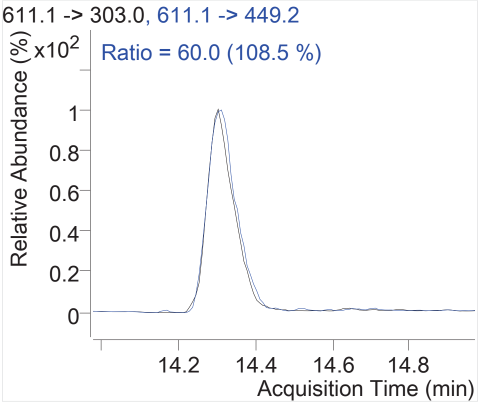

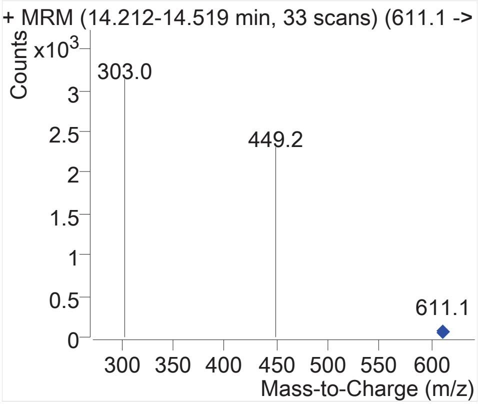

Hyperoside

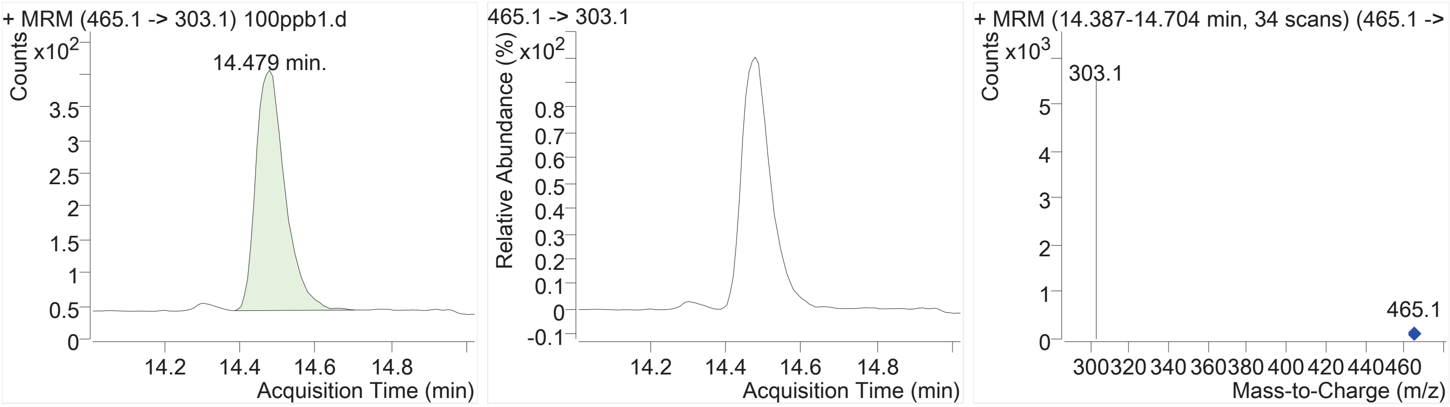

Rosmarinic acid

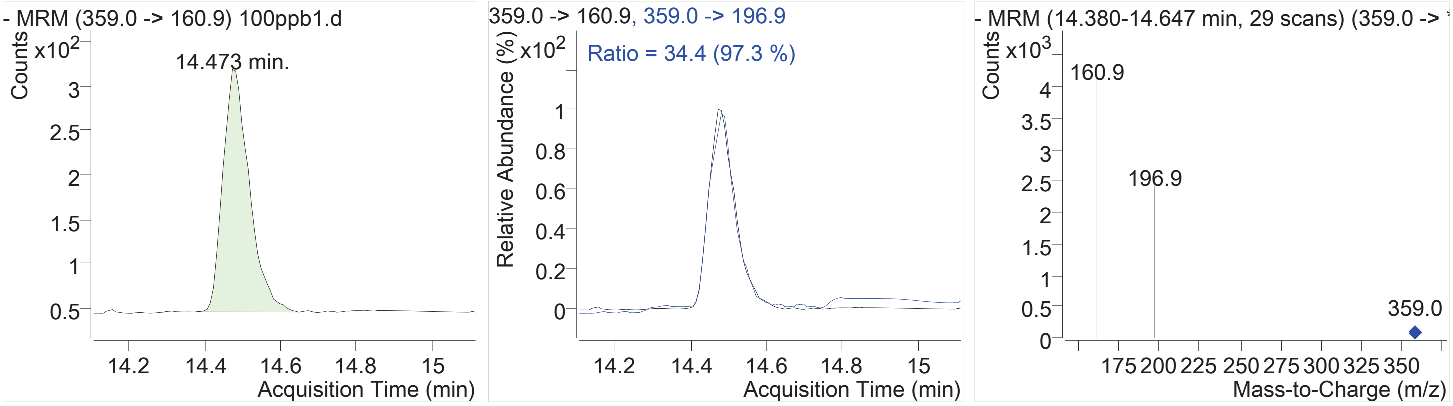

Apigenin 7-glucoside

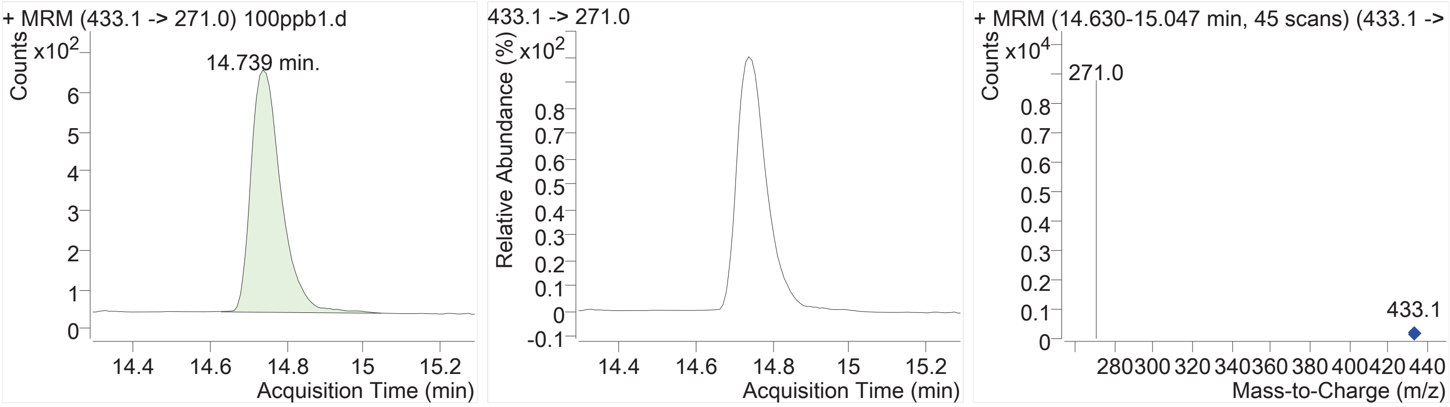

Pinoresinol

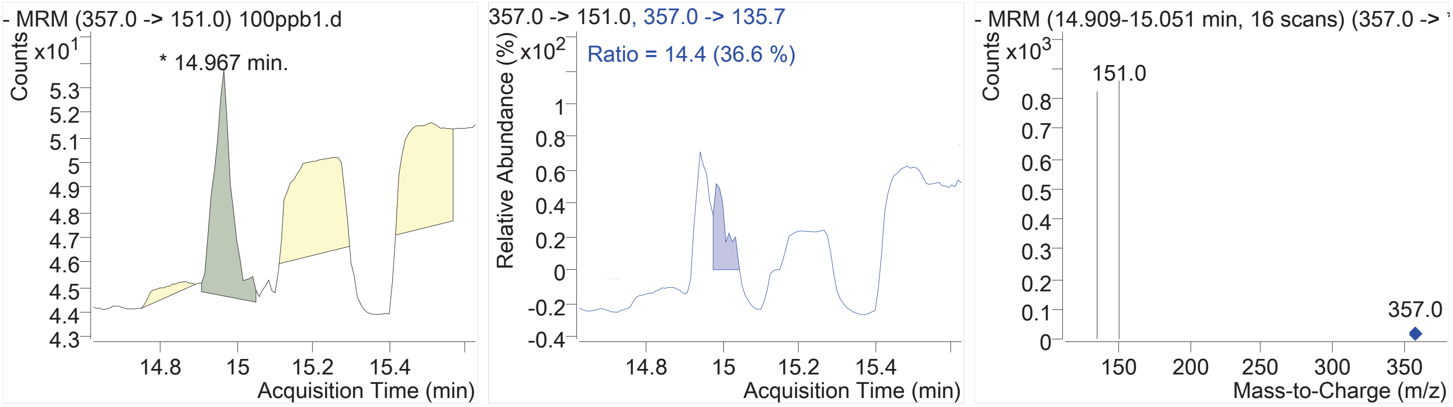

2-Hydroxycinnamic acid

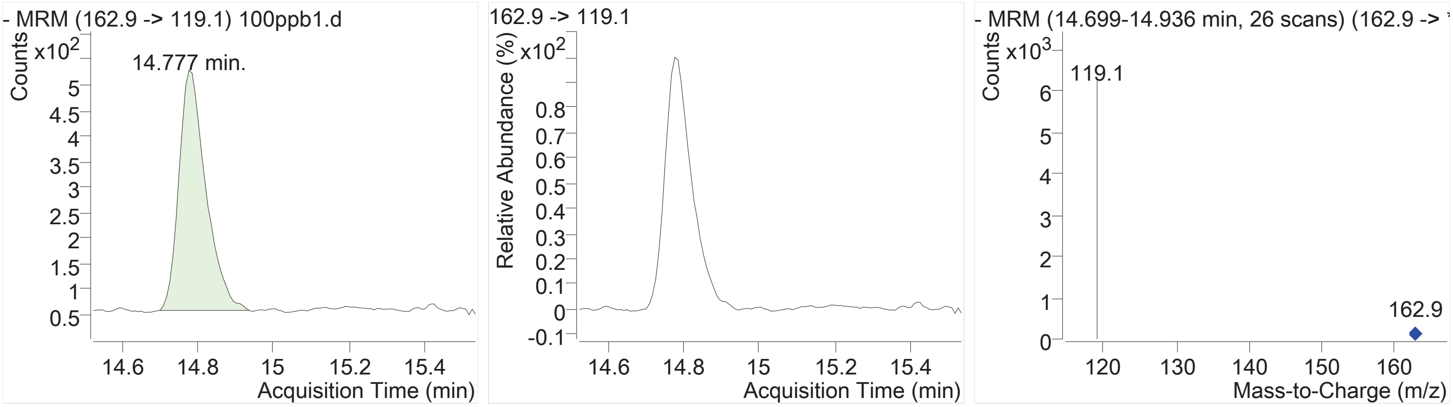

Eriodictyol

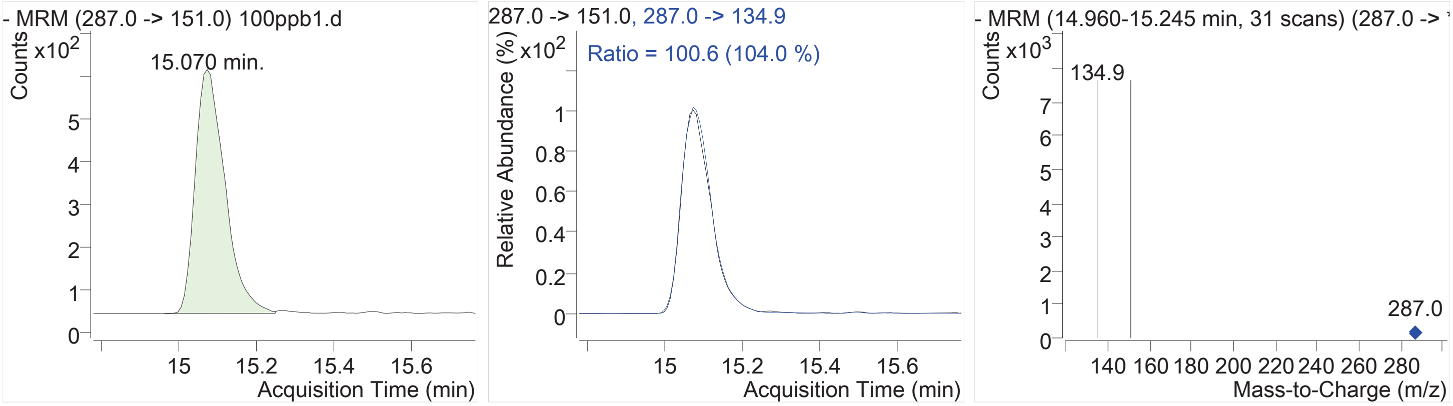

Quercetin

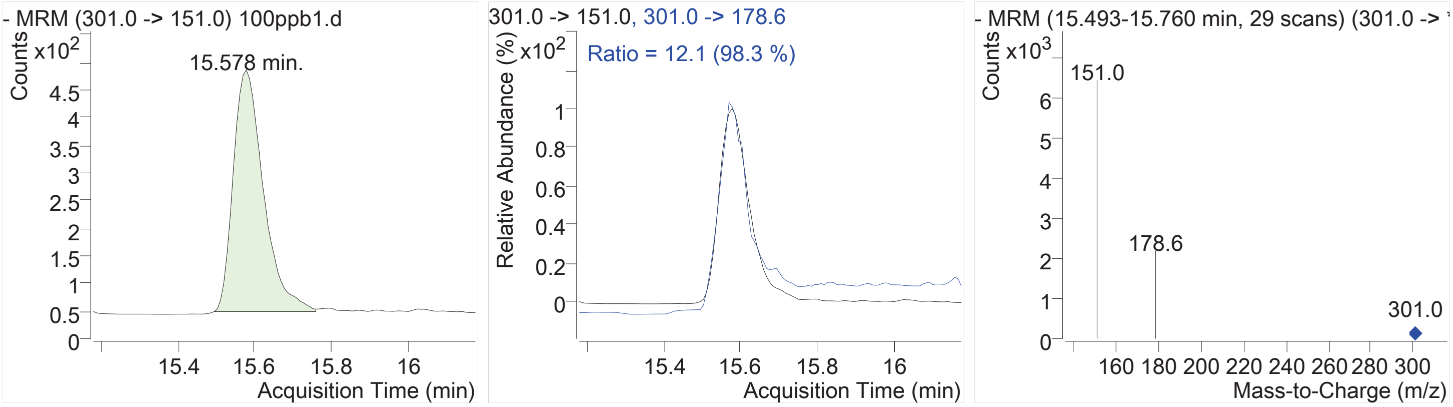

Luteolin

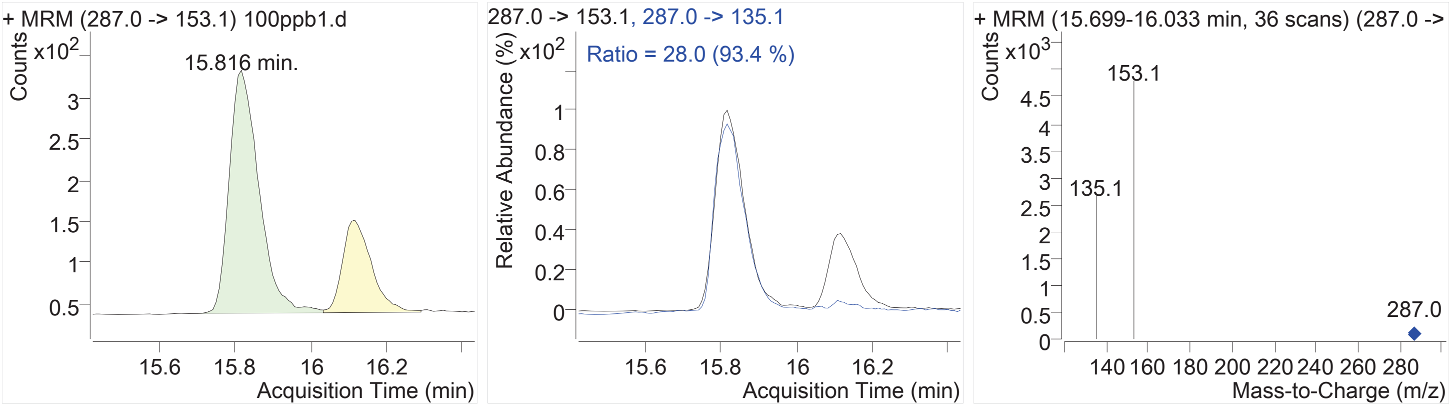

Kaempferol

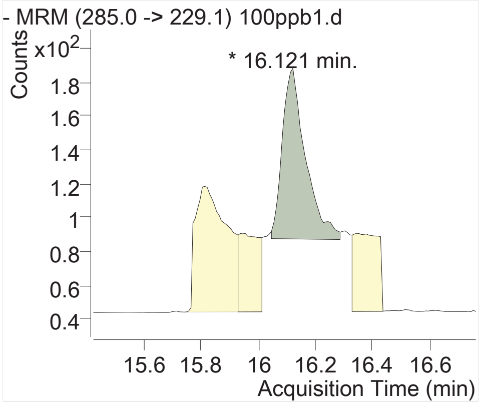

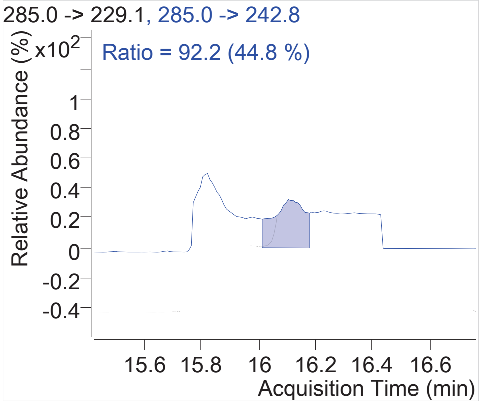

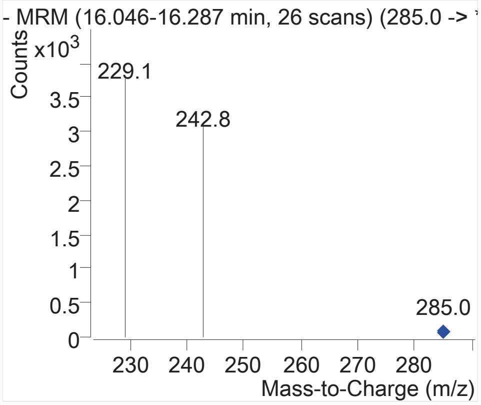

Apigenin

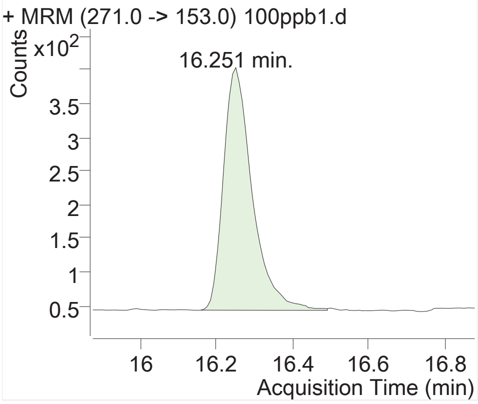

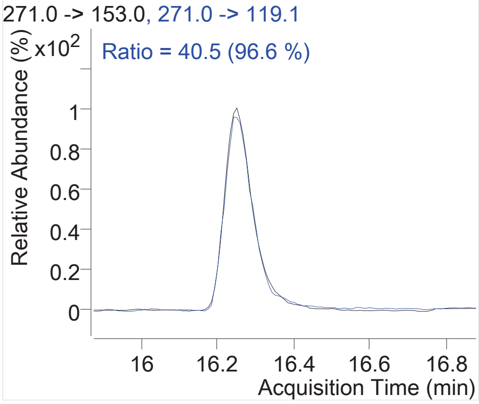

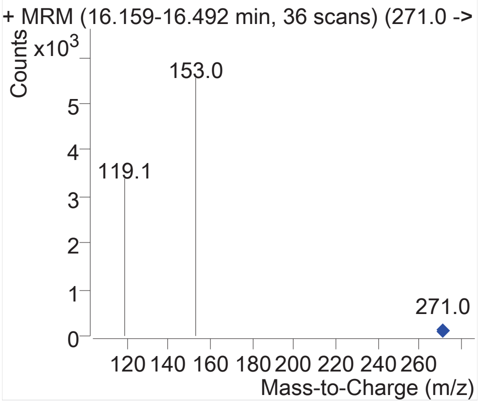

Quantitative Analysis Complete Report

**Batch Path**	D:\MassHunter\Data\2022ekim\061022cengizhoca\QuantResults\071022.batch.bin		
**Analysis Time**	10/11/2022 1:33:26 PM	**Analyst Name**	Defam-PC\admin
**Report Time**	10/11/2022 1:34:11 PM	**Reporter Name**	admin
**Last Calib Update**	10/11/2022 1:33:17 PM	**Batch State**	Processed
**Quant Batch Version**	B.07.01	**Quant Report Version**	B.07.01

**Acq. Time**	10/6/2022 6:26:55 PM	**Data File**	100ppb2.d
**Sample Type**	Cal	**Sample Name**	100ppb2
**Dilution**	1	**Acq. Method**	FENOLIK_DMRM2021-31bilesen.m

Sample Chromatogram

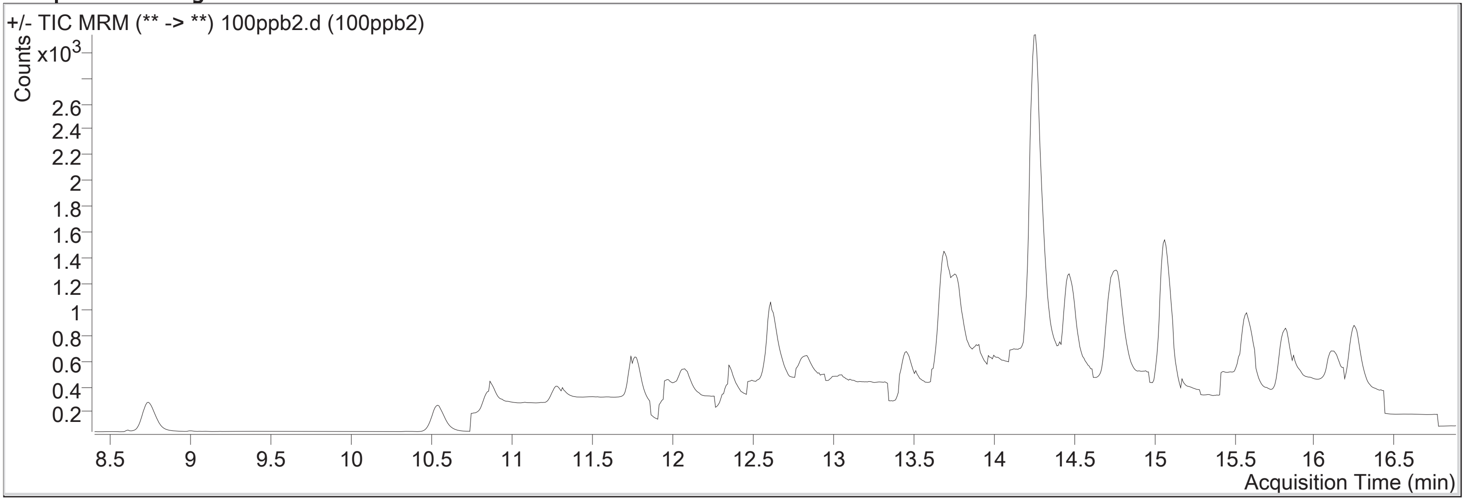

Compound	Transition	RT	Resp.	Final Conc	Units
Gallic acid	168.9 -> 125.0	8.741	1358	105.5540	ng/ml
Protocatechuic acid	152.9 -> 108.9	10.543	1198	104.1706	ng/ml
Pyrocatechol	109.0 -> 52.9	10.797	44	128.2957	ng/ml
3,4-Dihydroxyphenylacetic acid	167.0 -> 123.0	10.860	926	107.9185	ng/ml
(+)-Catechin	289.0 -> 245.0	11.277	506	125.8558	ng/ml
2,5-Dihydroxybenzoic acid	152.9 -> 109.0	11.971	722	114.6293	ng/ml
Chlorogenic acid	355.0 -> 163.0	11.777	2273	112.5017	ng/ml
3-Hydroxybenzoic acid	137.0 -> 93.0	12.828	430	95.5195	ng/ml
4-Hydroxybenzoic acid	136.9 -> 93.1	12.081	1218	96.6080	ng/ml
(-)-Epicatechin	291.0 -> 139.1	12.351	794	103.1343	ng/ml
Caffeic acid	179.0 -> 135.0	12.616	3185	108.5444	ng/ml
Syringic acid	196.9 -> 181.9	12.740	86	122.5376	ng/ml
Vanillin	151.0 -> 136.0	13.045	379	92.6379	ng/ml
Verbascoside	623.0 -> 160.8	13.459	1133	100.0341	ng/ml
Taxifolin	303.0 -> 285.1	13.695	3529	94.0705	ng/ml
p-Coumaric acid	162.9 -> 119.0	13.784	3079	110.8228	ng/ml
Sinapic acid	222.9 -> 207.9	13.848	323	106.1399	ng/ml
Ferulic acid	193.0 -> 134.0	13.925	648	124.5307	ng/ml
Luteolin 7-glucoside	447.1 -> 285.0	14.255	11834	96.3644	ng/ml
Hesperidin	611.1 -> 303.0	14.302	877	110.6730	ng/ml
Hyperoside	465.1 -> 303.1	14.470	1966	103.4669	ng/ml
Rosmarinic acid	359.0 -> 160.9	14.473	1299	102.5878	ng/ml
Apigenin 7-glucoside	433.1 -> 271.0	14.739	3542	104.0308	ng/ml
Pinoresinol	357.0 -> 151.0	14.950	28	83.4822	ng/ml
2-Hydroxycinnamic acid	162.9 -> 119.1	14.786	2217	102.7210	ng/ml
Eriodictyol	287.0 -> 151.0	15.070	3082	103.0961	ng/ml
Quercetin	301.0 -> 151.0	15.578	2527	103.3176	ng/ml
Luteolin	287.0 -> 153.1	15.825	1760	106.2186	ng/ml
Kaempferol	285.0 -> 229.1	16.121	594	115.0103	ng/ml

Compound	Transition	RT	Resp.	Final Conc	Units
Apigenin	271.0 -> 153.0	16.260	1904	97.1300	ng/ml

Gallic acid

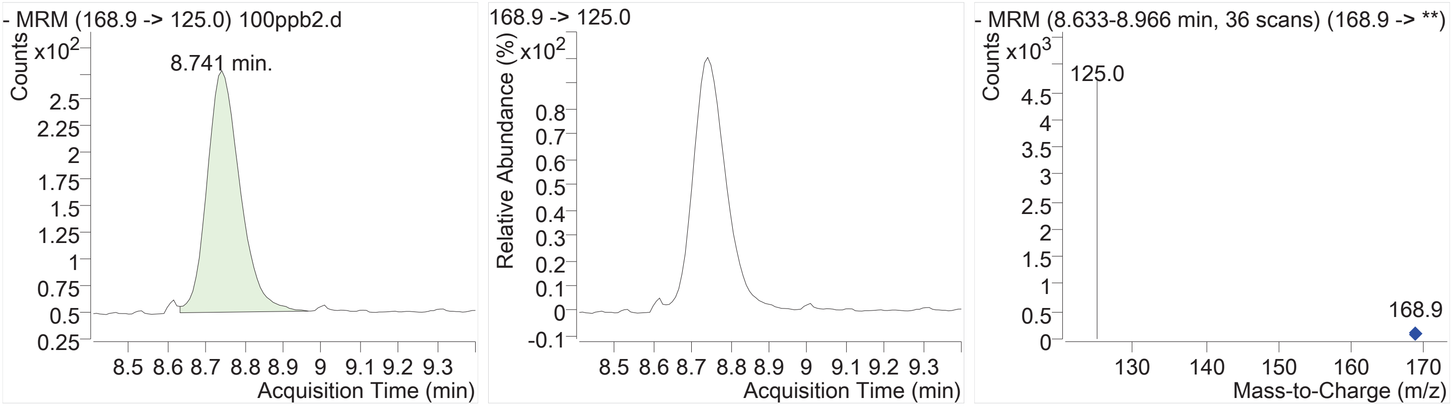

Protocatechuic acid

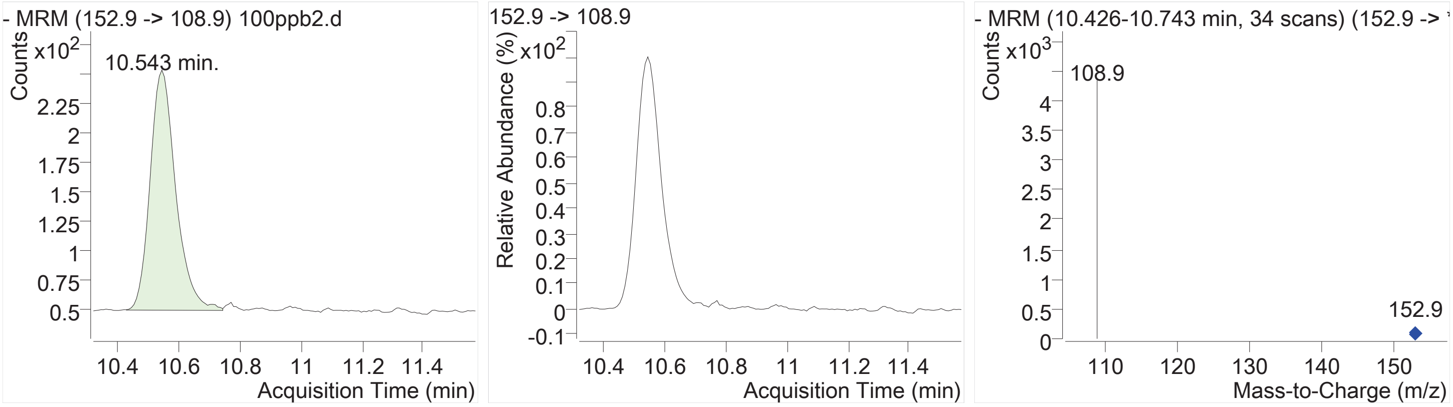

Pyrocatechol

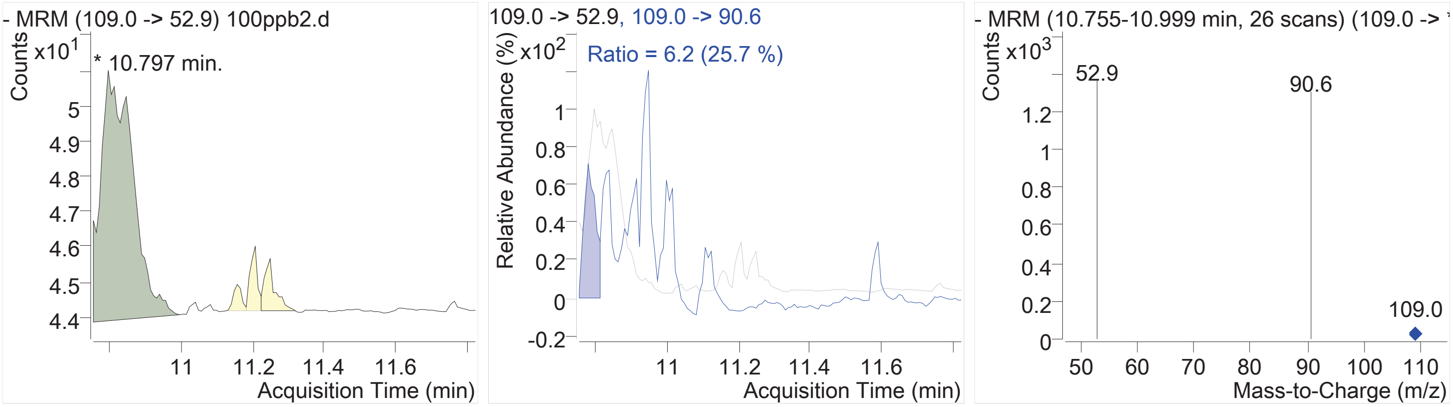

3,4-Dihydroxyphenylacetic acid

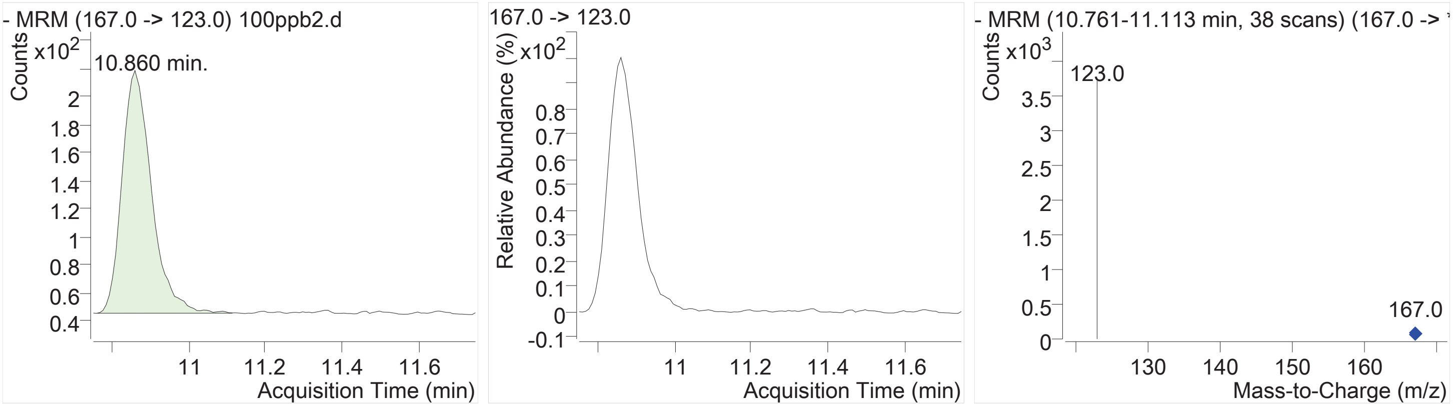

Agilent Technologies

(+)-Catechin

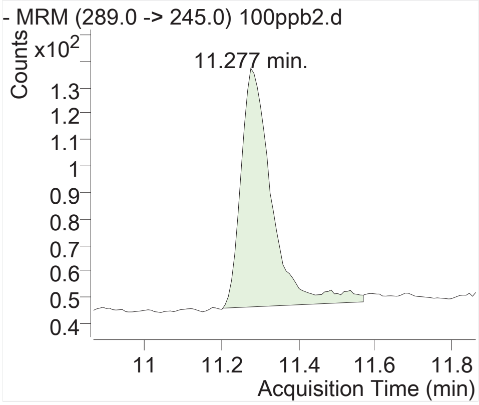

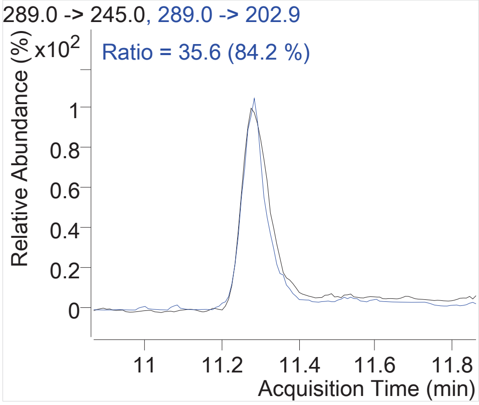

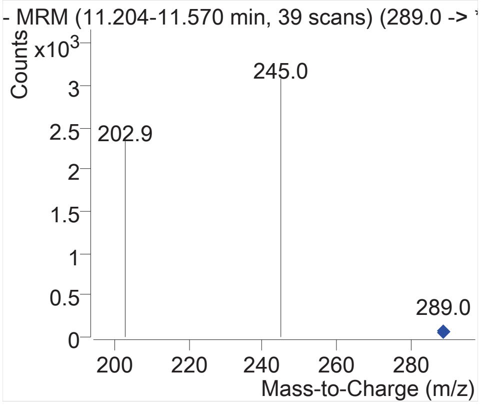

2,5-Dihydroxybenzoic acid

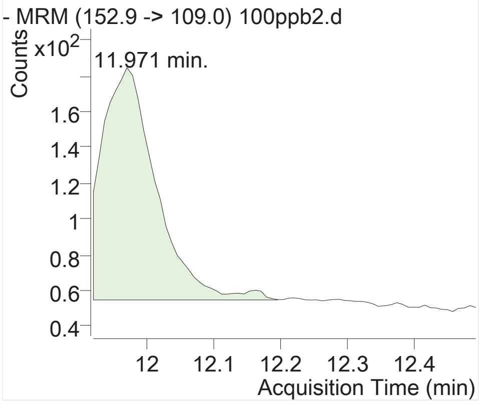

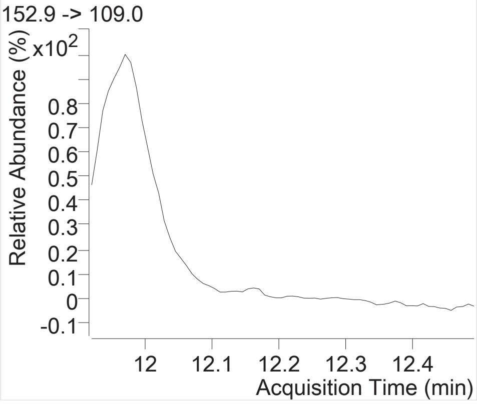

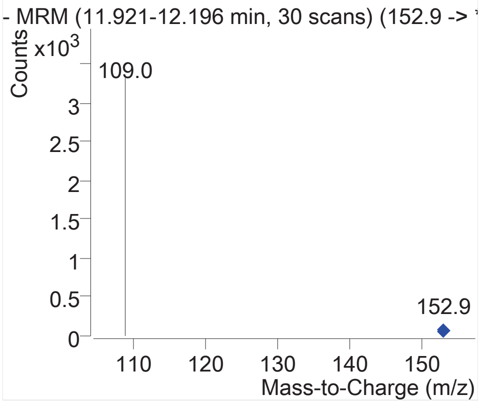

Chlorogenic acid

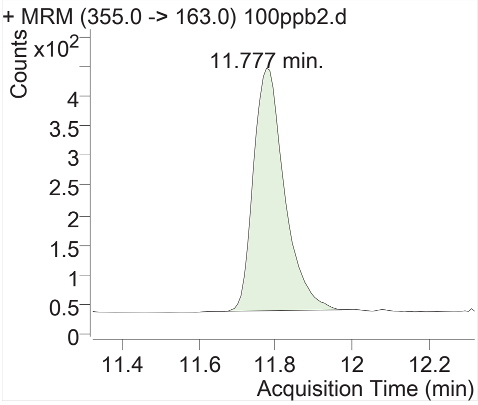

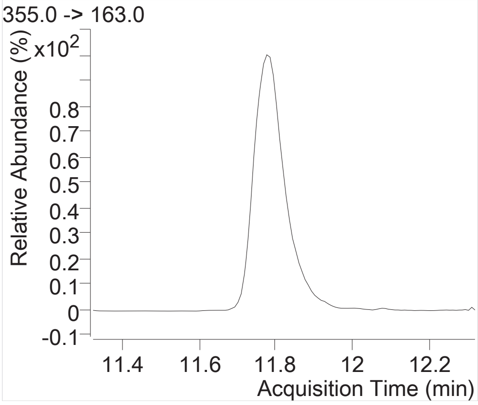

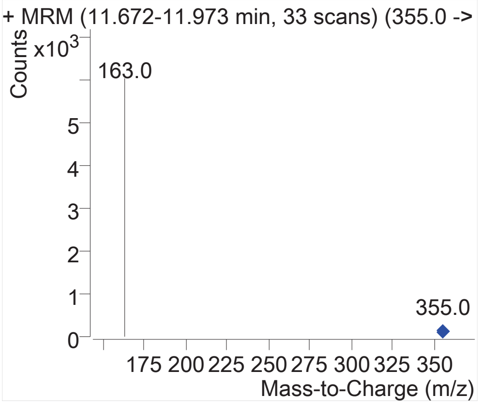

3-Hydroxybenzoic acid

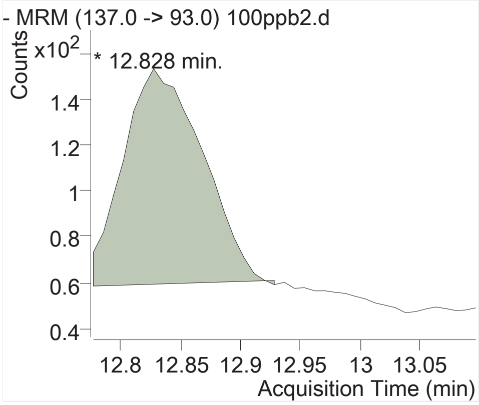

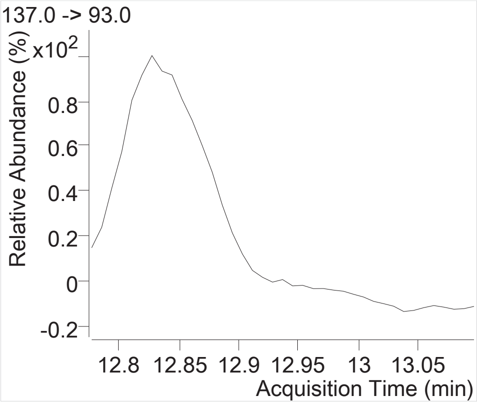

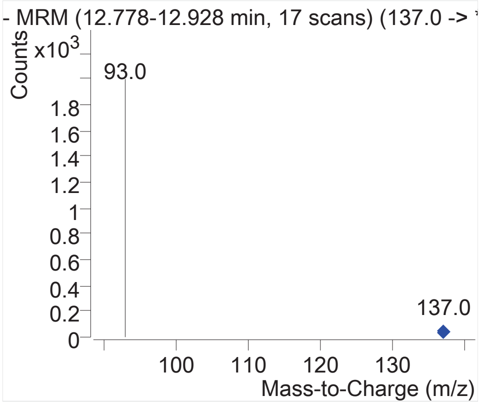

4-Hydroxybenzoic acid

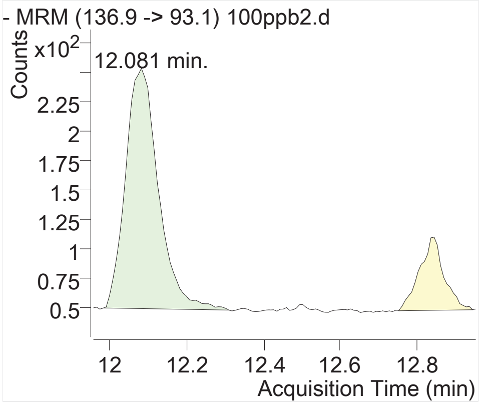

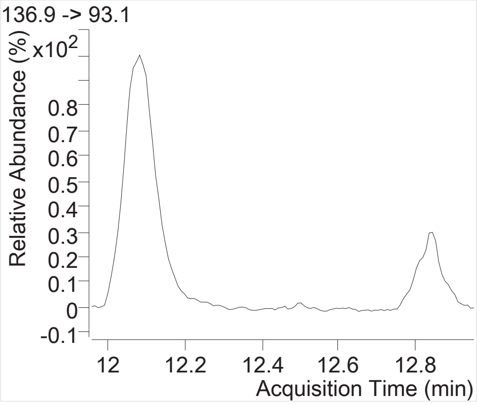

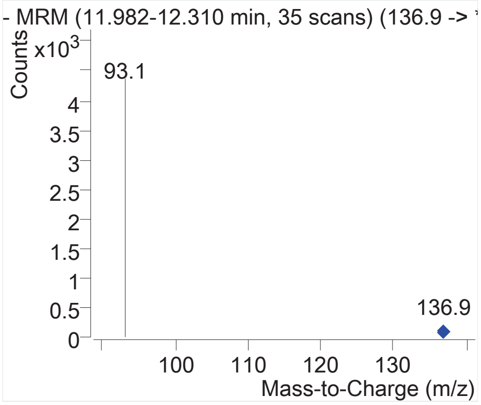

(-)-Epicatechin

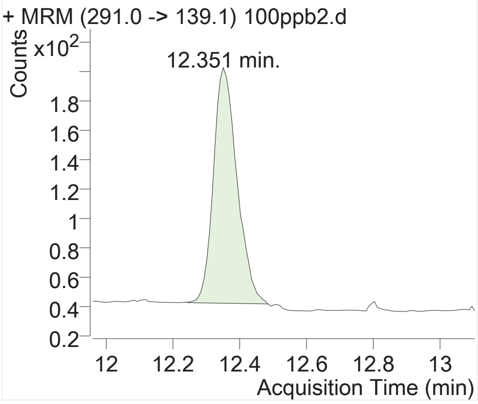

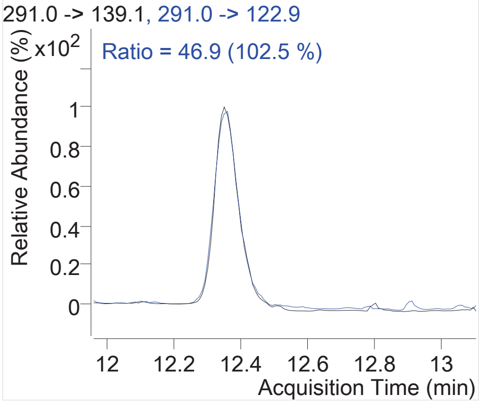

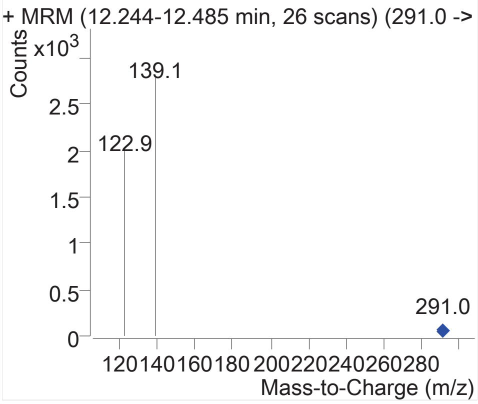

Caffeic acid

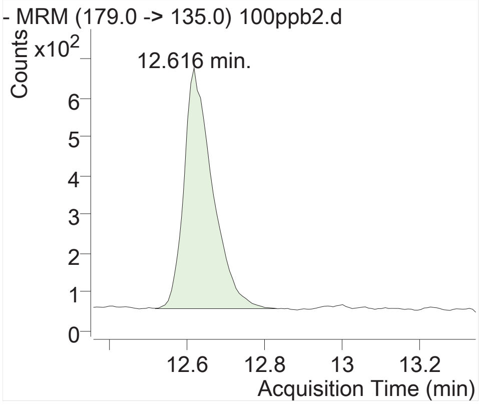

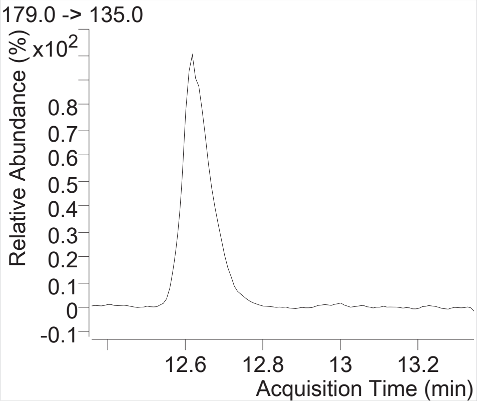

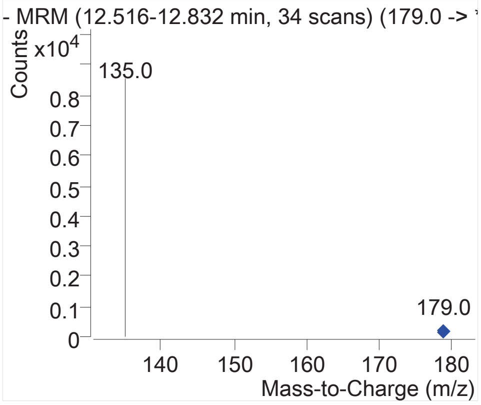

Syringic acid

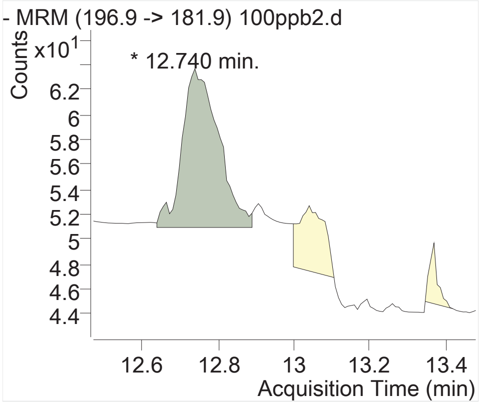

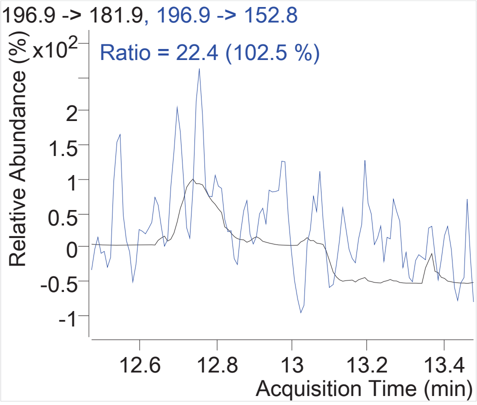

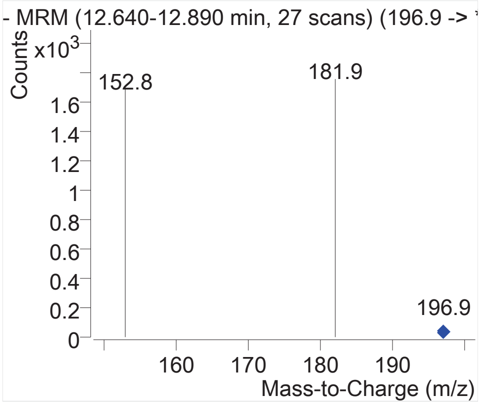

Vanillin

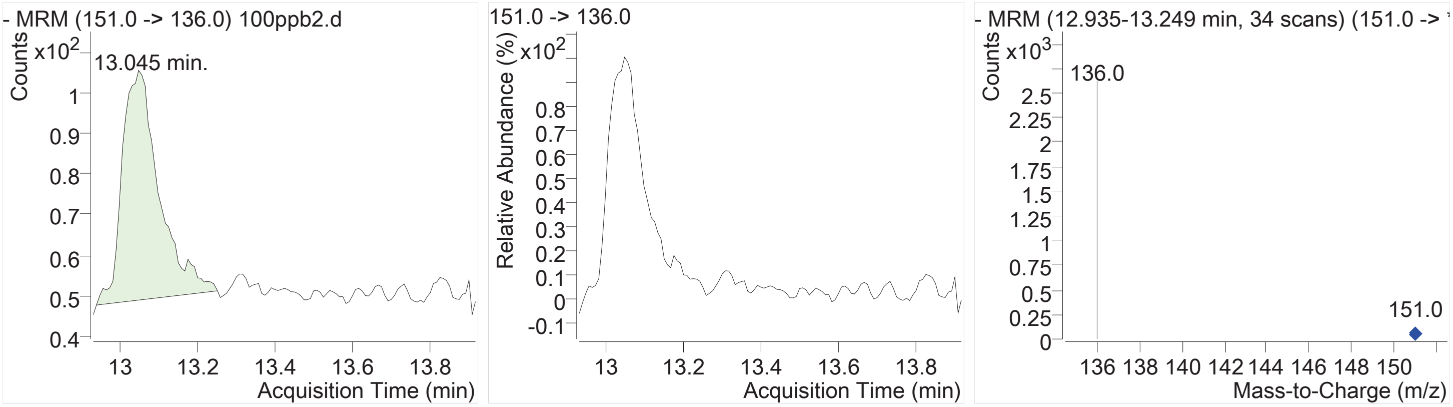

Verbascoside

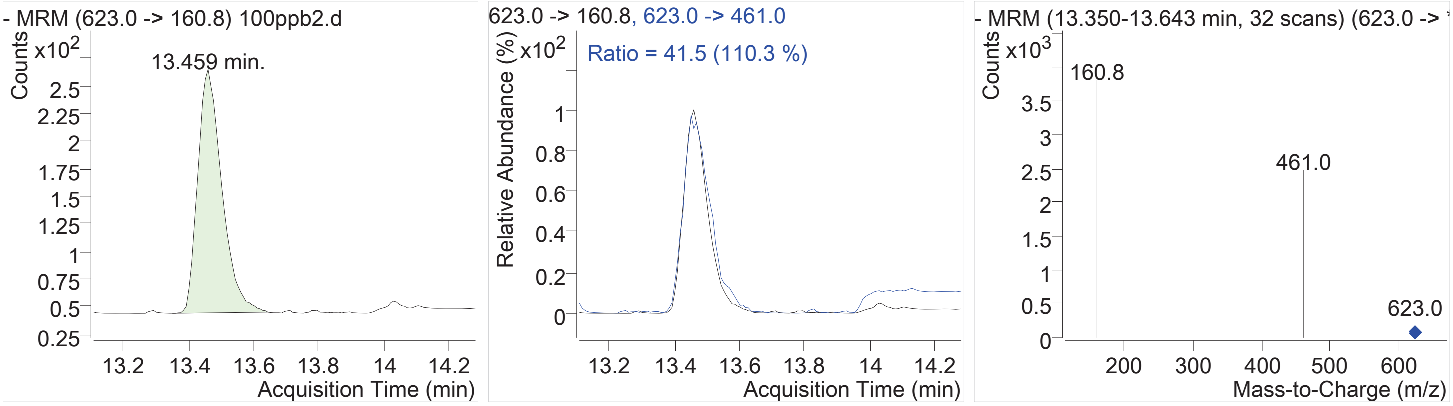

Taxifolin

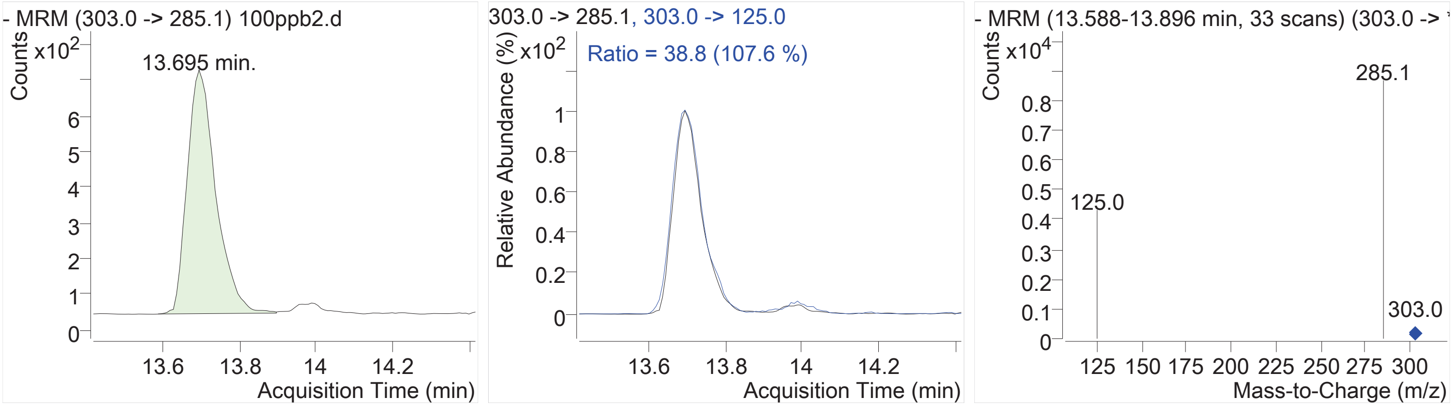

p-Coumaric acid

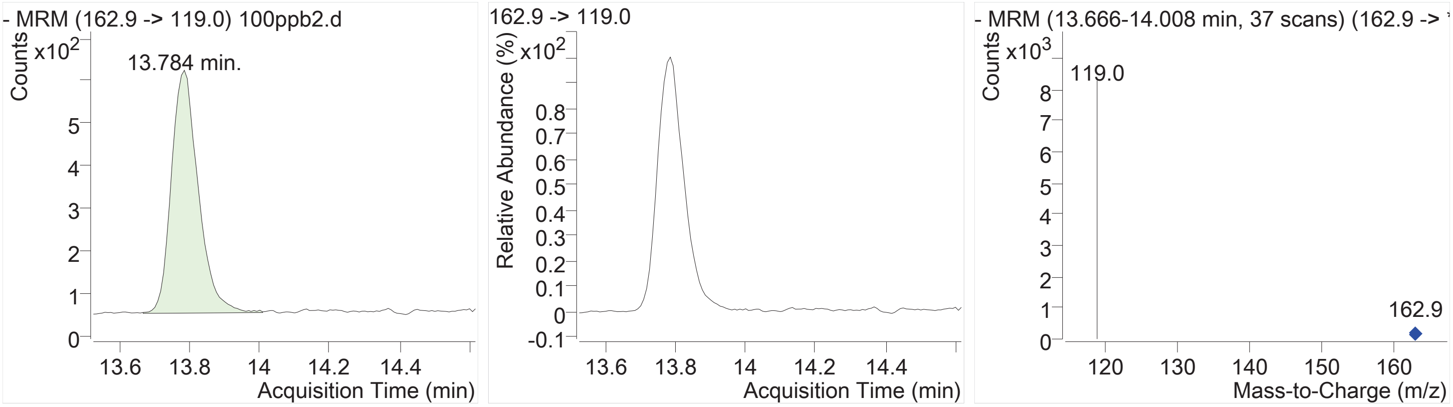

Sinapic acid

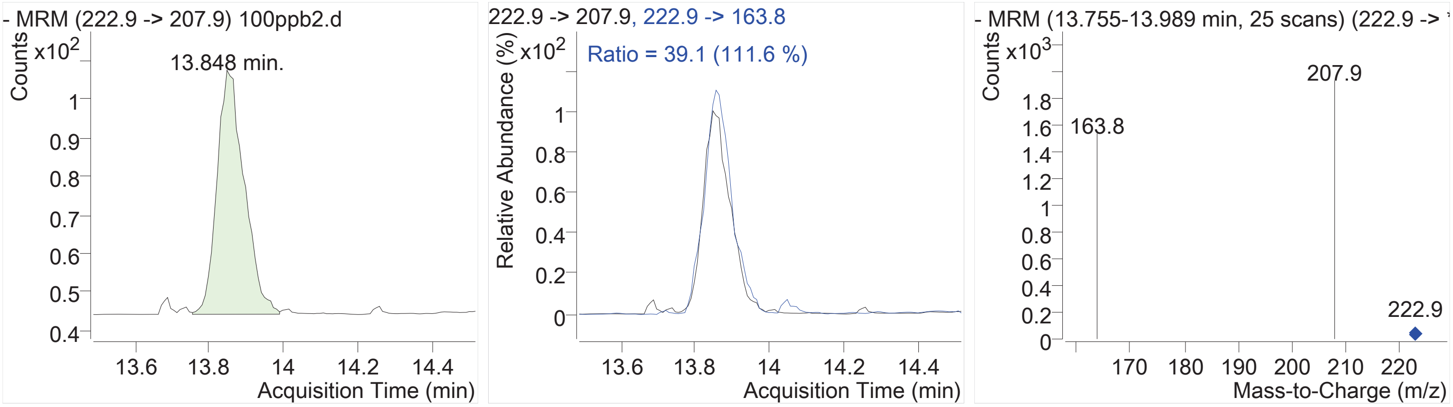

Ferulic acid

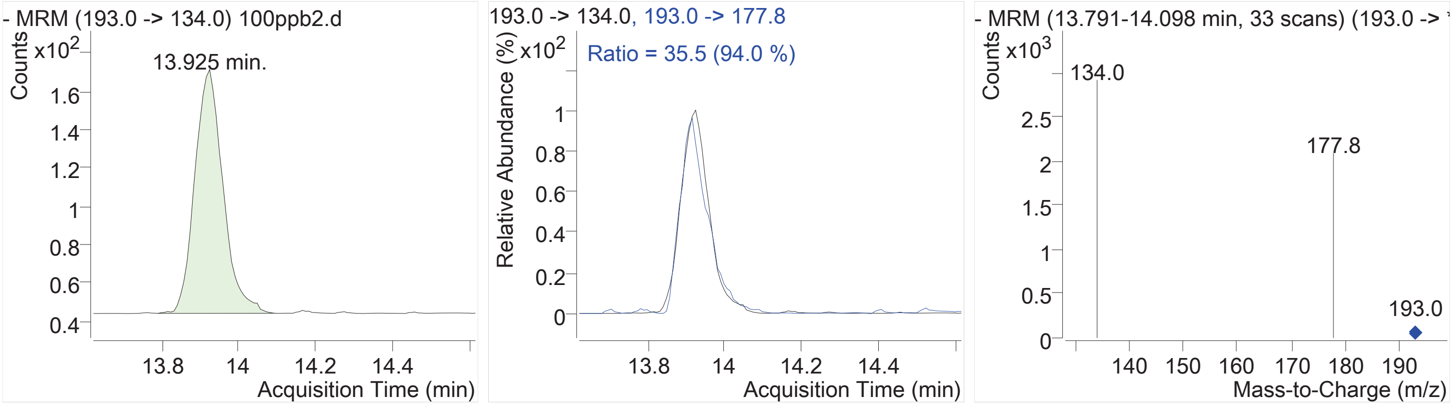

Luteolin 7-glucoside

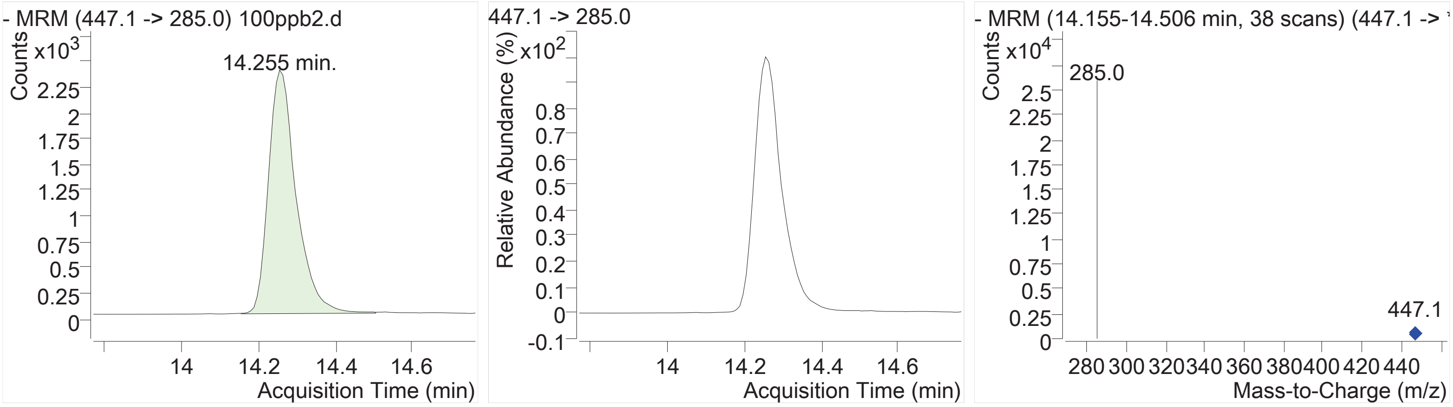

Hesperidin

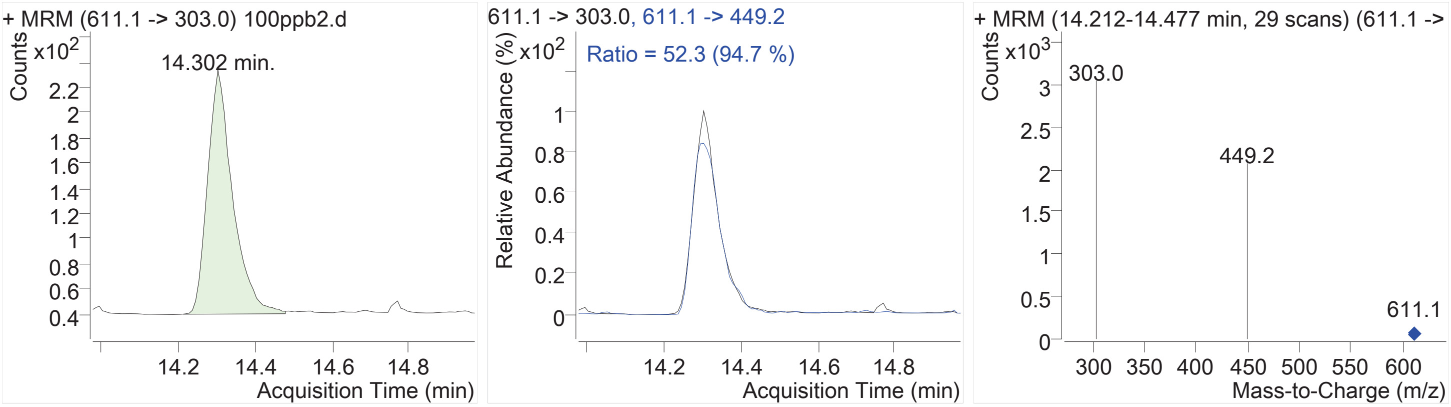

Hyperoside

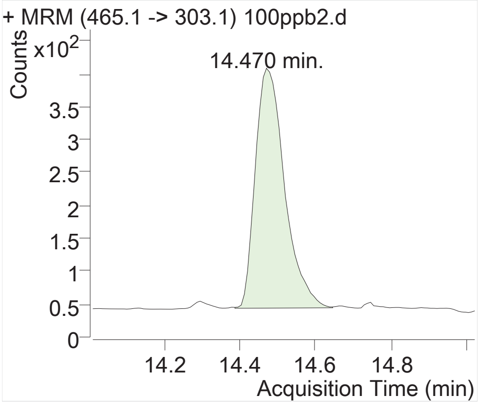

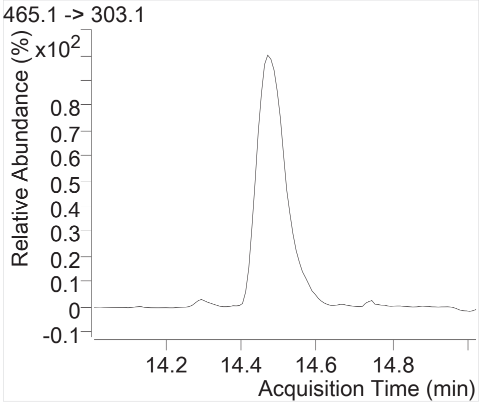

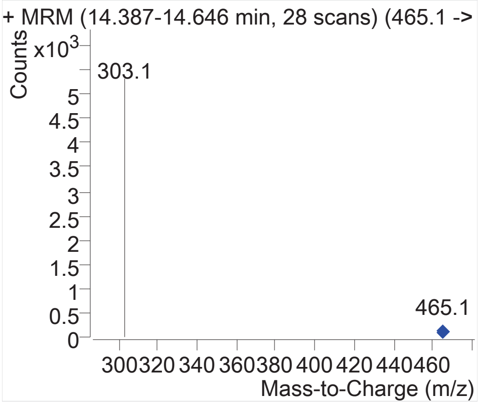

Rosmarinic acid

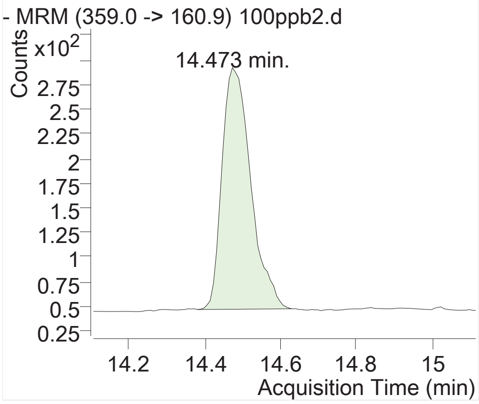

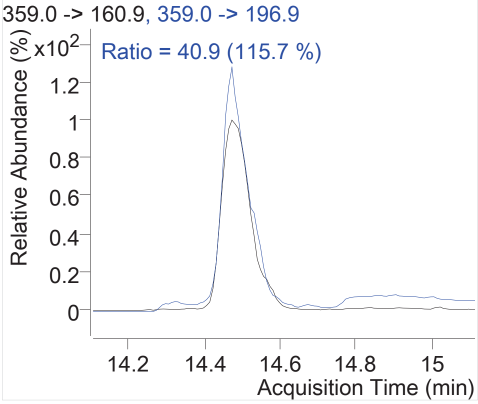

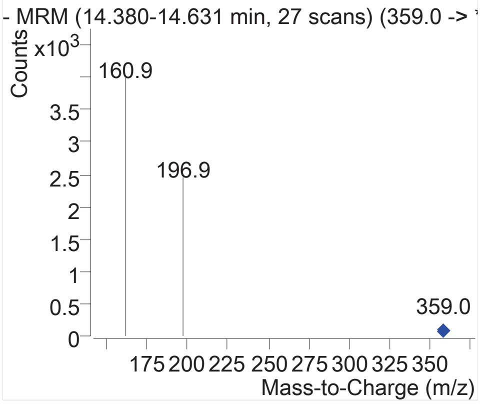

Apigenin 7-glucoside

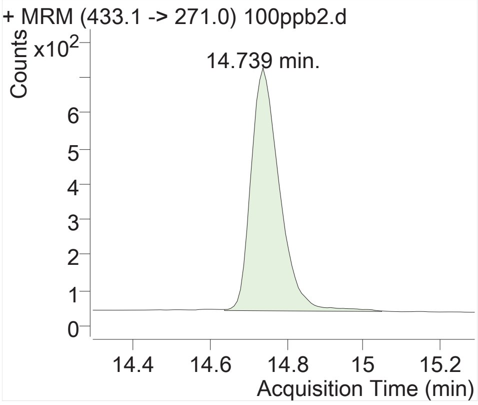

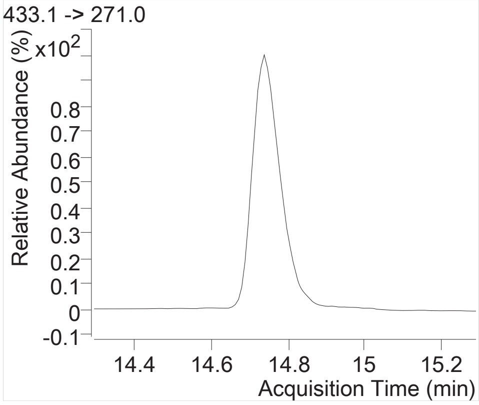

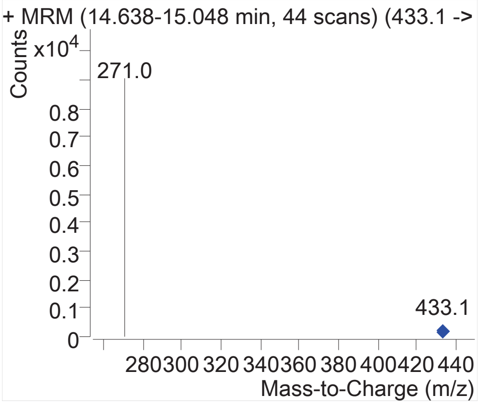

Pinoresinol

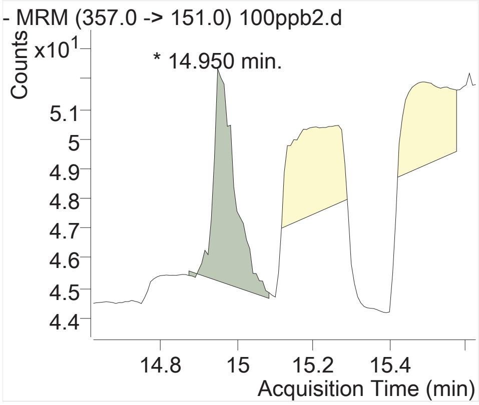

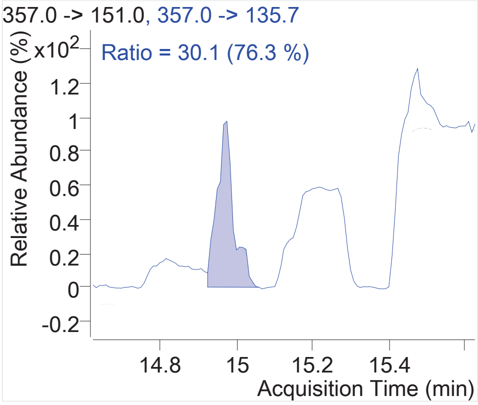

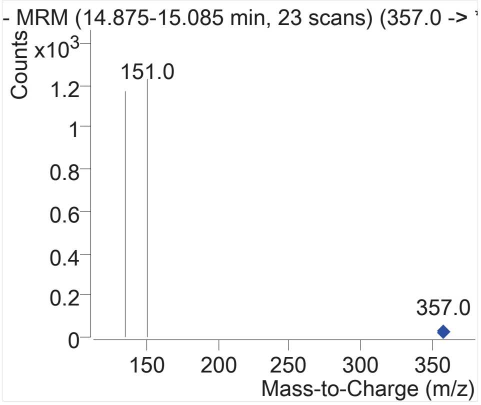

2-Hydroxycinnamic acid

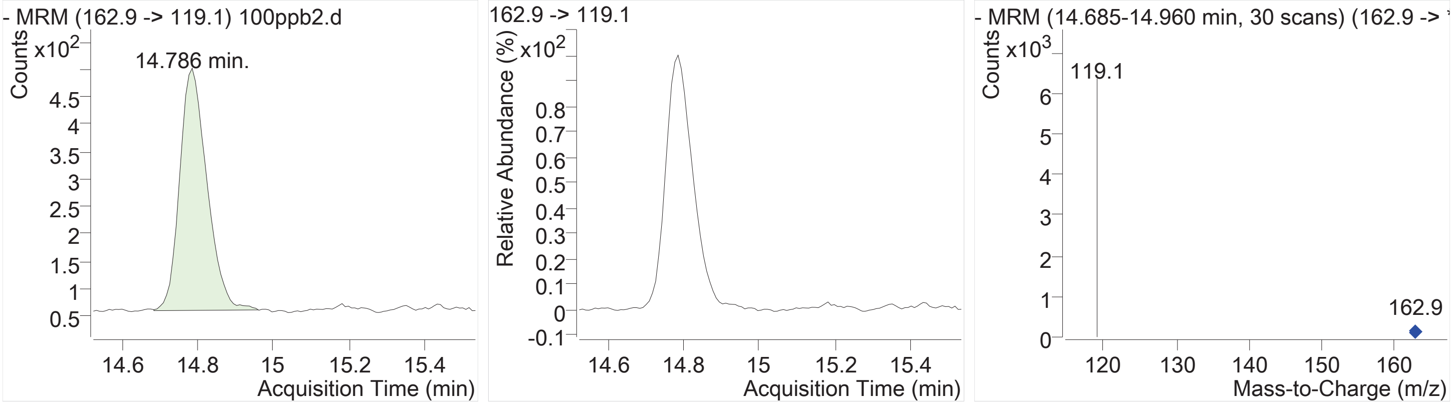

Eriodictyol

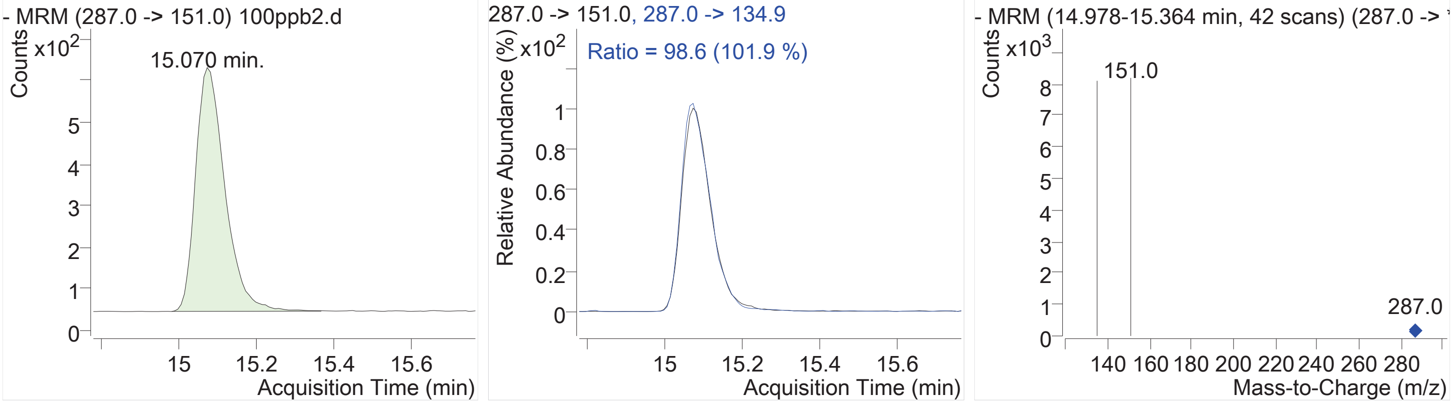

Quercetin

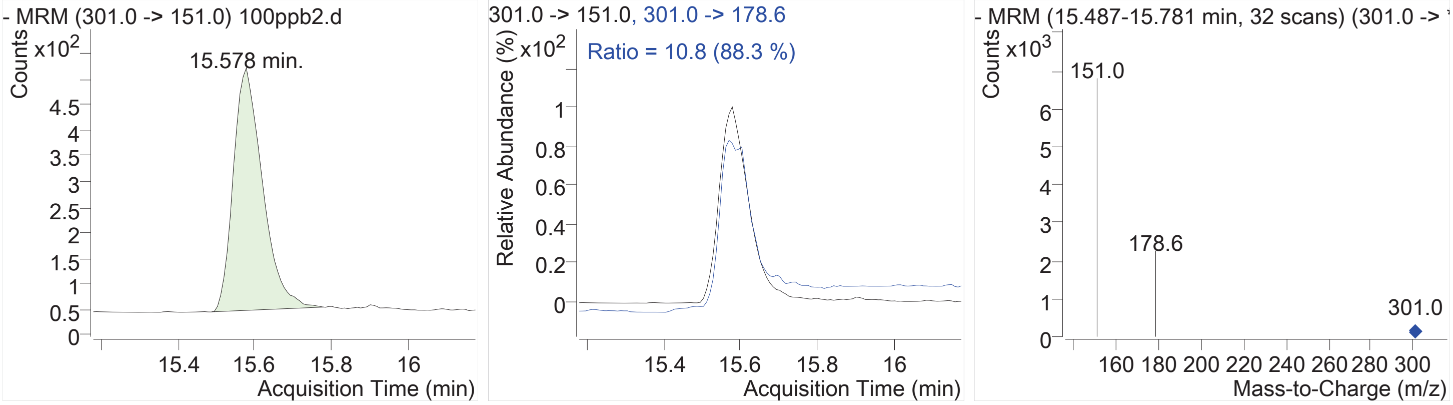

Luteolin

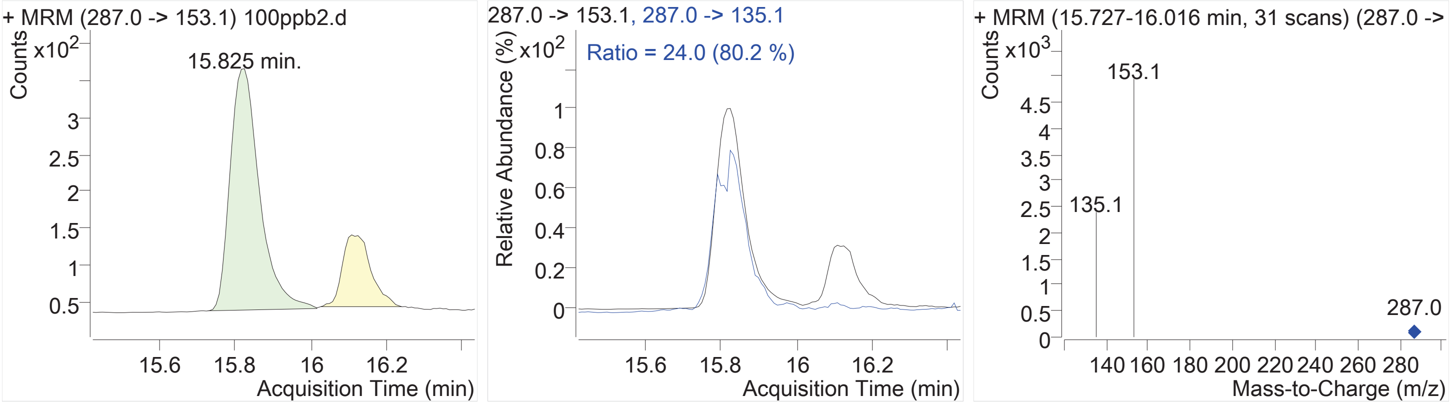

Kaempferol

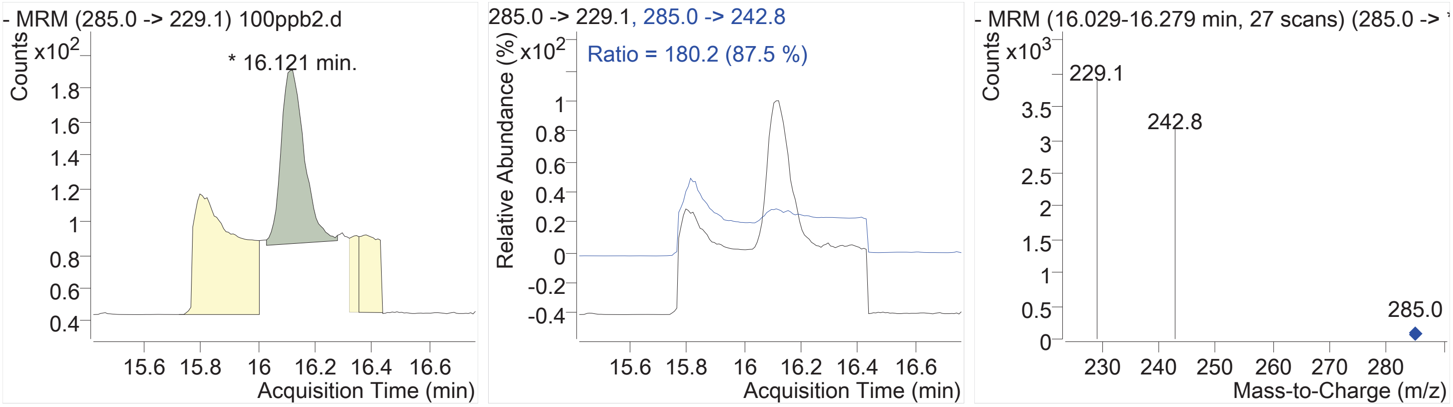

Apigenin

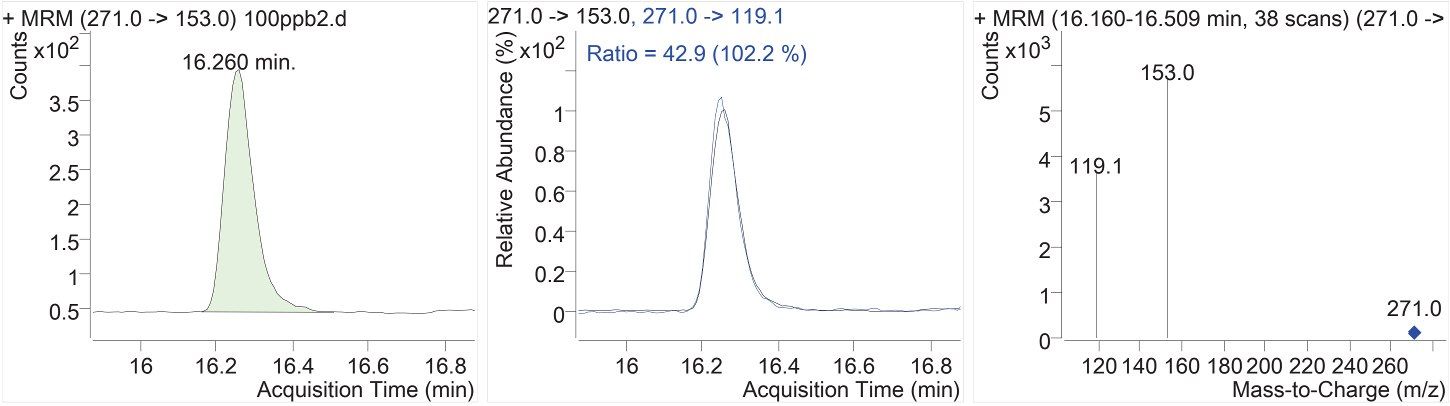

Quantitative Analysis Complete Report

**Batch Path**	D:\MassHunter\Data\2022ekim\061022cengizhoca\QuantResults\071022.batch.bin		
**Analysis Time**	10/11/2022 1:33:26 PM	**Analyst Name**	Defam-PC\admin
**Report Time**	10/11/2022 1:34:14 PM	**Reporter Name**	admin
**Last Calib Update**	10/11/2022 1:33:17 PM	**Batch State**	Processed
**Quant Batch Version**	B.07.01	**Quant Report Version**	B.07.01

**Acq. Time**	10/6/2022 6:48:34 PM	**Data File**	100ppb3.d
**Sample Type**	Cal	**Sample Name**	100ppb3
**Dilution**	1	**Acq. Method**	FENOLIK_DMRM2021-31bilesen.m

Sample Chromatogram

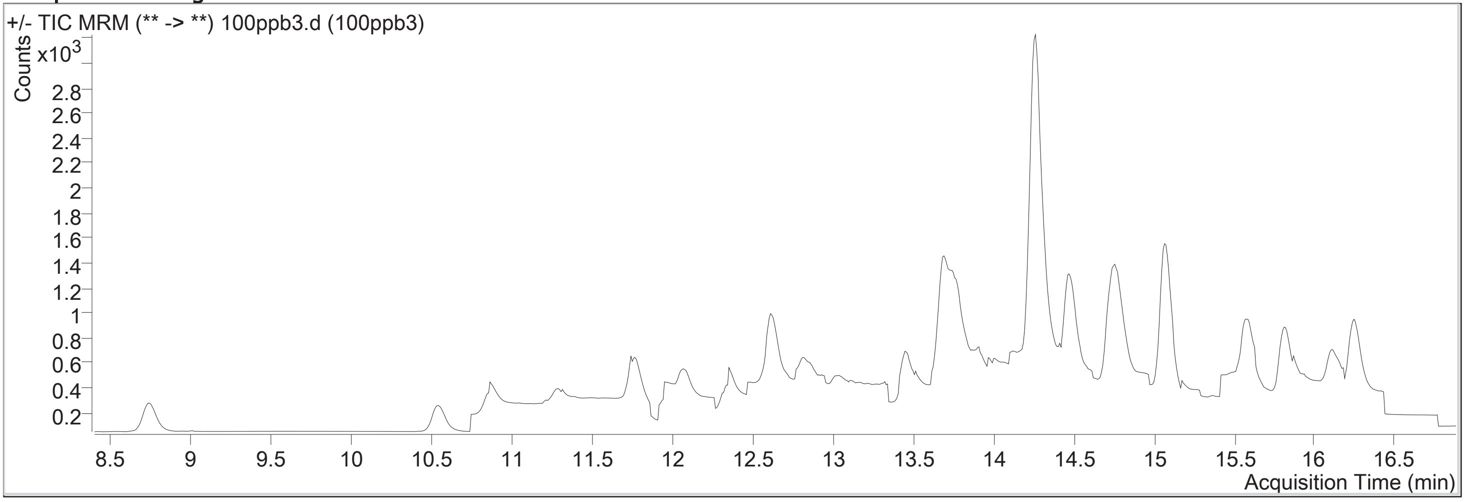

Compound	Transition	RT	Resp.	Final Conc	Units
Gallic acid	168.9 -> 125.0	8.741	1360	105.7217	ng/ml
Protocatechuic acid	152.9 -> 108.9	10.543	1231	107.3067	ng/ml
Pyrocatechol	109.0 -> 52.9	10.805	32	87.7827	ng/ml
3,4-Dihydroxyphenylacetic acid	167.0 -> 123.0	10.860	966	113.0111	ng/ml
(+)-Catechin	289.0 -> 245.0	11.294	403	94.1036	ng/ml
2,5-Dihydroxybenzoic acid	152.9 -> 109.0	11.954	684	108.6916	ng/ml
Chlorogenic acid	355.0 -> 163.0	11.777	2379	117.4567	ng/ml
3-Hydroxybenzoic acid	137.0 -> 93.0	12.819	534	114.8329	ng/ml
4-Hydroxybenzoic acid	136.9 -> 93.1	12.072	1272	101.7121	ng/ml
(-)-Epicatechin	291.0 -> 139.1	12.351	816	105.9278	ng/ml
Caffeic acid	179.0 -> 135.0	12.616	3025	101.6371	ng/ml
Syringic acid	196.9 -> 181.9	12.765	73	104.2785	ng/ml
Vanillin	151.0 -> 136.0	13.045	394	97.2655	ng/ml
Verbascoside	623.0 -> 160.8	13.451	1182	104.9154	ng/ml
Taxifolin	303.0 -> 285.1	13.695	3736	102.3347	ng/ml
p-Coumaric acid	162.9 -> 119.0	13.784	3173	114.5153	ng/ml
Sinapic acid	222.9 -> 207.9	13.856	343	112.6130	ng/ml
Ferulic acid	193.0 -> 134.0	13.925	637	122.4015	ng/ml
Luteolin 7-glucoside	447.1 -> 285.0	14.255	12280	101.2657	ng/ml
Hesperidin	611.1 -> 303.0	14.310	925	116.3887	ng/ml
Hyperoside	465.1 -> 303.1	14.470	2113	110.8718	ng/ml
Rosmarinic acid	359.0 -> 160.9	14.481	1445	114.2891	ng/ml
Apigenin 7-glucoside	433.1 -> 271.0	14.739	3776	110.9796	ng/ml
Pinoresinol	357.0 -> 151.0	14.942	27	80.8620	ng/ml
2-Hydroxycinnamic acid	162.9 -> 119.1	14.777	2296	106.4425	ng/ml
Eriodictyol	287.0 -> 151.0	15.078	3150	105.8178	ng/ml
Quercetin	301.0 -> 151.0	15.578	2534	103.6220	ng/ml
Luteolin	287.0 -> 153.1	15.816	1849	111.7955	ng/ml
Kaempferol	285.0 -> 229.1	16.105	580	111.4005	ng/ml

Quantitative Analysis Complete Report

Compound	Transition	RT	Resp.	Final Conc	Units
Apigenin	271.0 -> 153.0	16.251	2155	110.7457	ng/ml

Gallic acid

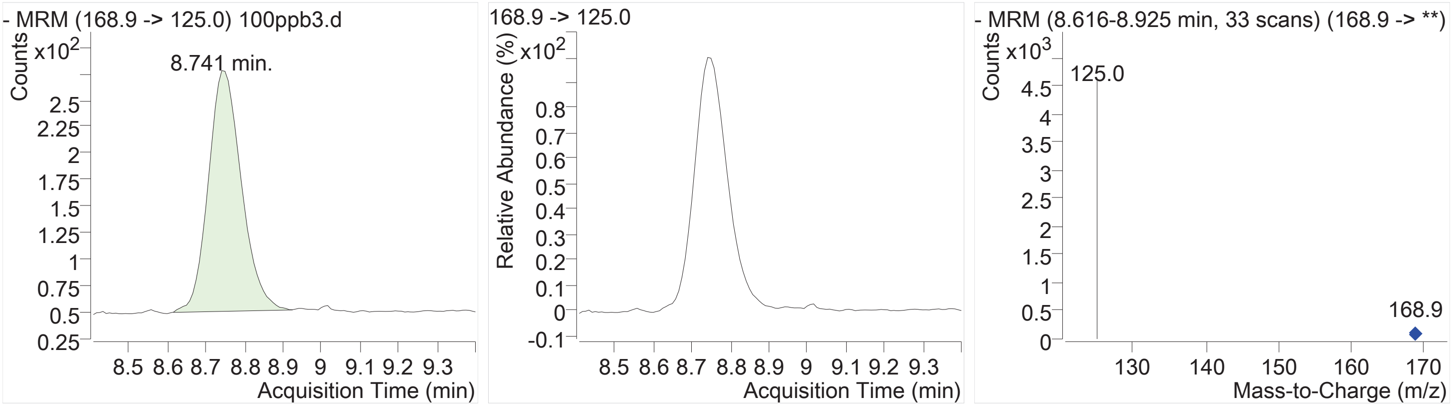

Protocatechuic acid

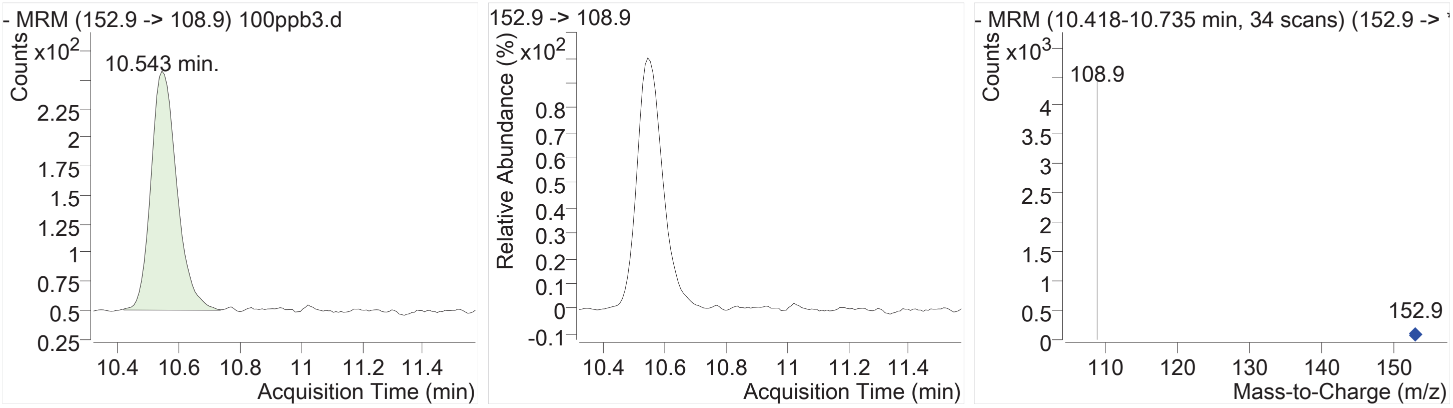

Pyrocatechol

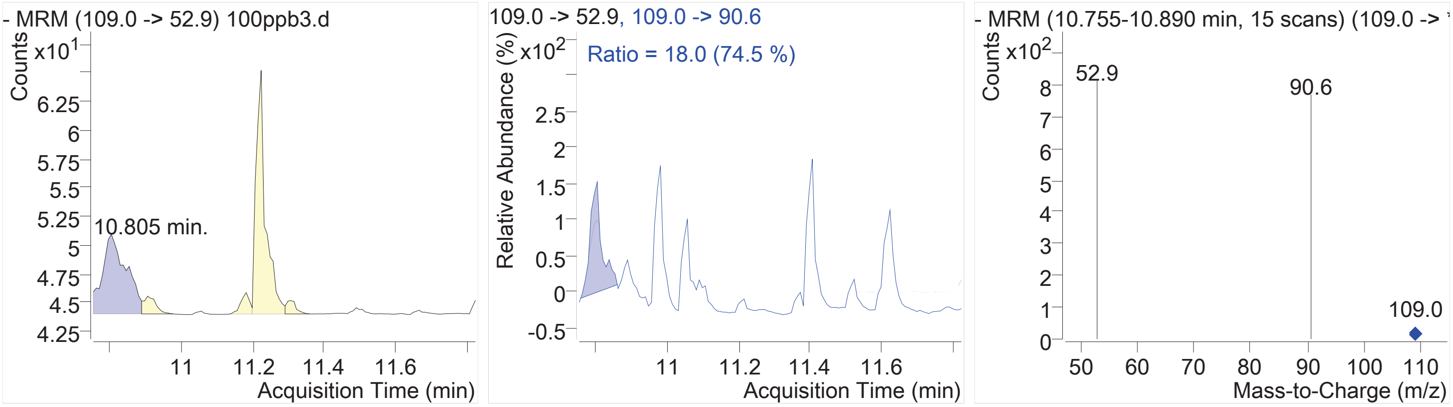

3,4-Dihydroxyphenylacetic acid

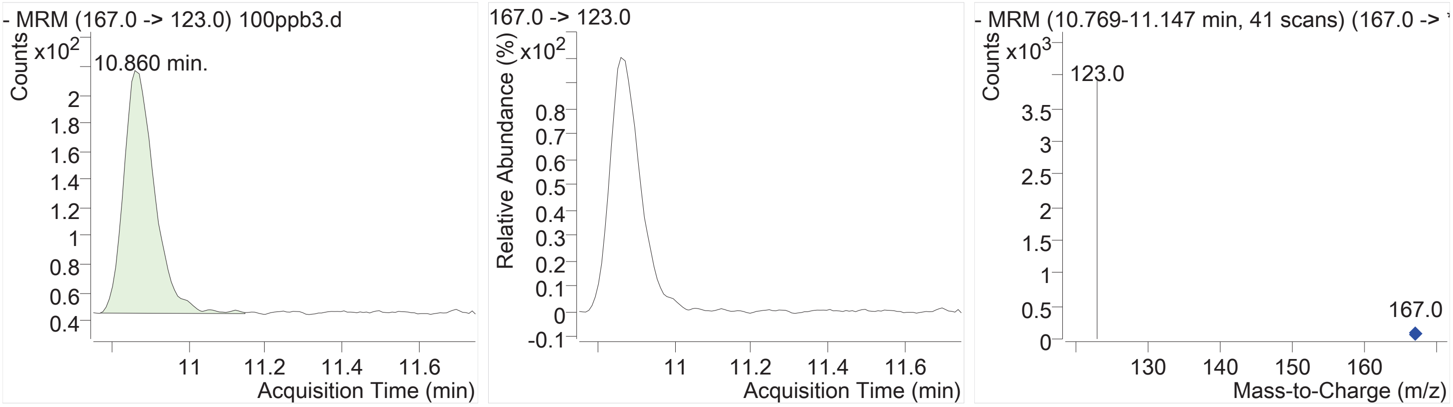

(+)-Catechin

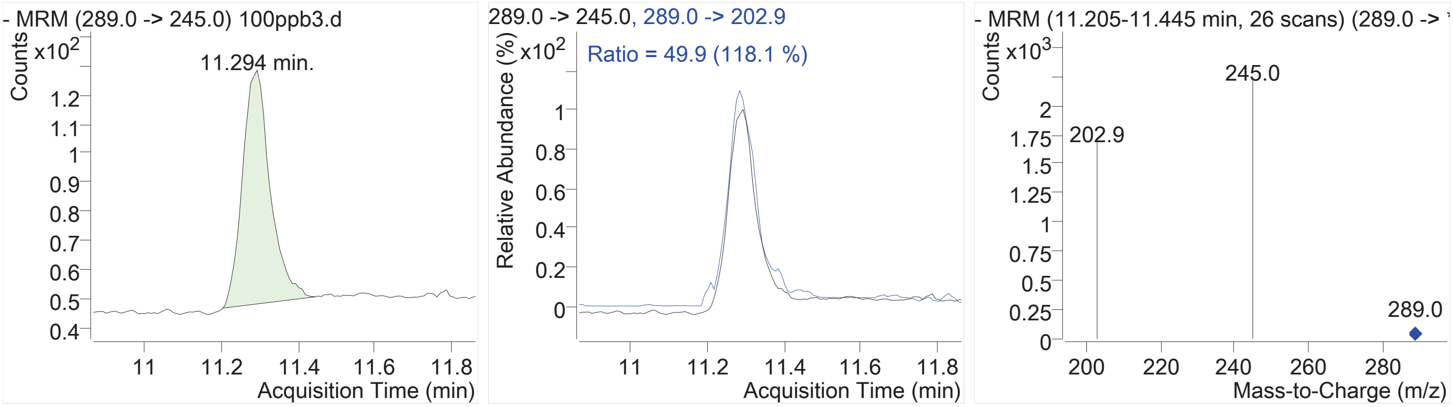

2,5-Dihydroxybenzoic acid

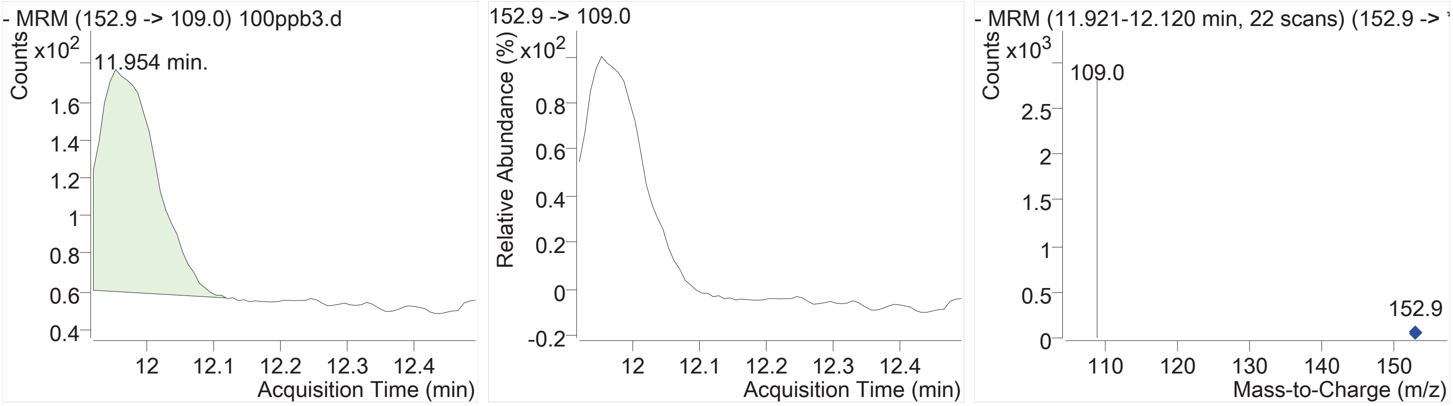

Chlorogenic acid

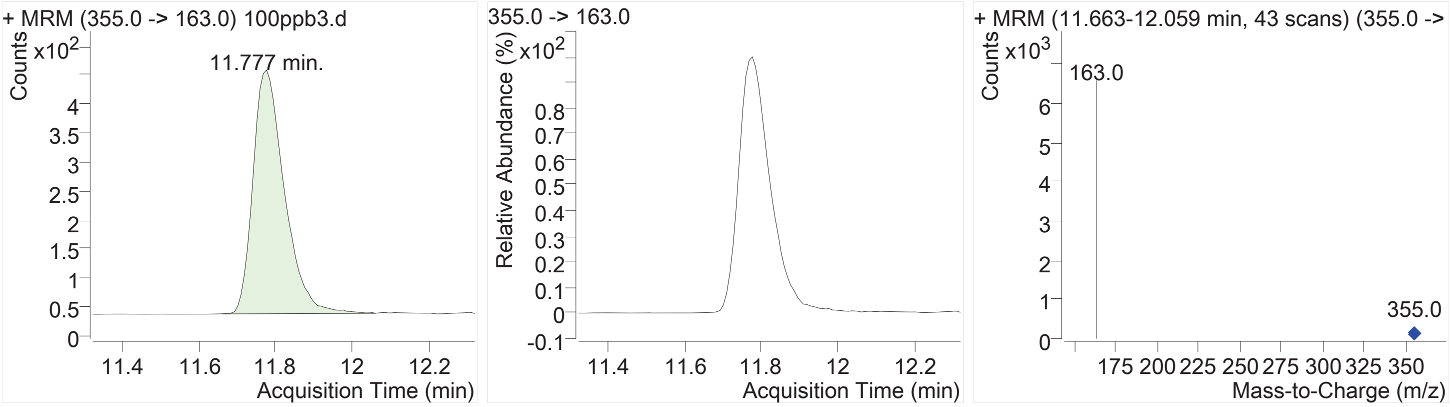

3-Hydroxybenzoic acid

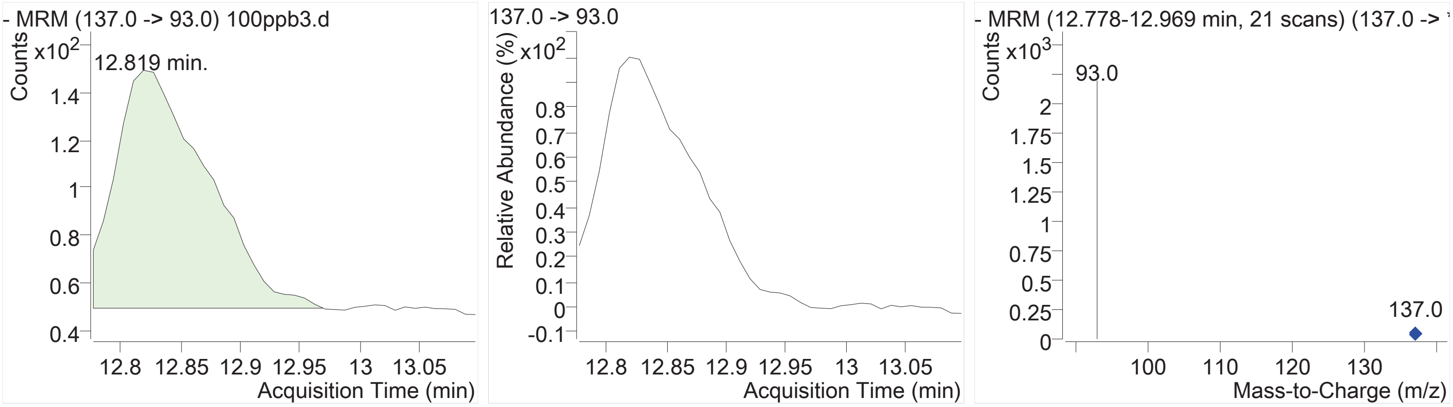

4-Hydroxybenzoic acid

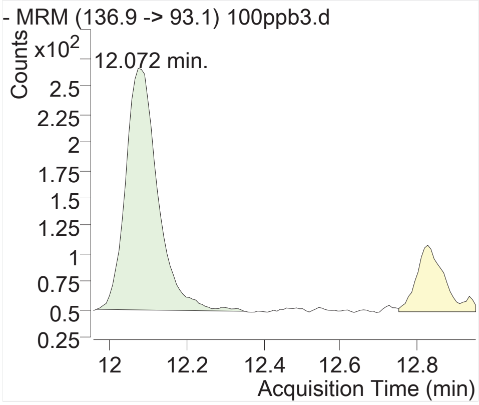

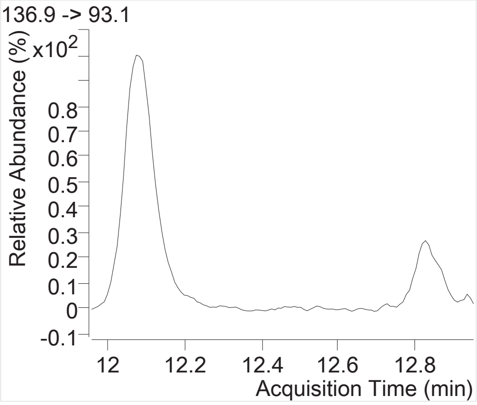

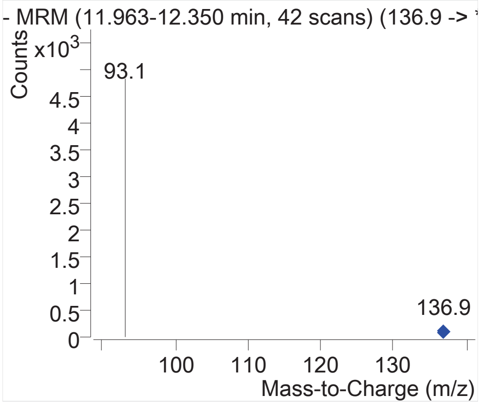

(-)-Epicatechin

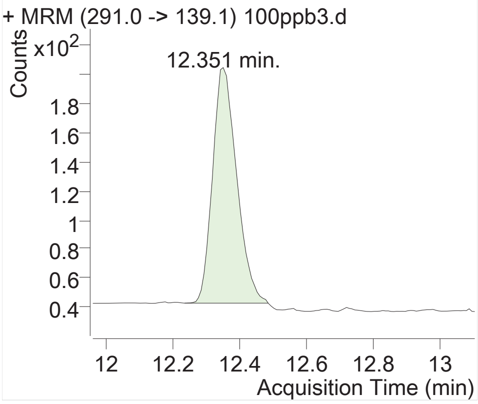

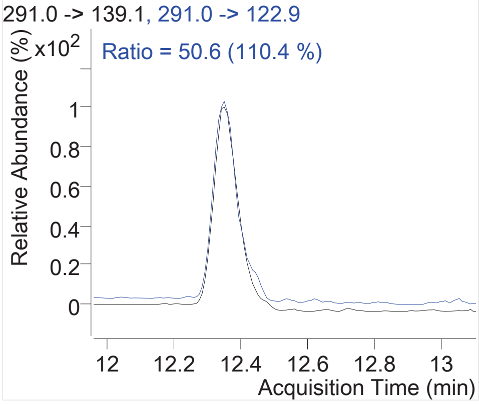

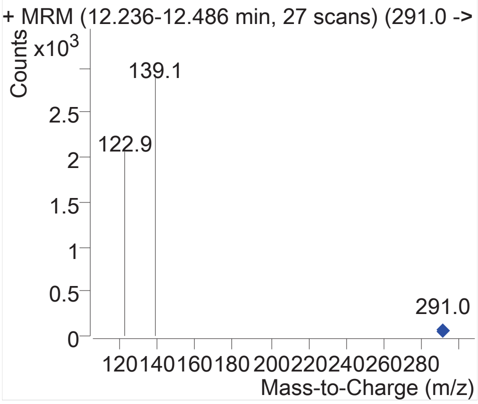

Caffeic acid

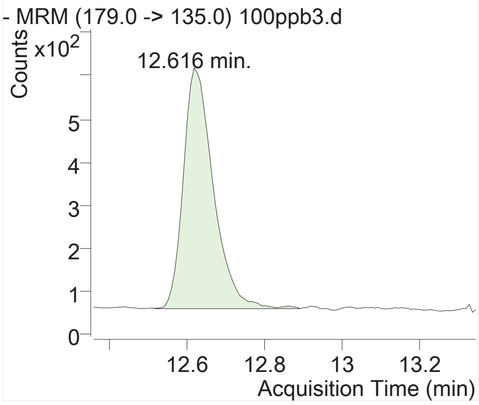

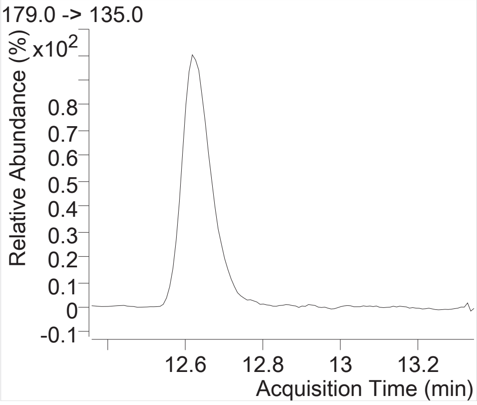

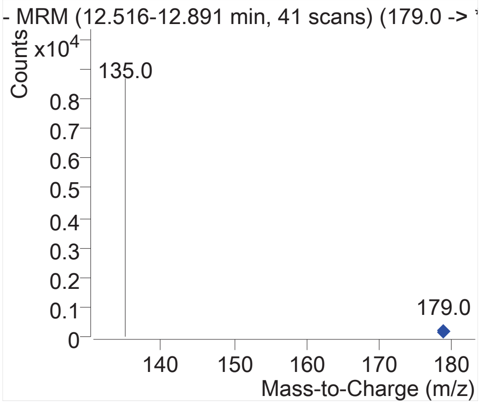

Syringic acid

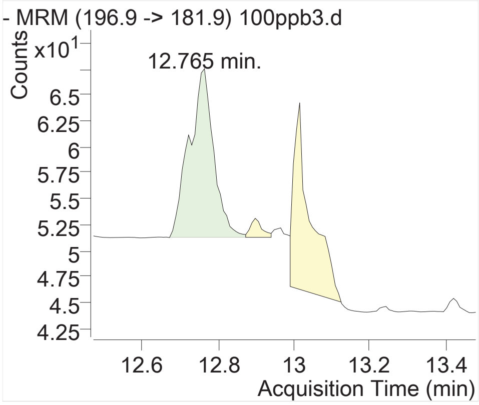

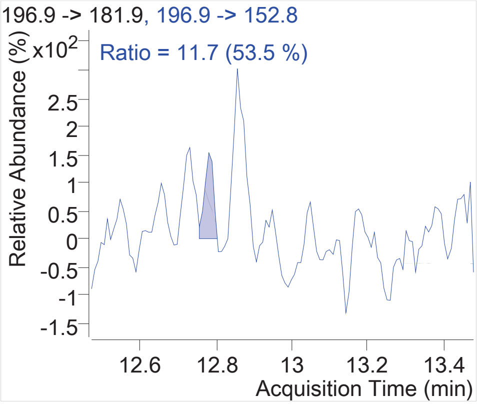

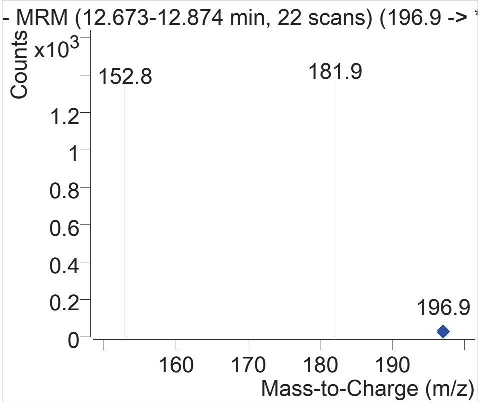

Vanillin

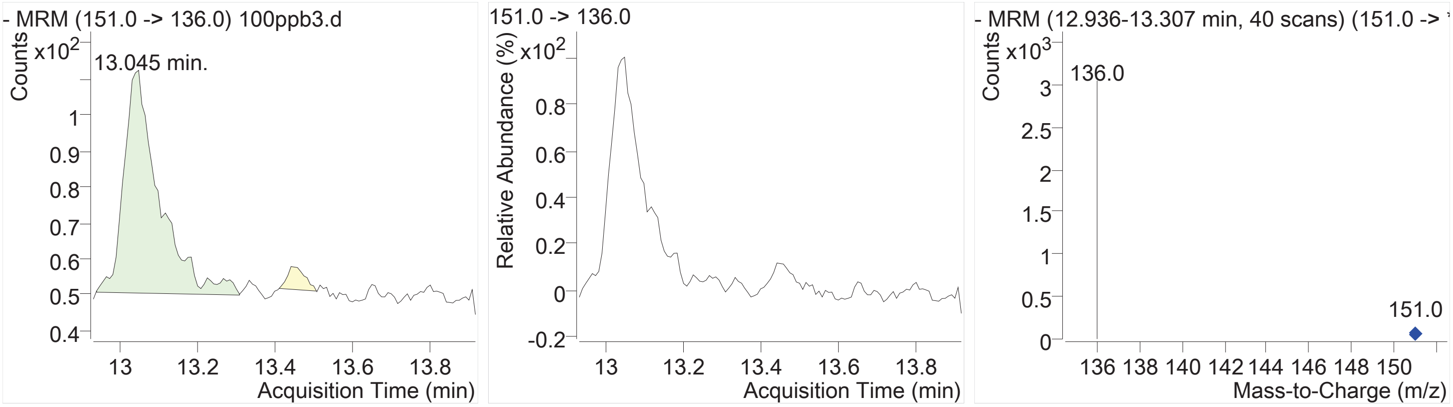

Verbascoside

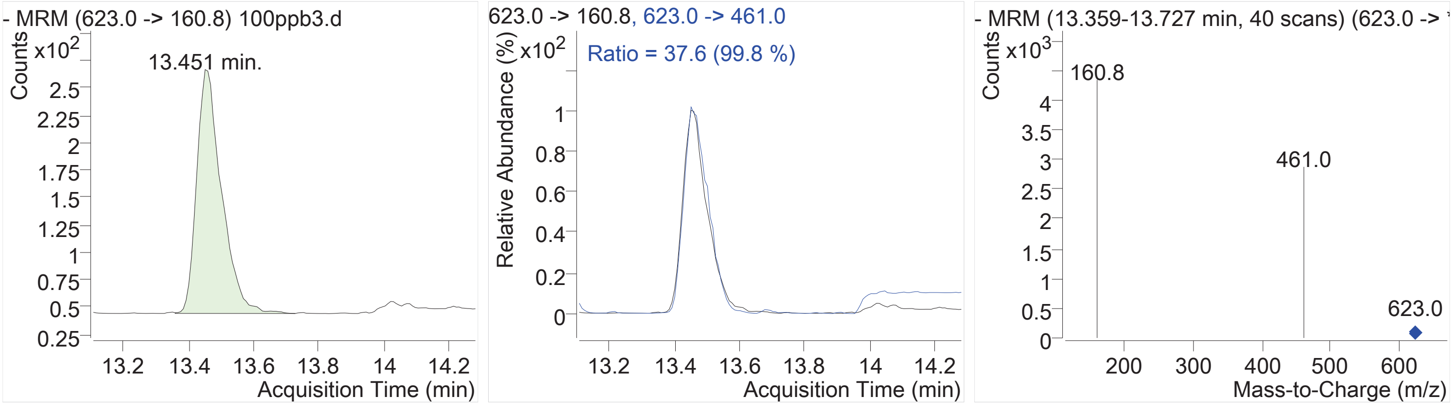

Taxifolin

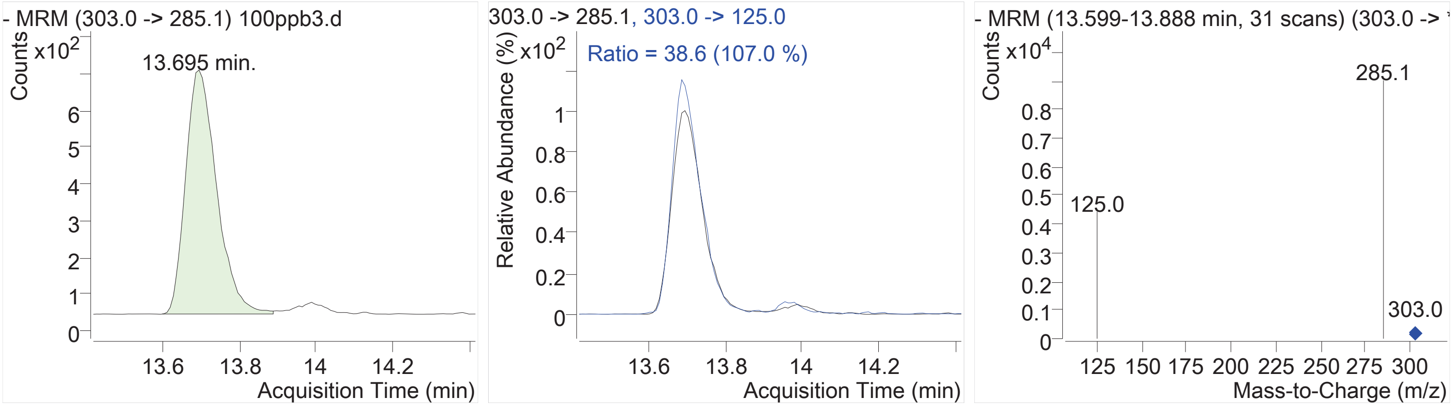

p-Coumaric acid

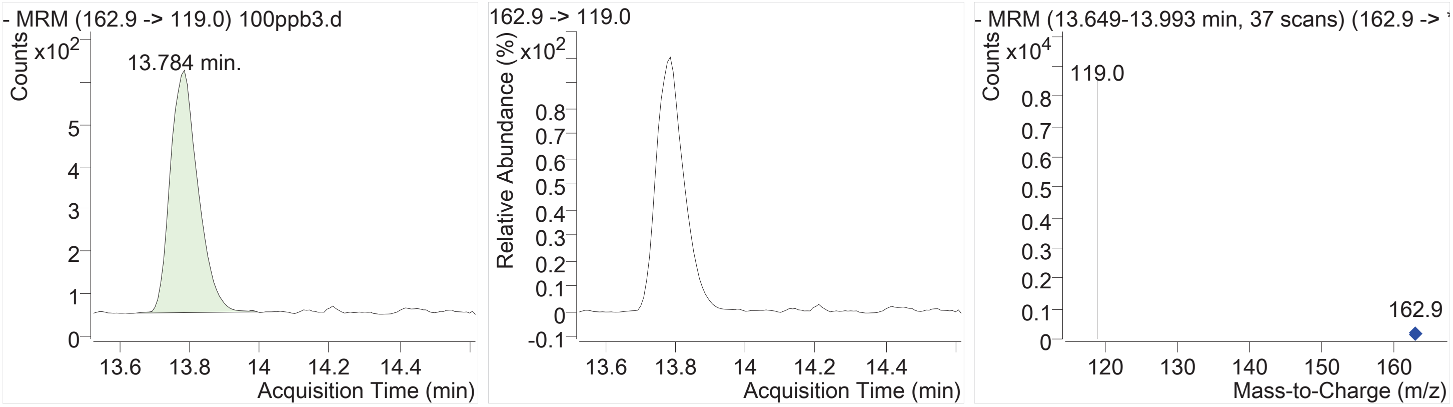

Sinapic acid

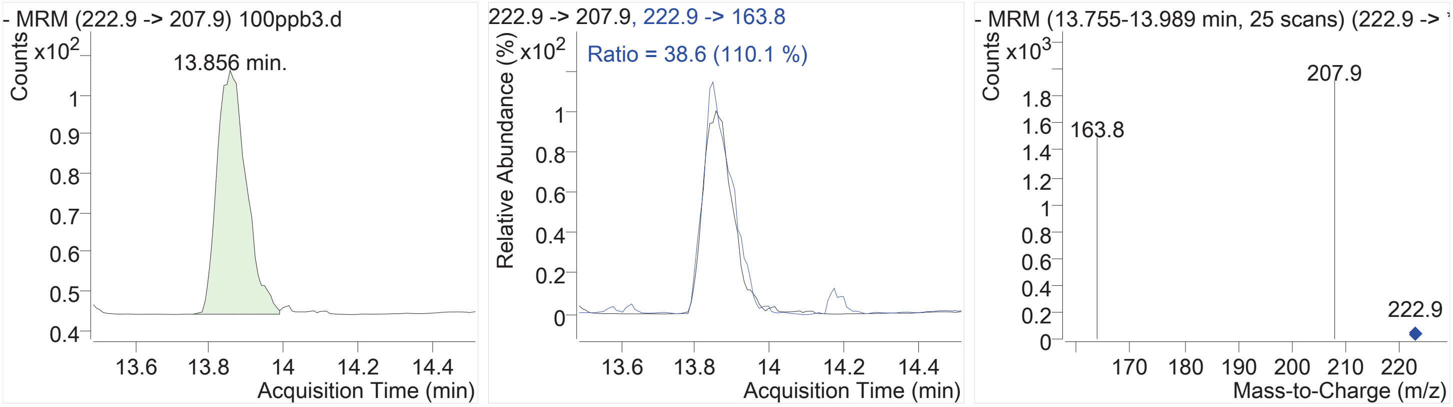

Ferulic acid

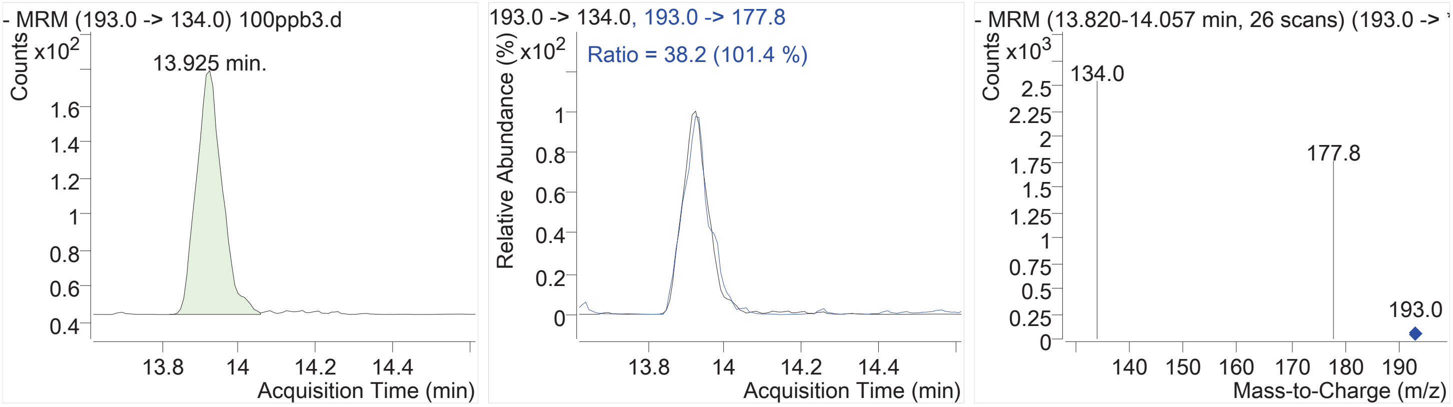

Luteolin 7-glucoside

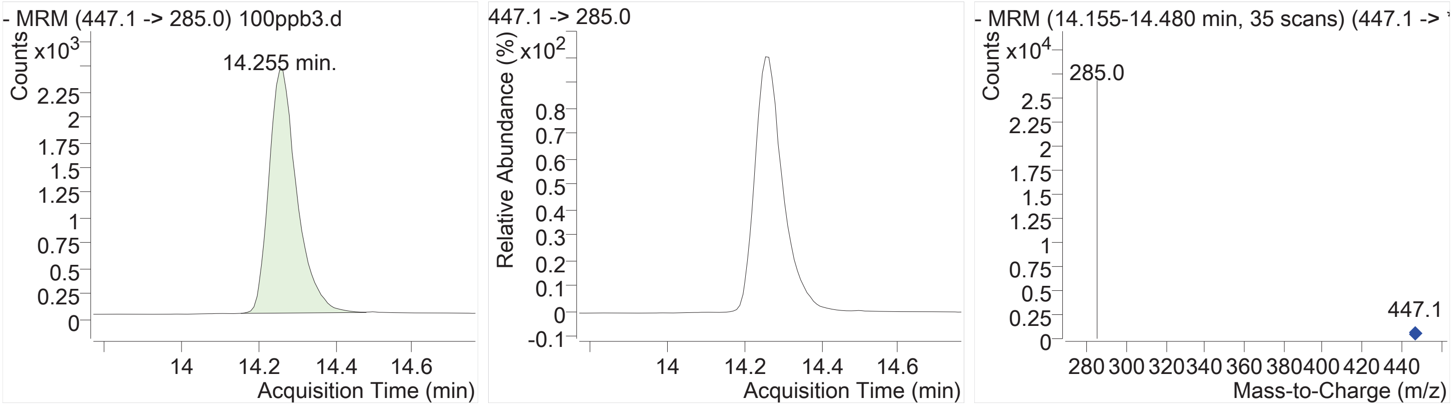

Hesperidin

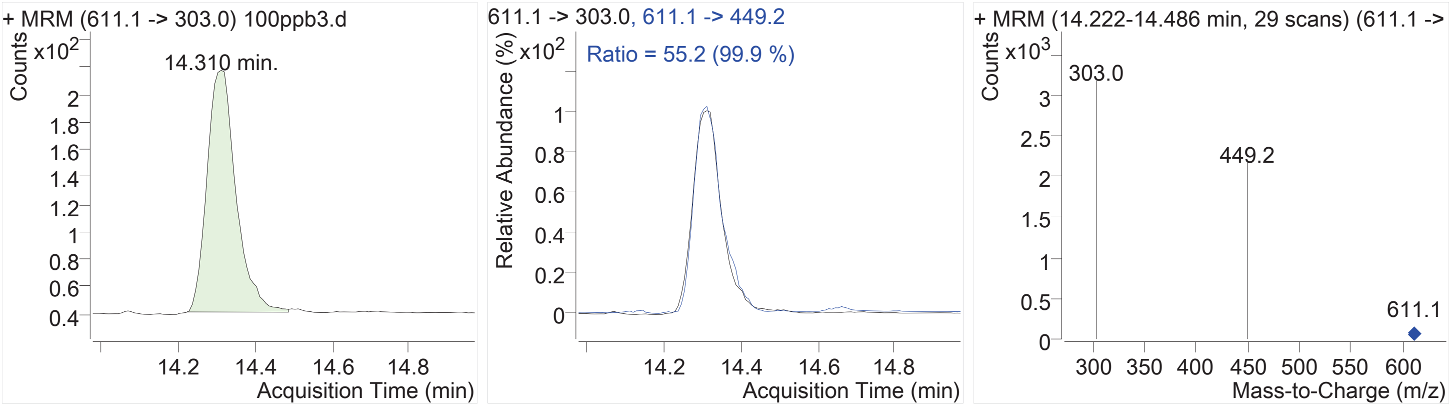

Agilent Technologies

Hyperoside

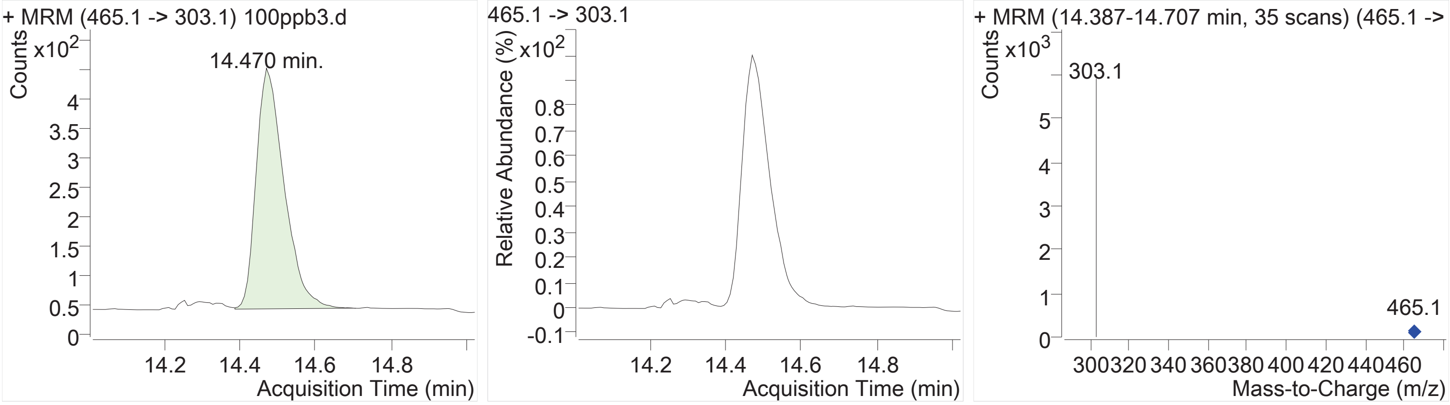

Rosmarinic acid

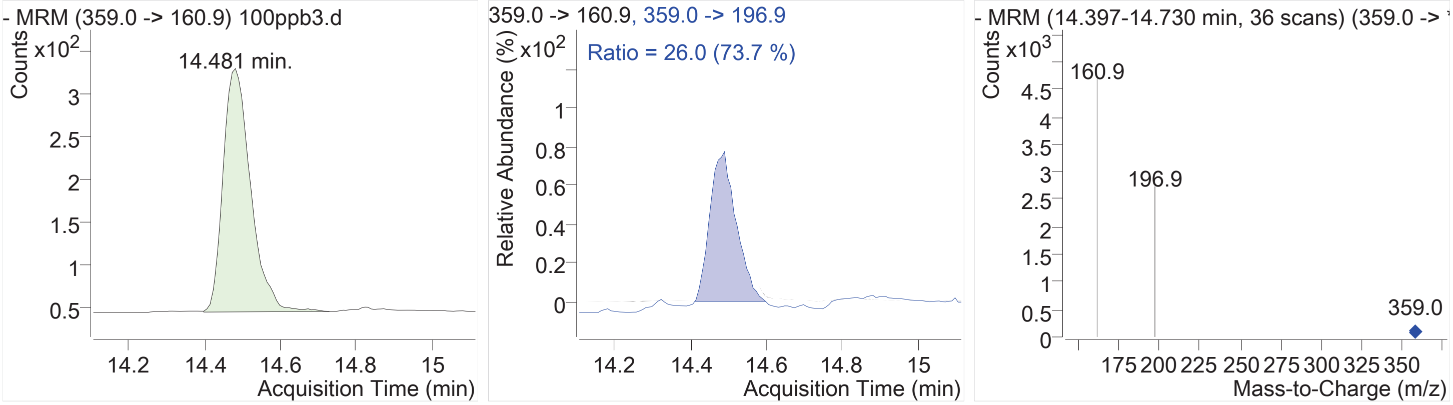

Apigenin 7-glucoside

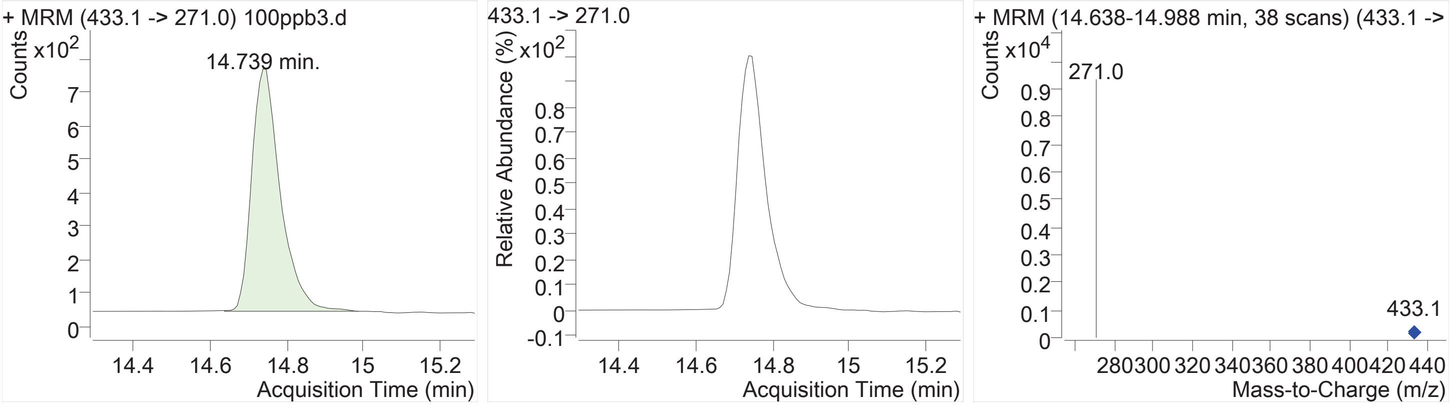

Pinoresinol

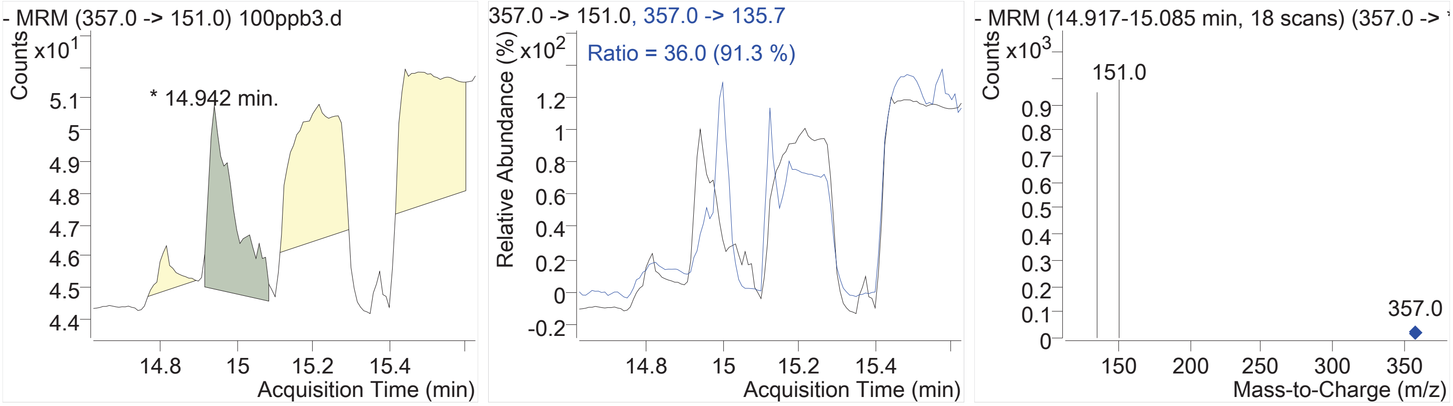

 Generated at 1:33 PM on 10/11/2022

2-Hydroxycinnamic acid

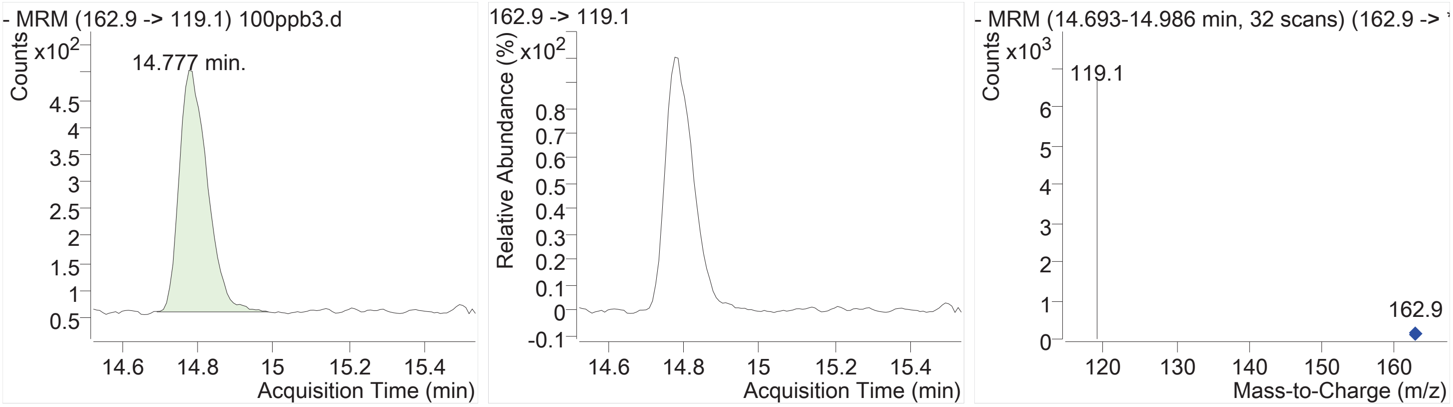

Eriodictyol

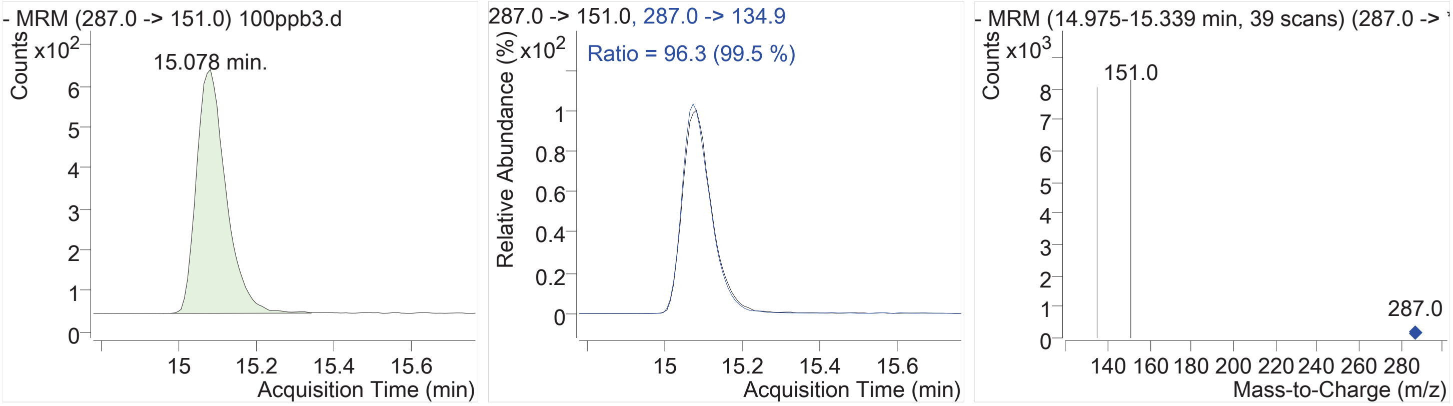

Quercetin

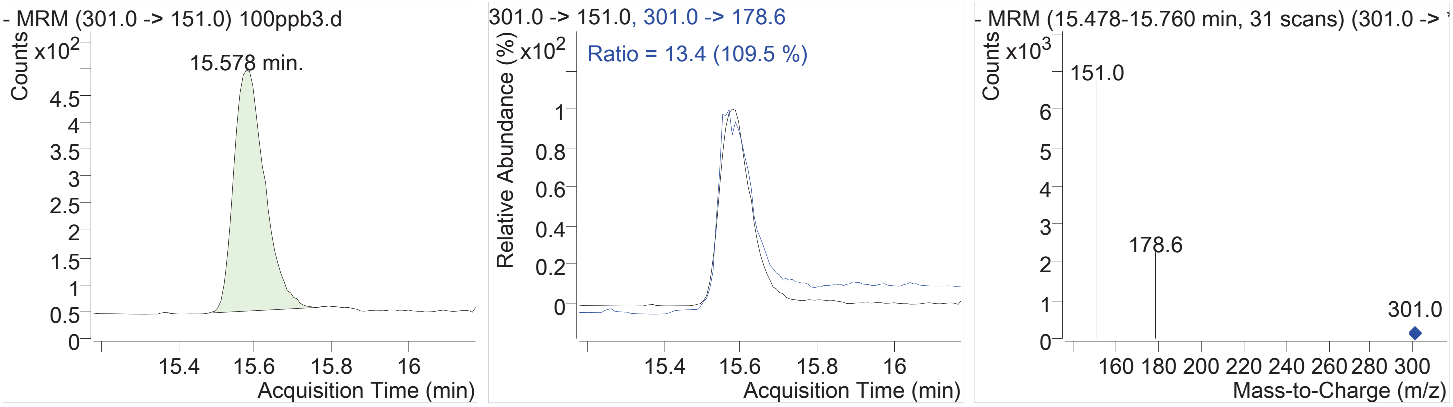

Luteolin

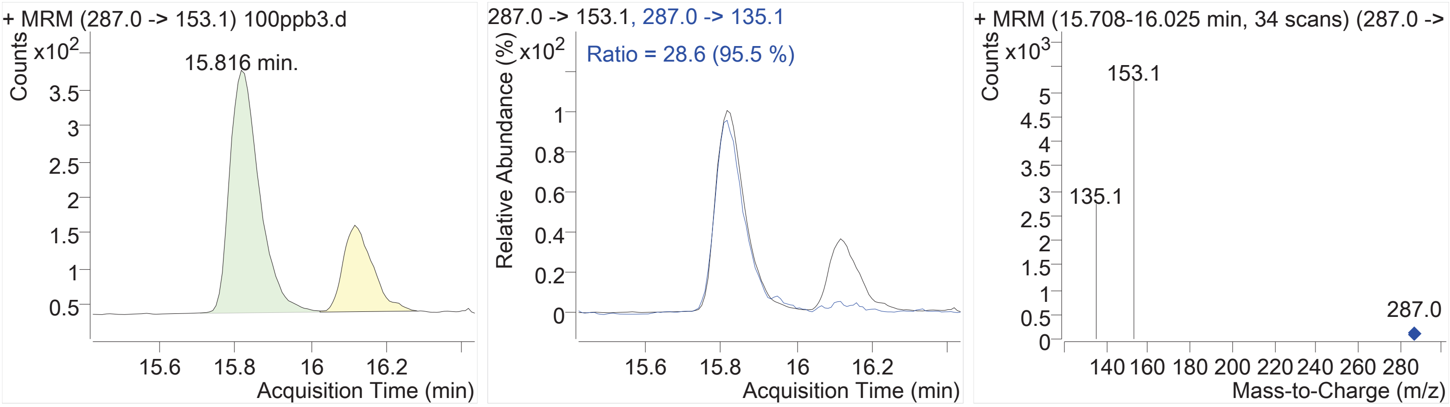

Kaempferol

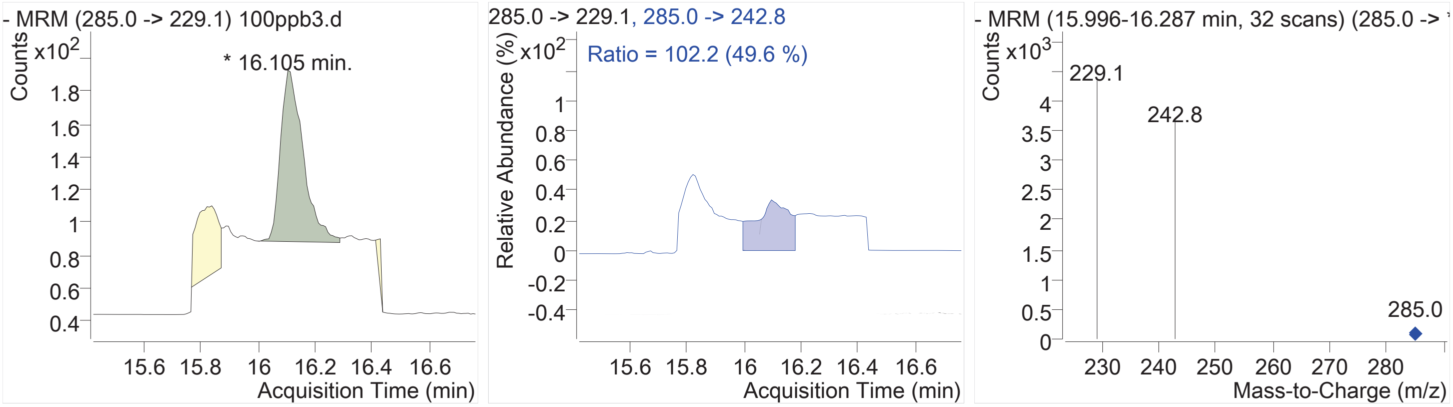

Apigenin

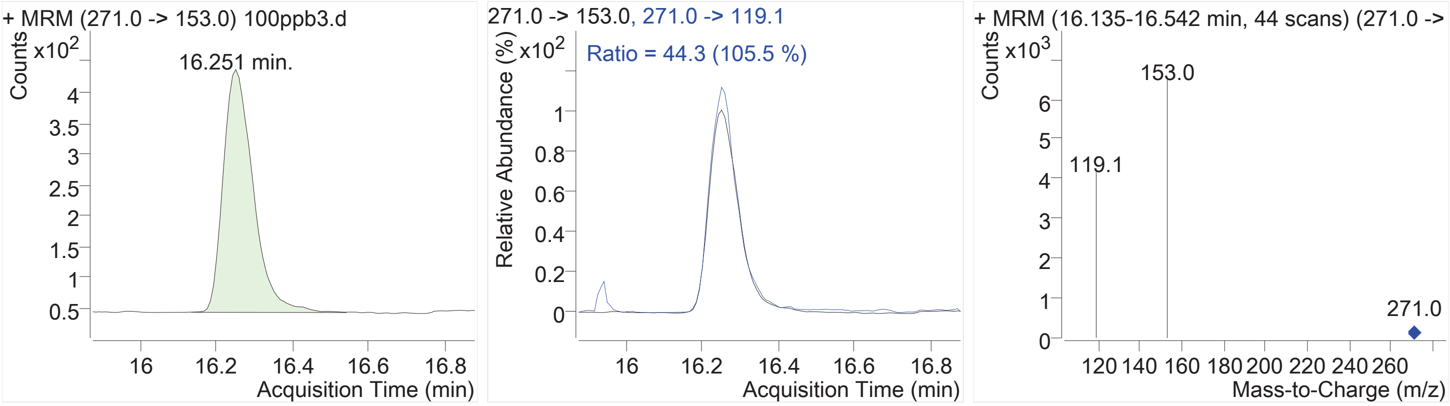

Quantitative Analysis Complete Report

Agilent Technologies

**Batch Path**	D:\MassHunter\Data\2022ekim\061022cengizhoca\QuantResults\071022.batch.bin		
**Analysis Time**	10/11/2022 1:33:26 PM	**Analyst Name**	Defam-PC\admin
**Report Time**	10/11/2022 1:34:17 PM	**Reporter Name**	admin
**Last Calib Update**	10/11/2022 1:33:17 PM	**Batch State**	Processed
**Quant Batch Version**	B.07.01	**Quant Report Version**	B.07.01

**Acq. Time**	10/6/2022 7:10:16 PM	**Data File**	250ppb1.d
**Sample Type**	Cal	**Sample Name**	250ppb1
**Dilution**	1	**Acq. Method**	FENOLIK_DMRM2021-31bilesen.m

Sample Chromatogram

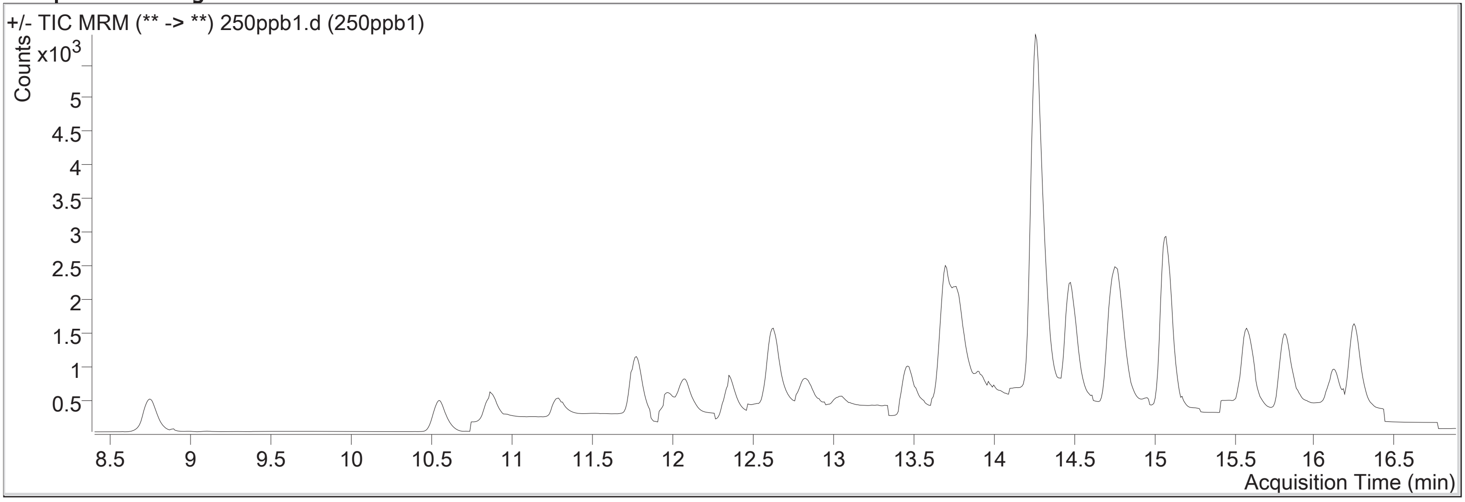

Compound	Transition	RT	Resp.	Final Conc	Units
Gallic acid	168.9 -> 125.0	8.750	2981	258.1111	ng/ml
Protocatechuic acid	152.9 -> 108.9	10.551	2690	248.4797	ng/ml
Pyrocatechol	109.0 -> 52.9	10.831	79	243.5675	ng/ml
3,4-Dihydroxyphenylacetic acid	167.0 -> 123.0	10.871	2023	246.3737	ng/ml
(+)-Catechin	289.0 -> 245.0	11.294	1006	280.2250	ng/ml
2,5-Dihydroxybenzoic acid	152.9 -> 109.0	11.971	1642	260.0777	ng/ml
Chlorogenic acid	355.0 -> 163.0	11.785	5367	257.7291	ng/ml
3-Hydroxybenzoic acid	137.0 -> 93.0	12.836	1239	246.6953	ng/ml
4-Hydroxybenzoic acid	136.9 -> 93.1	12.081	2764	241.2311	ng/ml
(-)-Epicatechin	291.0 -> 139.1	12.361	1942	247.7801	ng/ml
Caffeic acid	179.0 -> 135.0	12.633	6437	248.8870	ng/ml
Syringic acid	196.9 -> 181.9	12.757	190	267.7617	ng/ml
Vanillin	151.0 -> 136.0	13.053	942	261.5460	ng/ml
Verbascoside	623.0 -> 160.8	13.467	2508	237.9791	ng/ml
Taxifolin	303.0 -> 285.1	13.703	7653	258.6576	ng/ml
p-Coumaric acid	162.9 -> 119.0	13.792	6865	258.6283	ng/ml
Sinapic acid	222.9 -> 207.9	13.856	735	235.9321	ng/ml
Ferulic acid	193.0 -> 134.0	13.934	1264	242.7344	ng/ml
Luteolin 7-glucoside	447.1 -> 285.0	14.264	25423	245.6612	ng/ml
Hesperidin	611.1 -> 303.0	14.310	2063	254.5285	ng/ml
Hyperoside	465.1 -> 303.1	14.479	4890	251.4230	ng/ml
Rosmarinic acid	359.0 -> 160.9	14.481	3025	240.6256	ng/ml
Apigenin 7-glucoside	433.1 -> 271.0	14.747	8380	247.9263	ng/ml
Pinoresinol	357.0 -> 151.0	14.959	73	261.3065	ng/ml
2-Hydroxycinnamic acid	162.9 -> 119.1	14.786	5293	247.7823	ng/ml
Eriodictyol	287.0 -> 151.0	15.078	6962	257.2121	ng/ml
Quercetin	301.0 -> 151.0	15.578	5693	241.7059	ng/ml
Luteolin	287.0 -> 153.1	15.825	3905	241.7447	ng/ml
Kaempferol	285.0 -> 229.1	16.130	1210	268.8920	ng/ml

Compound	Transition	RT	Resp.	Final Conc	Units
Apigenin	271.0 -> 153.0	16.251	4746	251.0300	ng/ml

Gallic acid

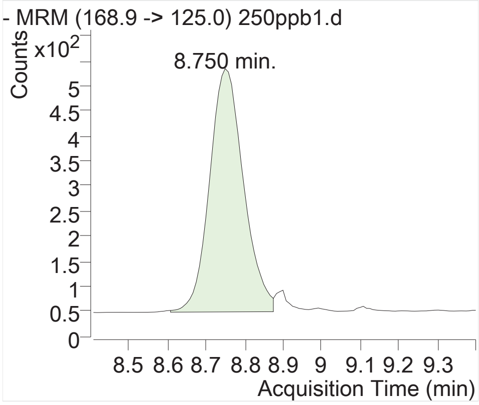

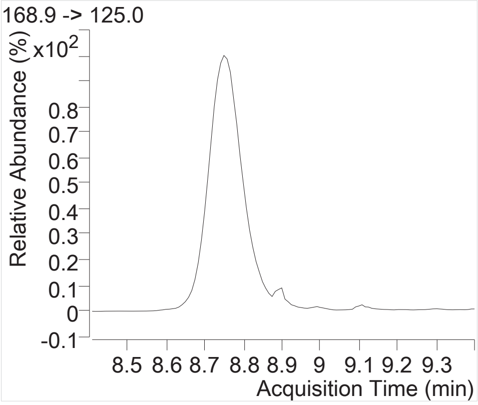

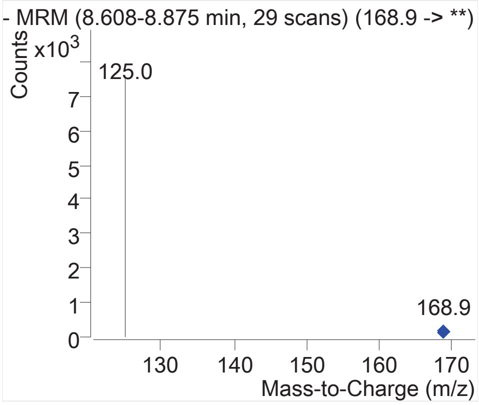

Protocatechuic acid

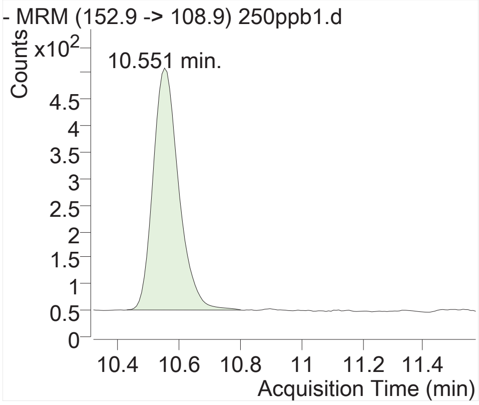

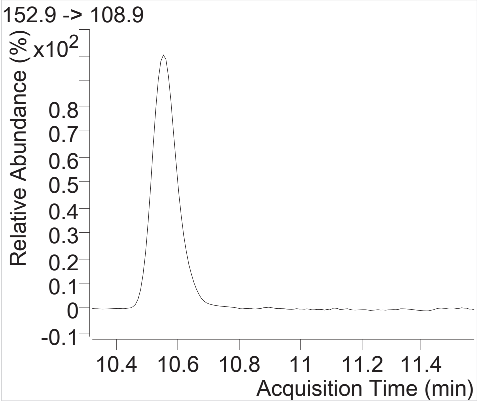

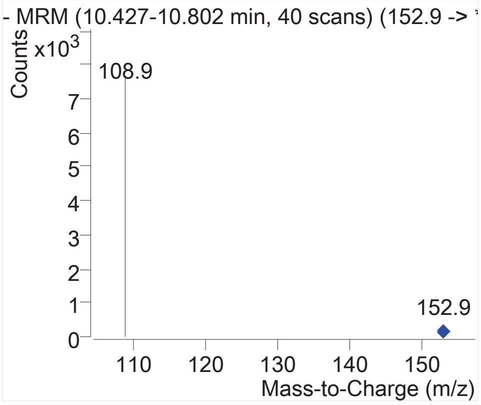

Pyrocatechol

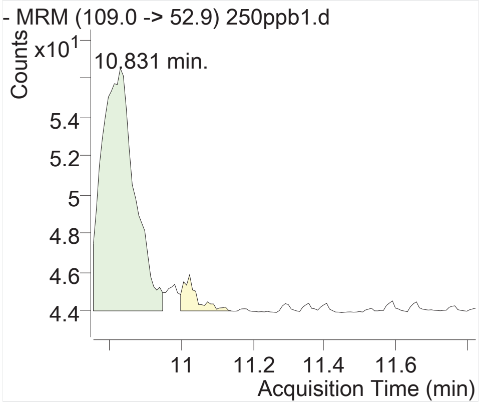

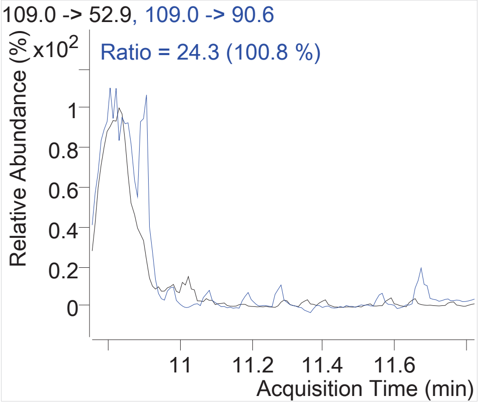

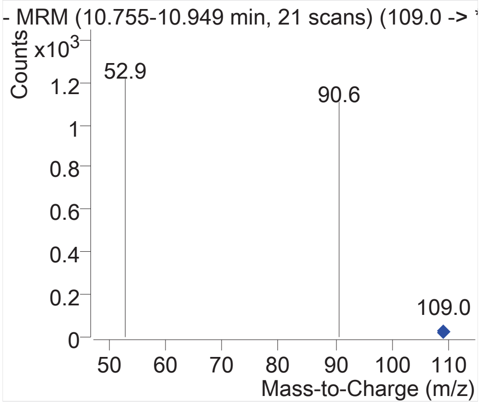

3,4-Dihydroxyphenylacetic acid

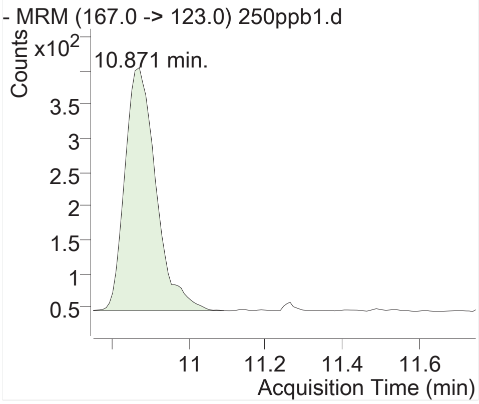

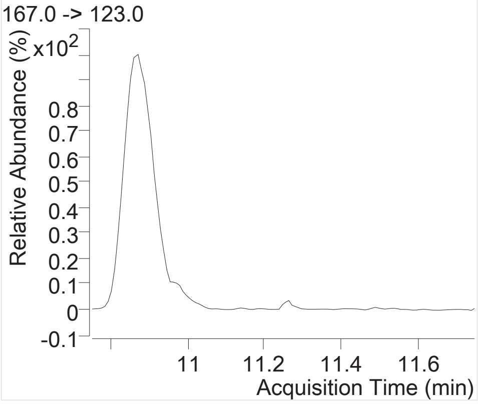

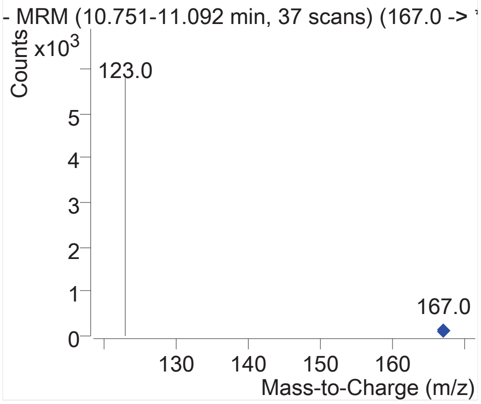

(+)-Catechin

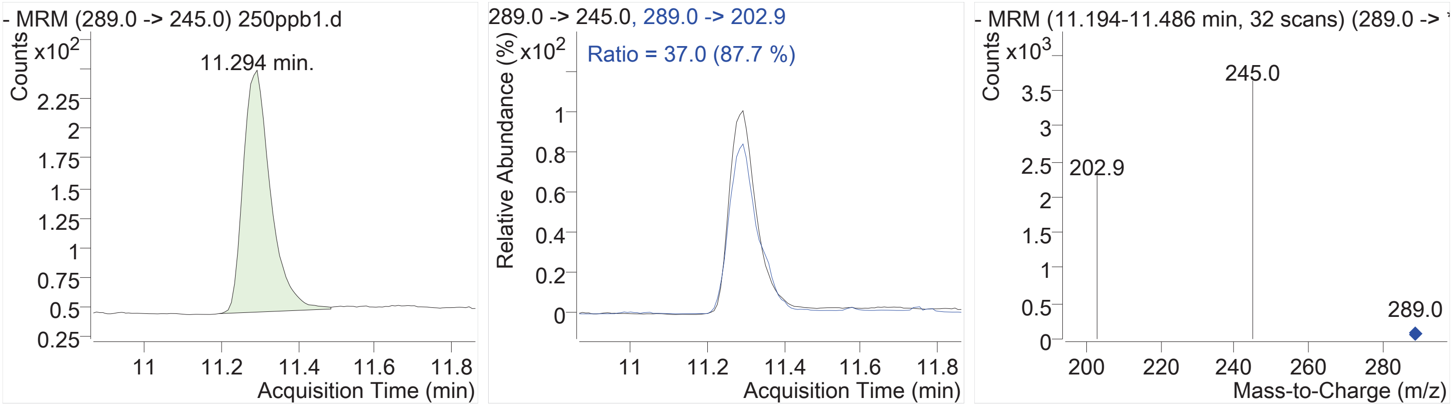

2,5-Dihydroxybenzoic acid

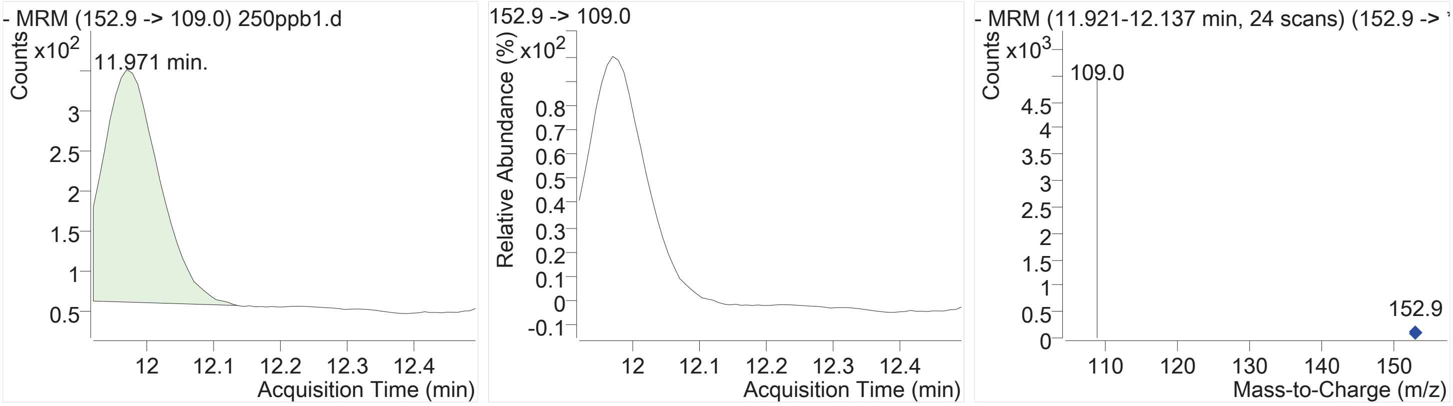

Chlorogenic acid

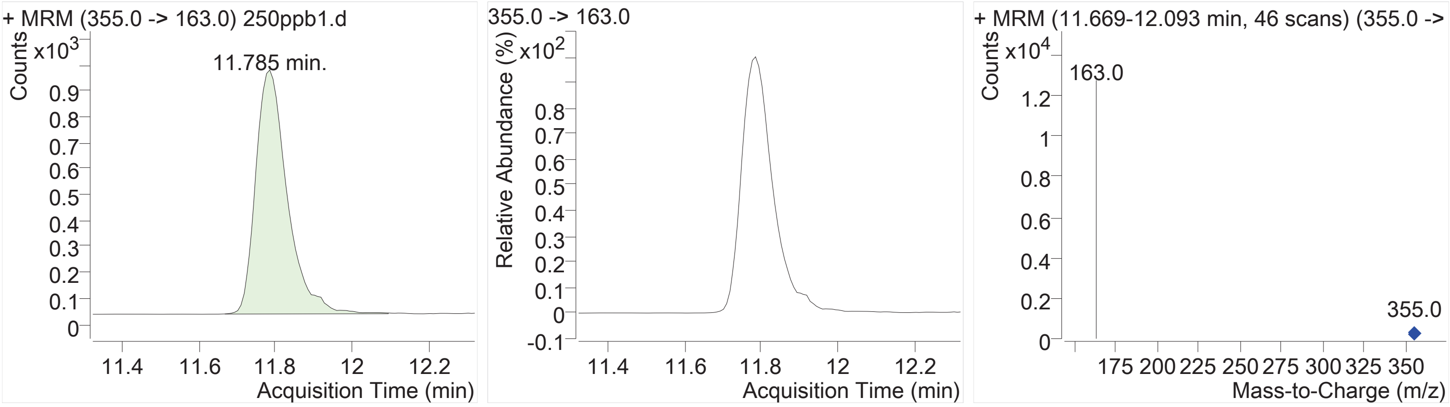

3-Hydroxybenzoic acid

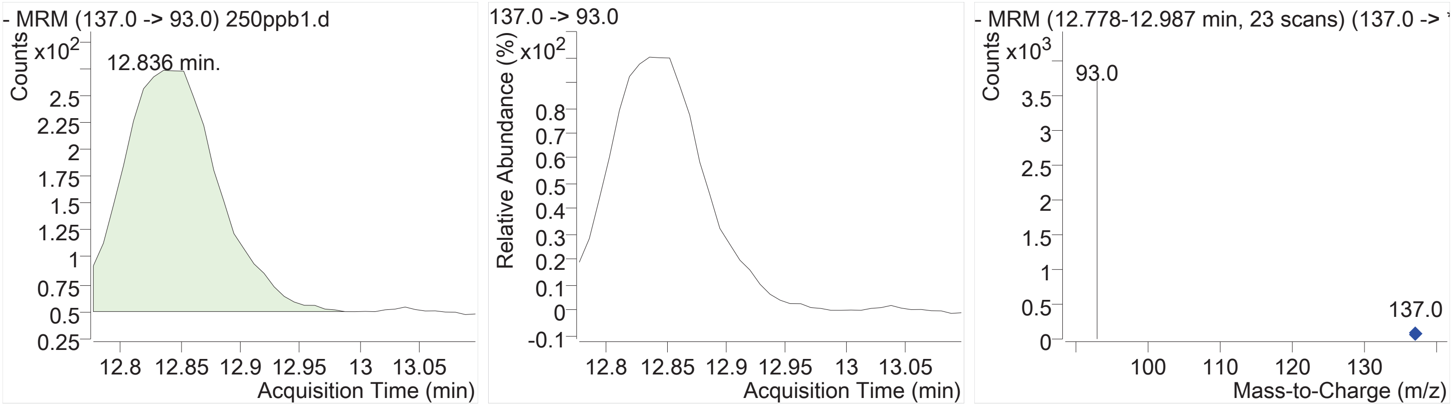

4-Hydroxybenzoic acid

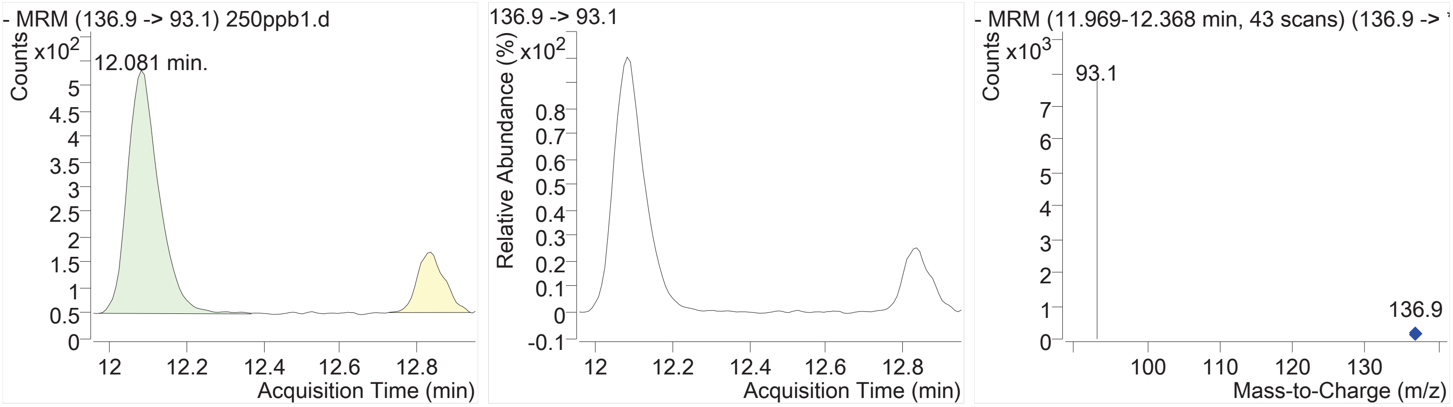

(-)-Epicatechin

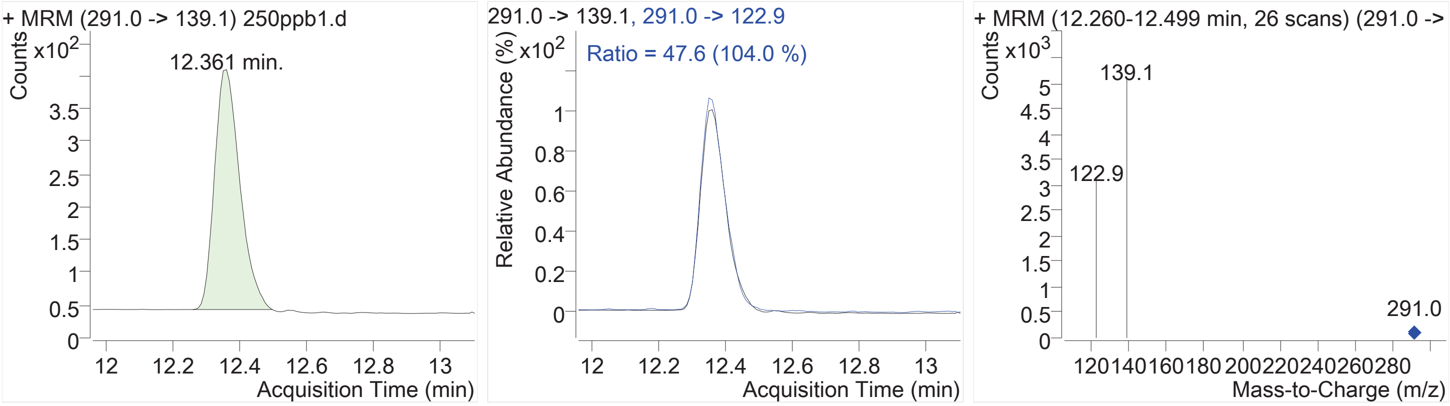

Caffeic acid

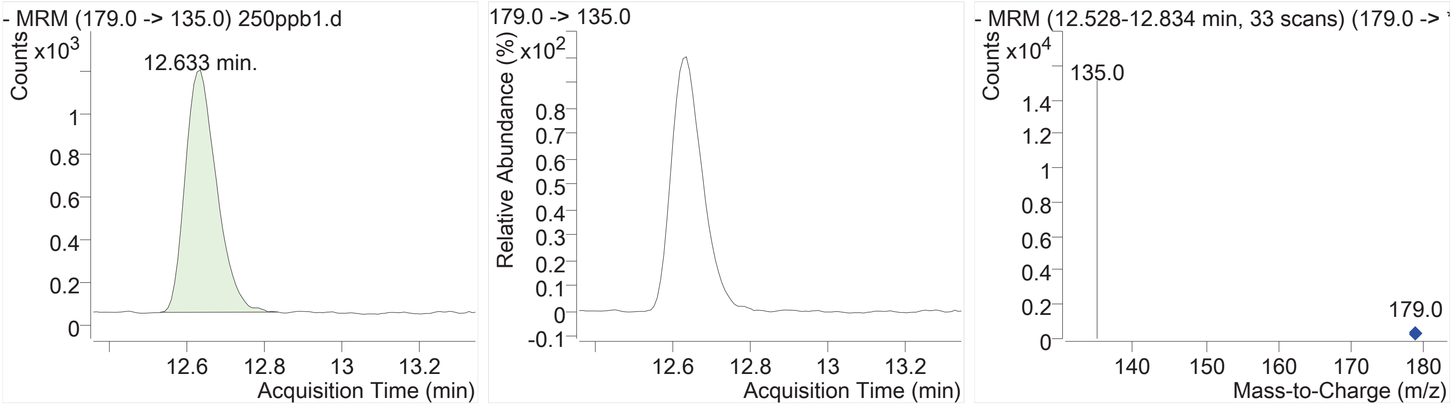

Syringic acid

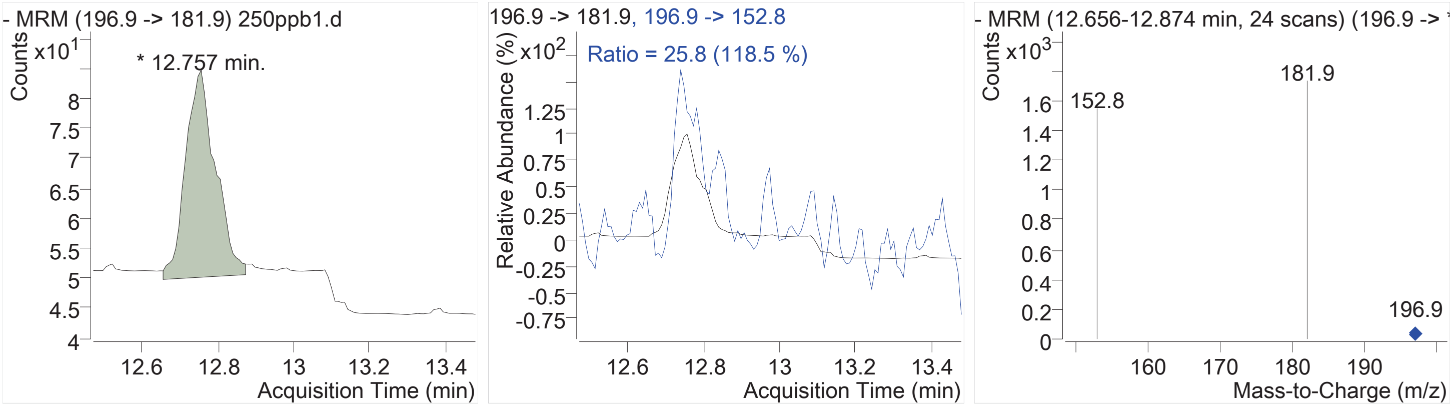

Vanillin

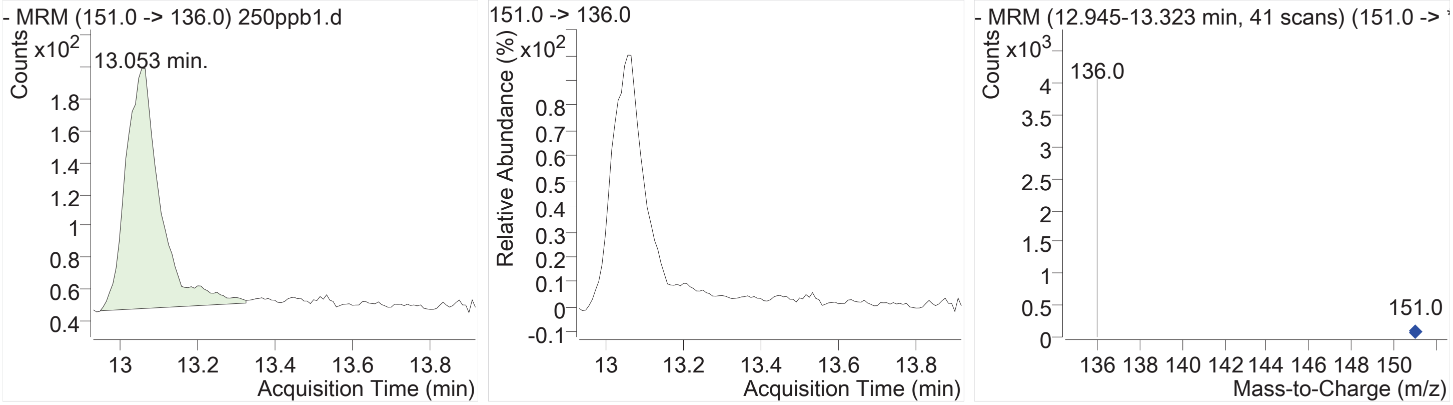

Verbascoside

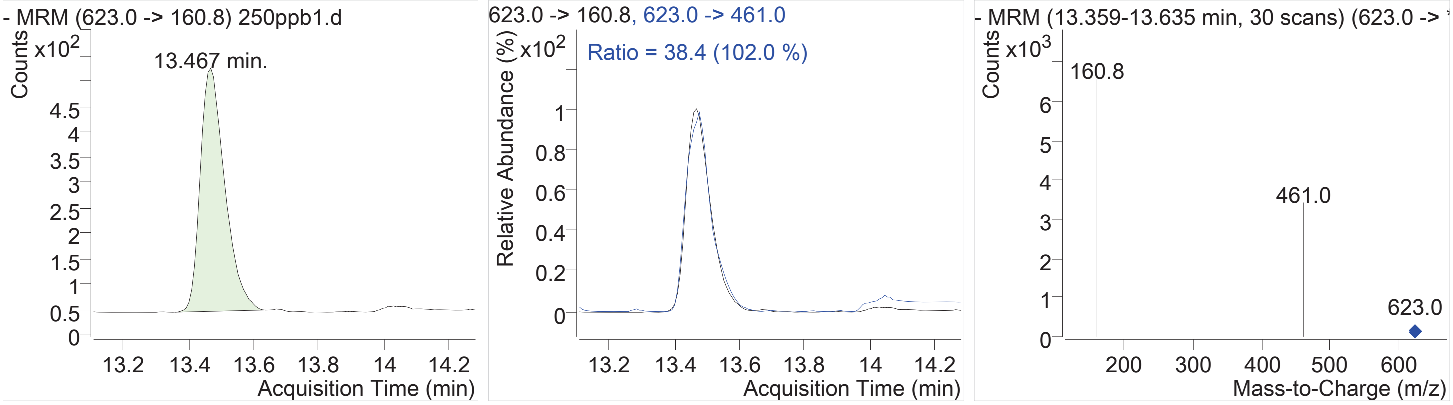

Taxifolin

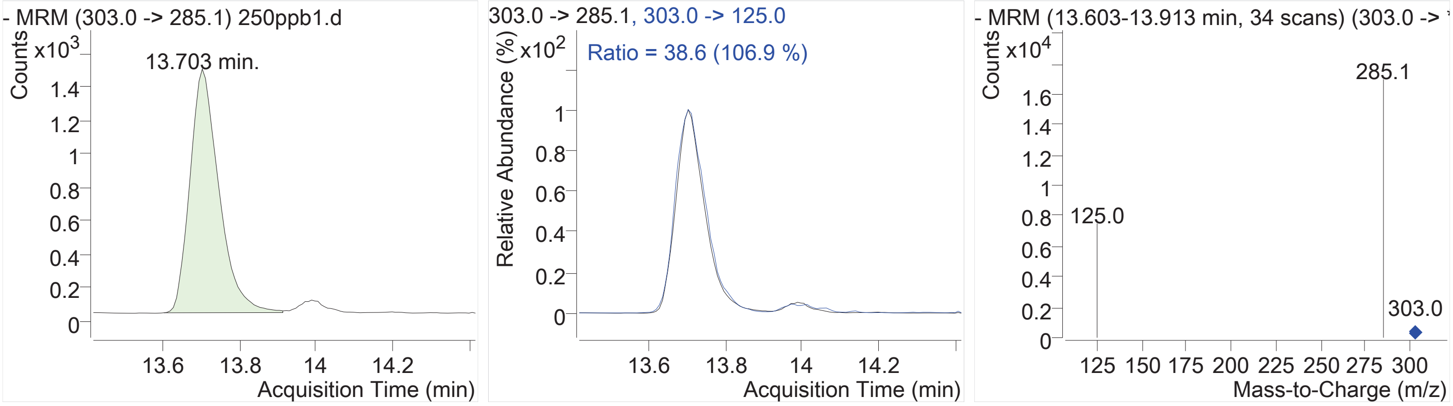

p-Coumaric acid

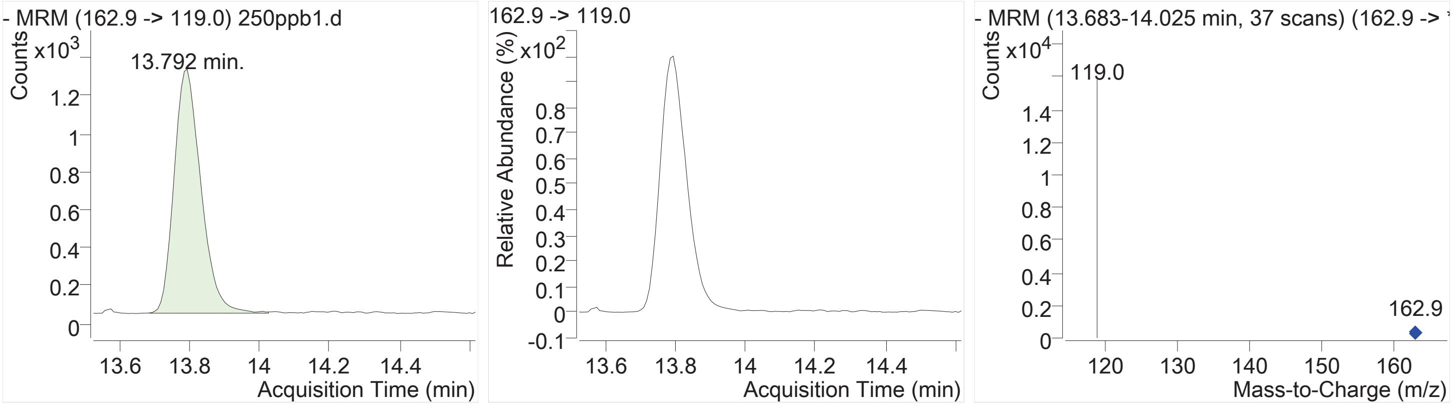

Agilent Technologies

Sinapic acid

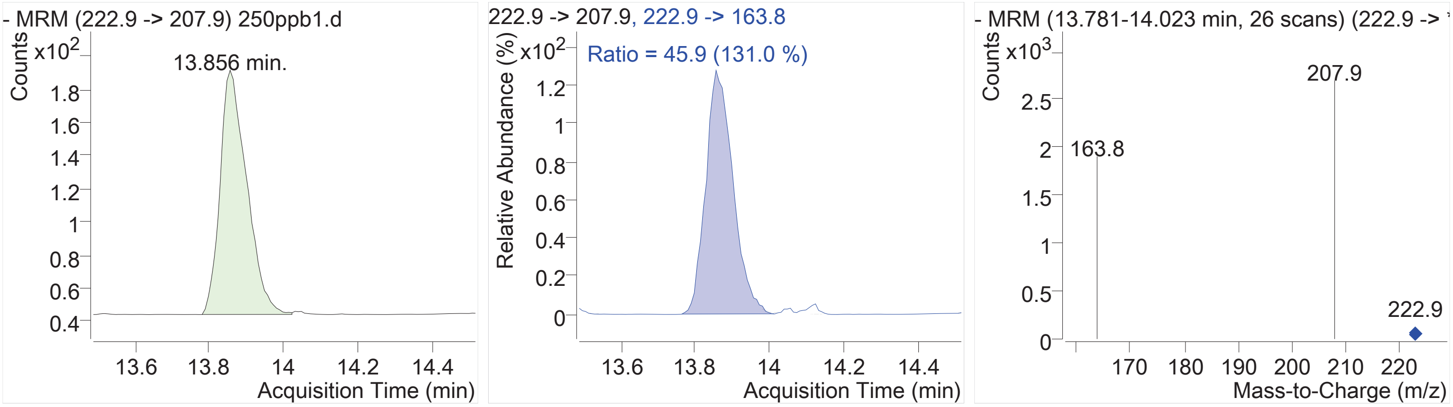

Ferulic acid

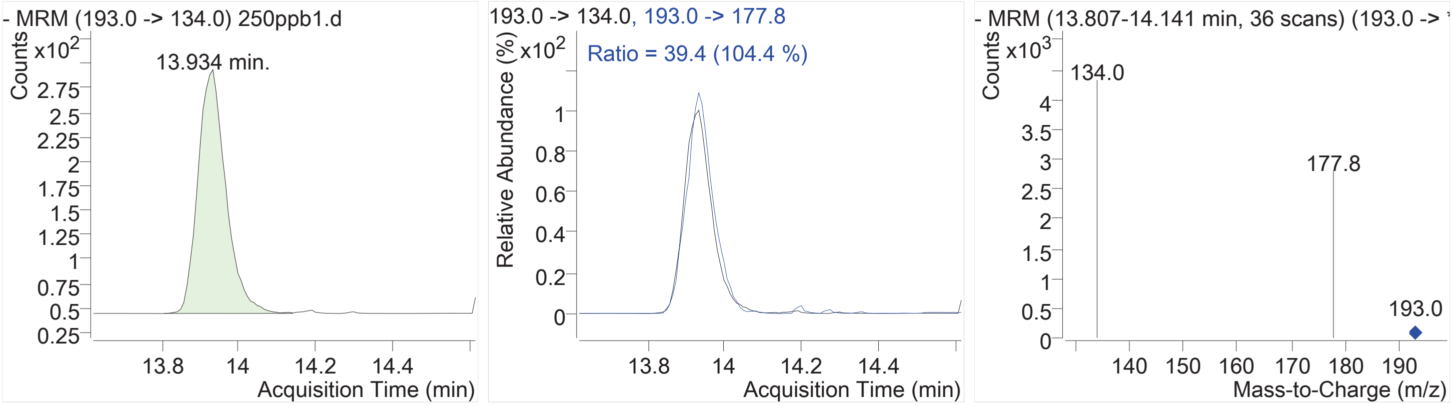

Luteolin 7-glucoside

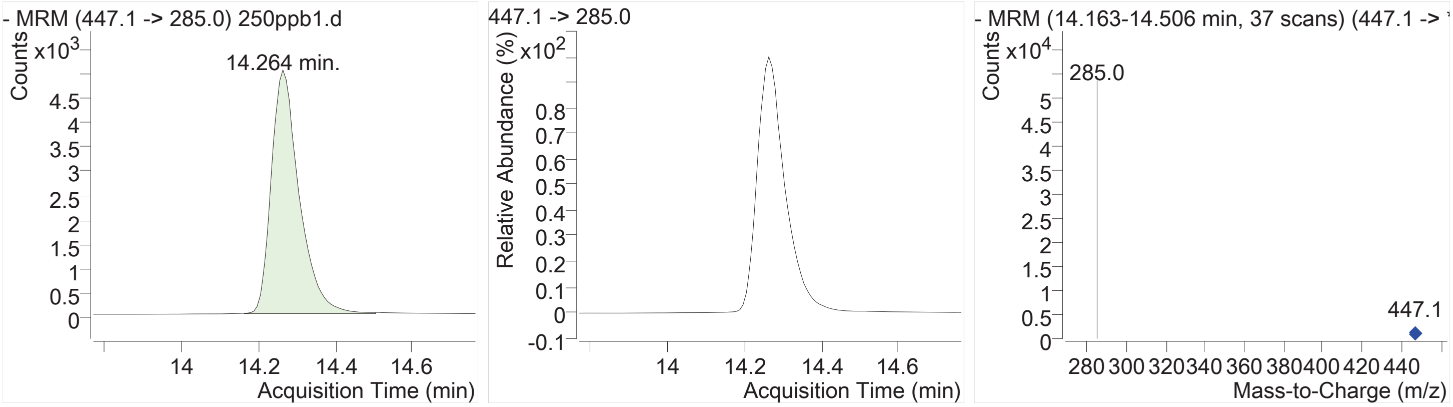

Hesperidin

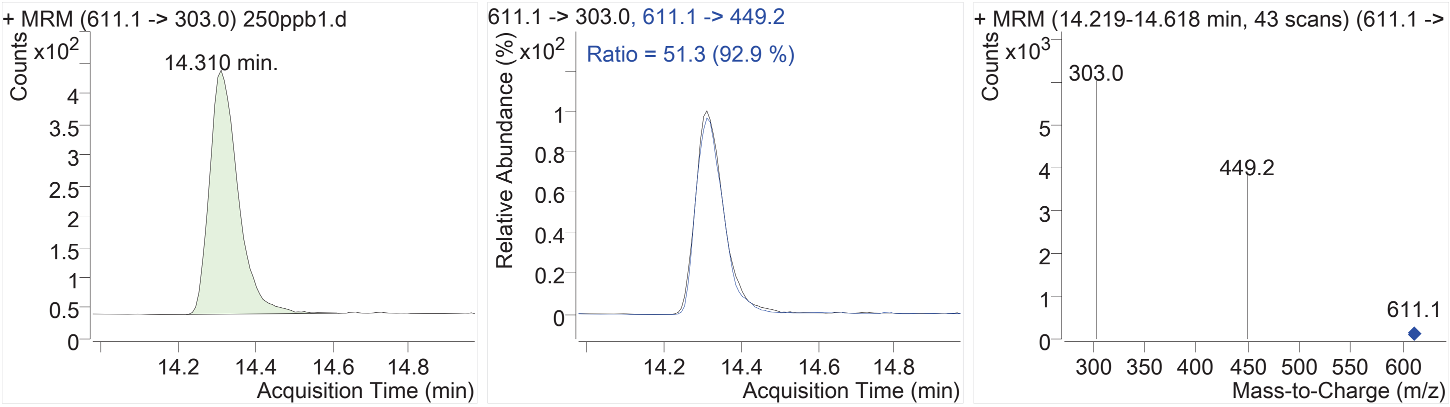

Hyperoside

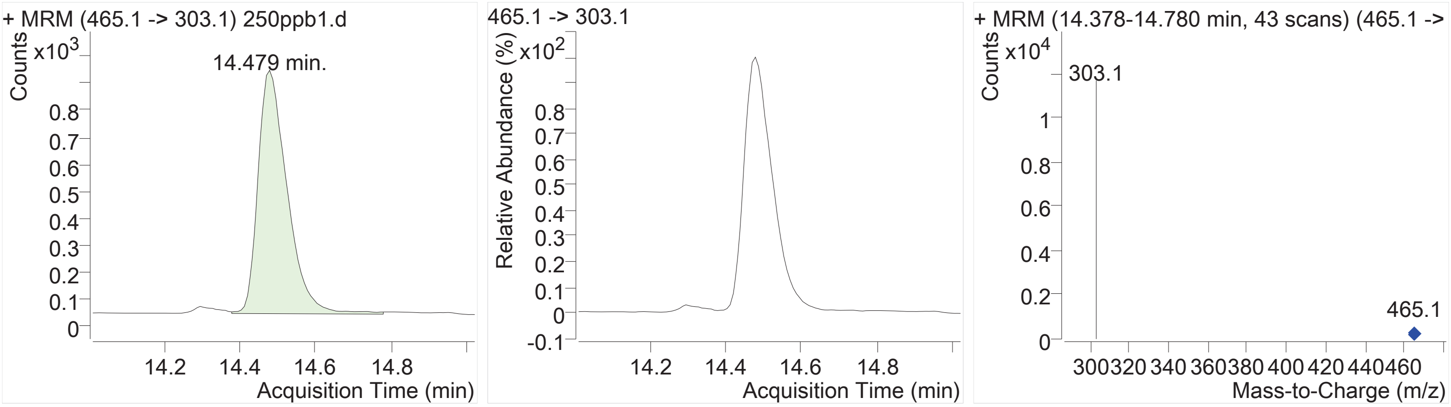

Rosmarinic acid

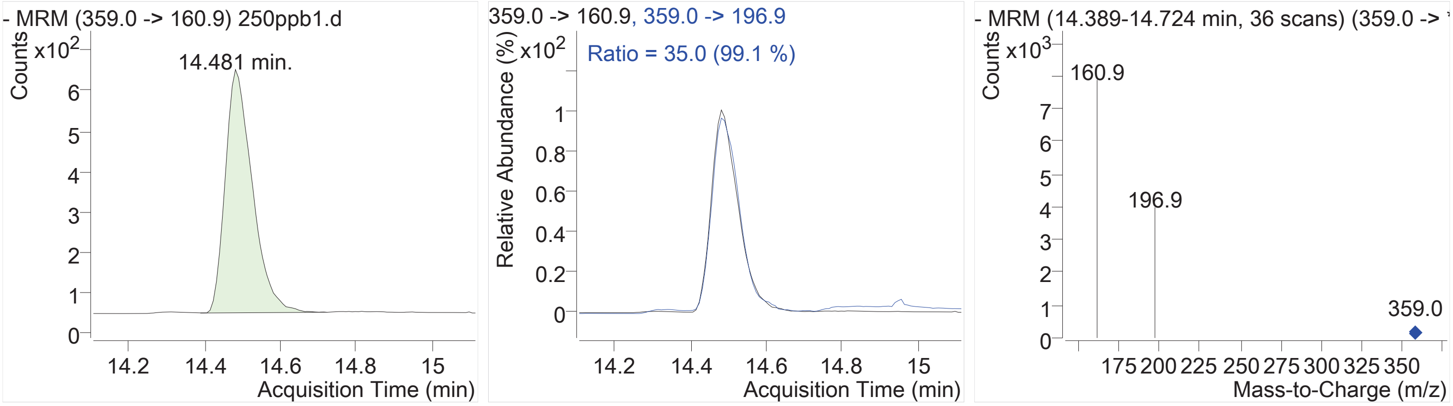

Apigenin 7-glucoside

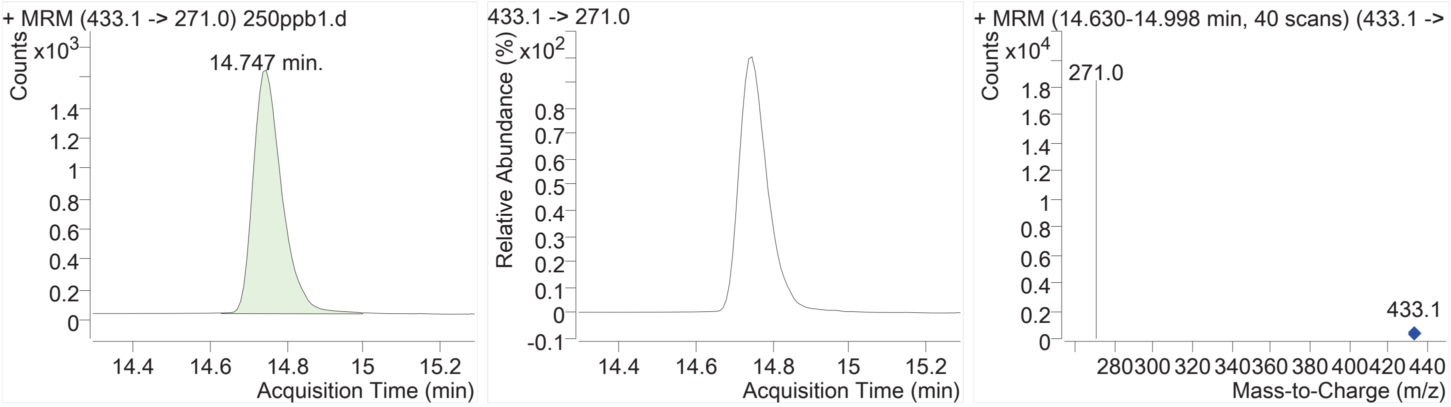

Pinoresinol

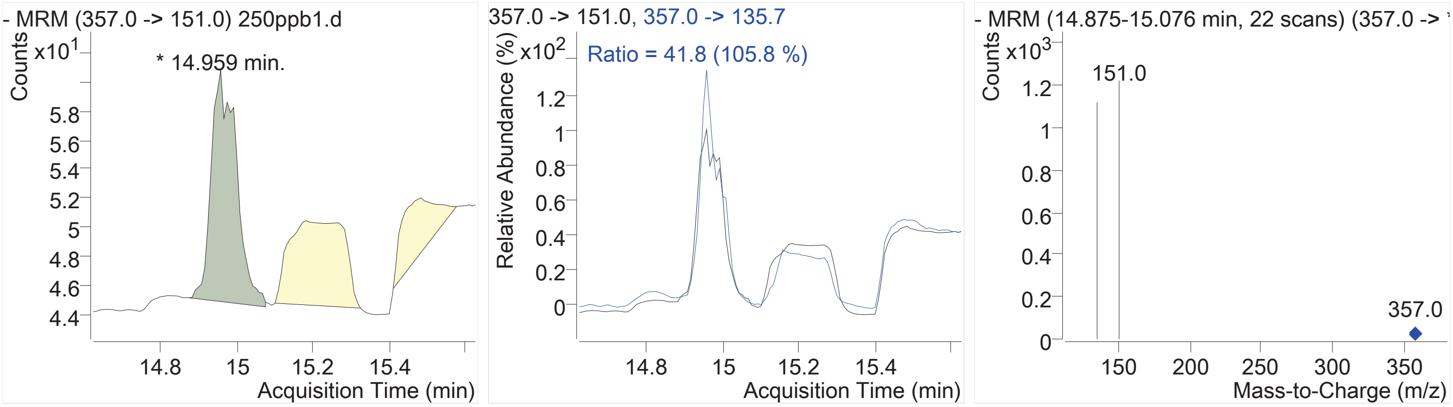

2-Hydroxycinnamic acid

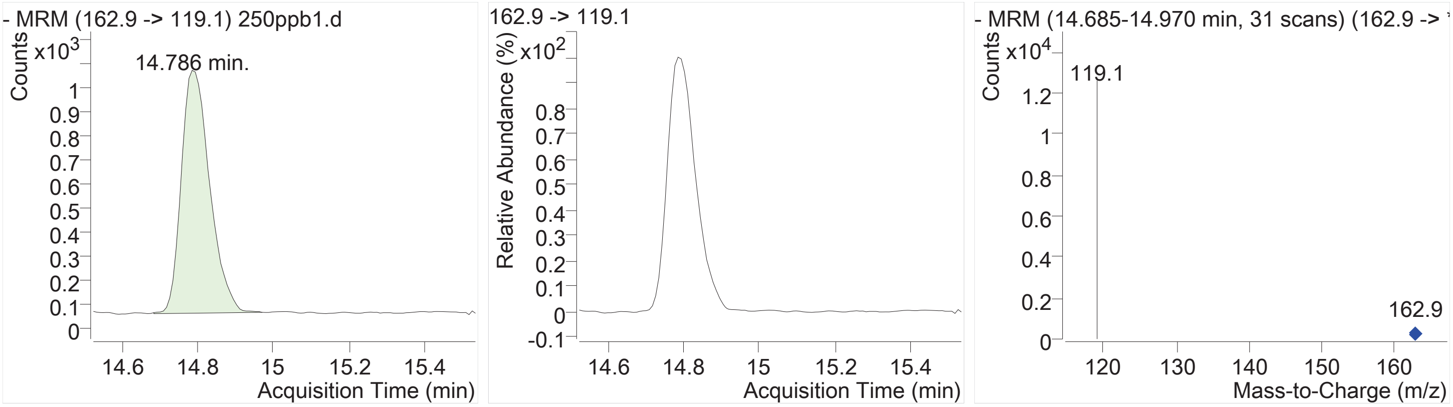

Eriodictyol

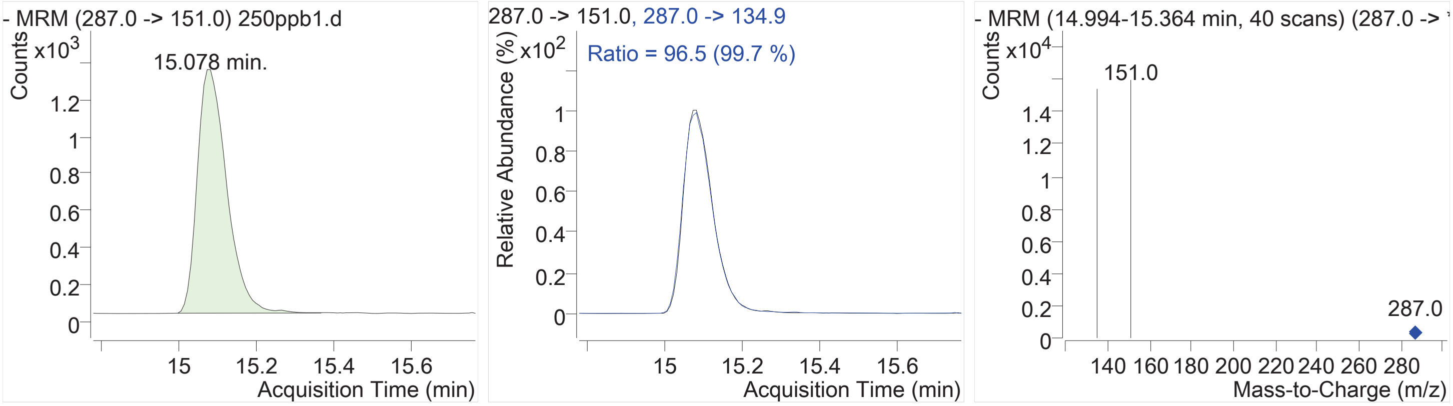

Quercetin

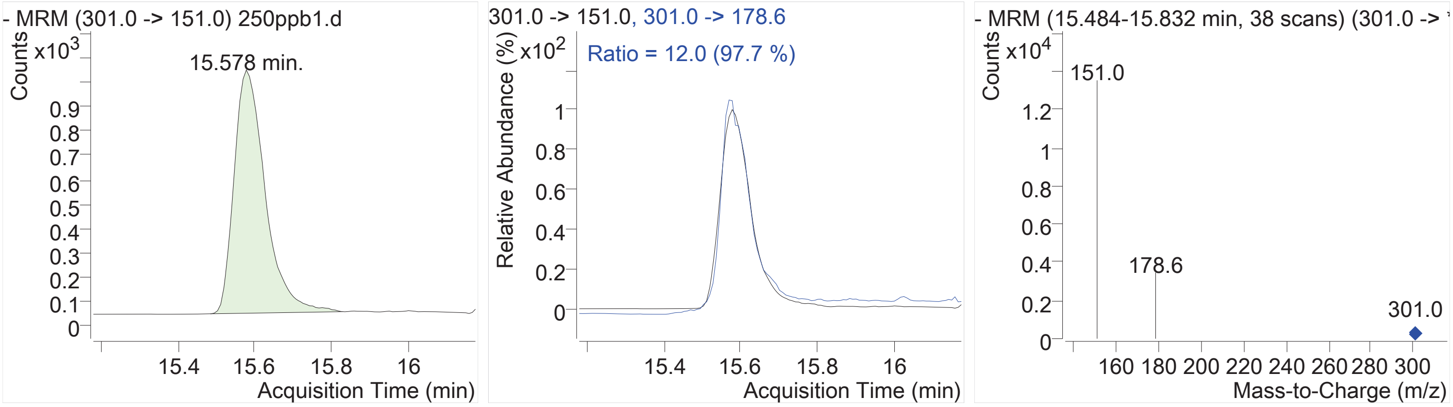

Luteolin

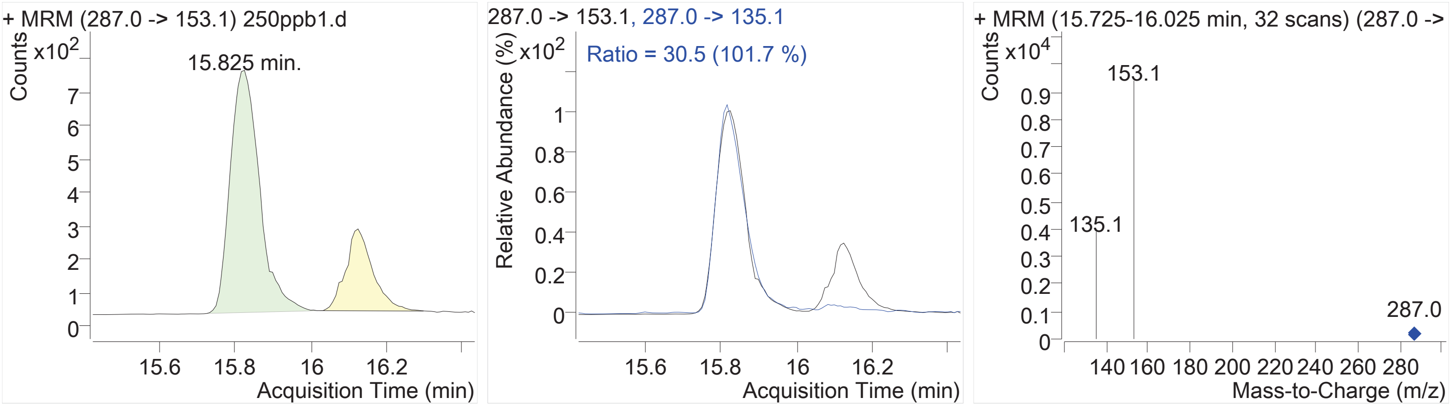

Kaempferol

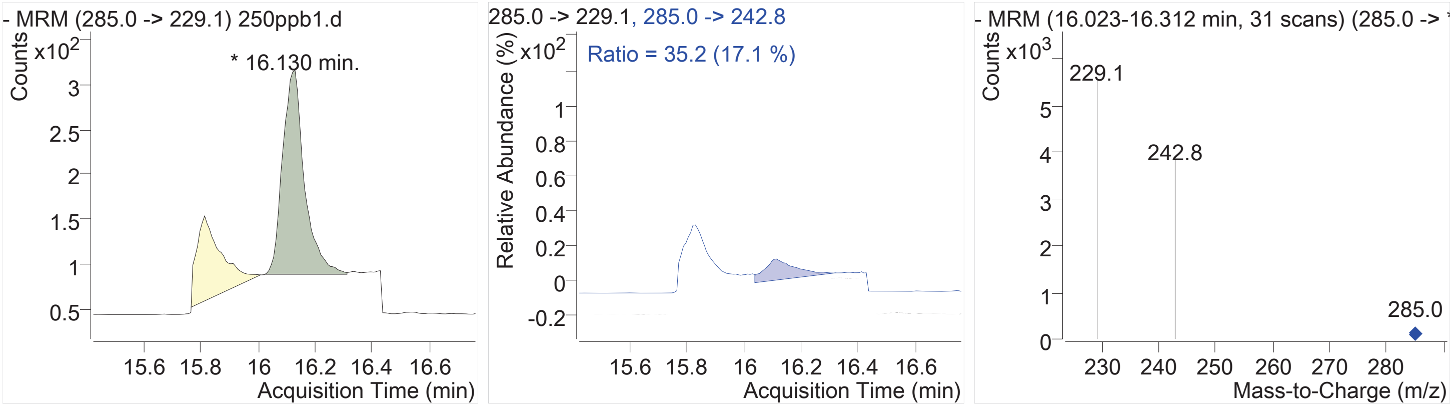

Apigenin

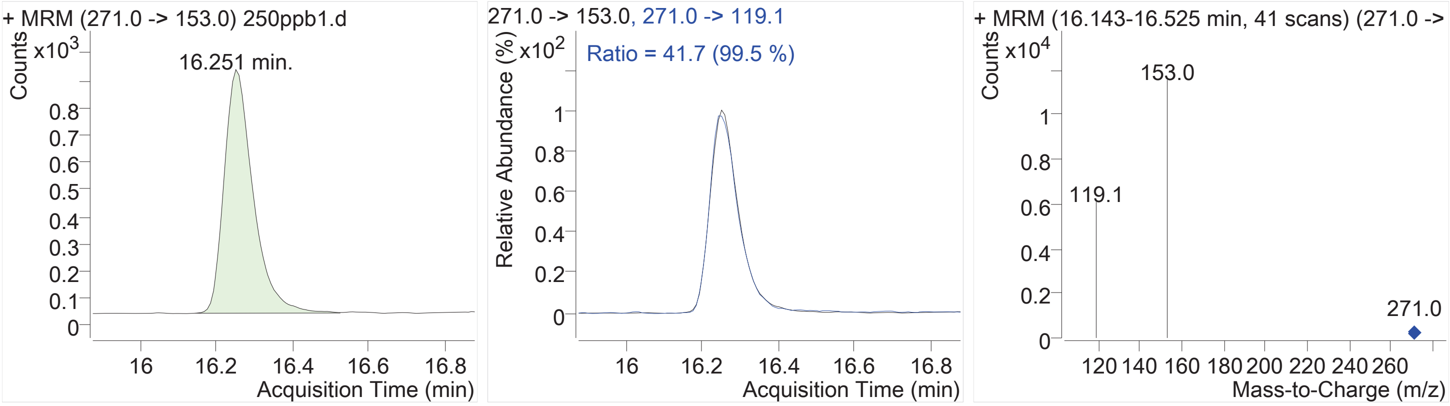

Quantitative Analysis Complete Report

Agilent Technologies

**Batch Path**	D:\MassHunter\Data\2022ekim\061022cengizhoca\QuantResults\071022.batch.bin		
**Analysis Time**	10/11/2022 1:33:26 PM	**Analyst Name**	Defam-PC\admin
**Report Time**	10/11/2022 1:34:20 PM	**Reporter Name**	admin
**Last Calib Update**	10/11/2022 1:33:17 PM	**Batch State**	Processed
**Quant Batch Version**	B.07.01	**Quant Report Version**	B.07.01

**Acq. Time**	10/6/2022 7:31:58 PM	**Data File**	250ppb2.d
**Sample Type**	Cal	**Sample Name**	250ppb2
**Dilution**	1	**Acq. Method**	FENOLIK_DMRM2021-31bilesen.m

Sample Chromatogram

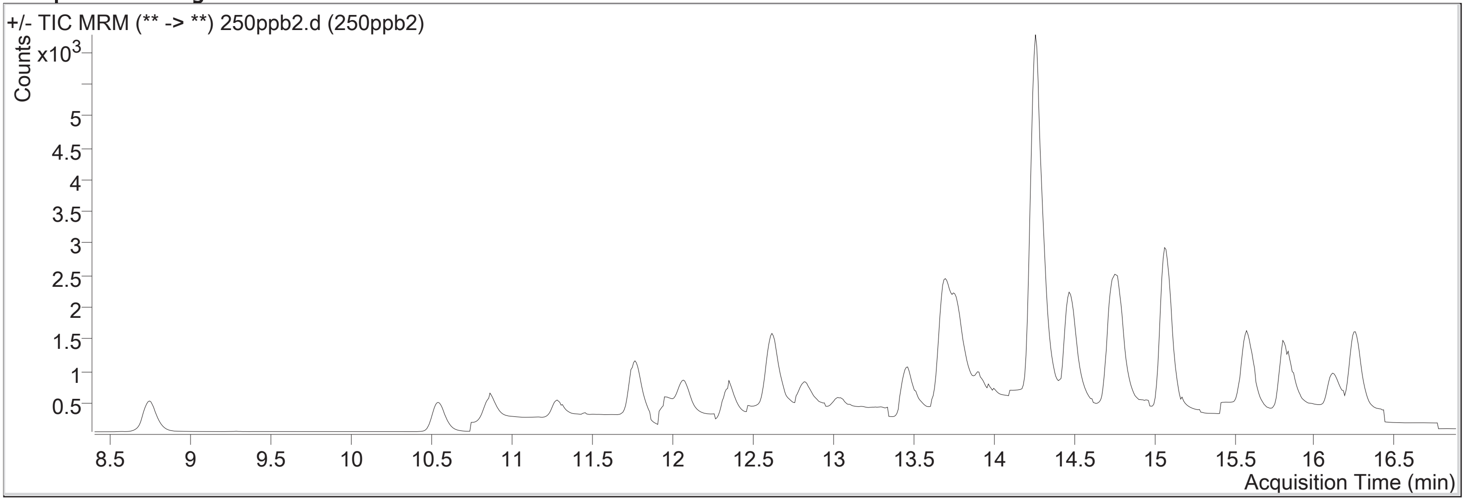

Compound	Transition	RT	Resp.	Final Conc	Units
Gallic acid	168.9 -> 125.0	8.750	2978	257.8373	ng/ml
Protocatechuic acid	152.9 -> 108.9	10.543	2679	247.4624	ng/ml
Pyrocatechol	109.0 -> 52.9	10.814	78	240.7014	ng/ml
3,4-Dihydroxyphenylacetic acid	167.0 -> 123.0	10.860	2093	255.2710	ng/ml
(+)-Catechin	289.0 -> 245.0	11.286	994	276.3034	ng/ml
2,5-Dihydroxybenzoic acid	152.9 -> 109.0	11.954	1503	238.0886	ng/ml
Chlorogenic acid	355.0 -> 163.0	11.777	5356	257.2116	ng/ml
3-Hydroxybenzoic acid	137.0 -> 93.0	12.836	1158	231.6025	ng/ml
4-Hydroxybenzoic acid	136.9 -> 93.1	12.072	2922	255.9975	ng/ml
(-)-Epicatechin	291.0 -> 139.1	12.351	1912	244.0519	ng/ml
Caffeic acid	179.0 -> 135.0	12.624	6580	255.0503	ng/ml
Syringic acid	196.9 -> 181.9	12.748	167	235.9900	ng/ml
Vanillin	151.0 -> 136.0	13.037	884	244.1660	ng/ml
Verbascoside	623.0 -> 160.8	13.459	2607	247.8597	ng/ml
Taxifolin	303.0 -> 285.1	13.695	7668	259.2388	ng/ml
p-Coumaric acid	162.9 -> 119.0	13.784	6933	261.2789	ng/ml
Sinapic acid	222.9 -> 207.9	13.856	803	257.6513	ng/ml
Ferulic acid	193.0 -> 134.0	13.916	1327	254.9409	ng/ml
Luteolin 7-glucoside	447.1 -> 285.0	14.264	26577	258.3454	ng/ml
Hesperidin	611.1 -> 303.0	14.310	1805	223.2001	ng/ml
Hyperoside	465.1 -> 303.1	14.479	4636	238.5983	ng/ml
Rosmarinic acid	359.0 -> 160.9	14.481	3169	252.1411	ng/ml
Apigenin 7-glucoside	433.1 -> 271.0	14.739	8641	255.6770	ng/ml
Pinoresinol	357.0 -> 151.0	14.967	48	162.4476	ng/ml
2-Hydroxycinnamic acid	162.9 -> 119.1	14.786	5239	245.2251	ng/ml
Eriodictyol	287.0 -> 151.0	15.070	7007	259.0168	ng/ml
Quercetin	301.0 -> 151.0	15.578	5892	250.3854	ng/ml
Luteolin	287.0 -> 153.1	15.808	3846	238.0102	ng/ml
Kaempferol	285.0 -> 229.1	16.121	1107	243.0488	ng/ml

Compound	Transition	RT	Resp.	Final Conc	Units
Apigenin	271.0 -> 153.0	16.260	4753	251.3925	ng/ml

Gallic acid

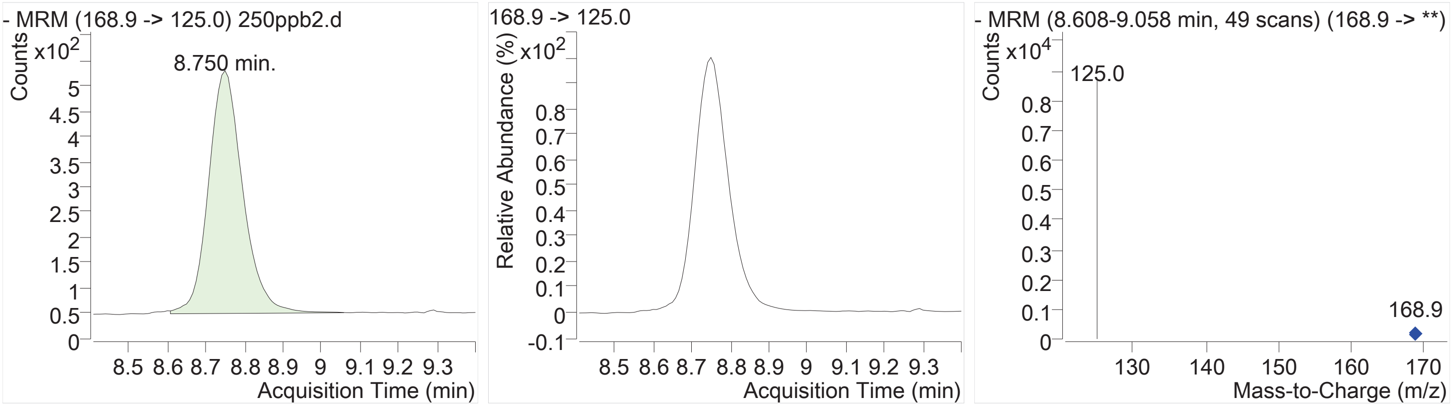

Protocatechuic acid

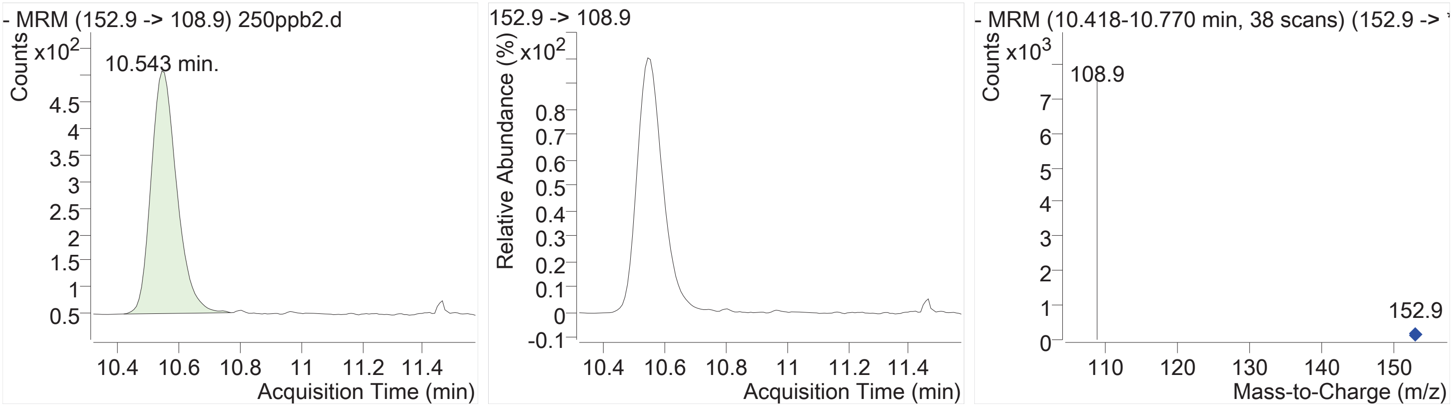

Pyrocatechol

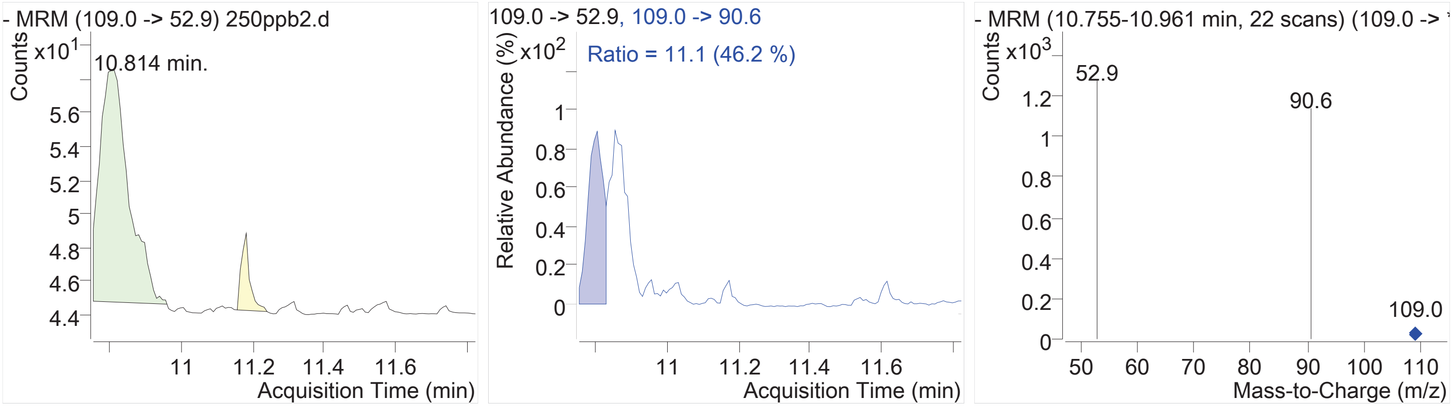

3,4-Dihydroxyphenylacetic acid

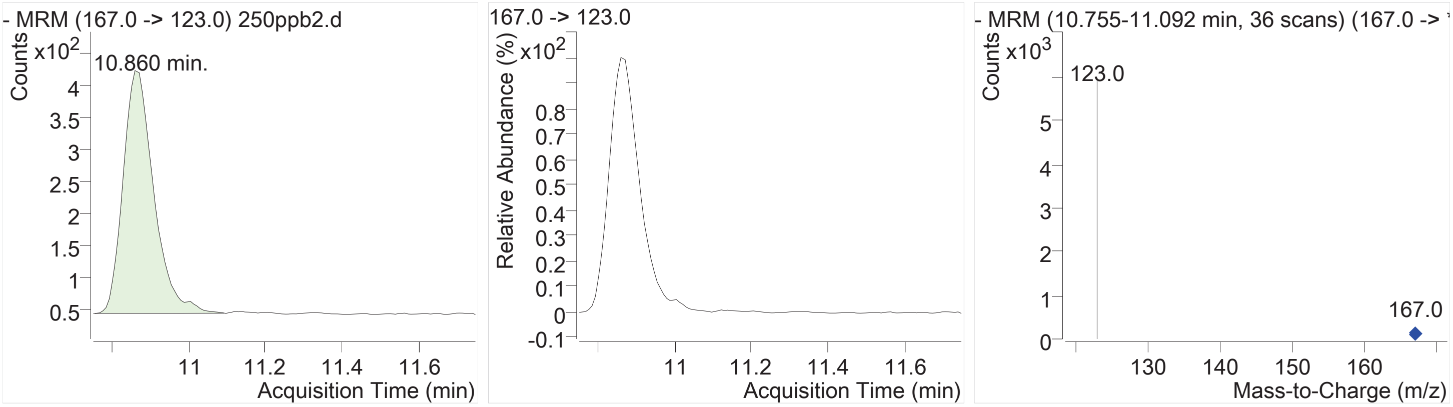

(+)-Catechin

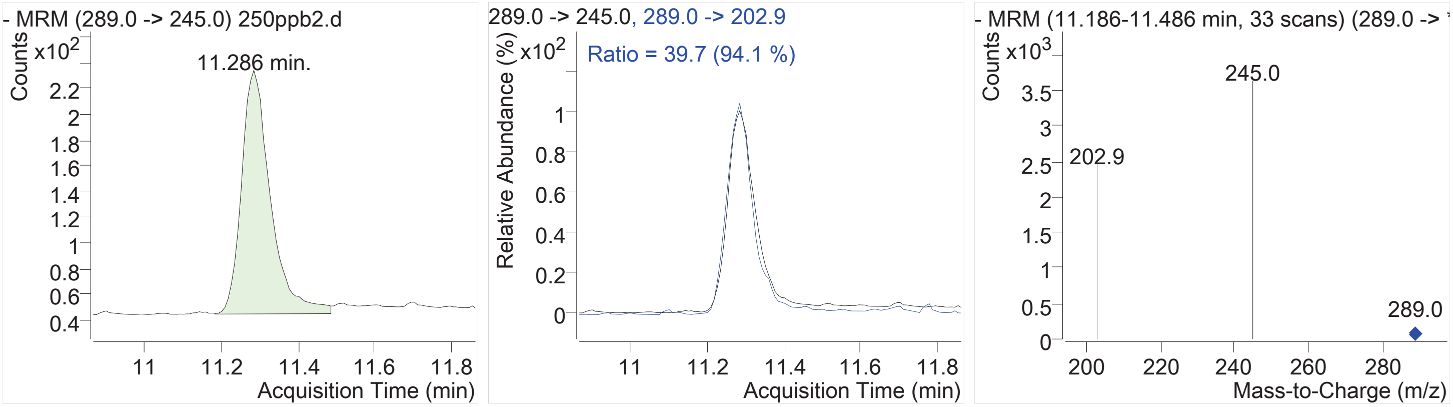

2,5-Dihydroxybenzoic acid

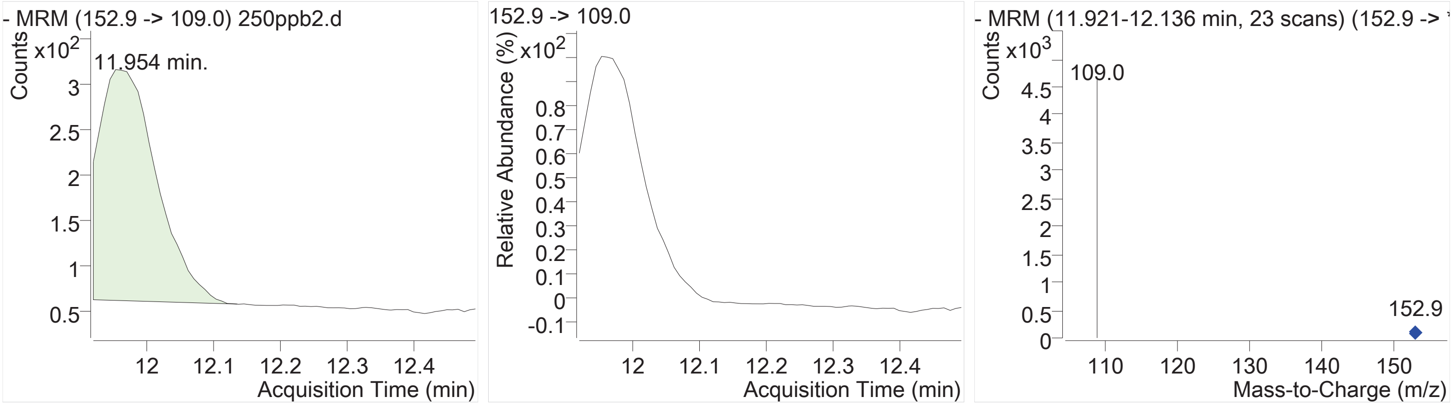

Chlorogenic acid

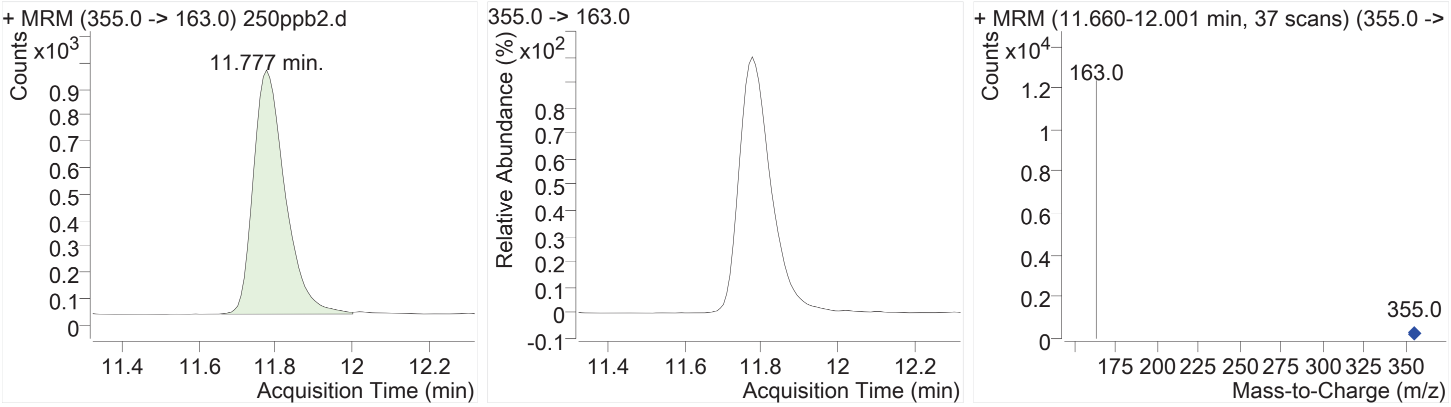

3-Hydroxybenzoic acid

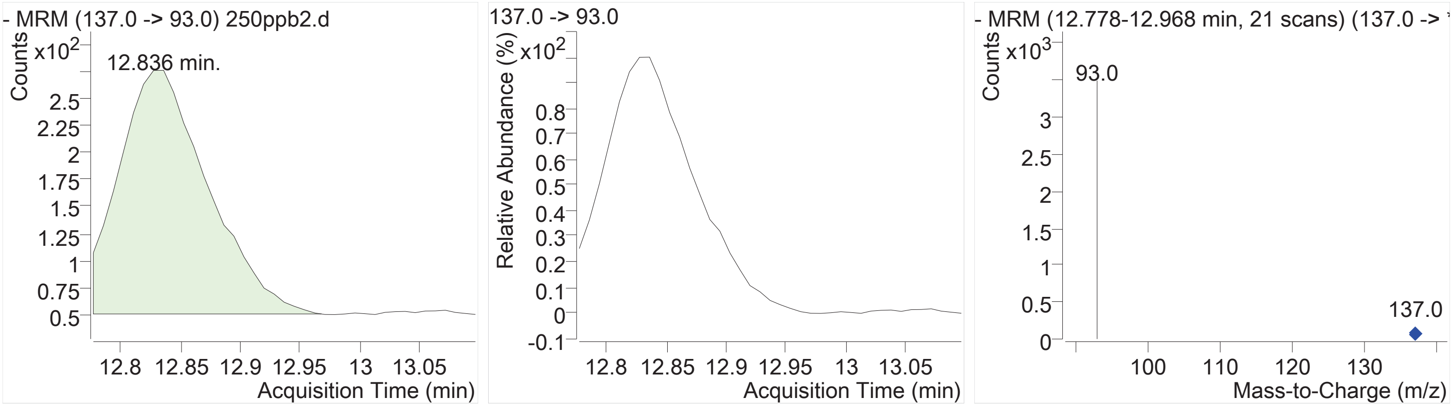

4-Hydroxybenzoic acid

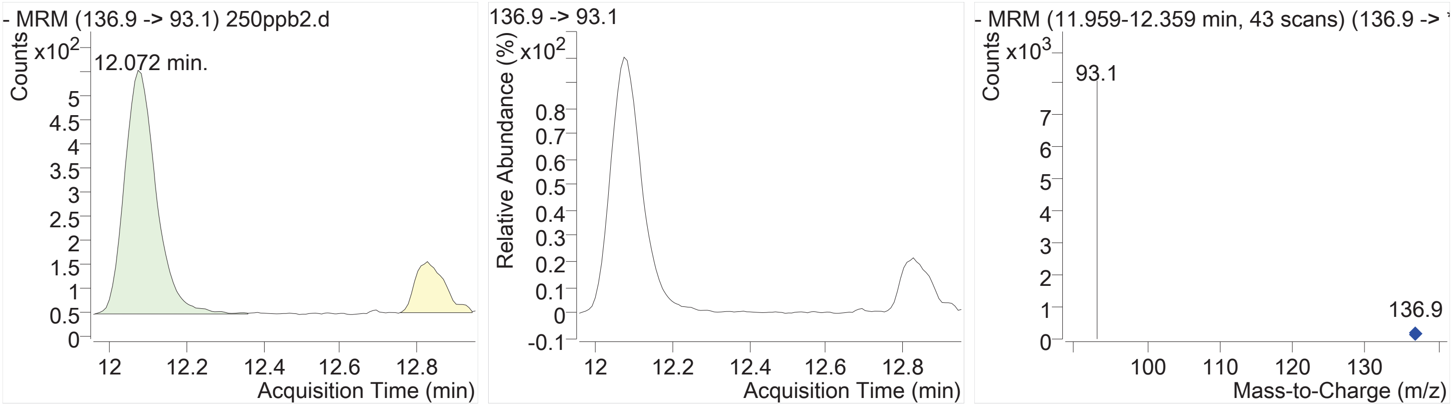

(-)-Epicatechin

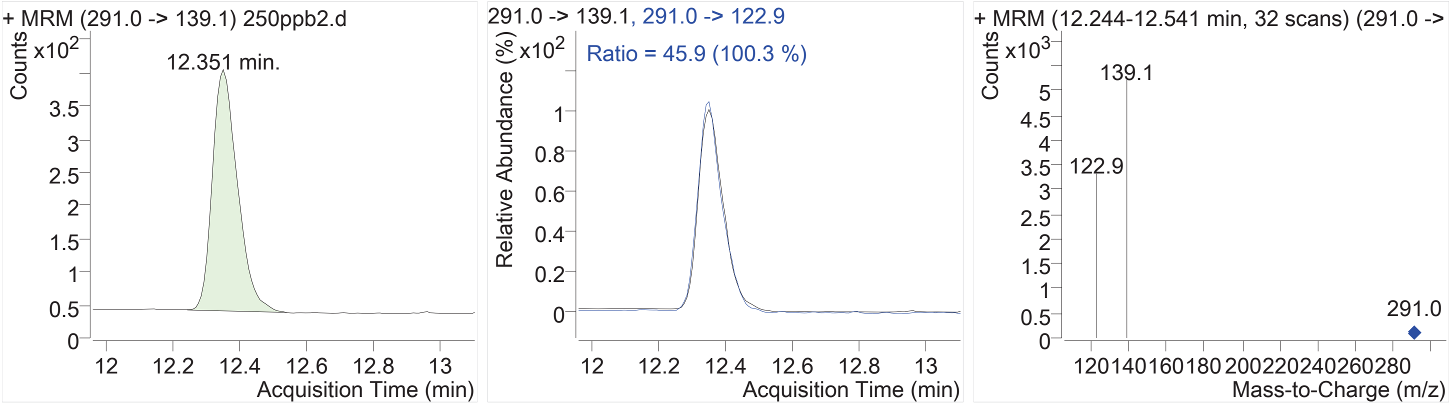

Caffeic acid

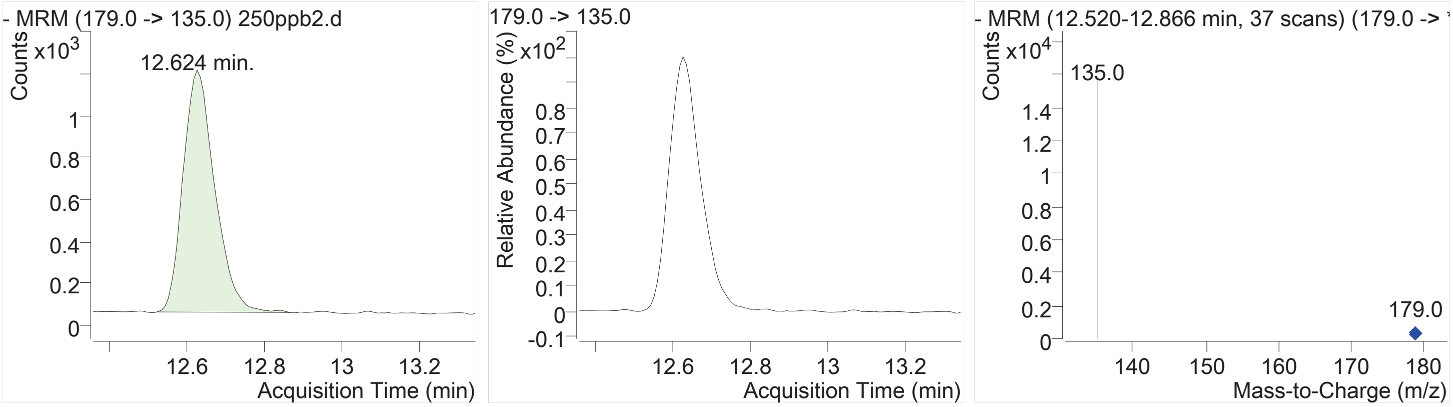

Syringic acid

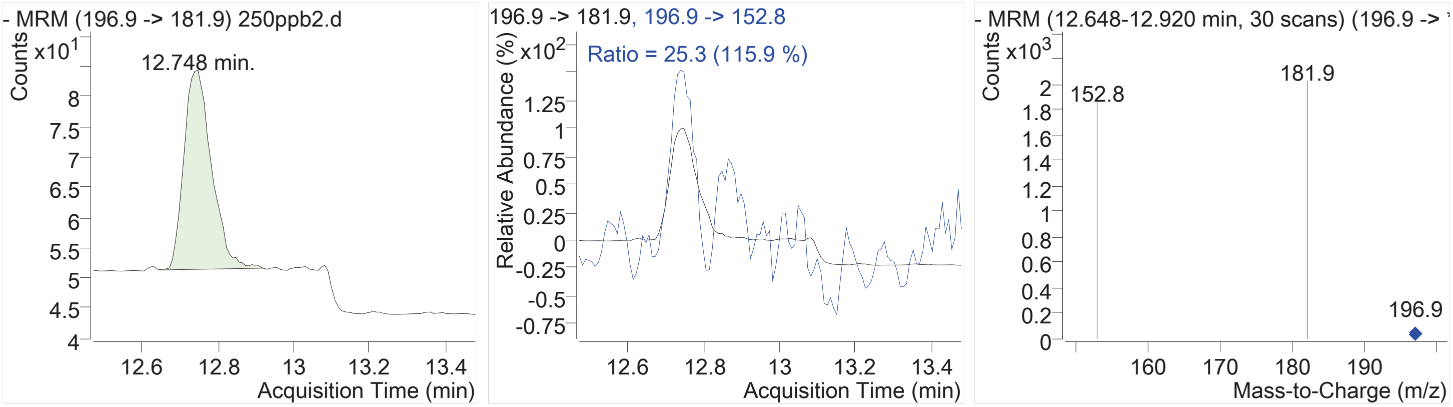

Agilent Technologies

Vanillin

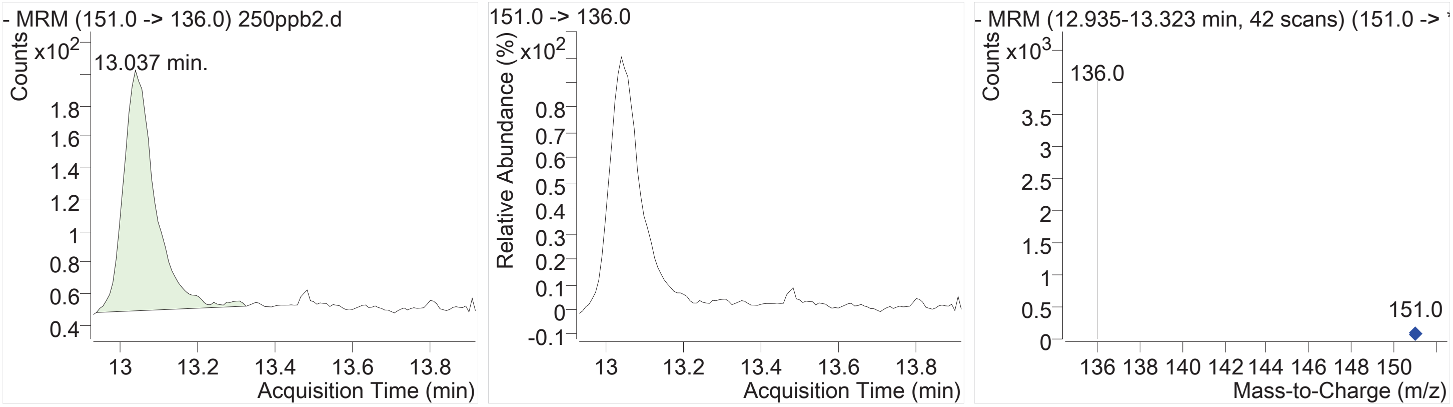

Verbascoside

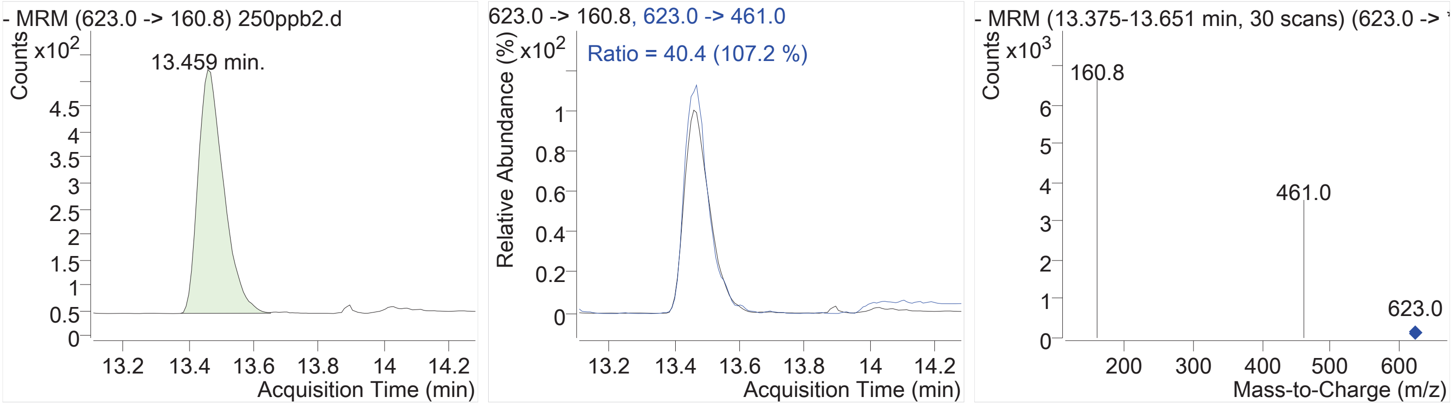

Taxifolin

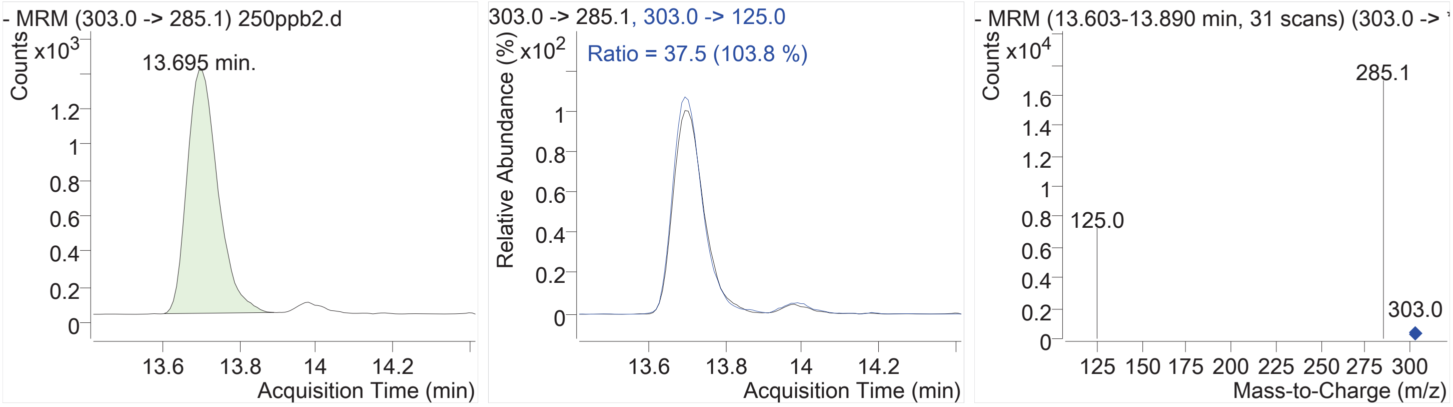

p-Coumaric acid

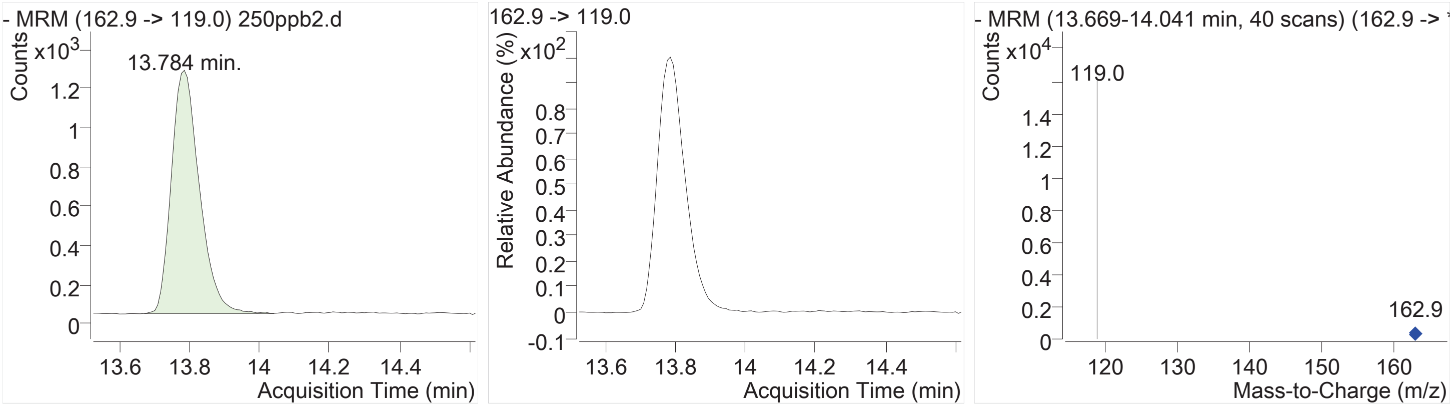

Sinapic acid

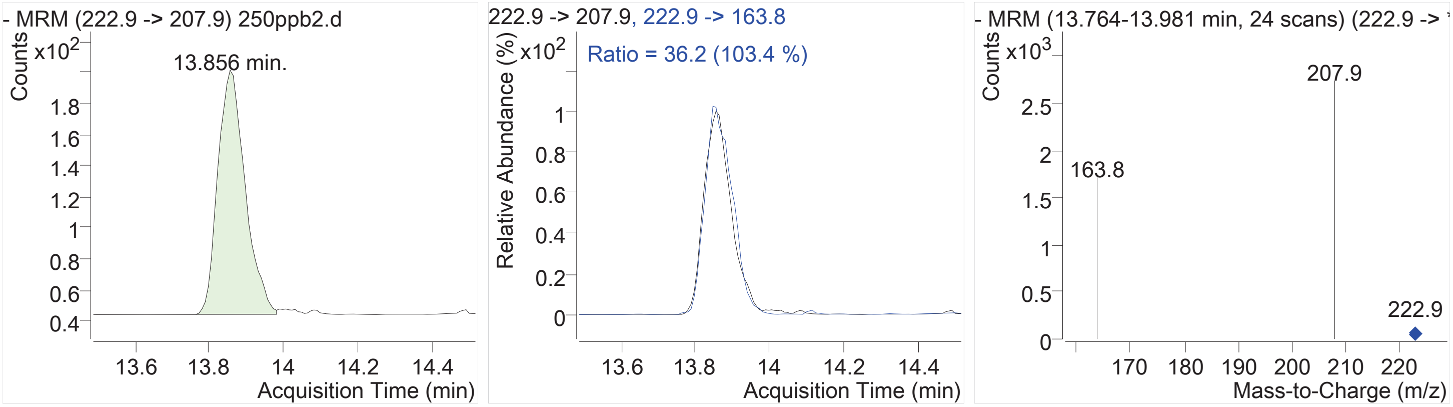

Ferulic acid

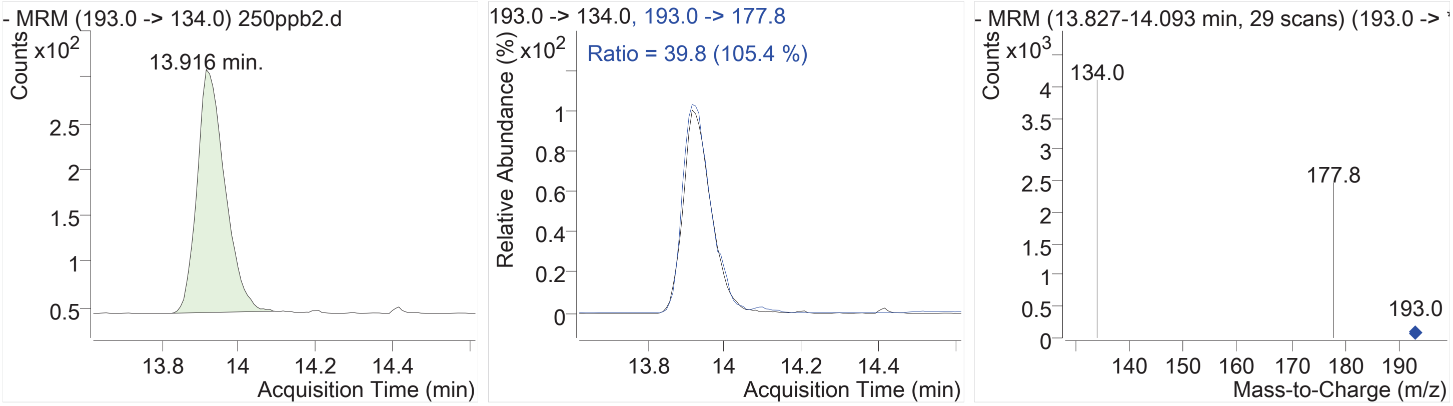

Luteolin 7-glucoside

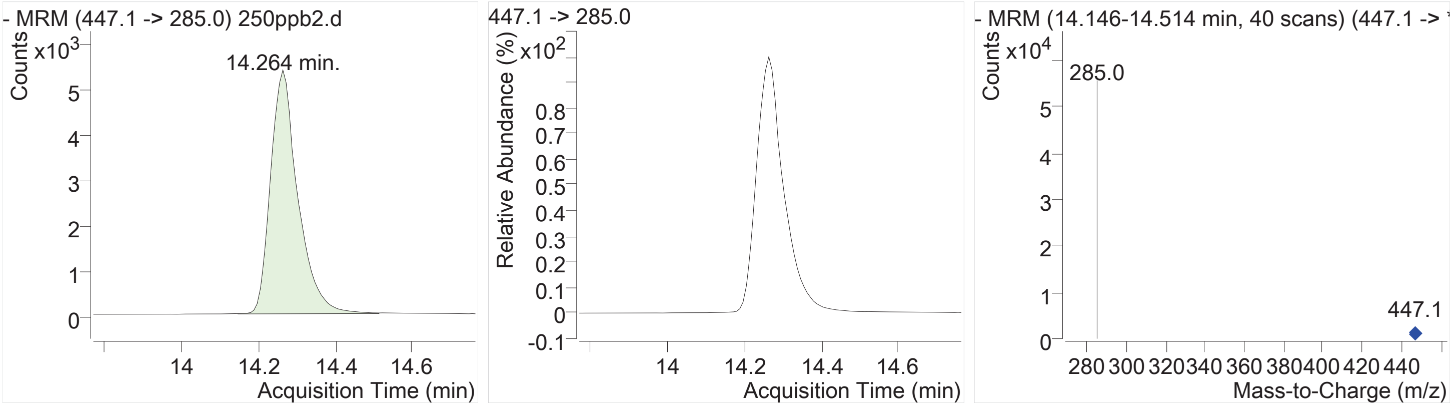

Hesperidin

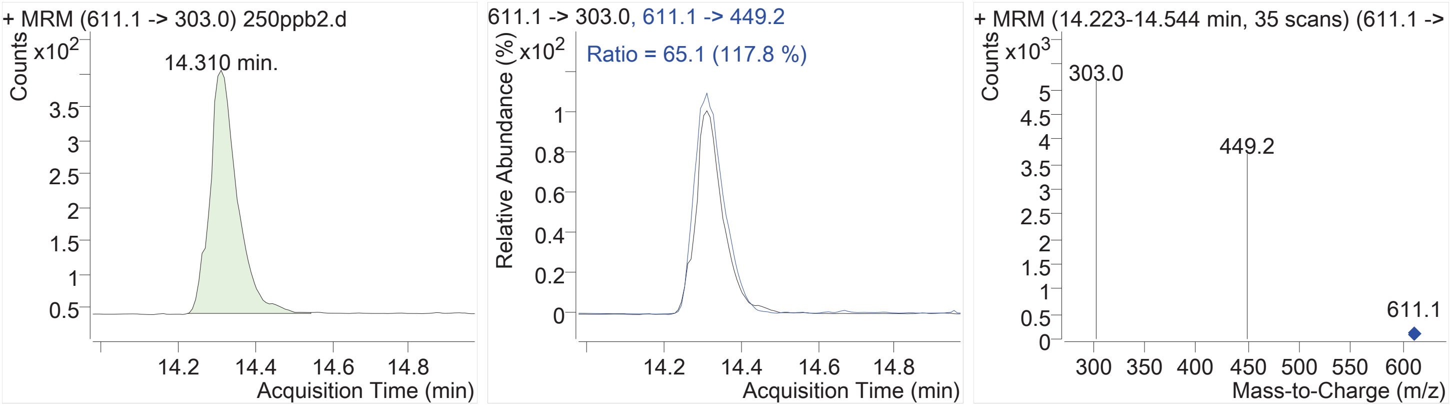

Hyperoside

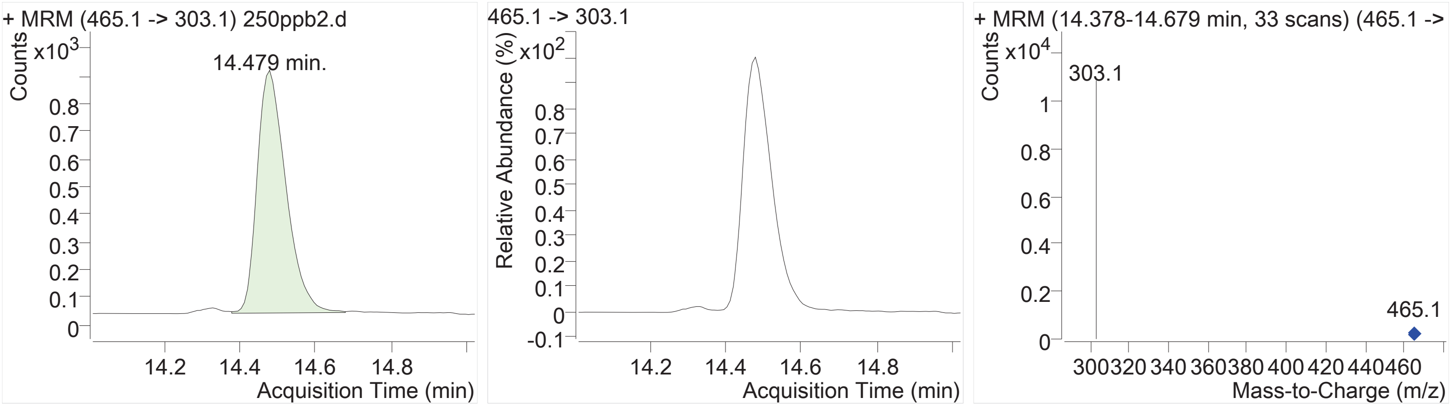

Rosmarinic acid

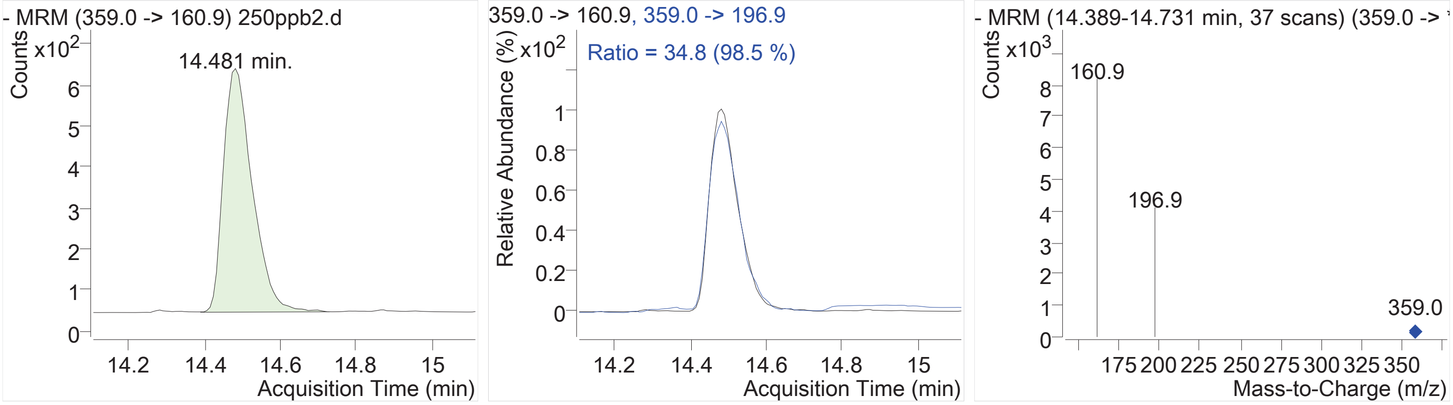

Apigenin 7-glucoside

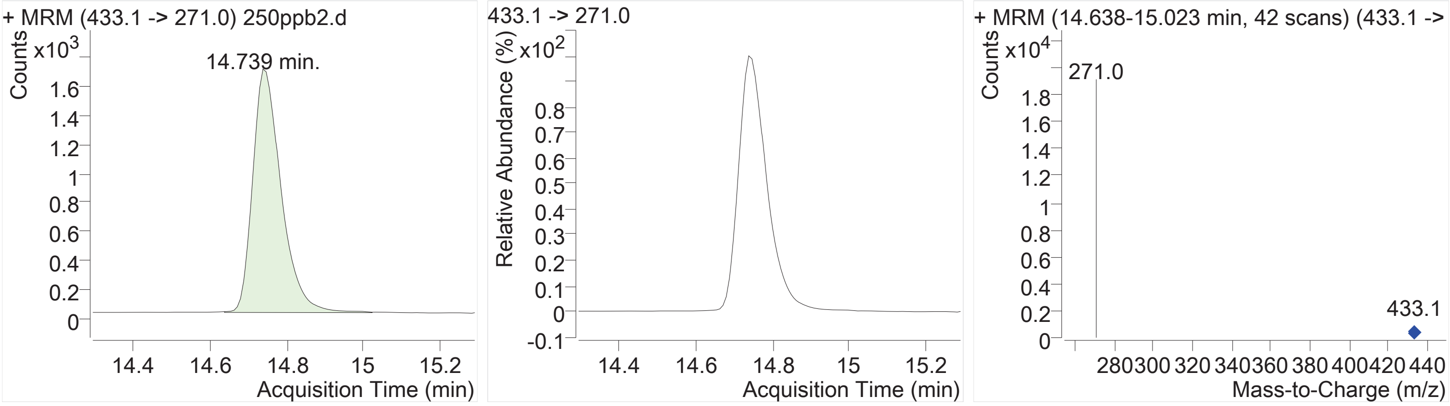

Pinoresinol

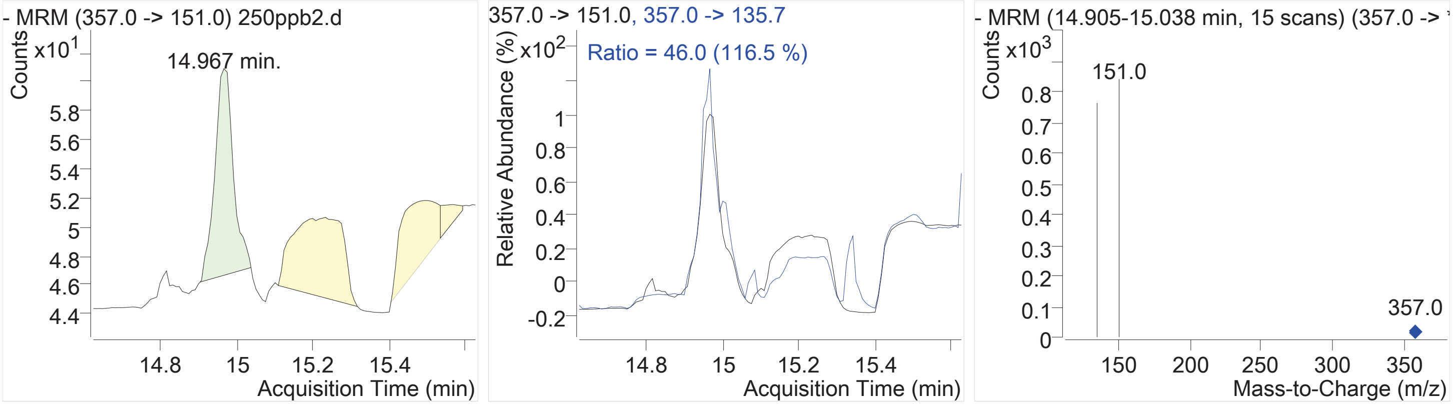

2-Hydroxycinnamic acid

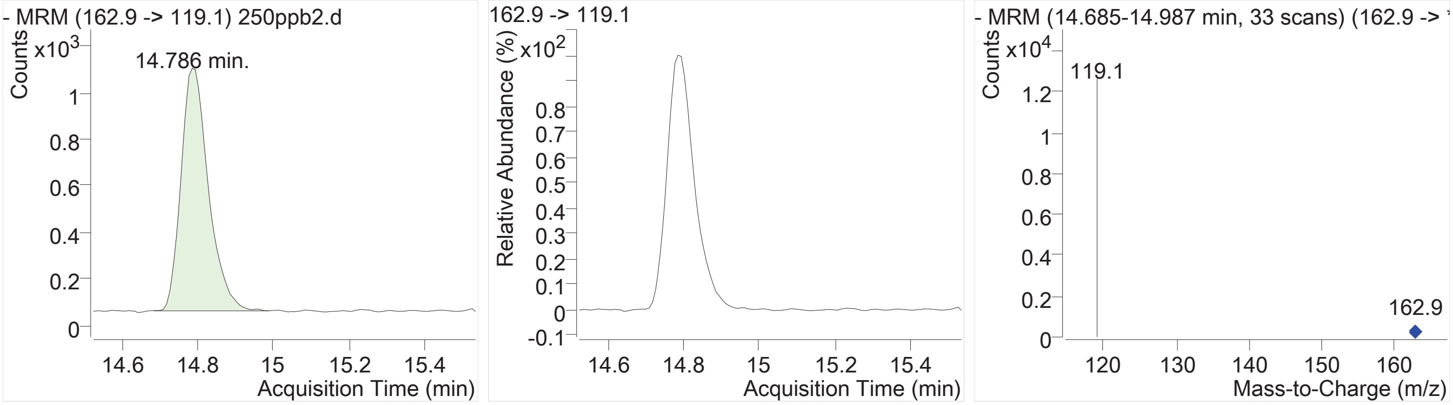

Eriodictyol

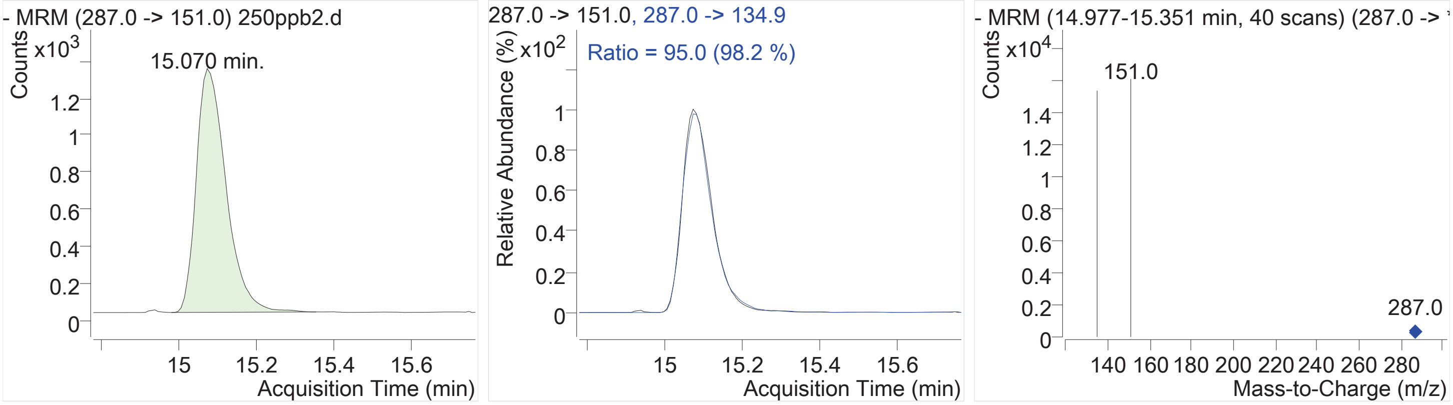

Quercetin

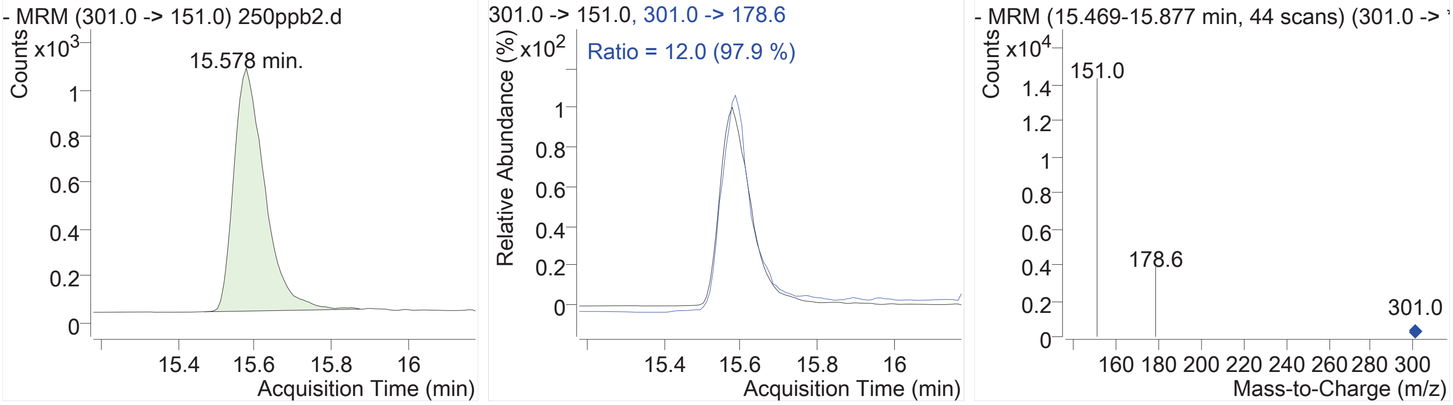

Luteolin

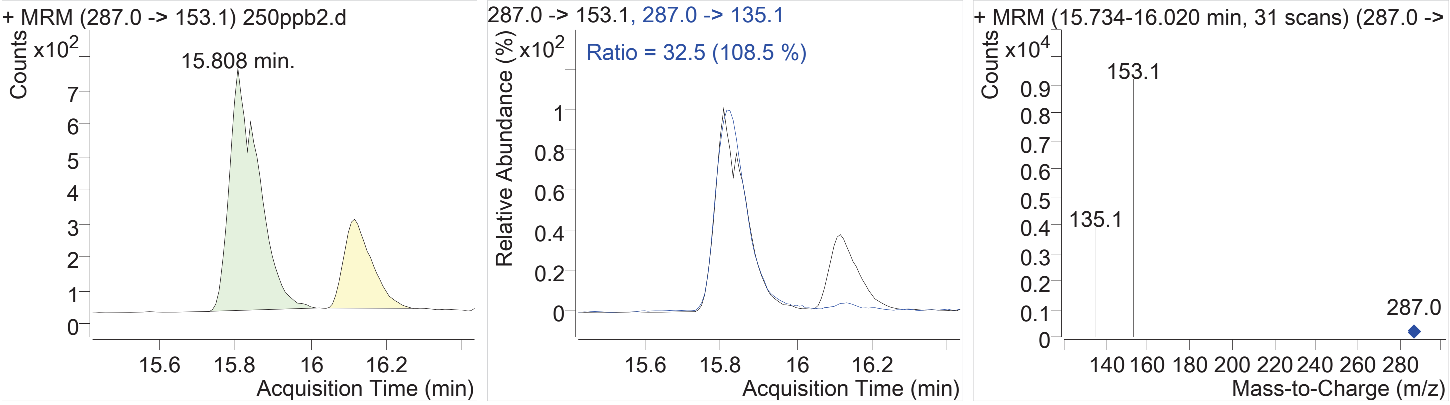

Kaempferol

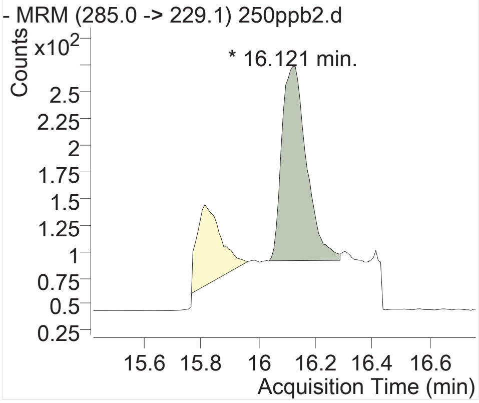

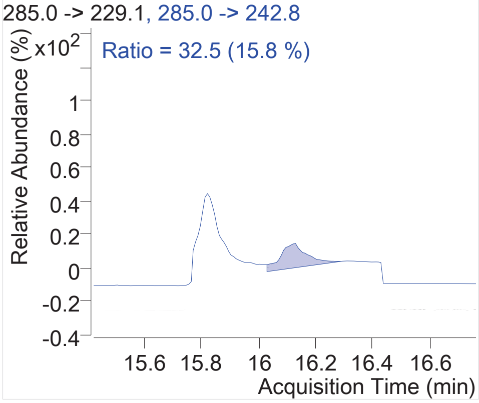

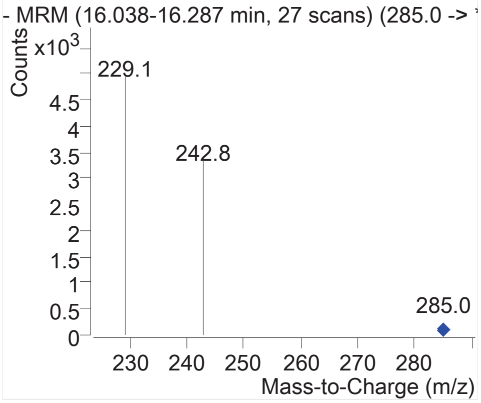

Apigenin

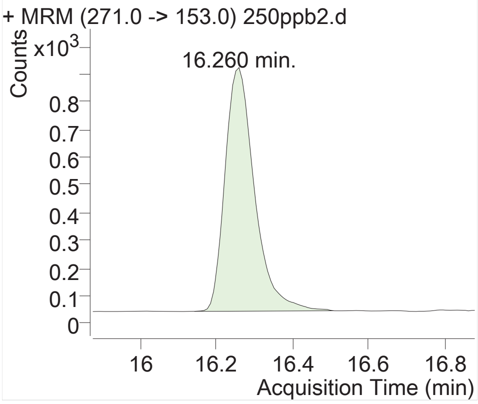

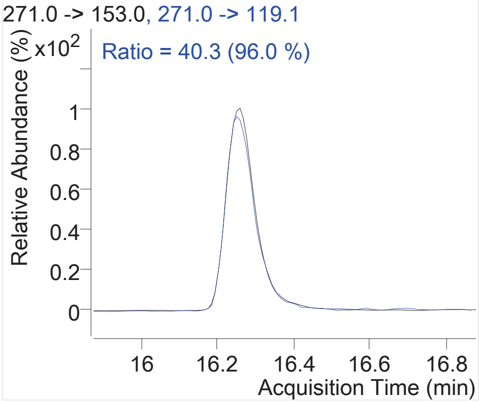

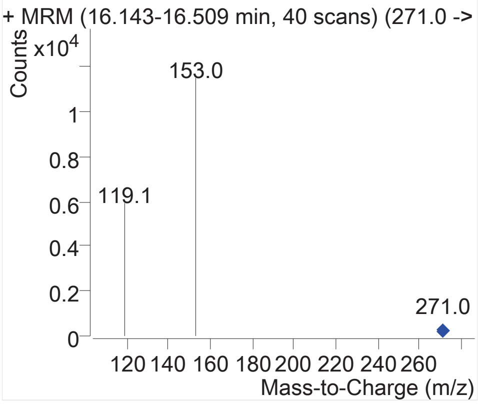

Quantitative Analysis Complete Report

Agilent Technologies

**Batch Path**	D:\MassHunter\Data\2022ekim\061022cengizhoca\QuantResults\071022.batch.bin		
**Analysis Time**	10/11/2022 1:33:26 PM	**Analyst Name**	Defam-PC\admin
**Report Time**	10/11/2022 1:34:23 PM	**Reporter Name**	admin
**Last Calib Update**	10/11/2022 1:33:17 PM	**Batch State**	Processed
**Quant Batch Version**	B.07.01	**Quant Report Version**	B.07.01

**Acq. Time**	10/6/2022 7:53:39 PM	**Data File**	250ppb3.d
**Sample Type**	Cal	**Sample Name**	250ppb3
**Dilution**	1	**Acq. Method**	FENOLIK_DMRM2021-31bilesen.m

Sample Chromatogram

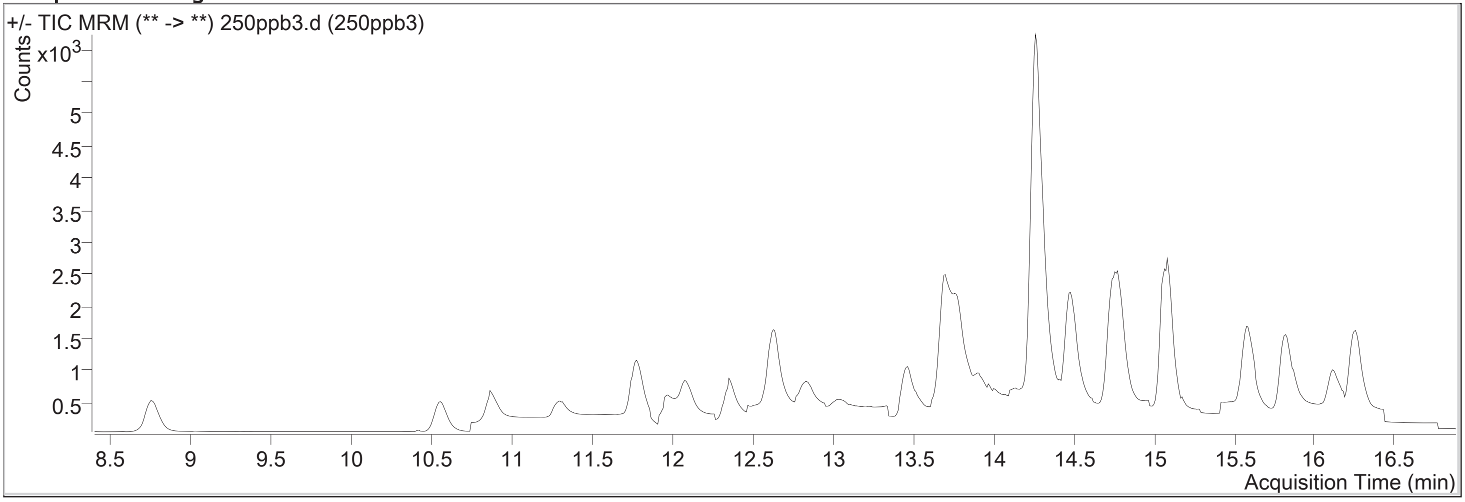

Compound	Transition	RT	Resp.	Final Conc	Units
Gallic acid	168.9 -> 125.0	8.758	2962	256.3445	ng/ml
Protocatechuic acid	152.9 -> 108.9	10.559	2684	247.9033	ng/ml
Pyrocatechol	109.0 -> 52.9	10.874	115	360.4919	ng/ml
3,4-Dihydroxyphenylacetic acid	167.0 -> 123.0	10.871	2067	252.0275	ng/ml
(+)-Catechin	289.0 -> 245.0	11.302	959	265.7187	ng/ml
2,5-Dihydroxybenzoic acid	152.9 -> 109.0	11.971	1572	248.8941	ng/ml
Chlorogenic acid	355.0 -> 163.0	11.785	5296	254.3829	ng/ml
3-Hydroxybenzoic acid	137.0 -> 93.0	12.845	1273	253.1454	ng/ml
4-Hydroxybenzoic acid	136.9 -> 93.1	12.089	2843	248.6277	ng/ml
(-)-Epicatechin	291.0 -> 139.1	12.361	1940	247.5823	ng/ml
Caffeic acid	179.0 -> 135.0	12.633	6725	261.3145	ng/ml
Syringic acid	196.9 -> 181.9	12.765	181	255.5301	ng/ml
Vanillin	151.0 -> 136.0	13.053	807	221.0151	ng/ml
Verbascoside	623.0 -> 160.8	13.459	2670	254.1681	ng/ml
Taxifolin	303.0 -> 285.1	13.695	7709	260.8713	ng/ml
p-Coumaric acid	162.9 -> 119.0	13.784	6909	260.3475	ng/ml
Sinapic acid	222.9 -> 207.9	13.848	796	255.4346	ng/ml
Ferulic acid	193.0 -> 134.0	13.925	1281	245.9975	ng/ml
Luteolin 7-glucoside	447.1 -> 285.0	14.264	26253	254.7851	ng/ml
Hesperidin	611.1 -> 303.0	14.310	2096	258.5218	ng/ml
Hyperoside	465.1 -> 303.1	14.479	4525	232.9429	ng/ml
Rosmarinic acid	359.0 -> 160.9	14.481	2932	233.1788	ng/ml
Apigenin 7-glucoside	433.1 -> 271.0	14.747	8633	255.4256	ng/ml
Pinoresinol	357.0 -> 151.0	14.967	54	186.4562	ng/ml
2-Hydroxycinnamic acid	162.9 -> 119.1	14.786	5392	252.4420	ng/ml
Eriodictyol	287.0 -> 151.0	15.087	6297	230.8125	ng/ml
Quercetin	301.0 -> 151.0	15.586	6108	259.8316	ng/ml
Luteolin	287.0 -> 153.1	15.825	4280	265.4377	ng/ml
Kaempferol	285.0 -> 229.1	16.113	1138	250.8880	ng/ml

Compound	Transition	RT	Resp.	Final Conc	Units
Apigenin	271.0 -> 153.0	16.260	4803	254.0694	ng/ml

Gallic acid

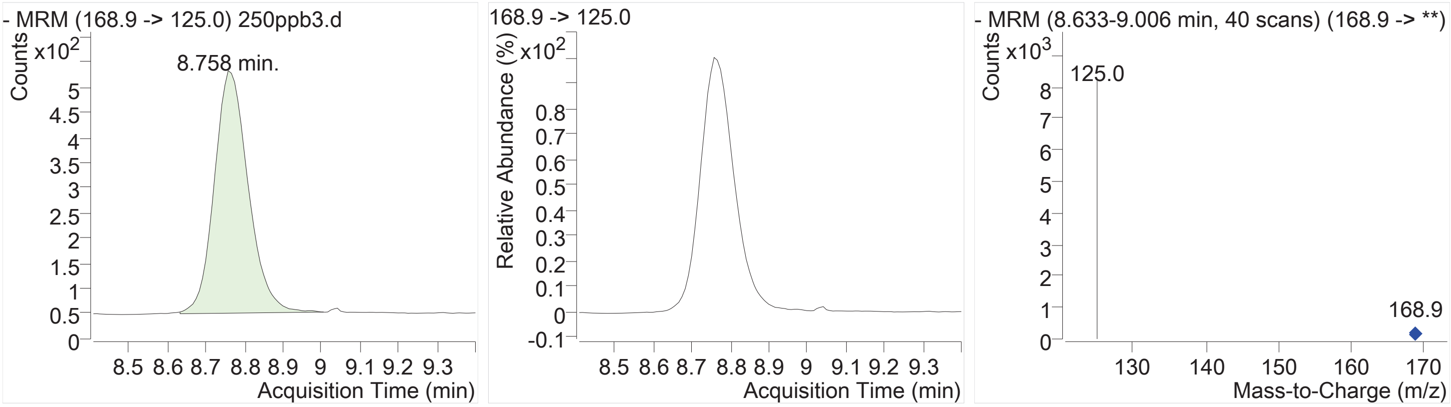

Protocatechuic acid

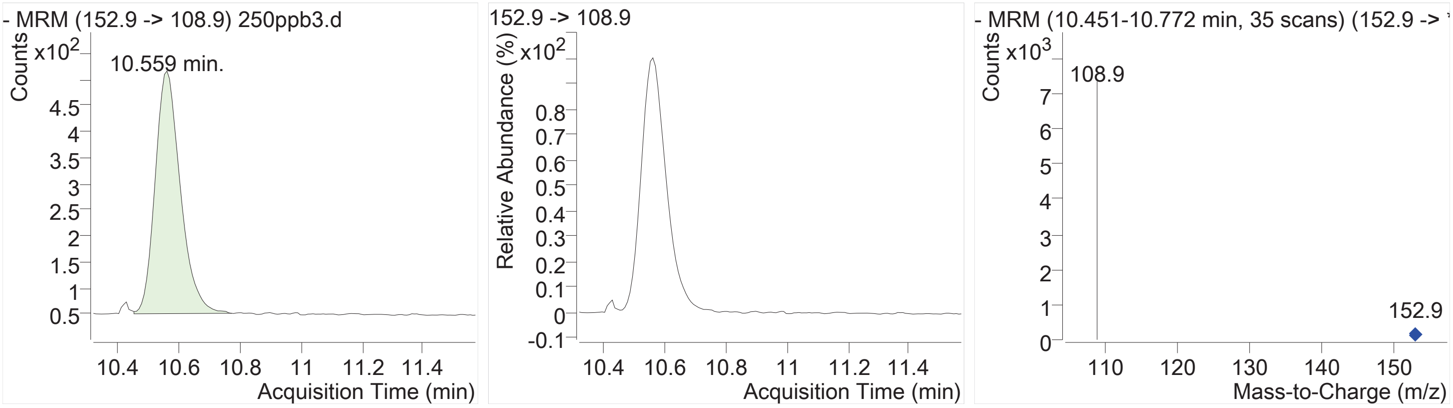

Pyrocatechol

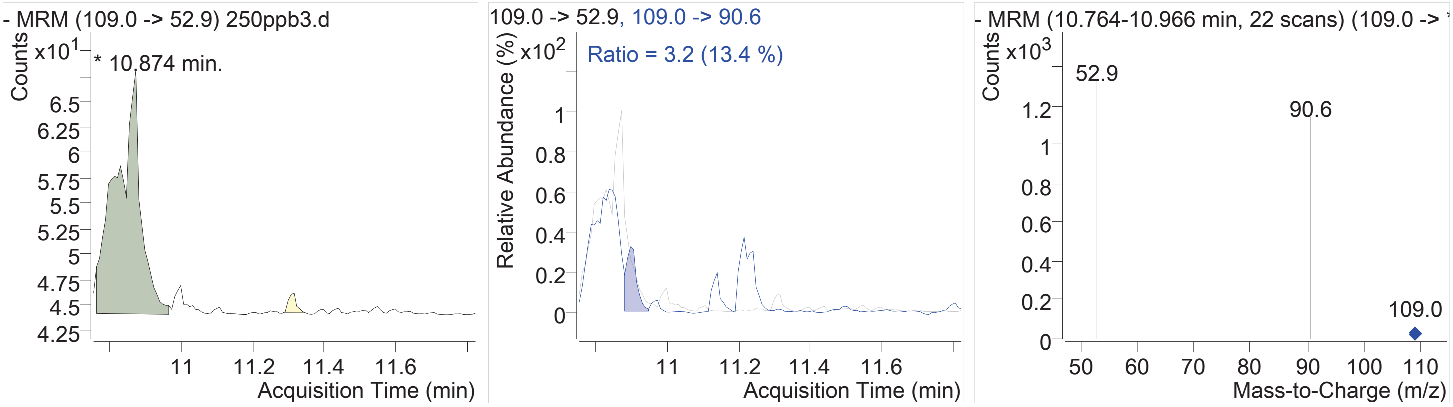

3,4-Dihydroxyphenylacetic acid

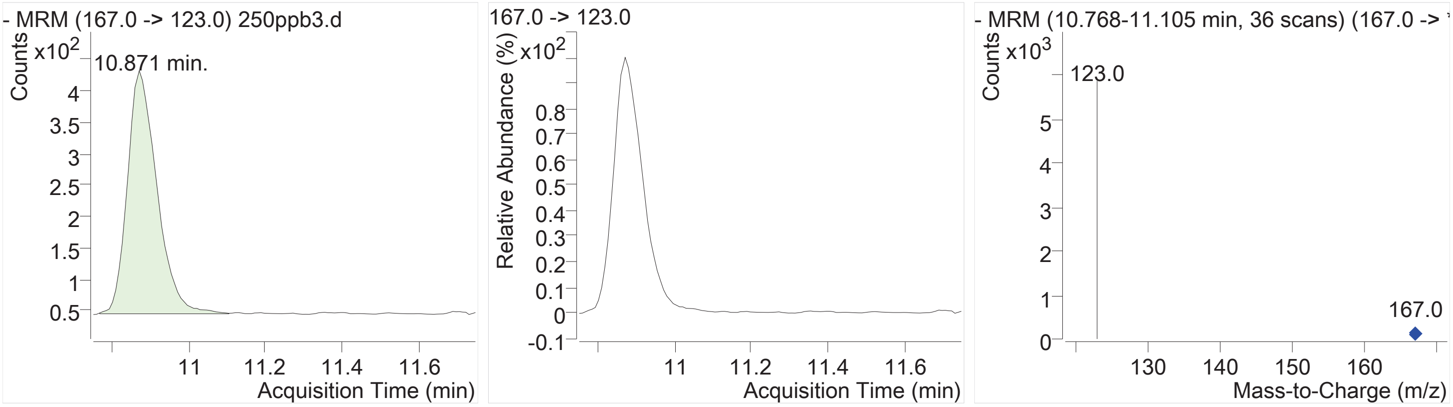

(+)-Catechin

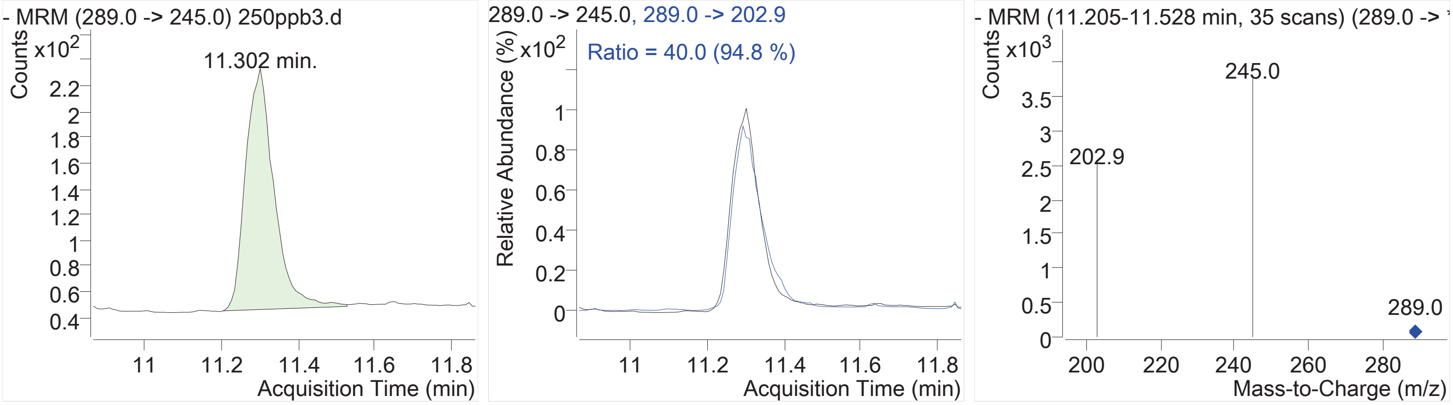

2,5-Dihydroxybenzoic acid

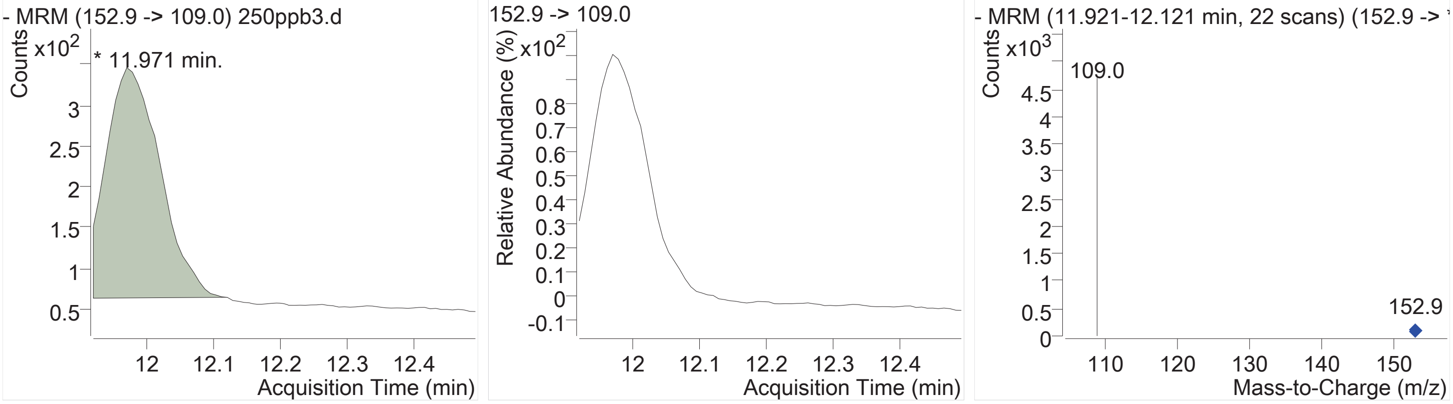

Chlorogenic acid

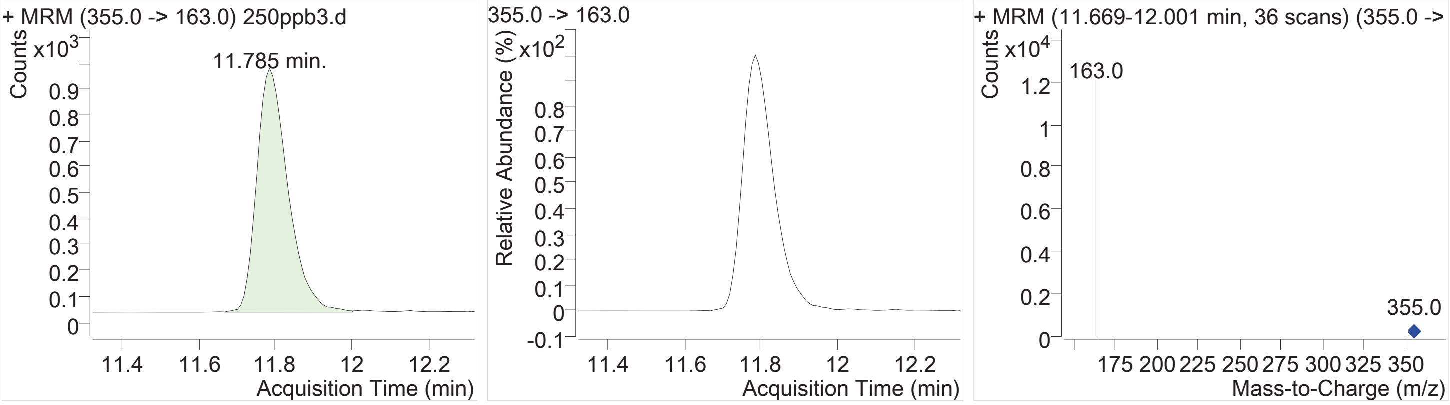

3-Hydroxybenzoic acid

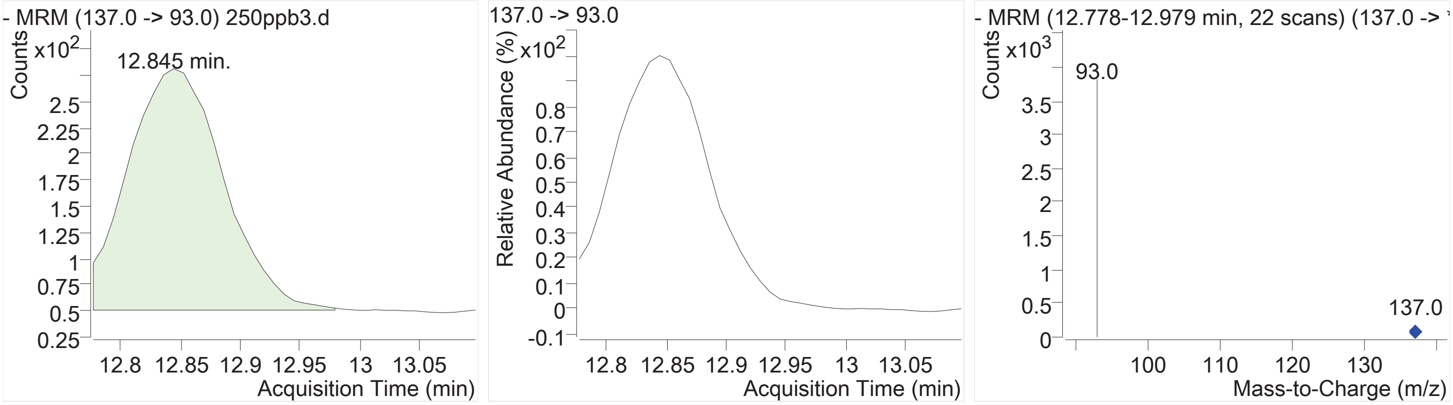

4-Hydroxybenzoic acid

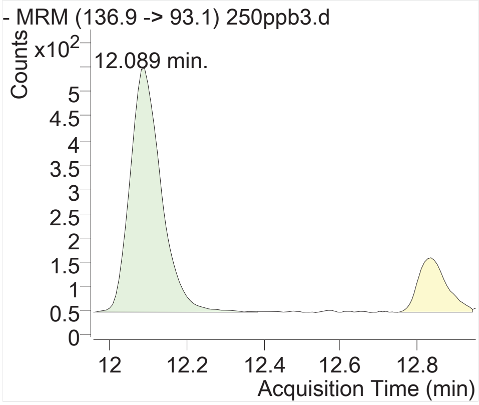

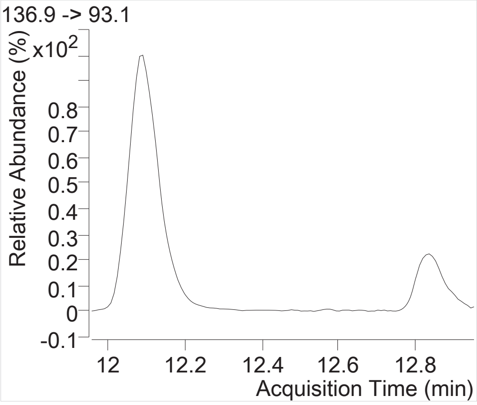

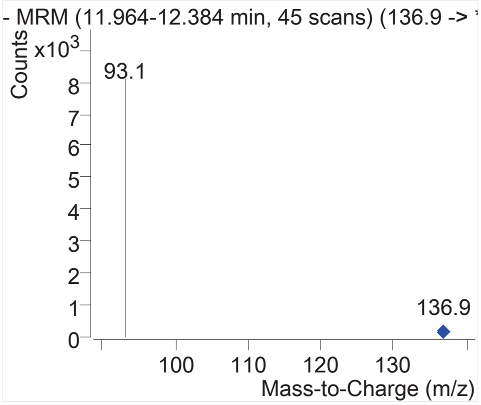

(-)-Epicatechin

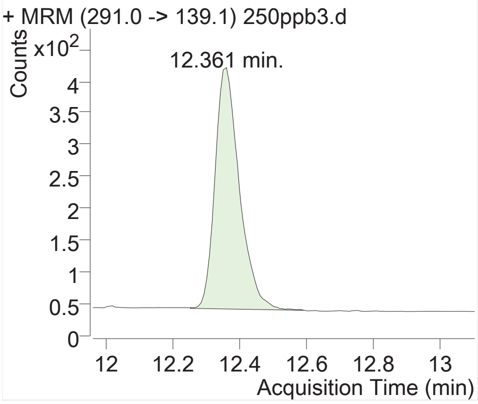

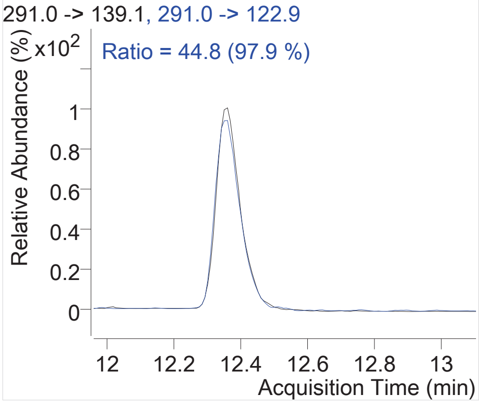

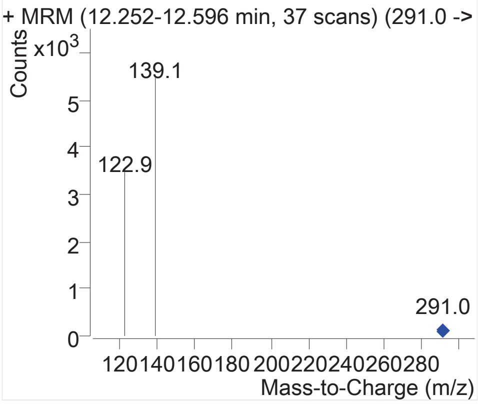

Caffeic acid

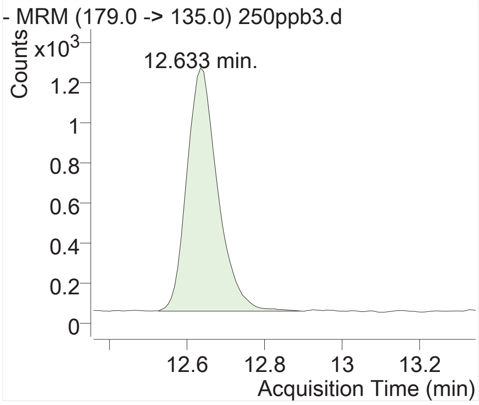

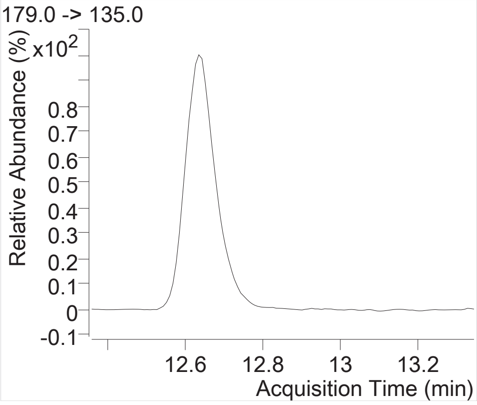

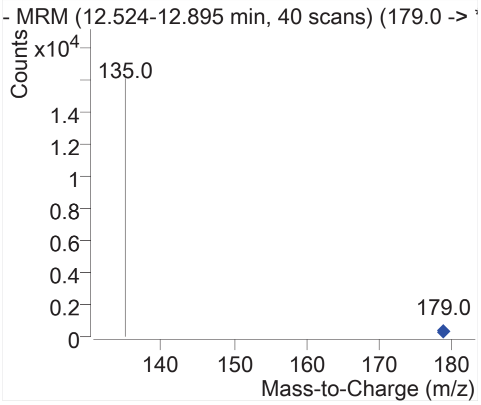

Syringic acid

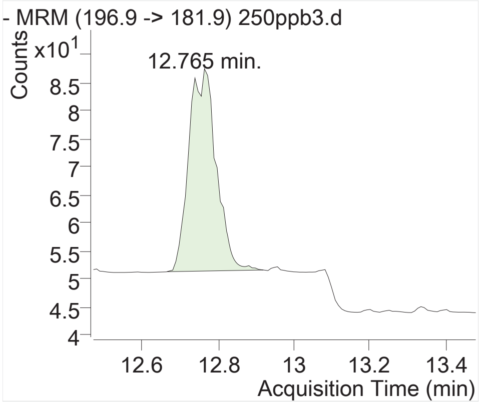

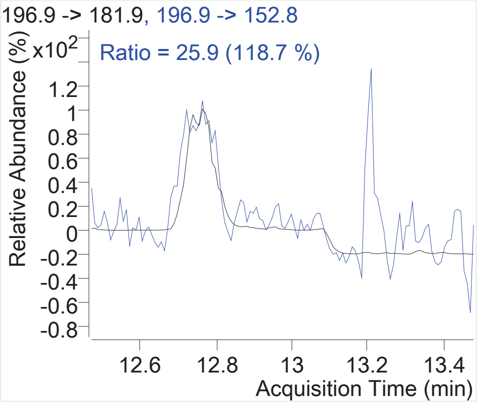

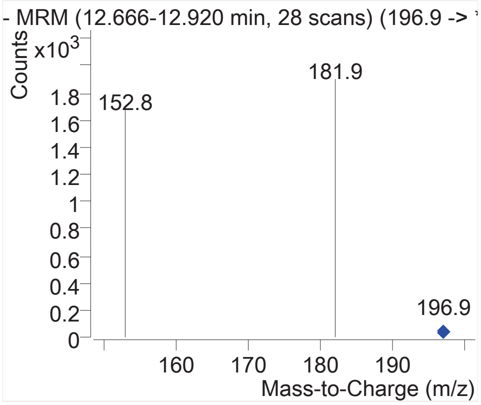

Vanillin

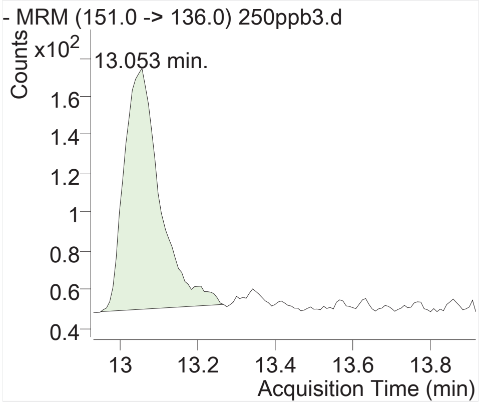

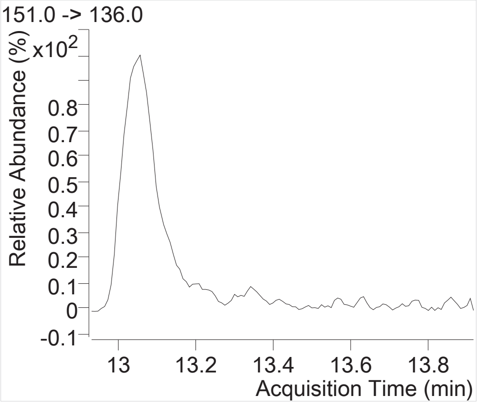

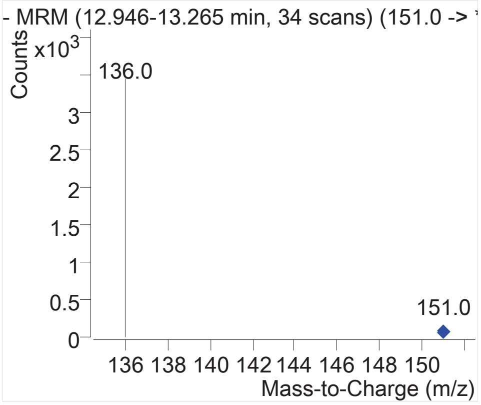

Verbascoside

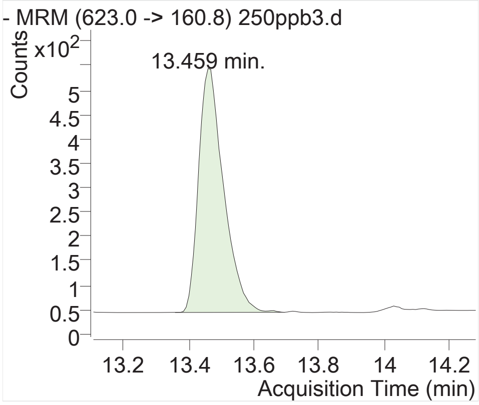

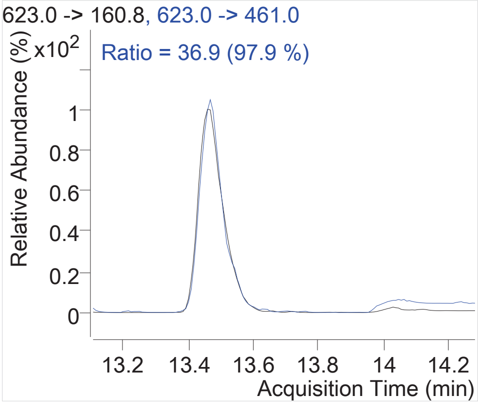

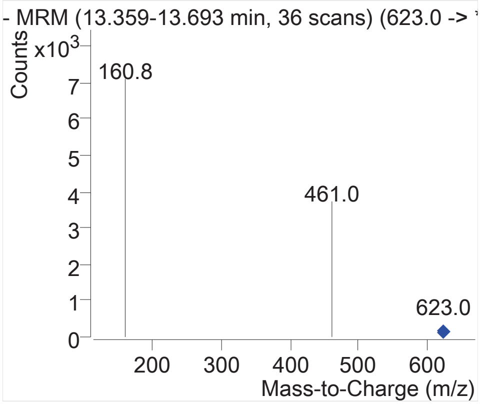

Taxifolin

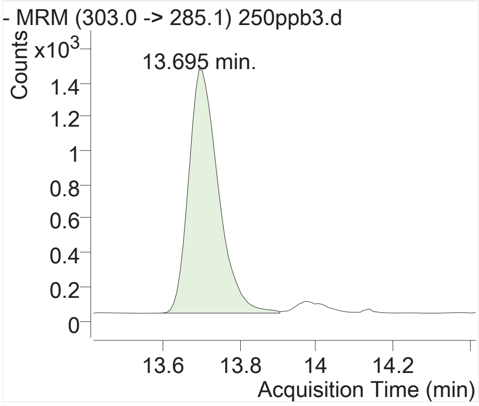

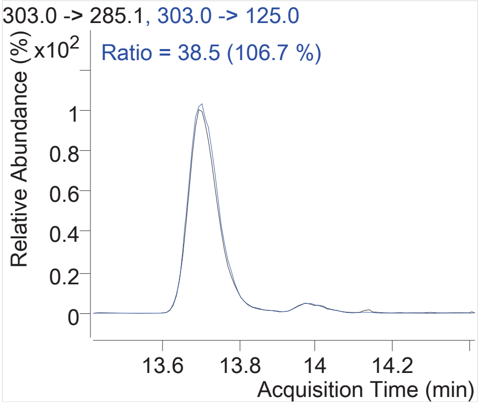

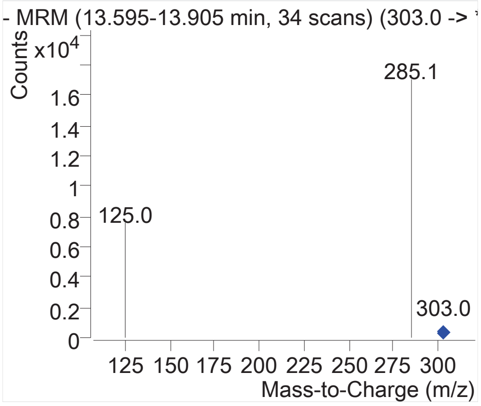

p-Coumaric acid

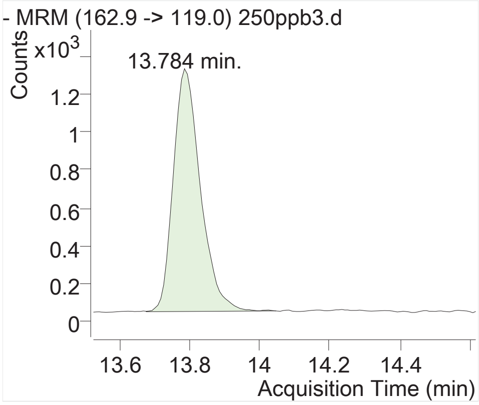

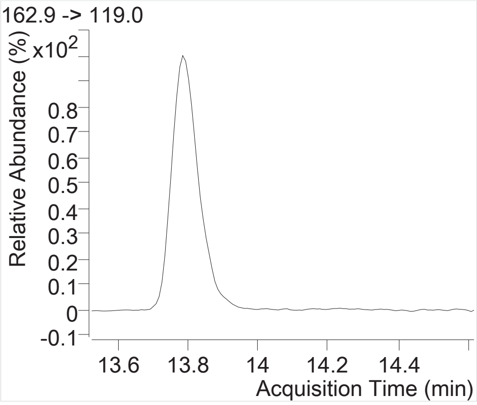

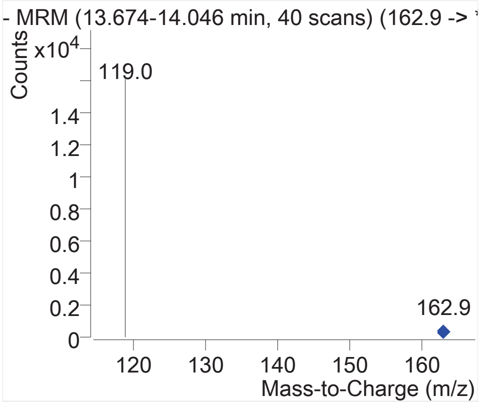

Sinapic acid

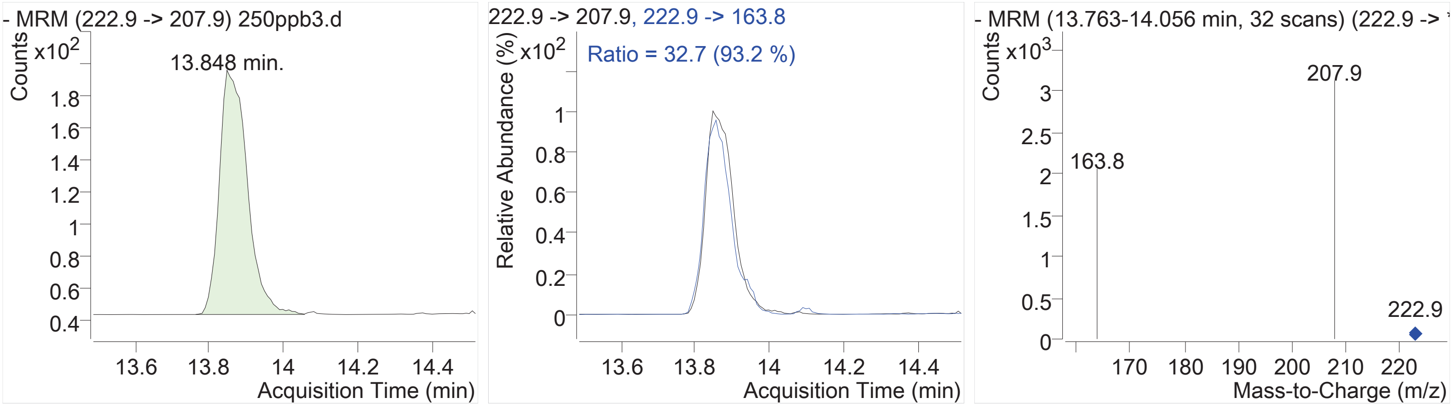

Ferulic acid

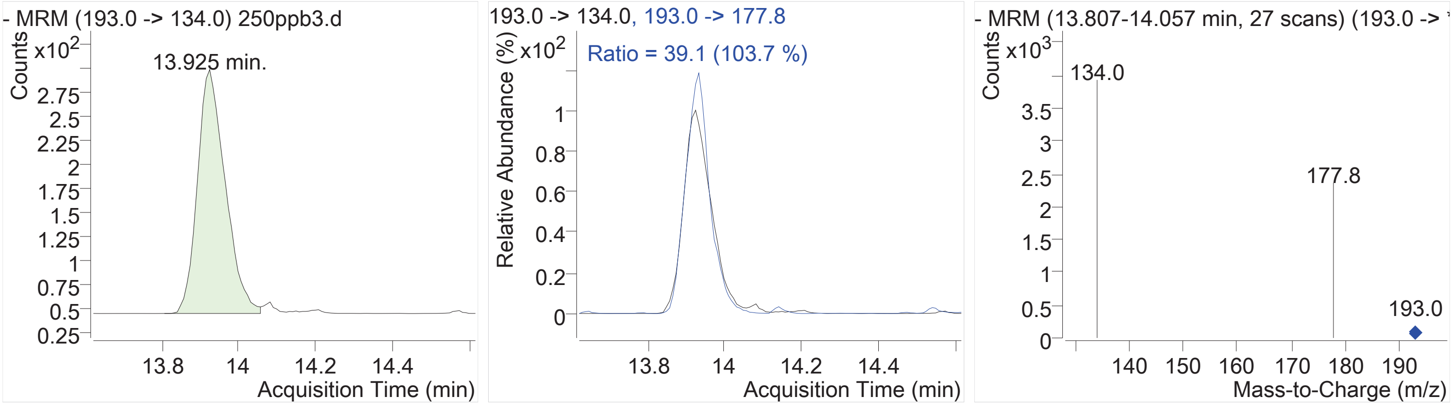

Luteolin 7-glucoside

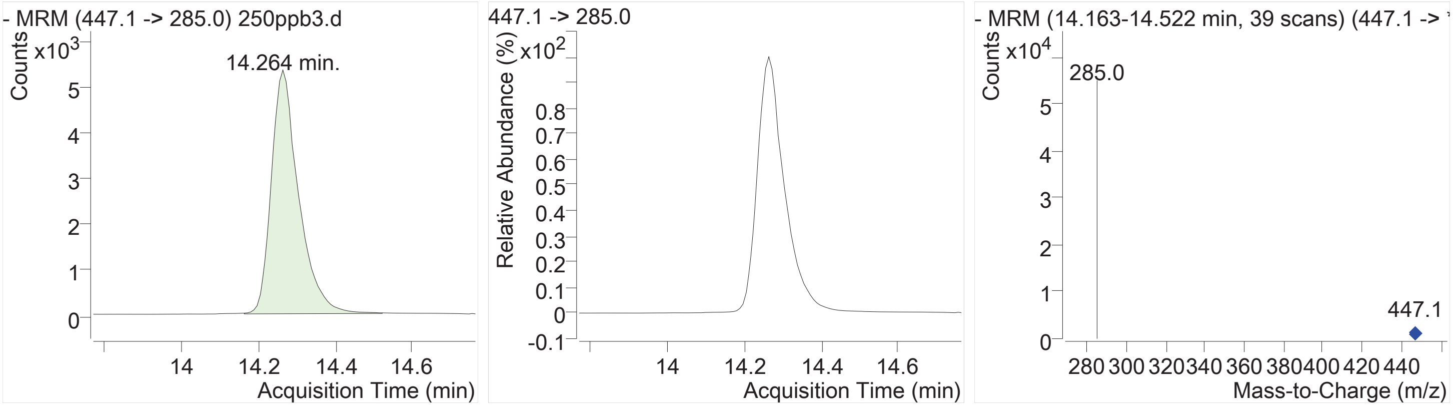

Hesperidin

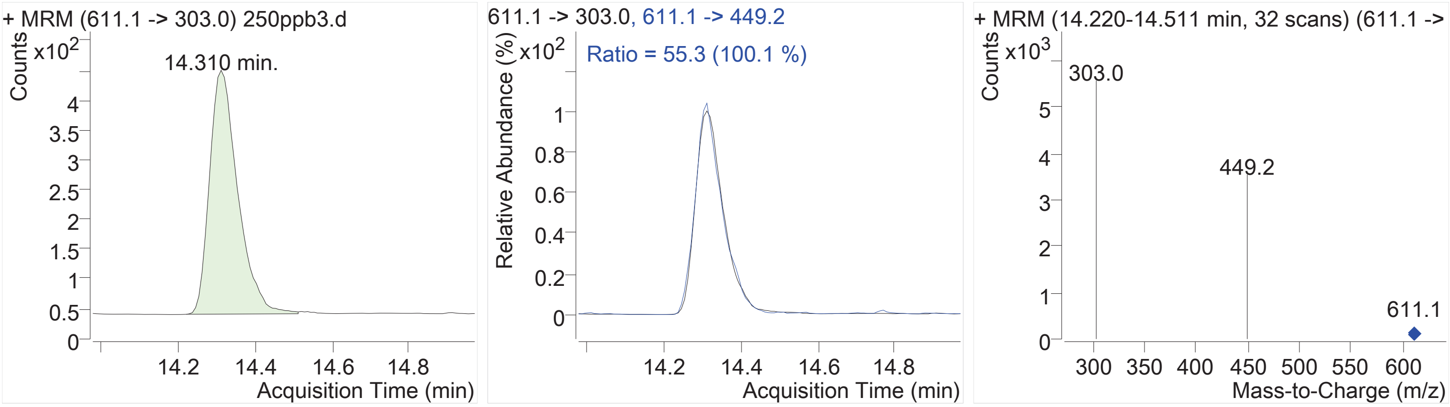

Agilent Technologies

Hyperoside

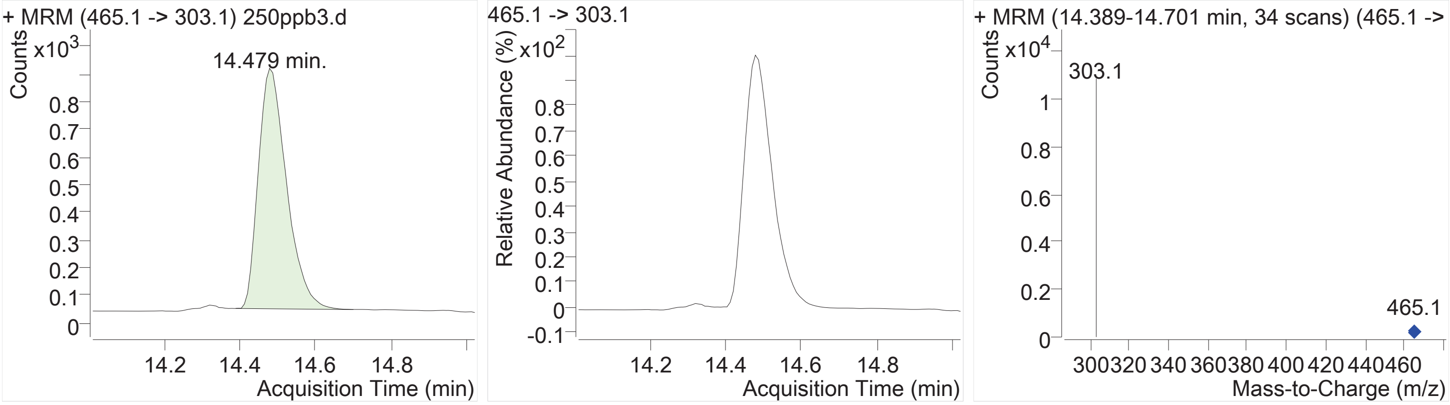

Rosmarinic acid

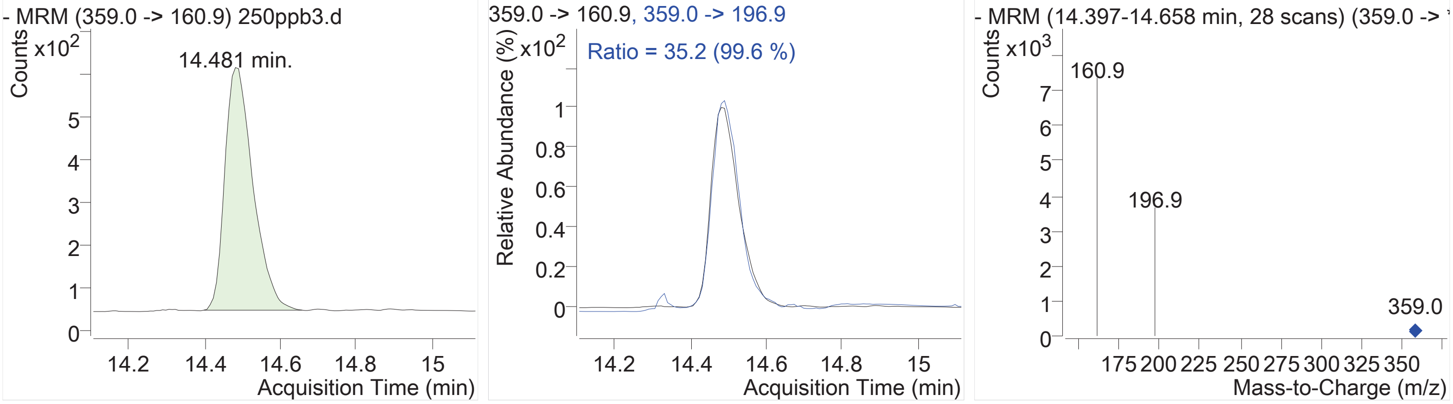

Apigenin 7-glucoside

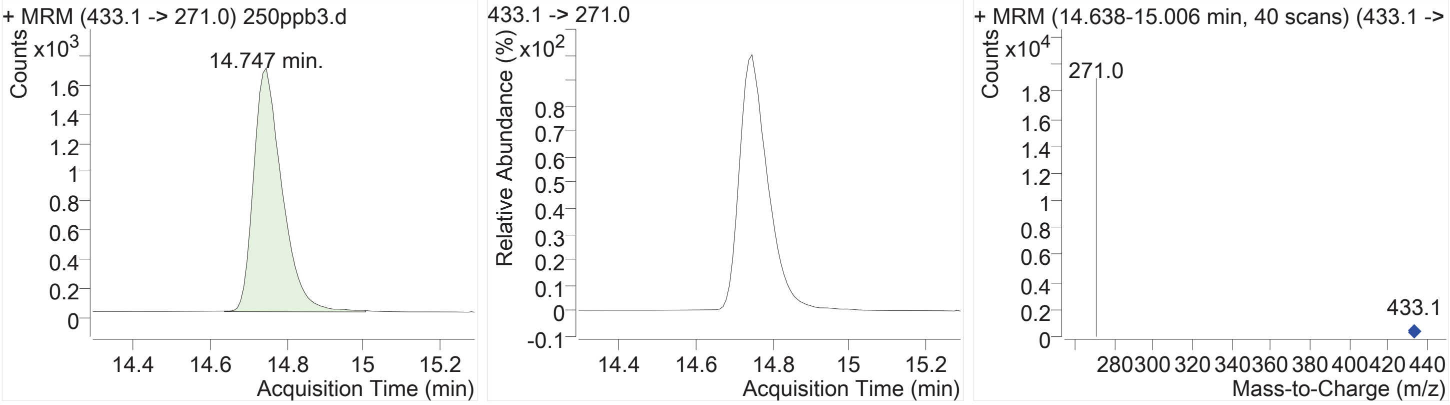

Pinoresinol

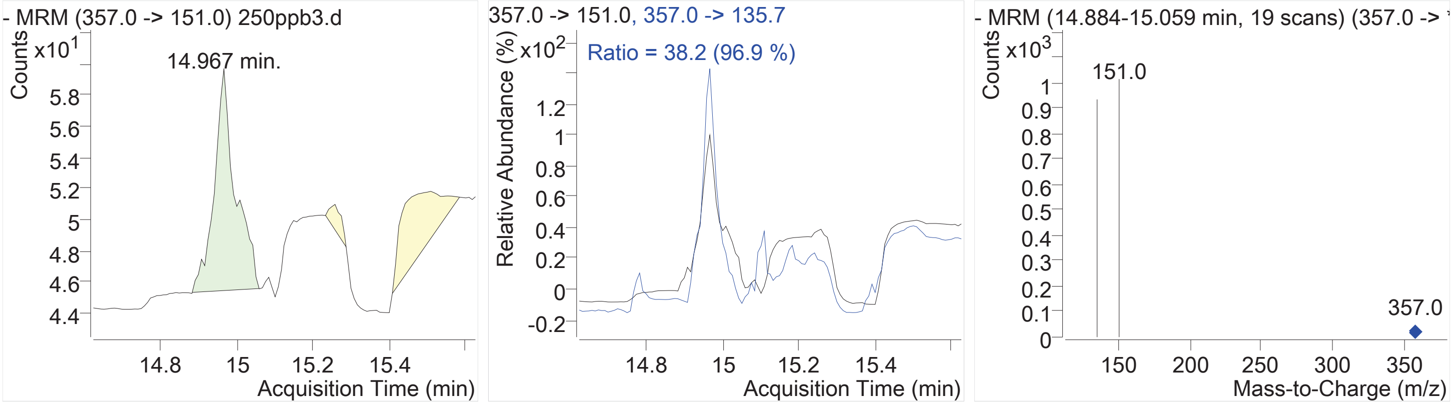

2-Hydroxycinnamic acid

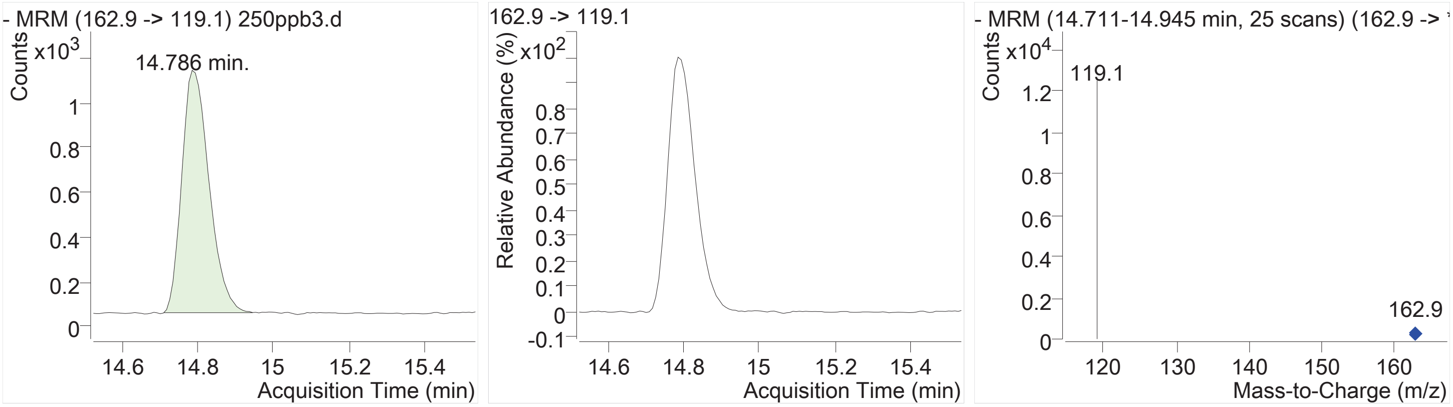

Eriodictyol

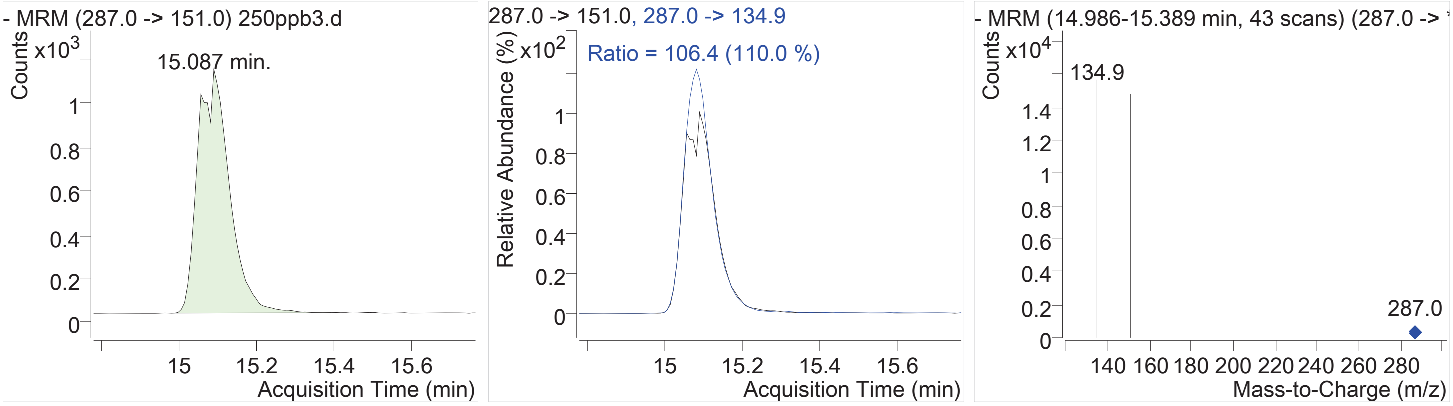

Quercetin

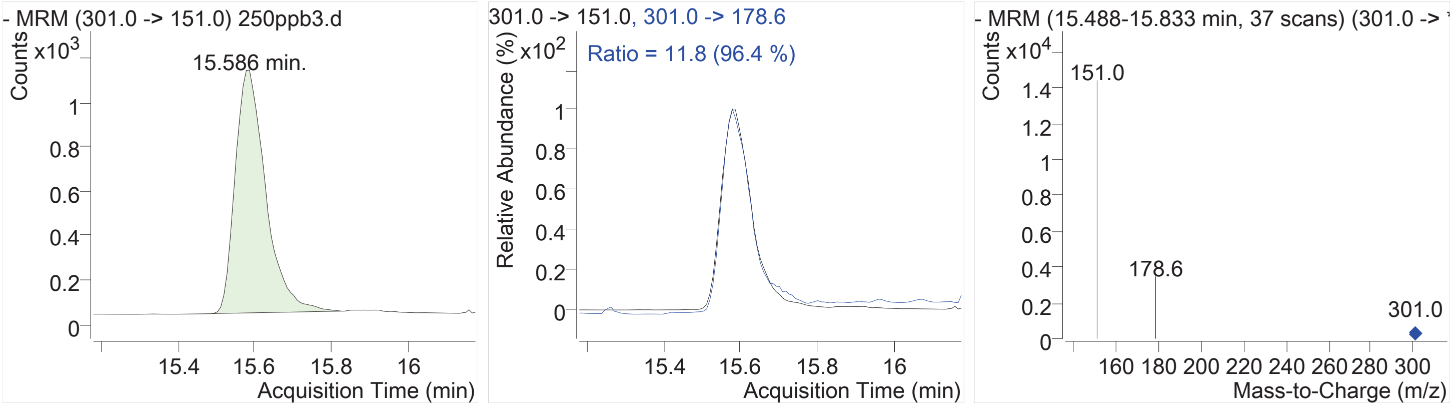

Luteolin

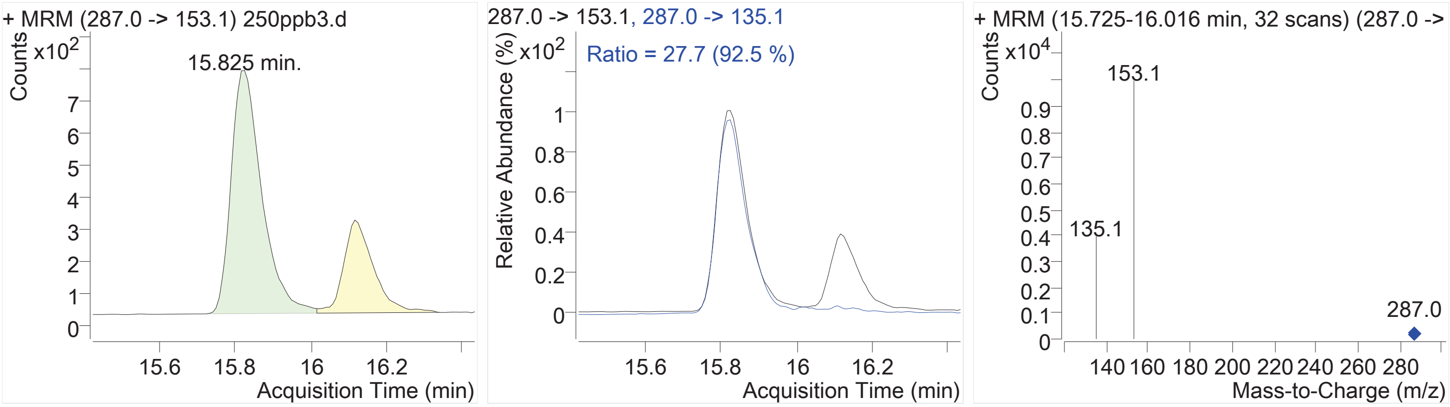

Agilent Technologies

Kaempferol

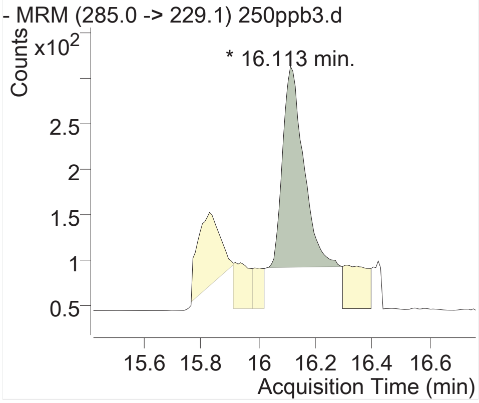

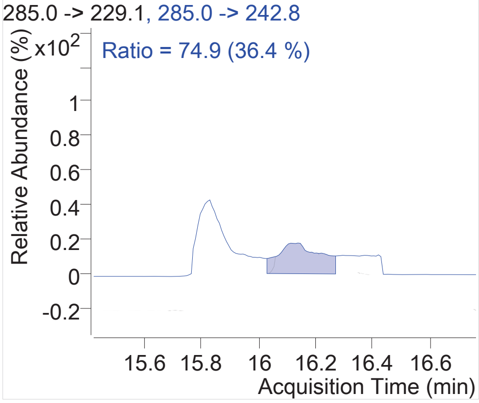

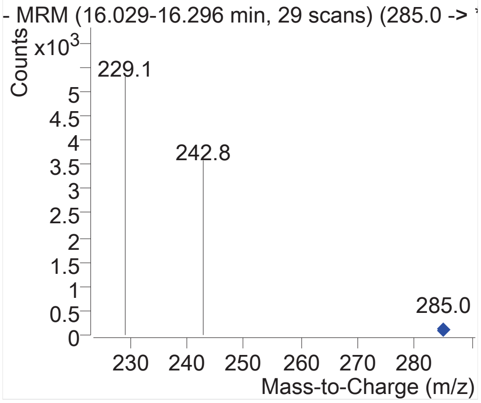

Apigenin

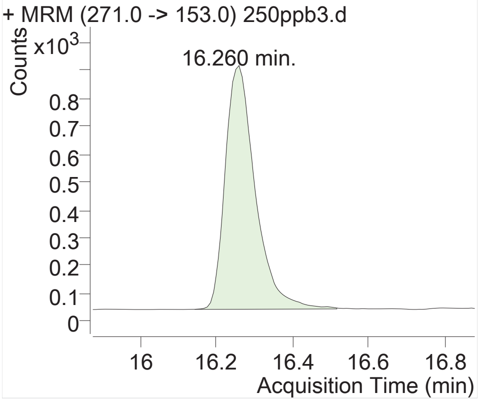

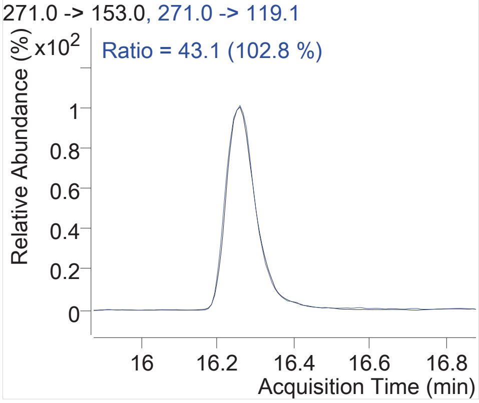

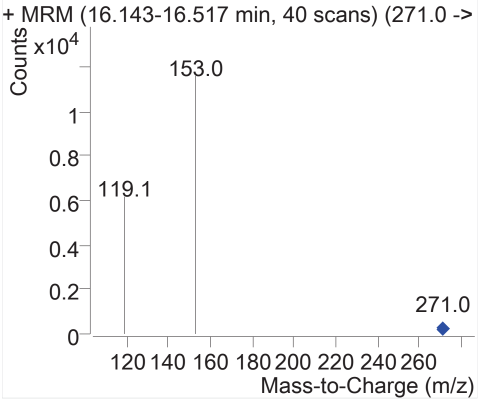

Quantitative Analysis Complete Report

**Batch Path**	D:\MassHunter\Data\2022ekim\061022cengizhoca\QuantResults\071022.batch.bin		
**Analysis Time**	10/11/2022 1:33:26 PM	**Analyst Name**	Defam-PC\admin
**Report Time**	10/11/2022 1:34:26 PM	**Reporter Name**	admin
**Last Calib Update**	10/11/2022 1:33:17 PM	**Batch State**	Processed
**Quant Batch Version**	B.07.01	**Quant Report Version**	B.07.01

**Acq. Time**	10/6/2022 8:15:20 PM	**Data File**	500ppb1.d
**Sample Type**	Cal	**Sample Name**	500ppb1
**Dilution**	1	**Acq. Method**	FENOLIK_DMRM2021-31bilesen.m

Sample Chromatogram

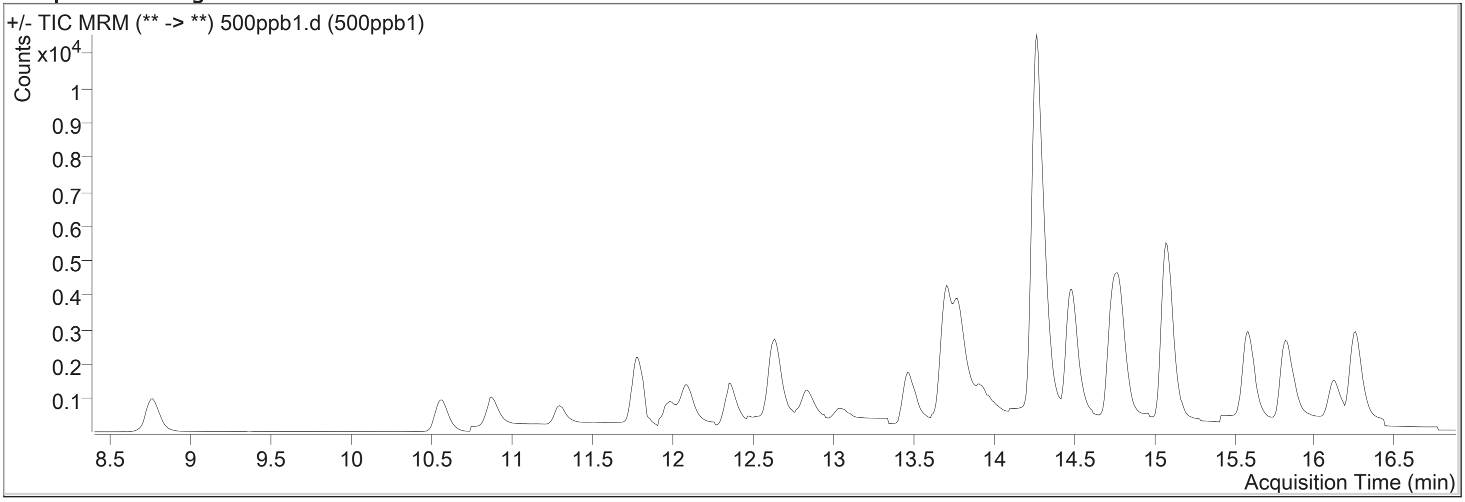

Compound	Transition	RT	Resp.	Final Conc	Units
Gallic acid	168.9 -> 125.0	8.766	5975	539.5334	ng/ml
Protocatechuic acid	152.9 -> 108.9	10.568	5544	524.6661	ng/ml
Pyrocatechol	109.0 -> 52.9	10.831	151	479.3011	ng/ml
3,4-Dihydroxyphenylacetic acid	167.0 -> 123.0	10.879	4201	521.2669	ng/ml
(+)-Catechin	289.0 -> 245.0	11.302	1764	514.1201	ng/ml
2,5-Dihydroxybenzoic acid	152.9 -> 109.0	11.988	3299	521.8580	ng/ml
Chlorogenic acid	355.0 -> 163.0	11.793	10372	492.6535	ng/ml
3-Hydroxybenzoic acid	137.0 -> 93.0	12.845	2642	509.1901	ng/ml
4-Hydroxybenzoic acid	136.9 -> 93.1	12.089	5913	535.6665	ng/ml
(-)-Epicatechin	291.0 -> 139.1	12.369	4072	516.2789	ng/ml
Caffeic acid	179.0 -> 135.0	12.641	13194	540.5373	ng/ml
Syringic acid	196.9 -> 181.9	12.748	353	494.8108	ng/ml
Vanillin	151.0 -> 136.0	13.053	1941	561.2160	ng/ml
Verbascoside	623.0 -> 160.8	13.467	5431	531.0617	ng/ml
Taxifolin	303.0 -> 285.1	13.712	15077	554.9196	ng/ml
p-Coumaric acid	162.9 -> 119.0	13.792	13899	533.2423	ng/ml
Sinapic acid	222.9 -> 207.9	13.864	1571	499.7134	ng/ml
Ferulic acid	193.0 -> 134.0	13.934	2647	508.0690	ng/ml
Luteolin 7-glucoside	447.1 -> 285.0	14.264	51277	529.7120	ng/ml
Hesperidin	611.1 -> 303.0	14.319	4319	528.3534	ng/ml
Hyperoside	465.1 -> 303.1	14.487	10086	514.4364	ng/ml
Rosmarinic acid	359.0 -> 160.9	14.489	6474	516.4535	ng/ml
Apigenin 7-glucoside	433.1 -> 271.0	14.747	17437	517.2855	ng/ml
Pinoresinol	357.0 -> 151.0	14.967	131	489.3227	ng/ml
2-Hydroxycinnamic acid	162.9 -> 119.1	14.794	11011	517.3781	ng/ml
Eriodictyol	287.0 -> 151.0	15.078	14052	538.8617	ng/ml
Quercetin	301.0 -> 151.0	15.586	12336	532.0472	ng/ml
Luteolin	287.0 -> 153.1	15.825	8430	527.5986	ng/ml
Kaempferol	285.0 -> 229.1	16.121	2318	545.5338	ng/ml

Agilent Technologies

Compound	Transition	RT	Resp.	Final Conc	Units
Apigenin	271.0 -> 153.0	16.260	9884	529.1451	ng/ml

Gallic acid

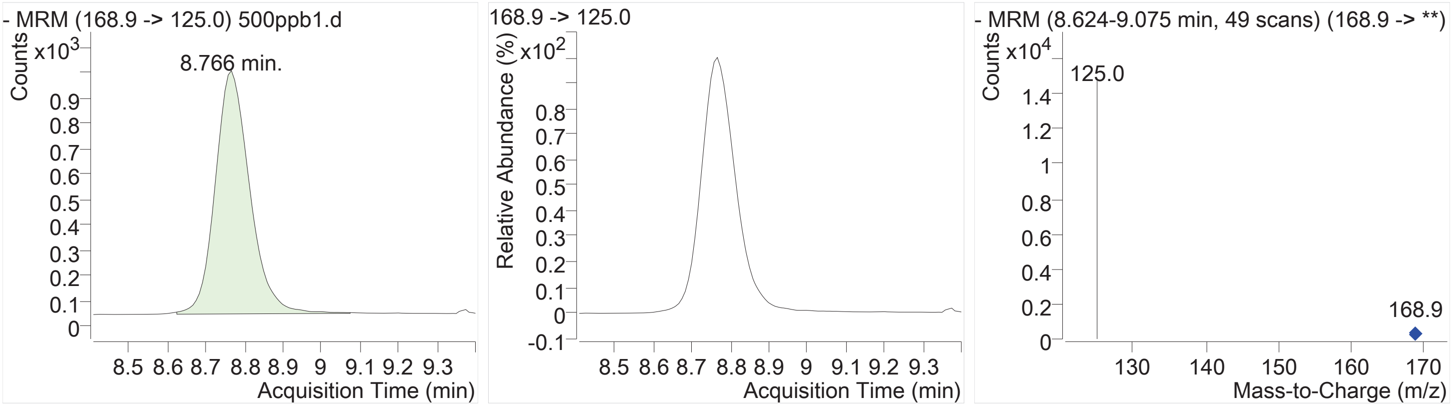

Protocatechuic acid

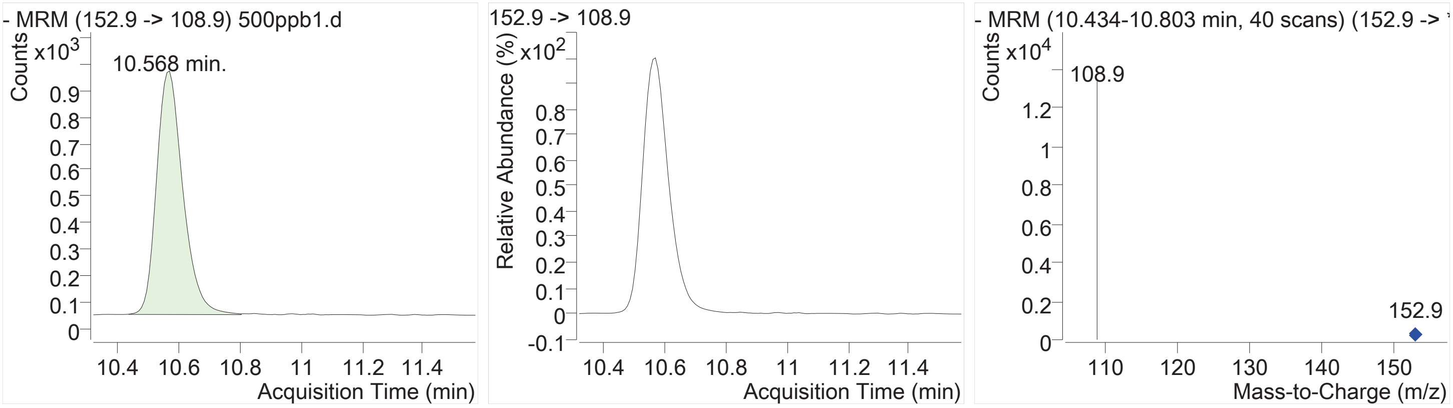

Pyrocatechol

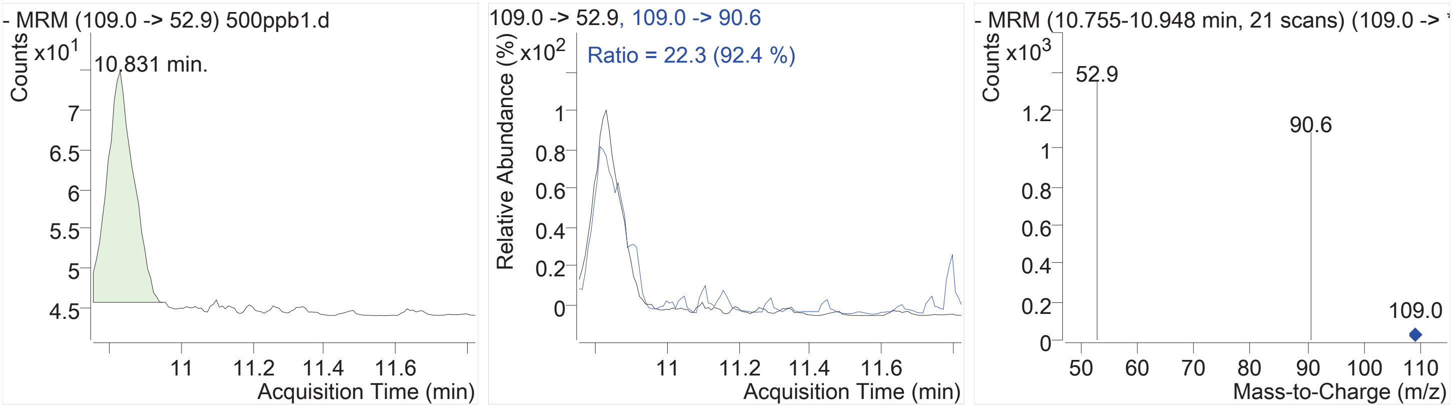

3,4-Dihydroxyphenylacetic acid

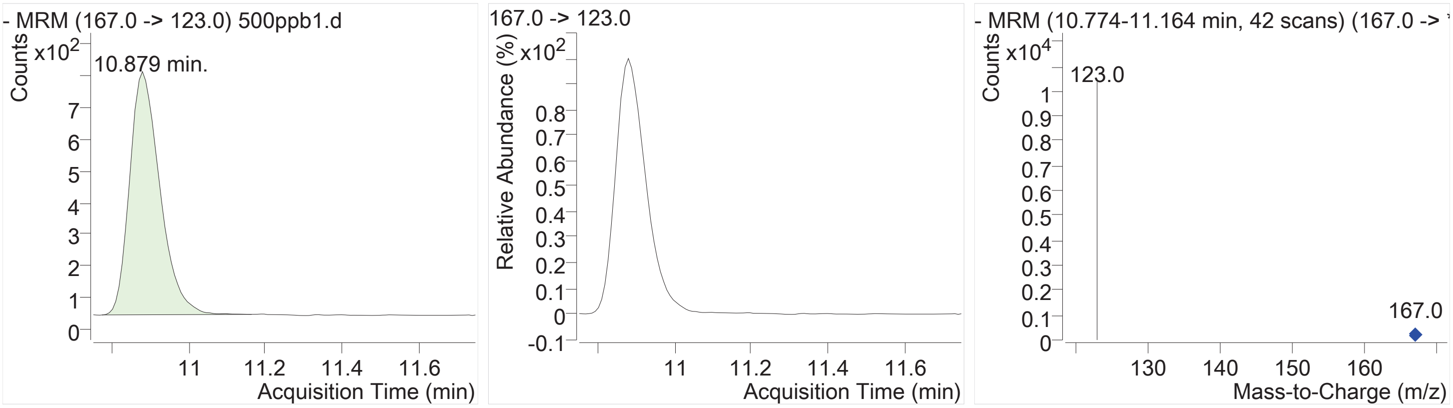

(+)-Catechin

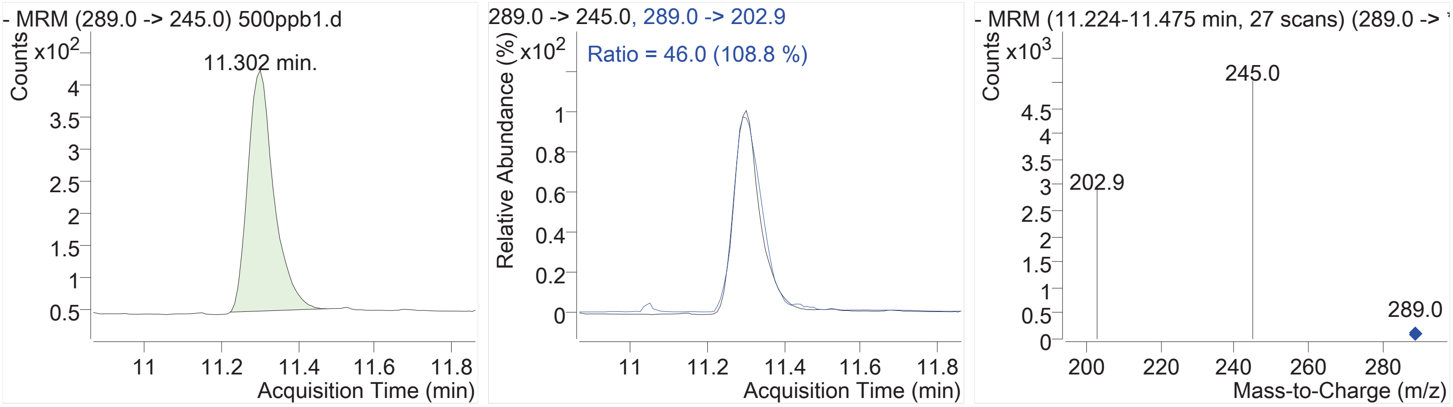

2,5-Dihydroxybenzoic acid

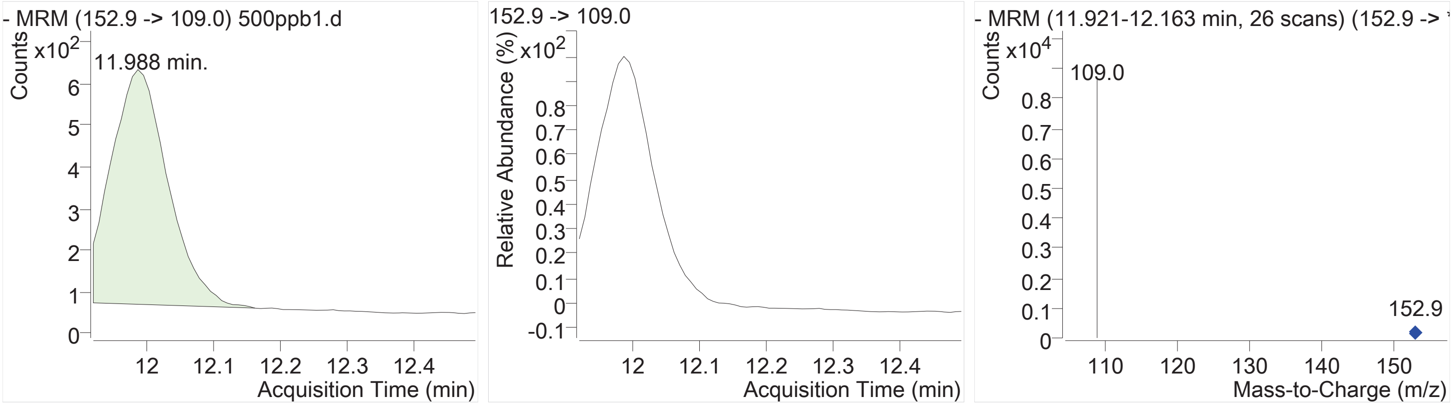

Chlorogenic acid

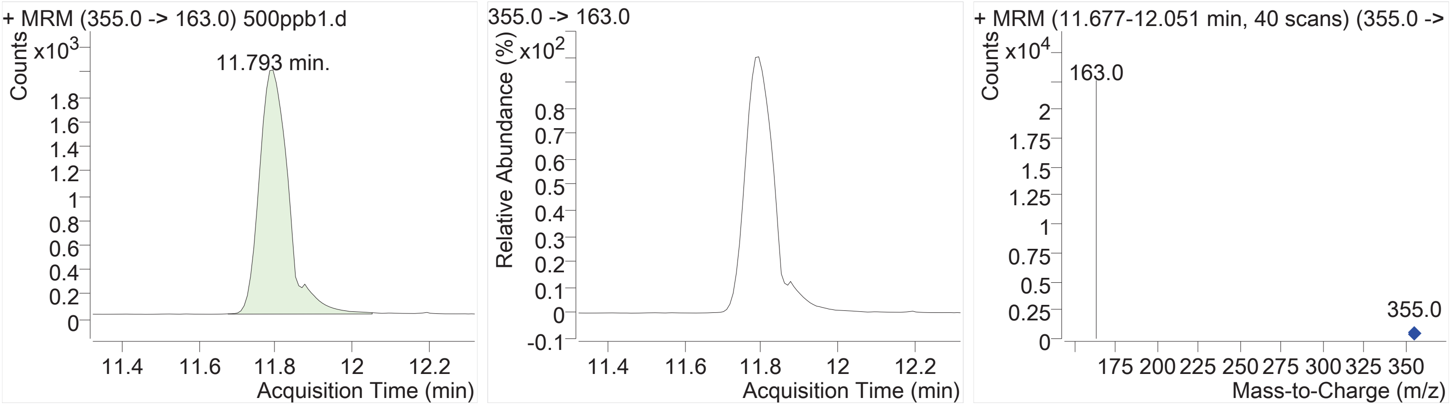

3-Hydroxybenzoic acid

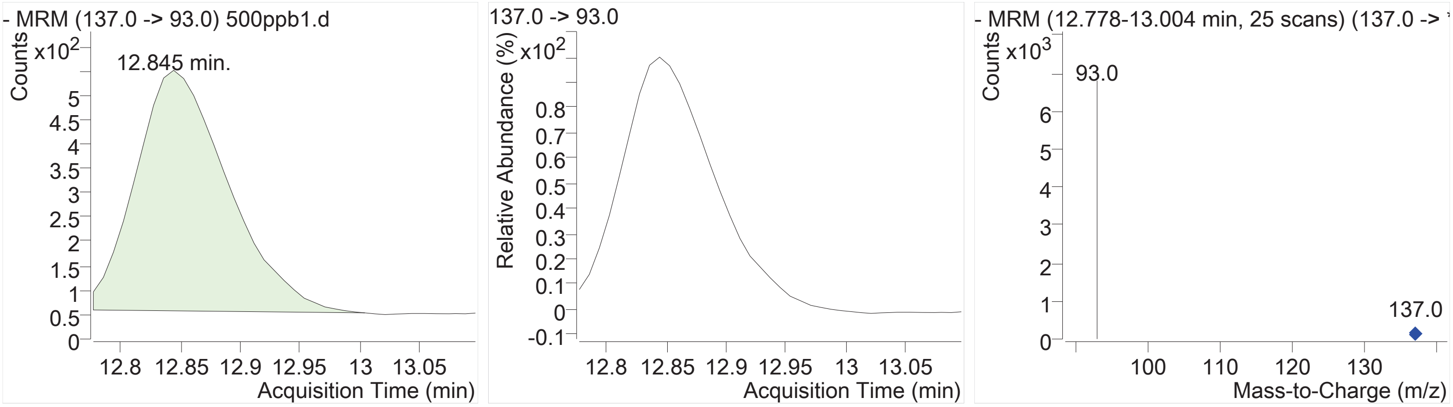

4-Hydroxybenzoic acid

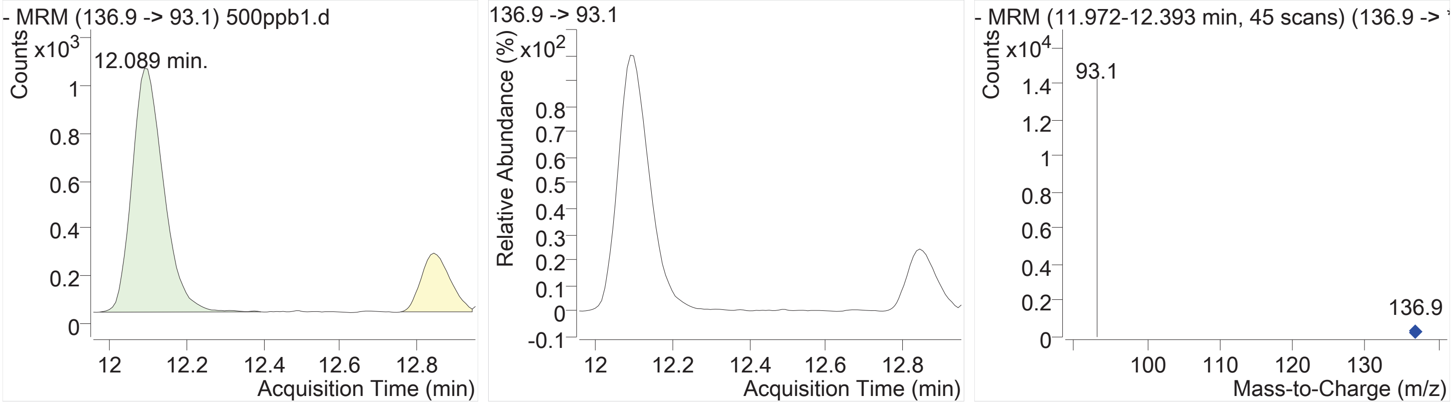

(-)-Epicatechin

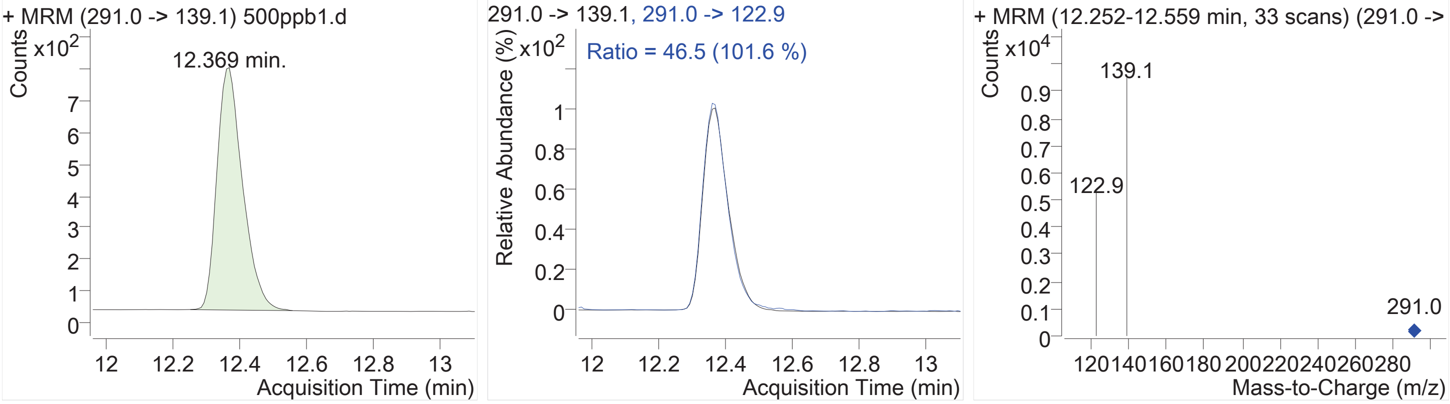

Caffeic acid

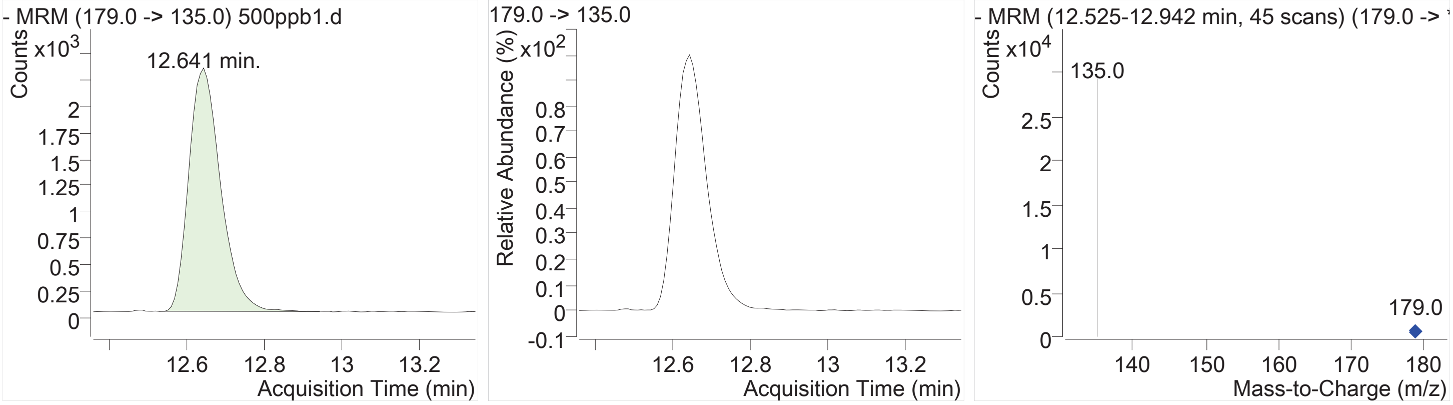

Syringic acid

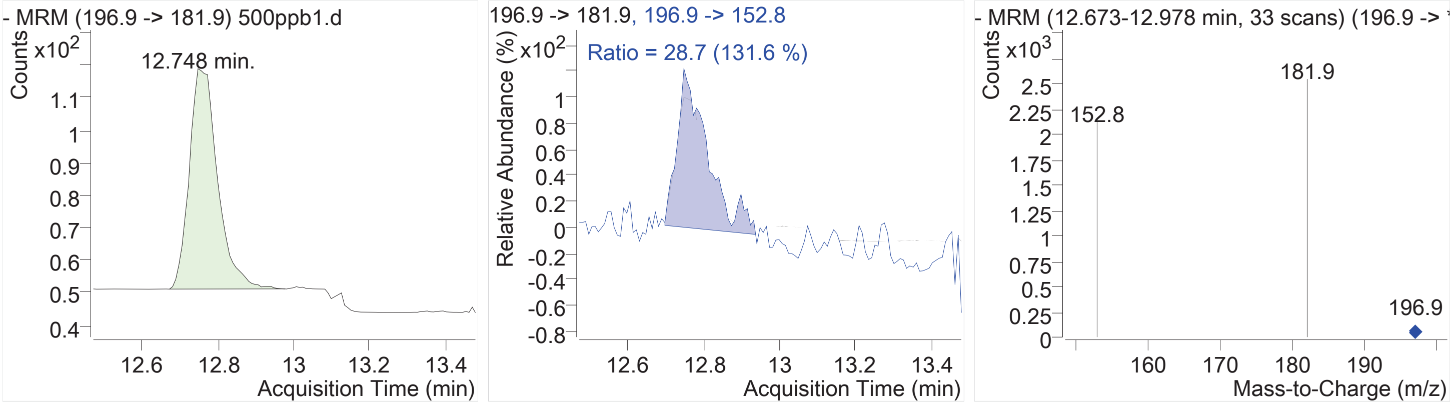

Vanillin

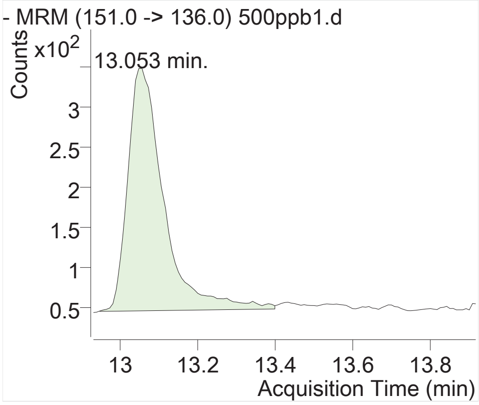

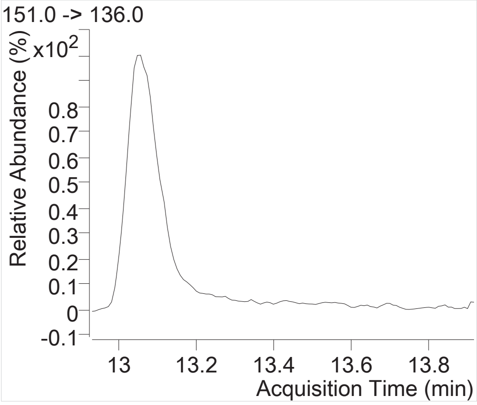

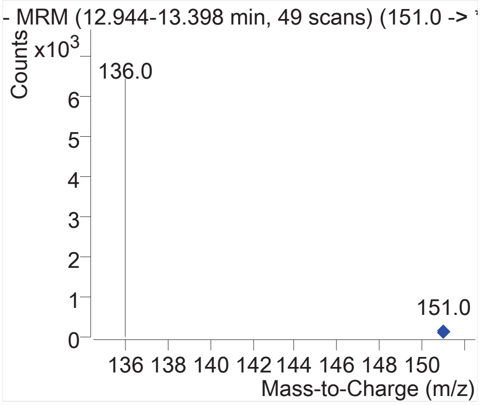

Verbascoside

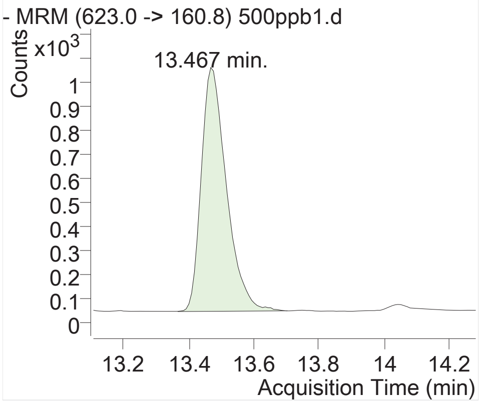

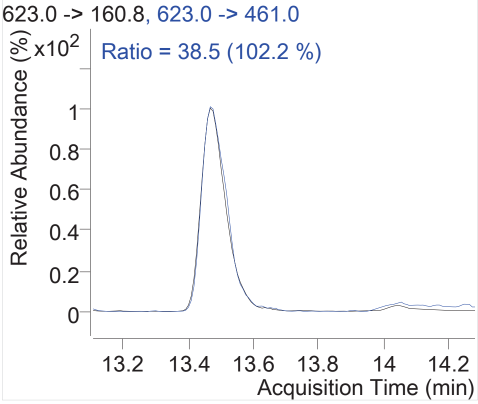

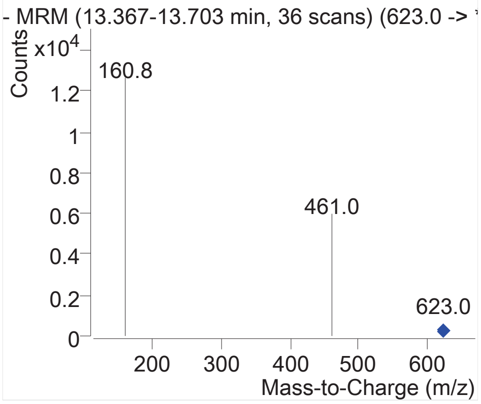

Taxifolin

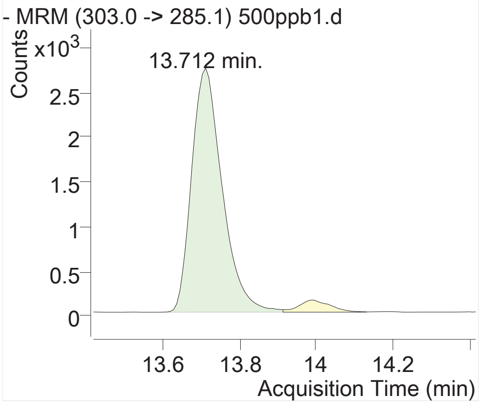

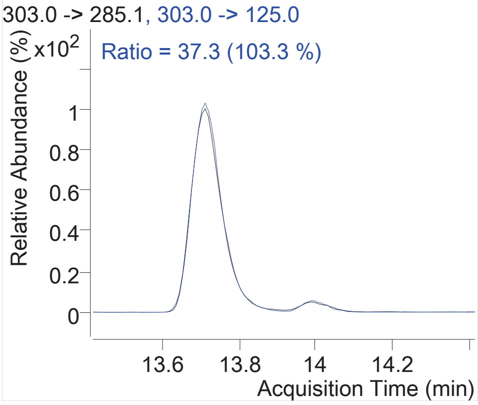

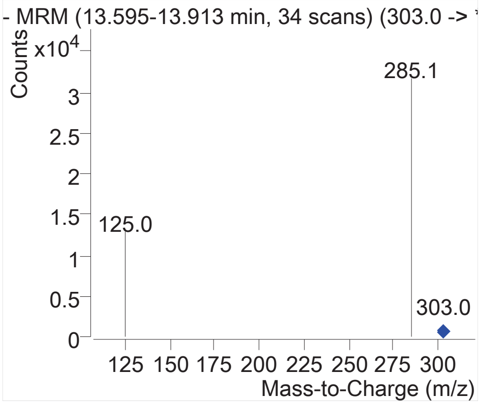

p-Coumaric acid

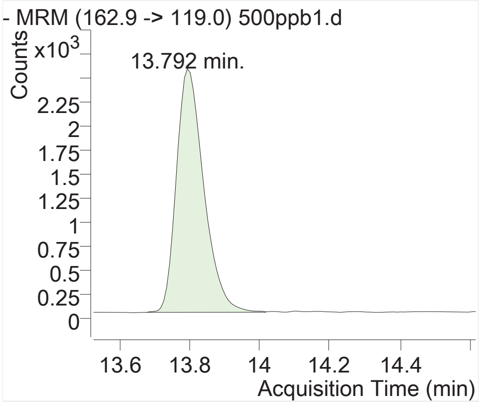

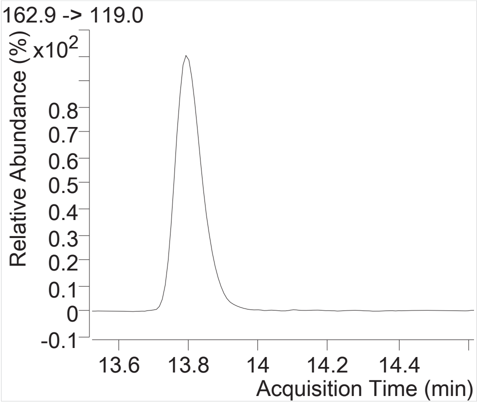

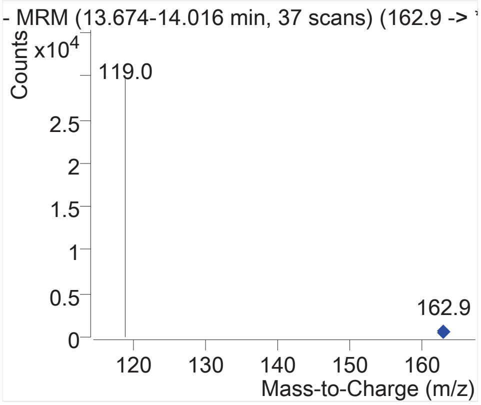

Sinapic acid

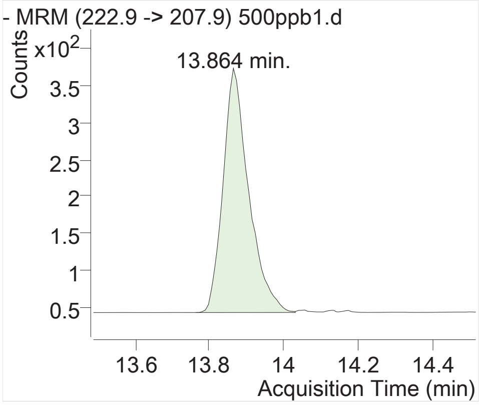

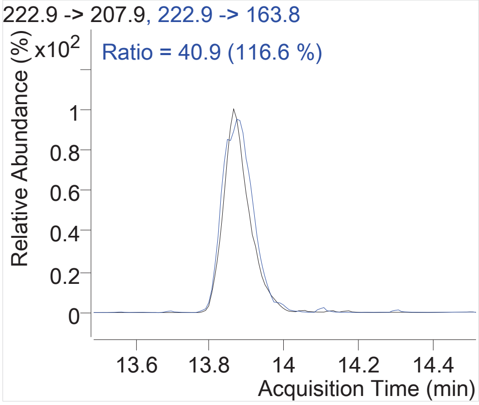

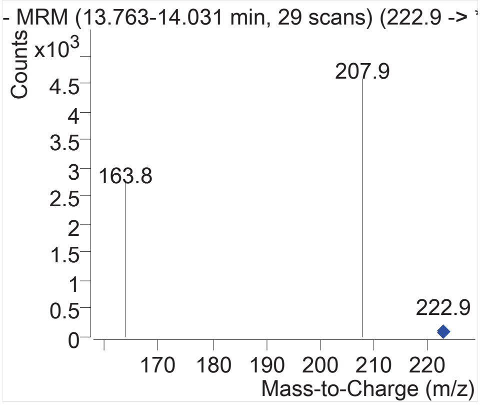

Ferulic acid

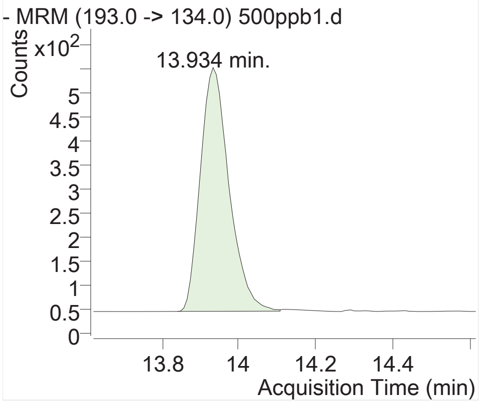

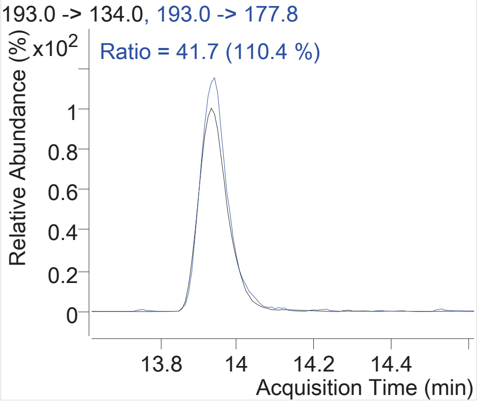

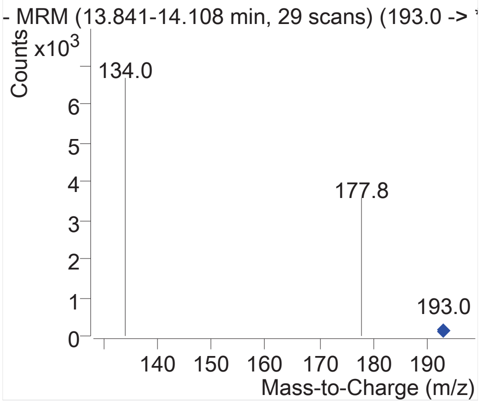

Luteolin 7-glucoside

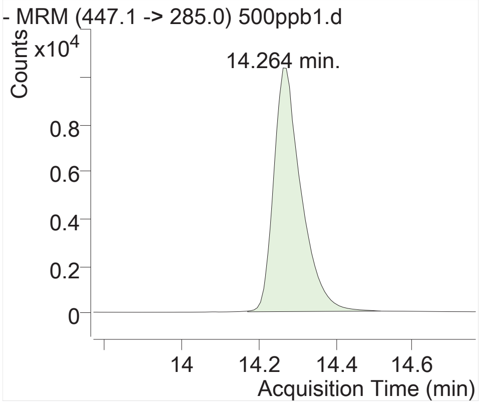

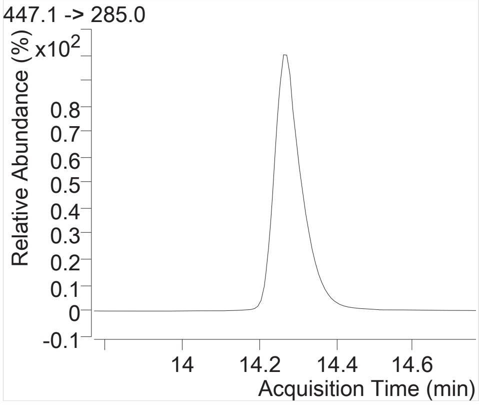

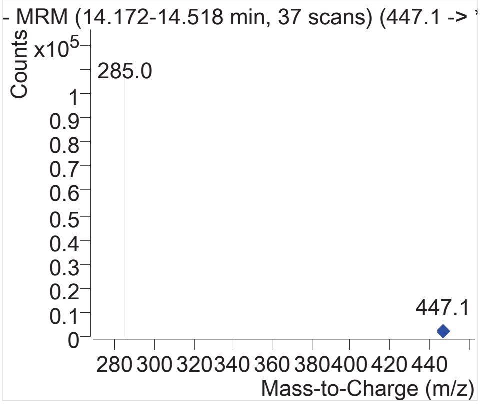

Hesperidin

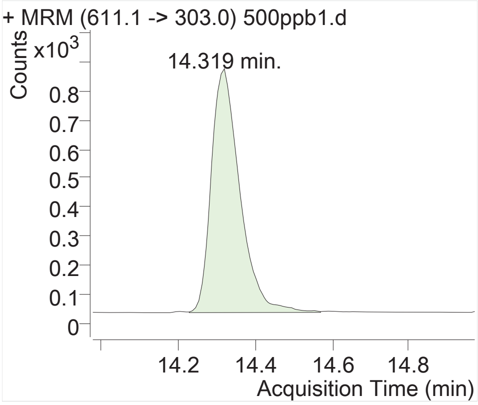

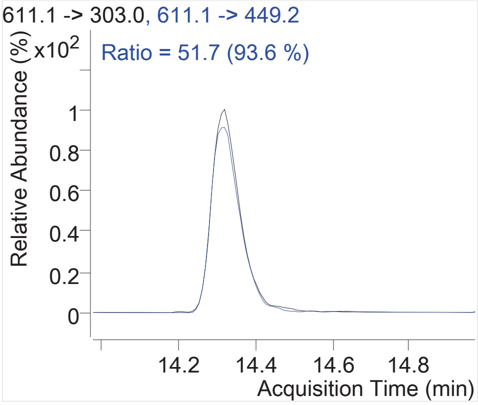

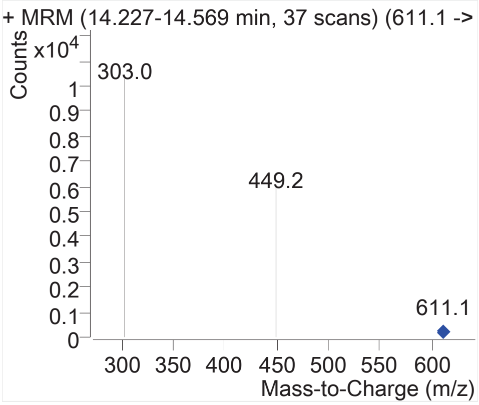

Hyperoside

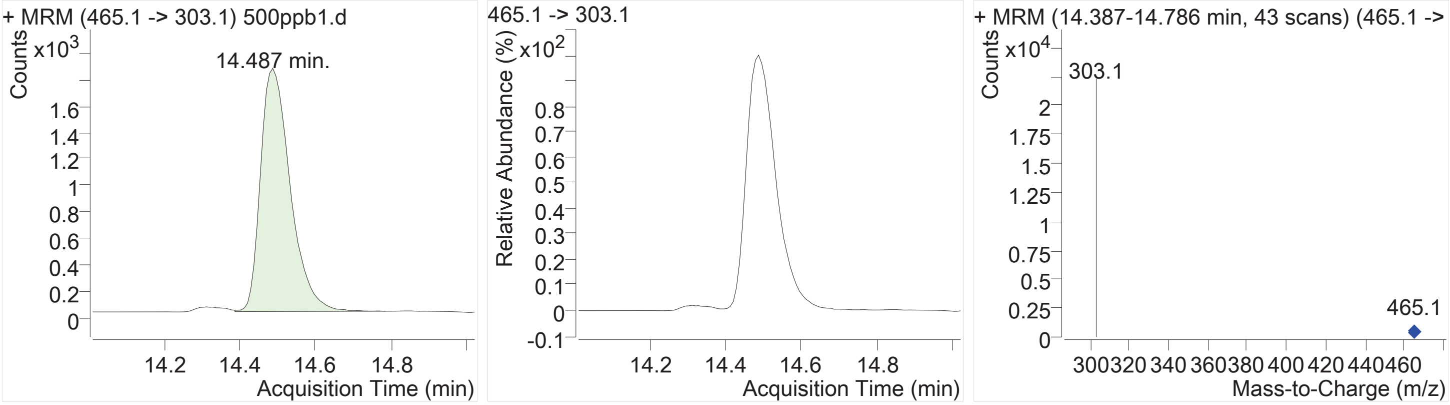

Rosmarinic acid

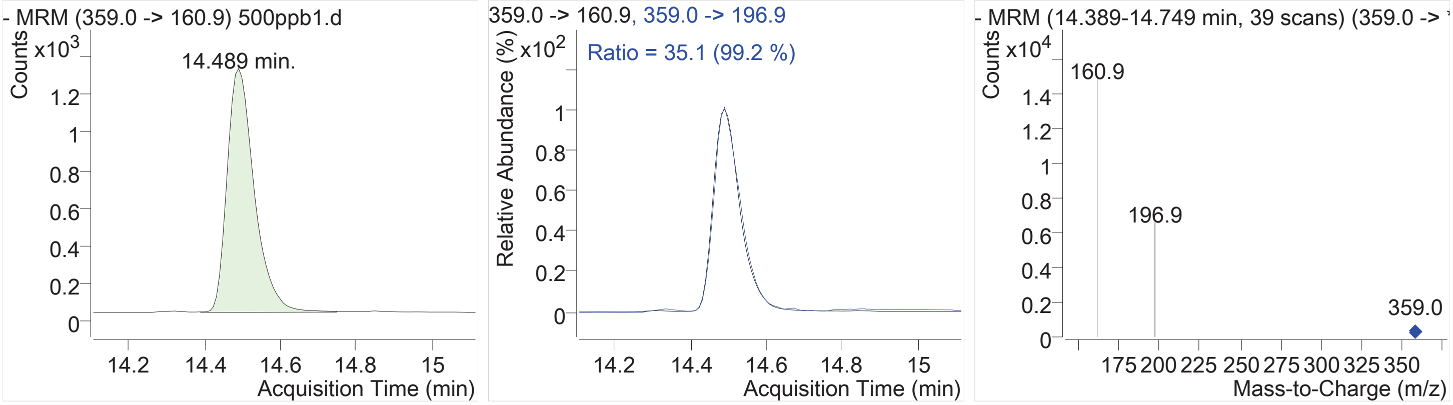

Apigenin 7-glucoside

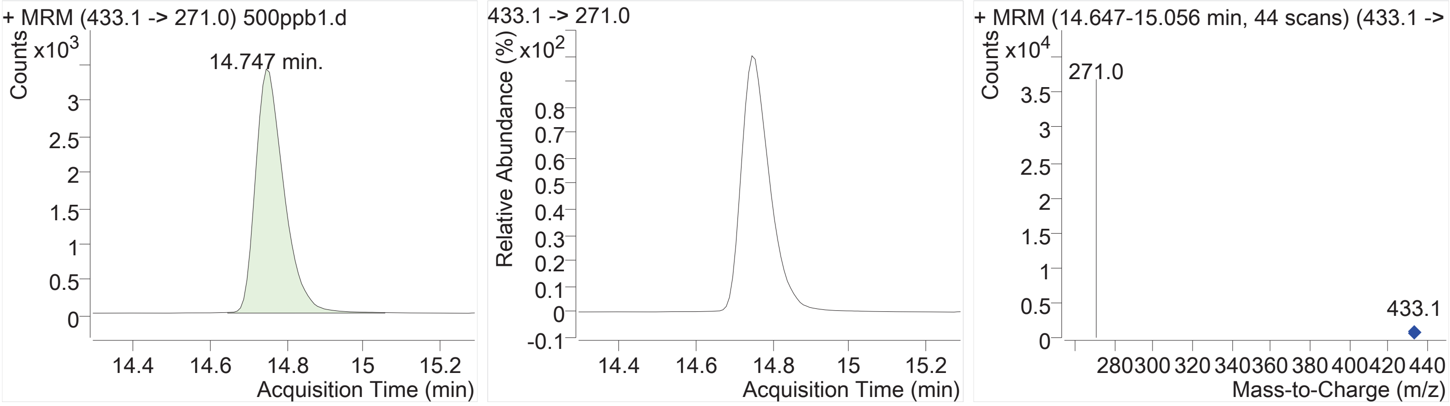

Pinoresinol

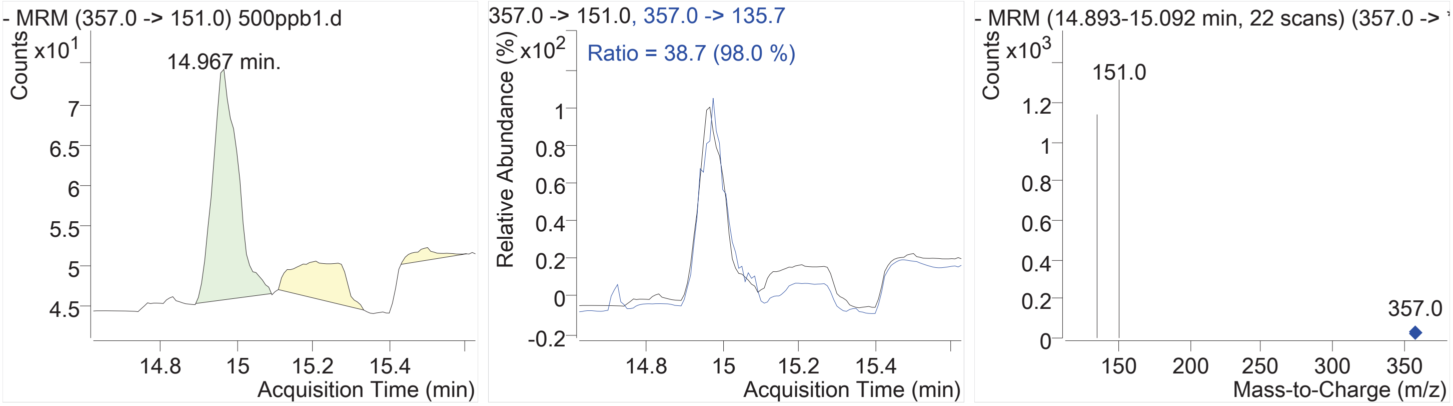

2-Hydroxycinnamic acid

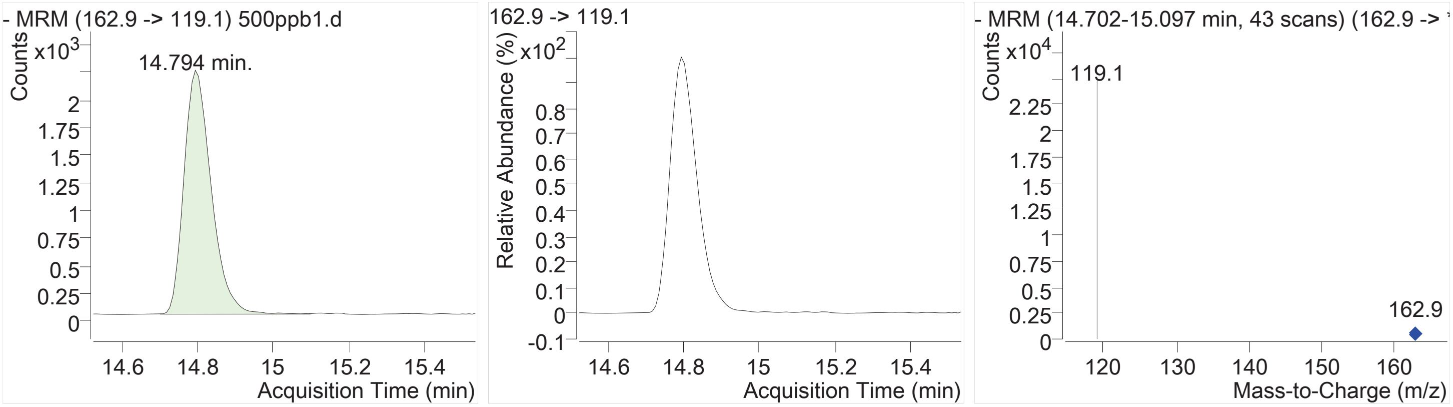

Eriodictyol

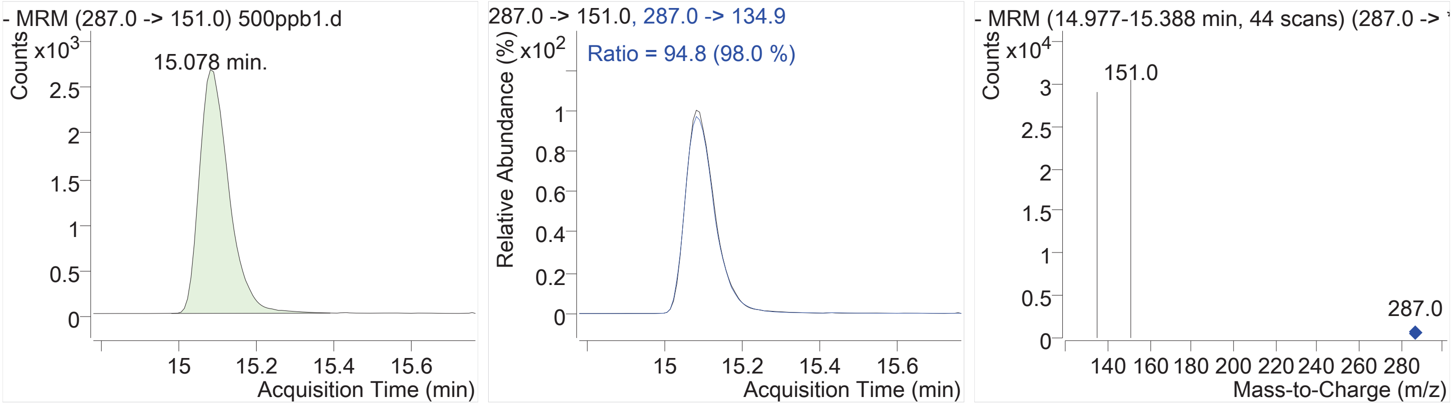

Quercetin

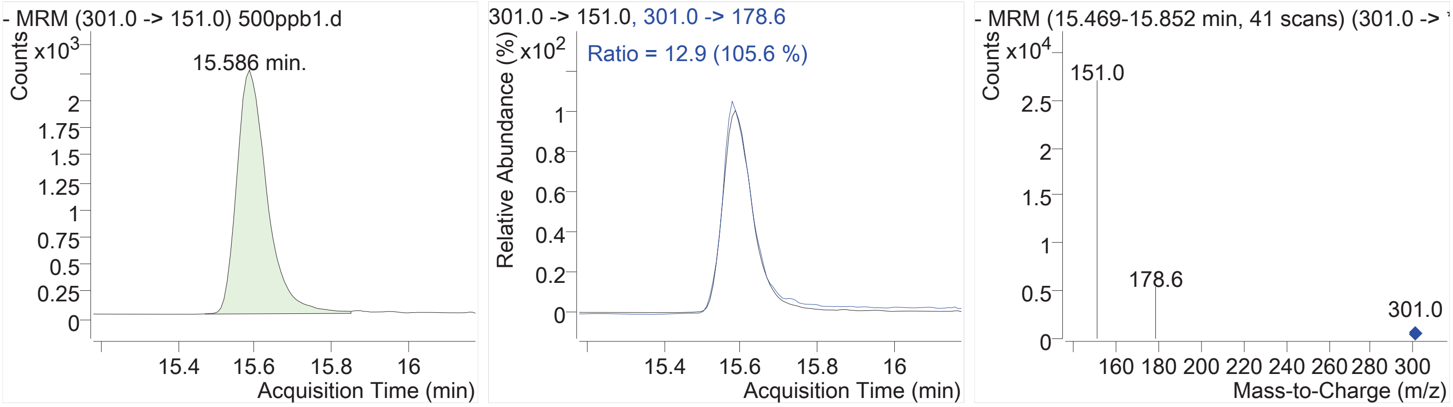

Luteolin

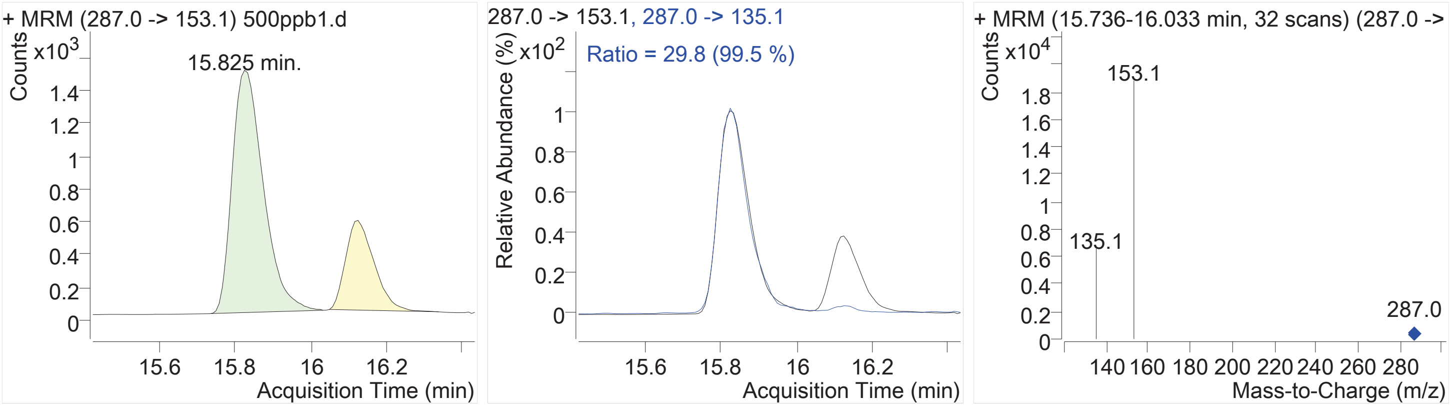

Agilent Technologies

Kaempferol

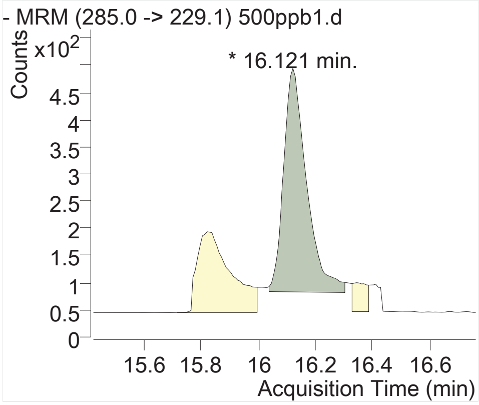

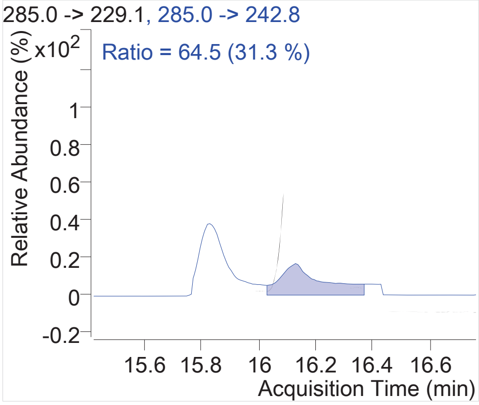

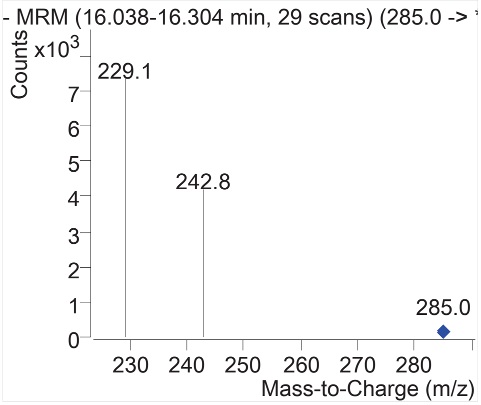

Apigenin

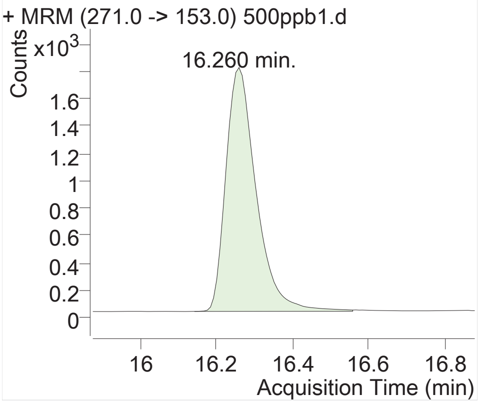

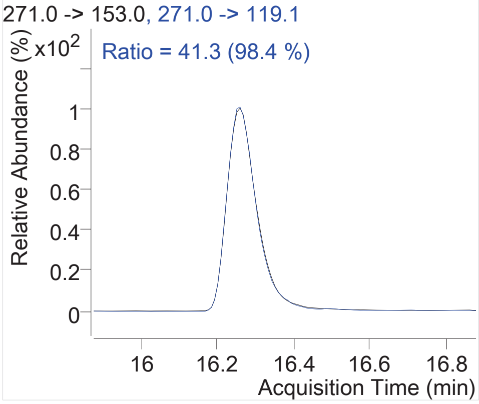

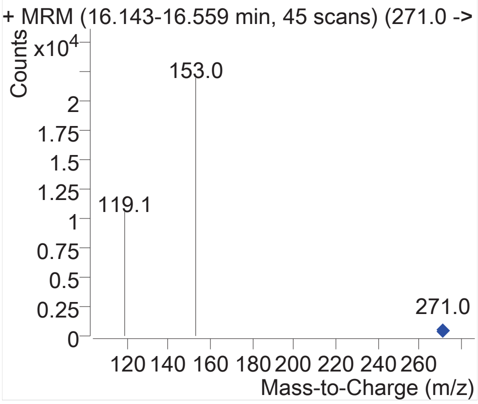

Quantitative Analysis Complete Report

**Batch Path**	D:\MassHunter\Data\2022ekim\061022cengizhoca\QuantResults\071022.batch.bin		
**Analysis Time**	10/11/2022 1:33:26 PM	**Analyst Name**	Defam-PC\admin
**Report Time**	10/11/2022 1:34:29 PM	**Reporter Name**	admin
**Last Calib Update**	10/11/2022 1:33:17 PM	**Batch State**	Processed
**Quant Batch Version**	B.07.01	**Quant Report Version**	B.07.01

**Acq. Time**	10/6/2022 8:37:01 PM	**Data File**	500ppb2.d
**Sample Type**	Cal	**Sample Name**	500ppb2
**Dilution**	1	**Acq. Method**	FENOLIK_DMRM2021-31bilesen.m

Sample Chromatogram

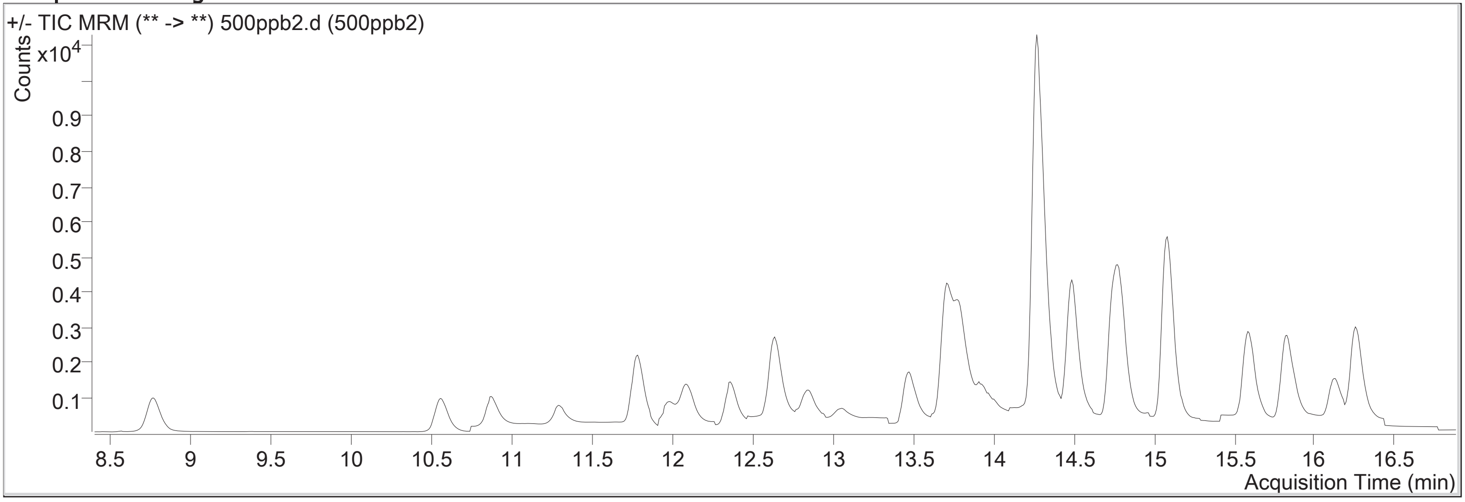

Compound	Transition	RT	Resp.	Final Conc	Units
Gallic acid	168.9 -> 125.0	8.775	6025	544.2341	ng/ml
Protocatechuic acid	152.9 -> 108.9	10.559	5576	527.7816	ng/ml
Pyrocatechol	109.0 -> 52.9	10.831	151	480.0042	ng/ml
3,4-Dihydroxyphenylacetic acid	167.0 -> 123.0	10.879	4171	517.5872	ng/ml
(+)-Catechin	289.0 -> 245.0	11.294	1869	546.2426	ng/ml
2,5-Dihydroxybenzoic acid	152.9 -> 109.0	11.988	3589	567.7118	ng/ml
Chlorogenic acid	355.0 -> 163.0	11.793	11244	533.5764	ng/ml
3-Hydroxybenzoic acid	137.0 -> 93.0	12.853	2732	525.9839	ng/ml
4-Hydroxybenzoic acid	136.9 -> 93.1	12.089	5860	530.6833	ng/ml
(-)-Epicatechin	291.0 -> 139.1	12.369	4097	519.3311	ng/ml
Caffeic acid	179.0 -> 135.0	12.641	13046	534.1772	ng/ml
Syringic acid	196.9 -> 181.9	12.765	418	586.0106	ng/ml
Vanillin	151.0 -> 136.0	13.062	1761	507.1726	ng/ml
Verbascoside	623.0 -> 160.8	13.476	5287	516.6662	ng/ml
Taxifolin	303.0 -> 285.1	13.712	14931	549.1162	ng/ml
p-Coumaric acid	162.9 -> 119.0	13.801	13748	527.3728	ng/ml
Sinapic acid	222.9 -> 207.9	13.864	1652	525.1850	ng/ml
Ferulic acid	193.0 -> 134.0	13.934	2724	522.9891	ng/ml
Luteolin 7-glucoside	447.1 -> 285.0	14.272	51110	527.8735	ng/ml
Hesperidin	611.1 -> 303.0	14.319	4313	527.5245	ng/ml
Hyperoside	465.1 -> 303.1	14.495	10310	525.7668	ng/ml
Rosmarinic acid	359.0 -> 160.9	14.489	6530	520.9417	ng/ml
Apigenin 7-glucoside	433.1 -> 271.0	14.755	17975	533.2879	ng/ml
Pinoresinol	357.0 -> 151.0	14.967	132	492.0144	ng/ml
2-Hydroxycinnamic acid	162.9 -> 119.1	14.802	11001	516.9123	ng/ml
Eriodictyol	287.0 -> 151.0	15.087	14212	545.2018	ng/ml
Quercetin	301.0 -> 151.0	15.586	12240	527.8523	ng/ml
Luteolin	287.0 -> 153.1	15.833	8691	544.1054	ng/ml
Kaempferol	285.0 -> 229.1	16.130	2325	547.3324	ng/ml

Compound	Transition	RT	Resp.	Final Conc	Units
Apigenin	271.0 -> 153.0	16.260	10152	543.6772	ng/ml

Gallic acid

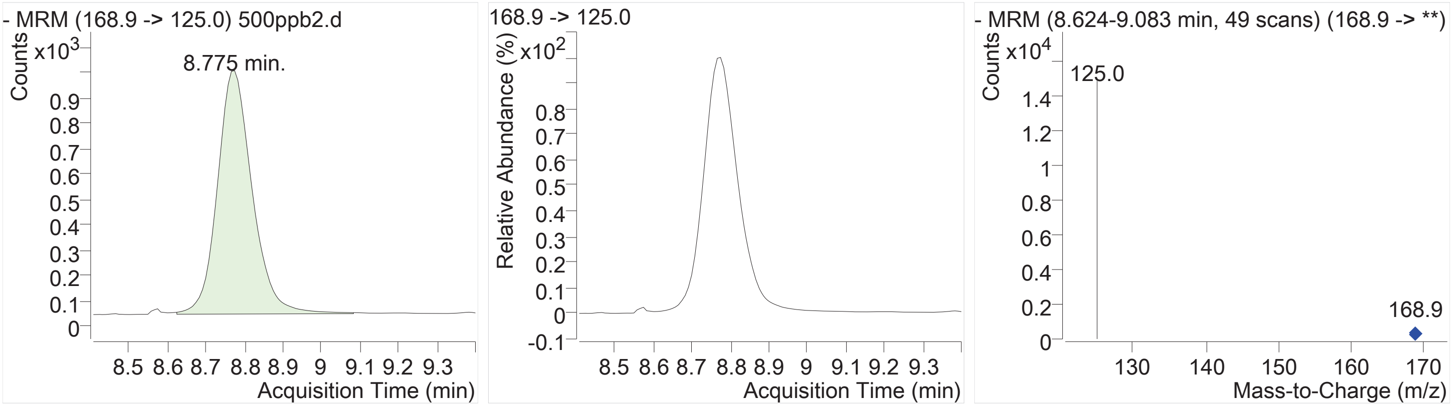

Protocatechuic acid

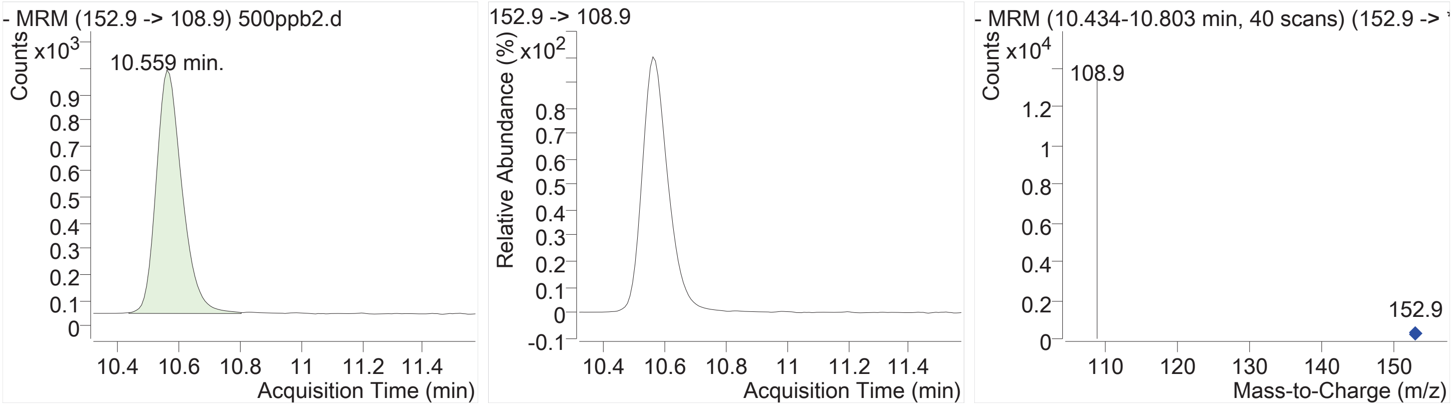

Pyrocatechol

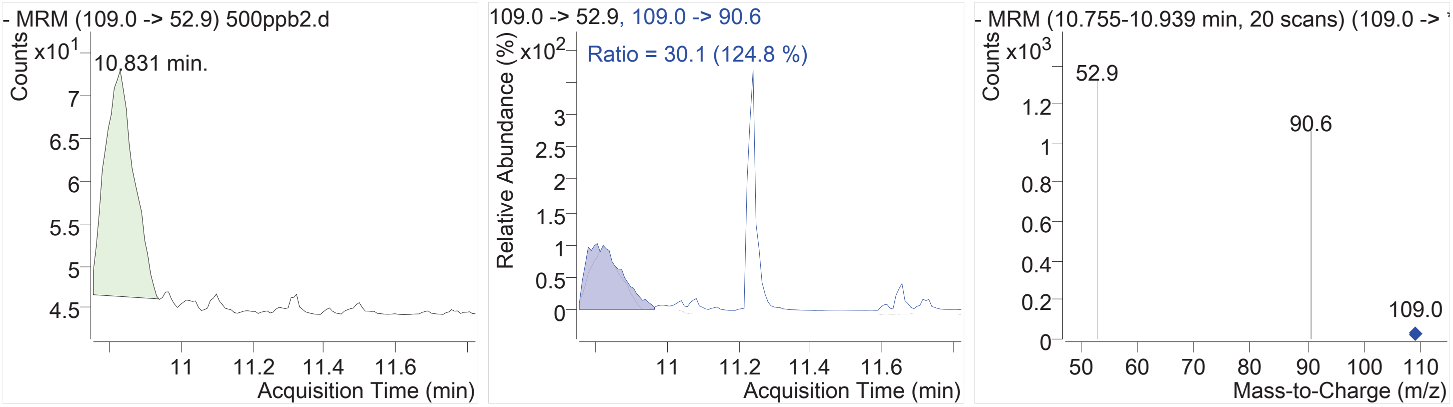

3,4-Dihydroxyphenylacetic acid

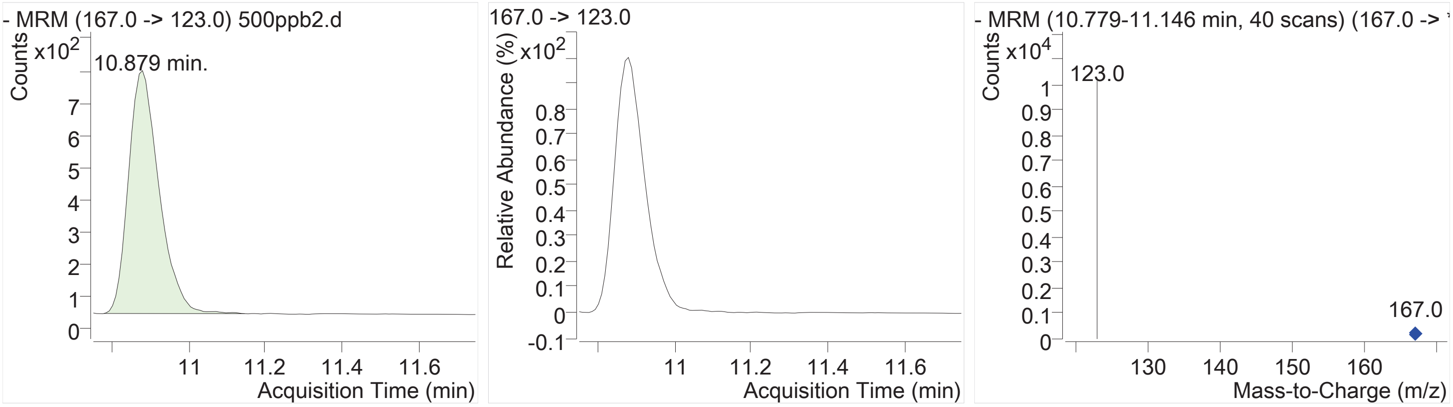

(+)-Catechin

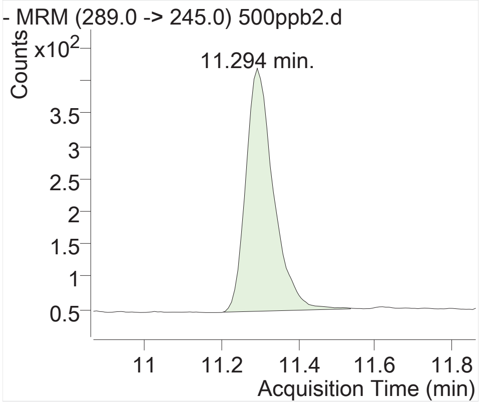

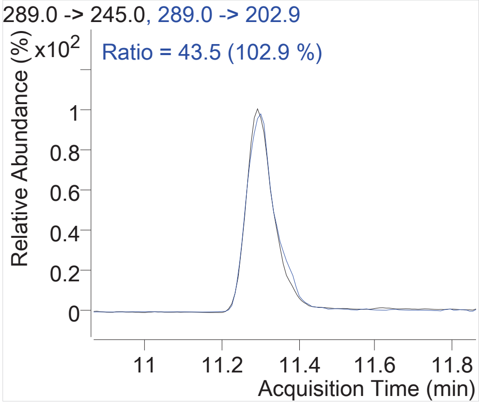

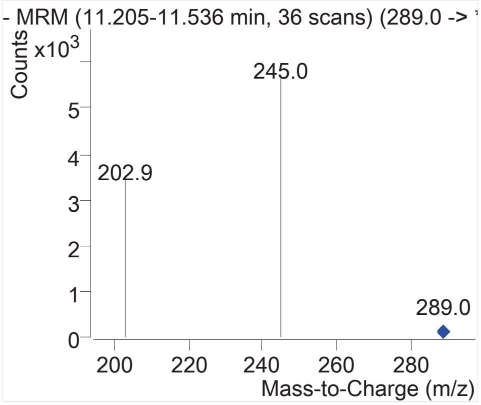

2,5-Dihydroxybenzoic acid

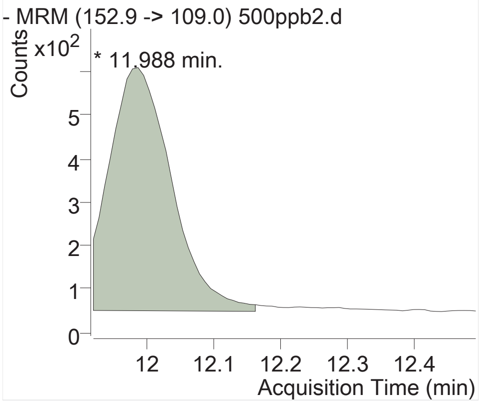

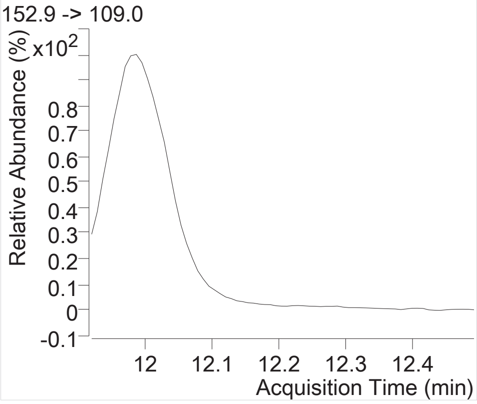

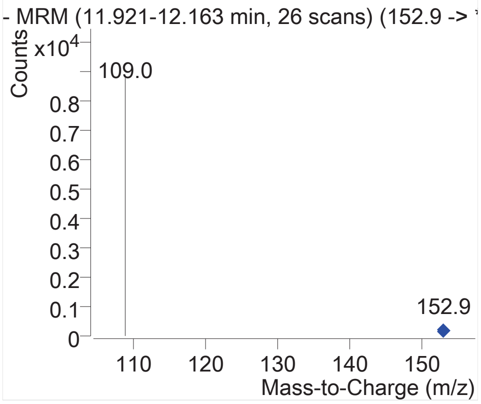

Chlorogenic acid

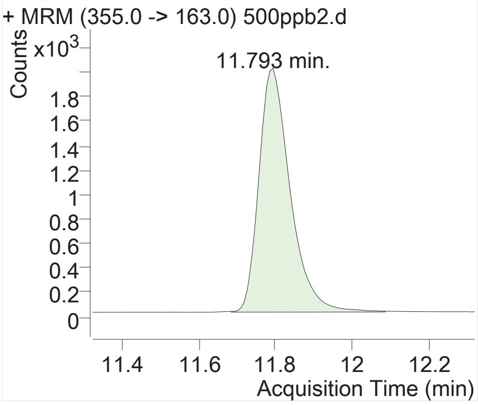

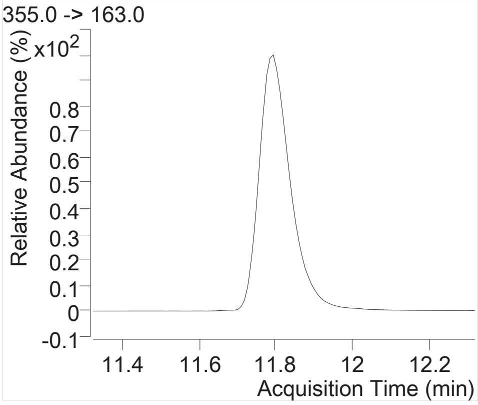

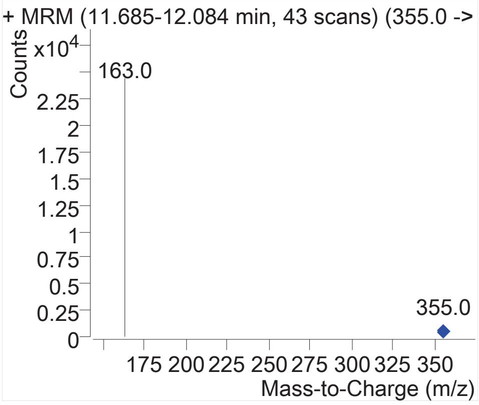

3-Hydroxybenzoic acid

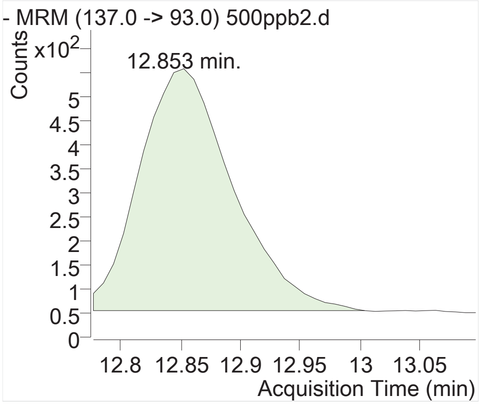

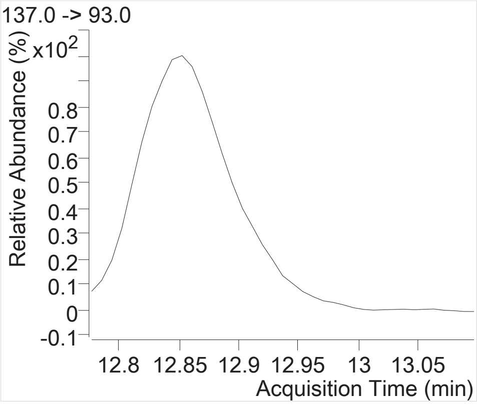

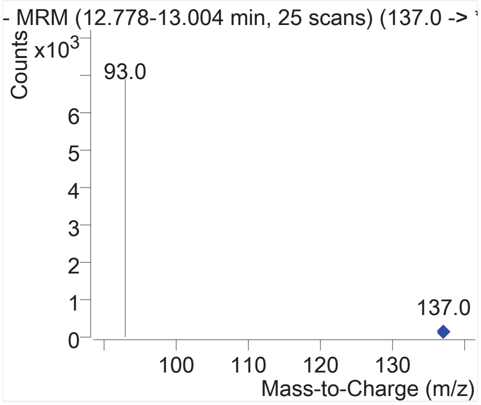

4-Hydroxybenzoic acid

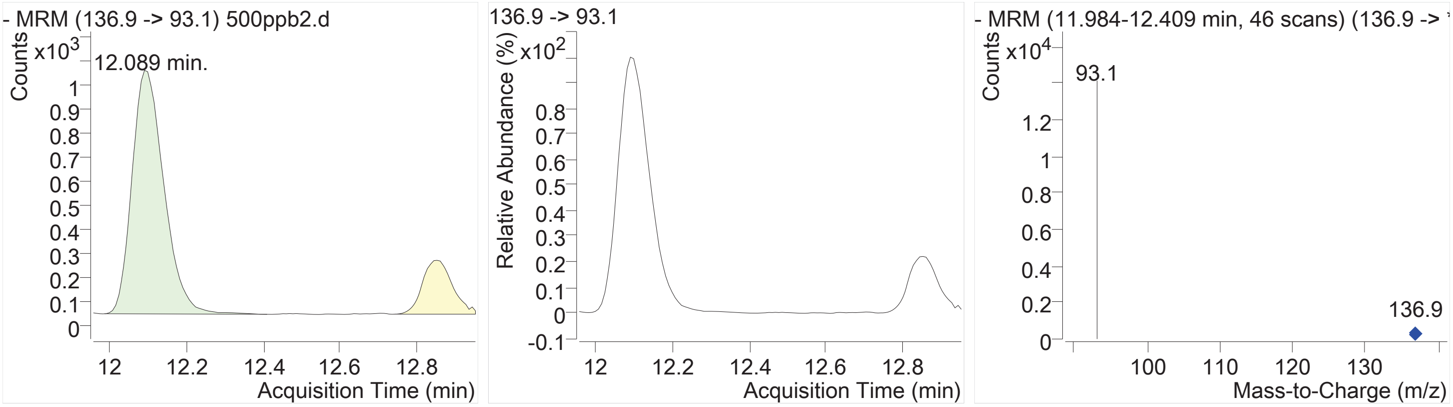

(-)-Epicatechin

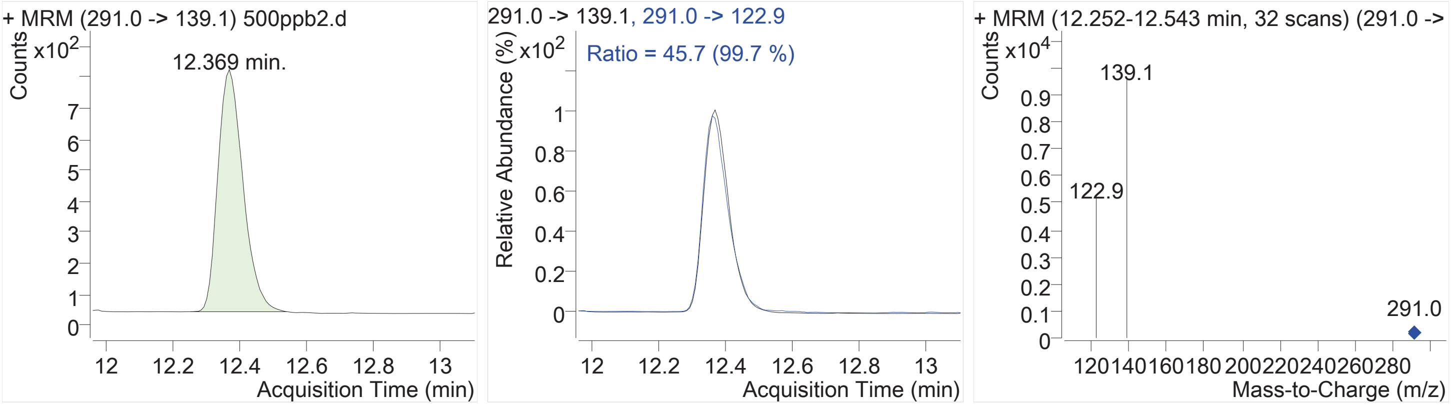

Caffeic acid

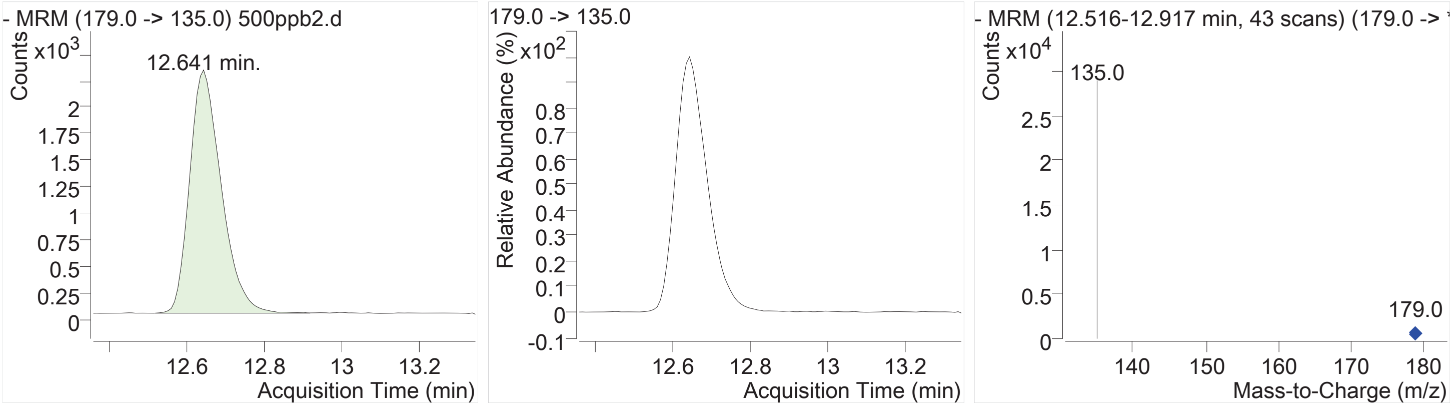

Syringic acid

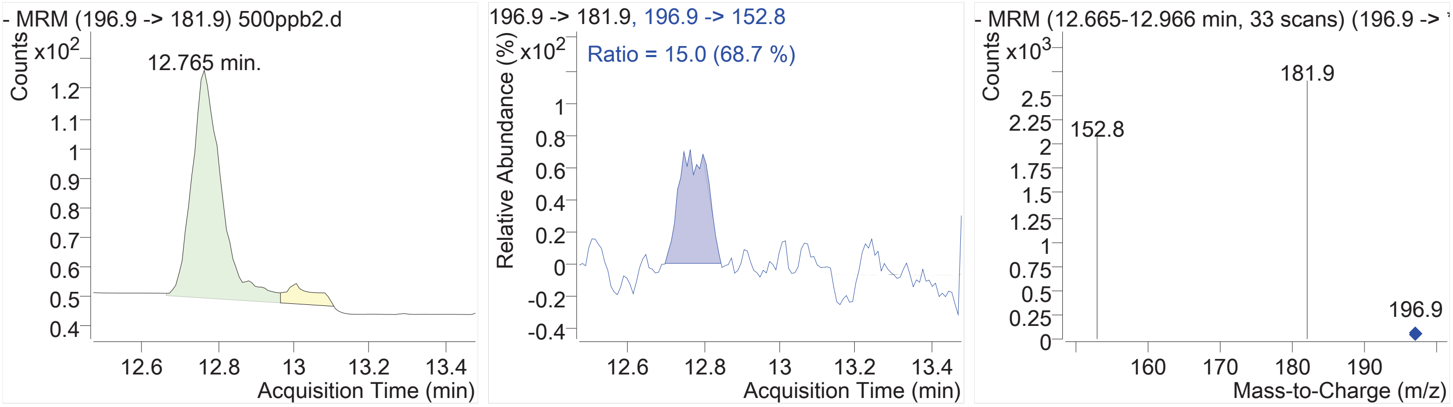

Agilent Technologies

Vanillin

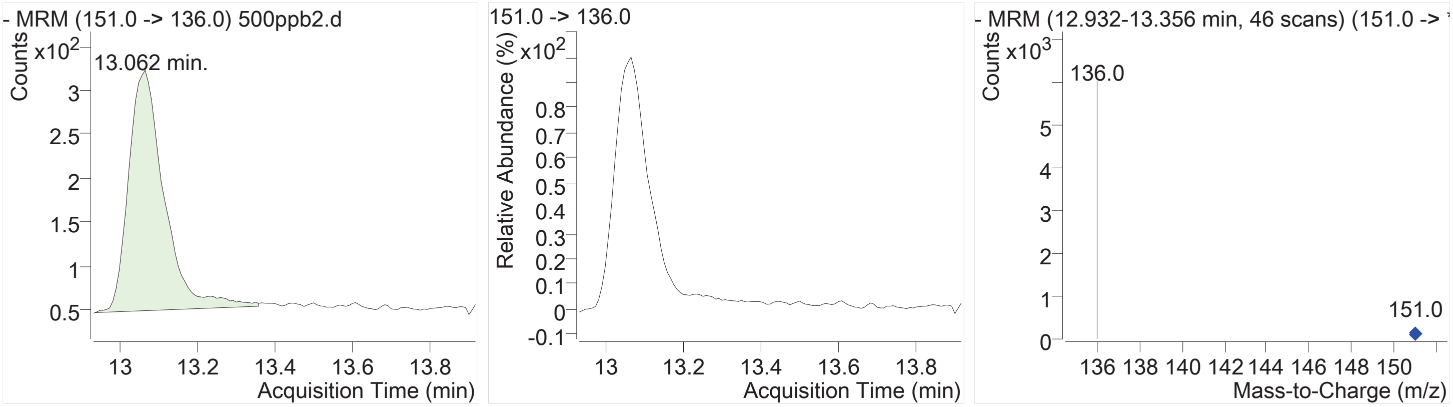

Verbascoside

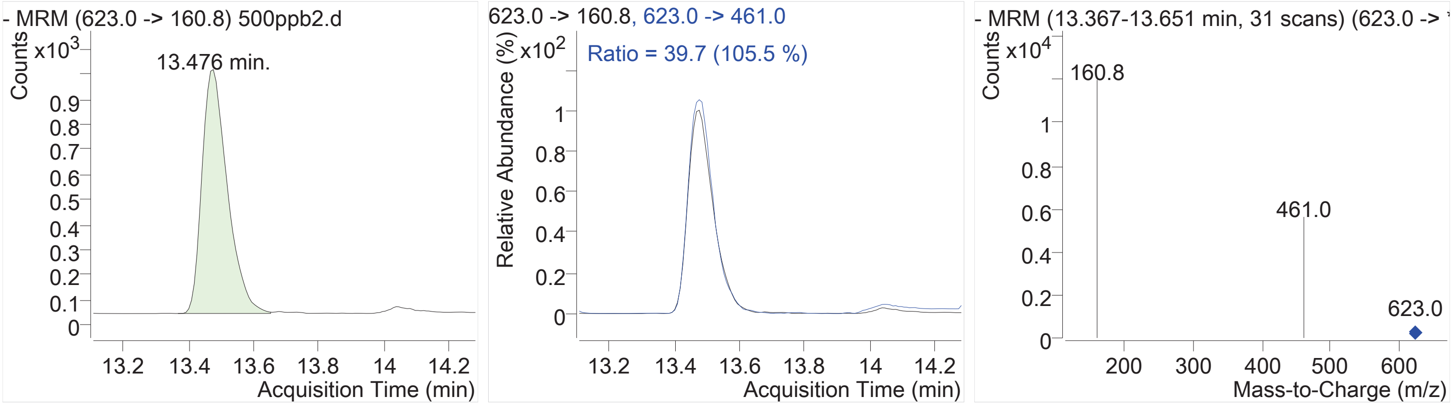

Taxifolin

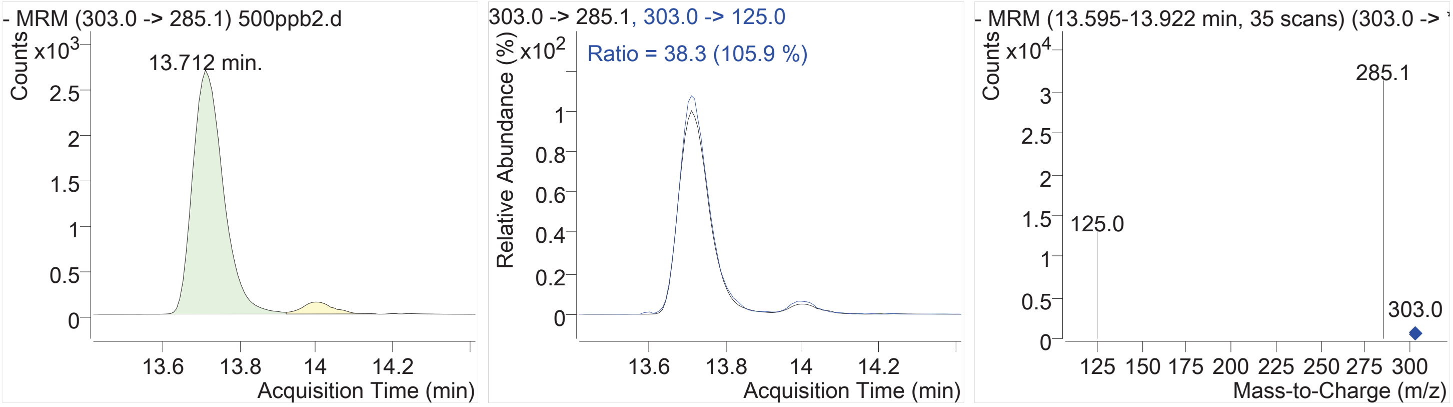

p-Coumaric acid

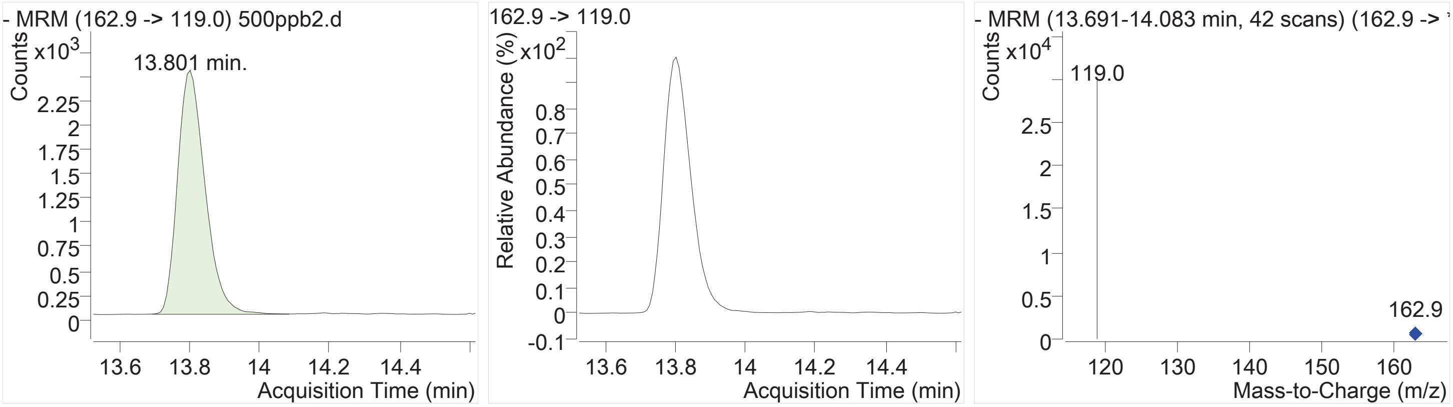

Sinapic acid

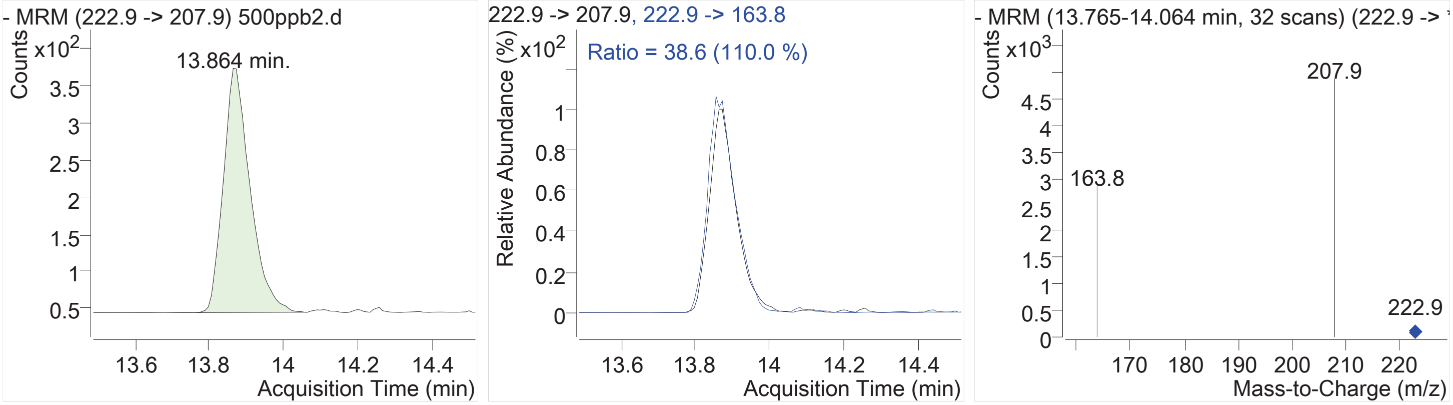

Ferulic acid

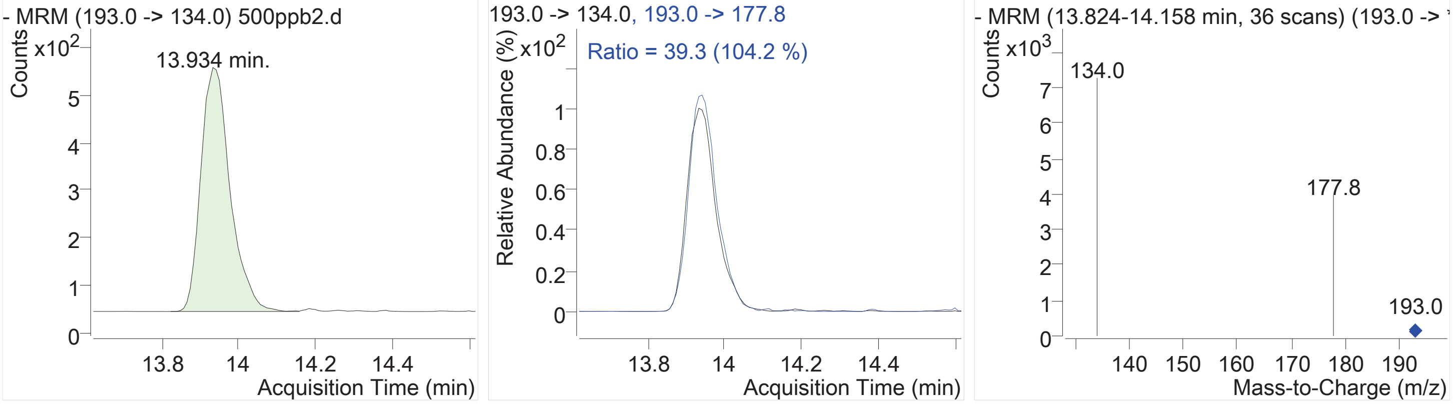

Luteolin 7-glucoside

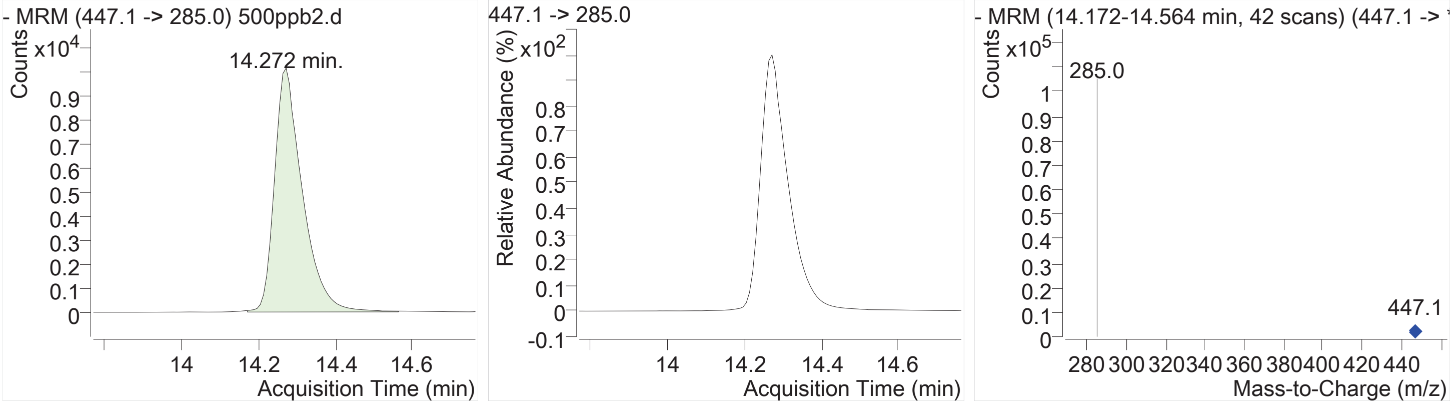

Hesperidin

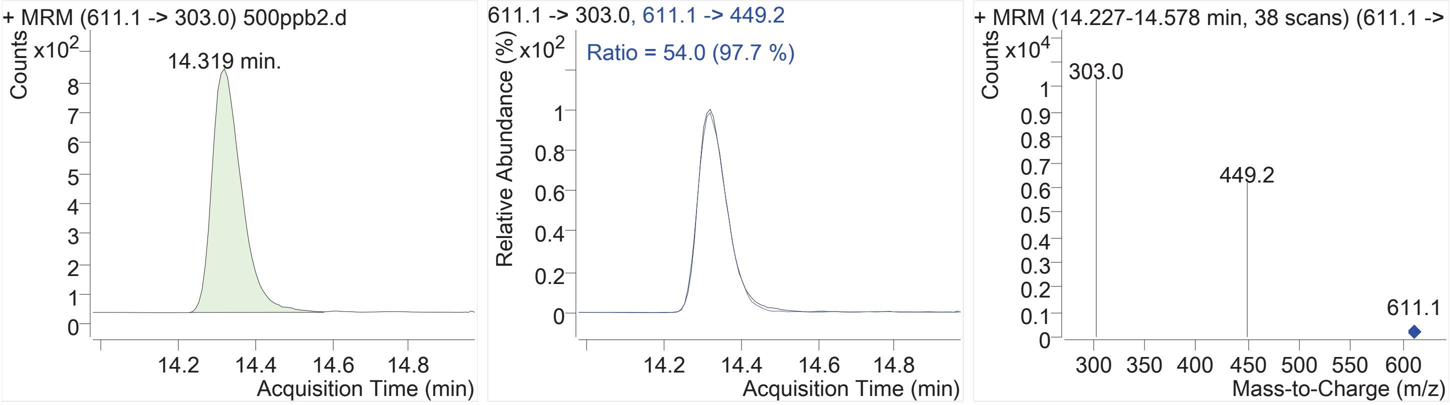

Hyperoside

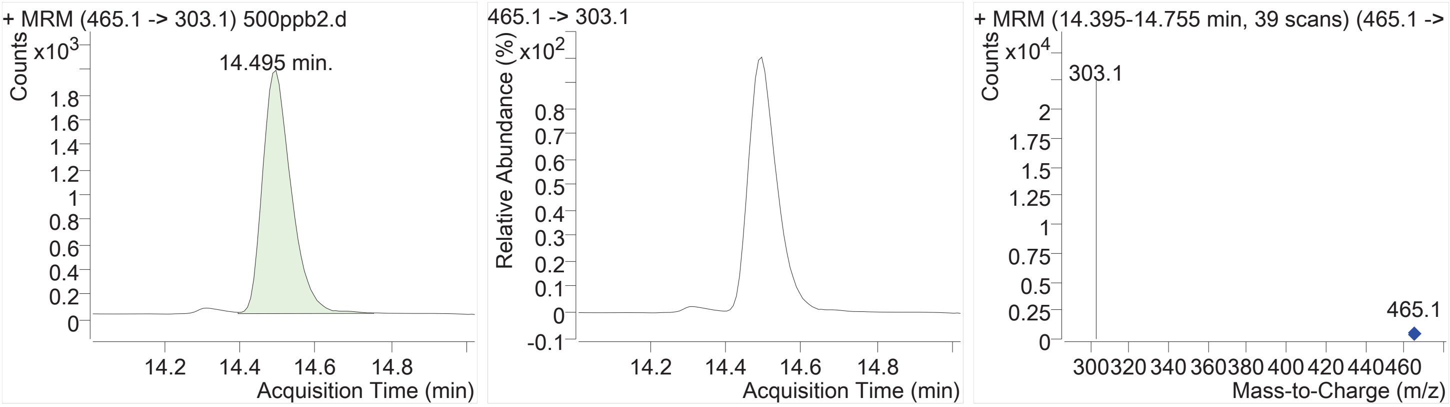

Rosmarinic acid

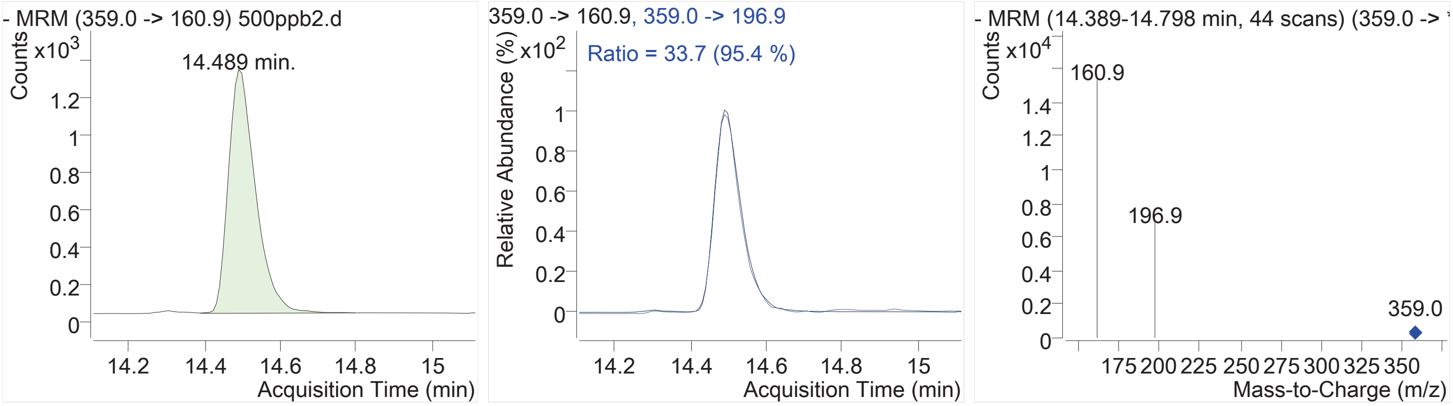

Apigenin 7-glucoside

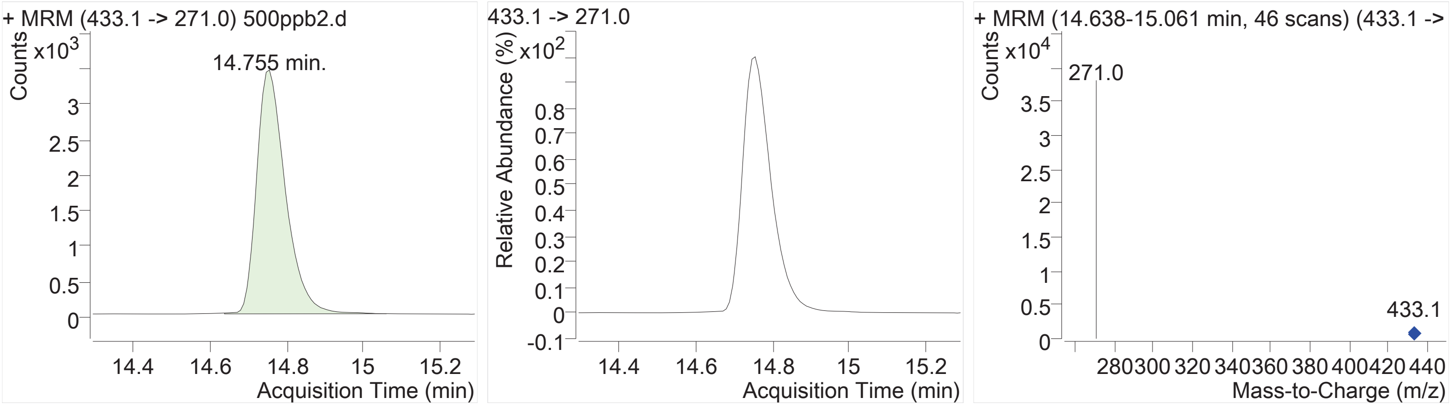

Pinoresinol

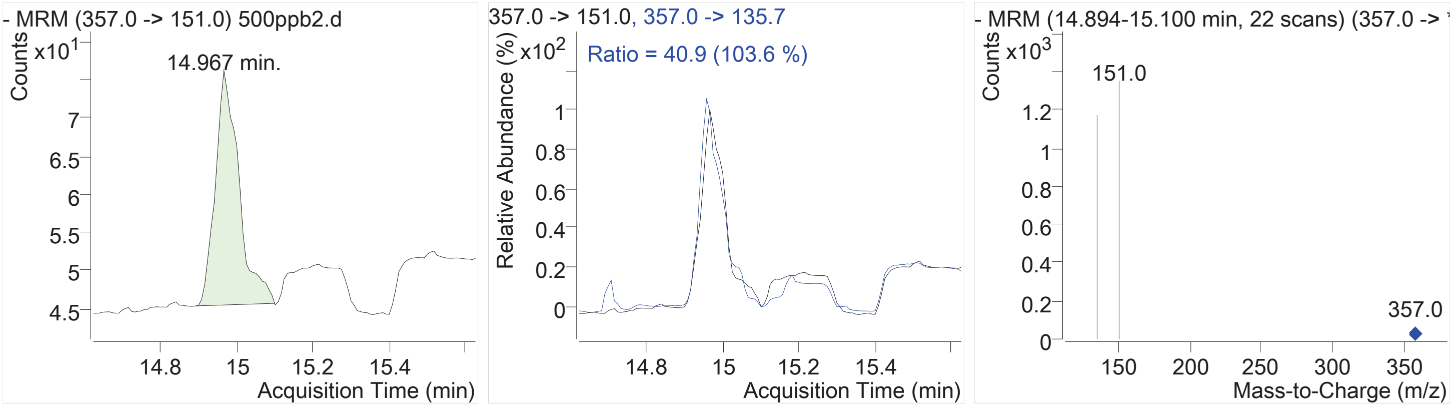

2-Hydroxycinnamic acid

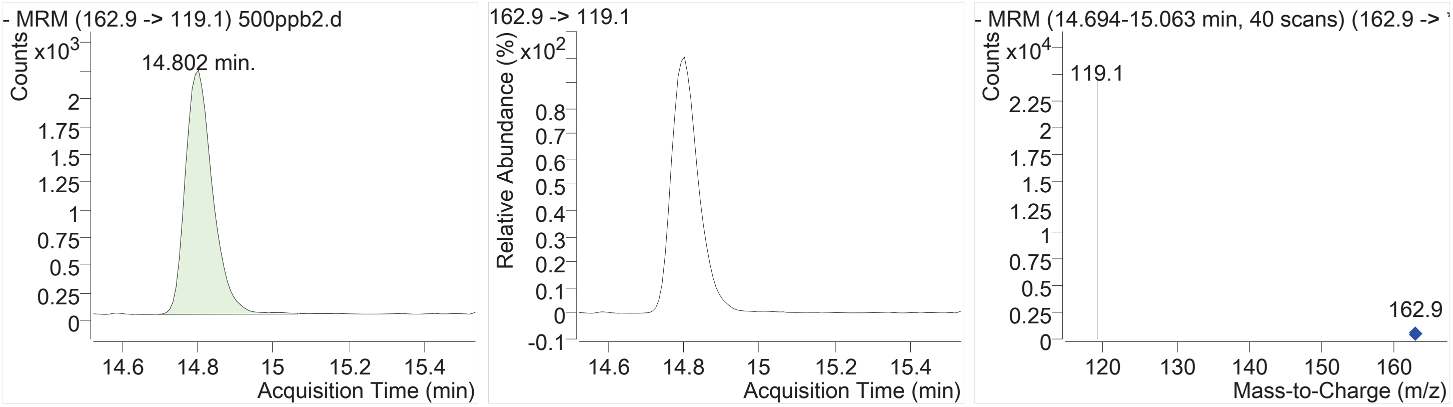

Eriodictyol

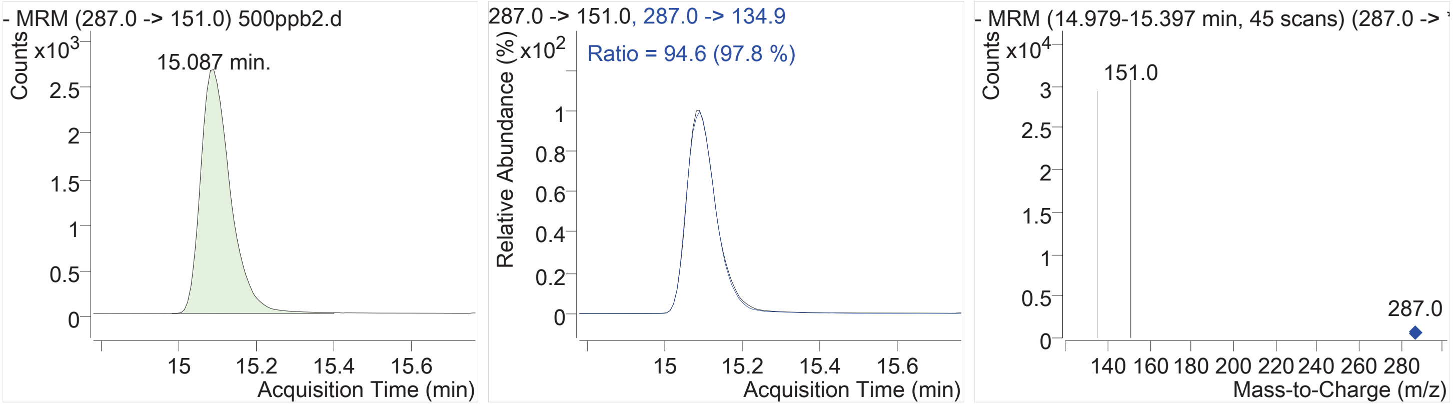

Quercetin

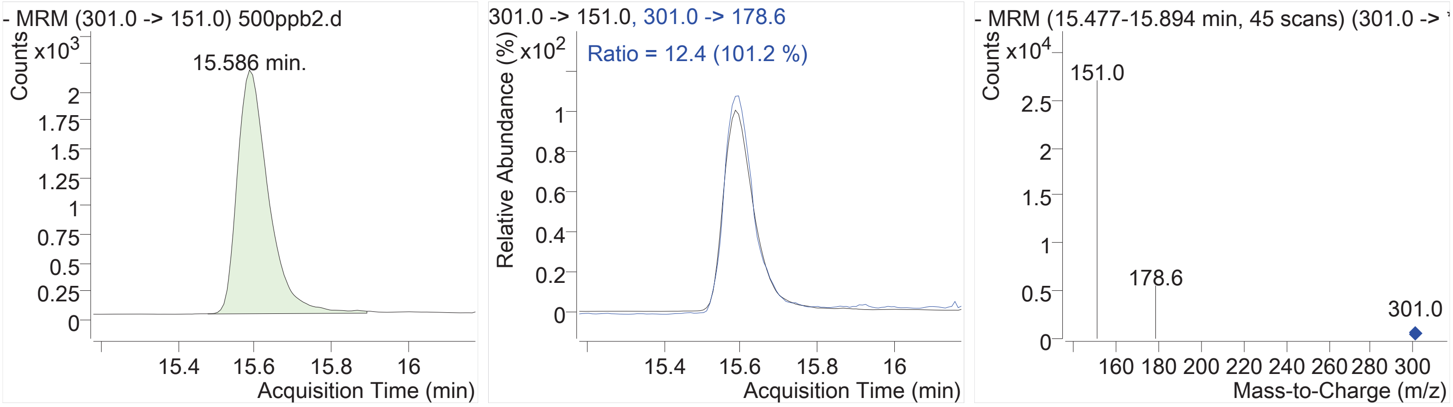

Luteolin

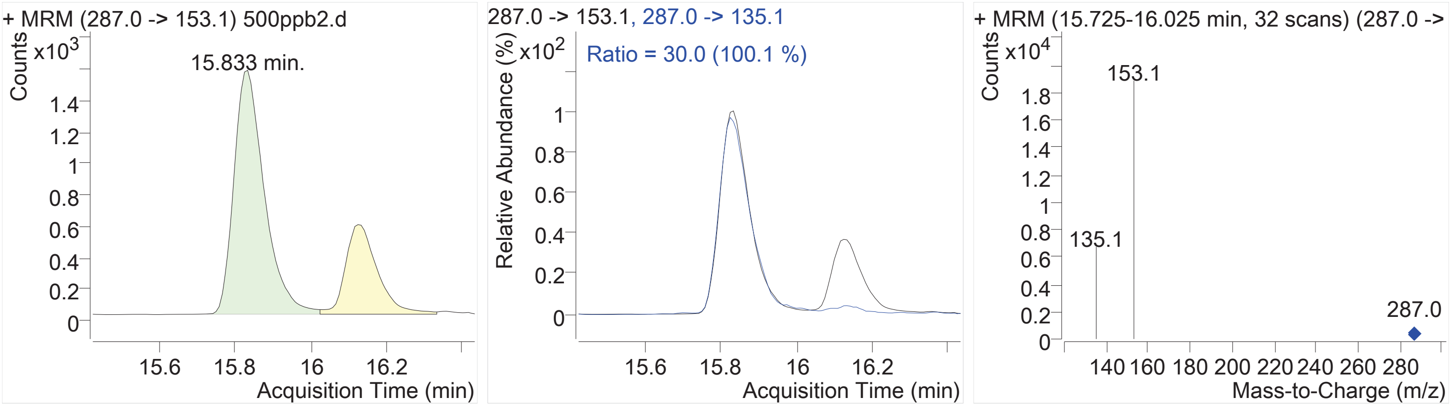

Kaempferol

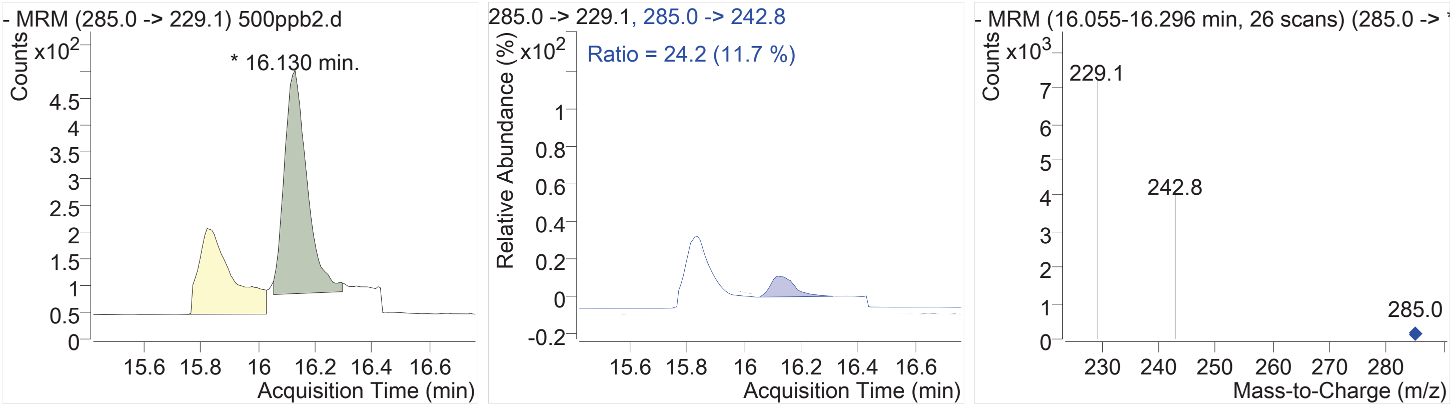

Apigenin

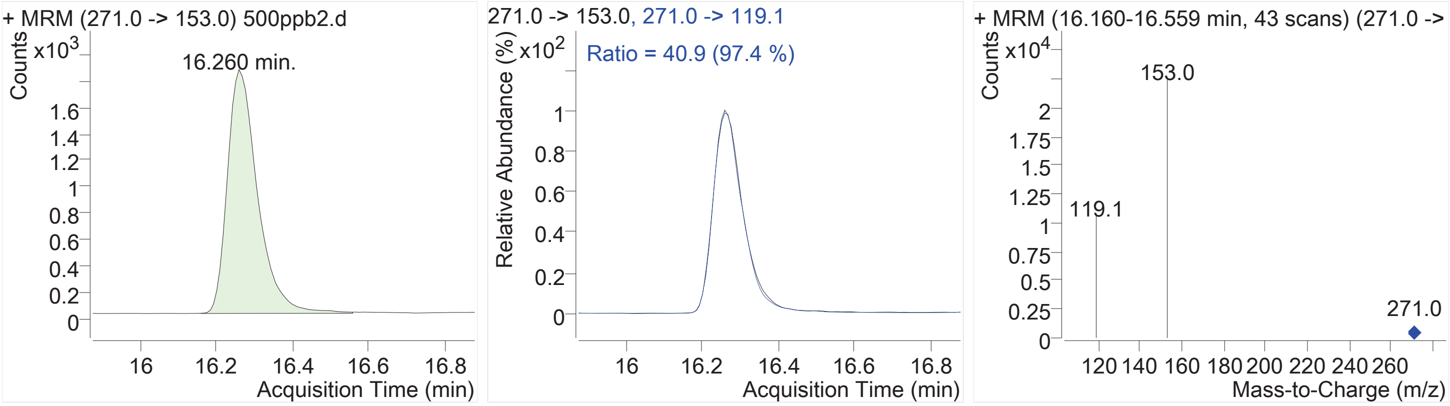

Quantitative Analysis Complete Report

Agilent Technologies

**Batch Path**	D:\MassHunter\Data\2022ekim\061022cengizhoca\QuantResults\071022.batch.bin		
**Analysis Time**	10/11/2022 1:33:26 PM	**Analyst Name**	Defam-PC\admin
**Report Time**	10/11/2022 1:34:32 PM	**Reporter Name**	admin
**Last Calib Update**	10/11/2022 1:33:17 PM	**Batch State**	Processed
**Quant Batch Version**	B.07.01	**Quant Report Version**	B.07.01

**Acq. Time**	10/6/2022 8:58:41 PM	**Data File**	500ppb3.d
**Sample Type**	Cal	**Sample Name**	500ppb3
**Dilution**	1	**Acq. Method**	FENOLIK_DMRM2021-31bilesen.m

Sample Chromatogram

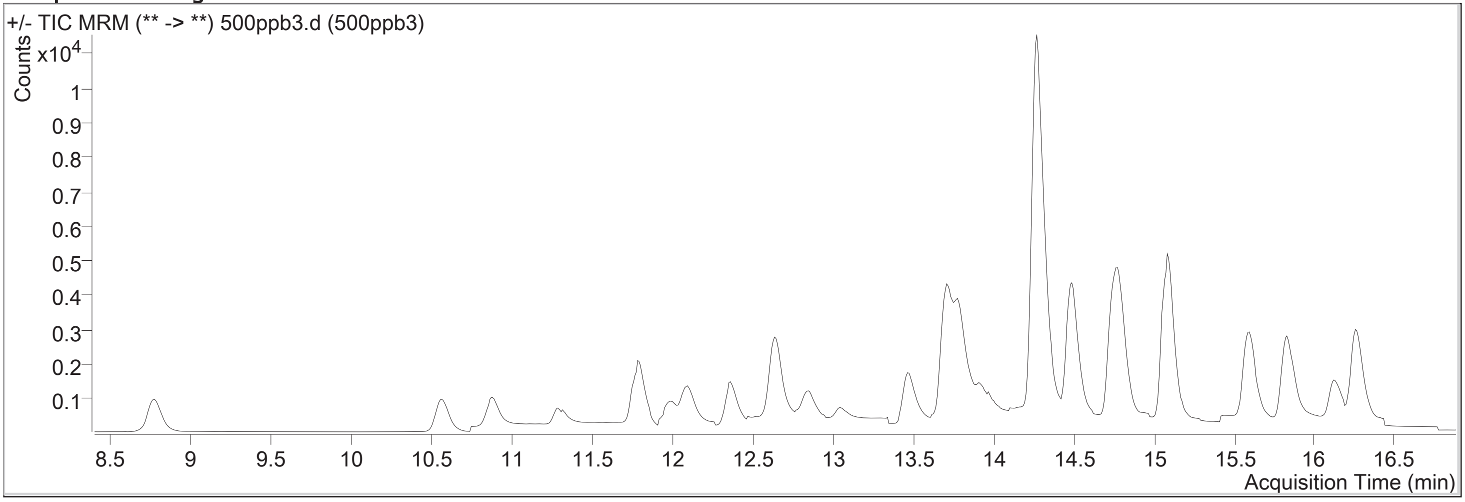

Compound	Transition	RT	Resp.	Final Conc	Units
Gallic acid	168.9 -> 125.0	8.775	5984	540.3413	ng/ml
Protocatechuic acid	152.9 -> 108.9	10.568	5523	522.5972	ng/ml
Pyrocatechol	109.0 -> 52.9	10.839	180	575.7839	ng/ml
3,4-Dihydroxyphenylacetic acid	167.0 -> 123.0	10.879	4208	522.1573	ng/ml
(+)-Catechin	289.0 -> 245.0	11.286	1635	474.1699	ng/ml
2,5-Dihydroxybenzoic acid	152.9 -> 109.0	11.988	3731	590.1980	ng/ml
Chlorogenic acid	355.0 -> 163.0	11.793	10663	506.2754	ng/ml
3-Hydroxybenzoic acid	137.0 -> 93.0	12.853	2698	519.6662	ng/ml
4-Hydroxybenzoic acid	136.9 -> 93.1	12.097	5837	528.5849	ng/ml
(-)-Epicatechin	291.0 -> 139.1	12.369	4210	533.5963	ng/ml
Caffeic acid	179.0 -> 135.0	12.641	13298	545.0329	ng/ml
Syringic acid	196.9 -> 181.9	12.765	387	543.2235	ng/ml
Vanillin	151.0 -> 136.0	13.053	1724	496.2029	ng/ml
Verbascoside	623.0 -> 160.8	13.467	5258	513.7557	ng/ml
Taxifolin	303.0 -> 285.1	13.712	15410	568.2097	ng/ml
p-Coumaric acid	162.9 -> 119.0	13.792	13718	526.1957	ng/ml
Sinapic acid	222.9 -> 207.9	13.864	1657	526.5985	ng/ml
Ferulic acid	193.0 -> 134.0	13.934	2768	531.3465	ng/ml
Luteolin 7-glucoside	447.1 -> 285.0	14.272	51994	537.5842	ng/ml
Hesperidin	611.1 -> 303.0	14.310	4334	530.1521	ng/ml
Hyperoside	465.1 -> 303.1	14.487	10630	541.9716	ng/ml
Rosmarinic acid	359.0 -> 160.9	14.489	6508	519.1533	ng/ml
Apigenin 7-glucoside	433.1 -> 271.0	14.755	18072	536.1615	ng/ml
Pinoresinol	357.0 -> 151.0	14.959	112	415.5208	ng/ml
2-Hydroxycinnamic acid	162.9 -> 119.1	14.794	10976	515.7341	ng/ml
Eriodictyol	287.0 -> 151.0	15.078	13840	530.4438	ng/ml
Quercetin	301.0 -> 151.0	15.594	12566	542.1020	ng/ml
Luteolin	287.0 -> 153.1	15.833	8647	541.3173	ng/ml
Kaempferol	285.0 -> 229.1	16.121	2193	514.3050	ng/ml

Quantitative Analysis Complete Report

Agilent Technologies

Compound	Transition	RT	Resp.	Final Conc	Units
Apigenin	271.0 -> 153.0	16.260	10133	542.6208	ng/ml

Gallic acid

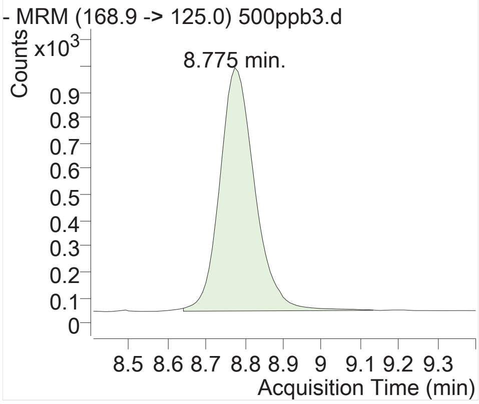

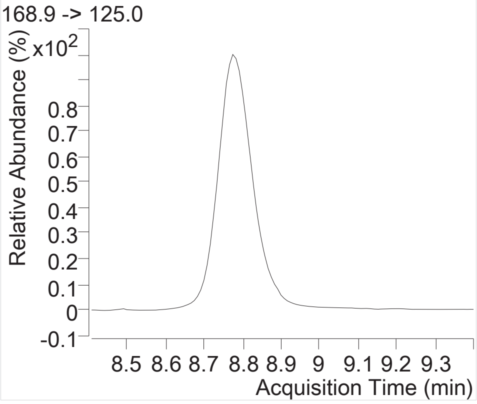

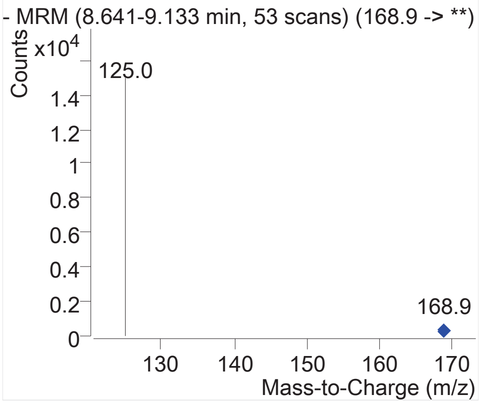

Protocatechuic acid

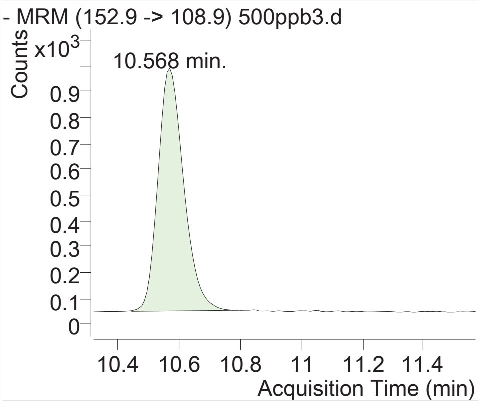

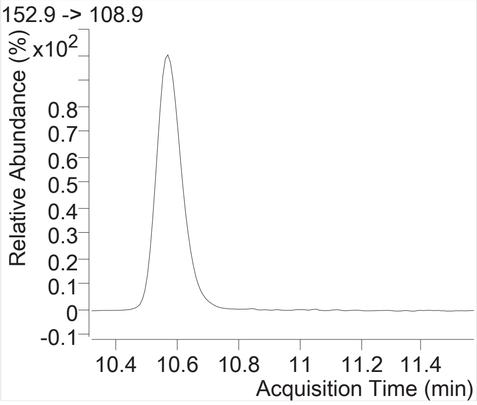

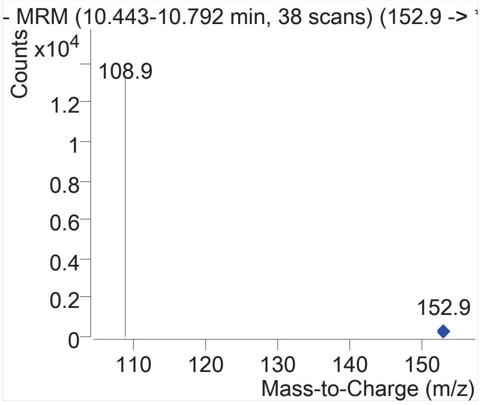

Pyrocatechol

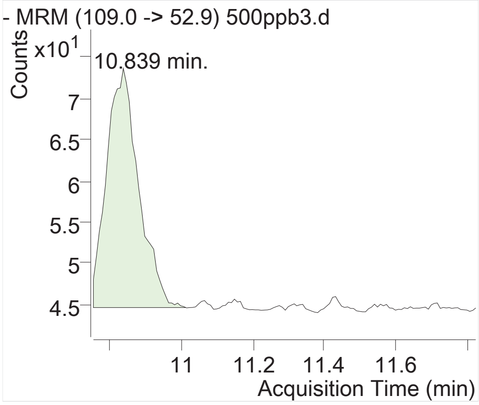

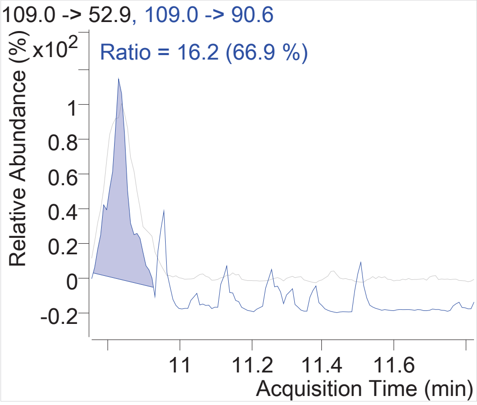

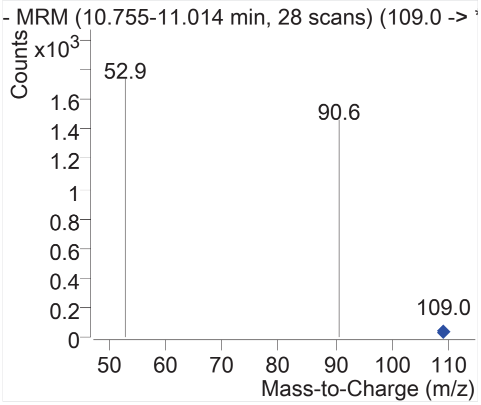

3,4-Dihydroxyphenylacetic acid

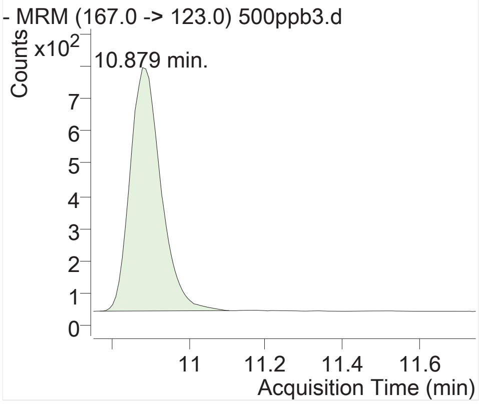

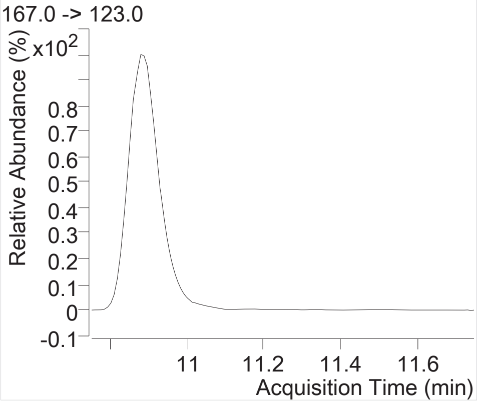

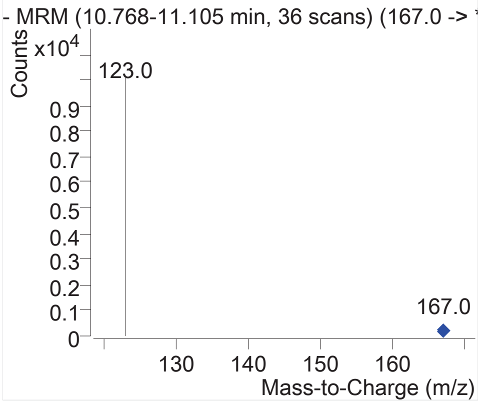

(+)-Catechin

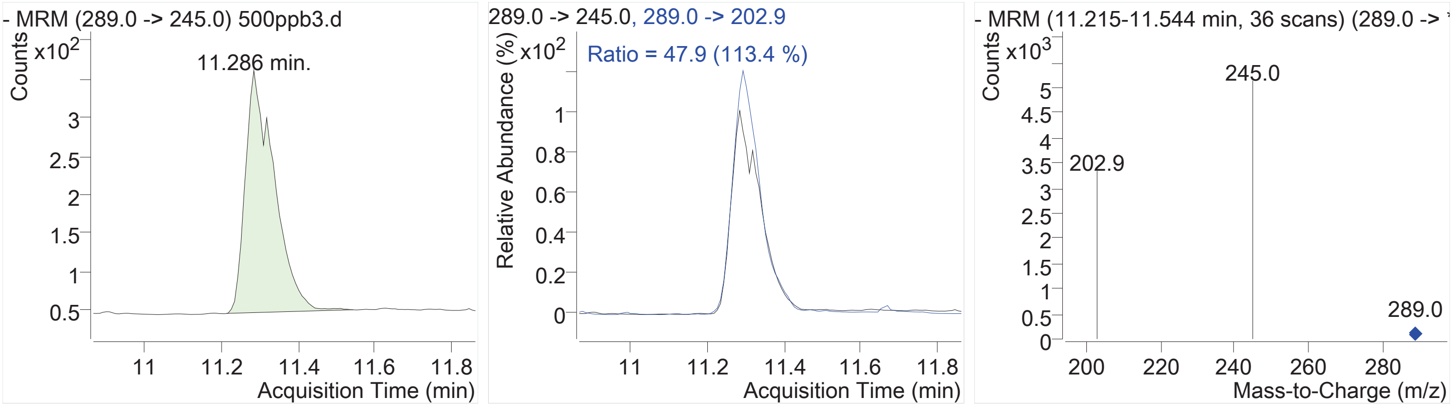

2,5-Dihydroxybenzoic acid

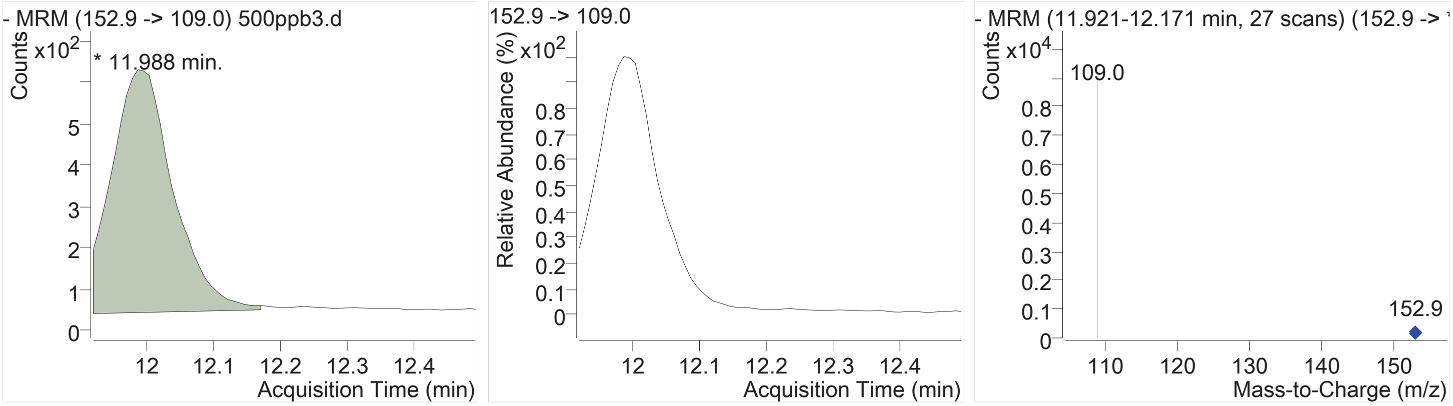

Chlorogenic acid

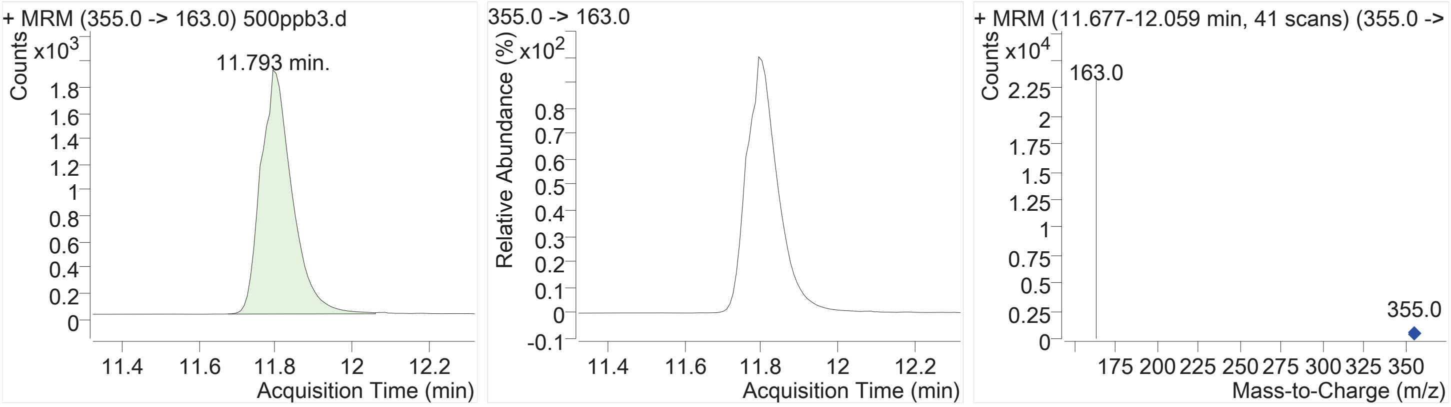

3-Hydroxybenzoic acid

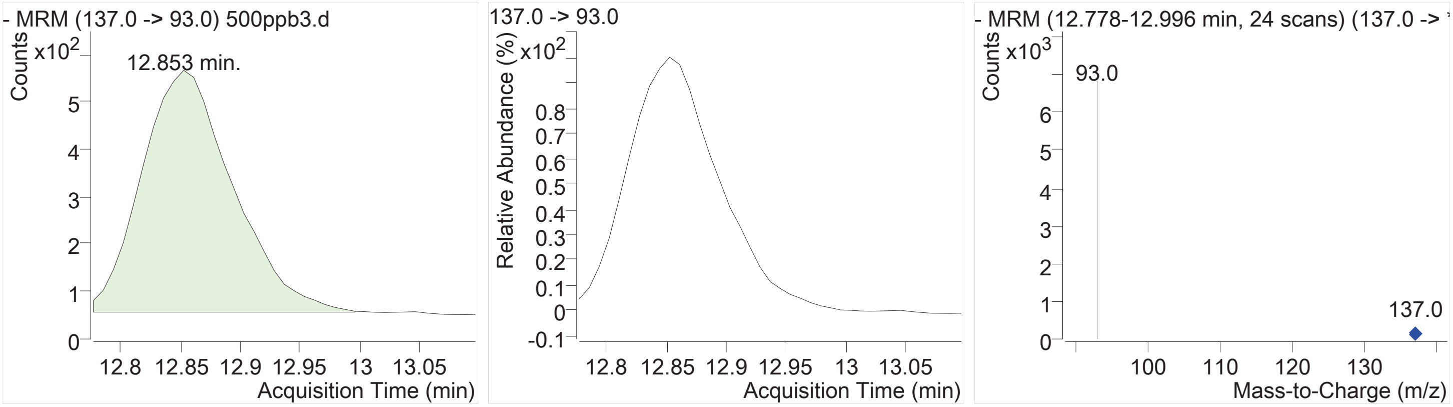

4-Hydroxybenzoic acid

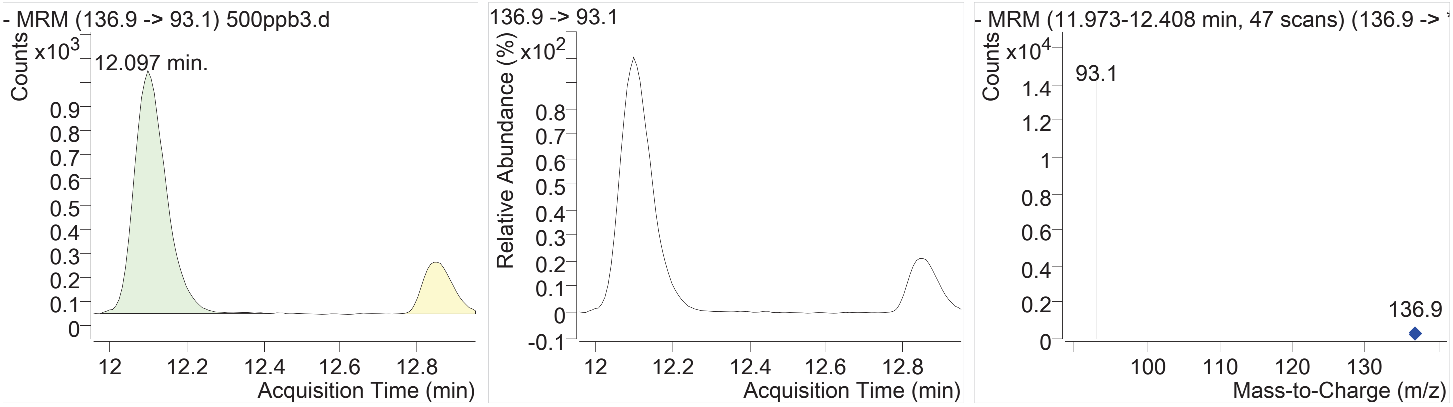

(-)-Epicatechin

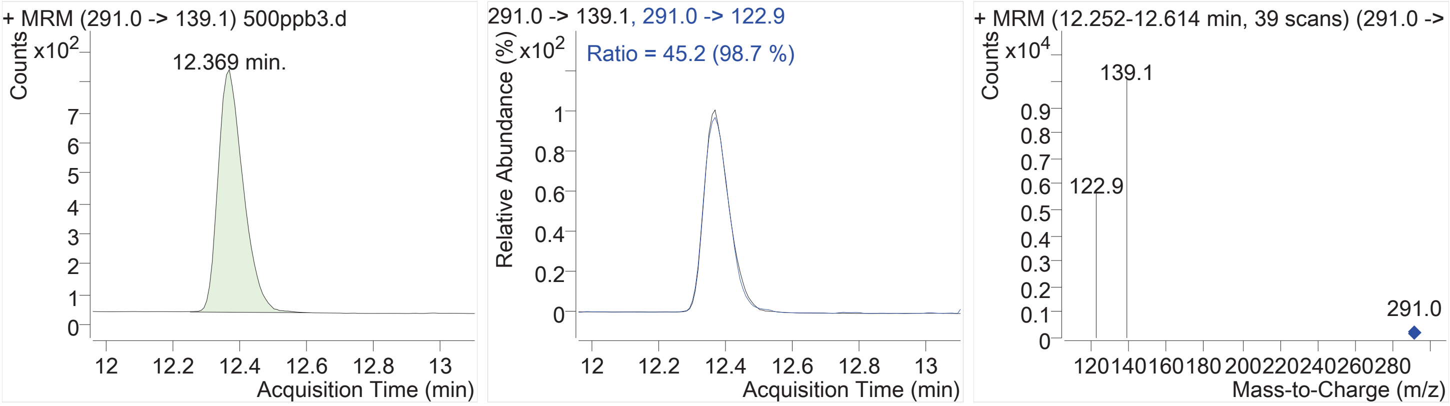

Caffeic acid

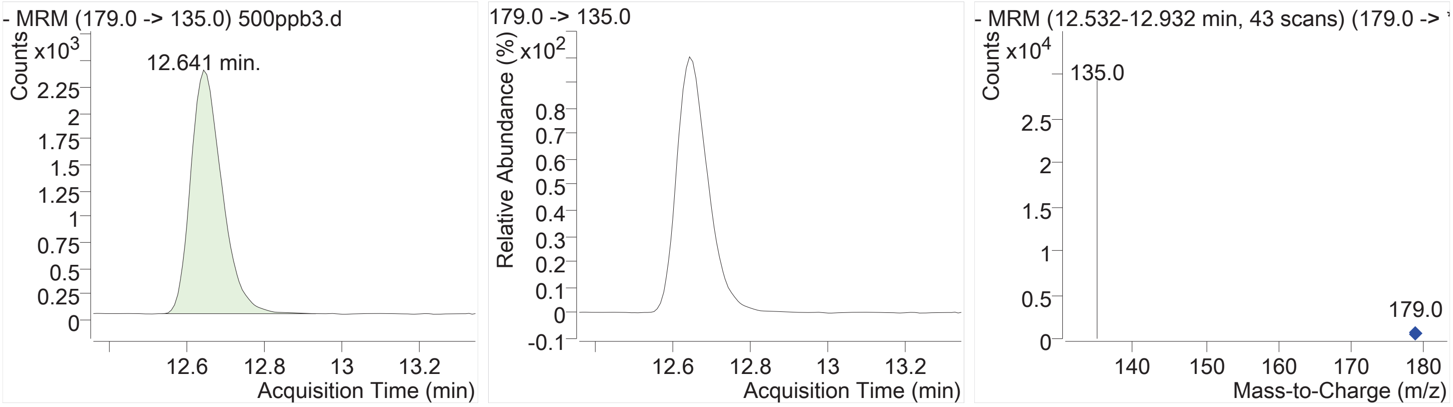

Syringic acid

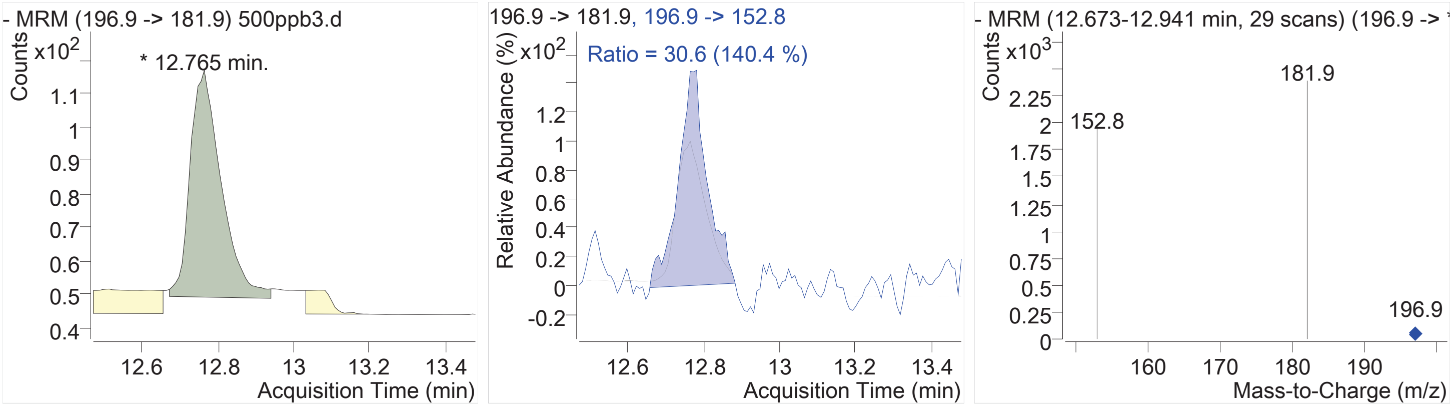

Vanillin

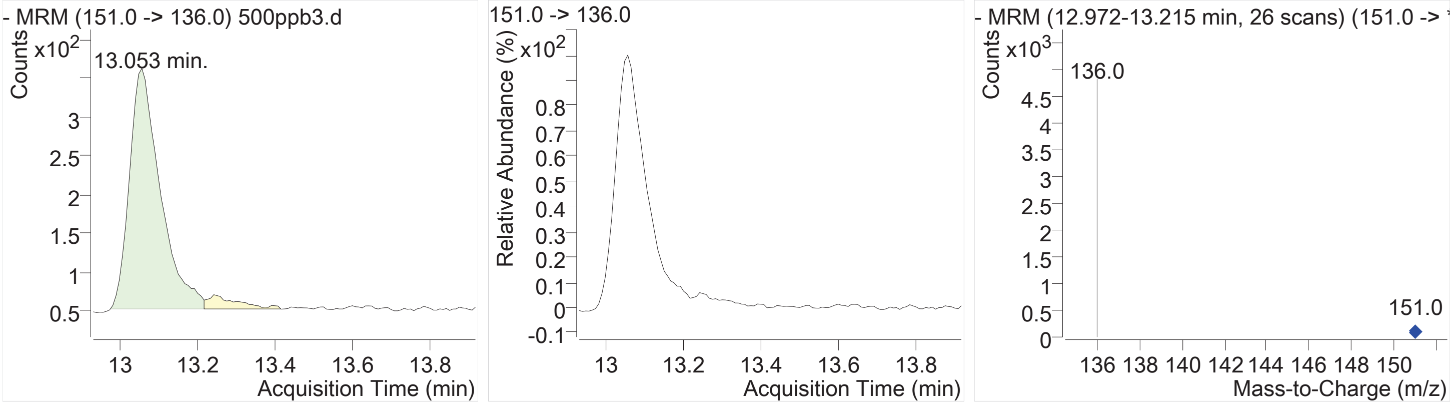

Verbascoside

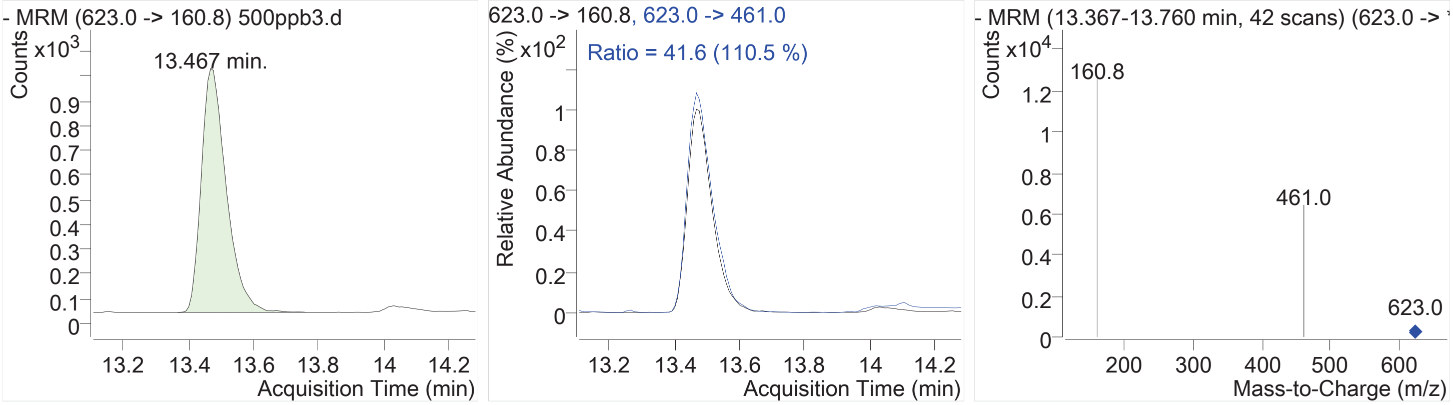

Taxifolin

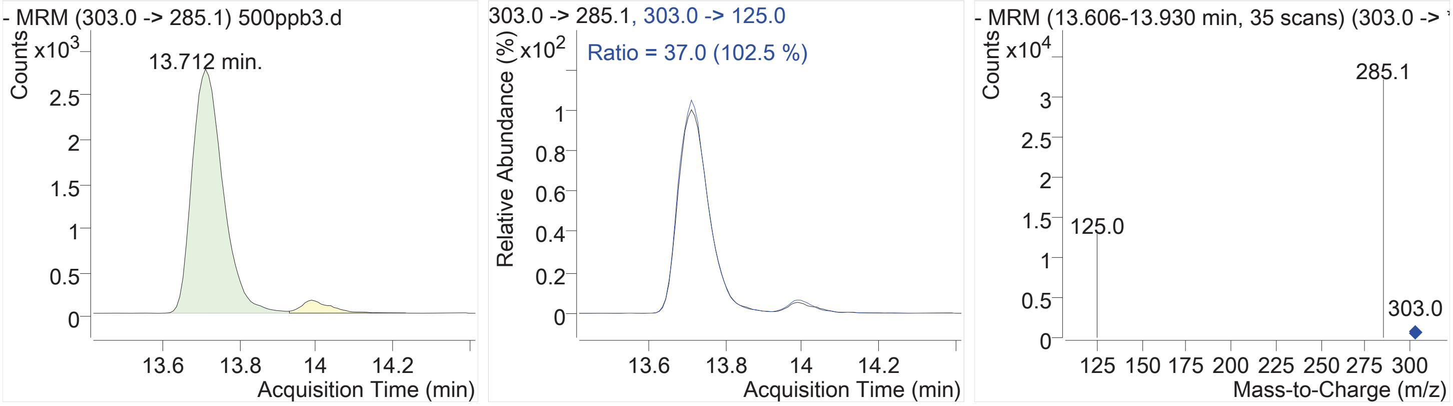

p-Coumaric acid

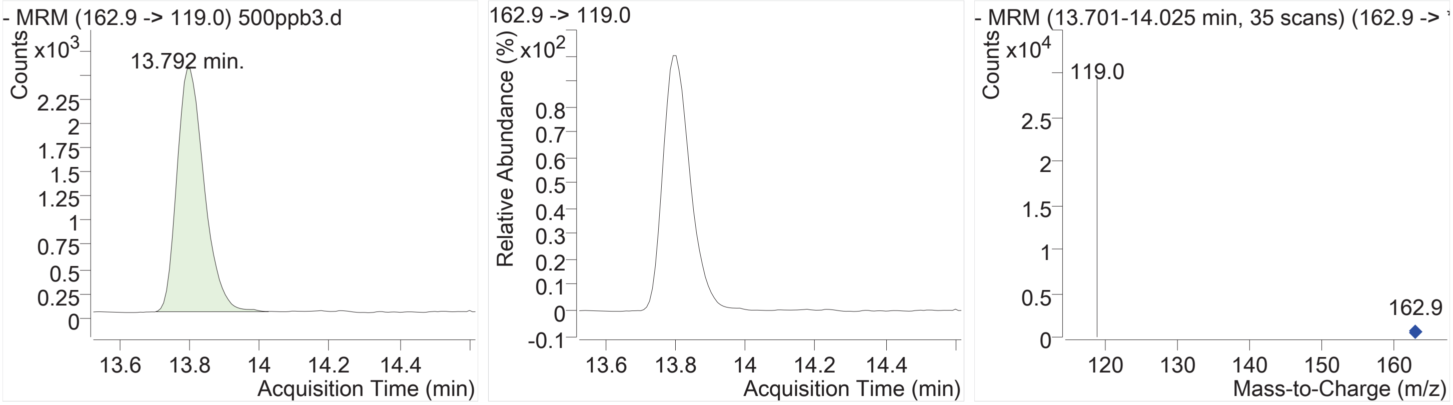

Sinapic acid

- MRM (222.9 -> 207.9) 500ppb3.d

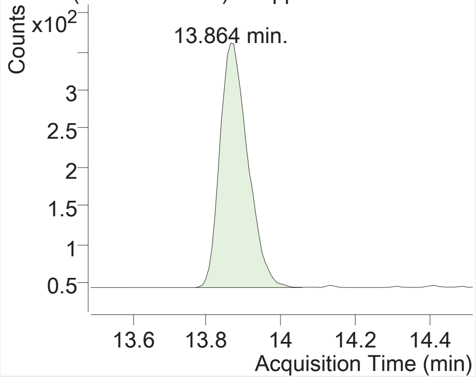

222.9 -> 207.9, 222.9 -> 163.8

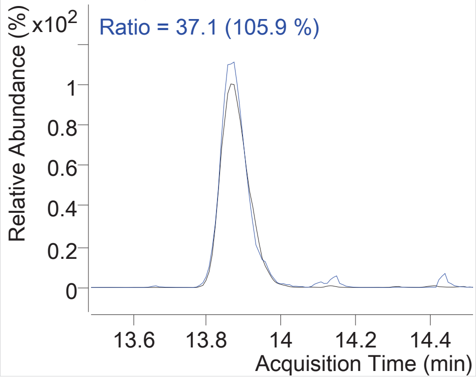

- MRM (13.768-14.056 min, 31 scans) (222.9 ->

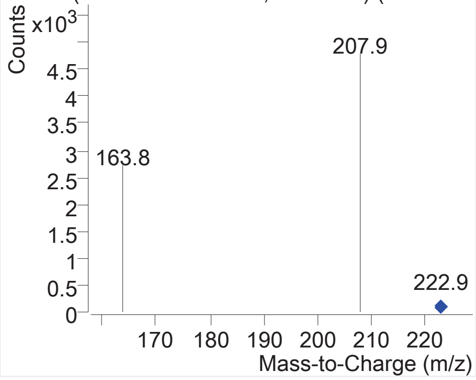

Ferulic acid

- MRM (193.0 -> 134.0) 500ppb3.d

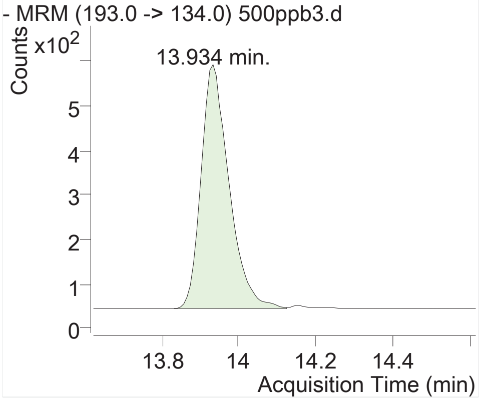

193.0 -> 134.0, 193.0 -> 177.8

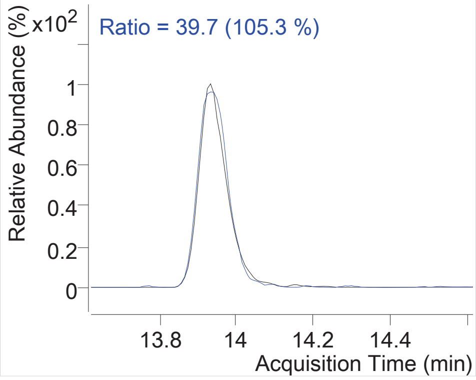

- MRM (13.833-14.125 min, 32 scans) (193.0 ->

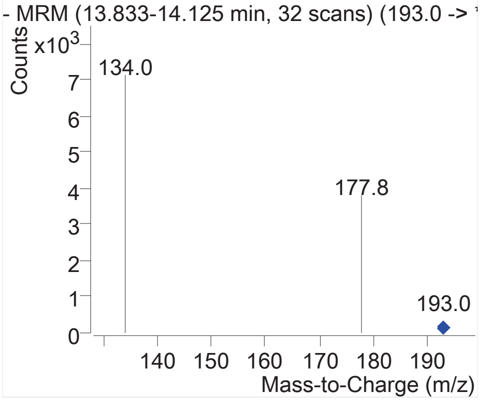

Luteolin 7-glucoside

- MRM (447.1 -> 285.0) 500ppb3.d

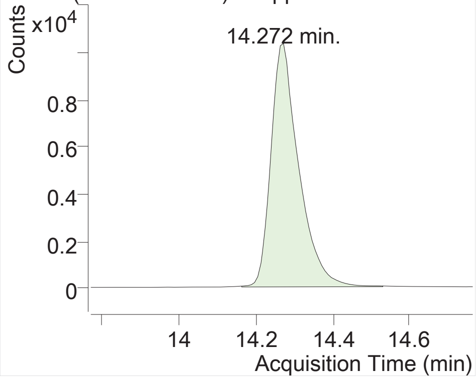

447.1 -> 285.0

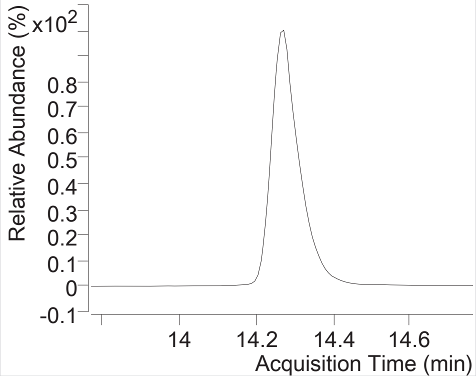

- MRM (14.163-14.531 min, 40 scans) (447.1 ->

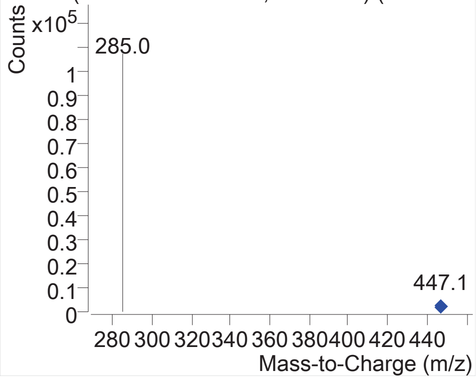

Hesperidin

+ MRM (611.1 -> 303.0) 500ppb3.d

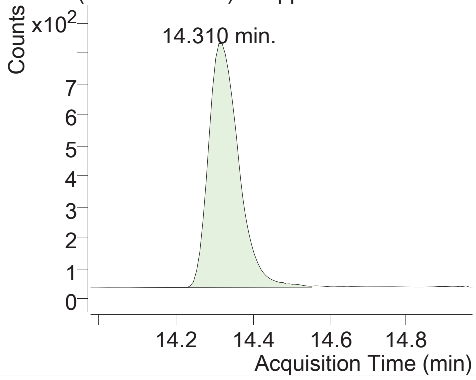

611.1 -> 303.0, 611.1 -> 449.2

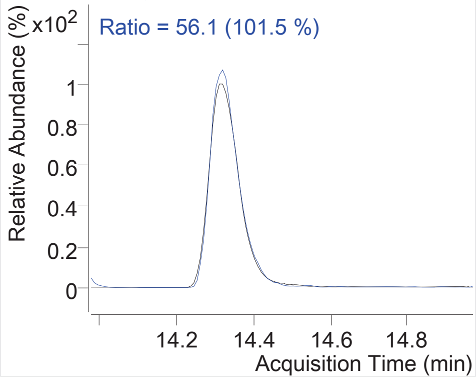

+ MRM (14.227-14.553 min, 35 scans) (611.1 ->

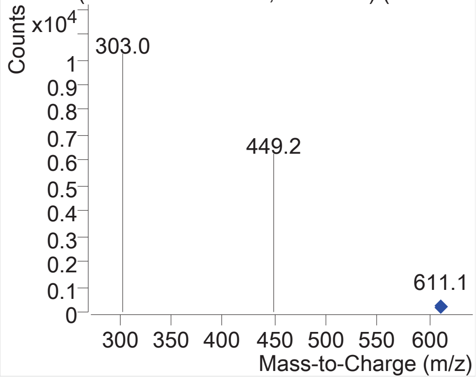

Hyperoside

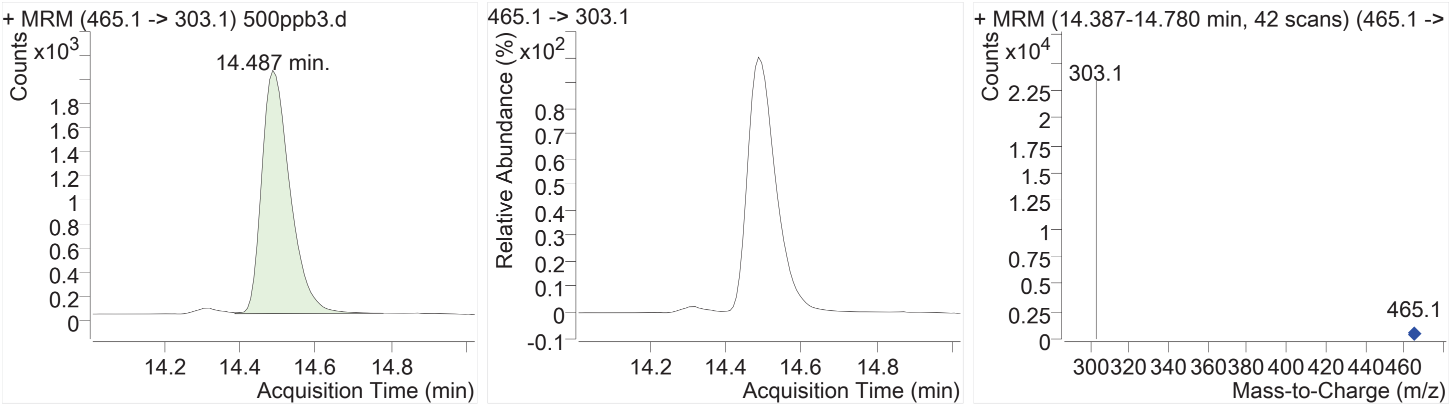

Rosmarinic acid

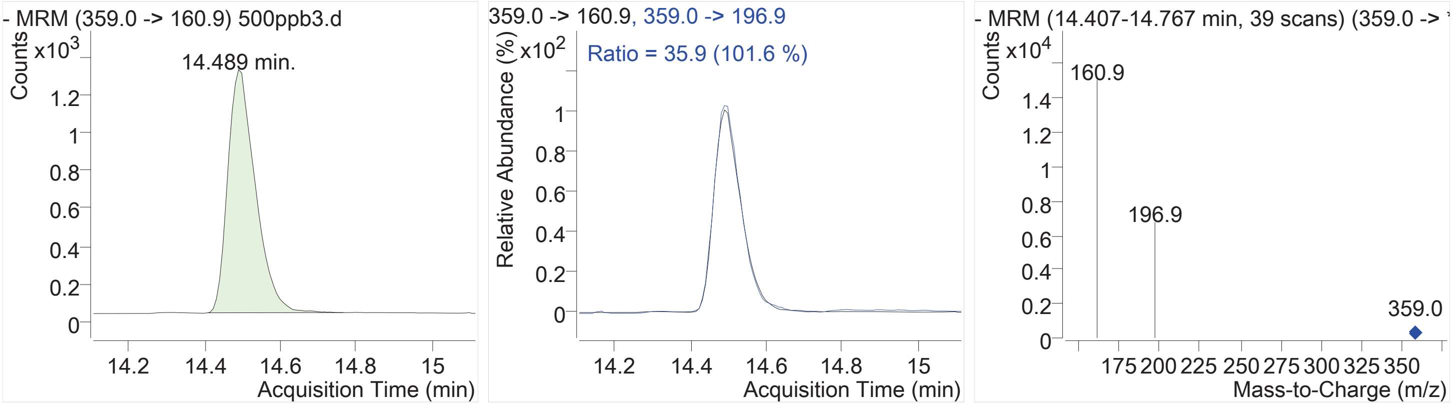

Apigenin 7-glucoside

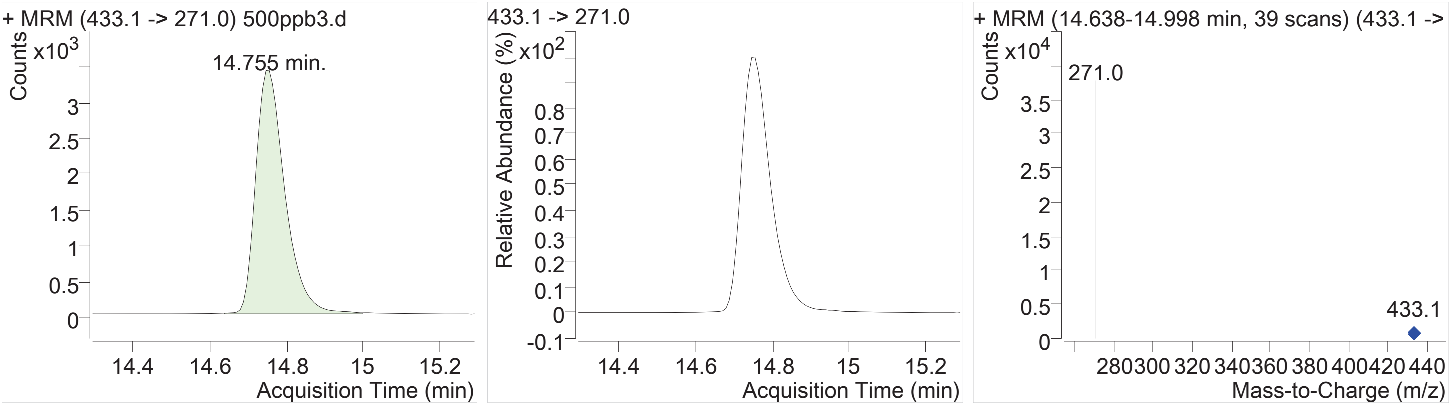

Pinoresinol

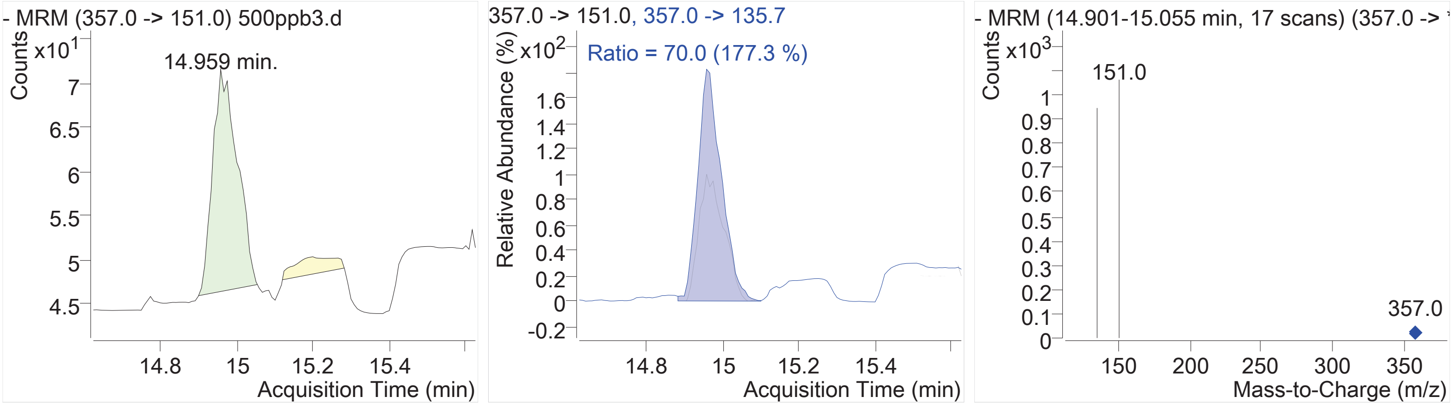

2-Hydroxycinnamic acid

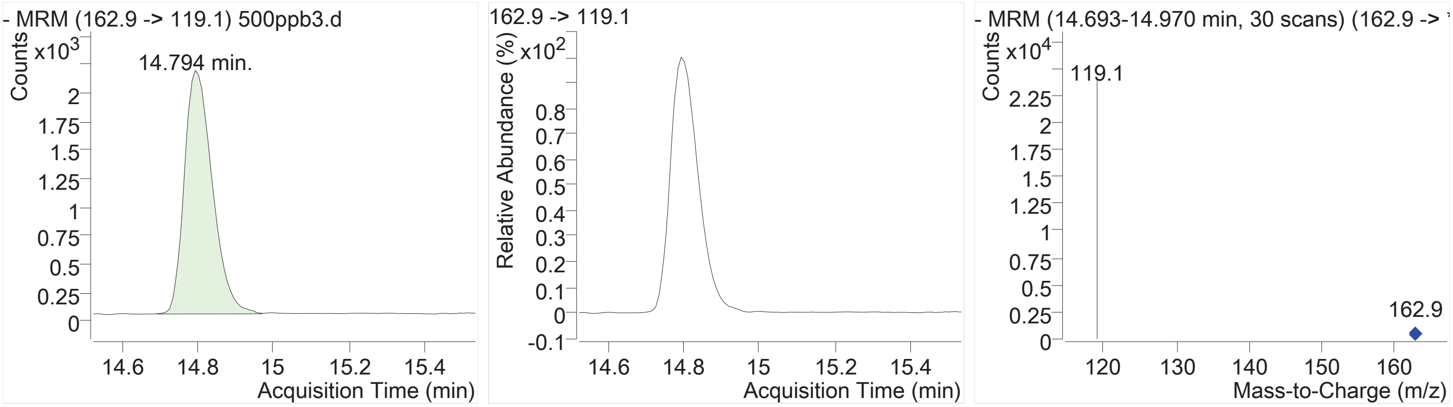

Eriodictyol

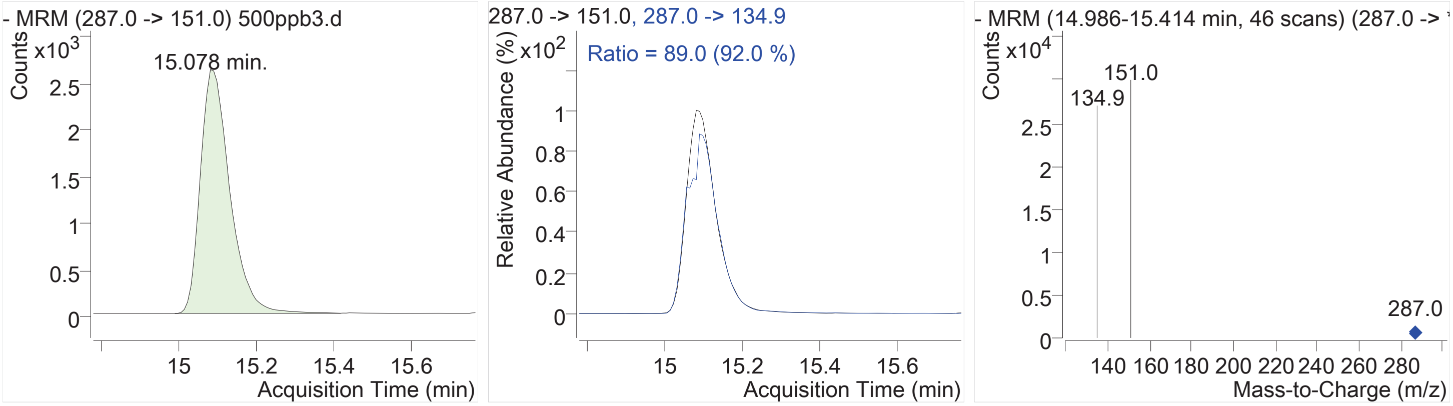

Quercetin

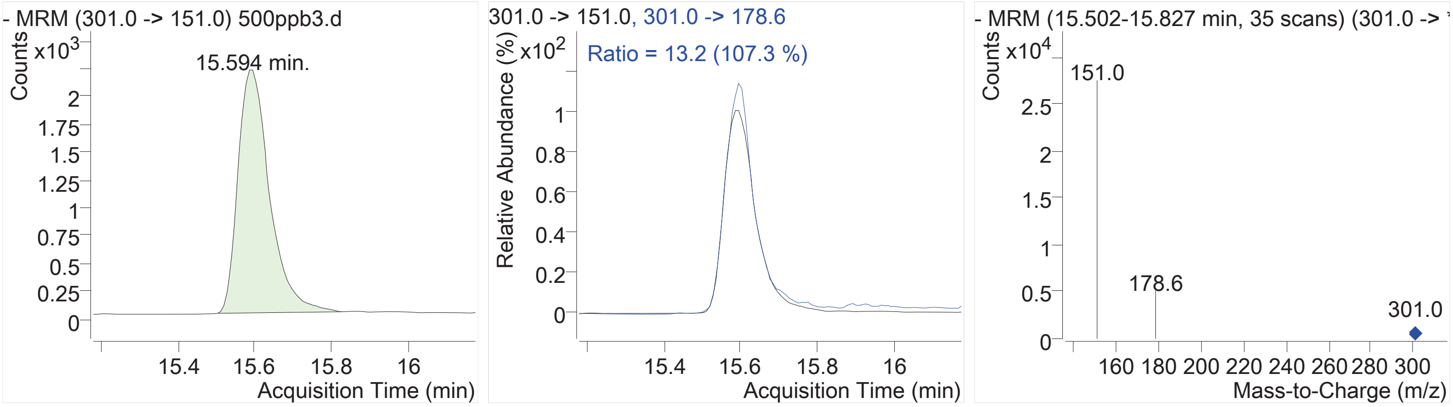

Luteolin

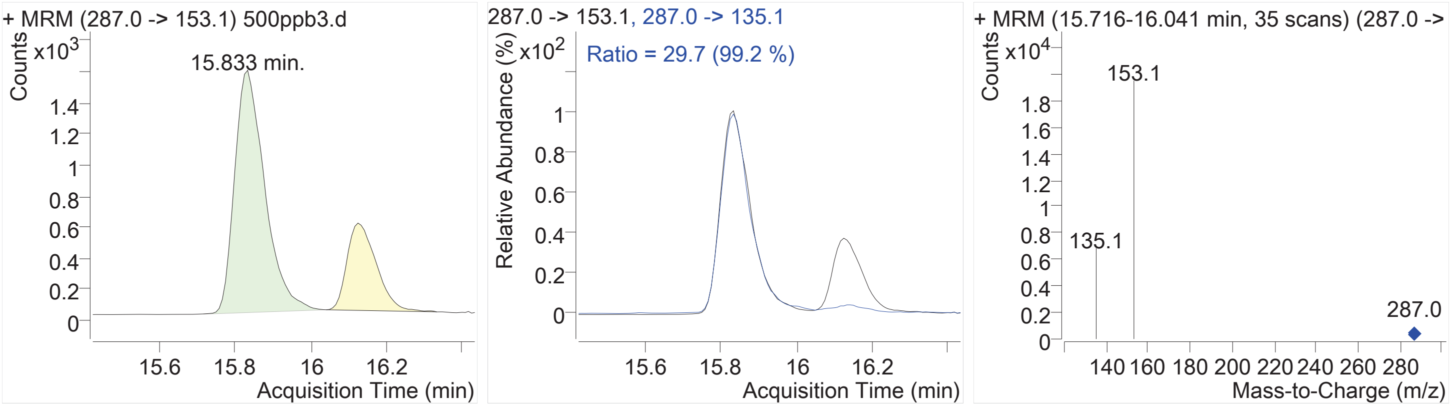

Kaempferol

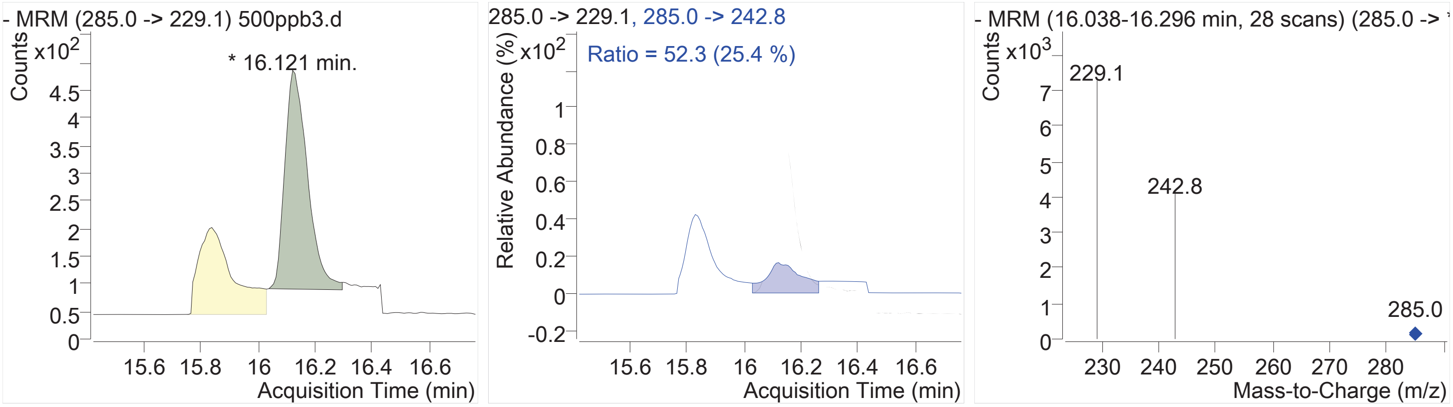

Apigenin

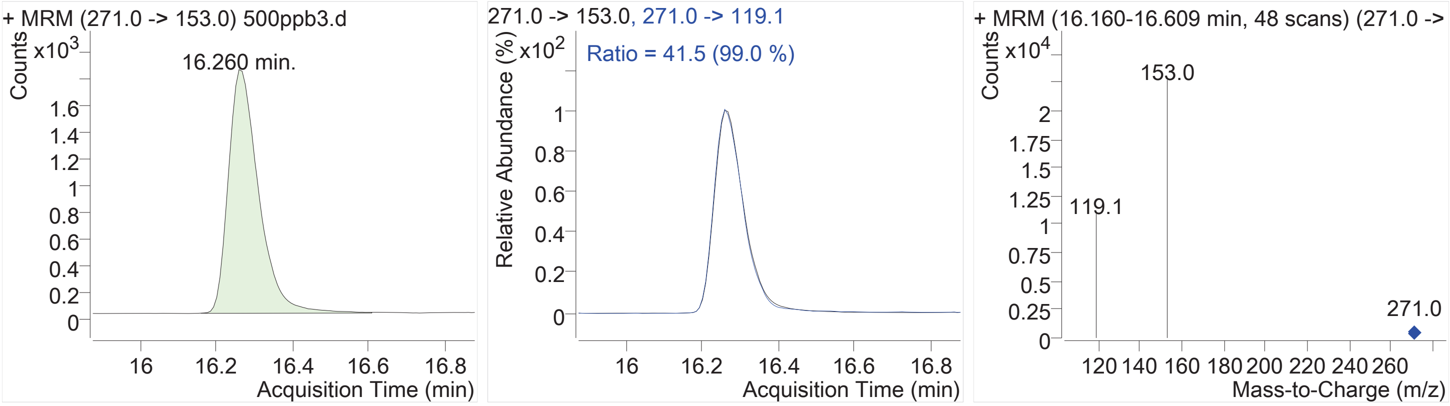

Quantitative Analysis Complete Report

**Batch Path**	D:\MassHunter\Data\2022ekim\061022cengizhoca\QuantResults\071022.batch.bin		
**Analysis Time**	10/11/2022 1:33:26 PM	**Analyst Name**	Defam-PC\admin
**Report Time**	10/11/2022 1:34:34 PM	**Reporter Name**	admin
**Last Calib Update**	10/11/2022 1:33:17 PM	**Batch State**	Processed
**Quant Batch Version**	B.07.01	**Quant Report Version**	B.07.01

**Acq. Time**	10/6/2022 9:20:23 PM	**Data File**	1000ppb1.d
**Sample Type**	Cal	**Sample Name**	1000ppb1
**Dilution**	1	**Acq. Method**	FENOLIK_DMRM2021-31bilesen.m

Sample Chromatogram

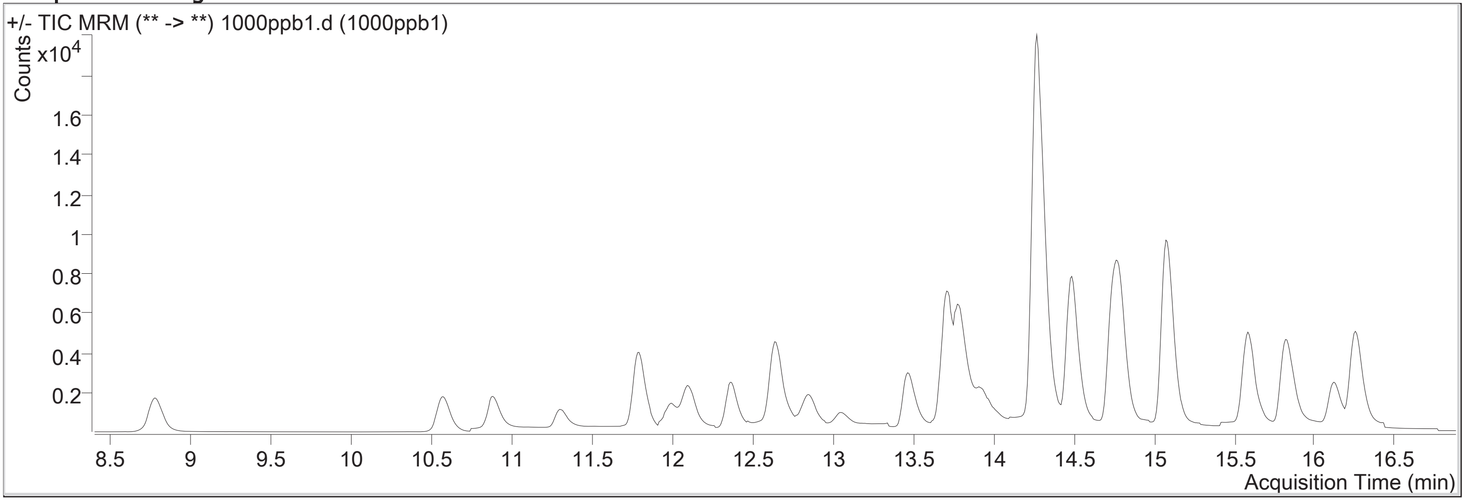

Compound	Transition	RT	Resp.	Final Conc	Units
Gallic acid	168.9 -> 125.0	8.783	11032	1014.7633	ng/ml
Protocatechuic acid	152.9 -> 108.9	10.576	10639	1017.6295	ng/ml
Pyrocatechol	109.0 -> 52.9	10.831	315	1020.7597	ng/ml
3,4-Dihydroxyphenylacetic acid	167.0 -> 123.0	10.888	8499	1063.7717	ng/ml
(+)-Catechin	289.0 -> 245.0	11.302	3351	1003.7100	ng/ml
2,5-Dihydroxybenzoic acid	152.9 -> 109.0	11.996	6657	1052.4536	ng/ml
Chlorogenic acid	355.0 -> 163.0	11.802	21670	1022.9514	ng/ml
3-Hydroxybenzoic acid	137.0 -> 93.0	12.861	5429	1030.3545	ng/ml
4-Hydroxybenzoic acid	136.9 -> 93.1	12.097	11222	1032.0475	ng/ml
(-)-Epicatechin	291.0 -> 139.1	12.369	8001	1011.3057	ng/ml
Caffeic acid	179.0 -> 135.0	12.641	24200	1015.6110	ng/ml
Syringic acid	196.9 -> 181.9	12.773	734	1026.8009	ng/ml
Vanillin	151.0 -> 136.0	13.053	3457	1016.2171	ng/ml
Verbascoside	623.0 -> 160.8	13.467	10297	1019.1654	ng/ml
Taxifolin	303.0 -> 285.1	13.712	25309	963.2799	ng/ml
p-Coumaric acid	162.9 -> 119.0	13.801	25973	1004.6535	ng/ml
Sinapic acid	222.9 -> 207.9	13.873	3258	1031.5171	ng/ml
Ferulic acid	193.0 -> 134.0	13.934	5358	1028.3674	ng/ml
Luteolin 7-glucoside	447.1 -> 285.0	14.272	94912	1009.1075	ng/ml
Hesperidin	611.1 -> 303.0	14.319	8206	1000.0435	ng/ml
Hyperoside	465.1 -> 303.1	14.487	20105	1021.5063	ng/ml
Rosmarinic acid	359.0 -> 160.9	14.489	12837	1025.2666	ng/ml
Apigenin 7-glucoside	433.1 -> 271.0	14.747	33663	999.8474	ng/ml
Pinoresinol	357.0 -> 151.0	14.950	263	1011.2129	ng/ml
2-Hydroxycinnamic acid	162.9 -> 119.1	14.794	21790	1025.6140	ng/ml
Eriodictyol	287.0 -> 151.0	15.078	25696	1001.3855	ng/ml
Quercetin	301.0 -> 151.0	15.586	23388	1015.1219	ng/ml
Luteolin	287.0 -> 153.1	15.825	15722	988.3047	ng/ml
Kaempferol	285.0 -> 229.1	16.121	3962	955.9408	ng/ml

Compound	Transition	RT	Resp.	Final Conc	Units
Apigenin	271.0 -> 153.0	16.260	18564	999.0581	ng/ml

Gallic acid

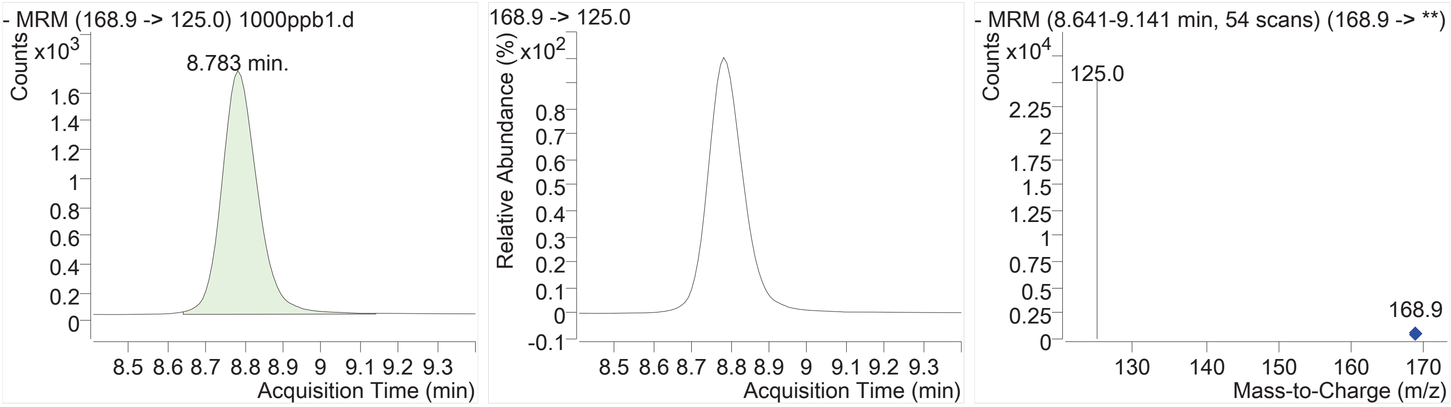

Protocatechuic acid

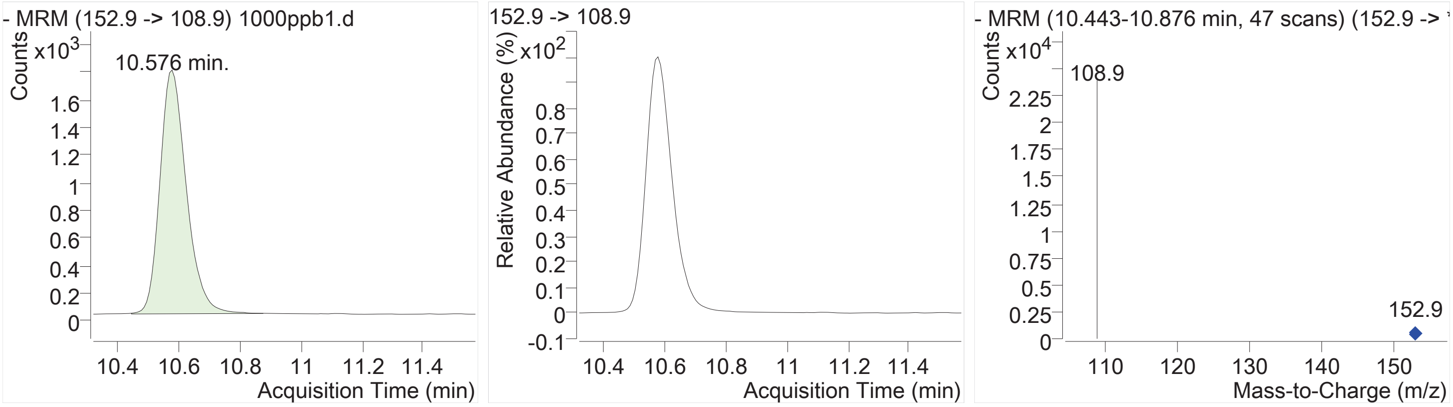

Pyrocatechol

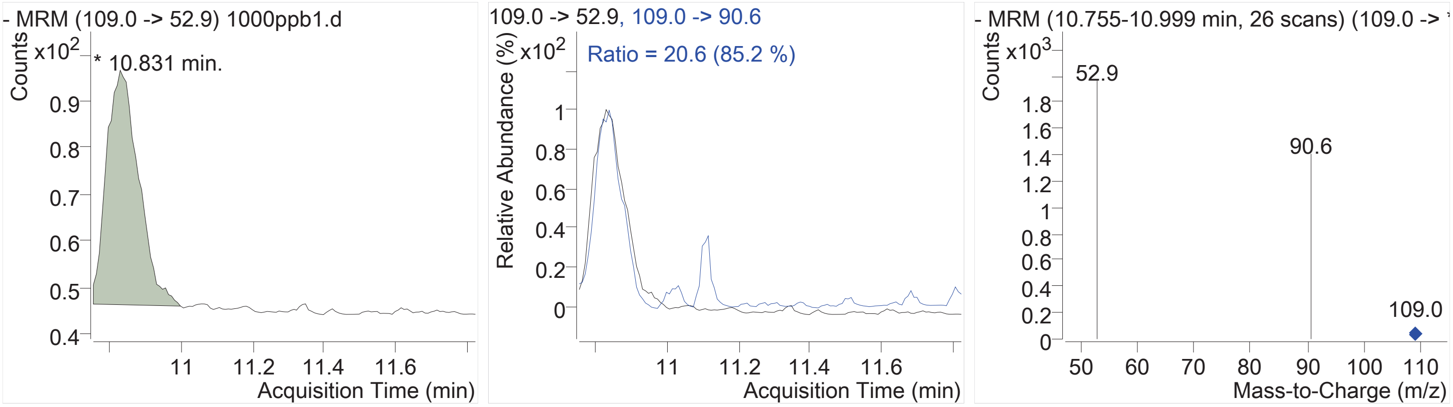

3,4-Dihydroxyphenylacetic acid

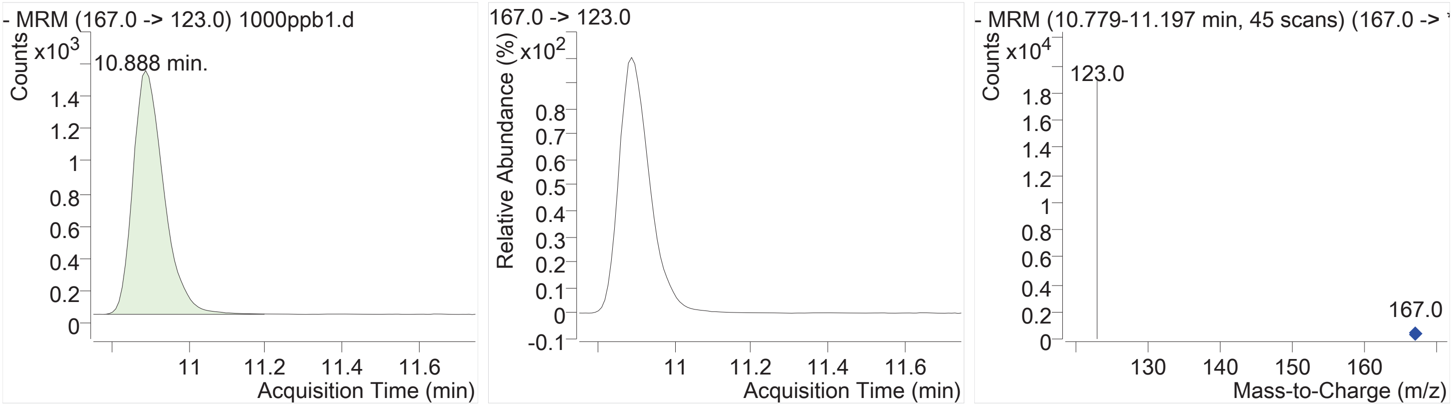

Agilent Technologies

(+)-Catechin

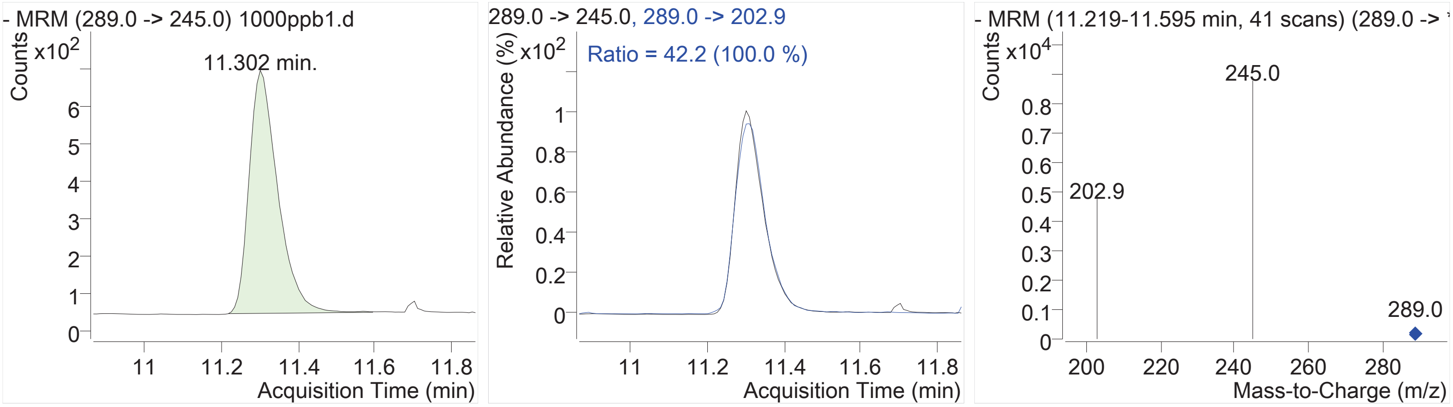

2,5-Dihydroxybenzoic acid

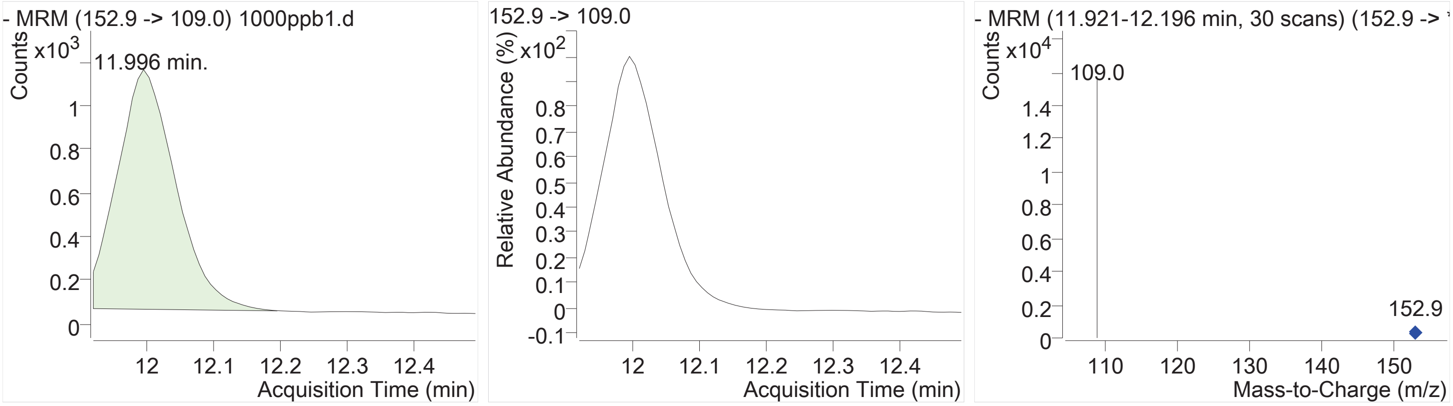

Chlorogenic acid

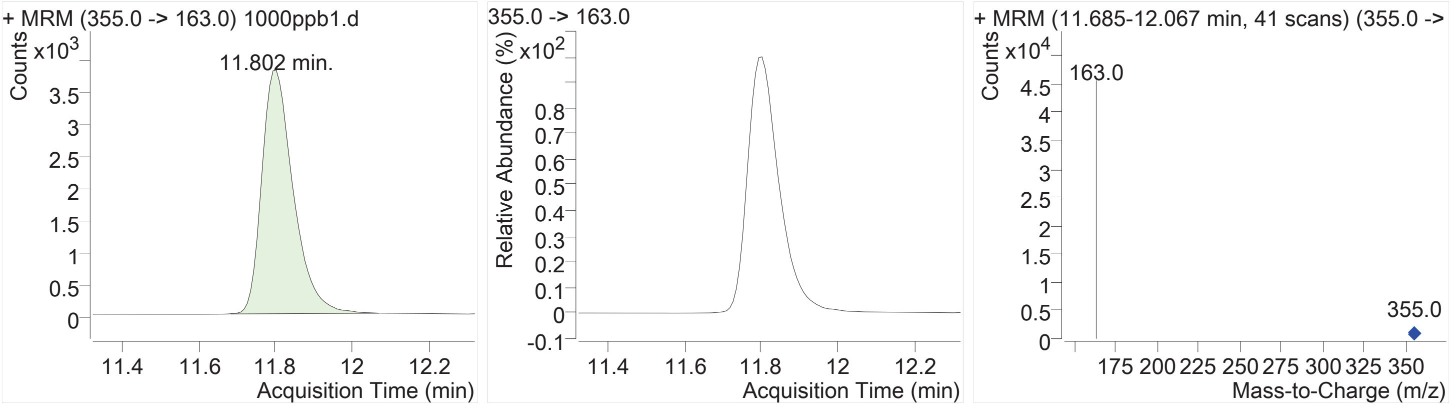

3-Hydroxybenzoic acid

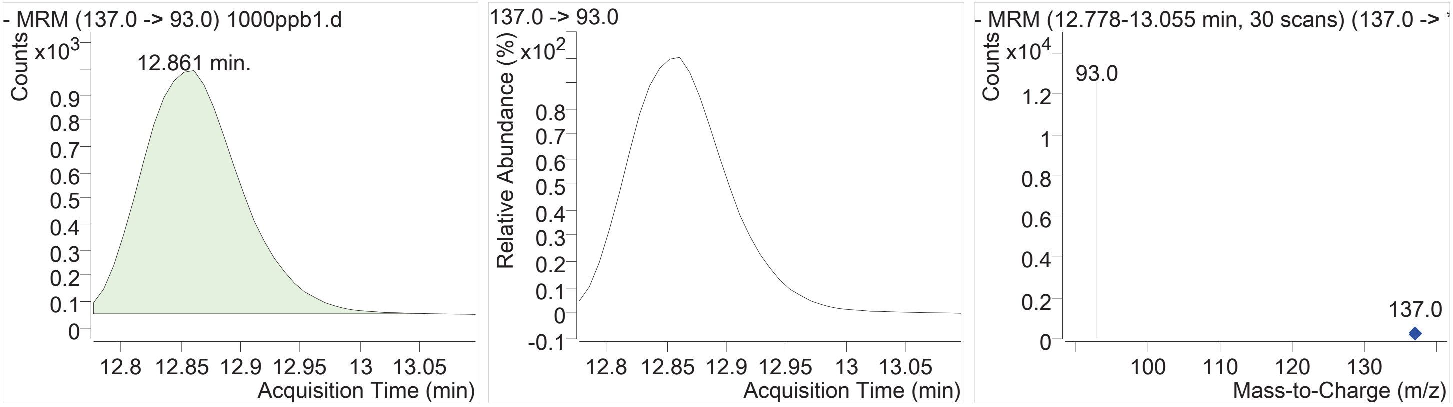

4-Hydroxybenzoic acid

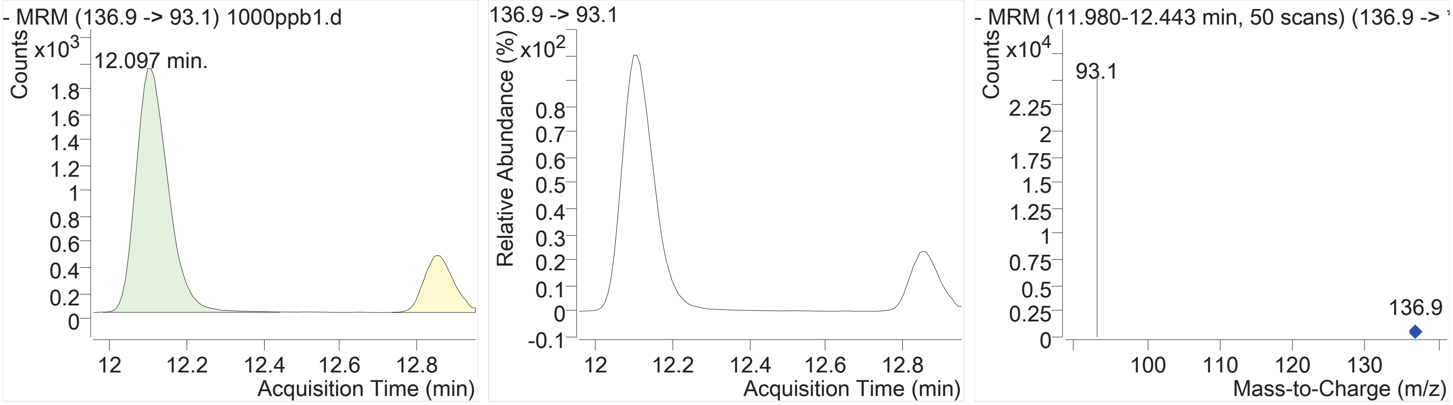

(-)-Epicatechin

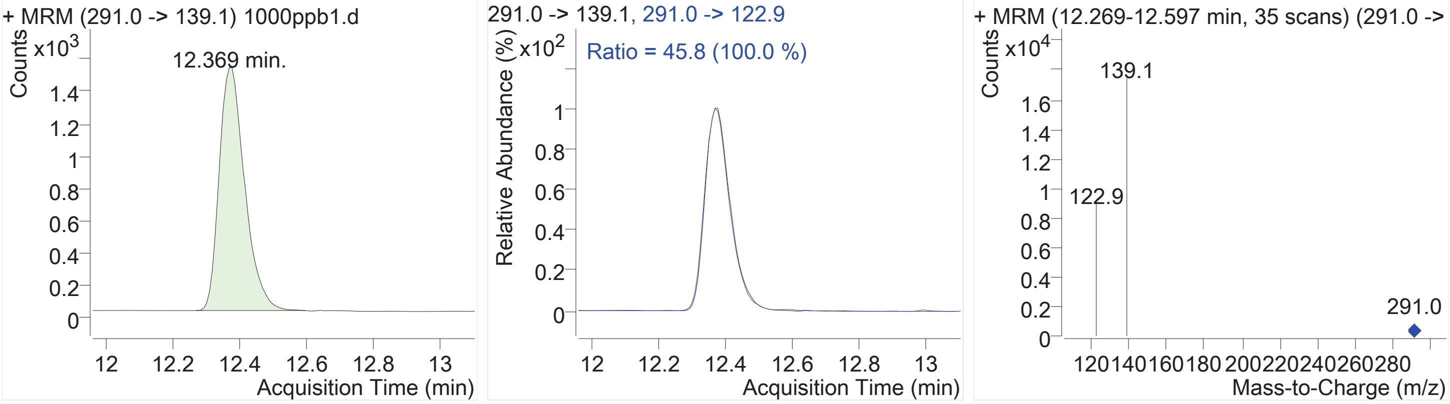

Caffeic acid

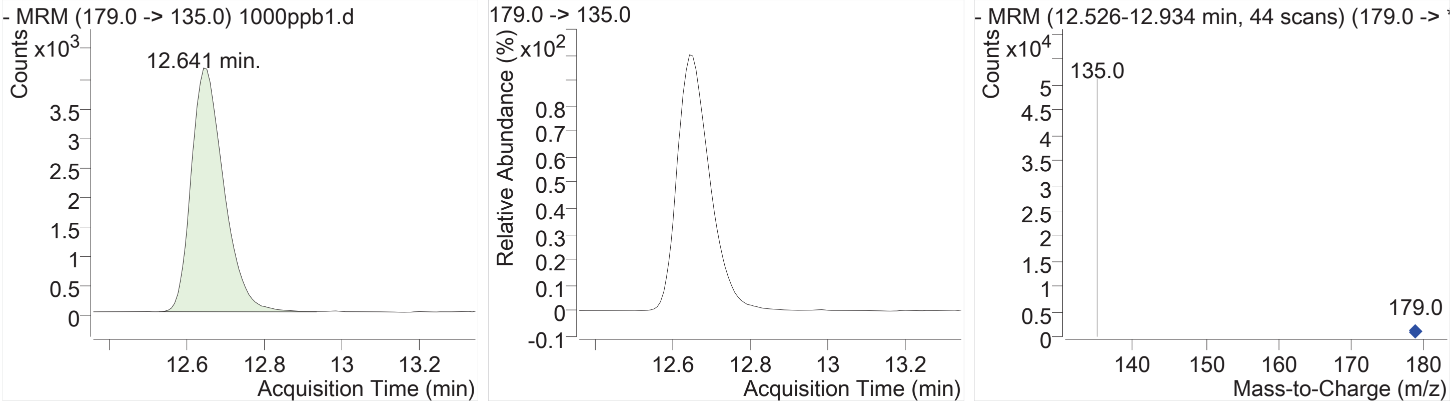

Syringic acid

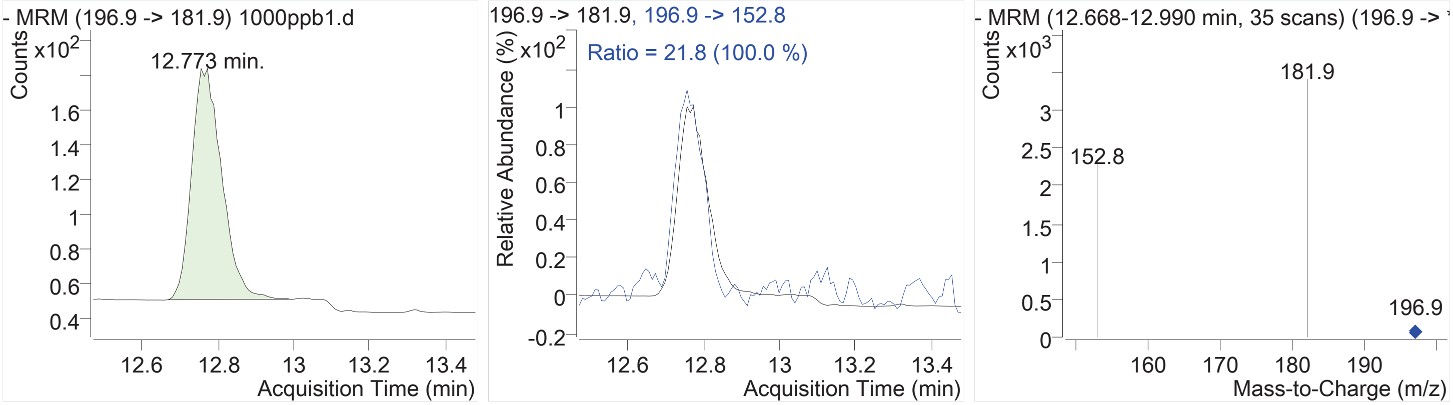

Vanillin

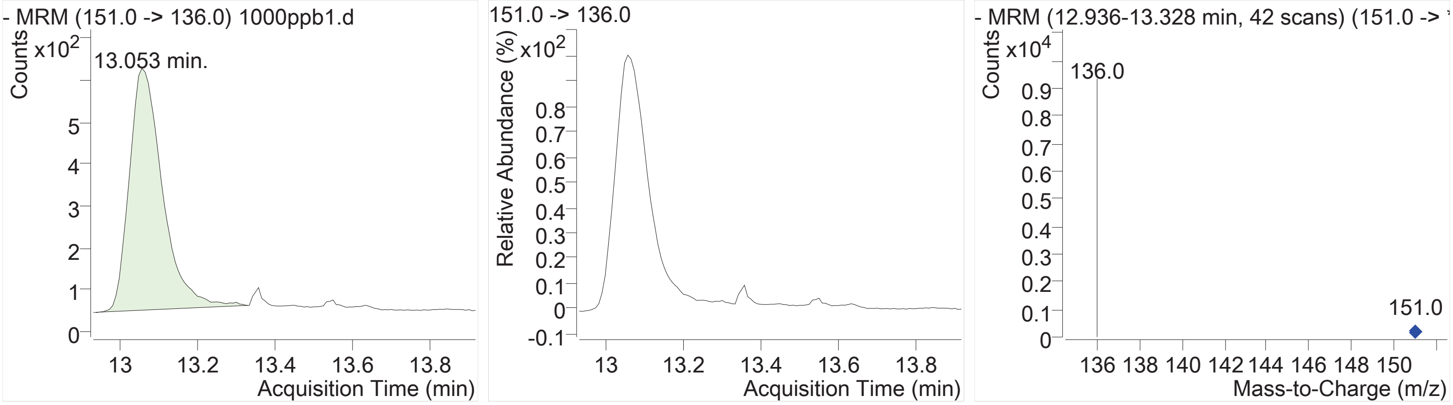

Verbascoside

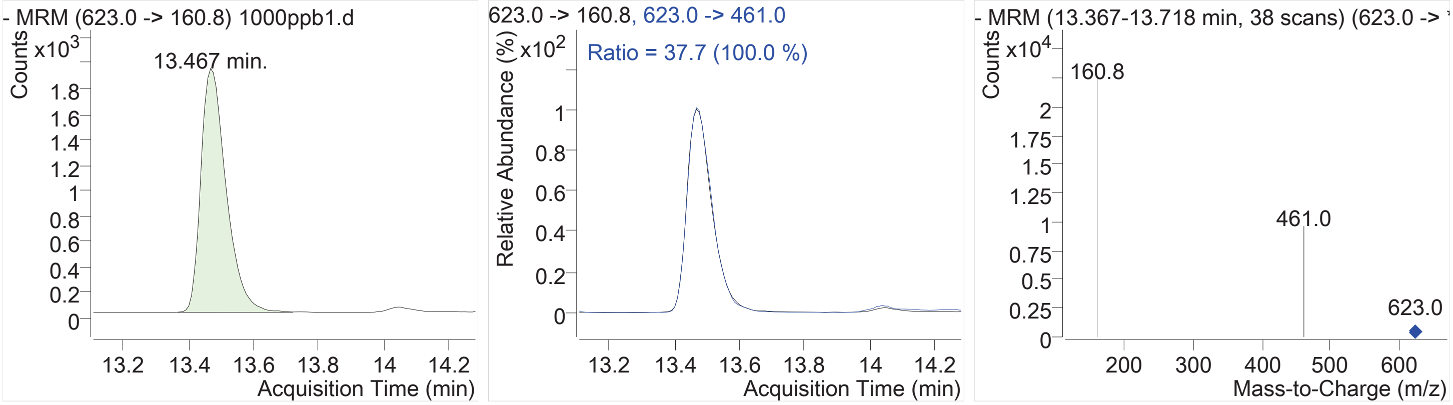

Taxifolin

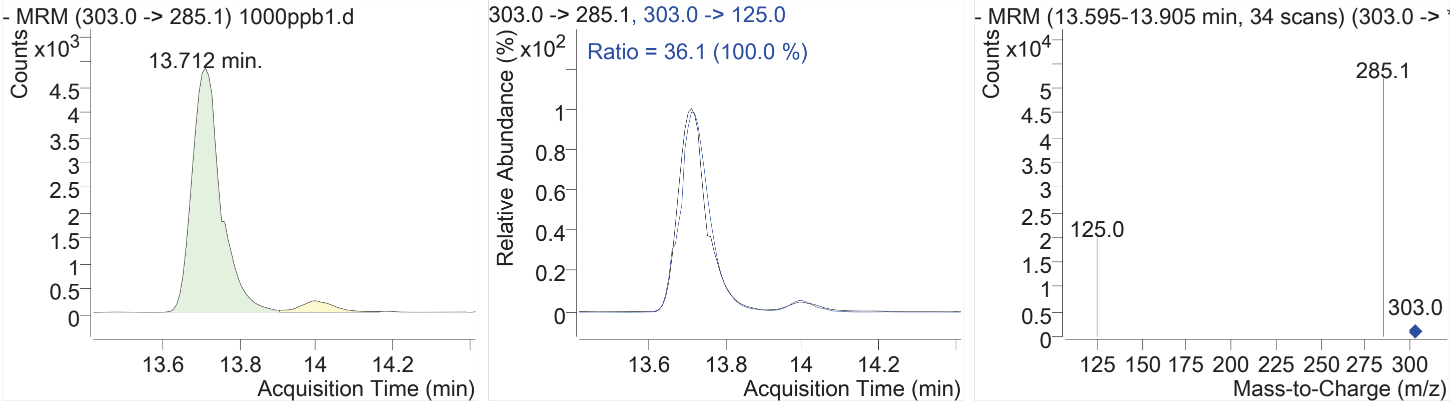

p-Coumaric acid

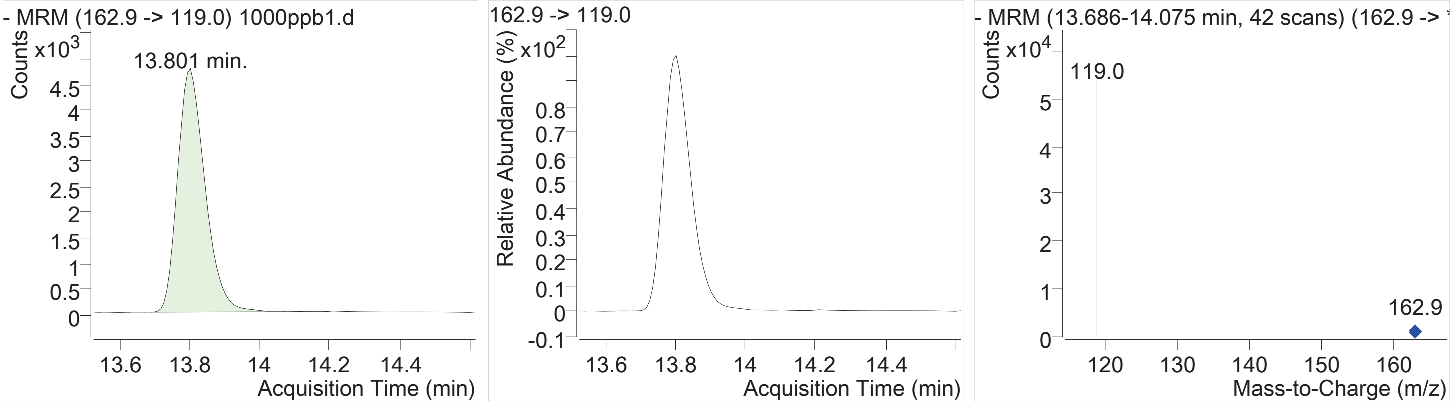

Sinapic acid

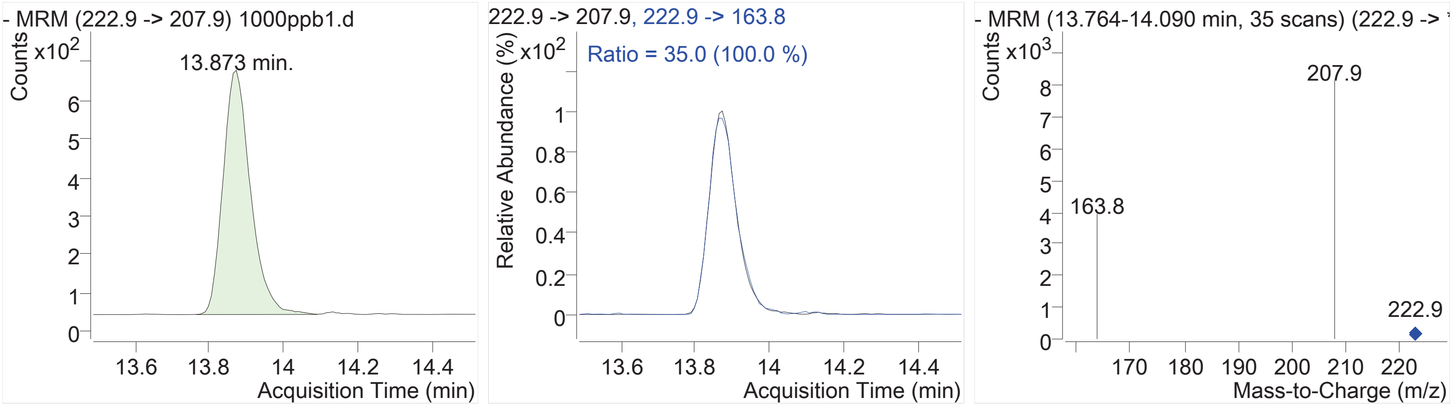

Ferulic acid

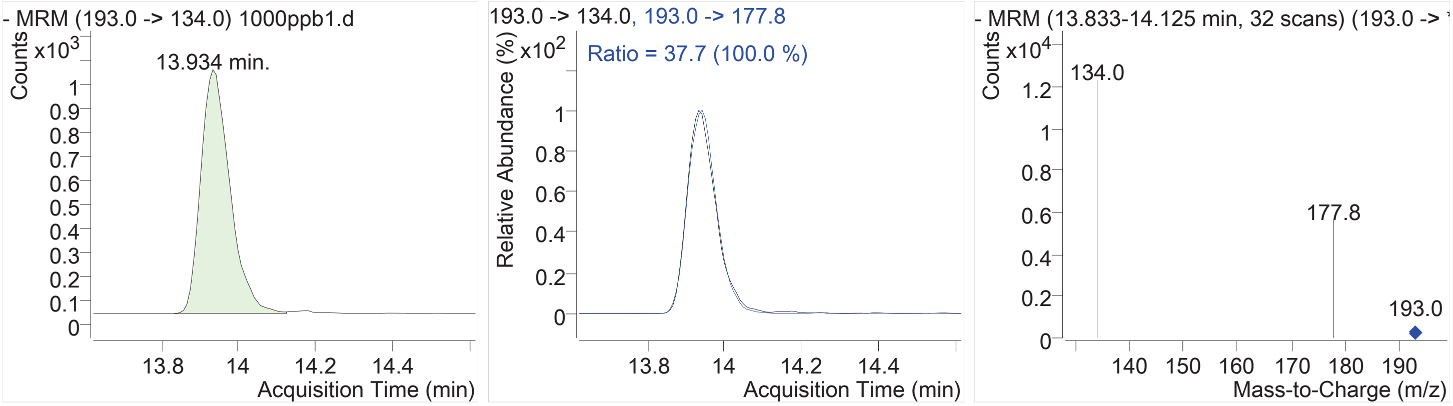

Luteolin 7-glucoside

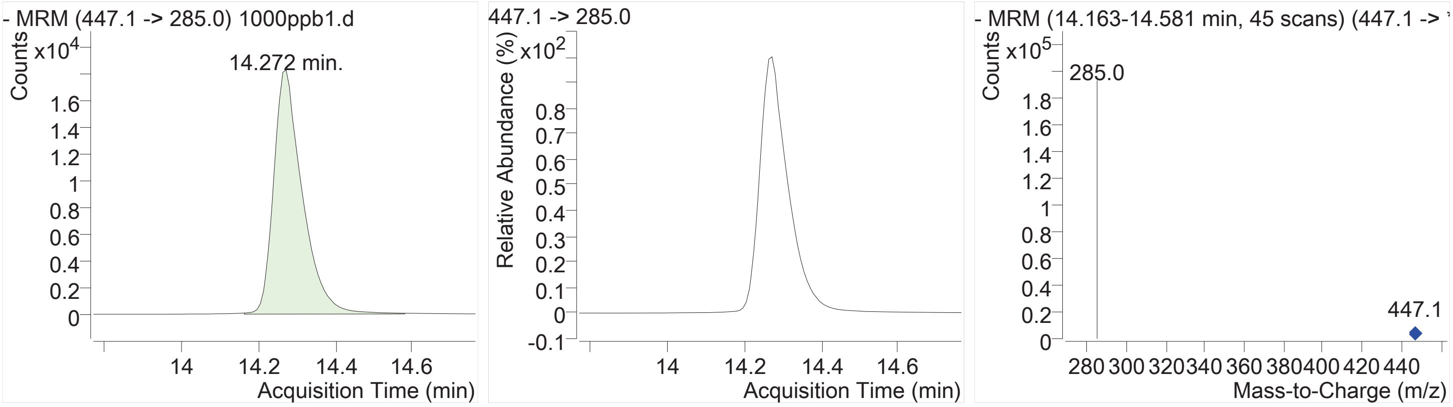

Hesperidin

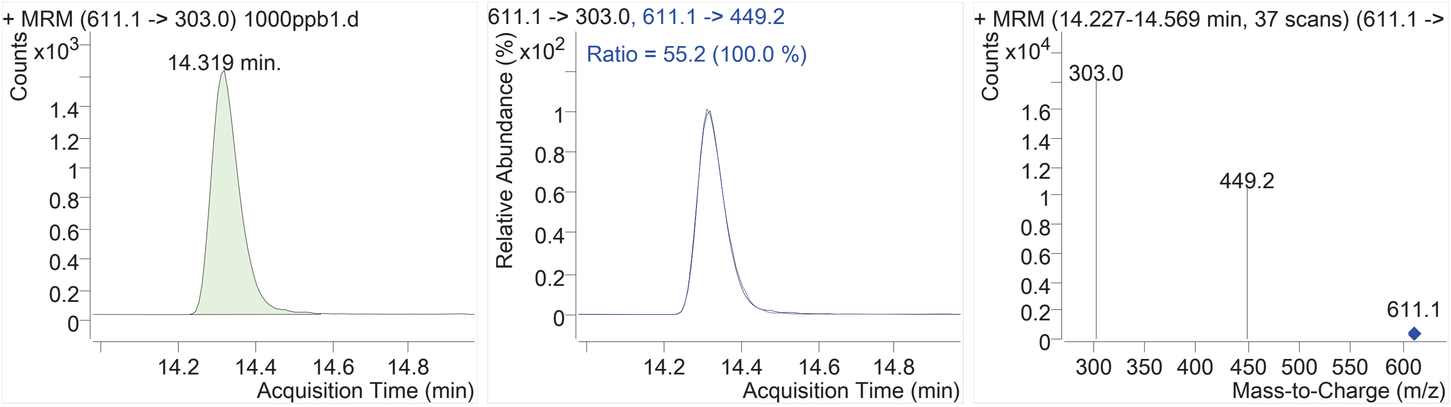

Agilent Technologies

Hyperoside

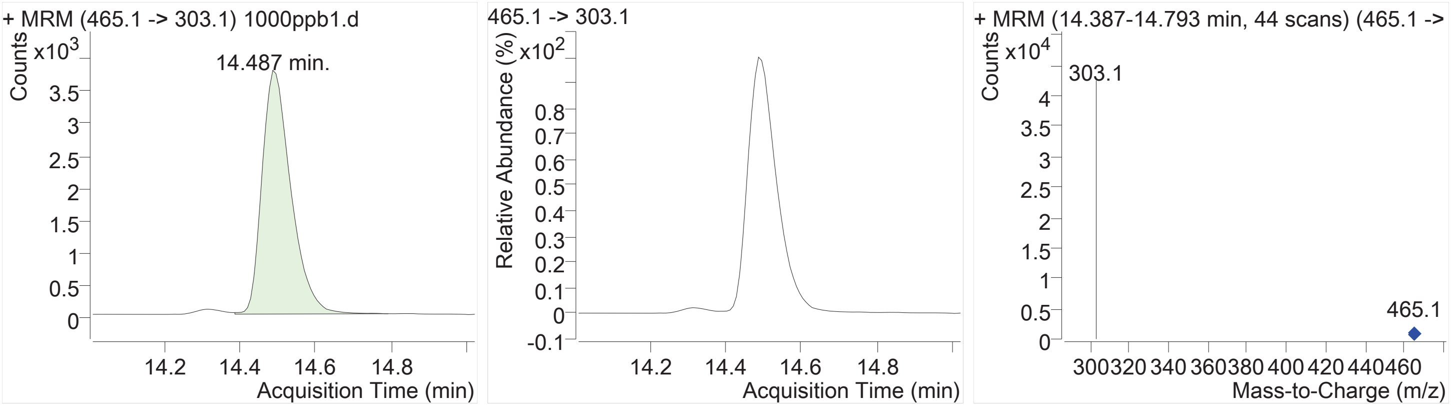

Rosmarinic acid

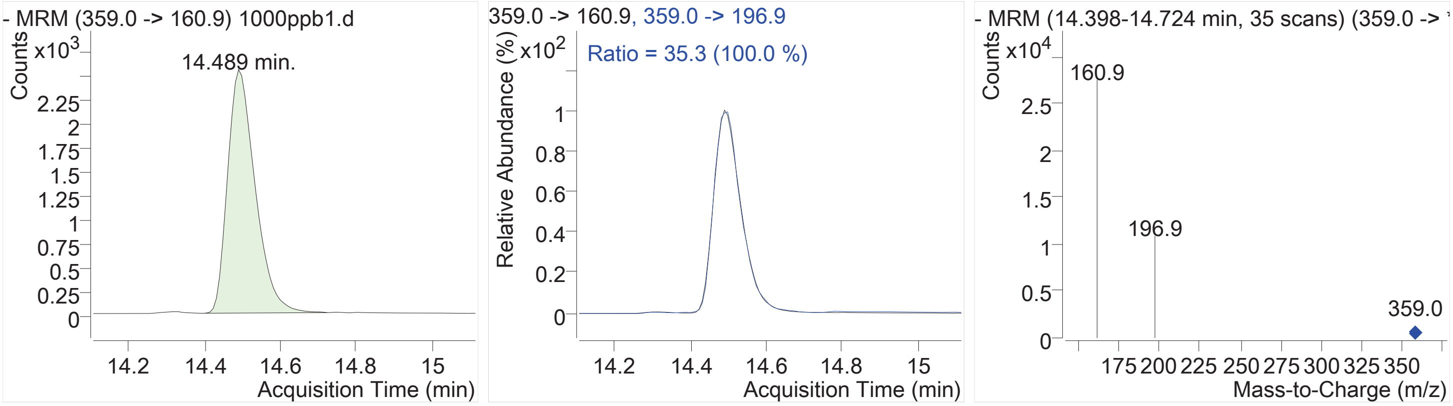

Apigenin 7-glucoside

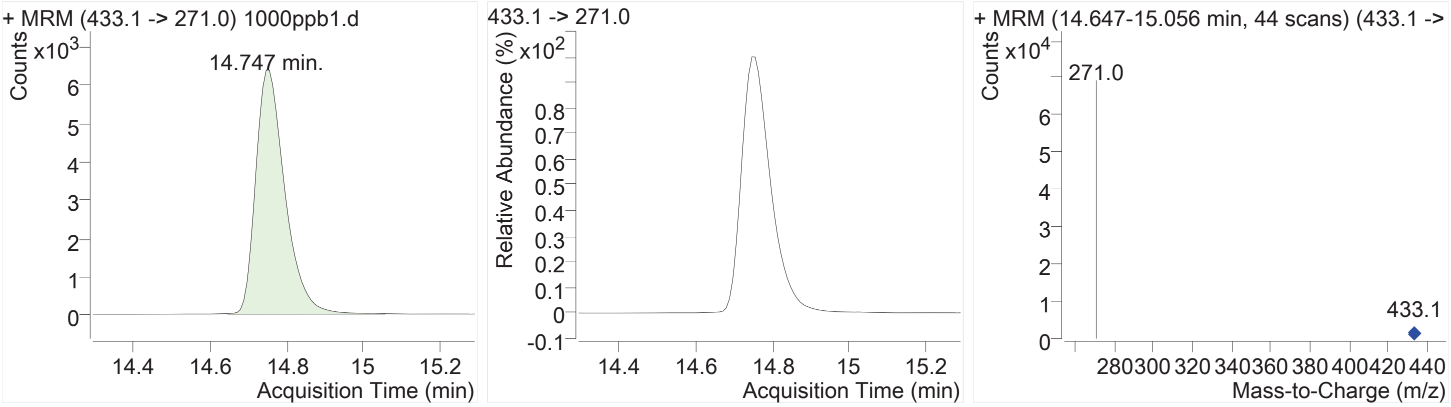

Pinoresinol

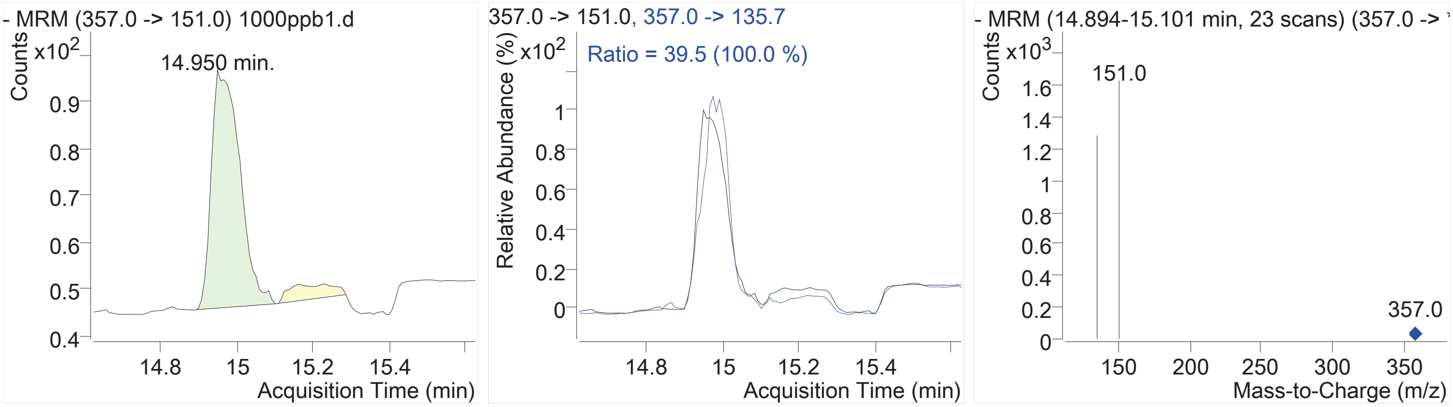

2-Hydroxycinnamic acid

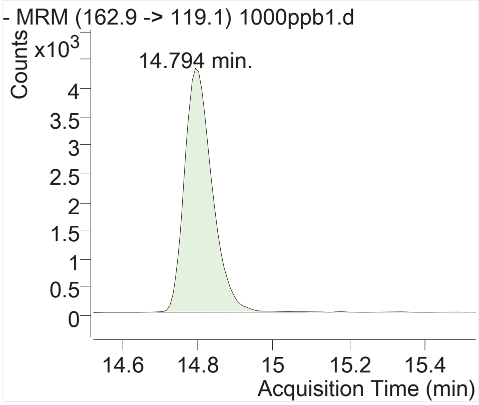

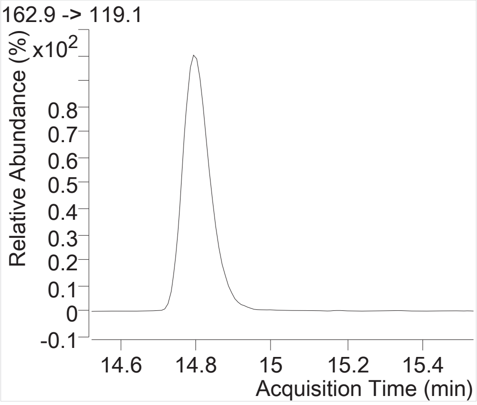

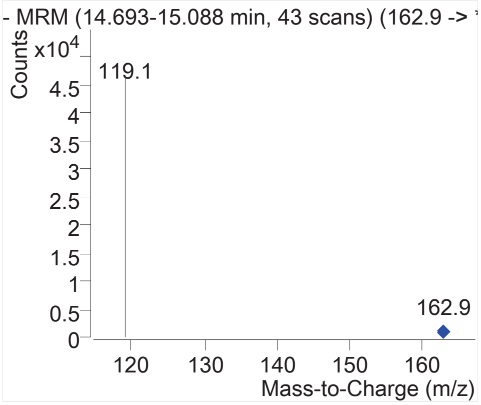

Eriodictyol

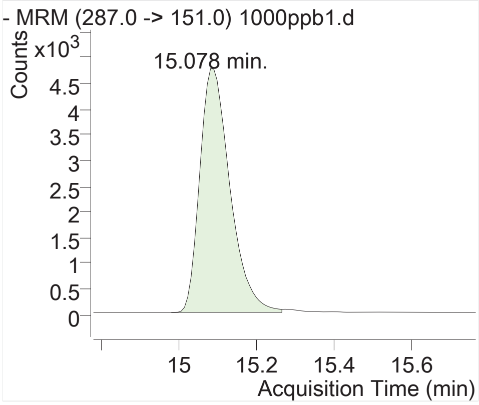

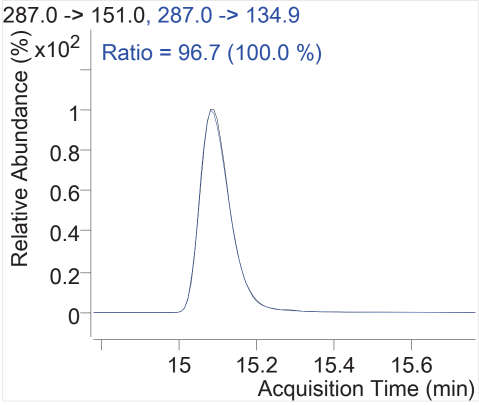

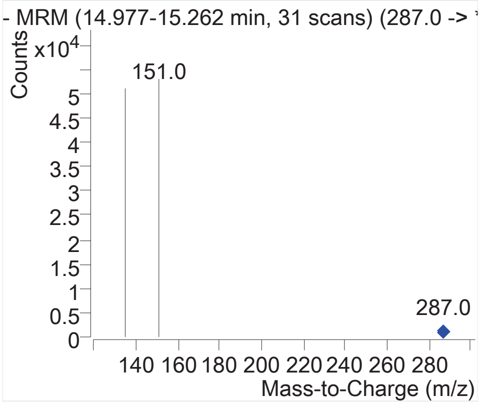

Quercetin

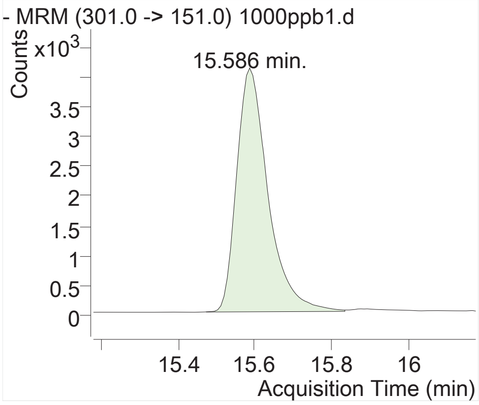

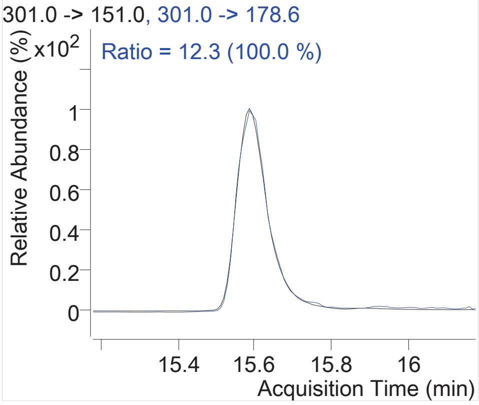

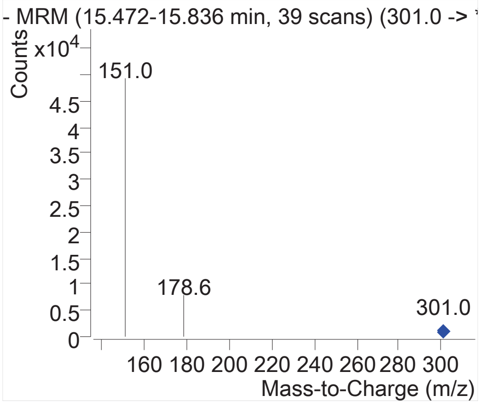

Luteolin

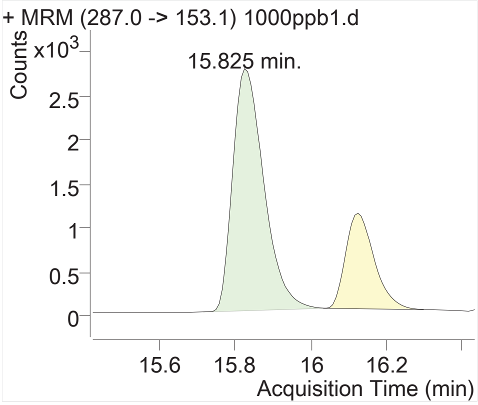

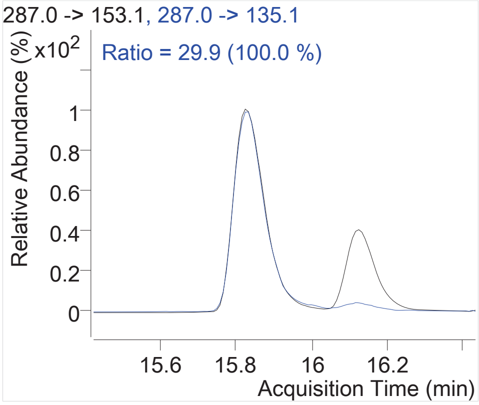

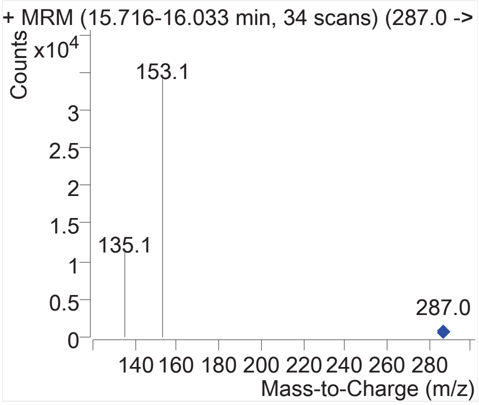

Kaempferol

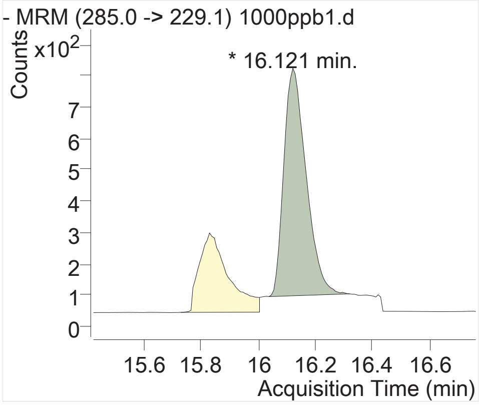

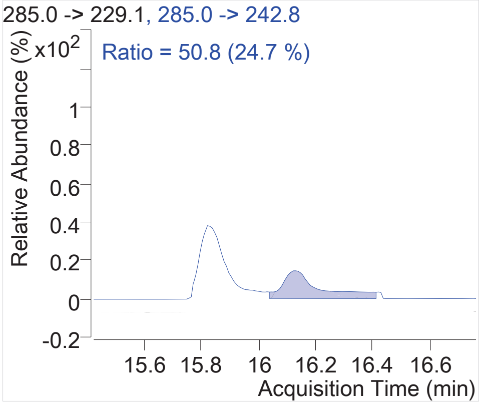

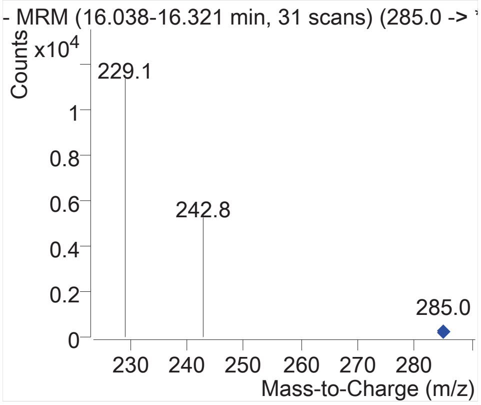

Apigenin

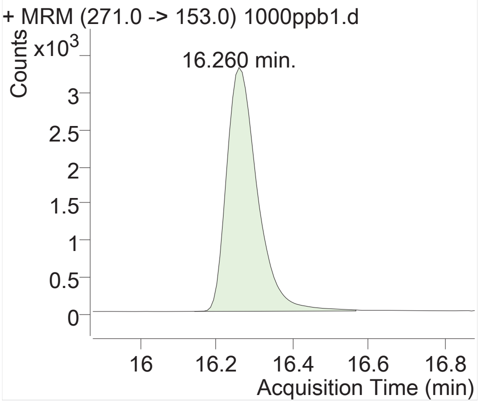

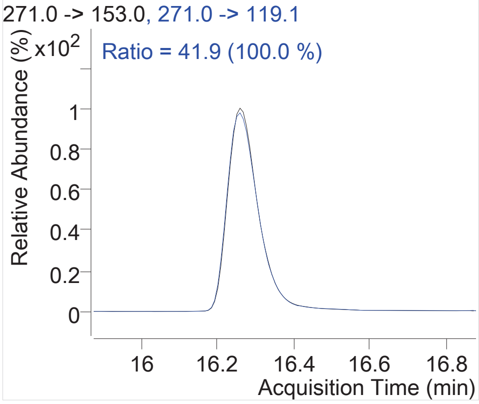

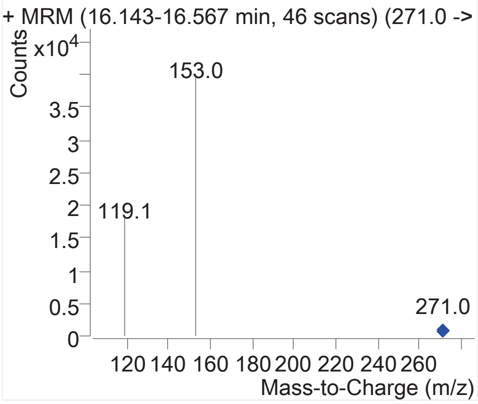

Quantitative Analysis Complete Report

**Batch Path**	D:\MassHunter\Data\2022ekim\061022cengizhoca\QuantResults\071022.batch.bin		
**Analysis Time**	10/11/2022 1:33:26 PM	**Analyst Name**	Defam-PC\admin
**Report Time**	10/11/2022 1:34:37 PM	**Reporter Name**	admin
**Last Calib Update**	10/11/2022 1:33:17 PM	**Batch State**	Processed
**Quant Batch Version**	B.07.01	**Quant Report Version**	B.07.01

**Acq. Time**	10/6/2022 9:42:03 PM	**Data File**	1000ppb2.d
**Sample Type**	Cal	**Sample Name**	1000ppb2
**Dilution**	1	**Acq. Method**	FENOLIK_DMRM2021-31bilesen.m

Sample Chromatogram

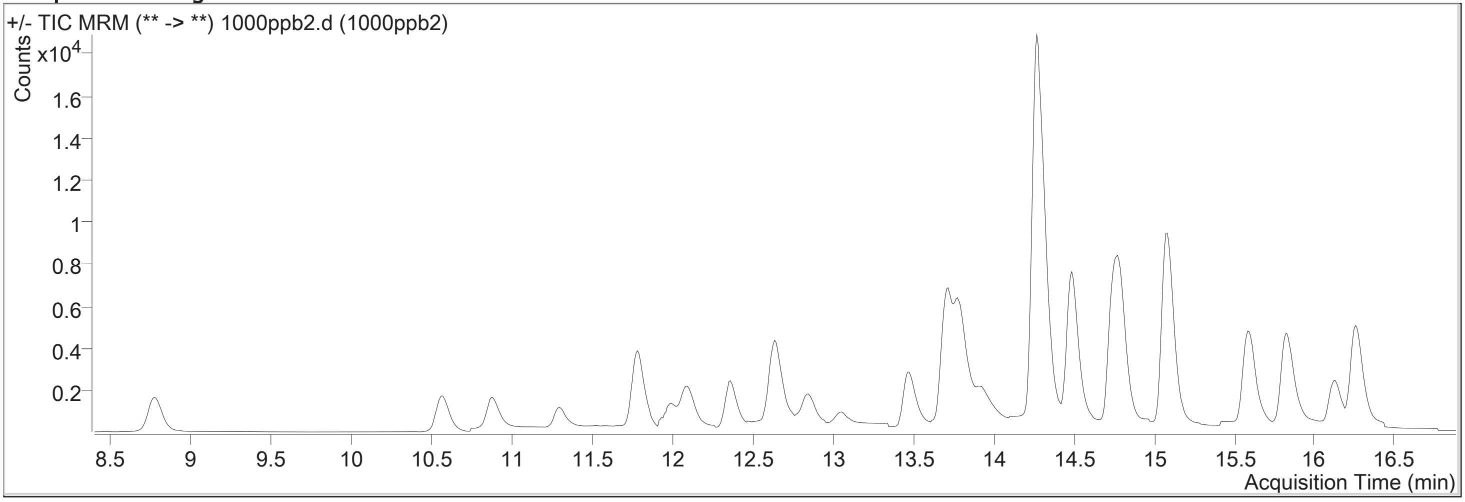

Compound	Transition	RT	Resp.	Final Conc	Units
Gallic acid	168.9 -> 125.0	8.783	10374	952.9453	ng/ml
Protocatechuic acid	152.9 -> 108.9	10.568	10071	962.6984	ng/ml
Pyrocatechol	109.0 -> 52.9	10.822	309	1000.9603	ng/ml
3,4-Dihydroxyphenylacetic acid	167.0 -> 123.0	10.888	7824	978.6204	ng/ml
(+)-Catechin	289.0 -> 245.0	11.302	3317	993.0585	ng/ml
2,5-Dihydroxybenzoic acid	152.9 -> 109.0	11.996	6265	990.5379	ng/ml
Chlorogenic acid	355.0 -> 163.0	11.793	20594	972.4178	ng/ml
3-Hydroxybenzoic acid	137.0 -> 93.0	12.853	4978	946.1062	ng/ml
4-Hydroxybenzoic acid	136.9 -> 93.1	12.097	10421	957.1763	ng/ml
(-)-Epicatechin	291.0 -> 139.1	12.369	7749	979.6259	ng/ml
Caffeic acid	179.0 -> 135.0	12.641	23033	965.2409	ng/ml
Syringic acid	196.9 -> 181.9	12.757	708	990.2416	ng/ml
Vanillin	151.0 -> 136.0	13.062	3313	972.8767	ng/ml
Verbascoside	623.0 -> 160.8	13.476	9803	969.6688	ng/ml
Taxifolin	303.0 -> 285.1	13.712	25452	969.0002	ng/ml
p-Coumaric acid	162.9 -> 119.0	13.801	24851	960.8410	ng/ml
Sinapic acid	222.9 -> 207.9	13.873	3138	993.7140	ng/ml
Ferulic acid	193.0 -> 134.0	13.934	5066	972.2530	ng/ml
Luteolin 7-glucoside	447.1 -> 285.0	14.272	89837	953.3428	ng/ml
Hesperidin	611.1 -> 303.0	14.319	7866	958.7951	ng/ml
Hyperoside	465.1 -> 303.1	14.495	19114	971.3325	ng/ml
Rosmarinic acid	359.0 -> 160.9	14.489	12233	976.9791	ng/ml
Apigenin 7-glucoside	433.1 -> 271.0	14.755	32942	978.4026	ng/ml
Pinoresinol	357.0 -> 151.0	14.967	255	979.0101	ng/ml
2-Hydroxycinnamic acid	162.9 -> 119.1	14.794	20803	979.0892	ng/ml
Eriodictyol	287.0 -> 151.0	15.087	25161	980.1297	ng/ml
Quercetin	301.0 -> 151.0	15.586	22272	966.3349	ng/ml
Luteolin	287.0 -> 153.1	15.825	15325	963.2163	ng/ml
Kaempferol	285.0 -> 229.1	16.130	3807	917.4037	ng/ml

Agilent Technologies

Compound	Transition	RT	Resp.	Final Conc	Units
Apigenin	271.0 -> 153.0	16.260	18232	981.0949	ng/ml

Gallic acid

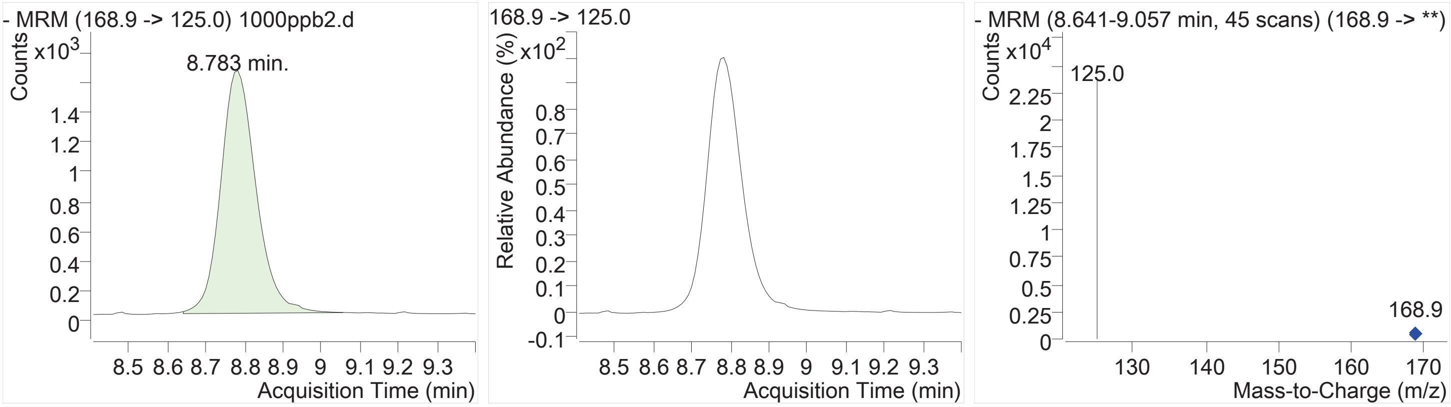

Protocatechuic acid

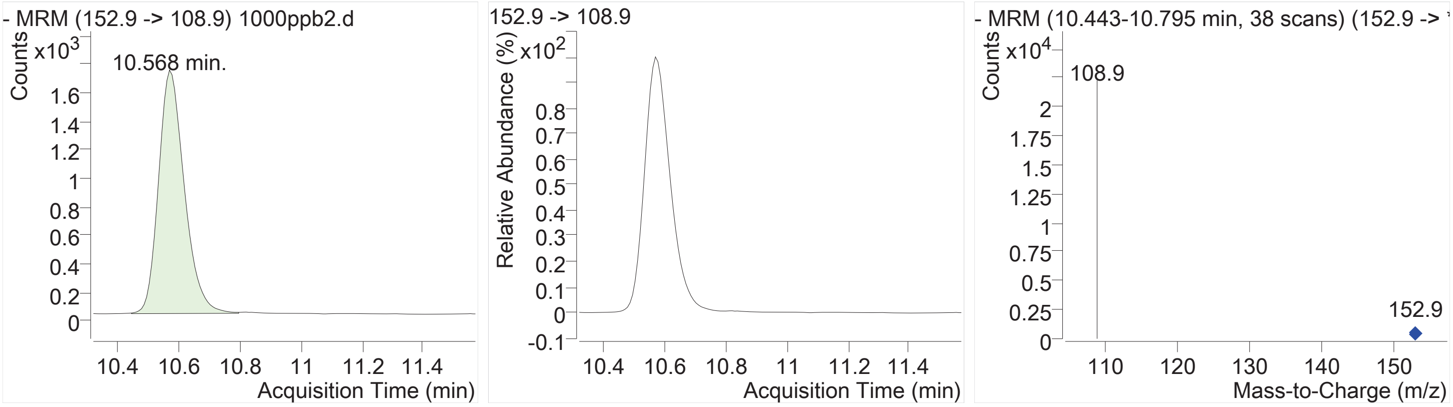

Pyrocatechol

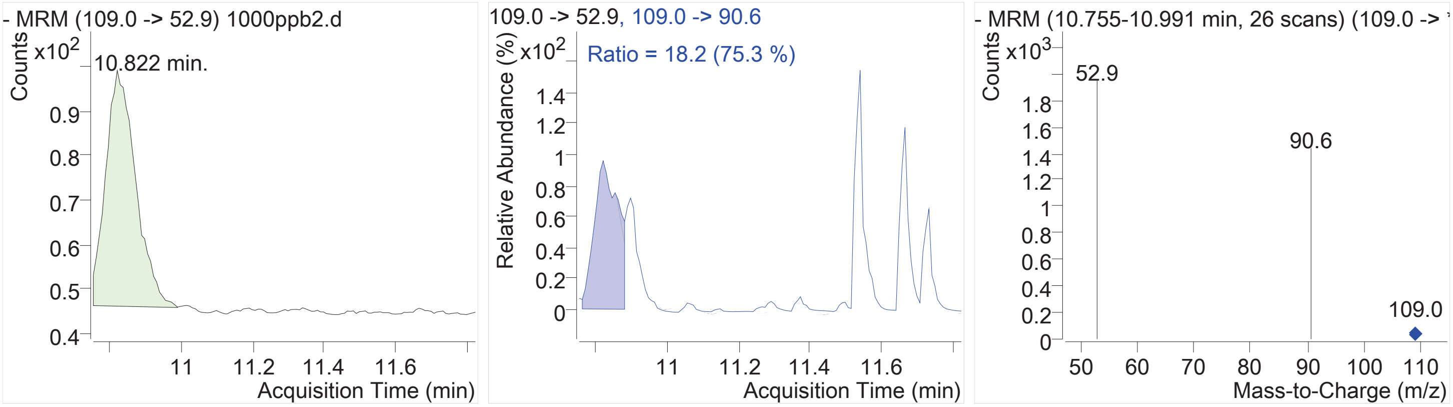

3,4-Dihydroxyphenylacetic acid

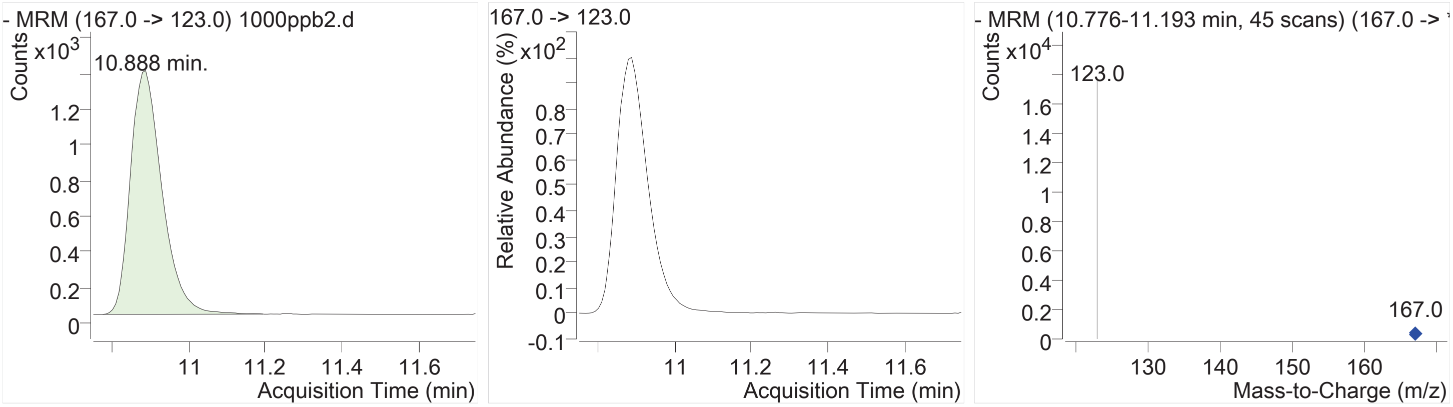

(+)-Catechin

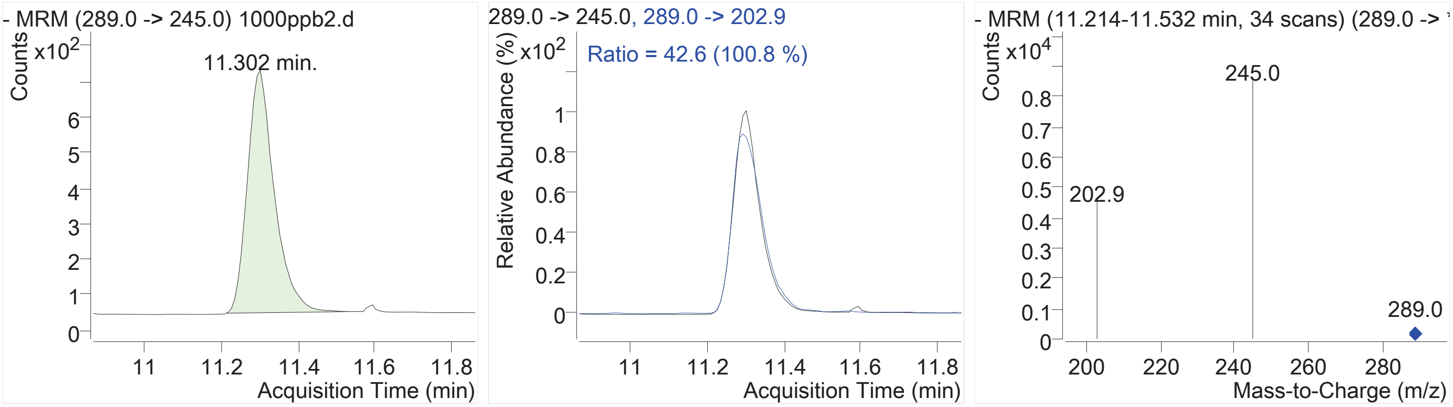

2,5-Dihydroxybenzoic acid

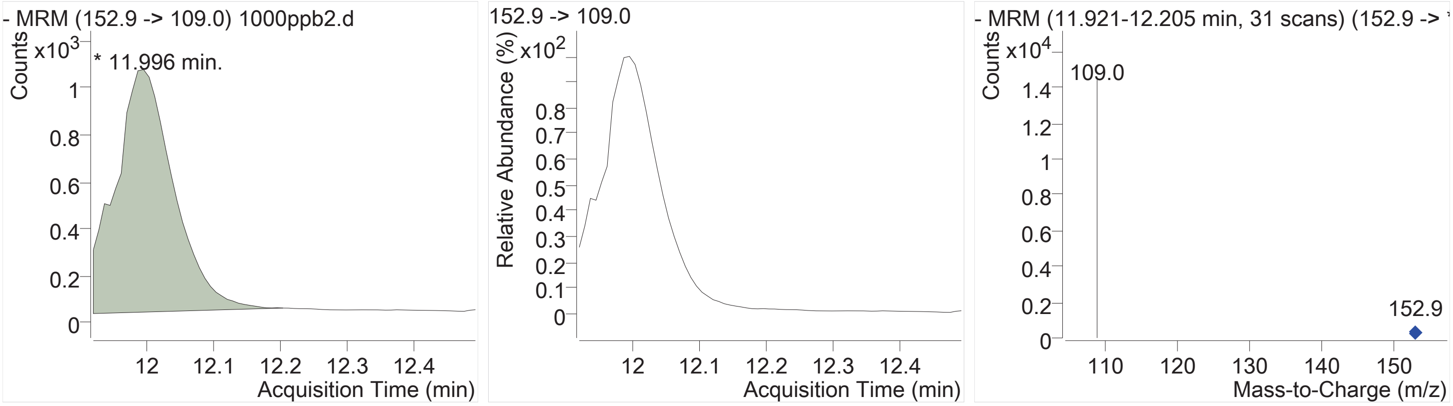

Chlorogenic acid

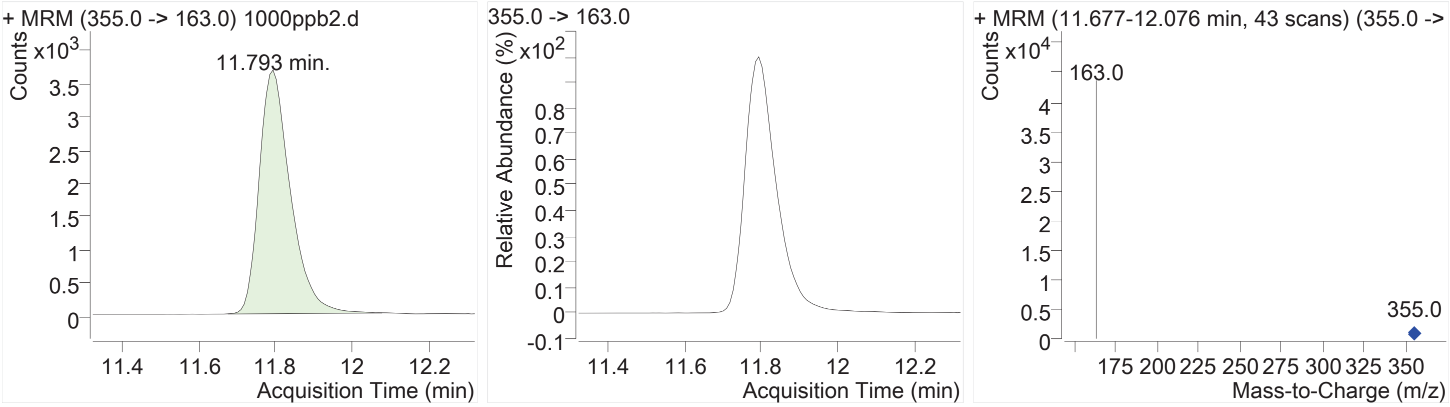

3-Hydroxybenzoic acid

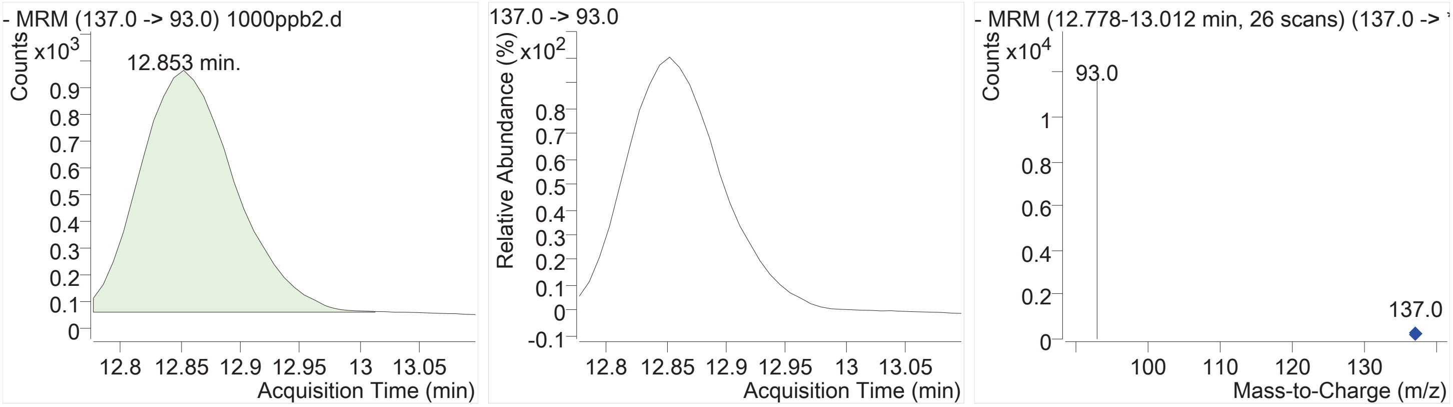

Agilent Technologies

4-Hydroxybenzoic acid

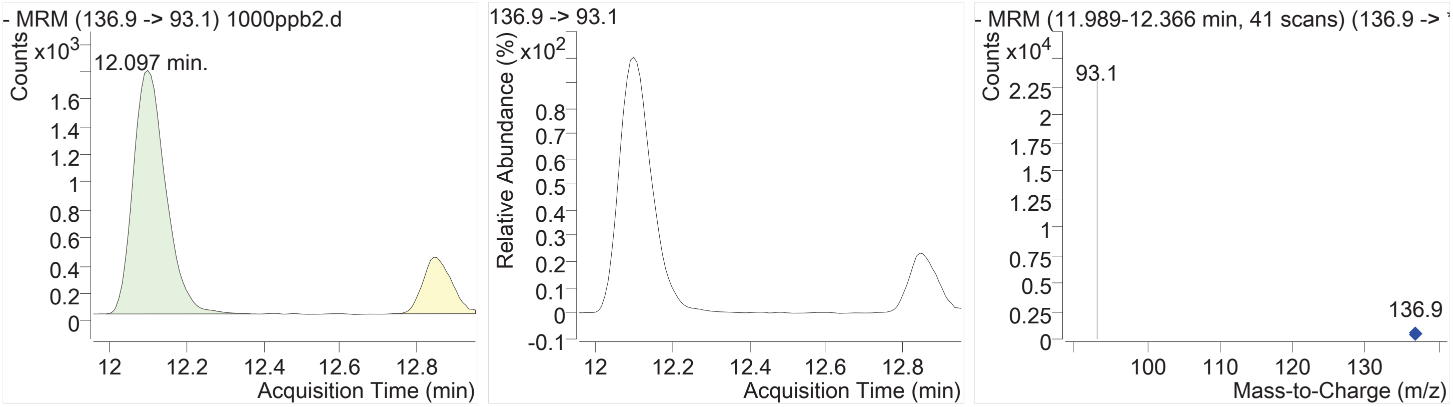

(-)-Epicatechin

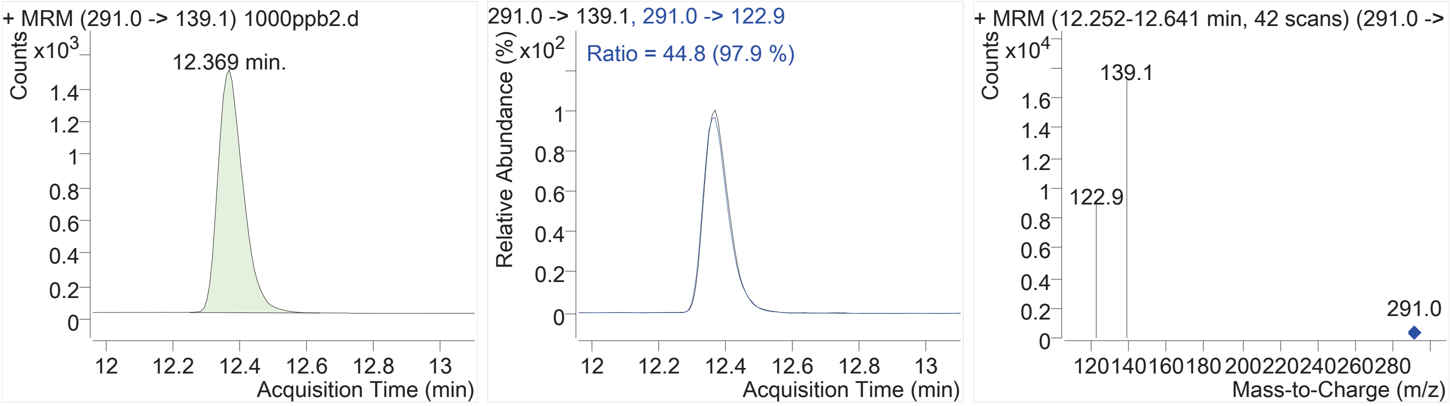

Caffeic acid

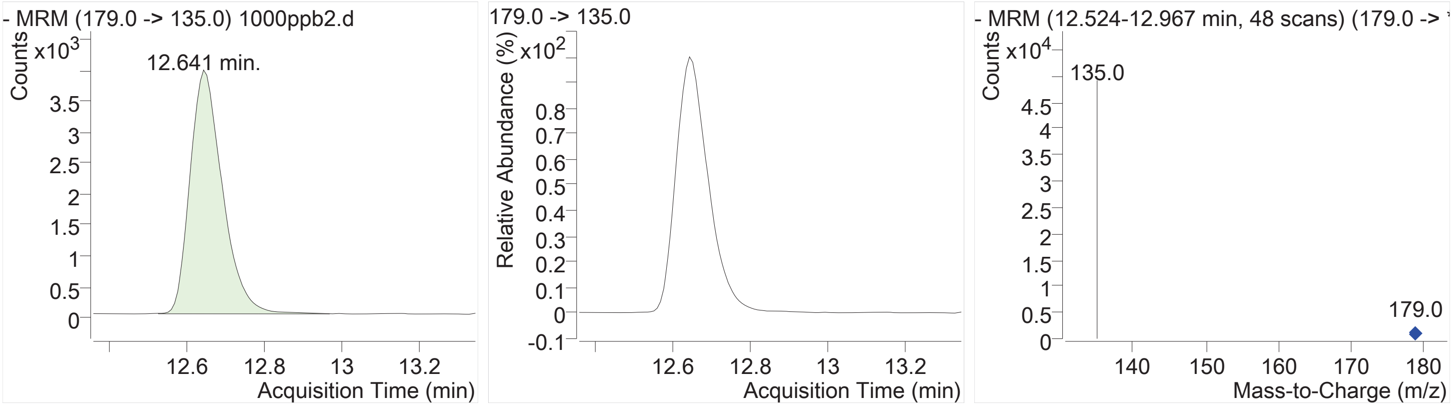

Syringic acid

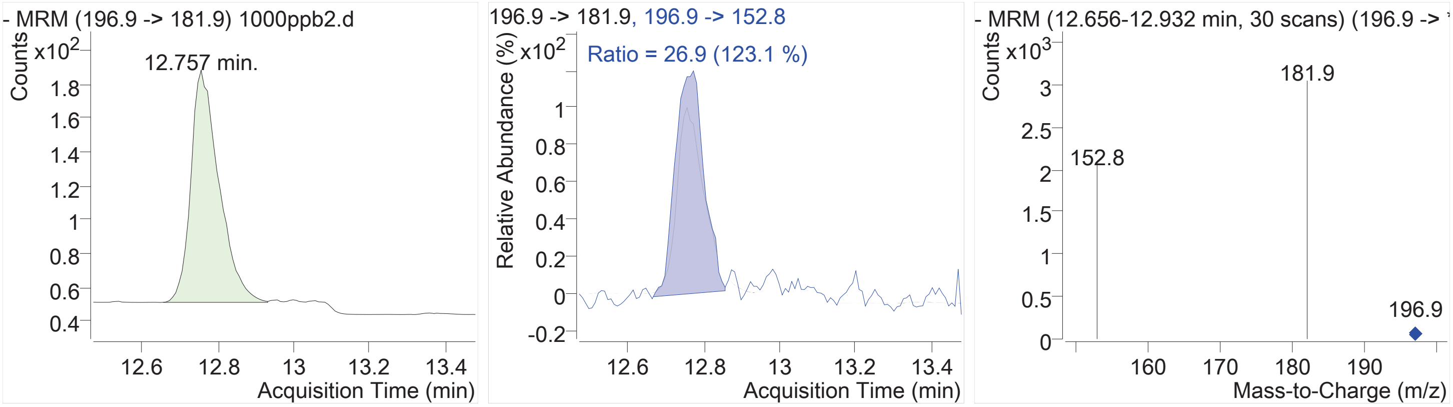

Vanillin

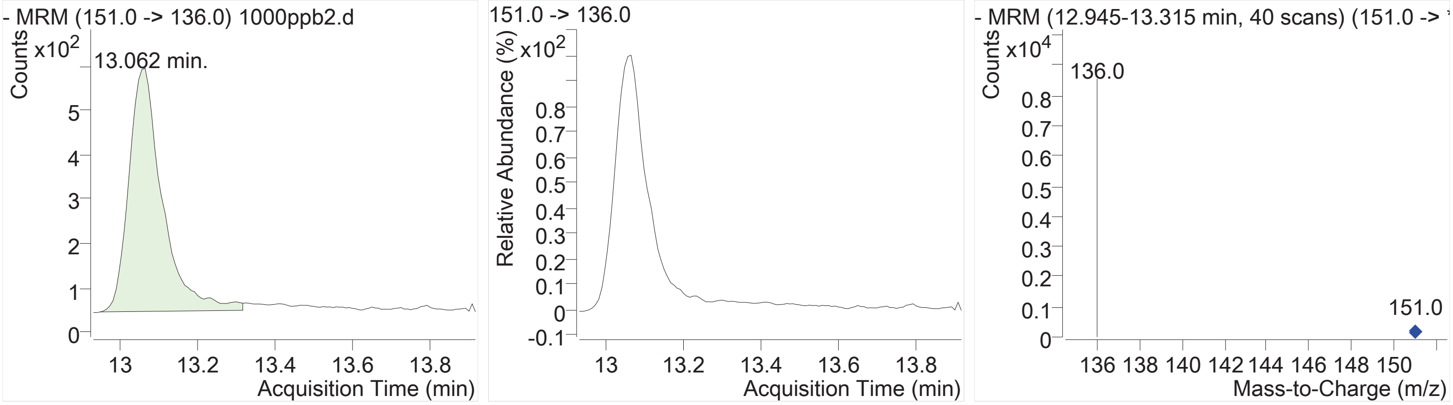

Verbascoside

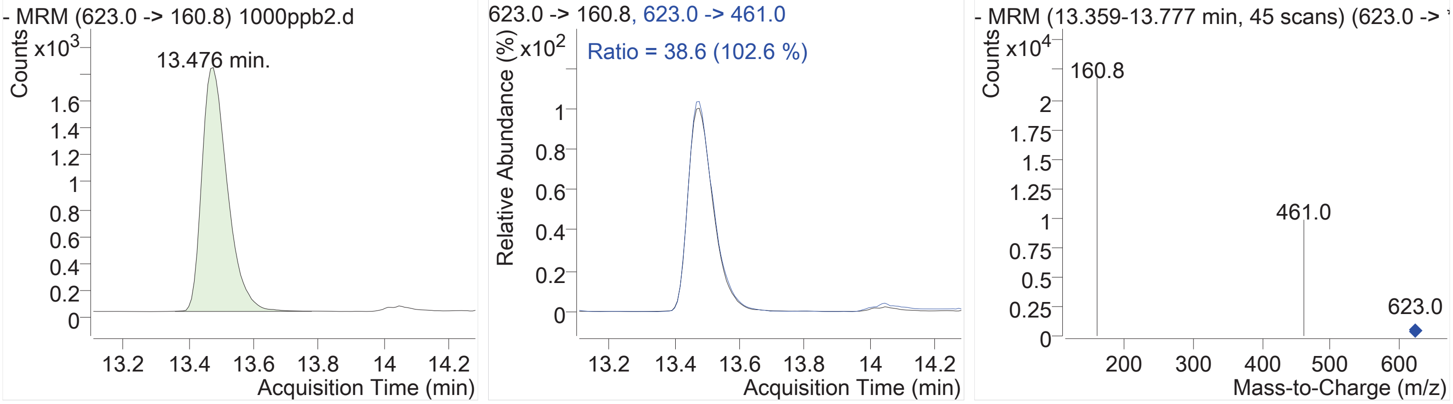

Taxifolin

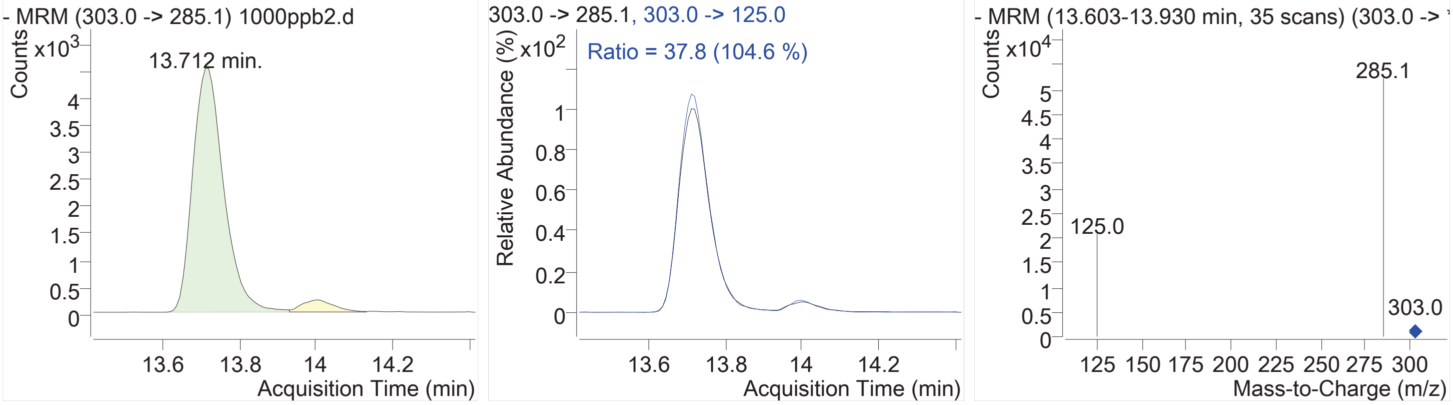

p-Coumaric acid

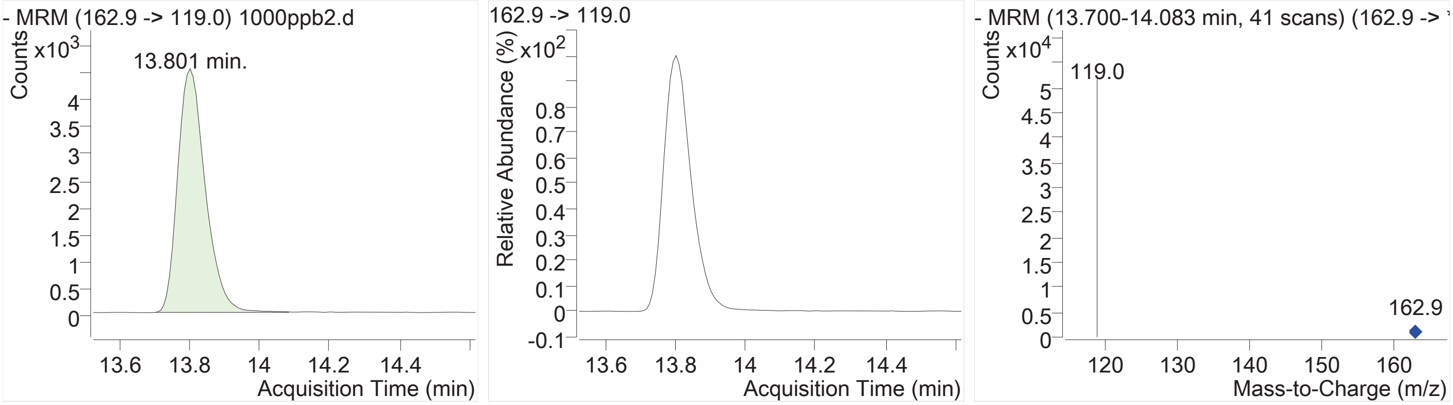

Agilent Technologies

Sinapic acid

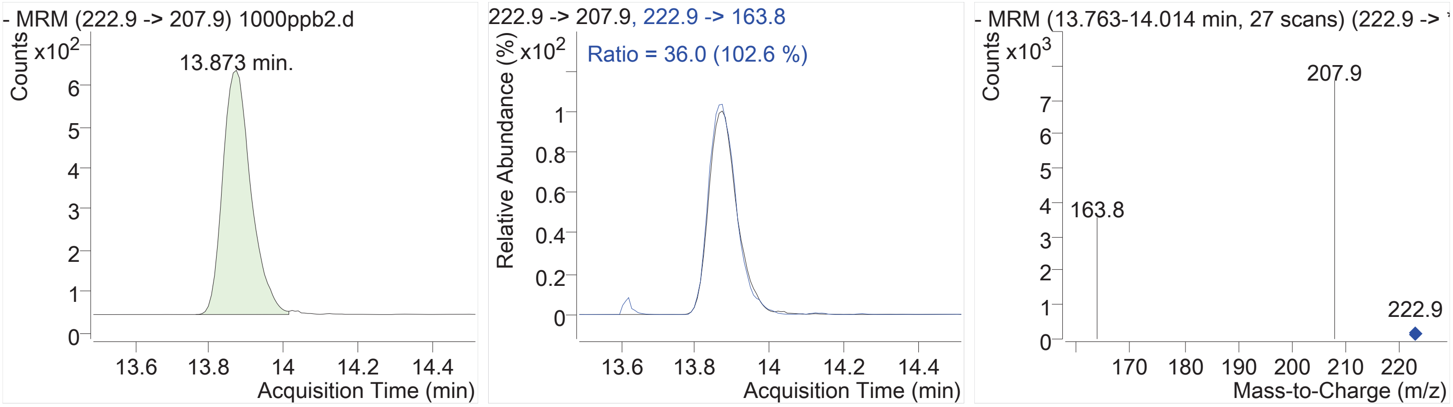

Ferulic acid

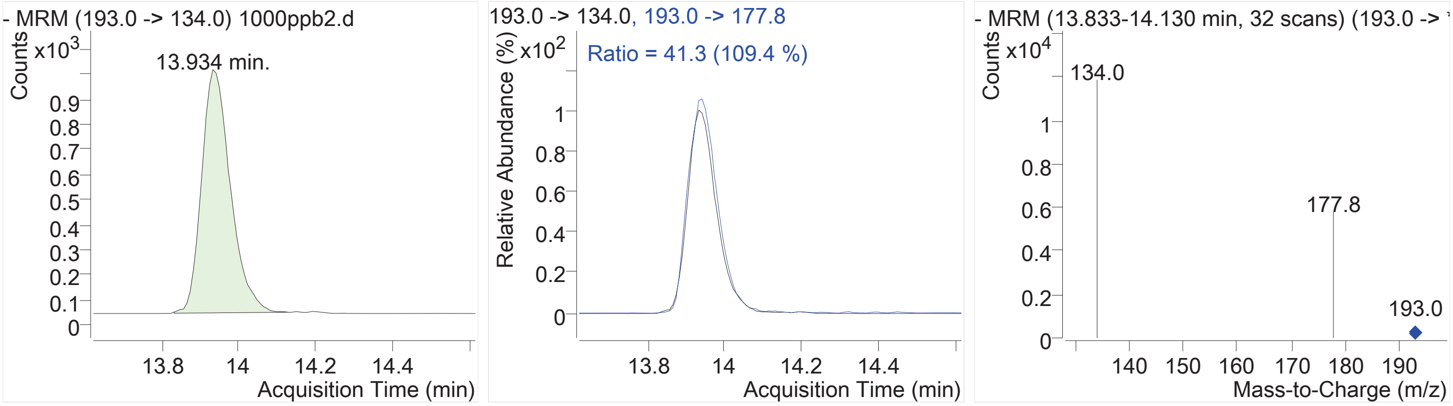

Luteolin 7-glucoside

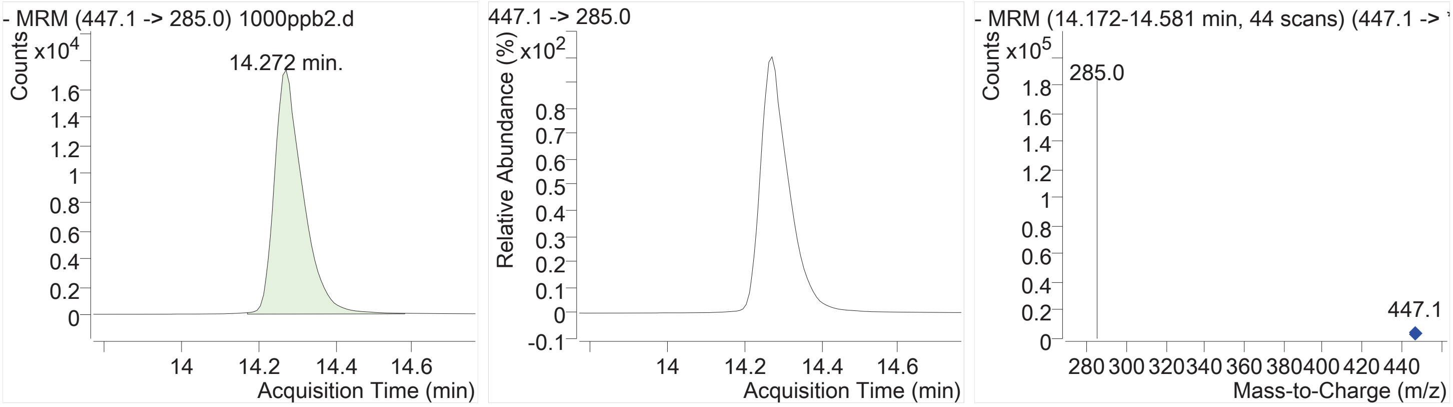

Hesperidin

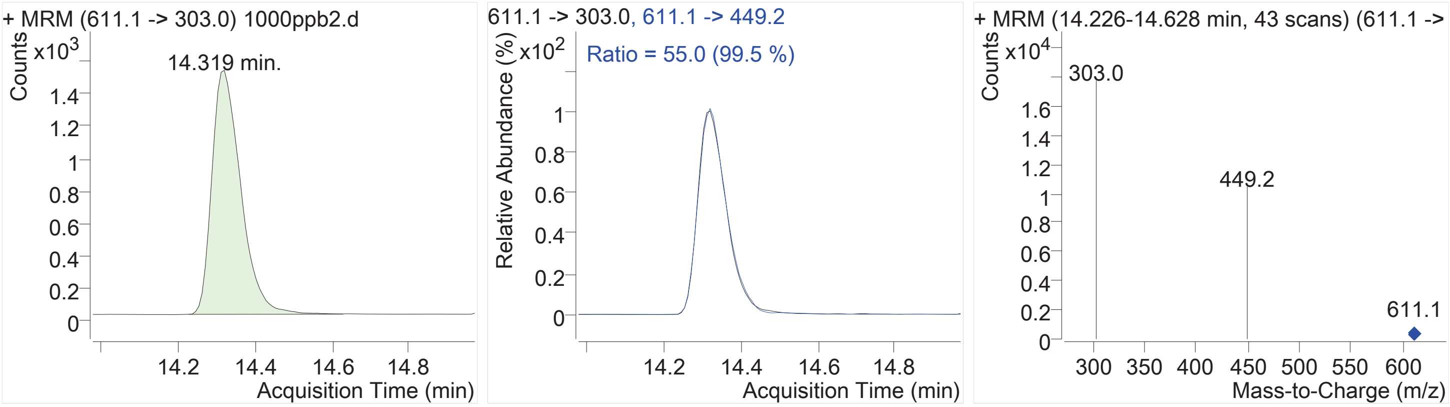

Hyperoside

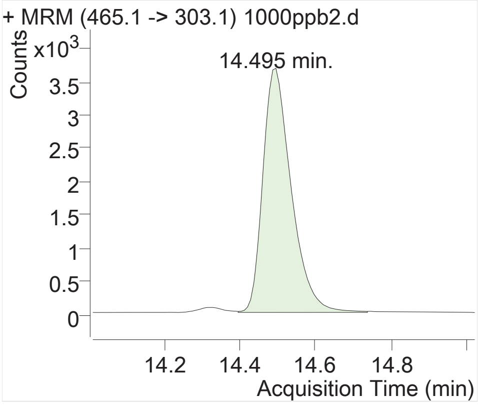

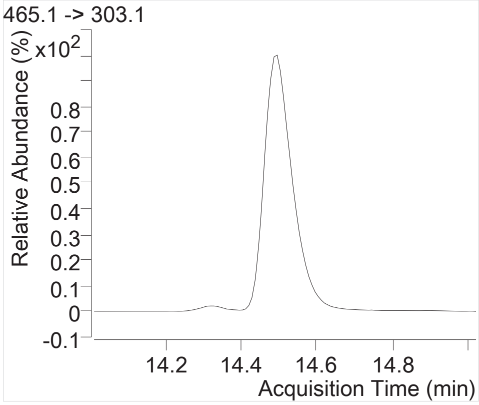

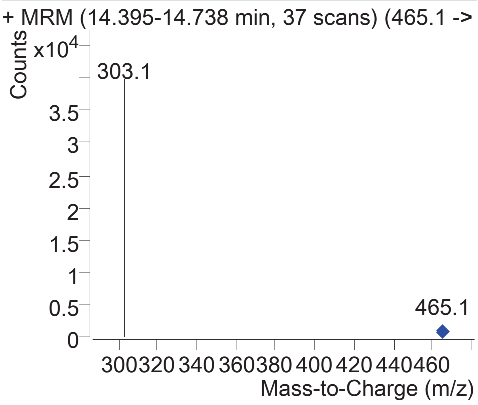

Rosmarinic acid

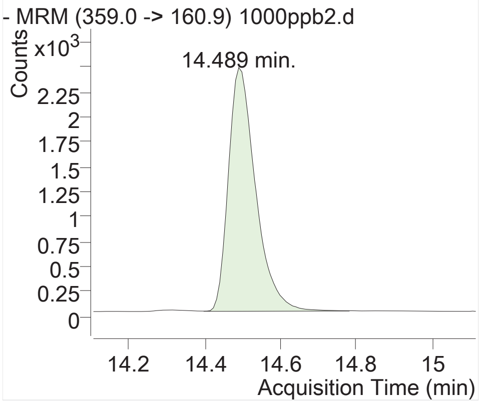

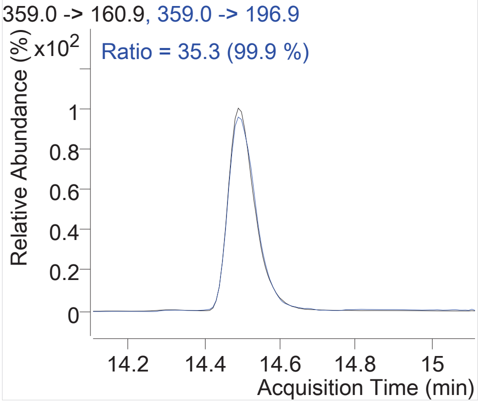

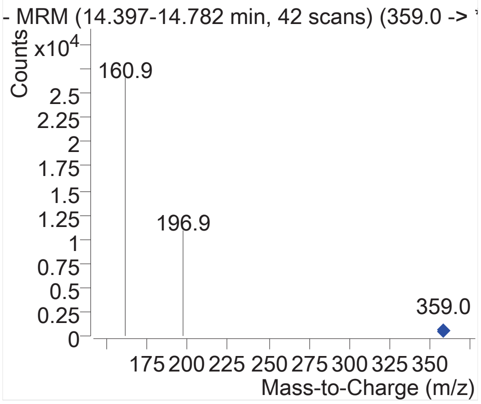

Apigenin 7-glucoside

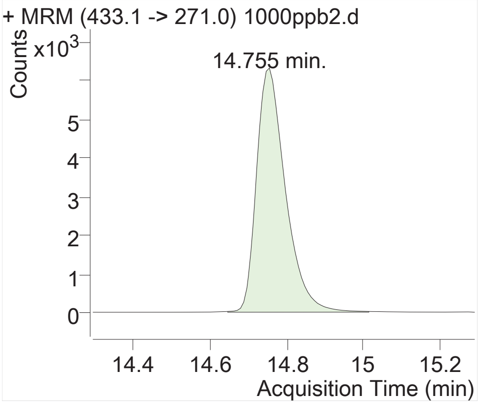

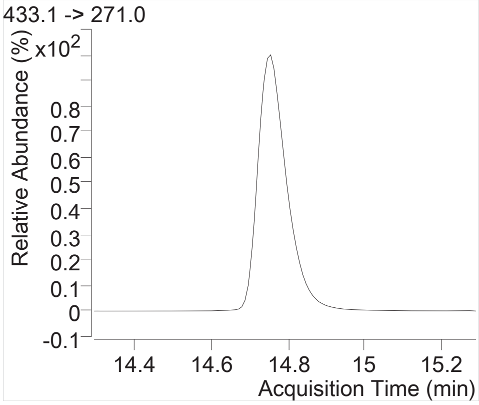

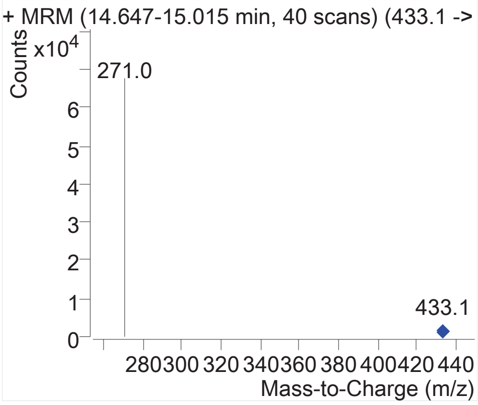

Pinoresinol

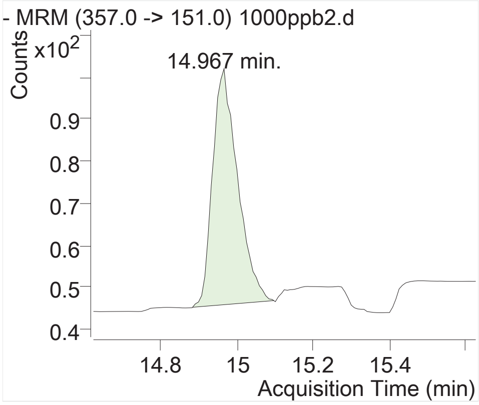

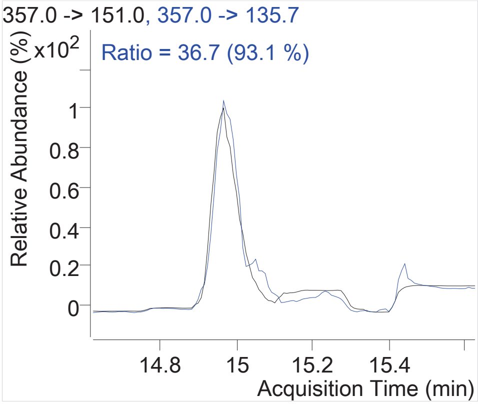

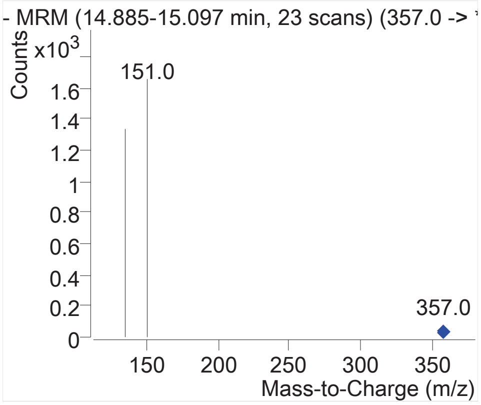

2-Hydroxycinnamic acid

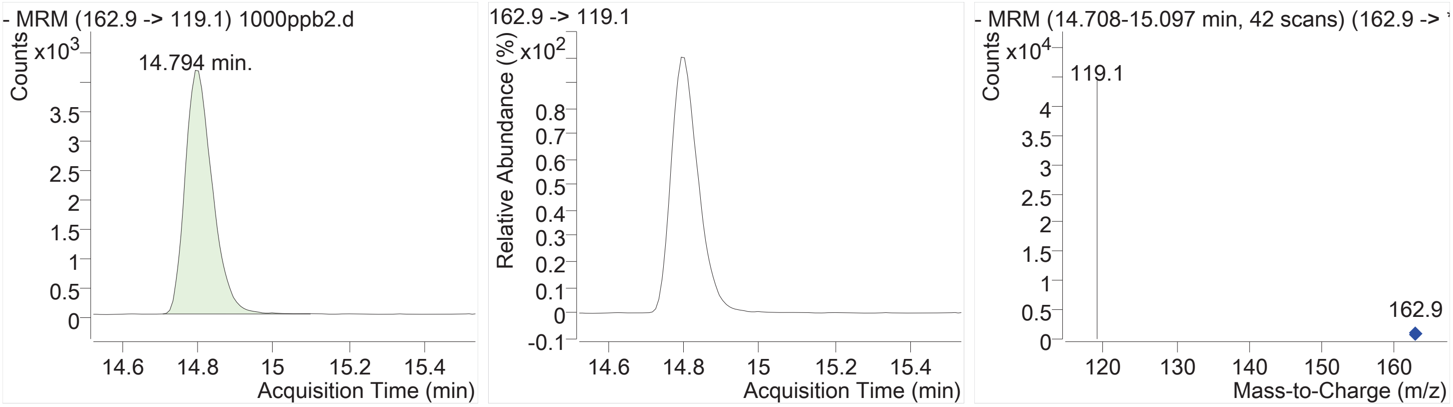

Eriodictyol

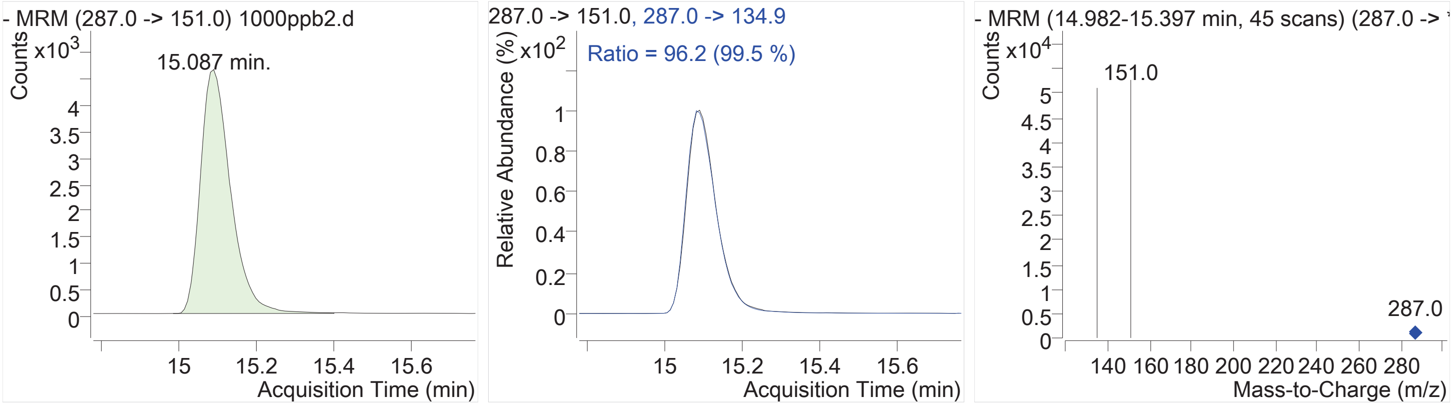

Quercetin

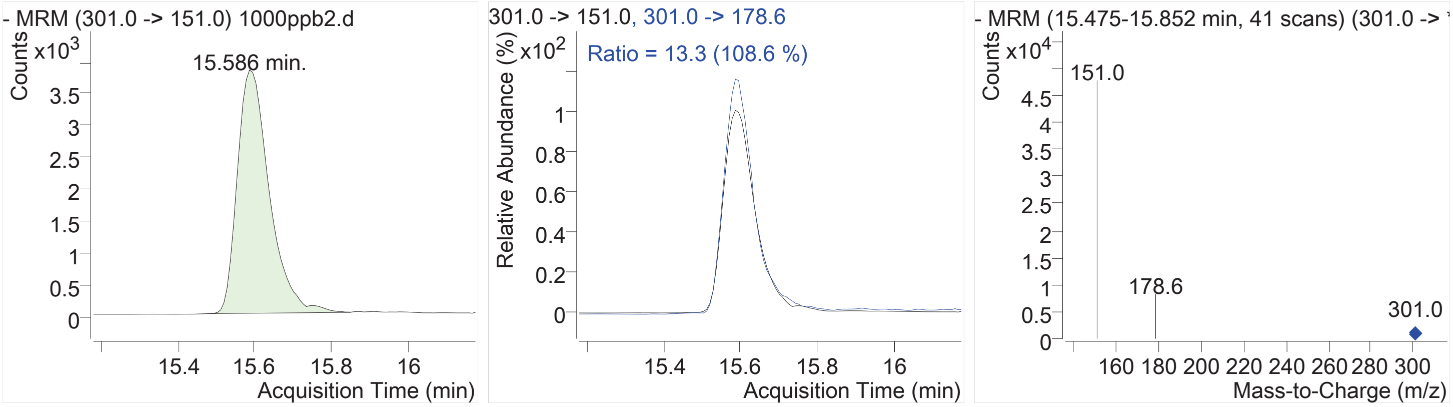

Luteolin

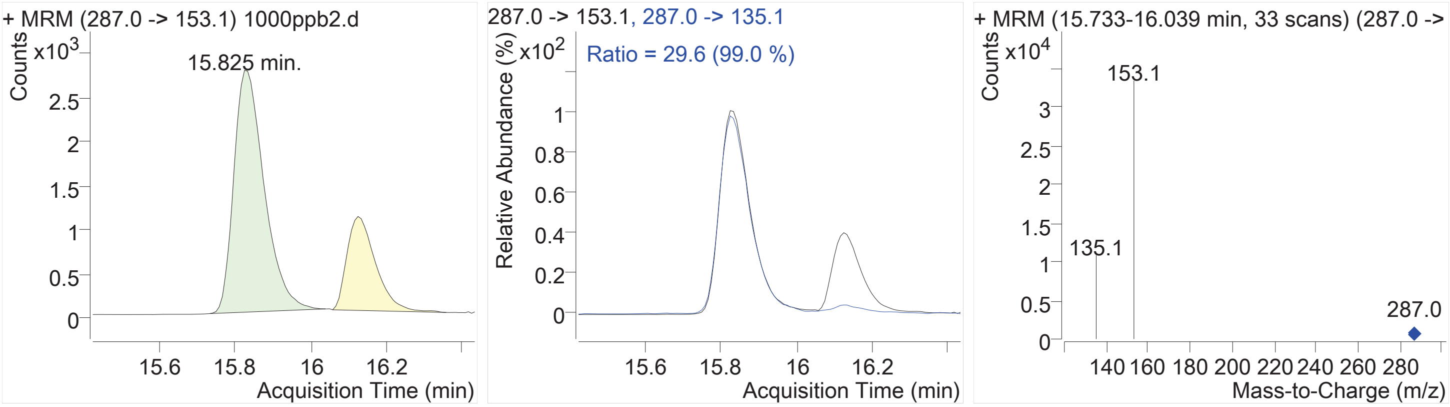

Quantitative Analysis Complete Report

Kaempferol

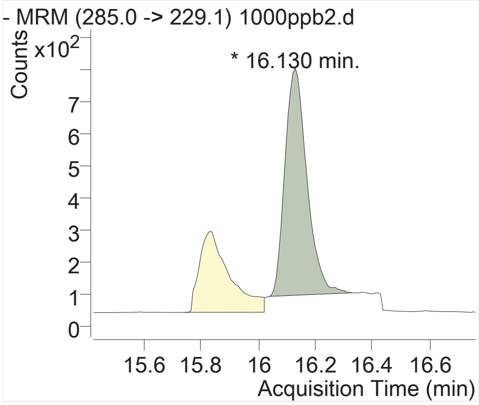

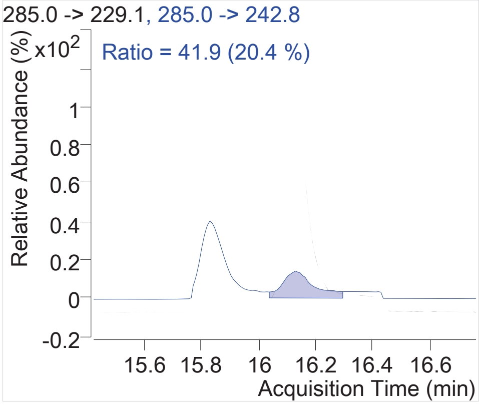

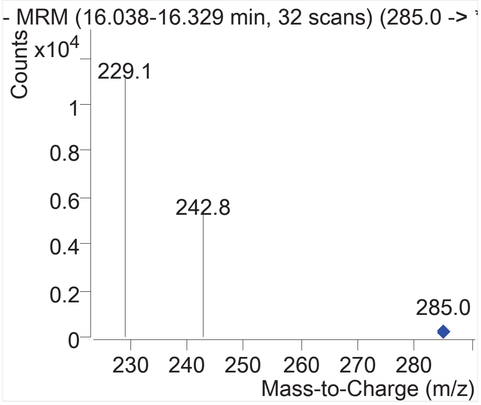

Apigenin

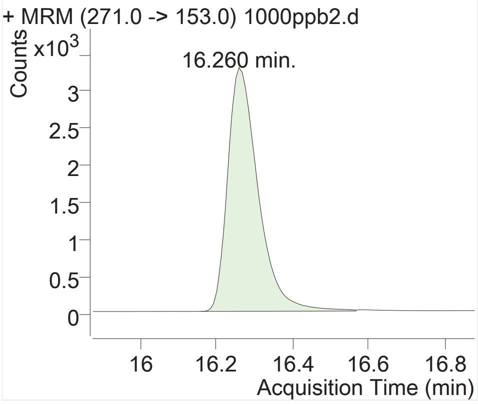

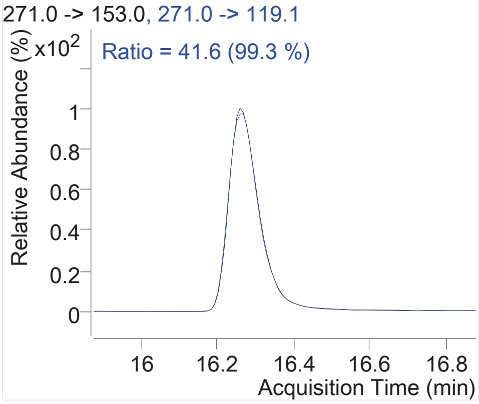

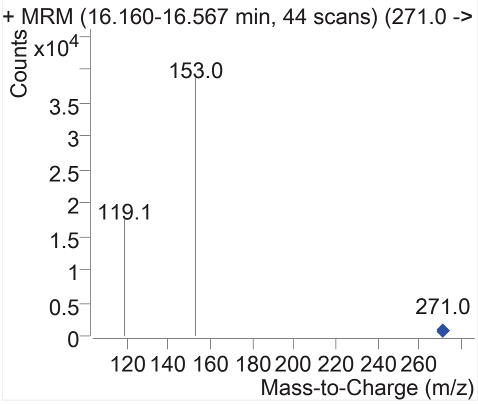

Quantitative Analysis Complete Report

**Batch Path**	D:\MassHunter\Data\2022ekim\061022cengizhoca\QuantResults\071022.batch.bin		
**Analysis Time**	10/11/2022 1:33:26 PM	**Analyst Name**	Defam-PC\admin
**Report Time**	10/11/2022 1:34:40 PM	**Reporter Name**	admin
**Last Calib Update**	10/11/2022 1:33:17 PM	**Batch State**	Processed
**Quant Batch Version**	B.07.01	**Quant Report Version**	B.07.01

**Acq. Time**	10/6/2022 10:03:44 PM	**Data File**	1000ppb3.d
**Sample Type**	Cal	**Sample Name**	1000ppb3
**Dilution**	1	**Acq. Method**	FENOLIK_DMRM2021-31bilesen.m

Sample Chromatogram

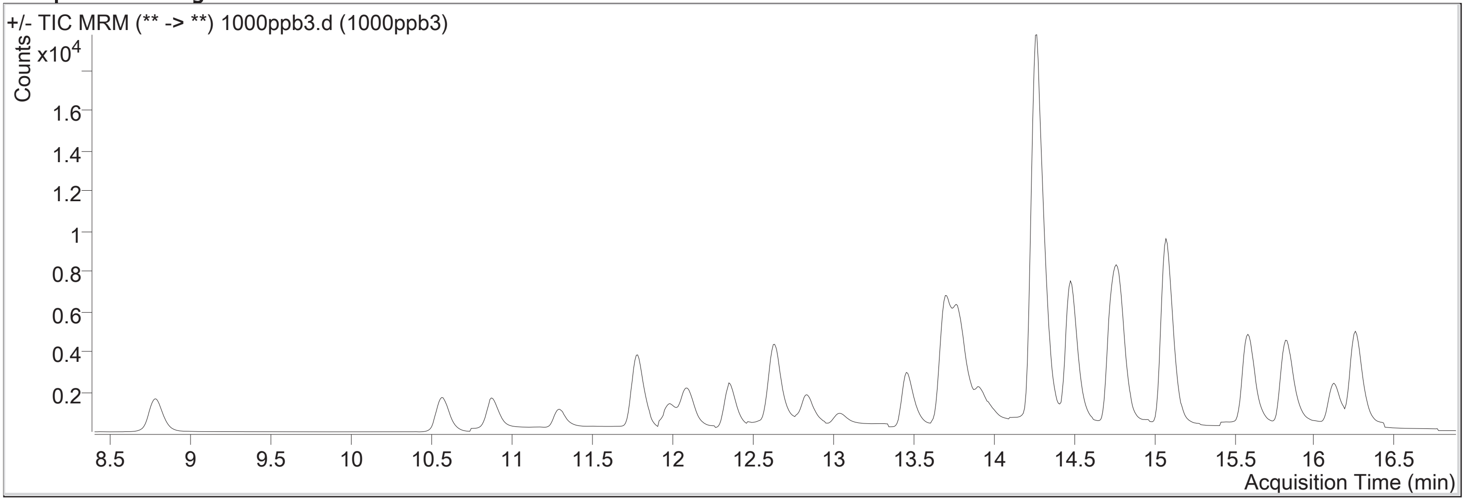

Compound	Transition	RT	Resp.	Final Conc	Units
Gallic acid	168.9 -> 125.0	8.783	10522	966.9094	ng/ml
Protocatechuic acid	152.9 -> 108.9	10.568	10291	983.9114	ng/ml
Pyrocatechol	109.0 -> 52.9	10.839	339	1099.9595	ng/ml
3,4-Dihydroxyphenylacetic acid	167.0 -> 123.0	10.879	7913	989.7819	ng/ml
(+)-Catechin	289.0 -> 245.0	11.302	3236	968.2655	ng/ml
2,5-Dihydroxybenzoic acid	152.9 -> 109.0	11.988	6313	998.1250	ng/ml
Chlorogenic acid	355.0 -> 163.0	11.793	20777	981.0432	ng/ml
3-Hydroxybenzoic acid	137.0 -> 93.0	12.845	4136	788.6899	ng/ml
4-Hydroxybenzoic acid	136.9 -> 93.1	12.097	10523	966.6949	ng/ml
(-)-Epicatechin	291.0 -> 139.1	12.361	7737	978.0745	ng/ml
Caffeic acid	179.0 -> 135.0	12.641	22884	958.7847	ng/ml
Syringic acid	196.9 -> 181.9	12.757	705	986.9296	ng/ml
Vanillin	151.0 -> 136.0	13.053	3356	985.9814	ng/ml
Verbascoside	623.0 -> 160.8	13.459	9947	984.0769	ng/ml
Taxifolin	303.0 -> 285.1	13.703	25705	979.0896	ng/ml
p-Coumaric acid	162.9 -> 119.0	13.792	25426	983.2819	ng/ml
Sinapic acid	222.9 -> 207.9	13.864	2993	947.8402	ng/ml
Ferulic acid	193.0 -> 134.0	13.925	5032	965.8144	ng/ml
Luteolin 7-glucoside	447.1 -> 285.0	14.264	93226	990.5837	ng/ml
Hesperidin	611.1 -> 303.0	14.310	8209	1000.3602	ng/ml
Hyperoside	465.1 -> 303.1	14.487	19141	972.7189	ng/ml
Rosmarinic acid	359.0 -> 160.9	14.481	12204	974.6178	ng/ml
Apigenin 7-glucoside	433.1 -> 271.0	14.747	32915	977.6059	ng/ml
Pinoresinol	357.0 -> 151.0	14.976	265	1018.8690	ng/ml
2-Hydroxycinnamic acid	162.9 -> 119.1	14.794	20640	971.3714	ng/ml
Eriodictyol	287.0 -> 151.0	15.078	24746	963.6427	ng/ml
Quercetin	301.0 -> 151.0	15.586	22333	969.0342	ng/ml
Luteolin	287.0 -> 153.1	15.825	15823	994.6560	ng/ml
Kaempferol	285.0 -> 229.1	16.121	4089	987.7973	ng/ml

Agilent Technologies

Compound	Transition	RT	Resp.	Final Conc	Units
Apigenin	271.0 -> 153.0	16.260	17898	962.9949	ng/ml

Gallic acid

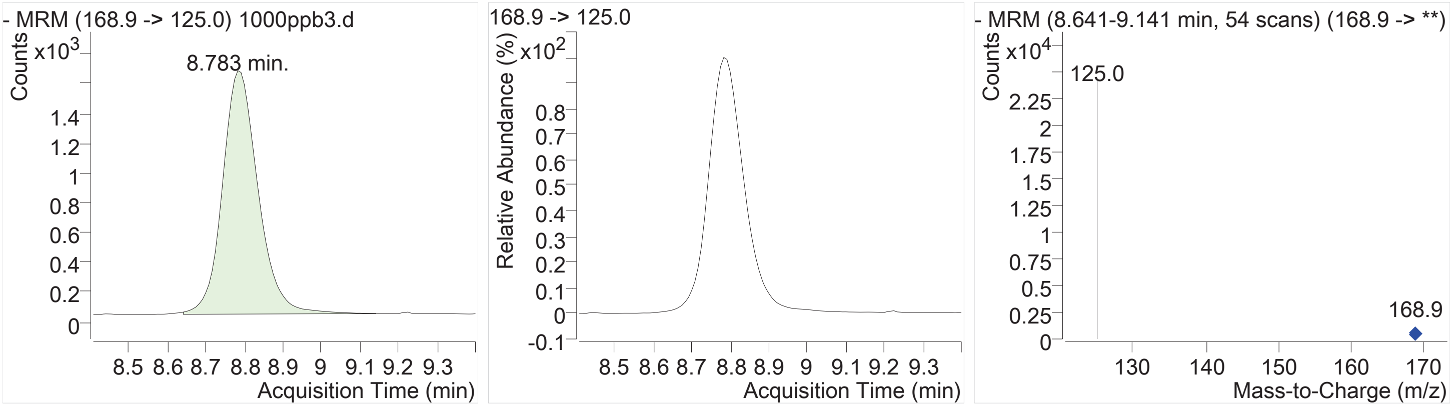

Protocatechuic acid

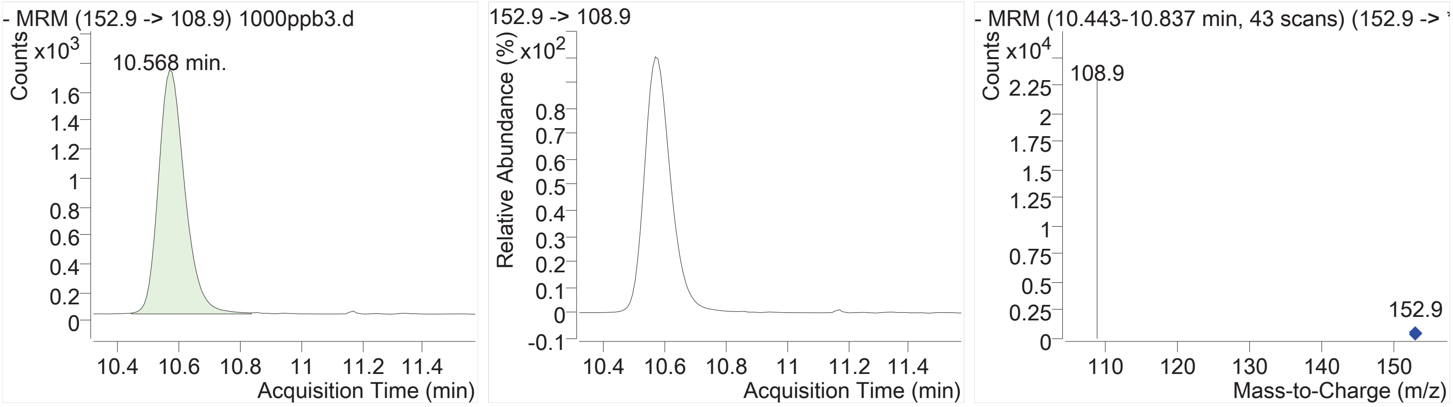

Pyrocatechol

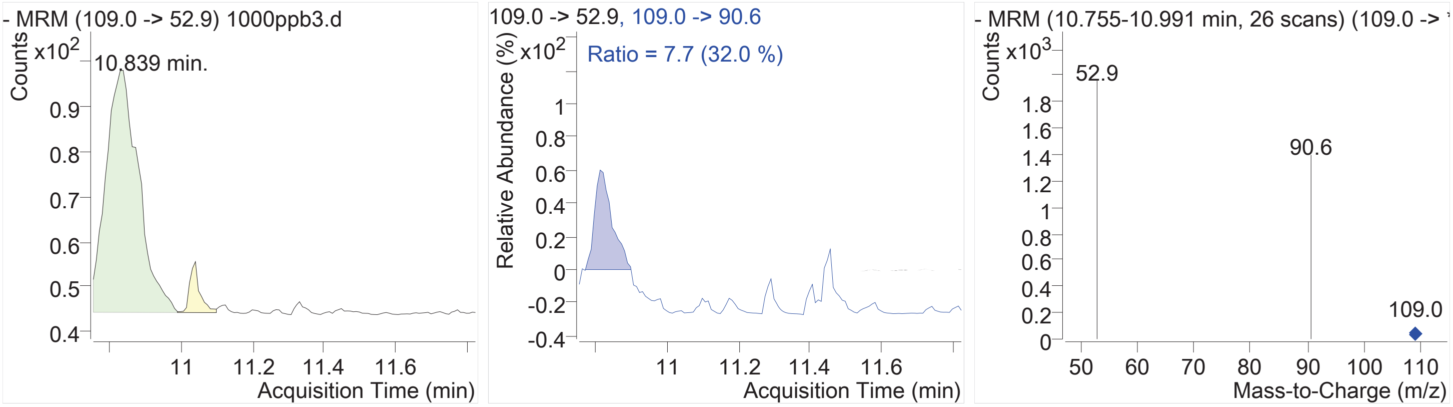

3,4-Dihydroxyphenylacetic acid

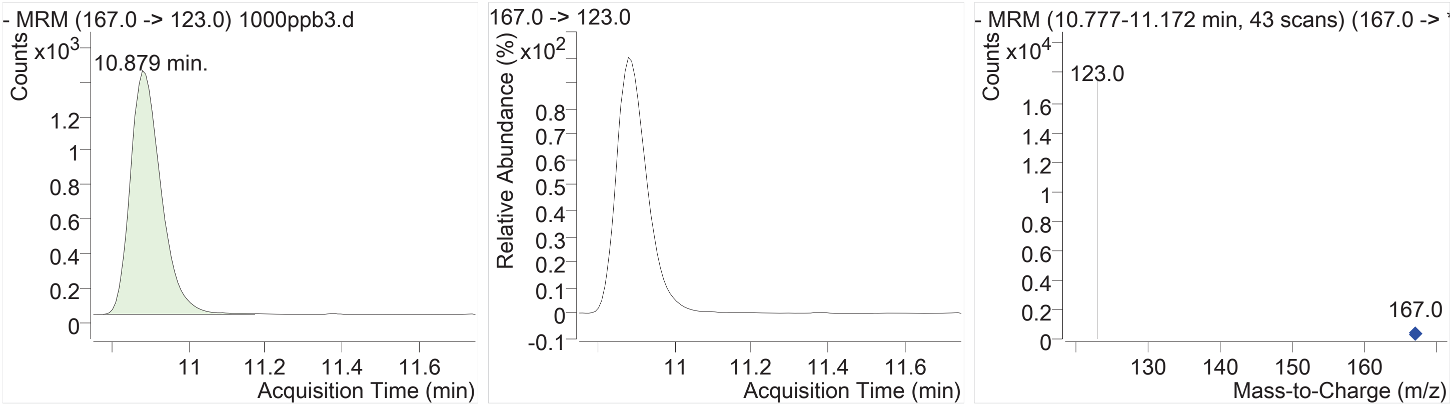

(+)-Catechin

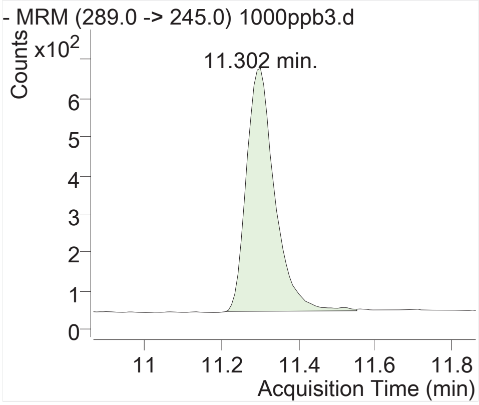

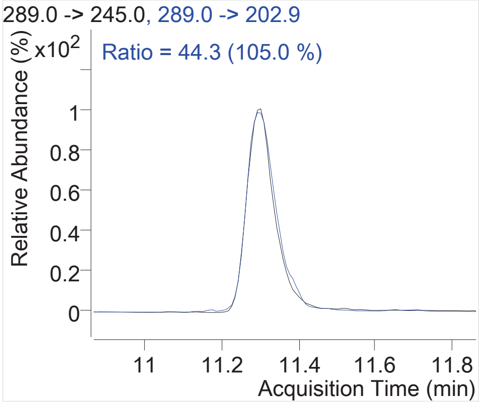

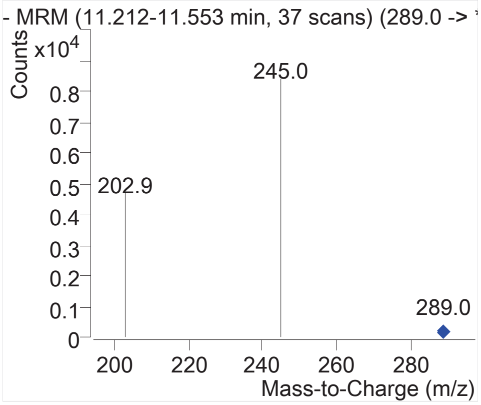

2,5-Dihydroxybenzoic acid

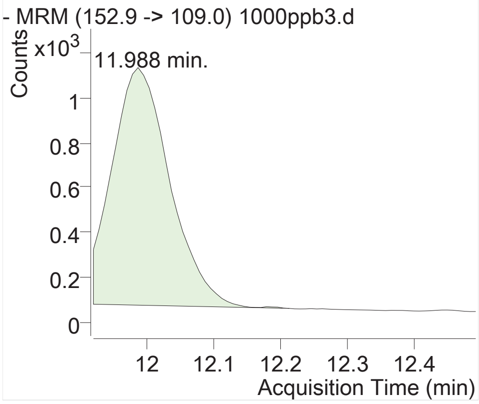

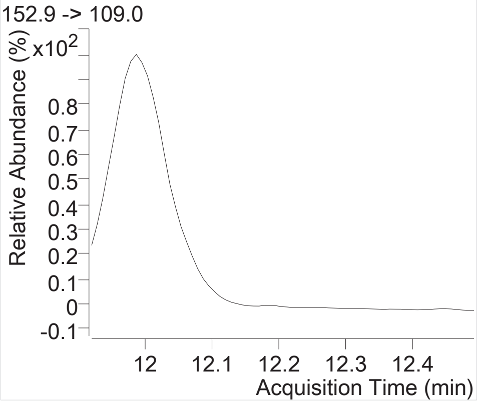

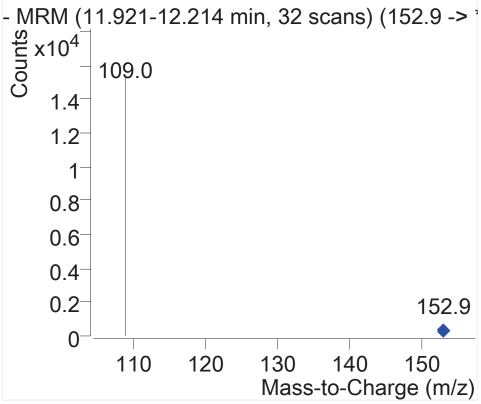

Chlorogenic acid

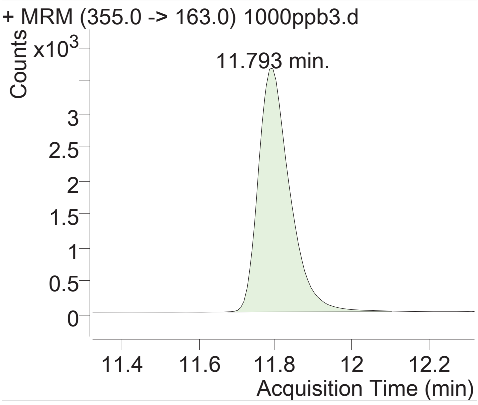

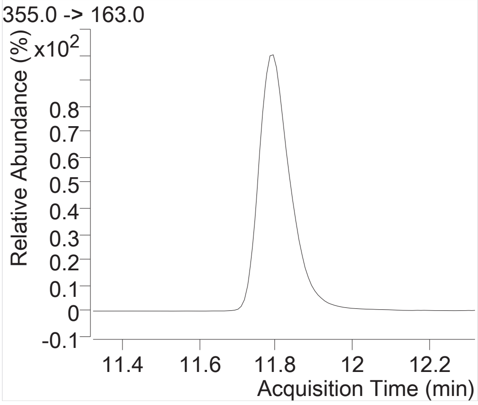

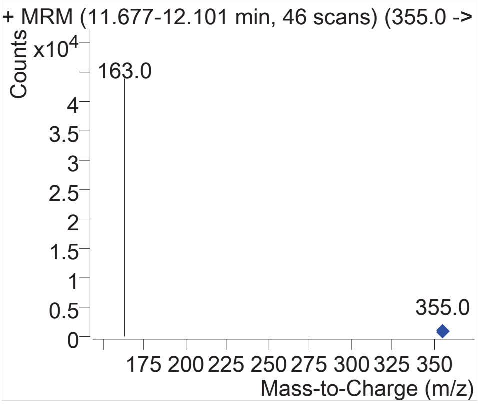

3-Hydroxybenzoic acid

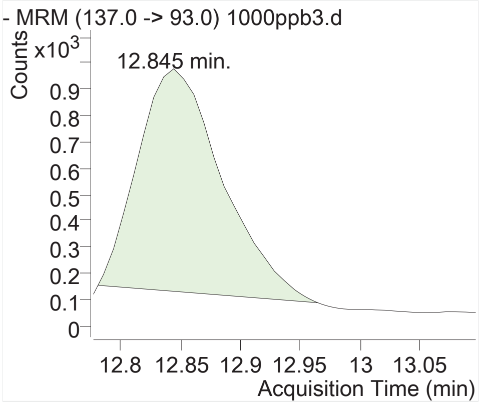

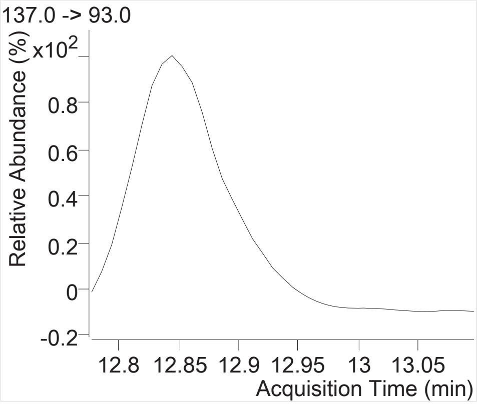

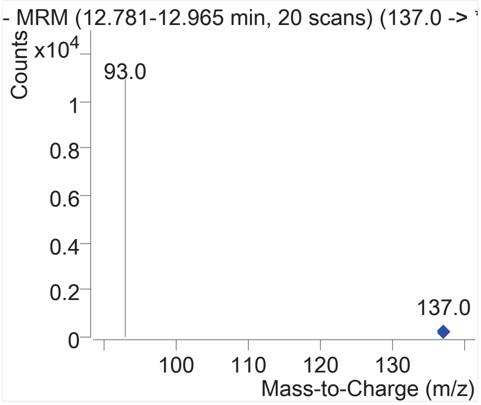

4-Hydroxybenzoic acid

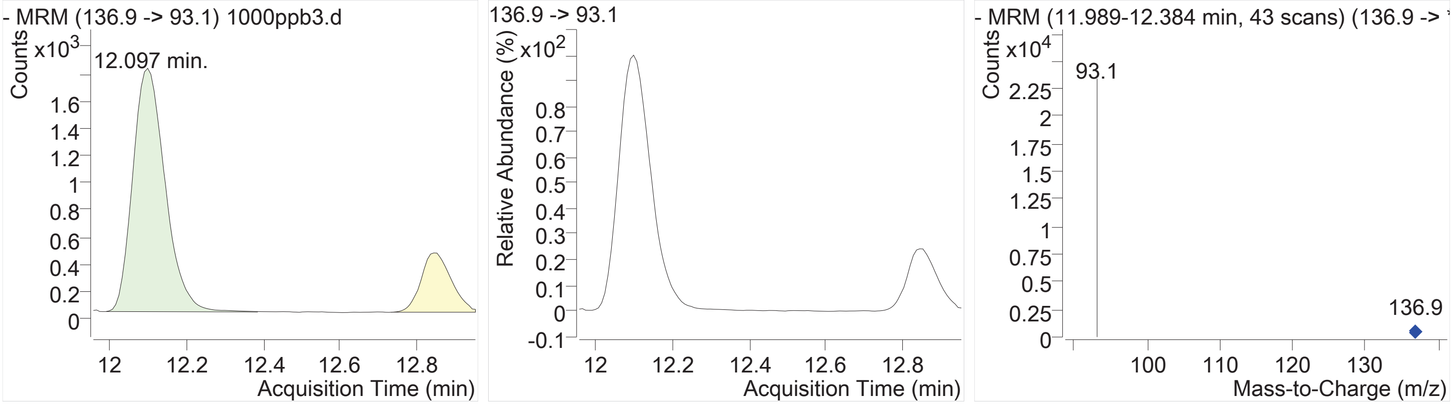

(-)-Epicatechin

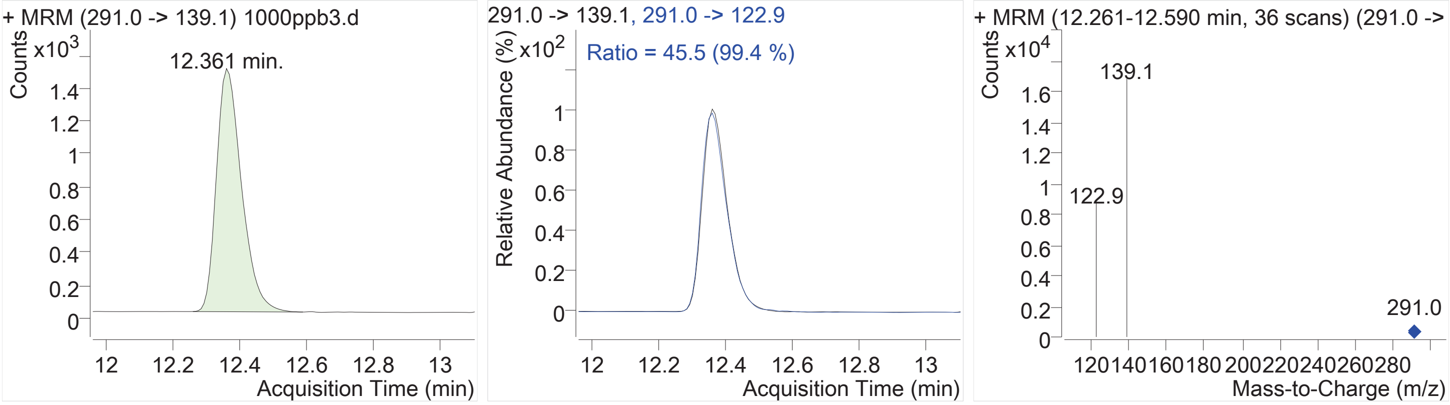

Caffeic acid

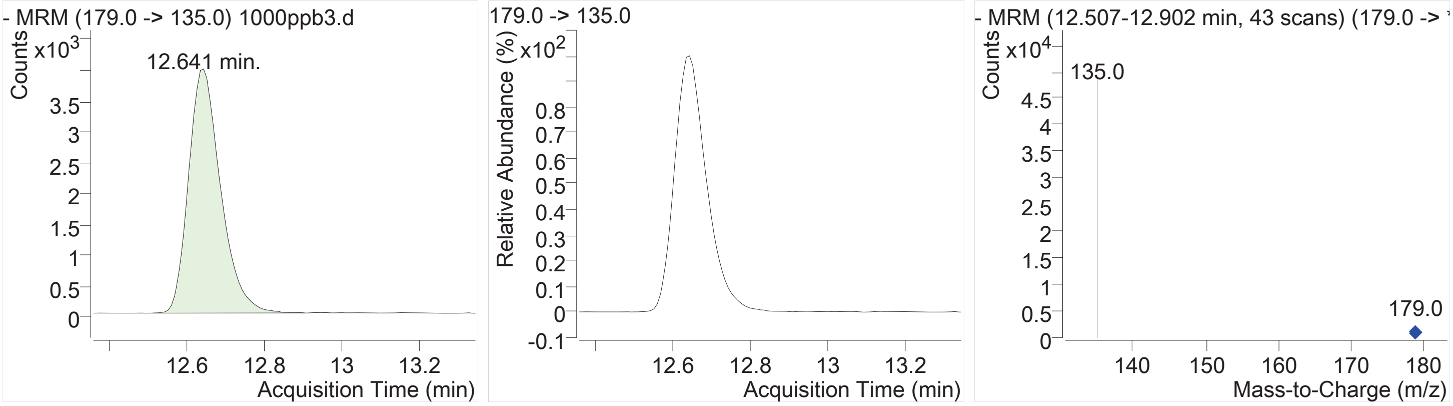

Syringic acid

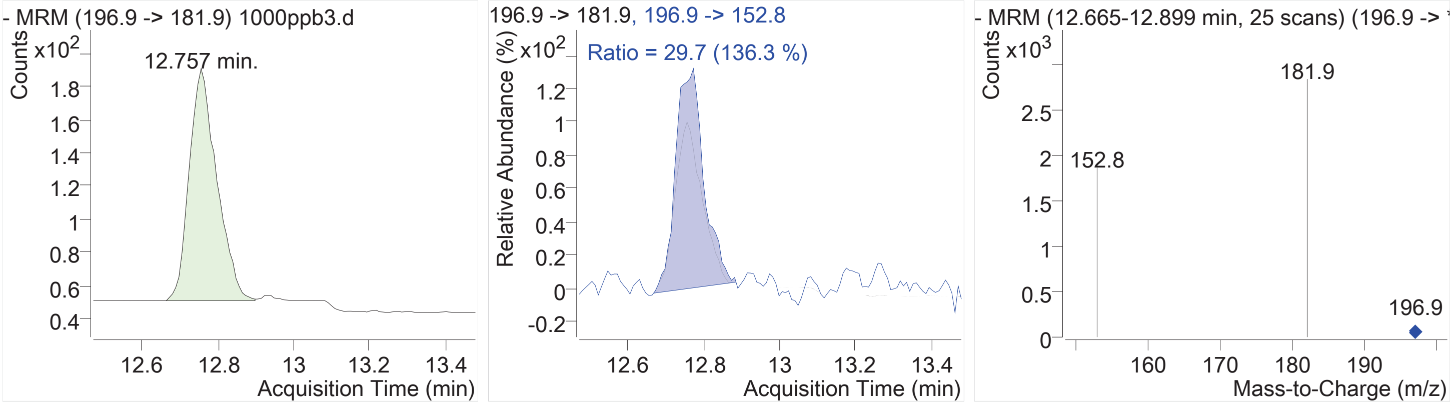

Vanillin

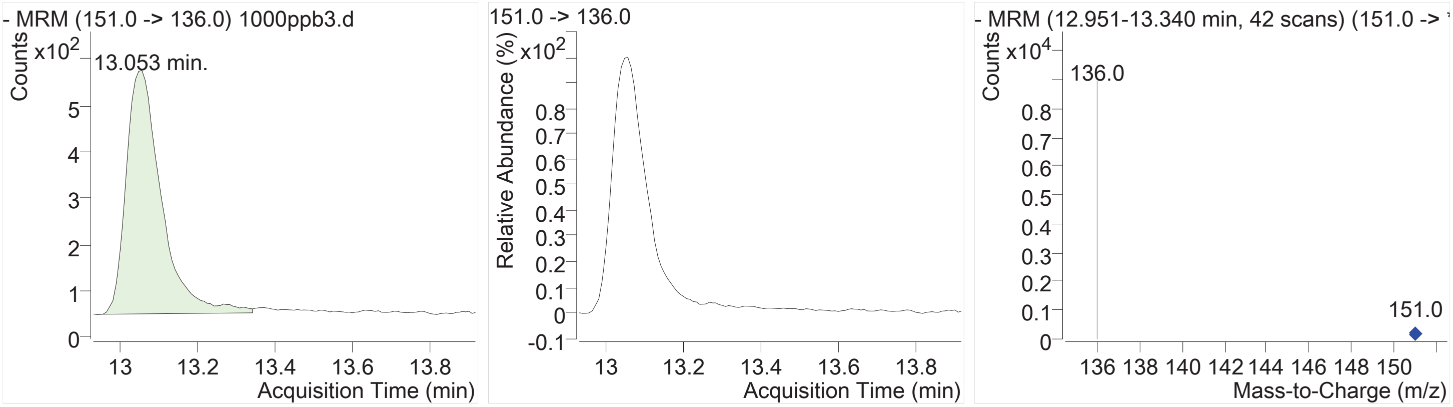

Verbascoside

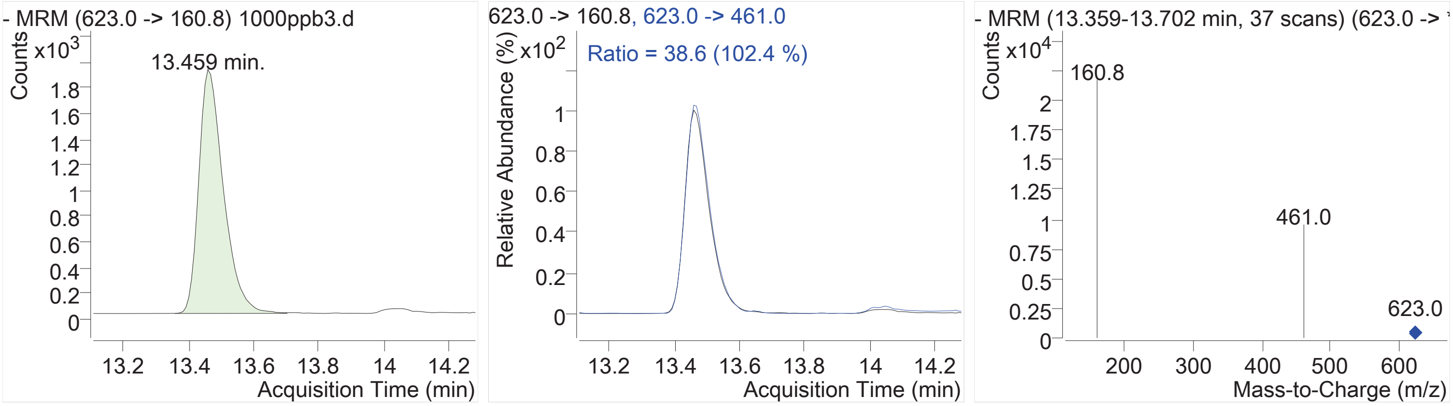

Taxifolin

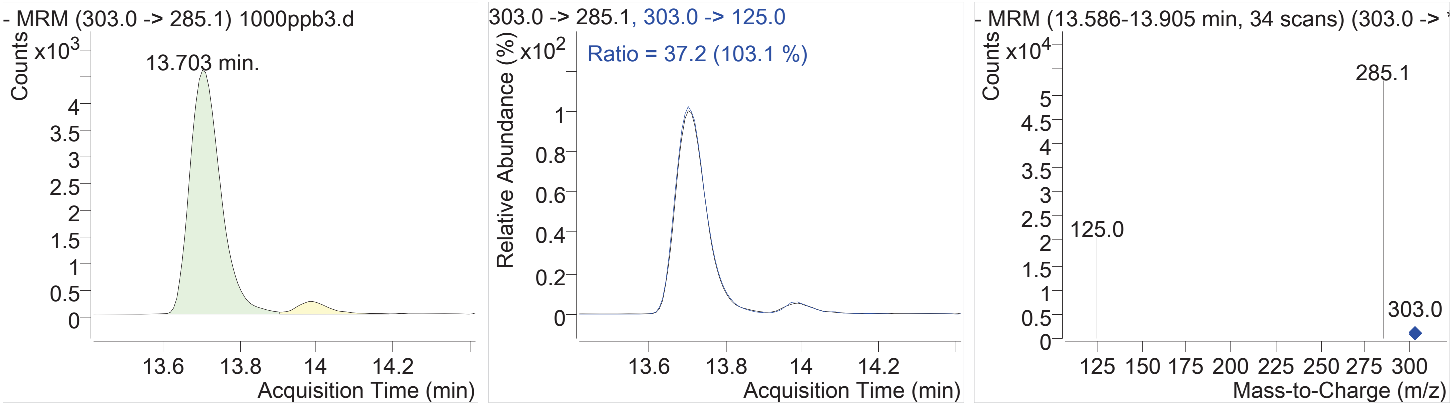

p-Coumaric acid

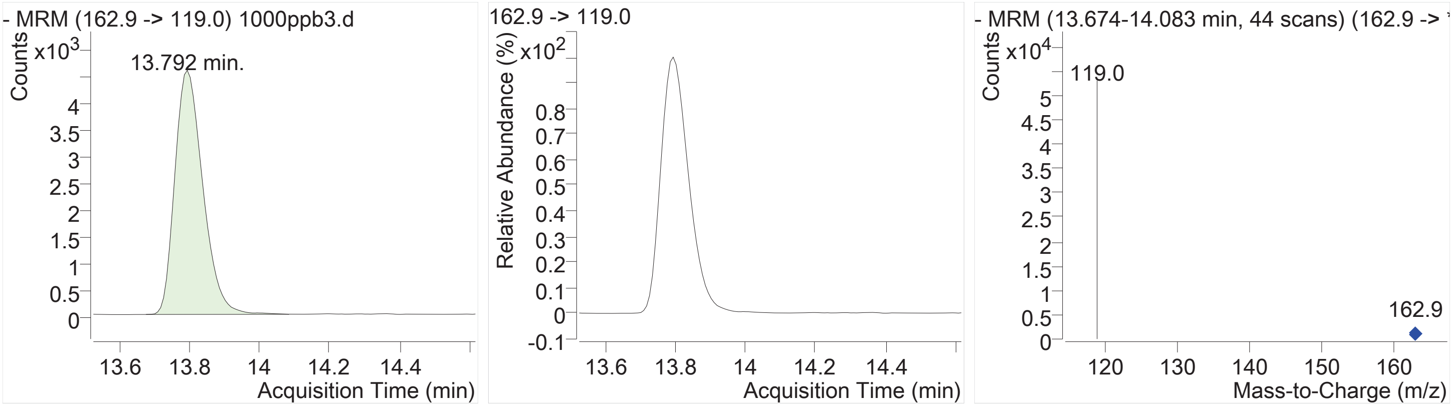

Agilent Technologies

Sinapic acid

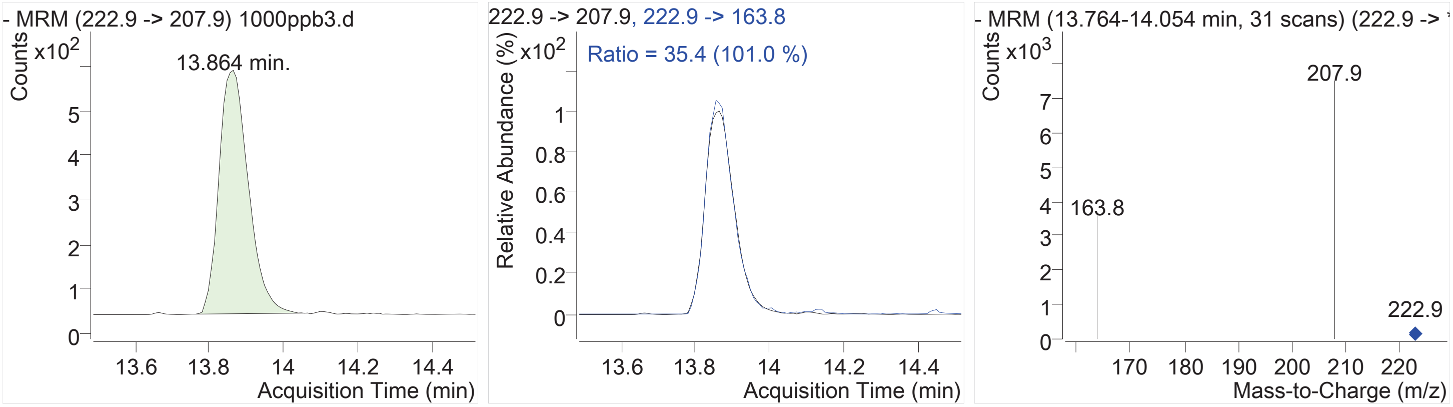

Ferulic acid

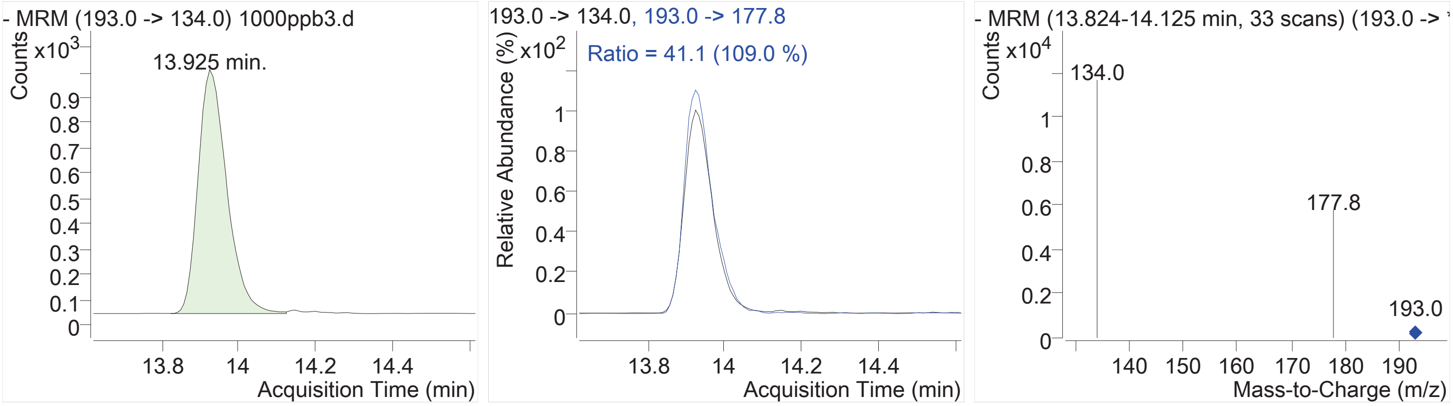

Luteolin 7-glucoside

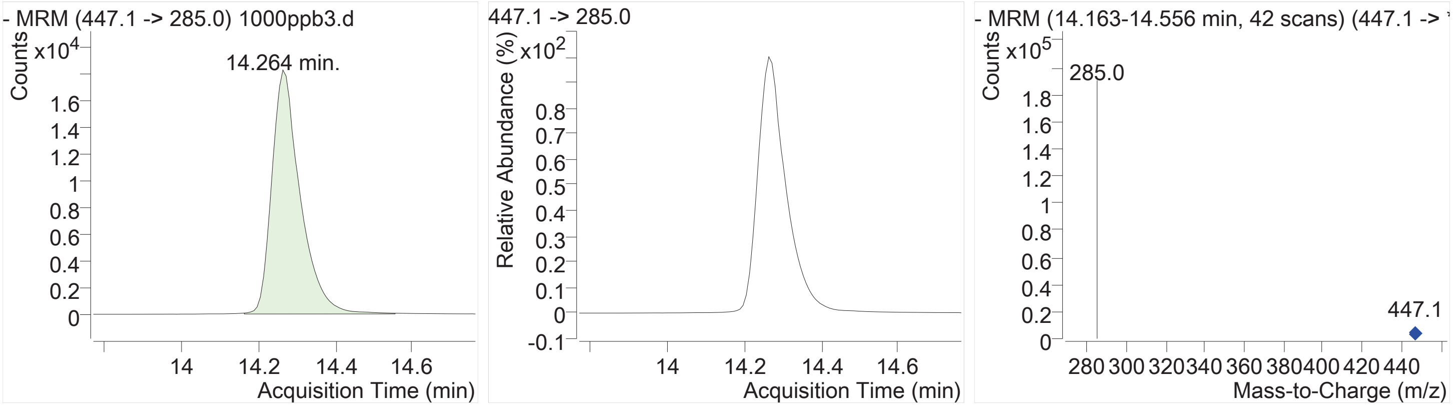

Hesperidin

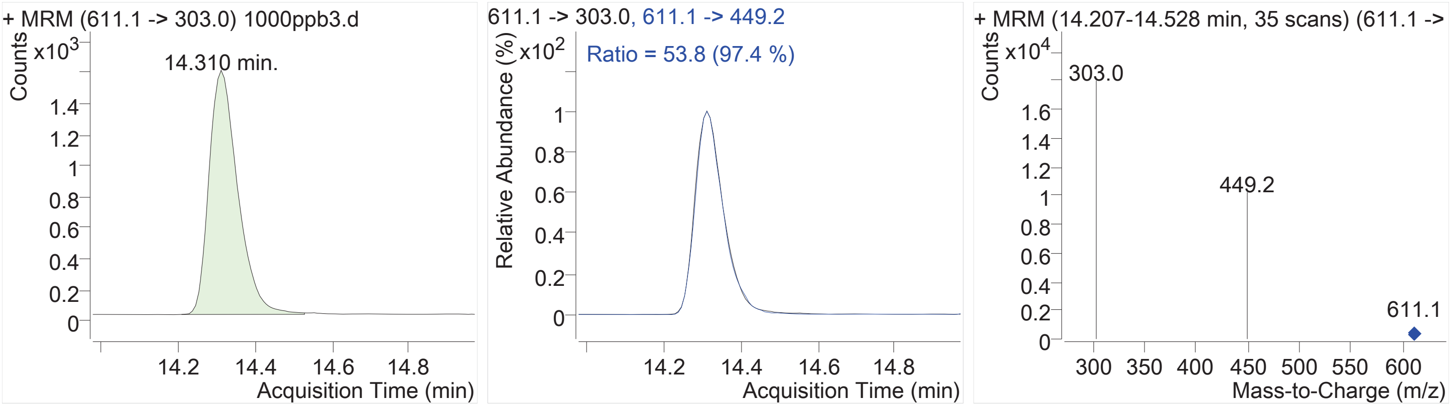

Hyperoside

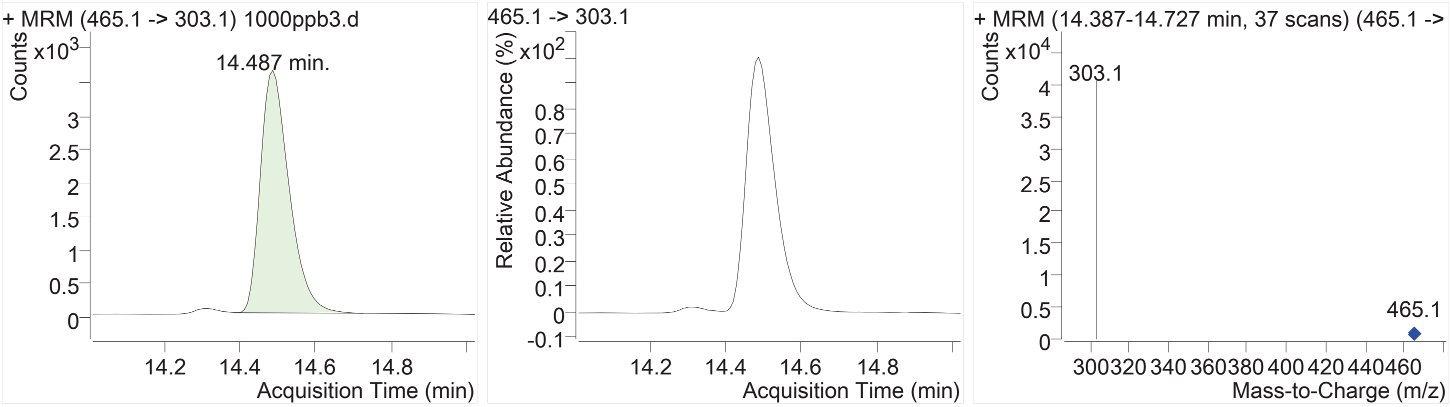

Rosmarinic acid

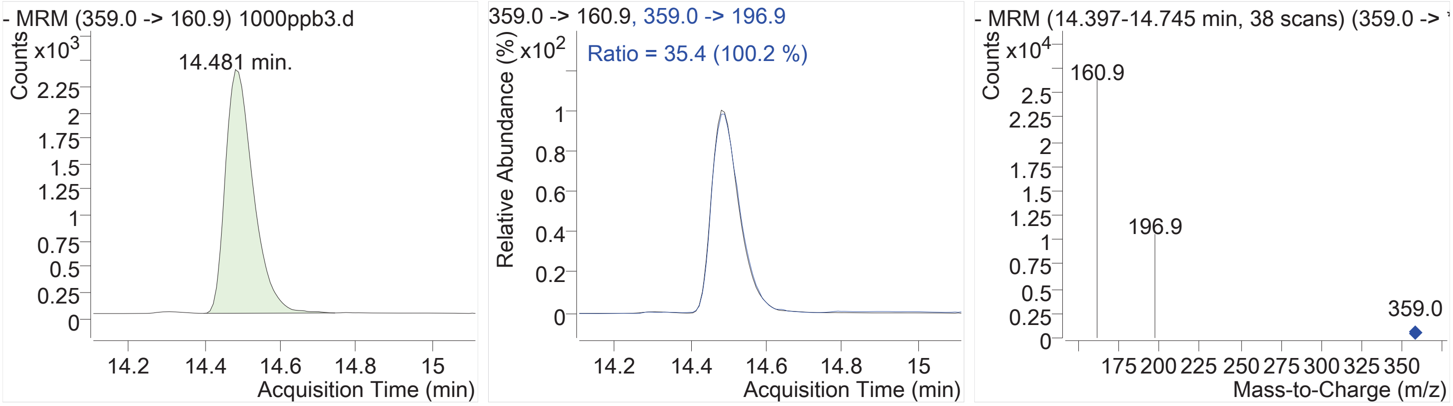

Apigenin 7-glucoside

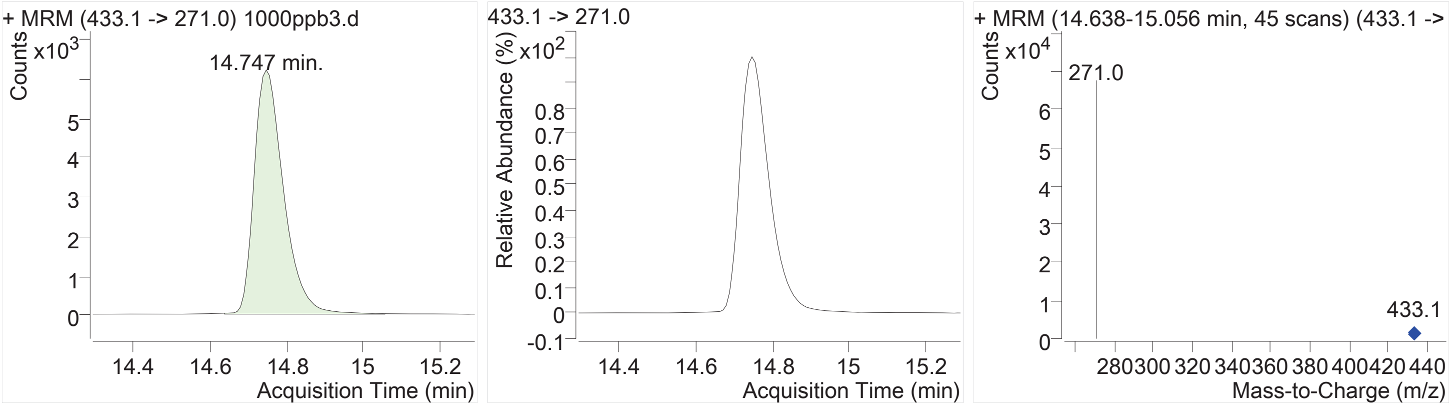

Pinoresinol

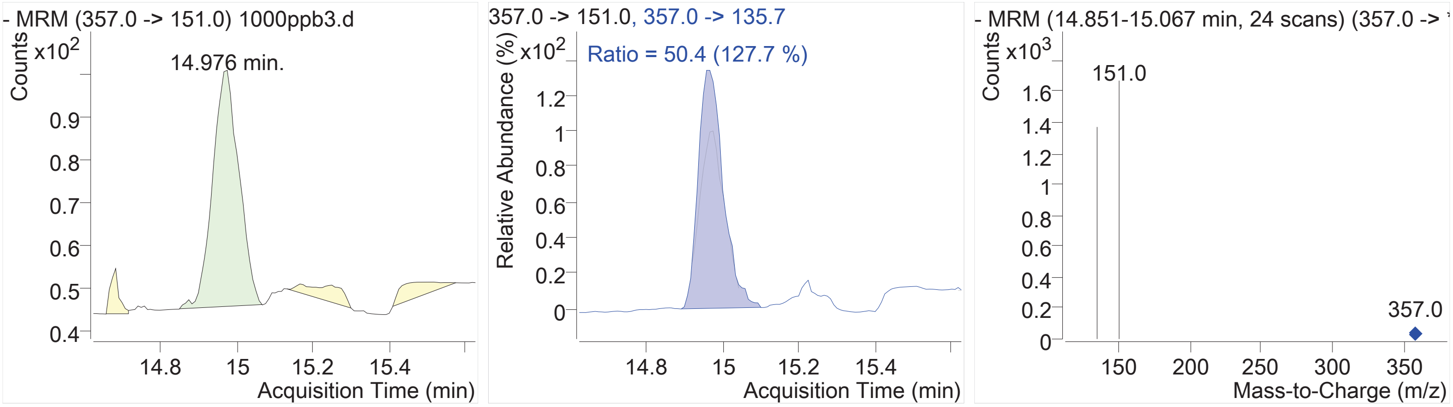

2-Hydroxycinnamic acid

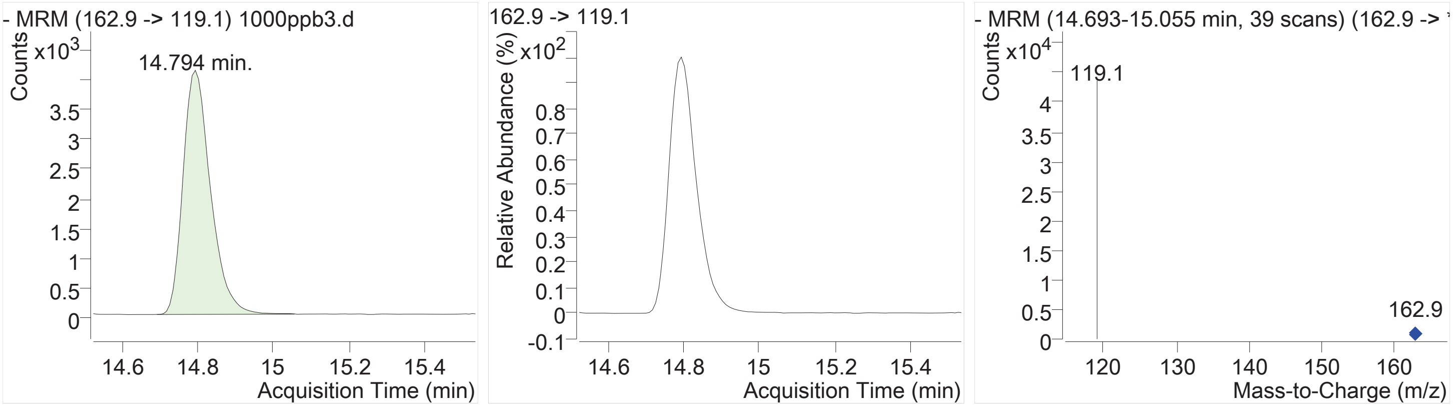

Eriodictyol

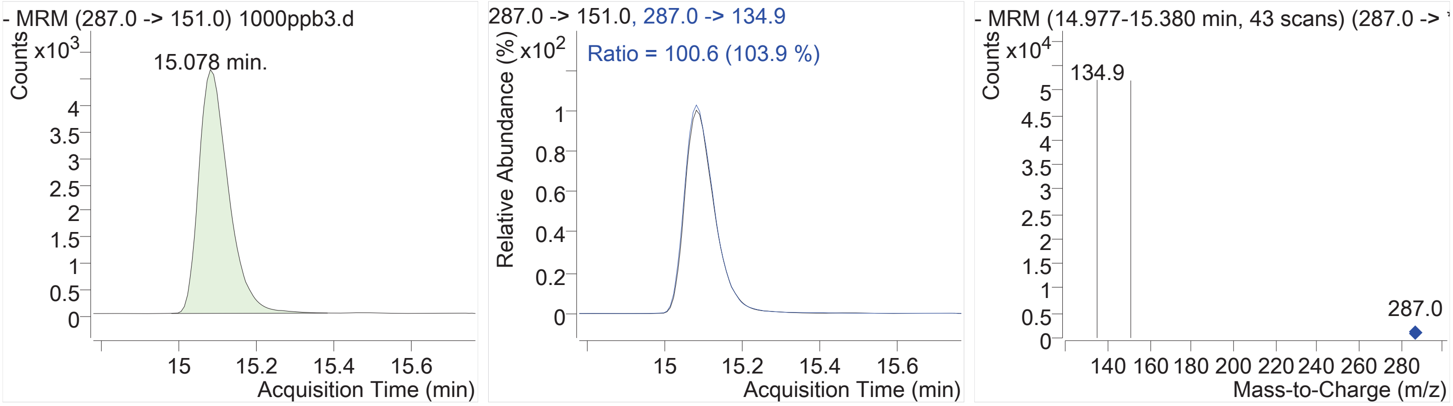

Quercetin

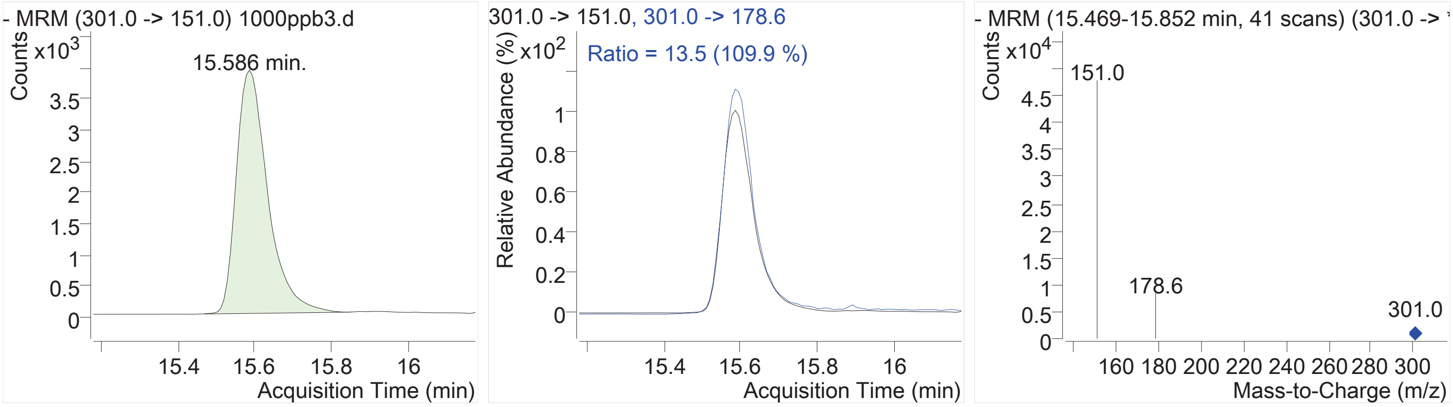

Luteolin

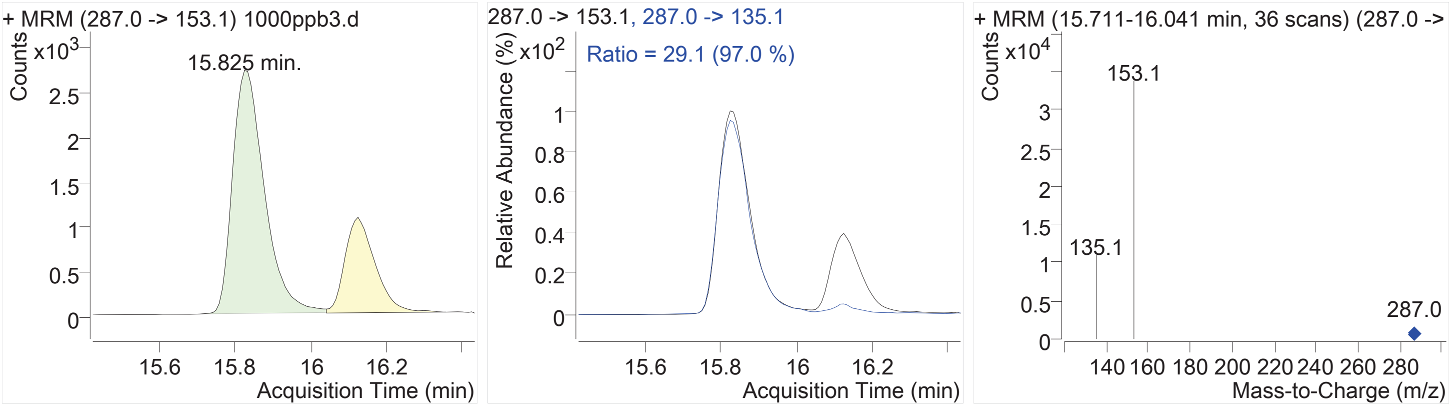

Kaempferol

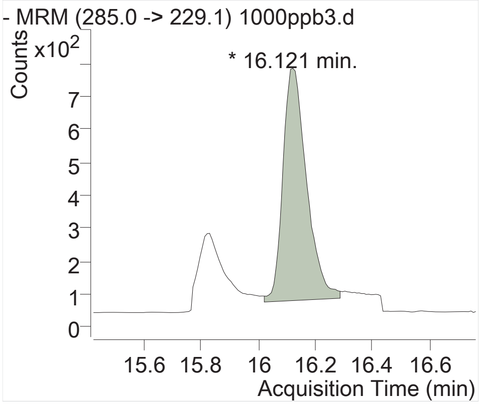

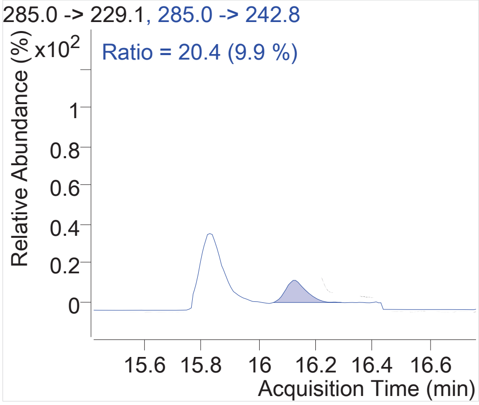

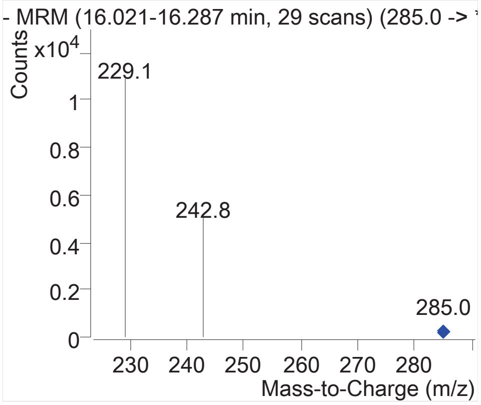

Apigenin

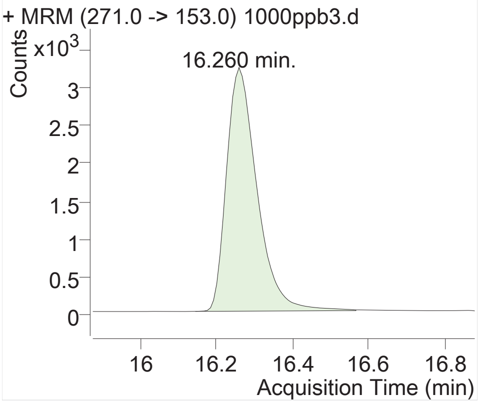

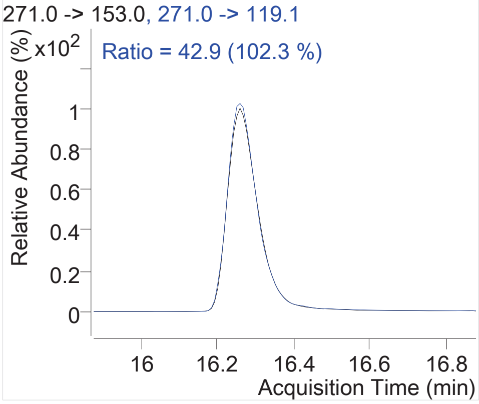

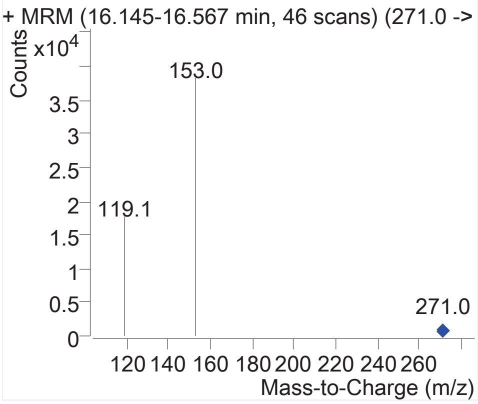

Quantitative Analysis Complete Report

**Batch Path**	D:\MassHunter\Data\2022ekim\061022cengizhoca\QuantResults\071022.batch.bin		
**Analysis Time**	10/11/2022 1:33:26 PM	**Analyst Name**	Defam-PC\admin
**Report Time**	10/11/2022 1:36:48 PM	**Reporter Name**	admin
**Last Calib Update**	10/11/2022 1:33:17 PM	**Batch State**	Processed
**Quant Batch Version**	B.07.01	**Quant Report Version**	B.07.01

**Acq. Time**	10/7/2022 1:36:19 PM	**Data File**	22-15A-1.d
**Sample Type**	Sample	**Sample Name**	22-15A-1
**Dilution**	1	**Acq. Method**	FENOLIK_DMRM2021-31bilesen.m

Sample Chromatogram

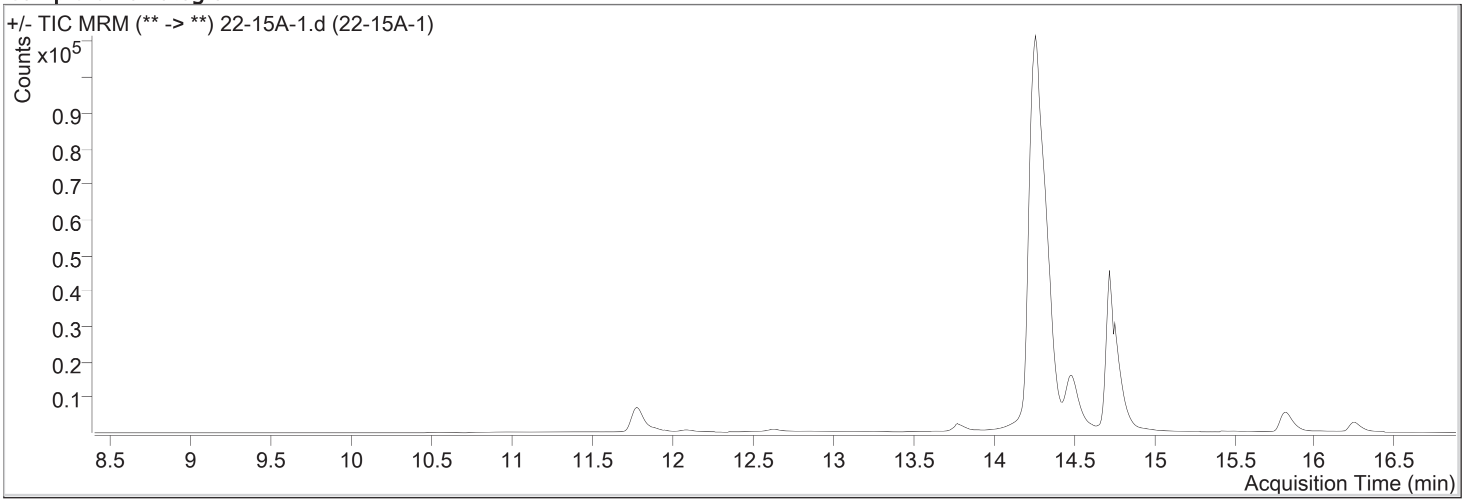

Compound	Transition	RT	Resp.	Final Conc	Units
Gallic acid	168.9 -> 125.0	8.800	6	ND	ng/ml
Protocatechuic acid	152.9 -> 108.9	10.568	796	65.1982	ng/ml
Pyrocatechol	109.0 -> 52.9	10.814	0	ND	ng/ml
3,4-Dihydroxyphenylacetic acid	167.0 -> 123.0	10.996	17	ND	ng/ml
(+)-Catechin	289.0 -> 245.0	11.236	1	ND	ng/ml
2,5-Dihydroxybenzoic acid	152.9 -> 109.0	12.004	90	14.7663	ng/ml
Chlorogenic acid	355.0 -> 163.0	11.785	43941	2068.2917	ng/ml
3-Hydroxybenzoic acid	137.0 -> 93.0	12.853	32	21.0380	ng/ml
4-Hydroxybenzoic acid	136.9 -> 93.1	12.089	2766	241.3607	ng/ml
(-)-Epicatechin	291.0 -> 139.1	12.293	3	3.4232	ng/ml
Caffeic acid	179.0 -> 135.0	12.633	3333	114.9407	ng/ml
Syringic acid	196.9 -> 181.9	12.757	146	206.7221	ng/ml
Vanillin	151.0 -> 136.0	13.045	211	42.1012	ng/ml
Verbascoside	623.0 -> 160.8	13.292	3	ND	ng/ml
Taxifolin	303.0 -> 285.1	13.712	5	ND	ng/ml
p-Coumaric acid	162.9 -> 119.0	13.792	9368	356.3704	ng/ml
Sinapic acid	222.9 -> 207.9	13.856	21	10.8654	ng/ml
Ferulic acid	193.0 -> 134.0	13.934	252	48.5086	ng/ml
Luteolin 7-glucoside	447.1 -> 285.0	14.264	844830	9248.0826	ng/ml
Hesperidin	611.1 -> 303.0	14.461	6147	750.1395	ng/ml
Hyperoside	465.1 -> 303.1	14.504	27466	1394.0576	ng/ml
Rosmarinic acid	359.0 -> 160.9	14.481	23495	1877.5640	ng/ml
Apigenin 7-glucoside	433.1 -> 271.0	14.730	209380	6225.7884	ng/ml
Pinoresinol	357.0 -> 151.0	14.959	480	1866.0389	ng/ml
2-Hydroxycinnamic acid	162.9 -> 119.1	14.987	12	ND	ng/ml
Eriodictyol	287.0 -> 151.0	15.078	110	ND	ng/ml
Quercetin	301.0 -> 151.0	15.586	23	ND	ng/ml
Luteolin	287.0 -> 153.1	15.825	20541	1292.7200	ng/ml
Kaempferol	285.0 -> 229.1	16.155	43	ND	ng/ml

Compound	Transition	RT	Resp.	Final Conc	Units
Apigenin	271.0 -> 153.0	16.251	9842	526.8651	ng/ml

Gallic acid

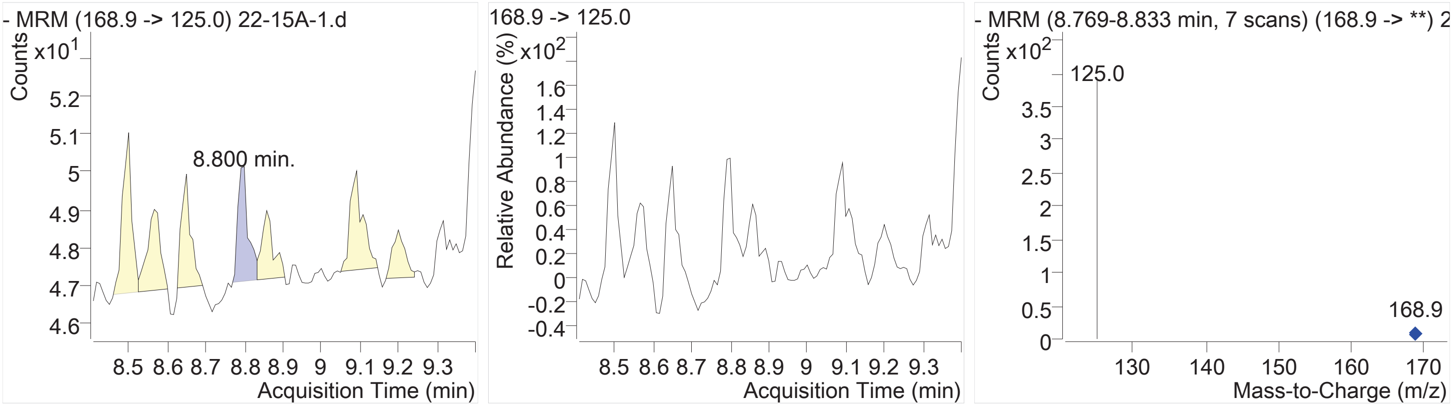

Protocatechuic acid

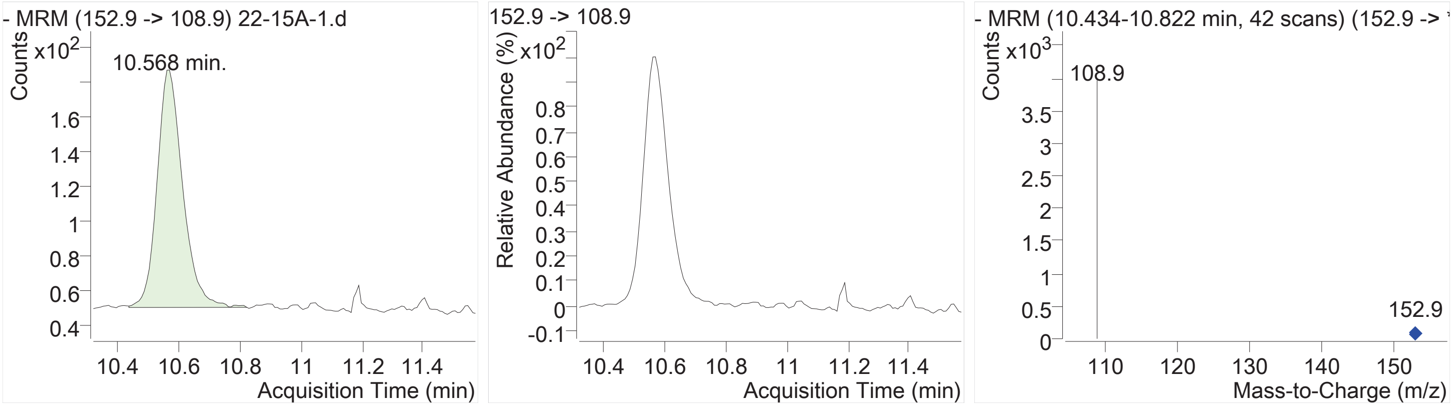

Pyrocatechol

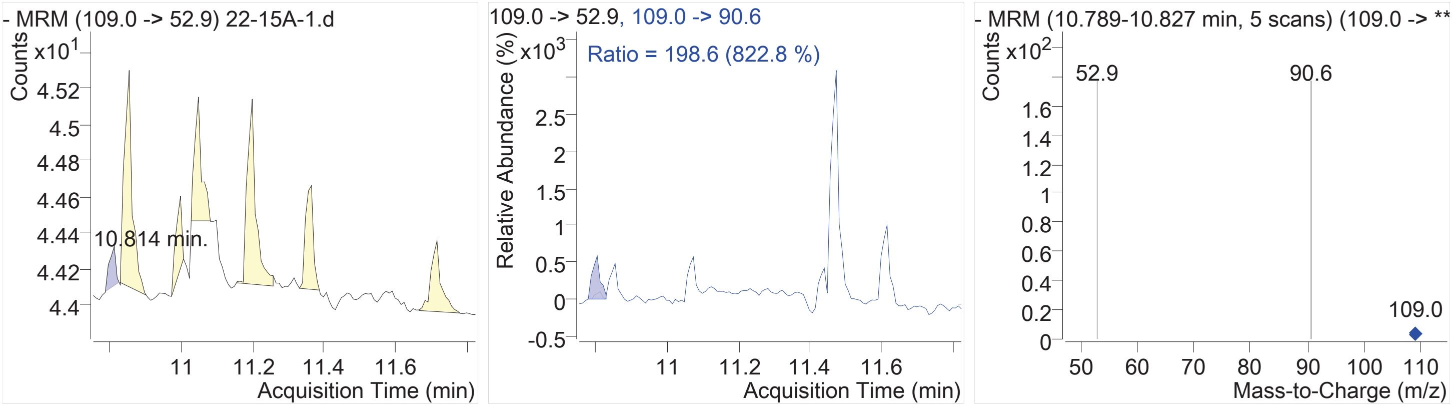

3,4-Dihydroxyphenylacetic acid

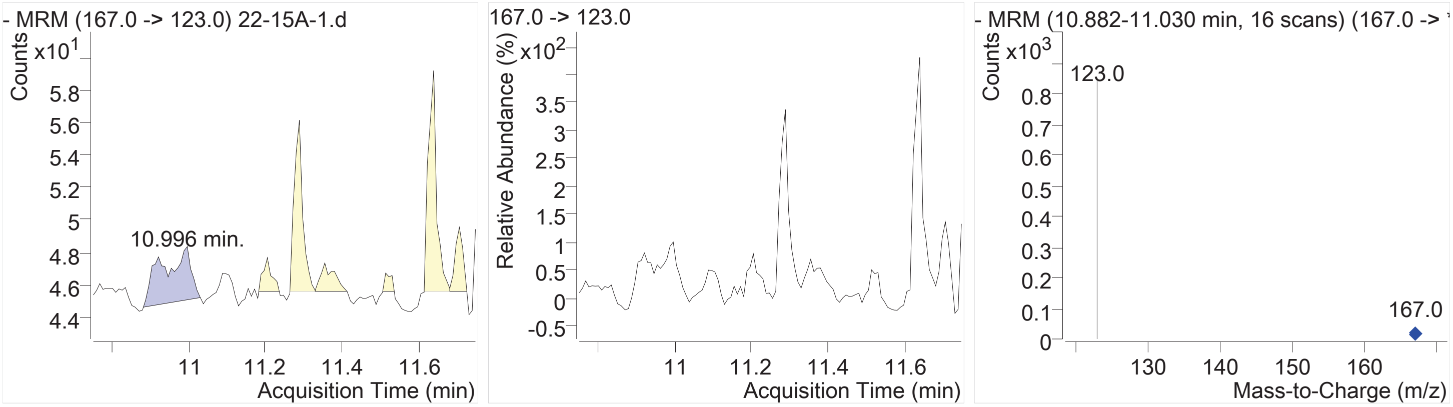

(+)-Catechin

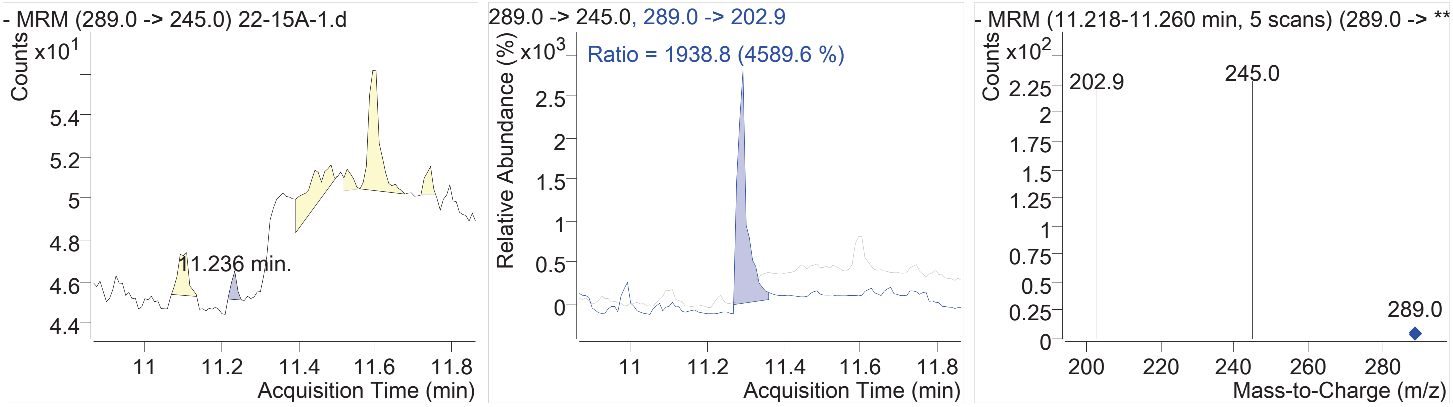

2,5-Dihydroxybenzoic acid

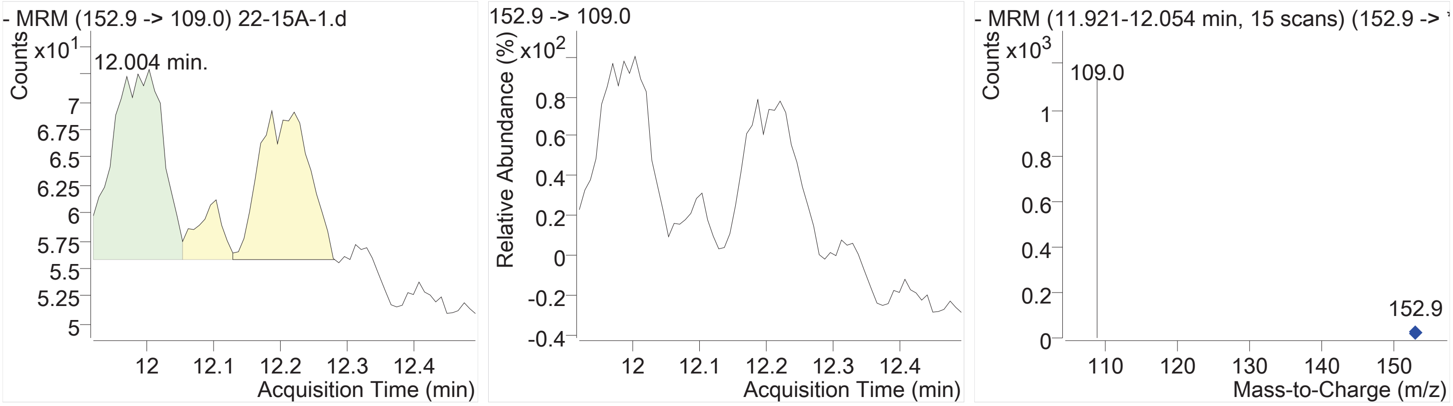

Chlorogenic acid

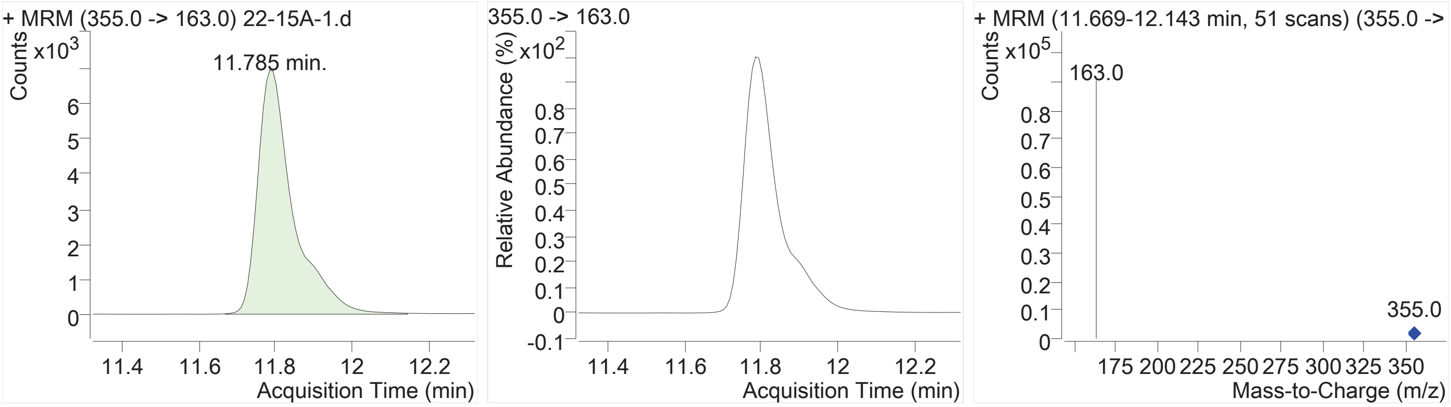

3-Hydroxybenzoic acid

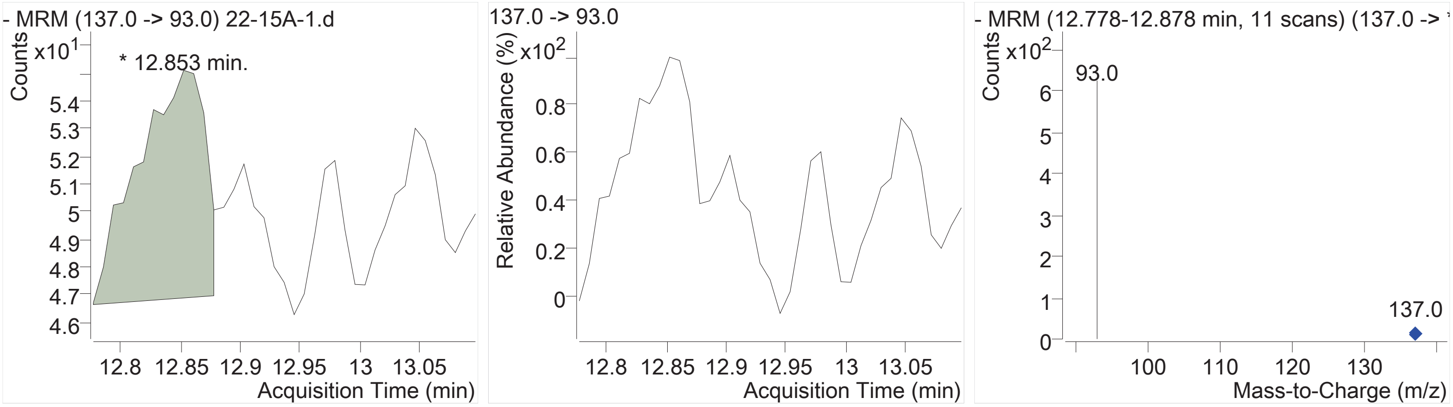

Agilent Technologies

4-Hydroxybenzoic acid

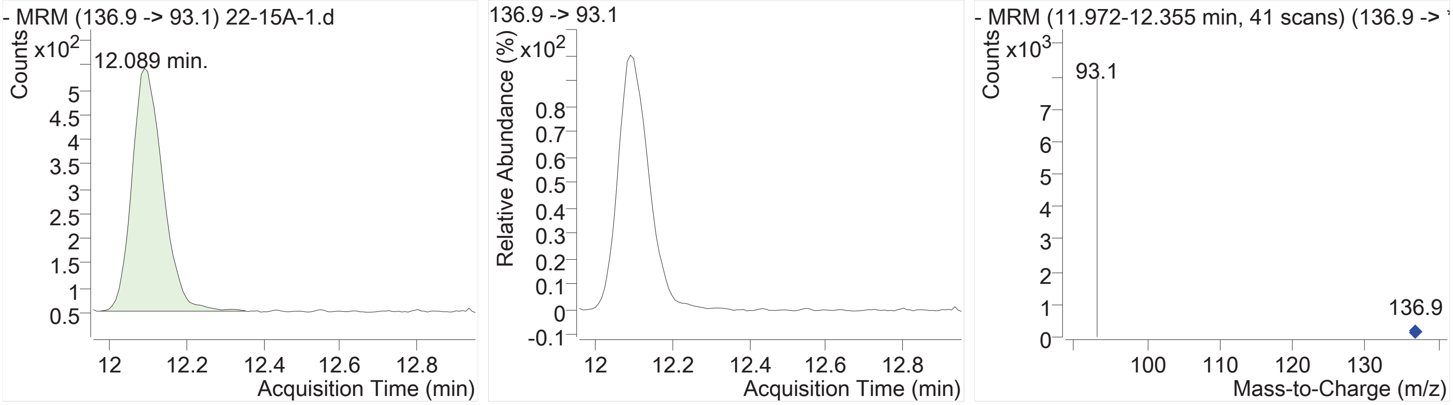

(-)-Epicatechin

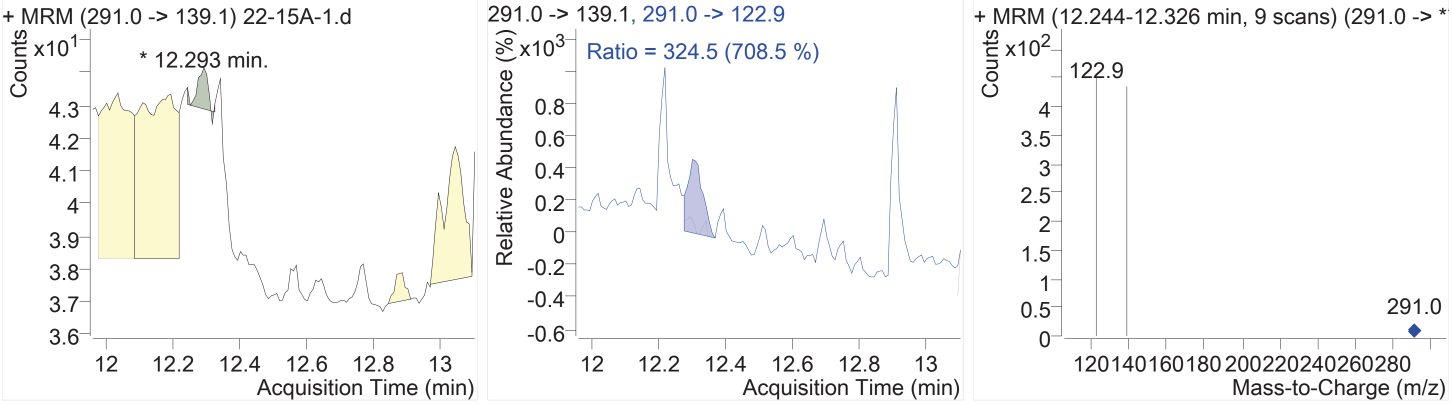

Caffeic acid

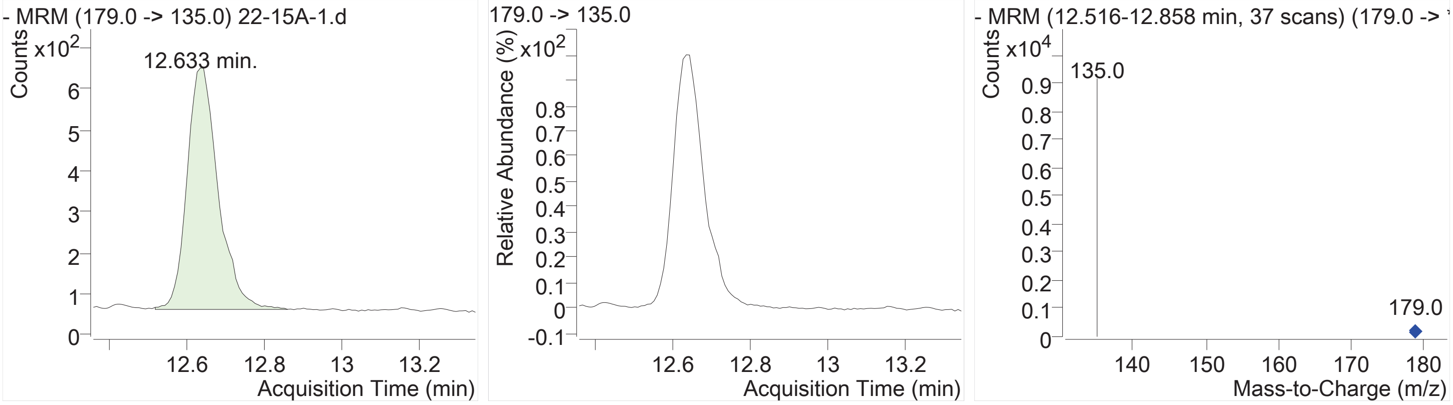

Syringic acid

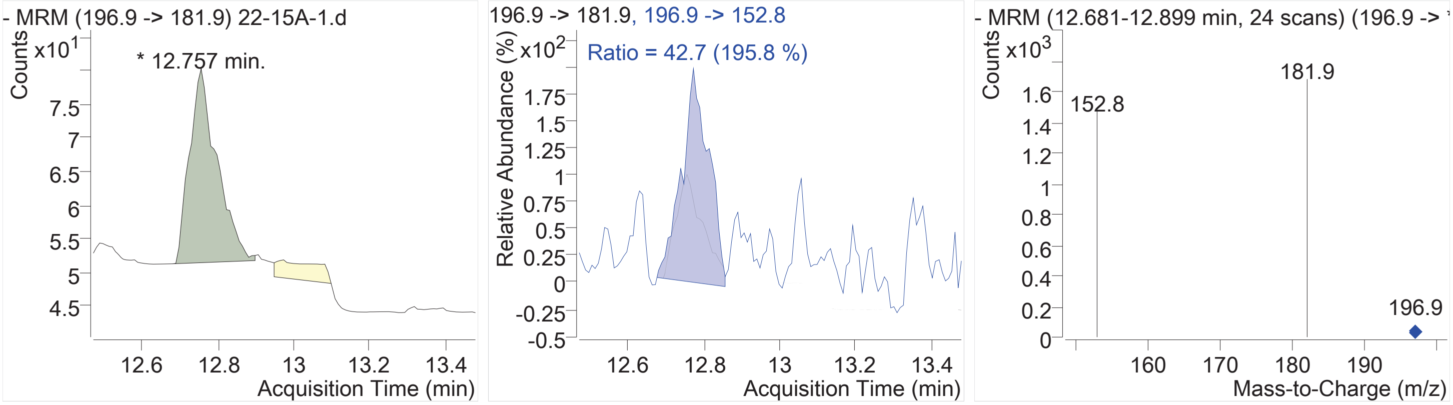

Vanillin

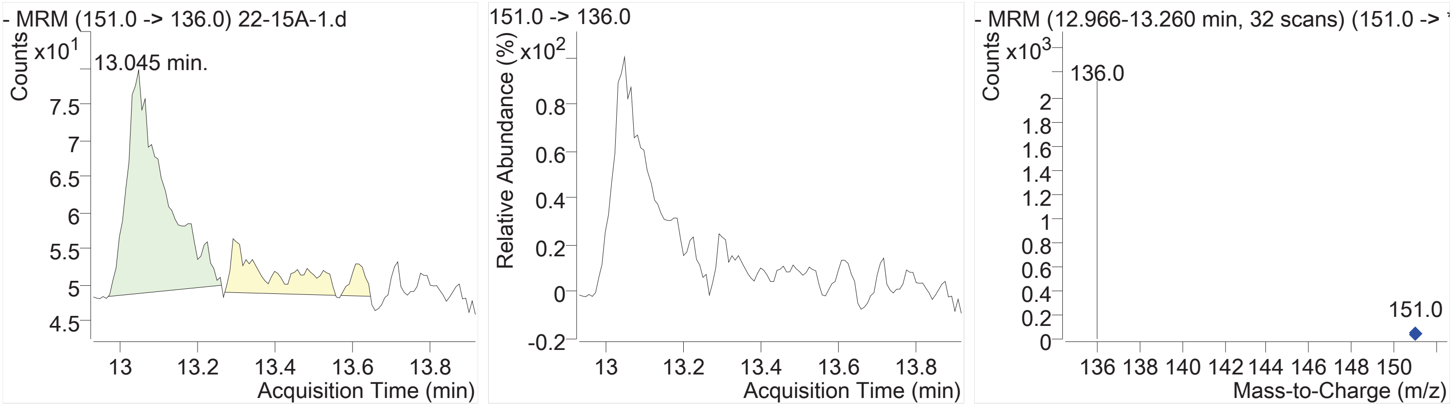

Verbascoside

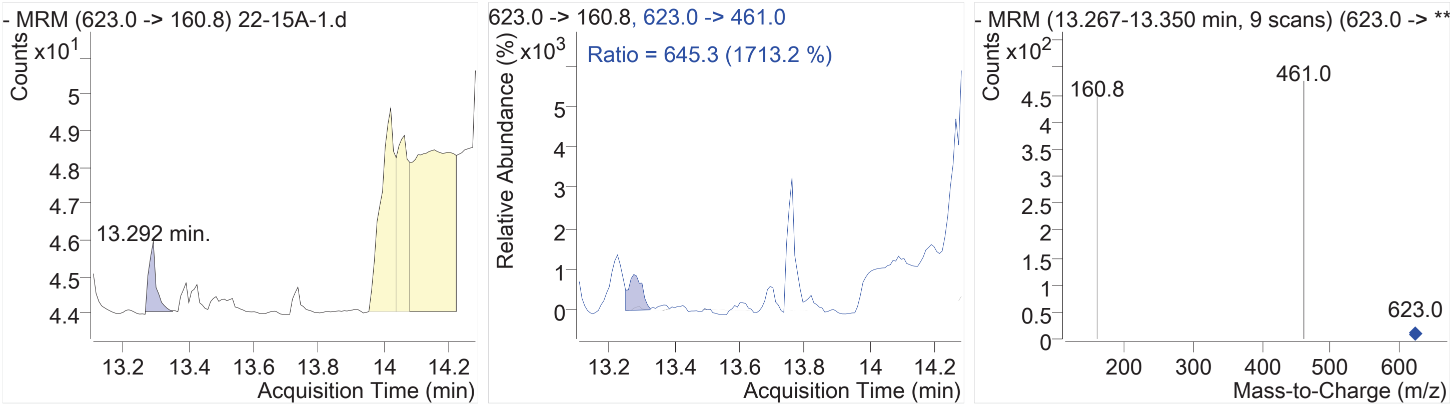

Taxifolin

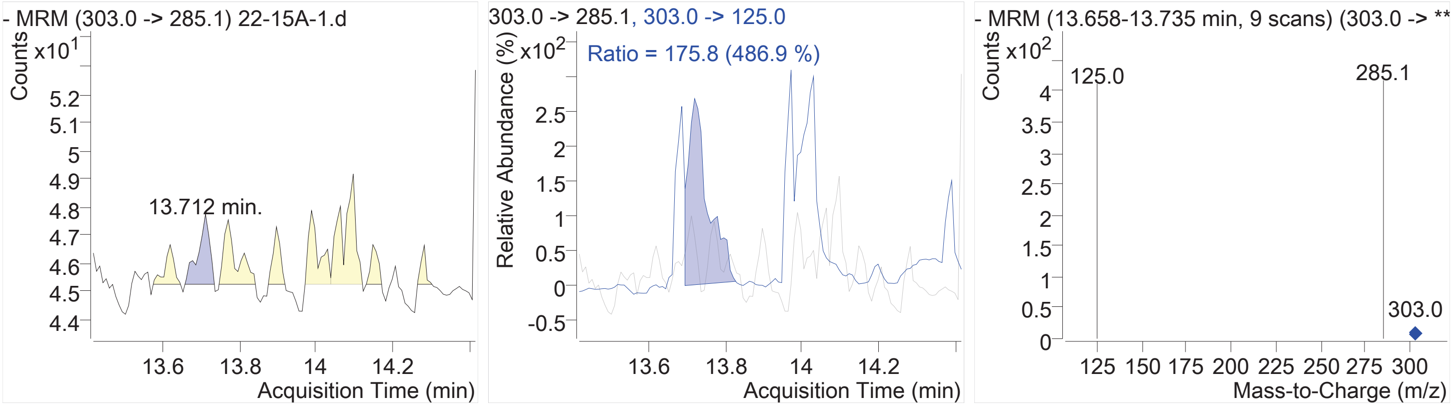

p-Coumaric acid

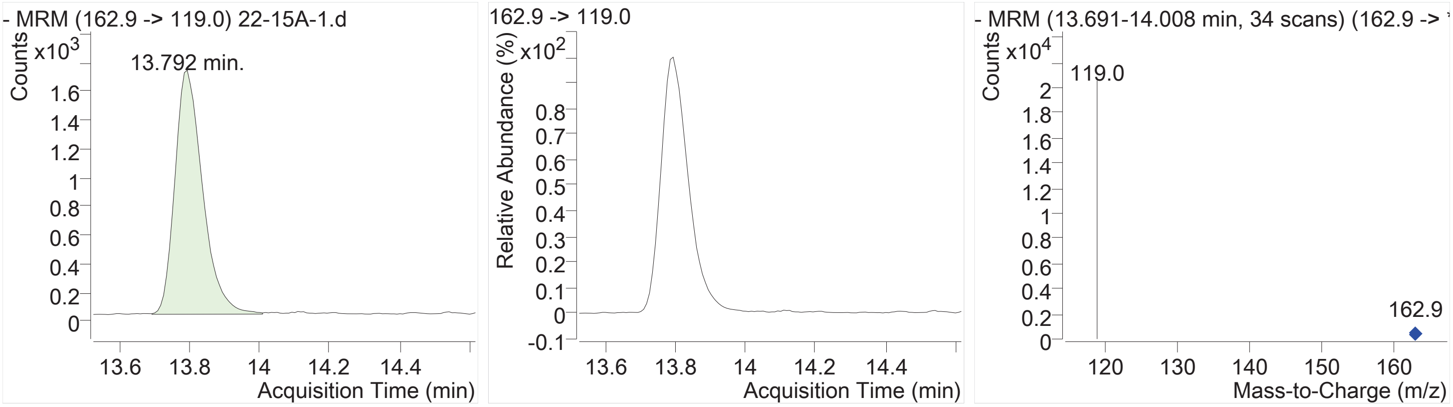

Sinapic acid

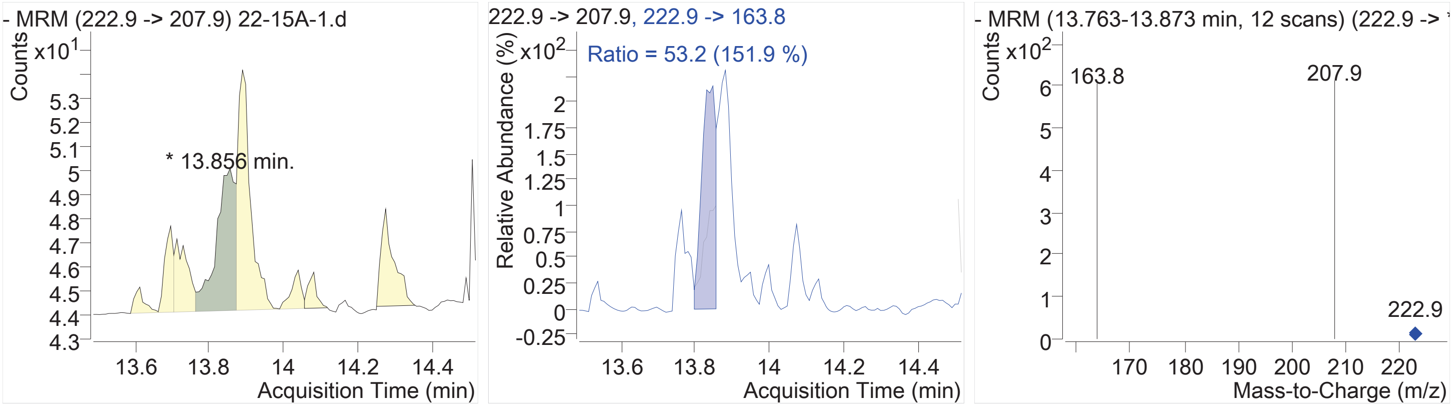

Ferulic acid

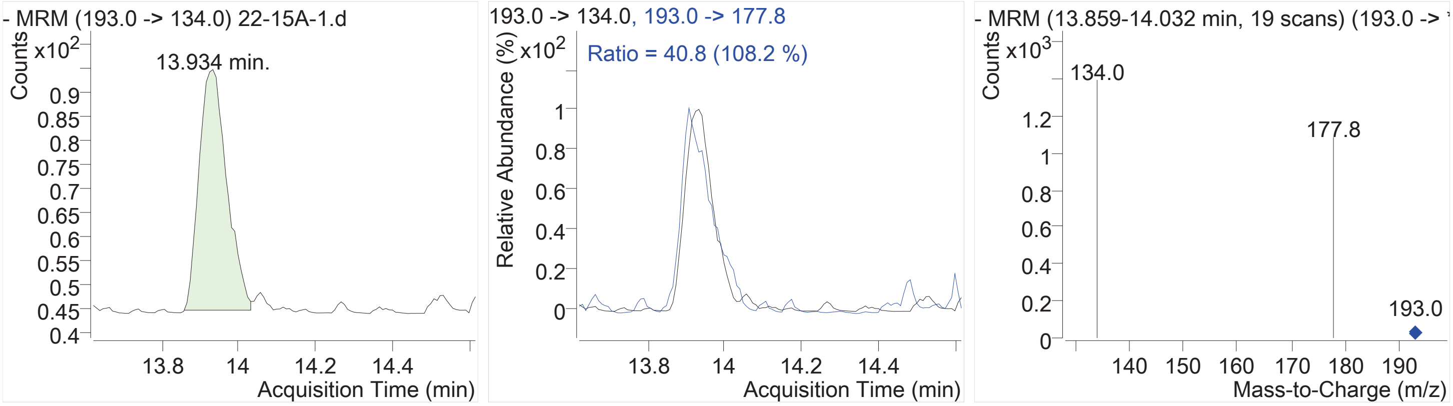

Luteolin 7-glucoside

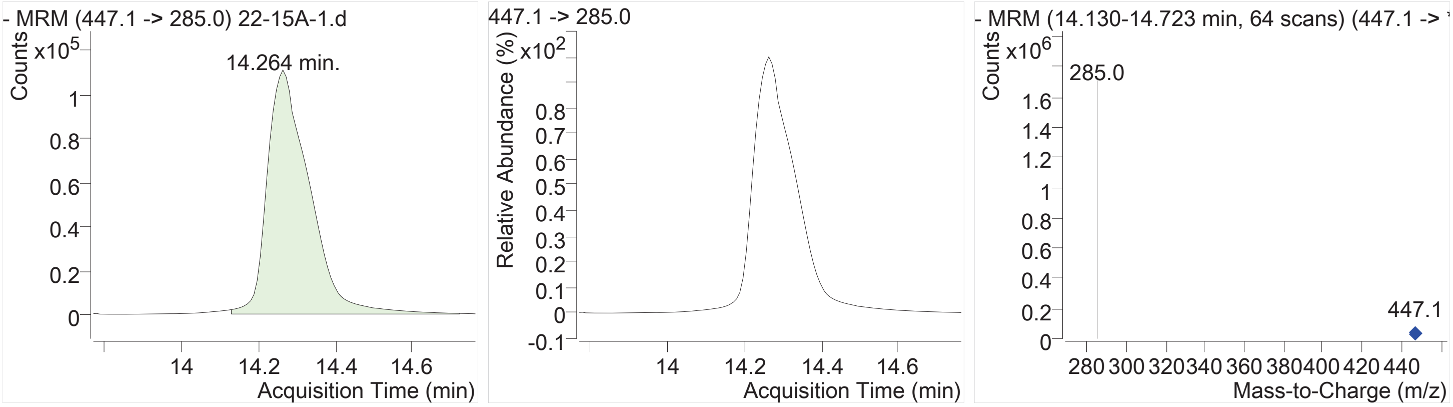

Hesperidin

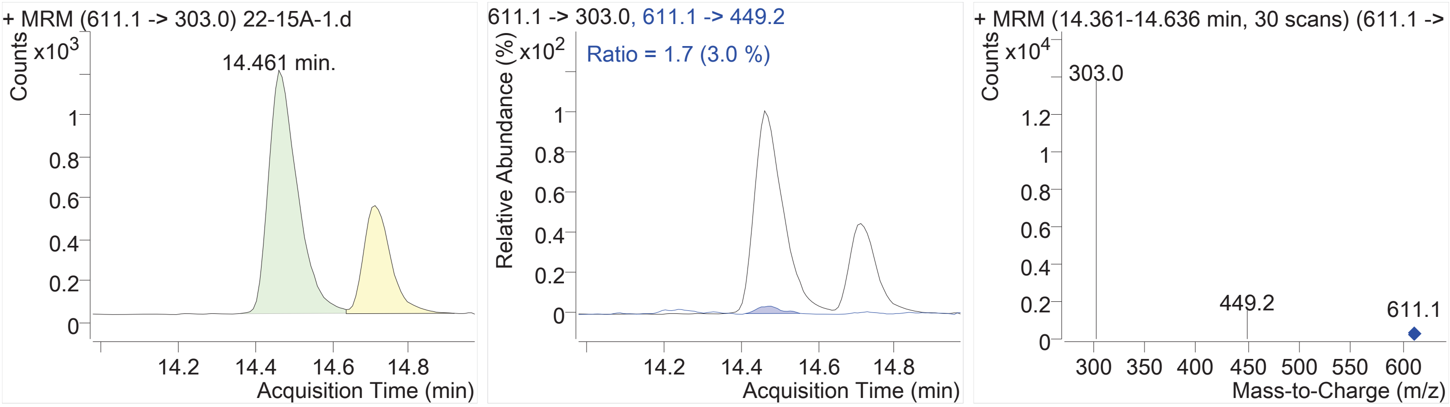

Agilent Technologies

Hyperoside

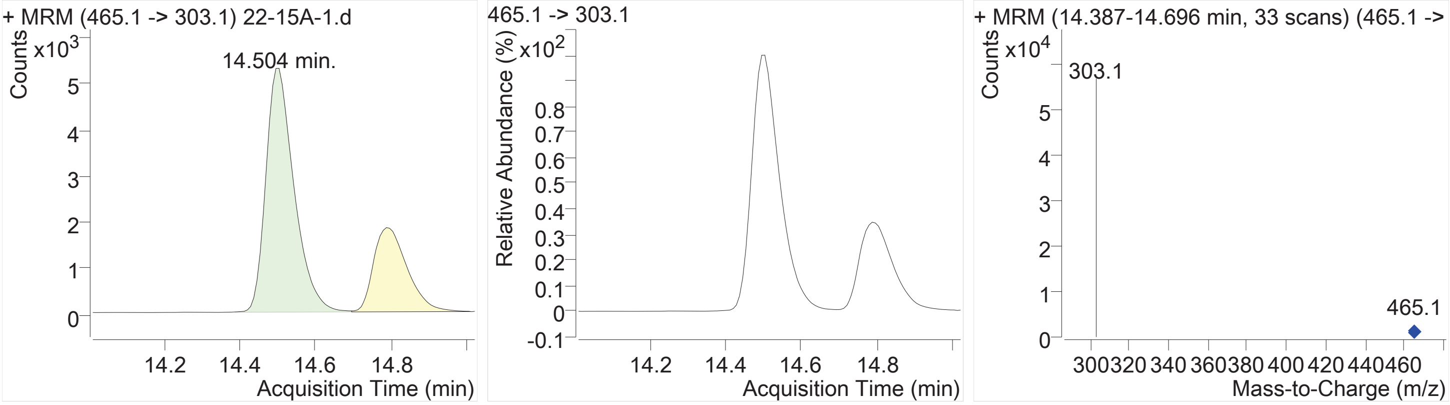

Rosmarinic acid

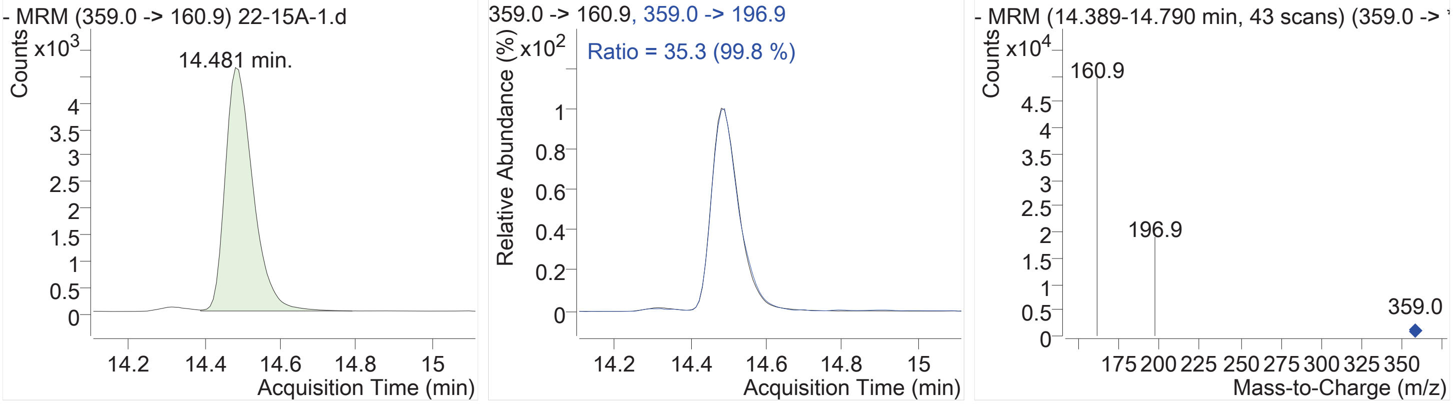

Apigenin 7-glucoside

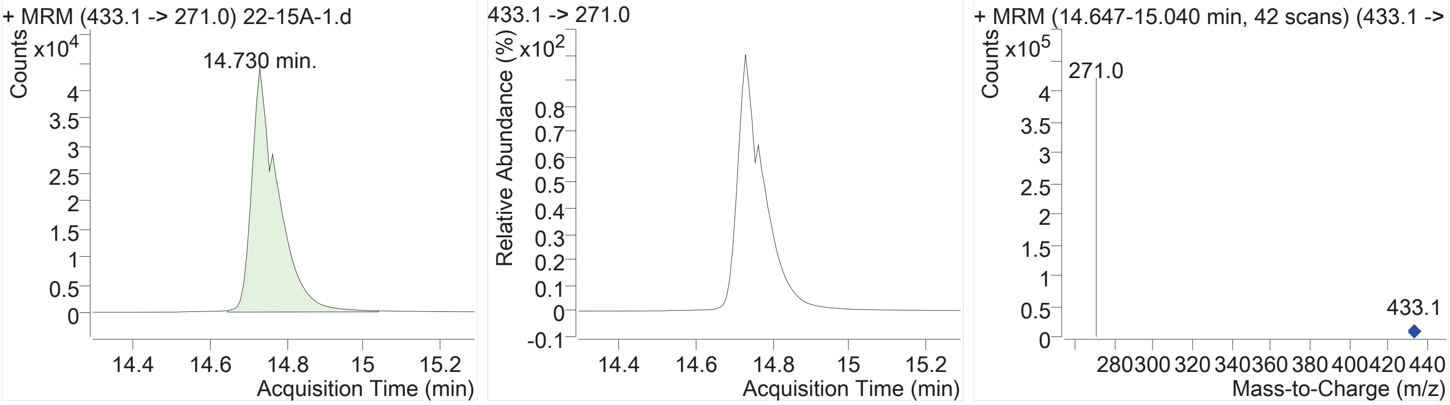

Pinoresinol

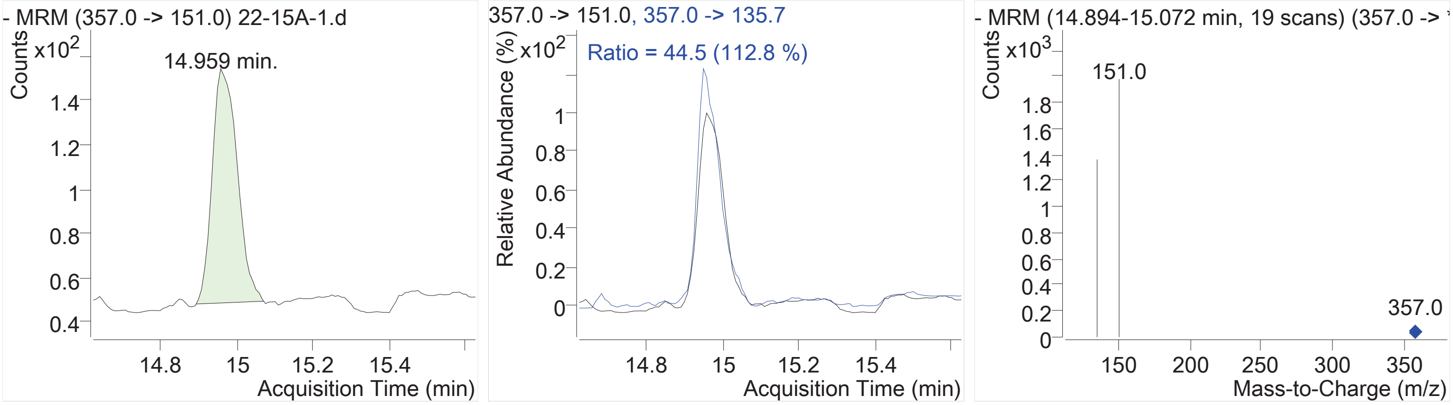

2-Hydroxycinnamic acid

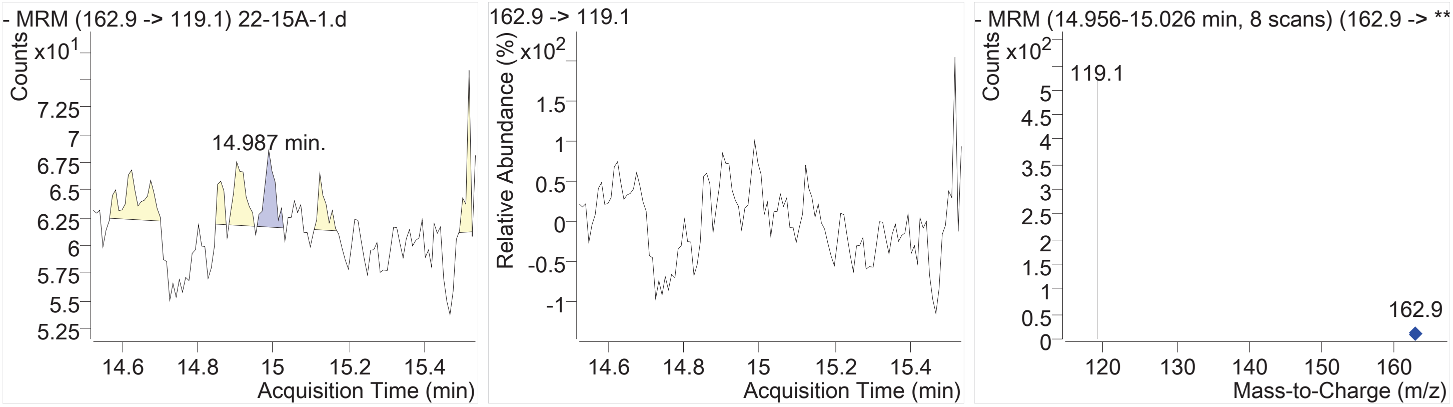

Eriodictyol

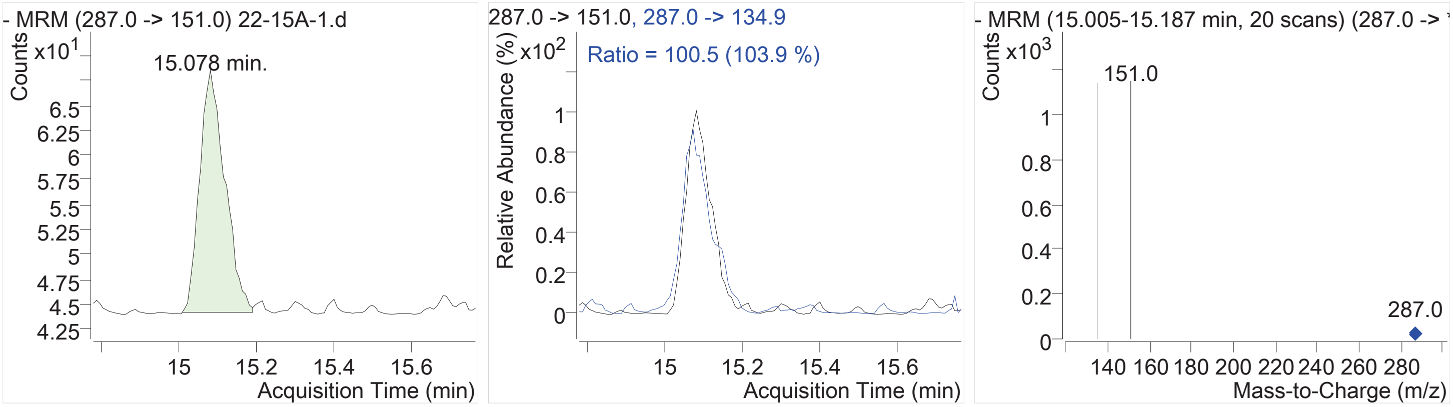

Quercetin

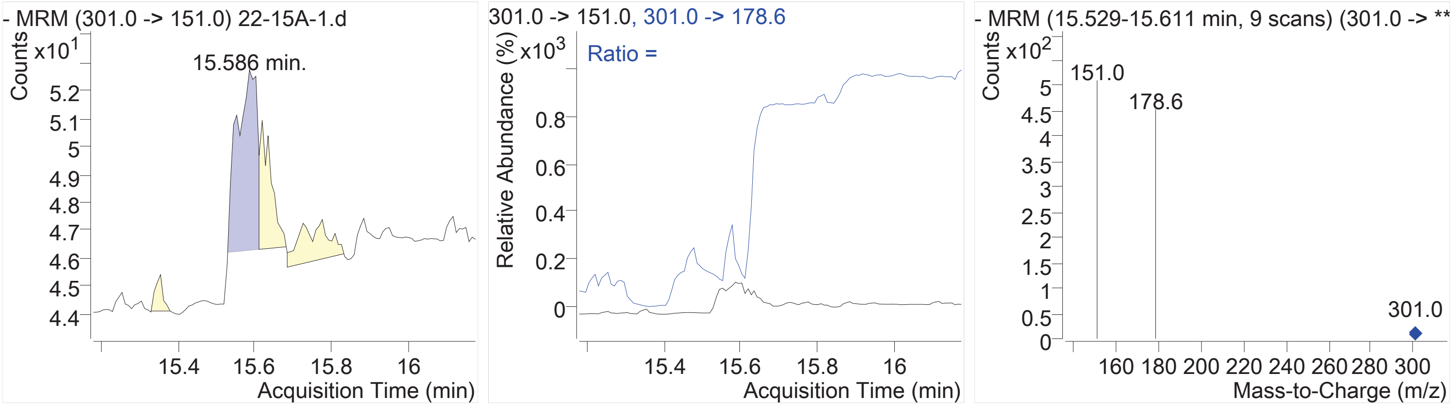

Luteolin

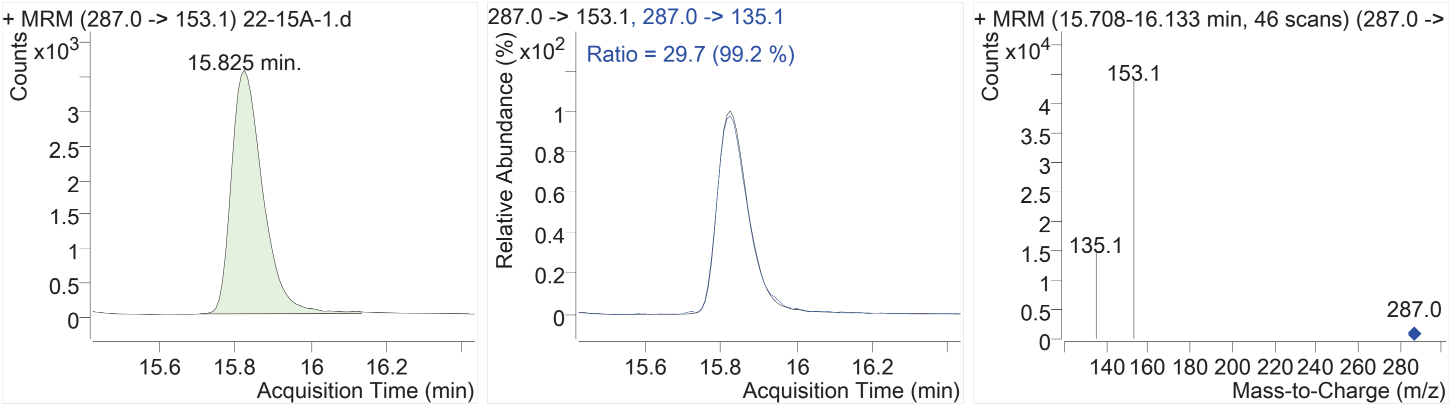

Kaempferol

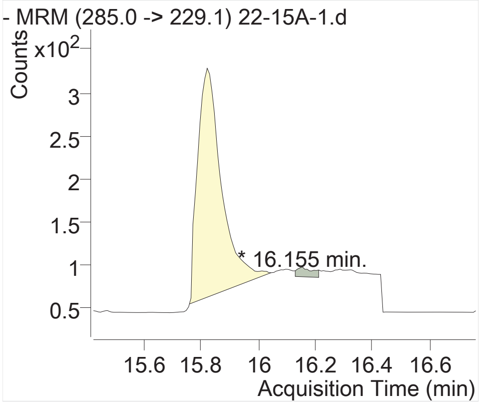

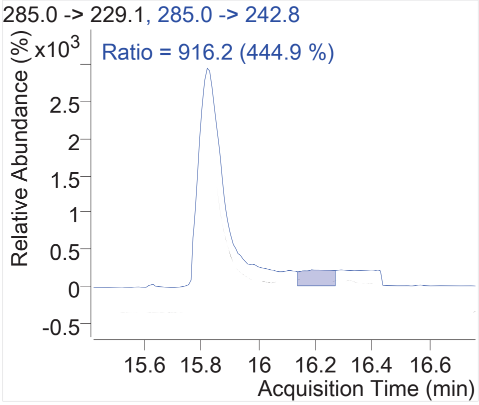

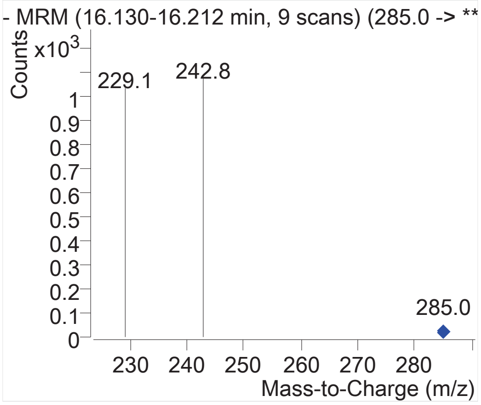

Apigenin

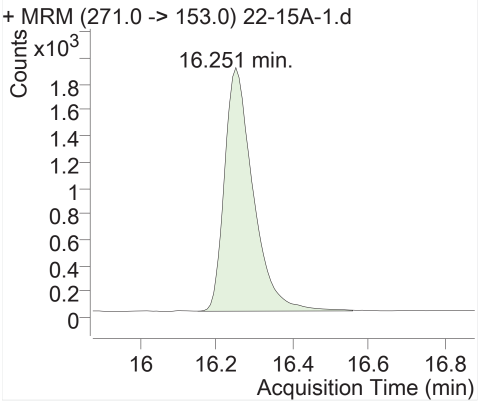

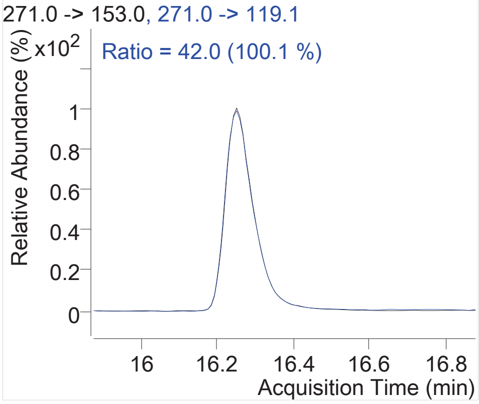

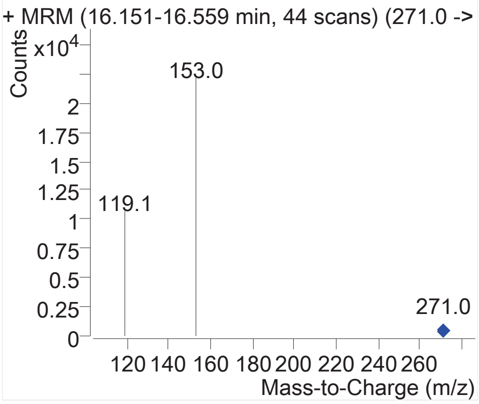

Quantitative Analysis Complete Report

Agilent Technologies

**Batch Path**	D:\MassHunter\Data\2022ekim\061022cengizhoca\QuantResults\071022.batch.bin		
**Analysis Time**	10/11/2022 1:33:26 PM	**Analyst Name**	Defam-PC\admin
**Report Time**	10/11/2022 1:36:51 PM	**Reporter Name**	admin
**Last Calib Update**	10/11/2022 1:33:17 PM	**Batch State**	Processed
**Quant Batch Version**	B.07.01	**Quant Report Version**	B.07.01

**Acq. Time**	10/7/2022 1:57:58 PM	**Data File**	22-15A-2.d
**Sample Type**	Sample	**Sample Name**	22-15A-2
**Dilution**	1	**Acq. Method**	FENOLIK_DMRM2021-31bilesen.m

Sample Chromatogram

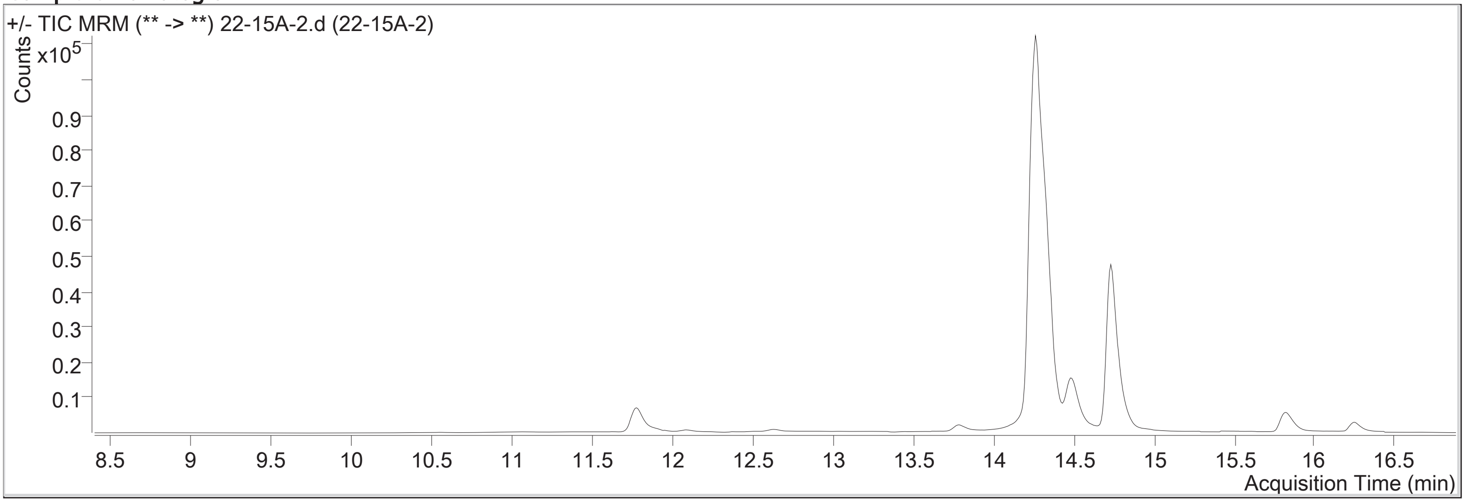

Compound	Transition	RT	Resp.	Final Conc	Units
Gallic acid	168.9 -> 125.0	8.883	5	ND	ng/ml
Protocatechuic acid	152.9 -> 108.9	10.559	817	67.2465	ng/ml
Pyrocatechol	109.0 -> 52.9	10.822	1	ND	ng/ml
3,4-Dihydroxyphenylacetic acid	167.0 -> 123.0	11.046	2	ND	ng/ml
(+)-Catechin	289.0 -> 245.0	11.419	2	ND	ng/ml
2,5-Dihydroxybenzoic acid	152.9 -> 109.0	11.971	105	17.0672	ng/ml
Chlorogenic acid	355.0 -> 163.0	11.785	43651	2054.7021	ng/ml
3-Hydroxybenzoic acid	137.0 -> 93.0	12.836	13	17.5453	ng/ml
4-Hydroxybenzoic acid	136.9 -> 93.1	12.089	2801	244.7058	ng/ml
(-)-Epicatechin	291.0 -> 139.1	12.285	5	3.7248	ng/ml
Caffeic acid	179.0 -> 135.0	12.633	3068	103.4612	ng/ml
Syringic acid	196.9 -> 181.9	12.782	138	194.9232	ng/ml
Vanillin	151.0 -> 136.0	13.045	190	35.8794	ng/ml
Verbascoside	623.0 -> 160.8	13.685	2	ND	ng/ml
Taxifolin	303.0 -> 285.1	13.670	8	ND	ng/ml
p-Coumaric acid	162.9 -> 119.0	13.792	9271	352.5828	ng/ml
Sinapic acid	222.9 -> 207.9	13.856	37	15.9931	ng/ml
Ferulic acid	193.0 -> 134.0	13.925	259	49.9390	ng/ml
Luteolin 7-glucoside	447.1 -> 285.0	14.264	841347	9209.8113	ng/ml
Hesperidin	611.1 -> 303.0	14.469	6381	778.5929	ng/ml
Hyperoside	465.1 -> 303.1	14.504	27122	1376.6424	ng/ml
Rosmarinic acid	359.0 -> 160.9	14.481	19857	1586.6509	ng/ml
Apigenin 7-glucoside	433.1 -> 271.0	14.739	229895	6835.9352	ng/ml
Pinoresinol	357.0 -> 151.0	14.967	463	1799.1250	ng/ml
2-Hydroxycinnamic acid	162.9 -> 119.1	14.945	19	ND	ng/ml
Eriodictyol	287.0 -> 151.0	15.087	105	ND	ng/ml
Quercetin	301.0 -> 151.0	15.553	9	ND	ng/ml
Luteolin	287.0 -> 153.1	15.825	20896	1315.1641	ng/ml
Kaempferol	285.0 -> 229.1	16.130	48	ND	ng/ml

Compound	Transition	RT	Resp.	Final Conc	Units
Apigenin	271.0 -> 153.0	16.251	9665	517.2847	ng/ml

Gallic acid

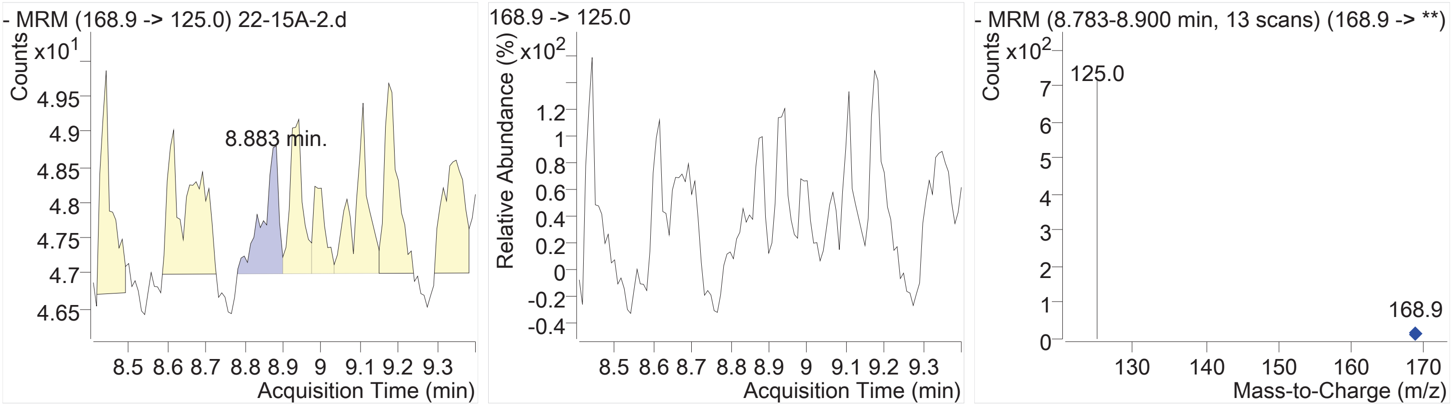

Protocatechuic acid

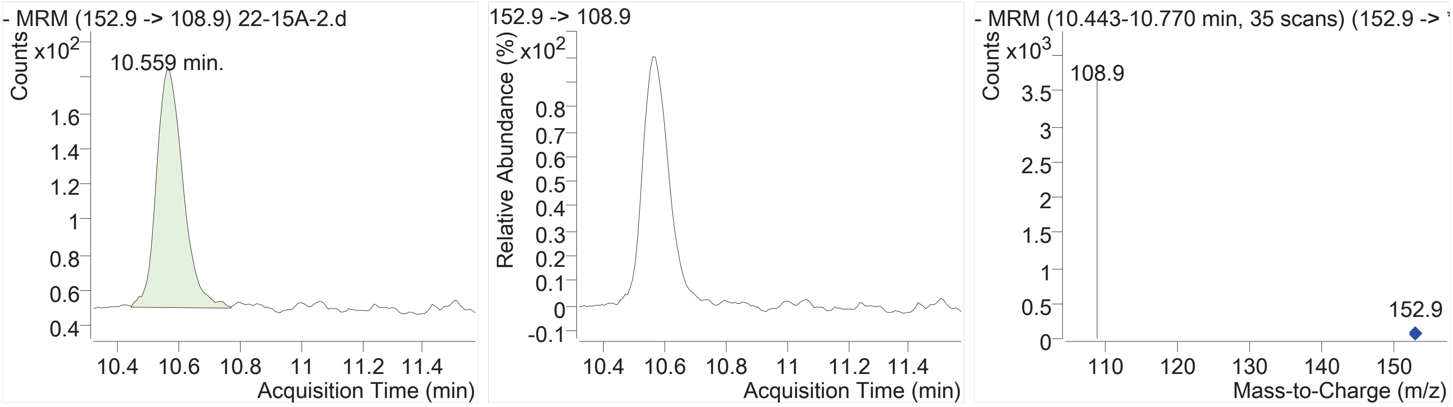

Pyrocatechol

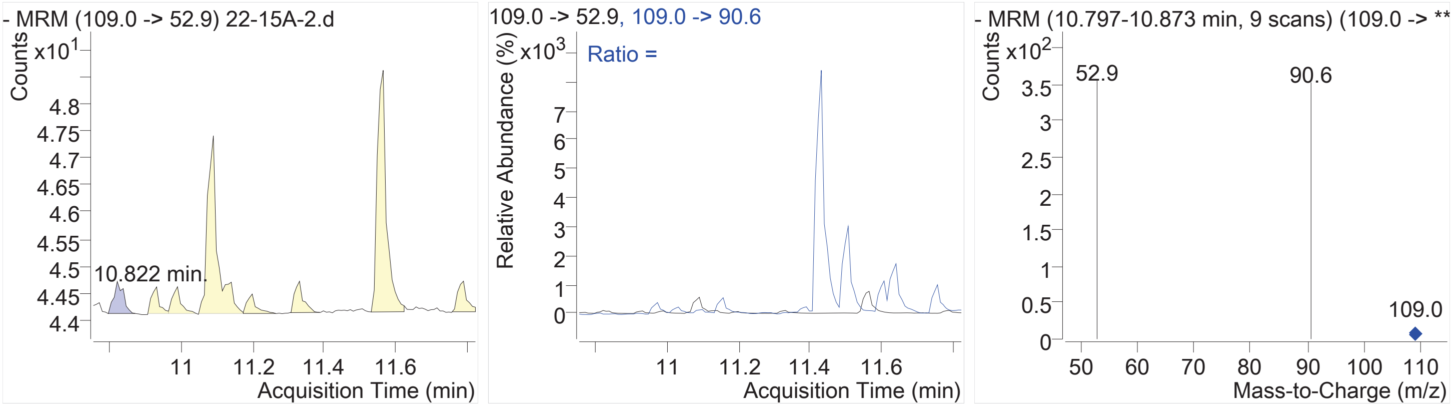

3,4-Dihydroxyphenylacetic acid

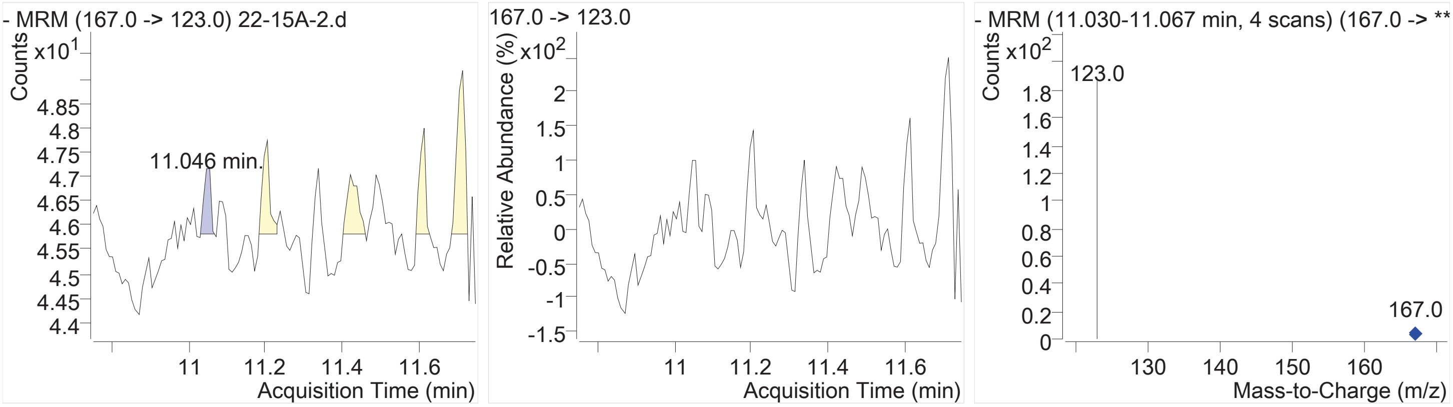

 Generated at 1:33 PM on 10/11/2022

(+)-Catechin

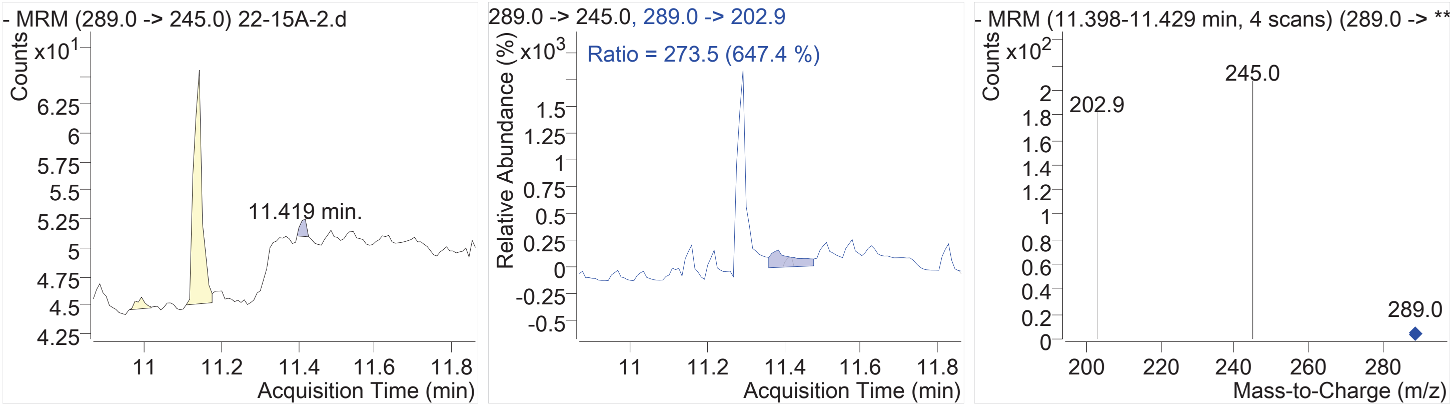

2,5-Dihydroxybenzoic acid

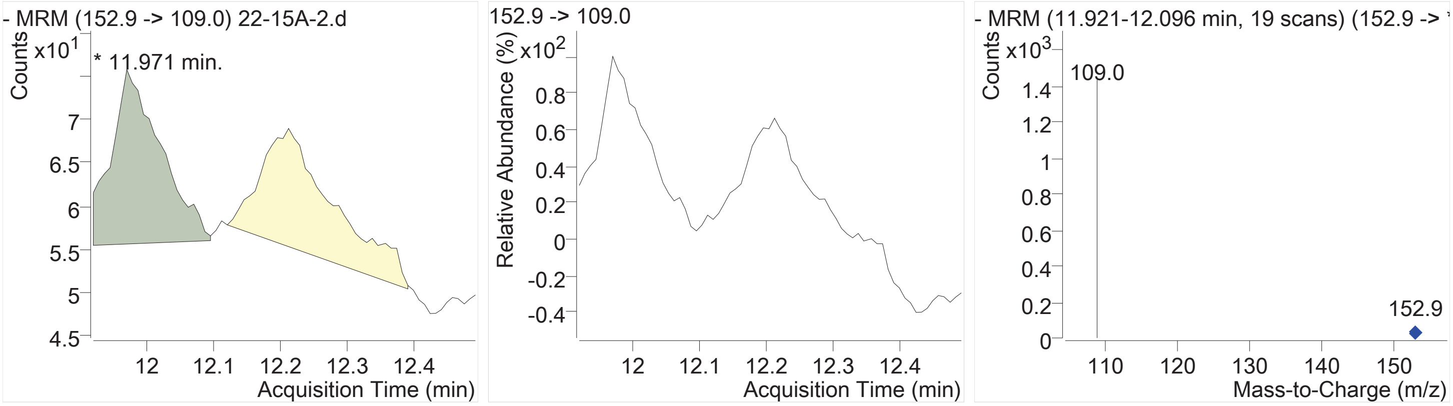

Chlorogenic acid

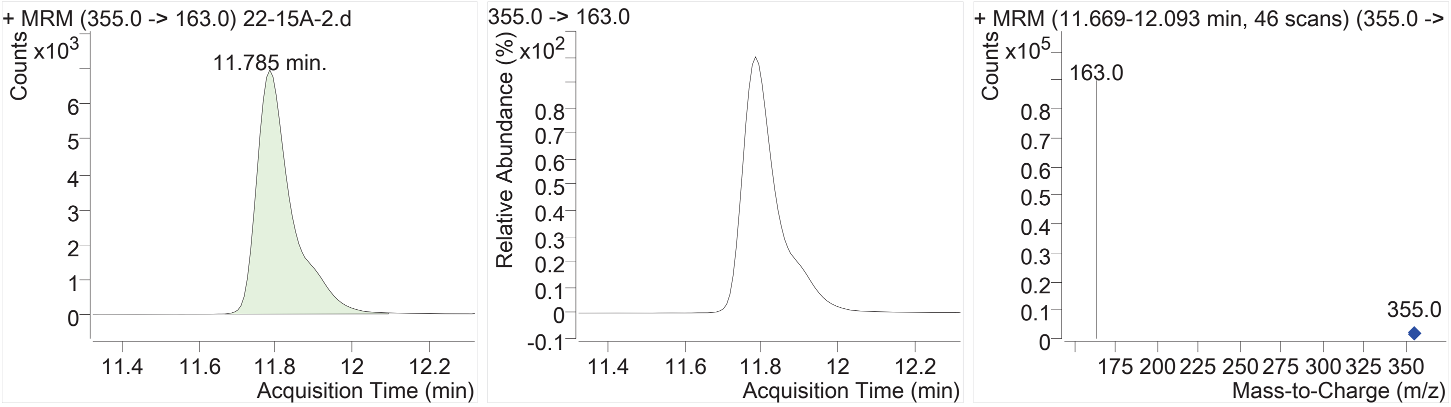

3-Hydroxybenzoic acid

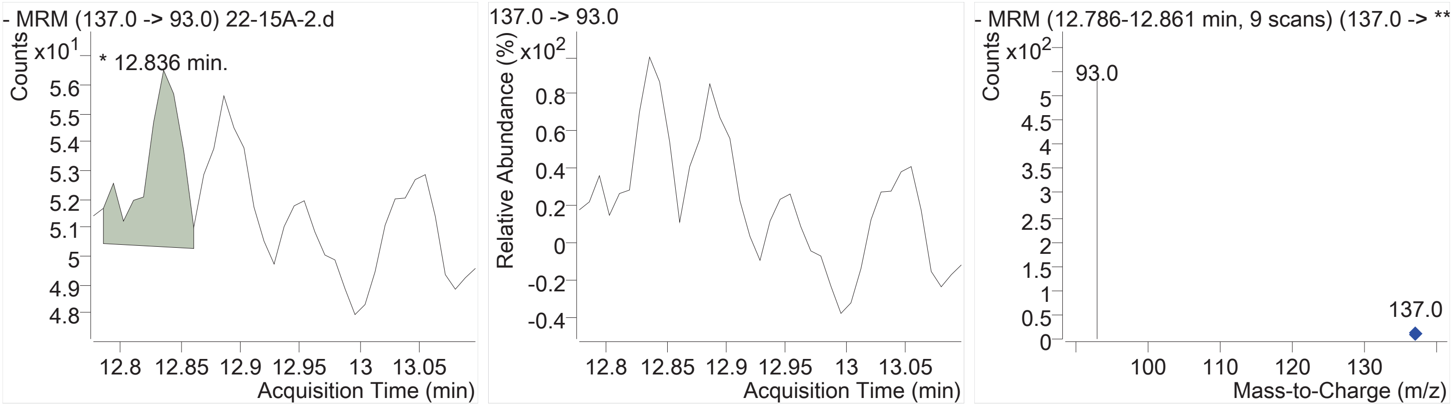

4-Hydroxybenzoic acid

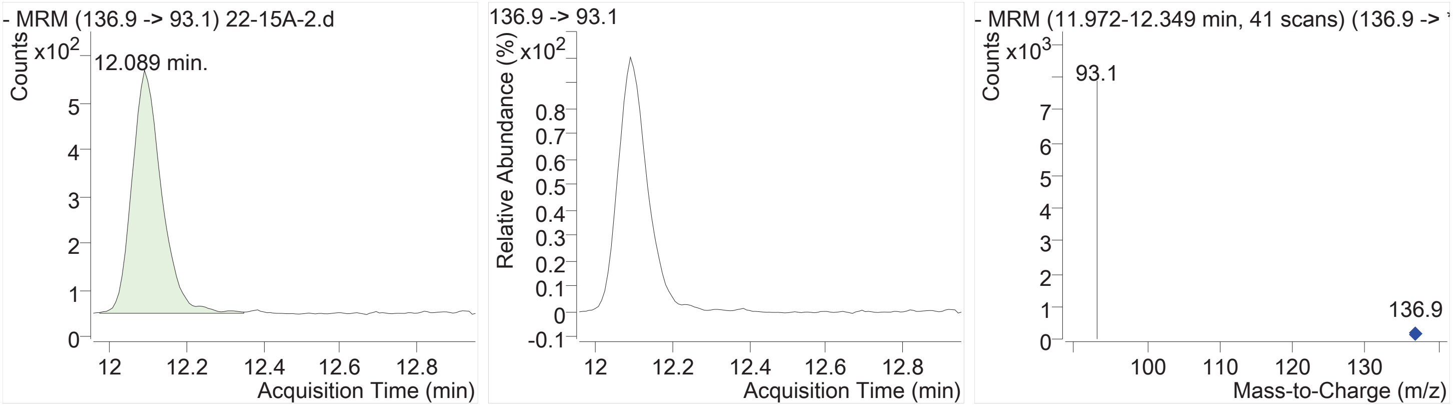

(-)-Epicatechin

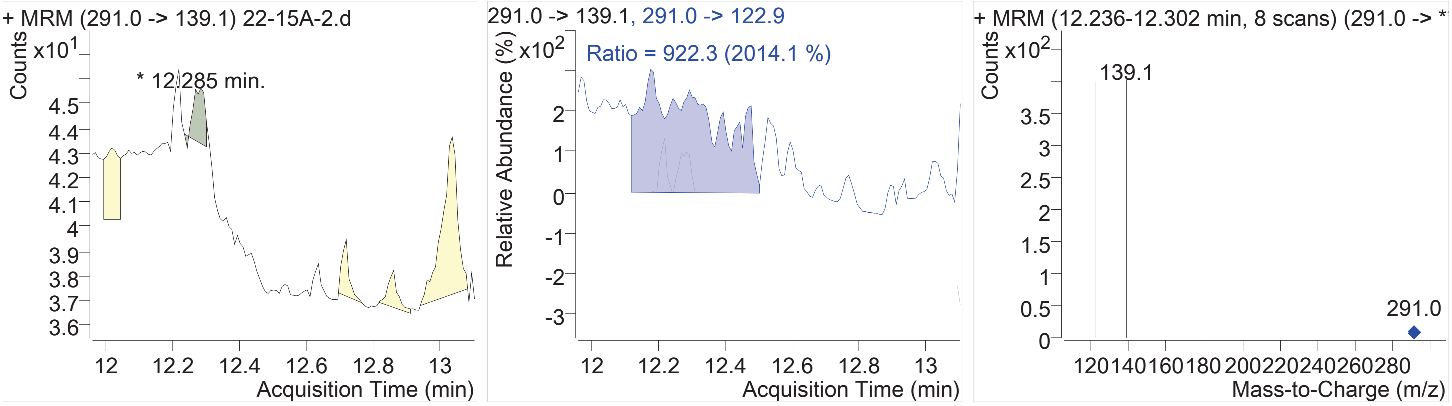

Caffeic acid

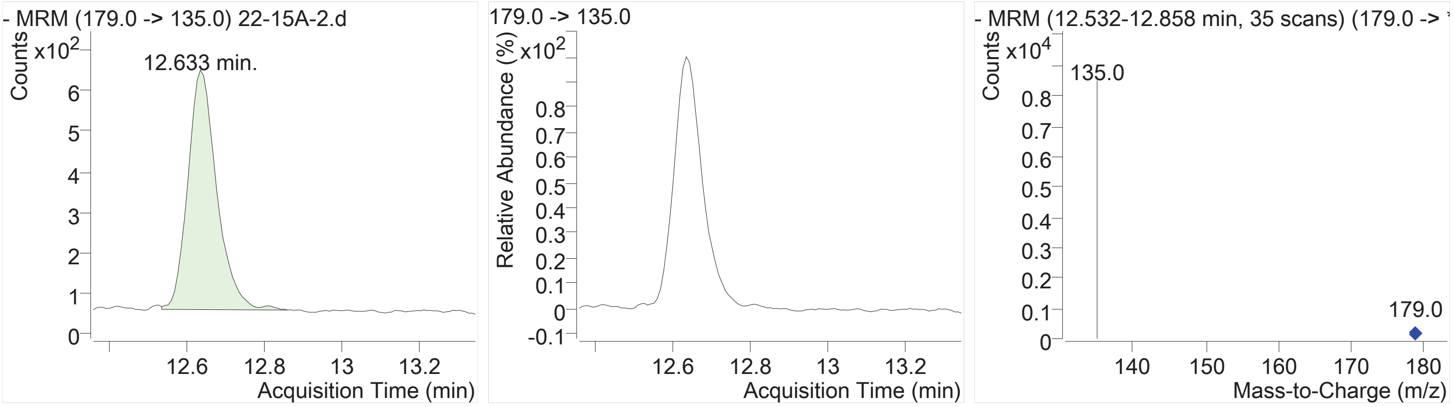

Syringic acid

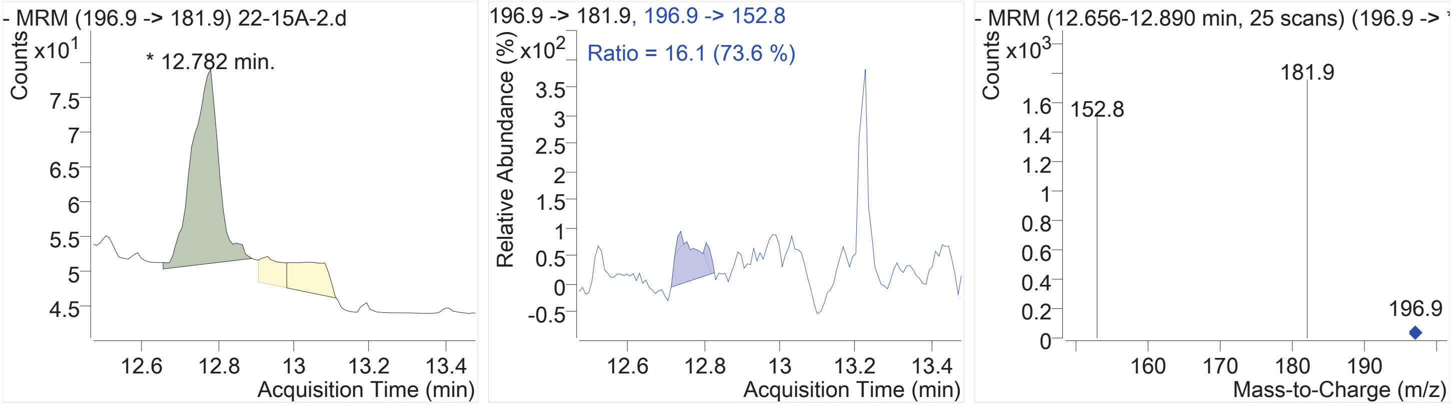

Vanillin

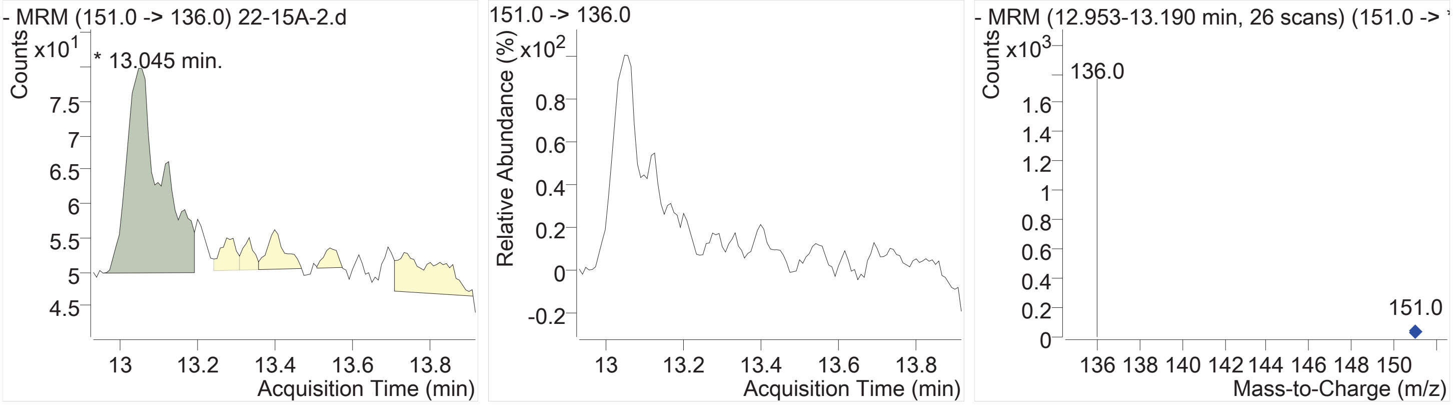

Verbascoside

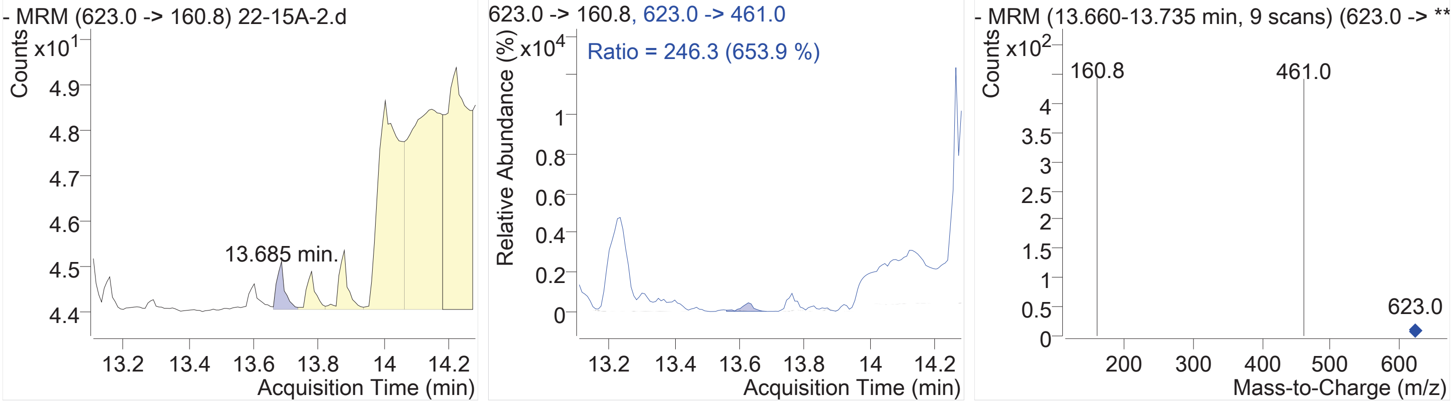

Taxifolin

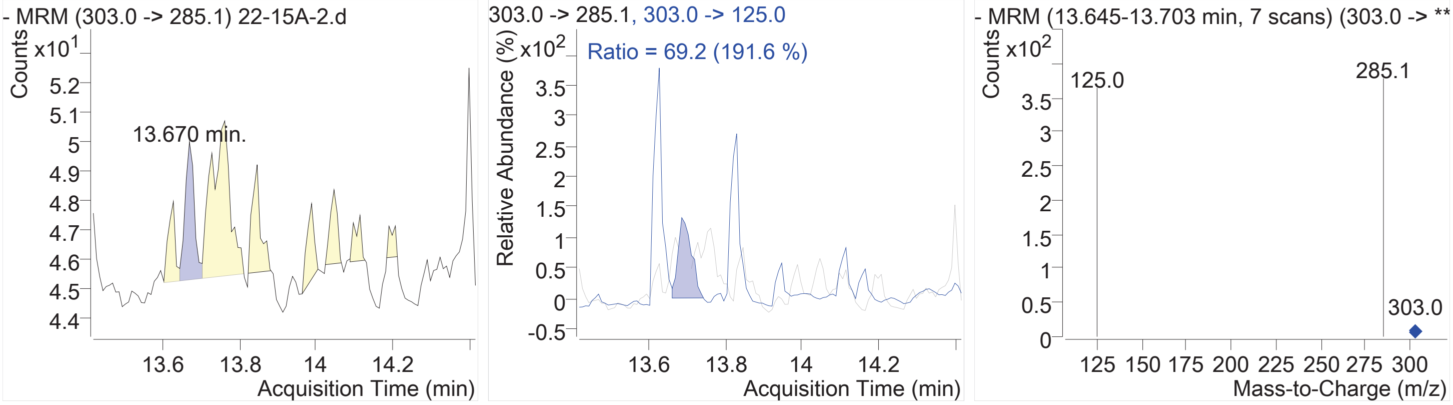

p-Coumaric acid

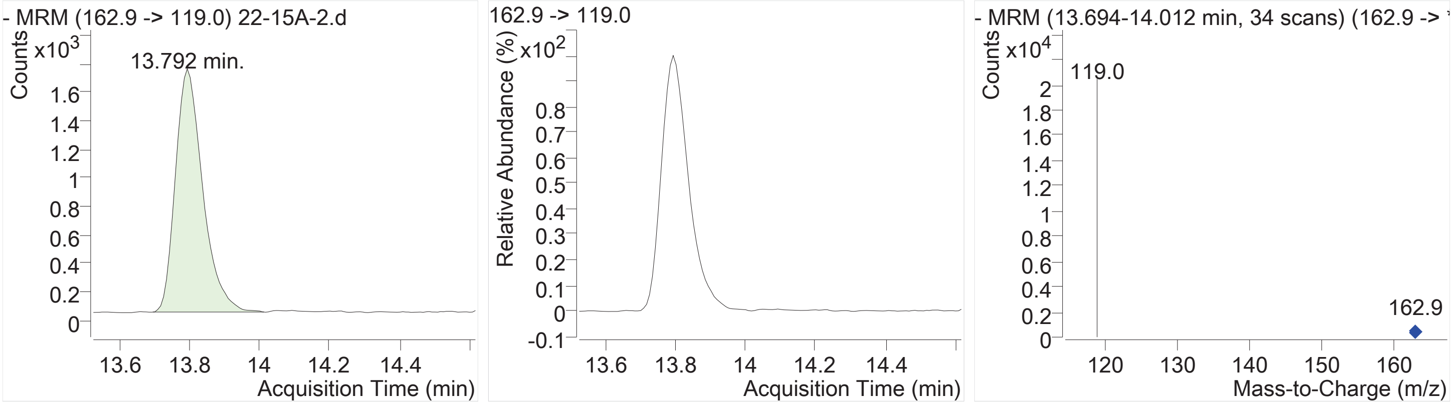

Sinapic acid

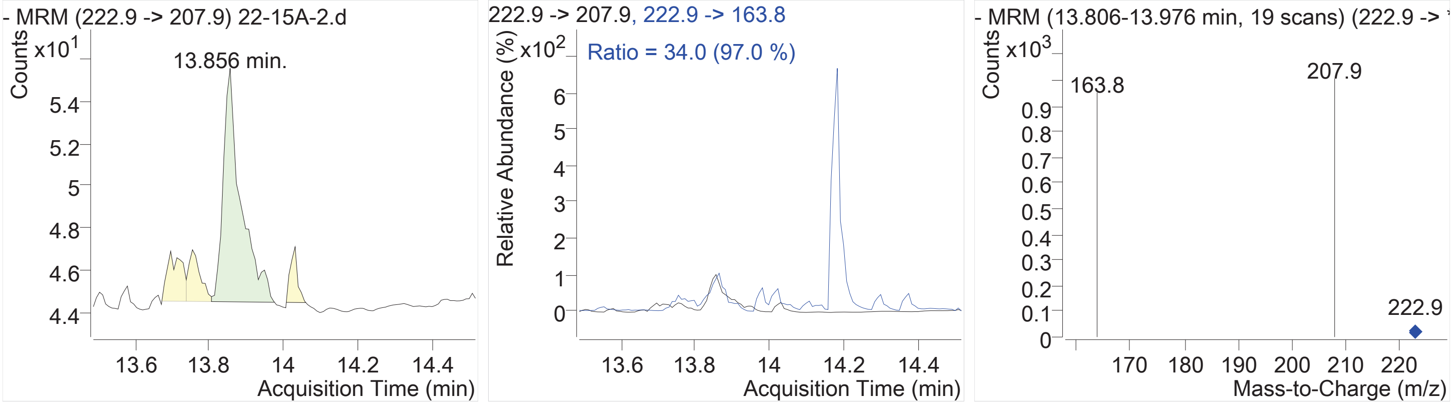

Ferulic acid

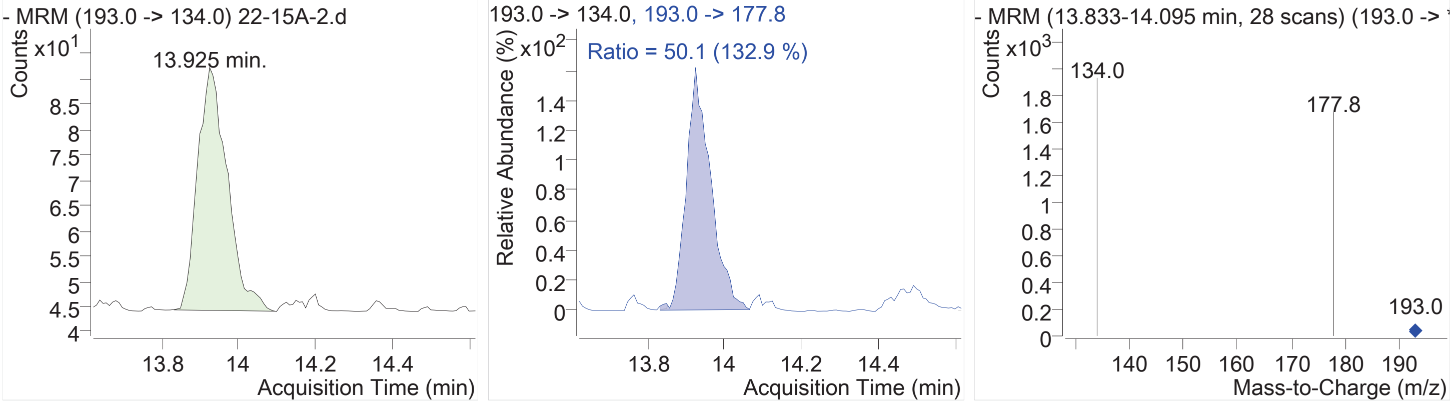

Luteolin 7-glucoside

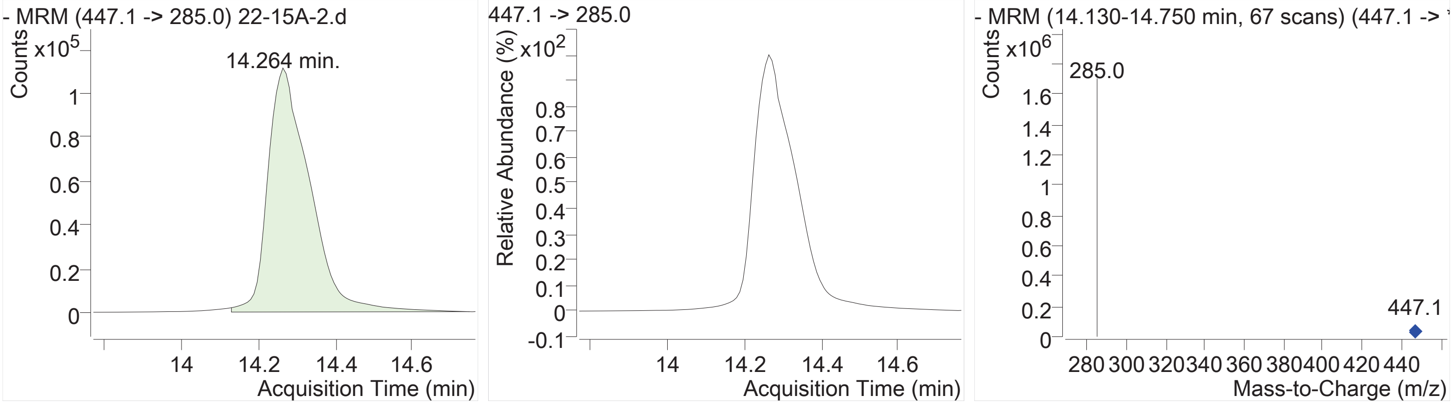

Hesperidin

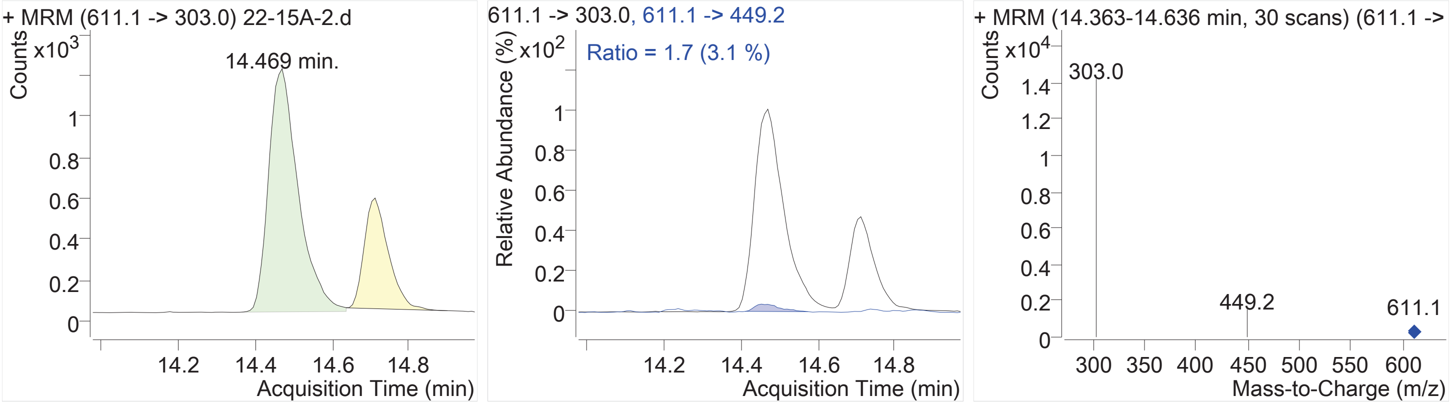

Agilent Technologies

Hyperoside

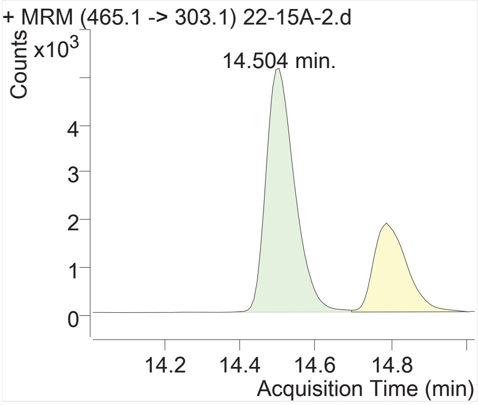

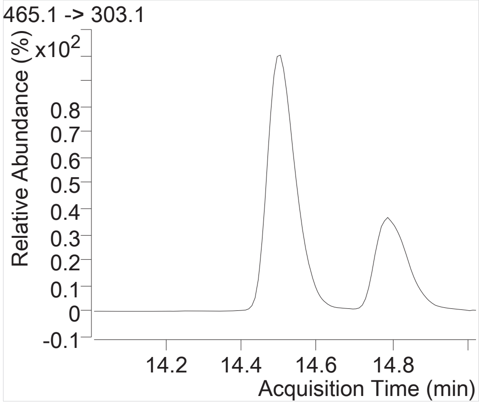

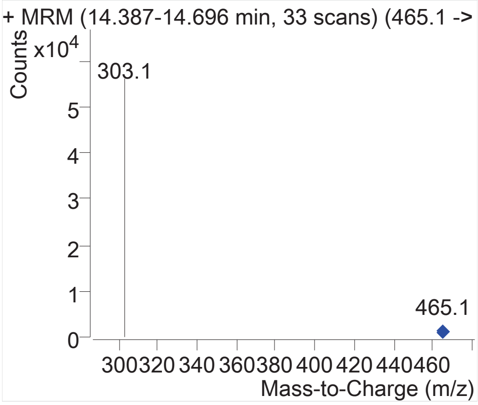

Rosmarinic acid

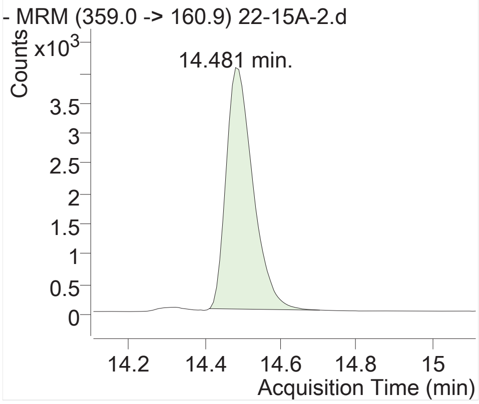

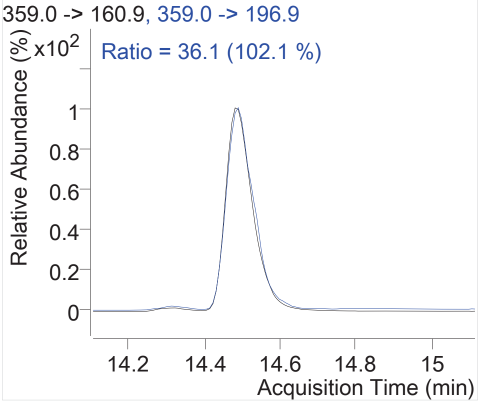

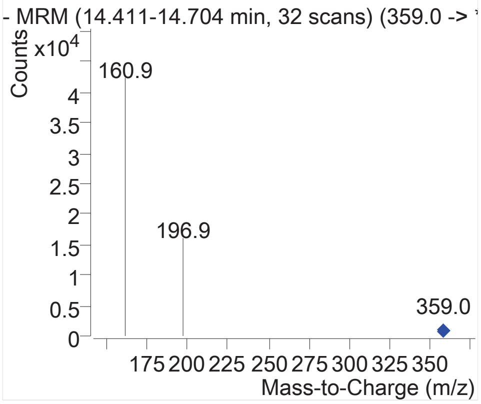

Apigenin 7-glucoside

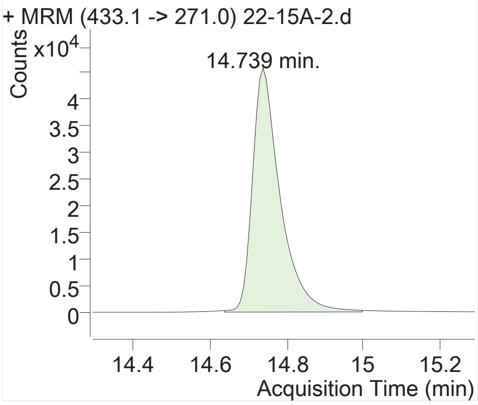

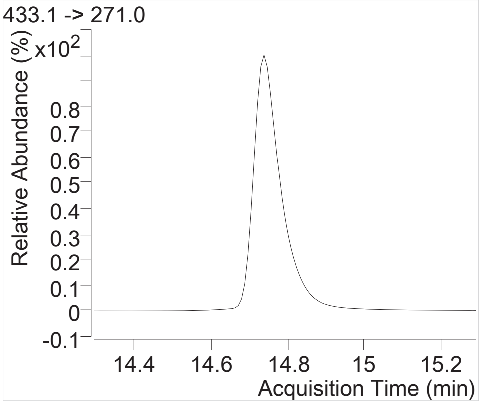

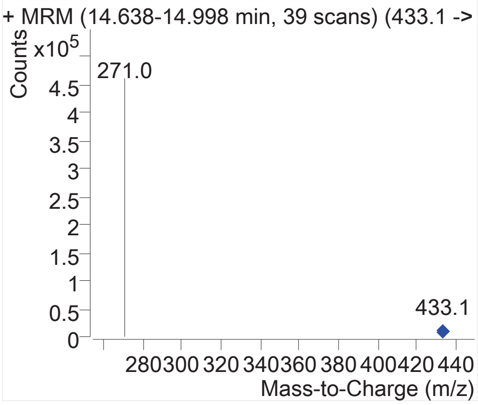

Pinoresinol

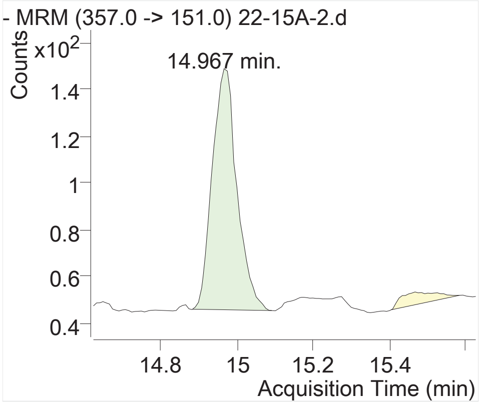

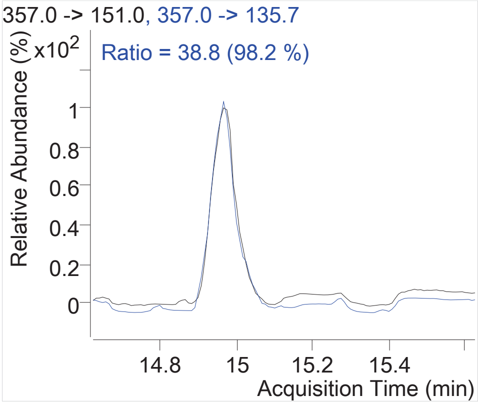

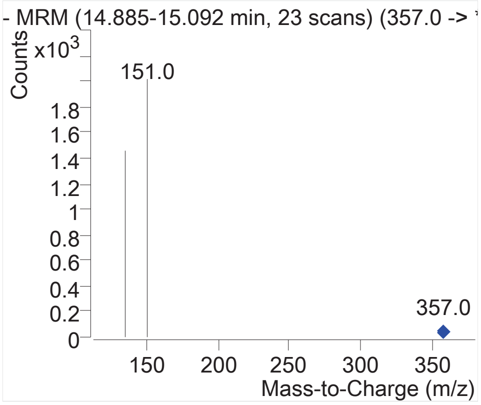

2-Hydroxycinnamic acid

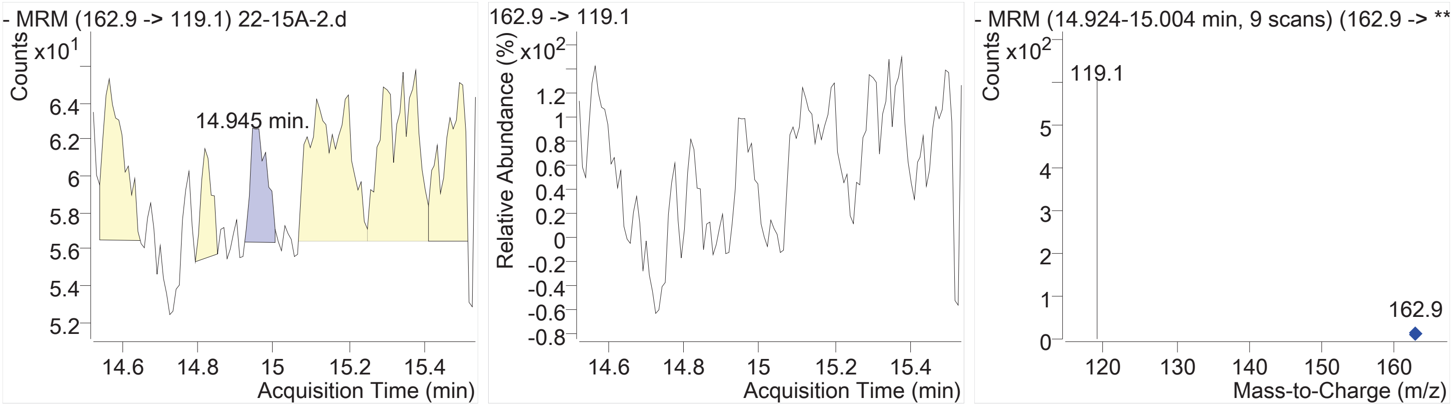

Eriodictyol

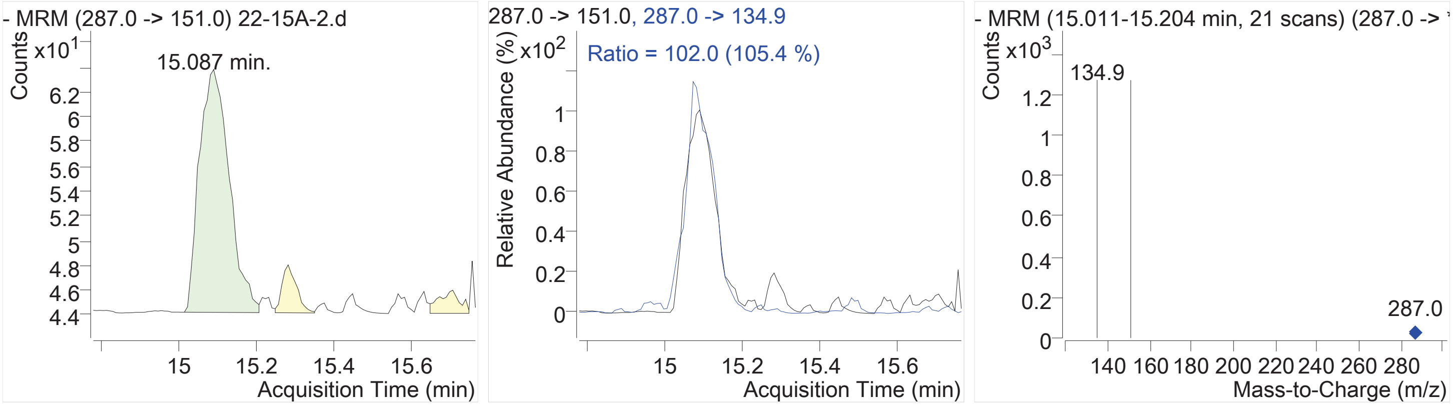

Quercetin

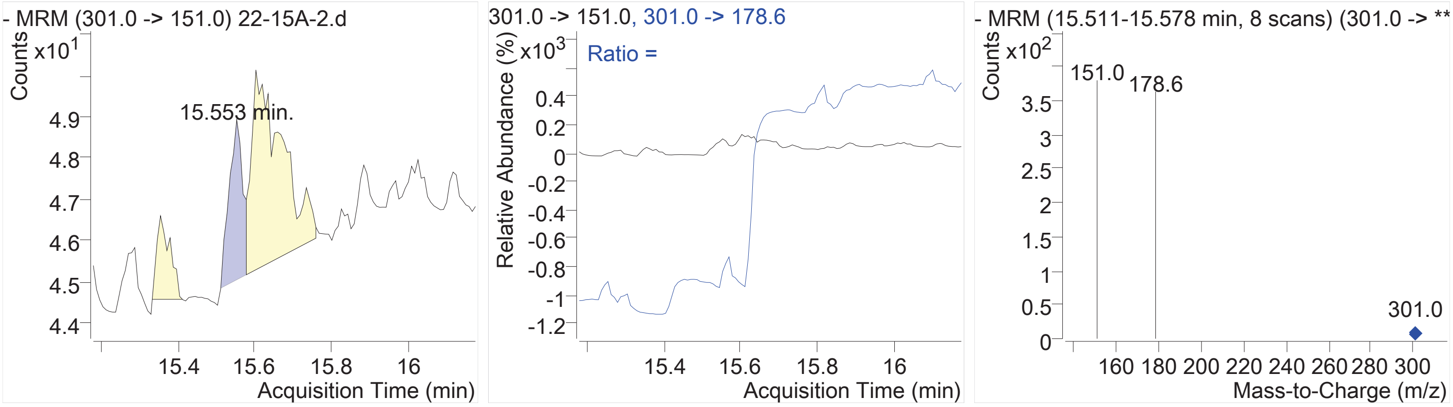

Luteolin

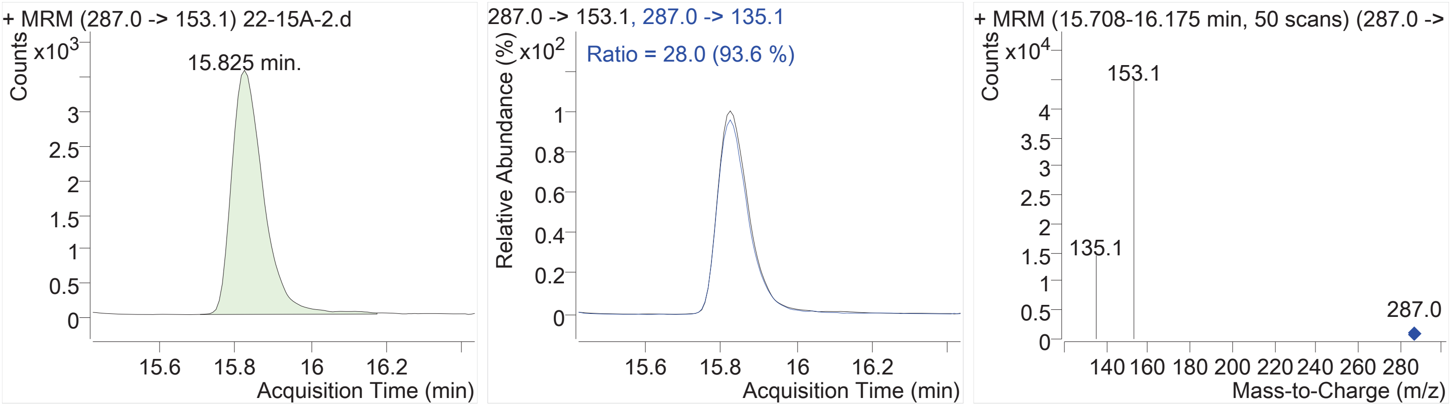

Agilent Technologies

Kaempferol

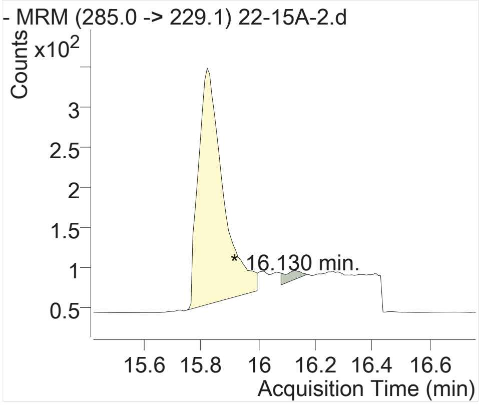

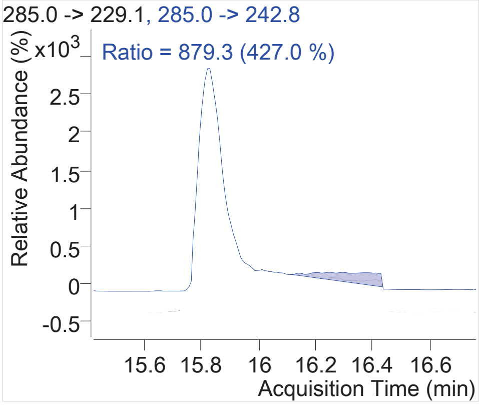

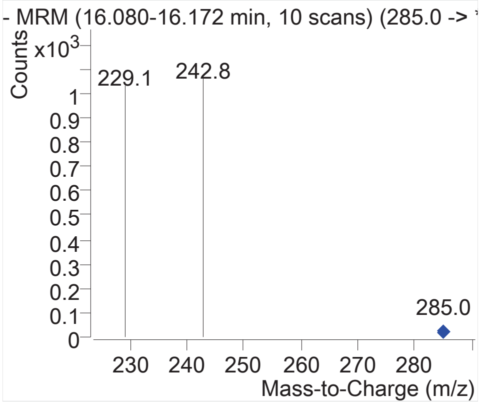

Apigenin

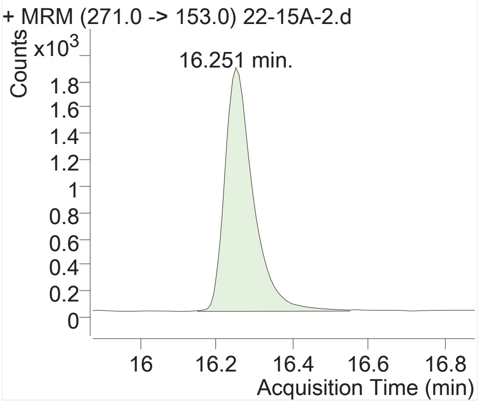

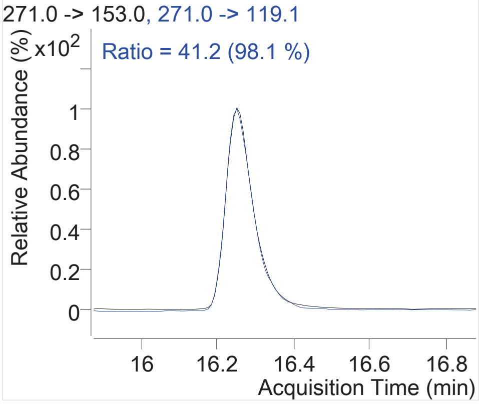

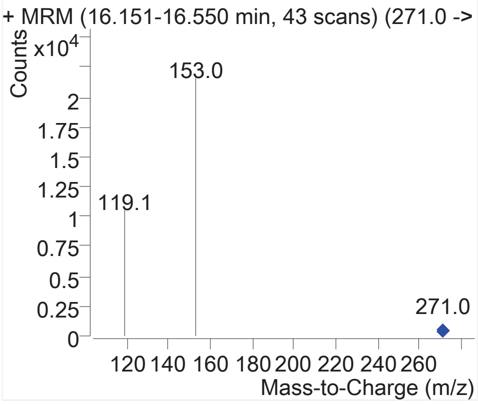

Quantitative Analysis Complete Report

**Batch Path**	D:\MassHunter\Data\2022ekim\061022cengizhoca\QuantResults\071022.batch.bin		
**Analysis Time**	10/11/2022 1:33:26 PM	**Analyst Name**	Defam-PC\admin
**Report Time**	10/11/2022 1:36:54 PM	**Reporter Name**	admin
**Last Calib Update**	10/11/2022 1:33:17 PM	**Batch State**	Processed
**Quant Batch Version**	B.07.01	**Quant Report Version**	B.07.01

**Acq. Time**	10/7/2022 2:19:39 PM	**Data File**	22-15A-3.d
**Sample Type**	Sample	**Sample Name**	22-15A-3
**Dilution**	1	**Acq. Method**	FENOLIK_DMRM2021-31bilesen.m

Sample Chromatogram

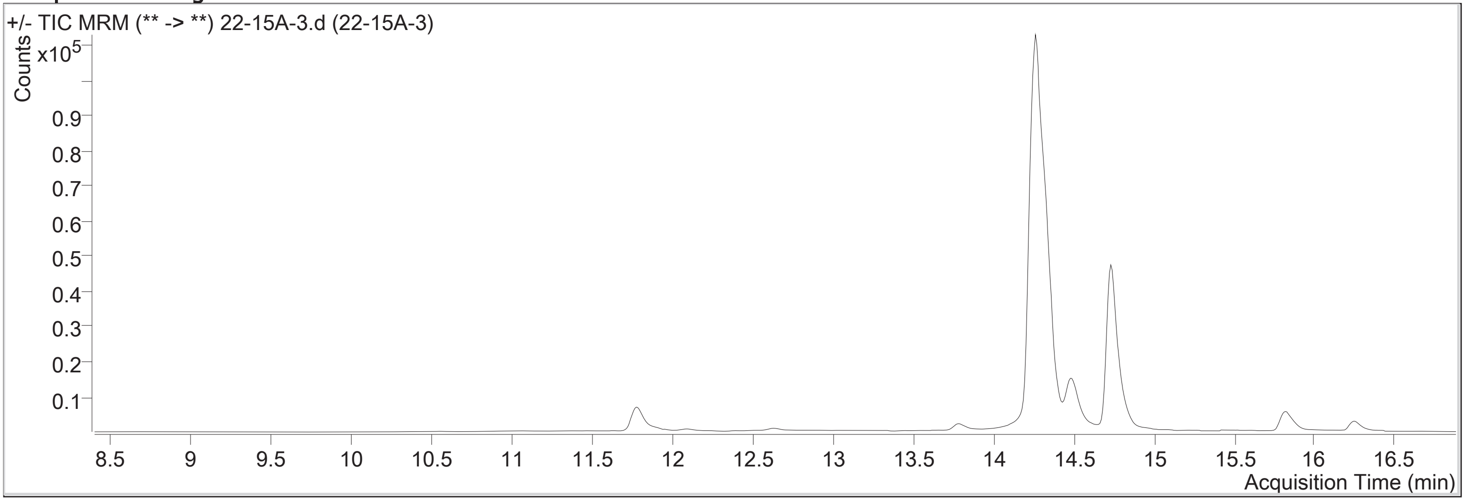

Compound	Transition	RT	Resp.	Final Conc	Units
Gallic acid	168.9 -> 125.0	8.791	2	ND	ng/ml
Protocatechuic acid	152.9 -> 108.9	10.559	819	67.5074	ng/ml
Pyrocatechol	109.0 -> 52.9	11.074	2	ND	ng/ml
3,4-Dihydroxyphenylacetic acid	167.0 -> 123.0	11.355	3	ND	ng/ml
(+)-Catechin	289.0 -> 245.0	11.378	37	ND	ng/ml
2,5-Dihydroxybenzoic acid	152.9 -> 109.0	12.013	112	18.3196	ng/ml
Chlorogenic acid	355.0 -> 163.0	11.785	43407	2043.2324	ng/ml
3-Hydroxybenzoic acid	137.0 -> 93.0	12.811	14	17.6302	ng/ml
4-Hydroxybenzoic acid	136.9 -> 93.1	12.097	2798	244.4272	ng/ml
(-)-Epicatechin	291.0 -> 139.1	12.302	3	3.4495	ng/ml
Caffeic acid	179.0 -> 135.0	12.633	3246	111.1733	ng/ml
Syringic acid	196.9 -> 181.9	12.782	150	212.5223	ng/ml
Vanillin	151.0 -> 136.0	13.053	236	49.7218	ng/ml
Verbascoside	623.0 -> 160.8	13.618	1	ND	ng/ml
Taxifolin	303.0 -> 285.1	13.661	33	ND	ng/ml
p-Coumaric acid	162.9 -> 119.0	13.792	9484	360.8727	ng/ml
Sinapic acid	222.9 -> 207.9	13.864	32	14.6022	ng/ml
Ferulic acid	193.0 -> 134.0	13.925	257	49.5839	ng/ml
Luteolin 7-glucoside	447.1 -> 285.0	14.264	847693	9279.5317	ng/ml
Hesperidin	611.1 -> 303.0	14.469	6270	765.1268	ng/ml
Hyperoside	465.1 -> 303.1	14.504	27019	1371.4377	ng/ml
Rosmarinic acid	359.0 -> 160.9	14.481	19640	1569.3202	ng/ml
Apigenin 7-glucoside	433.1 -> 271.0	14.739	229675	6829.3646	ng/ml
Pinoresinol	357.0 -> 151.0	14.959	520	2025.8040	ng/ml
2-Hydroxycinnamic acid	162.9 -> 119.1	14.735	26	ND	ng/ml
Eriodictyol	287.0 -> 151.0	15.070	131	ND	ng/ml
Quercetin	301.0 -> 151.0	15.635	12	ND	ng/ml
Luteolin	287.0 -> 153.1	15.825	20875	1313.8207	ng/ml
Kaempferol	285.0 -> 229.1	16.105	38	ND	ng/ml

Quantitative Analysis Complete Report

Agilent Technologies

Compound	Transition	RT	Resp.	Final Conc	Units
Apigenin	271.0 -> 153.0	16.251	9965	533.5579	ng/ml

Gallic acid

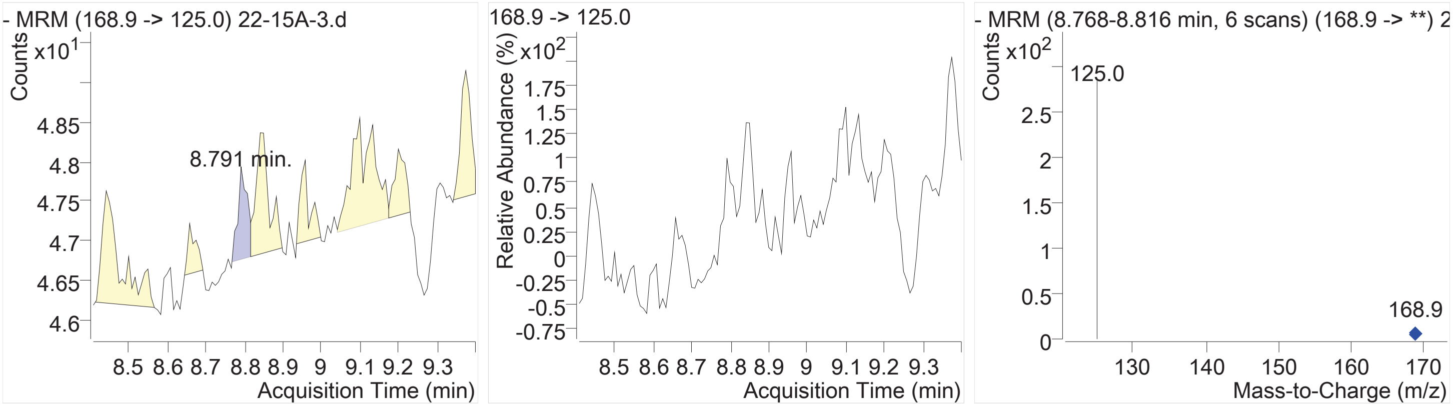

Protocatechuic acid

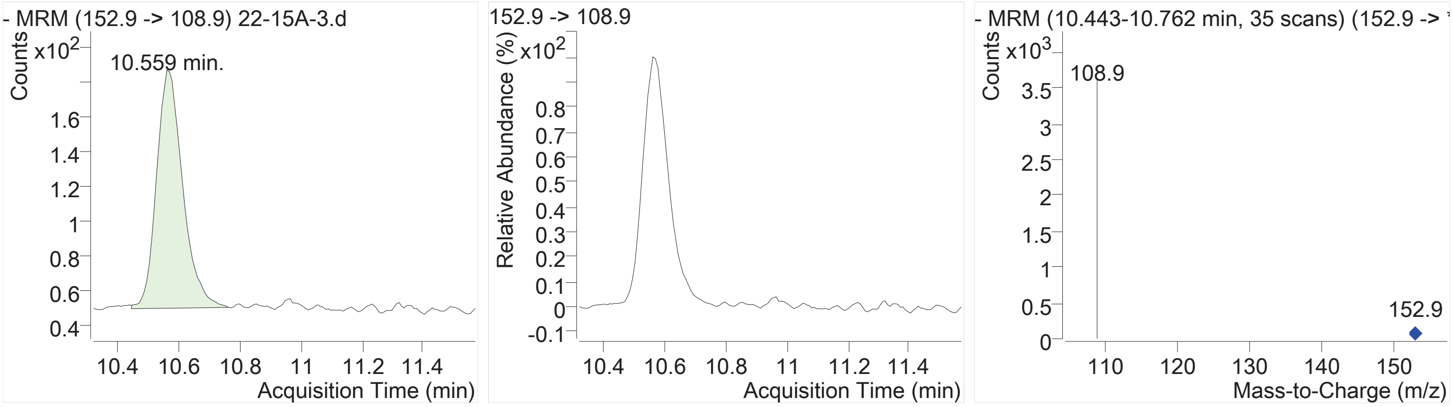

Pyrocatechol

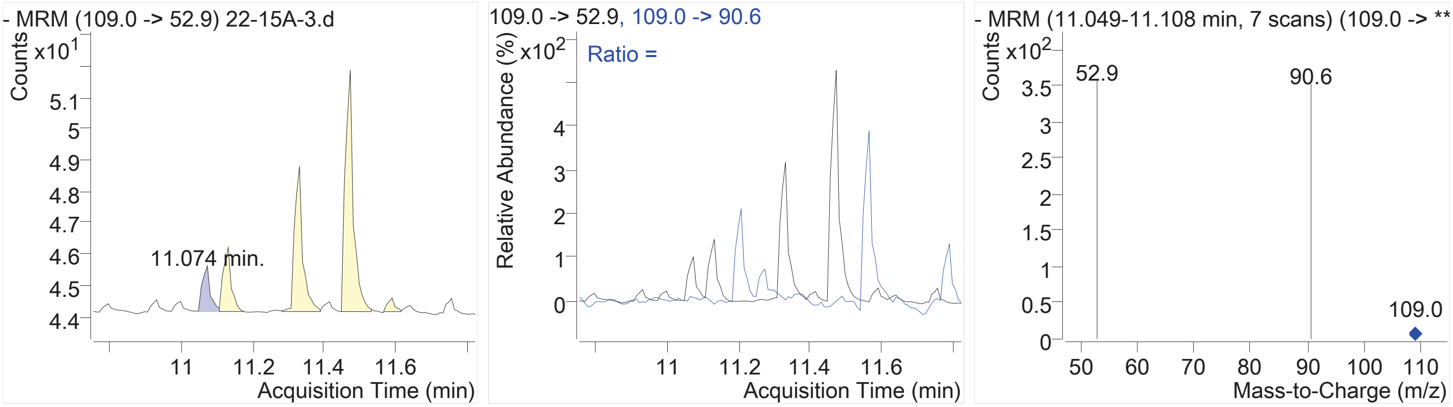

3,4-Dihydroxyphenylacetic acid

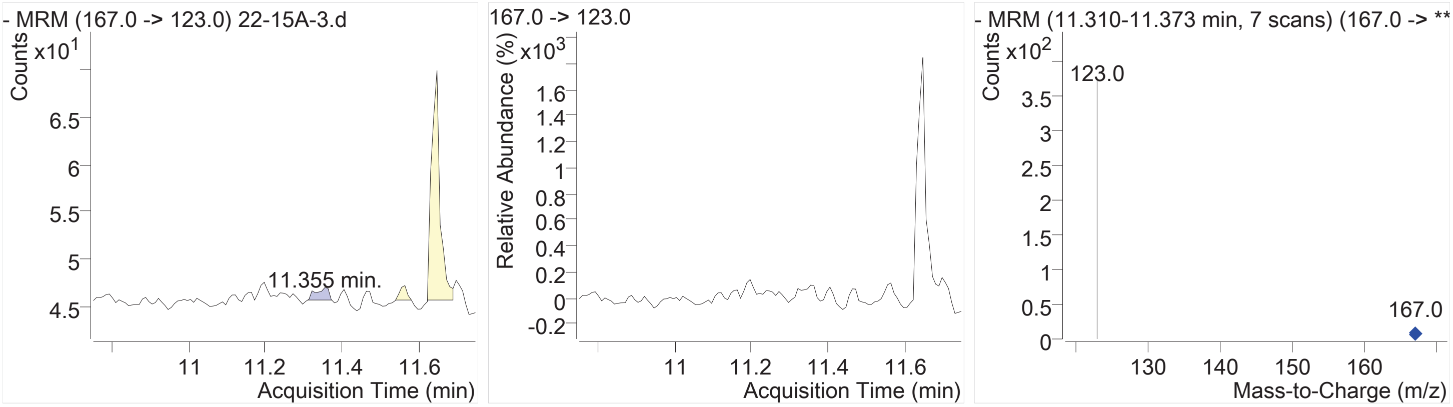

Agilent Technologies

(+)-Catechin

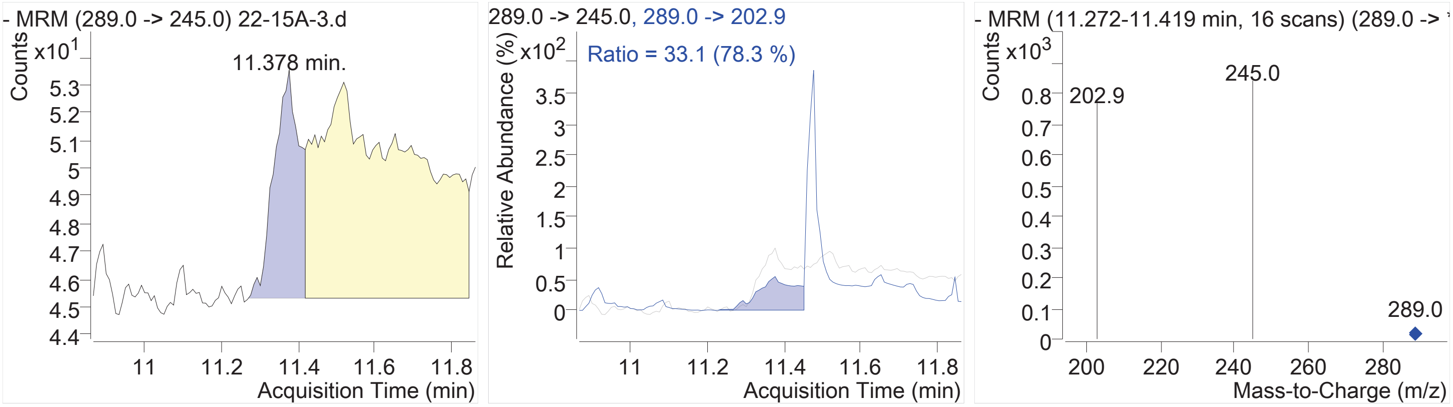

2,5-Dihydroxybenzoic acid

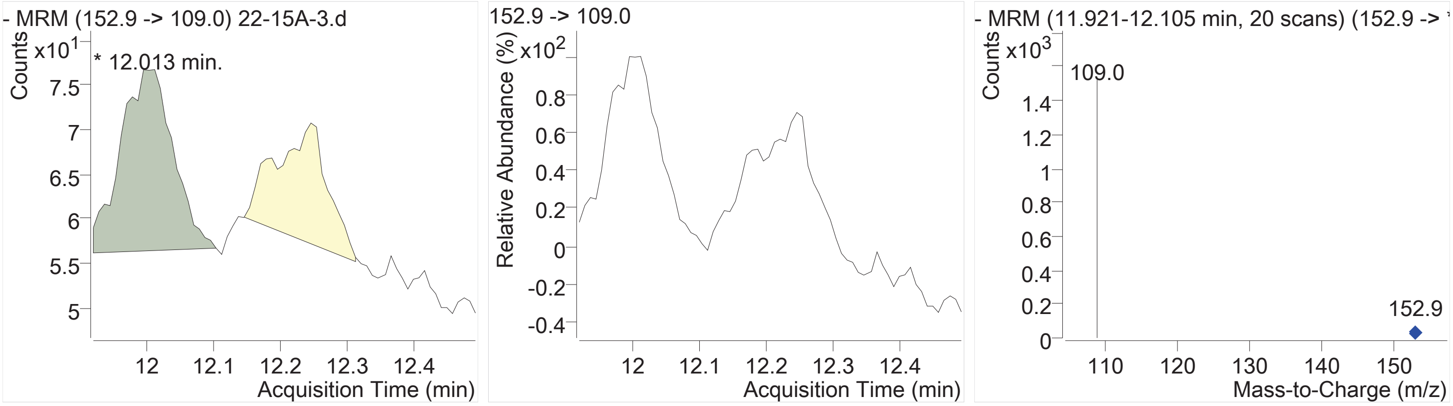

Chlorogenic acid

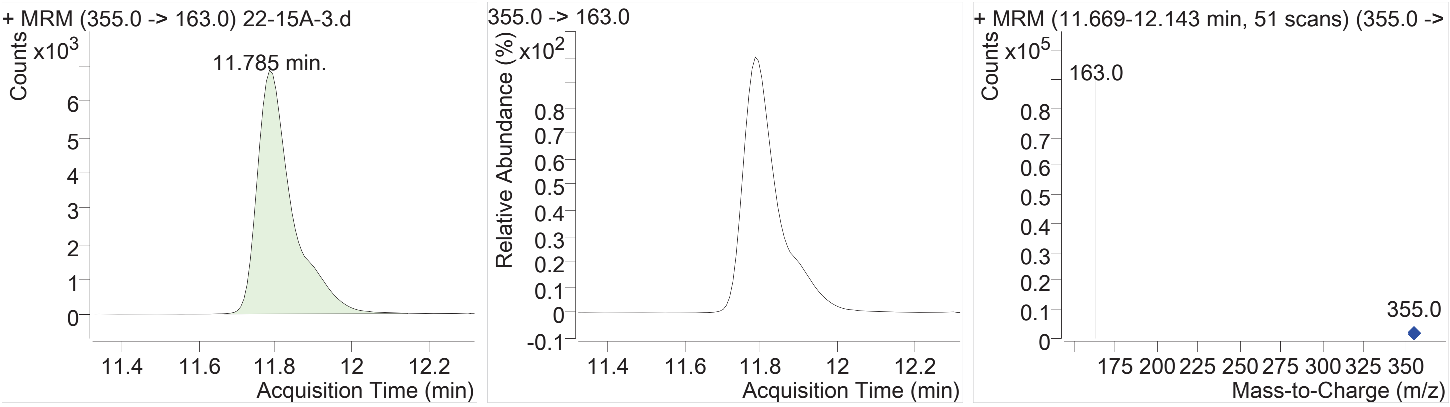

3-Hydroxybenzoic acid

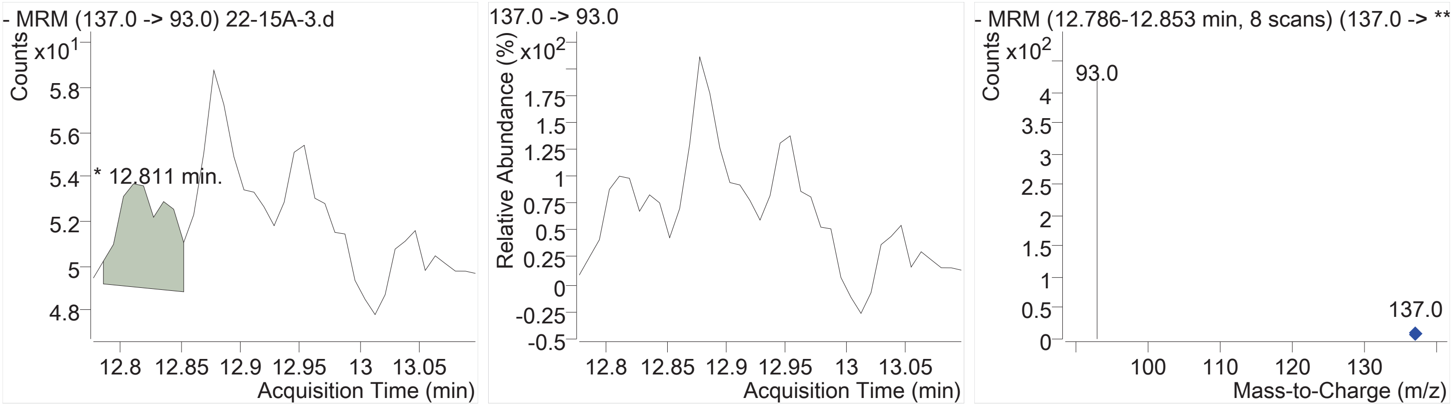

4-Hydroxybenzoic acid

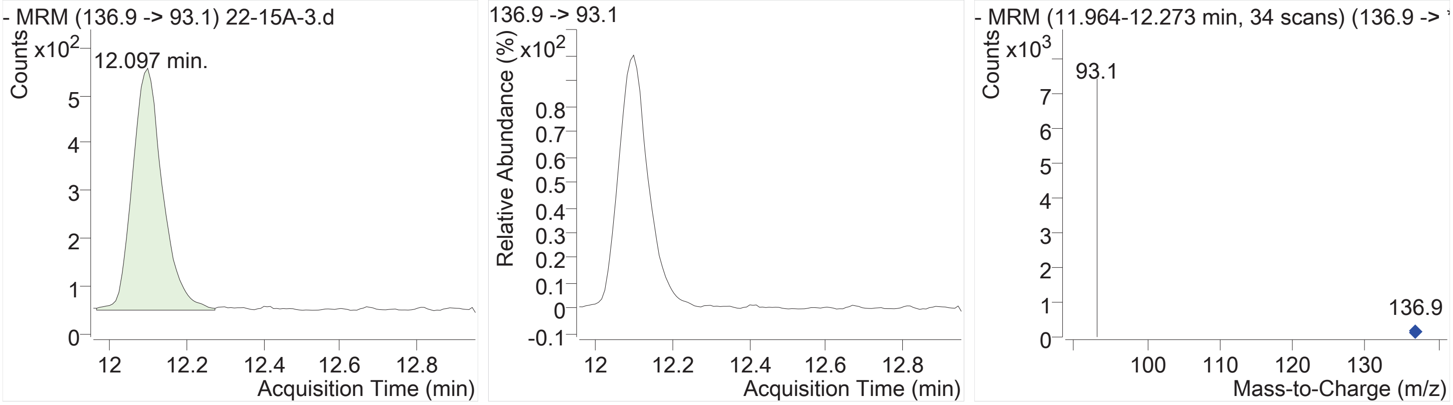

(-)-Epicatechin

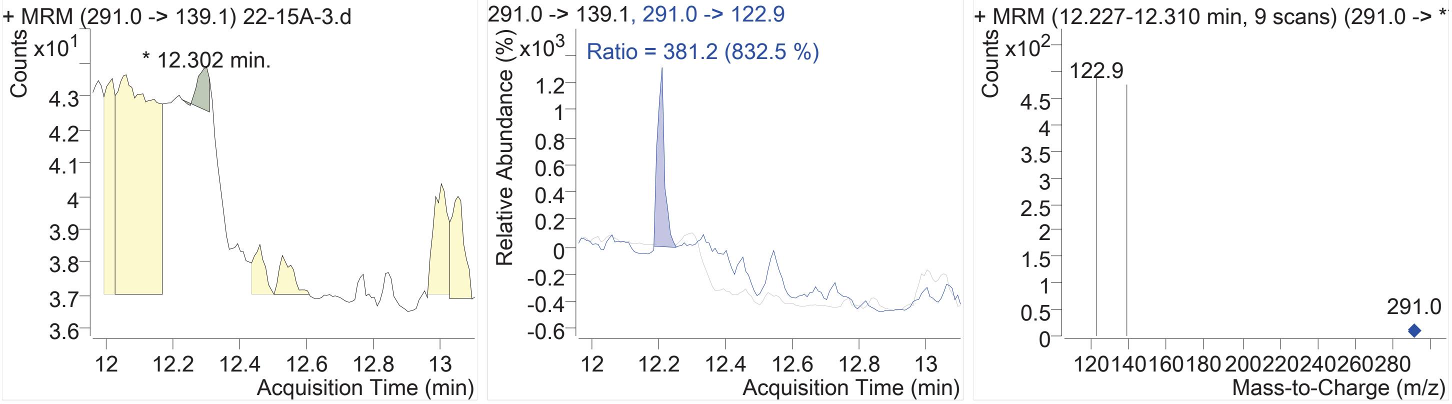

Caffeic acid

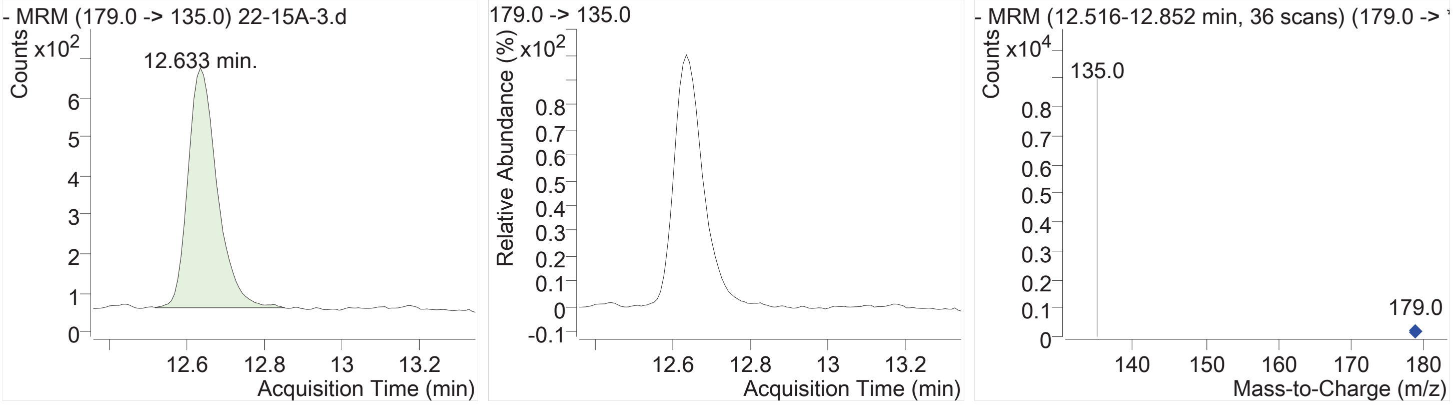

Syringic acid

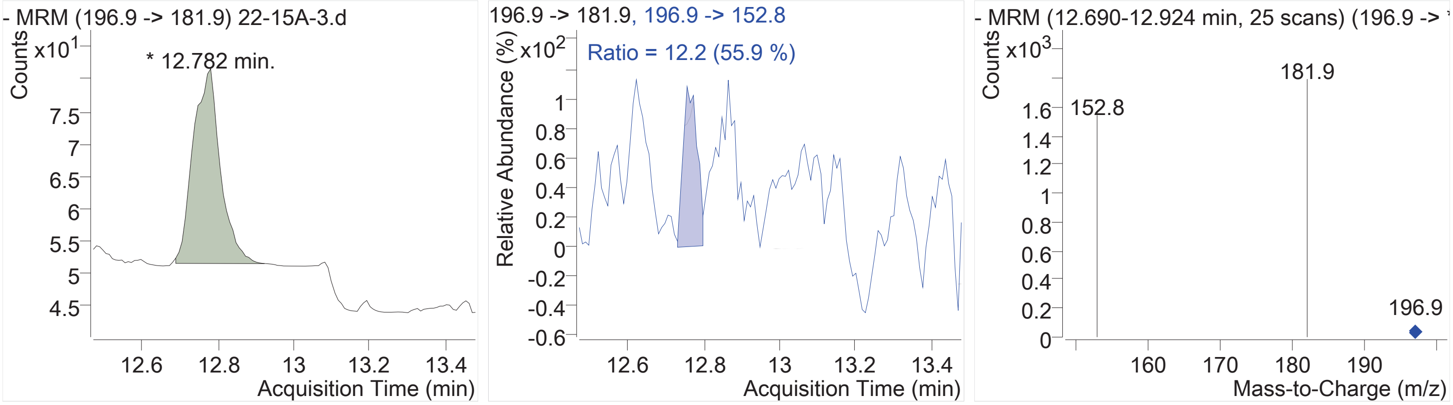

Vanillin

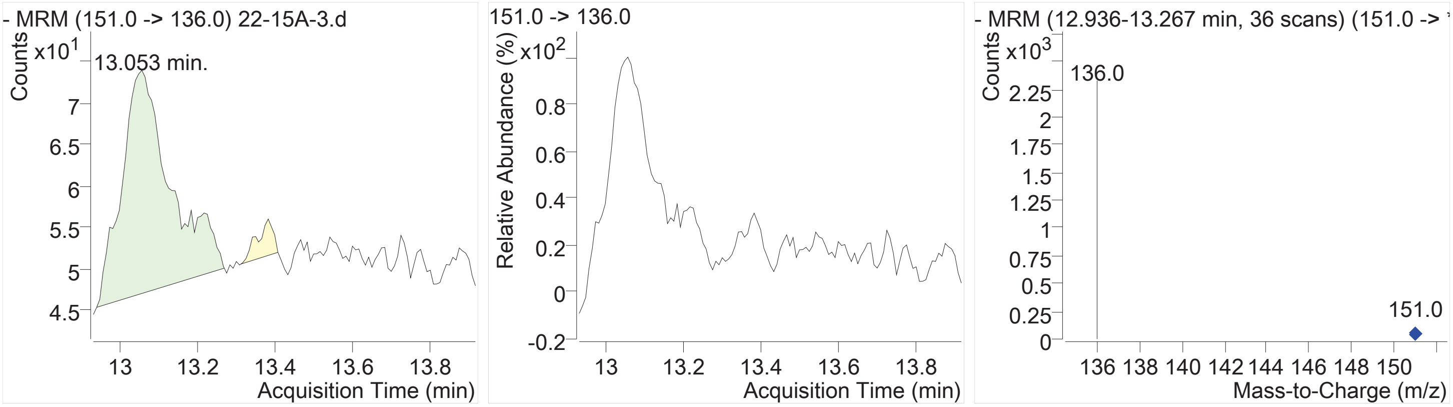

Verbascoside

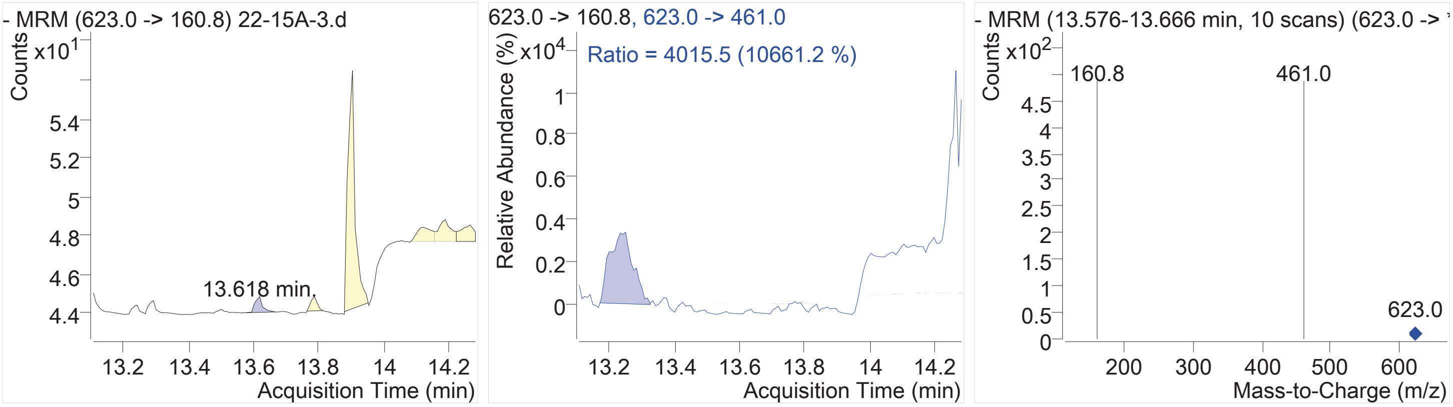

Taxifolin

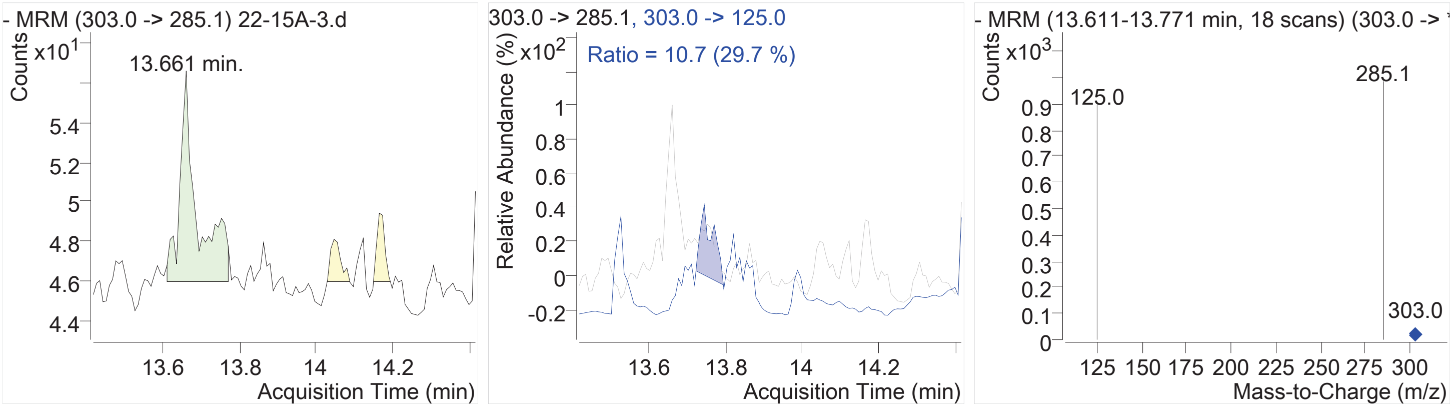

p-Coumaric acid

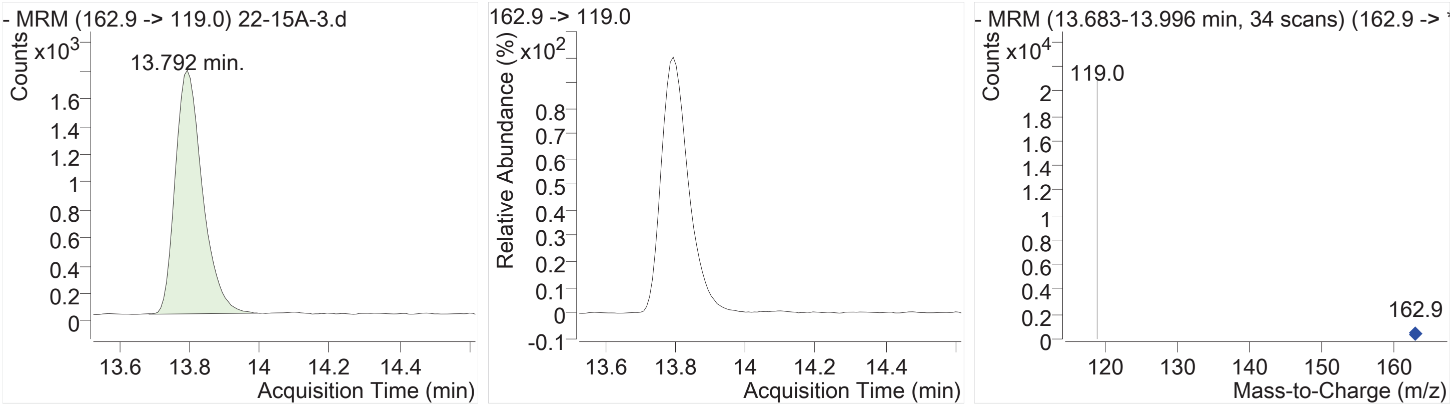

Sinapic acid

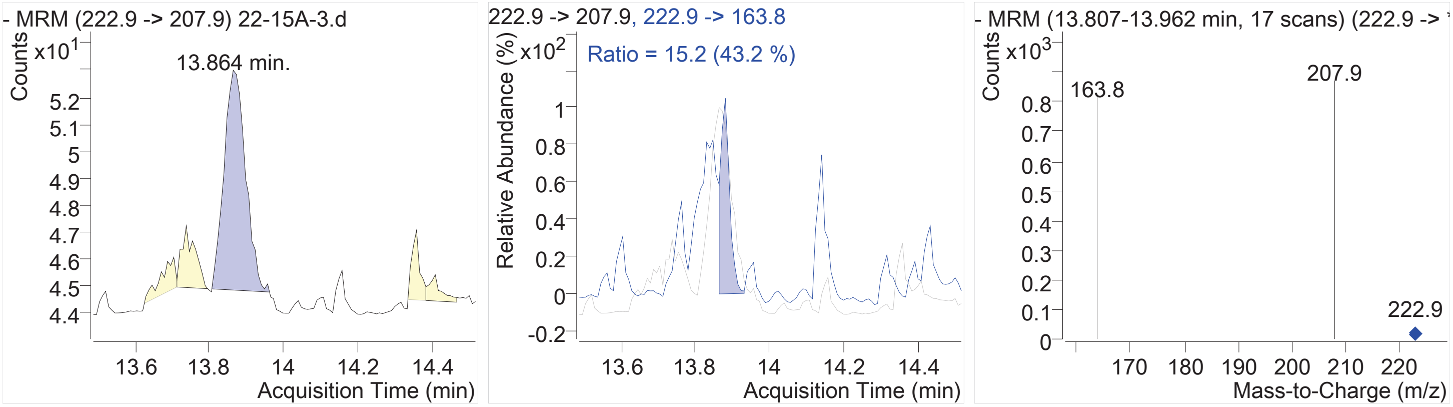

Ferulic acid

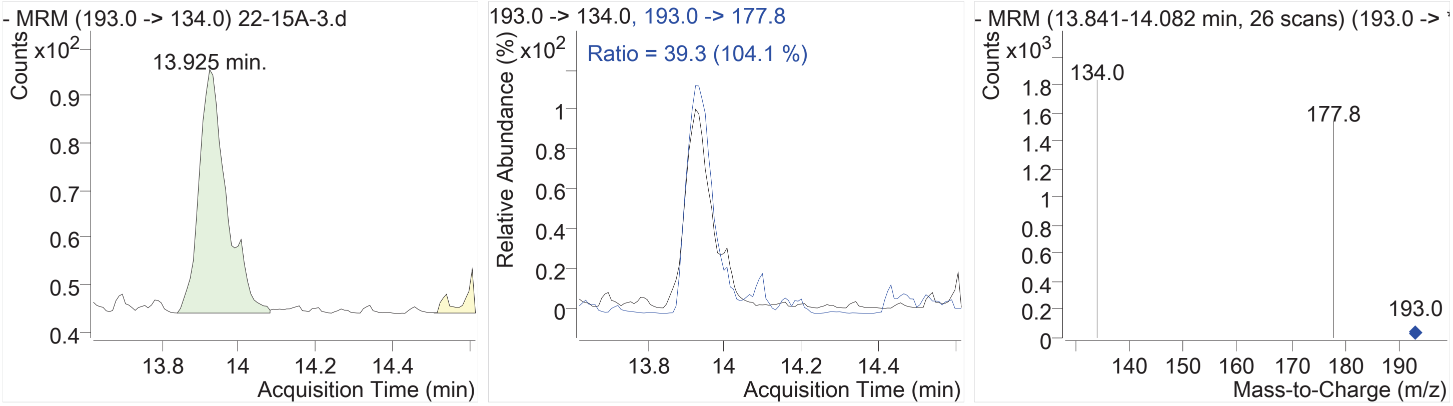

Luteolin 7-glucoside

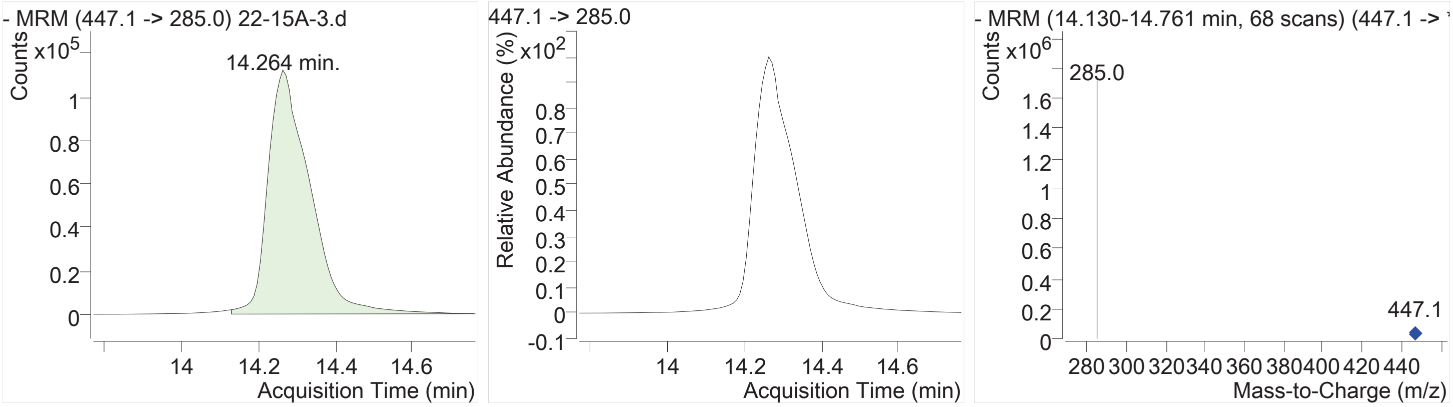

Hesperidin

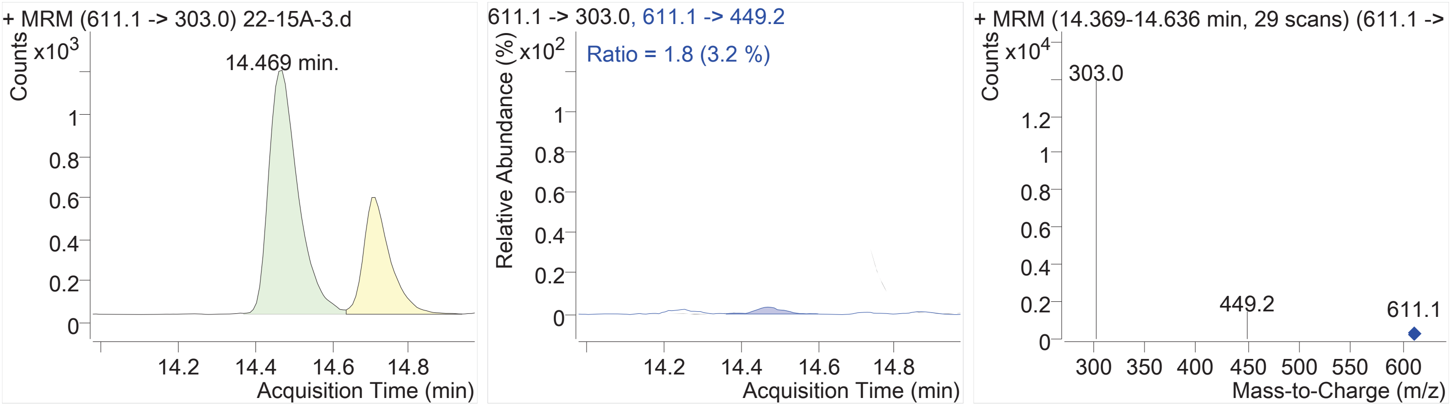

Hyperoside

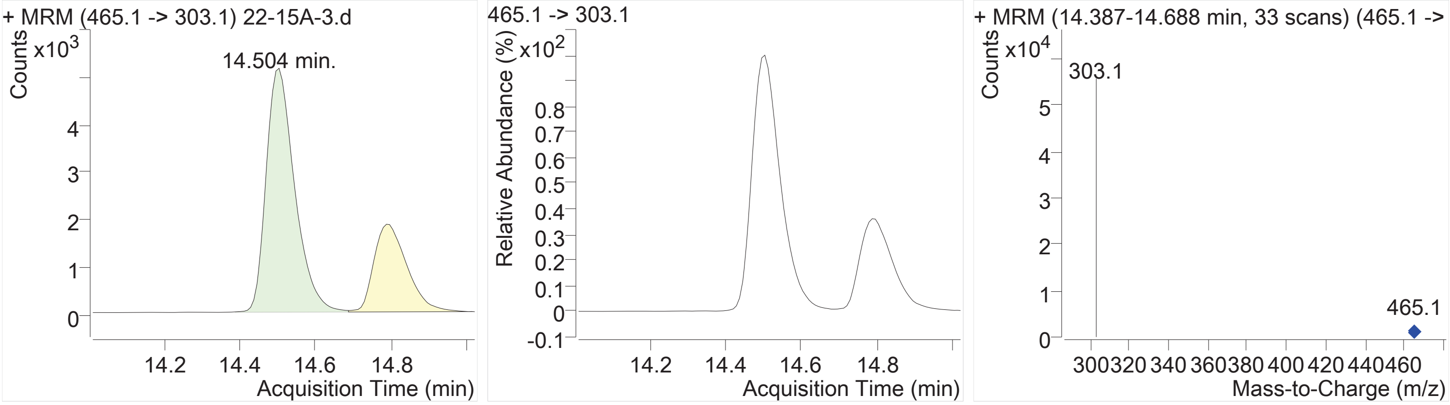

Rosmarinic acid

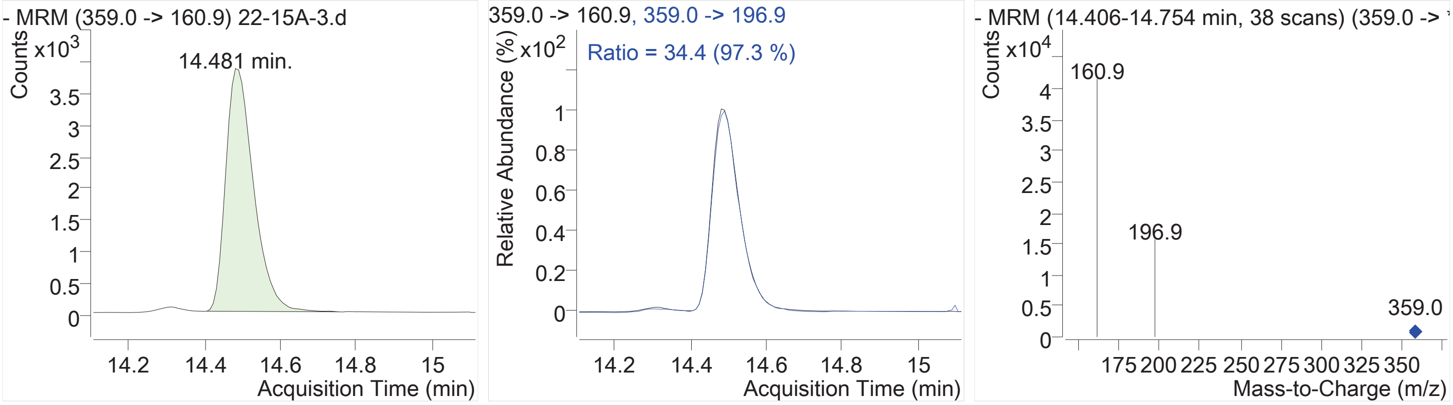

Apigenin 7-glucoside

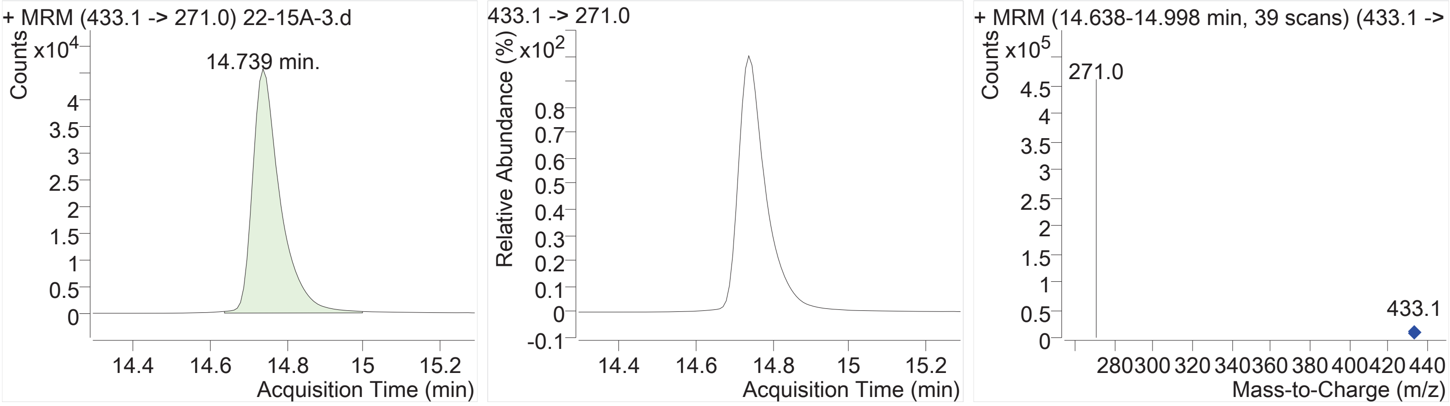

Pinoresinol

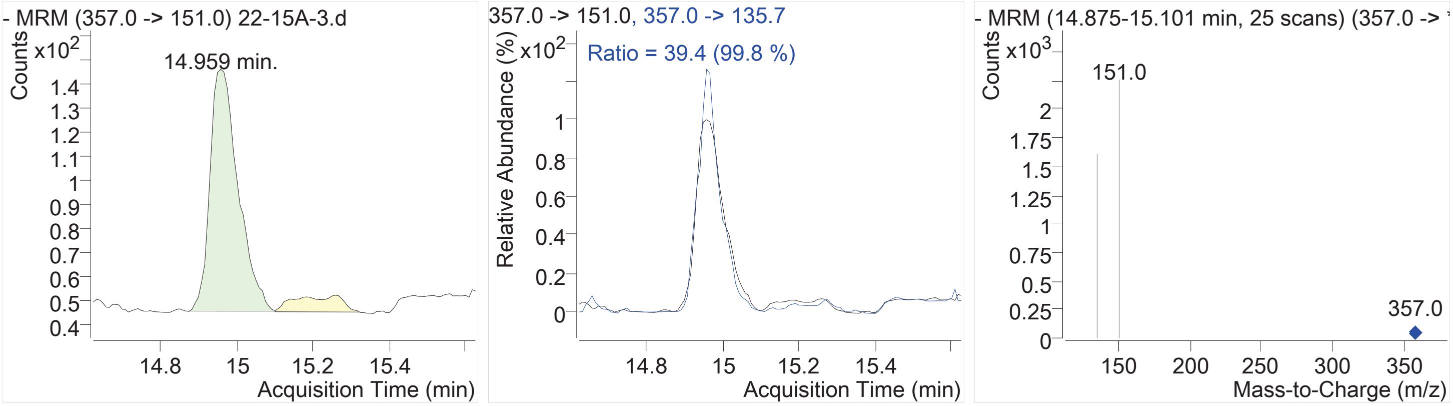

2-Hydroxycinnamic acid

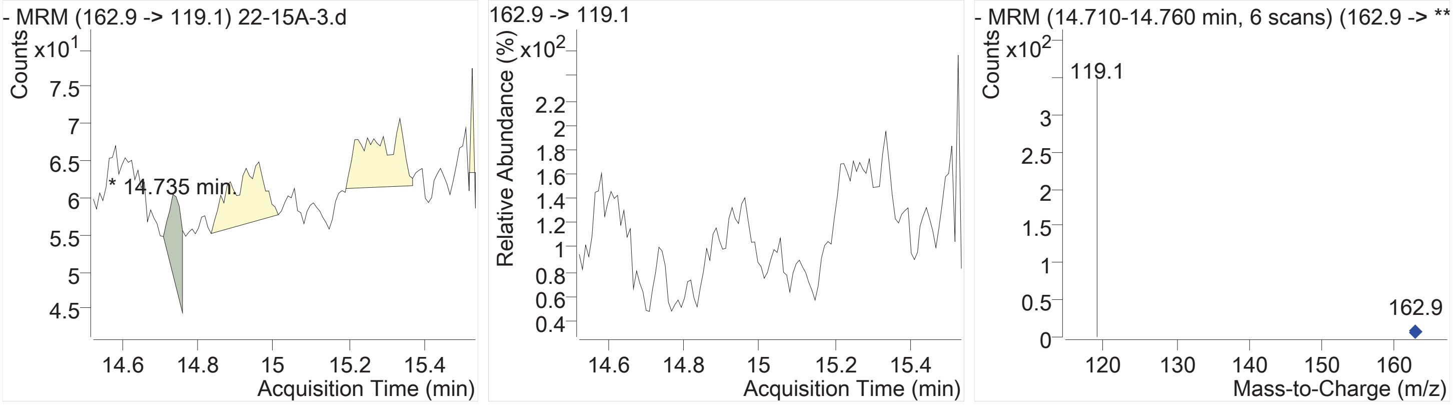

Eriodictyol

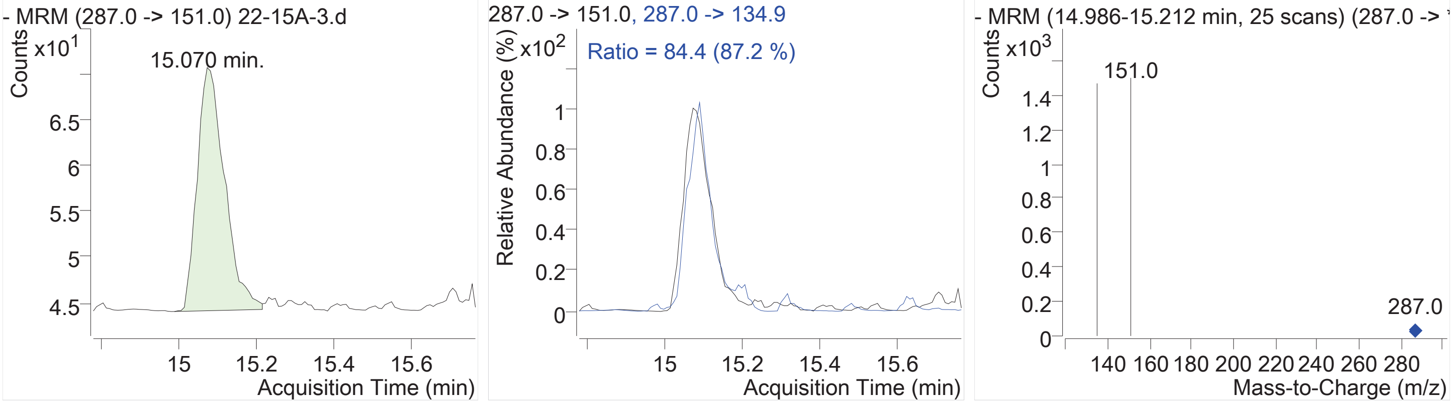

Quercetin

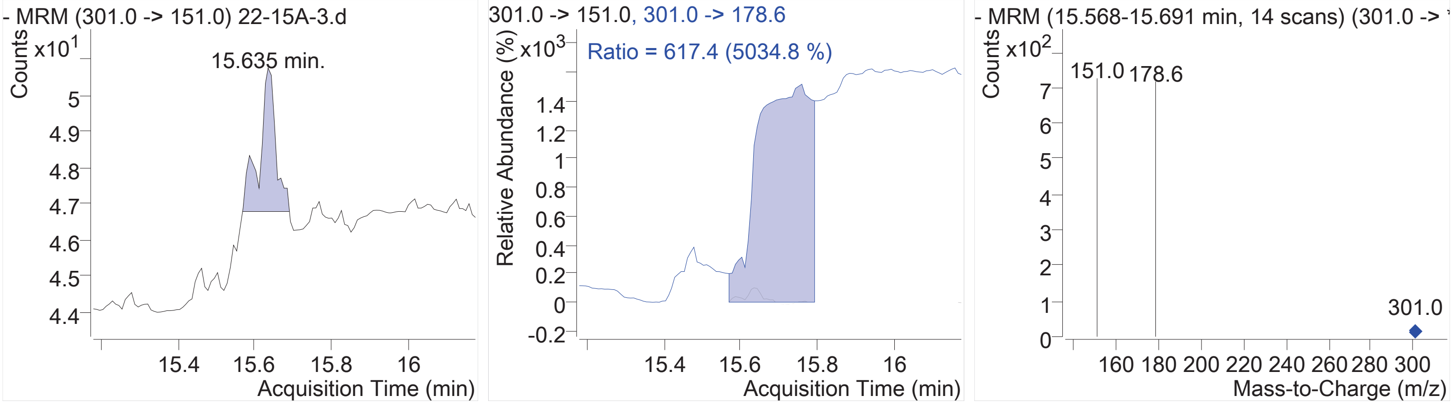

Luteolin

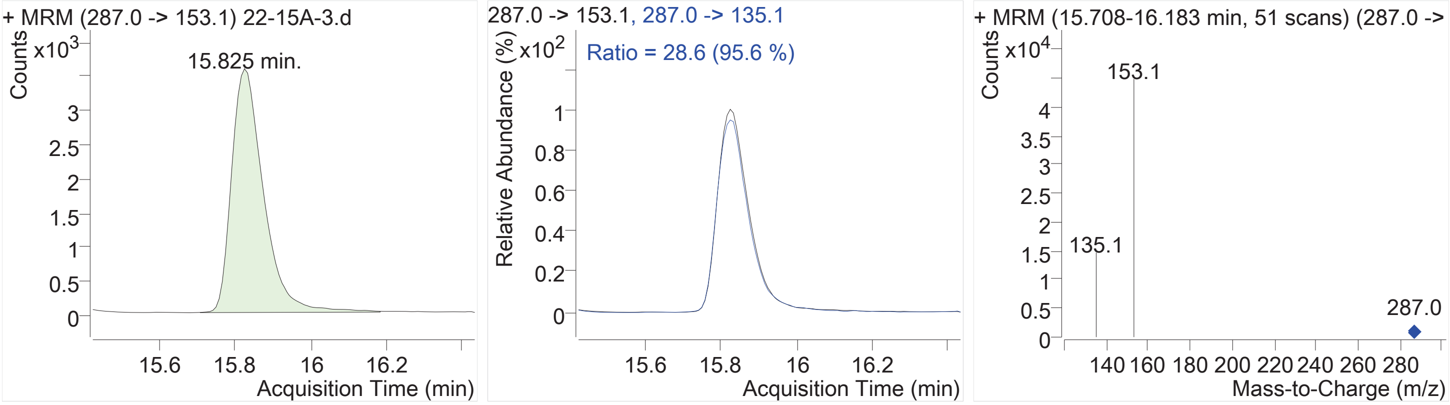

Kaempferol

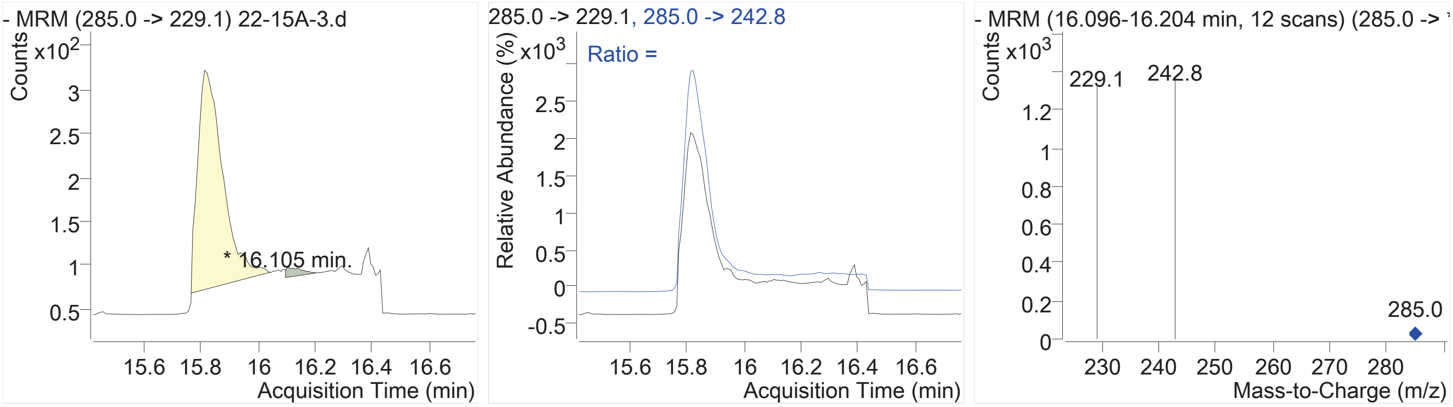

Apigenin

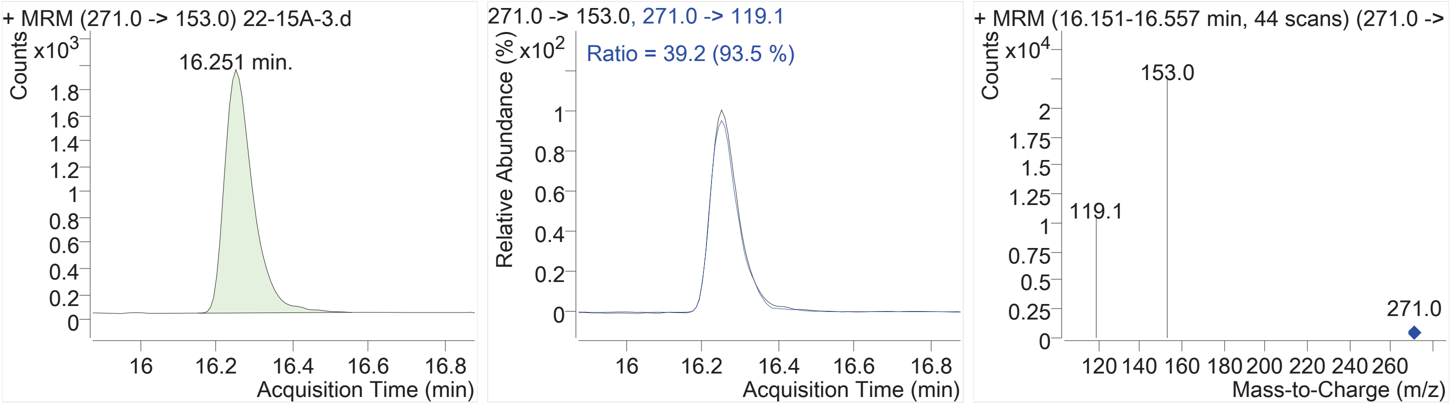

Quantitative Analysis Complete Report

Agilent Technologies

**Batch Path**	D:\MassHunter\Data\2022ekim\061022cengizhoca\QuantResults\071022.batch.bin		
**Analysis Time**	10/11/2022 1:33:26 PM	**Analyst Name**	Defam-PC\admin
**Report Time**	10/11/2022 1:39:29 PM	**Reporter Name**	admin
**Last Calib Update**	10/11/2022 1:33:17 PM	**Batch State**	Processed
**Quant Batch Version**	B.07.01	**Quant Report Version**	B.07.01

**Acq. Time**	10/8/2022 9:07:21 AM	**Data File**	22-15B-1.d
**Sample Type**	Sample	**Sample Name**	22-15B-1
**Dilution**	1	**Acq. Method**	FENOLIK_DMRM2021-31bilesen.m

Sample Chromatogram

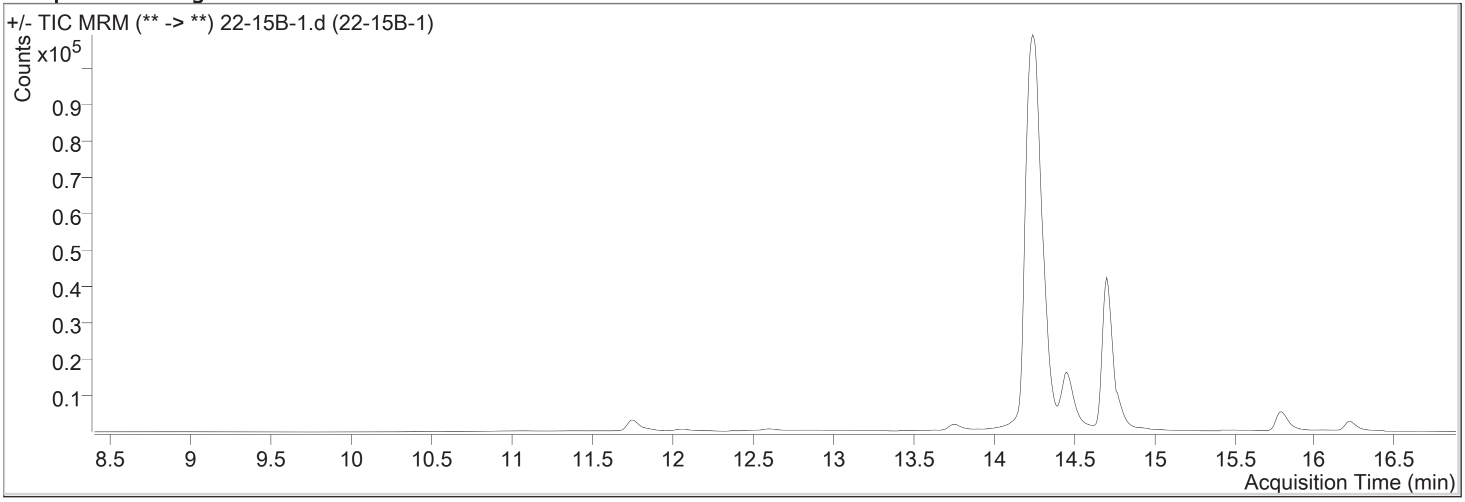

Compound	Transition	RT	Resp.	Final Conc	Units
Gallic acid	168.9 -> 125.0	8.716	2	ND	ng/ml
Protocatechuic acid	152.9 -> 108.9	10.551	522	38.7392	ng/ml
Pyrocatechol	109.0 -> 52.9	10.772	3	ND	ng/ml
3,4-Dihydroxyphenylacetic acid	167.0 -> 123.0	10.954	6	ND	ng/ml
(+)-Catechin	289.0 -> 245.0	11.186	12	ND	ng/ml
2,5-Dihydroxybenzoic acid	152.9 -> 109.0	11.963	60	9.9936	ng/ml
Chlorogenic acid	355.0 -> 163.0	11.760	19375	915.2301	ng/ml
3-Hydroxybenzoic acid	137.0 -> 93.0	12.778	9	16.6615	ng/ml
4-Hydroxybenzoic acid	136.9 -> 93.1	12.064	2510	217.4602	ng/ml
(-)-Epicatechin	291.0 -> 139.1	12.277	9	4.2483	ng/ml
Caffeic acid	179.0 -> 135.0	12.608	2148	63.7779	ng/ml
Syringic acid	196.9 -> 181.9	12.740	150	212.3293	ng/ml
Vanillin	151.0 -> 136.0	13.028	192	36.5526	ng/ml
Verbascoside	623.0 -> 160.8	14.154	75	ND	ng/ml
Taxifolin	303.0 -> 285.1	13.695	19	ND	ng/ml
p-Coumaric acid	162.9 -> 119.0	13.758	8401	318.6026	ng/ml
Sinapic acid	222.9 -> 207.9	13.814	47	19.1009	ng/ml
Ferulic acid	193.0 -> 134.0	13.908	221	42.5636	ng/ml
Luteolin 7-glucoside	447.1 -> 285.0	14.247	799450	8749.5163	ng/ml
Hesperidin	611.1 -> 303.0	14.436	5185	633.4380	ng/ml
Hyperoside	465.1 -> 303.1	14.470	20155	1024.0287	ng/ml
Rosmarinic acid	359.0 -> 160.9	14.456	32002	2557.8949	ng/ml
Apigenin 7-glucoside	433.1 -> 271.0	14.713	194854	5793.7795	ng/ml
Pinoresinol	357.0 -> 151.0	14.942	505	1964.9871	ng/ml
2-Hydroxycinnamic acid	162.9 -> 119.1	15.155	6	ND	ng/ml
Eriodictyol	287.0 -> 151.0	15.045	160	ND	ng/ml
Quercetin	301.0 -> 151.0	15.569	104	ND	ng/ml
Luteolin	287.0 -> 153.1	15.800	18931	1191.0294	ng/ml
Kaempferol	285.0 -> 229.1	16.096	43	ND	ng/ml

Quantitative Analysis Complete Report

Agilent Technologies

Compound	Transition	RT	Resp.	Final Conc	Units
Apigenin	271.0 -> 153.0	16.226	9343	499.8910	ng/ml

Gallic acid

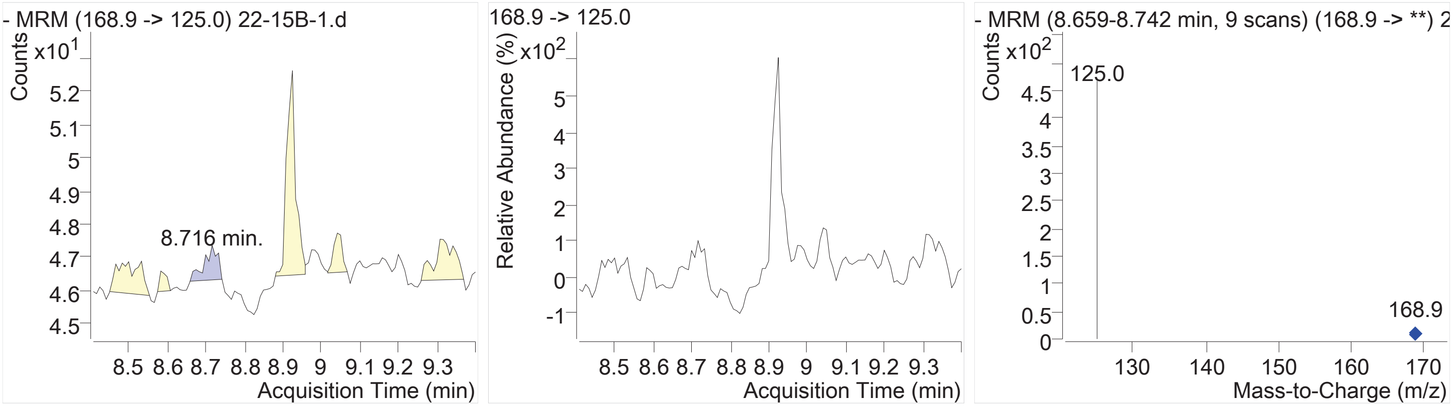

Protocatechuic acid

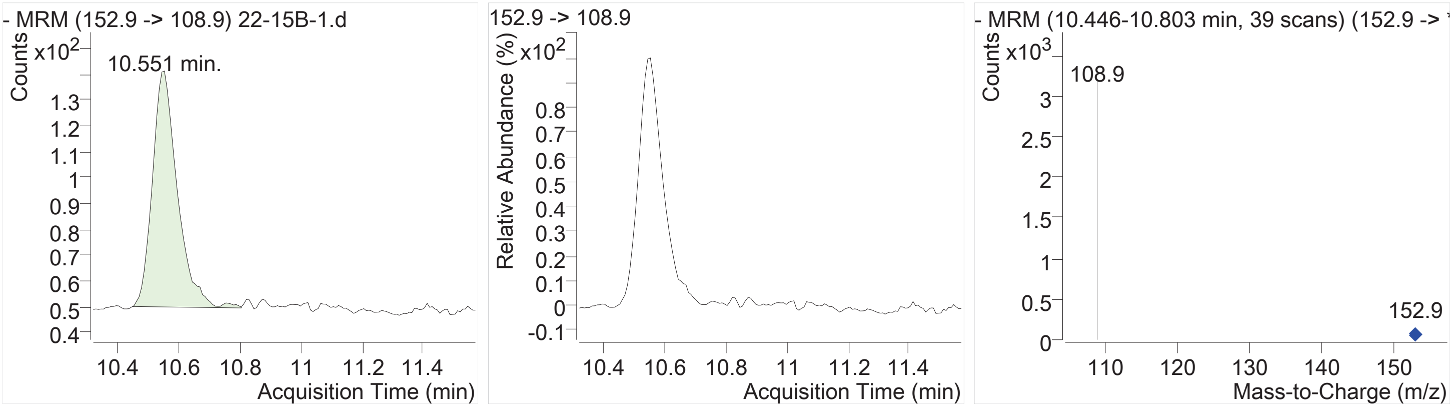

Pyrocatechol

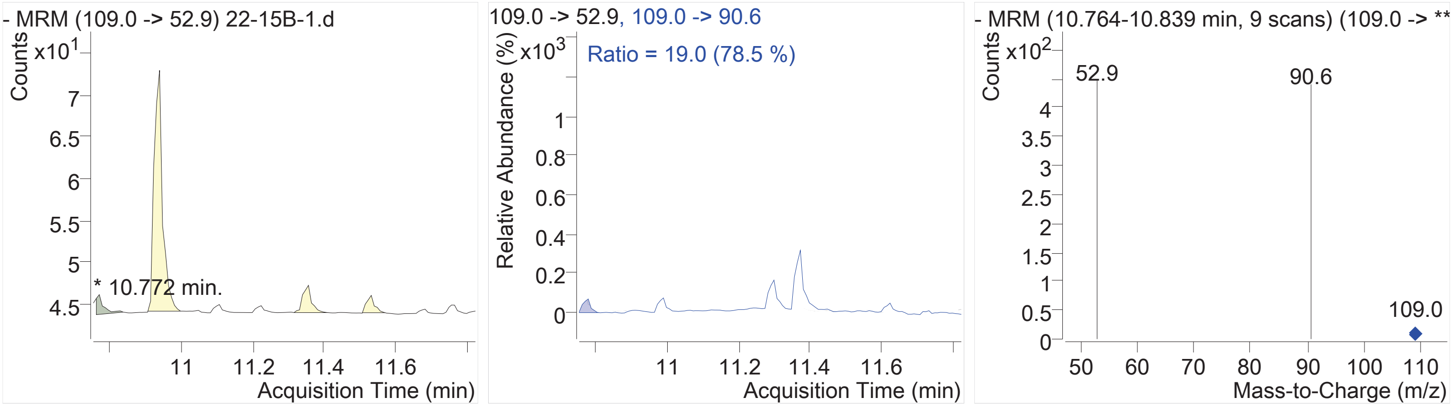

3,4-Dihydroxyphenylacetic acid

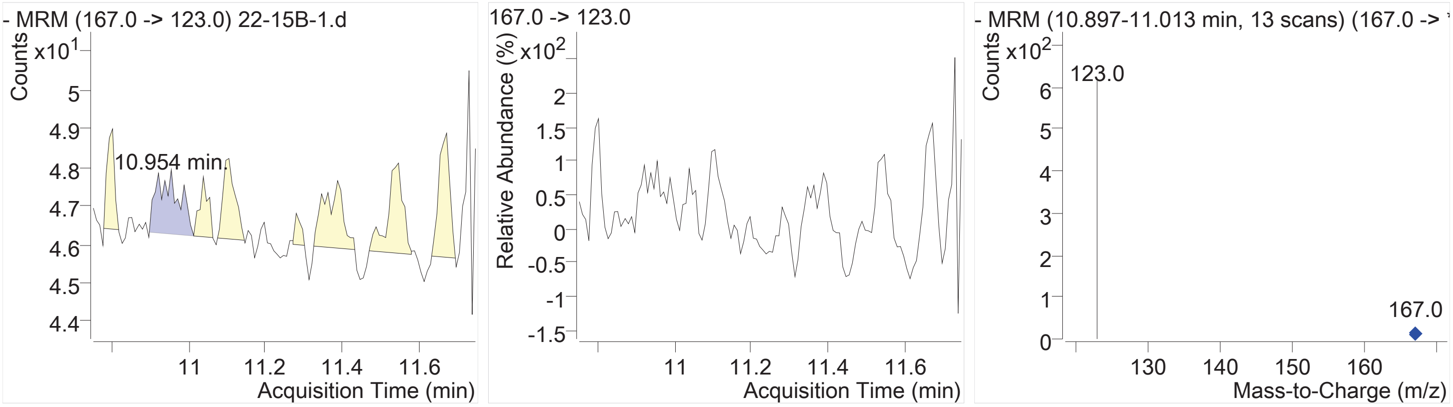

(+)-Catechin

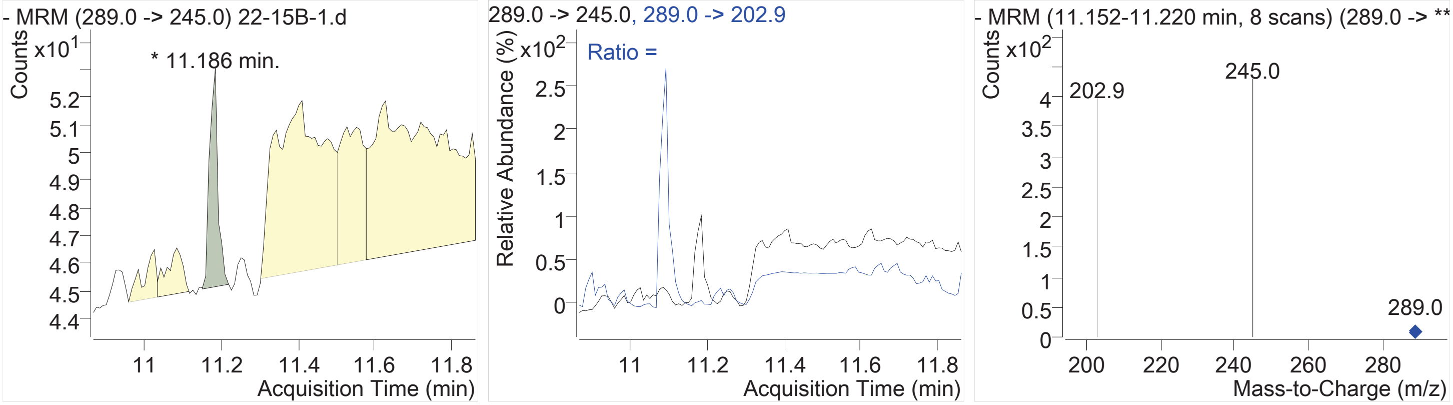

2,5-Dihydroxybenzoic acid

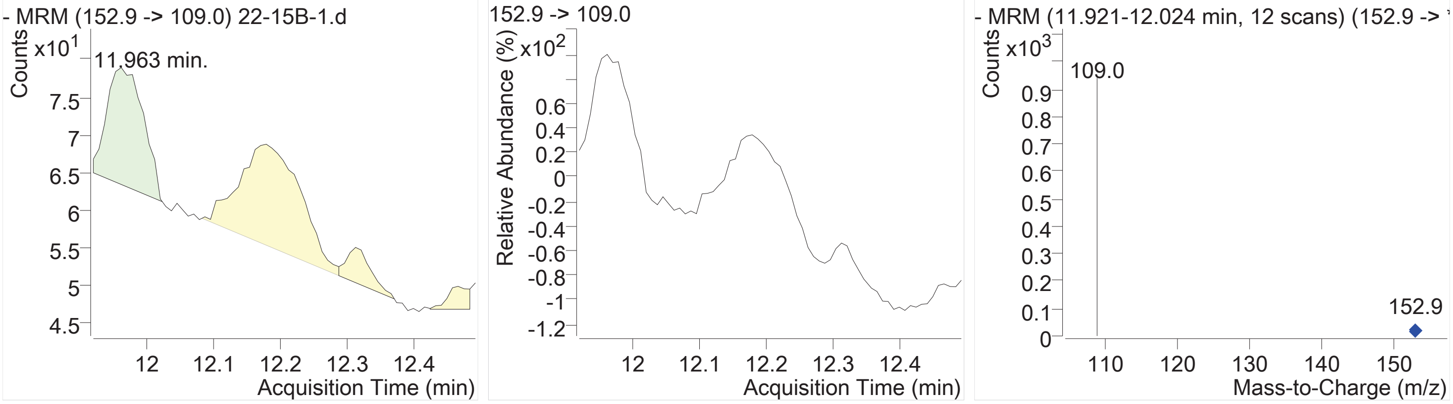

Chlorogenic acid

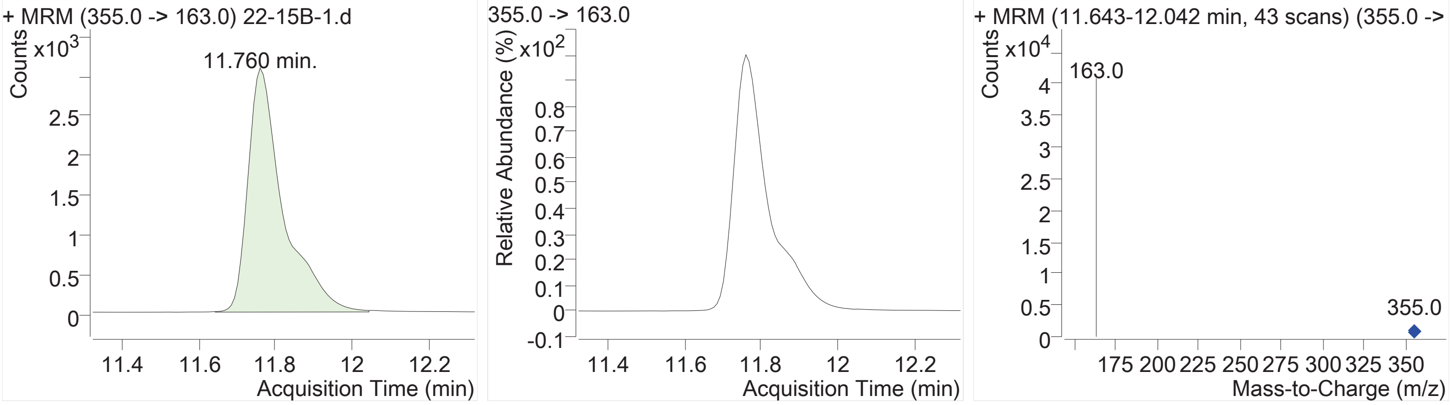

3-Hydroxybenzoic acid

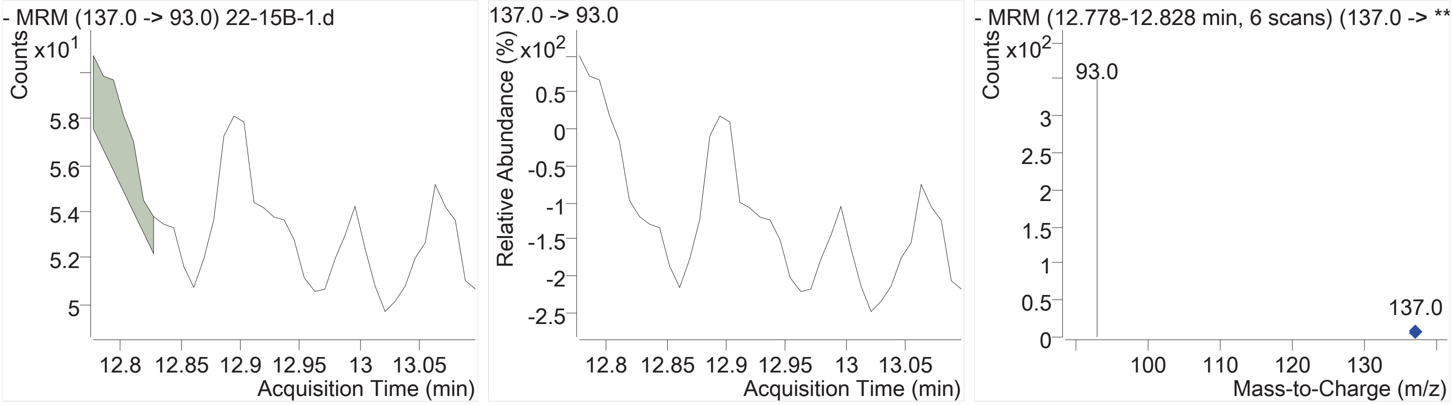

4-Hydroxybenzoic acid

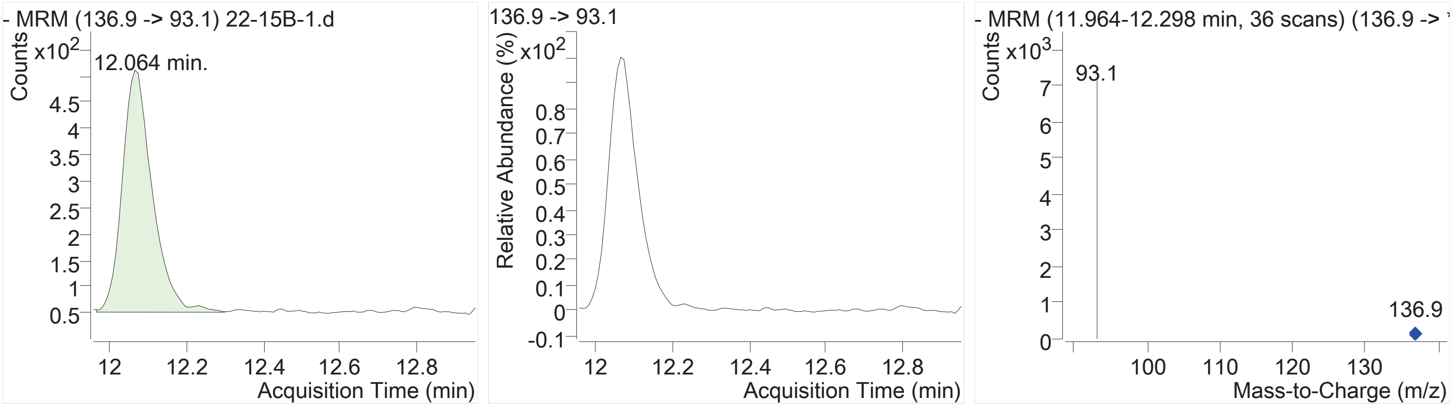

(-)-Epicatechin

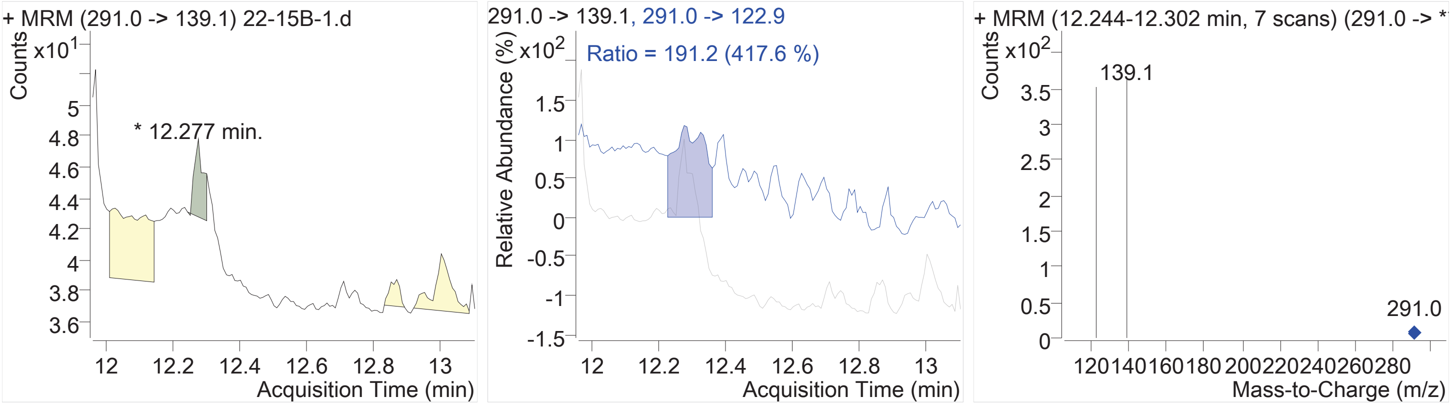

Caffeic acid

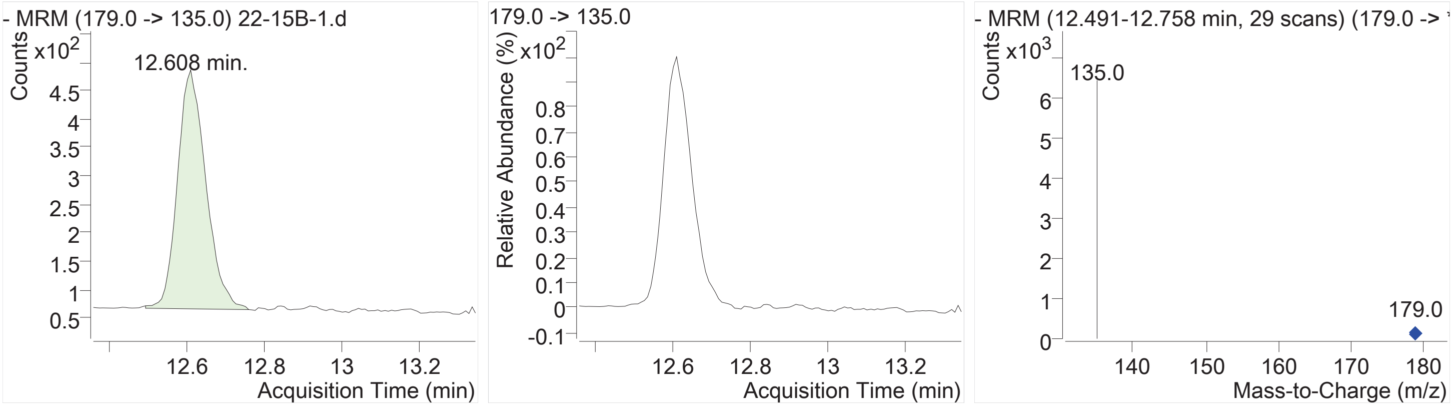

Syringic acid

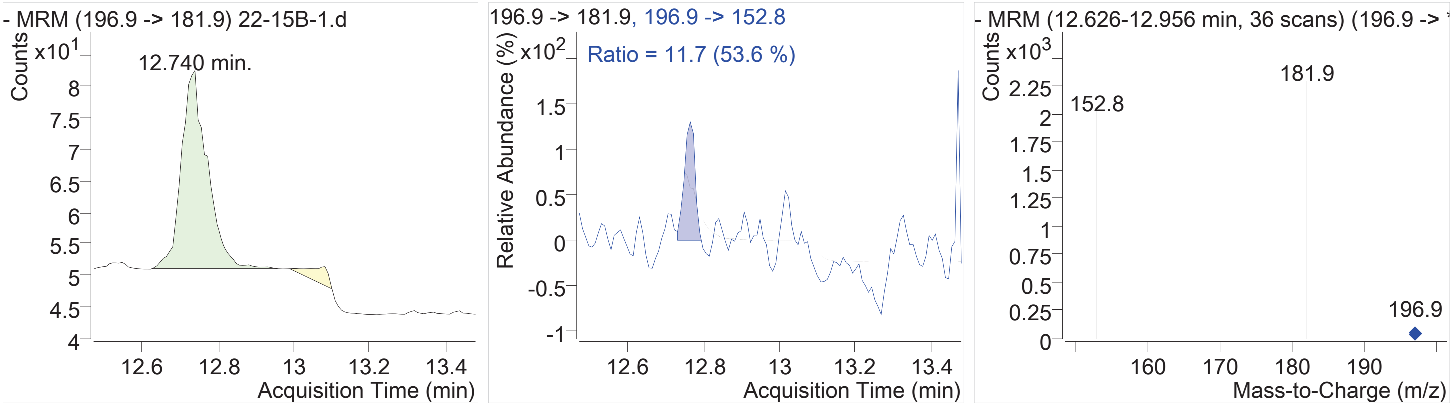

Vanillin

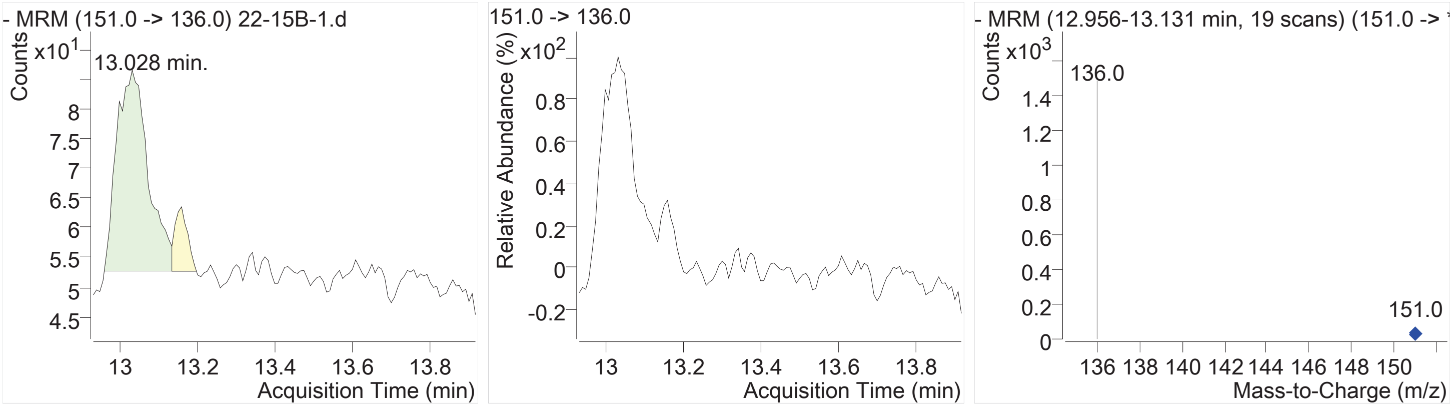

Verbascoside

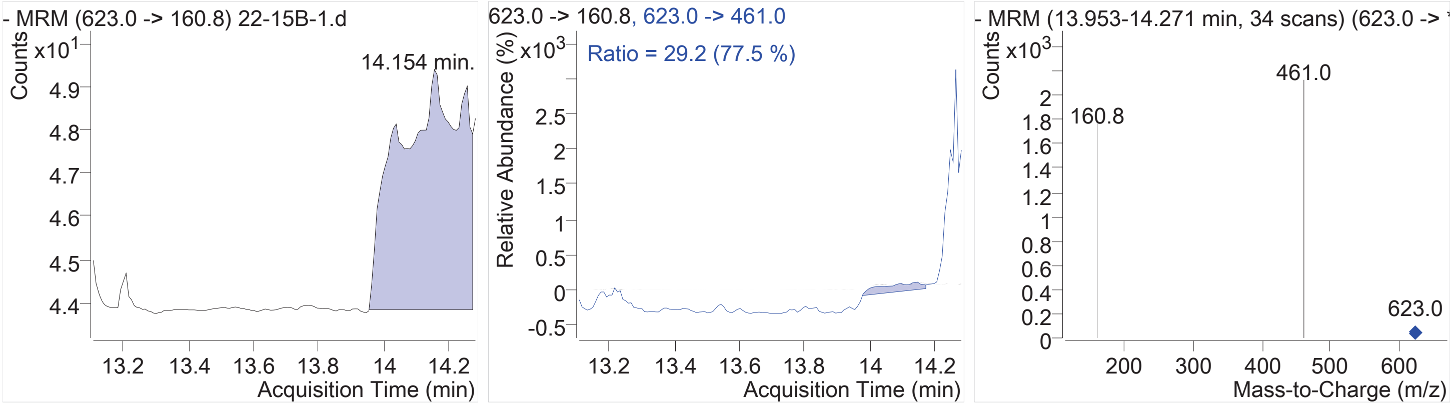

Taxifolin

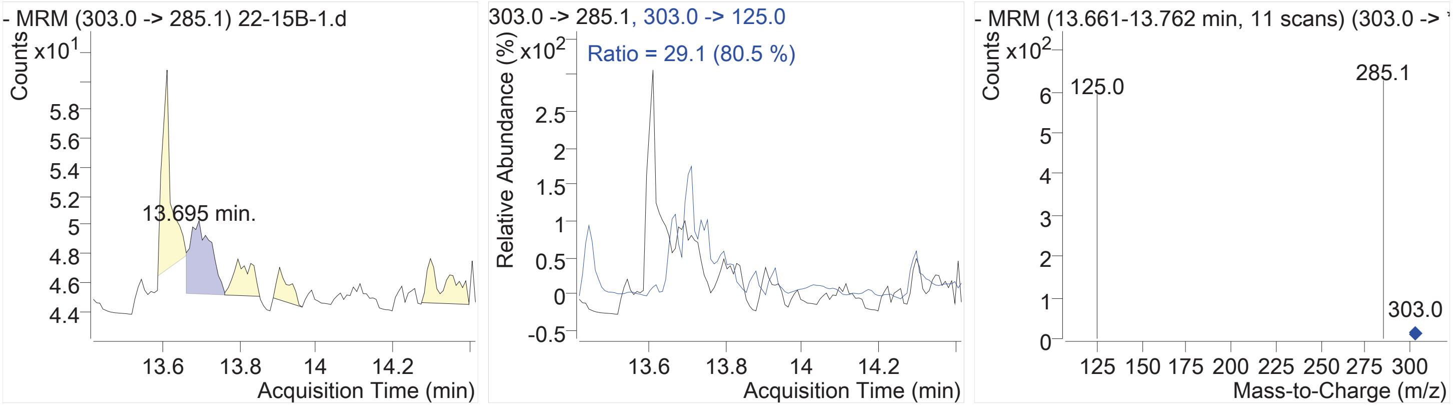

p-Coumaric acid

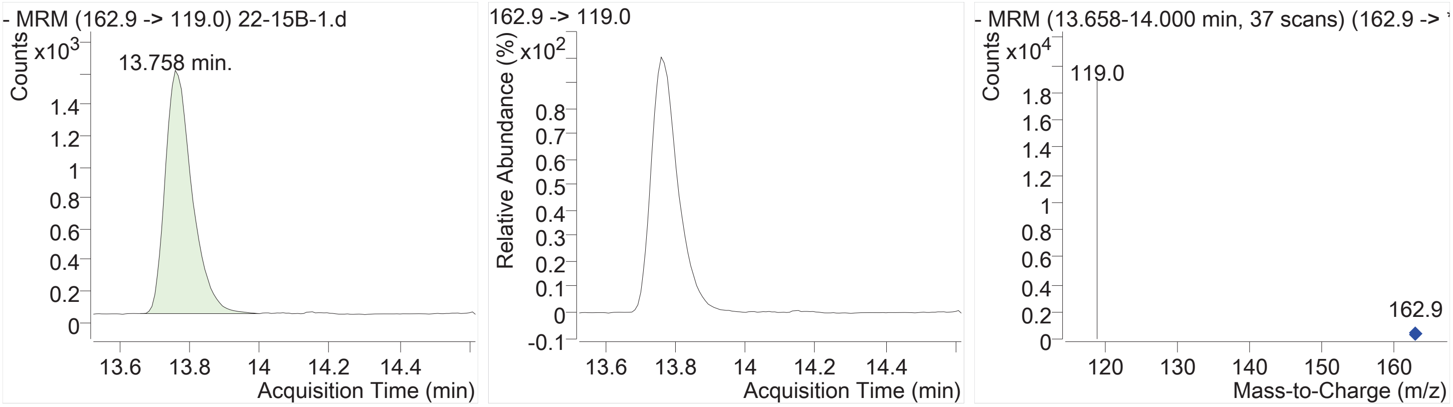

Sinapic acid

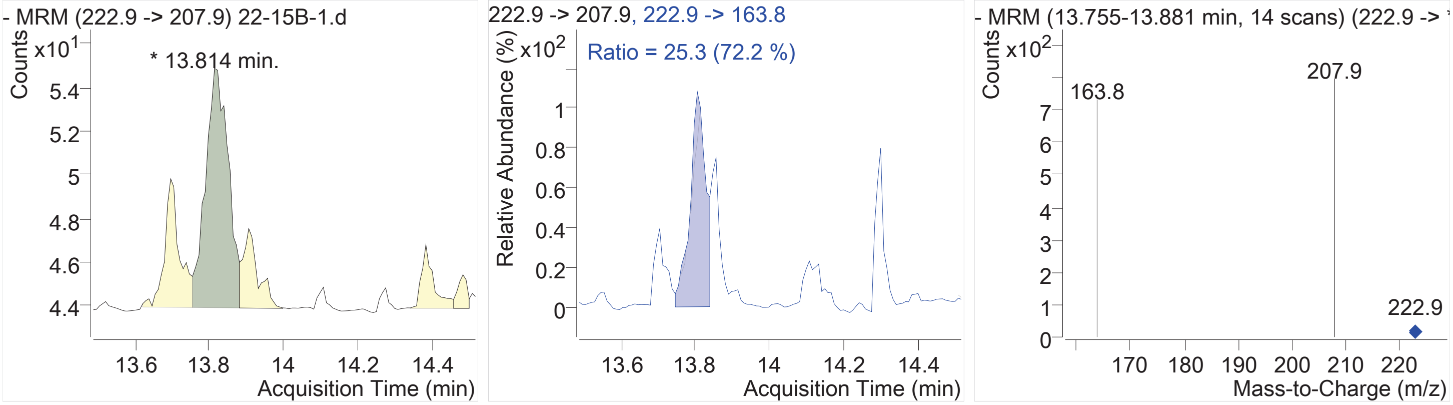

Ferulic acid

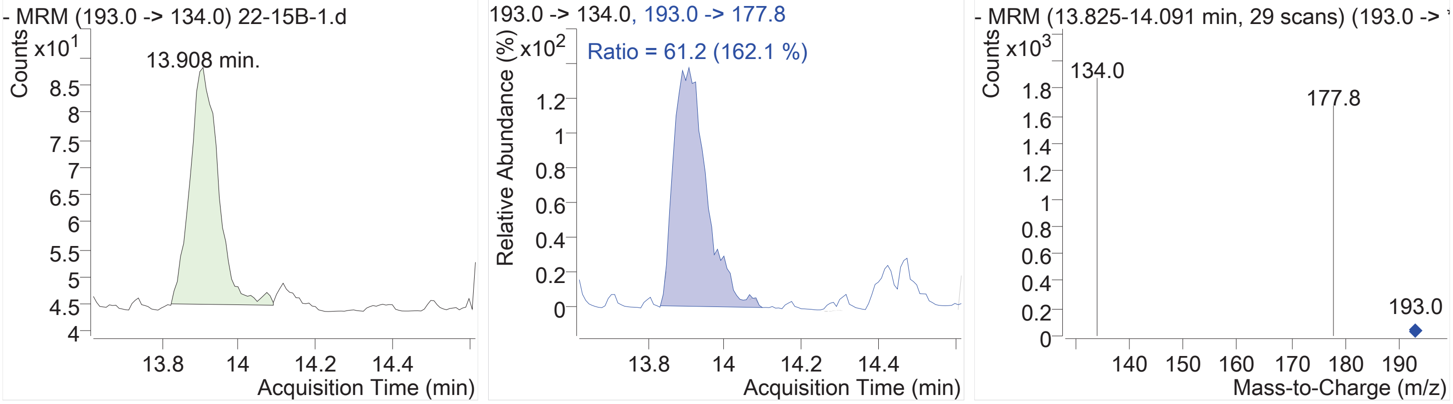

Luteolin 7-glucoside

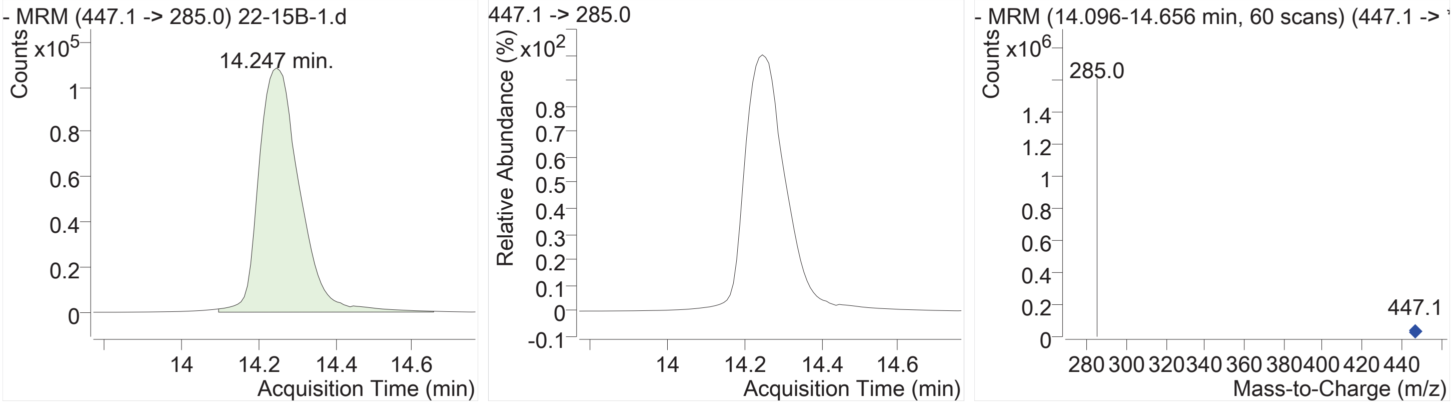

Hesperidin

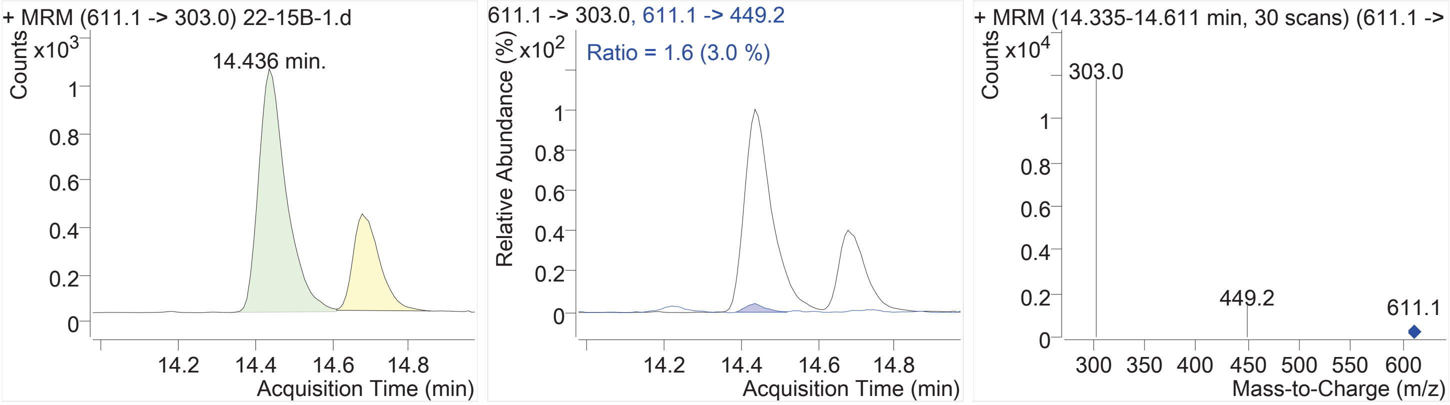

Hyperoside

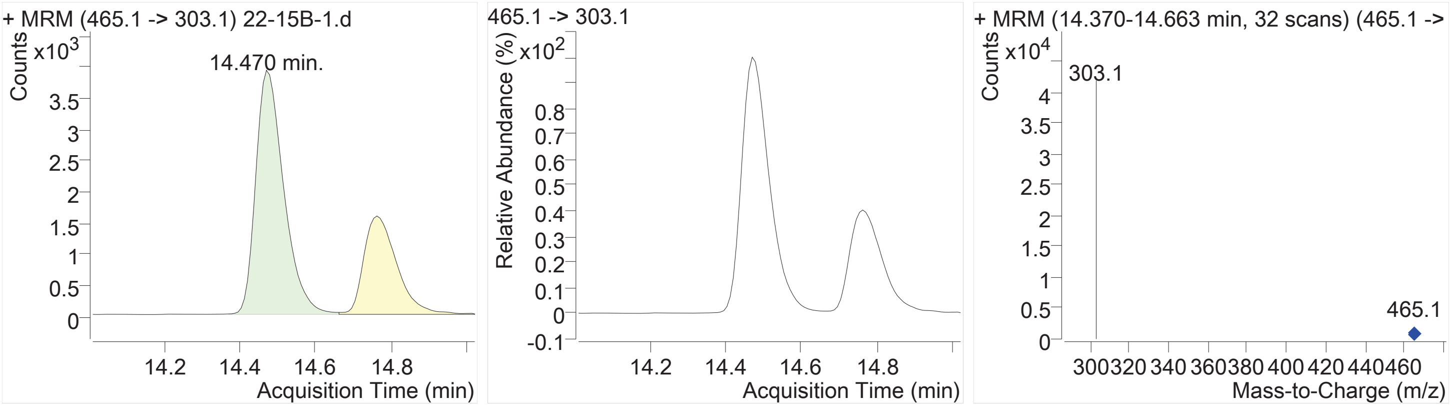

Rosmarinic acid

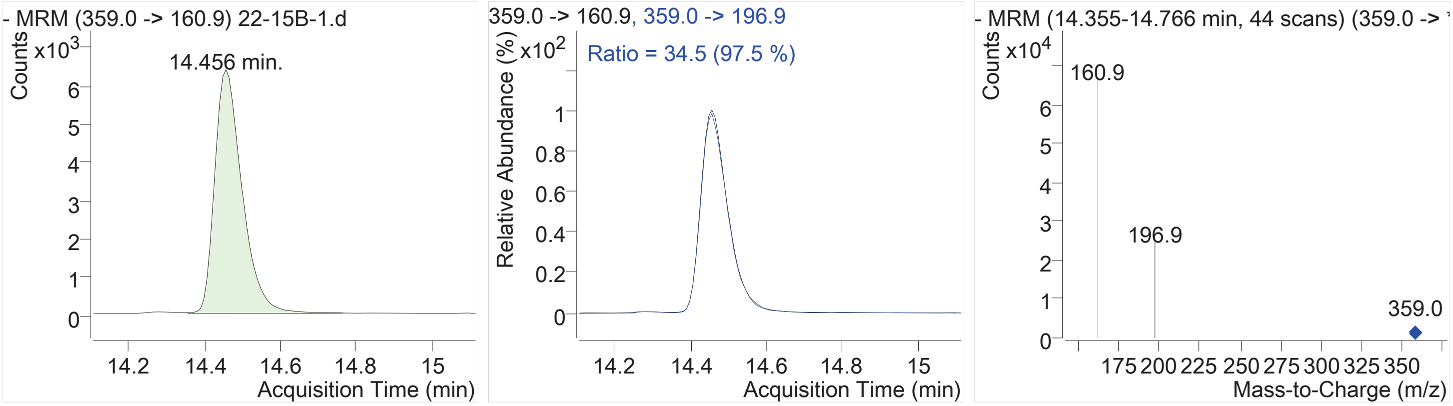

Apigenin 7-glucoside

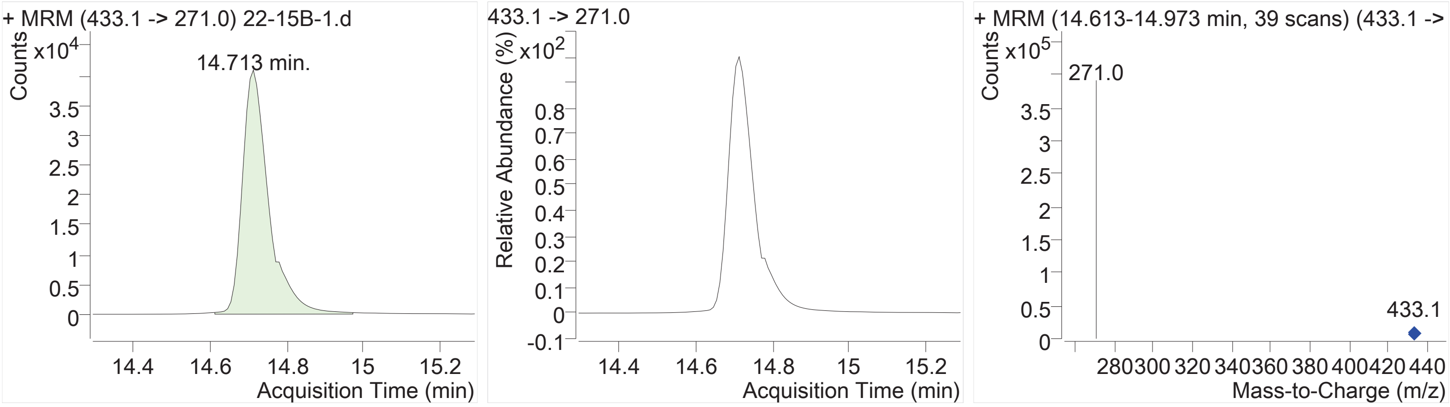

Pinoresinol

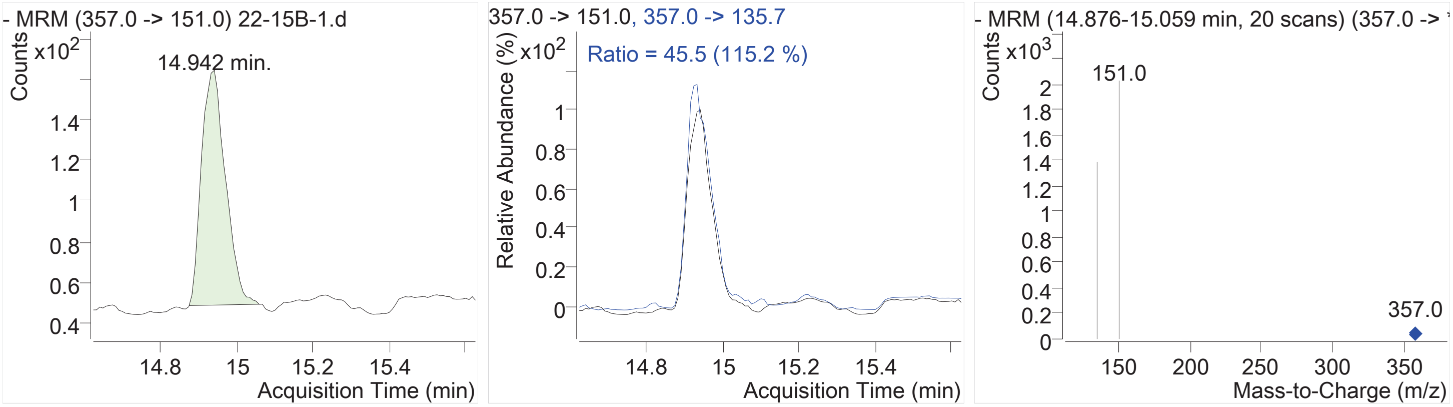

2-Hydroxycinnamic acid

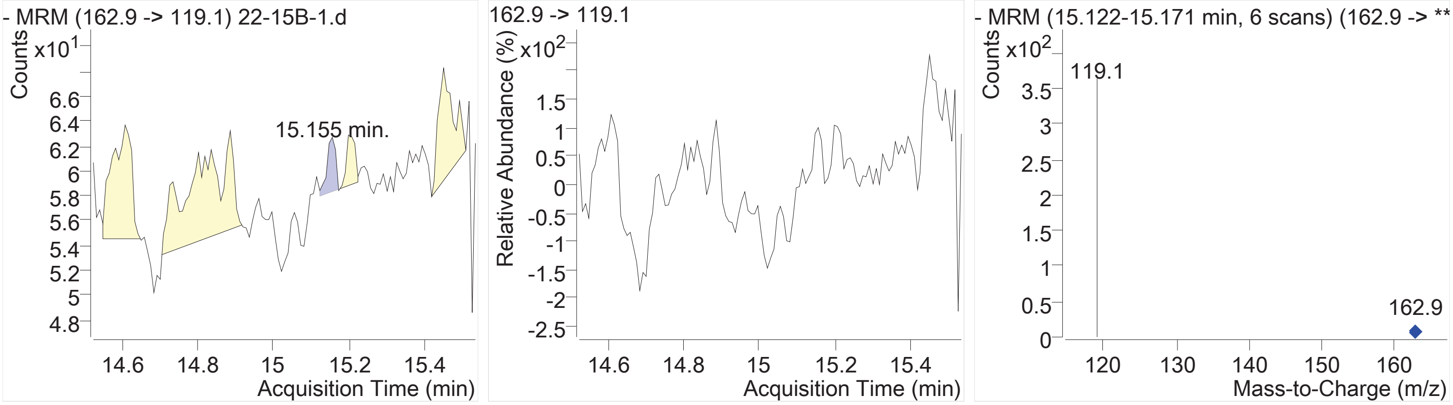

Eriodictyol

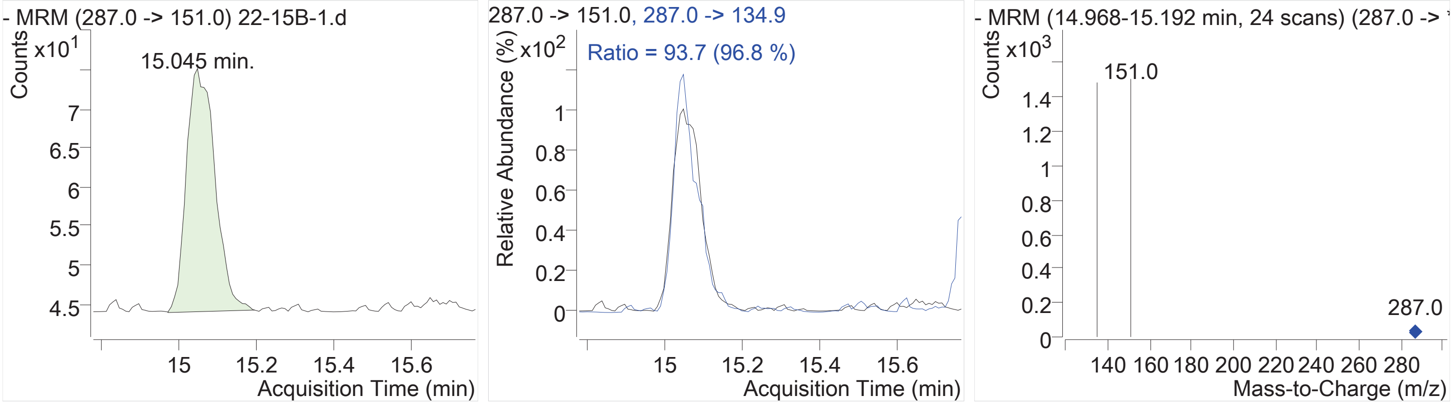

Quercetin

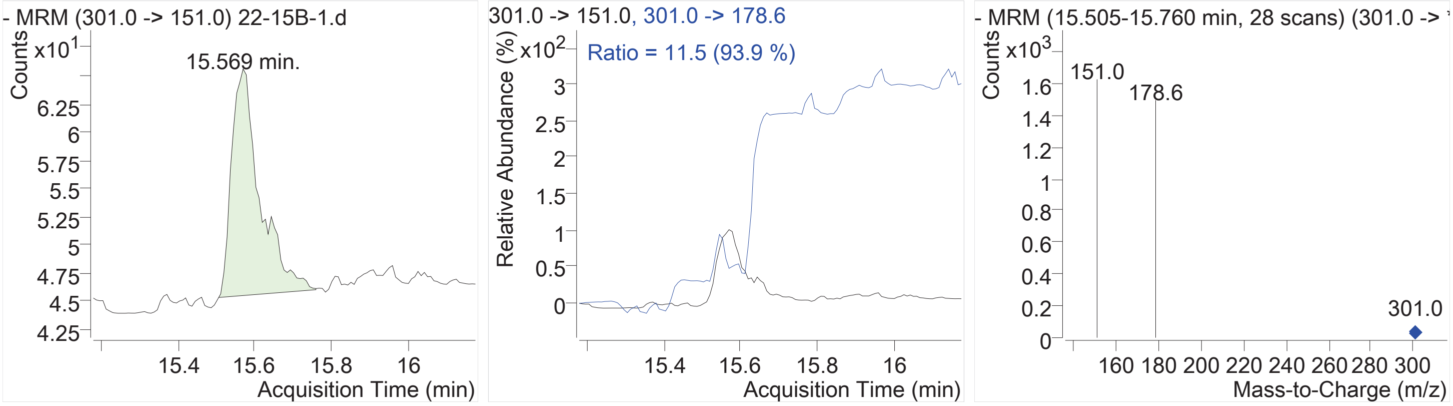

Luteolin

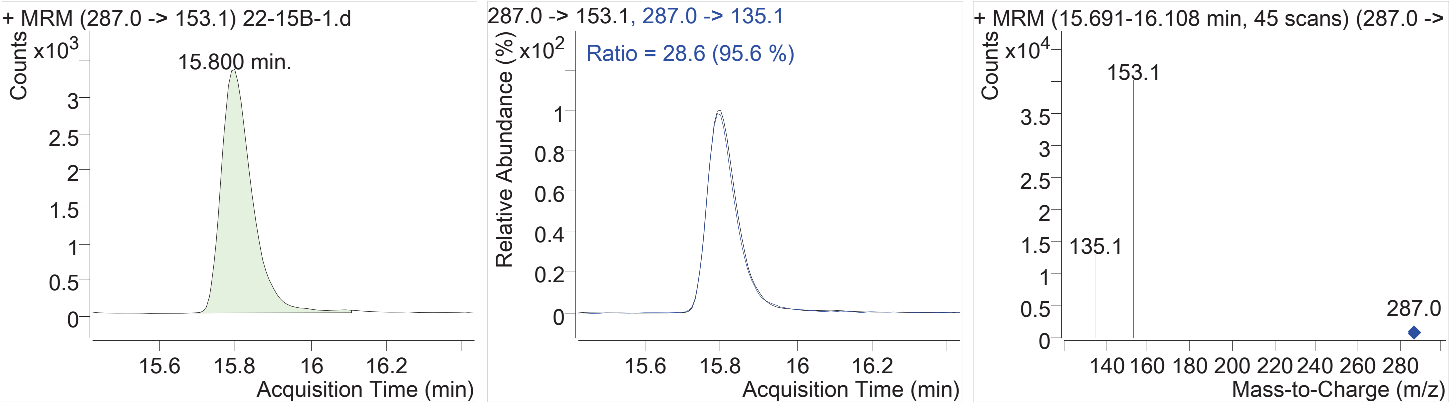

Kaempferol

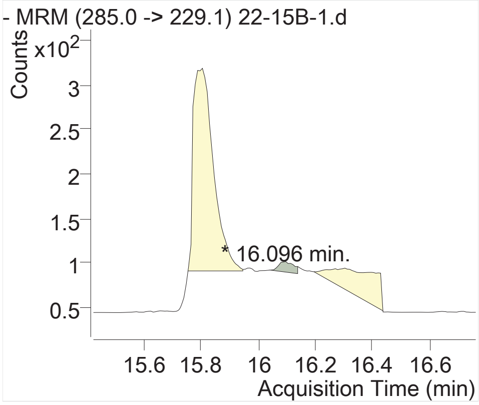

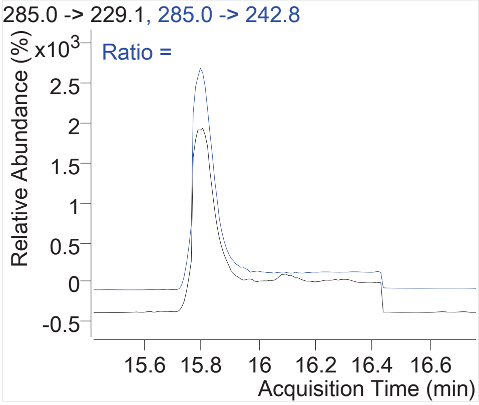

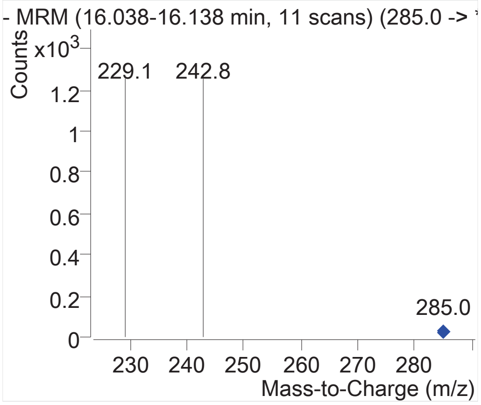

Apigenin

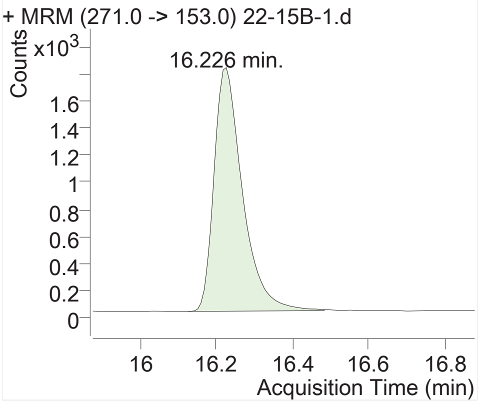

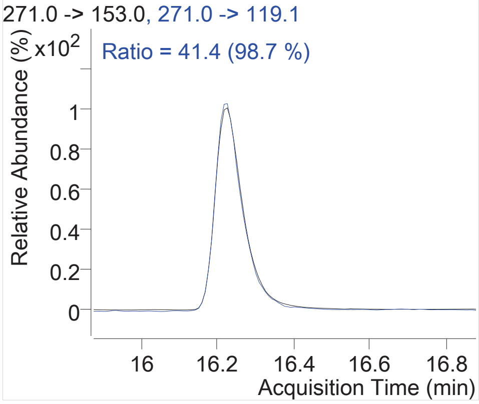

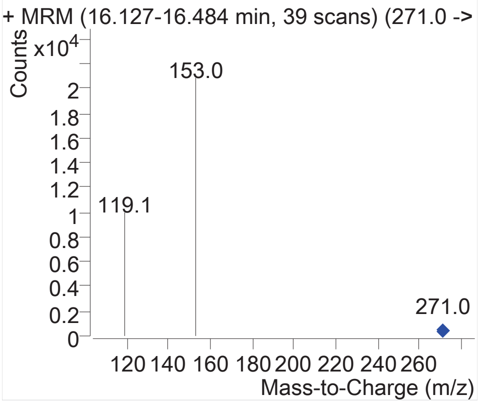

Quantitative Analysis Complete Report

Agilent Technologies

**Batch Path**	D:\MassHunter\Data\2022ekim\061022cengizhoca\QuantResults\071022.batch.bin		
**Analysis Time**	10/11/2022 1:33:26 PM	**Analyst Name**	Defam-PC\admin
**Report Time**	10/11/2022 1:39:32 PM	**Reporter Name**	admin
**Last Calib Update**	10/11/2022 1:33:17 PM	**Batch State**	Processed
**Quant Batch Version**	B.07.01	**Quant Report Version**	B.07.01

**Acq. Time**	10/8/2022 9:29:01 AM	**Data File**	22-15B-2.d
**Sample Type**	Sample	**Sample Name**	22-15B-2
**Dilution**	1	**Acq. Method**	FENOLIK_DMRM2021-31bilesen.m

Sample Chromatogram

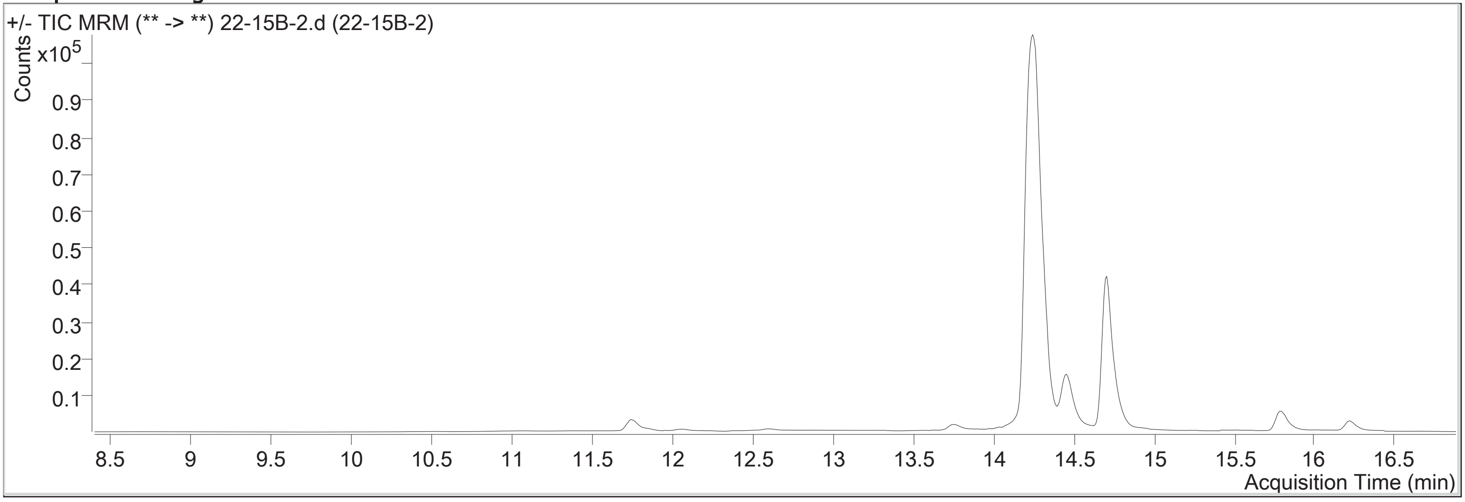

Compound	Transition	RT	Resp.	Final Conc	Units
Gallic acid	168.9 -> 125.0	8.900	3	ND	ng/ml
Protocatechuic acid	152.9 -> 108.9	10.543	551	41.5173	ng/ml
Pyrocatechol	109.0 -> 52.9	10.822	1	ND	ng/ml
3,4-Dihydroxyphenylacetic acid	167.0 -> 123.0	10.843	11	ND	ng/ml
(+)-Catechin	289.0 -> 245.0	11.219	9	ND	ng/ml
2,5-Dihydroxybenzoic acid	152.9 -> 109.0	11.954	58	9.6473	ng/ml
Chlorogenic acid	355.0 -> 163.0	11.752	19721	931.4722	ng/ml
3-Hydroxybenzoic acid	137.0 -> 93.0	12.819	14	17.6661	ng/ml
4-Hydroxybenzoic acid	136.9 -> 93.1	12.064	2376	204.9290	ng/ml
(-)-Epicatechin	291.0 -> 139.1	12.302	6	3.8968	ng/ml
Caffeic acid	179.0 -> 135.0	12.608	2192	65.6664	ng/ml
Syringic acid	196.9 -> 181.9	12.723	124	175.6957	ng/ml
Vanillin	151.0 -> 136.0	13.012	232	48.6369	ng/ml
Verbascoside	623.0 -> 160.8	13.317	18	ND	ng/ml
Taxifolin	303.0 -> 285.1	13.661	28	ND	ng/ml
p-Coumaric acid	162.9 -> 119.0	13.758	8546	324.2599	ng/ml
Sinapic acid	222.9 -> 207.9	13.823	43	17.8946	ng/ml
Ferulic acid	193.0 -> 134.0	13.899	260	50.1531	ng/ml
Luteolin 7-glucoside	447.1 -> 285.0	14.247	792794	8676.3882	ng/ml
Hesperidin	611.1 -> 303.0	14.427	5000	610.9026	ng/ml
Hyperoside	465.1 -> 303.1	14.470	20224	1027.5511	ng/ml
Rosmarinic acid	359.0 -> 160.9	14.456	27875	2227.8208	ng/ml
Apigenin 7-glucoside	433.1 -> 271.0	14.713	200726	5968.4125	ng/ml
Pinoresinol	357.0 -> 151.0	14.925	623	2430.9142	ng/ml
2-Hydroxycinnamic acid	162.9 -> 119.1	14.743	4	ND	ng/ml
Eriodictyol	287.0 -> 151.0	15.036	142	ND	ng/ml
Quercetin	301.0 -> 151.0	15.544	100	ND	ng/ml
Luteolin	287.0 -> 153.1	15.791	19162	1205.5929	ng/ml
Kaempferol	285.0 -> 229.1	16.096	52	ND	ng/ml

Compound	Transition	RT	Resp.	Final Conc	Units
Apigenin	271.0 -> 153.0	16.226	9394	502.6294	ng/ml

Gallic acid

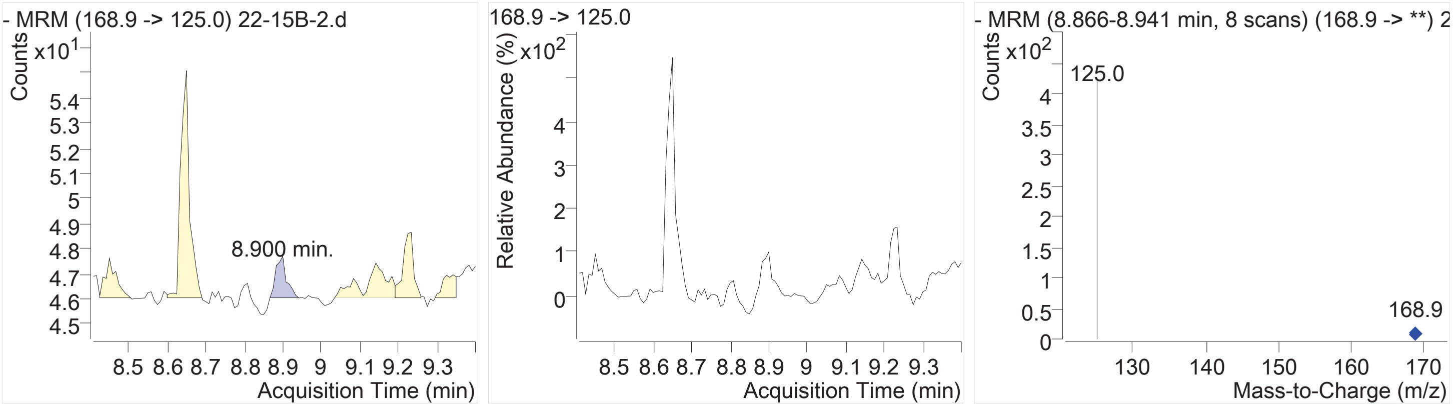

Protocatechuic acid

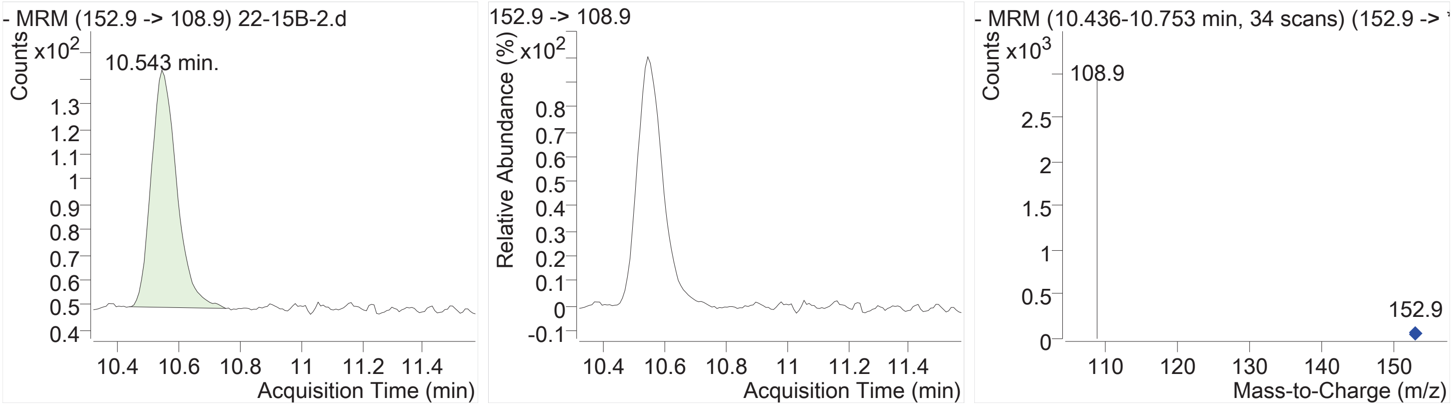

Pyrocatechol

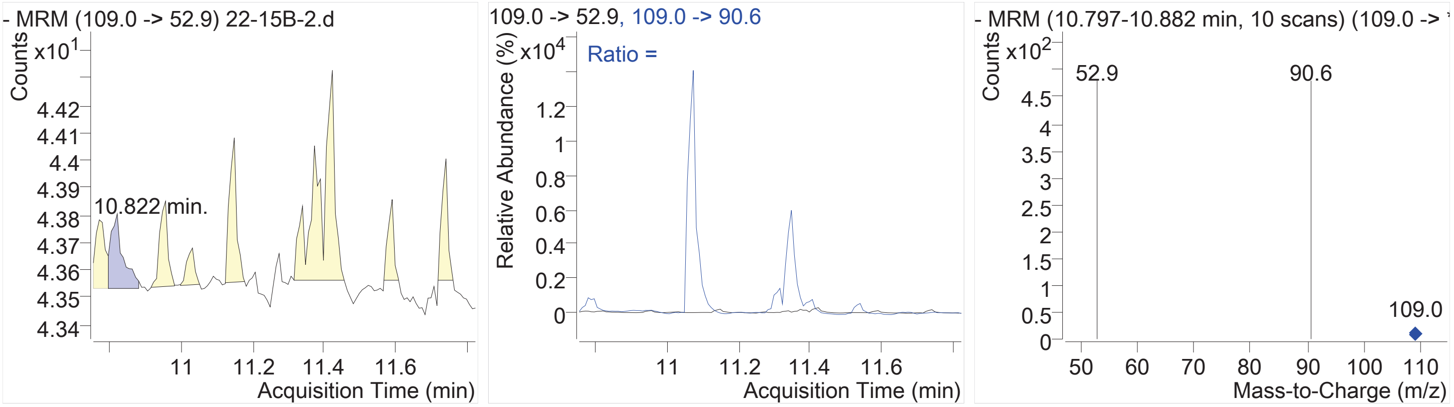

3,4-Dihydroxyphenylacetic acid

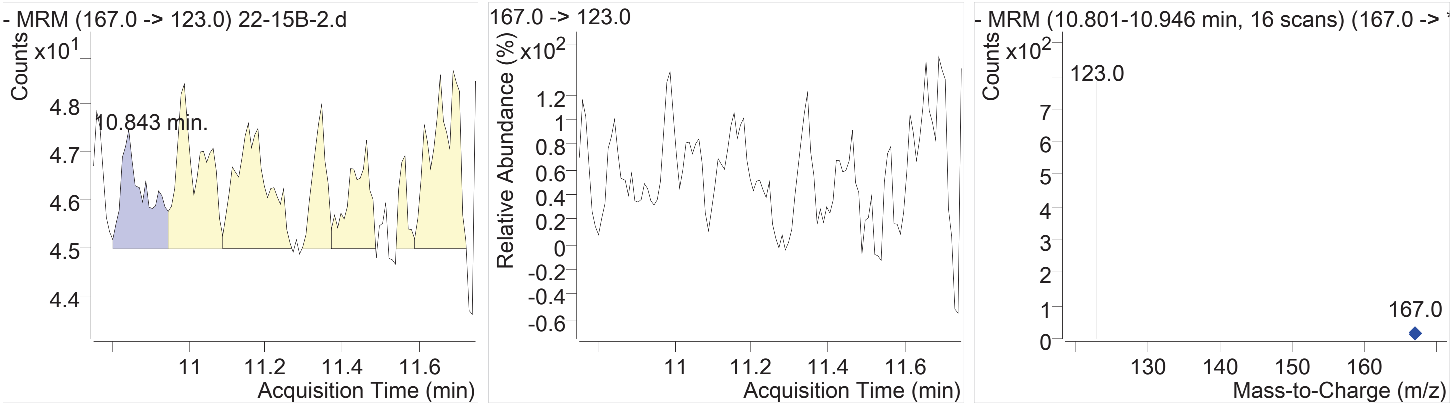

Agilent Technologies

(+)-Catechin

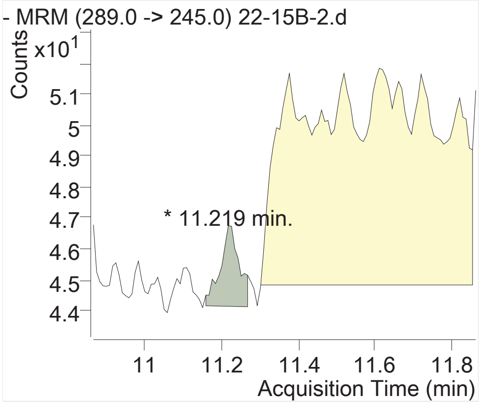

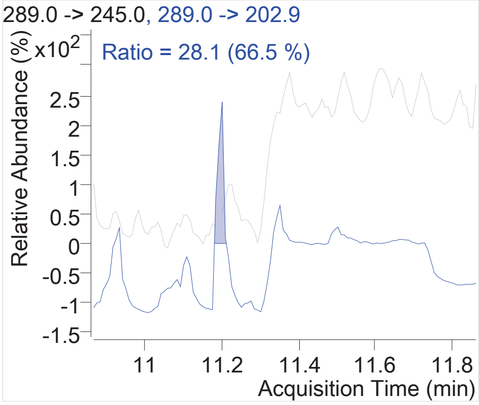

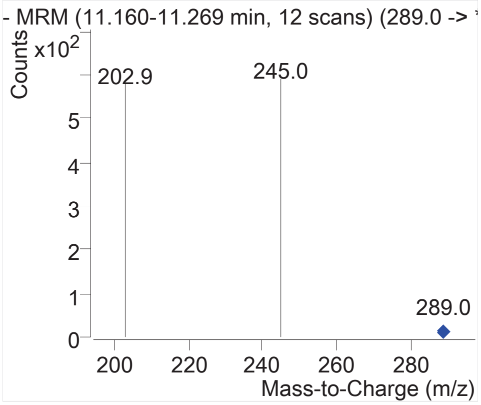

2,5-Dihydroxybenzoic acid

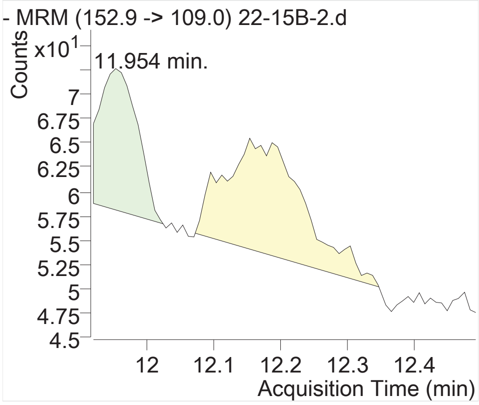

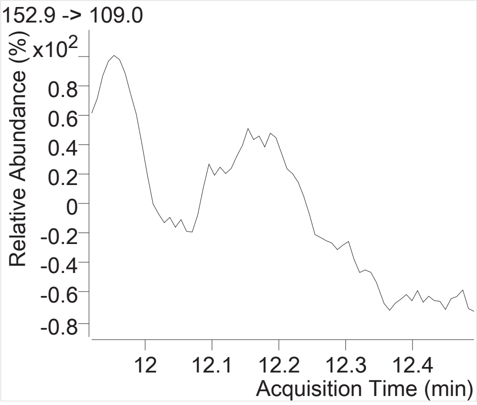

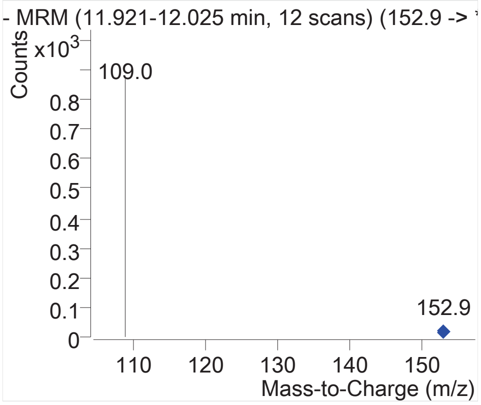

Chlorogenic acid

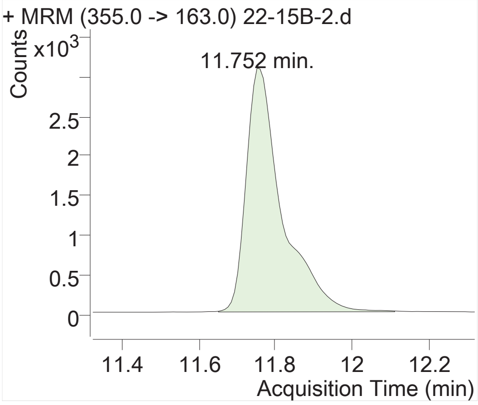

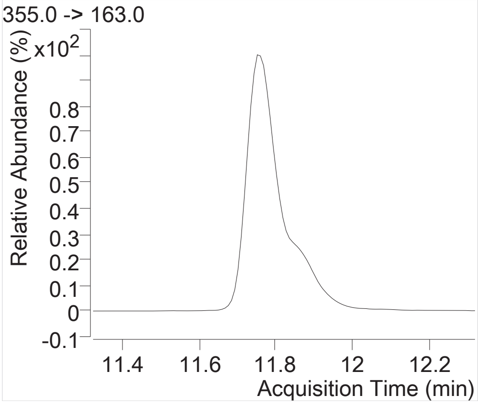

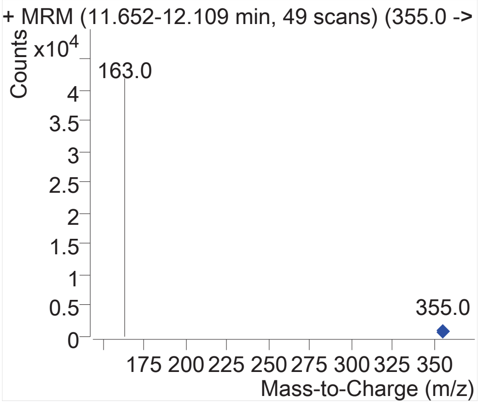

3-Hydroxybenzoic acid

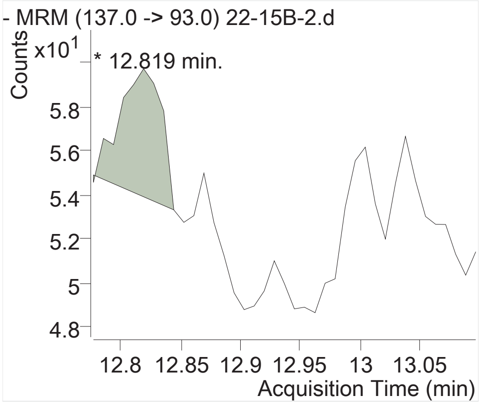

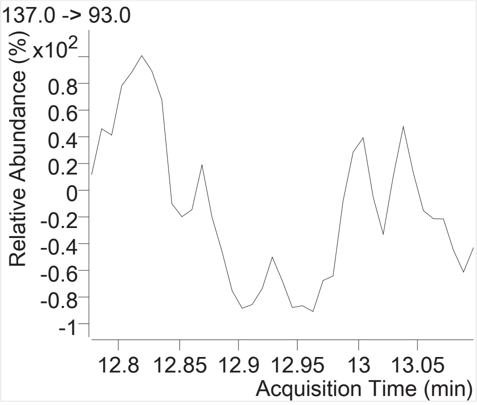

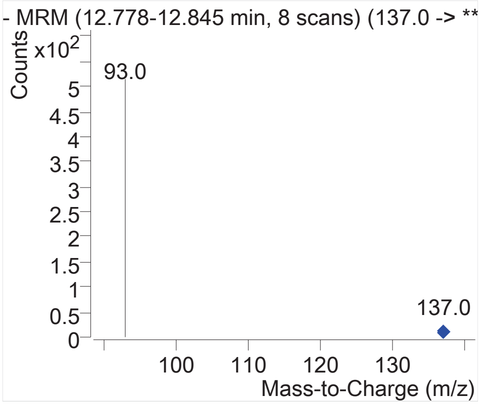

Generated at 1:33 PM on 10/11/2022

4-Hydroxybenzoic acid

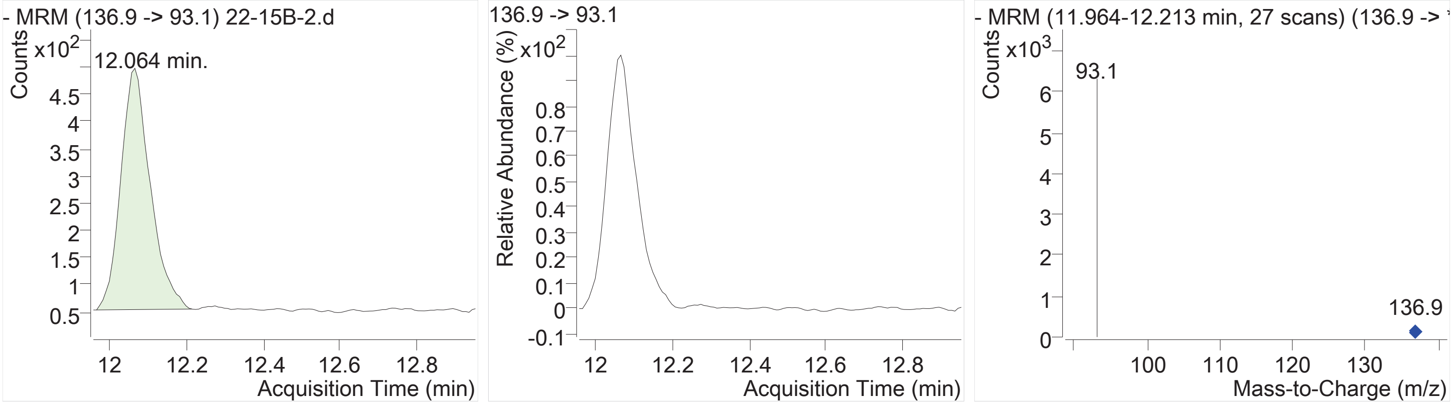

(-)-Epicatechin

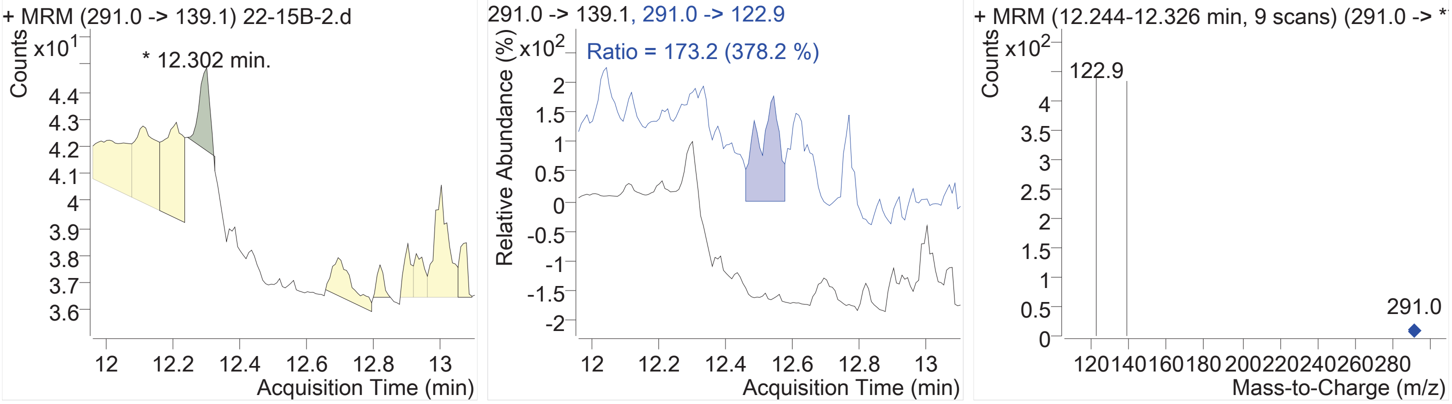

Caffeic acid

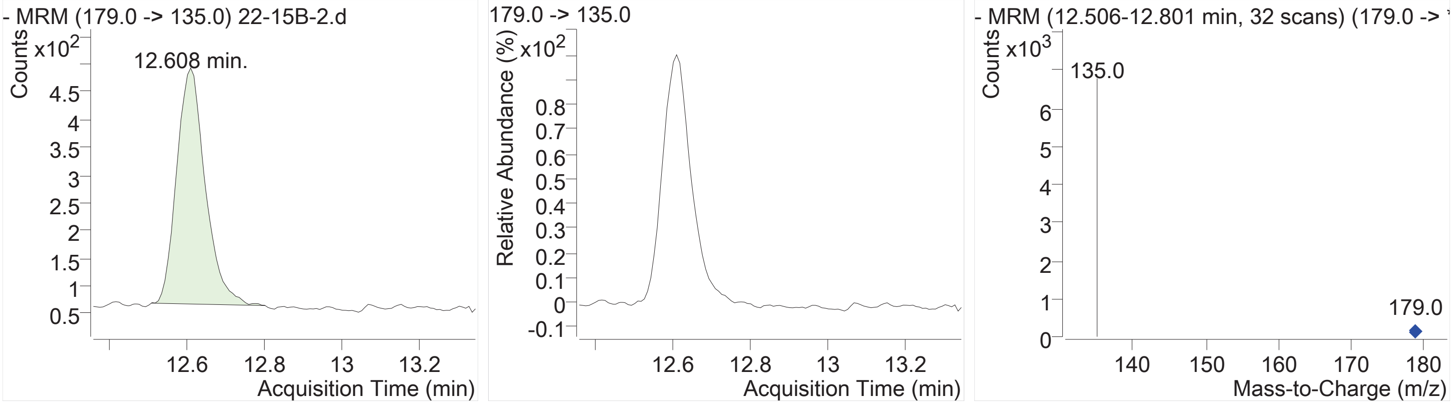

Syringic acid

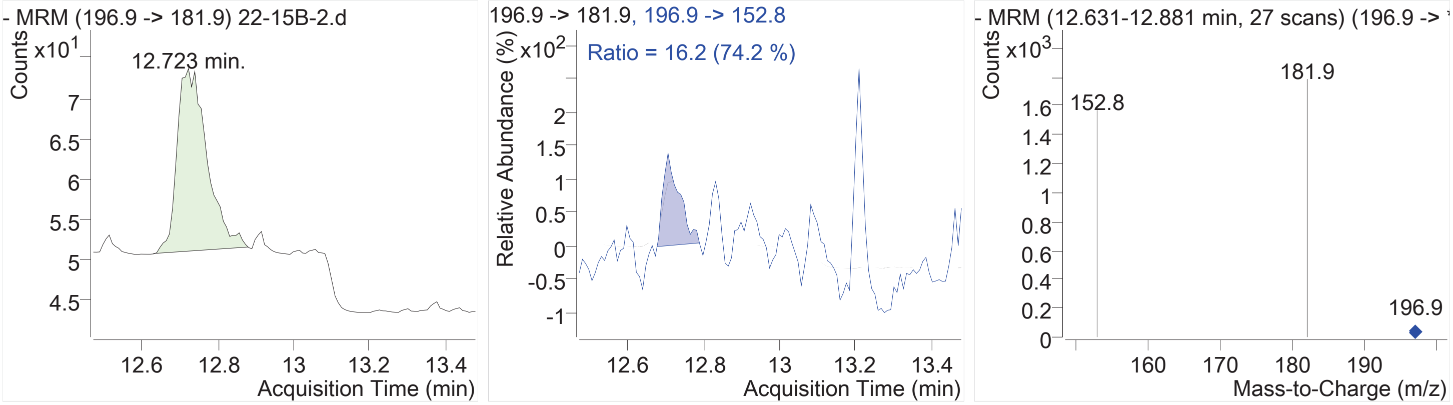

Vanillin

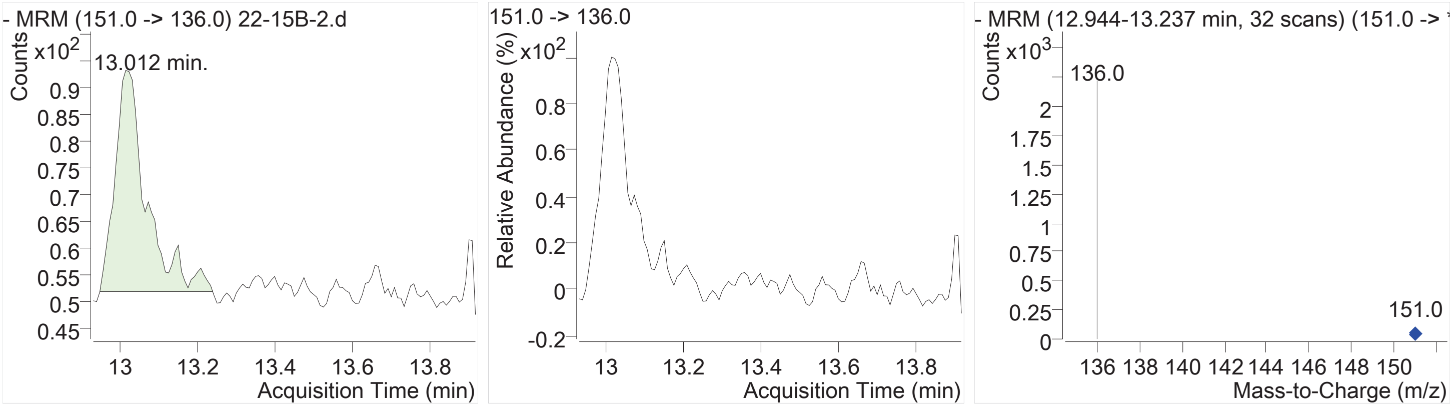

Verbascoside

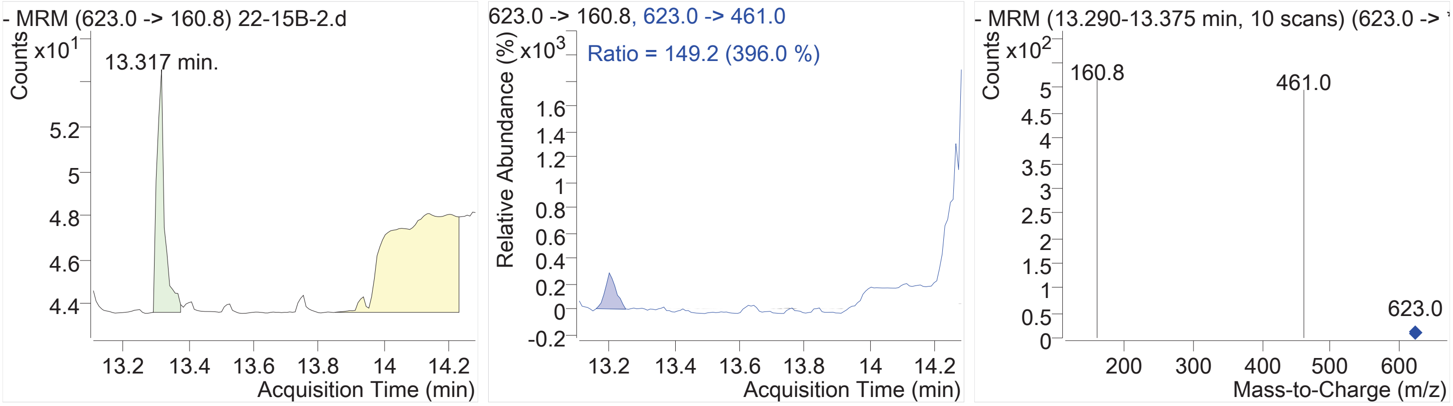

Taxifolin

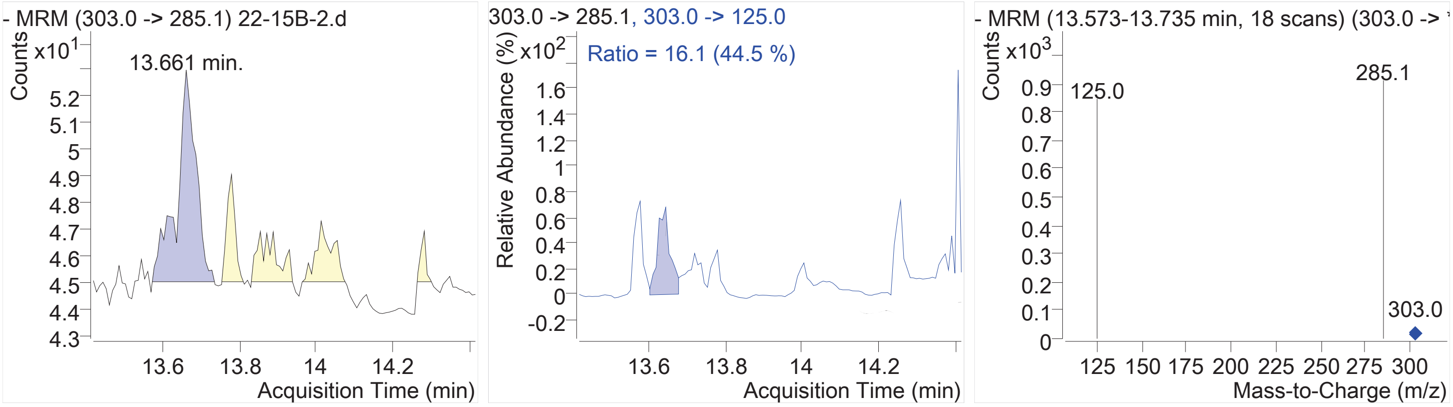

p-Coumaric acid

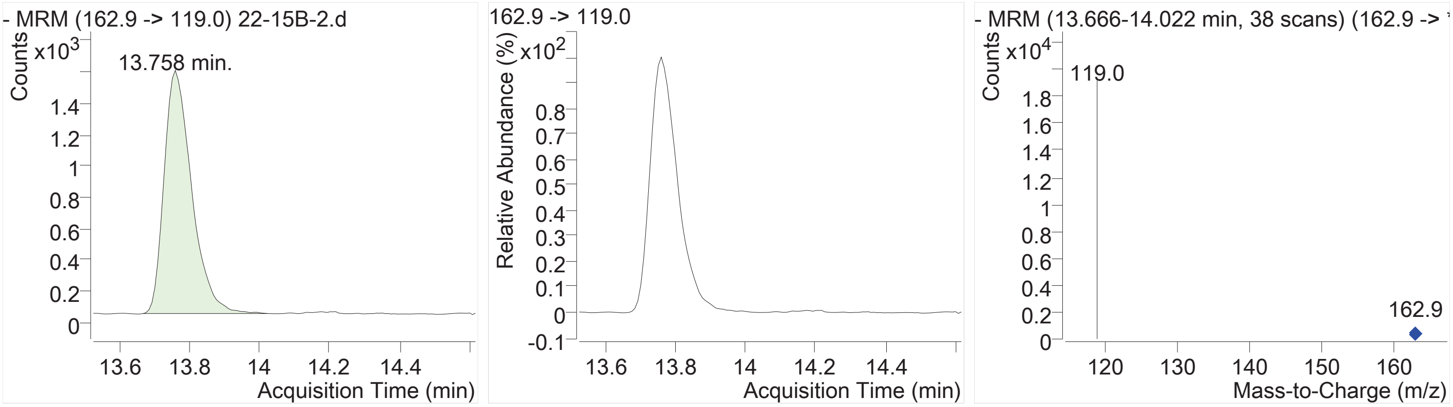

Sinapic acid

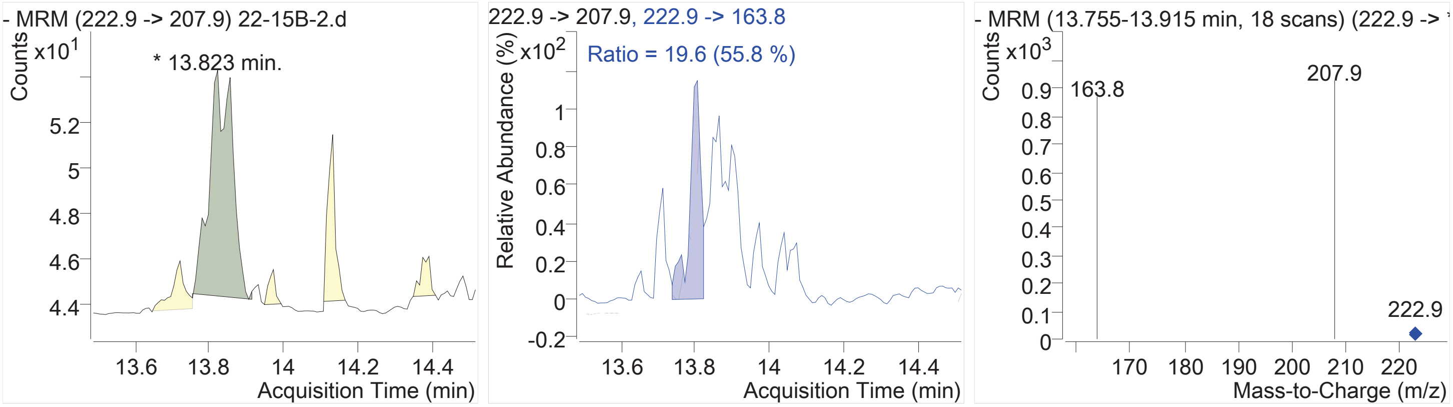

Ferulic acid

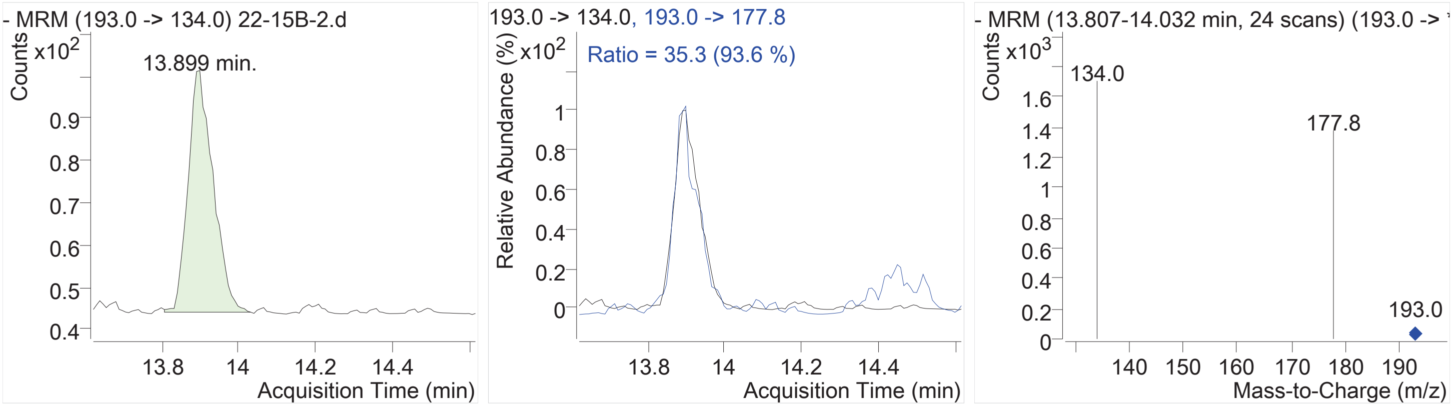

Luteolin 7-glucoside

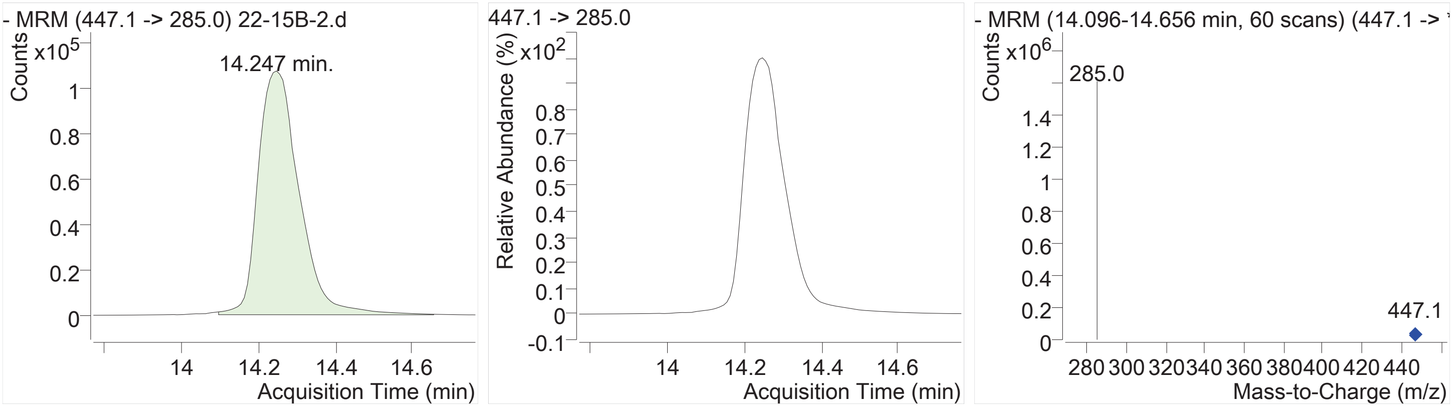

Hesperidin

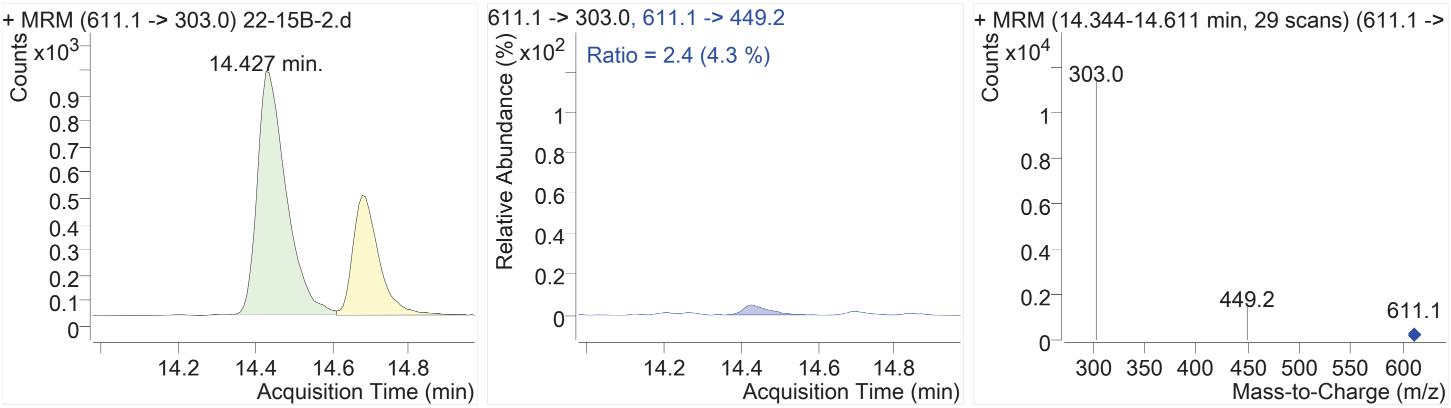

Hyperoside

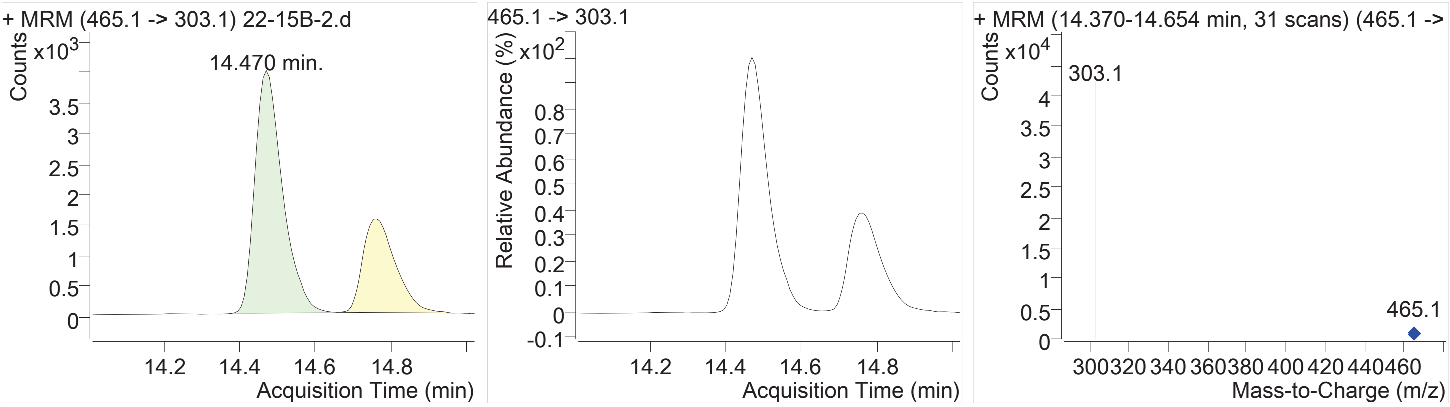

Rosmarinic acid

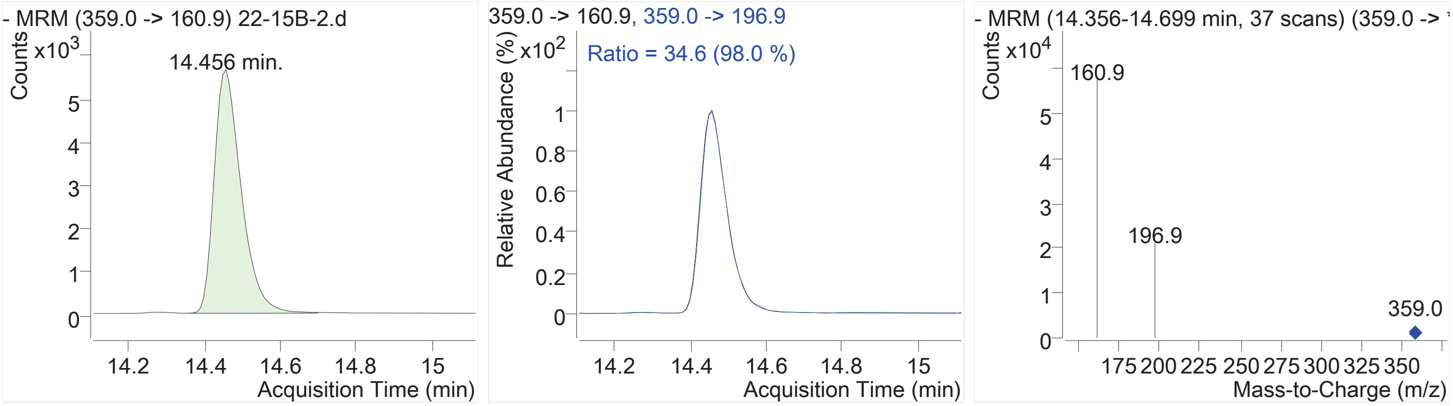

Apigenin 7-glucoside

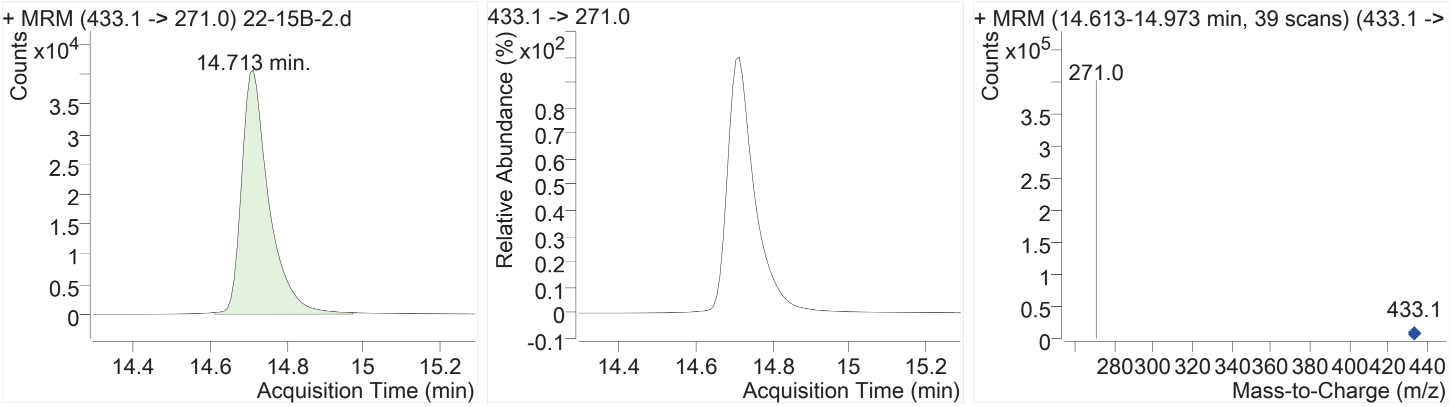

Pinoresinol

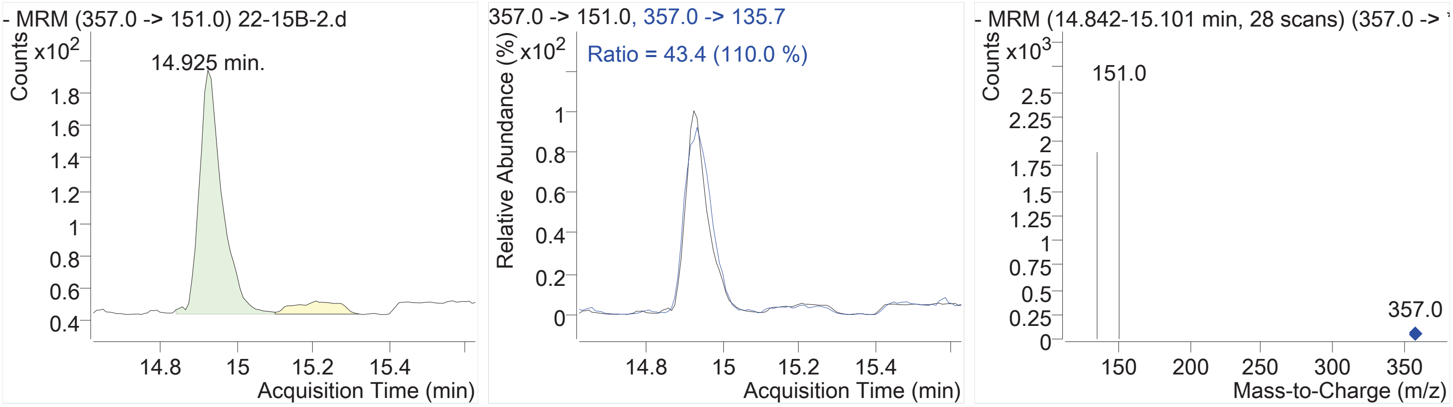

2-Hydroxycinnamic acid

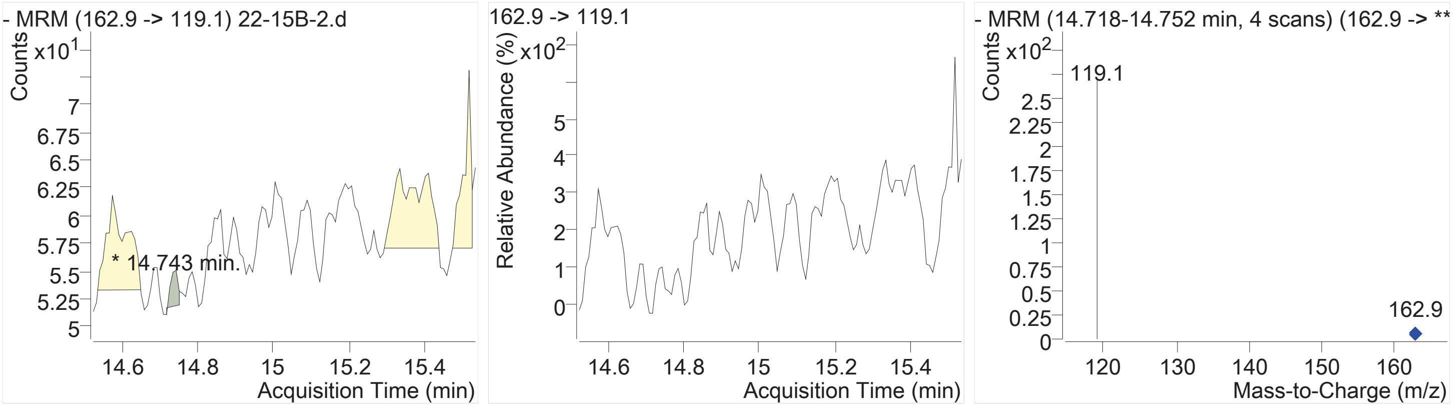

Eriodictyol

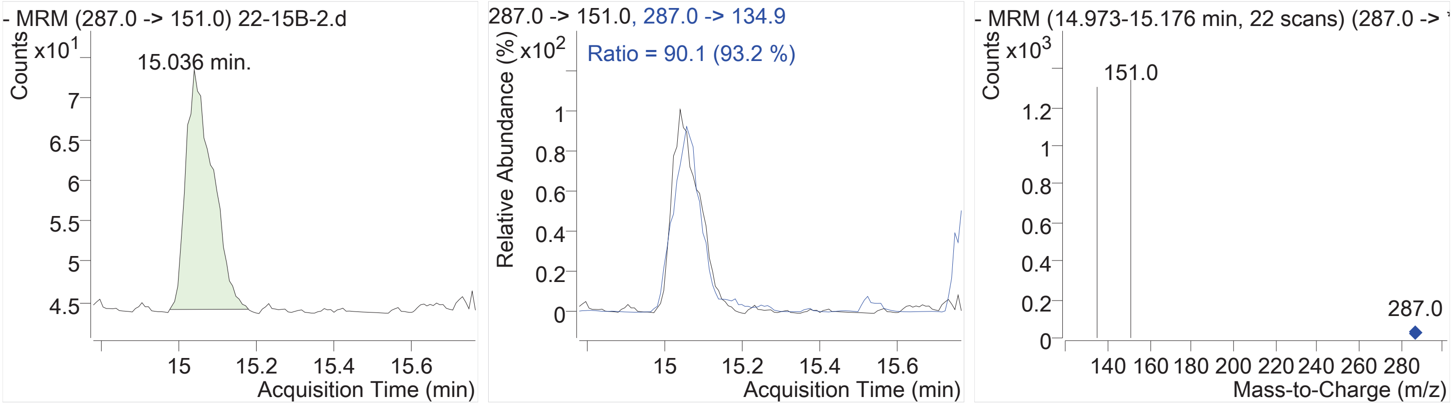

Quercetin

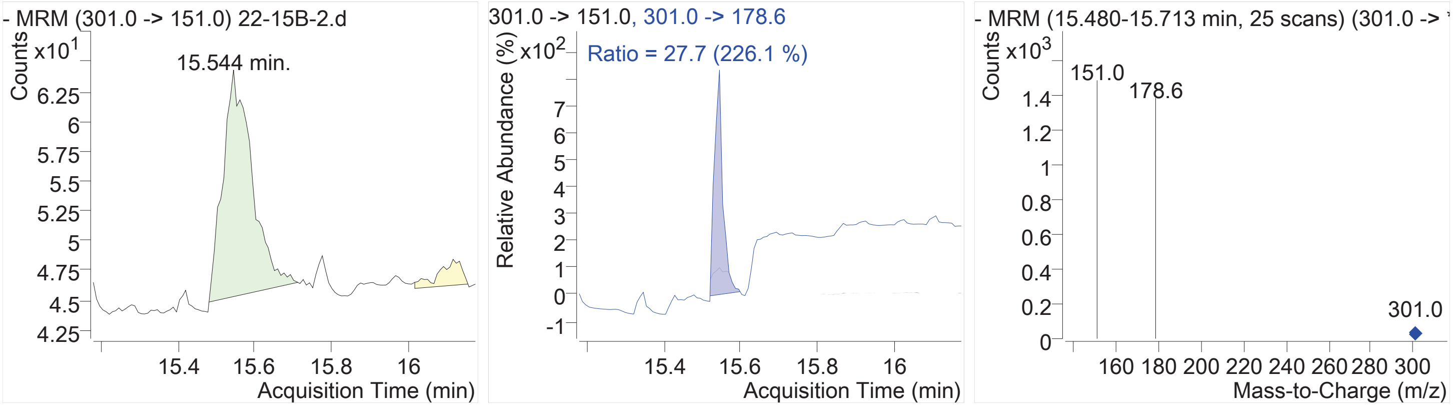

Luteolin

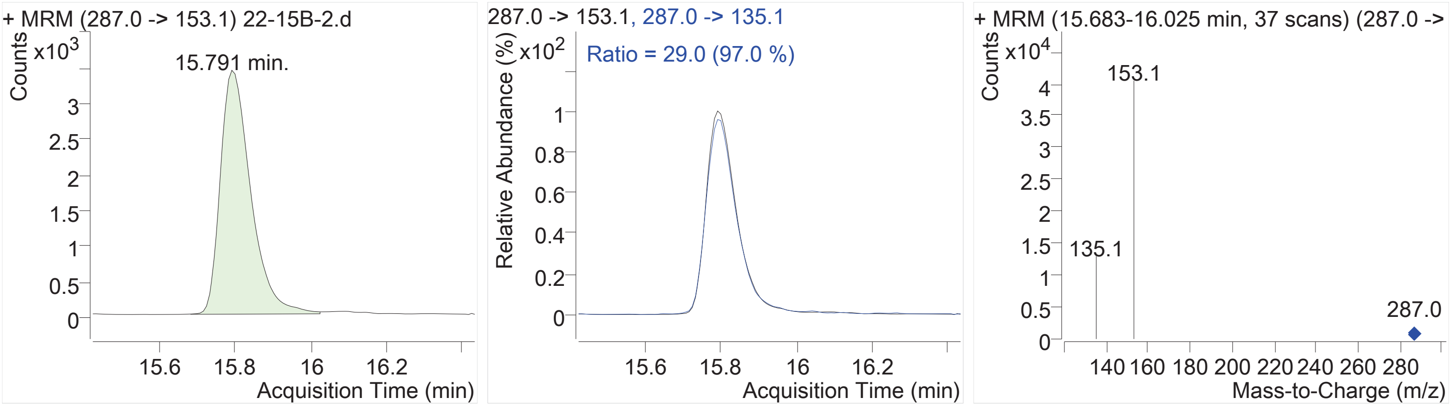

Agilent Technologies

Kaempferol

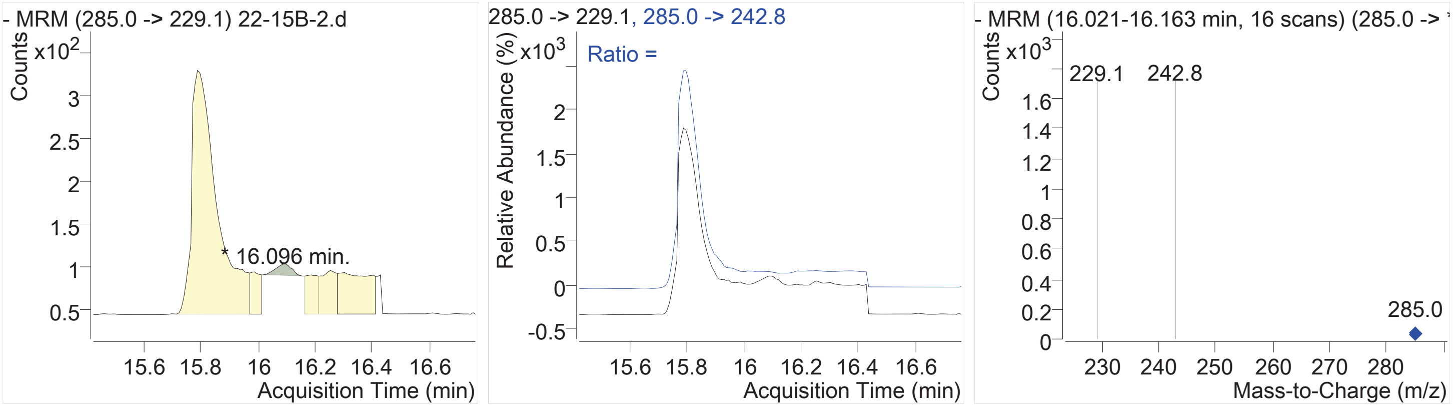

Apigenin

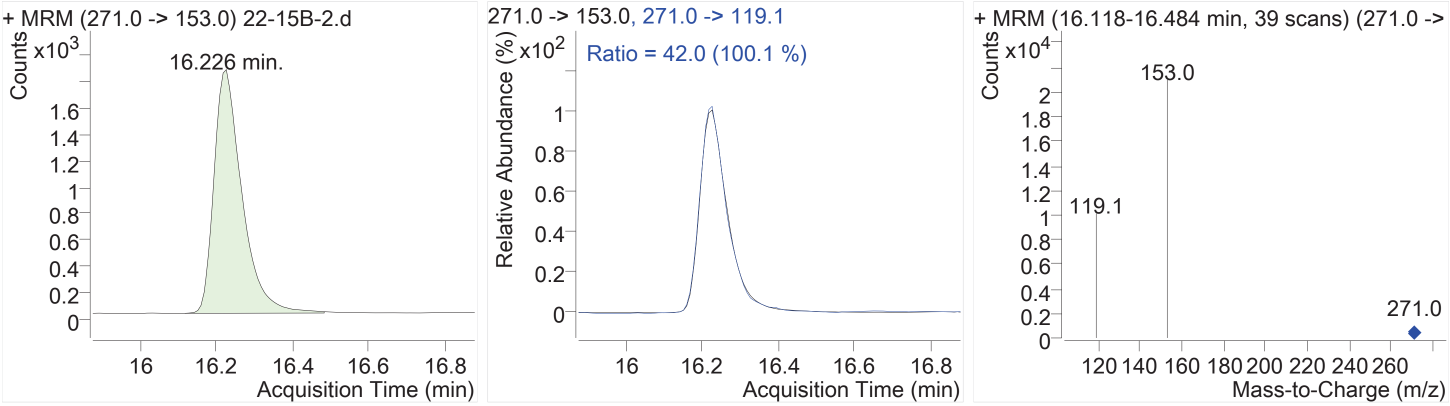

Quantitative Analysis Complete Report

Agilent Technologies

**Batch Path**	D:\MassHunter\Data\2022ekim\061022cengizhoca\QuantResults\071022.batch.bin		
**Analysis Time**	10/11/2022 1:33:26 PM	**Analyst Name**	Defam-PC\admin
**Report Time**	10/11/2022 1:39:35 PM	**Reporter Name**	admin
**Last Calib Update**	10/11/2022 1:33:17 PM	**Batch State**	Processed
**Quant Batch Version**	B.07.01	**Quant Report Version**	B.07.01

**Acq. Time**	10/8/2022 9:50:42 AM	**Data File**	22-15B-3.d
**Sample Type**	Sample	**Sample Name**	22-15B-3
**Dilution**	1	**Acq. Method**	FENOLIK_DMRM2021-31bilesen.m

Sample Chromatogram

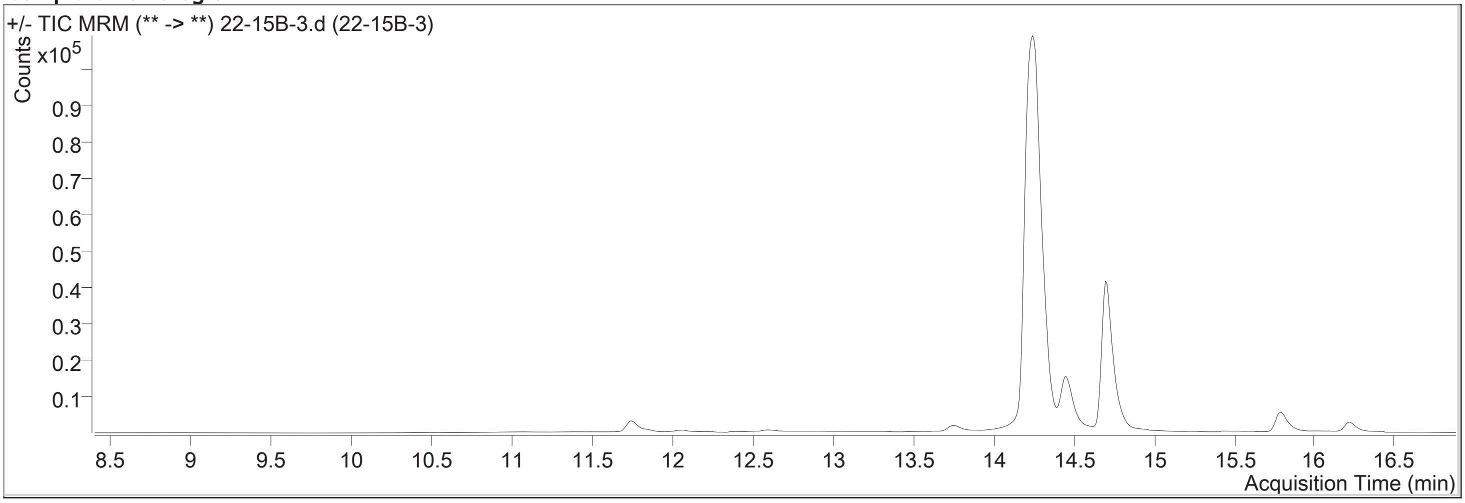

Compound	Transition	RT	Resp.	Final Conc	Units
Gallic acid	168.9 -> 125.0	8.825	6	ND	ng/ml
Protocatechuic acid	152.9 -> 108.9	10.543	514	37.9121	ng/ml
Pyrocatechol	109.0 -> 52.9	10.839	1	ND	ng/ml
3,4-Dihydroxyphenylacetic acid	167.0 -> 123.0	10.954	7	ND	ng/ml
(+)-Catechin	289.0 -> 245.0	11.461	24	ND	ng/ml
2,5-Dihydroxybenzoic acid	152.9 -> 109.0	11.946	67	11.2150	ng/ml
Chlorogenic acid	355.0 -> 163.0	11.752	19492	920.7244	ng/ml
3-Hydroxybenzoic acid	137.0 -> 93.0	12.836	17	18.2555	ng/ml
4-Hydroxybenzoic acid	136.9 -> 93.1	12.064	2424	209.4151	ng/ml
(-)-Epicatechin	291.0 -> 139.1	12.302	4	3.6080	ng/ml
Caffeic acid	179.0 -> 135.0	12.599	2242	67.8166	ng/ml
Syringic acid	196.9 -> 181.9	12.723	132	187.6359	ng/ml
Vanillin	151.0 -> 136.0	13.028	184	34.2396	ng/ml
Verbascoside	623.0 -> 160.8	13.576	3	ND	ng/ml
Taxifolin	303.0 -> 285.1	13.728	8	ND	ng/ml
p-Coumaric acid	162.9 -> 119.0	13.758	8441	320.1794	ng/ml
Sinapic acid	222.9 -> 207.9	13.831	52	20.7404	ng/ml
Ferulic acid	193.0 -> 134.0	13.899	220	42.3990	ng/ml
Luteolin 7-glucoside	447.1 -> 285.0	14.247	808190	8845.5367	ng/ml
Hesperidin	611.1 -> 303.0	14.436	5167	631.2382	ng/ml
Hyperoside	465.1 -> 303.1	14.470	20404	1036.6394	ng/ml
Rosmarinic acid	359.0 -> 160.9	14.448	27626	2207.9561	ng/ml
Apigenin 7-glucoside	433.1 -> 271.0	14.705	198669	5907.2303	ng/ml
Pinoresinol	357.0 -> 151.0	14.925	548	2133.1541	ng/ml
2-Hydroxycinnamic acid	162.9 -> 119.1	14.911	9	ND	ng/ml
Eriodictyol	287.0 -> 151.0	15.045	130	ND	ng/ml
Quercetin	301.0 -> 151.0	15.544	64	ND	ng/ml
Luteolin	287.0 -> 153.1	15.791	19274	1212.7005	ng/ml
Kaempferol	285.0 -> 229.1	16.071	65	ND	ng/ml

Quantitative Analysis Complete Report

Agilent Technologies

Compound	Transition	RT	Resp.	Final Conc	Units
Apigenin	271.0 -> 153.0	16.226	9272	496.0243	ng/ml

Gallic acid

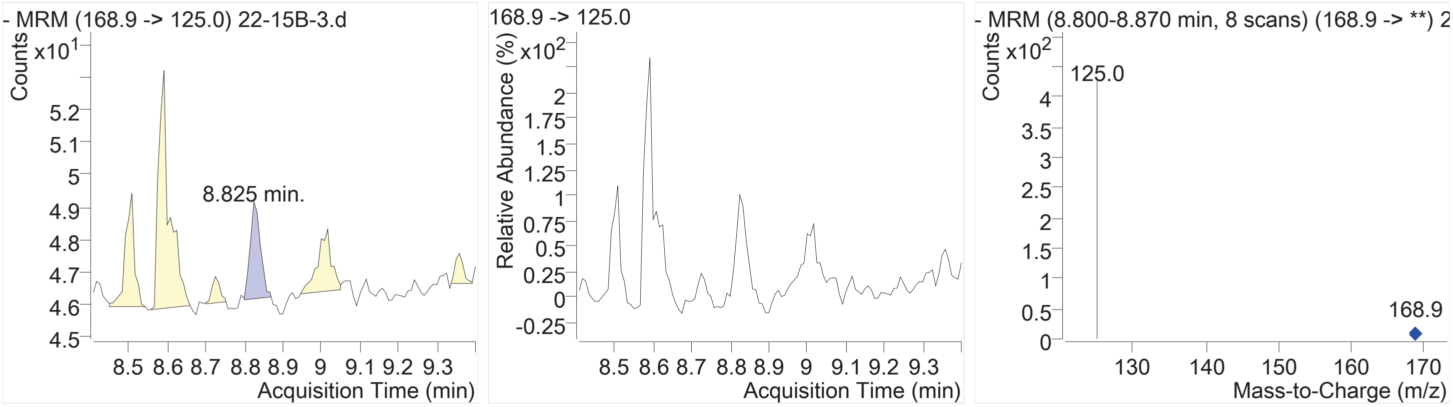

Protocatechuic acid

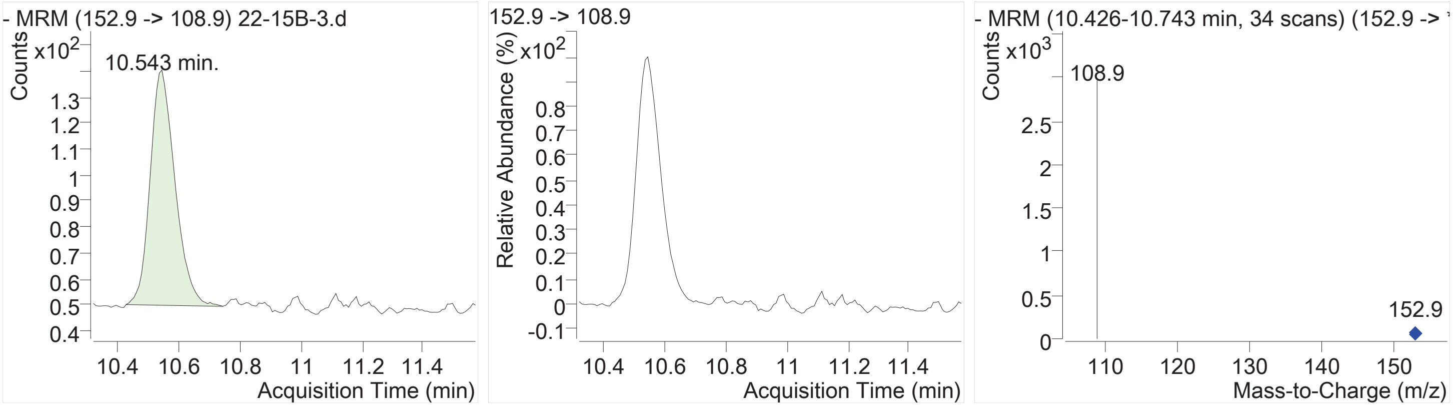

Pyrocatechol

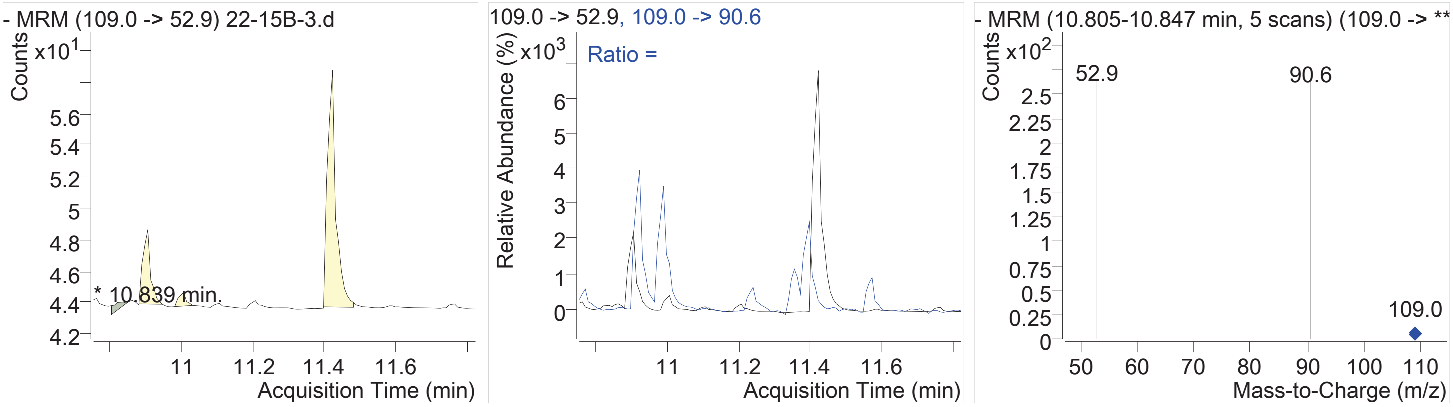

3,4-Dihydroxyphenylacetic acid

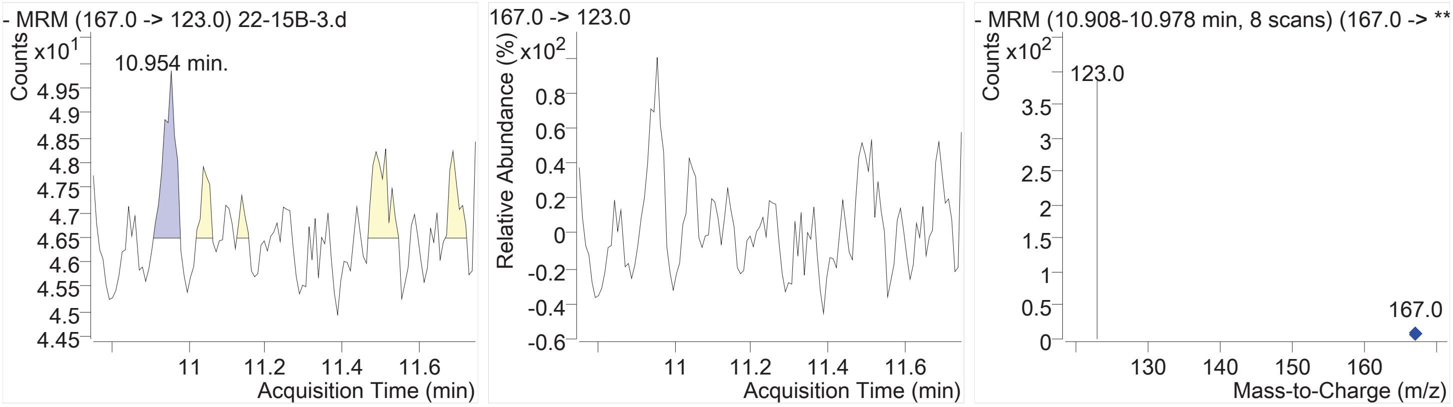

(+)-Catechin

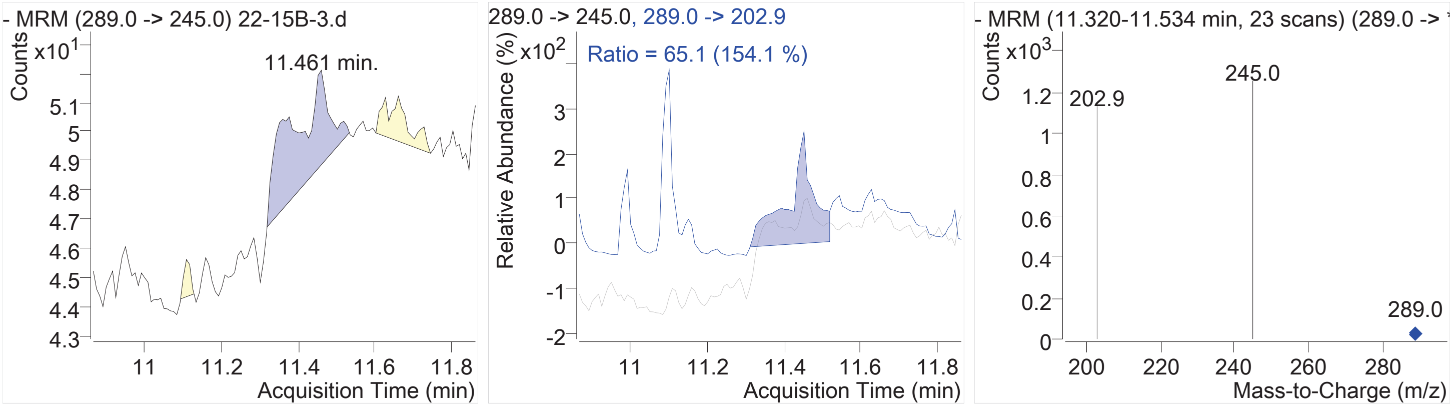

2,5-Dihydroxybenzoic acid

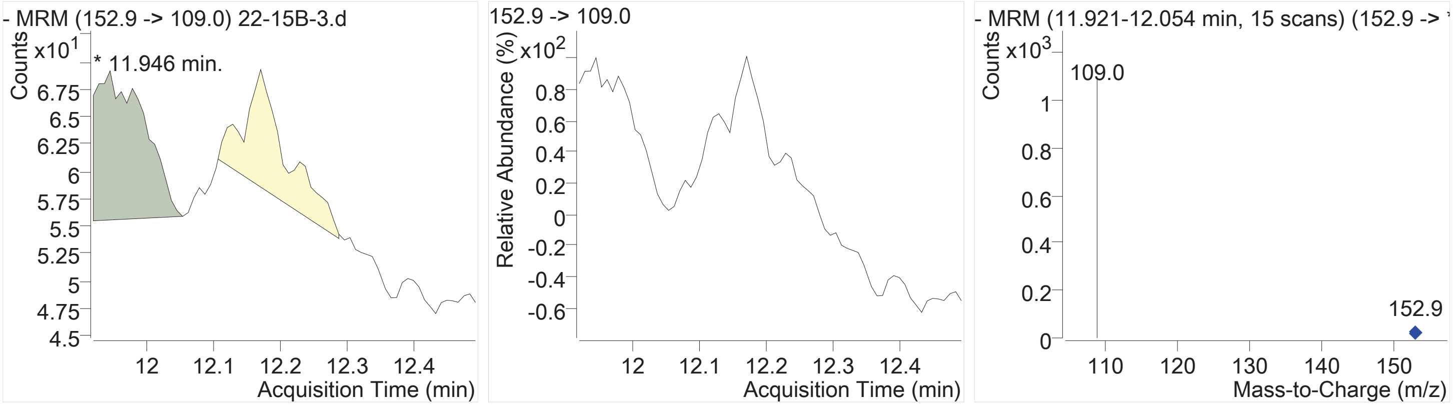

Chlorogenic acid

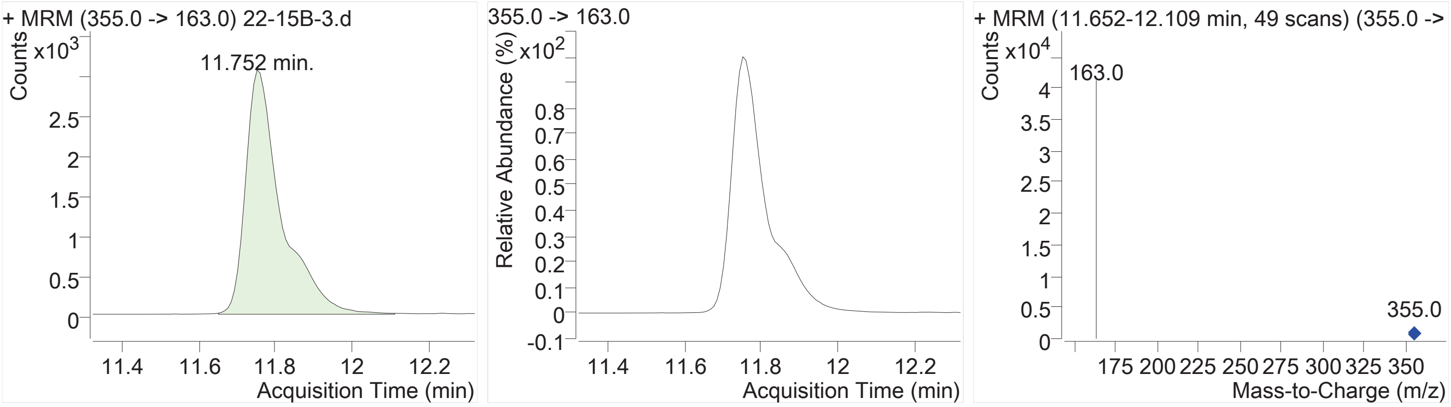

3-Hydroxybenzoic acid

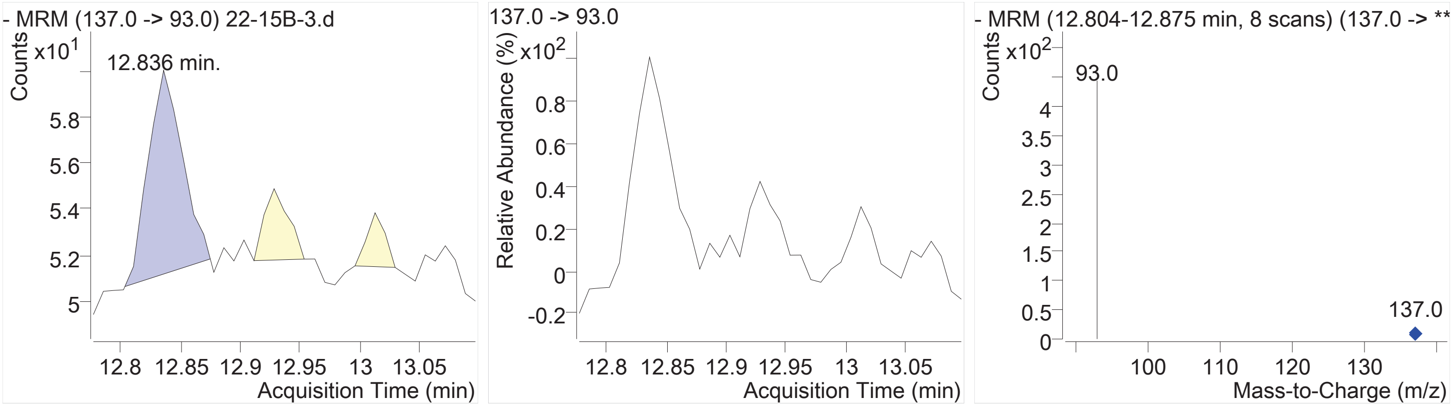

4-Hydroxybenzoic acid

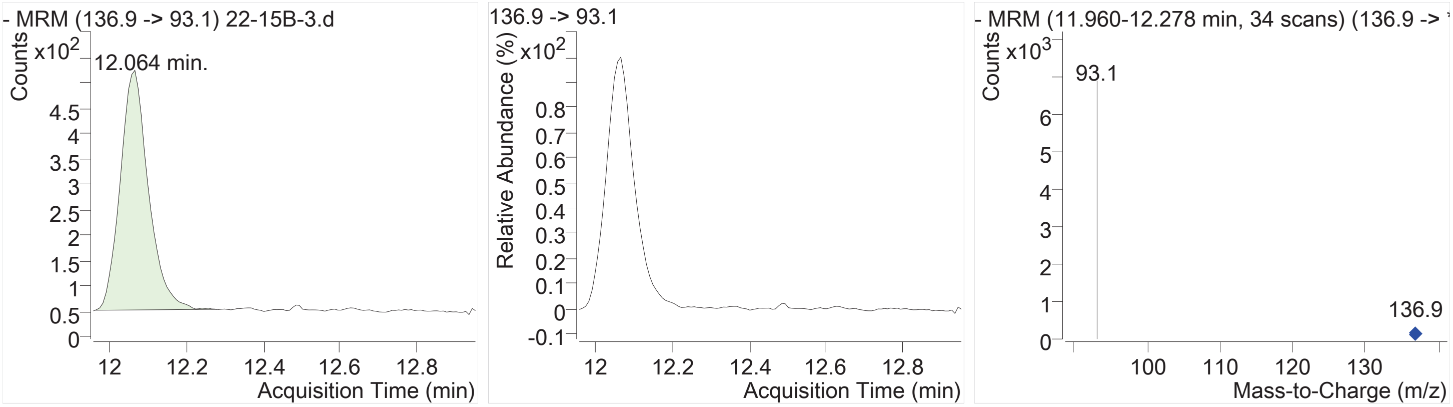

(-)-Epicatechin

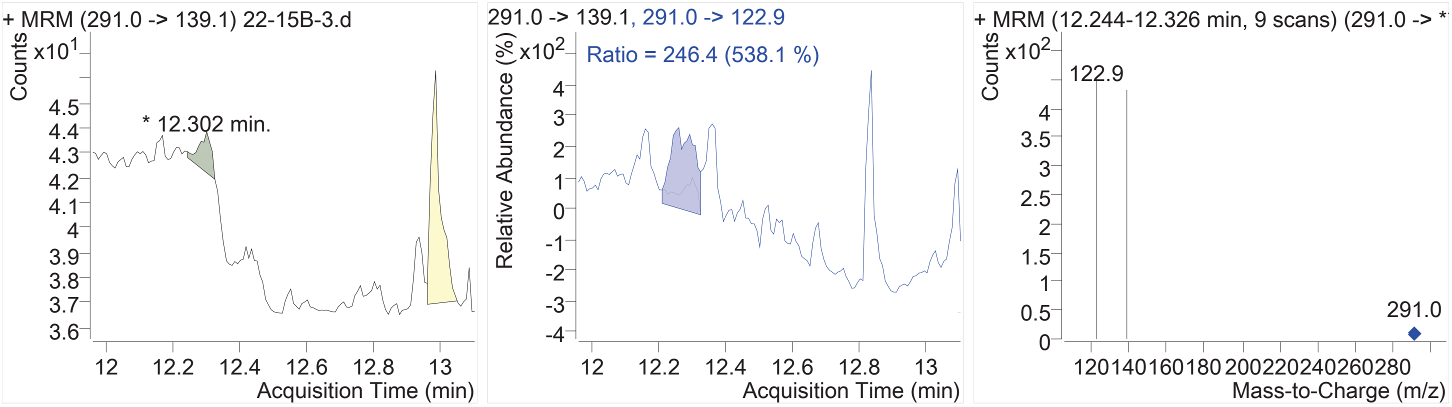

Caffeic acid

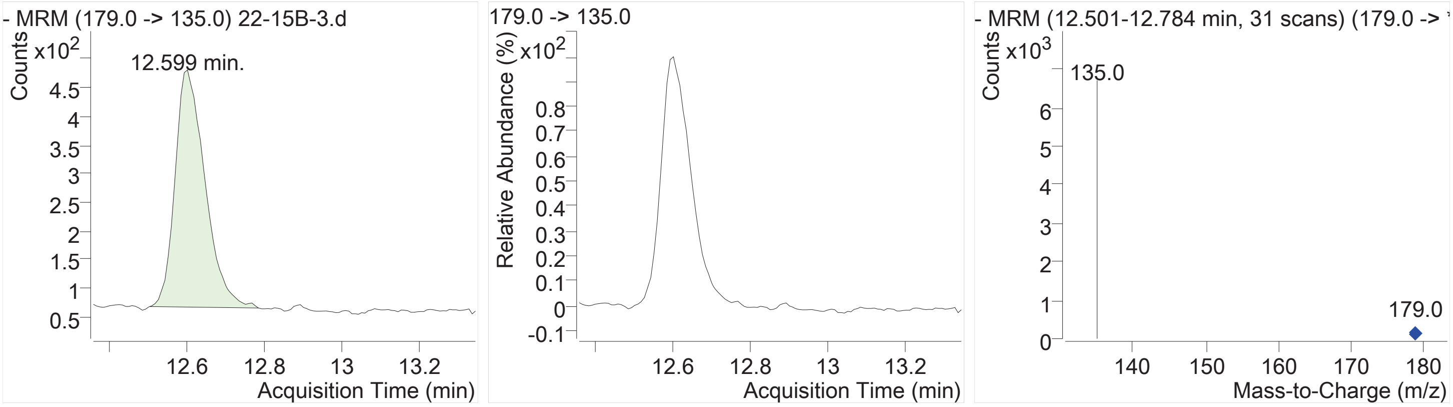

Syringic acid

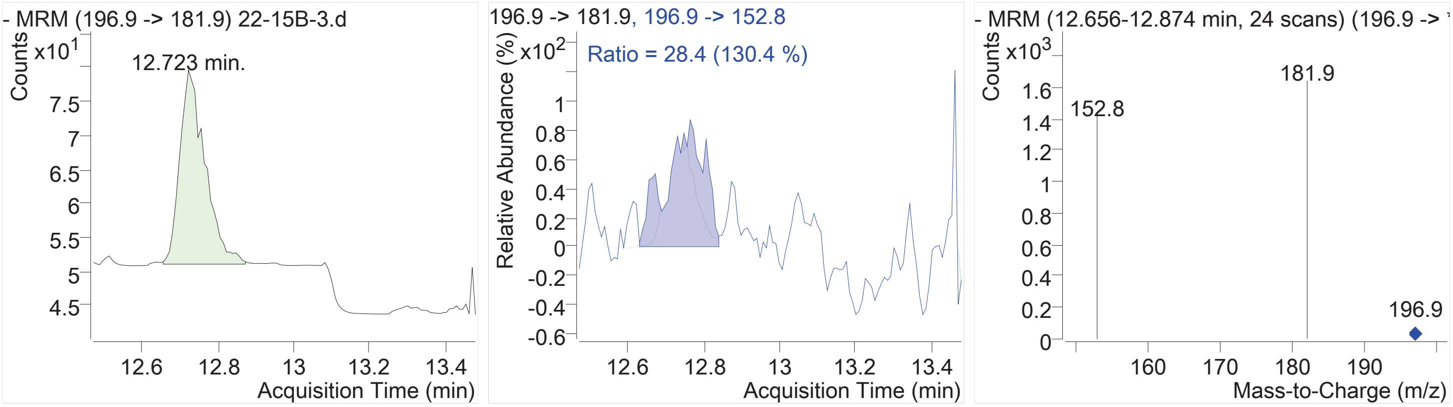

Vanillin

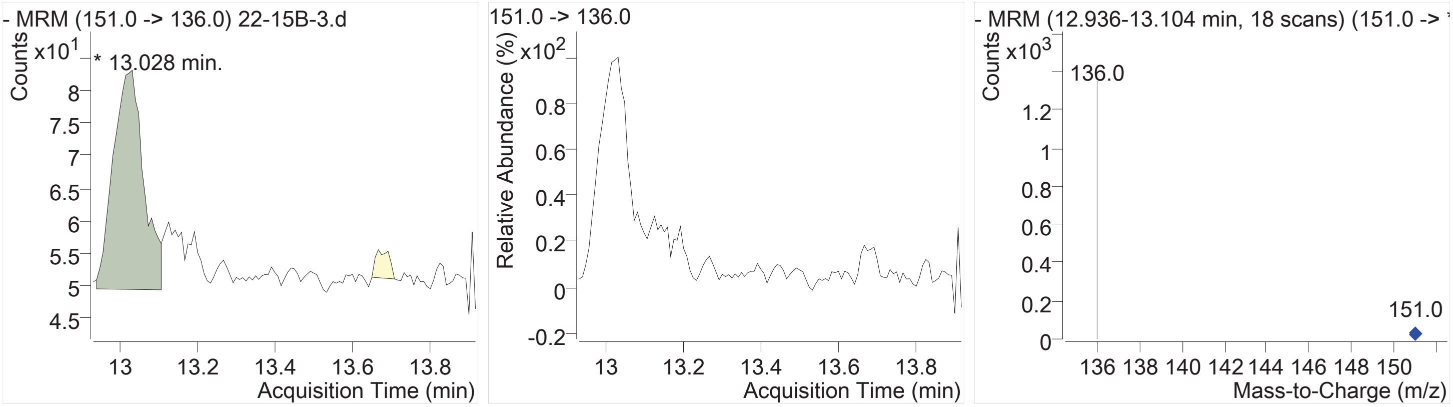

Verbascoside

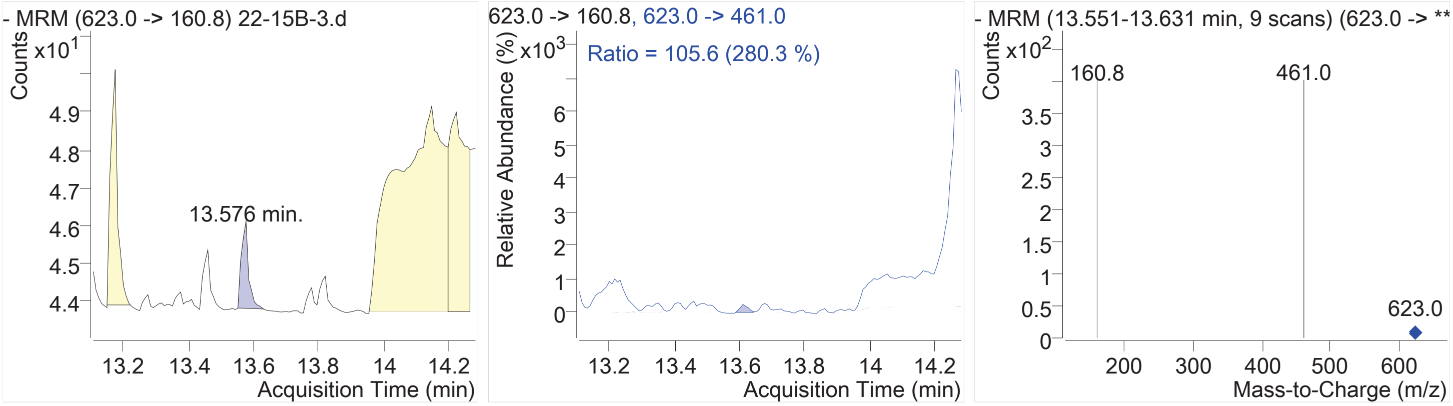

Taxifolin

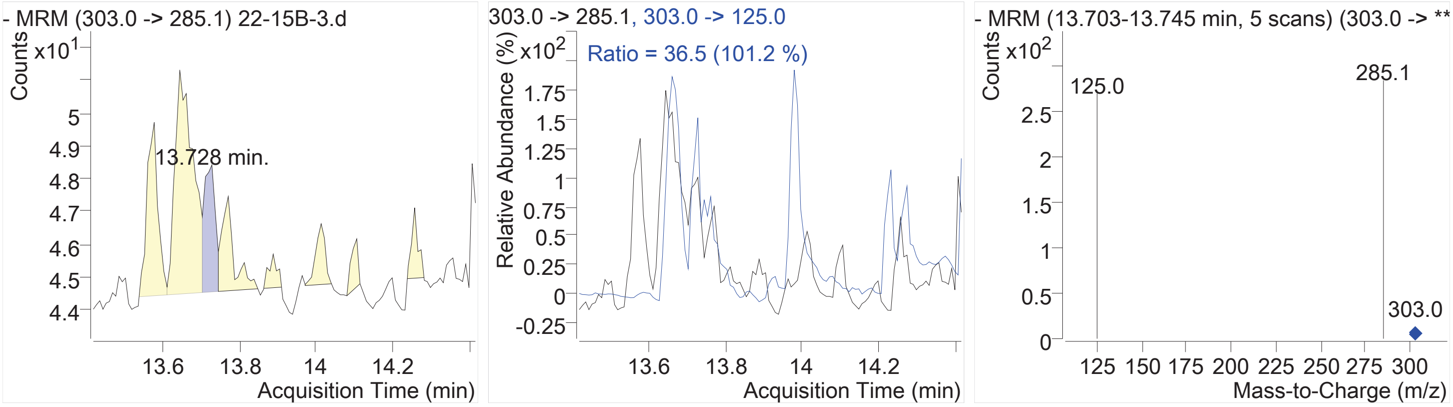

p-Coumaric acid

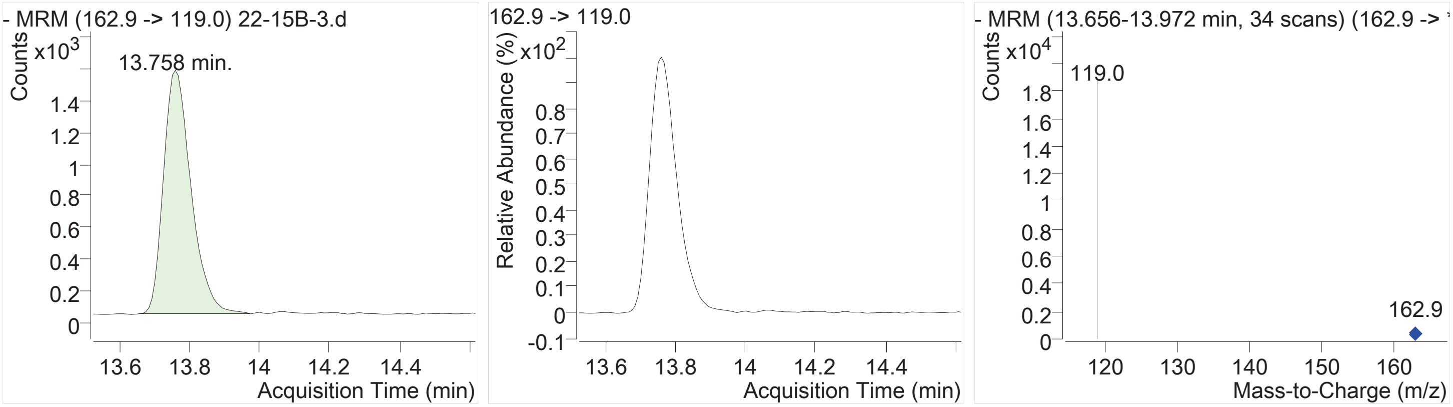

Sinapic acid

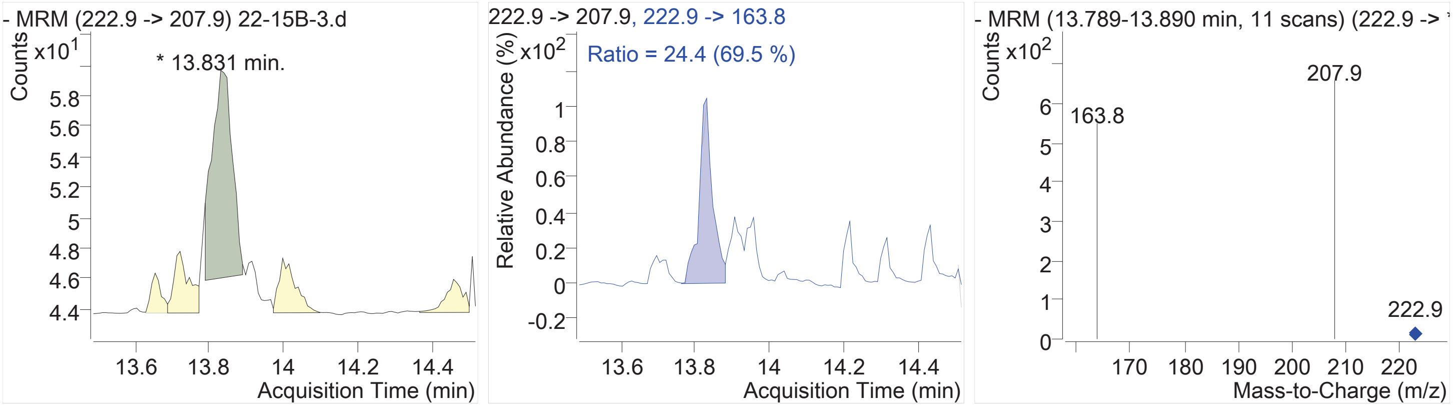

Ferulic acid

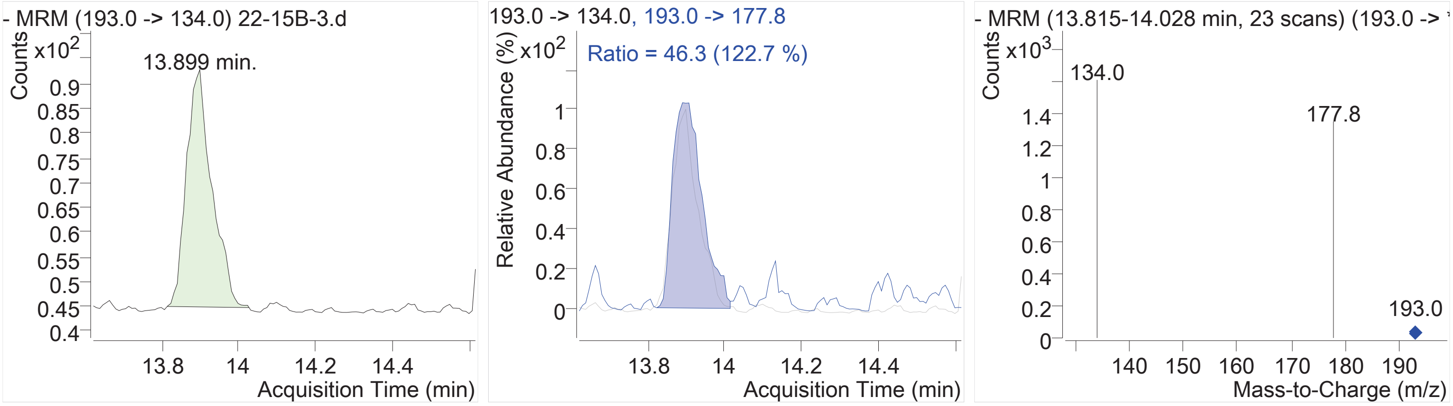

Luteolin 7-glucoside

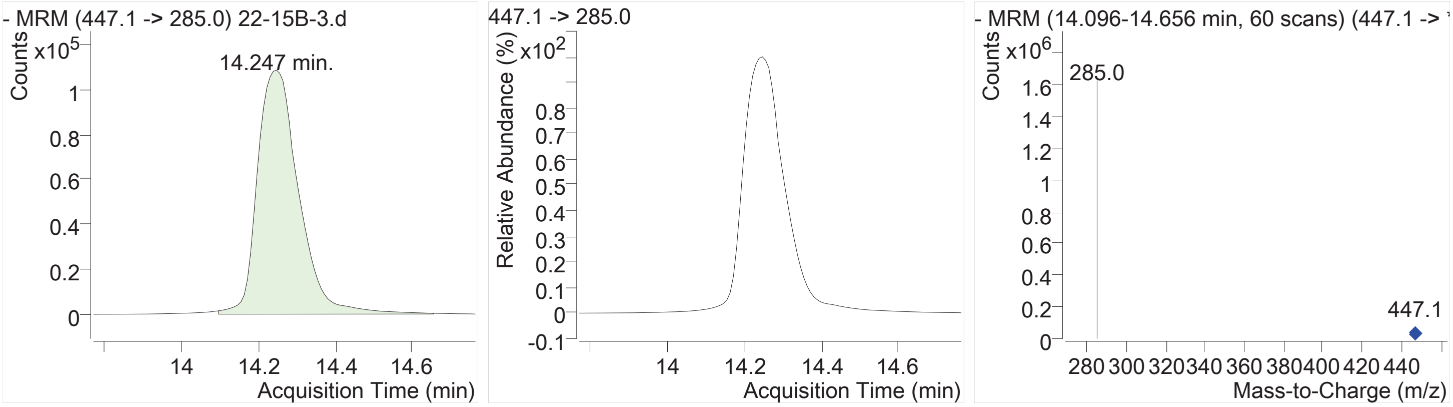

Hesperidin

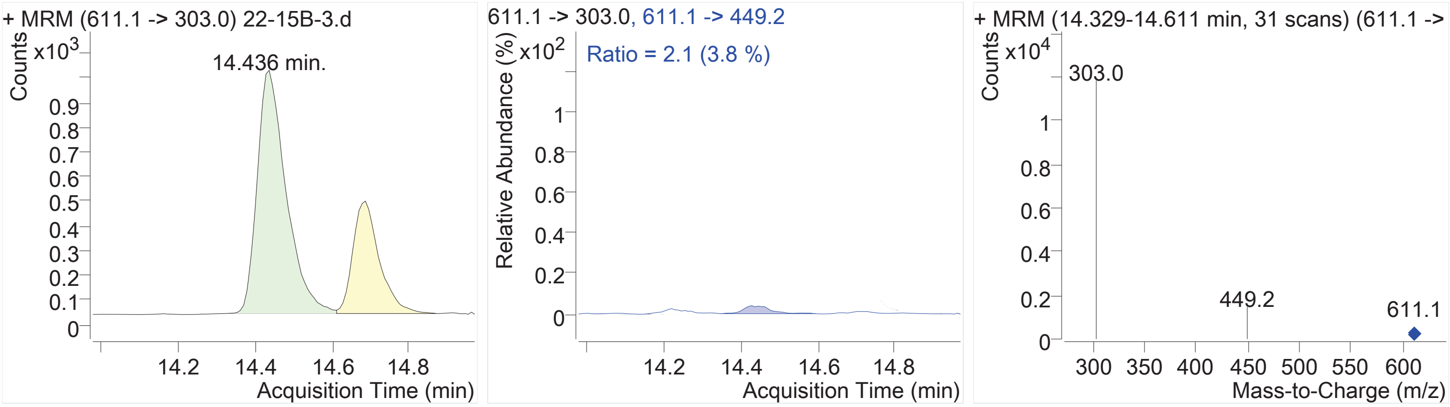

Agilent Technologies

Hyperoside

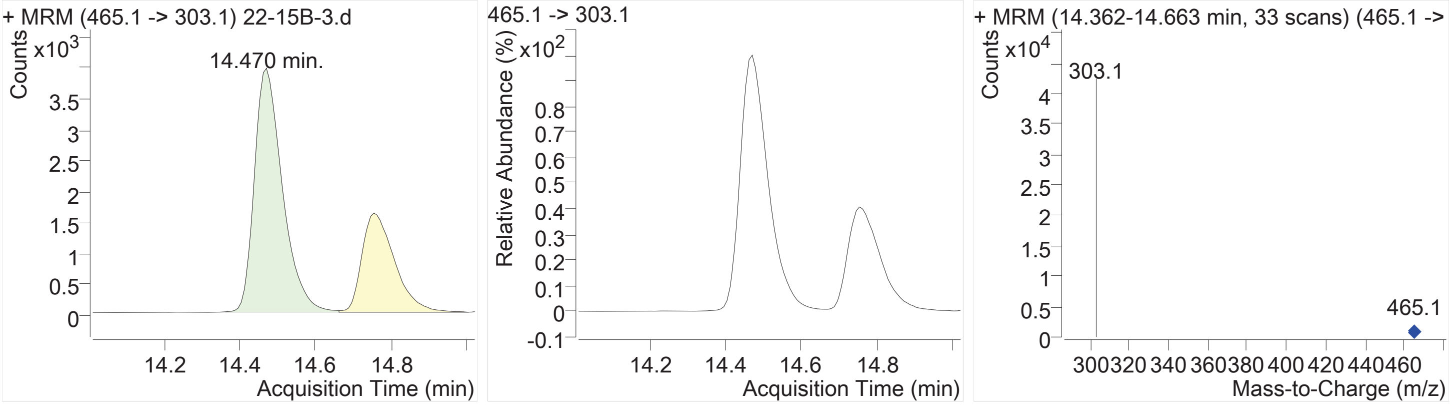

Rosmarinic acid

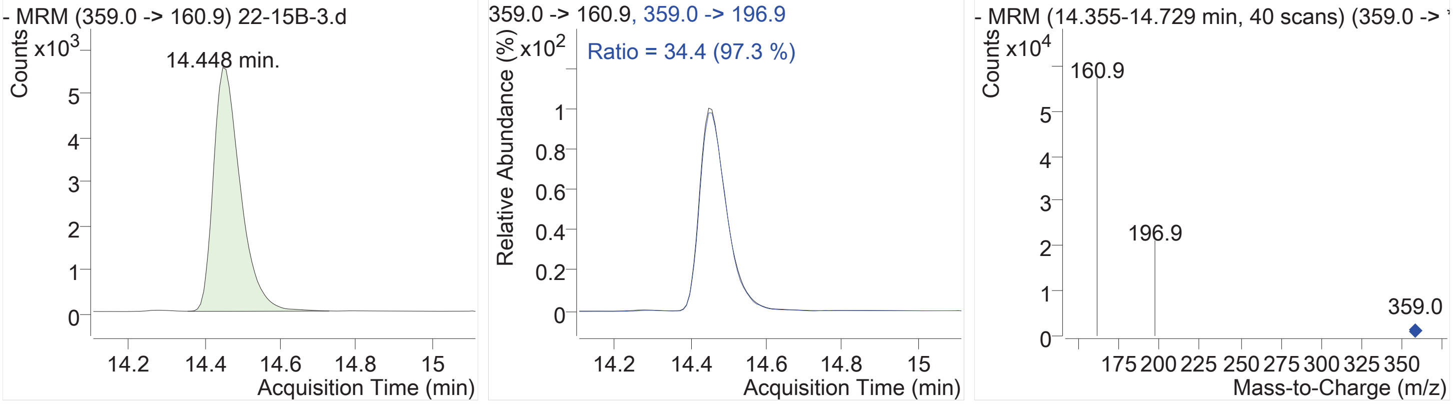

Apigenin 7-glucoside

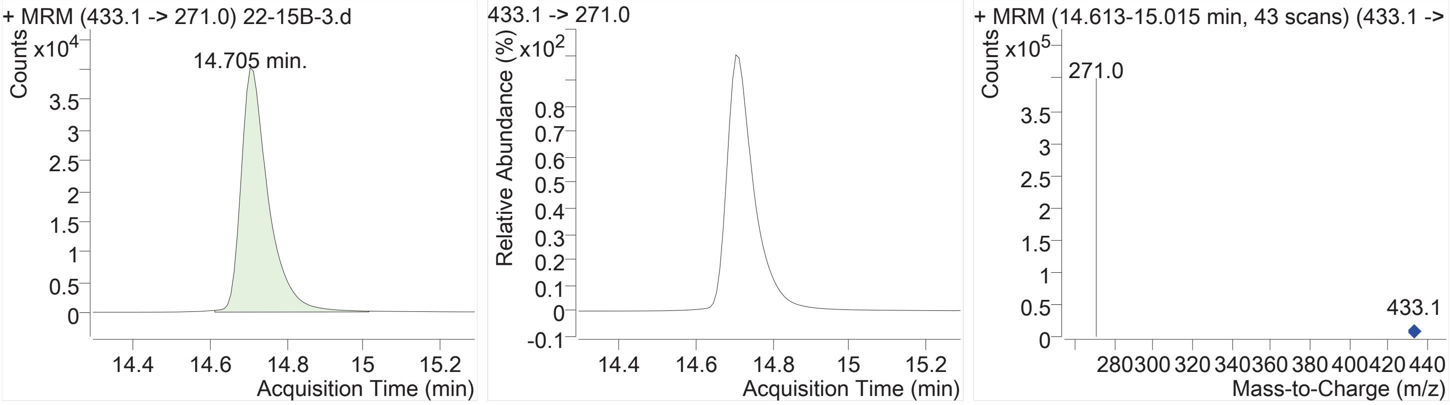

Pinoresinol

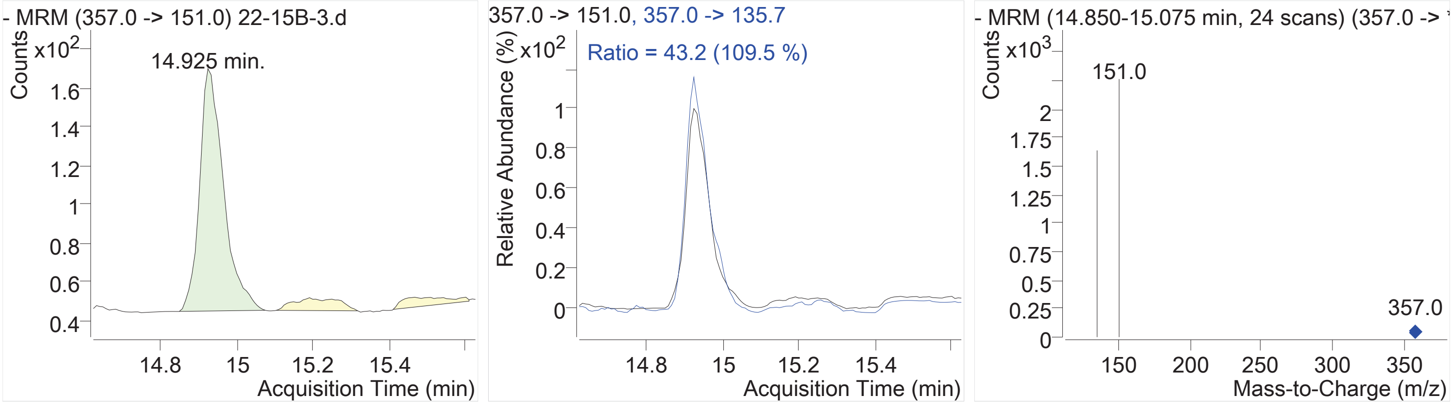

2-Hydroxycinnamic acid

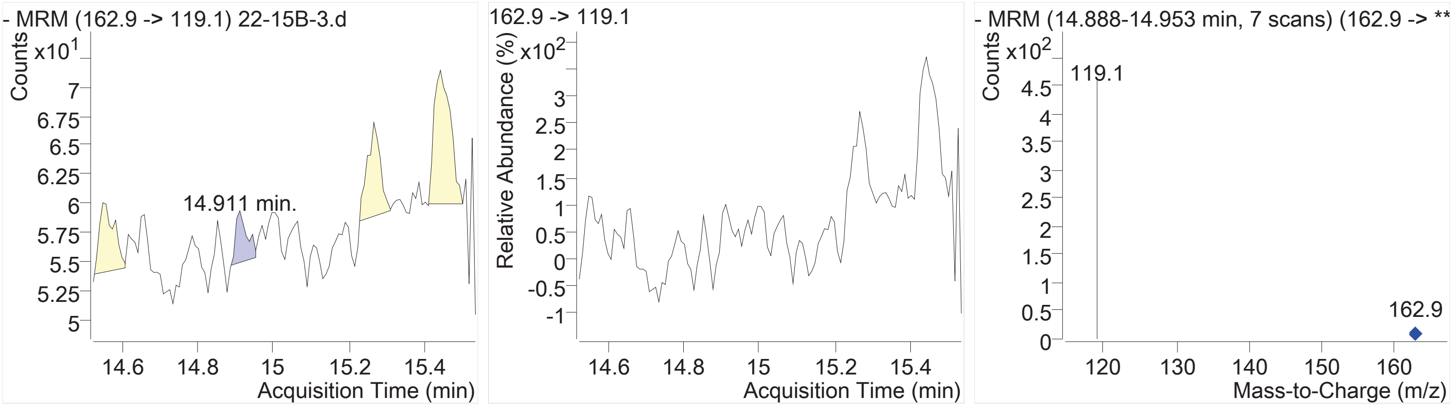

Eriodictyol

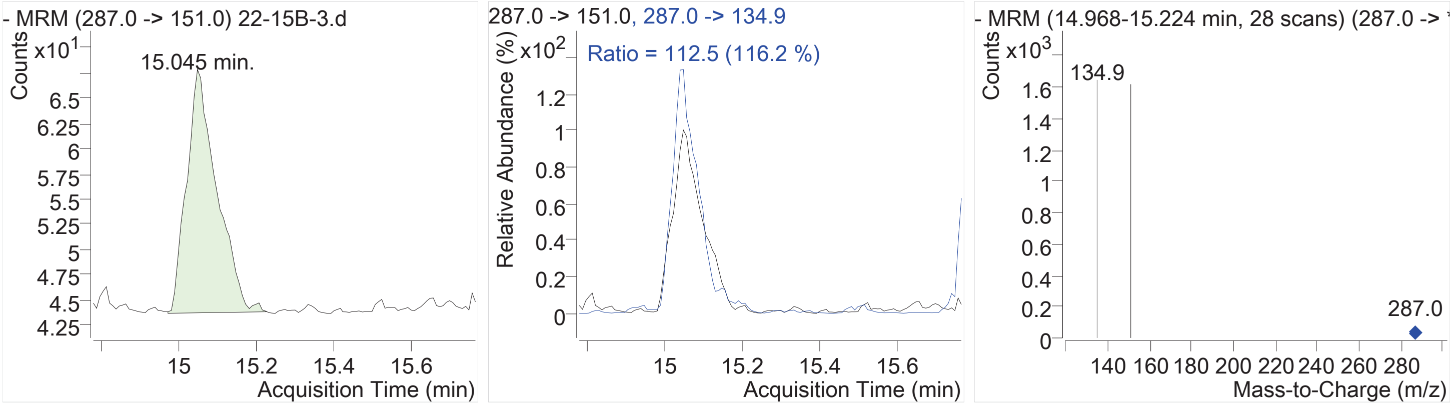

Quercetin

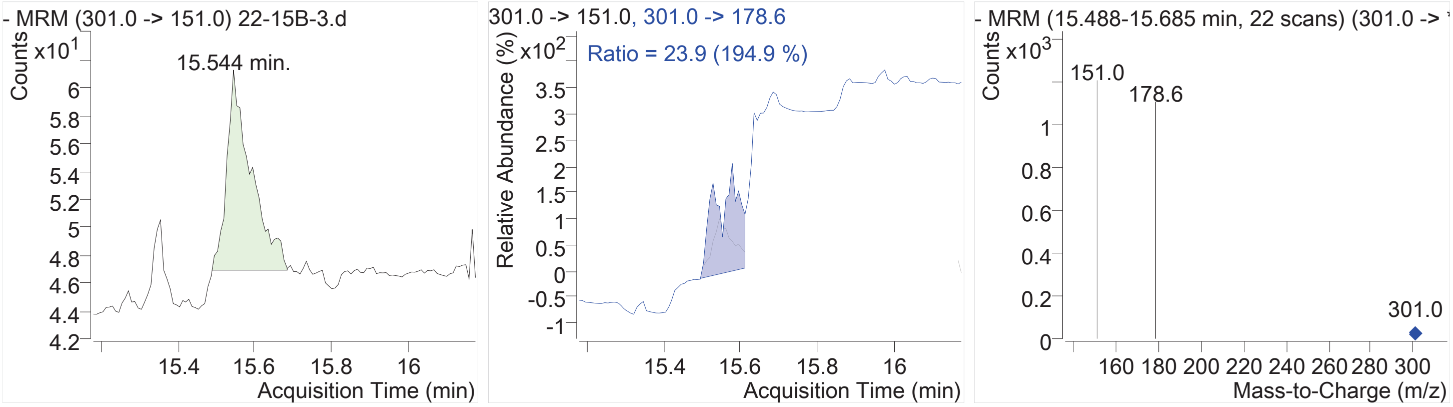

Luteolin

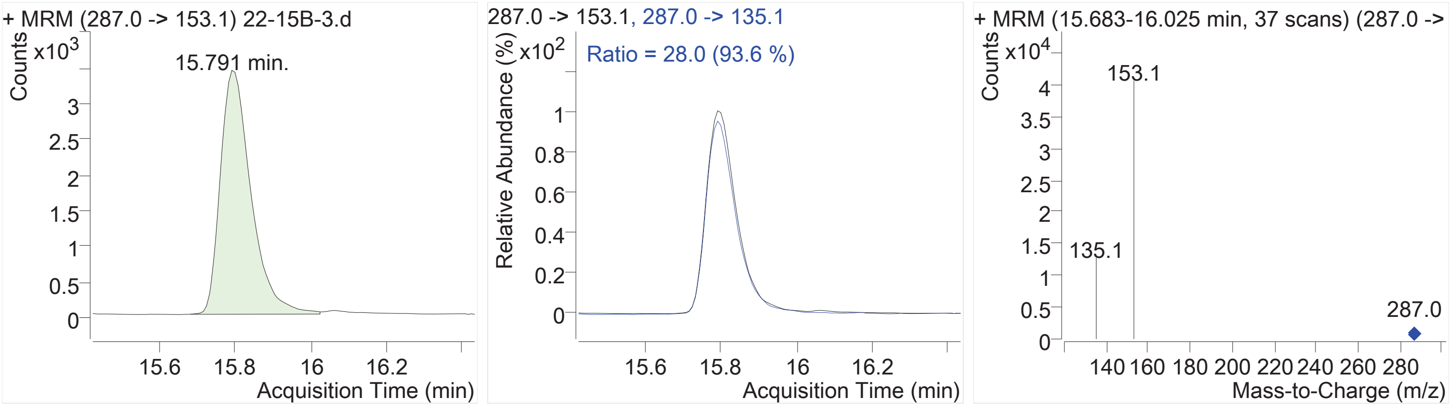

Kaempferol

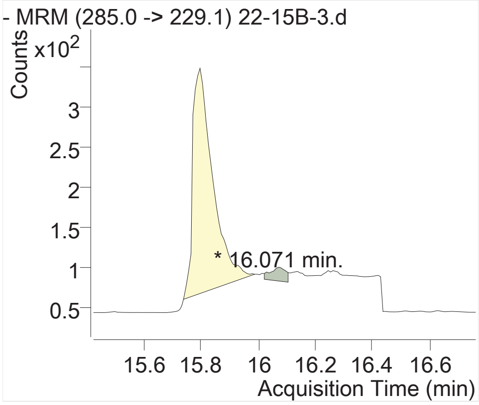

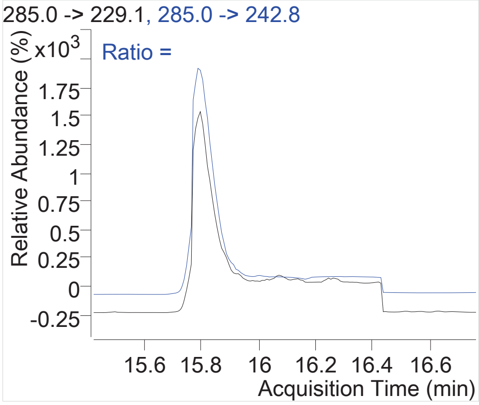

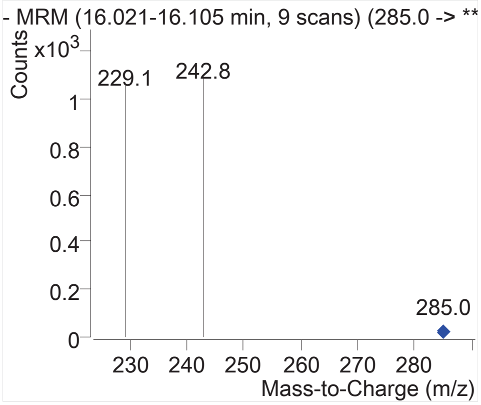

Apigenin

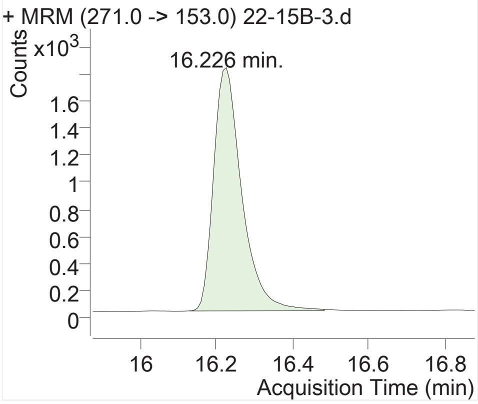

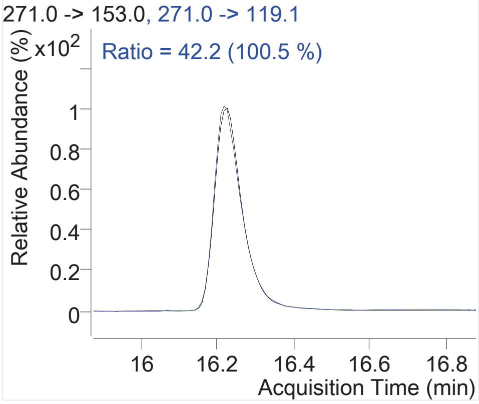

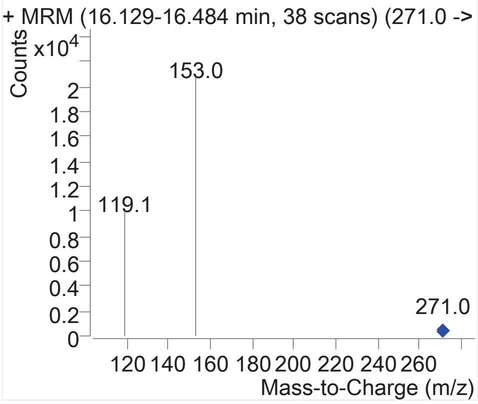

Quantitative Analysis Complete Report

Agilent Technologies

**Batch Path**	D:\MassHunter\Data\2022ekim\061022cengizhoca\QuantResults\071022.batch.bin		
**Analysis Time**	10/11/2022 1:33:26 PM	**Analyst Name**	Defam-PC\admin
**Report Time**	10/11/2022 1:42:11 PM	**Reporter Name**	admin
**Last Calib Update**	10/11/2022 1:33:17 PM	**Batch State**	Processed
**Quant Batch Version**	B.07.01	**Quant Report Version**	B.07.01

**Acq. Time**	10/9/2022 4:39:16 AM	**Data File**	22-15C-1.d
**Sample Type**	Sample	**Sample Name**	22-15C-1
**Dilution**	1	**Acq. Method**	FENOLIK_DMRM2021-31bilesen.m

Sample Chromatogram

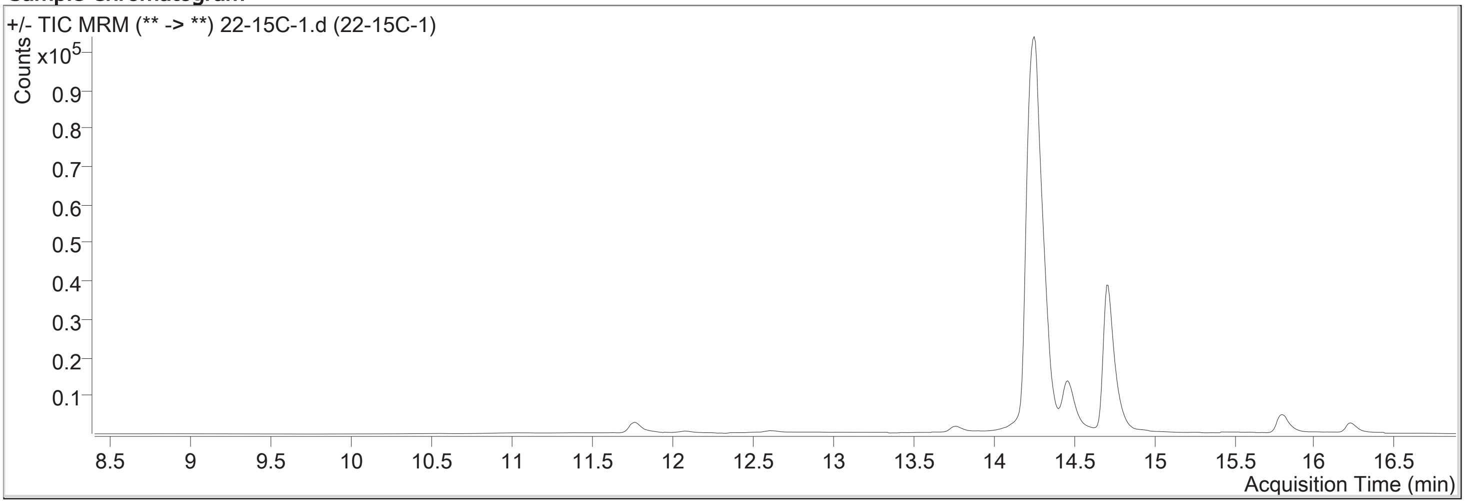

Compound	Transition	RT	Resp.	Final Conc	Units
Gallic acid	168.9 -> 125.0	8.783	2	ND	ng/ml
Protocatechuic acid	152.9 -> 108.9	10.559	546	41.0202	ng/ml
Pyrocatechol	109.0 -> 52.9	10.805	3	ND	ng/ml
3,4-Dihydroxyphenylacetic acid	167.0 -> 123.0	10.921	5	ND	ng/ml
(+)-Catechin	289.0 -> 245.0	11.386	35	ND	ng/ml
2,5-Dihydroxybenzoic acid	152.9 -> 109.0	11.979	62	10.3447	ng/ml
Chlorogenic acid	355.0 -> 163.0	11.777	18178	859.0215	ng/ml
3-Hydroxybenzoic acid	137.0 -> 93.0	12.828	13	17.4722	ng/ml
4-Hydroxybenzoic acid	136.9 -> 93.1	12.081	2367	204.0907	ng/ml
(-)-Epicatechin	291.0 -> 139.1	12.293	4	3.6051	ng/ml
Caffeic acid	179.0 -> 135.0	12.624	2434	76.1257	ng/ml
Syringic acid	196.9 -> 181.9	12.740	109	154.9537	ng/ml
Vanillin	151.0 -> 136.0	13.028	140	20.7779	ng/ml
Verbascoside	623.0 -> 160.8	13.710	3	ND	ng/ml
Taxifolin	303.0 -> 285.1	13.653	29	ND	ng/ml
p-Coumaric acid	162.9 -> 119.0	13.766	8028	304.0516	ng/ml
Sinapic acid	222.9 -> 207.9	13.831	26	12.6472	ng/ml
Ferulic acid	193.0 -> 134.0	13.899	229	44.2342	ng/ml
Luteolin 7-glucoside	447.1 -> 285.0	14.255	748291	8187.4585	ng/ml
Hesperidin	611.1 -> 303.0	14.444	4561	557.7034	ng/ml
Hyperoside	465.1 -> 303.1	14.479	17769	903.2709	ng/ml
Rosmarinic acid	359.0 -> 160.9	14.464	24720	1975.5095	ng/ml
Apigenin 7-glucoside	433.1 -> 271.0	14.713	185729	5522.3942	ng/ml
Pinoresinol	357.0 -> 151.0	14.934	499	1942.3786	ng/ml
2-Hydroxycinnamic acid	162.9 -> 119.1	15.063	27	ND	ng/ml
Eriodictyol	287.0 -> 151.0	15.053	129	ND	ng/ml
Quercetin	301.0 -> 151.0	15.569	80	ND	ng/ml
Luteolin	287.0 -> 153.1	15.800	17156	1078.8880	ng/ml
Kaempferol	285.0 -> 229.1	16.080	18	ND	ng/ml

Agilent Technologies

Compound	Transition	RT	Resp.	Final Conc	Units
Apigenin	271.0 -> 153.0	16.226	8997	481.1655	ng/ml

Gallic acid

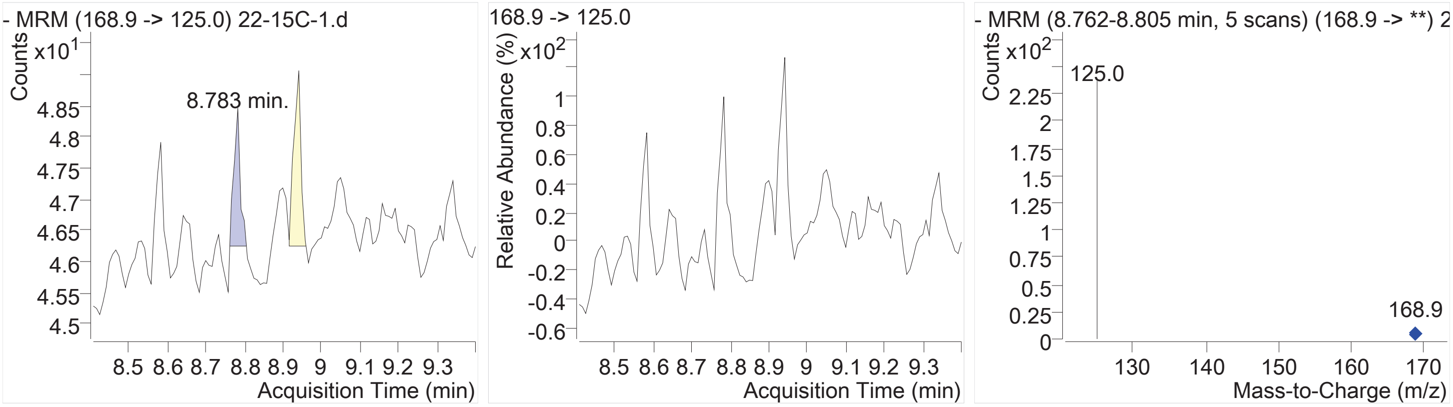

Protocatechuic acid

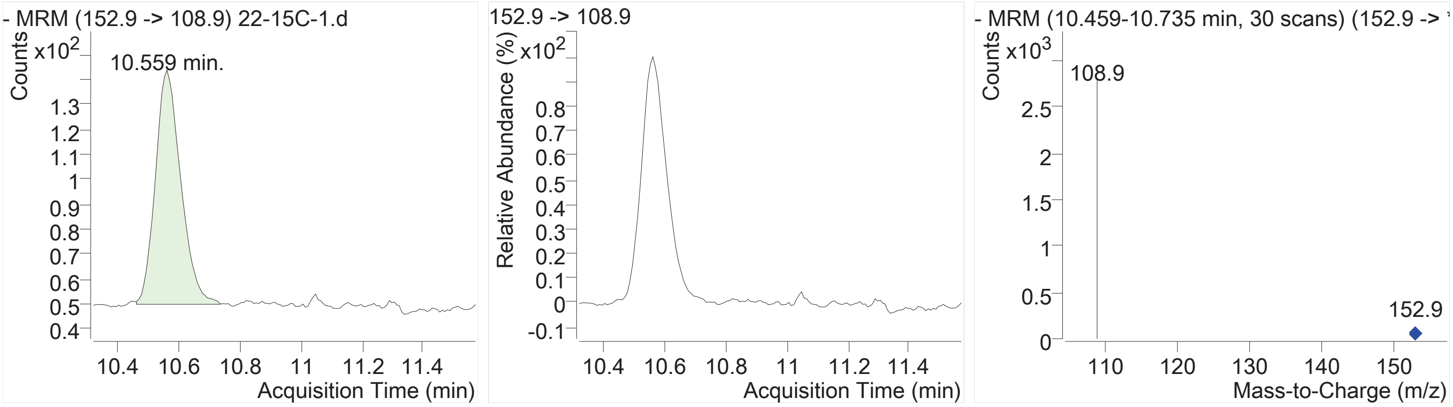

Pyrocatechol

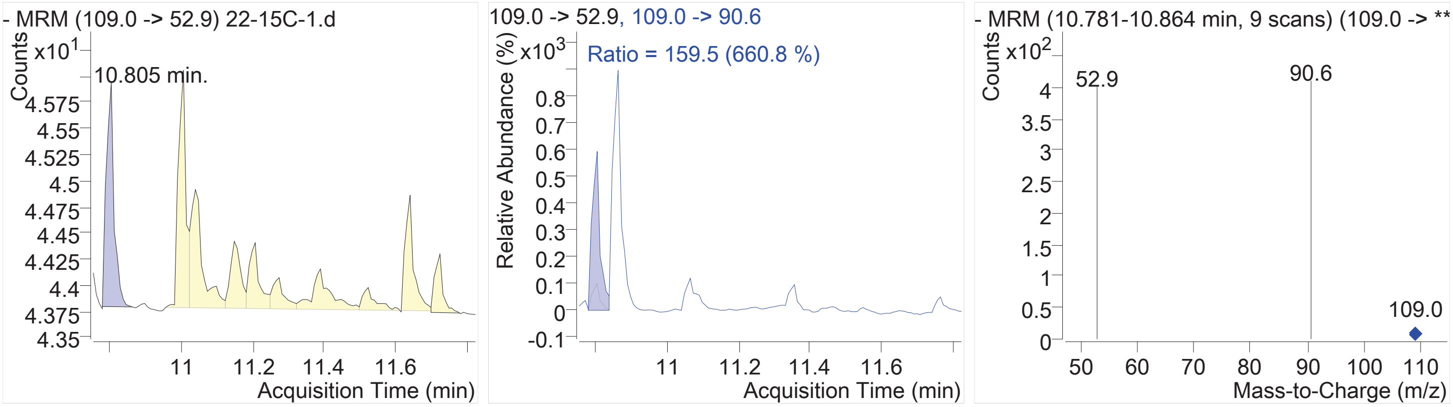

3,4-Dihydroxyphenylacetic acid

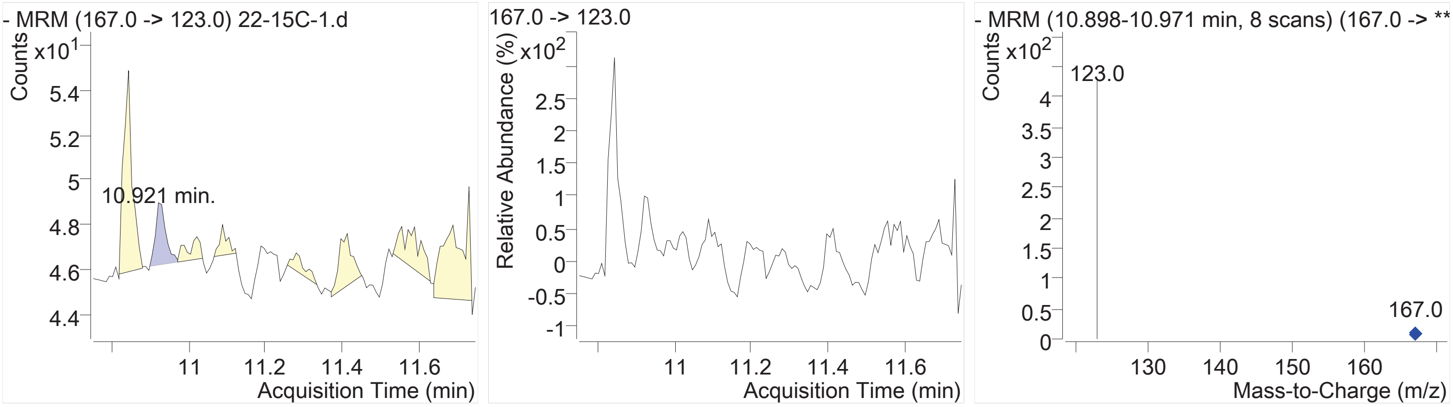

(+)-Catechin

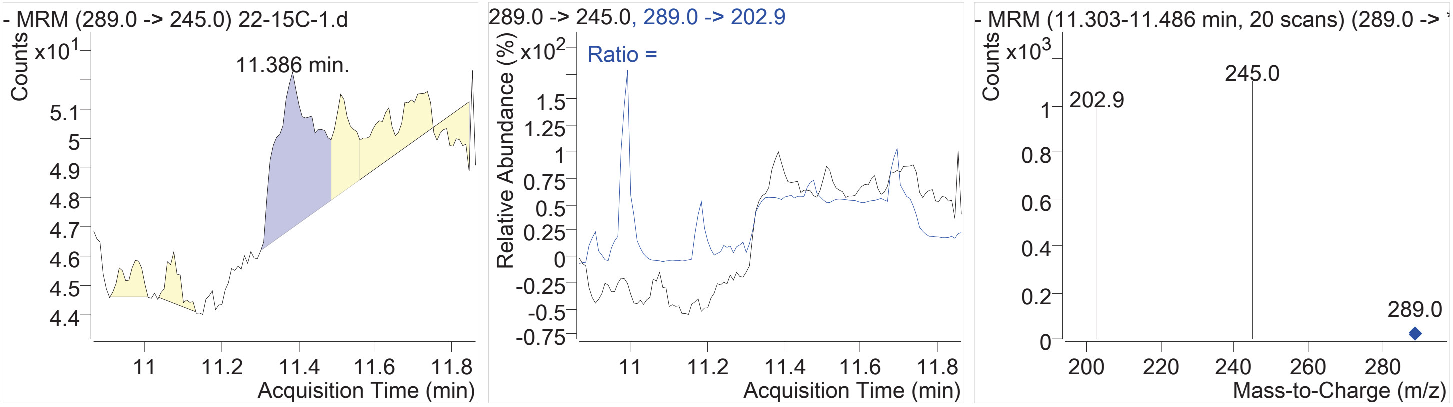

2,5-Dihydroxybenzoic acid

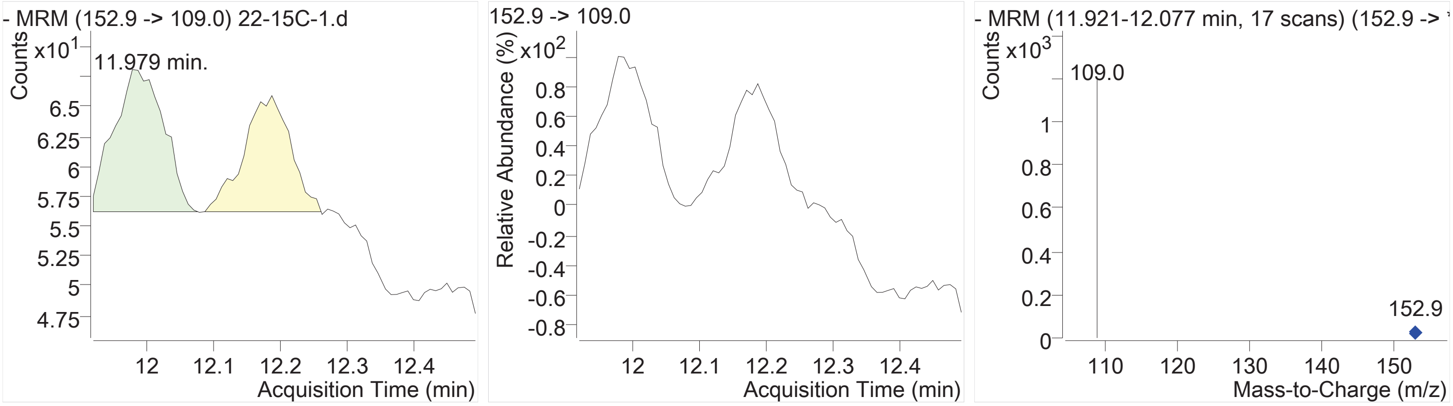

Chlorogenic acid

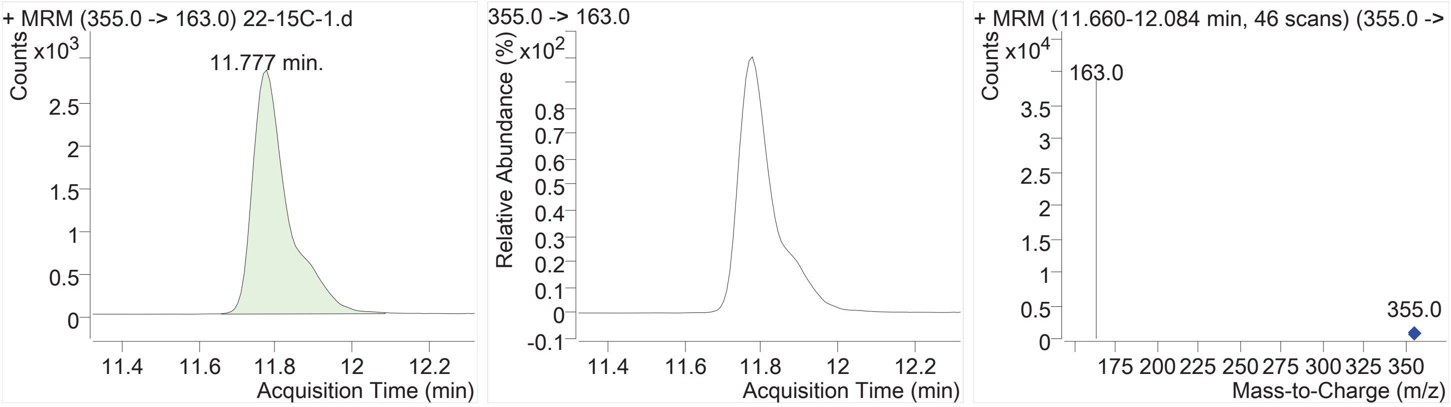

3-Hydroxybenzoic acid

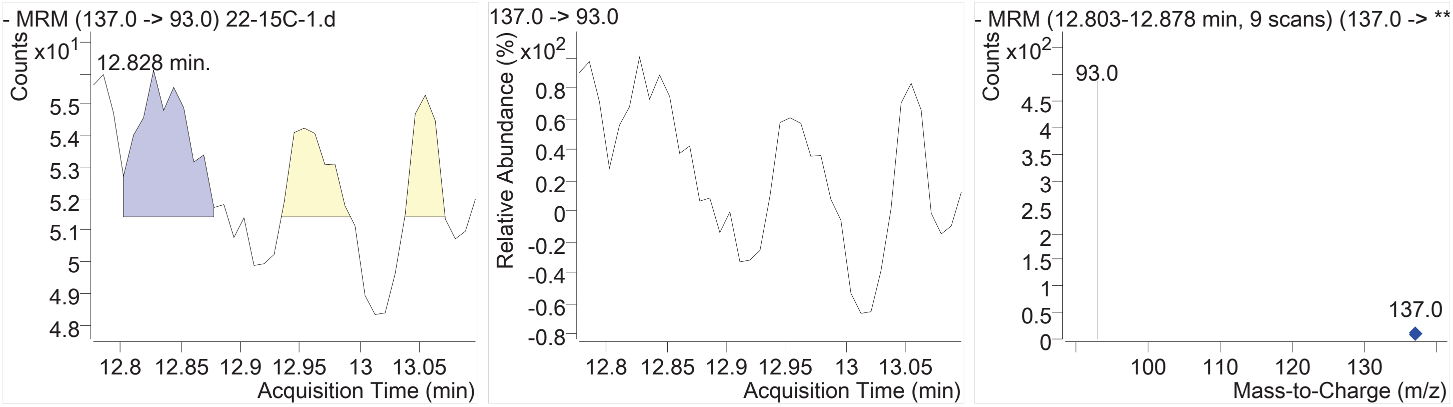

4-Hydroxybenzoic acid

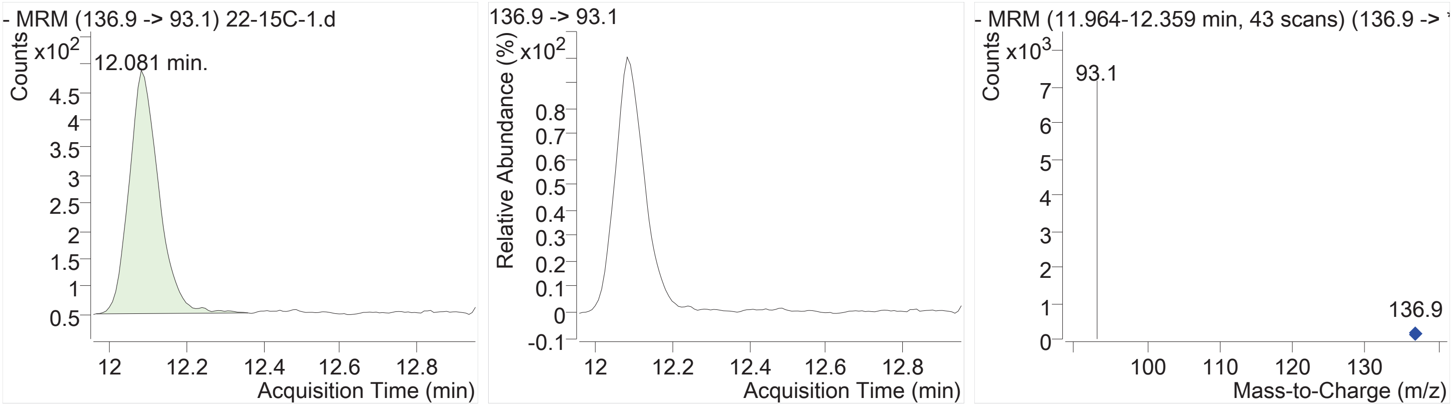

(-)-Epicatechin

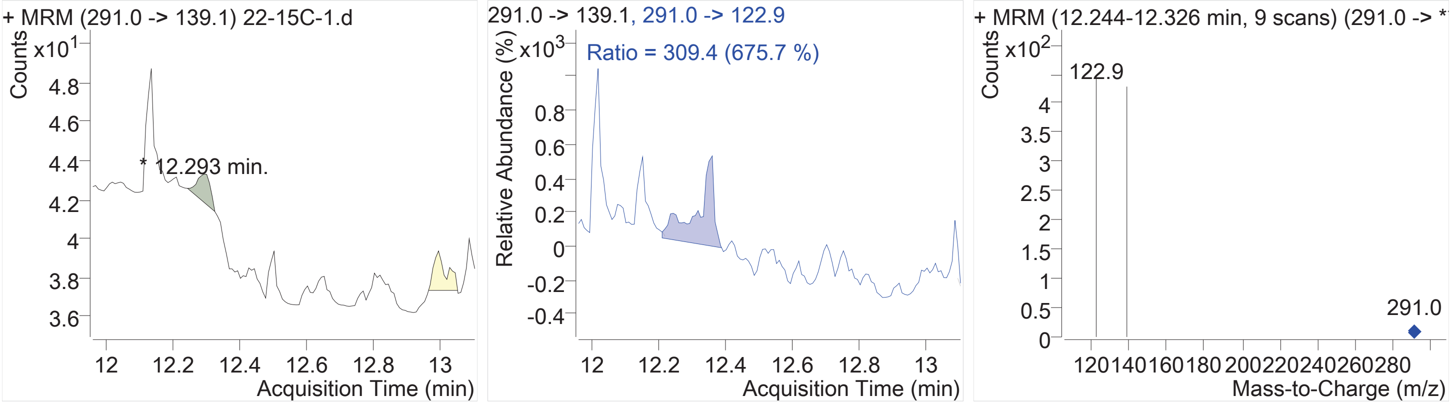

Caffeic acid

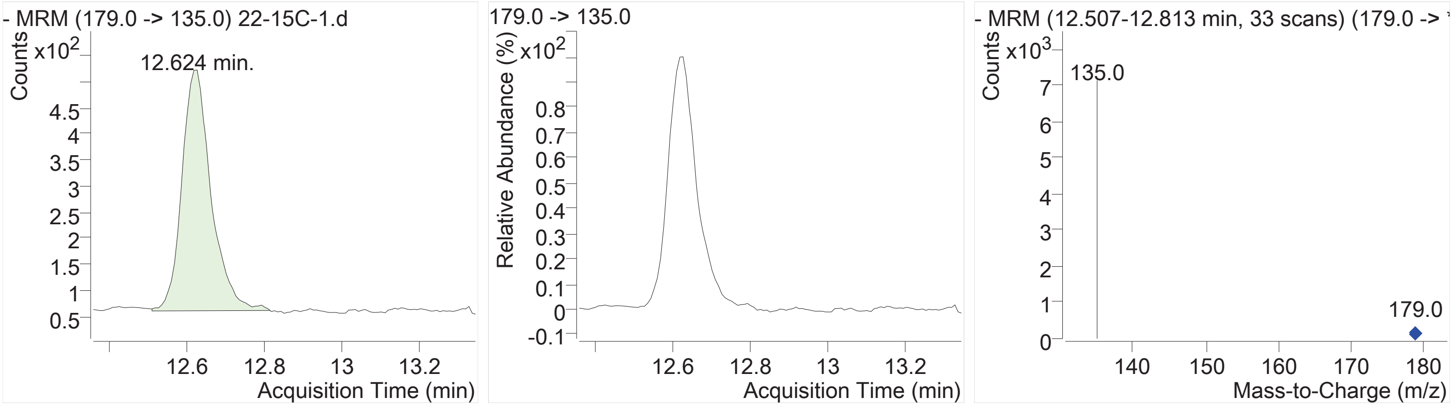

Syringic acid

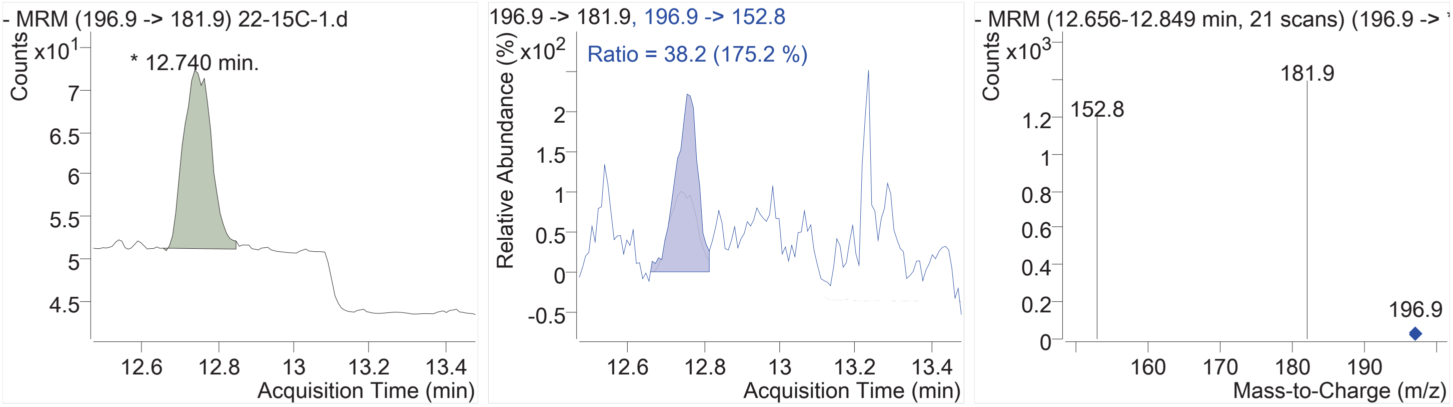

Agilent Technologies

Vanillin

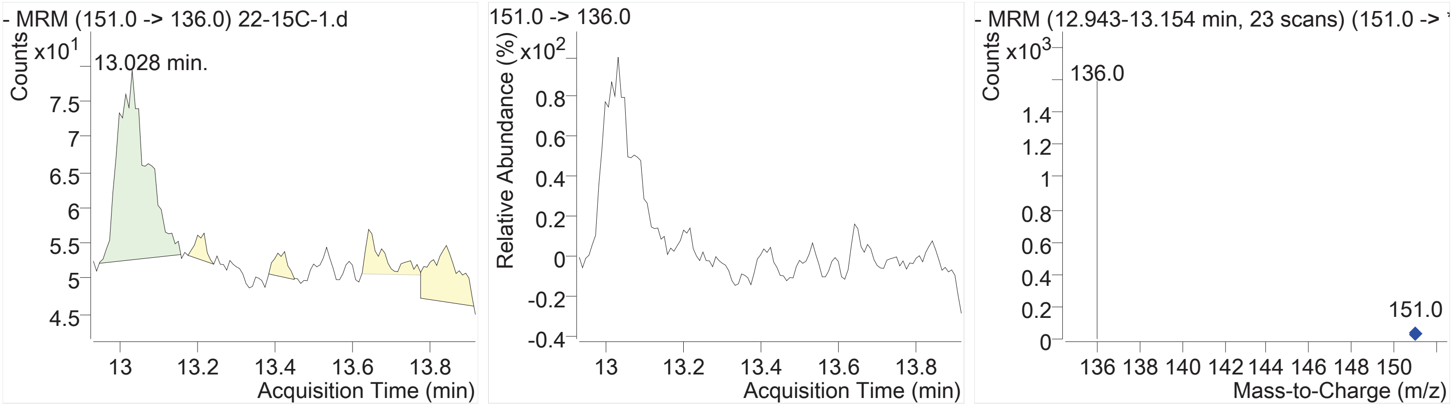

Verbascoside

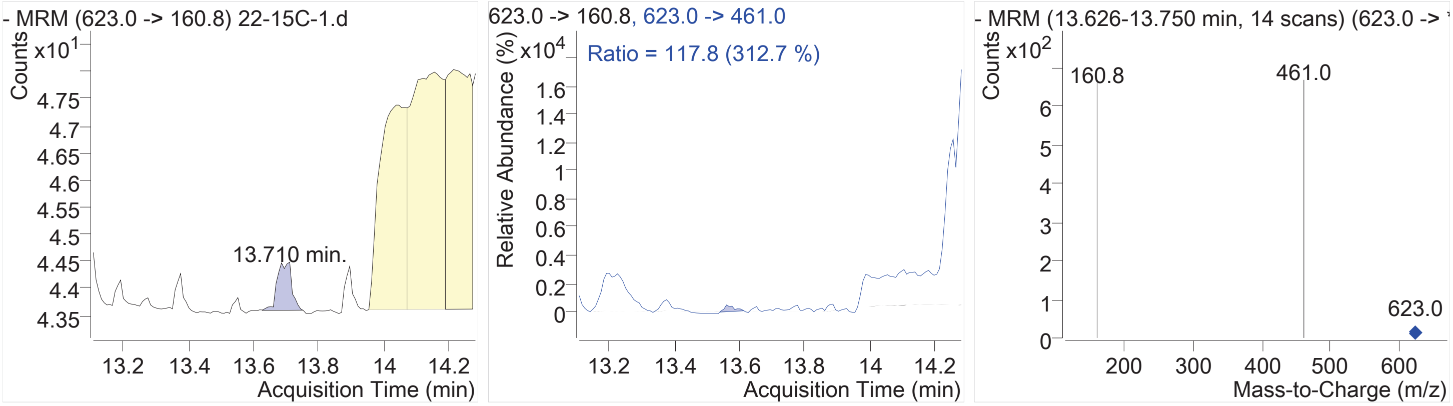

Taxifolin

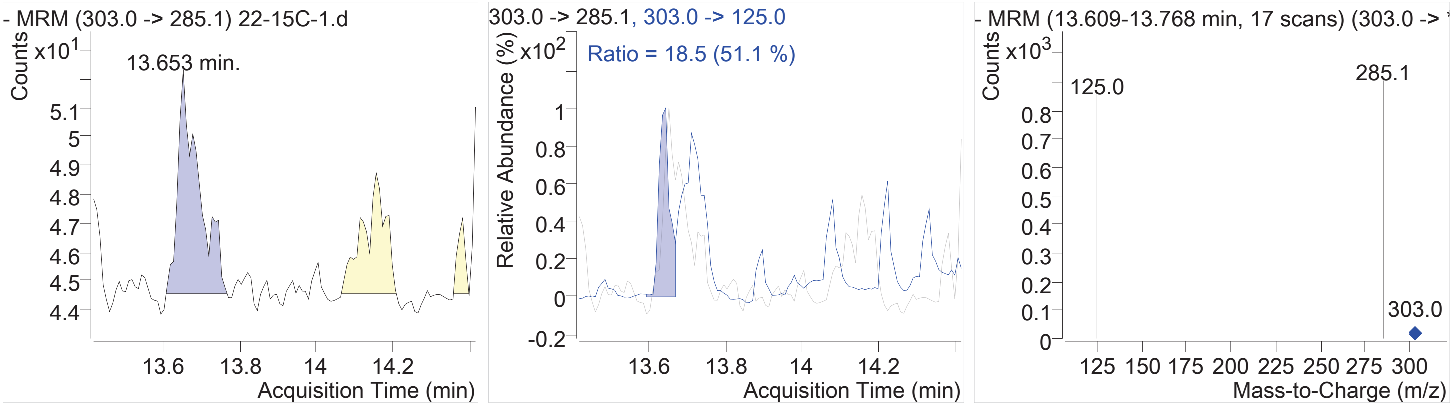

p-Coumaric acid

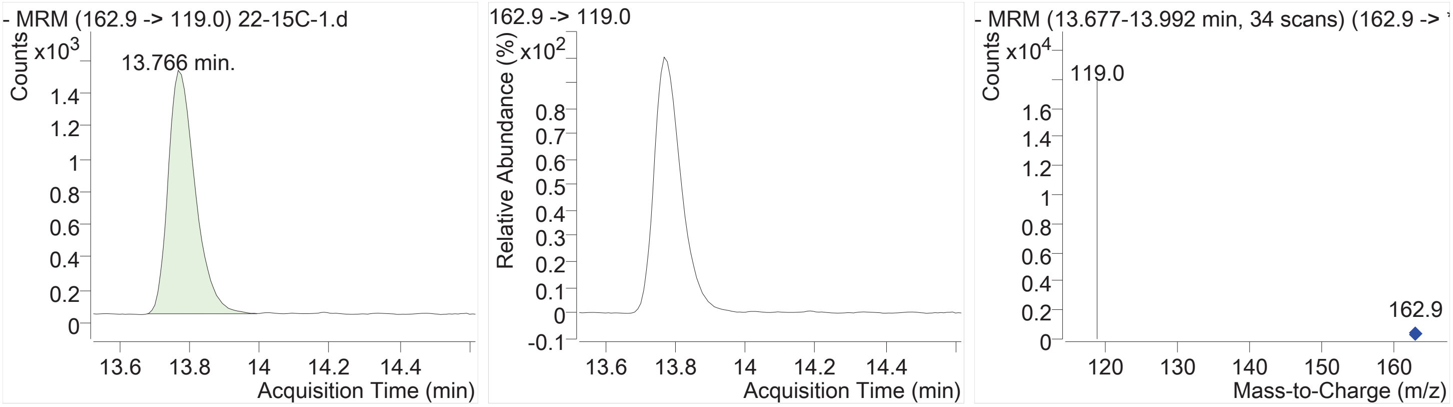

Sinapic acid

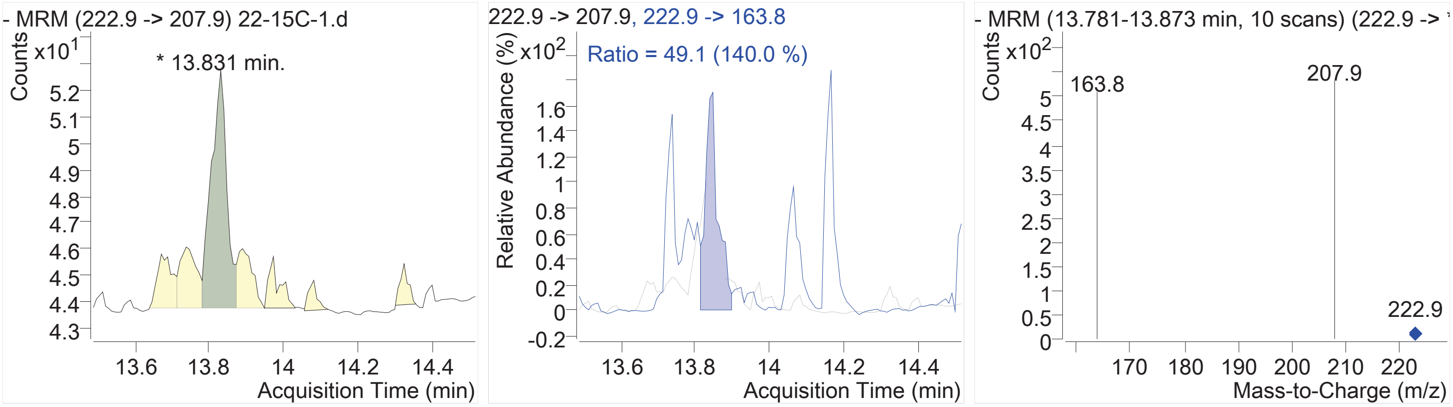

Ferulic acid

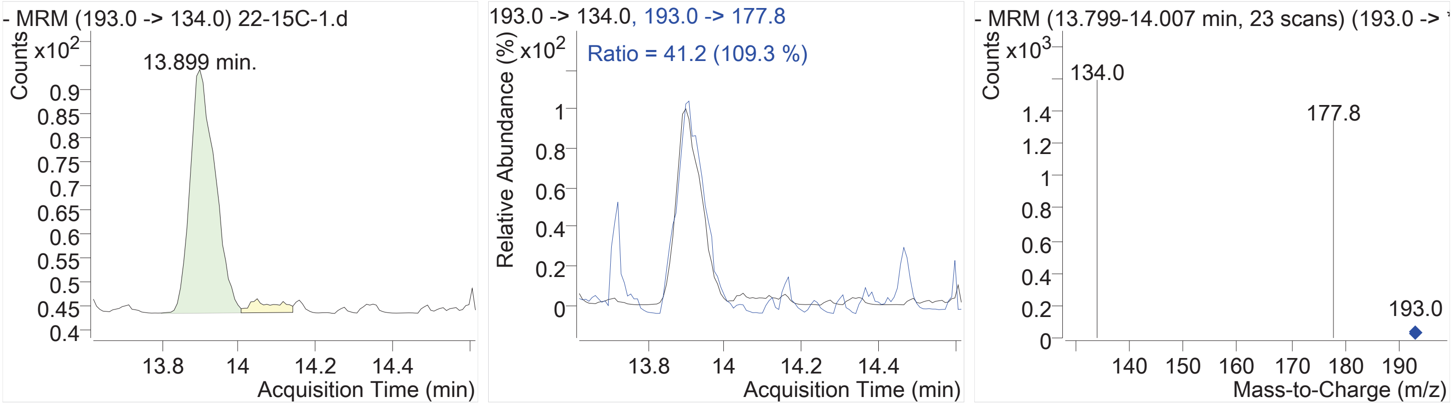

Luteolin 7-glucoside

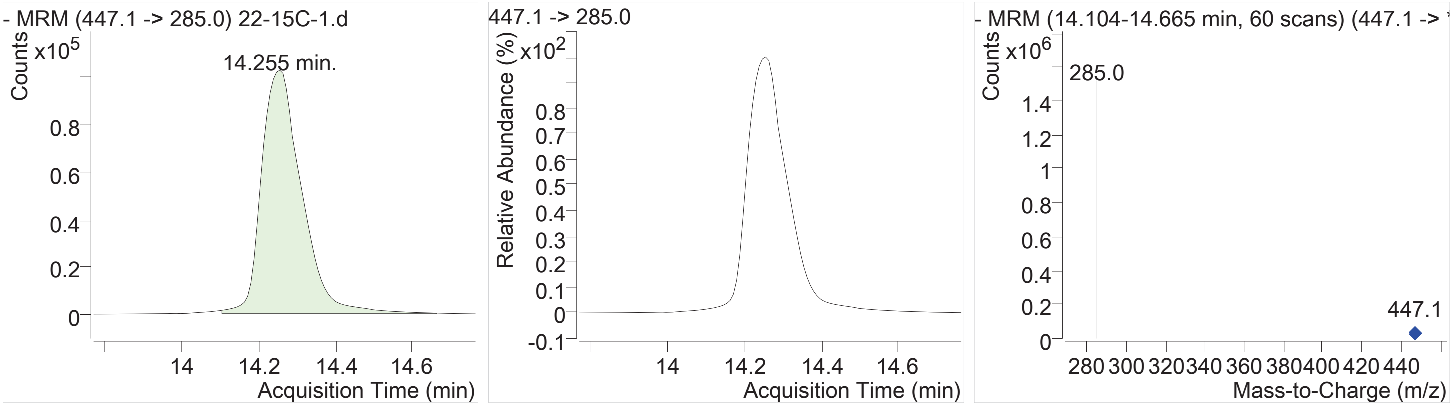

Hesperidin

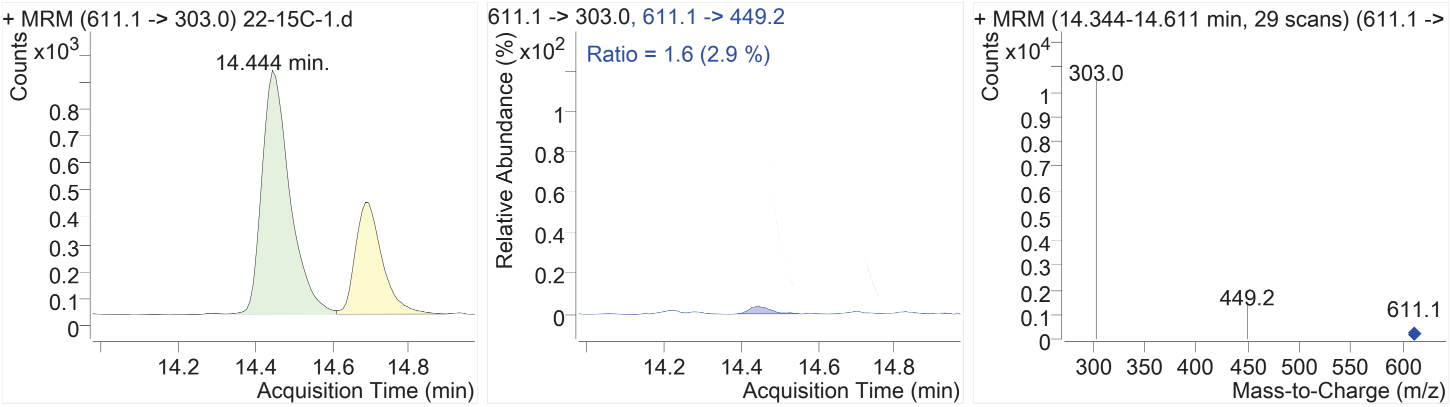

Hyperoside

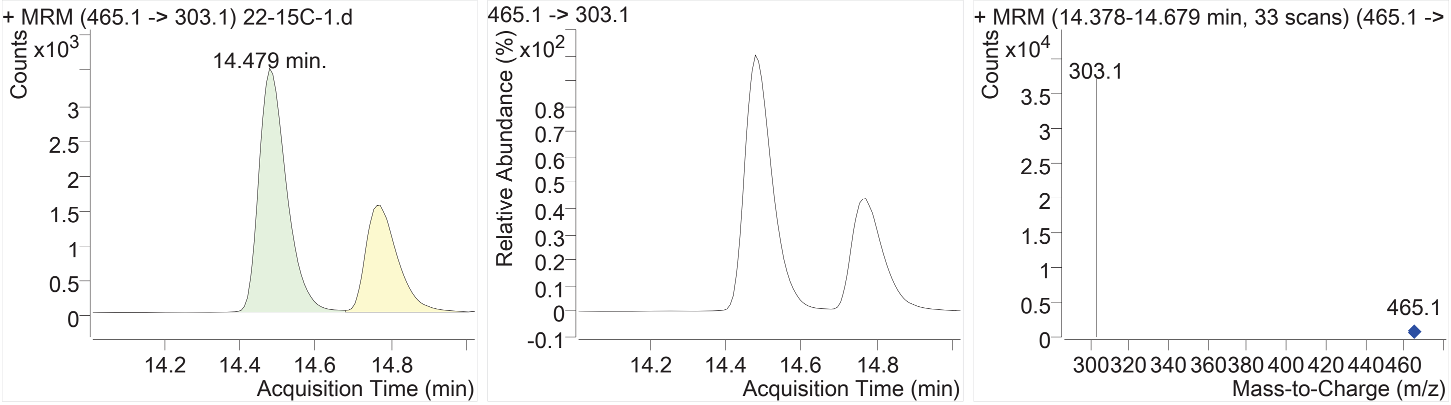

Rosmarinic acid

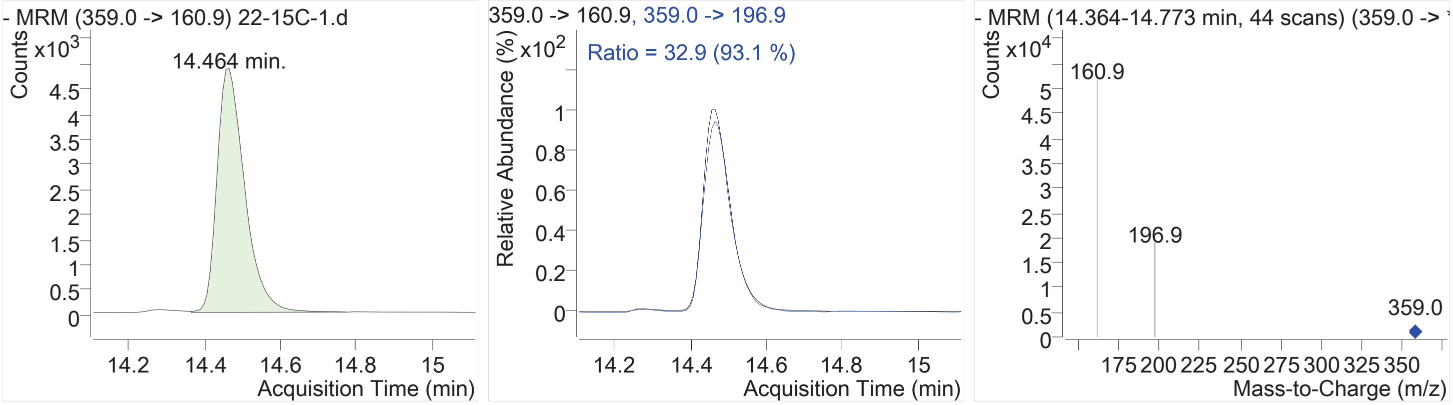

Apigenin 7-glucoside

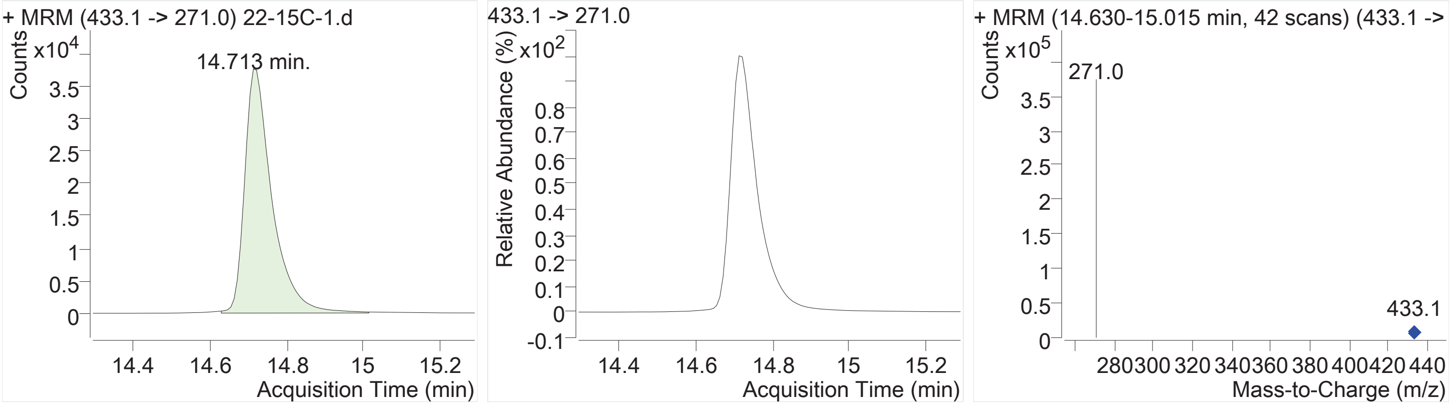

Pinoresinol

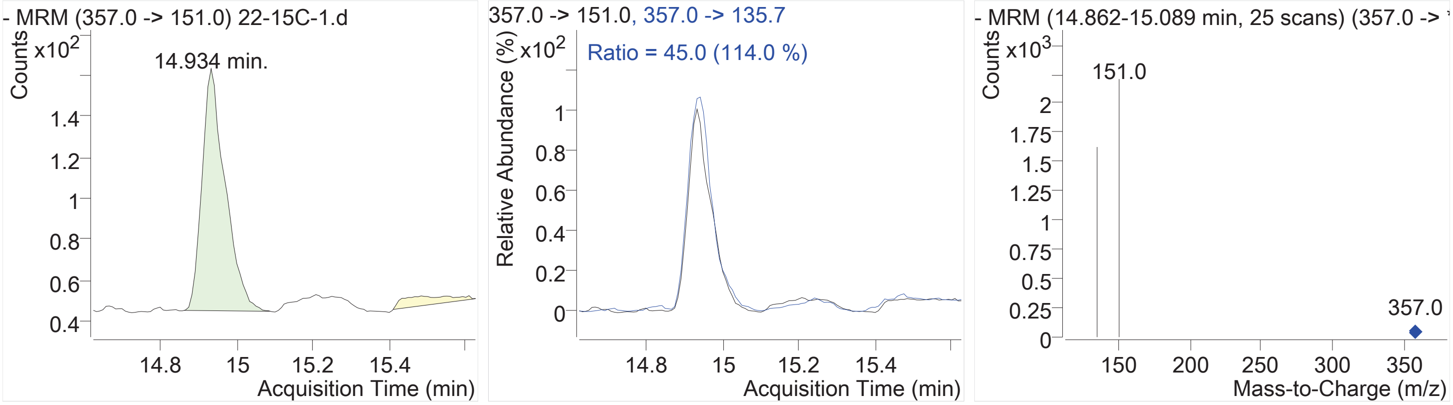

2-Hydroxycinnamic acid

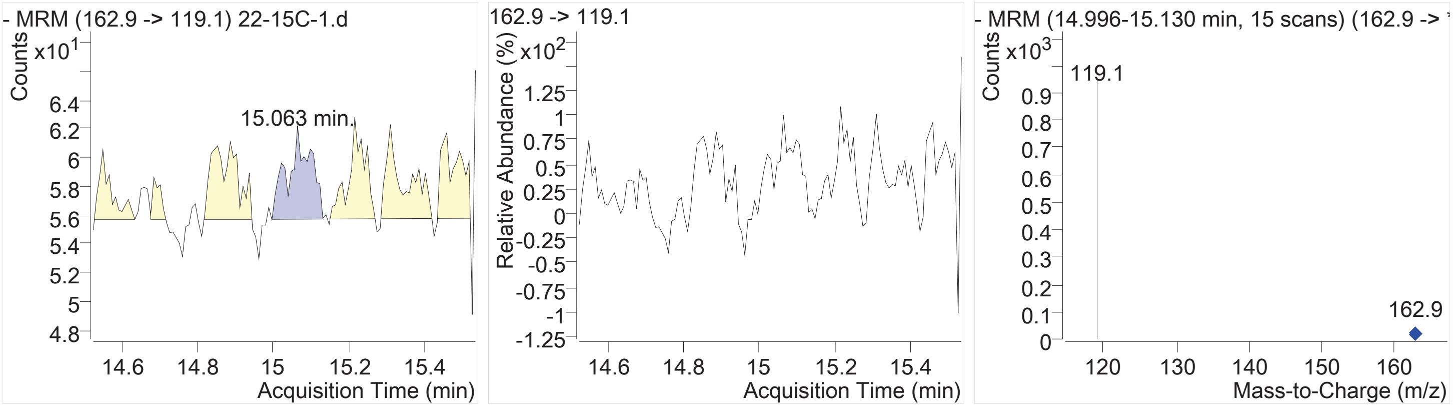

Eriodictyol

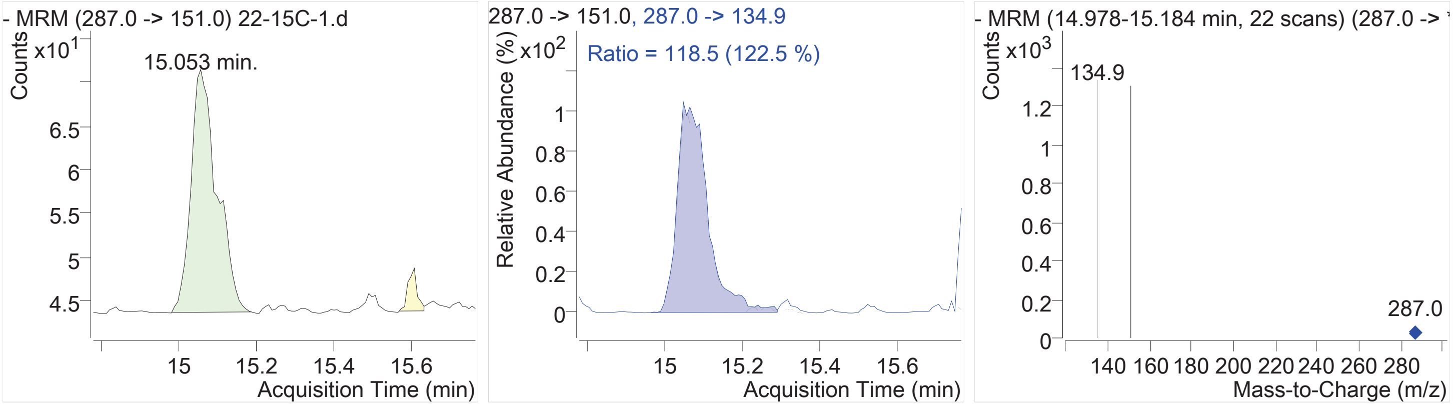

Quercetin

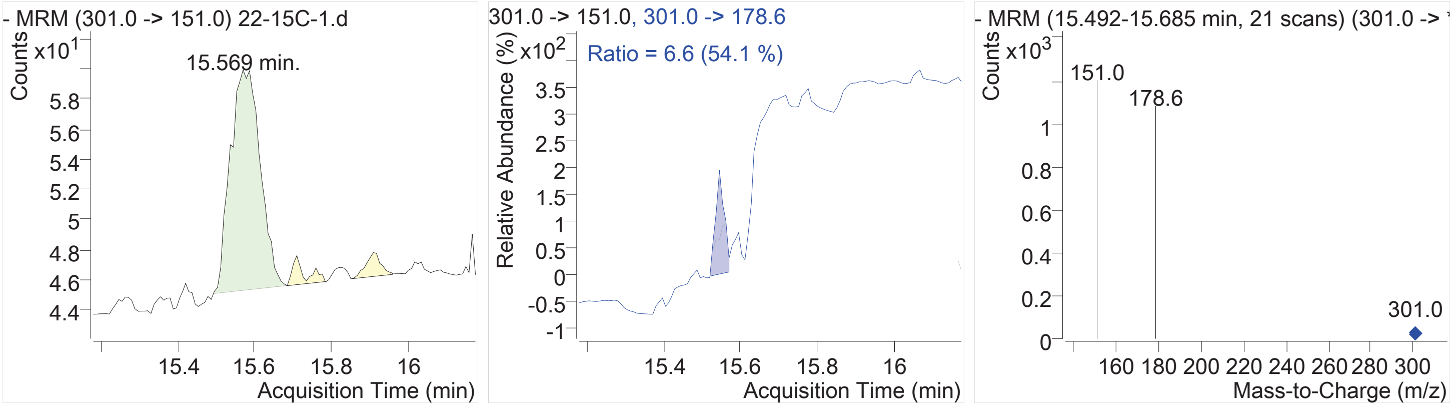

Luteolin

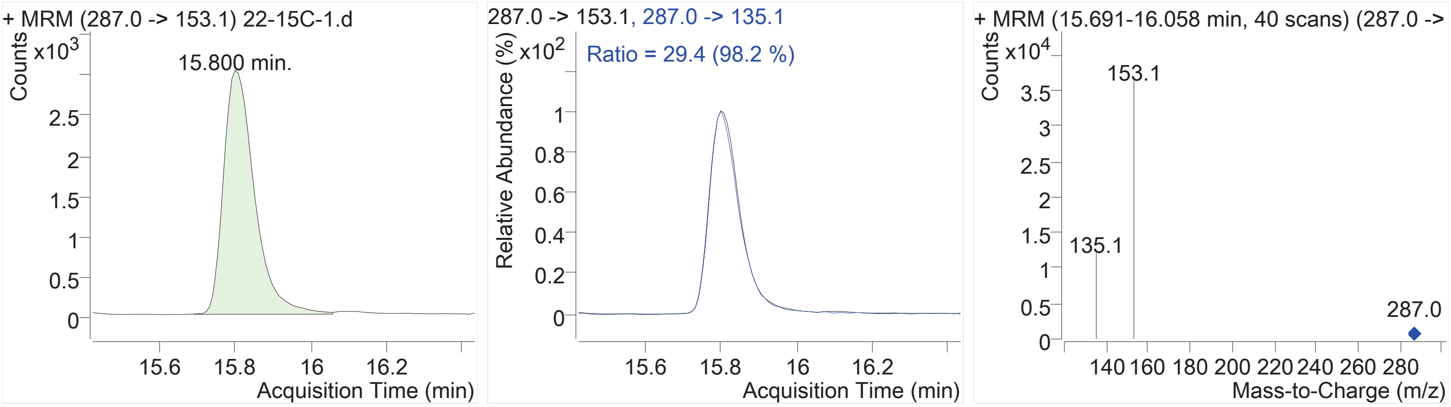

Agilent Technologies

Kaempferol

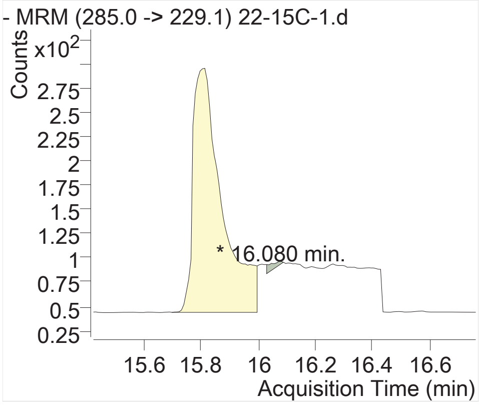

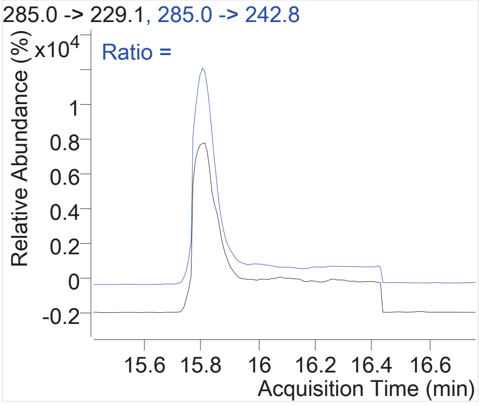

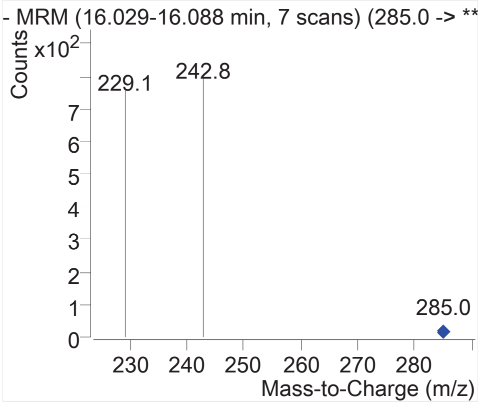

Apigenin

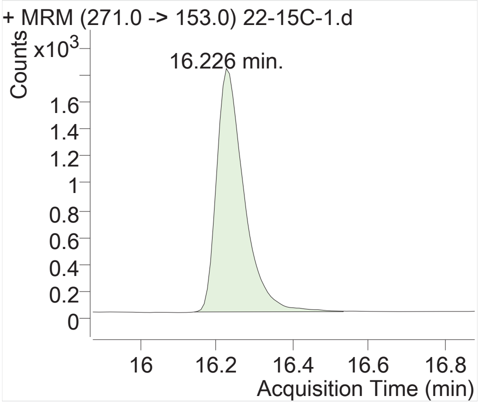

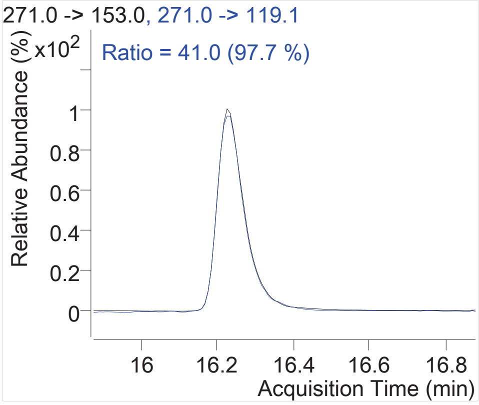

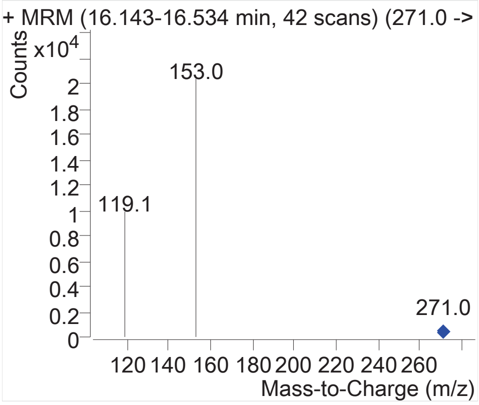

Quantitative Analysis Complete Report

**Batch Path**	D:\MassHunter\Data\2022ekim\061022cengizhoca\QuantResults\071022.batch.bin		
**Analysis Time**	10/11/2022 1:33:26 PM	**Analyst Name**	Defam-PC\admin
**Report Time**	10/11/2022 1:42:14 PM	**Reporter Name**	admin
**Last Calib Update**	10/11/2022 1:33:17 PM	**Batch State**	Processed
**Quant Batch Version**	B.07.01	**Quant Report Version**	B.07.01

**Acq. Time**	10/9/2022 5:00:58 AM	**Data File**	22-15C-2.d
**Sample Type**	Sample	**Sample Name**	22-15C-2
**Dilution**	1	**Acq. Method**	FENOLIK_DMRM2021-31bilesen.m

Sample Chromatogram

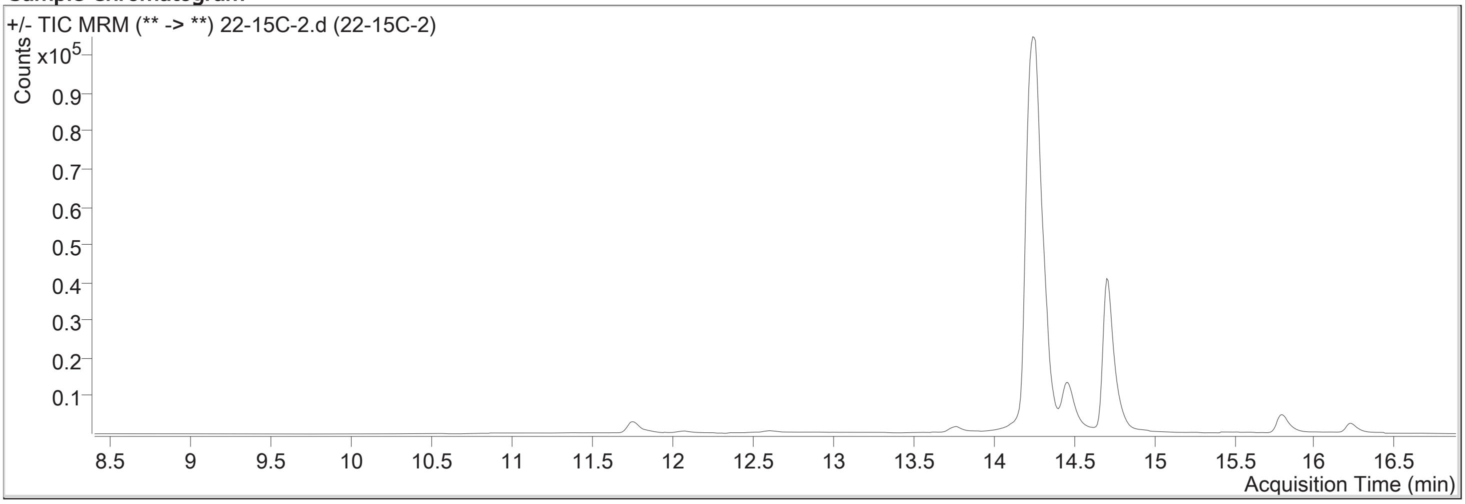

Compound	Transition	RT	Resp.	Final Conc	Units
Gallic acid	168.9 -> 125.0	8.808	4	ND	ng/ml
Protocatechuic acid	152.9 -> 108.9	10.559	527	39.1793	ng/ml
Pyrocatechol	109.0 -> 52.9	10.864	4	ND	ng/ml
3,4-Dihydroxyphenylacetic acid	167.0 -> 123.0	10.888	1	ND	ng/ml
(+)-Catechin	289.0 -> 245.0	11.386	68	ND	ng/ml
2,5-Dihydroxybenzoic acid	152.9 -> 109.0	11.954	57	9.6157	ng/ml
Chlorogenic acid	355.0 -> 163.0	11.760	19487	920.4916	ng/ml
3-Hydroxybenzoic acid	137.0 -> 93.0	12.819	7	16.3213	ng/ml
4-Hydroxybenzoic acid	136.9 -> 93.1	12.072	2520	218.3544	ng/ml
(-)-Epicatechin	291.0 -> 139.1	12.293	4	3.6638	ng/ml
Caffeic acid	179.0 -> 135.0	12.616	2331	71.6853	ng/ml
Syringic acid	196.9 -> 181.9	12.731	120	171.0326	ng/ml
Vanillin	151.0 -> 136.0	13.020	173	30.8544	ng/ml
Verbascoside	623.0 -> 160.8	13.852	2	ND	ng/ml
Taxifolin	303.0 -> 285.1	13.745	10	ND	ng/ml
p-Coumaric acid	162.9 -> 119.0	13.766	7856	297.3239	ng/ml
Sinapic acid	222.9 -> 207.9	13.823	33	14.8436	ng/ml
Ferulic acid	193.0 -> 134.0	13.899	213	41.1756	ng/ml
Luteolin 7-glucoside	447.1 -> 285.0	14.247	768189	8406.0607	ng/ml
Hesperidin	611.1 -> 303.0	14.444	4580	559.9765	ng/ml
Hyperoside	465.1 -> 303.1	14.479	18172	923.6934	ng/ml
Rosmarinic acid	359.0 -> 160.9	14.456	23258	1858.6418	ng/ml
Apigenin 7-glucoside	433.1 -> 271.0	14.713	195029	5798.9756	ng/ml
Pinoresinol	357.0 -> 151.0	14.934	545	2122.7550	ng/ml
2-Hydroxycinnamic acid	162.9 -> 119.1	14.911	19	ND	ng/ml
Eriodictyol	287.0 -> 151.0	15.045	113	ND	ng/ml
Quercetin	301.0 -> 151.0	15.561	90	ND	ng/ml
Luteolin	287.0 -> 153.1	15.800	17077	1073.8532	ng/ml
Kaempferol	285.0 -> 229.1	16.096	10	ND	ng/ml

Compound	Transition	RT	Resp.	Final Conc	Units
Apigenin	271.0 -> 153.0	16.226	8819	471.5055	ng/ml

Gallic acid

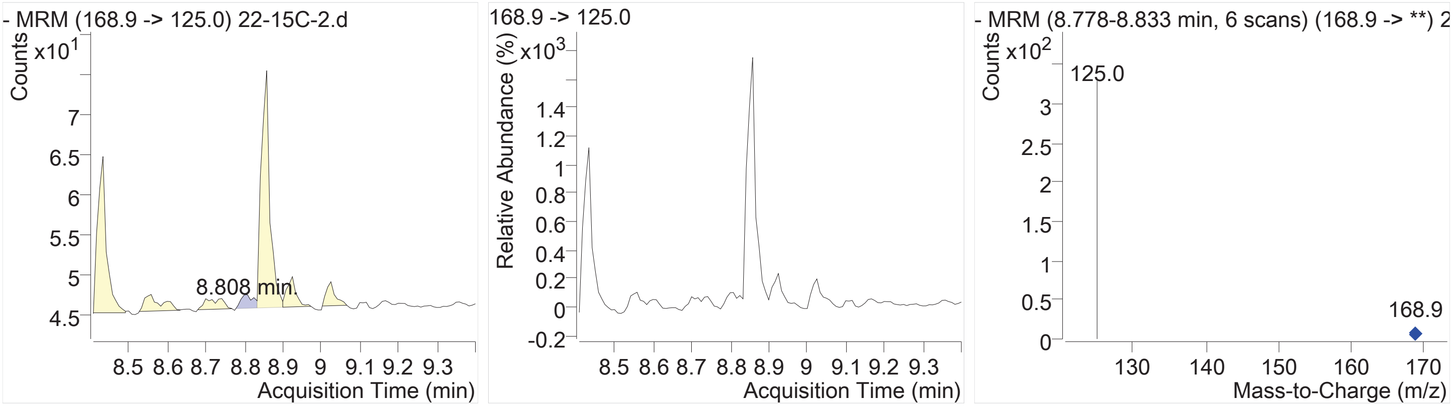

Protocatechuic acid

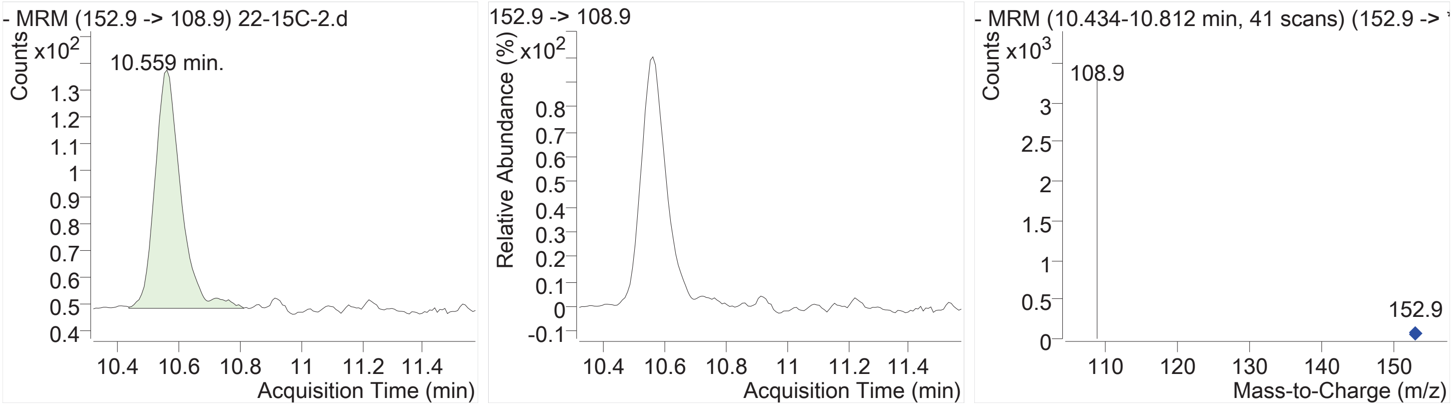

Pyrocatechol

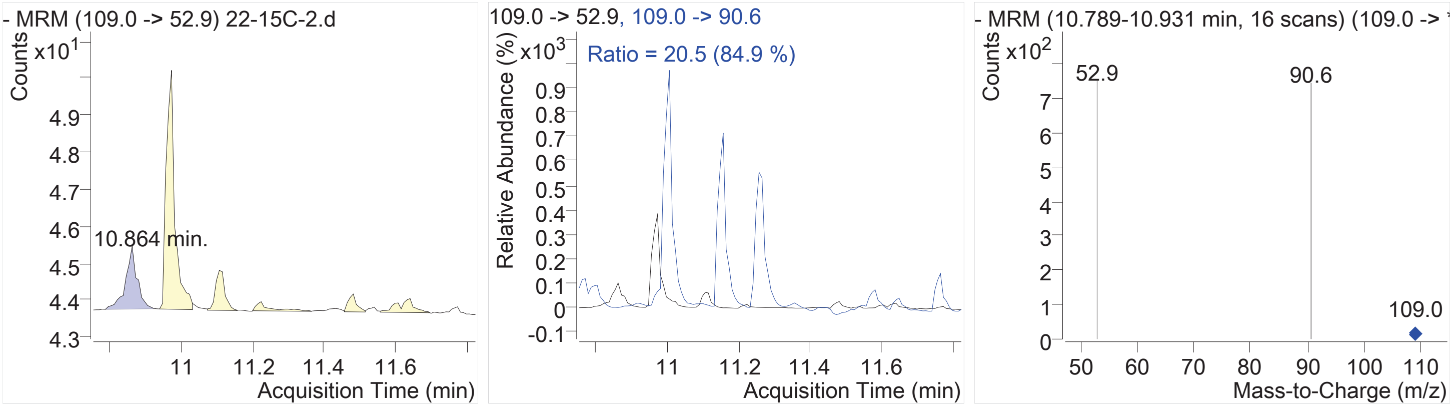

3,4-Dihydroxyphenylacetic acid

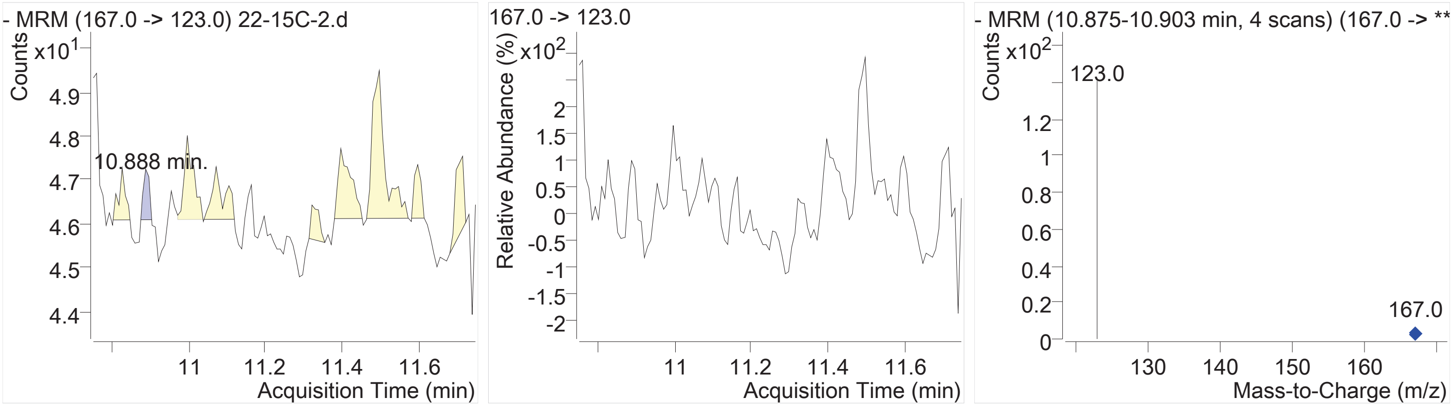

(+)-Catechin

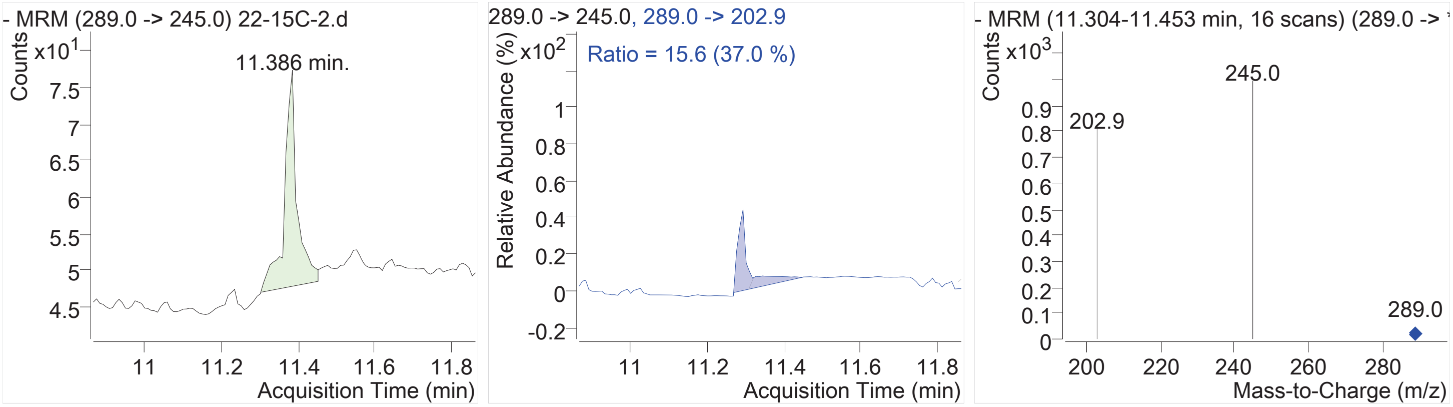

2,5-Dihydroxybenzoic acid

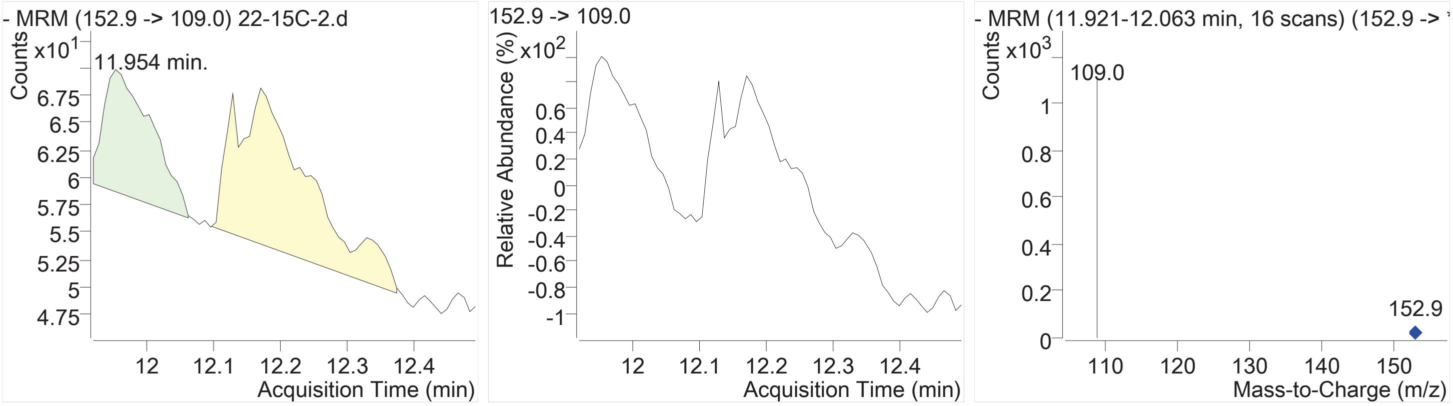

Chlorogenic acid

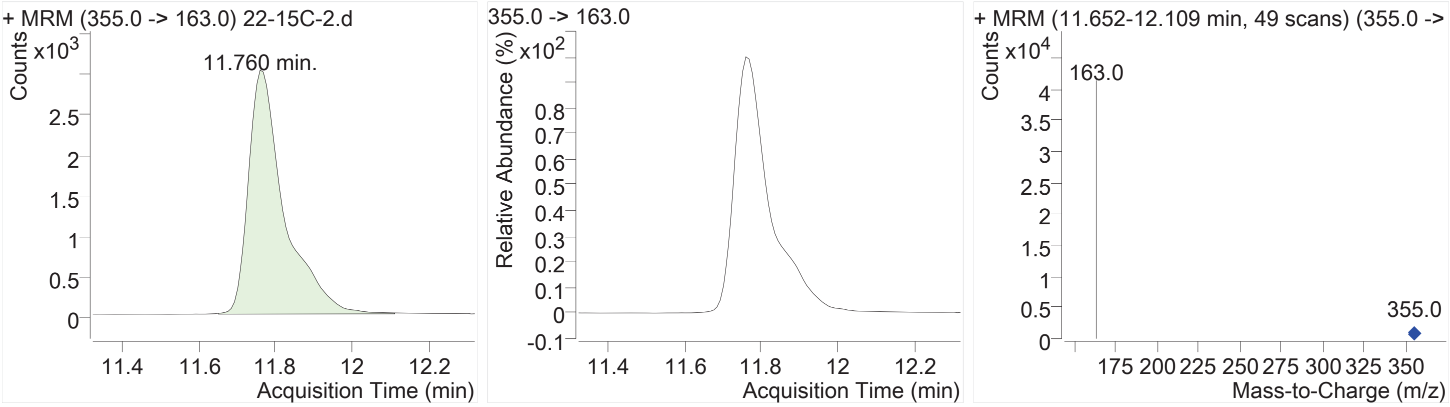

3-Hydroxybenzoic acid

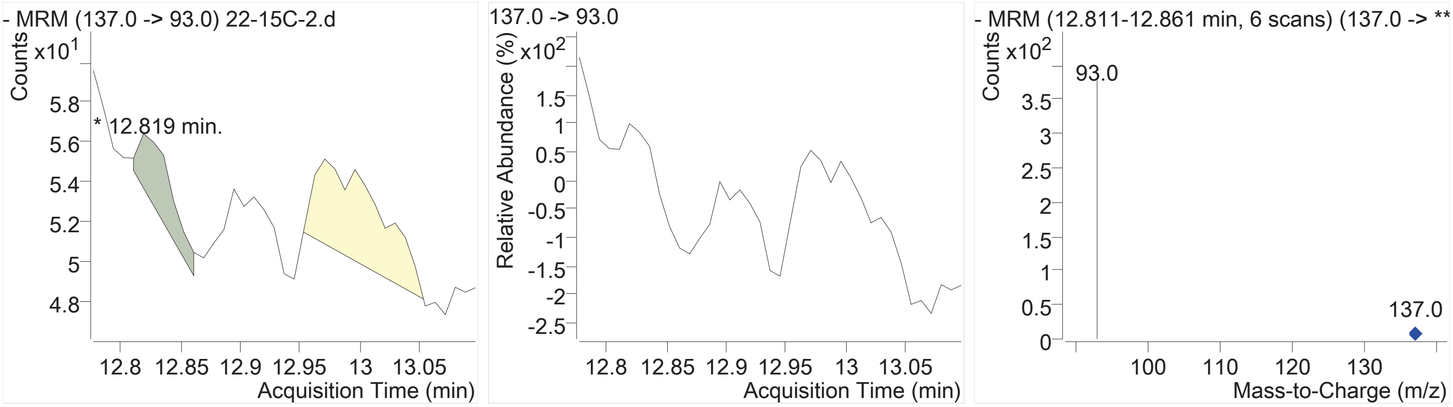

4-Hydroxybenzoic acid

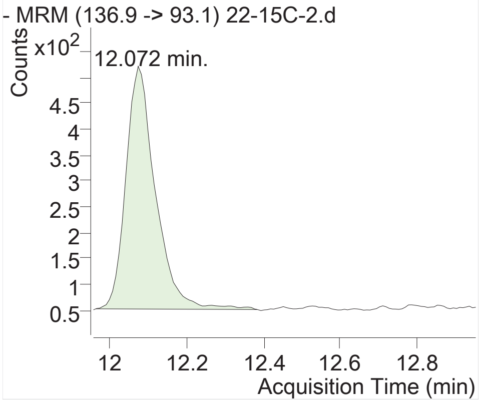

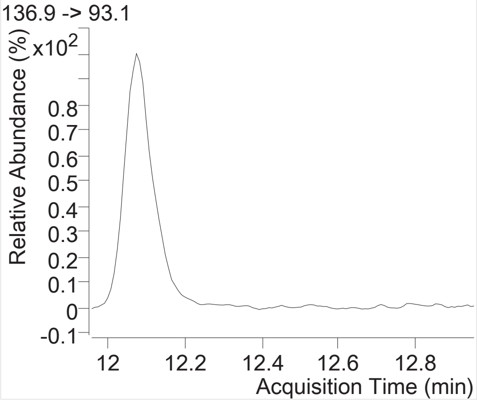

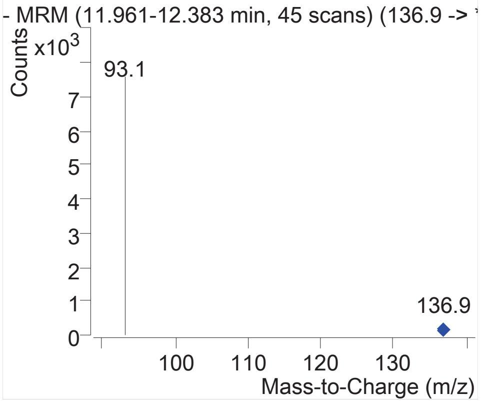

(-)-Epicatechin

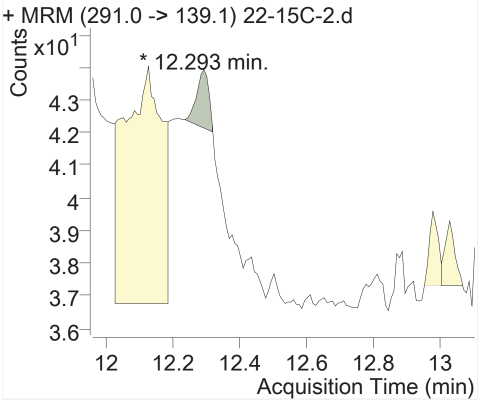

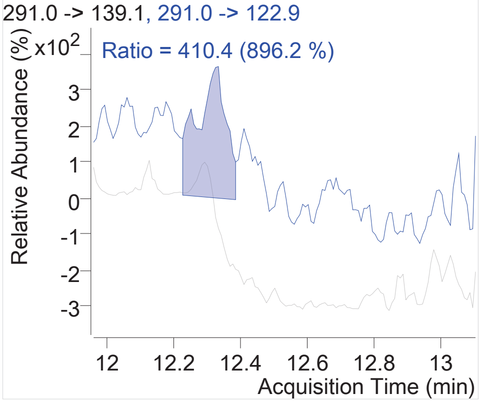

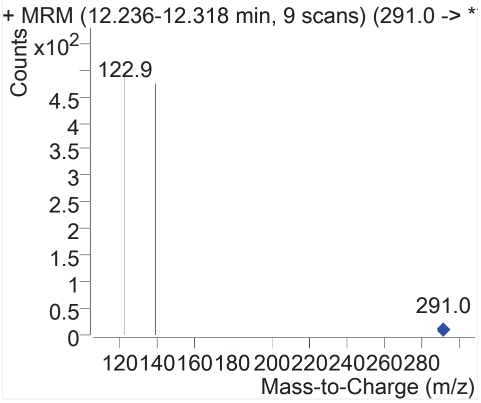

Caffeic acid

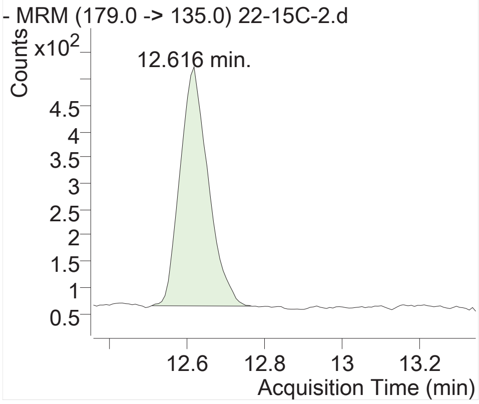

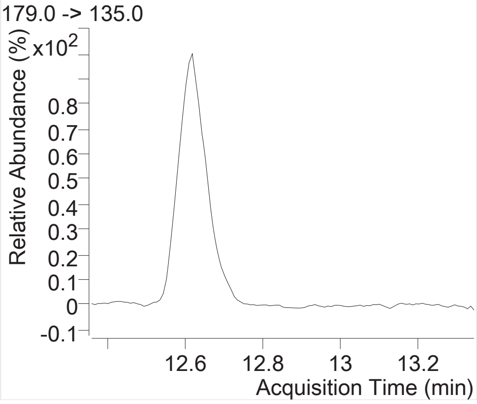

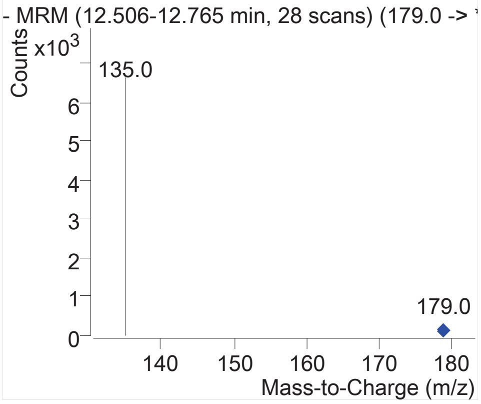

Syringic acid

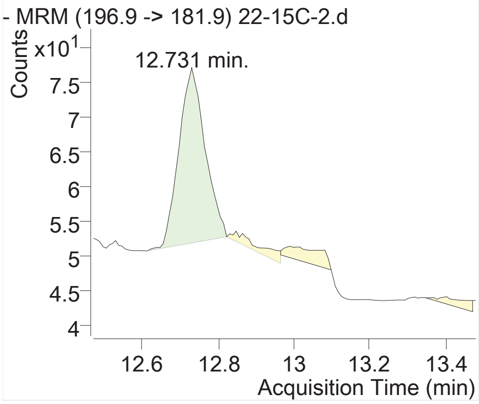

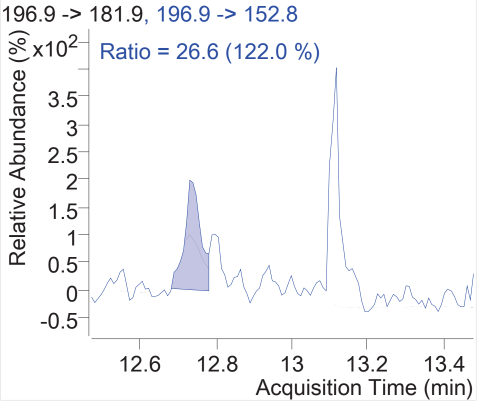

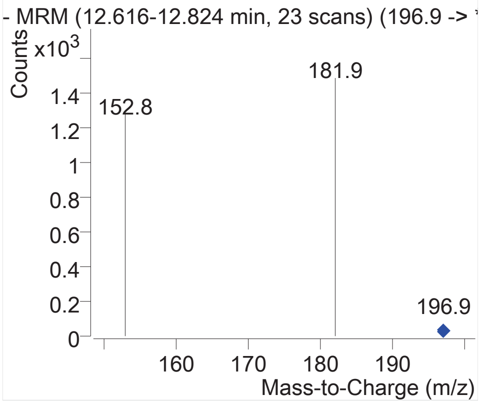

Vanillin

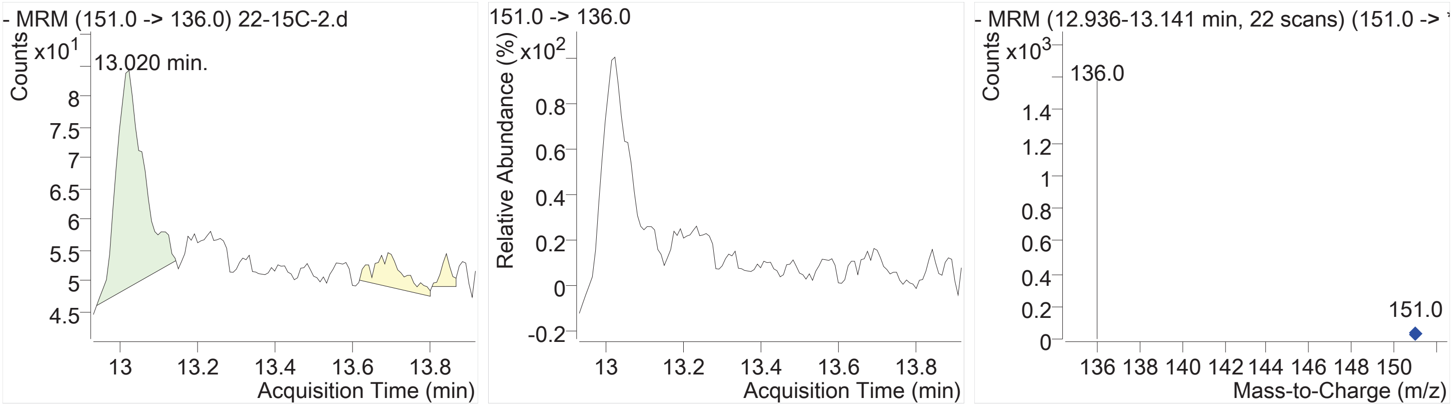

Verbascoside

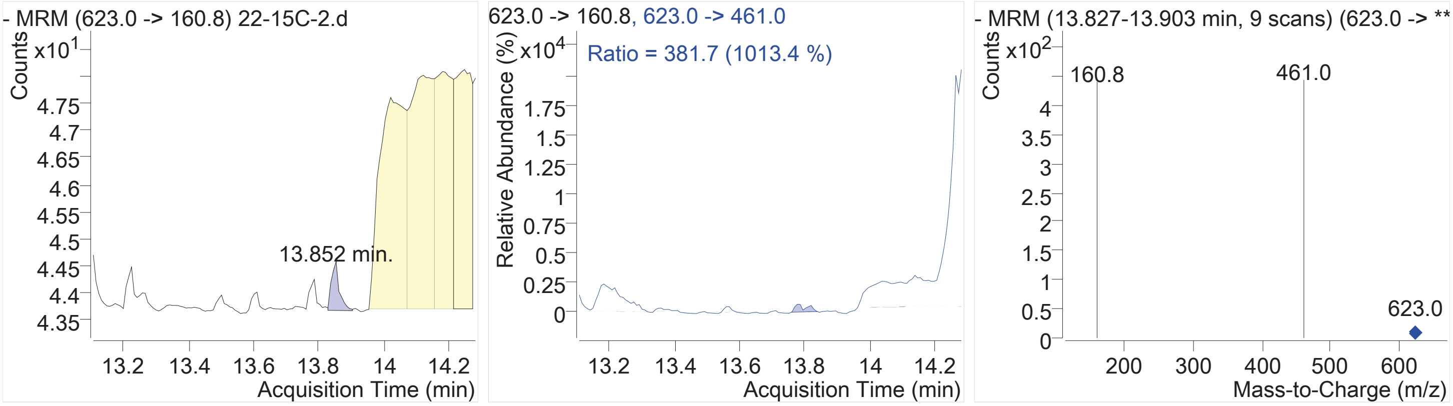

Taxifolin

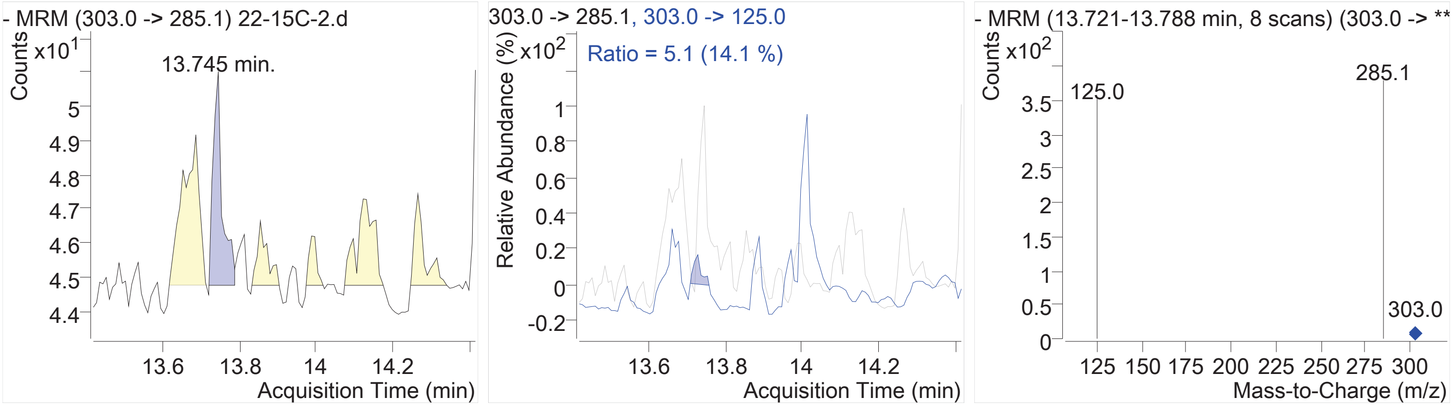

p-Coumaric acid

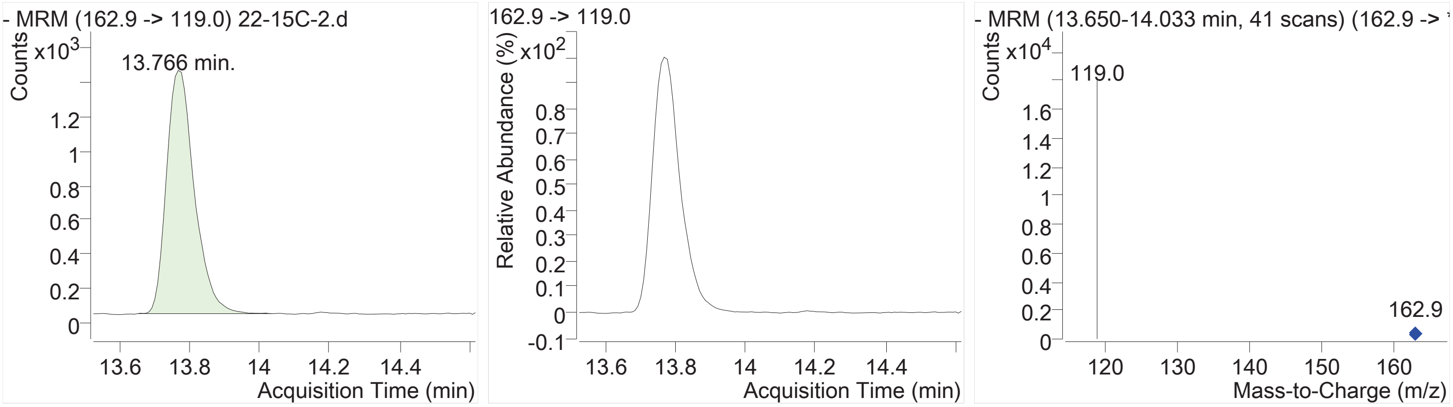

Sinapic acid

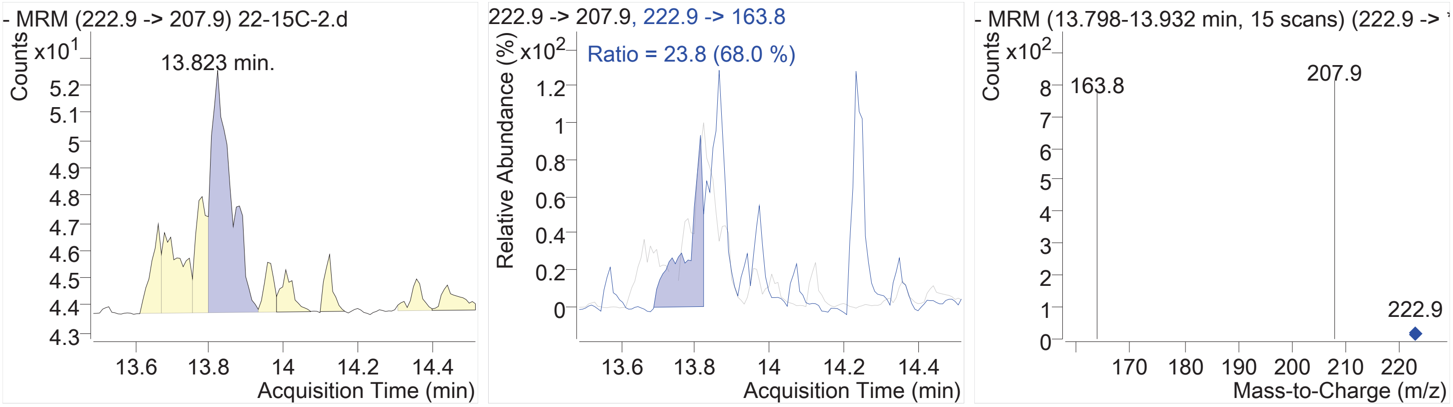

Ferulic acid

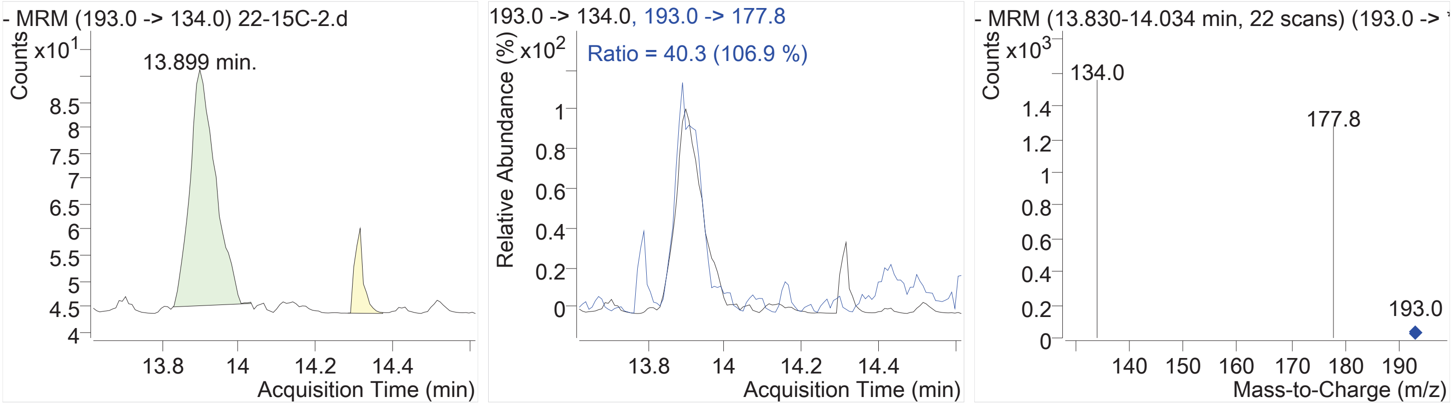

Luteolin 7-glucoside

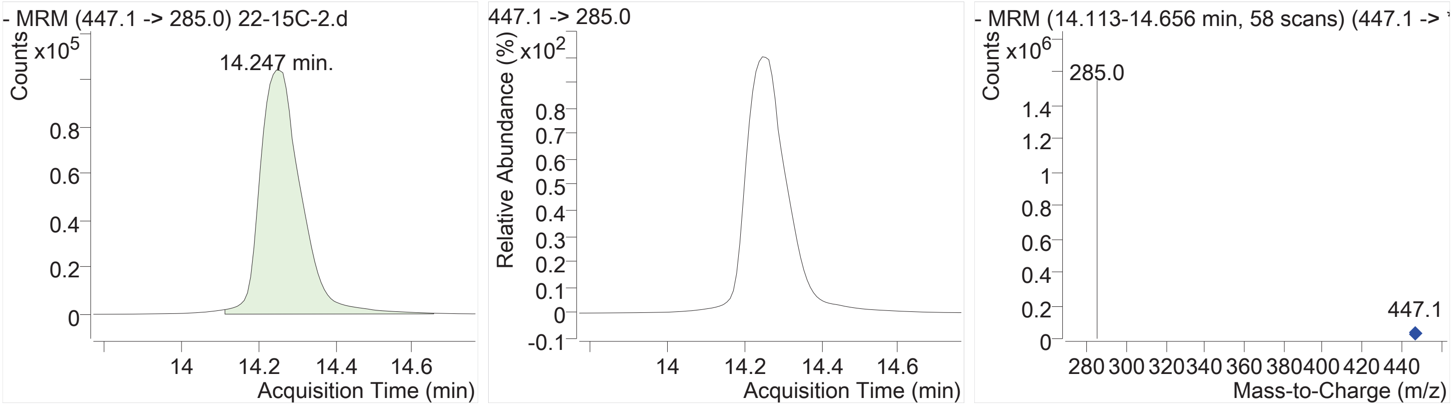

Hesperidin

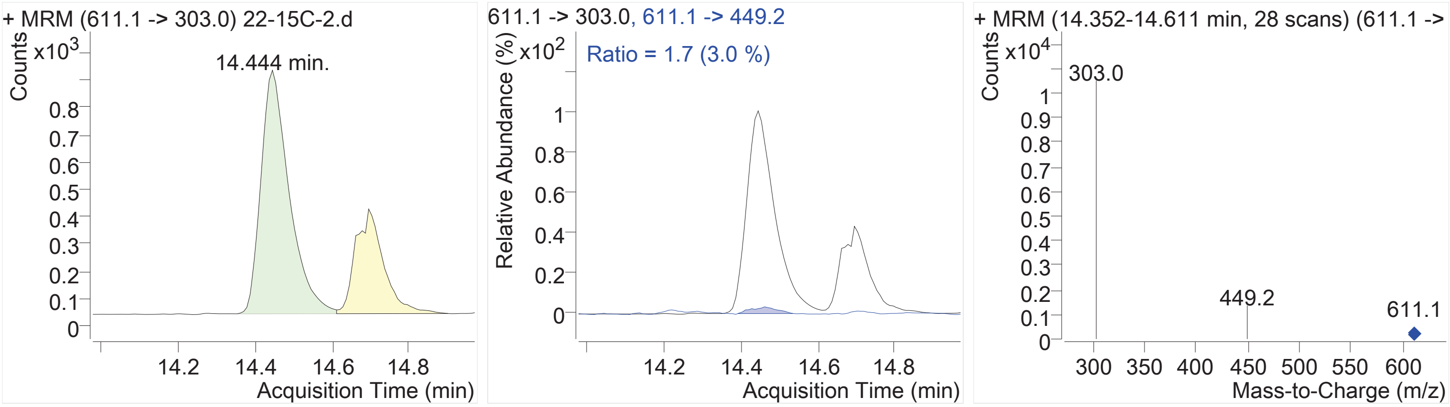

Hyperoside

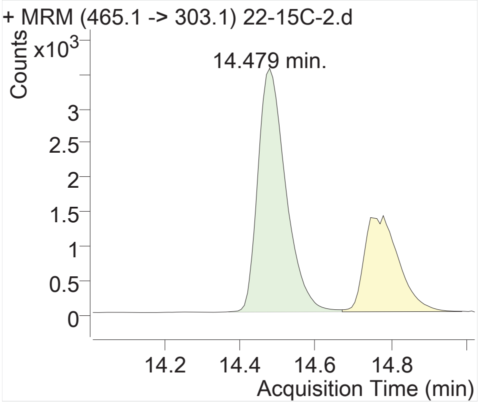

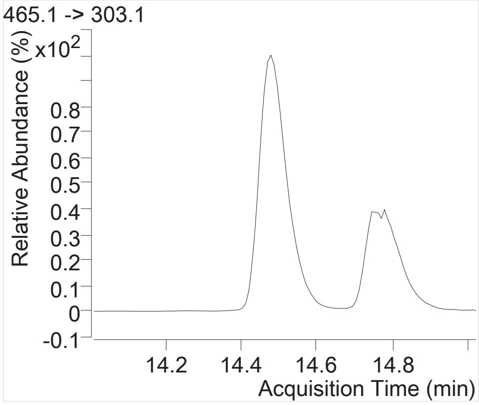

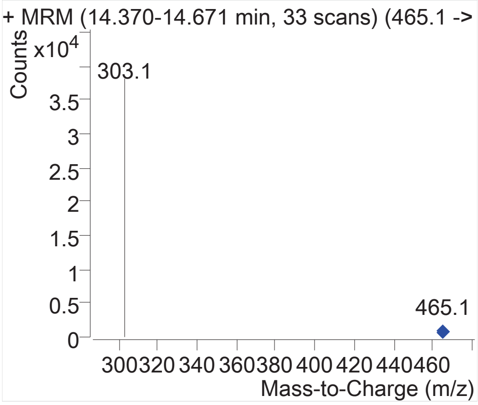

Rosmarinic acid

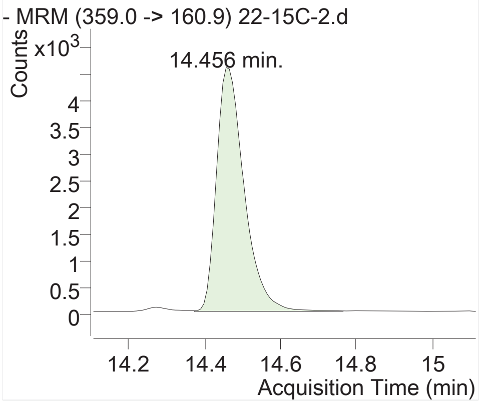

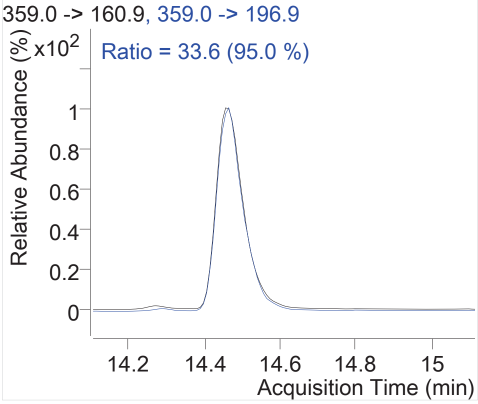

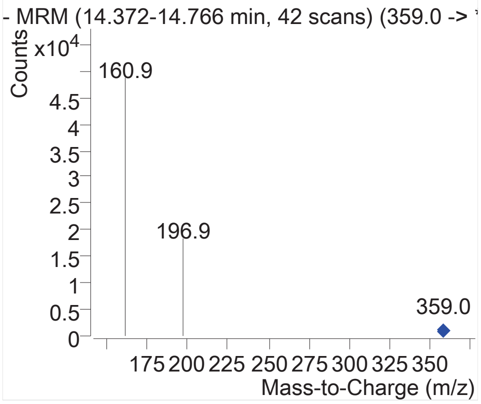

Apigenin 7-glucoside

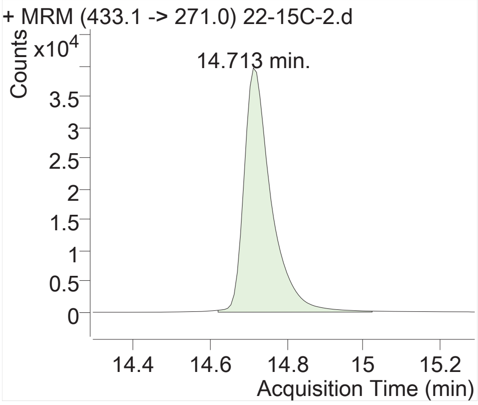

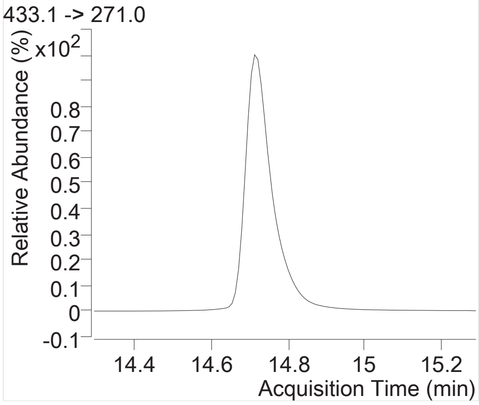

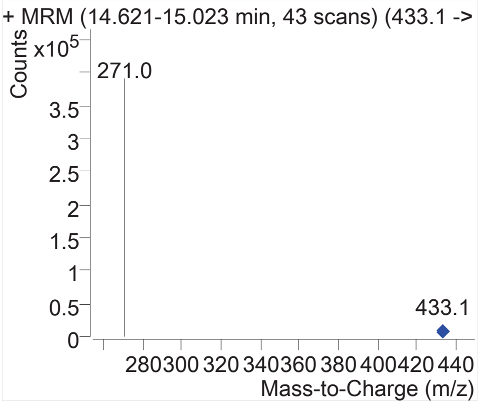

Pinoresinol

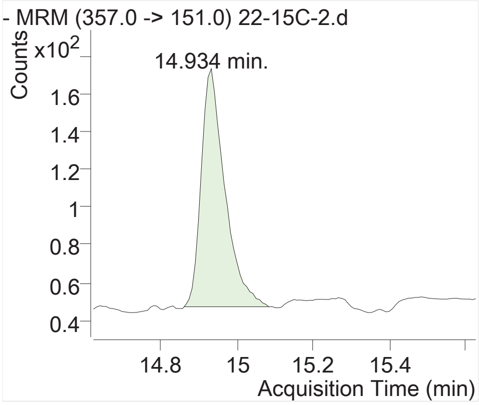

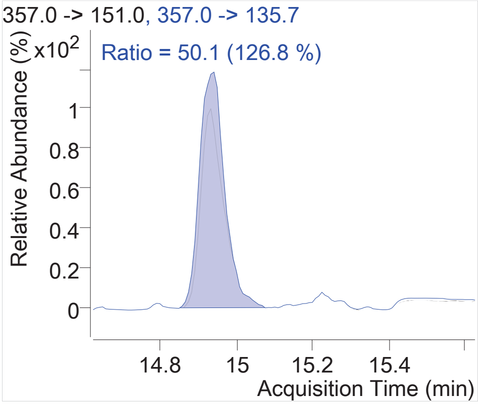

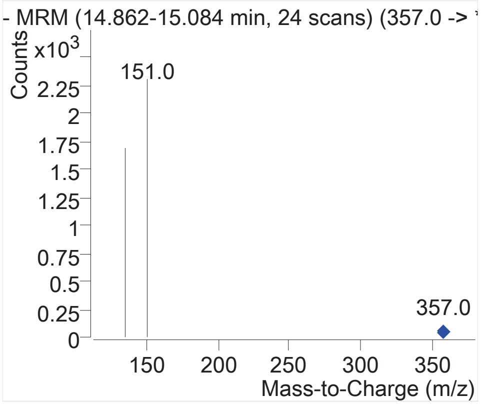

2-Hydroxycinnamic acid

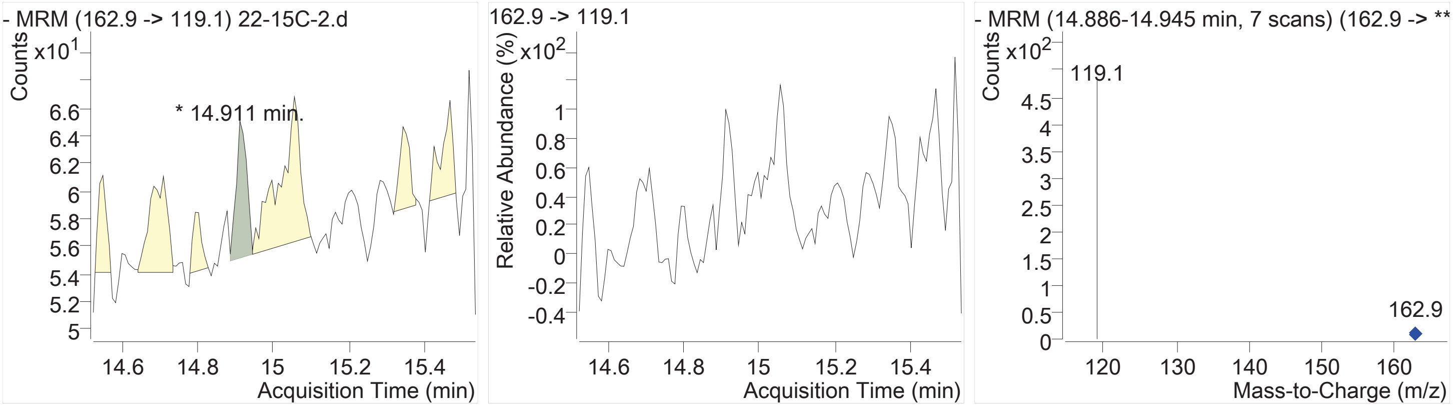

Eriodictyol

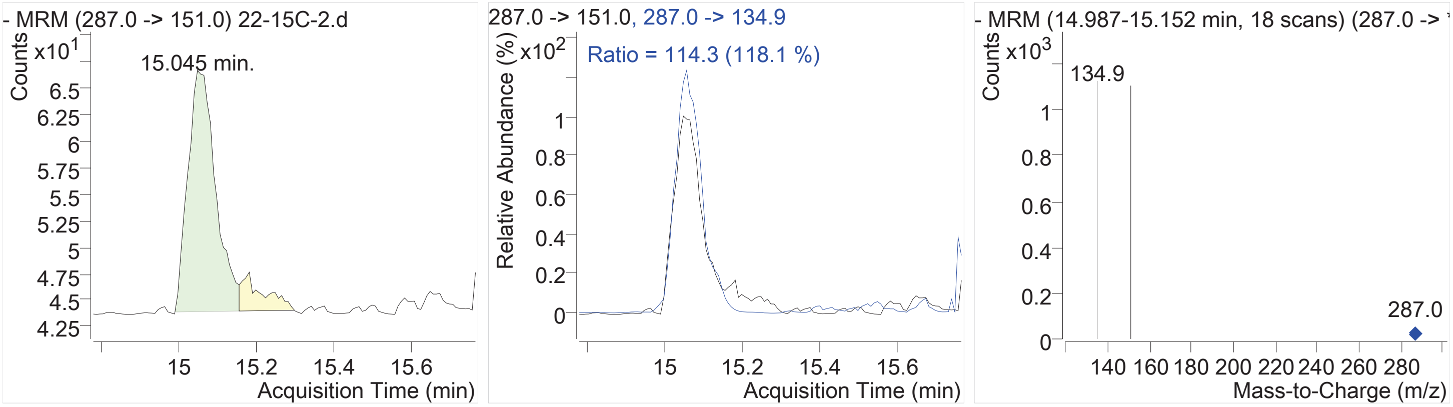

Quercetin

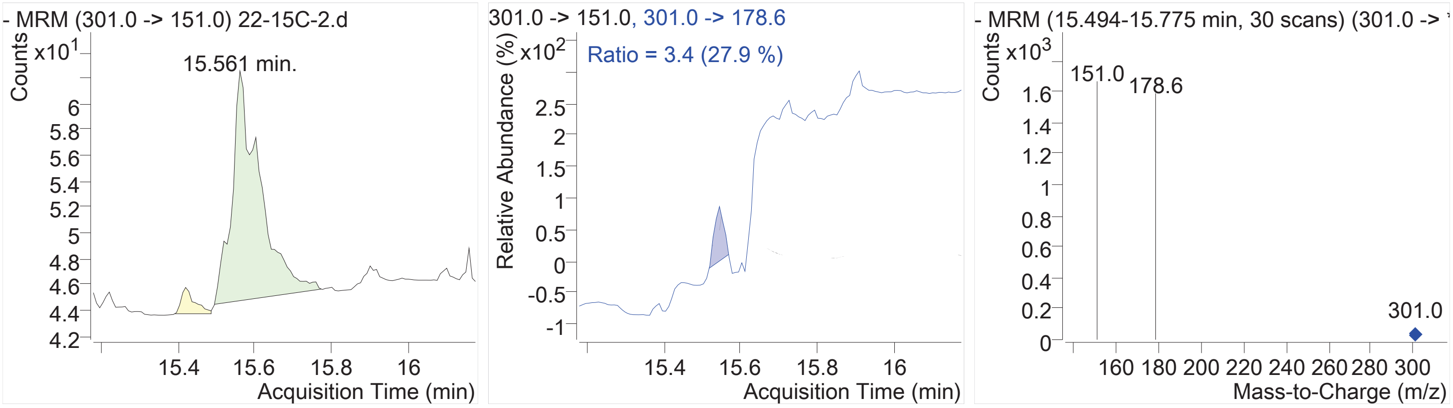

Luteolin

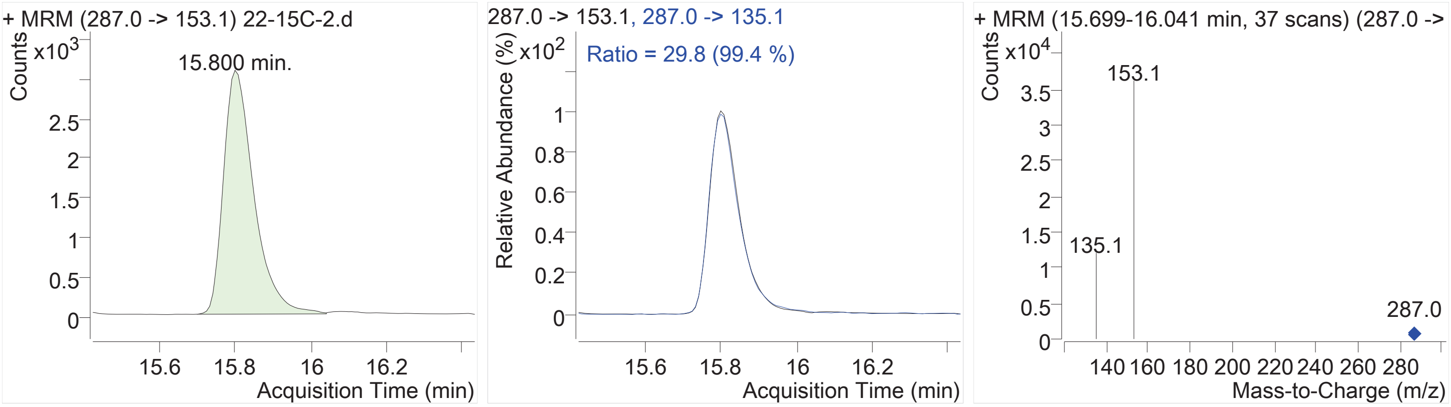

Kaempferol

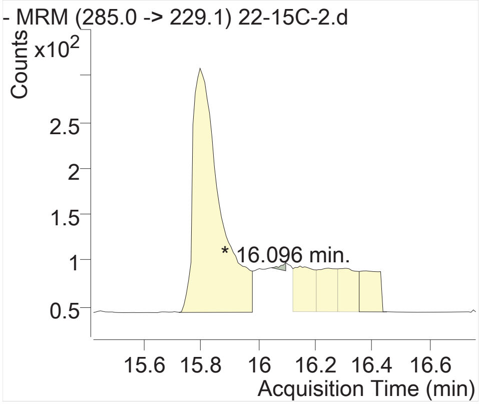

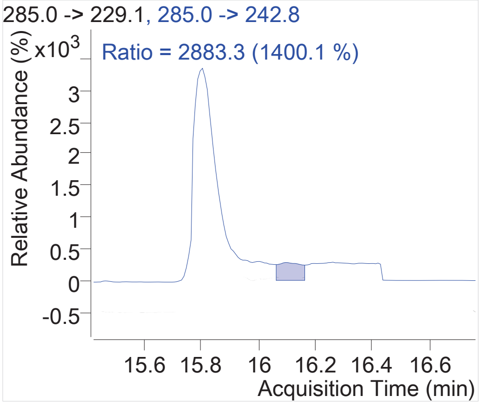

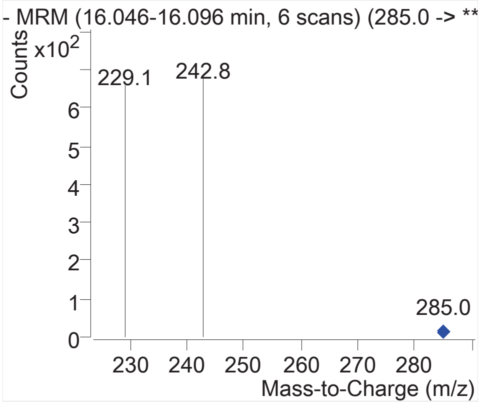

Apigenin

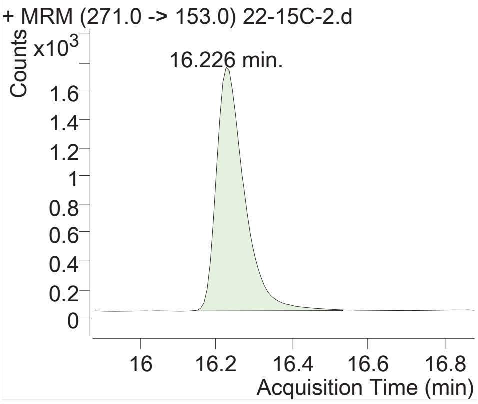

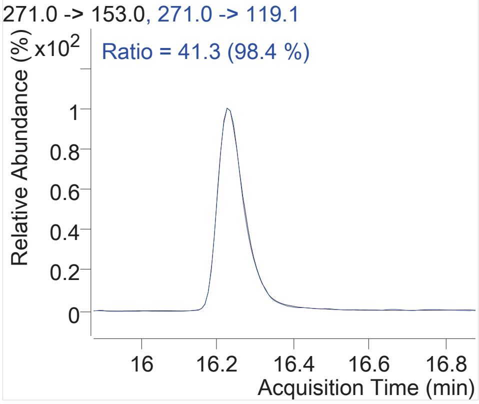

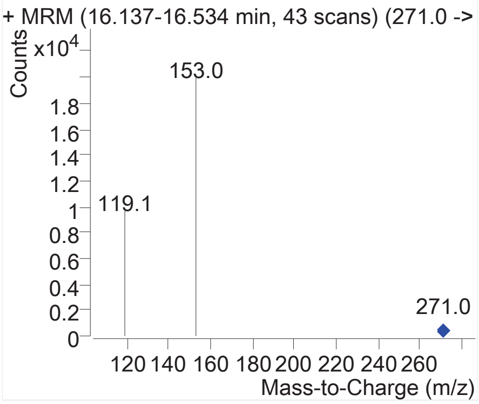

Quantitative Analysis Complete Report

**Batch Path**	D:\MassHunter\Data\2022ekim\061022cengizhoca\QuantResults\071022.batch.bin		
**Analysis Time**	10/11/2022 1:33:26 PM	**Analyst Name**	Defam-PC\admin
**Report Time**	10/11/2022 1:42:17 PM	**Reporter Name**	admin
**Last Calib Update**	10/11/2022 1:33:17 PM	**Batch State**	Processed
**Quant Batch Version**	B.07.01	**Quant Report Version**	B.07.01

**Acq. Time**	10/9/2022 5:22:40 AM	**Data File**	22-15C-3.d
**Sample Type**	Sample	**Sample Name**	22-15C-3
**Dilution**	1	**Acq. Method**	FENOLIK_DMRM2021-31bilesen.m

Sample Chromatogram

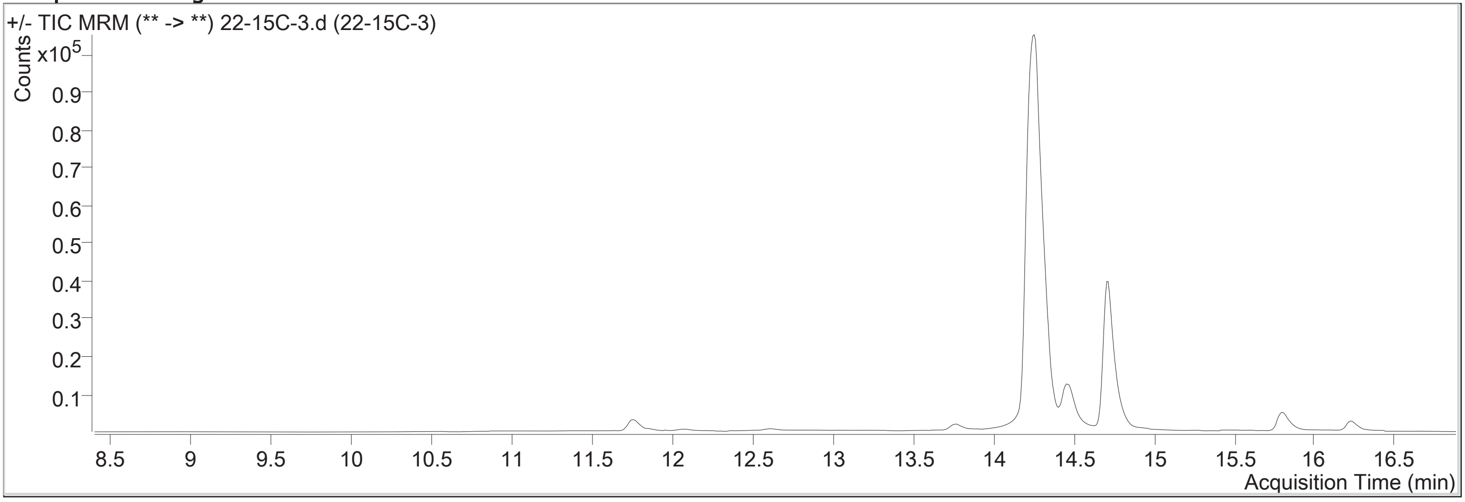

Compound	Transition	RT	Resp.	Final Conc	Units
Gallic acid	168.9 -> 125.0	8.766	5	ND	ng/ml
Protocatechuic acid	152.9 -> 108.9	10.559	512	37.7295	ng/ml
Pyrocatechol	109.0 -> 52.9	10.797	0	ND	ng/ml
3,4-Dihydroxyphenylacetic acid	167.0 -> 123.0	10.810	2	ND	ng/ml
(+)-Catechin	289.0 -> 245.0	11.069	11	ND	ng/ml
2,5-Dihydroxybenzoic acid	152.9 -> 109.0	11.963	55	9.2542	ng/ml
Chlorogenic acid	355.0 -> 163.0	11.768	19031	899.0568	ng/ml
3-Hydroxybenzoic acid	137.0 -> 93.0	12.836	10	16.8853	ng/ml
4-Hydroxybenzoic acid	136.9 -> 93.1	12.072	2332	200.8389	ng/ml
(-)-Epicatechin	291.0 -> 139.1	12.302	7	3.9700	ng/ml
Caffeic acid	179.0 -> 135.0	12.616	2291	69.9383	ng/ml
Syringic acid	196.9 -> 181.9	12.723	118	168.1878	ng/ml
Vanillin	151.0 -> 136.0	13.045	169	29.7363	ng/ml
Verbascoside	623.0 -> 160.8	14.229	74	ND	ng/ml
Taxifolin	303.0 -> 285.1	13.670	8	ND	ng/ml
p-Coumaric acid	162.9 -> 119.0	13.766	8338	316.1573	ng/ml
Sinapic acid	222.9 -> 207.9	13.831	38	16.4048	ng/ml
Ferulic acid	193.0 -> 134.0	13.908	245	47.2855	ng/ml
Luteolin 7-glucoside	447.1 -> 285.0	14.255	763750	8357.2973	ng/ml
Hesperidin	611.1 -> 303.0	14.444	4375	535.1084	ng/ml
Hyperoside	465.1 -> 303.1	14.479	18217	925.9603	ng/ml
Rosmarinic acid	359.0 -> 160.9	14.464	21826	1744.0792	ng/ml
Apigenin 7-glucoside	433.1 -> 271.0	14.713	188747	5612.1636	ng/ml
Pinoresinol	357.0 -> 151.0	14.934	540	2104.9272	ng/ml
2-Hydroxycinnamic acid	162.9 -> 119.1	14.945	22	ND	ng/ml
Eriodictyol	287.0 -> 151.0	15.053	131	ND	ng/ml
Quercetin	301.0 -> 151.0	15.578	53	ND	ng/ml
Luteolin	287.0 -> 153.1	15.800	17300	1088.0007	ng/ml
Kaempferol	285.0 -> 229.1	16.088	28	ND	ng/ml

Quantitative Analysis Complete Report

Compound	Transition	RT	Resp.	Final Conc	Units
Apigenin	271.0 -> 153.0	16.235	8922	477.1049	ng/ml

Gallic acid

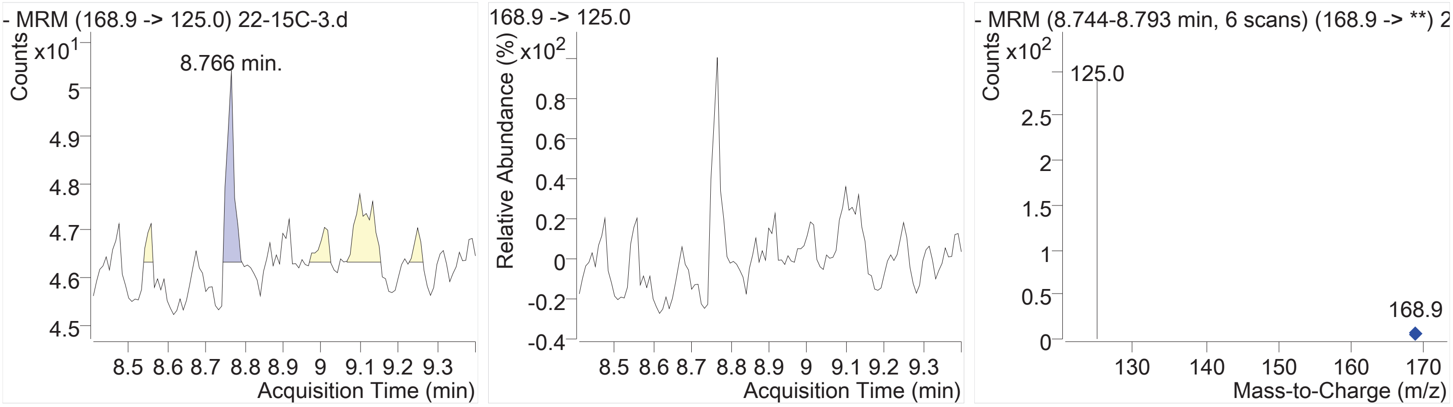

Protocatechuic acid

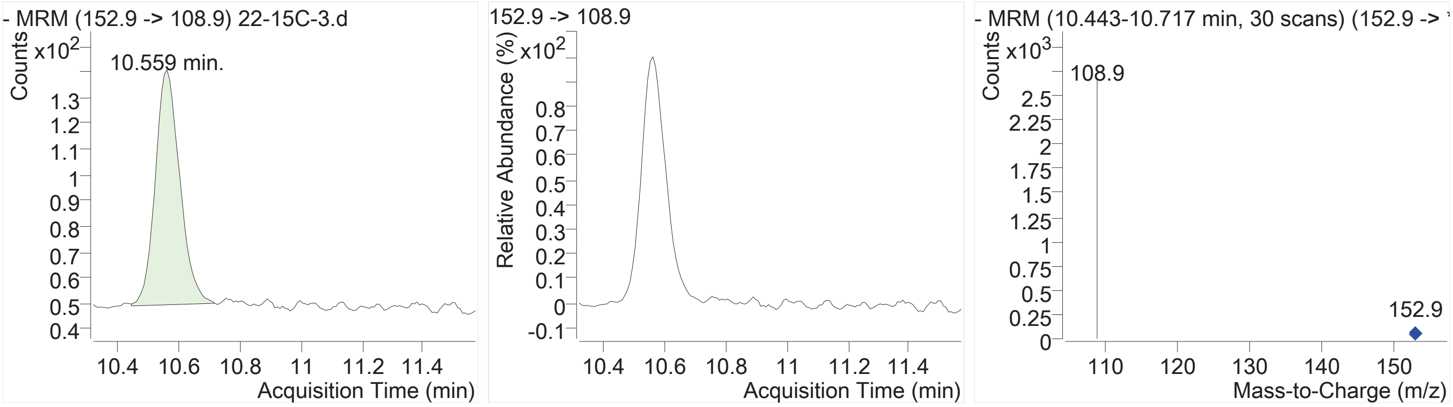

Pyrocatechol

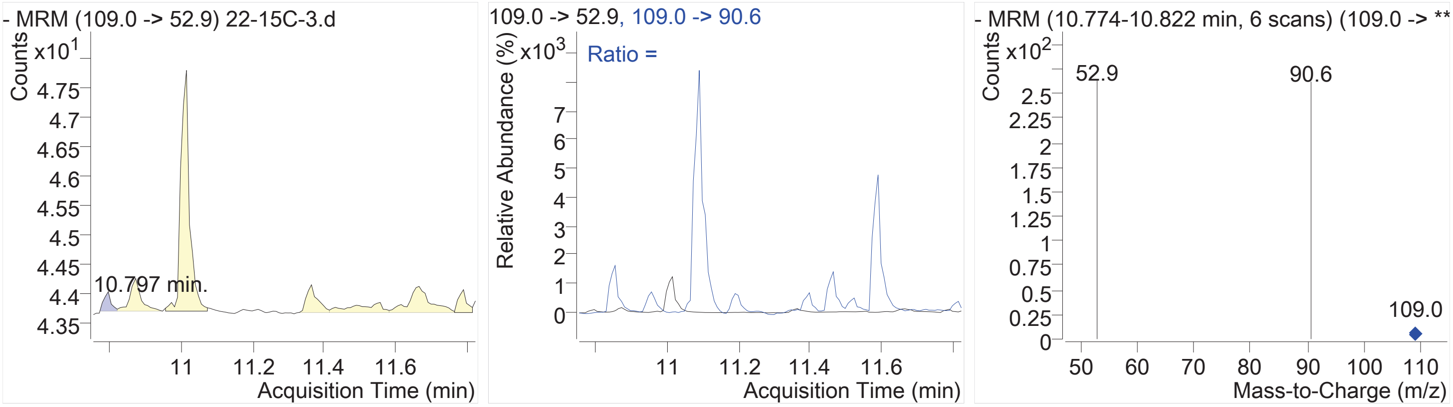

3,4-Dihydroxyphenylacetic acid

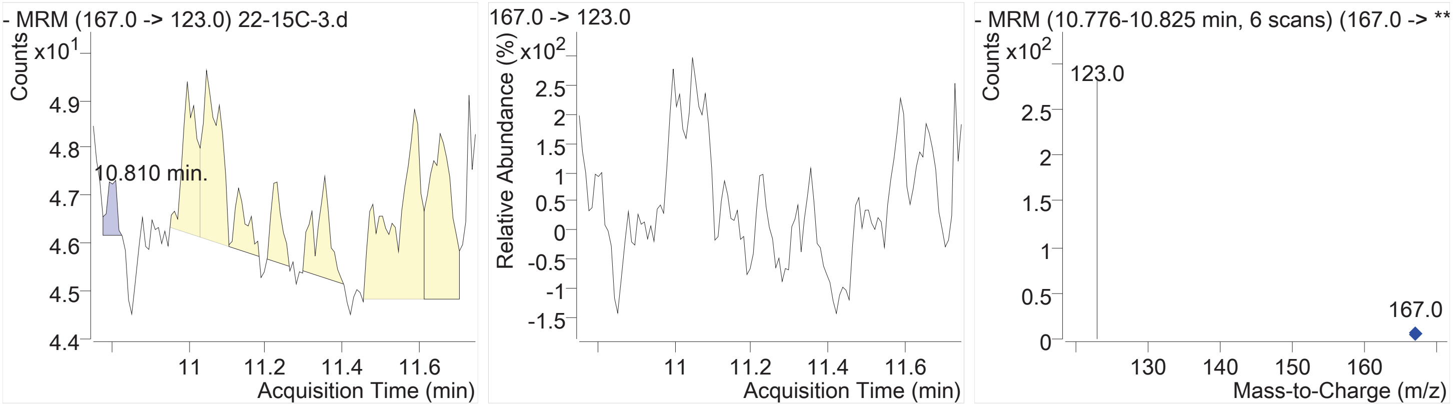

(+)-Catechin

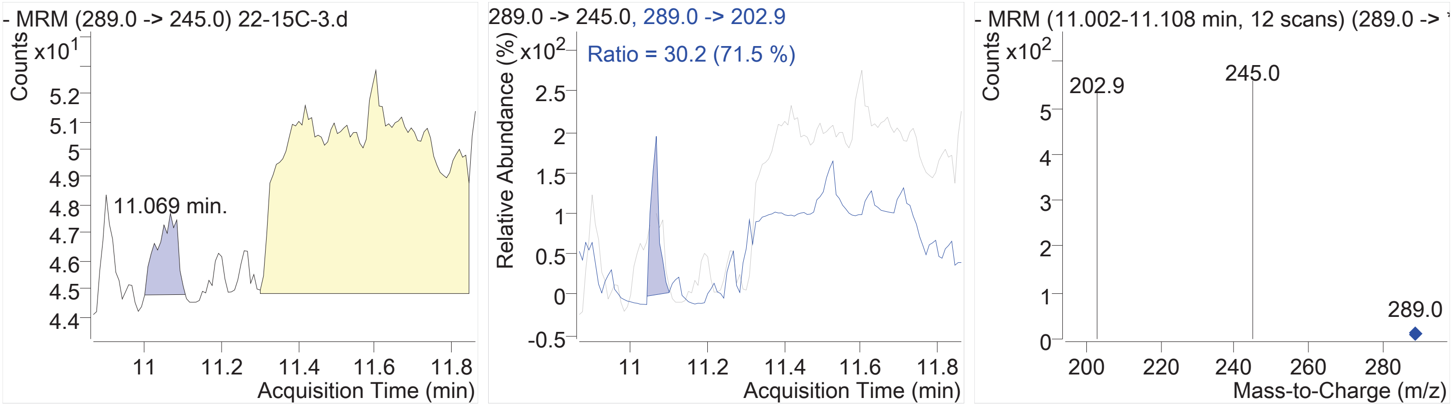

2,5-Dihydroxybenzoic acid

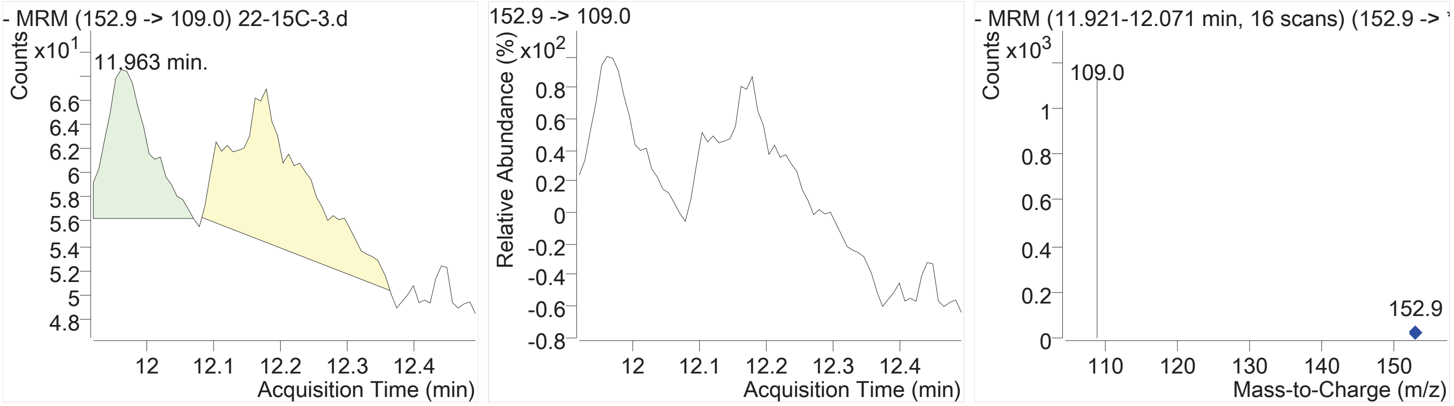

Chlorogenic acid

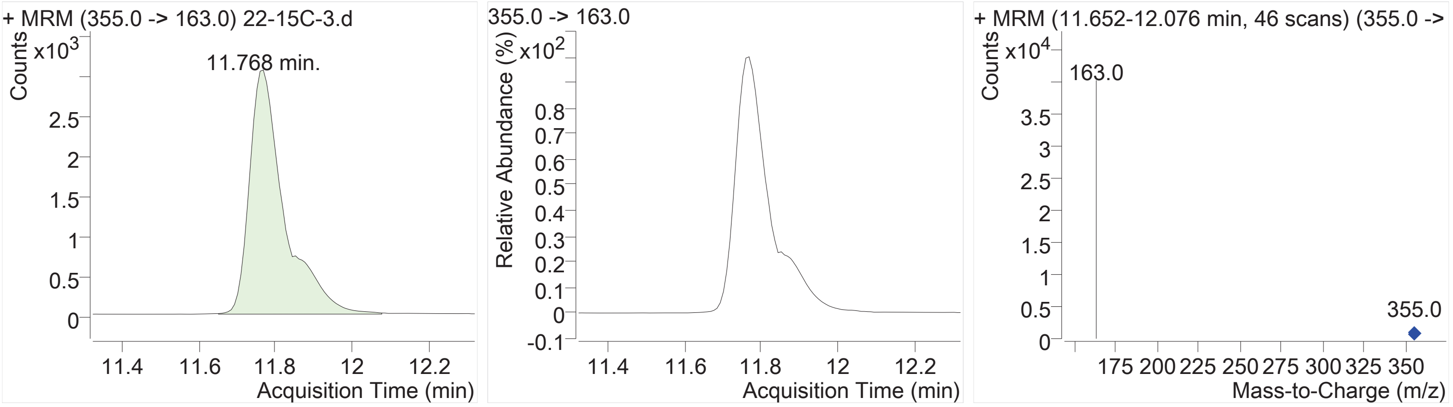

3-Hydroxybenzoic acid

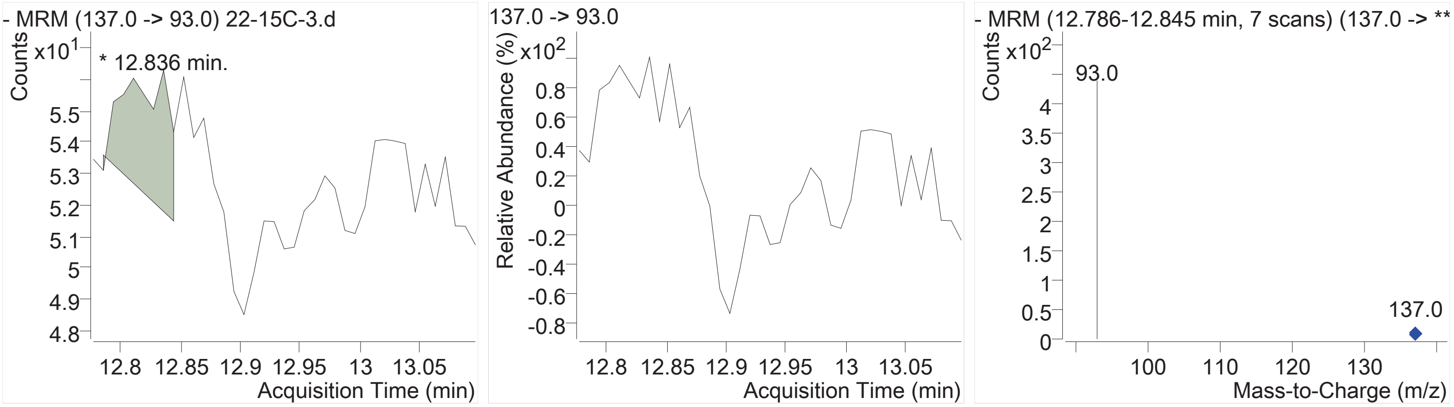

4-Hydroxybenzoic acid

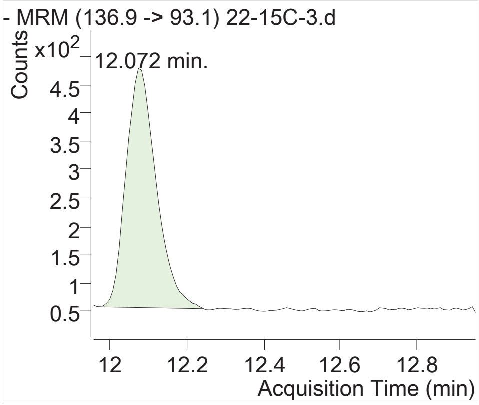

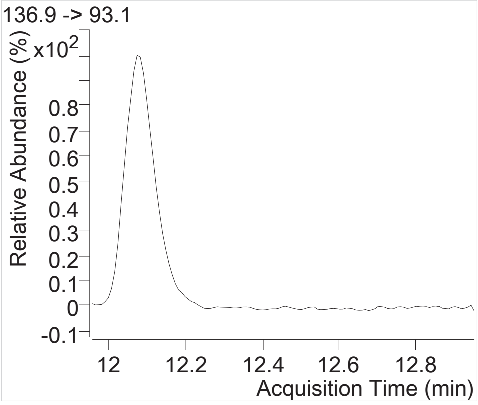

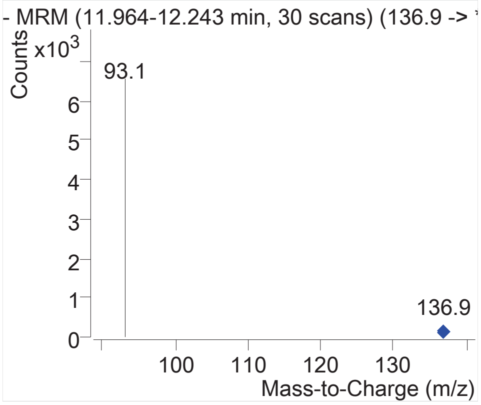

(-)-Epicatechin

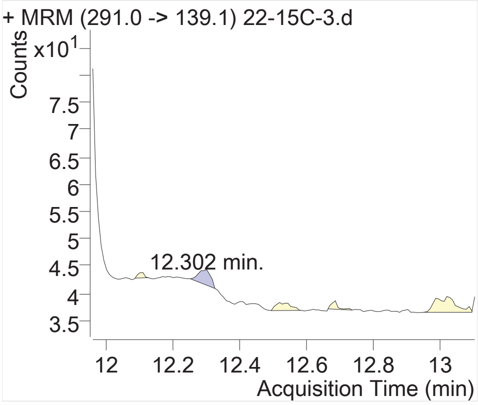

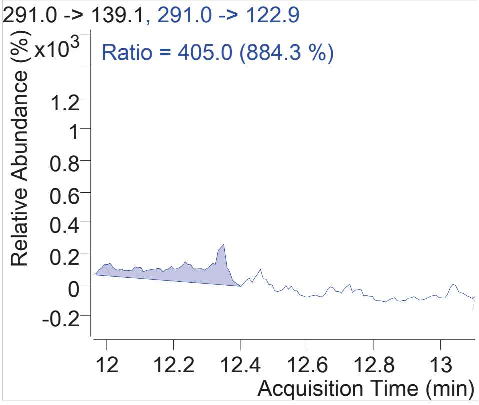

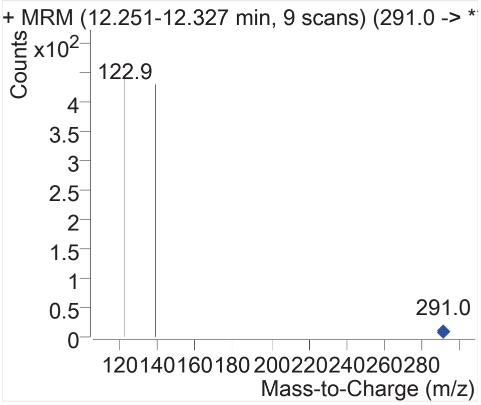

Caffeic acid

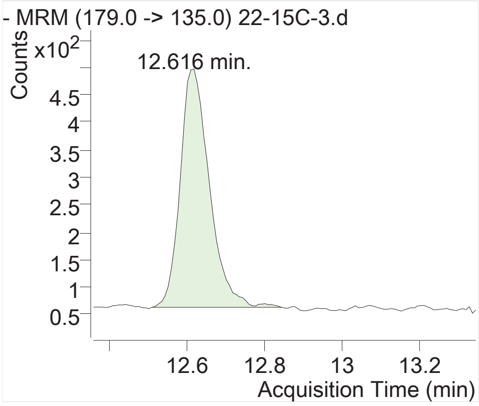

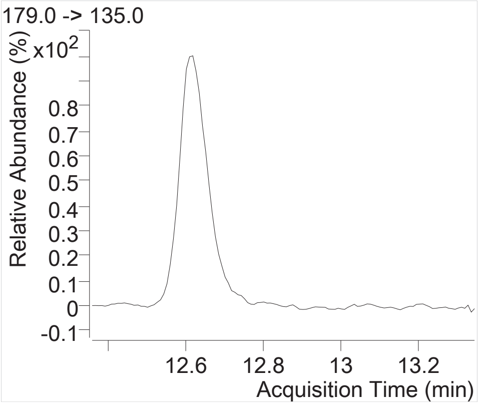

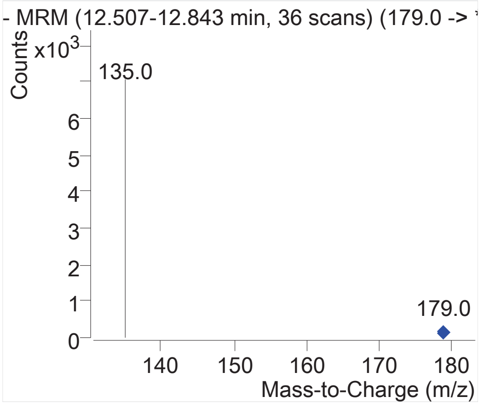

Syringic acid

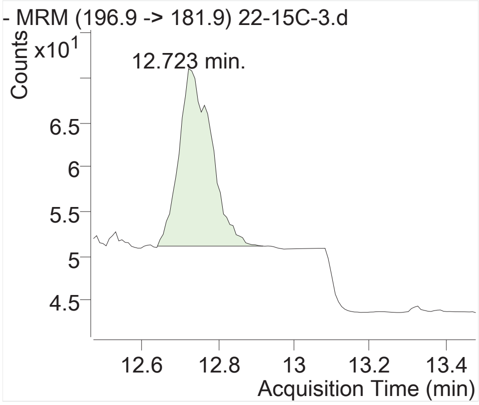

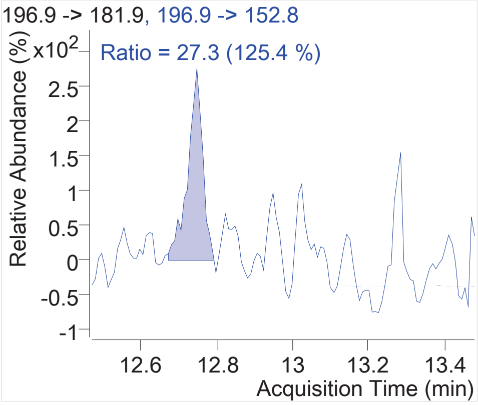

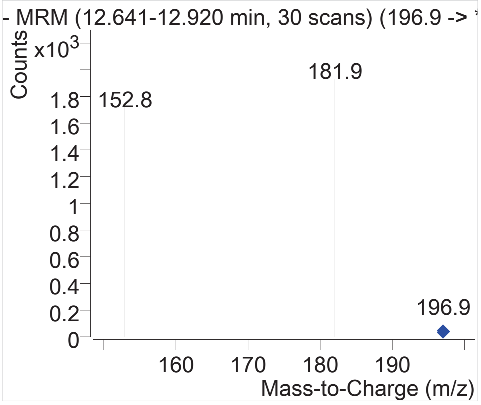

Vanillin

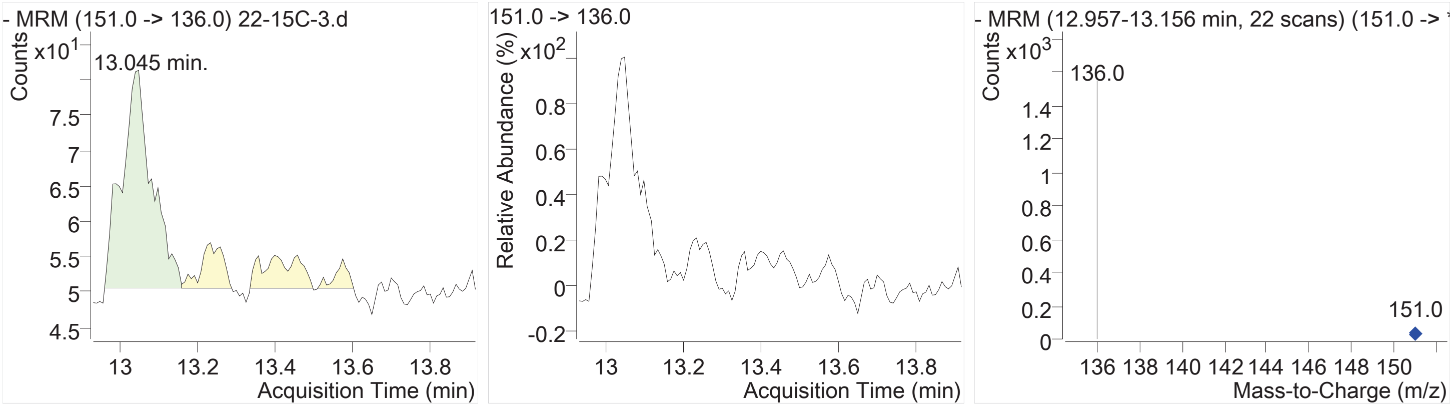

Verbascoside

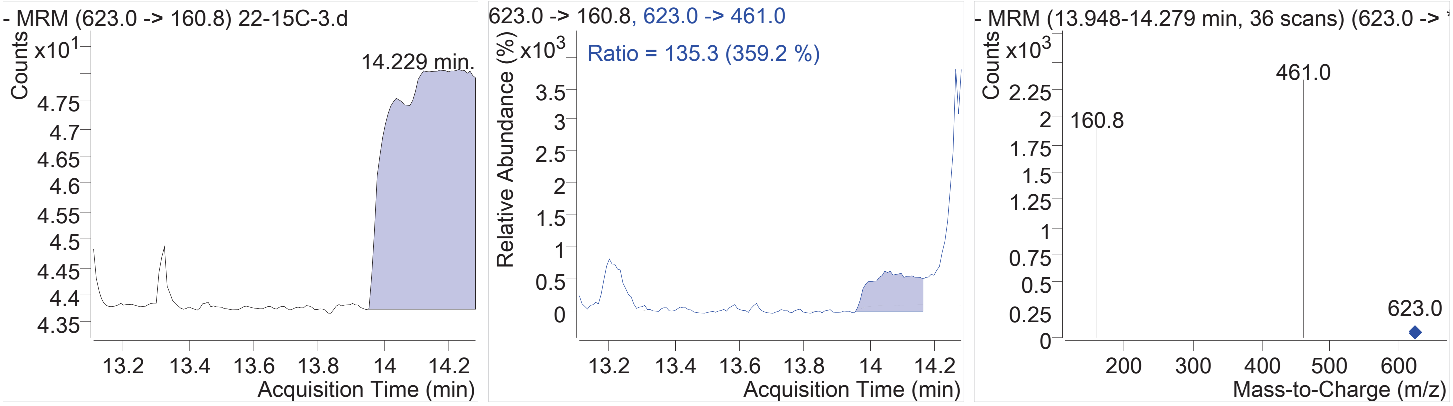

Taxifolin

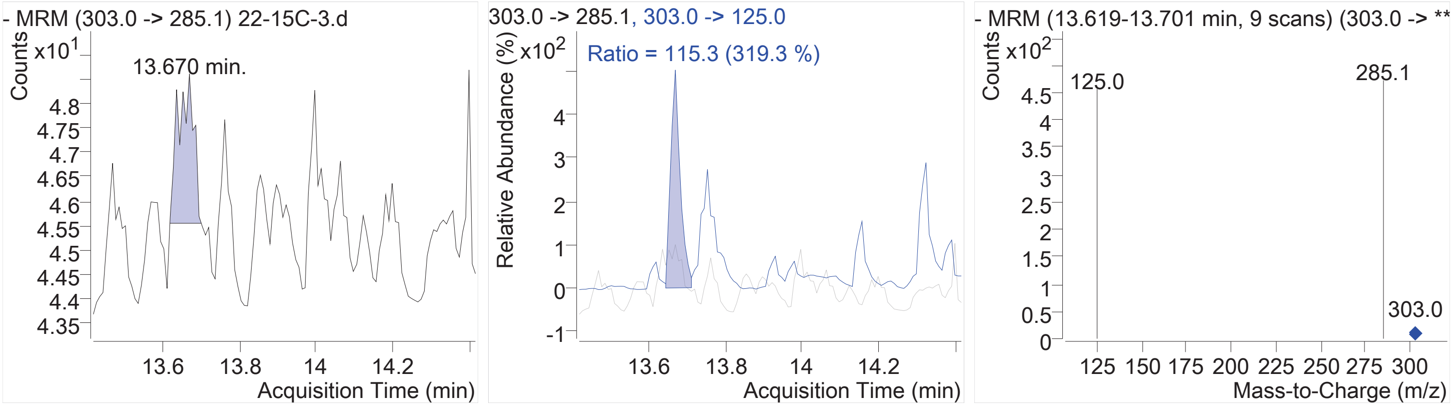

p-Coumaric acid

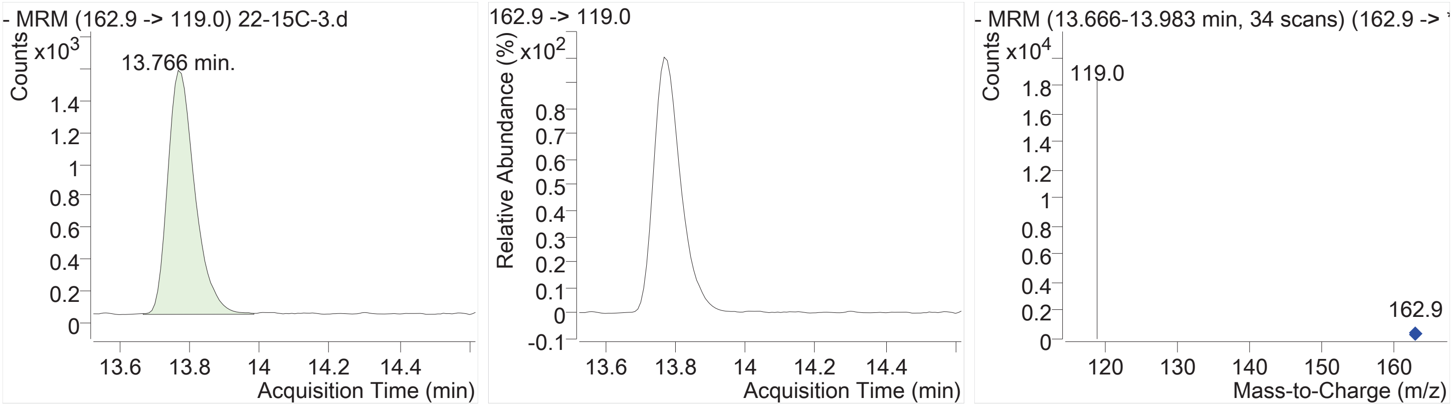

Sinapic acid

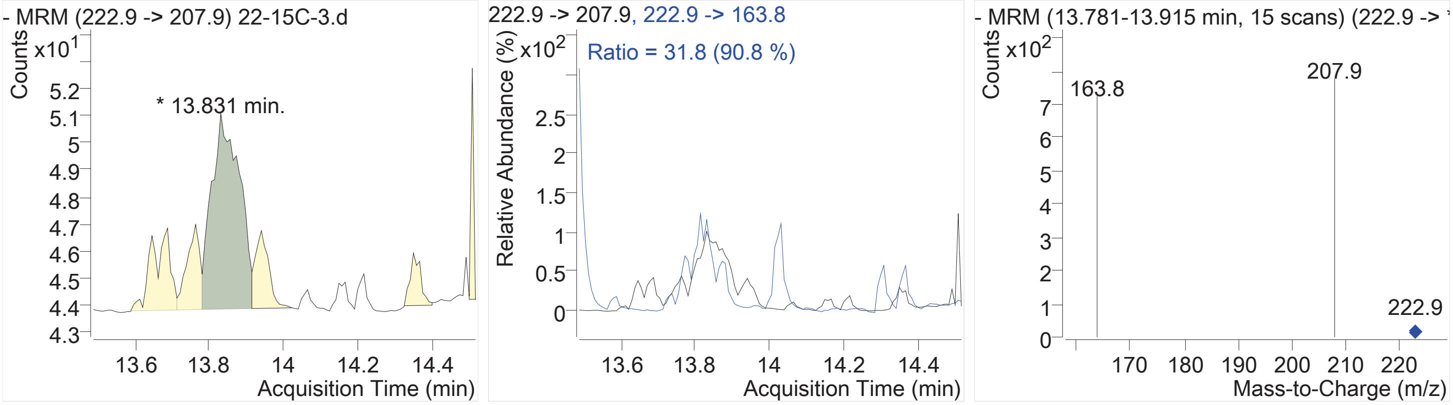

Ferulic acid

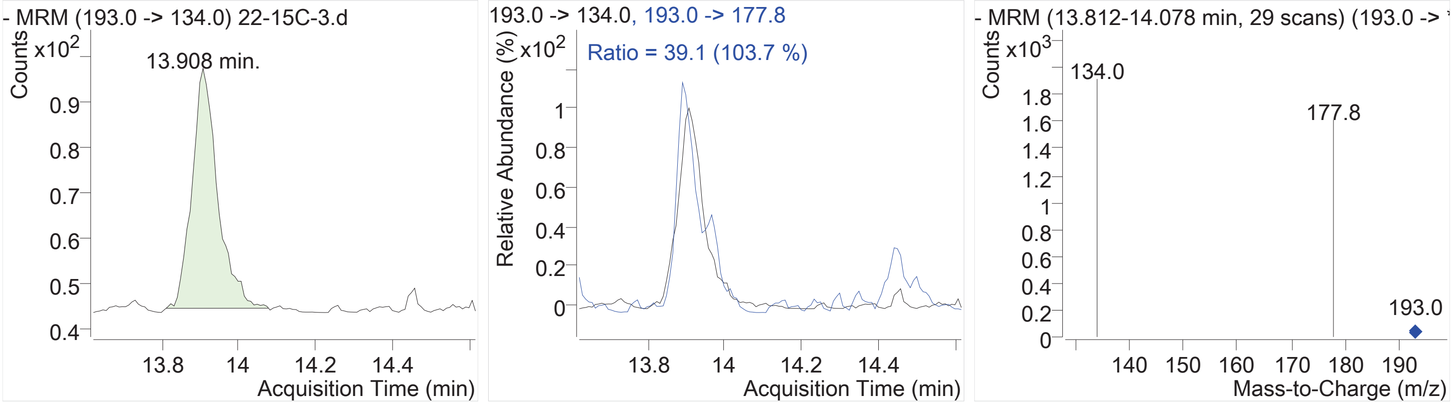

Luteolin 7-glucoside

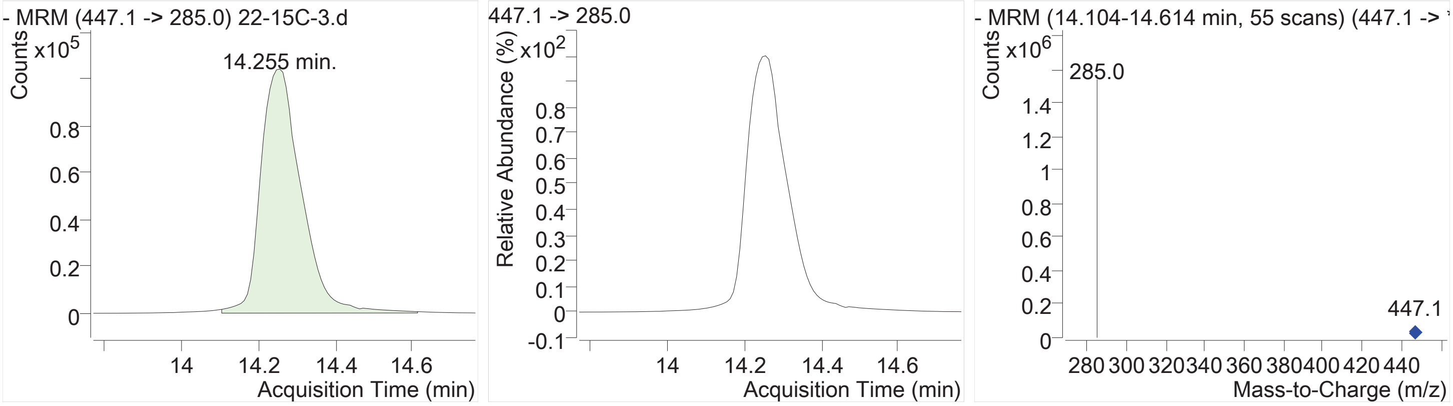

Hesperidin

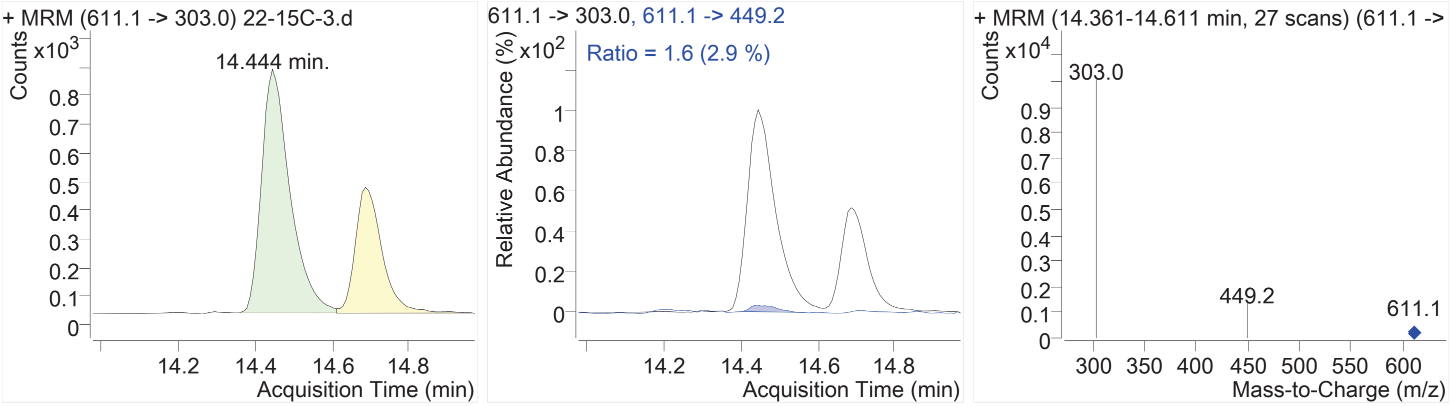

Hyperoside

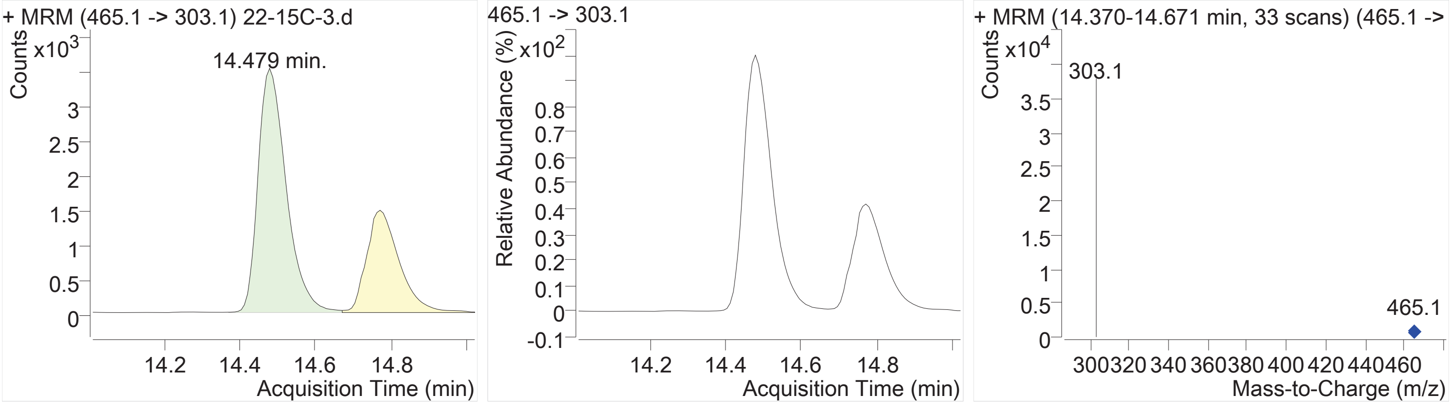

Rosmarinic acid

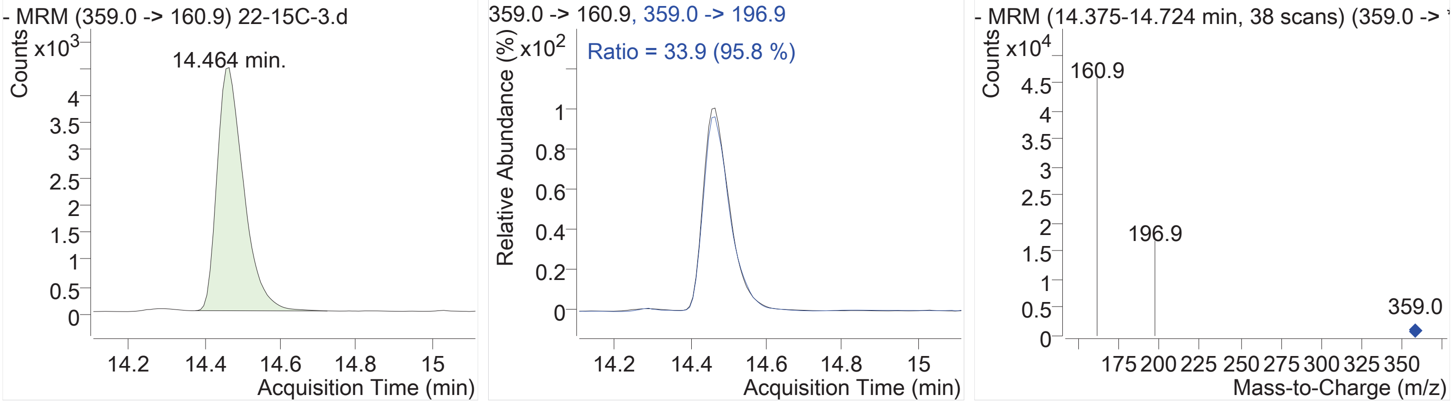

Apigenin 7-glucoside

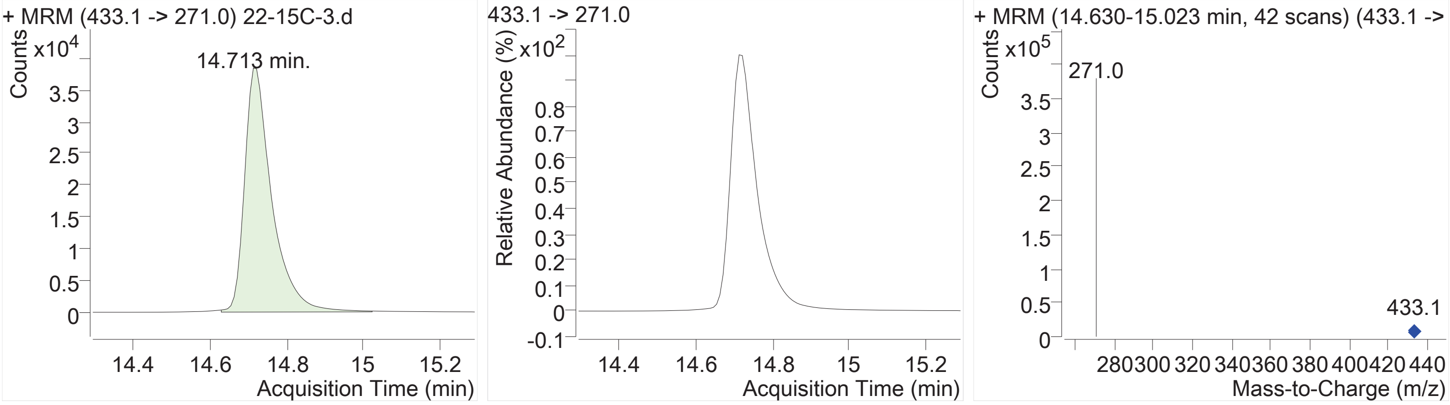

Pinoresinol

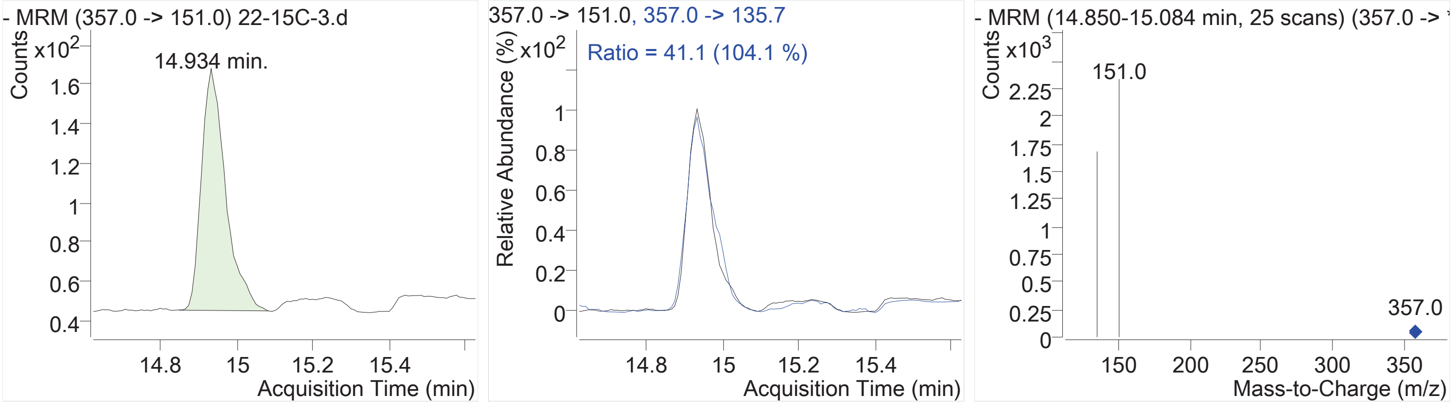

2-Hydroxycinnamic acid

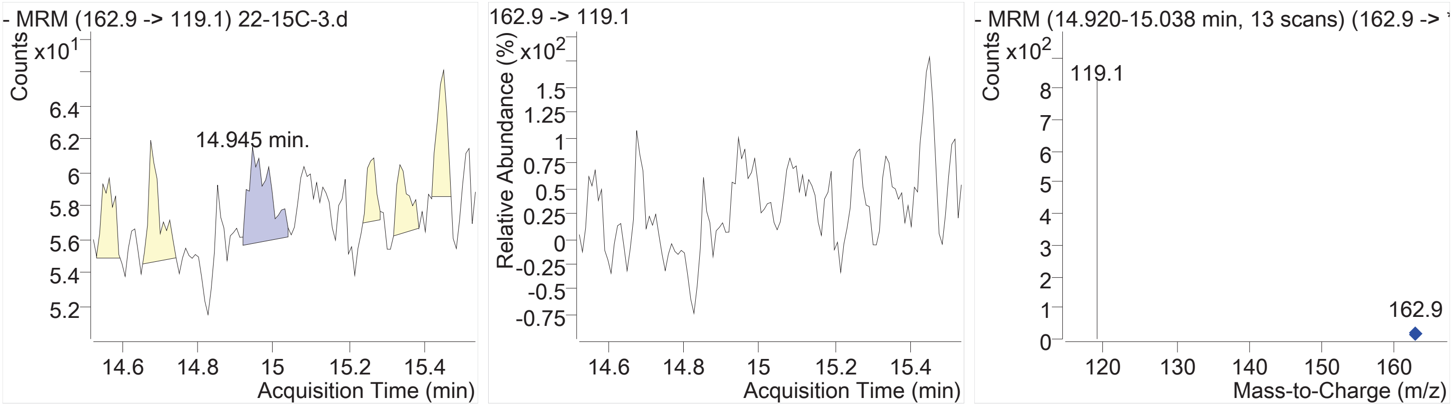

Eriodictyol

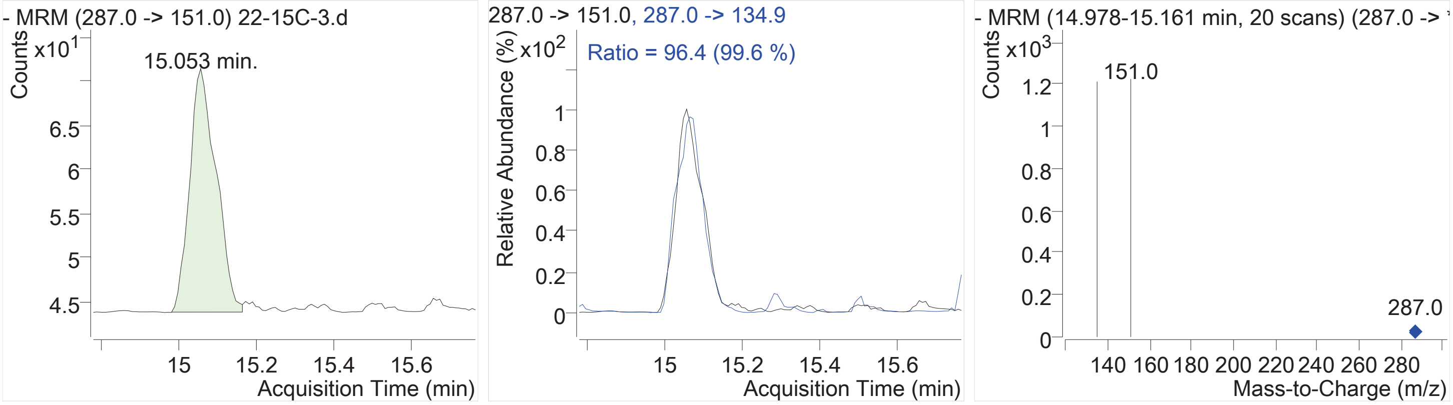

Quercetin

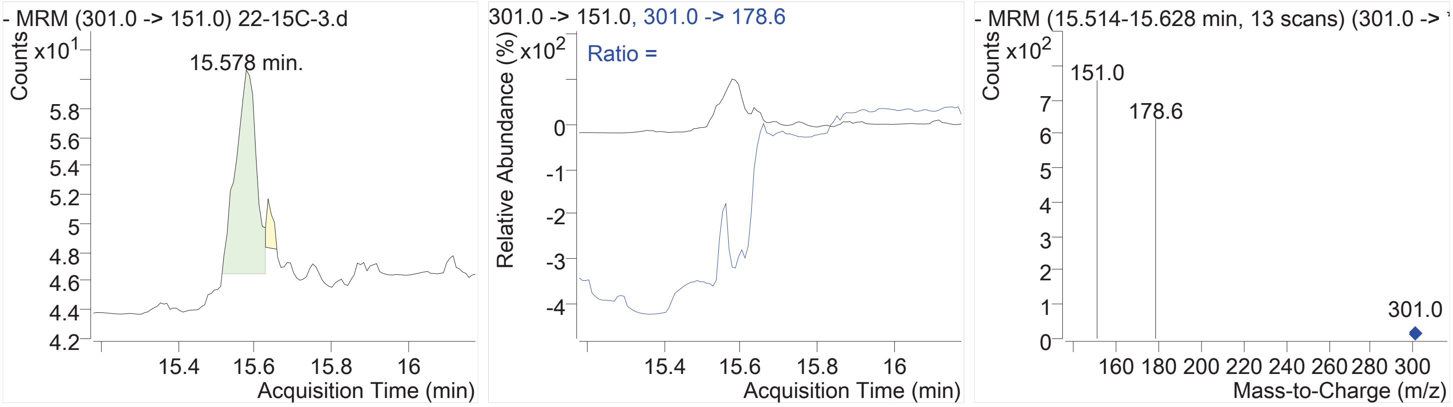

Luteolin

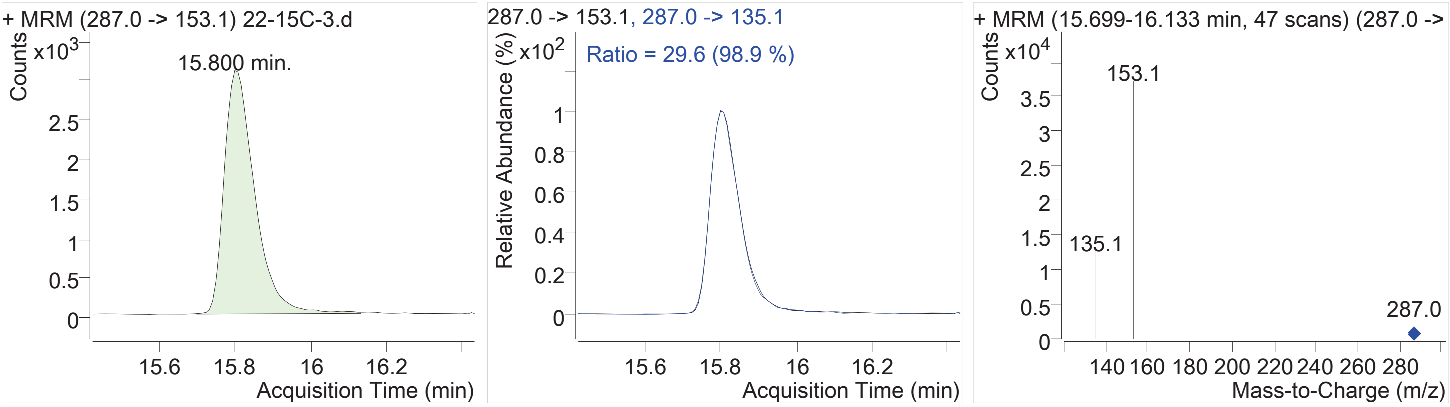

Kaempferol

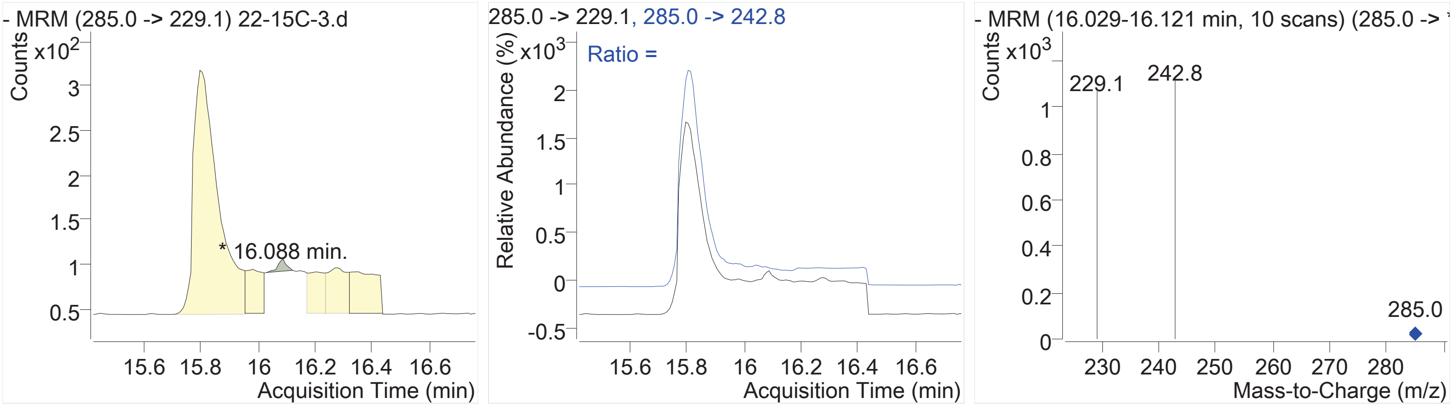

Apigenin

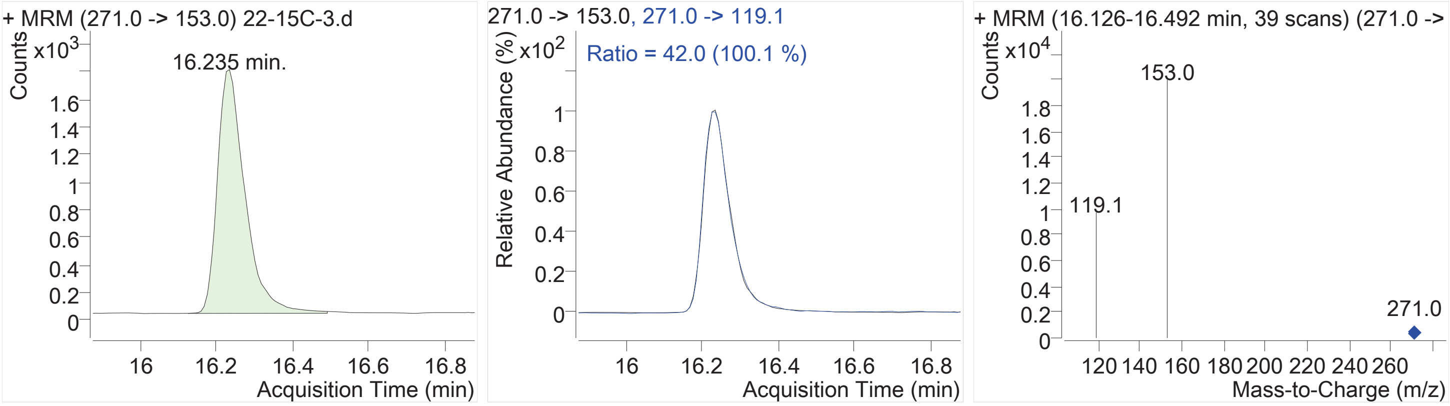

Supplement: S1 Data — (PDF) [file pone.0350995.s007.pdf]
